# Catalytic asymmetric reductive hydroalkylation of enamides and enecarbamates to chiral aliphatic amines

**DOI:** 10.1038/s41467-021-21600-x

**Published:** 2021-02-26

**Authors:** Jia-Wang Wang, Yan Li, Wan Nie, Zhe Chang, Zi-An Yu, Yi-Fan Zhao, Xi Lu, Yao Fu

**Affiliations:** Hefei National Laboratory for Physical Sciences at the Microscale, CAS Key Laboratory of Urban Pollutant Conversion, Anhui Province Key Laboratory of Biomass Clean Energy, iChEM, University of Science and Technology of China, Hefei, 230026 China

**Keywords:** Asymmetric catalysis, Reaction mechanisms, Synthetic chemistry methodology

## Abstract

To increase the reliability and success rate of drug discovery, efforts have been made to increase the C(*sp*^3^) fraction and avoid flat molecules. *sp*^3^-Rich enantiopure amines are most frequently encountered as chiral auxiliaries, synthetic intermediates for pharmaceutical agents and bioactive natural products. Streamlined construction of chiral aliphatic amines has long been regarded as a paramount challenge. Mainstream approaches, including hydrogenation of enamines and imines, C–H amination, and alkylation of imines, were applied for the synthesis of chiral amines with circumscribed skeleton structures; typically, the chiral carbon centre was adjacent to an auxiliary aryl or ester group. Herein, we report a mild and general nickel-catalysed asymmetric reductive hydroalkylation to effectively convert enamides and enecarbamates into drug-like α-branched chiral amines and derivatives. This reaction involves the regio- and stereoselective hydrometallation of an enamide or enecarbamate to generate a catalytic amount of enantioenriched alkylnickel intermediate, followed by C–C bond formation via alkyl electrophiles.

## Abstract (web summary)

Enantiopure aliphatic amines are frequently encountered as chiral auxiliaries and synthetic intermediates for bioactive compounds. Here, the authors report a mild nickel-catalysed asymmetric reductive hydroalkylation to convert enamides and enecarbamates into α-branched chiral amines and derivatives.

## Introduction

**Fig. 1:**
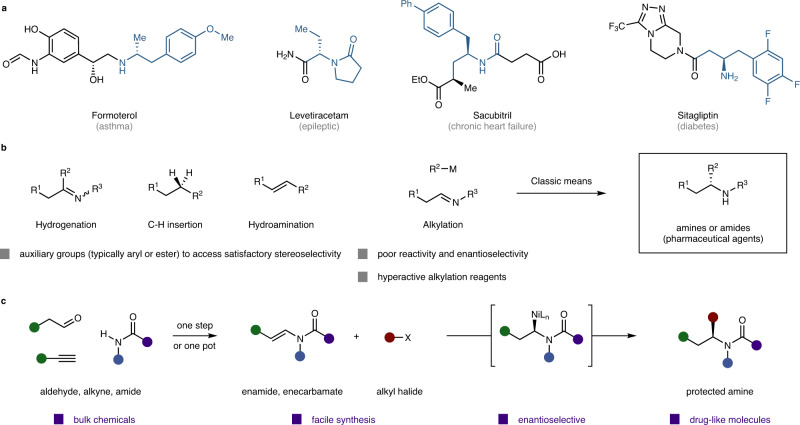
Design of a modular enamide/enecarbamate reductive hydroalkylation for the synthesis of chiral aliphatic amines. **a** Representative drug molecules demonstrating the universal existence of chiral aliphatic amines in biologically active molecules. **b** Catalytic asymmetric reductive hydroalkylation of enamides and enecarbamates enables rapid access to the privileged chiral aliphatic amines, complementing mainstream approaches that are limited by auxiliary groups to access satisfactory stereoselectivity. **c** Synthetic analysis and proposed mechanism of enamide and enecarbamate reductive hydroalkylation. Enamides and enecarbamates are prepared via facile syntheses from bulk chemicals—aldehydes, alkynes and amides. It was assumed that this reaction involved regio- and stereoselective hydrometallation of an enamide or enecarbamate to generate a catalytic amount of enantioenriched alkylnickel intermediate, followed by C–C bond formation with alkyl electrophile to yield chiral amine.

Enantiopure amines are frequently encountered as chiral auxiliaries and synthetic intermediates for pharmaceutical agents and bioactive natural products (Fig. 1a)^1,2^. Nearly half of small-molecule pharmaceuticals among the top 200 drugs by retail sales in 2019 contain an enantioenriched aliphatic amine as the key structural element^3^. To synthesise amines efficiently^4–8^, especially those chiral ones, exciting strides in the fields of hydrogenation of enamines and imines^9–13^, C–H amination^14–16^ and hydroamination of alkenes^17–21^ have been made to supplement the nucleophilic substitution reactions of the corresponding chiral alcohols (Fig. 1b). However, the primary problem, inadequate substrate scope, still remains. In the aforementioned classical methods, especially the hydrogenation and hydroamination of unsaturated bonds, chiral amines with circumscribed skeleton structures could be successfully synthesised; typically, the chiral carbon centre should be adjacent to an auxiliary aryl^13^ or ester group^22^. As an important process, Buchwald realised the efficient hydroamination of unactivated internal olefin to α-branched amine with an excellent enantioselectivity^17^. Very recently, Hartwig realised the direct, enantioselective intermolecular hydroamination of unactivated alkene lacking a directing group^21^. As another prevalent method to selectively synthesise enantioenriched amides is the catalytic asymmetric alkylation of imines through carbon–carbon bond formation. However, only a few catalytic enantioselective alkylations of imines with particular hyperactive alkylation reagents, typically relatively air- and moisture-sensitive Grignard reagents^23^ or zinc reagents^24,25^, have been reported. Poorly electrophilic alkyl-substituted imines presented poor reactivity, and the C–N double bond geometrical isomer mixture led to unsatisfactory enantioselectivities.

In designing a synthetically useful method for synthesising *sp*^3^-rich chiral amines and derivatives to meet the increased demand in drug discovery, we innovated an operationally facile reaction using easily synthesised enamides with readily available and batch-stable alkyl halides under reductive conditions. Our group devoted efforts to develop olefin reductive alkylation reactions^26–30^. Nowadays, olefin reductive hydroalkylation has been one of the most appealing methods for alkyl–alkyl formation^31–35^. In the elegant work from Fu’s group at Caltech^32^, enantioconvergent coupling of a broad scope of racemic alkyl electrophiles with olefins was achieved. Furthermore, conversion of unsaturated compounds other than olefins to construct alkyl–alkyl bonds may open the door to efficient methods for chiral centre construction^36^. Herein we report a reductive hydroalkylation of generalised organic unsaturated bonds, namely, the reductive hydroalkylation process of enamides and enecarbamates (Fig. 1c)^37–40^. This reaction involves in situ hydrometallation of enamide or enecarbamate to generate prospective enantioenriched alkylnickel intermediates, which subsequently react with alkyl halides to form C(*sp*^3^)–C(*sp*^3^) bonds, representing an enantioselective control mode different from the well-known nickel-catalysed electrophile–nucleophile cross-coupling reactions developed by Fu at Caltech^41,42^. This reaction enables the construction of structurally complex and multifunctional amides and β-aminoboronates with excellent functional-group compatibility suitable for late-stage elaboration applications. This reaction also facilitates the design and development of drug-like nitrogen-containing chiral molecules that are difficult to access by other synthetic routes, resulting in widespread application in the pharmaceutical sector and synthetic chemistry field.

## Results and discussion

### Substrates scope

**Fig. 2:**
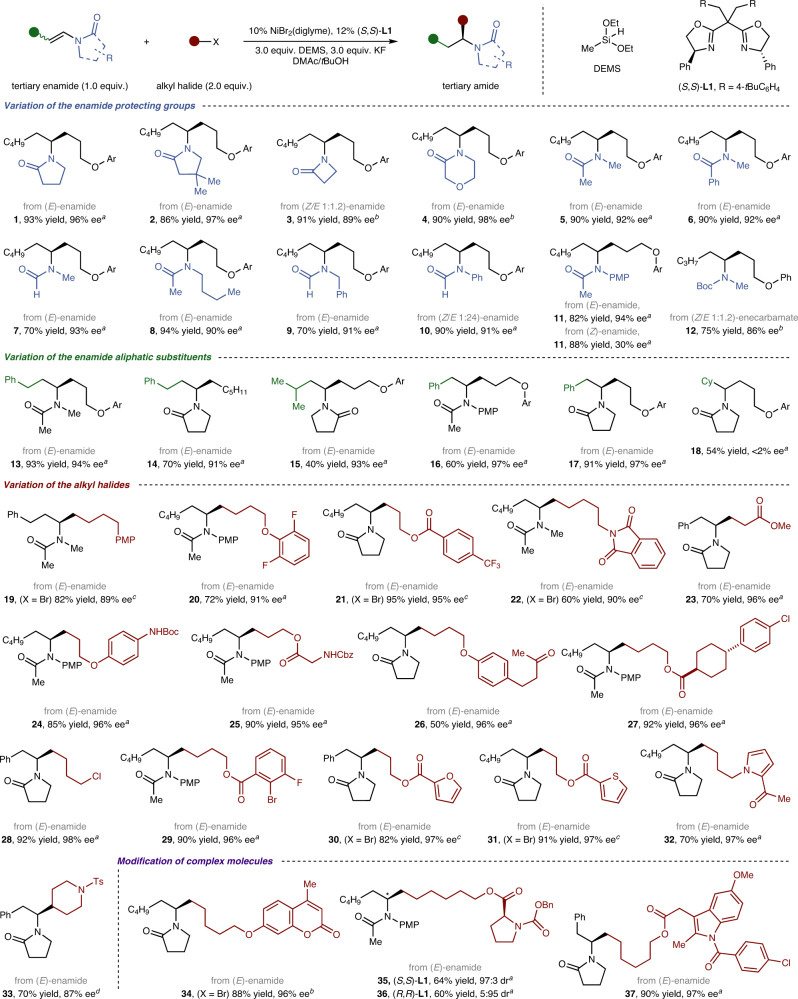
Asymmetric reductive hydroalkylation of tertiary enamides. ^*a*^DMAc:*t*BuOH (v/v = 5:4, 0.25 M), 25 °C, 20 h, isolated yield, 0.20 mmol scale. ^*b*^DMAc:*t*BuOH (v/v = 5:4, 0.25 M), 5 °C, 30 h, isolated yield, 0.20 mmol scale. ^*c*^DMAc:*t*BuOH (v/v = 5:4, 0.25 M), NaI (0.5 equiv.), 35 °C, 20 h, isolated yield, 0.20 mmol scale. ^*d*^DMAc:*t*BuOH (v/v = 5:4, 0.25 M), 50 °C, 20 h, isolated yield, 0.20 mmol scale. ^*e*^Diastereocenters marked with an asterisk symbol. Ar = 2-naphthyl, Diglyme = 2-methoxyethyl ether, DMAc = *N*,*N*-dimethylacetamide, PMP = *p*-methoxyphenyl, Boc = *tert*-butoxycarbonyl, Cbz = carbobenzyloxy, Ts = tosyl, Bn = benzyl, dr = diastereomeric ratio.

At the beginning of this study, we determined that this enamide reductive hydroalkylation could occur when a chiral nickel/bisoxazoline catalyst was combined with diethoxymethylsilane (DEMS) and KF in a DMAc/*t*BuOH mixed solvent. For more details for optimisation of reaction conditions, see Supplementary Table 1. Under our optimised conditions, a fairly broad scope of tertiary enamides served as active substrates to deliver the desired products (Fig. 2). We noticed that the major enantiomer of the product was determined by the configuration of the ligands (see Supplementary Note 4), and the (*E*)- or (*Z*)-enamides markedly affected the enantioselectivities (**11**). The (*E*)-substrate afforded a much higher level of enantioselectivity (94% enantiomeric excess). Fortunately, (*E*)-substrate was indeed the major isomer in the synthesis of tertiary enamide from the corresponding aldehyde. Although the amino-protecting groups varied in terms of steric and electronic properties, high yields (70–94% yield) and high enantioselectivities (86–97% e.e. (enantiomeric excess)) were achieved in all cases (**1**–**12**). The alkyl substituents on the enamide were also investigated; steric hindrance has a typical effect on the coupling efficiency (**13**–**17**). In the case of less bulky alkyl substituents, good yields were achieved, and vice versa. The enantioselectivity was hardly affected by the alkyl substituents, with the only exception being β,β-dialkyl-substituted enamides (**18**), which were produced in moderate yield with complete loss of enantioselectivity. *N*-styrylenamides (**16, 17**), which exhibit competitive reactivity at the benzylic position, were also good substrates to deliver the single *N*-α-alkylation-selective products.

With respect to alkyl electrophiles, both alkyl iodides and alkyl bromides could be converted efficiently (**19–33**). Relatively inert alkyl bromides (**19**, **21**, **22**) required higher reaction temperatures and a NaI additive to achieve satisfactory outcomes. A wide array of functional groups was compatible; for example, a phthalimide (**22**), an alkyl ester moiety (**23**), an amide possessing N–H bonds (**24**, **25**) and a base-sensitive ketone (**26**) were well tolerated. This reaction could also be conducted in the presence of an aryl chloride (**27**), an alkyl chloride (**28**) and an aryl bromide (**29**), thus providing an exceptional opportunity for further transformation at the preserved carbon–halogen bonds. In addition, several highly reactive electron-rich heterocycles (**30–32**)—which could undergo facile Friedel-Crafts reaction or C–H activation—were retained during the transformation. Finally, the late-stage diversification of complex molecules illustrated the high degree of compatibility of diverse functional groups for this enamide reductive hydroalkylation (**34**–**37**).

**Fig. 3:**
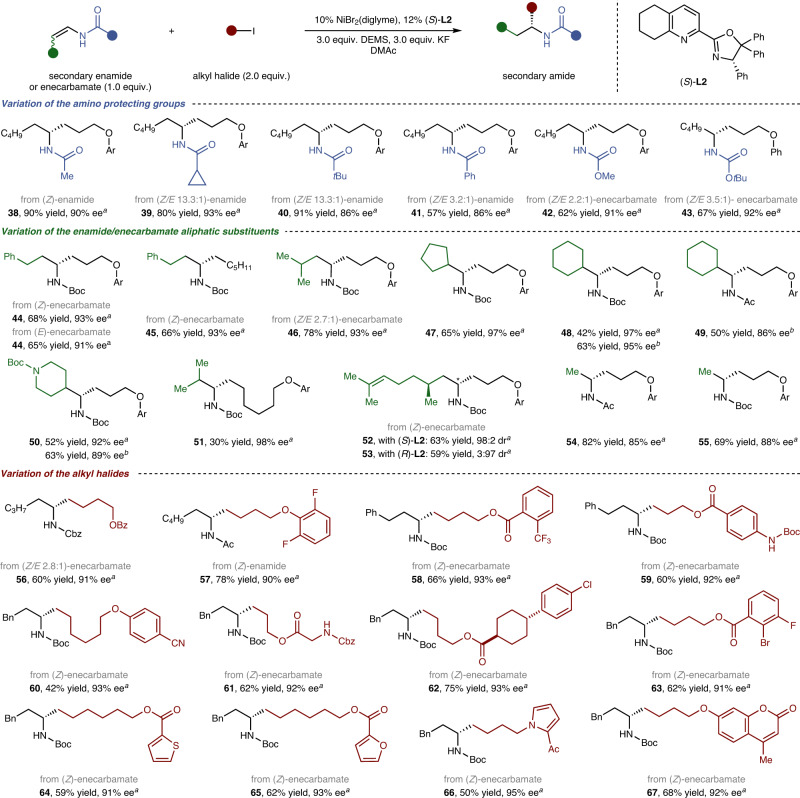
Asymmetric reductive hydroalkylation of secondary enamides or enecarbamates. ^*a*^DMAc (0.2 M), −27 °C, 40 h, isolated yield, 0.20 mmol scale. ^*b*^DMAc (0.2 M), 0 °C, 40 h, isolated yield, 0.20 mmol scale. ^*c*^Diastereocenters marked with an asterisk symbol. Ar = 2-naphthyl, Ac = acetyl, Bz = benzoyl.

Next, we aimed to expand the scope of asymmetric reductive hydroalkylation to include a second family of substrates, specifically, secondary enamides or enecarbamates (Fig. 3). Whereas reactions using the aforementioned method result in poor yield and enantioselectivity, as depicted in Fig. 2, secondary enamides or enecarbamates performed well under modified conditions after considerable effort. For more details for optimisation of reaction conditions, see Supplementary Table 2. Different from the tertiary enamides, in the cases of secondary enamides or enecarbamates, both (*E*)- and (*Z*)-substrates afforded comparable coupling yields and enantioselectivities (**44**). We could use the mixture of two isomers without additional isomer separation process. The scope of reductive hydroalkylation proved broad with respect to both the enamide, the enecarbamate and the alkyl halide (**38–67**). The amino-protecting group and aliphatic substituent could be altered and could tolerate many functional groups without any detrimental effect on the coupling yield or enantioselectivity (**38–55**). The improved flexibility of the amino-protecting groups, especially the ability to work with Boc (*tert*-butoxycarbonyl)-protecting groups (e.g. **43**–**48, 50–53**, **58–67**), ameliorated the practicability and applicability of this reaction. Furthermore, even more sterically encumbered β,β-dialkyl-substituted ones (**47–51**) could be converted to the desired products with excellent enantioselectivities, which was significantly different from the results obtained when using tertiary enamides. Similarly, a wide range of synthetically useful functional groups, such as an internal alkene (**52,** **53**), an aryl fluoride (**57**), a trifluoromethyl (**58**) and a cyano (**60**), were tolerated. Privileged heterocyclic motifs such as thiophene (**64**), furan (**65**), pyrrole (**66**) and coumarin (**67**) are commonly found in medicinal drugs and pose no problems during transformation.

### Mechanism study

**Fig. 4:**
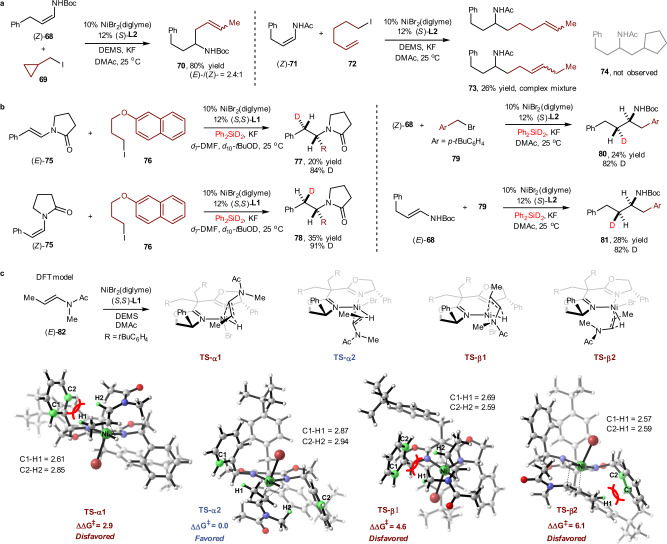
Mechanistic studies of enamide reductive hydroalkylation. **a** Radical clock experiments. (**b**) Deuterium-labelling experiments. (**c**) DFT calculations. DFT calculations were also performed at the B3LYP-D3(BJ)/6-311G(d,p)-SDD-SMD(DMA)//B3LYP-D3(BJ)/6-31G*-LANL2DZ-SMD(DMA) level of theory. For more details, see Supplementary Notes 3 and 5.

To examine the reaction mechanism of enamide/enecarbamate reductive hydroalkylation, we carried out radical clock experiments (Fig. 4a). Alkyl iodide (**69**) containing a cyclopropyl ring is a frequently used radical clock substrate and was applied under standard reaction conditions. The ring-opened product (**70**) was obtained in 80% isolated yield, though the hypothetical terminal double bond migrated to the interior. We also tested the reductive hydroalkylation of 5-iodopent-1-ene (**72**). The assumed ring-cyclized product (**74**) was not observed, but a complex mixture of linear coupling products (**73**) was obtained, which revealed that the migration of vinyl double bonds was a very rapid primitive step. Despite all this, it was concluded that the activation of alkyl halides proceeded through a radical pathway. Then, we took advantage of deuterated silane (Ph_2_SiD_2_) to study the stereochemistry of this reaction (Fig. 4b). Deuterium-labelling experiments revealed that this reductive hydroalkylation was completely diastereoselective. The stereochemical results indicated that this reaction proceeded through a Ni-D intermediate formation and *syn*-addition to an enamide or enecarbamate to generate prospective enantioenriched alkylnickel intermediates (for more details, see Supplementary Note 3)^43^. Totally, a chiral organometallic intermediate was formed and the chiral centre was located on nucleophilic site (enamide or enecarbamate), the stereochemical control was determined by the highly regio- and enantioselective Ni-H insertion. By contrast, in the work of Fu at Caltech, an achiral organometallic intermediate was formed and chiral centre was located on alkyl electrophile site, the stereochemical control came from oxidative addition and/or reductive elimination^32^. Thus, the essential nature of asymmetric catalysis, namely the enantioselectivity control site and determining step, was different between G. C. Fu’s work and our discovery. In addition, we ruled out the possibility of *Z*/*E* isomerization (for more details, see Supplementary Note 4). Finally, DFT calculations were carried out (see Supplementary Note 5 and Supplementary Data 1), and the simulation results were highly consistent with the results of the mechanistic study (Fig. 4c). The optimised structures and relative Gibbs free energies of the four transition states in the Ni-H insertion step are summarised. As shown, **TS-α2** corresponds to the major (*R*)-*N*-α-alkylation product, and it has the lowest free energy, which is in line with the experimental outcomes. Examining the structures, it can be seen that the C1–H1 and C2–H2 bond lengths in **TS-α2** are 2.87 Å and 2.94 Å, respectively, which are longer than the related ones in the other competing TSs corresponding to (*S*)-*N*-α-alkylation products and *N*-*β*-alkylation products (i.e. **TS-α1**, **TS-β1** and **TS-β2**). Therefore, the favoured transition state **TS-α2** has larger C–H bond distances between methyl groups of the substrate and phenyl groups of the ligand, resulting in the lower steric hindrance, and thus the lowered activation barrier therein. In addition, we also examined the effect of the (*Z*)-configuration on the enantioselectivity by calculating **TS-α1** and **TS-α2** with the (*Z*)-substrate (for more details, see Supplementary Note 5). The free energy gap between **TS-α1-Z** and **TS-α2-Z** (**Z** signifies the (*Z*)-substrate) is decreased to 1.6 kcal/mol compared to that of 2.9 kcal/mol between **TS-α1** and **TS-α2**, which is consistent with the higher level of enantioselectivity of the (*E*)-substrate. Together, the mechanistic study and DFT calculations confirmed our proposed mechanism and reaction design, as shown in Fig. 1c.

### Substrates scope of β-aminoboronates

**Fig. 5:**
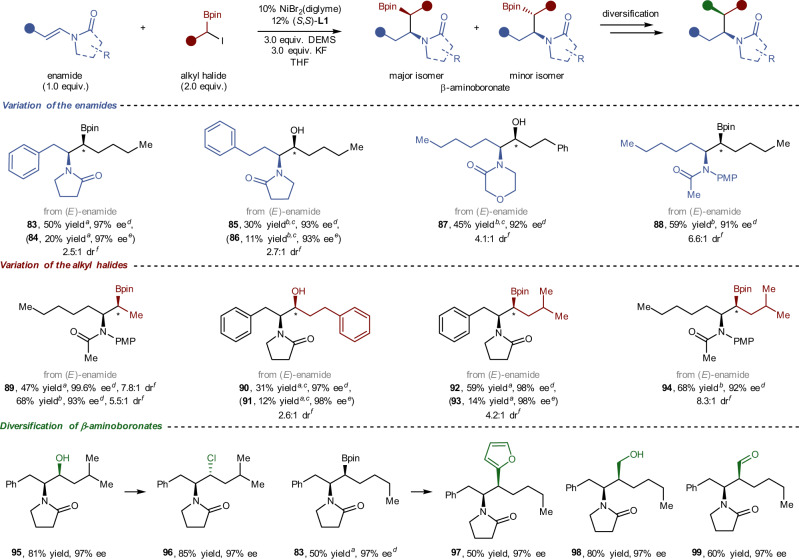
Asymmetric reductive hydroalkylation of enamides with racemic α-haloboronates to access β-aminoboronates. ^*a*^25 °C, 24 h, isolated yield, 0.20 mmol scale. ^*b*^40 °C, 24 h, isolated yield, 0.20 mmol scale. ^*c*^The product was isolated after the oxidisation of the corresponding alkylborate. ^*d*^enantiomeric excess of major isomer after oxidisation. ^*e*^enantiomeric excess of minor isomer after oxidisation. ^*f*^The dr value was determined via ^1^H NMR analysis of crude β-aminoboronates or HPLC analysis of crude β-aminoalcohols. ^*g*^Diastereocenters marked with an asterisk symbol. Bpin = boronic acid pinacol ester.

Chiral amino alcohol skeleton structures are ubiquitously found in bioactive natural products or drug molecules. They are also extensively used as chiral pools, ligands or catalysts for asymmetric catalysis. The best known route to obtain amino alcohols—reduction of amino acids—requires prior multistep synthesis using the highly toxic metal cyanide^44^. Building on our preceding foundation, we establish that enamide reductive hydroalkylation with racemic α-haloboronates to access β-aminoboronates can be accomplished (Fig. 5, **83–94**). Our method, based on classical alkylborate transformations and the Mitsunobu reaction, provided a modular and simplified strategy for the synthesis of chiral amino alcohols and other useful families of enantioenriched molecules (e.g. heterocycles, aldehydes and alkyl halides) with little loss of stereochemistry at both the boron-bound and nitrogen-bound carbons (**95**–**99**). A wide array of enamides and α-haloboronates, which varied in amino-protecting groups and side chains, served as suitable coupling partners in this enamide reductive hydroalkylation reaction. Reductive hydroalkylation proceeded well with consistently good yields and high levels of stereochemical control on the carbon adjacent to the *N*-atom with all substrates. Although mediocre diastereoselectivities on the carbon adjacent to the *B*-atom were observed in a few cases (e.g. **83**–**86**), these two diastereoisomers could be prepared in a single reaction and then separated through column chromatography.

### Synthetic transformations

**Fig. 6:**
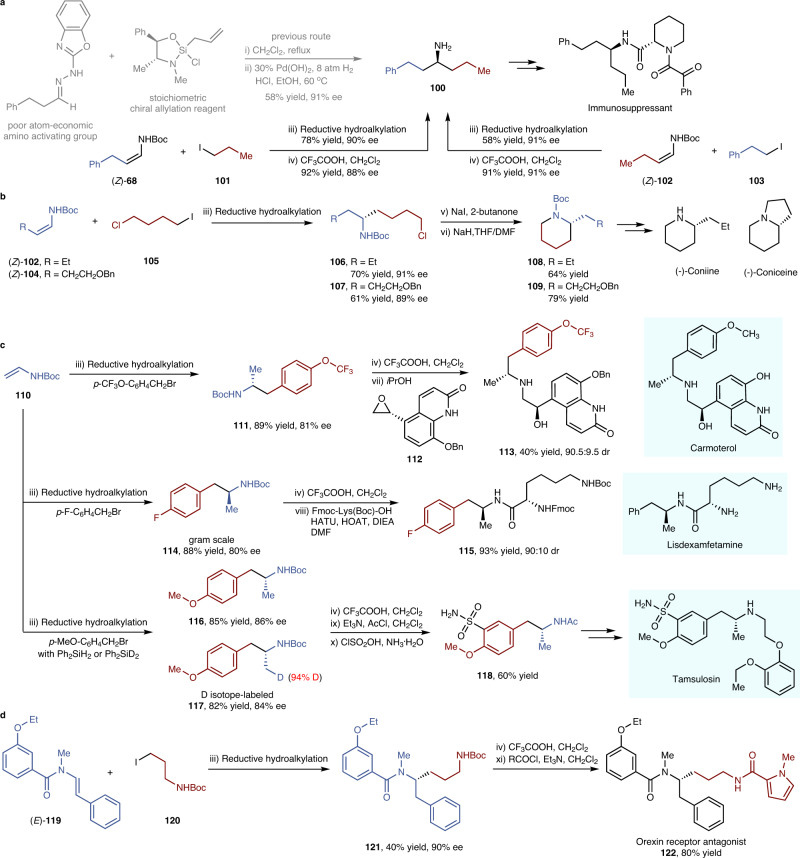
Synthetic transformations of reductive hydroalkylation products. **a** Comparison of enecarbamate reductive hydroalkylation with conventional methods for the synthesis of the key intermediate of an immunosuppressant agent. **b** Synthesis of alkaloids with enecarbamate reductive hydroalkylation as a key step. **c** Modular synthesis of isotope- or fluorine-labelled central nervous system drugs via enecarbamate reductive hydroalkylation. **d** Formal synthesis of drug analogues that contain a chiral carbon adjusted to a tertiary amide. All yields refer to isolated yield of purified product. For more details for reaction conditions, see Supplementary Note 1. THF = tetrahydrofuran, DMF = *N*,*N*-dimethylformamide, Fmoc = 9-fluorenylmethoxycarbonyl, Fmoc-Lys(Boc)-OH = (*S*)-2-((((9*H*-fluoren-9-yl)methoxy)carbonyl)amino)-6-((*tert*-butoxycarbonyl)amino)hexanoic acid, HATU = 2-(7-azabenzotriazol-1-yl)-*N*,*N*,*N*’,*N*’-tetramethyluronium hexafluorophosphate, HOAT = 1-hydroxy-7-azabenzotriazole, DIEA = *N*,*N*-diisopropylethylamine.

Given that chiral amine motifs exist in many small-molecule pharmaceutical agents and physiologically active natural compounds, the convenient synthesis of such molecules and medicinally valuable derivatives would demonstrate the availability and practicability of our reaction (Fig. 6). A representative chiral amine **100**, the key intermediate of an immunosuppressive agent, features two sterically and electronically similar alkyl substrates. The conventional method to access **100** requires time-consuming and tedious synthesis using a stoichiometric chiral allylation reagent, a poor atom-economic amino-activating group and high hydrogen pressure^45^. By capitalising on the flexible retrosynthetic analysis of our reductive hydroalkylation, amine **100** could be conveniently accessed through the use of readily available raw materials via an asymmetric catalytic method (Fig. 6a). This reductive hydroalkylation was also useful for the total synthesis of alkaloids, such as coniine and coniceine. The commercially available raw material **105** was employed in the reductive hydroalkylation, and desired products **106** and **107** were obtained in an efficient fashion. Subsequent intramolecular cyclization on the accommodated alkyl chloride groups yielded the pivotal intermediates and synthesis targets (Fig. 6b, **108**, **109**). An advantage of the current catalytic asymmetric reductive hydroalkylation is its substantial capacity to quickly and efficiently access 1-arylpropan-2-amines (e.g. amphetamine), which are key components of central nervous system drugs. The vast array of benzyl bromides and benzyl alcohols could be easily programmed into arylpropanamines with high structural and functional diversity. Various isotope- or fluorine-labelled pharmaceutical agents, such as trifluoromethyl-labelled Carmoterol (an asthma medicine), fluorine-labelled Lisdexamfetamine (an attention deficit and hyperactivity disorder medicine) and deuterium isotope-labelled Tamsulosin (a urinary system medicine), were produced via brief synthetic routes (Fig. 6c, **113**, **115**, **118**), highlighting the suitability of this reductive hydroalkylation in drug discovery. Additionally, reductive hydroalkylation of tertiary enamides also shows promise in tailor-made applications in the synthesis of the orexin receptor antagonist **122**, which contains a chiral carbon adjacent to a tertiary amide (Fig. 6d).

In summary, we describe a streamlined method to access chiral aliphatic amines and their derivatives through nickel-catalysed asymmetric reductive hydroalkylation of enamides and enecarbamates with alkyl halides. This reaction complements the substrate scope of traditional chiral amine synthesis strategies, overcomes the limitation of essential and finite auxiliary groups, and thus facilitates the chiral amine synthesis. We believe that the ready accessibility of raw materials, convenient implementation and efficiency of this highly stereoselective amine synthesis will lead to many applications in organic chemistry and pharmaceutical chemistry^46,47^.

## Methods

### General procedure for asymmetric reductive hydroalkylation of tertiary enamides

In the air, a 10 mL screw-cap test tube equipped with a magnetic stirrer was charged with NiBr_2_(diglyme) (0.02 mmol, 10 mol%), (*S*,*S*)-**L1** (0.024 mmol, 12 mol%). The test tube was evacuated and backfilled with argon for three times, then DMAc/*t*BuOH (*v*/*v* = 5:4, 0.8 mL) was added and the mixture was stirred at room temperature for 30 min. Meanwhile, in the air, another 10 mL screw-cap test tube equipped with a magnetic stirrer was charged with KF (0.6 mmol, 3.0 equiv.) (if enamide or alkyl halide was a solid, it was also added at this time). The test tube was evacuated and backfilled with argon for three times. Next, the solution of the catalyst was added in one portion via syringe, followed by the enamide (0.2 mmol, 1.0 equiv.) and alkyl halide (0.4 mmol, 2.0 equiv.). The resulting solution was stirred for 2 min at 0 °C, DEMS (0.6 mmol, 3.0 equiv.) was added dropwise via syringe and the solution was kept stirring for 5 min at 0 °C, then was stirred at 25 °C for 20 h. The reaction mixture was diluted with H_2_O followed by extraction with EtOAc, dried with anhydrous Na_2_SO_4_ and concentrated in vacuo. The residue was purified by flash column chromatography on silica gel to give the target product.

### General procedure for asymmetric reductive hydroalkylation of secondary enamides and enecarbamates

In the air, a 10 mL screw-cap test tube equipped with a magnetic stirrer was charged with NiBr_2_ (diglyme) (0.02 mmol, 10 mol%), (*S*)-**L2** (0.024 mmol, 12 mol%). The test tube was evacuated and backfilled with argon for three times, then DMAc (1.0 mL) was added and the mixture was stirred at room temperature for 30 min. Meanwhile, in the air, another 10 mL screw-cap test tube equipped with a magnetic stirrer was charged with KF (0.6 mmol, 3.0 equiv.) (if alkyl halide was a solid, it was also added at this time). The test tube was evacuated and backfilled with argon for three times, and the test tube was then placed in an EtOH cooling bath at −27 °C. Next, the solution of the catalyst was added in one portion via syringe, the reaction mixture was stirred at −27 °C for 10 min, DEMS (0.6 mmol, 3.0 equiv.) was added dropwise via syringe over 1 min, and then the reaction mixture was stirred for another 5 min. Finally, the enamide or enecarbamate (0.2 mmol, 1.0 equiv.) and alkyl halide (0.4 mmol, 2.0 equiv.) were added. The reaction mixture was stirred at −27 °C for 40 h. The reaction mixture was diluted with H_2_O followed by extraction with EtOAc, dried with anhydrous Na_2_SO_4_ and concentrated in vacuo. The residue was purified by flash column chromatography on silica gel to give the target product.

## Supplementary information

Supplementary Information

Peer Review File

Description of Additional Supplementary Files

Supplementary Data 1

## Data Availability

The authors declare that the data supporting the findings of this study are available within the article and Supplementary Information files, or from the corresponding author upon reasonable request. The experimental procedures, computational results and characterisation of all new compounds are provided in the Supplementary Information. The X-ray crystallographic coordinates for structures reported in this study have been deposited at the Cambridge Crystallographic Data Centre (CCDC), under deposition numbers CCDC 2032484 (**90-OH**). These data can be obtained free of charge from The Cambridge Crystallographic Data Centre (www.ccdc.cam.ac.uk/data_request/cif).
