# Supplementary material for: Catalytic asymmetric reductive hydroalkylation of enamides and enecarbamates to chiral aliphatic amines
Source: Nat Commun. 2021 Feb 26;12:1313. doi: 10.1038/s41467-021-21600-x (PMC7910428; doi:10.1038/s41467-021-21600-x)
Supplement: Supplementary file 1 — Supplementary Information [file 41467_2021_21600_MOESM1_ESM.pdf]

Supplementary Information

Catalytic asymmetric reductive hydroalkylation of enamides and enecarbamates to chiral aliphatic amines

Wang, et al.

Tale of Contents

Supplementary Methods S3

General Information S3

Preparation of Alkyl Iodides S4

Preparation of Tertiary Enamides S13

Preparation of Secondary Enamides and Enecarbamates S21

Reductive Hydroalkylation of Tertiary Enamides S28

Reductive Hydroalkylation of Secondary Enamides and Enecarbamates S58

Reductive Hydroalkylation of Enamides with Racemic α-Haloboronates S81

Supplementary Note 1 ....S100

Synthetic Transformations of Enamide/Enecarbamate Reductive Hydroalkylation Products S100

Supplementary Note 2 ....S110

Assignment of Absolute Configuration...S110

Supplementary Note 3 ....S120

Mechanistic Studies...S120

Supplementary Note 4 ....S130

Effect of Configuration of Enamide and Ligand on Coupling Yields and Enantioselectiveties S130

Study of *Z/E* Interconversion of Enamide and Enecarbamate. S130

Supplementary Note 5 ....S132

Proposed Mechanism and DFT Calculations S132

Supplementary Figures S135

Supplementary References...S406

Supplementary Methods

General Information

Materials

The following chemicals were purchased and used as received: nickel(II) bromide 2-methoxyethyl ether complex (CAS: 312696-09-6, Aldrich, 459674-5G); diethoxymethylsilane (CAS: 2031-62-1, Adamas, 35415E); potassium fluoride (CAS: 7789-23-3, Acros, 21350250); tetrahydrofuran (CAS: 109-99-9, Adamas, 14018Q); *tert*-butanol (CAS: 75-65-0, J&K, 974855); *N*,*N*-dimethylacetamide (CAS: 127-19-5, Adamas, 18935J).

Caution: Eye protections are necessary when handling alkoxysilanes. Alkoxysilanes might be disproportionated to form pyrophoric SiH_4; please take suitable precautions.

Analytical Methods

^{1}H NMR, ^{13}C NMR, ^{11}B NMR and ^{19}F NMR spectra were recorded on Bruker 400 MHz spectrometer and Bruker 500 MHz spectrometer at 295 K in $CDCl_3$ unless otherwise noted. Data for ^{1}H NMR were reported as follows: chemical shift (δ ppm), multiplicity, coupling constant (Hz), and integration. Data for ^{13}C NMR were reported as follows: chemical shift (δ ppm), multiplicity, and coupling constant (Hz). Data for ^{11}B NMR were reported as follows: chemical shift (δ ppm), multiplicity, and coupling constant (Hz). Data for ^{19}F NMR were reported as follows: chemical shift (δ ppm), multiplicity, coupling constant (Hz). Chemical shifts were reported using the residual solvent $CHCl_3$ as the internal reference for ^{1}H NMR (δ = 7.260 ppm) and $CDCl_3$ peak as the internal reference for ^{13}C NMR (δ = 77.160 ppm). High-resolution mass spectral analysis (HRMS) data were acquired on Water XEVO G2 Q-TOF (Waters Corporation). HPLC analysis were carried out on a Shimadzu system with Daicel columns. Gas chromatographic (GC) analysis was acquired on a Shimadzu GC-2010 plus Series GC system equipped with a flame-ionization detector. Organic solutions were concentrated

under reduced pressure on Buchi rotary evaporator. Column chromatographic purification of products was accomplished using forced-flow chromatography on Silica Gel (300-400 mesh).

Preparation of Alkyl Iodides

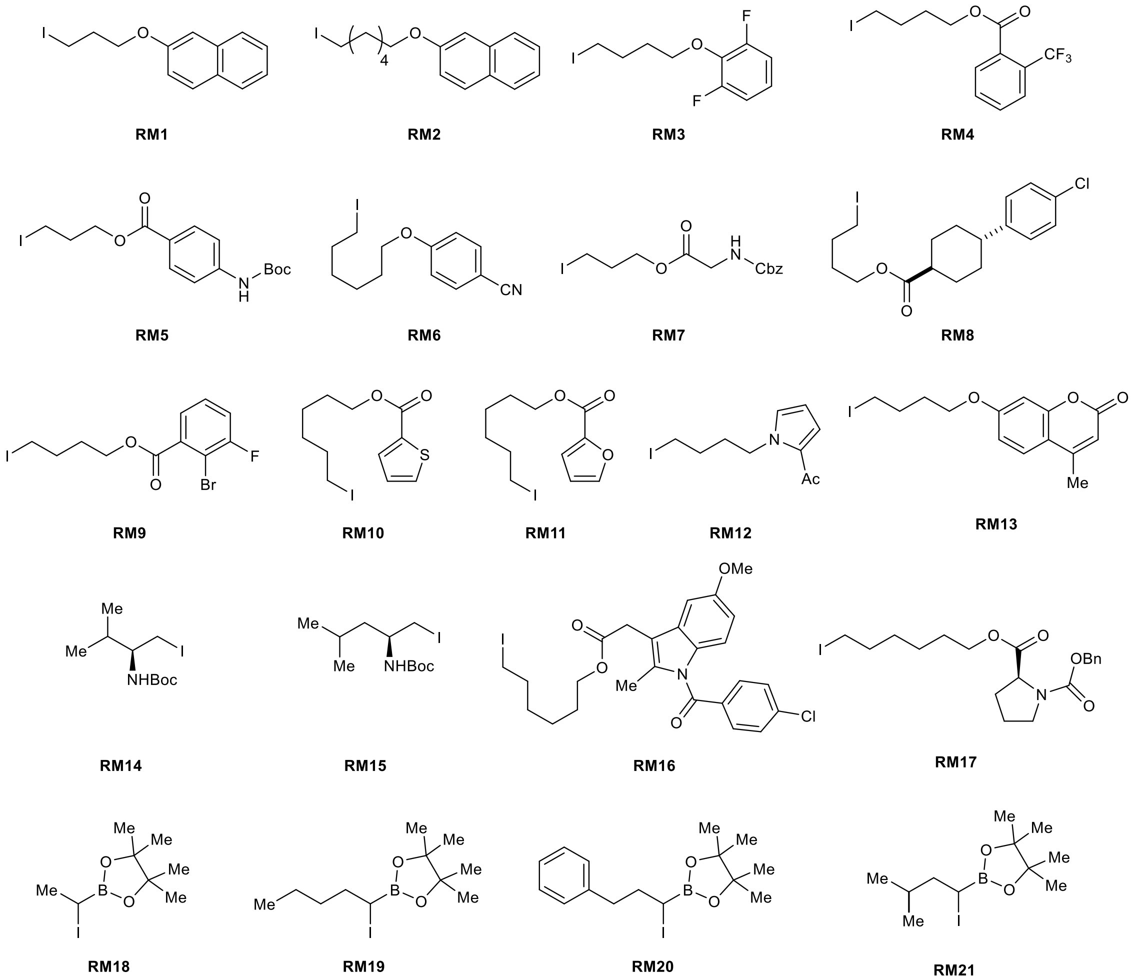

Supplementary Figure 1. List of Alkyl Iodides

General procedure A1 (GP-A1)

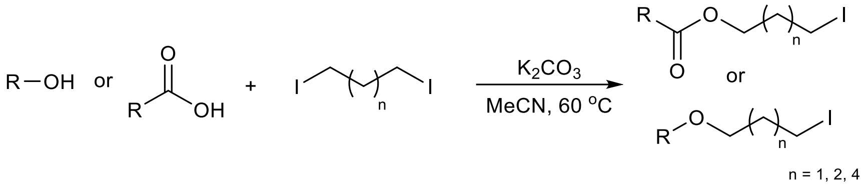

According to the reported literature[1], alkyl iodide (**RM1-RM13**, **RM16-RM17**) were conveniently synthesized according to **GP-A1** in gram scale.

General procedure A2 (GP-A2)

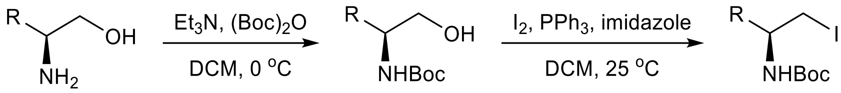

According to the reported literature[2], alkyl iodide (**RM14-RM15**) were conveniently synthesized according to **GP-A2** in gram scale.

General procedure A3 (GP-A3)

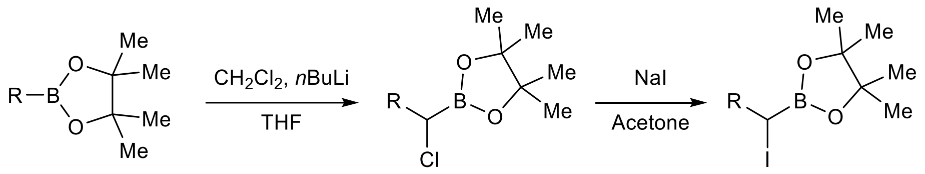

According to the reported literature[3], alkyl iodide (**RM18-RM20**) were conveniently synthesized according to **GP-A3** in gram scale.

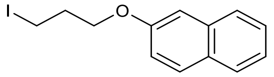

2-(3-iodopropoxy)naphthalene (**RM1**)

RM1 was obtained as white solid.

^{1}H NMR (500 MHz, Chloroform-*d*) δ 7.85 – 7.74 (m, 3H), 7.53 – 7.45 (m, 1H), 7.43 – 7.34 (m, 1H), 7.23 – 7.13 (m, 2H), 4.16 (t, *J* = 5.8 Hz, 2H), 3.43 (t, *J* = 6.8 Hz, 2H), 2.35 (tt, *J* = 6.8, 5.8 Hz, 2H).

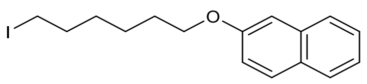

2-((6-iodohexyl)oxy)naphthalene (**RM2**)

RM2 was obtained as white solid.

^{1}H NMR (400 MHz, Chloroform-*d*) δ 7.85 – 7.73 (m, 3H), 7.52 – 7.44 (m, 1H), 7.41 – 7.33 (m, 1H), 7.22 – 7.13 (m, 2H), 4.09 (t, J = 6.4 Hz, 2H), 3.23 (t, J = 7.0 Hz, 2H), 1.94 – 1.80 (m, 4H), 1.59 – 1.46 (m, 4H).

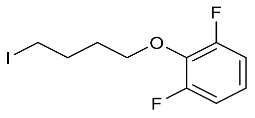

1,3-difluoro-2-(4-iodobutoxy)benzene (**RM3**)

RM3 was obtained as yellow oil.

^{1}H NMR (400 MHz, Chloroform-*d*) δ 7.02 – 6.80 (m, 3H), 4.14 (t, J = 6.0 Hz, 2H), 3.27 (t, J = 6.8 Hz, 2H), 2.13 – 2.01 (m, 2H), 1.94 – 1.77 (m, 2H).

^{13}C NMR (101 MHz, Chloroform-*d*) δ 156.21 (dd, J = 248.1, 5.5 Hz), 135.56 (t, J = 14.3 Hz), 122.83 (t, J = 9.3 Hz), 114.42 – 110.20 (m), 73.32 (t, J = 2.9 Hz), 30.79, 29.73, 6.51.

^{19}F NMR (376 MHz, Chloroform-*d*) δ 128.34 (dd, J = 7.8, 5.8 Hz).

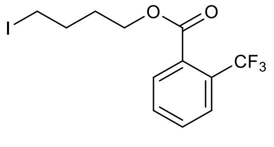

4-iodobutyl 2-(trifluoromethyl)benzoate (**RM4**)

RM4 was obtained as yellow oil.

^{1}H NMR (400 MHz, Chloroform-*d*) δ 7.84 – 7.68 (m, 2H), 7.70 – 7.53 (m, 2H), 4.36 (t, J = 6.2 Hz, 2H), 3.23 (t, J = 6.7 Hz, 2H), 2.04 – 1.80 (m, 4H).

^{13}C NMR (101 MHz, Chloroform-*d*) δ 166.93, 131.81, 131.22, 131.19, 130.22, 128.62 (q, J = 32.5 Hz), 126.71 (q, J = 5.4 Hz), 123.39 (q, J = 273.4 Hz), 64.85, 29.80, 29.39, 5.87.

^{19}F NMR (376 MHz, Chloroform-*d*) δ 59.35.

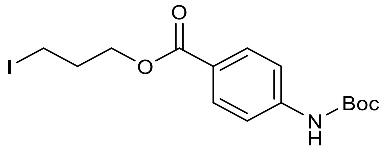

3-iodopropyl 4-((*tert*-butoxycarbonyl)amino)benzoate (**RM5**)

RM5 was obtained as white solid.

^{1}H NMR (400 MHz, Chloroform-*d*) δ 7.96 (d, *J* = 8.8 Hz, 2H), 7.44 (d, *J* = 8.8 Hz, 2H), 6.78 (s, 1H), 4.37 (t, *J* = 6.0 Hz, 2H), 3.30 (t, *J* = 6.9 Hz, 2H), 2.33 – 2.19 (m, 2H), 1.53 (s, 9H).

^{13}C NMR (101 MHz, Chloroform-*d*) δ 166.02, 152.17, 142.88, 130.91, 124.09, 117.35, 81.25, 64.35, 32.54, 28.27, 1.58.

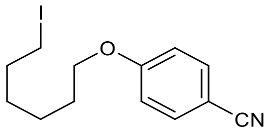

4-((6-iodohexyl)oxy)benzonitrile (**RM6**)

RM6 was obtained as white soild.

^{1}H NMR (400 MHz, Chloroform-*d*) δ 7.64 – 7.47 (m, 2H), 6.99 – 6.86 (m, 2H), 3.98 (t, *J* = 6.4 Hz, 2H), 3.18 (t, *J* = 6.9 Hz, 2H), 1.90 – 1.73 (m, 4H), 1.53 – 1.39 (m, 4H).

^{13}C NMR (101 MHz, Chloroform-*d*) δ 162.36, 133.99, 119.33, 115.20, 103.67, 68.16, 33.28, 30.16, 28.80, 24.97, 7.08.

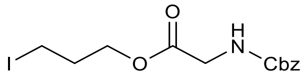

3-iodopropyl ((benzyloxy)carbonyl)glycinate (**RM7**)

RM7 was obtained as white soild.

^{1}H NMR (400 MHz, Chloroform-*d*) δ 7.40 – 7.28 (m, 5H), 5.35 (s, 1H), 5.12 (s, 2H), 4.22 (t, *J* = 6.0 Hz, 2H), 3.97 (d, *J* = 5.7 Hz, 2H), 3.19 (t, *J* = 6.8 Hz, 2H), 2.20 – 2.05 (m, 2H).

^{13}C NMR (101 MHz, Chloroform-*d*) δ 169.90, 156.28, 136.13, 128.53, 128.22, 128.11, 67.12, 64.99, 42.67, 32.04, 1.14.

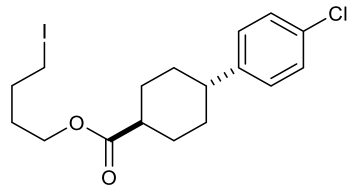

4-iodobutyl (1*r*,4*r*)-4-(4-chlorophenyl)cyclohexane-1-carboxylate (**RM8**)

RM8 was obtained as yellow oil.

^{1}H NMR (400 MHz, Chloroform-*d*) δ 7.25 (d, *J* = 8.1 Hz, 2H), 7.12 (d, *J* = 8.5 Hz, 2H), 4.11 (t, *J* = 6.3 Hz, 2H), 3.22 (t, *J* = 6.8 Hz, 2H), 2.49 (tt, *J* = 11.9, 3.4 Hz, 1H), 2.34 (tt, *J* = 12.2, 3.6 Hz, 1H), 2.15 – 2.05 (m, 2H), 1.99 – 1.86 (m, 4H), 1.82 – 1.71 (m, 2H), 1.65 – 1.52 (m, 2H), 1.50 – 1.36 (m, 2H).

^{13}C NMR (101 MHz, Chloroform-*d*) δ 175.77, 145.21, 131.62, 128.44, 128.08, 63.06, 42.93, 42.87, 33.17, 29.98, 29.57, 29.18, 5.99.

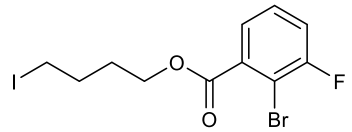

4-iodobutyl 2-bromo-3-fluorobenzoate (**RM9**)

RM9 was obtained as yellow oil.

^{1}H NMR (400 MHz, Chloroform-*d*) δ 7.55 (dt, *J* = 7.7, 1.3 Hz, 1H), 7.34 (td, *J* = 8.0, 5.1 Hz, 1H), 7.28 – 7.21 (m, 1H), 4.37 (t, *J* = 6.2 Hz, 2H), 3.24 (t, *J* = 6.7 Hz, 2H), 2.05 – 1.85 (m, 4H).

^{13}C NMR (101 MHz, Chloroform-*d*) δ 165.42 (d, *J* = 3.0 Hz), 159.47 (d, *J* = 247.4 Hz), 134.40, 128.49 (d, *J* = 8.0 Hz), 126.51 (d, *J* = 3.5 Hz), 119.13 (d, *J* = 23.5 Hz), 109.32 (d, *J* = 22.5 Hz), 64.69, 29.95, 29.46, 5.87.

^{19}F NMR (376 MHz, Chloroform-*d*) δ 102.79 (dd, *J* = 8.2, 5.2 Hz).

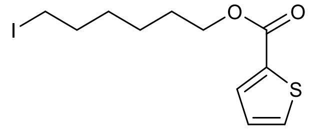

6-iodohexyl thiophene-2-carboxylate (**RM10**)

RM10 was obtained as yellow oil.

^{1}H NMR (400 MHz, Chloroform-*d*) δ 7.79 (dd, *J* = 3.7, 1.3 Hz, 1H), 7.54 (dd, *J* = 5.0, 1.3 Hz, 1H), 7.09 (dd, *J* = 5.0, 3.7 Hz, 1H), 4.29 (t, *J* = 6.6 Hz, 2H), 3.19 (t, *J* = 7.0 Hz, 2H), 1.88 – 1.72 (m, 4H), 1.46 (p, *J* = 3.1 Hz, 4H).

^{13}C NMR (101 MHz, Chloroform-*d*) δ 162.32, 133.38, 133.35, 132.30, 127.77, 65.02, 33.34, 30.15, 28.53, 25.00, 6.94.

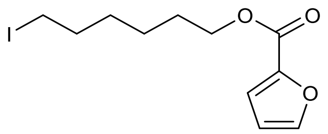

6-iodohexyl furan-2-carboxylate (**RM11**)

RM11 was obtained as yellow oil.

^{1}H NMR (400 MHz, Chloroform-*d*) δ 7.57 (dd, *J* = 1.8, 0.8 Hz, 1H), 7.16 (dd, *J* = 3.5, 0.9 Hz, 1H), 6.50 (dd, *J* = 3.5, 1.7 Hz, 1H), 4.29 (t, *J* = 6.7 Hz, 2H), 3.18 (t, *J* = 7.0 Hz, 2H), 1.87 – 1.72 (m, 4H), 1.48 – 1.40 (m, 4H).

^{13}C NMR (101 MHz, Chloroform-*d*) δ 158.79, 146.24, 144.75, 117.80, 111.81, 64.79, 33.28, 30.10, 28.49, 24.91, 6.89.

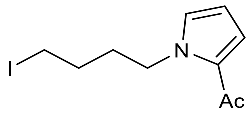

1-(1-(4-iodobutyl)-1*H*-pyrrol-2-yl)ethan-1-one (**RM12**)

RM12 was obtained as yellow oil.

^{1}H NMR (500 MHz, Chloroform-*d*) δ 6.96 (dd, *J* = 4.0, 1.7 Hz, 1H), 6.84 (dd, *J* = 2.5, 1.7 Hz, 1H), 6.13 (dd, *J* = 4.1, 2.5 Hz, 1H), 4.34 (t, *J* = 6.8 Hz, 2H), 3.16 (t, *J* = 6.7 Hz, 2H), 2.43 (s, 3H), 1.88 – 1.77 (m, 4H).

^{13}C NMR (126 MHz, Chloroform-*d*) δ 188.31, 130.11, 130.05, 120.45, 108.17, 48.59, 32.35, 30.54, 27.33, 5.85.

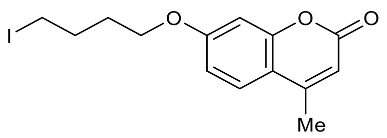

7-(4-iodobutoxy)-4-methyl-2*H*-chromen-2-one (**RM13**)

RM13 was obtained as white solid.

^{1}H NMR (400 MHz, Chloroform-*d*) δ 7.45 (d, *J* = 8.8 Hz, 1H), 6.81 (dd, *J* = 8.8, 2.5 Hz, 1H), 6.73 (d, *J* = 2.5 Hz, 1H), 6.12 – 6.04 (m, 1H), 4.01 (t, *J* = 6.0 Hz, 2H), 3.24 (t, *J* = 6.7 Hz, 2H), 2.36 (d, *J* = 1.2 Hz, 3H), 2.06 – 1.86 (m, 4H).

^{13}C NMR (101 MHz, Chloroform-*d*) δ 161.81, 161.22, 155.14, 152.58, 125.55, 113.52, 112.46, 111.85, 101.27, 67.23, 29.97, 29.87, 18.68, 6.22.

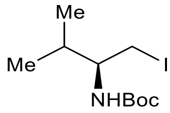

tert-butyl (*S*)-(1-iodo-3-methylbutan-2-yl)carbamate (**RM14**)

RM14 was obtained as white solid.

^{1}H NMR (400 MHz, Chloroform-*d*) δ 4.56 (d, *J* = 9.3 Hz, 1H), 3.46 – 3.27 (m, 2H), 3.17 – 3.04 (m, 1H), 1.85 – 1.69 (m, 1H), 1.45 (s, 9H), 0.96 (d, *J* = 6.8 Hz, 3H), 0.92 (d, *J* = 6.7 Hz, 3H).

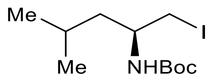

tert-butyl (*S*)-(1-iodo-4-methylpentan-2-yl)carbamate (**RM15**)

RM15 was obtained as white solid.

^{1}H NMR (500 MHz, Chloroform-*d*) δ 4.61 – 4.43 (m, 1H), 3.52 – 3.35 (m, 2H), 3.27 (dd, *J* = 10.0, 3.4 Hz, 1H), 1.68 – 1.56 (m, 1H), 1.44 (s, 9H), 1.34 (td, *J* = 7.0, 6.3, 1.7 Hz, 2H), 0.97 – 0.90 (m, 6H).

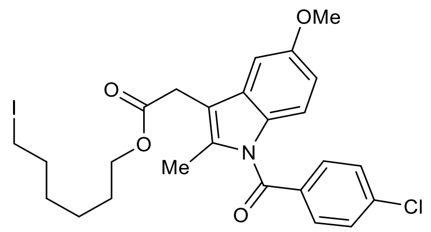

6-iodohexyl 2-(1-(4-chlorobenzoyl)-5-methoxy-2-methyl-1*H*-indol-3-yl)acetate (**RM16**)

RM16 was obtained as white solid.

^{1}H NMR (400 MHz, Chloroform-*d*) δ 7.66 (d, *J* = 8.5 Hz, 2H), 7.51 – 7.44 (m, 2H), 6.96 (d, *J* = 2.5 Hz, 1H), 6.86 (d, *J* = 9.0 Hz, 1H), 6.67 (dd, *J* = 9.0, 2.6 Hz, 1H), 4.09 (t, *J* = 6.6 Hz, 2H), 3.84 (s, 3H), 3.66 (s, 2H), 3.13 (t, *J* = 7.0 Hz, 2H), 2.39 (s, 3H), 1.75 (p, *J* = 7.0 Hz, 2H), 1.68 – 1.56 (m, 2H), 1.40 – 1.27 (m, 4H).

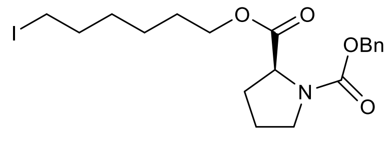

1-benzyl 2-(6-iodohexyl) (*S*)-pyrrolidine-1,2-dicarboxylate (**RM17**)

RM17 was obtained as white solid.

^{1}H NMR (400 MHz, Chloroform-*d*) δ 7.38 – 7.27 (m, 5H), 5.22 – 4.97 (m, 2H), 4.34 (ddd, *J* = 17.7, 8.6, 3.5 Hz, 1H), 4.12 (t, *J* = 6.6 Hz, 1H), 4.05 – 3.88 (m, 1H), 3.66 – 3.56 (m, 1H), 3.55 – 3.42 (m, 1H), 3.15 (dt, *J* = 10.5, 6.9 Hz, 2H), 2.30 – 2.11 (m, 1H), 2.05 – 1.85 (m, 3H), 1.84 – 1.70 (m, 2H), 1.69 – 1.58 (m, 1H), 1.55 – 1.45 (m, 1H), 1.44 – 1.30 (m, 3H), 1.28 – 1.18 (m, 1H).

^{13}C NMR (101 MHz, Chloroform-*d*) δ 172.87, 172.70, 154.84, 154.29, 136.73, 136.59, 128.46, 128.40, 127.96, 127.93, 127.84, 127.74, 66.96, 66.92, 64.95, 64.93, 59.30, 58.96, 46.95, 46.45, 33.30, 33.22, 31.00, 30.05, 30.01, 29.99, 28.38, 28.27, 24.79, 24.75, 24.33, 23.55, 7.02, 6.95.

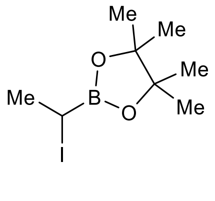

2-(1-iodoethyl)-4,4,5,5-tetramethyl-1,3,2-dioxaborolane (**RM18**)

RM18 was obtained as pale-yellow oil.

^{1}H NMR (500 MHz, Chloroform-*d*) δ 3.34 (q, J = 7.6 Hz, 1H), 1.82 (d, J = 7.6 Hz, 3H), 1.25 (d, J = 1.9 Hz, 12H).

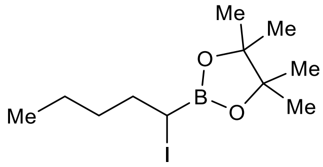

2-(1-iodopentyl)-4,4,5,5-tetramethyl-1,3,2-dioxaborolane (**RM19**)

RM19 was obtained as pale-yellow oil.

^{1}H NMR (400 MHz, Chloroform-*d*) δ 3.19 (t, *J* = 8.2 Hz, 1H), 2.00 – 1.71 (m, 2H), 1.47 – 1.18 (m, 16H), 0.88 (t, *J* = 7.0 Hz, 3H).

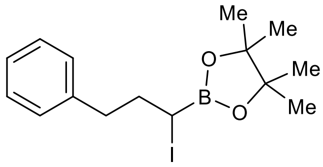

2-(1-iodo-3-phenylpropyl)-4,4,5,5-tetramethyl-1,3,2-dioxaborolane (**RM20**)

RM20 was obtained as pale-yellow oil.

^{1}H NMR (400 MHz, Chloroform-*d*) δ 7.34 – 7.26 (m, 2H), 7.23 – 7.16 (m, 3H), 3.22 (dd, *J* = 8.8, 7.2 Hz, 1H), 2.86 – 2.58 (m, 2H), 2.27 – 1.99 (m, 2H), 1.29 (d, *J* = 2.0 Hz, 12H).

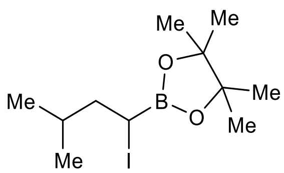

2-(1-iodo-3-methylbutyl)-4,4,5,5-tetramethyl-1,3,2-dioxaborolane (**RM21**)

RM21 was obtained as pale-yellow oil.

^{1}H NMR (400 MHz, Chloroform-*d*) δ 3.24 (dd, *J* = 9.0, 7.8 Hz, 1H), 1.90 – 1.75 (m, 1H), 1.70 – 1.48 (m, 2H), 1.23 (d, *J* = 3.1 Hz, 12H), 0.87 (d, *J* = 6.6 Hz, 3H), 0.82 (d, *J* = 6.5 Hz, 3H).

Preparation of Tertiary Enamides

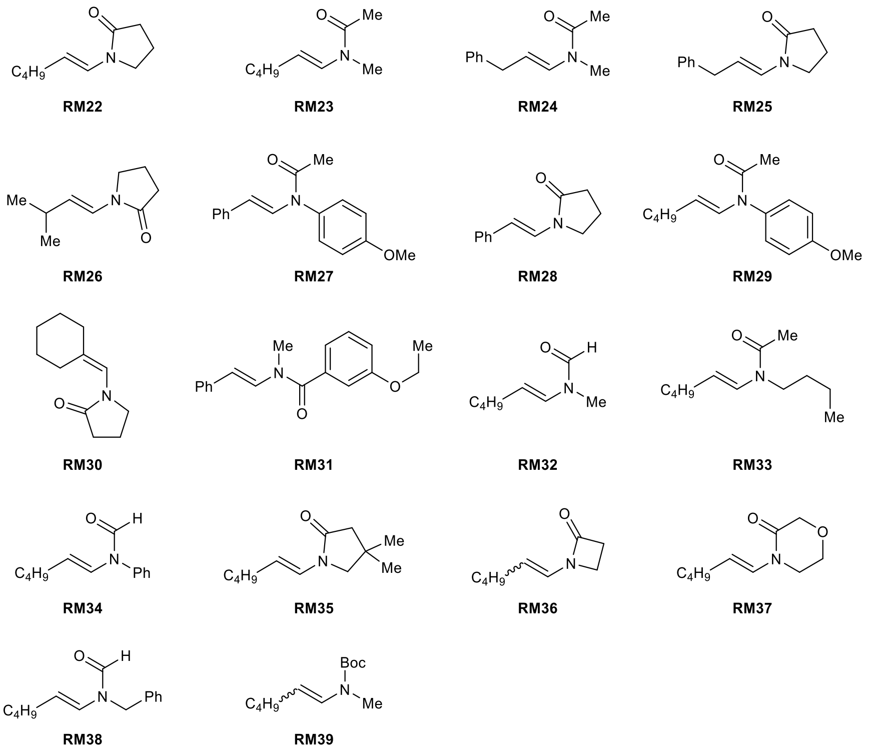

Supplementary Figure 2. List of tertiary enamides

General Procedure B1 (GP-B1)

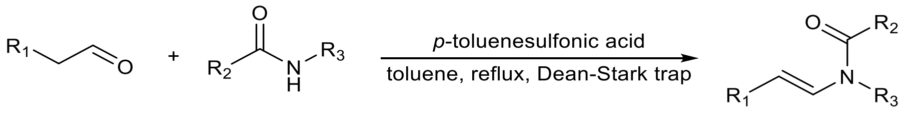

According to the reported literature[4], tertiary enamides (**RM22-RM31**) were conveniently synthesized according to **GP-B1** in gram scale.

General Procedure B2 (GP-B2)

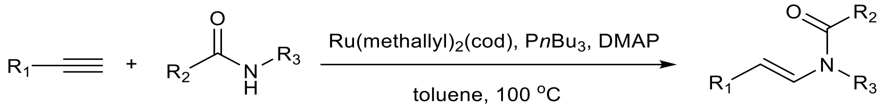

According to the reported literature[5], tertiary enamides (**RM32-RM39**) were conveniently synthesized according to **GP-B2** in gram scale.

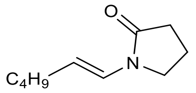

(*E*)-1-(hex-1-en-1-yl)pyrrolidin-2-one **(RM22)**

RM22 was obtained as colorless oil.

^{1}H NMR (400 MHz, Chloroform-*d*) δ 6.75 (d, *J* = 14.5 Hz, 1H), 4.84 (dt, *J* = 14.3, 7.1 Hz, 1H), 3.39 (t, *J* = 7.2 Hz, 2H), 2.36 (t, *J* = 8.9 Hz, 2H), 2.08 – 1.87 (m, 4H), 1.43 – 1.12 (m, 4H), 0.79 (t, *J* = 7.1 Hz, 3H).

^{13}C NMR (101 MHz, Chloroform-*d*) δ 172.66, 123.41, 112.40, 45.18, 32.12, 31.19, 29.62, 21.96, 17.31, 13.78.

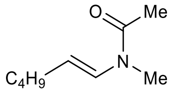

(*E*)-*N*-(hex-1-en-1-yl)-*N*-methylacetamide (**RM23**)

RM23 was obtained as colorless oil.

Mixture of rotational isomers, ratio = 29:71.

^{1}H NMR (400 MHz, Chloroform-*d*) δ 7.22 (dt, *J* = 14.5, 1.4 Hz, 0.29H), 6.52 (dt, *J* = 13.8, 1.3 Hz, 0.71H), 5.08 – 4.83 (m, 1H), 3.01 (s, 0.92H), 2.98 (s, 2.15H), 2.12 (s, 2.15H), 2.12 (s, 0.98H), 2.04 – 1.95 (m, 2H), 1.35 – 1.20 (m, 4H), 0.88 – 0.79 (m, 3H).

^{13}C NMR (101 MHz, Chloroform-*d*) δ 169.11, 168.84, 128.58, 126.68, 112.54, 111.67, 33.04, 32.41, 32.35, 30.05, 29.88, 29.60, 22.53, 22.07, 21.90, 13.86.

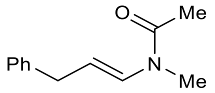

(*E*)-*N*-methyl-*N*-(3-phenylprop-1-en-1-yl)acetamide (**RM24**)

RM24 was obtained as colorless oil.

^{1}H NMR (400 MHz, Chloroform-*d*) δ 7.46 (dt, J = 14.4, 1.4 Hz, 0.34H), 7.35 – 7.13

(m, 5H), 6.67 (dt, J = 13.7, 1.4 Hz, 0.66H), 5.23 – 5.00 (m, 1H), 3.39 (dd, J = 7.2, 1.4 Hz, 2H), 3.05 (s, 3H), 2.17 (s, 3H).

^{13}C NMR (101 MHz, Chloroform-*d*) δ 169.02, 168.91, 140.99, 140.46, 129.82, 128.50, 128.40, 128.34, 128.30, 127.70, 126.24, 126.06, 110.41, 110.02, 36.65, 36.54, 33.02, 29.48, 22.58, 21.92.

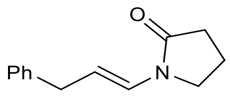

(*E*)-1-(3-phenylprop-1-en-1-yl)pyrrolidin-2-one (**RM25**)

RM25 was obtained as pale yellow oil.

^{1}H NMR (400 MHz, Chloroform-*d*) δ 7.25 – 7.09 (m, 5H), 6.95 (d, *J* = 14.4 Hz, 1H), 5.02 (dt, *J* = 14.5, 7.3 Hz, 1H), 3.39 (t, *J* = 7.3 Hz, 2H), 3.34 (d, *J* = 7.3 Hz, 2H), 2.38 (t, *J* = 7.8 Hz, 2H), 1.97 (p, *J* = 7.7 Hz, 2H).

^{13}C NMR (101 MHz, Chloroform-*d*) δ 172.76, 140.39, 128.17, 128.08, 125.86, 124.24, 110.75, 44.94, 36.20, 30.91, 17.08.

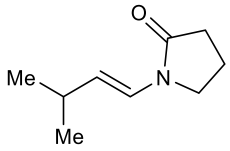

(*E*)-1-(3-methylbut-1-en-1-yl)pyrrolidin-2-one (**RM26**)

RM26 was obtained as pale yellow oil.

^{1}H NMR (400 MHz, Chloroform-*d*) δ 6.68 (d, *J* = 14.6 Hz, 1H), 4.77 (dd, *J* = 14.6, 7.1 Hz, 1H), 3.33 (t, *J* = 7.2 Hz, 2H), 2.30 (t, *J* = 8.0 Hz, 2H), 2.20 (hd, *J* = 6.8, 1.3 Hz, 1H), 1.93 (p, *J* = 7.6 Hz, 2H), 0.87 (s, 3H), 0.85 (s, 3H).

^{13}C NMR (101 MHz, Chloroform-*d*) δ 172.71, 121.32, 119.55, 45.05, 31.09, 29.01, 22.88, 17.17.

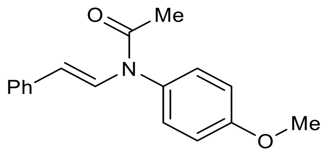

(*E*)-*N*-(4-methoxyphenyl)-*N*-styrylacetamide (**RM27**)

RM27 was obtained as white solid.

^{1}H NMR (400 MHz, Chloroform-*d*) δ 8.27 (d, *J* = 14.8 Hz, 1H), 7.33 – 7.22 (m, 4H), 7.19 – 7.11 (m, 3H), 7.09 – 7.00 (m, 2H), 5.35 (d, *J* = 14.8 Hz, 1H), 3.89 (s, 3H), 1.93 (s, 3H).

^{13}C NMR (101 MHz, Chloroform-*d*) δ 169.42, 159.73, 136.62, 132.14, 129.96, 128.71, 128.63, 126.47, 125.77, 115.35, 113.71, 55.62, 23.47.

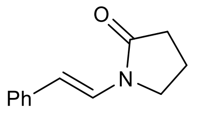

(*E*)-1-styrylpyrrolidin-2-one (**RM28**)

RM28 was obtained as white solid.

^{1}H NMR (400 MHz, Chloroform-*d*) δ 7.62 (d, *J* = 14.9 Hz, 1H), 7.39 – 7.33 (m, 2H), 7.29 (dd, *J* = 8.6, 6.9 Hz, 2H), 7.20 – 7.14 (m, 1H), 5.87 (d, *J* = 14.8 Hz, 1H), 3.76 – 3.54 (m, 2H), 2.53 (dd, *J* = 8.7, 7.6 Hz, 2H), 2.28 – 2.05 (m, 2H).

^{13}C NMR (101 MHz, Chloroform-*d*) δ 173.51, 136.44, 128.76, 126.65, 125.71, 123.64, 111.86, 45.32, 31.35, 17.52.

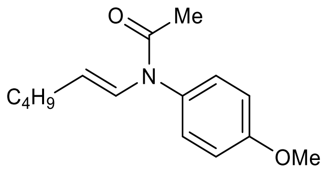

(*E*)-*N*-(hex-1-en-1-yl)-*N*-(4-methoxyphenyl)acetamide (**RM29**)

RM29 was obtained as brown oil.

^{1}H NMR (400 MHz, Chloroform-*d*) δ 7.36 (d, *J* = 14.3 Hz, 1H), 6.98 (d, *J* = 8.8 Hz, 2H), 6.88 (d, *J* = 8.9 Hz, 2H), 4.31 (dt, *J* = 14.3, 7.2 Hz, 1H), 3.75 (s, 3H), 1.92 – 1.84 (m, 2H), 1.74 (s, 3H), 1.23 – 1.05 (m, 4H), 0.74 (t, *J* = 7.0 Hz, 3H).

^{13}C NMR (101 MHz, Chloroform-*d*) δ 168.55, 159.23, 132.62, 129.68, 128.23, 114.92, 114.31, 55.31, 31.96, 29.52, 23.06, 21.97, 13.78.

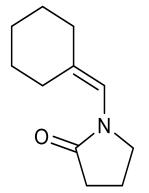

1-(cyclohexylidenemethyl)pyrrolidin-2-one (**RM30**)

RM30 was obtained as colorless oil.

^{1}H NMR (400 MHz, Chloroform-*d*) δ 5.78 (s, 1H), 3.47 (t, *J* = 7.0 Hz, 2H), 2.28 (t, *J* = 8.1 Hz, 2H), 2.14 – 1.86 (m, 6H), 1.68 – 1.31 (m, 6H).

^{13}C NMR (101 MHz, Chloroform-*d*) δ 174.71, 135.35, 116.08, 49.59, 33.77, 30.57, 28.70, 27.94, 27.10, 26.20, 18.63.

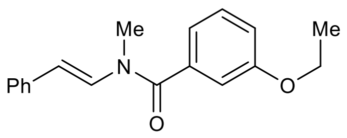

(*E*)-3-ethoxy-*N*-methyl-N-styrylbenzamide (**RM31**)

RM31 was obtained as colorless oil.

^{1}H NMR (400 MHz, Chloroform-*d*) δ 7.38 – 7.30 (m, 2H), 7.27 – 7.20 (m, 3H), 7.17 – 7.09 (m, 2H), 7.07 – 6.97 (m, 3H), 6.01 (d, *J* = 14.5 Hz, 1H), 4.04 (qd, *J* = 7.0, 1.4 Hz, 2H), 3.33 (s, 3H), 1.40 (td, *J* = 7.0, 1.4 Hz, 3H).

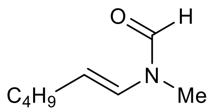

(*E*)-*N*-(hex-1-en-1-yl)-*N*-methylformamide (**RM32**)

RM32 was obtained as brown oil.

Mixture of rotational isomers, ratio = 30:70.

^{1}H NMR (400 MHz, Chloroform-*d*) δ 8.16 (s, 0.69H), 7.94 (s, 0.30H), 7.00 (dt, *J* = 14.3, 1.5 Hz, 0.30H), 6.36 (dt, *J* = 13.9, 1.4 Hz, 0.70H), 5.07 – 4.93 (m, 1H), 2.94 (s, 0.93H), 2.89 (s, 2.09H), 2.08 – 1.90 (m, 2H), 1.41 – 1.13 (m, 4H), 0.91 – 0.69 (m, 3H).

^{13}C NMR (101 MHz, Chloroform-*d*) δ 161.99, 160.62, 128.06, 124.11, 113.13, 111.39, 32.89, 32.13, 32.05, 29.76, 29.62, 27.32, 21.98, 21.97, 13.77.

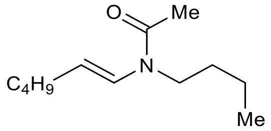

(*E*)-*N*-butyl-*N*-(hex-1-en-1-yl)acetamide (**RM33**)

RM33 was obtained as brown oil.

Mixture of rotational isomers, ratio = 28:72.

^{1}H NMR (400 MHz, Chloroform-*d*) δ 7.09 (d, *J* = 14.7 Hz, 0.28H), 6.36 (d, *J* = 13.9 Hz, 0.72H), 5.01 – 4.92 (m, 1H), 3.59 – 3.47 (m, 1.45H), 3.42 – 3.33 (m, 0.57H), 2.09 (s, 3H), 2.04 – 1.95 (m, 2H), 1.56 – 1.38 (m, 2H), 1.34 – 1.20 (m, 6H), 0.92 – 0.79 (m, 6H).

^{13}C NMR (101 MHz, Chloroform-*d*) δ 168.71, 168.52, 127.55, 125.26, 113.07, 111.35, 45.57, 42.59, 32.41, 32.31, 30.19, 30.04, 29.68, 28.84, 22.21, 22.10, 22.07, 20.19, 20.14, 13.87, 13.82, 13.75.

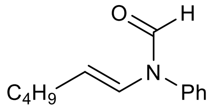

(*E*)-*N*-(hex-1-en-1-yl)-*N*-phenylformamide (**RM34**)

RM34 was obtained as brown oil, > 20:1 *E:Z*.

Mixture of rotational isomers, ratio = 16:80.

^{1}H NMR (400 MHz, Chloroform-*d*) δ 8.52 (s, 0.16H), 8.44 – 8.34 (m, 0.04H), 8.14 (s, 0.80H), 7.51 – 7.34 (m, 3H), 7.27 – 7.15 (m, 2.80H), 6.53 (d, J = 13.8 Hz, 0.16H), 6.44 (d, J = 8.8 Hz, 0.04H), 5.04 – 4.89 (m, 1H), 2.13 – 1.94 (m, 2H), 1.40 – 1.20 (m, 4H), 0.96 – 0.80 (m, 3H).

^{13}C NMR (101 MHz, Chloroform-*d*) δ 161.42, 160.22, 138.40, 136.85, 129.62, 129.32, 128.34, 128.25, 127.68, 127.55, 126.94, 124.95, 117.15, 116.65, 31.65, 29.59, 22.00, 13.77.

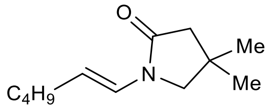

(*E*)-1-(hex-1-en-1-yl)-4,4-dimethylpyrrolidin-2-one (**RM35**)

RM35 was obtained as pale yellow oil.

^{1}H NMR (400 MHz, Chloroform-*d*) δ 6.77 (d, *J* = 14.5 Hz, 1H), 4.78 (dt, *J* = 14.3, 7.1 Hz, 1H), 3.10 (s, 2H), 2.19 (s, 2H), 2.03 – 1.87 (m, 2H), 1.37 – 1.16 (m, 4H), 1.07 (s, 6H), 0.78 (t, *J* = 7.1 Hz, 3H).

^{13}C NMR (101 MHz, Chloroform-*d*) δ 171.86, 123.43, 112.08, 58.34, 46.51, 32.37, 32.11, 29.58, 27.96, 21.97, 13.81.

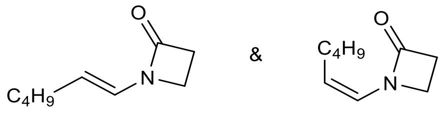

1-(hex-1-en-1-yl)azetidin-2-one (**RM36**)

RM36 was obtained as pale yellow oil, 55:45 *Z:E*.

^{1}H NMR (400 MHz, Chloroform-*d*) δ 6.42 (d, *J* = 14.2 Hz, 0.45H), 6.19 (d, *J* = 9.4 Hz, 0.55H), 4.95 (dt, *J* = 14.3, 7.1 Hz, 0.46H), 4.61 (dt, *J* = 9.5, 7.8 Hz, 0.55H), 3.62 (t, *J* = 4.5 Hz, 1.11H), 3.30 (t, *J* = 4.4 Hz, 0.92H), 2.97 (t, *J* = 4.5 Hz, 1.11H), 2.89 (t, *J* = 4.4 Hz, 0.92H), 2.17 – 2.05 (m, 1.16H), 2.01 – 1.86 (m, 0.97H), 1.37 – 1.20 (m, 4H), 0.92 – 0.71 (m, 3H).

^{13}C NMR (101 MHz, Chloroform-*d*) δ 165.23, 163.67, 121.57, 119.44, 113.43, 111.91, 42.06, 38.05, 37.76, 35.76, 32.34, 31.83, 29.05, 26.24, 22.18, 21.99, 13.86, 13.81.

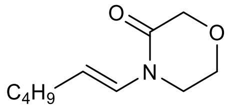

(*E*)-4-(hex-1-en-1-yl)morpholin-3-one (**RM37**)

RM37 was obtained as pale yellow oil.

^{1}H NMR (400 MHz, Chloroform-*d*) δ 7.18 (d, *J* = 14.6 Hz, 1H), 4.97 (dt, *J* = 14.4, 7.2 Hz, 1H), 4.10 (s, 2H), 3.84 (t, *J* = 5.2 Hz, 2H), 3.35 (t, *J* = 4.9 Hz, 2H), 2.09 – 1.93 (m, 2H), 1.34 – 1.17 (m, 4H), 0.79 (t, *J* = 7.1 Hz, 3H).

^{13}C NMR (101 MHz, Chloroform-*d*) δ 164.59, 124.81, 112.22, 67.94, 63.29, 43.50, 32.01, 29.60, 21.92, 13.72.

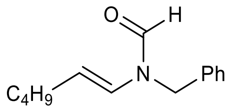

(*E*)-*N*-benzyl-*N*-(hex-1-en-1-yl)formamide (**RM38**)

RM38 was obtained as pale yellow oil.

Mixture of rotational isomers, ratio = 31:69.

^{1}H NMR (400 MHz, Chloroform-*d*) δ 8.41 (s, 0.68H), 8.21 (s, 0.30H), 7.39 – 7.14 (m, 5H), 7.02 (d, *J* = 14.6 Hz, 0.31H), 6.33 (d, *J* = 14.1 Hz, 0.69H), 5.10 – 4.95 (m, 1H), 4.74 (s, 1.38H), 4.61 (s, 0.60H), 2.03 – 1.78 (m, 2H), 1.33 – 1.07 (m, 4H), 0.89 – 0.70 (m, 3H).

^{13}C NMR (101 MHz, Chloroform-*d*) δ 162.21, 161.30, 136.06, 135.97, 128.70, 128.49, 127.56, 127.18, 126.99, 126.65, 126.13, 122.71, 115.25, 113.80, 49.91, 44.68, 31.89, 31.80, 29.83, 29.70, 21.88, 21.85, 13.80, 13.79.

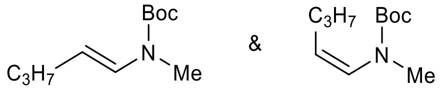

tert-butyl methyl(pent-1-en-1-yl)carbamate (**RM39**)

RM39 was obtained as pale yellow oil, 41:59 *Z:E*.

^{1}H NMR (500 MHz, Chloroform-*d*) δ 6.97 (d, *J* = 14.3 Hz, 0.25H), 6.79 (d, *J* = 14.2 Hz, 0.34H), 6.26 – 5.97 (m, 0.41H), 4.89 – 4.79 (m, 0.41H), 4.77 – 4.68 (m, 0.59H), 3.00 (s, 1.24H), 2.96 (s, 1.75H), 2.24 – 1.84 (m, 2H), 1.46 (s, 5.36H), 1.44 (s, 3.61H), 1.41 – 1.30 (m, 2H), 0.88 (m, 3H).

Preparation of Secondary Enamides and Enecarbamates

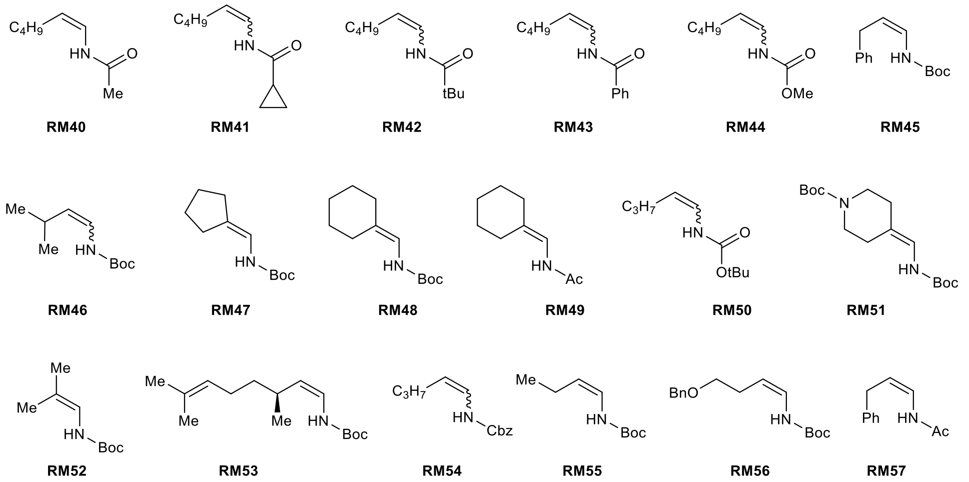

Supplementary Figure 3. List of secondary enamides and enecarbamates

General procedure C1 (GP-C1)

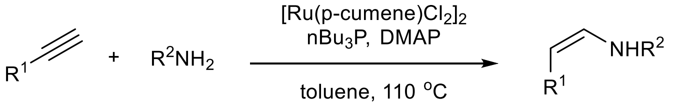

According to the reported literatures[6], secondary enamides (**RM40-RM42**) were conveniently synthesized according to **GP-C1** in gram scale.

General procedure C2 (GP-C2)

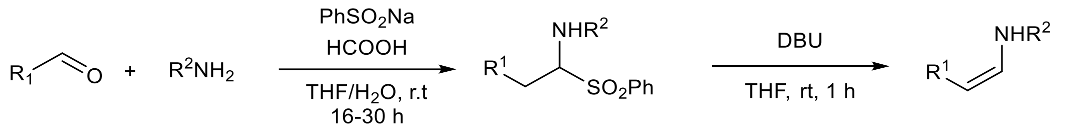

According to the reported literature[7], secondary enamides and enecarbamates (**RM43-RM57**) were conveniently synthesized according to **GP-C2** in gram scale.

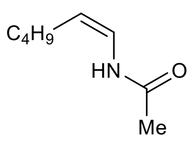

(*Z*)-*N*-(hex-1-en-1-yl)acetamide (**RM40**)

RM40 was obtained as yellow oil.

^{1}H NMR (500 MHz, Chloroform-*d*) δ 7.42 – 7.16 (m, 1H), 6.72 – 6.60 (m, 1H), 4.69 (dt, *J* = 9.2, 7.4 Hz, 1H), 2.05 (s, 3H), 2.01 – 1.95 (m, 2H), 1.40 – 1.26 (m, 4H), 0.88 (t, *J* = 7.1 Hz, 3H).

^{13}C NMR (126 MHz, Chloroform-*d*) δ 167.46, 120.78, 111.35, 31.51, 25.44, 23.32, 22.32, 13.92.

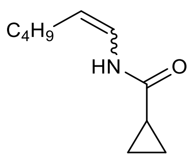

N-(hex-1-en-1-yl)cyclopropanecarboxamide (**RM41**)

RM41 was obtained as yellow oil, 93:7 *Z:E.*

^{1}H NMR (400 MHz, Chloroform-*d*) δ 7.73 – 7.52 (m, 1H), 6.66 (ddt, *J* = 10.8, 9.0, 1.7 Hz, 1H), 5.10 (dt, *J* = 14.3, 7.2 Hz, 0.07H), 4.64 (dt, *J* = 9.1, 7.4 Hz, 0.93H), 2.00 (qd, *J* = 7.2, 1.8 Hz, 2H), 1.48 (tt, *J* = 7.9, 4.5 Hz, 1H), 1.40 – 1.24 (m, 4H), 1.04 – 0.94 (m, 2H), 0.92 – 0.82 (m, 3H), 0.82 – 0.69 (m, 2H).

^{13}C NMR (101 MHz, Chloroform-*d*) δ 171.46, 120.91, 110.85, 31.60, 25.45, 22.34, 14.68, 13.97, 7.86.

HRMS (ESI) calcd for $C_{10}H_{18}NO^{+}$ [$(M+H)^{+}$] 168.1383, found 168.1380.

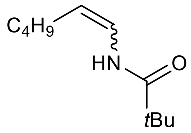

N-(hex-1-en-1-yl)pivalamide (**RM42**)

RM42 was obtained as yellow oil, 93:7 *Z:E.*

^{1}H NMR (400 MHz, Chloroform-*d*) δ 7.14 (s, 1H), 6.69 (ddt, *J* = 10.7, 8.9, 1.7 Hz, 1H), 5.14 (dt, *J* = 14.3, 7.1 Hz, 0.07H), 4.72 (dtd, *J* = 8.1, 7.3, 0.7 Hz, 0.93H), 1.97 (qd, *J* = 7.2, 1.7 Hz, 2H), 1.41 – 1.29 (m, 4H), 1.22 (s, 9H), 0.90 (t, *J* = 7.1 Hz, 3H).

The NMR data are consistent with the reported literature[8].

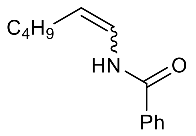

N-(hex-1-en-1-yl)benzamide (**RM43**)

RM43 was obtained as yellow oil, 76:24 *Z:E.*

^{1}H NMR (400 MHz, Chloroform-*d*) δ 7.96 (d, *J* = 10.4 Hz, 0.24H), 7.84 – 7.74 (m, 2H), 7.68 (d, *J* = 10.3 Hz, 0.76H), 7.56 – 7.39 (m, 3H), 6.99 – 6.85 (m, 1H), 5.32 (dt, *J* = 14.3, 7.2 Hz, 0.24H), 4.86 (dt, *J* = 9.0, 7.4 Hz, 0.76H), 2.09 (qd, *J* = 7.3, 1.8 Hz, 2H), 1.47 – 1.30 (m, 4H), 0.95 – 0.85 (m, 3H).

The NMR data are consistent with the reported literature[9].

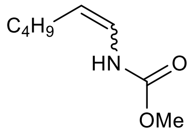

methyl hex-1-en-1-ylcarbamate (**RM44**)

RM44 was obtained as yellow oil, 69:31 *Z:E.*

^{1}H NMR (400 MHz, Chloroform-*d*) δ 6.62 – 6.13 (m, 2H), 4.98 (dt, *J* = 14.2, 7.2 Hz, 0.31H), 4.59 (dq, *J* = 11.6, 7.3, 5.3 Hz, 0.69H), 3.76 – 3.59 (m, 3H), 2.18 – 1.78 (m, 2H), 1.44 – 1.08 (m, 4H), 0.87 (t, *J* = 6.8 Hz, 3H).

^{13}C NMR (101 MHz, Chloroform-*d*) δ 154.33, 123.43, 121.94, 110.99, 109.31, 52.40, 32.14, 31.48, 29.27, 25.12, 22.29, 22.06, 13.89.

HRMS (ESI) calcd for $C_8H_{16}NO_2^+$ [$(M+H)^+$] 158.1176, found 158.1173.

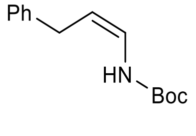

tert-butyl (*Z*)-(3-phenylprop-1-en-1-yl)carbamate (**RM45**)

RM2 was obtained as white solid.

^{1}H NMR (500 MHz, Chloroform-*d*) δ 7.34 – 7.27 (m, 2H), 7.25 – 7.17 (m, 3H), 6.57 (t, *J* = 10.2 Hz, 1H), 6.25 (d, *J* = 11.3 Hz, 1H), 4.81 (q, *J* = 8.0 Hz, 1H), 3.33 (dd, *J* = 7.4, 1.6 Hz, 2H), 1.48 (s, 9H).

The NMR data are consistent with the reported literature[7].

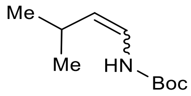

tert-butyl (3-methylbut-1-en-1-yl)carbamate (**RM46**)

RM46 was obtained as white solid, 73:27 *Z:E.*

^{1}H NMR (500 MHz, Chloroform-*d*) δ 6.46 – 6.26 (m, 1H), 6.18 (d, *J* = 11.8 Hz, 1H), 4.94 (dd, *J* = 14.2, 6.8 Hz, 0.27H), 4.44 (t, *J* = 9.2 Hz, 0.73H), 2.46 – 2.22 (m, 1H), 1.46 (m, 9H), 0.99 (dd, *J* = 6.7, 5.0 Hz, 6H).

The NMR data are consistent with the reported literature[10].

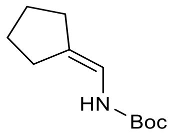

tert-butyl (cyclopentylidenemethyl)carbamate (**RM47**)

RM47 was obtained as white solid.

^{1}H NMR (400 MHz, Chloroform-*d*) δ 6.36 (dt, *J* = 10.6, 2.3 Hz, 1H), 5.83 (d, *J* = 10.6 Hz, 1H), 2.31 – 2.20 (m, 2H), 2.16 – 2.06 (m, 2H), 1.74 – 1.57 (m, 4H), 1.47 (s, 9H).

^{13}C NMR (101 MHz, Chloroform-*d*) δ 152.82, 122.98, 114.43, 79.98, 31.11, 28.32, 27.35, 26.81, 26.31.

HRMS (ESI) calcd for $C_{11}H_{20}NO_2^+$ [(M+H)$^+$] 198.1489, found 198.1487.

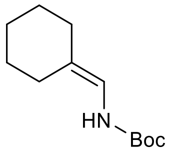

tert-butyl (cyclohexylidenemethyl)carbamate (**RM48**)

RM48 was obtained as white solid.

^{1}H NMR (400 MHz, Chloroform-*d*) δ 6.18 (d, *J* = 10.6 Hz, 1H), 6.02 (d, *J* = 11.1 Hz, 1H), 2.08 – 1.93 (m, 5H), 1.53 – 1.48 (m, 5H), 1.43 (s, 9H).

^{13}C NMR (101 MHz, Chloroform-*d*) δ 153.19, 120.01, 114.79, 79.93, 33.42, 28.32,

28.16, 27.20, 26.91, 26.65.

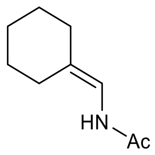

N-(cyclohexylidenemethyl)acetamide (**RM49**)

RM49 was obtained as white solid.

^{1}H NMR (400 MHz, Chloroform-*d*) δ 7.53 – 7.32 (m, 1H), 6.43 (dt, *J* = 10.3, 1.3 Hz, 1H), 2.10 – 2.02 (m, 4H), 2.01 (s, 3H), 1.52 – 1.45 (m, 6H).

The NMR data are consistent with the reported literature[11].

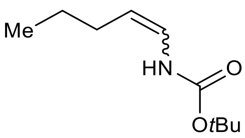

tert-butyl pent-1-en-1-ylcarbamate (**RM50**)

RM50 was obtained as white solid, 78:22 *Z:E.*

^{1}H NMR (400 MHz, Chloroform-*d*) δ 6.49 – 6.27 (m, 1H), 6.16 (d, *J* = 11.5 Hz, 1H), 4.93 (dt, *J* = 14.2, 7.2 Hz, 0.22H), 4.57 (q, *J* = 7.8 Hz, 0.78H), 1.94 (dqd, *J* = 14.6, 7.3, 1.4 Hz, 2H), 1.55 – 1.34 (m, 11H), 0.97 – 0.85 (m, 3H).

The NMR data are consistent with the reported literature[12].

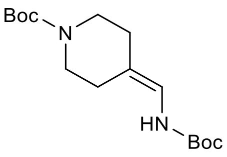

tert-butyl 4-(((*tert*-butoxycarbonyl)amino)methylene)piperidine-1-carboxylate (**RM51**)

RM51 was obtained as white solid.

^{1}H NMR (500 MHz, Chloroform-*d*) δ 6.33 (d, *J* = 10.8 Hz, 1H), 6.02 (d, *J* = 11.1 Hz, 1H), 3.38 (t, *J* = 5.7 Hz, 4H), 2.17 – 2.02 (m, 4H), 1.45 (s, 18H).

^{13}C NMR (101 MHz, Chloroform-*d*) δ 154.71, 153.11, 117.12, 115.08, 80.26, 79.53, 45.81, 43.49, 32.29, 28.43, 28.27, 26.74.

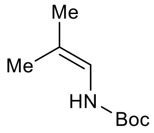

tert-butyl (2-methylprop-1-en-1-yl)carbamate (**RM52**)

RM52 was obtained as white solid.

^{1}H NMR (400 MHz, Chloroform-*d*) δ 6.29 – 6.13 (m, 1H), 5.95 (d, *J* = 10.6 Hz, 1H), 1.65 (d, *J* = 1.5 Hz, 3H), 1.54 (d, *J* = 1.4 Hz, 3H), 1.44 (s, 9H).

The NMR data are consistent with the reported literature[10].

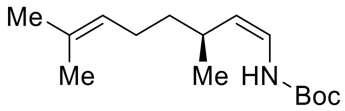

tert-butyl (*S,Z*)-(3,7-dimethylocta-1,6-dien-1-yl)carbamate (**RM53**)

RM53 was obtained as colorless oil.

^{1}H NMR (400 MHz, Chloroform-*d*) δ 6.44 – 6.29 (m, 1H), 6.16 (d, *J* = 11.3 Hz, 1H), 5.14 – 5.01 (m, 1H), 4.33 (t, *J* = 9.5 Hz, 1H), 2.25 – 2.14 (m, 1H), 2.01 – 1.91 (m, 2H), 1.73 – 1.67 (m, 3H), 1.58 (s, 3H), 1.46 (s, 9H), 1.42 – 1.34 (m, 1H), 1.30 – 1.21 (m, 1H), 0.97 (d, *J* = 6.6 Hz, 3H).

^{13}C NMR (101 MHz, Chloroform-*d*) δ 152.93, 132.40, 124.84, 121.25, 114.48, 80.21, 37.48, 29.91, 28.33, 25.71, 25.63, 21.46, 17.79.

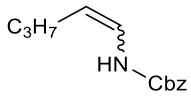

benzyl pent-1-en-1-ylcarbamate (**RM54**)

RM54 was obtained as colorless oil, 74:26 *Z:E.*

^{1}H NMR (400 MHz, Chloroform-*d*) δ 7.48 – 7.28 (m, 5H), 6.55 – 6.34 (m, 2H), 5.24 – 5.09 (m, 2H), 5.00 (dt, *J* = 14.4, 7.2 Hz, 0.26H), 4.64 (q, *J* = 7.6 Hz, 0.74H), 1.94 (dq, *J* = 22.5, 7.3 Hz, 2H), 1.40 (dp, *J* = 14.5, 7.3 Hz, 2H), 0.91 (dt, *J* = 10.0, 7.3 Hz, 3H).

The NMR data are consistent with the reported literature[13].

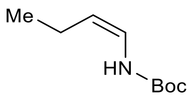

tert-butyl (*Z*)-but-1-en-1-ylcarbamate (**RM55**)

RM55 was obtained as white solid.

^{1}H NMR (500 MHz, Chloroform-*d*) δ 6.37 (t, *J* = 10.0 Hz, 1H), 6.12 (s, 1H), 4.68 – 4.42 (m, 1H), 2.07 – 1.90 (m, 2H), 1.47 (s, 9H), 1.08 – 0.96 (m, 3H).

The NMR data are consistent with the reported literature[7].

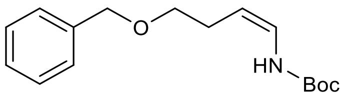

tert-butyl (*Z*)-(4-(benzyloxy)but-1-en-1-yl)carbamate (**RM56**)

RM56 was obtained as colorless oil.

^{1}H NMR (500 MHz, Chloroform-*d*) δ 7.38 – 7.27 (m, 5H), 6.99 (d, *J* = 10.8 Hz, 1H), 6.51 (t, *J* = 9.8 Hz, 1H), 4.67 (q, *J* = 8.0 Hz, 1H), 4.53 (s, 2H), 3.54 (t, *J* = 6.1 Hz, 2H), 2.32 – 2.20 (m, 2H), 1.43 (s, 9H).

^{13}C NMR (126 MHz, Chloroform-*d*) δ 153.15, 138.12, 128.42, 127.68, 127.65, 124.91, 104.63, 79.99, 73.30, 70.57, 28.29, 26.52.

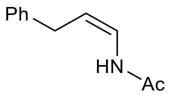

(*Z*)-*N*-(3-phenylprop-1-en-1-yl)acetamide (**RM57**)

RM57 was obtained as colorless oil.

^{1}H NMR (500 MHz, Chloroform-*d*) δ 7.34 – 7.30 (m, 2H), 7.24 – 7.20 (m, 3H), 7.05 (s, 1H), 6.83 (ddt, *J* = 10.8, 9.1, 1.7 Hz, 1H), 4.95 (dt, *J* = 8.9, 7.4 Hz, 1H), 3.39 (dt, *J* = 7.4, 1.1 Hz, 2H), 1.99 (s, 3H).

Reductive Hydroalkylation of Tertiary Enamides

Supplementary Table 1. Optimization of the Reaction Conditions for Tertiary Enamides

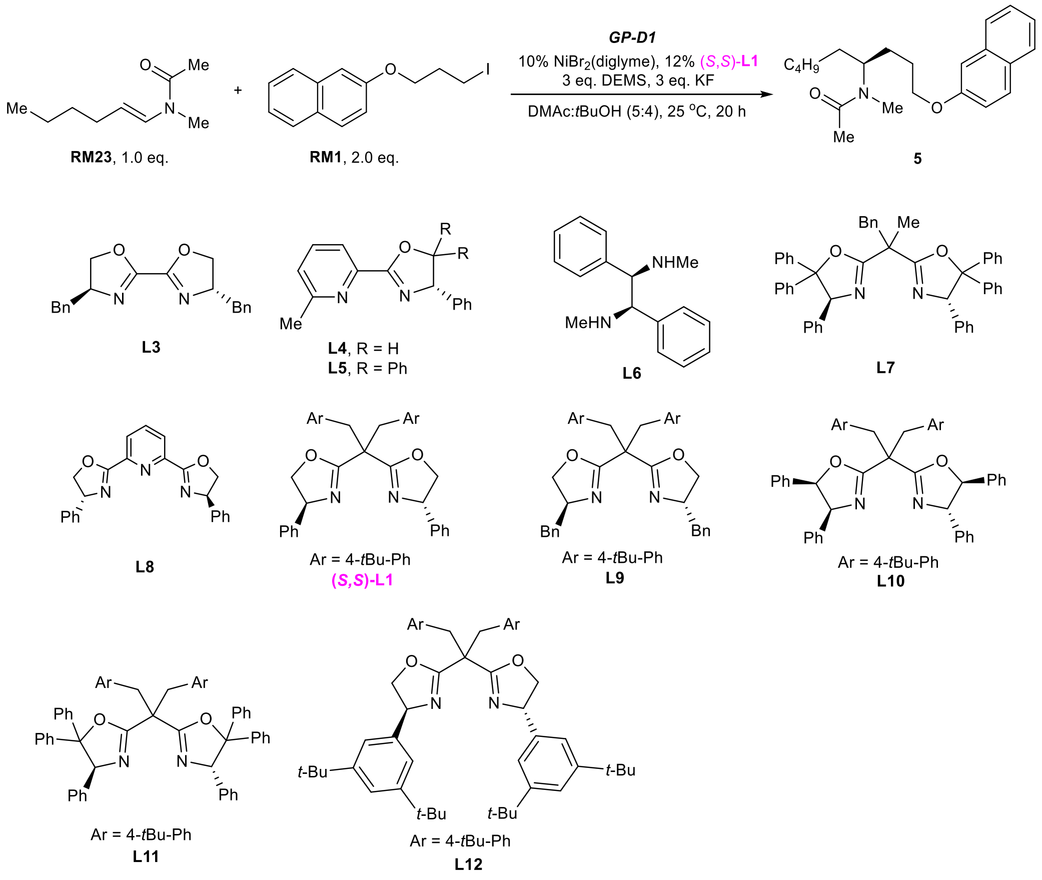

entry	nickel source	ligand	silane	base	solvent	yield of **5** (%)	e.e. of **5** (%)
1	NiBr$_2$(diglyme)	(*S*,*S*)-**L1**	DEMS	KF	DMAc/*t*BuOH (5:4)	**95 (90**[b]**)**	**92**
2	NiBr$_2$(diglyme)	**L3**	DEMS	KF	DMAc/*t*BuOH (5:4)	9	6
3	NiBr$_2$(diglyme)	**L4**	DEMS	KF	DMAc/*t*BuOH (5:4)	5	39
4	NiBr$_2$(diglyme)	**L5**	DEMS	KF	DMAc/*t*BuOH (5:4)	19	70
5	NiBr$_2$(diglyme)	**L6**	DEMS	KF	DMAc/*t*BuOH (5:4)	trace	N.D.
6	NiBr$_2$(diglyme)	**L7**	DEMS	KF	DMAc/*t*BuOH (5:4)	4	30
7	NiBr$_2$(diglyme)	**L8**	DEMS	KF	DMAc/*t*BuOH (5:4)	trace	N.D.
8	NiBr$_2$(diglyme)	**L9**	DEMS	KF	DMAc/*t*BuOH (5:4)	trace	N.D.
9	NiBr$_2$(diglyme)	**L10**	DEMS	KF	DMAc/*t*BuOH (5:4)	27	87

10	$NiBr_2$(diglyme)	**L11**	DEMS	KF	DMAc/*t*BuOH (5:4)	85	84
11	$NiBr_2$(diglyme)	**L12**	DEMS	KF	DMAc/*t*BuOH (5:4)	30	83
12	$NiBr_2$(diglyme)	(*S*,*S*)-**L1**	$(EtO)_3SiH$	KF	DMAc/*t*BuOH (5:4)	75	90
13	$NiBr_2$(diglyme)	(*S*,*S*)-**L1**	$MeEt_2SiH$	KF	DMAc/*t*BuOH (5:4)	trace	N.D.
14	$NiBr_2$(diglyme)	(*S*,*S*)-**L1**	DEMS	$K_3PO_4(H_2O)$	DMAc/*t*BuOH (5:4)	14	85
15	$NiBr_2$(diglyme)	(*S*,*S*)-**L1**	DEMS	K_3PO_4	DMAc/*t*BuOH (5:4)	37	87
16	$NiBr_2$(diglyme)	(*S*,*S*)-**L1**	DEMS	$KHCO_3$	DMAc/*t*BuOH (5:4)	24	85
17	$NiBr_2$(diglyme)	(*S*,*S*)-**L1**	DEMS	Na_2CO_3	DMAc/*t*BuOH (5:4)	30	87
18	$NiBr_2$(diglyme)	(*S*,*S*)-**L1**	DEMS	CsF	DMAc/*t*BuOH (5:4)	27	85
19	$NiBr_2$(diglyme)	(*S*,*S*)-**L1**	DEMS	K_2CO_3	DMAc/*t*BuOH (5:4)	trace	N.D.
20	$NiBr_2$(diglyme)	(*S*,*S*)-**L1**	DEMS	Cs_2CO_3	DMAc/*t*BuOH (5:4)	5	N.D.
21	$NiBr_2$(diglyme)	(*S*,*S*)-**L1**	DEMS	KF	glyme	5	81
22	$NiBr_2$(diglyme)	(*S*,*S*)-**L1**	DEMS	KF	DCE	trace	N.D.
23	$NiBr_2$(diglyme)	(*S*,*S*)-**L1**	DEMS	KF	2-Me THF	7	87
24	$NiBr_2$(diglyme)	(*S*,*S*)-**L1**	DEMS	KF	*t*BuOH	28	83
25	$NiBr_2$(diglyme)	(*S*,*S*)-**L1**	DEMS	KF	DMAc	70	89
26	$NiBr_2$(diglyme)	(*S*,*S*)-**L1**	DEMS	KF	Diglyme	13	77
27	$NiBr_2$(diglyme)	(*S*,*S*)-**L1**	DEMS	KF	toluene	trace	N.D.
28	$NiBr_2$(diglyme)	(*S*,*S*)-**L1**	DEMS	KF	DMAc/THF(1:1)	68	87
29	$NiBr_2$(diglyme)	(*S*,*S*)-**L1**	DEMS	KF	DMAc/DCE (1:1)	60	73
30	$NiBr_2$(diglyme)	(*S*,*S*)-**L1**	DEMS	KF	DMAc/*t*BuOH(1:1)	85	90

[a] Conditions: **RM23** (0.2 mmol, 1.0 equiv.), **RM1** (0.4 mmol, 2.0 equiv.), nickel source (0.02 mmol, 10 mol%), ligand (0.024 mmol, 12 mol%), silane (0.6 mmol, 3 equiv.), base (0.6 mmol, 3 equiv.), solvent (0.8 mL), 25 °C, 20 h. Triphenylmethane was used as an internal standard. GC yield. [b] Isolated yield. Bn = benzyl. Glyme = 1,2-dimethoxyethane. DCE = 1,2-dichloroethane. Diglyme = 2-methoxyethyl ether. THF = tetrahydrofuran. DMAc = *N*,*N*-dimethylacetamide. DEMS = diethoxymethylsilane. e.e. = enantiomeric excess. N.D. = not detected.

In the air, a 10 mL screw-cap test tube equipped with a magnetic stirrer was charged

with nickel source (0.02 mmol, 10 mol%), ligand (0.024 mmol, 12 mol%). The test tube was evacuated and backfilled with argon for three times, then solvent (0.8 mL) was added and the mixture was stirred at room temperature for 30 min.

Meanwhile, in the air, another 10 mL screw-cap test tube equipped with a magnetic stirrer was charged with base (0.6 mmol, 3.0 equiv.) (if enamide or alkyl halide is a solid, it was also added at this time). The test tube was evacuated and backfilled with argon for three times. Next, the solution of the catalyst (see above) was added in one portion via syringe, followed by the enamide (0.2 mmol, 1.0 equiv.) and alkyl halide (0.4 mmol, 2.0 equiv.). The resulting solution was stirred for 2 min at 0 °C, silane (0.6 mmol, 3.0 equiv.) was added dropwise via syringe and the solution was kept stirring for 5 min at 0 °C, then was stirred at 25 °C for 20 h. The GC yield was determined using triphenylmethane as an internal standard.

General Procedure for Asymmetric Reductive Hydroalkylation of Tertiary Enamides

General Procedure D1 (GP-D1): Asymmetric Reductive Hydroalkylation with Primary Alkyl Iodides (Fig. 2). In the air, a 10 mL screw-cap test tube equipped with a magnetic stirrer was charged with $NiBr_2$(diglyme) (0.02 mmol, 10 mol%), ligand (0.024 mmol, 12 mol%). The test tube was evacuated and backfilled with argon for three times, then DMAc/*t*BuOH (5:4, 0.8 mL) was added and the mixture was stirred at room temperature for 30 min.

Meanwhile, in the air, another 10 mL screw-cap test tube equipped with a magnetic stirrer was charged with potassium fluoride (0.6 mmol, 3.0 equiv.) (if enamide or alkyl halide is a solid, it was also added at this time). The test tube was evacuated and backfilled with argon for three times. Next, the solution of the catalyst (see above) was added in one portion via syringe, followed by the enamide (0.2 mmol, 1.0 equiv.) and alkyl halide (0.4 mmol, 2.0 equiv.). The resulting solution was stirred for 2 min at 0 °C, DEMS (0.6 mmol, 3.0 equiv.) was added dropwise via syringe and the solution was kept stirring for 5 min at 0 °C, then was stirred at 25 °C for 20 h. The reaction mixture

was diluted with H_2O followed by extraction with EtOAc, dried with anhydrous Na_2SO_4 and concentrated in vacuo. The residue was purified by flash column chromatography on silica gel to give the target product.

General Procedure D2 (GP-D2): Asymmetric Reductive Hydroalkylation with Primary Alkyl Iodides (Fig. 2). The procedure is the same as **GP-D1**, except for reaction temperature and time: the reaction mixture was stirred at 5 °C for 30 h.

General Procedure D3 (GP-D3): Asymmetric Reductive Hydroalkylation with Primary Alkyl Bromides (Fig. 2). In the air, a 10 mL screw-cap test tube equipped with a magnetic stirrer was charged with $NiBr_2$(diglyme) (0.02 mmol, 10 mol%), ligand (0.024 mmol, 12 mol%). The test tube was evacuated and backfilled with argon for three times, then DMAc/*t*BuOH (5:4, 0.8 mL) was added and the mixture was stirred at room temperature for 30 min.

Meanwhile, in the air, another 10 mL screw-cap test tube equipped with a magnetic stirrer was charged with potassium fluoride (0.6 mmol, 3.0 equiv.) and NaI (0.1 mmol, 0.5 equiv.) (if enamide or alkyl halide is a solid, it was also added at this time). The test tube was evacuated and backfilled with argon for three times. Next, the solution of the catalyst (see above) was added in one portion via syringe, followed by the enamide (0.2 mmol, 1.0 equiv.) and alkyl halide (0.4 mmol, 2.0 equiv.). The resulting solution was stirred for 2 min at 0 °C, DEMS (0.6 mmol, 3.0 equiv.) was added dropwise via syringe and the solution was kept stirring for 5 min at 0 °C, then was stirred at 40 °C for 20 h. The reaction mixture was diluted with H_2O followed by extraction with EtOAc, dried with anhydrous Na_2SO_4 and concentrated in vacuo. The residue was purified by flash column chromatography on silica gel to give the target product.

General Procedure D4 (GP-D4): Asymmetric Reductive Hydroalkylation with Secondary Alkyl Iodides (Fig. 2). The procedure is the same as **GP-D1**, except for reaction temperature: the reaction mixture was stirred at 50 °C for 20 h.

Examples Described in Figure 2

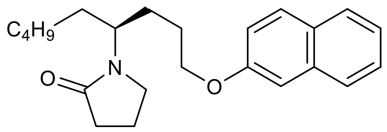

(*S*)-1-(1-(naphthalen-2-yloxy)nonan-4-yl)pyrrolidin-2-one (**1**)

Following **GP-D1**, **1** was obtained as pale-yellow oil (65.7 mg, 93% yield, 96% ee).

^{1}H NMR (500 MHz, Chloroform-*d*) δ 7.83 – 7.68 (m, 3H), 7.49 – 7.39 (m, 1H), 7.36 – 7.28 (m, 1H), 7.17 – 7.08 (m, 2H), 4.28 – 4.13 (m, 1H), 4.12 – 4.00 (m, 2H), 3.39 – 3.14 (m, 2H), 2.41 (t, *J* = 8.0 Hz, 2H), 2.08 – 1.90 (m, 2H), 1.85 – 1.74 (m, 2H), 1.73 – 1.60 (m, 2H), 1.54 – 1.42 (m, 2H), 1.38 – 1.12 (m, 6H), 0.87 (t, *J* = 6.8 Hz, 3H).

^{13}C NMR (126 MHz, Chloroform-*d*) δ 175.54, 156.96, 134.70, 129.43, 129.01, 127.70, 126.84, 126.43, 123.63, 118.99, 106.76, 67.37, 50.82, 41.80, 32.71, 31.74, 31.67, 29.00, 26.21, 26.07, 22.66, 18.40, 14.13.

HRMS (ESI) calcd for $C_{23}H_{32}NO_2^+$ $[(M+H)^+]$ 354.2428, found 354.2426.

HPLC analysis: The ee was determined to be 96% on a CHIRALCEL OD-H column (20% *i*PrOH in hexane, 2.0 mL/min, 40 °C); retention times for compound obtained using (*S*,*S*)-**L1**: 3.8 min (minor), 4.2 min (major).

$[\alpha]_D$ (20.0 ℃, c = 1.0 in $CHCl_3$) = -5.34 °, 96% ee, from (*S*,*S*)-**L1**.

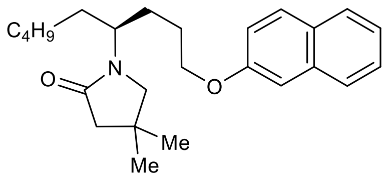

(*S*)-4,4-dimethyl-1-(1-(naphthalen-2-yloxy)nonan-4-yl)pyrrolidin-2-one (**2**)

Following **GP-D1**, **2** was obtained as pale-yellow oil (65.5 mg, 86% yield, 97% ee).

^{1}H NMR (400 MHz, Chloroform-*d*) δ 7.79 – 7.67 (m, 3H), 7.42 (ddd, *J* = 8.2, 6.8, 1.3 Hz, 1H), 7.31 (ddd, *J* = 8.1, 6.8, 1.2 Hz, 1H), 7.16 – 7.09 (m, 2H), 4.22 – 4.12 (m, 1H), 4.11 – 4.02 (m, 2H), 2.96 (dd, *J* = 2.2 Hz, 2H), 2.24 (s, 2H), 1.88 – 1.74 (m, 2H), 1.72

– 1.53 (m, 2H), 1.50 – 1.39 (m, 2H), 1.36 – 1.20 (m, 6H), 1.13 (d, *J* = 13.7 Hz, 6H), 0.93 – 0.82 (m, 3H).

^{13}C NMR (101 MHz, Chloroform-*d*) δ 174.73, 156.86, 134.61, 129.34, 128.92, 127.62, 126.75, 126.34, 123.54, 118.94, 106.65, 67.26, 54.96, 50.40, 46.74, 32.65, 32.59, 31.65, 29.00, 28.07, 28.01, 26.13, 26.01, 22.58, 14.05.

HRMS (ESI) calcd for $C_{25}H_{36}NO_2^+$ [(M+H)$^+$] 382.2741, found 382.2747.

HPLC analysis: The ee was determined to be 97% on a CHIRALCEL OD-H column (10% *i*PrOH in hexane, 1.0 mL/min, 40 °C); retention times for compound obtained using (*S*,*S*)-**L1**: 11.7 min (minor), 14.4 min (major).

$[\alpha]_D$ (20.0 °C, c = 1.0 in $CHCl_3$) = -3.34 °; 97% ee, from (*S*,*S*)-**L1**.

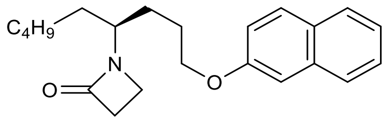

(*S*)-1-(1-(naphthalen-2-yloxy)nonan-4-yl)azetidin-2-one (**3**)

Following **GP-D2**, **3** was obtained as yellow oil (61.9 mg, 91% yield, 89% ee).

^{1}H NMR (400 MHz, Chloroform-*d*) δ 7.80 – 7.64 (m, 3H), 7.49 – 7.39 (m, 1H), 7.37 – 7.29 (m, 1H), 7.16 – 7.08 (m, 2H), 4.16 – 4.01 (m, 2H), 3.82 – 3.65 (m, 1H), 3.23 – 3.09 (m, 2H), 2.94 – 2.81 (m, 2H), 1.94 – 1.81 (m, 2H), 1.72 – 1.63 (m, 2H), 1.54 – 1.44 (m, 2H), 1.37 – 1.19 (m, 6H), 0.97 – 0.79 (m, 3H).

^{13}C NMR (101 MHz, Chloroform-*d*) δ 168.03, 156.84, 134.59, 129.39, 128.94, 127.64, 126.76, 126.38, 123.60, 118.88, 106.61, 67.18, 51.63, 35.56, 35.42, 32.99, 31.56, 29.48, 26.32, 26.14, 22.57, 14.05.

HRMS (ESI) calcd for $C_{22}H_{30}NO_2^+$ [(M+H)$^+$] 340.2271, found 340.2275.

HPLC analysis: The ee was determined to be 89% on a CHIRALCEL OD-H column (15% *i*PrOH in hexane, 1.5 mL/min, 40 °C); retention times for compound obtained using (*S*,*S*)-**L1**: 6.4 min (minor), 7.0 min (major).

$[\alpha]_D$ (20.0 °C, c = 1.0 in $CHCl_3$) = -8.39 °; 89% ee, from (*S*,*S*)-**L1**.

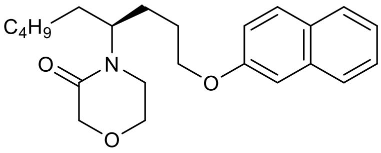

(*S*)-4-(1-(naphthalen-2-yloxy)nonan-4-yl)morpholin-3-one (**4**)

Following **GP-D2**, **4** was obtained as light red oil (66.5 mg, 90% yield, 98% ee).

^{1}H NMR (400 MHz, Chloroform-*d*) δ 7.74 (td, *J* = 8.9, 8.3, 6.2 Hz, 3H), 7.43 (ddd, *J* = 8.2, 6.8, 1.3 Hz, 1H), 7.33 (ddd, *J* = 8.1, 6.8, 1.2 Hz, 1H), 7.18 – 7.08 (m, 2H), 4.73 (p, *J* = 7.6 Hz, 1H), 4.23 (s, 2H), 4.09 (td, *J* = 6.1, 2.6 Hz, 2H), 3.97 – 3.78 (m, 2H), 3.20 (t, *J* = 5.0 Hz, 2H), 1.86 – 1.77 (m, 2H), 1.73 – 1.65 (m, 2H), 1.55 – 1.43 (m, 2H), 1.36 – 1.22 (m, 6H), 0.88 (t, *J* = 6.8 Hz, 3H).

^{13}C NMR (101 MHz, Chloroform-*d*) δ 167.15, 156.92, 134.68, 129.47, 129.02, 127.73, 126.84, 126.47, 123.68, 118.96, 106.74, 68.18, 67.33, 64.16, 51.57, 39.44, 31.94, 31.76, 28.29, 26.06, 25.94, 22.66, 14.16.

HRMS (ESI) calcd for $C_{23}H_{32}NO_3^+$ [(M+H)$^+$] 370.2377, found 370.2380.

HPLC analysis: The ee was determined to be 98% on a CHIRALPAK AD-H column (10% *i*PrOH in hexane, 1.0 mL/min, 40 °C); retention times for compound obtained using (*S,S*)-**L1**: 10.1 min (minor), 10.8 min (major).

$[\alpha]_D$ (20.0 °C, c = 1.0 in $CHCl_3$) = -7.03 °; 98% ee, from (*S,S*)-**L1**.

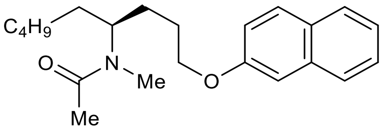

(*S*)-*N*-methyl-*N*-(1-(naphthalen-2-yloxy) nonan-4-yl)acetamide (**5**)

Following **GP-D1**, **5** was obtained as pale-yellow oil (61.4 mg, 90% yield, 92% ee).

Mixture of rotational isomers, ratio = 55:45.

^{1}H NMR (500 MHz, Chloroform-*d*) δ 7.83 – 7.67 (m, 3H), 7.49 – 7.39 (m, 1H), 7.37 – 7.28 (m, 1H), 7.17 – 7.08 (m, 2H), 4.74 (p, *J* = 7.6 Hz, 0.55H), 4.14 – 4.00 (m, 2H), 3.71 (tt, *J* = 9.3, 5.1 Hz, 0.46H), 2.77 (s, 1.59H), 2.75 (s, 1.33H), 2.14 (s, 1.38H), 2.10 (s, 1.60H), 1.82 – 1.67 (m, 3H), 1.66 – 1.58 (m, 1H), 1.56 – 1.46 (m, 1H), 1.45 – 1.39 (m, 1H), 1.36 – 1.14 (m, 6H), 0.99 – 0.83 (m, 3H).

<u>^{13}C NMR</u> (101 MHz, Chloroform-*d*) δ 171.46, 171.20, 156.98, 156.86, 134.67, 134.60, 129.52, 129.37, 129.03, 128.94, 127.72, 127.67, 126.81, 126.78, 126.48, 126.38, 123.72, 123.56, 118.99, 118.86, 106.67, 106.58, 67.52, 67.45, 58.09, 51.79, 33.25, 32.54, 31.84, 31.80, 29.95, 29.05, 28.76, 26.23, 26.12, 26.07, 26.01, 25.80, 22.65, 22.62, 22.49, 22.12, 14.13, 14.11.

<u>HRMS (ESI)</u> calcd for $C_{22}H_{32}NO_2^+$ [$(M+H)^+$] 342.2428, found 342.2428.

<u>HPLC analysis:</u> The ee was determined to be 92% on a CHIRALPAK AD-H column (7% *i*PrOH in hexane, 0.7 mL/min, 40 °C); retention times for compound obtained using (*S*,*S*)-**L1**: 13.3 min (minor), 14.1 min (major).

$[\alpha]_D$ (20.0 ℃, c = 1.0 in $CHCl_3$) = -6.22 °; 92% ee, from (*S*,*S*)-**L1**.

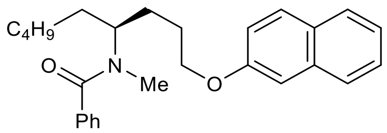

(*S*)-*N*-methyl-*N*-(1-(naphthalen-2-yloxy)nonan-4-yl)benzamide (**6**)

Following **GP-D1**, **6** was obtained as pale-yellow oil (72.6 mg, 90% yield, 93% ee). Mixture of rotational isomers, ratio = 45:55.

<u>^{1}H NMR</u> (500 MHz, Chloroform-*d*) δ 7.80 – 7.69 (m, 3H), 7.49 – 7.40 (m, 1H), 7.41 – 7.30 (m, 6H), 7.19 – 7.13 (m, 1H), 7.14 – 7.07 (m, 0.67H), 7.07 – 7.03 (m, 0.48H), 5.01 – 4.79 (m, 0.45H), 4.26 – 4.07 (m, 1H), 3.99 – 3.93 (m, 1H), 3.76 – 3.62 (m, 0.57H), 2.95 (s, 1.77H), 2.73 (s, 1.25H), 2.01 – 1.88 (m, 1H), 1.84 – 1.64 (m, 3H), 1.62 – 1.50 (m, 1H), 1.50 – 1.25 (m, 5H), 1.24 – 1.13 (m, 2H), 1.05 – 0.80 (m, 3H).

<u>^{13}C NMR</u> (126 MHz, Chloroform-*d*) δ 172.82, 172.43, 156.97, 156.85, 137.55, 137.29, 134.71, 134.63, 129.48, 129.42, 129.28, 129.07, 129.02, 128.53, 128.50, 127.73, 127.71, 127.69, 126.84, 126.80, 126.76, 126.65, 126.46, 126.40, 123.70, 123.61, 119.02, 118.87, 106.84, 106.69, 67.55, 67.29, 58.15, 52.32, 33.15, 32.34, 31.81, 31.78, 30.71, 29.82, 28.67, 26.28, 26.13, 26.12, 26.03, 22.71, 22.58, 14.13, 14.06.

<u>HRMS (ESI)</u> calcd for $C_{27}H_{34}NO_2^+$ [$(M+H)^+$] 404.2584, found 404.2578.

<u>HPLC analysis:</u> The ee was determined to be 92% on a CHIRALPAK AS-H column (10% *i*PrOH in hexane, 0.7 mL/min, 40 °C); retention times for compound obtained

using (*S*,*S*)-**L1**: 7.4 min (minor), 7.9 min (major).

[α]D (20.0 ℃, c = 1.0 in $CHCl_3$) = -11.52 °, 92% ee, from (*S*,*S*)-**L1**.

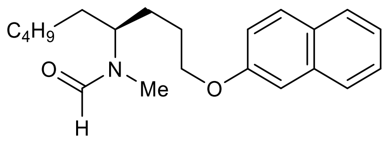

(*S*)-*N*-methyl-*N*-(1-(naphthalen-2-yloxy)nonan-4-yl)formamide (**7**)

Following **GP-D1**, 7 was obtained as pale-yellow oil (45.8 mg, 70% yield, 93% ee). Mixture of rotational isomers, ratio = 25:75.

^{1}H NMR (400 MHz, Chloroform-*d*) δ 8.14 (s, 1H), 7.86 – 7.65 (m, 3H), 7.48 – 7.38 (m, 1H), 7.39 – 7.28 (m, 1H), 7.19 – 7.08 (m, 2H), 4.47 (p, *J* = 7.5 Hz, 0.23H), 4.18 – 3.98 (m, 2H), 3.49 – 3.30 (m, 0.75H), 2.79 (s, 0.75H), 2.75 (s, 2.25H), 1.84 – 1.68 (m, 4H), 1.67 – 1.45 (m, 2H), 1.37 – 1.12 (m, 6H), 0.97 – 0.79 (m, 3H).

^{13}C NMR (101 MHz, Chloroform-*d*) δ 163.60, 163.23, 156.88, 156.76, 134.59, 134.54, 129.45, 129.33, 128.96, 128.90, 127.66, 127.61, 126.75, 126.72, 126.43, 126.34, 123.66, 123.54, 118.90, 118.80, 106.59, 106.51, 67.22, 67.20, 58.56, 50.65, 32.44, 31.74, 31.60, 31.44, 29.26, 28.91, 28.13, 26.04, 25.91, 25.87, 25.78, 24.28, 22.57, 22.54, 14.06, 14.03.

HRMS (ESI) calcd for $C_{21}H_{30}NO_2^+$ [$(M+H)^+$] 328.2277, found 328.2271.

HPLC analysis: The ee was determined to be 93% on a CHIRALPAK AS-H column (10% *i*PrOH in hexane, 1.0 mL/min, 40 °C); retention times for compound obtained using (*S*,*S*)-**L1**: 10.8 min (minor), 9.6 min (major).

[α]D (20.0 ℃, c = 1.0 in $CHCl_3$) = -3.37 °, 93% ee, from (*S*,*S*)-**L1**.

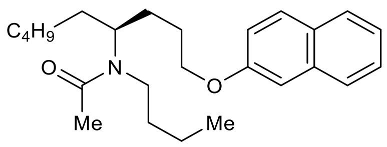

(*S*)-*N*-butyl-*N*-(1-(naphthalen-2-yloxy)nonan-4-yl)acetamide (**8**)

Following **GP-D1**, **8** was obtained as yellow oil (72.0 mg, 94% yield, 90% ee). Mixture of rotational isomers, ratio = 46:54.

^{1}H NMR (400 MHz, Chloroform-*d*) δ 7.83 – 7.68 (m, 3H), 7.49 – 7.38 (m, 1H), 7.38 – 7.27 (m, 1H), 7.18 – 7.07 (m, 2H), 4.57 (s, 0.46H), 4.20 – 3.98 (m, 2H), 3.80 – 3.57 (m, 0.54H), 3.25 – 2.97 (m, 2H), 2.13 (s, 1.50H), 2.11 (s, 1.77H), 1.90 – 1.73 (m, 2H), 1.76 – 1.65 (m, 2H), 1.65 – 1.52 (m, 2H), 1.54 – 1.44 (m, 2H), 1.42 – 1.16 (m, 8H), 0.95 (q, *J* = 7.8 Hz, 3H), 0.89 (q, *J* = 6.5 Hz, 3H).

^{13}C NMR (101 MHz, Chloroform-*d*) δ 171.47, 170.91, 156.99, 156.85, 134.66, 134.60, 129.51, 129.36, 129.02, 128.93, 127.71, 127.66, 126.79, 126.77, 126.47, 126.36, 123.70, 123.54, 118.98, 118.84, 106.64, 106.55, 67.59, 67.56, 58.89, 53.73, 44.06, 41.00, 34.10, 33.51, 33.06, 31.90, 31.89, 31.10, 30.75, 29.80, 26.44, 26.37, 26.30, 26.26, 22.67, 22.65, 22.62, 22.14, 20.91, 20.65, 14.14, 14.12, 13.96, 13.82.

HRMS (ESI) calcd for $C_{25}H_{38}NO_2^+$ [(M+H)$^+$] 384.2903, found 384.2901.

HPLC analysis: The ee was determined to be 90% on a CHIRALCEL OD-H column (20% *i*PrOH in hexane, 2.0 mL/min, 40 °C); retention times for compound obtained using (*S*,*S*)-**L1**: 3.7 min (minor), 2.8 min (major).

$[\alpha]_D$ (20.0 °C, c = 1.0 in $CHCl_3$) = -2.77 °; 90% ee, from (*S*,*S*)-**L1**.

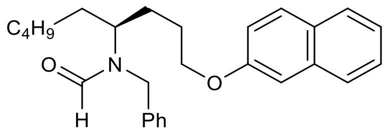

(*S*)-*N*-benzyl-*N*-(1-(naphthalen-2-yloxy)nonan-4-yl)formamide (**9**)

Following **GP-D1**, **9** was obtained as pale-yellow oil (56.4 mg, 70% yield, 91% ee). Mixture of rotational isomers, ratio = 24:76.

^{1}H NMR (400 MHz, Chloroform-*d*) δ 8.39 (s, 0.23H), 8.25 (s, 0.73H), 7.94 – 7.63 (m, 3H), 7.47 – 7.18 (m, 7H), 7.14 – 6.97 (m, 2H), 4.65 – 4.40 (m, 1.46H), 4.29 (s, 0.71H), 4.15 – 4.05 (m, 0.11H), 3.97 – 3.69 (m, 1.94H), 3.49 – 3.30 (m, 0.71H), 1.79 – 1.54 (m, 4H), 1.56 – 1.37 (m, 2H), 1.32 – 0.99 (m, 6H), 0.89 – 0.71 (m, 3H).

^{13}C NMR (101 MHz, Chloroform-*d*) δ 164.24, 163.72, 156.93, 156.80, 138.27, 137.46, 134.64, 134.58, 129.47, 129.37, 129.00, 128.95, 128.85, 128.60, 128.53, 128.13, 128.06, 127.72, 127.69, 127.51, 126.79, 126.76, 126.47, 126.39, 123.70, 123.59,

118.99, 118.87, 106.63, 106.57, 67.36, 67.20, 59.62, 53.36, 48.80, 44.00, 33.83, 32.79, 31.66, 31.47, 30.53, 29.19, 26.24, 26.05, 26.01, 22.59, 22.54, 14.11, 14.06.

HRMS (ESI) calcd for $C_{27}H_{34}NO_2^+$ $[(M+H)^+]$ 404.2590, found 404.2593.

HPLC analysis: The ee was determined to be 91% on a CHIRALPAK AD-H column (8% *i*PrOH in hexane, 0.8 mL/min, 40 °C); retention times for compound obtained using (*S*,*S*)-**L1**: 16.3 min (minor), 15.2 min (major).

$[\alpha]_D$ (20.0 °C, c = 1.0 in $CHCl_3$) = 6.36 °; 91% ee, from (*S*,*S*)-**L1**.

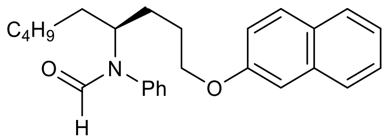

(*S*)-*N*-(1-(naphthalen-2-yloxy)nonan-4-yl)-*N*-phenylformamide (**10**)

Following **GP-D1**, **10** was obtained as brown oil (70.0 mg, 90% yield, 91% ee).

Mixture of rotational isomers, ratio = 85:15.

^{1}H NMR (500 MHz, Chloroform-*d*) δ 8.37 (s, 0.12H), 8.35 (s, 0.84H), 7.81 – 7.70 (m, 3H), 7.48 – 7.30 (m, 5H), 7.21 (d, *J* = 7.6 Hz, 2H), 7.16 – 7.10 (m, 2H), 4.74 – 4.55 (m, 0.85H), 4.20 – 4.05 (m, 2H), 3.84 – 3.65 (m, 0.14H), 2.14 – 1.89 (m, 2H), 1.88 – 1.72 (m, 2H), 1.69 – 1.47 (m, 2H), 1.46 – 1.37 (m, 2H), 1.35 – 1.21 (m, 4H), 1.03 – 0.80 (m, 3H).

^{13}C NMR (101 MHz, Chloroform-*d*) δ 163.77, 163.66, 156.95, 156.82, 139.20, 136.78, 134.68, 134.63, 129.60, 129.51, 129.46, 129.43, 129.09, 129.02, 128.64, 128.13, 127.99, 127.78, 127.74, 126.84, 126.55, 126.45, 123.79, 123.65, 119.01, 118.89, 106.67, 106.62, 67.47, 67.38, 61.15, 55.07, 34.24, 33.53, 31.80, 31.70, 30.95, 29.98, 26.55, 26.49, 26.38, 22.67, 14.16.

HRMS (ESI) calcd for $C_{26}H_{32}NO_2^+$ $[(M+H)^+]$ 390.2433, found 390.2434.

HPLC analysis: The ee was determined to be 91% on a CHIRALPAK AS-H column (7% *i*PrOH in hexane, 0.7 mL/min, 40 °C); retention times for compound obtained using (*S*,*S*)-**L1**: 13.5 min (minor), 11.5 min (major).

$[\alpha]_D$ (20.0 °C, c = 1.0 in $CHCl_3$) = 9.12 °; 91% ee, from (*S*,*S*)-**L1**.

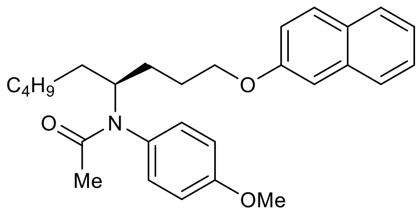

(*S*)-*N*-(4-methoxyphenyl)-*N*-(1-(naphthalen-2-yloxy)nonan-4-yl)acetamide (**11**)

Following **GP-D1**, **11** was obtained as yellow oil (71.0 mg, 82% yield, 94% ee).

^{1}H NMR (400 MHz, Chloroform-*d*) δ 7.85 – 7.66 (m, 3H), 7.48 – 7.39 (m, 1H), 7.37 – 7.28 (m, 1H), 7.21 – 7.10 (m, 2H), 7.10 – 7.00 (m, 2H), 6.95 – 6.87 (m, 2H), 4.97 – 4.80 (m, 1H), 4.25 – 4.04 (m, 2H), 3.84 (s, 3H), 2.11 – 1.89 (m, 2H), 1.79 (s, 3H), 1.55 (q, *J* = 7.5 Hz, 2H), 1.41 (q, *J* = 5.9 Hz, 2H), 1.37 – 1.21 (m, 6H), 0.89 (t, *J* = 6.8 Hz, 3H).

^{13}C NMR (101 MHz, Chloroform-*d*) δ 171.78, 159.27, 157.01, 134.71, 131.84, 131.00, 129.43, 128.99, 127.72, 126.84, 126.43, 123.62, 119.04, 114.51, 106.69, 67.63, 55.56, 54.20, 33.47, 32.02, 29.67, 26.61, 23.70, 22.74, 14.22.

HRMS (ESI) calcd for $C_{28}H_{36}NO_3^+$ [$(M+H)^+$] 434.2695, found 434.2698.

HPLC analysis: The ee was determined to be 94% on a CHIRALPAK AD-H column (15% *i*PrOH in hexane, 1.5 mL/min, 40 °C); retention times for compound obtained using (*S,S*)-**L1**: 5.4 min (minor), 4.6 min (major).

$[\alpha]_D$ (20.0 ℃, c = 1.0 in $CHCl_3$) = 15.35 °; 94% ee, from (*S,S*)-**L1**.

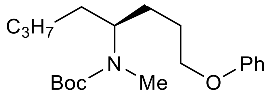

tert-butyl (*S*)-methyl(1-phenoxyoctan-4-yl)carbamate (**12**)

Following **GP-D2**, **12** was obtained as pale yellow oil (50.3 mg, 75% yield, 86% ee).

Mixture of rotational isomers, ratio = 43:57.

^{1}H NMR (400 MHz, Chloroform-*d*) δ 7.33 – 7.22 (m, 2H), 6.98 – 6.84 (m, 3H), 4.28 – 4.12 (m, 0.43H), 4.07 – 3.87 (m, 2.61H), 2.67 (s, 1.72H), 2.62 (s, 1.32H), 1.89 – 1.65 (m, 2H), 1.63 – 1.52 (m, 2H), 1.46 (s, 4.68H), 1.45 (s, 4.56H), 1.42 – 1.14 (m, 6H), 0.99 – 0.82 (m, 3H).

13C NMR (101 MHz, Chloroform-*d*) δ 159.12, 159.10, 156.60, 156.55, 129.53, 129.49, 120.69, 120.57, 114.59, 114.54, 79.39, 79.06, 67.56, 67.32, 54.65, 53.81, 32.63, 32.45, 29.30, 29.06, 28.60, 28.51, 28.46, 27.31, 26.69, 26.23, 26.16, 22.65, 22.48, 14.17.

HRMS (ESI) calcd for $C_{20}H_{34}NO_3^+$ [(M+H)$^+$] 336.2539, found 336.2535.

HPLC analysis: The ee was determined to be 86% on a CHIRALCEL OD-H column (10% *i*PrOH in hexane, 1.0 mL/min, 40 °C); retention times for compound obtained using (*S,S*)-**L1**: 3.9 min (minor), 3.5 min (major).

$[\alpha]_D$ (20.0 ℃, c = 1.0 in $CHCl_3$) = 1.42 °, 86% ee, from (*S,S*)-**L1**.

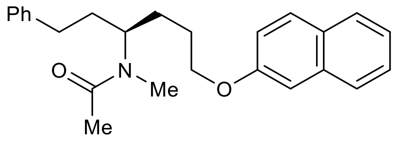

(*S*)-*N*-methyl-*N*-(6-(naphthalen-2-yloxy)-1-phenylhexan-3-yl)acetamide (**13**)

Following **GP-D1**, **13** was obtained as pale yellow oil (69.8 mg, 93% yield, 94% ee). Mixture of rotational isomers, ratio = 45:55.

1H NMR (400 MHz, Chloroform-*d*) δ 7.85 – 7.70 (m, 3H), 7.57 – 7.40 (m, 1H), 7.40 – 7.25 (m, 3H), 7.26 – 7.09 (m, 5H), 4.87 (p, *J* = 7.5 Hz, 0.55H), 4.19 – 3.98 (m, 2H), 3.79 – 3.66 (m, 0.45H), 2.82 (s, 1.31H), 2.76 (s, 1.70H), 2.71 – 2.44 (m, 2H), 2.10 (s, 1.73H), 1.97 (s, 1.31H), 1.95 – 1.57 (m, 6H).

13C NMR (101 MHz, Chloroform-*d*) δ 171.62, 171.25, 156.92, 156.79, 141.92, 140.88, 134.64, 134.57, 129.52, 129.38, 129.02, 128.94, 128.67, 128.43, 128.32, 128.29, 127.72, 127.66, 126.79, 126.76, 126.49, 126.38, 126.29, 125.92, 123.73, 123.58, 118.95, 118.82, 106.66, 106.57, 67.39, 67.30, 56.99, 51.96, 34.51, 34.39, 32.93, 32.32, 29.75, 29.12, 28.74, 26.07, 26.01, 25.91, 22.48, 21.88.

HRMS (ESI) calcd for $C_{25}H_{30}NO_2^+$ [(M+H)$^+$] 376.2277, found 376.2278.

HPLC analysis: The ee was determined to be 94% on a CHIRALPAK AD-H column (8% *i*PrOH in hexane, 0.8 mL/min, 40 °C); retention times for compound obtained using (*S,S*)-**L1**: 19.6 min (minor), 21.0 min (major).

$[\alpha]_D$ (20.0 ℃, c = 1.0 in $CHCl_3$) = 7.92 °, 94% ee, from (*S,S*)-**L1**.

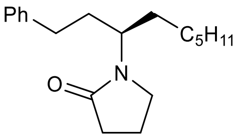

(*R*)-1-(1-phenylnonan-3-yl)pyrrolidin-2-one (**14**)

Following **GP-D1**, **14** was obtained as pale yellow oil (40.2 mg, 70% yield, 91% ee).

^{1}H NMR (400 MHz, Chloroform-*d*) δ 7.31 – 7.22 (m, 2H), 7.21 – 7.12 (m, 3H), 4.16 (p, *J* = 7.5, 7.0 Hz, 1H), 3.33 – 3.14 (m, 2H), 2.68 – 2.57 (m, 1H), 2.55 – 2.45 (m, 1H), 2.44 – 2.34 (m, 2H), 2.03 – 1.92 (m, 1H), 1.92 – 1.83 (m, 1H), 1.80 – 1.71 (m, 2H), 1.51 – 1.40 (m, 2H), 1.36 – 1.12 (m, 8H), 0.86 (t, *J* = 6.9 Hz, 3H).

^{13}C NMR (101 MHz, Chloroform-*d*) δ 175.52, 141.99, 128.48, 128.37, 125.98, 51.38, 41.92, 34.41, 33.07, 32.72, 31.85, 31.69, 29.24, 26.31, 22.70, 18.33, 14.20.

HRMS (ESI) calcd for $C_{19}H_{30}NO^+$ [(M+H)$^+$] 288.2327, found 288.2325.

HPLC analysis: The ee was determined to be 91% on a CHIRALCEL OD-H column (10% *i*PrOH in hexane, 1.0 mL/min, 40 °C); retention times for compound obtained using (*S*,*S*)-**L1**: 5.1 min (minor), 5.8 min (major).

$[\alpha]_D$ (20.0 °C, c = 1.0 in $CHCl_3$) = 16.87 °; 91% ee, from (*S*,*S*)-**L1**.

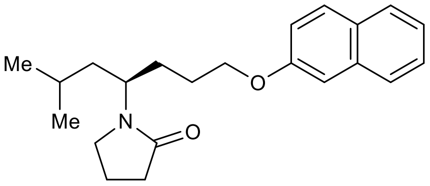

(*R*)-1-(6-methyl-1-(naphthalen-2-yloxy)heptan-4-yl)pyrrolidin-2-one (**15**)

Following **GP-D1**, **15** was obtained as pale-yellow oil (27.1 mg, 40% yield, 93% ee).

^{1}H NMR (400 MHz, Chloroform-*d*) δ 7.80 – 7.68 (m, 3H), 7.49 – 7.38 (m, 1H), 7.37 – 7.28 (m, 1H), 7.18 – 7.09 (m, 2H), 4.40 – 4.21 (m, 1H), 4.15 – 4.00 (m, 2H), 3.30 – 3.19 (m, 2H), 2.40 (t, *J* = 9.0 Hz, 2H), 2.02 – 1.91 (m, 2H), 1.85 – 1.72 (m, 2H), 1.70 – 1.61 (m, 2H), 1.56 – 1.38 (m, 2H), 1.31 – 1.17 (m, 1H), 0.94 (d, *J* = 6.5 Hz, 3H), 0.91 (d, *J* = 6.5 Hz, 3H).

^{13}C NMR (101 MHz, Chloroform-*d*) δ 175.34, 156.96, 134.69, 129.43, 128.99, 127.71, 126.84, 126.43, 123.63, 119.01, 106.73, 67.38, 48.71, 41.81, 41.67, 31.66, 29.31, 26.16, 25.13, 23.53, 22.11, 18.39.

HRMS (ESI) calcd for $C_{22}H_{30}NO_2^+$ [(M+H)$^+$] 340.2277, found 340.2278.

HPLC analysis: The ee was determined to be 93% on a CHIRALCEL OD-H column (15% *i*PrOH in hexane, 1.5 mL/min, 40 °C); retention times for compound obtained using (*S,S*)-**L1**: 6.1 min (minor), 7.4 min (major).

[α]$_D$ (20.0 °C, c = 1.0 in $CHCl_3$) = -7.69 °; 93% ee, from (*S,S*)-**L1**.

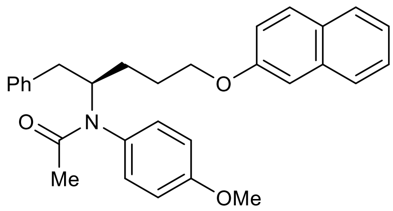

(*R*)-*N*-(4-methoxyphenyl)-*N*-(5-(naphthalen-2-yloxy)-1-phenylpentan-2-yl)acetamide (**16**)

Following **GP-D1**, **16** was obtained as brown oil (54.4 mg, 60% yield, 97% ee).

^{1}H NMR (400 MHz, Chloroform-*d*) δ 7.85 – 7.66 (m, 3H), 7.51 – 7.39 (m, 1H), 7.38 – 7.26 (m, 3H), 7.26 – 7.18 (m, 3H), 7.16 – 7.07 (m, 2H), 6.99 – 6.77 (m, 4H), 5.17 (s, 1H), 4.21 – 4.09 (m, 1H), 4.08 – 3.93 (m, 1H), 3.83 (s, 3H), 2.88 (dd, *J* = 14.3, 7.7 Hz, 1H), 2.64 (dd, *J* = 14.3, 7.6 Hz, 1H), 2.15 – 1.98 (m, 1H), 1.97 – 1.85 (m, 1H), 1.83 – 1.61 (m, 5H).

^{13}C NMR (101 MHz, Chloroform-*d*) δ 171.78, 159.29, 156.94, 138.87, 134.69, 132.38, 131.07, 130.62, 129.41, 129.14, 128.99, 128.59, 127.72, 126.84, 126.43, 126.39, 123.63, 119.01, 114.47, 106.71, 67.51, 55.55, 39.92, 28.85, 26.61, 23.76.

HRMS (ESI) calcd for $C_{30}H_{32}NO_3^+$ [(M+H)$^+$] 454.2382, found 454.2388.

HPLC analysis: The ee was determined to be 97% on a CHIRALPAK AS-H column (10% *i*PrOH in hexane, 1.0 mL/min, 40 °C); retention times for compound obtained using (*S,S*)-**L1**: 5.3 min (minor), 6.8 min (major).

[α]$_D$ (20.0 °C, c = 1.0 in $CHCl_3$) = 27.0 °; 97% ee, from (*S,S*)-**L1**.

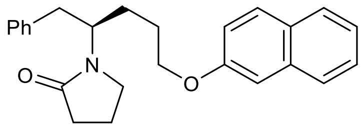

(R)-1-(5-(naphthalen-2-yloxy)-1-phenylpentan-2-yl)pyrrolidin-2-one (**17**)

Following **GP-D1**, **17** was obtained as pale-yellow oil (67.9 mg, 91% yield, 97% ee).

^{1}H NMR (400 MHz, Chloroform-*d*) δ 7.76 – 7.68 (m, 3H), 7.45 – 7.38 (m, 1H), 7.34 – 7.29 (m, 1H), 7.29 – 7.23 (m, 2H), 7.21 – 7.16 (m, 3H), 7.13 – 7.08 (m, 2H), 4.55 – 4.36 (m, 1H), 4.13 – 3.98 (m, 2H), 3.22 (t, J = 7.0 Hz, 2H), 2.91 – 2.73 (m, 2H), 2.37 – 2.23 (m, 1H), 2.26 – 2.13 (m, 1H), 1.92 – 1.69 (m, 6H).

^{13}C NMR (101 MHz, Chloroform-*d*) δ 175.32, 156.85, 138.10, 134.62, 129.39, 128.95, 128.93, 128.48, 127.66, 126.79, 126.50, 126.40, 123.61, 118.94, 106.68, 67.20, 51.93, 42.58, 39.20, 31.47, 28.26, 26.19, 18.30.

HRMS (ESI) calcd for $C_{25}H_{28}NO_2^+$ [(M+H)$^+$] 374.2120, found 374.2117.

HPLC analysis: The ee was determined to be 97% on a CHIRALPAK AD-H column (15% *i*PrOH in hexane, 1.5 mL/min, 40 ºC); retention times for compound obtained using (S,S)-**L1**: 6.3 min (minor), 7.6 min (major).

[α]$_D$ (20.0 ℃, c = 1.0 in $CHCl_3$) = -1.58 º; 97% ee, from (S,S)-**L1**.

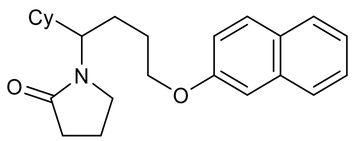

1-(4-(argiooxy)-1-cyclohexylbutyl)pyrrolidin-2-one (**18**)

Following **GP-D1**, **18** was obtained as pale-yellow oil (39.4 mg, 54% yield, <2% ee).

^{1}H NMR (400 MHz, Chloroform-*d*) δ 7.80 – 7.68 (m, 3H), 7.48 – 7.38 (m, 1H), 7.37 – 7.28 (m, 1H), 7.17 – 7.08 (m, 2H), 4.26 – 3.99 (m, 2H), 3.91 – 3.80 (m, 1H), 3.44 – 3.14 (m, 2H), 2.43 (t, J = 8.1 Hz, 2H), 2.05 – 1.88 (m, 3H), 1.87 – 1.80 (m, 1H), 1.79 – 1.69 (m, 4H), 1.66 – 1.49 (m, 3H), 1.43 – 1.33 (m, 1H), 1.29 – 1.10 (m, 3H), 1.08 – 0.93 (m, 2H).

^{13}C NMR (101 MHz, Chloroform-*d*) δ 175.90, 156.96, 134.70, 129.43, 129.00, 127.71, 126.85, 126.44, 123.64, 119.00, 106.77, 67.38, 55.76, 42.51, 39.88, 31.55, 30.43, 30.02, 26.38, 26.17, 26.15, 26.13, 25.49, 18.49.

HRMS (ESI) calcd for $C_{24}H_{32}NO_2^+$ [(M+H)$^+$] 366.2433, found 366.2433.

HPLC analysis: The ee was determined to be <2% on a CHIRALCEL OD-H column (10% *i*PrOH in hexane, 1.0 mL/min, 40 °C); retention times for compound obtained using (*S,S*)-**L1**: 11.9 min (minor), 15.6 min (major).

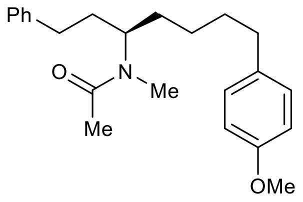

(*R*)-*N*-(7-(4-methoxyphenyl)-1-phenylheptan-3-yl)-*N*-methylacetamide (**19**)

Following **GP-D3**, **19** was obtained as pale-yellow oil (57.9 mg, 82% yield, 89% ee). Mixture of rotational isomers, ratio = 48:52.

^{1}H NMR (400 MHz, Chloroform-*d*) δ 7.34 – 7.23 (m, 2H), 7.25 – 7.11 (m, 3H), 7.07 (dd, *J* = 8.6, 3.2 Hz, 2H), 6.82 (dd, *J* = 8.6, 3.9 Hz, 2H), 4.74 (p, *J* = 7.6 Hz, 0.49H), 3.78 (s, 1.42H), 3.78 (s, 1.51H), 3.68 – 3.51 (m, 0.55H), 2.75 (s, 1.55H), 2.70 (s, 1.47H), 2.67 – 2.41 (m, 4H), 2.07 (s, 1.48H), 1.91 (s, 1.65H), 1.87 – 1.77 (m, 1H), 1.75 – 1.66 (m, 1H), 1.65 – 1.39 (m, 4H), 1.36 – 1.15 (m, 2H).

^{13}C NMR (101 MHz, Chloroform-*d*) δ 171.46, 171.25, 157.84, 157.72, 142.17, 141.05, 134.70, 134.36, 129.35, 129.29, 128.69, 128.45, 128.35, 128.32, 126.30, 125.91, 113.85, 113.76, 57.28, 55.36, 52.36, 34.94, 34.93, 34.57, 34.38, 33.04, 32.99, 32.42, 32.36, 31.75, 31.56, 29.23, 25.98, 25.95, 25.84, 22.53, 21.87.

HRMS (ESI) calcd for $C_{23}H_{32}NO_2^+$ [(M+H)$^+$] 354.2433, found 354.2438.

HPLC analysis: The ee was determined to be 89% on a CHIRALPAK AD-H column (3% *i*PrOH in hexane, 0.3 mL/min, 40 °C); retention times for compound obtained using (*S,S*)-**L1**: 98.4 min (minor), 94.0 min (major).

$[\alpha]_D$ (20.0 °C, c = 1.0 in $CHCl_3$) = 10.46 °, 89% ee, from (*S,S*)-**L1**.

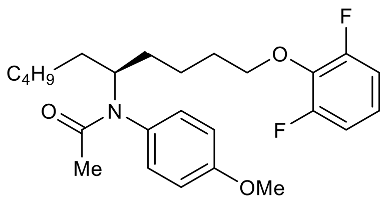

(*S*)-*N*-(1-(2,6-difluorophenoxy)decan-5-yl)-*N*-(4-methoxyphenyl)acetamide (**20**)

Following **GP-D1**, **20** was obtained as yellow oil (62.3 mg, 72% yield, 91% ee).

^{1}H NMR (400 MHz, Chloroform-*d*) δ 7.01 (s, 2H), 6.97 – 6.81 (m, 5H), 4.78 (p, *J* = 6.9 Hz, 1H), 4.25 – 4.04 (m, 2H), 3.82 (s, 3H), 1.86 – 1.61 (m, 6H), 1.60 – 1.47 (m, 1H), 1.44 – 1.18 (m, 10H), 0.97 – 0.79 (m, 3H).

^{13}C NMR (101 MHz, Chloroform-*d*) δ 171.57, 159.19, 156.32 (dd, *J* = 248.0, 5.8 Hz), 135.89 (t, *J* = 14.0 Hz), 132.11, 130.91, 122.58 (t, *J* = 9.3 Hz), 114.47, 112.23 (dd, *J* = 16.6, 6.4 Hz), 74.48 (t, *J* = 3.0 Hz), 55.53, 54.59, 33.35, 32.85, 32.03, 30.04, 26.60, 23.66, 23.00, 22.74, 14.21.

^{19}F NMR (377 MHz, Chloroform-*d*) δ -128.24 (t, *J* = 6.8 Hz).

HRMS (ESI) calcd for $C_{25}H_{34}NO_3F_2^+$ [$(M+H)^+$] 434.2507, found 434.2506.

HPLC analysis: The ee was determined to be 91% on a CHIRALPAK AD-H column (15% *i*PrOH in hexane, 0.5 mL/min, 40 °C); retention times for compound obtained using (*S*,*S*)-**L1**: 24.3 min (minor), 22.9 min (major).

$[\alpha]_D$ (20.0 ℃, c = 1.0 in $CHCl_3$) = 7.47 °; 91% ee, from (*S*,*S*)-**L1**.

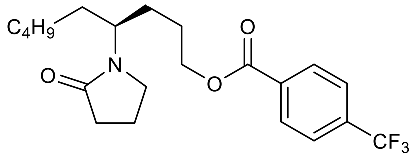

(*S*)-4-(2-oxopyrrolidin-1-yl)nonyl 4-(trifluoromethyl)benzoate (**21**)

Following **GP-D3**, **21** was obtained as pale-yellow oil (75.8 mg, 95% yield, 95% ee).

^{1}H NMR (400 MHz, Chloroform-*d*) δ 8.11 (d, *J* = 8.1 Hz, 2H), 7.67 (d, *J* = 8.2 Hz, 2H), 4.40 – 4.25 (m, 2H), 4.20 – 4.05 (m, 1H), 3.30 – 3.13 (m, 2H), 2.40 (t, *J* = 8.0 Hz, 2H), 1.99 (p, *J* = 7.7 Hz, 2H), 1.80 – 1.62 (m, 2H), 1.61 – 1.50 (m, 2H), 1.48 – 1.37 (m, *J* = 5.7 Hz, 2H), 1.33 – 1.09 (m, 6H), 0.83 (t, *J* = 6.8 Hz, 3H).

^{13}C NMR (101 MHz, Chloroform-*d*) δ 175.50, 165.37, 134.41 (q, *J* = 32.5 Hz), 133.58, 130.00, 125.45 (q, *J* = 3.7 Hz), 123.69 (q, *J* = 272.7 Hz), 65.25, 50.67, 41.71, 32.53, 31.62, 31.54, 28.94, 25.98, 25.68, 22.58, 18.37, 14.04.

^{19}F NMR (377 MHz, Chloroform-*d*) δ -63.12.

HRMS (ESI) calcd for $C_{21}H_{29}NO_3F_3^+$ [(M+H)$^+$] 400.2100, found 400.2094.

HPLC analysis: The ee was determined to be 95% on a CHIRALCEL OD-H column (15% *i*PrOH in hexane, 1.5 mL/min, 40 °C); retention times for compound obtained using (*S,S*)-**L1**: 3.9 min (minor), 3.5 min (major).

$[\alpha]_D$ (20.0 ℃, c = 1.0 in $CHCl_3$) = -2.61 °, 95% ee, from (*S,S*)-**L1**.

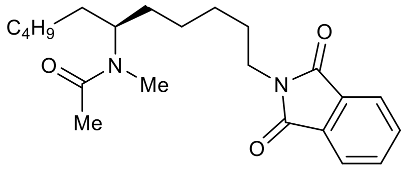

(*S*)-*N*-(1-(1,3-dioxoisoindolin-2-yl)undecan-6-yl)-*N*-methylacetamide (**22**)

Following **GP-D3**, **22** was obtained as colorless oil (44.6 mg, 60% yield, 90% ee).

Mixture of rotational isomers, ratio = 48:52.

^{1}H NMR (400 MHz, Chloroform-*d*) δ 7.86 – 7.76 (m, 2H), 7.74 – 7.64 (m, 2H), 4.58 (p, *J* = 7.7 Hz, 0.48H), 3.70 – 3.59 (m, 2H), 3.61 – 3.50 (m, 0.52H), 2.72 (s, 1.54H), 2.66 (s, 1.60H), 2.08 (s, 1.53H), 2.07 (s, 1.51H), 1.73 – 1.56 (m, 2H), 1.49 – 1.38 (m, 2H), 1.38 – 1.08 (m, 12H), 0.94 – 0.65 (m, 3H).

^{13}C NMR (101 MHz, Chloroform-*d*) δ 171.38, 171.11, 168.53, 134.01, 133.95, 132.22, 132.17, 123.27, 123.23, 58.28, 52.27, 37.95, 37.86, 33.19, 33.14, 32.45, 32.32, 31.83, 29.13, 28.50, 26.86, 26.82, 26.06, 26.00, 25.86, 22.64, 22.61, 22.40, 21.96, 14.12, 14.10.

HRMS (ESI) calcd for $C_{22}H_{33}N_2O_3^+$ [(M+H)$^+$] 373.2391, found 373.2494.

HPLC analysis: The ee was determined to be 90% on a CHIRALPAK AD-H column (7% *i*PrOH in hexane, 0.7 mL/min, 40 °C); retention times for compound obtained using (*S,S*)-**L1**: 27.8 min (minor), 29.3 min (major).

$[\alpha]_D$ (20.0 ℃, c = 1.0 in $CHCl_3$) = -1.50 °, 90% ee, from (*S,S*)-**L1**.

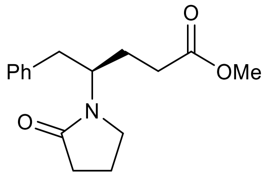

methyl (*R*)-4-(2-oxopyrrolidin-1-yl)-5-phenylpentanoate (**23**)

Following **GP-D1**, **23** was obtained as pale-yellow oil (38.5 mg, 70% yield, 96% ee).

^{1}H NMR (400 MHz, Chloroform-*d*) δ 7.30 – 7.23 (m, 2H), 7.18 (t, *J* = 7.6 Hz, 3H), 4.36 (p, *J* = 8.2 Hz, 1H), 3.64 (s, 3H), 3.21 (t, *J* = 6.9 Hz, 2H), 2.82 (d, *J* = 2.6 Hz, 1H), 2.80 (d, *J* = 3.9 Hz, 1H), 2.38 – 2.14 (m, 4H), 1.98 – 1.77 (m, 4H).

^{13}C NMR (101 MHz, Chloroform-*d*) δ 175.47, 173.68, 137.81, 128.92, 128.56, 126.63, 52.09, 51.79, 42.85, 39.03, 31.42, 31.14, 26.86, 18.34.

HRMS (ESI) calcd for $C_{16}H_{22}NO_3^+$ [(M+H)$^+$] 276.1600, found 276.1597.

HPLC analysis: The ee was determined to be 91% on a CHIRALPAK AD-H column (15% *i*PrOH in hexane, 1.5 mL/min, 40 °C); retention times for compound obtained using (*S*,*S*)-**L1**: 4.7 min (minor), 5.4 min (major).

$[\alpha]_D$ (20.0 °C, c = 1.0 in $CHCl_3$) = -14.59 °, 96% ee, from (*S*,*S*)-**L1**.

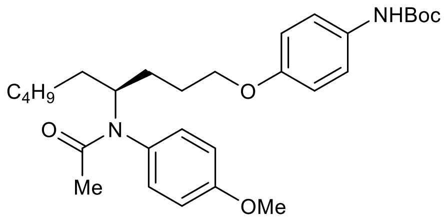

tert-butyl (*S*)-(4-((4-(*N*-(4-methoxyphenyl)acetamido)nonyl)oxy)phenyl)carbamate (**24**)

Following **GP-D1**, **24** was obtained as yellow oil (84.7 mg, 85% yield, 96% ee).

^{1}H NMR (400 MHz, Chloroform-*d*) δ 7.94 (d, *J* = 8.7 Hz, 2H), 7.46 (d, *J* = 8.6 Hz, 2H), 7.12 (s, 1H), 6.99 (d, *J* = 8.6 Hz, 2H), 6.84 (d, *J* = 36.4 Hz, 2H), 4.84 (p, *J* = 7.2 Hz, 1H), 4.41 – 4.18 (m, 2H), 3.79 (s, 3H), 1.98 – 1.79 (m, 2H), 1.77 (s, 3H), 1.50 (s, 9H), 1.46 – 1.33 (m, 4H), 1.32 – 1.16 (m, 6H), 0.91 – 0.70 (m, 3H).

^{13}C NMR (101 MHz, Chloroform-*d*) δ 171.82, 166.37, 159.26, 152.41, 143.05, 131.60, 130.92, 130.82, 124.45, 117.45, 114.78, 114.32, 81.11, 64.66, 55.51, 54.28, 33.49, 31.93, 29.54, 28.35, 26.54, 26.33, 23.60, 22.68, 14.17.

HRMS (ESI) calcd for $C_{29}H_{42}N_2O_5Na^+$ [(M+Na)$^+$] 521.2991, found 521.2988.

HPLC analysis: The ee was determined to be 96% on a CHIRALPAK AD-H column (10% *i*PrOH in hexane, 1.0 mL/min, 40 °C); retention times for compound obtained using (*S,S*)-**L1**: 17.8 min (minor), 30.9 min (major).

[α]$_D$ (20.0 ℃, c = 1.0 in $CHCl_3$) = 10.51 °; 96% ee, from (*S,S*)-**L1**.

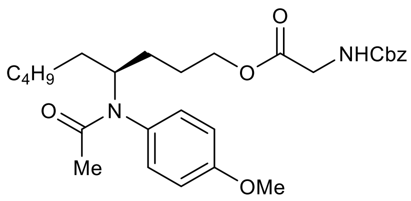

(*S*)-4-(*N*-(4-methoxyphenyl)acetamido)nonyl ((benzyloxy)carbonyl)glycinate (**25**)

Following **GP-D1**, **25** was obtained as pale-yellow oil (89.7 mg, 90% yield, 95% ee).

^{1}H NMR (400 MHz, Chloroform-*d*) δ 7.38 – 7.27 (m, 5H), 6.97 (d, *J* = 8.9 Hz, 2H), 6.91 (d, *J* = 9.0 Hz, 2H), 5.51 (t, *J* = 5.1 Hz, 1H), 5.11 (s, 2H), 4.78 (p, *J* = 7.2 Hz, 1H), 4.22 – 4.07 (m, 2H), 3.96 (d, *J* = 5.7 Hz, 2H), 3.81 (s, 3H), 1.83 – 1.67 (m, 5H), 1.46 – 1.14 (m, 10H), 0.87 (t, *J* = 6.8 Hz, 3H).

^{13}C NMR (101 MHz, Chloroform-*d*) δ 171.70, 170.12, 159.26, 156.40, 136.34, 131.62, 130.77, 128.56, 128.20, 128.12, 114.52, 67.07, 65.45, 55.50, 54.02, 42.85, 33.27, 31.90, 29.44, 26.48, 25.79, 23.57, 22.64, 14.15.

HRMS (ESI) calcd for $C_{28}H_{39}N_2O_6^+$ [(M+H)$^+$] 499.2808, found 499.2813.

HPLC analysis: The ee was determined to be 95% on a CHIRALPAK AD-H column (15% *i*PrOH in hexane, 1.5 mL/min, 40 °C); retention times for compound obtained using (*S,S*)-**L1**: 16.5 min (minor), 13.2 min (major).

[α]$_D$ (20.0 ℃, c = 1.0 in $CHCl_3$) = -0.50 °; 95% ee, from (*S,S*)-**L1**.

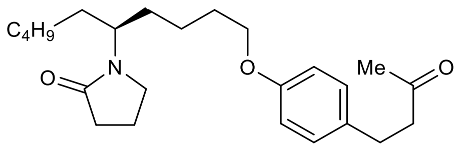

(*S*)-1-(1-(4-(3-oxobutyl)phenoxy)decan-5-yl)pyrrolidin-2-one (**26**)

Following **GP-D1**, **26** was obtained as colorless oil (38.7 mg, 50% yield, 96% ee).

^{1}H NMR (400 MHz, Chloroform-*d*) δ 7.06 (d, *J* = 8.6 Hz, 2H), 6.77 (d, *J* = 8.6 Hz, 2H), 4.07 (p, *J* = 7.4 Hz, 1H), 3.96 – 3.81 (m, 2H), 3.20 (t, *J* = 7.0 Hz, 2H), 2.85 – 2.76 (m, 2H), 2.74 – 2.66 (m, 2H), 2.39 (t, *J* = 8.0 Hz, 2H), 2.11 (s, 3H), 2.04 – 1.91 (m, 2H), 1.87 – 1.75 (m, 1H), 1.75 – 1.61 (m, 1H), 1.54 – 1.34 (m, 5H), 1.32 – 1.10 (m, 7H), 0.85 (t, *J* = 6.9 Hz, 3H).

^{13}C NMR (101 MHz, Chloroform-*d*) δ 208.32, 175.31, 157.45, 132.91, 129.25, 114.52, 67.65, 50.90, 45.53, 41.78, 32.54, 32.23, 31.71, 31.63, 30.20, 29.04, 28.97, 26.03, 22.87, 22.62, 18.39, 14.10.

HRMS (ESI) calcd for $C_{24}H_{38}NO_3^+$ [$(M+H)^+$] 388.2852, found 388.2849.

HPLC analysis: The ee was determined to be 96% on a CHIRALCEL OD-H column (15% *i*PrOH in hexane, 1.5 mL/min, 40 °C); retention times for compound obtained using (*S,S*)-**L1**: 5.9 min (minor), 8.3 min (major).

$[\alpha]_D$ (20.0 °C, c = 1.0 in $CHCl_3$) = -1.26 °; 96% ee, from (*S,S*)-**L1**.

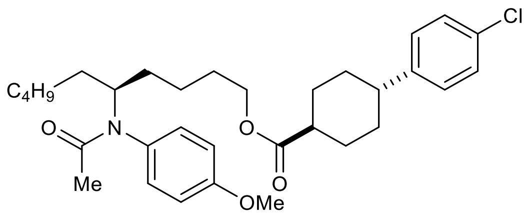

(*S*)-5-(*N*-(4-methoxyphenyl)acetamido)decyl (1*r*,4*S*)-4-(4-chlorophenyl)cyclohexane-1-carboxylate (**27**)

Following **GP-D1**, **27** was obtained as pale-yellow oil (99.6 mg, 92% yield, 96% ee).

^{1}H NMR (400 MHz, Chloroform-*d*) δ 7.23 (d, *J* = 8.4 Hz, 2H), 7.10 (d, *J* = 8.5 Hz, 2H), 6.98 (d, *J* = 8.2 Hz, 2H), 6.90 (d, *J* = 8.9 Hz, 2H), 4.77 (p, *J* = 6.9 Hz, 1H), 4.06 (t, *J* = 6.5 Hz, 2H), 3.82 (s, 3H), 2.47 (tt, *J* = 11.9, 3.4 Hz, 1H), 2.32 (tt, *J* = 12.1, 3.6 Hz, 1H),

2.09 (dd, J = 13.5, 3.6 Hz, 2H), 1.99 – 1.88 (m, 2H), 1.75 (s, 3H), 1.71 – 1.53 (m, 4H), 1.53 – 1.18 (m, 14H), 0.87 (t, J = 6.9 Hz, 3H).

^{13}C NMR (101 MHz, Chloroform-*d*) δ 175.94, 171.50, 159.21, 145.35, 131.95, 131.68, 130.81, 128.52, 128.17, 114.47, 64.24, 55.51, 54.37, 43.05, 43.02, 33.30, 33.23, 32.83, 31.99, 29.29, 28.70, 26.55, 23.63, 23.21, 22.69, 14.19.

HRMS (ESI) calcd for $C_{32}H_{45}NO_4Cl^+$ [(M+H)$^+$] 542.3037, found 542.3040.

HPLC analysis: The ee was determined to be 96% on a CHIRALPAK AD-H column (7% *i*PrOH in hexane, 0.7 mL/min, 40 °C); retention times for compound obtained using (*S*,*S*)-**L1**: 22.4 min (minor), 23.6 min (major).

[α]$_D$ (20.0 ℃, c = 1.0 in $CHCl_3$) = 5.13 °, 96% ee, from (*S*,*S*)-**L1**.

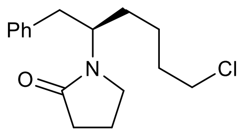

(*R*)-1-(6-chloro-1-phenylhexan-2-yl)pyrrolidin-2-one (**28**)

Following **GP-D1**, **28** was obtained as pale-yellow oil (51.3 mg, 92% yield, 98% ee).

^{1}H NMR (400 MHz, Chloroform-*d*) δ 7.29 – 7.24 (m, 2H), 7.21 – 7.15 (m, 3H), 4.41 – 4.30 (m, 1H), 3.59 – 3.43 (m, 2H), 3.32 – 3.14 (m, 2H), 2.91 – 2.67 (m, 2H), 2.49 – 2.27 (m, 1H), 2.26 – 2.13 (m, 1H), 1.98 – 1.76 (m, 3H), 1.75 – 1.64 (m, 1H), 1.64 – 1.46 (m, 2H), 1.46 – 1.30 (m, 2H).

^{13}C NMR (101 MHz, Chloroform-*d*) δ 175.27, 138.15, 128.93, 128.50, 126.52, 52.08, 44.91, 42.77, 39.13, 32.10, 31.51, 30.96, 23.58, 18.39.

HRMS (ESI) calcd for $C_{16}H_{23}NOCl^+$ [(M+H)$^+$] 280.1468, found 280.1469.

HPLC analysis: The ee was determined to be 98% on a CHIRALPAK AS-H column (15% *i*PrOH in hexane, 1.5 mL/min, 40 °C); retention times for compound obtained using (*S*,*S*)-**L1**: 2.9 min (minor), 3.8 min (major).

[α]$_D$ (20.0 ℃, c = 1.0 in $CHCl_3$) = -6.00 °, 98% ee, from (*S*,*S*)-**L1**.

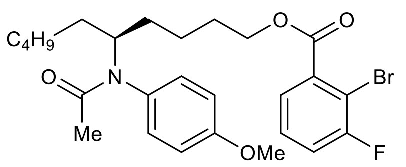

(S)-5-(N-(4-methoxyphenyl)acetamido)decyl 2-bromo-3-fluorobenzoate (**29**)

Following **GP-D1**, **29** was obtained as brown oil (93.8 mg, 90% yield, 96% ee).

^{1}H NMR (400 MHz, Chloroform-d) δ 7.56 (dt, J = 7.7, 1.3 Hz, 1H), 7.33 (td, J = 8.0, 5.1 Hz, 1H), 7.26 – 7.20 (m, 1H), 6.98 (d, J = 8.4 Hz, 2H), 6.92 – 6.83 (m, 2H), 4.78 (p, J = 6.8 Hz, 1H), 4.34 (t, J = 6.5 Hz, 2H), 3.82 (s, 3H), 1.89 – 1.79 (m, 2H), 1.75 (s, 3H), 1.65 – 1.49 (m, 2H), 1.36 (q, J = 7.5 Hz, 4H), 1.32 – 1.17 (m, 6H), 0.87 (t, J = 6.4 Hz, 3H).

^{13}C NMR (101 MHz, Chloroform-d) δ 171.61, 165.63 (d, J = 2.9 Hz), 159.61 (d, J = 247.2 Hz), 159.23, 134.74, 131.95, 130.83, 129.26, 128.56 (d, J = 8.0 Hz), 126.66 (d, J = 3.6 Hz), 119.13 (d, J = 23.5 Hz), 114.80, 114.51, 109.44 (d, J = 22.1 Hz), 65.96, 55.55, 54.40, 33.29, 32.81, 32.01, 28.61, 26.59, 23.67, 23.35, 22.72, 14.21.

^{19}F NMR (471 MHz, Chloroform-d) δ -102.97 (dd, J = 8.5, 5.3 Hz).

HRMS (ESI) calcd for $C_{26}H_{34}NO_4BrF^+$ $[(M+H)^+]$ 522.1655, found 522.1661.

HPLC analysis: The ee was determined to be 96% on a CHIRALPAK AD-H column (10% iPrOH in hexane, 1.0 mL/min, 40 °C); retention times for compound obtained using (S,S)-**L1**: 14.3 min (minor), 11.4 min (major).

$[\alpha]_D$ (20.0 °C, c = 1.0 in $CHCl_3$) = 2.65 °, 96% ee, from (S,S)-**L1**.

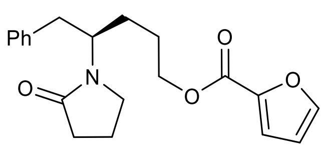

(R)-4-(2-oxopyrrolidin-1-yl)-5-phenylpentyl furan-2-carboxylate (**30**)

Following **GP-D3**, **30** was obtained as pale-yellow oil (55.9 mg, 82% yield, 97% ee).

^{1}H NMR (400 MHz, Chloroform-d) δ 7.56 (d, J = 1.6 Hz, 1H), 7.30 – 7.21 (m, 2H), 7.22 – 7.14 (m, 3H), 7.13 (d, J = 3.5 Hz, 1H), 6.49 (dd, J = 3.5, 1.7 Hz, 1H), 4.41 (p, J = 7.9 Hz, 1H), 4.27 (td, J = 6.1, 2.4 Hz, 2H), 3.33 – 3.14 (m, 2H), 2.87 – 2.71 (m, 2H), 2.50 – 2.27 (m, 1H), 2.26 – 2.14 (m, 1H), 2.04 – 1.78 (m, 2H), 1.76 – 1.57 (m, 4H).

^{13}C NMR (101 MHz, Chloroform-*d*) δ 175.38, 158.78, 146.38, 144.71, 137.99, 128.92, 128.51, 126.55, 117.99, 111.93, 64.48, 52.07, 42.74, 39.11, 31.46, 28.10, 25.79, 18.36.

HRMS (ESI) calcd for $C_{20}H_{24}NO_4^+$ [(M+H)$^+$] 342.1075, found 342.1078.

HPLC analysis: The ee was determined to be 97% on a CHIRALPAK AS-H column (15% *i*PrOH in hexane, 1.5 mL/min, 40 °C); retention times for compound obtained using (*S*,*S*)-**L1**: 4.4 min (minor), 7.1 min (major).

$[\alpha]_D$ (20.0 °C, c = 1.0 in $CHCl_3$) = -5.73 °; 97% ee, from (*S*,*S*)-**L1**.

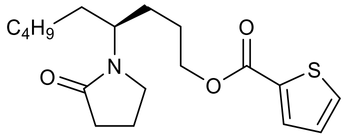

(*S*)-4-(2-oxopyrrolidin-1-yl)nonyl thiophene-2-carboxylate (**31**)

Following **GP-D3**, **31** was obtained as pale-yellow oil (61.3 mg, 91% yield, 97% ee).

^{1}H NMR (400 MHz, Chloroform-*d*) δ 7.77 (dd, *J* = 3.8, 1.3 Hz, 1H), 7.53 (dd, *J* = 5.0, 1.3 Hz, 1H), 7.08 (dd, *J* = 5.0, 3.7 Hz, 1H), 4.33 – 4.19 (m, 2H), 4.17 – 4.04 (m, 1H), 3.22 (t, *J* = 7.0 Hz, 2H), 2.40 (t, *J* = 8.0 Hz, 2H), 1.99 (p, *J* = 7.5 Hz, 2H), 1.77 – 1.48 (m, 4H), 1.48 – 1.37 (m, 2H), 1.34 – 1.10 (m, 6H), 0.84 (d, *J* = 6.9 Hz, 3H).

^{13}C NMR (101 MHz, Chloroform-*d*) δ 175.50, 162.27, 133.88, 133.45, 132.38, 127.84, 64.80, 50.76, 41.72, 32.59, 31.65, 31.58, 28.88, 25.99, 25.77, 22.60, 18.37, 14.08.

HRMS (ESI) calcd for $C_{18}H_{28}NO_3S^+$ [(M+H)$^+$] 338.1970, found 338.1973.

HPLC analysis: The ee was determined to be 97% on a CHIRALCEL OD-H column (15% *i*PrOH in hexane, 1.5 mL/min, 40 °C); retention times for compound obtained using (*S*,*S*)-**L1**: 4.1 min (minor), 4.8 min (major).

$[\alpha]_D$ (20.0 °C, c = 1.0 in $CHCl_3$) = -4.23 °; 97% ee, from (*S*,*S*)-**L1**.

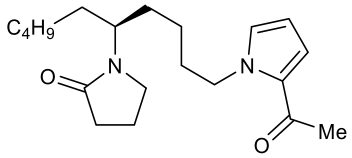

(*S*)-1-(1-(2-acetyl-1*H*-pyrrol-1-yl)decan-5-yl)pyrrolidin-2-one (**32**)

Following **GP-D1**, **32** was obtained as brown oil (46.5 mg, 70% yield, 97% ee).

^{1}H NMR (400 MHz, Chloroform-*d*) δ 6.93 (dd, *J* = 4.0, 1.7 Hz, 1H), 6.85 – 6.79 (m, 1H), 6.09 (dd, *J* = 4.1, 2.5 Hz, 1H), 4.33 – 4.17 (m, 2H), 4.01 (p, *J* = 7.6 Hz, 1H), 3.23 – 3.09 (m, 2H), 2.40 (s, 3H), 2.41 – 2.32 (m, 2H), 1.95 (p, *J* = 7.3 Hz, 2H), 1.82 – 1.58 (m, 2H), 1.48 – 1.34 (m, 4H), 1.30 – 1.10 (m, 8H), 0.84 (t, *J* = 6.9 Hz, 3H).

^{13}C NMR (101 MHz, Chloroform-*d*) δ 188.34, 175.35, 130.34, 130.08, 120.41, 108.02, 50.94, 49.67, 41.80, 32.52, 32.15, 31.70, 31.62, 31.28, 27.43, 26.03, 23.39, 22.62, 18.37, 14.11.

HRMS (ESI) calcd for $C_{20}H_{33}N_2O_2^+$ [(M+H)$^+$] 333.2542, found 333.2537.

HPLC analysis: The ee was determined to be 97% on a CHIRALCEL OD-H column (15% *i*PrOH in hexane, 1.5 mL/min, 40 °C); retention times for compound obtained using (*S,S*)-**L1**: 4.1 min (minor), 3.6 min (major).

$[\alpha]_D$ (20.0 ℃, c = 1.0 in $CHCl_3$) = -0.38 °; 97% ee, from (*S,S*)-**L1**.

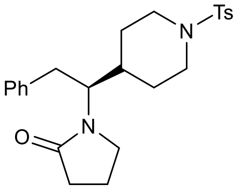

(*S*)-1-(2-phenyl-1-(1-tosylpiperidin-4-yl)ethyl)pyrrolidin-2-one (**33**)

Following **GP-D4**, **33** was obtained as yellow oil (59.6 mg, 70% yield, 87% ee).

^{1}H NMR (500 MHz, Chloroform-*d*) δ 7.63 (d, *J* = 8.1 Hz, 2H), 7.32 (d, *J* = 8.0 Hz, 2H), 7.24 (d, *J* = 7.4 Hz, 2H), 7.17 (t, *J* = 7.3 Hz, 1H), 7.12 (d, *J* = 7.3 Hz, 2H), 3.98 – 3.71 (m, 3H), 3.11 (q, *J* = 8.5 Hz, 1H), 2.98 (dd, *J* = 14.2, 4.6 Hz, 1H), 2.89 (q, *J* = 7.9, 7.4 Hz, 1H), 2.80 – 2.70 (m, 1H), 2.43 (s, 3H), 2.35 – 2.17 (m, 3H), 2.14 – 2.03 (m, 1H), 1.89 (d, *J* = 13.1 Hz, 1H), 1.80 – 1.62 (m, 4H), 1.53 – 1.34 (m, 2H).

^{13}C NMR (126 MHz, Chloroform-*d*) δ 175.44, 143.67, 138.07, 133.12, 129.73, 128.76, 128.53, 127.88, 126.61, 57.89, 46.33, 46.13, 45.81, 37.94, 35.37, 31.50, 29.22, 28.78, 21.65, 18.65.

HRMS (ESI) calcd for $C_{24}H_{31}N_2O_3S^+$ [(M+H)$^+$] 427.2055, found 427.2053.

HPLC analysis: The ee was determined to be 87% on a CHIRALCEL OD-H column (15% *i*PrOH in hexane, 1.5 mL/min, 40 °C); retention times for compound obtained

using (*S*,*S*)-**L1**: 20.2 min (minor), 17.5 min (major).

[α]D (20.0 ℃, c = 1.0 in $CHCl_3$) = -26.98 °; 87% ee, from (*S*,*S*)-**L1**.

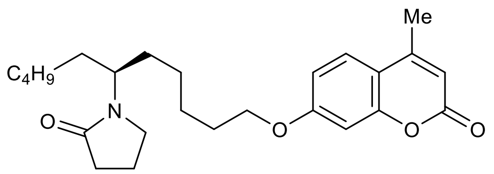

(*S*)-1-(1-((4-methyl-2-oxo-2*H*-chromen-7-yl)oxy)undecan-6-yl)pyrrolidin-2-one (**34**)

Following **GP-D3**, **34** was obtained as pale-yellow oil (72.7 mg, 88% yield, 96% ee).

^{1}H NMR (400 MHz, Chloroform-*d*) δ 7.45 (d, *J* = 8.8 Hz, 1H), 6.81 (dd, *J* = 8.8, 2.6 Hz, 1H), 6.74 (d, *J* = 2.5 Hz, 1H), 6.08 (d, *J* = 1.3 Hz, 1H), 4.04 (p, *J* = 7.5 Hz, 1H), 3.96 (t, *J* = 6.4 Hz, 2H), 3.19 (t, *J* = 7.0 Hz, 2H), 2.43 – 2.31 (m, 5H), 1.96 (p, *J* = 7.7 Hz, 2H), 1.76 (p, *J* = 6.7 Hz, 2H), 1.55 – 1.34 (m, 6H), 1.33 – 1.11 (m, 8H), 0.83 (t, *J* = 6.9 Hz, 3H).

^{13}C NMR (101 MHz, Chloroform-*d*) δ 175.32, 162.20, 161.43, 155.28, 152.71, 125.56, 113.46, 112.60, 111.81, 101.41, 68.47, 50.88, 41.76, 32.54, 32.46, 31.68, 31.62, 28.97, 26.10, 26.01, 25.85, 22.60, 18.74, 18.37, 14.08.

HRMS (ESI) calcd for $C_{25}H_{36}NO_4^+$ [$(M+H)^+$] 414.2644, found 414.2640.

HPLC analysis: The ee was determined to be 96% on a CHIRALCEL OD-H column (15% *i*PrOH in hexane, 1.5 mL/min, 40 °C); retention times for compound obtained using (*S*,*S*)-**L1**: 14.7 min (minor), 12.7 min (major).

[α]D (20.0 ℃, c = 1.0 in $CHCl_3$) = -1.70 °; 96% ee, from (*S*,*S*)-**L1**.

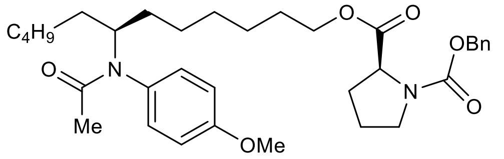

1-benzyl 2-((*S*)-7-(*N*-(4-methoxyphenyl)acetamido)dodecyl) (*S*)-pyrrolidine-1,2-dicarboxylate (**35**)

Following **GP-D1**, **35** was obtained as yellow oil (74.2 mg, 64% yield, 97:3 dr).

^{1}H NMR (400 MHz, Chloroform-*d*) δ 7.38 – 7.32 (m, 2H), 7.32 – 7.26 (m, 3H), 6.98 (d, *J* = 9.0 Hz, 2H), 6.90 (d, *J* = 8.0 Hz, 2H), 5.15 (dd, *J* = 14.0, 12.5 Hz, 1H), 5.07 (dd, *J* = 13.9, 12.5 Hz, 1H), 4.75 (p, *J* = 6.8 Hz, 1H), 4.34 (ddd, *J* = 20.7, 8.6, 3.5 Hz, 1H), 4.11 (t, *J* = 6.7 Hz, 1H), 4.04 – 3.91 (m, 1H), 3.82 (s, 3H), 3.67 – 3.56 (m, 1H), 3.56 – 3.39 (m, 1H), 2.28 – 2.15 (m, 1H), 2.06 – 1.83 (m, 4H), 1.75 (s, 3H), 1.69 – 1.57 (m, 1H), 1.55 – 1.45 (m, 1H), 1.42 – 1.18 (m, 15H), 0.87 (t, *J* = 6.5 Hz, 3H).

^{13}C NMR (101 MHz, Chloroform-*d*) δ 172.99, 172.80, 159.17, 154.94, 154.40, 136.83, 136.69, 132.06, 130.88, 128.53, 128.47, 128.03, 127.97, 127.94, 127.82, 114.44, 67.04, 67.00, 65.26, 65.22, 59.38, 59.06, 55.53, 54.60, 54.57, 47.01, 46.51, 33.29, 33.24, 32.02, 31.07, 30.07, 29.39, 29.35, 28.63, 28.54, 26.77, 26.76, 26.58, 25.89, 25.85, 24.38, 23.70, 23.61, 22.72, 14.21.

HRMS (ESI) calcd for $C_{34}H_{48}N_2O_6Na^+$ $[(M+Na)^+]$ 603.3434, found 603.3425.

HPLC analysis: The dr was determined to be 97:3 on a CHIRALCEL OD-H column (7% *i*PrOH in hexane, 0.7 mL/min, 40 °C); retention times for compound obtained using (*S*,*S*)-**L1**: 49.1 min (minor), 44.6 min (major).

$[\alpha]_D$ (20.0 °C, c = 1.0 in $CHCl_3$) = 26.09 °; 97:3 dr, from (*S*,*S*)-**L1**.

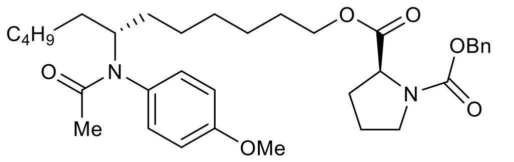

1-benzyl 2-((*R*)-7-(*N*-(4-methoxyphenyl)acetamido)dodecyl) (*S*)-pyrrolidine-1,2-dicarboxylate (**36**)

Following **GP-D1**, **36** was obtained as yellow oil (69.6 mg, 60% yield, 5:95 dr).

^{1}H NMR (400 MHz, Chloroform-*d*) δ 7.39 – 7.33 (m, 2H), 7.33 – 7.27 (m, 3H), 6.98 (d, *J* = 8.5 Hz, 2H), 6.91 (d, *J* = 8.5 Hz, 2H), 5.15 (dd, *J* = 14.8, 12.5 Hz, 1H), 5.08 (t, *J* = 12.9 Hz, 1H), 4.74 (p, *J* = 7.0 Hz, 1H), 4.35 (ddd, *J* = 20.3, 8.6, 3.5 Hz, 1H), 4.12 (tt, *J* = 6.7, 2.9 Hz, 1H), 3.97 (td, *J* = 6.7, 3.6 Hz, 1H), 3.83 (s, 3H), 3.70 – 3.57 (m, 1H), 3.56 – 3.43 (m, 1H), 2.31 – 2.15 (m, 1H), 2.08 – 1.85 (m, 4H), 1.76 (s, 3H), 1.69 – 1.58 (m, 1H), 1.56 – 1.46 (m, 1H), 1.45 – 1.17 (m, 15H), 0.88 (t, *J* = 6.8 Hz, 3H).

^{13}C NMR (101 MHz, Chloroform-*d*) δ 173.02, 172.83, 171.64, 159.22, 154.97, 154.43, 136.85, 136.72, 132.02, 130.86, 128.56, 128.49, 128.05, 128.00, 127.97, 127.85, 114.48, 67.07, 67.03, 65.28, 65.25, 59.41, 59.09, 55.56, 54.77, 54.68, 47.04, 46.54, 33.26, 32.05, 31.10, 30.10, 29.40, 29.38, 28.64, 28.56, 26.79, 26.61, 25.88, 24.41, 23.65, 23.64, 22.74, 14.23.

HRMS (ESI) calcd for $C_{34}H_{49}N_2O_6^+$ [$(M+H)^+$] 581.3591, found 581.3597.

HPLC analysis: The dr was determined to be 5:95 on a CHIRALCEL OD-H column (7% *i*PrOH in hexane, 0.7 mL/min, 40 °C); retention times for compound obtained using (*R*,*R*)-**L1**: 44.2 min (minor), 47.6 min (major).

$[\alpha]_D$ (20.0 °C, c = 1.0 in $CHCl_3$) = 19.17 °; 5:95 dr, from (*R*,*R*)-**L1**.

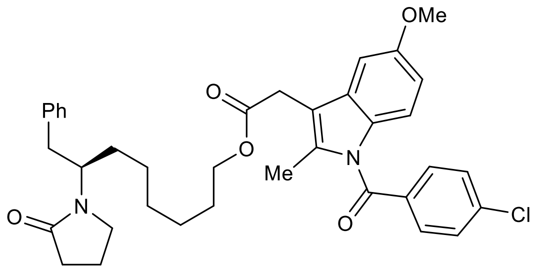

(*R*)-7-(2-oxopyrrolidin-1-yl)-8-phenyloctyl 2-(1-(4-chlorobenzoyl)-5-methoxy-2-methyl-1*H*-indol-3-yl)acetate (**37**)

Following **GP-D1**, **37** was obtained as yellow oil (113.1 mg, 90% yield, 97% ee).

^{1}H NMR (400 MHz, Chloroform-*d*) δ 7.65 (d, *J* = 8.5 Hz, 2H), 7.46 (d, *J* = 8.4 Hz, 2H), 7.31 – 7.22 (m, 2H), 7.22 – 7.13 (m, 3H), 6.95 (d, *J* = 2.5 Hz, 1H), 6.86 (d, *J* = 8.9 Hz, 1H), 6.65 (dd, *J* = 9.0, 2.5 Hz, 1H), 4.34 (p, *J* = 8.4 Hz, 1H), 4.06 (t, *J* = 6.6 Hz, 2H), 3.82 (s, 3H), 3.64 (s, 2H), 3.31 – 3.11 (m, 2H), 2.95 – 2.68 (m, 2H), 2.37 (s, 3H), 2.36 – 2.14 (m, 2H), 2.01 – 1.79 (m, 2H), 1.66 – 1.53 (m, 2H), 1.54 – 1.41 (m, 2H), 1.38 – 1.12 (m, 6H).

^{13}C NMR (101 MHz, Chloroform-*d*) δ 175.26, 171.02, 168.35, 156.07, 139.30, 138.24, 135.93, 133.98, 131.24, 130.84, 130.74, 129.19, 128.92, 128.45, 126.46, 115.00, 112.80, 111.71, 101.35, 65.14, 55.76, 52.30, 42.79, 39.10, 31.78, 31.52, 30.47, 29.02, 28.57, 26.32, 25.86, 18.37, 13.47.

HRMS (ESI) calcd for $C_{37}H_{42}N_2O_5Cl^+$ [$(M+H)^+$] 629.2782, found 629.2787.

HPLC analysis: The ee was determined to be 97% on a CHIRALPAK AS-H column (20% *i*PrOH in hexane, 2.0 mL/min, 40 °C); retention times for compound obtained using (*S*,*S*)-**L1**: 3.8 min (minor), 6.4 min (major).

$[\alpha]_D$ (20.0 ℃, c = 1.0 in $CHCl_3$) = -2.83 °; 97% ee, from (*S*,*S*)-**L1**.

Reductive Hydroalkylation of Secondary Enamides and Enecarbamates

Supplementary Table 2. Optimization of the Reaction Conditions for Secondary Enamides and Enecarbamates

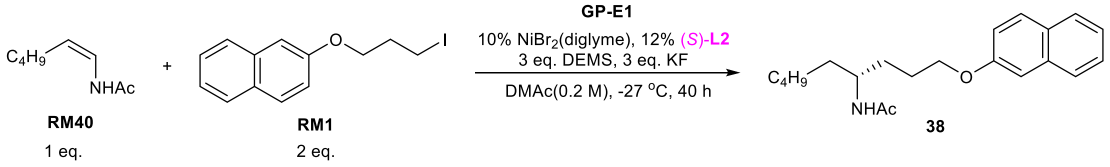

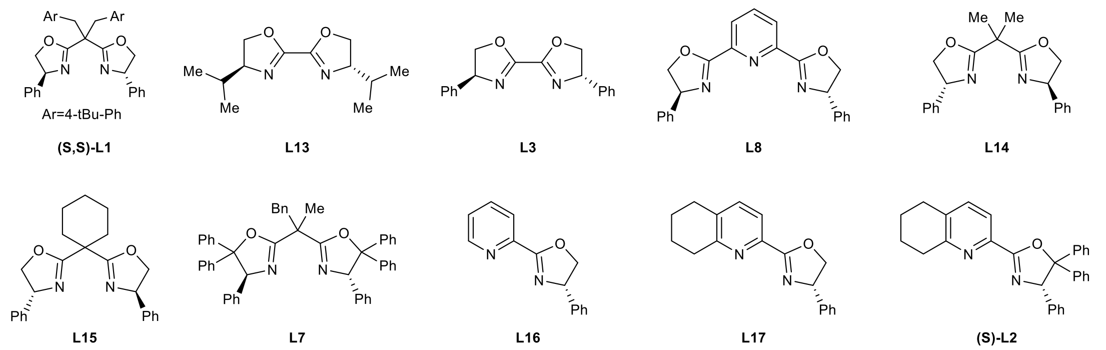

entry	nickel source	ligand	silane	base	solvent	temperature	yield of **38** (%)	e.e. of **38** (%)
1	$NiBr_2$(diglyme)	(*S*)-**L2**	DEMS	KF	DMAc	-27 °C	**95 (90[b])**	**90**
2	$NiBr_2$(diglyme)	(S,*S*)-**L1**	DEMS	KF	DMAc	25 °C	70	45
2	$NiBr_2$(diglyme)	**L13**	DEMS	KF	DMAc	25 °C	37	70
3	$NiBr_2$(diglyme)	**L3**	DEMS	KF	DMAc	25 °C	45	5
4	$NiBr_2$(diglyme)	**L8**	DEMS	KF	DMAc	25 °C	36	17
5	$NiBr_2$(diglyme)	**L14**	DEMS	KF	DMAc	25 °C	49	56
6	$NiBr_2$(diglyme)	**L15**	DEMS	KF	DMAc	25 °C	49	39
7	$NiBr_2$(diglyme)	**L7**	DEMS	KF	DMAc	25 °C	49	15
8	$NiBr_2$(diglyme)	**L16**	DEMS	KF	DMAc	25 °C	42	20
9	$NiBr_2$(diglyme)	**L17**	DEMS	KF	DMAc	25 °C	78	77
10	$NiBr_2$(diglyme)	(*S*)-**L2**	$(EtO)_3SiH$	KF	DMAc	25 °C	75	70
11	$NiBr_2$(diglyme)	(*S*)-**L2**	$MeEt_2SiH$	KF	DMAc	25 °C	trace	N.D
12	$NiBr_2$(diglyme)	(*S*)-**L2**	DEMS	$K_3PO_4(H_2O)$	DMAc	25 °C	57	71

13	$NiBr_2$(diglyme)	(*S*)-**L2**	DEMS	K_3PO_4	DMAc	25 °C	81	73
14	$NiBr_2$(diglyme)	(*S*)-**L2**	DEMS	$KHCO_3$	DMAc	25 °C	92	76
15	$NiBr_2$(diglyme)	(*S*)-**L2**	DEMS	Na_2CO_3	DMAc	25 °C	80	76
16	$NiBr_2$(diglyme)	(*S*)-**L2**	DEMS	CsF	DMAc	25 °C	68	76
17	$NiBr_2$(diglyme)	(*S*)-**L2**	DEMS	K_2CO_3	DMAc	25 °C	83	76
18	$NiBr_2$(diglyme)	(*S*)-**L2**	DEMS	Cs_2CO_3	DMAc	25 °C	56	76
19	$NiBr_2$(diglyme)	(*S*)-**L2**	DEMS	KF	DMF	25 °C	78	71
20	$NiBr_2$(diglyme)	(*S*)-**L2**	DEMS	KF	NMP	25 °C	73	72
21	$NiBr_2$(diglyme)	(*S*)-**L2**	DEMS	KF	2-Me THF	25 °C	79	52
22	$NiBr_2$(diglyme)	(*S*)-**L2**	DEMS	KF	DCE	25 °C	3	19
23	$NiBr_2$(diglyme)	(*S*)-**L2**	DEMS	KF	CH_3CN	25 °C	80	31
24	$NiBr_2$(diglyme)	(*S*)-**L2**	DEMS	KF	1,4-dioxane	25 °C	trace	N.D.
25	$NiBr_2$(diglyme)	(*S*)-**L2**	DEMS	KF	toluene	25 °C	3	53
26	$NiBr_2$(diglyme)	(*S*)-**L2**	DEMS	KF	DMAc	25 °C	96	78
27	$NiBr_2$(diglyme)	(*S*)-**L2**	DEMS	KF	DMAc	-27 °C	95	90

[a] Conditions: **RM40** (0.2 mmol, 1.0 equiv.), **RM1** (0.4 mmol, 2.0 equiv.), nickel source (0.02 mmol, 10 mol%), ligand (0.024 mmol, 12 mol%), silane (0.6 mmol, 3 equiv.), base (0.6 mmol, 3 equiv.), solvent (1.0 mL), -27 °C, 40 h. Triphenylmethane was used as an internal standard. GC yield. [b] Isolated yield. Bn = benzyl. Glyme = 1,2-dimethoxyethane. DCE = 1,2-dichloroethane. Diglyme = 2-methoxyethyl ether. THF = tetrahydrofuran. DMAc = *N*,*N*-dimethylacetamide. DEMS = diethoxymethylsilane. e.e. = enantiomeric excess. N.D. = not detected.

In the air, a 10 mL screw-cap test tube equipped with a magnetic stirrer was charged with nickel source (0.02 mmol, 10 mol%), ligand (0.024 mmol, 12 mol%). The test tube was evacuated and backfilled with argon for three times, then solvent (1.0 mL) was added and the mixture was stirred at room temperature for 30 min.

Meanwhile, in the air, another 10 mL screw-cap test tube equipped with a magnetic stirrer was charged with base (0.6 mmol, 3.0 equiv.) (if enamide/enecarbamate or alkyl halide is a solid, it was also added at this time). The test tube was evacuated and

backfilled with argon for three times, and the test tube was then placed in an EtOH cooling bath at –27 °C. Next, the solution of the catalyst (see above) was added in one portion via syringe, the reaction mixture was stirred at –27 °C for 10 min, and then the enamide/enecarbamate (0.2 mmol, 1.0 equiv.) and alkyl halide (0.4 mmol, 2.0 equiv.) was added dropwise via syringe, silane (0.6 mmol, 3.0 equiv.) was added dropwise via syringe over 1 min. The reaction mixture was stirred at –27 °C for 40 h. The GC yield was determined using triphenylmethane as an internal standard.

General Procedure for Asymmetric Reductive Hydroalkylation of Secondary Enamides and Enecarbamates

General Procedure E1 (GP-E1): Asymmetric Reductive Hydroalkylation with Primary Alkyl Iodides (Fig. 3). In the air, a 10 mL screw-cap test tube equipped with a magnetic stirrer was charged with $NiBr_2$(diglyme) (0.02 mmol, 10 mol%), ligand (0.024 mmol, 12 mol%). The test tube was evacuated and backfilled with argon for three times, then DMAc (1.0 mL) was added and the mixture was stirred at room temperature for 30 min.

Meanwhile, in the air, another 10 mL screw-cap test tube equipped with a magnetic stirrer was charged with KF (0.6 mmol, 3.0 equiv.) (if alkyl halide is a solid, it was also added at this time). The test tube was evacuated and backfilled with argon for three times, and the test tube was then placed in an EtOH cooling bath at –27 °C. Next, the solution of the catalyst (see above) was added in one portion via syringe, the reaction mixture was stirred at –27 °C for 10 min, and then DEMS (0.6 mmol, 3.0 equiv.) was added dropwise via syringe over 1 min, the reaction mixture was stirred for another 5 min, followed by the enamide/enecarbamate (0.2 mmol, 1.0 equiv.) and alkyl halide (0.4 mmol, 2.0 equiv.). The reaction mixture was stirred at –27 °C for 40 h. The reaction mixture was diluted with H_2O followed by extraction with EtOAc, dried with anhydrous Na_2SO_4 and concentrated in vacuo. The residue was purified by flash column chromatography on silica gel to give the target product.

General Procedure E2 (GP-E2): Asymmetric Reductive Hydroalkylation with Primary Alkyl Iodides (Fig. 3&6). In the air, a 10 mL screw-cap test tube equipped with a magnetic stirrer was charged with $NiBr_2$(diglyme) (0.02 mmol, 10 mol%), ligand (0.024 mmol, 12 mol%). The test tube was evacuated and backfilled with argon for three times, then DMAc (1.0 mL) was added and the mixture was stirred at room temperature for 30 min.

Meanwhile, in the air, another 10 mL screw-cap test tube equipped with a magnetic stirrer was charged with KF (0.6 mmol, 3.0 equiv.) (if enamide/enecarbamate or halide is a solid, it was also added at this time). The test tube was evacuated and backfilled with argon for three times, and the test tube was then placed in an EtOH cooling bath at –27 °C. Next, the solution of the catalyst (see above) was added in one portion via syringe, the reaction mixture was stirred at –27 °C for 10 min, and then the enamide/enecarbamate (0.2 mmol, 1.0 equiv.) and alkyl halide (0.4 mmol, 2.0 equiv.) was added dropwise via syringe, DEMS (0.6 mmol, 3.0 equiv.) was added dropwise via syringe over 1 min. The reaction mixture was stirred at –27 °C for 40 h. The reaction mixture was diluted with H_2O followed by extraction with EtOAc, dried with anhydrous Na_2SO_4 and concentrated in vacuo. The residue was purified by flash column chromatography on silica gel to give the target product.

General Procedure E3 (GP-E3): Asymmetric Reductive Hydroalkylation with Primary Alkyl Iodides (Fig. 3). In the air, a 10 mL screw-cap test tube equipped with a magnetic stirrer was charged with $NiBr_2$(diglyme) (0.02 mmol, 10 mol%), ligand (0.024 mmol, 12 mol%). The test tube was evacuated and backfilled with argon for three times, then DMAc (1.0 mL) was added and the mixture was stirred at room temperature for 30 min.

Meanwhile, in the air, another 10 mL screw-cap test tube equipped with a magnetic stirrer was charged with KF (0.6 mmol, 3.0 equiv.) (if enamide/enecarbamate or halide is a solid, it was also added at this time). The test tube was evacuated and backfilled

with argon for three times, and the test tube was then placed in an EtOH cooling bath at 0 °C. Next, the solution of the catalyst (see above) was added in one portion via syringe, the reaction mixture was stirred at 0 °C for 10 min, and then the enamide/enecarbamate (0.2 mmol, 1.0 equiv.) and alkyl halide (0.4 mmol, 2.0 equiv.) was added dropwise via syringe, DEMS (0.6 mmol, 3.0 equiv.) was added dropwise via syringe over 1 min. The reaction mixture was stirred at 0 °C for 40 h. The reaction mixture was diluted with H_2O followed by extraction with EtOAc, dried with anhydrous Na_2SO_4 and concentrated in vacuo. The residue was purified by flash column chromatography on silica gel to give the target product.

Examples Described in Figure 3

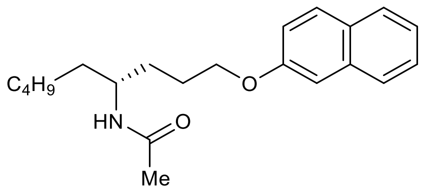

(*R*)-*N*-(1-(naphthalen-2-yloxy)nonan-4-yl)acetamide (**38**)

Following **GP-E1**, **38** was obtained as white solid (58.9 mg, 90%, 90% ee).

^{1}H NMR (500 MHz, Chloroform-*d*) δ 7.81 – 7.65 (m, 3H), 7.47 – 7.39 (m, 1H), 7.36 – 7.30 (m, 1H), 7.17 – 7.08 (m, 2H), 5.39 (d, *J* = 9.1 Hz, 1H), 4.08 (t, *J* = 6.2 Hz, 2H), 4.04 – 3.95 (m, 1H), 1.98 (s, 3H), 1.93 – 1.80 (m, 2H), 1.80 – 1.70 (m, 1H), 1.60 – 1.47 (m, 2H), 1.44 – 1.21 (m, 7H), 0.88 (t, *J* = 6.7 Hz, 3H).

^{13}C NMR (126 MHz, Chloroform-*d*) δ 169.78, 156.85, 134.57, 129.35, 128.90, 127.61, 126.72, 126.34, 123.55, 118.86, 106.63, 67.55, 49.08, 35.35, 31.80, 31.75, 25.76, 25.57, 23.50, 22.58, 14.04.

HRMS (ESI) calcd for $C_{21}H_{30}NO_2^+$ [$(M+H)^+$] 328.2271, found 328.2272.

HPLC analysis: The ee was determined to be 90% on a CHIRALPAK AD-H column (10% *i*PrOH in hexane, 1.0 mL/min, 40 °C); retention times for compound obtained using (*S*)-**L2**: 6.7 min (minor), 7.1 min (major).

$[\alpha]_D$ (20.0 °C, c = 1.0 in $CHCl_3$) = +7.46°; 90% ee, from (*S*)-**L2**.

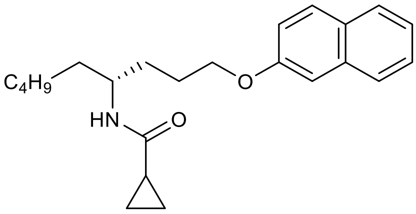

(*R*)-*N*-(1-(naphthalen-2-yloxy)nonan-4-yl)cyclopropanecarboxamide (**39**)

Following **GP-E1**, **39** was obtained as white solid (56.5 mg,80%, 93% ee).

^{1}H NMR (500 MHz, Chloroform-*d*) δ 7.82 – 7.68 (m, 3H), 7.51 – 7.40 (m, 1H), 7.38 – 7.30 (m, 1H), 7.18 – 7.08 (m, 2H), 5.42 (d, *J* = 9.0 Hz, 1H), 4.09 (t, *J* = 6.2 Hz, 2H), 4.06 – 3.96 (m, 1H), 1.96 – 1.81 (m, 2H), 1.81 – 1.73 (m, 1H), 1.62 – 1.51 (m, 2H), 1.45 – 1.25 (m, 8H), 1.02 – 0.93 (m, 2H), 0.88 (t, *J* = 6.8 Hz, 3H), 0.77 – 0.65 (m, 2H).

^{13}C NMR (126 MHz, Chloroform-*d*) δ 173.16, 156.85, 134.55, 129.32, 128.85, 127.59, 126.72, 126.31, 123.51, 118.87, 106.56, 67.56, 49.07, 35.53, 31.95, 31.77, 25.77, 25.58, 22.58, 14.90, 14.06, 7.02, 7.00.

HRMS (ESI) calcd for $C_{23}H_{32}NO_2^+$ [(M+H)$^+$] 354.2428, found 354.2425.

HPLC analysis: The ee was determined to be 93% on a CHIRALPAK IG column (8% *i*PrOH in hexane, 0.8 mL/min, 40 °C); retention times for compound obtained using (*S*)-**L2**: 24.4 min (major), 25.6 min (minor).

$[\alpha]_D$ (20.0 °C, c = 1.0 in $CHCl_3$) = +7.89°; 93% ee, from (*S*)-**L2**.

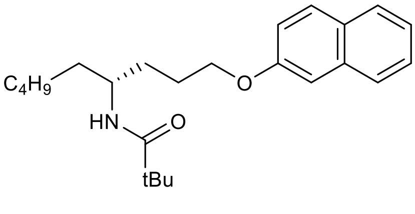

(*R*)-*N*-(1-(naphthalen-2-yloxy)nonan-4-yl)pivalamide (**40**)

Following **GP-E1**, **40** was obtained as white solid (67.2 mg, 91%, 86% ee).

^{1}H NMR (500 MHz, Chloroform-*d*) δ 7.80 – 7.67 (m, 3H), 7.48 – 7.39 (m, 1H), 7.39 – 7.29 (m, 1H), 7.20 – 7.10 (m, 2H), 5.36 (d, *J* = 9.0 Hz, 1H), 4.09 (td, *J* = 6.1, 1.6 Hz, 2H), 4.06 – 3.97 (m, 1H), 1.91 – 1.81 (m, 2H), 1.81 – 1.71 (m, 1H), 1.59 – 1.48 (m, 2H), 1.44 – 1.36 (m, 1H), 1.35 – 1.24 (m, 6H), 1.21 (s, 9H), 0.88 (t, *J* = 6.5 Hz, 3H).

^{13}C NMR (101 MHz, Chloroform-*d*) δ 178.10, 156.90, 134.61, 129.36, 128.92, 127.63, 126.76, 126.34, 123.55, 118.93, 106.66, 67.59, 48.63, 38.76, 35.44, 32.04, 31.73, 27.73, 25.79, 25.60, 22.59, 14.04.

HRMS (ESI) calcd for $C_{24}H_{36}NO_2^+$ $[(M+H)^+]$ 370.2741, found 370.2741.

HPLC analysis: The ee was determined to be 86% on a CHIRALCEL OJ-H column (10% *i*PrOH in hexane, 1.0 mL/min, 40 °C); retention times for compound obtained using (*S*)-**L2**: 12.8 min (major), 15.0 min (minor).

$[\alpha]_D$ (20.0 °C, c = 1.0 in $CHCl_3$) = +5.15°; 86% ee, from (*S*)-**L2**.

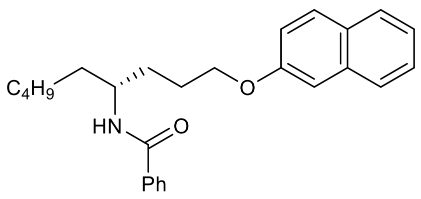

(*R*)-*N*-(1-(naphthalen-2-yloxy)nonan-4-yl)benzamide (**41**)

Following **GP-E2**, **41** was obtained as white solid (44.4 mg, 57%, 86% ee).

^{1}H NMR (500 MHz, Chloroform-*d*) δ 7.78 – 7.66 (m, 5H), 7.51 – 7.45 (m, 1H), 7.44 – 7.37 (m, 3H), 7.35 – 7.29 (m, 1H), 7.14 – 7.09 (m, 2H), 5.92 (d, *J* = 9.1 Hz, 1H), 4.30 – 4.18 (m, 1H), 4.12 (td, *J* = 6.1, 1.3 Hz, 2H), 2.02 – 1.92 (m, 2H), 1.91 – 1.84 (m, 1H), 1.74 – 1.59 (m, 3H), 1.57 – 1.48 (m, 1H), 1.45 – 1.36 (m, 2H), 1.33 – 1.28 (m, 3H), 0.88 (t, 3H).

^{13}C NMR (126 MHz, Chloroform-*d*) δ 167.29, 156.83, 134.92, 134.57, 131.34, 129.36, 128.93, 128.57, 127.60, 126.79, 126.74, 126.32, 123.55, 118.85, 106.71, 67.56, 49.54, 35.52, 31.98, 31.77, 25.83, 25.68, 22.58, 14.03.

HRMS (ESI) calcd for $C_{26}H_{32}NO_2^+$ $[(M+H)^+]$ 390.2428, found 390.2428.

HPLC analysis: The ee was determined to be 86% on a CHIRALCEL OD-H column (20% *i*PrOH in hexane, 2.0 mL/min, 40 °C); retention times for compound obtained using (*S*)-**L2**: 8.5 min (minor), 9.6 min (major).

$[\alpha]_D$ (20.0 °C, c = 1.0 in $CHCl_3$) = -3.57°; 86% ee, from (*S*)-**L2**.

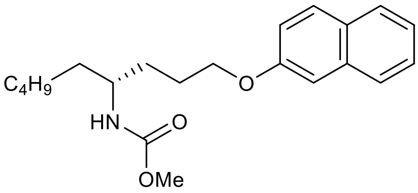

methyl (*R*)-(1-(naphthalen-2-yloxy)nonan-4-yl)carbamate (**42**)

Following **GP-E2**, **42** was obtained as colorless oil (42.6 mg, 62%, 91% ee).

^{1}H NMR (400 MHz, Chloroform-*d*) δ 7.80 – 7.68 (m, 3H), 7.47 – 7.40 (m, 1H), 7.36 – 7.29 (m, 1H), 7.18 – 7.09 (m, 2H), 4.48 (d, *J* = 9.2 Hz, 1H), 4.08 (t, *J* = 6.2 Hz, 2H), 3.79 – 3.58 (m, 4H), 1.98 – 1.82 (m, 2H), 1.80 – 1.70 (m, 1H), 1.60 – 1.47 (m, 2H), 1.43 – 1.24 (m, 7H), 0.92 – 0.84 (m, 3H).

^{13}C NMR (101 MHz, Chloroform-*d*) δ 156.90, 156.87, 134.57, 129.33, 128.90, 127.61, 126.71, 126.31, 123.52, 118.91, 106.55, 67.58, 51.98, 51.04, 35.60, 32.09, 31.73, 25.69, 25.49, 22.57, 14.04.

HRMS (ESI) calcd for $C_{21}H_{29}NO_3Na^+$ [$(M+Na)^+$] 366.2039, found 366.2047.

HPLC analysis: The ee was determined to be 91% on a CHIRALPAK AS-H column (3% *i*PrOH in hexane, 0.5 mL/min, 40 °C); retention times for compound obtained using (*S*)-**L2**: 13.7 min (minor), 15.4 min (major).

$[\alpha]_D$ (20.0 °C, c = 1.0 in $CHCl_3$) = +6.92°; 91% ee, from (*S*)-**L2**.

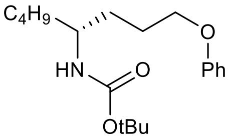

tert-butyl (*R*)-(1-phenoxyoctan-4-yl)carbamate (**43**)

Following **GP-E2**, **43** was obtained as white solid (43.0 mg, 67%, 92% ee).

^{1}H NMR (500 MHz, Chloroform-*d*) δ 7.30 – 7.26 (m, 2H), 6.96 – 6.86 (m, 3H), 4.32 (d, *J* = 9.3 Hz, 1H), 3.97 (t, *J* = 6.3 Hz, 2H), 3.67 – 3.46 (m, 1H), 1.92 – 1.76 (m, 2H), 1.72 – 1.64 (m, 1H), 1.54 – 1.41 (m, 11H), 1.38 – 1.25 (m, 5H), 0.89 (t, *J* = 6.2 Hz, 3H).

^{13}C NMR (101 MHz, Chloroform-*d*) δ 158.96, 155.76, 129.42, 120.55, 114.47, 78.92, 67.57, 50.40, 35.42, 32.10, 28.43, 28.04, 25.82, 22.64, 14.06.

HRMS (ESI) calcd for $C_{19}H_{31}NO_3Na^+$ [$(M+Na)^+$] 344.2196, found 344.2194.

HPLC analysis: The ee was determined to be 92% on a CHIRALPAK AD-H column (10% *i*PrOH in hexane, 1.0 mL/min, 40 °C); retention times for compound obtained using (*S*)-**L2**: 4.3 min (major), 4.8 min (minor).

$[\alpha]_D$ (20.0 °C, c = 1.0 in $CHCl_3$) = +2.09°; 92% ee, from (*S*)-**L2**.

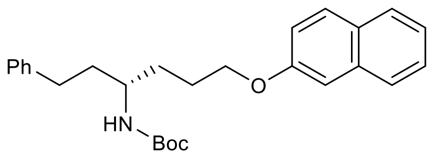

tert-butyl (*R*)-(6-(naphthalen-2-yloxy)-1-phenylhexan-3-yl)carbamate (**44**)

Following **GP-E2**, **44** was obtained as white solid (57.1 mg, 68%, 93% ee).

^{1}H NMR (500 MHz, Chloroform-*d*) δ 7.80 – 7.69 (m, 3H), 7.47 – 7.41 (m, 1H), 7.36 – 7.31 (m, 1H), 7.31 – 7.26 (m, 2H), 7.23 – 7.17 (m, 3H), 7.16 – 7.09 (m, 2H), 4.41 (d, *J* = 9.3 Hz, 1H), 4.09 (t, *J* = 6.3 Hz, 2H), 3.79 – 3.54 (m, 1H), 2.79 – 2.61 (m, 2H), 1.98 – 1.81 (m, 3H), 1.80 – 1.67 (m, 2H), 1.64 – 1.55 (m, 1H), 1.47 (s, 9H).

^{13}C NMR (126 MHz, Chloroform-*d*) δ 156.90, 155.75, 141.93, 134.57, 129.34, 128.91, 128.41, 128.37, 127.61, 126.71, 126.31, 125.86, 123.52, 118.91, 106.60, 79.11, 67.58, 50.32, 37.71, 32.41, 32.27, 28.43, 25.76.

HRMS (ESI) calcd for $C_{27}H_{33}NO_3Na^+$ [(M+Na)$^+$] 442.2352, found 442.2359.

HPLC analysis: The ee was determined to be 93% on a CHIRALPAK AD-H column (15% *i*PrOH in hexane, 1.5 mL/min, 40 °C); retention times for compound obtained using (*S*)-**L2**: 4.4 min (major), 5.7 min (minor).

$[\alpha]_D$ (20.0 °C, c = 1.0 in $CHCl_3$) = -2.81°; 93% ee, from (*S*)-**L2**.

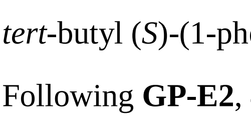

tert-butyl (*S*)-(1-phenylnonan-3-yl)carbamate (**45**)

Following **GP-E2**, **45** was obtained as colorless oil (42.2 mg, 66%, 93% ee).

^{1}H NMR (500 MHz, Chloroform-*d*) δ 7.32 – 7.26 (m, 2H), 7.19 (d, *J* = 7.4 Hz, 3H), 4.31 (d, *J* = 9.4 Hz, 1H), 3.69 – 3.42 (m, 1H), 2.76 – 2.54 (m, 2H), 1.84 – 1.73 (m, 1H), 1.69 – 1.61 (m, 1H), 1.53 – 1.43 (m, 10H), 1.38 – 1.22 (m, 9H), 0.88 (t, *J* = 6.8 Hz, 3H).

^{13}C NMR (126 MHz, Chloroform-*d*) δ 155.71, 142.21, 128.36, 125.77, 78.92, 50.60, 37.60, 35.69, 32.41, 31.80, 29.23, 28.44, 25.79, 22.59, 14.08.

HRMS (ESI) calcd for $C_{20}H_{33}NO_2Na^+$ [$(M+Na)^+$] 342.2403, found 342.2403.

HPLC analysis: The ee was determined to be 93% on a CHIRALCEL OD-H column (10% *i*PrOH in hexane, 1.0 mL/min, 40 °C); retention times for compound obtained using (*S*)-**L2**: 3.5 min (major), 3.9 min (minor).

$[\alpha]_D$ (20.0 °C, c = 1.0 in $CHCl_3$) = -7.72°; 93% ee, from (*S*)-**L2**.

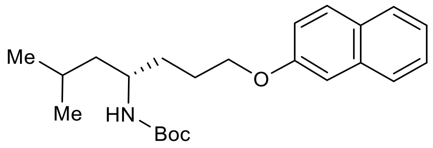

tert-butyl (*S*)-(6-methyl-1-(naphthalen-2-yloxy)heptan-4-yl)carbamate (**46**)

Following **GP-E2**, **46** was obtained as white solid (57.9 mg, 78%, 93% ee).

^{1}H NMR (500 MHz, Chloroform-*d*) δ 7.79 – 7.70 (m, 3H), 7.47 – 7.40 (m, 1H), 7.36 – 7.30 (m, 1H), 7.18 – 7.11 (m, 2H), 4.30 (d, *J* = 9.3 Hz, 1H), 4.09 (td, *J* = 6.3, 2.0 Hz, 2H), 3.82 – 3.60 (m, 1H), 1.98 – 1.82 (m, 2H), 1.77 – 1.65 (m, 2H), 1.54 – 1.41 (m, 10H), 1.36 – 1.25 (m, 2H), 0.93 (t, *J* = 6.6 Hz, 6H).

^{13}C NMR (126 MHz, Chloroform-*d*) δ 156.95, 155.69, 134.58, 129.30, 128.89, 127.61, 126.71, 126.29, 123.49, 118.94, 106.59, 78.90, 67.73, 48.53, 45.11, 32.75, 28.43, 25.69, 24.93, 23.13, 22.29.

HRMS (ESI) calcd for $C_{23}H_{33}NO_3Na^+$ [$(M+Na)^+$] 394.2352, found 394.2354.

HPLC analysis: The ee was determined to be 93% on a CHIRALPAK AD-H column (10% *i*PrOH in hexane, 1.0 mL/min, 40 °C); retention times for compound obtained using (*S*)-**L2**: 5.6 min (major), 7.5 min (minor).

$[\alpha]_D$ (20.0 °C, c = 1.0 in $CHCl_3$) = +9.82°; 93% ee, from (*S*)-**L2**.

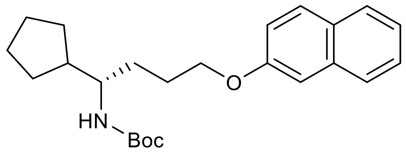

tert-butyl (*S*)-(1-cyclopentyl-4-(naphthalen-2-yloxy)butyl)carbamate (**47**)

Following **GP-E2**, **47** was obtained as white solid (49.8 mg, 65%, 97% ee).

^{1}H NMR (400 MHz, Chloroform-*d*) δ 7.80 – 7.69 (m, 3H), 7.46 – 7.39 (m, 1H), 7.37 – 7.29 (m, 1H), 7.18 – 7.09 (m, 2H), 4.37 (d, *J* = 9.8 Hz, 1H), 4.09 (t, *J* = 6.2 Hz, 2H), 3.63 – 3.38 (m, 1H), 2.02 – 1.92 (m, 1H), 1.91 – 1.78 (m, 3H), 1.78 – 1.69 (m, 2H), 1.66 – 1.59 (m, 2H), 1.57 – 1.49 (m, 2H), 1.49 – 1.40 (m, 10H), 1.31 – 1.22 (m, 2H).

^{13}C NMR (101 MHz, Chloroform-*d*) δ 156.98, 156.04, 134.58, 129.30, 128.88, 127.60, 126.71, 126.28, 123.48, 118.95, 106.59, 78.89, 67.72, 54.32, 45.39, 31.30, 29.78, 28.81, 28.44, 25.85, 25.58, 25.25.

HRMS (ESI) calcd for $C_{24}H_{33}NO_3Na^+$ [$(M+Na)^+$] 406.2352, found 406.2358.

HPLC analysis: The ee was determined to be 97% on a CHIRALPAK AD-H column (10% *i*PrOH in hexane, 1.0 mL/min, 40 °C); retention times for compound obtained using (*S*)-**L2**: 6.1 min (major), 9.0 min (minor).

$[\alpha]_D$ (20.0 °C, c = 1.0 in $CHCl_3$) = -4.83°; 97% ee, from (*S*)-**L2**.

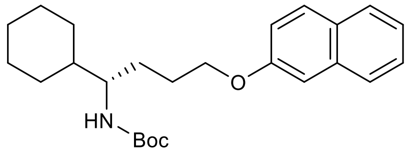

tert-butyl (*S*)-(1-cyclohexyl-4-(naphthalen-2-yloxy)butyl)carbamate (**48**)

Following **GP-E2**, **48** was obtained as white solid (33.4 mg, 42%, 97% ee).

^{1}H NMR (500 MHz, Chloroform-*d*) δ 7.78 – 7.68 (m, 3H), 7.46 – 7.40 (m, 1H), 7.36 – 7.30 (m, 1H), 7.17 – 7.10 (m, 2H), 4.38 (d, *J* = 9.9 Hz, 1H), 4.09 (t, *J* = 6.2 Hz, 2H), 3.56 – 3.35 (m, 1H), 1.99 – 1.81 (m, 2H), 1.80 – 1.70 (m, 4H), 1.70 – 1.60 (m, 2H), 1.52 – 1.41 (m, 10H), 1.42 – 1.33 (m, 1H), 1.29 – 1.18 (m, 2H), 1.18 – 0.94 (m, 3H).

^{13}C NMR (101 MHz, Chloroform-*d*) δ 156.96, 156.03, 134.58, 129.30, 128.88, 127.61, 126.71, 126.29, 123.49, 118.94, 106.59, 78.90, 67.71, 54.87, 42.50, 29.65, 29.16, 28.45, 28.22, 26.44, 26.29, 26.27, 26.10.

HRMS (ESI) calcd for $C_{25}H_{35}NO_3Na^+$ [$(M+Na)^+$] 420.2509, found 420.2516.

HPLC analysis: The ee was determined to be 98% on a CHIRALCEL OD-H column (10% *i*PrOH in hexane, 1.0 mL/min, 40 °C); retention times for compound obtained using (*S*)-**L2**: 6.3 min (major), 7.3 min (minor).

[α]D (20.0 °C, c = 1.0 in $CHCl_3$) = -4.99°; 98% ee, from (*S*)-**L2**.

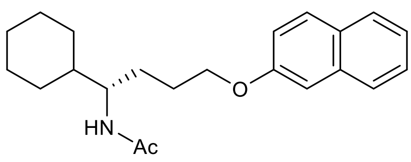

(*S*)-*N*-(1-cyclohexyl-4-(naphthalen-2-yloxy)butyl)acetamide (**49**)

Following **GP-E2**, **49** was obtained as white solid (33.9 mg, 50%, 86% ee).

^{1}H NMR (500 MHz, Chloroform-*d*) δ 7.79 – 7.68 (m, 3H), 7.46 – 7.40 (m, 1H), 7.35 – 7.30 (m, 1H), 7.16 – 7.10 (m, 2H), 5.41 (d, *J* = 9.5 Hz, 1H), 4.13 – 4.01 (m, 2H), 3.96 – 3.84 (m, 1H), 2.00 (s, 3H), 1.93 – 1.63 (m, 8H), 1.55 – 1.45 (m, 1H), 1.44 – 1.35 (m, 1H), 1.28 – 0.95 (m, 5H).

^{13}C NMR (101 MHz, Chloroform-*d*) δ 169.96, 156.86, 134.56, 129.34, 128.89, 127.60, 126.72, 126.34, 123.54, 118.85, 106.62, 67.52, 53.39, 42.14, 29.63, 28.70, 28.47, 26.40, 26.20, 26.01, 23.51.

HRMS (ESI) calcd for $C_{22}H_{30}NO_2^+$ [$(M+H)^+$] 340.2271, found 340.2271.

HPLC analysis: The ee was determined to be 86% on a CHIRALPAK AD-H column (15% *i*PrOH in hexane, 1.5 mL/min, 40 °C); retention times for compound obtained using (*S*)-**L2**: 3.5 min (minor), 4.3 min (major).

[α]D (20.0 °C, c = 1.0 in $CHCl_3$) = -2.83°; 86% ee, from (*S*)-**L2**.

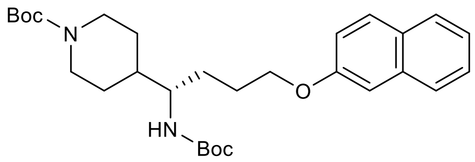

tert-butyl (*S*)-4-(1-((tert-butoxycarbonyl)amino)-4-(naphthalen-2-yloxy)butyl)piperidine-1-carboxylate (**50**)

Following **GP-E2**, **50** was obtained as colorless oil (51.8 mg, 52%, 92% ee).

^{1}H NMR (500 MHz, Chloroform-*d*) δ 7.77 – 7.70 (m, 3H), 7.45 – 7.40 (m, 1H), 7.35 – 7.29 (m, 1H), 7.16 – 7.10 (m, 2H), 4.38 (d, *J* = 9.8 Hz, 1H), 4.27 – 3.98 (m, 4H), 3.63 – 3.49 (m, 1H), 2.64 (s, 2H), 1.99 – 1.91 (m, 1H), 1.89 – 1.82 (m, 1H), 1.81 – 1.74 (m, 1H), 1.73 – 1.59 (m, 3H), 1.55 – 1.40 (m, 19H), 1.31 – 1.18 (m, 2H).

^{13}C NMR (126 MHz, Chloroform-*d*) δ 156.89, 155.98, 154.83, 134.59, 129.37, 128.96, 127.63, 126.72, 126.34, 123.57, 118.86, 106.70, 79.44, 79.27, 67.51, 54.17, 43.84, 41.14, 29.07, 28.47, 28.43, 26.06.

HRMS (ESI) calcd for $C_{29}H_{42}N_2O_5Na^+$ [(M+Na)$^+$] 521.2986, found 521.3001.

HPLC analysis: The ee was determined to be 92% on a CHIRALPAK AD-H column (7% *i*PrOH in hexane, 0.7 mL/min, 40 ^{o}C); retention times for compound obtained using (*S*)-**L2**: 20.5 min (major), 35.6 min (minor).

$[\alpha]_D$ (20.0 °C, c = 1.0 in $CHCl_3$) = -7.05°; 92% ee, from (*S*)-**L2**.

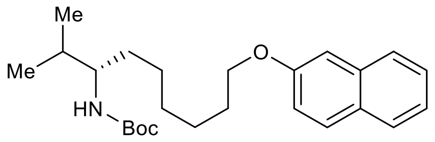

tert-butyl (*S*)-(2-methyl-9-(naphthalen-2-yloxy)nonan-3-yl)carbamate (**51**)

Following **GP-E2**, **51** was obtained as white solid (24.0 mg, 30%, 98% ee).

^{1}H NMR (500 MHz, Chloroform-*d*) δ 7.86 – 7.64 (m, 3H), 7.49 – 7.39 (m, 1H), 7.37 – 7.30 (m, 1H), 7.20 – 7.06 (m, 2H), 4.27 (d, *J* = 9.8 Hz, 1H), 4.07 (t, *J* = 6.5 Hz, 2H), 3.52 – 3.28 (m, 1H), 1.94 – 1.79 (m, 2H), 1.78 – 1.68 (m, 1H), 1.61 – 1.31 (m, 16H), 1.31 – 1.22 (m, 1H), 0.90 (d, *J* = 6.8 Hz, 3H), 0.86 (d, *J* = 6.7 Hz, 3H).

^{13}C NMR (126 MHz, Chloroform-*d*) δ 157.08, 156.05, 134.61, 129.29, 128.87, 127.62, 126.68, 126.27, 123.44, 119.02, 106.54, 78.79, 67.93, 55.48, 32.50, 32.10, 29.36, 29.20, 28.44, 26.19, 26.09, 19.17, 17.57.

HRMS (ESI) calcd for $C_{25}H_{37}NO_3Na^+$ [(M+Na)$^+$] 422.2665, found 422.2665.

HPLC analysis: The ee was determined to be 98% on a CHIRALPAK AD-H column (10% *i*PrOH in hexane, 1.0 mL/min, 40 ^{o}C); retention times for compound obtained using (*S*)-**L2**: 5.2 min (major), 5.6 min (minor).

$[\alpha]_D$ (20.0 °C, c = 1.0 in $CHCl_3$) = +1.89°; 98% ee, from (*S*)-**L2**.

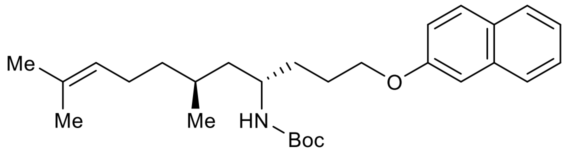

tert-butyl ((4*S*,6*S*)-6,10-dimethyl-1-(naphthalen-2-yloxy)undec-9-en-4-yl)carbamate (**52**)

Following **GP-E2**, **52** was obtained as colorless oil (57.1 mg, 63%, 98:2 d.r).

^{1}H NMR (500 MHz, Chloroform-*d*) δ 7.82 – 7.68 (m, 3H), 7.48 – 7.40 (m, 1H), 7.39 – 7.29 (m, 1H), 7.18 – 7.11 (m, 2H), 5.13 – 5.04 (m, 1H), 4.25 (d, *J* = 9.3 Hz, 1H), 4.09 (td, *J* = 6.3, 2.7 Hz, 2H), 3.83 – 3.59 (m, 1H), 2.08 – 1.82 (m, 4H), 1.65 (d, *J* = 38.2 Hz, 6H), 1.58 – 1.43 (m, 11H), 1.42 – 1.35 (m, 1H), 1.42 – 1.11 (m, 4H), 1.01 – 0.84 (m, 3H).

^{13}C NMR (101 MHz, Chloroform-*d*)δ 156.94, 155.75, 134.57, 131.19, 129.30, 128.88, 127.60, 126.71, 126.28, 124.72, 123.48, 118.93, 106.56, 78.90, 67.72, 48.30, 43.25, 37.54, 33.21, 29.17, 28.42, 25.77, 25.72, 25.48, 19.44, 17.68.

HRMS (ESI) calcd for $C_{28}H_{42}NO_3^+$ [(M+H)$^+$] 440.3159, found 440.3147.

HPLC analysis: The dr was determined to be 98:2 on a CHIRALPAK AD-H column (5% *i*PrOH in hexane, 0.5 mL/min, 40 °C); retention times for compound obtained using (*S*)-**L2**: 12.6 min (major), 15.3 min (minor).

$[\alpha]_D$ (20.0 °C, c = 1.0 in $CHCl_3$) = +3.59°; 98:2 dr, from (*S*)-**L2**.

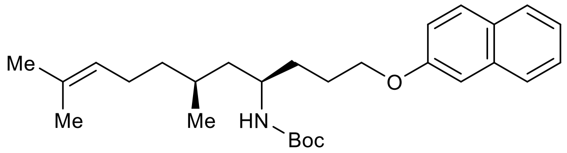

tert-butyl ((4*R*,6*S*)-6,10-dimethyl-1-(naphthalen-2-yloxy)undec-9-en-4-yl)carbamate (**53**)

Following **GP-E2**, **53** was obtained as colorless oil (51.7 mg, 57%, 3:97 d.r).

^{1}H NMR (500 MHz, Chloroform-*d*) δ 7.78 – 7.68 (m, 3H), 7.46 – 7.39 (m, 1H), 7.32 (t, *J* = 7.5 Hz, 1H), 7.18 – 7.10 (m, 2H), 5.20 – 5.02 (m, 1H), 4.31 (d, *J* = 9.2 Hz, 1H), 4.17 – 4.03 (m, 2H), 3.81 – 3.54 (m, 1H), 2.07 – 1.82 (m, 4H), 1.78 – 1.58 (m, 6H), 1.57 – 1.36 (m, 13H), 1.33 – 1.21 (m, 2H), 1.19 – 1.08 (m, 1H), 0.97 – 0.84 (m, 3H).

^{13}C NMR (101 MHz, Chloroform-*d*) δ 156.94, 155.57, 134.57, 131.16, 129.31, 128.88, 127.61, 126.71, 126.29, 124.72, 123.49, 118.93, 106.58, 78.93, 67.71, 48.34, 43.33, 36.60, 32.21, 29.41, 28.43, 25.72, 25.60, 25.37, 19.83, 17.67.

HRMS (ESI) calcd for $C_{28}H_{42}NO_3^+$ [$(M+H)^+$] 440.3159, found 440.3152.

HPLC analysis: The dr was determined to be 3:97 on a CHIRALPAK AD-H column (5% *i*PrOH in hexane, 0.5 mL/min, 40 °C); retention times for compound obtained using (*R*)-**L2**: 12.7 min (major), 15.4 min (minor).

$[\alpha]_D$ (20.0 °C, c = 1.0 in $CHCl_3$) = -5.67°; 3:97 dr, from (*R*)-**L2**.

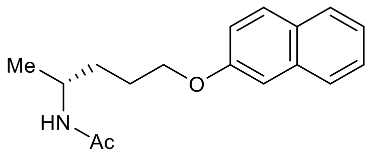

(*R*)-*N*-(5-(naphthalen-2-yloxy)pentan-2-yl)acetamide (**54**)

Following **GP-E2**, **54** was obtained as white solid (44.5 mg, 82%, 85% ee).

^{1}H NMR (500 MHz, Chloroform-*d*) δ 7.79 – 7.63 (m, 3H), 7.49 – 7.38 (m, 1H), 7.37 – 7.30 (m, 1H), 7.17 – 7.05 (m, 2H), 5.57 – 5.36 (m, 1H), 4.08 (t, *J* = 6.3 Hz, 3H), 1.97 (s, 3H), 1.87 (h, *J* = 7.2 Hz, 2H), 1.67 (dtd, *J* = 16.7, 13.8, 6.7 Hz, 2H), 1.19 (d, *J* = 6.6 Hz, 3H).

^{13}C NMR (101 MHz, Chloroform-*d*) δ 169.58, 156.82, 134.55, 129.37, 128.91, 127.61, 126.72, 126.35, 123.57, 118.83, 106.60, 67.47, 45.10, 33.50, 25.90, 23.54, 21.14.

HRMS (ESI) calcd for $C_{17}H_{21}NO_2Na^+$ [$(M+Na)^+$] 294.1464, found 294.1468.

HPLC analysis: The ee was determined to be 85% on a CHIRALCEL OD-H column (20% *i*PrOH in hexane, 2.0 mL/min, 40 °C); retention times for compound obtained using (*S*)-**L2**: 4.1 min (major), 4.7 min (minor).

$[\alpha]_D$ (20.0 °C, c = 1.0 in $CHCl_3$) = +12.35°; 85% ee, from (*S*)-**L2**.

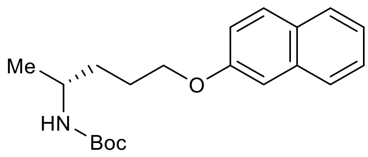

tert-butyl (*R*)-(5-(naphthalen-2-yloxy)pentan-2-yl)carbamate (**55**)

Following **GP-E2**, **55** was obtained as white solid (45.5 mg, 69%, 88% ee).

^{1}H NMR (500 MHz, Chloroform-*d*) δ 7.78 – 7.70 (m, 3H), 7.47 – 7.41 (m, 1H), 7.35 – 7.31 (m, 1H), 7.17 – 7.11 (m, 2H), 4.43 (s, 1H), 4.14 – 4.04 (m, 2H), 3.75 (s, 1H), 1.95

– 1.85 (m, 2H), 1.70 – 1.58 (m, 2H), 1.46 (s, 9H), 1.18 (d, *J* = 6.6 Hz, 3H).

^{13}C NMR (126 MHz, Chloroform-*d*) δ 156.92, 155.45, 134.58, 129.33, 128.92, 127.62, 126.71, 126.31, 123.52, 118.92, 106.60, 79.05, 67.65, 46.32, 33.90, 28.44, 25.92, 21.43.

HRMS (ESI) calcd for $C_{20}H_{27}NO_3Na^+$ [(M+Na)$^+$] 352.1883, found 352.1888.

HPLC analysis: The ee was determined to be 88% on a CHIRALPAK AD-H column (15% *i*PrOH in hexane, 1.5 mL/min, 40 ºC); retention times for compound obtained using (*S*)-**L2**: 3.4 min (major), 3.8min (minor).

[α]$_D$ (20.0 °C, c = 1.0 in $CHCl_3$) = +3.72°; 88% ee, from (*S*)-**L2**.

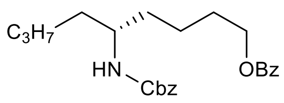

(*R*)-5-(((benzyloxy)carbonyl)amino)nonyl benzoate (**56**)

Following **GP-E2**, **56** was obtained as colorless oil (47.7 mg, 60%, 91% ee).

^{1}H NMR (500 MHz, Chloroform-*d*) δ 8.08 – 7.98 (m, 2H), 7.58 – 7.51 (m, 1H), 7.48 – 7.39 (m, 2H), 7.39 – 7.28 (m, 5H), 5.16 – 5.03 (m, 2H), 4.51 (d, *J* = 9.3 Hz, 1H), 4.31 (t, *J* = 6.6 Hz, 2H), 3.71 – 3.54 (m, 1H), 1.87 – 1.70 (m, 2H), 1.61 – 1.40 (m, 5H), 1.39 – 1.20 (m, 5H), 0.88 (t, *J* = 5.5 Hz, 3H).

^{13}C NMR (101 MHz, Chloroform-*d*) δ 166.67, 156.16, 136.66, 132.85, 130.38, 129.54, 128.51, 128.33, 128.07, 128.05, 66.55, 64.77, 51.15, 35.19, 35.10, 28.59, 27.96, 22.61, 22.35, 14.03.

HRMS (ESI) calcd for $C_{24}H_{31}NO_4Na^+$ [(M+Na)$^+$] 420.2145, found 420.2144.

HPLC analysis: The ee was determined to be 91% on a CHIRALCEL OD-H column (10% *i*PrOH in hexane, 1.0 mL/min, 40 ºC); retention times for compound obtained using (*S*)-**L2**: 9.9 min (major), 13.1min (minor).

[α]$_D$ (20.0 °C, c = 1.0 in $CHCl_3$) = -1.40°; 91% ee, from (*S*)-**L2**.

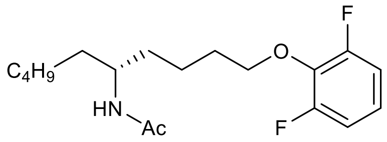

(*R*)-*N*-(1-(2,6-difluorophenoxy)decan-5-yl)acetamide (**57**)

Following **GP-E2**, **57** was obtained as white solid (51.1 mg, 78%, 90% ee).

^{1}H NMR (500 MHz, Chloroform-*d*) δ 6.98 – 6.80 (m, 3H), 5.18 (d, *J* = 9.1 Hz, 1H), 4.18 – 4.05 (m, 2H), 3.97 – 3.87 (m, 1H), 1.98 (s, 3H), 1.82 – 1.69 (m, 2H), 1.59 – 1.44 (m, 4H), 1.37 – 1.18 (m, 8H), 0.87 (t, *J* = 6.6 Hz, 3H).

^{13}C NMR (126 MHz, Chloroform-*d*) δ 169.96, 156.40 (dd, *J* = 247.9, 5.6 Hz), 135.84 (t, *J* = 14.2 Hz), 122.74 (t, *J* = 9.4 Hz), 112.23 (dd, *J* = 17.2, 5.6 Hz), 74.51 (t, *J* = 3.0 Hz), 49.50, 35.20, 34.77, 31.87, 29.88, 25.67, 23.46, 22.67, 22.07, 14.12.

^{19}F NMR (471 MHz, Chloroform-*d*) δ 128.37.

HRMS (ESI) calcd for $C_{18}H_{27}F_2NO_2Na^+$ [(M+Na)$^+$] 350.1902, found 350.1902.

HPLC analysis: The ee was determined to be 90% on a CHIRALPAK AD-H column (5% *i*PrOH in hexane, 0.5 mL/min, 40 °C); retention times for compound obtained using (*S*)-**L2**: 15.0 min (minor), 15.9min (major).

[α]$_D$ (20.0 °C, c = 1.0 in $CHCl_3$) = +0.96°; 90% ee, from (*S*)-**L2**.

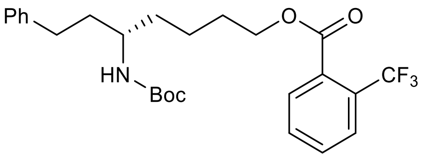

(*S*)-5-((*tert*-butoxycarbonyl)amino)-7-phenylheptyl 2-(trifluoromethyl)benzoate (**58**)

Following **GP-E2**, **58** was obtained as colorless oil (63.3 mg, 66%, 93% ee).

^{1}H NMR (500 MHz, Chloroform-*d*) δ 7.81 – 7.70 (m, 2H), 7.64 – 7.56 (m, 2H), 7.28 (d, *J* = 7.7 Hz, 2H), 7.22 – 7.12 (m, 3H), 4.54 – 4.20 (m, 3H), 3.72 – 3.42 (m, 1H), 2.77 – 2.55 (m, 2H), 1.84 – 1.72 (m, 3H), 1.69 – 1.60 (m, 1H), 1.59 – 1.49 (m, 2H), 1.47 – 1.37 (m, 11H).

^{13}C NMR (101 MHz, Chloroform-*d*) δ 167.00, 155.72, 141.98, 131.74, 131.43 (d, *J* = 2.4 Hz), 131.06, 130.16, 128.45 (q, *J* = 32.3 Hz), 128.39, 128.35, 126.64 (q, *J* = 5.4 Hz), 125.83, 123.39 (q, *J* = 273.4 Hz), 79.04, 65.94, 50.36, 37.57, 35.29, 32.39, 28.39, 28.29, 22.21.

^{19}F NMR (471 MHz, Chloroform-*d*) δ 59.33.

HRMS (ESI) calcd for $C_{26}H_{32}F_3NO_4Na^+$ [(M+Na)$^+$] 502.2175, found 502.2184.

HPLC analysis: The ee was determined to be 93% on a CHIRALCEL OD-H column

(10% *i*PrOH in hexane, 1.0 mL/min, 40 °C); retention times for compound obtained using (*S*)-**L2**: 7.3 min (major), 8.3min (minor).

[α]D (20.0 °C, c = 1.0 in $CHCl_3$) = -3.44°; 93% ee, from (*S*)-**L2**.

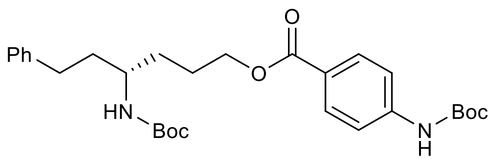

(*R*)-4-((*tert*-butoxycarbonyl)amino)-6-phenylhexyl-4-((*tert*-butoxycarbonyl)amino-benzoate (**59**)

Following **GP-E2**, **59** was obtained as white solid (61.5 mg, 60%, 92% ee).

^{1}H NMR (500 MHz, Chloroform-*d*) δ 7.96 (d, *J* = 8.8 Hz, 2H), 7.42 (d, *J* = 8.5 Hz, 2H), 7.28 (d, *J* = 7.7 Hz, 2H), 7.22 – 7.14 (m, 3H), 6.73 (s, 1H), 4.35 (d, *J* = 9.4 Hz, 1H), 4.29 (t, *J* = 6.5 Hz, 2H), 3.74 – 3.49 (m, 1H), 2.76 – 2.58 (m, 2H), 1.90 – 1.74 (m, 3H), 1.72 – 1.60 (m, 3H), 1.52 (s, 9H), 1.45 (s, 9H).

^{13}C NMR (101 MHz, Chloroform-*d*) δ 166.21, 155.70, 152.18, 142.68, 141.85, 130.85, 128.41, 128.35, 125.88, 124.51, 117.33, 81.21, 79.18, 64.60, 50.35, 37.63, 32.40, 32.24, 28.42, 28.27, 25.37.

HRMS (ESI) calcd for $C_{29}H_{40}N_2O_6Na^+$ [$(M+Na)^+$] 535.2778, found 535.2792.

HPLC analysis: The ee was determined to be 92% on a CHIRALPAK AD-H column (15% *i*PrOH in hexane, 1.5 mL/min, 40 °C); retention times for compound obtained using (*S*)-**L2**: 5.8 min (major), 6.2min (minor).

[α]D (20.0 °C, c = 1.0 in $CHCl_3$) = -6.59°; 92% ee, from (*S*)-**L2**.

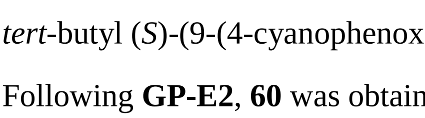

tert-butyl (*S*)-(9-(4-cyanophenoxy)-1-phenylnonan-3-yl)carbamate (**60**)

Following **GP-E2**, **60** was obtained as white solid (36.6 mg, 42%, 93% ee).

^{1}H NMR (500 MHz, Chloroform-*d*) δ 7.53 (d, *J* = 8.8 Hz, 2H), 7.25 – 7.22 (m, 2H), 7.18 – 7.12 (m, 3H), 6.89 (d, *J* = 8.8 Hz, 2H), 4.29 (d, *J* = 8.5 Hz, 1H), 3.95 (t, *J* = 6.5

Hz, 2H), 3.68 – 3.40 (m, 1H), 2.72 – 2.56 (m, 2H), 1.80 – 1.72 (m, 3H), 1.66 – 1.57 (m, 1H), 1.49 – 1.40 (m, 12H), 1.39 – 1.28 (m, 5H).

^{13}C NMR (126 MHz, Chloroform-*d*) δ 162.72, 156.04, 142.37, 134.24, 128.68, 128.64, 126.12, 119.62, 115.47, 103.93, 79.29, 68.59, 50.75, 37.90, 35.95, 32.71, 29.43, 29.17, 28.73, 26.17, 26.02.

HRMS (ESI) calcd for $C_{27}H_{36}N_2O_3Na^+$ [$(M+Na)^+$] 459.2618, found 459.2617.

HPLC analysis: The ee was determined to be 93% on a CHIRALPAK AD-H column (10% *i*PrOH in hexane, 1.0 mL/min, 40 ^{o}C); retention times for compound obtained using (*S*)-**L2**: 15.3 min (minor), 15.9min (major).

$[\alpha]_D$ (20.0 °C, c = 1.0 in $CHCl_3$) = -4.02°; 93% ee, from (*S*)-**L2**.

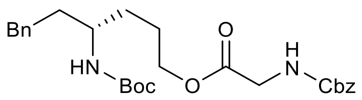

(*R*)-4-((*tert*-butoxycarbonyl)amino)-6-phenylhexyl ((benzyloxy)carbonyl)glycinate (**61**)

Following **GP-E2**, **61** was obtained as white solid (60.1 mg, 62%, 92% ee).

^{1}H NMR (400 MHz, Chloroform-*d*) δ 7.41 – 7.32 (m, 4H), 7.31 – 7.26 (m, 3H), 7.23 – 7.12 (m, 3H), 5.35 (s, 1H), 5.13 (s, 2H), 4.44 – 4.24 (m, 1H), 4.15 (s, 2H), 3.97 (d, *J* = 5.5 Hz, 2H), 3.72 – 3.42 (m, 1H), 2.77 – 2.51 (m, 2H), 1.85 – 1.54 (m, 5H), 1.45 (s, 9H), 1.31 – 1.22 (m, 1H).

^{13}C NMR (101 MHz, Chloroform-*d*) δ 170.01, 156.30, 155.72, 141.78, 136.22, 128.53, 128.42, 128.34, 128.19, 128.09, 125.90, 79.22, 67.06, 65.35, 50.14, 42.79, 37.67, 32.36, 32.20, 28.41, 25.04.

HRMS (ESI) calcd for $C_{27}H_{36}N_2O_6Na^+$ [$(M+Na)^+$] 507.2465, found 507.2468.

HPLC analysis: The ee was determined to be 92% on a CHIRALCEL OD-H column (20% *i*PrOH in hexane, 2.0 mL/min, 40 ^{o}C); retention times for compound obtained using (*S*)-**L2**: 7.0 min (major), 8.2min (minor).

$[\alpha]_D$ (20.0 °C, c = 1.0 in $CHCl_3$) = -4.39°; 92% ee, from (*S*)-**L2**.

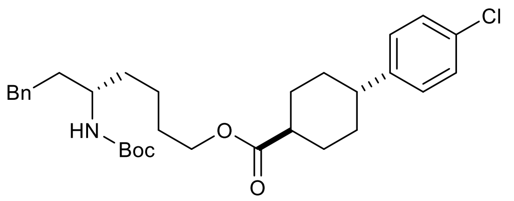

(*S*)-5-((*tert*-butoxycarbonyl)amino)-7-phenylheptyl (1*r*,4*S*)-4-(4 chlorophenyl) cyclohexane-1-carboxylate (**62**)

Following **GP-E2**, **62** was obtained as yellow oil (79.2 mg, 75%, 93% ee).

^{1}H NMR (500 MHz, Chloroform-*d*) δ 7.31 – 7.27 (m, 2H), 7.26 – 7.23 (m, 2H), 7.21 – 7.16 (m, 3H), 7.12 (d, *J* = 8.2 Hz, 2H), 4.32 (d, *J* = 9.3 Hz, 1H), 4.12 – 4.02 (m, 2H), 3.69 – 3.45 (m, 1H), 2.75 – 2.58 (m, 2H), 2.55 – 2.44 (m, 1H), 2.37 – 2.28 (m, 1H), 2.14 – 2.04 (m, 2H), 1.99 – 1.89 (m, 2H), 1.85 – 1.74 (m, 1H), 1.69 – 1.61 (m, 3H), 1.60 – 1.52 (m, 3H), 1.48 – 1.38 (m, 14H).

^{13}C NMR (126 MHz, Chloroform-*d*) δ 175.88, 155.72, 145.28, 141.97, 131.63, 128.45, 128.39, 128.34, 128.09, 125.85, 79.03, 64.09, 50.37, 42.97, 42.94, 37.59, 35.33, 33.21, 32.38, 29.21, 28.53, 28.43, 22.31.

HRMS (ESI) calcd for $C_{31}H_{42}ClNO_4Na^+$ [(M+Na)$^+$] 550.2694, found 550.2694.

HPLC analysis: The ee was determined to be 93% on a CHIRALPAK AD-H column (5% *i*PrOH in hexane, 0.5 mL/min, 40 °C); retention times for compound obtained using (*S*)-**L2**: 34.8 min (minor), 36.4min (major).

$[\alpha]_D$ (20.0 °C, c = 1.0 in $CHCl_3$) = -3.64°; 93% ee, from (*S*)-**L2**.

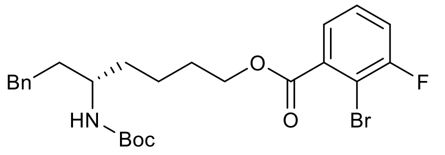

(*S*)-5-((*tert*-butoxycarbonyl)amino)-7-phenylheptyl 2-bromo-3-fluorobenzoate (**63**)

Following **GP-E2**, **63** was obtained as yellow oil (63.0 mg, 62%, 91% ee).

^{1}H NMR (500 MHz, Chloroform-*d*) δ 7.59 – 7.47 (m, 1H), 7.36 – 7.30 (m, 1H), 7.30 – 7.26 (m, 2H), 7.25 – 7.22 (m, 1H), 7.21 – 7.13 (m, 3H), 4.45 – 4.22 (m, 3H), 3.71 – 3.45 (m, 1H), 2.76 – 2.56 (m, 2H), 1.86 – 1.72 (m, 3H), 1.70 – 1.58 (m, 2H), 1.57 – 1.50 (m, 2H), 1.47 – 1.39 (m, 10H).

^{13}C NMR (101 MHz, Chloroform-*d*) δ 165.59 (d, *J* = 2.9 Hz), 159.50 (d, *J* = 247.3 Hz), 155.72, 141.93, 134.69, 128.44 (d, *J* = 7.3 Hz), 128.41, 128.35, 126.46 (d, *J* = 3.3 Hz), 125.86, 118.99 (d, *J* = 23.5 Hz), 109.30 (d, *J* = 22.5 Hz), 79.11, 65.80, 50.34, 37.62, 35.35, 32.40, 28.41, 28.41, 22.42.

^{19}F NMR (376 MHz, Chloroform-*d*) δ 103.00 (dd, *J* = 8.1, 5.1 Hz).

HRMS (ESI) calcd for $C_{25}H_{31}BrFNO_4Na^+$ [(M+Na)$^+$] 530.1312, found 530.1310.

HPLC analysis: The ee was determined to be 91% on a CHIRALPAK AD-H column (10% *i*PrOH in hexane, 1.0 mL/min, 40 °C); retention times for compound obtained using (*S*)-**L2**: 8.6 min (major), 10.4min (minor).

$[\alpha]_D$ (20.0 °C, c = 1.0 in $CHCl_3$) = -2.99°; 91% ee, from (*S*)-**L2**.

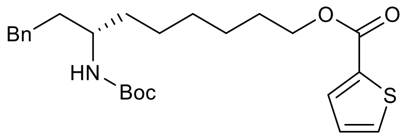

(*S*)-7-((*tert*-butoxycarbonyl)amino)-9-phenylnonyl thiophene-2-carboxylate (**64**)

Following **GP-E2**, **64** was obtained as yellow oil (52.6 mg, 59%, 91% ee).

^{1}H NMR (500 MHz, Chloroform-*d*) δ 7.79 (dd, *J* = 3.8, 1.3 Hz, 1H), 7.53 (dd, *J* = 5.0, 1.3 Hz, 1H), 7.28 (d, *J* = 7.7 Hz, 2H), 7.18 (d, *J* = 7.2 Hz, 3H), 7.13 – 7.06 (m, 1H), 4.33 (d, *J* = 9.4 Hz, 1H), 4.28 (t, *J* = 6.6 Hz, 2H), 3.68 – 3.44 (m, 1H), 2.73 – 2.58 (m, 2H), 1.81 – 1.71 (m, 3H), 1.67 – 1.59 (m, 1H), 1.51 – 1.43 (m, 10H), 1.43 – 1.31 (m, 7H).

^{13}C NMR (101 MHz, Chloroform-*d*) δ 162.33, 155.72, 142.11, 134.04, 133.25, 132.19, 128.36, 128.35, 127.70, 125.79, 78.96, 65.18, 50.50, 37.60, 35.61, 32.41, 29.12, 28.59, 28.43, 25.89, 25.71.

HRMS (ESI) calcd for $C_{25}H_{35}NO_4SNa^+$ [(M+Na)$^+$] 468.2179, found 468.2192.

HPLC analysis: The ee was determined to be 91% on a CHIRALPAK AD-H column (10% *i*PrOH in hexane, 1.0 mL/min, 40 °C); retention times for compound obtained using (*S*)-**L2**: 8.4 min (minor), 9.0 min (major).

$[\alpha]_D$ (20.0 °C, c = 1.0 in $CHCl_3$) = -4.13°; 91% ee, from (*S*)-**L2**.

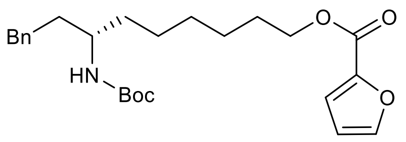

(S)-7-(($tert$-butoxycarbonyl)amino)-9-phenylnonyl furan-2-carboxylate (**65**)

Following **GP-E2**, **65** was obtained as yellow oil (53.3 mg, 62%, 93% ee).

^{1}H NMR (400 MHz, Chloroform-d) δ 7.56 (dd, J = 1.8, 0.8 Hz, 1H), 7.27 – 7.25 (m, 2H), 7.21 – 7.14 (m, 4H), 6.50 (dd, J = 3.5, 1.7 Hz, 1H), 4.28 (t, J = 6.7 Hz, 3H), 3.67 – 3.40 (m, 1H), 2.75 – 2.55 (m, 2H), 1.80 – 1.70 (m, 3H), 1.67 – 1.58 (m, 1H), 1.52 – 1.43 (m, 10H), 1.41 – 1.28 (m, 7H).

^{13}C NMR (126 MHz, Chloroform-d) δ 159.03, 155.90, 146.37, 145.05, 142.29, 128.56, 128.54, 125.99, 117.90, 111.96, 79.16, 65.17, 50.70, 37.77, 35.80, 32.59, 29.30, 28.79, 28.62, 26.03, 25.89.

HRMS (ESI) calcd for $C_{25}H_{35}NO_5Na^+$ [$(M+Na)^+$] 452.2407, found 452.2413.

HPLC analysis: The ee was determined to be 93% on a CHIRALPAK AD-H column (10% iPrOH in hexane, 1.0 mL/min, 40 °C); retention times for compound obtained using (S)-**L2**: 8.8 min (minor), 9.6 min (major).

$[\alpha]_D$ (20.0 °C, c = 1.0 in $CHCl_3$) = -4.52°; 93% ee, from (S)-**L2**.

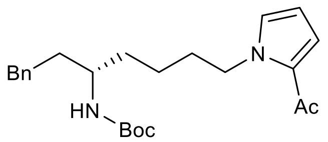

$tert$-butyl (S)-(7-(2-acetyl-1H-pyrrol-1-yl)-1-phenylheptan-3-yl)carbamate (**66**)

Following **GP-E2**, **66** was obtained as yellow oil (39.8 mg, 50%, 95% ee).

^{1}H NMR (500 MHz, Chloroform-d) δ 7.30 – 7.24 (m, 2H), 7.20 – 7.14 (m, 3H), 6.95 (dd, J = 4.1, 1.7 Hz, 1H), 6.83 (t, J = 2.1 Hz, 1H), 6.11 (dd, J = 4.1, 2.5 Hz, 1H), 4.40 – 4.20 (m, 3H), 3.66 – 3.42 (m, 1H), 2.72 – 2.57 (m, 2H), 2.42 (s, 3H), 1.79 – 1.68 (m, 3H), 1.67 – 1.59 (m, 1H), 1.52 – 1.43 (m, 10H), 1.42 – 1.30 (m, 3H).

^{13}C NMR (126 MHz, Chloroform-d) δ 188.26, 155.71, 142.03, 130.20, 130.04, 128.38, 128.36, 125.81, 120.33, 107.96, 79.00, 50.50, 49.56, 37.58, 35.28, 32.39, 31.32, 28.44, 27.33, 22.94.

HRMS (ESI) calcd for $C_{24}H_{34}N_2O_3Na^+$ [$(M+Na)^+$] 421.2461, found 421.2467.

HPLC analysis: The ee was determined to be 95% on a CHIRALPAK AD-H column (15% *i*PrOH in hexane, 1.5 mL/min, 40 °C); retention times for compound obtained using (*S*)-**L2**: 4.0 min (major), 4.3 min (minor).

$[\alpha]_D$ (20.0 °C, c = 1.0 in $CHCl_3$) = -1.23°; 95% ee, from (*S*)-**L2**.

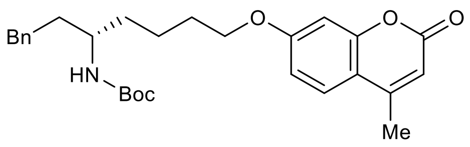

tert-butyl (*S*)-(7-((4-methyl-2-oxo-2H-chromen-7-yl)oxy)-1-phenylheptan-3-yl) carbamate (**67**)

Following **GP-E2**, **67** was obtained as yellow solid (63.3 mg, 68%, 92% ee).

^{1}H NMR (500 MHz, Chloroform-*d*) δ 7.48 (d, *J* = 8.8 Hz, 1H), 7.28 (d, *J* = 7.6 Hz, 2H), 7.22 – 7.14 (m, 3H), 6.83 (dd, *J* = 8.8, 2.5 Hz, 1H), 6.78 (d, *J* = 2.5 Hz, 1H), 6.14 – 6.11 (m, 1H), 4.32 (d, *J* = 9.4 Hz, 1H), 3.99 (dt, *J* = 6.4, 3.9 Hz, 2H), 3.74 – 3.49 (m, 1H), 2.76 – 2.58 (m, 2H), 2.39 (d, *J* = 1.2 Hz, 3H), 1.89 – 1.74 (m, 3H), 1.70 – 1.61 (m, 2H), 1.60 – 1.53 (m, 2H), 1.52 – 1.41 (m, 10H).

^{13}C NMR (126 MHz, Chloroform-*d*) δ 162.28, 161.53, 155.92, 155.41, 152.77, 142.12, 128.55, 128.51, 126.01, 125.64, 113.59, 112.76, 111.97, 101.49, 79.23, 68.50, 50.52, 37.74, 35.64, 32.58, 28.99, 28.59, 22.57, 18.82.

HRMS (ESI) calcd for $C_{28}H_{35}NO_5Na^+$ [$(M+Na)^+$] 488.2407, found 488.2417.

HPLC analysis: The ee was determined to be 92% on a CHIRALPAK AD-H column (15% *i*PrOH in hexane, 1.5 mL/min, 40 °C); retention times for compound obtained using (*S*)-**L2**: 10.0 min (major), 13.8 min (minor).

$[\alpha]_D$ (20.0 °C, c = 1.0 in $CHCl_3$) = -3.51°; 92% ee, from (*S*)-**L2**.

Reductive Hydroalkylation of Enamides with Racemic α-Haloboronates

General Procedure for Asymmetric Reductive Hydroalkylation of Enamides with Racemic α-Haloboronates

General Procedure F1 (GP-F1): In the air, a 10 mL screw-cap test tube equipped with a magnetic stirrer was charged with $NiBr_2$(diglyme) (0.05 mmol, 10 mol%), ligand (0.06 mmol, 12 mol%). The test tube was evacuated and backfilled with argon for three times, then THF (2 mL) was added and the mixture was stirred at room temperature for 30 min.

Meanwhile, in the air, another 10 mL screw-cap test tube equipped with a magnetic stirrer was charged with potassium fluoride (1.5 mmol, 3.0 equiv.) (if enamide or racemic α-haloboronates is a solid, it was also added at this time). The test tube was evacuated and backfilled with argon for three times. Next, the solution of the catalyst (see above) was added in one portion via syringe, followed by the enamide (0.5 mmol, 1.0 equiv.) and racemic α-haloboronates (1.0 mmol, 2.0 equiv.). The resulting solution was stirred for 2 min at 0 °C, DEMS (1.5 mmol, 3.0 equiv.) was added dropwise via syringe and the solution was kept stirring for 5 min at 0 °C, then was stirred at 25 °C for 30 h. The reaction mixture was diluted with H_2O followed by extraction with EtOAc, dried with anhydrous Na_2SO_4 and concentrated in vacuo. The residue was purified by flash column chromatography on silica gel to give the target product.

General Procedure F2 (GP-F2): The procedure is the same as **GP-F1**, except for reaction temperature: the reaction mixture was stirred at 40 °C for 30 h.

General Procedure F3 (GP-F3): General Procedure for Oxidation of β-Aminoboronates. The β-aminoboronates (0.2 mmol) was oxidized with $NaBO_3(4H_2O)$ (1.0 mmol, 5.0 equiv.) in THF/H_2O (1:1; 2 mL) at room temperature for 5 h. The reaction mixture was diluted with H_2O followed by extraction with EtOAc, dried with

anhydrous Na_2SO_4 and concentrated in vacuo. The residue was purified by flash column chromatography on silica gel to give the target product.

Examples Described in Figure 5

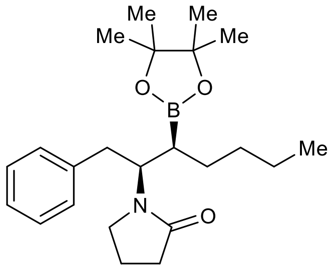

1-((2*S*,3*S*)-1-phenyl-3-(4,4,5,5-tetramethyl-1,3,2-dioxaborolan-2-yl)heptan-2-yl)pyrrolidin-2-one (**83**)

Following **GP-F1**, **83** was obtained as colorless oil (96.3 mg, 50% yield, 97% ee).

^{1}H NMR (500 MHz, Chloroform-*d*) δ 7.27 – 7.20 (m, 2H), 7.21 – 7.11 (m, 3H), 4.37 (td, *J* = 10.0, 5.0 Hz, 1H), 3.26 (td, *J* = 8.8, 5.6 Hz, 1H), 3.17 (td, *J* = 8.7, 5.9 Hz, 1H), 3.11 (dd, *J* = 14.3, 4.9 Hz, 1H), 2.71 (dd, *J* = 14.2, 10.5 Hz, 1H), 2.24 – 2.13 (m, 1H), 2.11 – 1.99 (m, 1H), 1.88 – 1.74 (m, 1H), 1.76 – 1.64 (m, 1H), 1.58 – 1.43 (m, 2H), 1.43 – 1.37 (m, 1H), 1.36 – 1.24 (m, 4H), 1.22 (s, 12H), 0.88 (t, *J* = 6.8 Hz, 3H).

^{13}C NMR (101 MHz, Chloroform-*d*) δ 174.80, 138.69, 129.02, 128.34, 126.35, 83.31, 54.58, 44.34, 37.68, 31.42, 28.63, 25.05, 24.99, 23.03, 18.27, 14.16.

^{11}B NMR (128 MHz, Chloroform-*d*) δ 33.62.

HRMS (ESI) calcd for $C_{23}H_{37}NO_3B^+$ $[(M+H)^+]$ 386.2866, found 386.2871.

$[\alpha]_D$ (20.0 ℃, c = 1.0 in $CHCl_3$) = -32.74 °, 97% ee, from (*S*,*S*)-**L1**.

HPLC analysis indicated 97% ee (compound **83-OH**) after oxidation.

^{1}H NMR analysis of crude β-aminoboronates (purified by flash column chromatogram-phy) indicated 2.5:1 dr.(compound **83**: compound **84**).

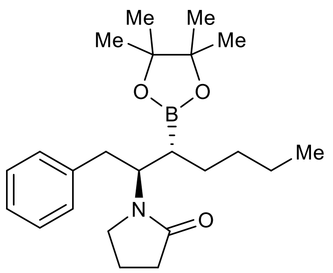

1-((2*S*,3*R*)-1-phenyl-3-(4,4,5,5-tetramethyl-1,3,2-dioxaborolan-2-yl)heptan-2-yl)pyrrolidin-2-one (**84**)

Following **GP-F1**, **84** was obtained as colorless oil (38.5 mg, 20% yield, 97% ee).

^{1}H NMR (500 MHz, Chloroform-*d*) δ 7.26 – 7.19 (m, 2H), 7.19 – 7.10 (m, 3H), 4.48 (td, *J* = 11.3, 3.8 Hz, 1H), 3.24 – 3.02 (m, 2H), 2.90 (dd, *J* = 14.4, 4.0 Hz, 1H), 2.66 (dd, *J* = 14.4, 11.6 Hz, 1H), 2.22 (ddd, *J* = 16.3, 9.3, 6.7 Hz, 1H), 2.03 (ddd, *J* = 16.3, 9.4, 6.5 Hz, 1H), 1.87 – 1.76 (m, 1H), 1.74 – 1.63 (m, 1H), 1.46 – 1.31 (m, 4H), 1.27 (s, 12H), 1.23 – 1.12 (m, 3H), 0.85 (t, *J* = 6.9 Hz, 3H).

^{13}C NMR (101 MHz, Chloroform-*d*) δ 175.27, 138.56, 128.77, 128.29, 126.35, 83.48, 53.64, 42.55, 38.85, 31.45, 31.17, 28.21, 27.60, 25.12, 24.83, 23.04, 18.38, 14.09.

^{11}B NMR (160 MHz, Chloroform-*d*) δ 34.10.

HRMS (ESI) calcd for $C_{23}H_{37}NO_3B^+$ $[(M+H)^+]$ 386.2866, found 386.2871.

$[\alpha]_D$ (20.0 ℃, c = 1.0 in $CHCl_3$) = 7.92 °, 97% ee, from (*S*,*S*)-**L1**.

HPLC analysis indicated 97% ee (compound **84-OH**) after oxidation.

^{1}H NMR analysis of crude β-aminoboronates (purified by flash column chromatogram-phy) indicated 2.5:1 dr.(compound **83**: compound **84**).

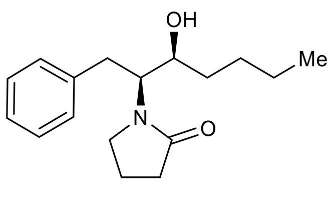

1-((2*S*,3*S*)-3-hydroxy-1-phenylheptan-2-yl)pyrrolidin-2-one (**83-OH**)

Following **GP-F3**, **83-OH** was obtained as yellow oil (48.4 mg, 88% yield, 97% ee).

^{1}H NMR (500 MHz, Chloroform-*d*) δ 7.32 – 7.24 (m, 2H), 7.24 – 7.16 (m, 3H), 3.74 – 3.58 (m, 2H), 3.24 (ddd, *J* = 9.8, 8.0, 5.6 Hz, 1H), 3.17 (dd, *J* = 13.9, 9.9 Hz, 1H),

2.97 (dd, J = 13.9, 5.6 Hz, 2H), 2.41 – 2.24 (m, 2H), 1.97 – 1.68 (m, 2H), 1.53 – 1.37 (m, 3H), 1.35 – 1.25 (m, 3H), 0.88 (t, J = 7.1 Hz, 3H).

^{13}C NMR (126 MHz, Chloroform-*d*) δ 176.71, 138.72, 129.12, 128.56, 126.56, 72.87, 61.87, 49.85, 35.16, 34.97, 31.71, 28.29, 22.81, 19.01, 14.16.

HRMS (ESI) calcd for $C_{17}H_{25}NO_2Na^+$ [$(M+Na)^+$] 298.1783, found 298.1779.

HPLC analysis: The ee was determined to be 97% on a CHIRALPAK AD-H column (8% *i*PrOH in hexane, 0.8 mL/min, 40 °C); retention times for compound obtained using (*S*,*S*)-**L1**: 11.4 min (minor), 13.3 min (major).

$[\alpha]_D$ (20.0 ℃, c = 1.0 in $CHCl_3$) = -67.54 °, 97% ee, from (*S*,*S*)-**L1**.

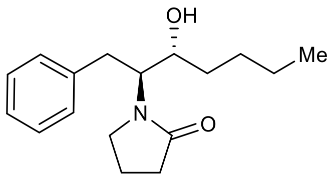

1-((2*S*,3*R*)-3-hydroxy-1-phenylheptan-2-yl)pyrrolidin-2-one (**84-OH**)

Following **GP-F3**, **84-OH** was obtained as yellow oil (47.8 mg, 87% yield, 96% ee).

^{1}H NMR (500 MHz, Chloroform-*d*) δ 7.33 – 7.24 (m, 2H), 7.24 – 7.12 (m, 3H), 3.98 – 3.83 (m, 1H), 3.59 (d, J = 11.6 Hz, 1H), 3.24 – 3.14 (m, 2H), 2.97 (dd, J = 14.3, 3.8 Hz, 1H), 2.78 – 2.67 (m, 1H), 2.37 – 2.23 (m, 2H), 1.84 – 1.69 (m, 2H), 1.65 – 1.55 (m, 1H), 1.56 – 1.45 (m, 2H), 1.42 – 1.29 (m, 3H), 0.92 (t, J = 6.9 Hz, 3H).

^{13}C NMR (126 MHz, Chloroform-*d*) δ 176.75, 139.11, 129.06, 128.60, 126.52, 74.19, 62.78, 49.66, 34.68, 31.92, 31.81, 28.50, 22.80, 18.93, 14.18.

HRMS (ESI) calcd for $C_{17}H_{25}NO_2Na^+$ [$(M+Na)^+$] 298.1783, found 298.1777.

HPLC analysis: The ee was determined to be 97% on a CHIRALPAK AD-H column (8% *i*PrOH in hexane, 0.8 mL/min, 40 °C); retention times for compound obtained using (*S*,*S*)-**L1**: 13.9 min (minor), 12.3 min (major).

$[\alpha]_D$ (20.0 ℃, c = 1.0 in $CHCl_3$) = -60.32 °, 97% ee, from (*S*,*S*)-**L1**.

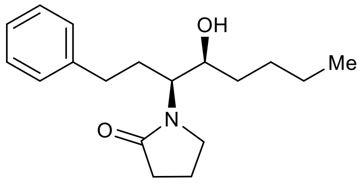

1-((3*S*,4*S*)-4-hydroxy-1-phenyloctan-3-yl)pyrrolidin-2-one (**85**)

Following concatenation of **GP-F1**, direct oxidation with $NaBO_3(4H_2O)$ (see **GP-F3**) and purification, **85** was obtained as colorless oil (43.4 mg, 30% total yield, 94% ee).

^{1}H NMR (500 MHz, Chloroform-*d*) δ 7.31 – 7.24 (m, 2H), 7.23 – 7.15 (m, 3H), 3.75 – 3.66 (m, 1H), 3.66 – 3.58 (m, 1H), 3.53 – 3.43 (m, 1H), 3.39 – 3.29 (m, 1H), 2.67 (ddd, *J* = 13.9, 9.4, 6.4 Hz, 1H), 2.55 (ddd, *J* = 14.0, 9.5, 6.0 Hz, 1H), 2.40 (t, *J* = 7.9 Hz, 2H), 2.11 – 1.88 (m, 4H), 1.49 – 1.37 (m, 3H), 1.37 – 1.26 (m, 3H), 0.89 (t, *J* = 7.1 Hz, 3H).

^{13}C NMR (101 MHz, Chloroform-*d*) δ 176.92, 141.56, 128.57, 128.39, 126.15, 72.99, 58.10, 47.12, 34.66, 32.88, 31.66, 30.30, 28.11, 22.81, 18.84, 14.20.

HRMS (ESI) calcd for $C_{18}H_{27}NO_2Na^+$ [$(M+Na)^+$] 312.1939, found 312.1936.

HPLC analysis: The ee was determined to be 94% on a CHIRALPAK AD-H column (10% *i*PrOH in hexane, 1.0 mL/min, 40 °C); retention times for compound obtained using (*S*,*S*)-**L1**: 7.1 min (minor), 11.1 min (major).

$[\alpha]_D$ (20.0 ℃, c = 1.0 in $CHCl_3$) = -4.88 °; 94% ee, from (*S*,*S*)-**L1**.

^{1}H NMR analysis of crude β-aminoboronates (purified by flash column chromatogram-phy) indicated 2.7:1 dr.

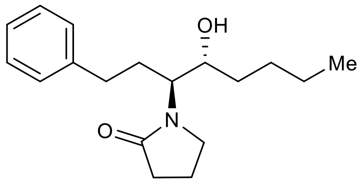

1-((3*S*,4*R*)-4-hydroxy-1-phenyloctan-3-yl)pyrrolidin-2-one (**86**)

Following concatenation of **GP-F1**, direct oxidation with $NaBO_3(4H_2O)$ (see **GP-F3**) and purification, **86** was obtained as colorless oil (15.9 mg, 11% total yield, 94% ee).

^{1}H NMR (500 MHz, Chloroform-*d*) δ 7.31 – 7.24 (m, 2H), 7.22 – 7.15 (m, 3H), 3.86 – 3.64 (m, 2H), 3.47 – 3.34 (m, 2H), 2.59 (td, *J* = 7.3, 6.3, 1.9 Hz, 2H), 2.39 (t, *J* = 8.1

Hz, 2H), 2.15 – 2.04 (m, 1H), 2.04 – 1.83 (m, 3H), 1.50 – 1.38 (m, 3H), 1.37 – 1.23 (m, 3H), 0.88 (t, *J* = 7.1 Hz, 3H).

^{13}C NMR (101 MHz, Chloroform-*d*) δ 176.48, 141.57, 128.56, 128.41, 126.17, 74.63, 58.19, 46.72, 34.36, 33.13, 31.67, 28.25, 27.57, 22.76, 18.81, 14.16.

HRMS (ESI) calcd for $C_{18}H_{27}NO_2Na^+$ [$(M+Na)^+$] 312.1939, found 312.1934.

HPLC analysis: The ee was determined to be 94% on a CHIRALCEL OD-H column (10% *i*PrOH in hexane, 1.0 mL/min, 40 °C); retention times for compound obtained using (*S,S*)-**L1**: 6.8 min (minor), 7.9 min (major).

$[\alpha]_D$ (20.0 °C, c = 1.0 in $CHCl_3$) = -1.79 °; 94% ee, from (*S,S*)-**L1**.

^{1}H NMR analysis of crude β-aminoboronates (purified by flash column chromatogram-phy) indicated 2.7:1 dr.

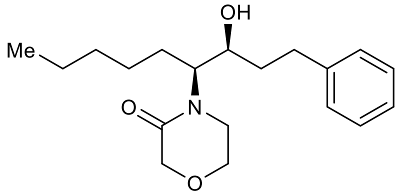

4-((3*S*,4*S*)-3-hydroxy-1-phenylnonan-4-yl)morpholin-3-one (**87**)

Following concatenation of **GP-F1**, direct oxidation with $NaBO_3(4H_2O)$ (see **GP-F3**) and purification, **87** was obtained as colorless oil (71.8 mg, 45% yield, 92% ee).

^{1}H NMR (500 MHz, Chloroform-*d*) δ 7.30 – 7.25 (m, 2H), 7.23 – 7.14 (m, 3H), 4.20 (s, 2H), 3.92 – 3.76 (m, 2H), 3.62 (s, 1H), 3.46 (ddd, *J* = 12.5, 6.2, 3.9 Hz, 1H), 3.27 (ddd, *J* = 12.4, 6.2, 3.8 Hz, 1H), 2.88 (ddd, *J* = 14.5, 9.2, 5.6 Hz, 1H), 2.70 (ddd, *J* = 13.7, 9.1, 7.1 Hz, 1H), 1.89 – 1.54 (m, 4H), 1.39 – 1.13 (m, 7H), 0.87 (t, *J* = 6.8 Hz, 3H).

^{13}C NMR (126 MHz, Chloroform-*d*) δ 168.36, 142.02, 128.57, 128.49, 125.94, 71.82, 68.06, 63.95, 36.78, 32.28, 31.70, 28.00, 25.98, 22.57, 14.07.

HRMS (ESI) calcd for $C_{19}H_{29}NO_3Na^+$ [$(M+Na)^+$] 342.2040, found 342.2049.

HPLC analysis: The ee was determined to be 92% on a CHIRALPAK AD-H column (10% *i*PrOH in hexane, 1.0 mL/min, 40 °C); retention times for compound obtained using (*S,S*)-**L1**: 7.1 min (minor), 9.6 min (major).

$[\alpha]_D$ (20.0 ℃, c = 1.0 in $CHCl_3$) = -13.08 °; 92% ee, from (*S*,*S*)-**L1**.

^{1}H NMR analysis of crude β-aminoboronates (purified by flash column chromatogram-phy) indicated 4.1:1 dr.

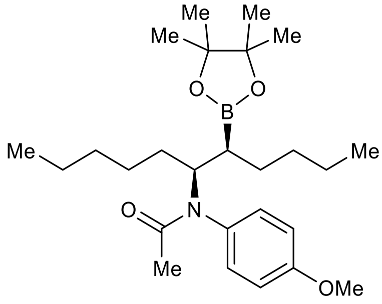

N-(4-methoxyphenyl)-*N*-((5*S*,6*S*)-5-(4,4,5,5-tetramethyl-1,3,2-dioxaborolan-2-yl)undecan-6-yl)acetamide (**88**)

Following **GP-F2**, **88** was obtained as yellow oil (131.4 mg, 59% yield, 91% ee).

^{1}H NMR (500 MHz, Chloroform-*d*) δ 7.40 (s, 1H), 7.03 – 6.75 (m, 3H), 4.75 (td, *J* = 9.9, 3.4 Hz, 1H), 3.81 (s, 3H), 1.70 (s, 3H), 1.66 – 1.57 (m, 1H), 1.48 – 1.41 (m, 1H), 1.39 – 1.25 (m, 10H), 1.22 (s, 6H), 1.20 (s, 6H), 1.17 – 1.10 (m, 2H), 1.04 (td, *J* = 10.0, 3.5 Hz, 1H), 0.92 – 0.78 (m, 6H).

^{13}C NMR (126 MHz, Chloroform-*d*) δ 171.07, 159.07, 133.07, 131.05, 114.39, 82.90, 57.58, 55.52, 32.04, 31.62, 30.50, 28.63, 26.47, 25.12, 25.01, 23.80, 23.13, 22.73, 14.24, 14.16.

^{11}B NMR (160 MHz, Chloroform-*d*) δ 31.60.

HRMS (ESI) calcd for $C_{26}H_{45}NO_4B^+$ [$(M+H)^+$] 446.3442, found 446.3444.

$[\alpha]_D$ (20.0 ℃, c = 1.0 in $CHCl_3$) = 1.06 °; 91% ee, from (*S*,*S*)-**L1**.

HPLC analysis indicated 91% ee (compound **88-OH**) after oxidation.

HPLC analysis of crude β-aminoalcohols (oxidized from crude β-aminoboronates) indicated 6.6:1 dr.

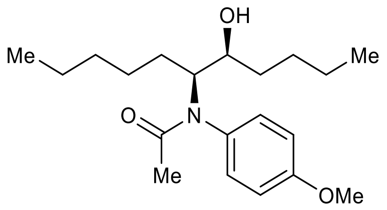

N-((5*S*,6*S*)-5-hydroxyundecan-6-yl)-*N*-(4-methoxyphenyl)acetamide (**88-OH**)

Following **GP-F3**, **88-OH** was obtained as brown oil (54.9 mg, 82% yield, 91% ee).

^{1}H NMR (400 MHz, Chloroform-*d*) δ 7.26 (s, 1H), 7.07 (s, 1H), 6.91 (d, *J* = 9.1 Hz, 2H), 4.00 (s, 1H), 3.83 (s, 3H), 3.41 (s, 1H), 1.91 – 1.72 (m, 4H), 1.55 – 1.19 (m, 14H), 0.94 – 0.80 (m, 6H).

^{13}C NMR (101 MHz, Chloroform-*d*) δ 173.54, 159.15, 129.77, 114.72, 71.86, 55.59, 35.37, 31.94, 28.72, 28.10, 26.60, 23.92, 22.93, 22.70, 14.24, 14.19.

HRMS (ESI) calcd for $C_{20}H_{34}NO_3^+$ [(M+H)$^+$] 336.2539, found 336.2529.

HPLC analysis: The ee was determined to be 91% on a CHIRALPAK AS-H column (10% *i*PrOH in hexane, 1.0 mL/min, 40 °C); retention times for compound obtained using (*S*,*S*)-**L1**: 5.9 min (minor), 6.8 min (major).

[α]$_D$ (20.0 ℃, c = 1.0 in $CHCl_3$) = -13.90 °; 91% ee, from (*S*,*S*)-**L1**.

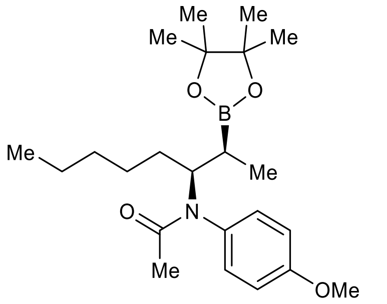

N-(4-methoxyphenyl)-*N*-((2*S*,3*S*)-2-(4,4,5,5-tetramethyl-1,3,2-dioxaborolan-2-yl)octan-3-yl)acetamide (**89**)

Following **GP-F1**, **89** was obtained as yellow oil (94.8 mg, 47% yield, 99.6% ee, 5.5:1 dr).

Following **GP-F2**, **89** was obtained as yellow oil (137.2 mg, 68% yield, 93% ee, 7.8:1 dr).

^{1}H NMR (500 MHz, Chloroform-*d*) δ 7.31 (s, 1H), 6.99 (s, 1H), 6.88 (d, *J* = 8.3 Hz, 2H), 4.58 (td, *J* = 9.4, 4.3 Hz, 1H), 3.79 (s, 3H), 1.70 (s, 3H), 1.66 – 1.56 (m, 1H), 1.42 – 1.04 (m, 20H), 0.89 (d, *J* = 7.4 Hz, 3H), 0.84 (t, *J* = 6.8 Hz, 3H).

^{13}C NMR (126 MHz, Chloroform-*d*) δ 171.11, 159.06, 133.17, 131.00, 114.38, 82.59, 59.12, 55.46, 31.97, 30.14, 26.35, 24.98, 24.85, 23.65, 22.65, 21.34, 14.17, 13.52.

^{11}B NMR (160 MHz, Chloroform-*d*) δ 30.40.

HRMS (ESI) calcd for $C_{23}H_{38}NO_4NaB^+$ [$(M+Na)^+$] 426.2792, found 426.2789.

[α]$_D$ (20.0 ℃, c = 1.0 in $CHCl_3$) = -0.35 °; 93% ee, from (*S*,*S*)-**L1**.

HPLC analysis indicated 93% ee (compound **89-OH**) after oxidation.

HPLC analysis of crude β-aminoalcohols (oxidized from crude β-aminoboronates) indicated 5.5:1 dr.

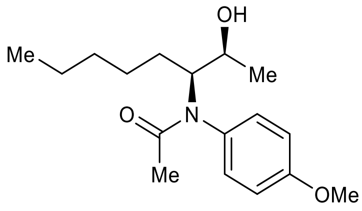

N-((2*S*,3*S*)-2-hydroxyoctan-3-yl)-*N*-(4-methoxyphenyl)acetamide (**89-OH**)

Following **GP-F3**, **89-OH** was obtained as yellow oil (46.9 mg, 80% yield, 93% ee).

^{1}H NMR (400 MHz, Chloroform-*d*) δ 7.26 (s, 1H), 7.06 (s, 1H), 6.94 – 6.79 (m, 2H), 4.08 (s, 1H), 3.81 (s, 3H), 3.65 – 3.52 (m, 1H), 1.80 (s, 3H), 1.45 – 1.34 (m, 3H), 1.33 – 1.14 (m, 8H), 0.87 (t, *J* = 7.0 Hz, 3H).

^{13}C NMR (101 MHz, Chloroform-*d*) δ 173.45, 159.10, 134.35, 129.89, 114.59, 67.83, 65.49, 55.48, 31.81, 28.62, 26.45, 23.73, 22.58, 21.83, 14.08.

HRMS (ESI) calcd for $C_{17}H_{27}NO_3Na^+$ [$(M+Na)^+$] 316.1889, found 316.1889.

HPLC analysis: The ee was determined to be 93% on a CHIRALPAK AD-H column (10% *i*PrOH in hexane, 1.0 mL/min, 40 °C); retention times for compound obtained using (*S*,*S*)-**L1**: 5.7 min (minor), 6.9 min (major).

[α]$_D$ (20.0 ℃, c = 1.0 in $CHCl_3$) = -9.62 °; 93% ee, from (*S*,*S*)-**L1**.

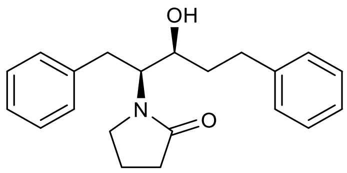

1-((2*S*,3*S*)-3-hydroxy-1,5-diphenylpentan-2-yl)pyrrolidin-2-one (**90**)

Following concatenation of **GP-F1**, direct oxidation with $NaBO_3(4H_2O)$ (see **GP-F3**) and purification, **90** was obtained as white solid (50.1 mg, 31% total yield, 97% ee).

^{1}H NMR (400 MHz, Chloroform-*d*) δ 7.29 (dtd, *J* = 8.3, 3.7, 1.2 Hz, 4H), 7.25 – 7.15 (m, 6H), 3.69 (dt, *J* = 9.1, 3.9 Hz, 1H), 3.59 (dt, *J* = 9.9, 4.7 Hz, 1H), 3.34 – 3.14 (m, 2H), 3.06 – 2.80 (m, 3H), 2.69 (ddd, *J* = 13.6, 9.7, 6.8 Hz, 1H), 2.41 – 2.25 (m, 2H), 1.94 – 1.59 (m, 4H).

^{13}C NMR (101 MHz, Chloroform-*d*) δ 176.93, 142.25, 138.69, 129.18, 128.68, 128.65, 128.54, 126.68, 125.97, 72.30, 62.99, 50.60, 37.45, 35.17, 32.57, 31.80, 19.03.

HRMS (ESI) calcd for $C_{21}H_{25}NO_2Na^+$ [$(M+Na)^+$] 346.1783, found 346.1784.

HPLC analysis: The ee was determined to be 97% on a CHIRALPAK AD-H column (10% *i*PrOH in hexane, 1.0 mL/min, 40 °C); retention times for compound obtained using (*S*,*S*)-**L1**: 10.9 min (minor), 13.1 min (major).

$[\alpha]_D$ (20.0 ℃, c = 1.0 in $CHCl_3$) = -7.26 °; 97% ee, from (*S*,*S*)-**L1**.

^{1}H NMR analysis of crude β-aminoboronates (purified by flash column chromatogram-phy) indicated 2.6:1 dr.

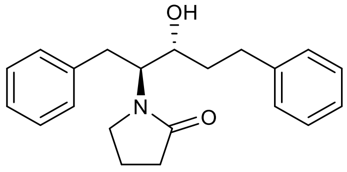

1-((2*S*,3*R*)-3-hydroxy-1,5-diphenylpentan-2-yl)pyrrolidin-2-one (**91**)

Following concatenation of **GP-F1**, direct oxidation with $NaBO_3(4H_2O)$ (see **GP-F3**) and purification, **91** was obtained as white glue (19.4 mg, 12% total yield, 98% ee).

^{1}H NMR (400 MHz, Chloroform-*d*) δ 7.34 – 7.11 (m, 10H), 3.94 (d, *J* = 9.0 Hz, 1H), 3.55 (d, *J* = 10.9 Hz, 1H), 3.28 – 3.09 (m, 2H), 3.04 – 2.86 (m, 2H), 2.71 (ddd, *J* = 13.7, 9.0, 7.0 Hz, 1H), 2.66 – 2.55 (m, 1H), 2.30 (t, *J* = 8.0 Hz, 2H), 2.07 – 1.88 (m, 1H),

1.86 – 1.63 (m, 3H).

^{13}C NMR (101 MHz, Chloroform-*d*) δ 176.94, 142.01, 138.94, 129.07, 128.71, 128.65, 128.60, 126.59, 126.07, 73.39, 63.29, 49.91, 36.81, 32.60, 31.94, 18.93.

HRMS (ESI) calcd for $C_{21}H_{25}NO_2Na^+$ [(M+Na)$^+$] 346.1783, found 346.1778.

HPLC analysis: The ee was determined to be 98% on a CHIRALPAK AD-H column (10% *i*PrOH in hexane, 1.0 mL/min, 40 °C); retention times for compound obtained using (*S,S*)-**L1**: 12.3 min (minor), 10.9 min (major).

[α]$_D$ (20.0 ℃, c = 1.0 in $CHCl_3$) = -33.54 °; 98% ee, from (*S,S*)-**L1**.

^{1}H NMR analysis of crude β-aminoboronates (purified by flash column chromatogram-phy) indicated 2.6:1 dr.

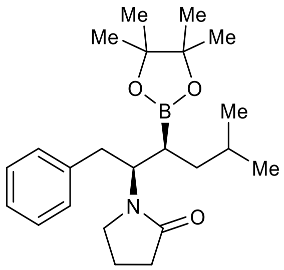

1-((2*S*,3*S*)-5-methyl-1-phenyl-3-(4,4,5,5-tetramethyl-1,3,2-dioxaborolan-2-yl)hexan-2-yl)pyrrolidin-2-one (**92**)

Following **GP-F1**, **92** was obtained as colorless oil (113.7 mg, 59% yield, 98% ee).

^{1}H NMR (500 MHz, Chloroform-*d*) δ 7.26 – 7.19 (m, 2H), 7.20 – 7.10 (m, 3H), 4.37 (ddd, *J* = 10.5, 9.0, 5.0 Hz, 1H), 3.30 (td, *J* = 8.8, 5.3 Hz, 1H), 3.17 (td, *J* = 8.8, 6.0 Hz, 1H), 3.11 (dd, *J* = 14.3, 5.0 Hz, 1H), 2.68 (dd, *J* = 14.3, 10.5 Hz, 1H), 2.15 (ddd, *J* = 16.2, 9.4, 6.3 Hz, 1H), 2.02 (ddd, *J* = 16.6, 9.6, 6.9 Hz, 1H), 1.87 – 1.74 (m, 1H), 1.76 – 1.63 (m, 1H), 1.62 – 1.40 (m, 3H), 1.29 – 1.17 (m, 13H), 0.88 (s, 3H), 0.86 (s, 3H).

^{13}C NMR (101 MHz, Chloroform-*d*) δ 174.54, 138.72, 128.98, 128.26, 126.26, 83.23, 54.42, 43.83, 38.30, 37.75, 31.37, 27.68, 26.39, 25.04, 25.02, 23.88, 22.03, 18.21.

^{11}B NMR (128 MHz, Chloroform-*d*) δ 32.69.

HRMS (ESI) calcd for $C_{23}H_{36}NO_3BNa^+$ [(M+Na)$^+$] 408.2686, found 408.2694.

[α]$_D$ (20.0 ℃, c = 1.0 in $CHCl_3$) = -35.70 °; 98% ee, from (*S,S*)-**L1**.

HPLC analysis indicated 98% ee (compound **95**) after oxidation.

^{1}H NMR analysis of crude β-aminoboronates (purified by flash column chromatography) indicated 4.2:1 dr.(compound **92**: compound **93**).

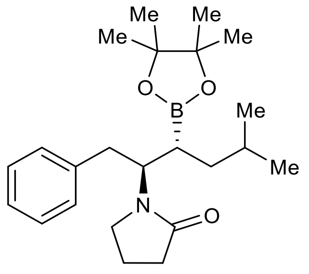

1-((2*S*,3*R*)-5-methyl-1-phenyl-3-(4,4,5,5-tetramethyl-1,3,2-dioxaborolan-2-yl)hexan-2-yl)pyrrolidin-2-one (**93**)

Following **GP-F1**, **93** was obtained as yellow oil (26.9 mg, 14% yield, 98% ee).

^{1}H NMR (500 MHz, Chloroform-*d*) δ 7.27 – 7.20 (m, 2H), 7.19 – 7.12 (m, 3H), 4.48 (td, *J* = 11.4, 4.0 Hz, 1H), 3.20 – 3.03 (m, 2H), 2.88 (dd, *J* = 14.4, 4.0 Hz, 1H), 2.69 (dd, *J* = 14.5, 11.6 Hz, 1H), 2.24 (ddd, *J* = 16.3, 9.2, 6.7 Hz, 1H), 2.04 (ddd, *J* = 16.3, 9.4, 6.4 Hz, 1H), 1.88 – 1.76 (m, 1H), 1.75 – 1.63 (m, 1H), 1.58 – 1.45 (m, 2H), 1.44 – 1.35 (m, 1H), 1.34 – 1.17 (m, 13H), 0.87 (dd, *J* = 6.5, 5.0 Hz, 6H).

^{13}C NMR (101 MHz, Chloroform-*d*) δ 175.38, 138.50, 128.80, 128.36, 126.42, 83.52, 53.85, 42.36, 38.94, 38.06, 31.49, 27.76, 25.89, 25.19, 24.90, 24.41, 21.84, 18.45.

^{11}B NMR (128 MHz, Chloroform-*d*) δ 33.03.

HRMS (ESI) calcd for $C_{23}H_{37}NO_3B^+$ $[(M+H)^+]$ 386.2866, found 386.2864.

$[\alpha]_D$ (20.0 ℃, c = 1.0 in $CHCl_3$) = 11.67 °, 98% ee, from (*S,S*)-**L1**.

HPLC analysis indicated 98% ee (compound **93-OH**) after oxidation.

^{1}H NMR analysis of crude β-aminoboronates (purified by flash column chromatography) indicated 4.2:1 dr.(compound **92**: compound **93**).

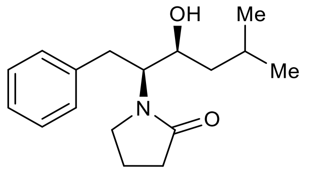

1-((2*S*,3*S*)-3-hydroxy-5-methyl-1-phenylhexan-2-yl)pyrrolidin-2-one (**95**)

Following **GP-F3**, **95** was obtained as white glue (48.4 mg, 88% yield, 98% ee).

^{1}H NMR (500 MHz, Chloroform-*d*) δ 7.30 – 7.25 (m, 2H), 7.23 – 7.17 (m, 3H), 3.75 (dt, *J* = 9.5, 3.9 Hz, 1H), 3.60 (dt, *J* = 9.9, 4.5 Hz, 1H), 3.28 – 3.15 (m, 2H), 2.97 (dd, *J* = 13.9, 5.4 Hz, 1H), 2.90 (q, *J* = 8.1 Hz, 1H), 2.40 – 2.25 (m, 2H), 1.90 – 1.73 (m, 3H), 1.41 (ddd, *J* = 14.2, 9.5, 5.1 Hz, 1H), 1.14 (ddd, *J* = 13.7, 8.8, 3.7 Hz, 1H), 0.91 (d, *J* = 6.6 Hz, 3H), 0.89 (d, *J* = 6.6 Hz, 3H).

^{13}C NMR (126 MHz, Chloroform-*d*) δ 176.75, 138.78, 129.12, 128.59, 126.59, 70.97, 62.66, 50.12, 44.36, 35.24, 31.76, 24.67, 23.73, 22.15, 19.04.

HRMS (ESI) calcd for $C_{17}H_{25}NO_2Na^+$ [$(M+Na)^+$] 298.1783, found 298.1778.

HPLC analysis: The ee was determined to be 98% on a CHIRALPAK AD-H column (10% *i*PrOH in hexane, 1.0 mL/min, 40 °C); retention times for compound obtained using (*S,S*)-**L1**: 6.9 min (minor), 7.3 min (major).

$[\alpha]_D$ (20.0 °C, c = 1.0 in $CHCl_3$) = -80.20 °, 98% ee, from (*S,S*)-**L1**.

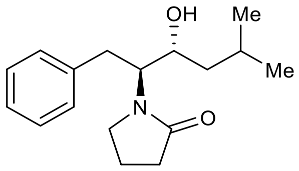

1-((2*S*,3*R*)-3-hydroxy-5-methyl-1-phenylhexan-2-yl)pyrrolidin-2-one **(93-OH)**

Following **GP-F3**, **93-OH** was obtained as white solid (49.0 mg, 89% yield, 98% ee).

^{1}H NMR (500 MHz, Chloroform-*d*) δ 7.30 – 7.26 (m, 2H), 7.24 – 7.14 (m, 3H), 4.45 (s, 1H), 4.07 – 3.94 (m, 1H), 3.63 – 3.50 (m, 1H), 3.26 – 3.13 (m, 2H), 2.97 (dd, *J* = 14.3, 3.9 Hz, 1H), 2.70 (ddd, *J* = 9.6, 8.3, 6.1 Hz, 1H), 2.36 – 2.26 (m, 2H), 1.90 – 1.70 (m, 4H), 1.64 – 1.54 (m, 1H), 0.96 (d, *J* = 6.7 Hz, 3H), 0.94 (d, *J* = 6.6 Hz, 3H).

^{13}C NMR (126 MHz, Chloroform-*d*) δ 176.82, 139.14, 129.10, 128.63, 126.55, 72.12, 63.37, 49.80, 44.07, 31.92, 31.89, 24.91, 23.70, 22.02, 18.95.

HRMS (ESI) calcd for $C_{17}H_{25}NO_2Na^+$ [$(M+Na)^+$] 298.1783, found 298.1780.

HPLC analysis: The ee was determined to be 98% on a CHIRALPAK AD-H column (10% *i*PrOH in hexane, 1.0 mL/min, 40 °C); retention times for compound obtained using (*S,S*)-**L1**: 8.6 min (minor), 7.8 min (major).

[α]D (20.0 ℃, c = 1.0 in $CHCl_3$) = -41.10 °; 98% ee, from (*S*,*S*)-**L1**.

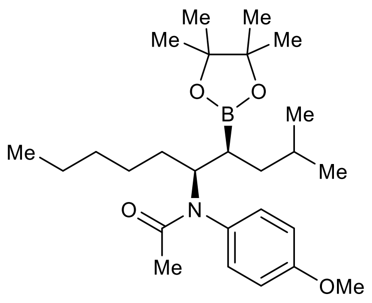

N-(4-methoxyphenyl)-*N*-((4*S*,5*S*)-2-methyl-4-(4,4,5,5-tetramethyl-1,3,2-dioxaborolan-2-yl)decan-5-yl)acetamide (**94**)

Following **GP-F2**, **94** was obtained as yellow oil (149.2 mg, 67% yield, 92% ee).

^{1}H NMR (500 MHz, Chloroform-*d*) δ 7.43 (s, 1H), 7.07 – 6.86 (m, 3H), 4.64 (td, *J* = 9.6, 4.0 Hz, 1H), 3.82 (s, 3H), 1.72 (s, 3H), 1.70 – 1.62 (m, 1H), 1.57 – 1.43 (m, 2H), 1.39 – 1.24 (m, 6H), 1.22 (s, 6H), 1.19 (s, 6H), 1.18 – 1.10 (m, 2H), 1.09 – 1.01 (m, 1H), 0.90 – 0.83 (m, 6H), 0.80 (d, *J* = 6.3 Hz, 3H).

^{13}C NMR (126 MHz, Chloroform-*d*) δ 171.16, 159.08, 133.20, 131.01, 114.41, 82.76, 58.55, 55.50, 38.41, 31.98, 30.57, 27.69, 26.47, 25.11, 25.01, 23.86, 23.80, 22.70, 22.14, 14.22.

^{11}B NMR (160 MHz, Chloroform-*d*) δ 30.51.

HRMS (ESI) calcd for $C_{26}H_{45}NO_4B^+$ $[(M+H)^+]$ 446.3442, found 446.3452.

[α]D (20.0 ℃, c = 1.0 in $CHCl_3$) = -0.14 °; 92% ee, from (*S*,*S*)-**L1**.

HPLC analysis indicated 92% ee (compound **94-OH**) after oxidation.

^{1}H NMR analysis indicated 8.3:1 dr.

HPLC analysis of crude β-aminoalcohols (oxidized from crude β-aminoboronates) indicated 8.3:1 dr.

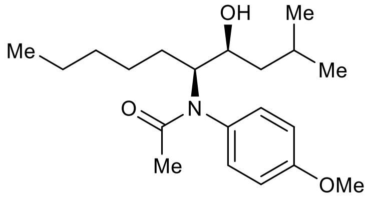

N-((4*S*,5*S*)-4-hydroxy-2-methyldecan-5-yl)-*N*-(4-methoxyphenyl)acetamide (**94-OH**)

Following **GP-F3**, **94** was obtained as yellow oil (53.6 mg, 80% yield, 92% ee).

^{1}H NMR (500 MHz, Chloroform-*d*) δ 7.26 (s, 1H), 7.08 (s, 1H), 6.95 – 6.87 (m, 2H), 3.93 (s, 1H), 3.83 (s, 3H), 3.52 (s, 1H), 1.95 – 1.85 (m, 1H), 1.81 (s, 3H), 1.56 – 1.18 (m, 10H), 0.94 (d, *J* = 6.7 Hz, 3H), 0.90 – 0.84 (m, 6H).

^{13}C NMR (126 MHz, Chloroform-*d*) δ 173.51, 159.16, 129.70, 114.76, 69.93, 55.60, 45.04, 31.96, 28.82, 26.66, 24.71, 24.13, 23.94, 22.71, 21.83, 14.19.

HRMS (ESI) calcd for $C_{20}H_{33}NO_3Na^+$ [(M+Na)$^+$] 358.2342, found 358.2350.

HPLC analysis: The ee was determined to be 92% on a CHIRALPAK AD-H column (8% *i*PrOH in hexane, 0.8 mL/min, 40 °C); retention times for compound obtained using (*S*,*S*)-**L1**: 5.4 min (minor), 5.8 min (major).

$[\alpha]_D$ (20.0 ℃, c = 1.0 in $CHCl_3$) = -16.79 °; 97% ee, from (*S*,*S*)-**L1**.

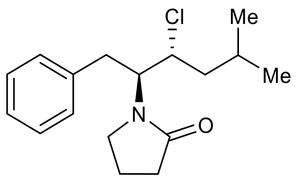

1-((2*S*,3*R*)-3-chloro-5-methyl-1-phenylhexan-2-yl)pyrrolidin-2-one (**96**)

According to the reported literature[14], **96** was obtained form **92-OH** as yellow oil (49.8 mg, 85% yield, 97% ee).

Triphenylphosphine (57.6 mg, 0.22 mmol) and **92-OH** (55.4 mg, 0.20 mmol) were stirred in CCl_4 (2 mL) in a pressure tube at 100 °C for 2 h. The reaction mixture was cooled to room temperature, the solvent was removed and the residue was purified by flash column chromatography on silica gel to give the target product.

^{1}H NMR (500 MHz, Chloroform-*d*) δ 7.33 – 7.24 (m, 2H), 7.23 – 7.15 (m, 3H), 4.47 (ddd, *J* = 11.1, 6.2, 4.2 Hz, 1H), 4.21 (ddd, *J* = 10.8, 6.1, 3.4 Hz, 1H), 3.37 – 3.27 (m, 2H), 3.29 – 3.19 (m, 1H), 2.99 (dd, *J* = 15.0, 11.6 Hz, 1H), 2.26 (ddd, *J* = 16.4, 9.4, 6.8 Hz, 1H), 2.11 (ddd, *J* = 16.5, 9.4, 6.5 Hz, 1H), 2.03 – 1.90 (m, 1H), 1.92 – 1.79 (m, 1H), 1.77 – 1.63 (m, 2H), 1.49 (ddd, *J* = 14.2, 9.6, 3.4 Hz, 1H), 0.97 (d, *J* = 6.7 Hz, 3H), 0.91 (d, *J* = 6.6 Hz, 3H).

^{13}C NMR (126 MHz, Chloroform-*d*) δ 175.52, 137.70, 128.66, 128.60, 126.63, 63.76, 57.31, 45.17, 44.50, 33.53, 31.17, 25.27, 23.41, 21.06, 18.81.

HRMS (ESI) calcd for $C_{17}H_{25}NOCl^+$ [$(M+H)^+$] 294.1625, found 294.1624.

HPLC analysis: The ee was determined to be 97% on a CHIRALPAK AD-H column (10% *i*PrOH in hexane, 1.0 mL/min, 40 °C); retention times for compound obtained using (*S,S*)-**L1**: 4.9 min (minor), 5.7 min (major).

$[\alpha]_D$ (20.0 ℃, c = 1.0 in $CHCl_3$) = 11.12 °, 97% ee, from (*S,S*)-**L1**.

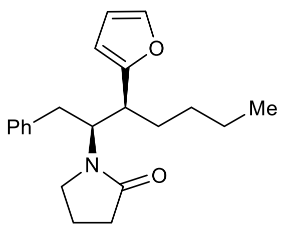

1-((2*S*,3*S*)-3-(furan-2-yl)-1-phenylheptan-2-yl)pyrrolidin-2-one (**97**)

Following the modified methods in the reported literature[15], **97** was obtained from **83** as pale-yellow oil (32.5 mg, 50% yield, 97% ee).

Under argon protection, a Schlenk tube was charged with furan (0.25 mmol, 1.25 equiv.). The solution was diluted with 1 mL THF. The flask was capped, removed from the box, and cooled to -78 °C. A solution of *n*BuLi (0.25 mmol, 1.25 equiv.) was added dropwise. The resulting mixture was warmed to rt and stirred for 1 h. Then the solution of 1b (0.20 mmol) in THF (1 mL) was added dropwise to the solution of lithiated furan at -78 °C. After 1 h at -78 °C, the resulting solution was added dropwise to a solution of NBS (71 mg, 2 equiv.) in THF (1 mL). After stirring at -78 °C for 1 h, a 20% aqueous solution of $Na_2S_2O_3$ was added. The mixture was warmed to rt and extracted with ethyl acetate (15 mL) twice. The combined organic layers were combined, dried over Na_2SO_4, and concentrated in vacuo. The residue was purified by flash column chromatography on silica gel to give the target product.

^{1}H NMR (500 MHz, Chloroform-*d*) δ 7.32 (d, *J* = 1.8 Hz, 1H), 7.30 – 7.23 (m, 2H), 7.23 – 7.14 (m, 3H), 6.28 (dd, *J* = 3.2, 1.9 Hz, 1H), 6.09 (d, *J* = 3.2 Hz, 1H), 4.31 (s, 1H), 3.15 (td, *J* = 8.8, 5.1 Hz, 1H), 3.09 (dd, *J* = 14.2, 5.0 Hz, 1H), 2.94 (dd, *J* = 14.3,

10.9 Hz, 1H), 2.88 – 2.81 (m, 1H), 2.76 – 2.67 (m, 1H), 2.11 – 1.94 (m, 2H), 1.82 – 1.72 (m, 2H), 1.63 – 1.49 (m, 2H), 1.38 – 1.08 (m, 4H), 0.85 (t, *J* = 7.1 Hz, 3H).

^{13}C NMR (101 MHz, Chloroform-*d*) δ 175.22, 155.81, 141.00, 138.58, 128.91, 128.49, 126.47, 110.27, 106.99, 57.08, 45.47, 42.01, 36.49, 31.55, 31.02, 29.70, 22.75, 18.83, 14.14.

HRMS (ESI) calcd for $C_{21}H_{27}NO_2Na^+$ $[(M+Na)^+]$ 348.1939, found 348.1937.

HPLC analysis: The ee was determined to be 97% on a CHIRALPAK AD-H column (10% *i*PrOH in hexane, 1.0 mL/min, 40 °C); retention times for compound obtained using (*S*,*S*)-**L1**: 6.7 min (minor), 7.3 min (major).

$[\alpha]_D$ (20.0 ℃, c = 1.0 in $CHCl_3$) = -29.50 °; 97% ee, from (*S*,*S*)-**L1**.

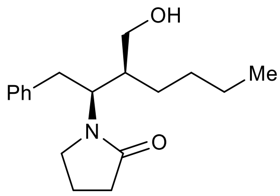

1-((2*S*,3*S*)-3-(hydroxymethyl)-1-phenylheptan-2-yl)pyrrolidin-2-one (**98**)

According to the reported literature[15], **98** was obtained from **83** as pale-yellow oil (46.3 mg, 80% yield, 97% ee).

Under argon protection, a Schlenk tube was charged with **83** (0.20 mmol, 1.0 equiv.), dibromomethane (0.5 mmol, 2.5 equiv.) and THF (2 mL). The flask was capped and cooled to -78 °C. A solution of *n*BuLi (0.44 mmol, 2.5 M in hexanes, 2.2 equiv.) was added dropwise. The resulting mixture was allowed to stir for 10 min, warmed to rt and stirred for an additional 2 h. The reaction mixture was cooled to 0 °C, and a premixed solution of NaOH (2 M, aq.)/30% H_2O_2 (2:1, 3 mL) was added dropwise. The resulting solution was warmed to rt and allowed to stir for 3 h before being quenched with water (10 mL). The mixture was extracted with ethyl acetate (15 mL) twice. The organic layers were combined, dried over Na_2SO_4 and concentrated in vacuo. The residue was purified by flash column chromatography on silica gel to give the target product.

^{1}H NMR (500 MHz, Chloroform-*d*) δ 7.30 – 7.22 (m, 2H), 7.20 – 7.10 (m, 3H), 4.39 (td, *J* = 11.8, 3.8 Hz, 1H), 3.58 (dd, *J* = 12.2, 2.4 Hz, 1H), 3.40 (dd, *J* = 12.3, 2.4 Hz,

1H), 3.27 – 3.15 (m, 2H), 3.07 (td, *J* = 8.9, 6.3 Hz, 1H), 2.89 (s, 1H), 2.70 (dd, *J* = 15.1, 12.1 Hz, 1H), 2.29 (ddd, *J* = 16.6, 9.3, 7.0 Hz, 1H), 2.14 (ddd, *J* = 16.6, 9.5, 6.2 Hz, 1H), 1.92 – 1.77 (m, 1H), 1.72 – 1.57 (m, 2H), 1.59 – 1.46 (m, 2H), 1.48 – 1.38 (m, 1H), 1.41 – 1.26 (m, 3H), 0.92 (t, *J* = 7.0 Hz, 3H).

^{13}C NMR (126 MHz, Chloroform-*d*) δ 176.95, 138.27, 128.61, 128.25, 126.49, 60.71, 52.65, 43.57, 42.62, 35.61, 31.08, 29.79, 27.88, 23.16, 18.42, 14.18.

HRMS (ESI) calcd for $C_{18}H_{27}NO_2Na^+$ [$(M+Na)^+$] 312.1939, found 312.1935.

HPLC analysis: The ee was determined to be 97% on a CHIRALPAK AD-H column (15% *i*PrOH in hexane, 1.5 mL/min, 40 °C); retention times for compound obtained using (*S,S*)-**L1**: 4.0 min (minor), 5.1 min (major).

$[\alpha]_D$ (20.0 °C, c = 1.0 in $CHCl_3$) = 0.89 °, 97% ee, from (*S,S*)-**L1**.

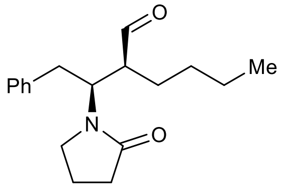

(*S*)-2-((*S*)-1-(2-oxopyrrolidin-1-yl)-2-phenylethyl)hexanal (**99**)

According to the reported literature[16], **99** was obtained as yellow oil (34.4 mg, 60% yield, 97% ee).

Under argon protection, a Schlenk tube was charged with dichloromethane (1.0 mmol, 5 equiv.) and THF (7.5 mL). The flask was capped and cooled to -78 °C. A solution of *n*BuLi (0.9 mmol, 2.5 M in hexanes, 4.5 equiv.) was added dropwise. The resulting mixture was allowed to stir for 45 min. A solution of **83** (0.2 mmol, 1.0 equiv.) in THF (2.5 mL) was added to the solution of Cl_2CHLi at –78 °C, and the resulting mixture was stirred at –30 °C for 45 min. Next, $ZnCl_2$ (1.0 mmol, 5.0 equiv.; the addition of ZnCl2 leads to a cleaner reaction) was added, and then the mixture was stirred at room temperature for 2 h. Water (pH = 7, phosphate buffer; 5 mL) and $NaBO_3(4H_2O)$ (1.0 mmol, 5.0 equiv.) were added, and the suspension was stirred at room temperature for 4 h. The mixture was extracted with ethyl acetate (15 mL) twice. The organic layers were combined, dried over Na_2SO_4 and concentrated in vacuo. The residue was purified

by flash column chromatography on silica gel to give the target product.

^{1}H NMR (400 MHz, Chloroform-*d*) δ 9.49 (d, J = 4.7 Hz, 1H), 7.28 (d, J = 106.4 Hz, 2H), 7.23 – 7.15 (m, 3H), 4.64 – 4.52 (m, 1H), 3.23 – 3.02 (m, 3H), 2.86 – 2.74 (m, 1H), 2.70 – 2.59 (m, 1H), 2.27 – 2.12 (m, 1H), 2.11 – 2.00 (m, 1H), 1.84 – 1.59 (m, 4H), 1.47 – 1.27 (m, 4H), 0.90 (t, J = 6.9 Hz, 3H).

^{13}C NMR (101 MHz, Chloroform-*d*) δ 203.54, 175.59, 137.24, 128.77, 128.66, 126.86, 55.36, 52.37, 44.34, 35.73, 31.16, 29.26, 27.14, 22.83, 18.47, 13.95.

HRMS (ESI) calcd for $C_{18}H_{26}NO_2^+$ [(M+Na)$^+$] 288.1964, found 288.1963.

HPLC analysis: The ee was determined to be 97% on a CHIRALPAK AD-H column (8% *i*PrOH in hexane, 0.8 mL/min, 40 °C); retention times for compound obtained using (*S,S*)-**L1**: 10.6 min (minor), 11.2 min (major).

$[\alpha]_D$ (20.0 ℃, c = 1.0 in $CHCl_3$) = -48.93 °; 97% ee, from (*S,S*)-**L1**.

Supplementary Note 1

Synthetic Transformations of Enamide/Enecarbamate Reductive Hydroalkylation Products

Examples Described in Figure 6

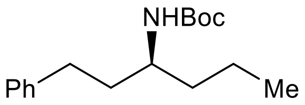

tert-butyl (*S*)-(1-phenylhexan-3-yl)carbamate (**100-Boc**)

Following **GP-E2**, from enecarbamate **(*Z*)-68** and alkyl halide **101**, **100-Boc** was obtained as white solid (43.2 mg, 78%, 90% ee).

Following **GP-E2**, from enecarbamate **(*Z*)-102** and alkyl halide **103**, **100-Boc** was obtained as white solid (32.2 mg, 58%, 91% ee).

^{1}H NMR (500 MHz, Chloroform-*d*) δ 7.29 – 7.25 (m, 2H), 7.18 (d, *J* = 7.6 Hz, 3H), 4.31 (s, 1H), 3.69 – 3.49 (m, 1H), 2.74 – 2.57 (m, 2H), 1.83 – 1.72 (m, 1H), 1.68 – 1.57 (m, 1H), 1.48 – 1.44 (m, 10H), 1.44 – 1.22 (m, 3H), 0.90 (t, *J* = 6.9 Hz, 3H).

^{13}C NMR (126 MHz, Chloroform-*d*) δ 155.75, 142.18, 128.37, 125.78, 78.90, 50.41, 37.93, 37.63, 32.40, 28.45, 19.07, 14.01.

HRMS (ESI) calcd for $C_{17}H_{27}NO_2Na^+$ $[(M+Na)^+]$ 300.1934, found 300.1936.

HPLC analysis: The ee was determined to be 90% & 91% on a CHIRALPAK AD-H column (10% *i*PrOH in hexane, 1.0 mL/min, 40 °C); retention times for compound obtained using (*S*)-**L2** & (*R*)-**L2**: 4.3 min (minor), 4.6 min (major).

$[\alpha]_D$ (20.0 °C, c = 1.0 in $CHCl_3$) = -7.90°; 90% ee, from (*S*)-**L2**.

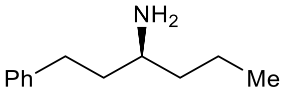

(*S*)-1-phenylhexan-3-amine (**100**)

According to the reported literature[17], from **(*Z*)-68** and **101**, **100** was obtained as white

solid (39.7 mg, 92%, 88% ee).

According to the reported literature[17], from **(*Z*)-102** and **103**, **100** was obtained as white solid (29.3 mg, 91%, 91% ee).

<u>^{1}H NMR</u> (400 MHz, Chloroform-*d*) δ 7.31 – 7.26 (m, 2H), 7.23 – 7.15 (m, 3H), 2.82 – 2.69 (m, 2H), 2.62 (ddd, *J* = 13.7, 10.3, 6.1 Hz, 1H), 1.80 – 1.70 (m, 1H), 1.64 – 1.53 (m, 3H), 1.47 – 1.38 (m, 2H), 1.35 – 1.26 (m, 2H), 0.92 (t, *J* = 6.9 Hz, 3H).

<u>^{13}C NMR</u> (101 MHz, Chloroform-*d*) δ 142.40, 128.35, 128.34, 125.73, 50.59, 40.25, 39.76, 32.58, 19.23, 14.20.

<u>HRMS (ESI)</u> calcd for $C_{12}H_{20}N^+$ [$(M+H)^+$] 178.1590, found 178.1592.

<u>HPLC analysis</u>: The ee was determined by the product of **100** with $(Boc)_2O$ and Et_3N to be 88% & 91% on a CHIRALPAK AD-H column (10% *i*PrOH in hexane, 1.0 mL/min, 40 °C); retention times for compound obtained using (*S*)-**L**2 & (*R*)-**L**2: 4.3 min (major), 4.6 min (minor).

$[\alpha]_D$ (20.0 °C, c = 1.0 in $CHCl_3$) = +7.81°; 88% ee, from (*S*)-**L2**.

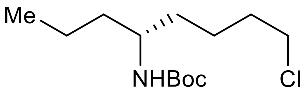

tert-butyl (*R*)-(8-chlorooctan-4-yl)carbamate (**106**)

Following **GP-E2**, **106** was obtained as colorless oil (36.9mg, 70%, 91% ee).

<u>^{1}H NMR</u> (500 MHz, Chloroform-*d*) δ 4.25 (d, *J* = 9.4 Hz, 1H), 3.64 – 3.38 (m, 3H), 1.84 – 1.69 (m, 2H), 1.53 – 1.38 (m, 13H), 1.38 – 1.26 (m, 4H), 0.93 – 0.86 (m, 3H).

<u>^{13}C NMR</u> (126 MHz, Chloroform-*d*) δ 155.77, 78.94, 50.18, 44.98, 37.76, 34.88, 32.44, 28.43, 23.19, 19.12, 14.02.

<u>HRMS (ESI)</u> calcd for $C_{13}H_{26}ClNO_2Na^+$ [$(M+Na)^+$] 286.1544, found 286.1542.

<u>HPLC analysis</u>: The ee was determined by the product of **106** with NaI to be 91% on a CHIRALPAK AD-H column (8% *i*PrOH in hexane, 0.8 mL/min, 40 °C); retention times for compound obtained using (*S*)-**L2**: 5.3 min (major), 5.6 min (minor).

$[\alpha]_D$ (20.0 °C, c = 1.0 in $CHCl_3$) = +4.60°; 91% ee, from (*S*)-**L2**.

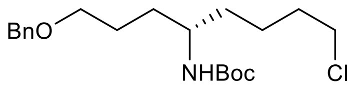

tert-butyl (*S*)-(1-(benzyloxy)-8-chlorooctan-4-yl)carbamate (**107**)

Following **GP-E2**, **107** was obtained as colorless oil (45.1mg, 62%, 89% ee).

^{1}H NMR (500 MHz, Chloroform-*d*) δ 7.37 – 7.31 (m, 4H), 7.30 – 7.26 (m, 1H), 4.49 (s, 2H), 4.33 (d, *J* = 9.0 Hz, 1H), 3.62 – 3.42 (m, 5H), 1.83 – 1.72 (m, 2H), 1.70 – 1.55 (m, 3H), 1.54 – 1.31 (m, 14H).

^{13}C NMR (126 MHz, Chloroform-*d*) δ 155.77, 138.52, 128.37, 127.65, 127.55, 79.01, 72.94, 70.08, 50.22, 44.93, 34.96, 32.38, 32.12, 28.43, 26.21, 23.21.

HRMS (ESI) calcd for $C_{20}H_{32}ClNO_3Na^+$ [$(M+Na)^+$] 392.1963, found 392.1967.

HPLC analysis: The ee was determined to be 89% on a CHIRALCEL OD-H column (5% *i*PrOH in hexane, 1.0 mL/min, 40 °C); retention times for compound obtained using (*S*)-**L2**: 7.0 min (major), 7.7 min (minor).

$[\alpha]_D$ (20.0 °C, c = 1.0 in $CHCl_3$) = +0.79°; 89% ee, from (*S*)-**L2**.

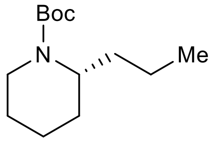

tert-butyl (*R*)-2-propylpiperidine-1-carboxylate (**108**)

According to the reported literature[18], **108** was obtained from **106** as colorless oil (29.1 mg, 64%).

^{1}H NMR (500 MHz, Chloroform-*d*) δ 4.19 (d, *J* = 8.8 Hz, 1H), 3.95 (d, *J* = 13.5 Hz, 1H), 2.73 (td, *J* = 13.3, 2.6 Hz, 1H), 1.69 – 1.60 (m, 1H), 1.59 – 1.49 (m, 5H), 1.44 (s, 9H), 1.40 – 1.31 (m, 2H), 1.29 – 1.21 (m, 2H), 0.90 (t, *J* = 7.3 Hz, 3H).

^{13}C NMR (101 MHz, Chloroform-*d*) δ 155.20, 78.95, 50.13, 38.64, 31.91, 28.50, 25.70, 19.48, 19.05, 14.09.

HRMS (ESI) calcd for $C_{13}H_{25}NO_2Na^+$ [$(M+Na)^+$] 250.1777, found 250.1776.

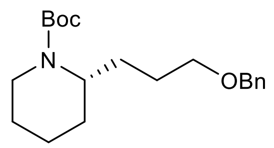

tert-butyl (*S*)-2-(3-(benzyloxy)propyl)piperidine-1-carboxylate (**109**)

According to the reported literature[18], **109** was obtained from **107** as colorless oil (52.7 mg, 79%).

<u>^{1}H NMR</u> (500 MHz, Chloroform-*d*) δ 7.37 – 7.31 (m, 4H), 7.30 – 7.26 (m, 1H), 4.50 (s, 2H), 4.21 (s, 1H), 3.96 (d, *J* = 13.5 Hz, 1H), 3.49 (qt, *J* = 9.3, 6.2 Hz, 2H), 2.79 – 2.70 (m, 1H), 1.82 – 1.70 (m, 2H), 1.61 – 1.52 (m, 7H), 1.44 (s, 9H), 1.41 – 1.34 (m, 1H).

<u>^{13}C NMR</u> (126 MHz, Chloroform-*d*) δ 155.16, 138.65, 128.35, 127.61, 127.50, 79.07, 72.85, 70.22, 50.26, 38.78, 29.71, 28.51, 26.60, 26.35, 25.66, 19.05.

<u>HRMS (ESI)</u> calcd for $C_{20}H_{31}NO_3Na^+$ [$(M+Na)^+$] 356.2196, found 356.2193.

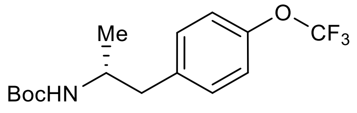

tert-butyl (*R*)-(1-(4-(trifluoromethoxy)phenyl)propan-2-yl)carbamate (**111**)

Following **GP-E2**, **111** was obtained as white solid (56.8 mg, 89%, 81% ee).

<u>^{1}H NMR</u> (400 MHz, Chloroform-*d*) δ 7.22 – 7.15 (m, 2H), 7.12 (d, J = 8.3 Hz, 2H), 4.39 (s, 1H), 3.89 (s, 1H), 2.86 – 2.60 (m, 2H), 1.40 (s, 9H), 1.08 (d, J = 6.7 Hz, 3H).

<u>^{13}C NMR</u> (126 MHz, Chloroform-*d*) δ 155.20, 147.87 (d, *J* = 2.3 Hz), 137.22, 130.76, 120.86, 120.56 (q, *J* = 256.6 Hz), 79.27, 47.45, 42.47, 28.39, 20.23.

<u>^{19}F NMR</u> (471 MHz, Chloroform-*d*) δ -57.94.

<u>HRMS (ESI)</u> calcd for $C_{15}H_{20}F_3NO_3Na^+$ [$(M+Na)^+$] 342.1287, found 342.1287.

<u>HPLC analysis</u>: The ee was determined to be 81% on a CHIRALPAK AD-H column (5% *i*PrOH in hexane, 1.0 mL/min, 25 °C); retention times for compound obtained using (*S*)-**L2**: 4.8 min (minor), 5.2 min (major).

$[\alpha]_D$ (20.0 °C, c = 1.0 in $CHCl_3$) = +2.16°; 81% ee, from (*S*)-**L2**.

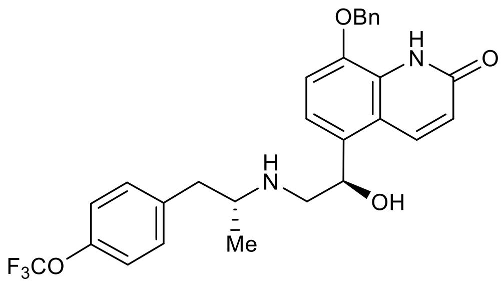

8-(benzyloxy)-5-((*R*)-1-hydroxy-2-(((*R*)-1-(4-(trifluoromethoxy)phenyl)propan-2-yl)amino)ethyl)quinolin-2(1*H*)-one (**113**)

According to the reported literature[19], **113** was obtained from **111** as colorless oil (41.0 mg, 40%, 90.5:9.5 dr).

<u>^{1}H NMR</u> (500 MHz, Chloroform-*d*) δ 8.07 (dd, *J* = 9.9, 2.2 Hz, 1H), 7.43 – 7.34 (m, 5H), 7.23 – 7.07 (m, 5H), 6.96 (dd, *J* = 8.3, 2.2 Hz, 1H), 6.61 (d, *J* = 9.9 Hz, 1H), 5.19 – 5.07 (m, 3H), 3.06 – 2.90 (m, 2H), 2.86 – 2.68 (m, 2H), 2.68 – 2.60 (m, 1H), 1.10 (d, *J* = 6.2 Hz, 3H).

<u>^{13}C NMR</u> (126 MHz, Chloroform-*d*) δ 161.49, 147.85, 144.02 (d, *J* = 2.0 Hz), 137.55, 136.67, 135.52, 131.14, 130.58, 129.07, 128.89, 128.70, 127.92, 122.25, 121.02, 120.51 (q, *J* = 257.0 Hz), 119.92, 117.38, 111.03, 71.01, 69.06, 54.93, 53.75, 42.59, 20.06.

<u>^{19}F NMR</u> (471 MHz, Chloroform-*d*) δ 57.85.

<u>HRMS (ESI)</u> calcd for $C_{28}H_{28}F_3N_2O_4^+$ [$(M+H)^+$] 513.1996, found 513.2010.

<u>HPLC analysis</u>: The dr was determined to be 90.5:9.5 on a CHIRALCEL OD-H column (15% *i*PrOH in hexane, 1.5 mL/min, 40 °C); retention times for compound obtained using (*S*)-**L2**: 17.4 min (major), 27.0 min (minor).

[α]$_D$ (20.0 °C, c = 1.0 in $CHCl_3$) = -31.45°; 90:10 dr, from (*S*)-**L2**.

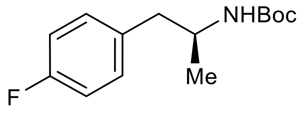

tert-butyl (*R*)-(1-(4-fluorophenyl)propan-2-yl)carbamate (**114**)

Following **GP-E2**, **114** was obtained as white solid (44.6 mg, 88%, 80% ee).

<u>^{1}H NMR</u> (400 MHz, Chloroform-*d*) (400 MHz, Chloroform-*d*) δ 7.19 – 7.07 (m, 2H), 6.96 (td, *J* = 8.8, 0.9 Hz, 2H), 4.37 (s, 1H), 3.86 (s, 1H), 2.79 (dd, *J* = 13.5, 5.7 Hz, 1H),

2.63 (dd, J = 13.5, 7.2 Hz, 1H), 1.41 (d, J = 1.0 Hz, 9H), 1.07 (dd, J = 6.6, 0.9 Hz, 3H).

^{13}C NMR (126 MHz, Chloroform-*d*) δ 161.62 (d, J = 244.0 Hz), 155.19, 134.00 (d, J = 3.2 Hz), 130.83 (d, J = 7.5 Hz), 115.08 (d, J = 21.0 Hz), 79.19, 47.52, 42.25, 28.40, 20.13.

^{19}F NMR (376 MHz, Chloroform-d) δ 117.10.

HRMS (ESI) calcd for $C_{14}H_{20}FNO_2Na^+$ [$(M+Na)^+$] 276.1370, found 276.1369.

HPLC analysis: The ee was determined to be 80% on a CHIRALPAK AD-H column (5% *i*PrOH in hexane, 1.0 mL/min, 25 °C); retention times for compound obtained using (*R*)-**L2**: 5.8 min (major), 6.3 min (minor).

$[\alpha]_D$ (20.0 °C, c = 1.0 in $CHCl_3$) = -3.21°; 80% ee, from (*R*)-**L2**.

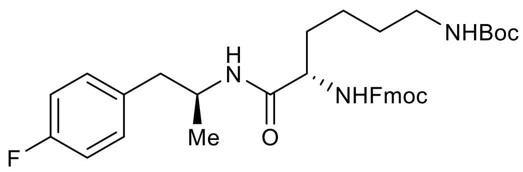

(9*H*-fluoren-9-yl)methyl *tert*-butyl ((*S*)-6-(((*S*)-1-(4-fluorophenyl)propan-2-yl)amino)-6-oxohexane-1,5-diyl)dicarbamate (**115**)

According to the reported literature[20], **115** was obtained from **114** as white solid (112.3 mg, 93%, 90:10 dr).

^{1}H NMR (500 MHz, Chloroform-*d*) δ 7.77 (d, J = 7.5 Hz, 2H), 7.59 (d, J = 7.5 Hz, 2H), 7.40 (t, J = 7.5 Hz, 2H), 7.31 (t, J = 7.4 Hz, 2H), 7.17 – 7.04 (m, 2H), 7.00 – 6.87 (m, 2H), 6.08 (s, 1H), 5.57 – 5.36 (m, 1H), 4.61 (s, 1H), 4.40 (dq, J = 15.2, 7.1 Hz, 2H), 4.19 (dt, J = 12.5, 6.7 Hz, 2H), 4.04 (s, 1H), 3.09 (s, 2H), 2.77 (dd, J = 13.8, 6.1 Hz, 1H), 2.65 (dd, J = 13.6, 7.1 Hz, 1H), 1.88 – 1.75 (m, 2H), 1.66 – 1.56 (m, 1H), 1.51 – 1.40 (m, 10H), 1.36 – 1.28 (m, 2H), 1.10 (d, J = 6.9 Hz, 3H).

^{13}C NMR (126 MHz, Chloroform-*d*) δ 170.87, 161.65 (d, J = 244.5 Hz), 156.32, 156.24, 143.76, 141.34, 133.59 (d, J = 3.0 Hz), 130.73 (d, J = 7.8 Hz), 127.79, 127.12, 125.02, 120.03, 115.17 (d, J = 21.0 Hz), 79.29, 67.06, 54.88, 47.16, 46.42, 41.66, 39.84, 31.93, 29.60, 28.44, 22.40, 19.92.

^{19}F NMR (471 MHz, Chloroform-*d*) δ 113.81 – 124.03 (m).

HRMS (ESI) calcd for $C_{35}H_{43}FN_3O_5^+$ [(M+H)$^+$] 604.3181, found 604.3182.

HPLC analysis: The dr was determined to be 90:10 on a CHIRALPAK AD-H column (15% *i*PrOH in hexane, 1.5 mL/min, 40 °C); retention times for compound obtained using (*R*)-**L2**: 6.7 min (minor), 8.0 min (major).

$[\alpha]_D$ (20.0 °C, c = 1.0 in $CHCl_3$) = -7.87°; 90:10 dr, from (*R*)-**L2**.

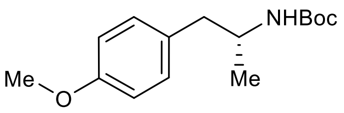

tert-butyl (*R*)-(1-(4-methoxyphenyl)propan-2-yl)carbamate (**116**)

Following **GP-E2**, Ph_2SiH_2 was used instead of DEMS, **116** was obtained as white solid (45.1 mg, 85%, 86% ee).

^{1}H NMR (500 MHz, Chloroform-*d*) δ 7.09 (d, *J* = 8.5 Hz, 2H), 6.83 (d, *J* = 8.6 Hz, 2H), 4.39 (s, 1H), 3.85 (s, 1H), 3.78 (s, 3H), 2.87 – 2.46 (m, 2H), 1.42 (s, 9H), 1.06 (d, *J* = 6.6 Hz, 3H).

^{13}C NMR (101 MHz, Chloroform-*d*) δ 158.15, 155.24, 130.45, 130.31, 113.73, 79.08, 55.24, 47.56, 42.07, 28.44, 20.11.

HRMS (ESI) calcd for $C_{15}H_{23}NO_3Na^+$ [(M+Na)$^+$] 288.1570, found 288.1575.

HPLC analysis: The ee was determined to be 86% on a CHIRALCEL OJ-H column (10% *i*PrOH in hexane, 1.0 mL/min, 25 °C); retention times for compound obtained using (*S*)-**L2**: 4.6 min (minor), 5.2 min (major).

$[\alpha]_D$ (20.0 °C, c = 1.0 in $CHCl_3$) = +6.26°; 86% ee, from (*S*)-**L2**.

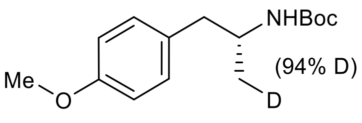

tert-butyl (*R*)-(1-(4-methoxyphenyl)propan-2-yl-3-d)carbamate (**117**)

Following **GP-E2**, Ph_2SiD_2 was used instead of DEMS, **117** was obtained as white solid (43.7 mg, 82%, 84% ee).

^{1}H NMR (500 MHz, Chloroform-*d*) (500 MHz, Chloroform-*d*) δ 7.08 (d, *J* = 8.5 Hz, 2H), 6.82 (d, *J* = 8.6 Hz, 2H), 4.39 (s, 1H), 3.84 (s, 1H), 3.78 (s, 3H), 2.76 (dd, *J* = 13.6,

5.6 Hz, 1H), 2.59 (dd, J = 13.5, 7.3 Hz, 1H), 1.42 (s, 9H), 1.05 (d, J = 6.8 Hz, 2H).

^{13}C NMR (126 MHz, Chloroform-d) δ 158.18, 155.27, 130.43, 113.76, 79.06, 55.23, 47.54, 42.08, 28.43, 20.33 – 19.37 (m).

HRMS (ESI) calcd for $C_{15}H_{22}DNO_3Na^+$ [(M+Na)$^+$] 289.1633, found 289.1632.

HPLC analysis: The ee was determined to be 84% on a CHIRALCEL OJ-H column (10% *i*PrOH in hexane, 1.0 mL/min, 25 °C); retention times for compound obtained using (*S*)-**L2**: 4.6 min (minor), 5.2 min (major).

$[\alpha]_D$ (20.0 °C, c = 1.0 in $CHCl_3$) = +9.10°; 84% ee, from (*S*)-**L2**.

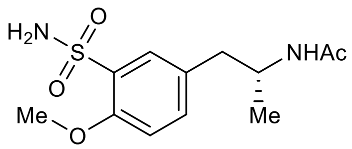

(*R*)-*N*-(1-(4-methoxy-3-sulfamoylphenyl)propan-2-yl)acetamide (**118**)

According to the reported literature[21], **118** was obtained from **116** as yellow solid (34.4 mg, 60%).

^{1}H NMR (500 MHz, DMSO-d_6) δ 7.79 (d, J = 8.0 Hz, 1H), 7.56 (d, J = 2.3 Hz, 1H), 7.37 (dd, J = 8.4, 2.3 Hz, 1H), 7.11 (d, J = 8.4 Hz, 1H), 7.01 (s, 2H), 3.93 – 3.83 (m, 4H), 2.68 (dd, J = 13.4, 7.1 Hz, 1H), 2.59 (dd, J = 13.5, 6.7 Hz, 1H), 1.75 (s, 3H), 1.00 (d, J = 6.6 Hz, 3H).

^{13}C NMR (101 MHz, DMSO-d_6) δ 168.38, 154.40, 134.22, 130.77, 130.67, 128.05, 112.41, 56.02, 46.04, 40.74, 22.77, 20.04.

HRMS (ESI) calcd for $C_{12}H_{19}N_2O_4S^+$ [(M+H)$^+$] 287.1060, found 287.1060.

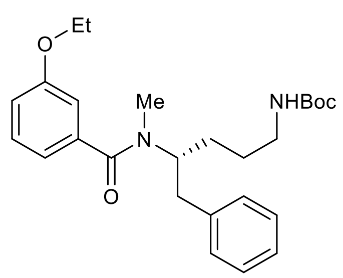

tert-butyl (*R*)-(4-(3-ethoxy-N-methylbenzamido)-5-phenylpentyl)carbamate (**121**)

Following **GP-D1**, **121** was obtained as pale-green oil (35.2 mg, 40% yield, 90% ee).

^{1}H NMR (500 MHz, Chloroform-*d*) δ 7.38 – 7.20 (m, 4H), 7.20 – 7.09 (m, 1H), 7.00 – 6.92 (m, 1H), 6.87 – 6.79 (m, 1H), 6.59 – 6.45 (m, 1H), 6.38 – 6.28 (m, 1H), 5.05 (s, 0.48H), 4.67 (s, 0.37H), 4.50 (s, 0.51H), 4.06 – 3.85 (m, 2H), 3.79 (tt, *J* = 9.7, 4.6 Hz, 0.50H), 3.24 – 2.90 (m, 4H), 2.88 – 2.78 (m, 1H), 2.74 – 2.51 (m, 2H), 1.92 – 1.53 (m, 4H), 1.43 (d, *J* = 6.1 Hz, 9H), 1.37 (t, *J* = 7.0 Hz, 3H).

^{13}C NMR (101 MHz, Chloroform-*d*) δ 172.68, 172.21, 158.87, 158.85, 156.17, 156.03, 138.48, 138.24, 137.96, 137.90, 129.59, 129.45, 129.36, 129.09, 128.77, 128.54, 126.85, 126.57, 118.52, 115.92, 115.47, 112.58, 112.10, 79.41, 79.25, 63.63, 63.60, 60.20, 40.55, 40.39, 39.74, 38.94, 29.94, 28.92, 28.54, 28.51, 27.19, 27.09, 26.78, 14.90, 14.88.

HRMS (ESI) calcd for $C_{26}H_{37}N_3O_4^+$ [$(M+H)^+$] 441.2748, found 441.2742.

HPLC analysis: The ee was determined to be 90% on a CHIRALPAK AD-H column (10% *i*PrOH in hexane, 1.0 mL/min, 40 °C); retention times for compound obtained using (*S,S*)-**L1**: 12.9 min (minor), 16.7 min (major).

$[\alpha]_D$ (20.0 ℃, c = 1.0 in $CHCl_3$) = -22.31 °, 92% ee, from (*S,S*)-**L1**.

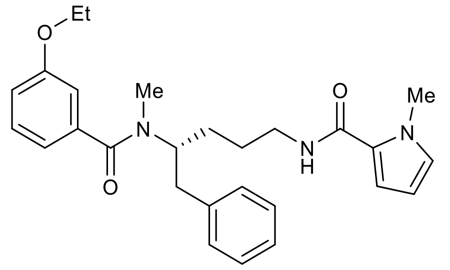

(*R*)-*N*-(4-(3-ethoxy-*N*-methylbenzamido)-5-phenylpentyl)-1-methyl-1*H*-pyrrole-2-carboxamide (**122**)

According to the reported literature[20], **122** was obtained from **121** as colorless oil (78.6 mg, 80% yield)

^{1}H NMR (500 MHz, Chloroform-*d*) δ 7.33 – 7.20 (m, 4H), 7.19 – 7.07 (m, 1H), 7.01 – 6.77 (m, 2H), 6.75 – 6.60 (m, 2H), 6.58 – 6.44 (m, 2H), 6.40 – 6.25 (m, 1H), 6.12 – 5.99 (m, 1H), 5.11 (s, 0.46H), 4.02 – 3.84 (m, 5H), 3.81 (m, 0.52H), 3.51 – 3.39 (m, 1H), 3.40 – 3.19 (m, 1H), 3.01 (s, 1.26H), 2.94 (dd, J = 14.3, 5.8 Hz, 0.61H), 2.88 – 2.74 (m, 1H), 2.71 – 2.55 (m, 2H), 1.85 – 1.58 (m, 2H), 1.56 – 1.39 (m, 2H), 1.35 (dt,

J = 12.0, 6.9 Hz, 3H).

^{13}C NMR (101 MHz, Chloroform-*d*) δ 172.68, 172.45, 162.25, 162.06, 158.84, 158.80, 138.30, 138.17, 129.58, 129.41, 129.32, 128.97, 128.73, 128.52, 127.96, 127.80, 126.81, 126.55, 125.88, 125.71, 118.53, 118.47, 115.99, 115.47, 112.52, 112.06, 111.81, 111.47, 107.22, 107.14, 63.59, 63.56, 60.19, 39.63, 39.10, 38.87, 38.85, 36.84, 36.78, 30.04, 29.78, 29.21, 26.96, 26.77, 26.22, 14.83.

HRMS (ESI) calcd for $C_{27}H_{34}N_3O_3^+$ [(M+H)$^+$] 448.2600, found 448.2600.

$[\alpha]_D$ (20.0 ℃, c = 1.0 in $CHCl_3$) = -14.59 °, 92% ee, from (*S*,*S*)-**L1**.

Supplementary Note 2

Assignment of Absolute Configuration

For chiral alkylamide, several amides with defined absolute configuration were synthesized for HPLC data comparison with the reaction product from (*S,S*)-**L1** or *S*-**L2**. For chiral β-aminoalcohol, a single-crystal x-ray crystallography data was provided.

Preparation of CP1&15B

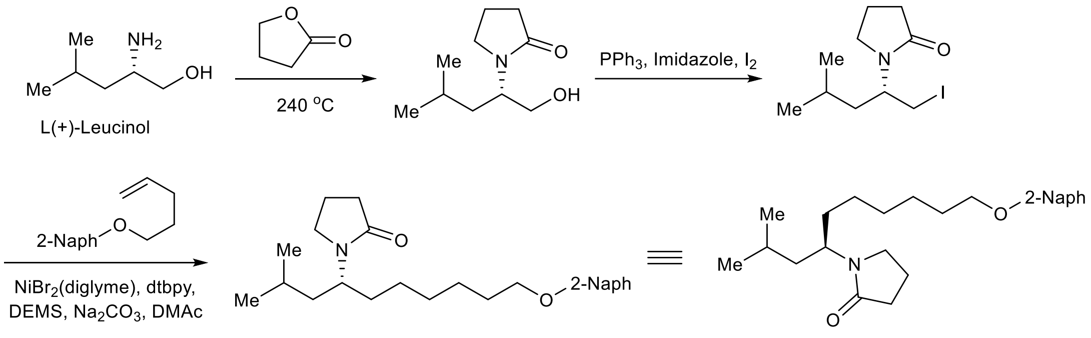

R-absolute configuration substrate **(CP1)**

According to the reported literature[22], **CP1** was synthesized.

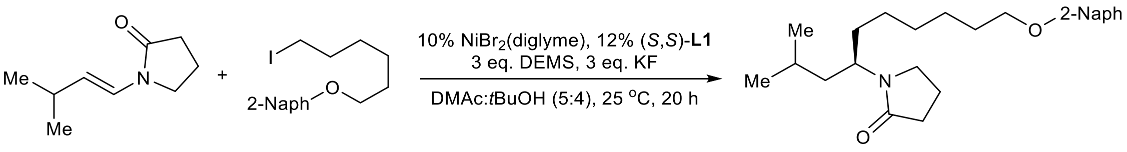

1.0 eq. 2.0 eq. **15B,** 42% yield, 95% ee

According to **GP-D1**, **15B** was synthesized.

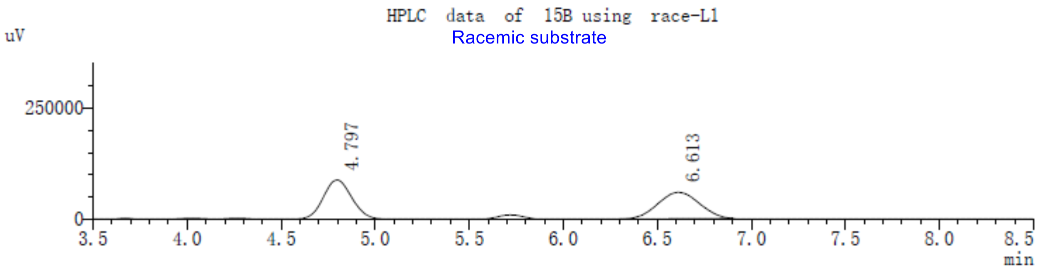

检测器A Ch1 214nm

Peak[#]	RetTime[min]	Height[uV]	Width[min]	Area[uV*s]	Area[%]
1	4.797	87827	0.164	921979	50.065
2	6.613	59560	0.246	919584	49.935

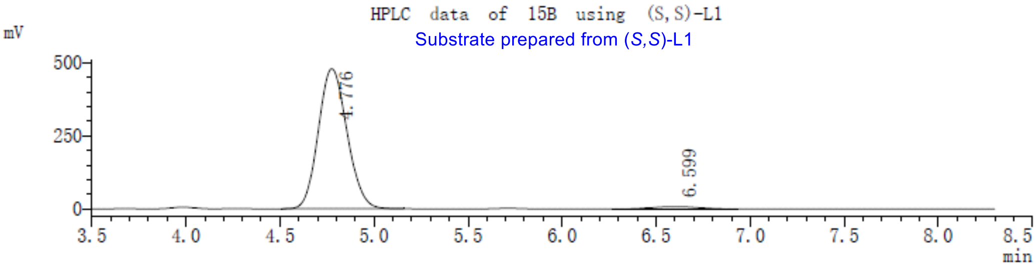

检测器A Ch1 214nm

Peak[#]	RetTime[min]	Height[uV]	Width[min]	Area[uV*s]	Area[%]
1	4.776	478654	0.166	5082052	97.768
2	6.599	7161	0.259	116020	2.232

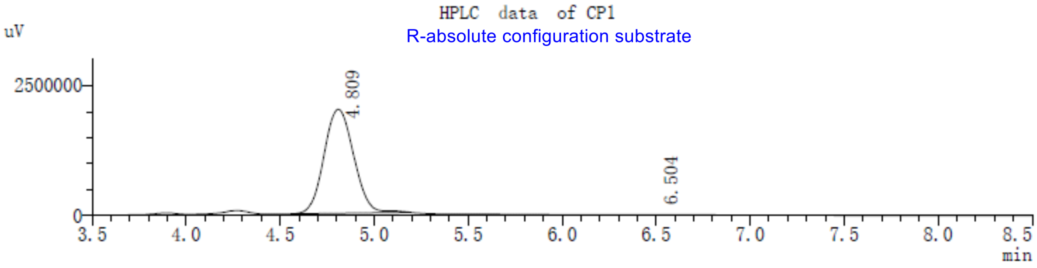

检测器A Ch1 214nm

Peak[#]	RetTime[min]	Height[uV]	Width[min]	Area[uV*s]	Area[%]
1	4.809	2005595	0.171	22067440	99.611
2	6.504	6954	--	86079	0.389

Supplementary Figure 4. HPLC spectra compared between **15B** and **CP1**

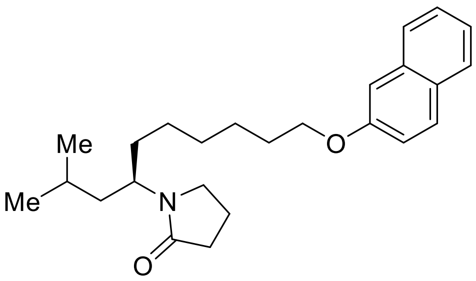

(*R*)-1-(2-methyl-10-(naphthalen-2-yloxy)decan-4-yl)pyrrolidin-2-one (**CP1**)

^{1}**H NMR** (500 MHz, Chloroform-*d*) δ 7.80 – 7.67 (m, 3H), 7.47 – 7.39 (m, 1H), 7.32 (t, J = 7.5 Hz, 1H), 7.18 – 7.10 (m, 2H), 4.29 – 4.13 (m, 1H), 4.06 (t, J = 6.4 Hz, 2H),

3.20 (t, J = 6.9 Hz, 2H), 2.40 (t, J = 7.9 Hz, 2H), 2.05 – 1.88 (m, 3H), 1.88 – 1.77 (m, 2H), 1.56 – 1.37 (m, 6H), 1.36 – 1.08 (m, 4H), 0.92 (d, J = 6.1 Hz, 3H), 0.90 (d, J = 6.2 Hz, 3H).

^{13}C NMR (126 MHz, Chloroform-*d*) δ 175.23, 157.19, 134.73, 129.41, 128.99, 127.73, 126.81, 126.40, 123.57, 119.13, 106.69, 68.02, 49.08, 41.77, 32.92, 31.73, 29.34, 29.27, 26.34, 26.14, 25.13, 23.57, 22.12, 18.46.

HRMS (ESI) calcd for $C_{25}H_{36}NO_2^+$ [$(M+H)^+$] 382.2276, found 382.2751.

HPLC analysis: The ee was determined to be 97% on a CHIRALCEL OD-H column (15% *i*PrOH in hexane, 1.5 mL/min, 40 °C); retention times for compound obtained using (*S,S*)-**L1**: 6.6 min (minor), 4.7 min (major).

$[\alpha]_D$ (20.0 ℃, c = 1.0 in $CHCl_3$) = -5.14 °; 95% ee, from (*S,S*)-**L1**.

Preparation of CP2&13B

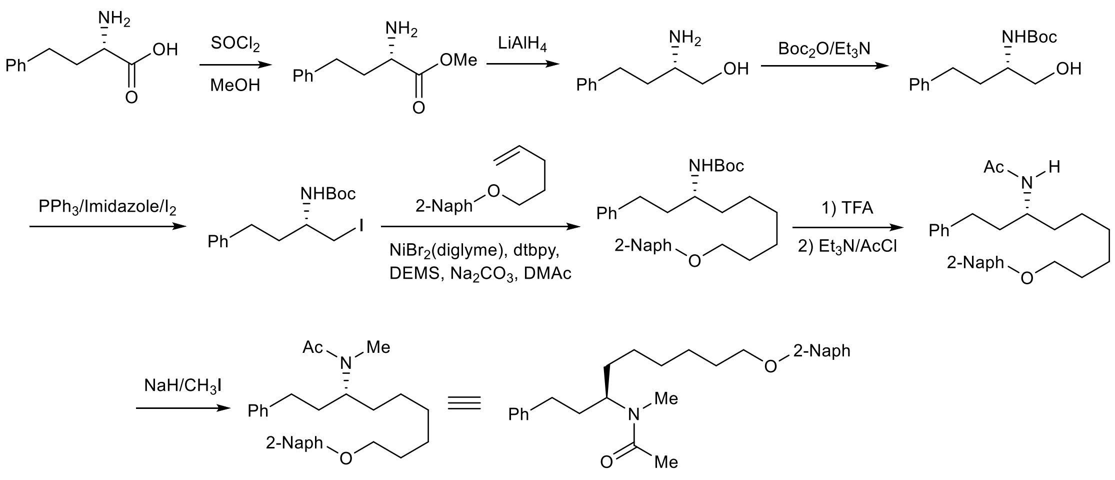

R-absolute configuration substrate **(CP2)**

According to the reported literature[22], **CP2** was synthesized.

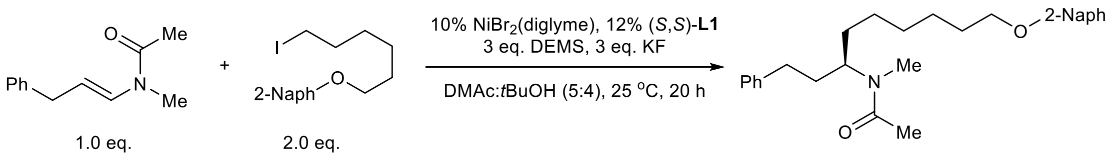

13B, 86% yield, 92%ee

According to **GP-D1**, **13B** was synthesized.

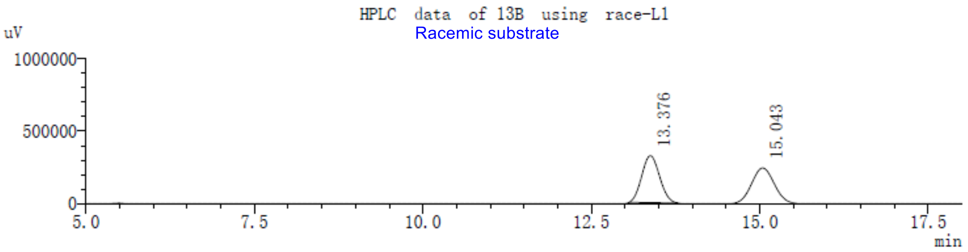

检测器A Ch1 214nm

Peak[#]	RetTime[min]	Height[uV]	Width[min]	Area[uV*s]	Area[%]
1	13.376	324219	0.292	5964381	50.507
2	15.043	247156	0.369	5844703	49.493

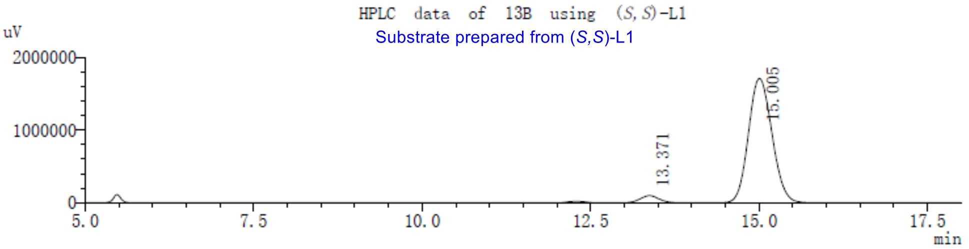

检测器A Ch1 214nm

Peak[#]	RetTime[min]	Height[uV]	Width[min]	Area[uV*s]	Area[%]
1	13.371	95218	0.292	1762677	4.073
2	15.005	1713259	0.380	41514944	95.927

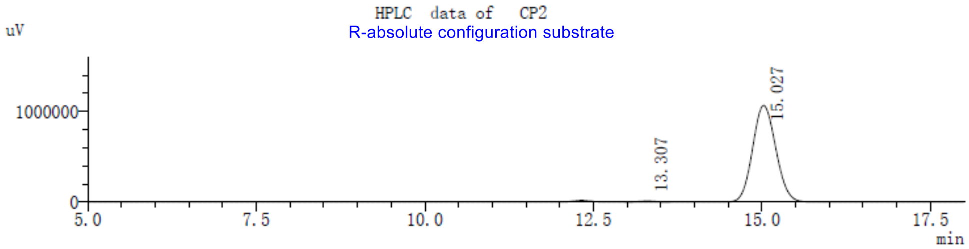

检测器A Ch1 214nm

Peak[#]	RetTime[min]	Height[uV]	Width[min]	Area[uV*s]	Area[%]
1	13.307	8943	0.317	173337	0.677
2	15.027	1063648	0.373	25416467	99.323

Supplementary Figure 5. HPLC spectra compared between **13B** and **CP2**

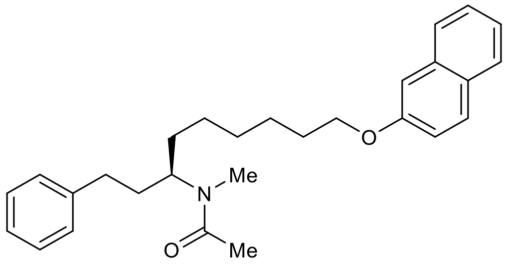

(*R*)-*N*-methyl-*N*-(9-(naphthalen-2-yloxy)-1-phenylnonan-3-yl)acetamide **(CP2)**

^{1}H NMR (500 MHz, Chloroform-*d*) δ 7.78 – 7.67 (m, 3H), 7.46 – 7.38 (m, 1H), 7.36 – 7.23 (m, 3H), 7.21 – 7.08 (m, 5H), 4.86 – 4.66 (m, 0.5H), 4.05 (td, J = 6.5, 3.7 Hz, 2H),

3.72 – 3.49 (m, 0.5H), 2.75 (s, 1.5H), 2.70 (s, 1.5H), 2.64 – 2.33 (m, 2H), 2.07 (s, 1.5H), 1.92 (s, 1.5H), 1.86 – 1.66 (m, 4H), 1.55 – 1.17 (m, 8H).

^{13}C NMR (101 MHz, Chloroform-*d*) δ 171.45, 171.23, 157.10, 157.04, 142.10, 141.01, 134.65, 134.63, 129.39, 129.34, 128.92, 128.89, 128.63, 128.40, 128.30, 128.26, 127.68, 127.66, 126.74, 126.73, 126.37, 126.33, 126.23, 125.85, 123.55, 123.50, 119.06, 119.01, 106.57, 106.55, 67.91, 67.77, 57.25, 52.33, 34.51, 34.33, 33.04, 32.93, 29.37, 29.28, 29.19, 29.13, 26.28, 26.17, 26.05, 25.90, 22.46, 21.82.

HRMS (ESI) calcd for $C_{21}H_{25}NO_2Na^+$ [$(M+Na)^+$] 346.1783, found 346.1784.

HPLC analysis: The ee was determined to be 92% on a CHIRALPAK AD-H column (10% *i*PrOH in hexane, 1.0 mL/min, 40 °C); retention times for compound obtained using (*S,S*)-**L1**: 13.3 min (minor), 15.0 min (major).

$[\alpha]_D$ (20.0 ℃, c = 1.0 in $CHCl_3$) = 9.02 °; 92% ee, from (*S,S*)-**L1**.

Preparation of CP3&46-b

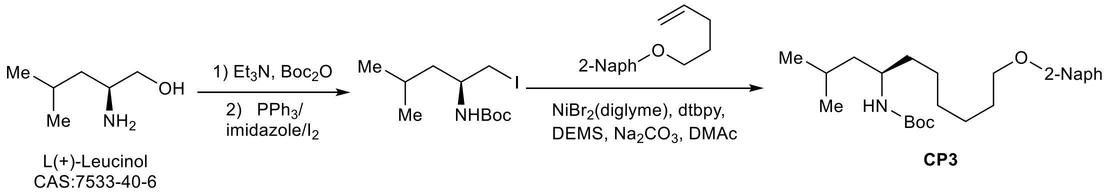

According to the reported literature[22], **CP3** was synthesized.

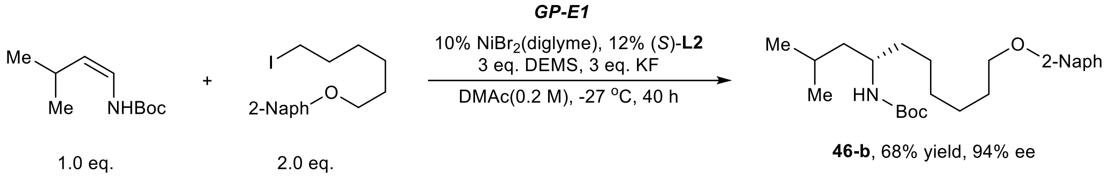

According to **GP-E1**, **46-b** was synthesized.

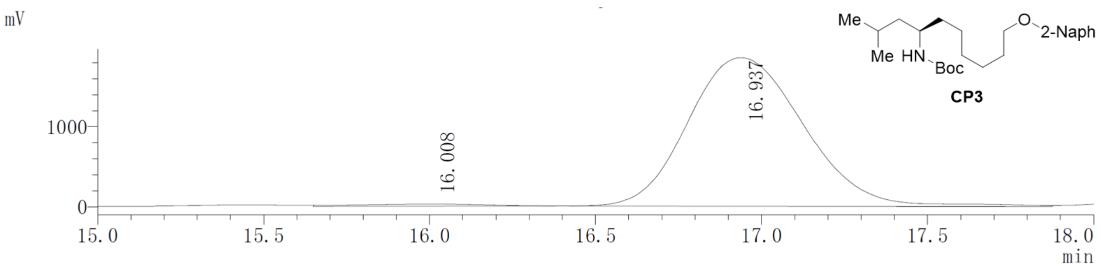

检测器A Ch1 214nm

Peak[#]	RetTime[min]	Height[uV]	Width[min]	Area[uV*s]	Area[%]
1	16.008	25663	0.478	691920	1.508
2	16.937	1857946	0.377	45183126	98.492

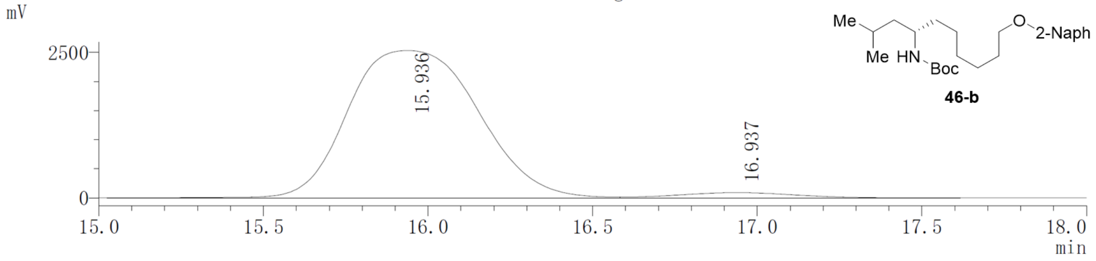

检测器A Ch1 214nm

Peak[#]	RetTime[min]	Height[uV]	Width[min]	Area[uV*s]	Area[%]
1	15.936	2534197	0.438	69513800	96.866
2	16.937	90841	0.387	2248935	3.134

Supplementary Figure 6. HPLC spectra compared between **CP3** and **46-b**

Preparation of CP4&51

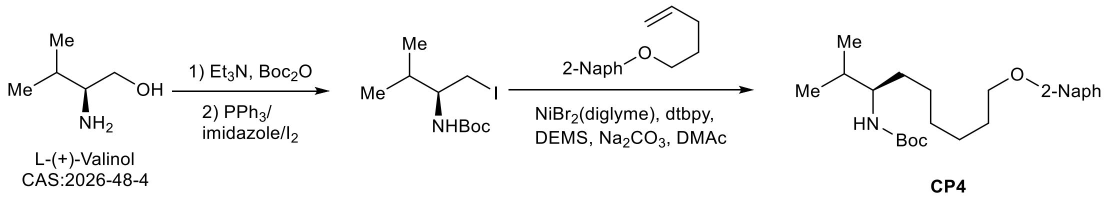

According to the reported literature[22], **CP4** was synthesized.

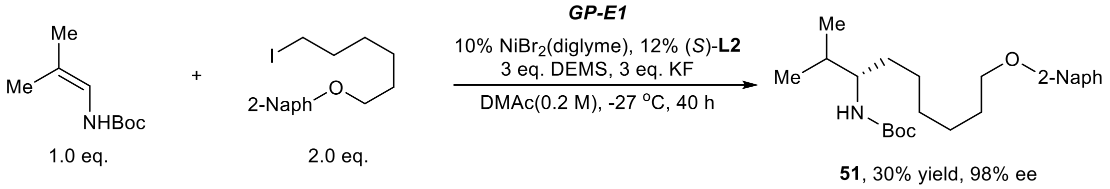

According to **GP-E1**, **51** was synthesized.

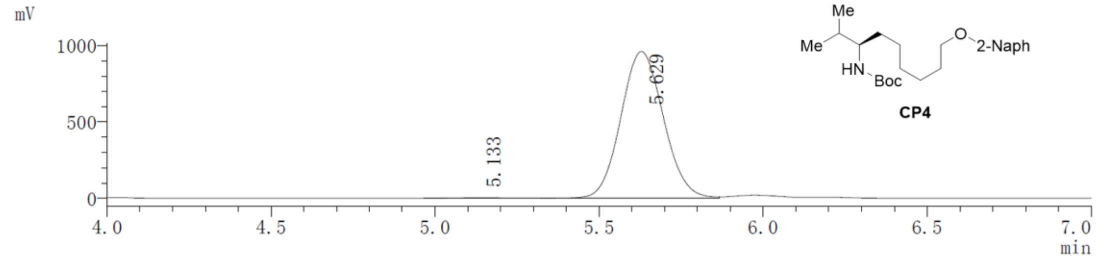

检测器A Ch1 214nm

Peak[#]	RetTime[min]	Height[uV]	Width[min]	Area[uV*s]	Area[%]
1	5.133	3654	0.173	41292	0.473
2	5.629	959766	0.140	8684894	99.527

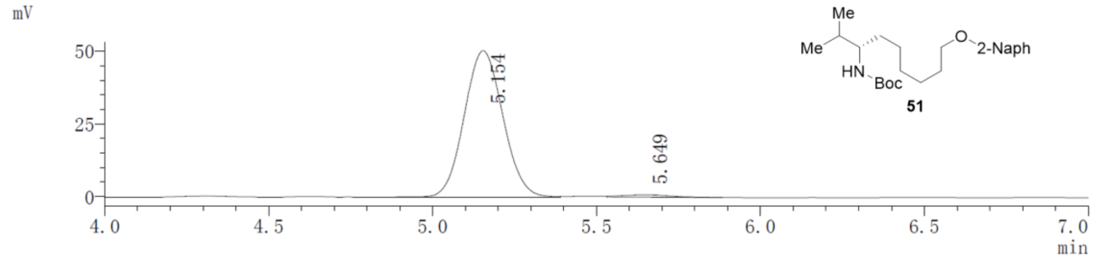

检测器A Ch1 214nm

Peak[#]	RetTime[min]	Height[uV]	Width[min]	Area[uV*s]	Area[%]
1	5.154	50461	0.128	417879	98.250
2	5.649	825	0.145	7444	1.750

Supplementary Figure 7. HPLC spectra compared between **CP4** and **51**

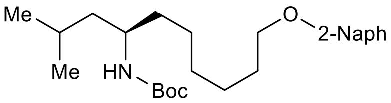

tert-butyl (*R*)-(2-methyl-10-(naphthalen-2-yloxy)decan-4-yl)carbamate (**CP3**)

^{1}H NMR (500 MHz, Chloroform-*d*) δ 7.89 – 7.68 (m, 3H), 7.43 (t, *J* = 7.6 Hz, 1H), 7.38 – 7.30 (m, 1H), 7.23 – 7.11 (m, 2H), 4.20 (d, *J* = 9.2 Hz, 1H), 4.14 – 4.01 (m, 2H), 3.72 – 3.52 (m, 1H), 1.93 – 1.81 (m, 2H), 1.71 – 1.63 (m, 1H), 1.60 – 1.31 (m, 17H), 1.29 – 1.22 (m, 2H), 1.08 – 0.85 (m, 6H).

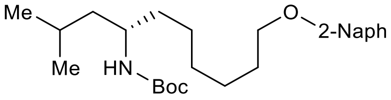

tert-butyl (*S*)-(2-methyl-10-(naphthalen-2-yloxy)decan-4-yl)carbamate (**46-b**)

Following **GP-E2**, **46-b** was obtained as white solid (56.2 mg, 68%, 94% ee).

^{1}H NMR (500 MHz, Chloroform-*d*) δ 7.78 – 7.69 (m, 3H), 7.46 – 7.40 (m, 1H), 7.35

– 7.29 (m, 1H), 7.17 – 7.11 (m, 2H), 4.19 (d, *J* = 9.4 Hz, 1H), 4.07 (t, *J* = 6.5 Hz, 2H), 3.70 – 3.51 (m, 1H), 1.90 – 1.80 (m, 2H), 1.70 – 1.63 (m, 1H), 1.55 – 1.31 (m, 17H), 1.27 – 1.21 (m, 2H), 0.91 (t, *J* = 6.0 Hz, 6H).

^{13}C NMR (126 MHz, Chloroform-*d*) δ 157.09, 155.65, 134.62, 129.29, 128.88, 127.62, 126.69, 126.27, 123.44, 119.02, 106.56, 78.78, 67.94, 48.74, 45.07, 36.19, 29.35, 29.19, 28.44, 26.09, 25.68, 24.93, 23.18, 22.31.

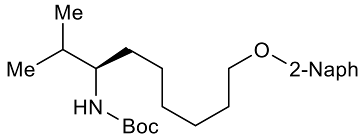

tert-butyl (*R*)-(2-methyl-9-(naphthalen-2-yloxy)nonan-3-yl)carbamate (**CP4**)

^{1}H NMR (500 MHz, Chloroform-*d*) δ 7.81 – 7.66 (m, 3H), 7.46 – 7.40 (m, 1H), 7.35 – 7.30 (m, 1H), 7.20 – 7.11 (m, 2H), 4.27 (d, 1H), 4.14 – 4.04 (m, 2H), 3.51 – 3.30 (m, 1H), 1.91 – 1.81 (m, 2H), 1.73 – 1.68 (m, 1H), 1.54 – 1.43 (m, 13H), 1.39 – 1.24 (m, 4H), 0.95 – 0.84 (m, 6H).

X-ray analysis data

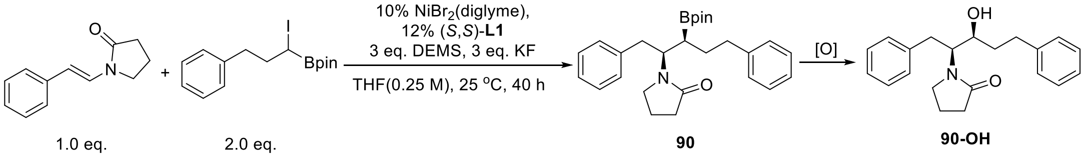

1-((2*S*,3*S*)-3-hydroxy-1,5-diphenylpentan-2-yl)pyrrolidin-2-one. 1-((2*S*,3*S*)-1,5-diphenyl-3-(4,4,5,5-tetramethyl-1,3,2-dioxaborolan-2-yl)pentan-2-yl)pyrrolidin-2-one (**90**) was prepared according to **GP-F1**, using (*S,S*)–**L1**. The boronate ester was stereospecifically oxidized as described in **GP-F3**, and the β-aminoalcohol was crystallized from ethyl acetate at 20 °C.

The absolute configuration of **90-OH** was unambiguously determined to be *S* at the oxygen-bound stereocenters and *S* at the nitrogen-bound stereocenters, as established by single-crystal x-ray crystallography. The absolute configurations of all other β-amino alcohols and β-aminoboronates were assigned by analogy.

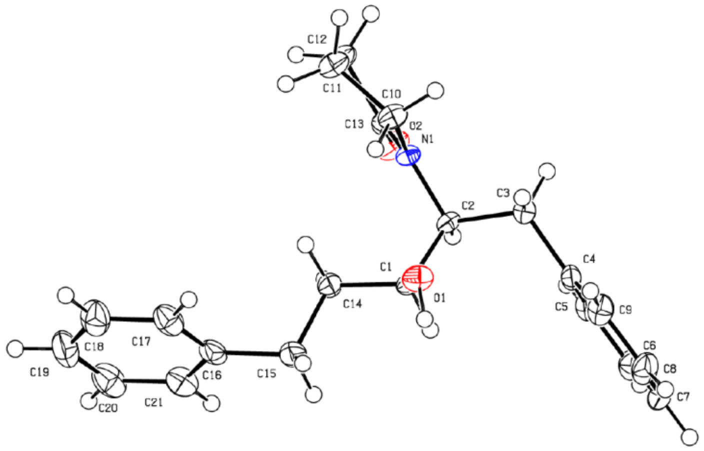

Supplementary Figure 8. ORTEP drawing of compound **90-OH** (CCDC number:2032484)

Supplementary Table 3. Crystal data and structure refinement for 200903wjw_0m.

Identification code	200903wjw_0m	
Empirical formula	C21 H25 N O2	
Formula weight	323.42	
Temperature	173.01 K	
Wavelength	1.34139 Å	
Crystal system	Orthorhombic	
Space group	$P2_12_12_1$	
Unit cell dimensions	a = 6.1839(3) Å	α= 90 °.
	b = 14.2345(7) Å	β= 90 °.
	c = 19.7754(10) Å	γ = 90 °.
Volume	1740.72(15) Å^3	
Z	4	
Density (calculated)	1.234 Mg/m^3	
Absorption coefficient	0.397 mm^{-1}	
F(000)	696	
Crystal size	0.08 x 0.06 x 0.05 mm^3	

Theta range for data collection	3.328 to 54.960 °.
Index ranges	-7<=h<=7, -17<=k<=17, -24<=l<=18
Reflections collected	18730
Independent reflections	3291 [R(int) = 0.0677]
Completeness to theta = 53.594 °	99.5 %
Absorption correction	Semi-empirical from equivalents
Max. and min. transmission	0.7508 and 0.5456
Refinement method	Full-matrix least-squares on F^2
Data / restraints / parameters	3291 / 0 / 218
Goodness-of-fit on F^2	1.075
Final R indices [I>2sigma(I)]	R1 = 0.0344, wR2 = 0.0771
R indices (all data)	R1 = 0.0370, wR2 = 0.0785
Absolute structure parameter	0.04(17)
Extinction coefficient	n/a
Largest diff. peak and hole	0.110 and -0.159 e.Å^{-3}

Supplementary Note 3

Mechanistic Studies

General Procedure G1 (GP-G1): Radical Experiments of Secondary Enamides/Enecarbamates (Fig. 4a). The procedure is the same as **GP-E2**, except for reaction temperature: the reaction mixture was stirred at 25 °C for 20 h.

General Procedure G2 (GP-G2): Deuterium-labelling Experiments of Tertiary Enamides (Fig. 4b). The procedure is the same as **GP-D1**, except for silane and solvent: Ph_2SiD_2 (0.6 mmol, 3.0 equiv.) (only used for the deuterium-labeling study) and d_7-DMF:d_{10}-tBuOD (5:4).

General Procedure G3 (GP-G3): Deuterium-labelling Experiments of Secondary Enecarbamates (Fig. 4b). The procedure is the same as **GP-E2**, except for reaction temperature and silane: the reaction mixture was stirred at 25 °C for 20 h, and Ph_2SiD_2 (0.6 mmol, 3.0 equiv.) (only used for the deuterium-labeling study).

Radical Experiments:

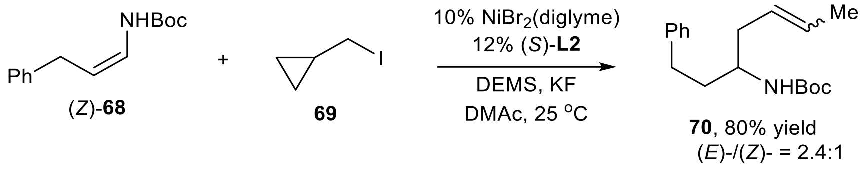

As shown above, a "radical clock" alkyl halide **69** was prepared to test the reaction, the ring-opened product **70** was obtained in 80% isolated yield, it was revealed that the activation of alkyl halides proceeded through a radical pathway.

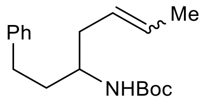

tert-butyl (1-phenylhept-5-en-3-yl)carbamate (**70**)

70 was prepared according to **GP-G1** from *tert*-butyl (*Z*)-(3-phenylprop-1-en-1-

yl)carbamate, 2.4: 1 *E:Z.* (46.3 mg, 80% isolated yield)

^{1}H NMR (400 MHz, Chloroform-*d*) δ 7.32 – 7.26 (m, 2H), 7.24 – 7.14 (m, 3H), 5.75 – 5.28 (m, 2H), 4.55 – 4.28 (m, 1H), 3.82 – 3.38 (m, 1H), 2.79 – 2.55 (m, 2H), 2.23 – 2.12 (m, 1H), 1.85 – 1.73 (m, 1H), 1.68 – 1.64 (m, 2.12H), 1.63 – 1.61 (m, 0.88H), 1.46 (s, 9H).

^{13}C NMR (101 MHz, Chloroform-*d*) δ 155.61, 142.06, 133.15, 128.60, 128.37, 128.33, 126.82, 126.56, 126.30, 125.82, 125.80, 125.67, 79.00, 50.57, 50.22, 45.28, 38.39, 37.60, 36.66, 36.55, 32.48, 29.70, 28.43, 28.23, 28.00, 25.29, 22.64, 18.06, 14.06, 13.01.

HRMS (ESI) calcd for $C_{18}H_{27}NO_2Na^+$ [$(M+Na)^+$] 312.1934, found 312.1935.

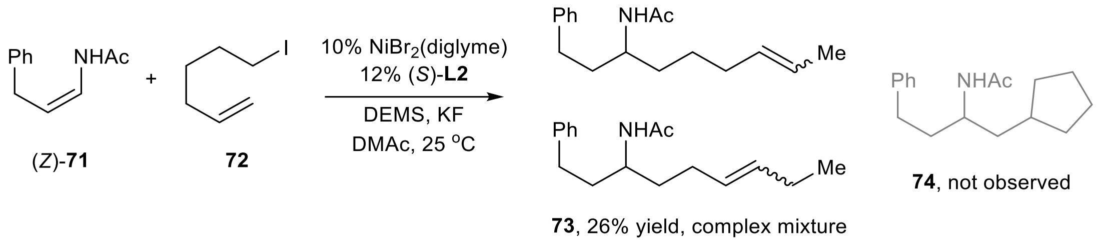

According to **GP-G1**, tested the reductive hydroalkylation of 5-iodopent-1-ene **72**. Analyzed the results by GC and NMR, the assumed ring-cyclized product **74** was not observed, and a complex mixture of linear coupling products **73** was obtained, which revealed that the migration of vinyl double bonds was a very rapid primitive step.

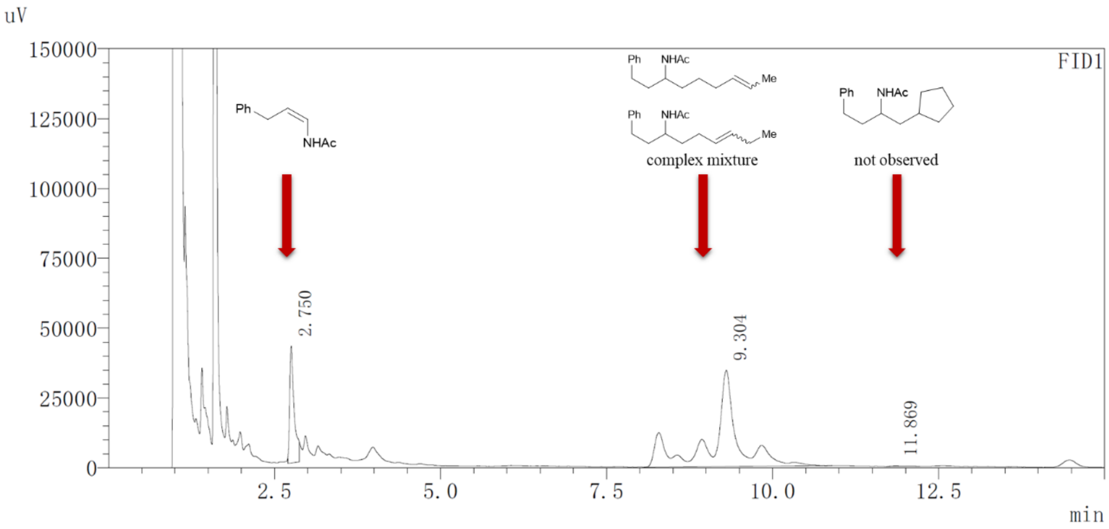

Supplementary Figure 9. GC spectra of the reaction solution shown above

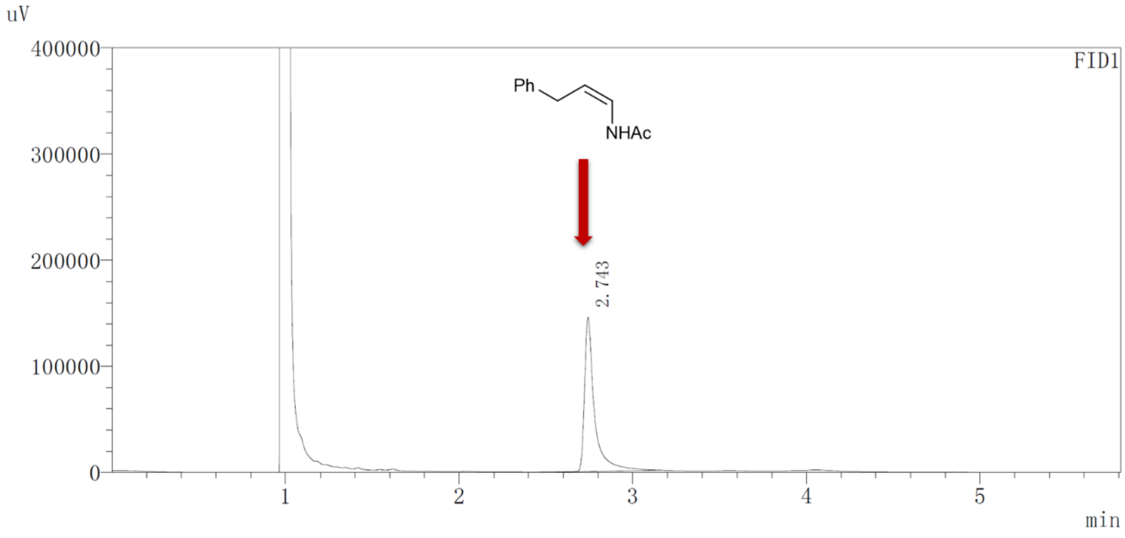

Supplementary Figure 10. GC spectra of ***Z*-71**

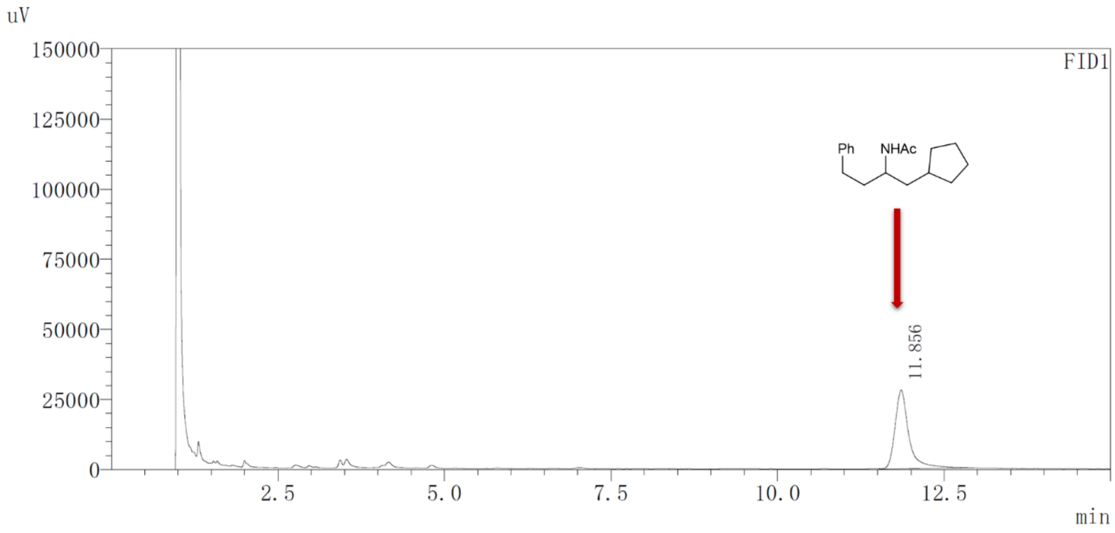

Supplementary Figure 11. GC spectra of **74**

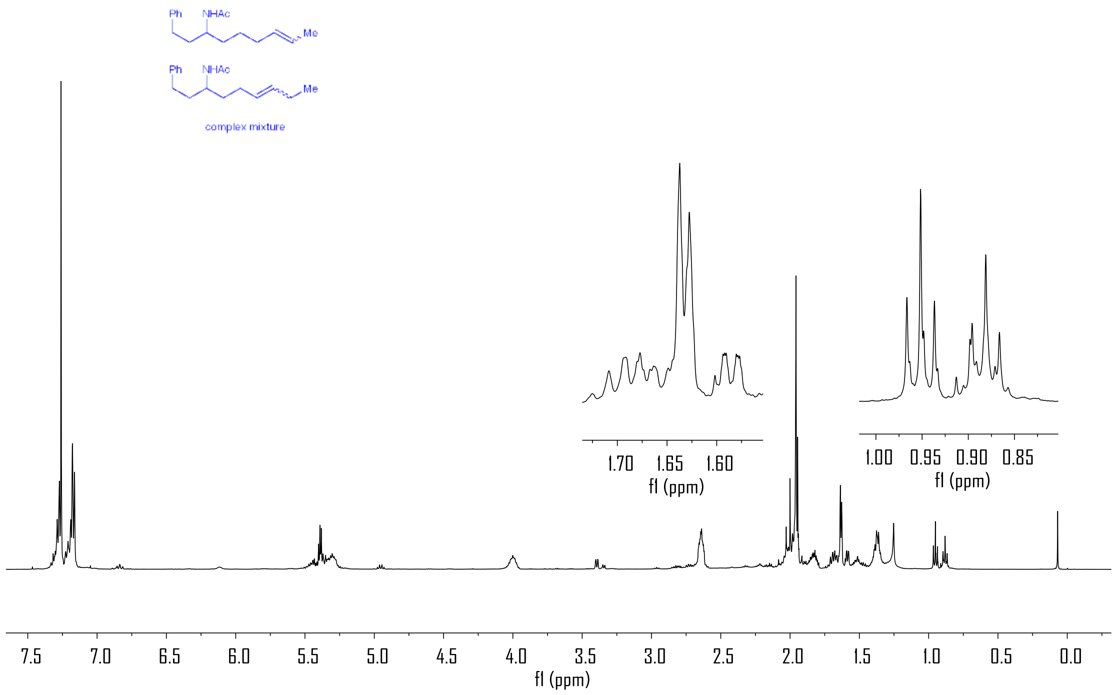

Supplementary Figure 12. ^{1}H NMR spectra for **73**

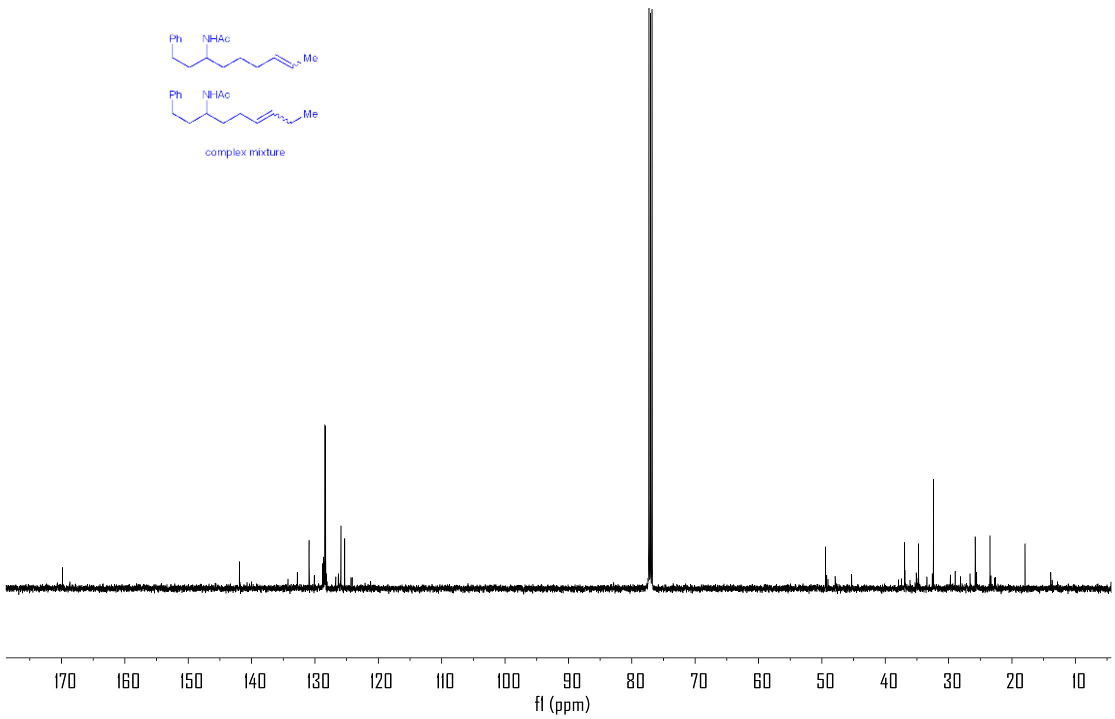

Supplementary Figure 13. ^{13}C NMR spectra for **73**

Deuterium-labelling experiments use (*S*,*S*)-L1 as ligand

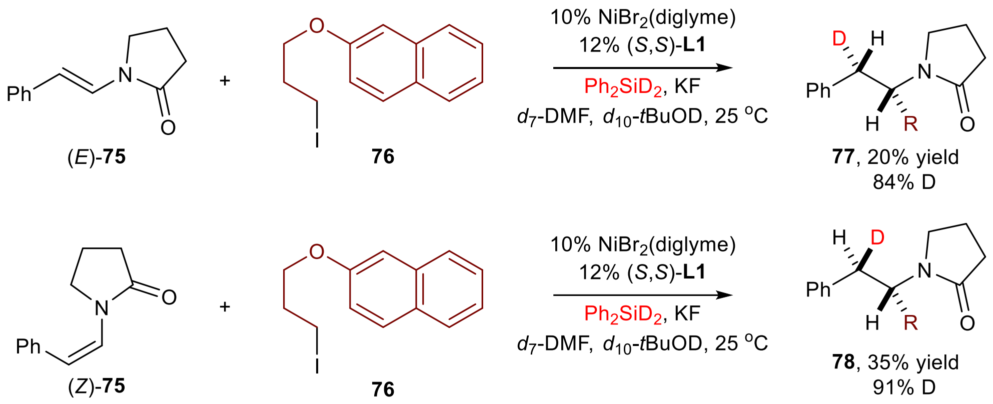

Deuterium-labelling experiments use racemic-L1 as ligand

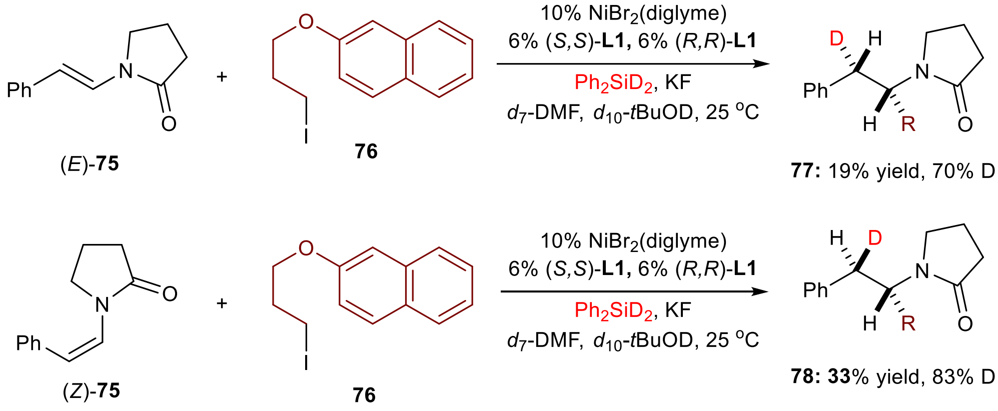

Deuterium-labelling experiments use dttbpy as ligand

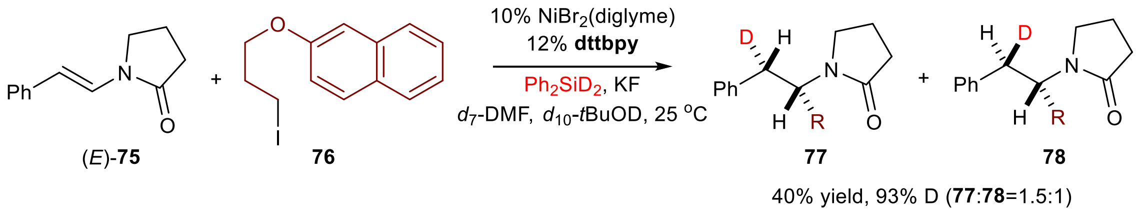

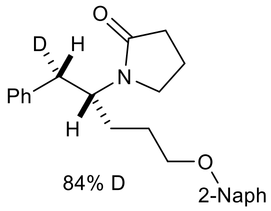

1-((1*S*,2*R*)-5-(naphthalen-2-yloxy)-1-phenylpentan-2-yl-1-d)pyrrolidin-2-one (**77**)

77 was prepared according to **GP-G2** from (*E*)-1-styrylpyrrolidin-2-one. (15.0 mg, 20%

isolated yield)

^{1}H NMR (500 MHz, Chloroform-*d*) δ 7.79 – 7.68 (m, 3H), 7.49 – 7.39 (m, 1H), 7.36 – 7.31 (m, 1H), 7.30 – 7.26 (m, 2H), 7.23 – 7.18 (m, 3H), 7.13 – 7.09 (m, 2H), 4.55 – 4.38 (m, 1H), 4.25 – 3.97 (m, 2H), 3.26 (t, J = 6.9 Hz, 2H), 2.80 (d, J = 8.7 Hz, 1H), 2.27 (dh, J = 32.6, 7.8 Hz, 2H), 1.98 – 1.73 (m, 6H).

^{2}H NMR (61 MHz, Chloroform-*d*) δ 2.86 (s).

HRMS (ESI) calcd for $C_{25}H_{27}DNO_2^+$ [$(M+H)^+$] 375.2182, found 375.2183.

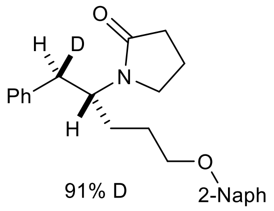

1-((1*R*,2*R*)-5-(naphthalen-2-yloxy)-1-phenylpentan-2-yl-1-d)pyrrolidin-2-one (**78**)

78 was prepared according to **GP-G2** from (*Z*)-1-styrylpyrrolidin-2-one. (26.2 mg, 35% isolated yield)

^{1}H NMR (400 MHz, Chloroform-*d*) δ 7.79 – 7.68 (m, 3H), 7.51 – 7.40 (m, 1H), 7.37 – 7.26 (m, 3H), 7.23 – 7.19 (m, 3H), 7.16 – 7.09 (m, 2H), 4.56 – 4.39 (m, 1H), 4.22 – 3.98 (m, 2H), 3.26 (t, J = 6.9 Hz, 2H), 2.85 (d, 1.09H), 2.40 – 2.13 (m, 2H), 1.94 – 1.73 (m, 6H).

^{13}C NMR (101 MHz, Chloroform-*d*) δ 175.43, 156.88, 138.08, 134.66, 129.44, 129.00, 128.97, 128.53, 127.71, 126.84, 126.56, 126.45, 123.66, 118.97, 106.73, 67.26, 51.98, 42.69, 38.89 (t, J = 19.0 Hz), 31.54, 28.30, 26.25, 18.36.

^{2}H NMR (61 MHz, Chloroform-*d*) δ 2.80 (s).

HRMS (ESI) calcd for $C_{25}H_{27}DNO_2^+$ [$(M+H)^+$] 375.2179, found 375.2182.

Determination of deuterium incorporation by NMR spectroscopy

According to the reported literature[23] and software simulation, the diastereotopic protons Ha and Hb have different chemical shifts when the hydrogen on the same carbon was replaced by deuterium. Incorporation of deuterium was analyzed by ^{1}H

NMR (Figure 1) relevant region is enlarged and aligned for comparison. For ^1H NMR, when deuterium is incorporated, the peak shape and integral of the corresponding position (δ 2.75 – 2.95 ppm) will change. Percent deuterium incorporation was derived from the integration value of this signal and the changes of peak type (multiple peaks to double peaks) means that Ni-D intermediate was through a syn-addition process to the enamide to generate prospective enantioenriched alkylnickel intermediates.

One phenomenon that needs to be mentioned is that when racemic ligands were used, the results of the reaction are similar to the results when use ligands with specific configuration expect for the slight decrease in deuterated ratio. However, when use **dttbpy** (4,4′-Di-*tert*-butyl-2,2′-dipyridyl) as ligand, two enantiomers of coupling products could be obtained.

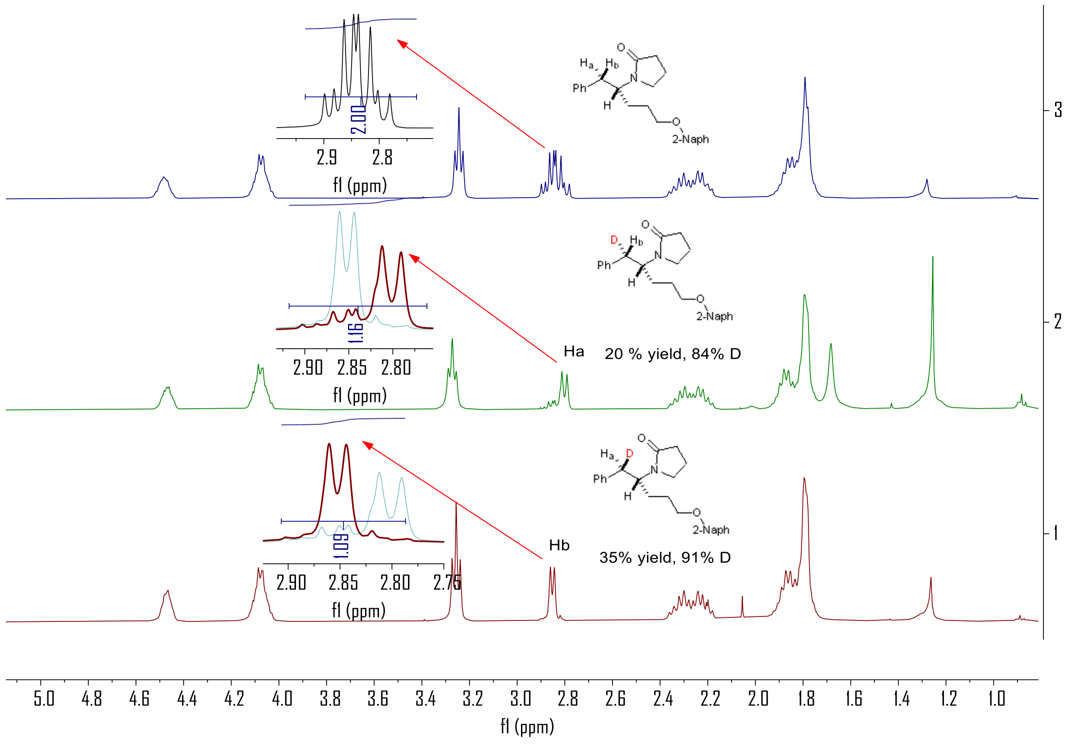

Supplementary Figure 14. local comparison of ^1H NMR spectra between **17**, **77** & **78**

The same result was also confirmed in deuterium-labelling experiments of secondary enecarbamate:

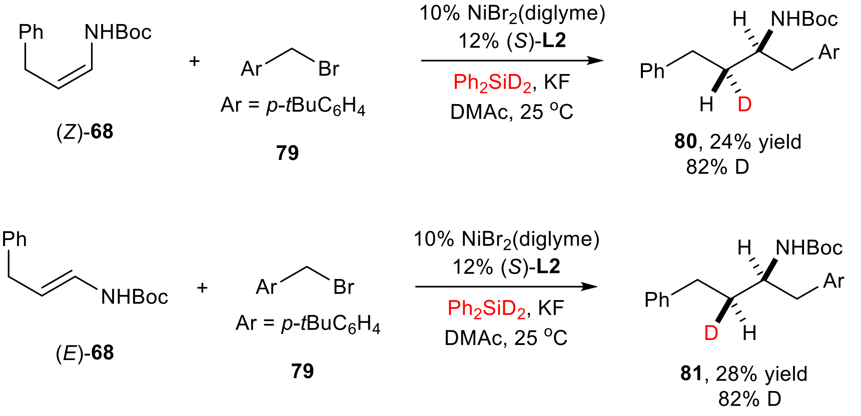

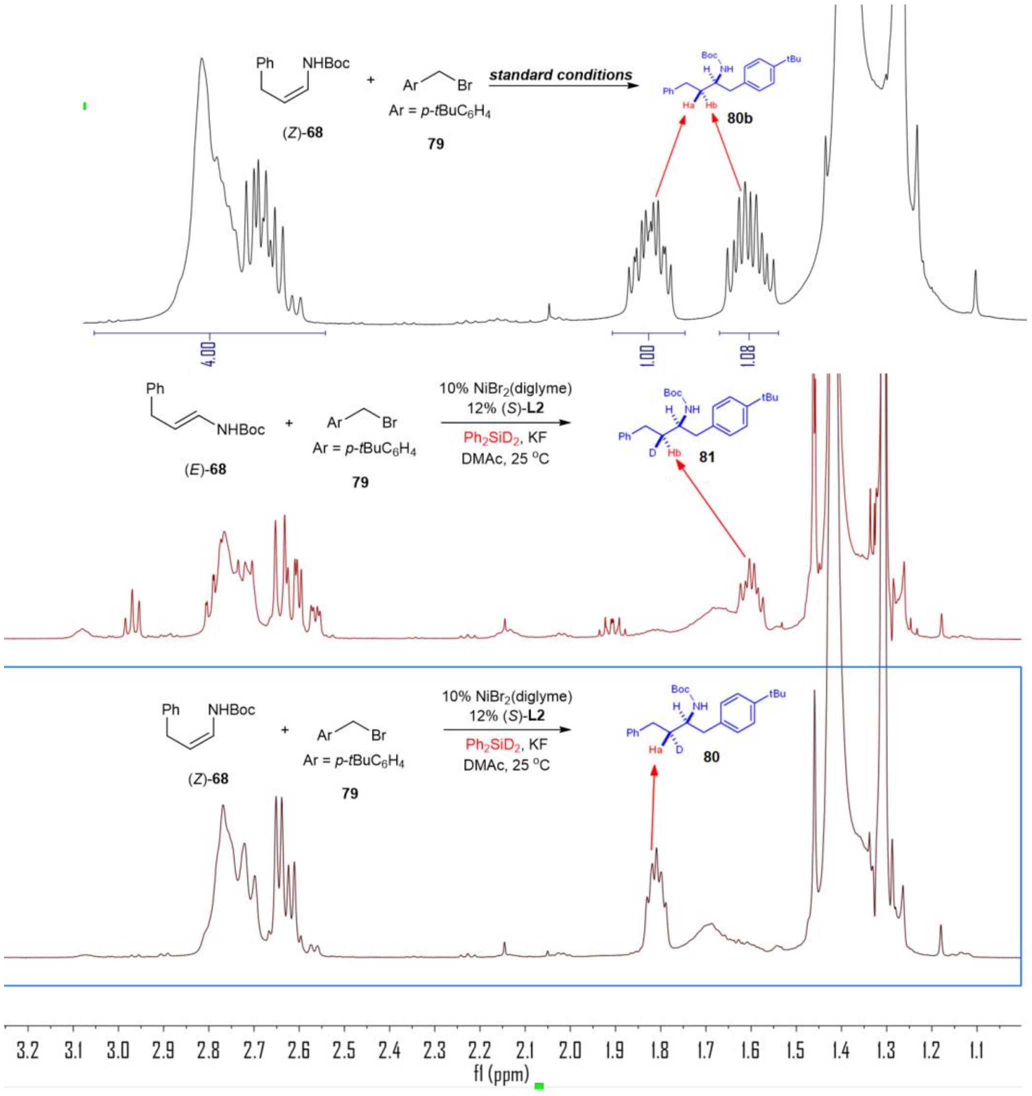

Supplementary Figure 15. local comparison of ^{1}H NMR spectra between **80** & **81**

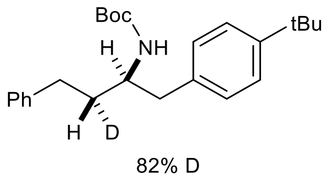

tert-butyl ((2*R*,3*R*)-1-(4-(tert-butyl)phenyl)-4-phenylbutan-2-yl-3-d)carbamate (**80**)

80 was prepared according to **GP-G3** from *tert*-butyl (*Z*)-(3-phenylprop-1-en-1-yl)carbamate. (18.3 mg, 24% isolated yield)

^{1}H NMR (500 MHz, Chloroform-*d*) δ 7.33 – 7.26 (m, 4H), 7.22 – 7.14 (m, 3H), 7.12 – 7.06 (m, 2H), 4.36 (d, *J* = 8.7 Hz, 1H), 3.94 – 3.67 (m, 1H), 2.84 – 2.57 (m, 4H), 1.86 – 1.77 (m, 1H), 1.64 – 1.56 (m, 0.18H) 1.46 – 1.26 (m, 18H).

^{13}C NMR (101 MHz, Chloroform-*d*) δ 155.48, 149.05, 141.90, 134.82, 129.21, 128.38, 128.36, 125.82, 125.20, 79.04, 51.26, 40.88, 35.80 (t, *J* = 16.5 Hz), 34.36, 32.42, 31.37, 28.40.

HRMS (ESI) calcd for $C_{25}H_{34}DNO_2Na^+$ [$(M+Na)^+$] 405.2623, found 405.2625.

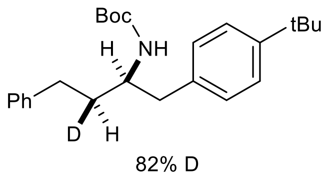

tert-butyl ((2*R*,3*S*)-1-(4-(tert-butyl)phenyl)-4-phenylbutan-2-yl-3-d)carbamate (**81**)

81 was prepared according to **GP-G3** from *tert*-butyl (*E*)-(3-phenylprop-1-en-1-yl)carbamate. (21.4 mg, 28% isolated yield)

^{1}H NMR (500 MHz, Chloroform-*d*) δ 7.33 – 7.26 (m, 4H), 7.22 – 7.13 (m, 3H), 7.13 – 7.04 (m, 2H), 4.36 (d, *J* = 10.5 Hz, 1H), 3.95 – 3.54 (m, 1H), 2.83 – 2.53 (m, 4H), 1.86 – 1.77 (m, 0.18H), 1.64 – 1.56 (m, 1H), 1.52 – 1.20 (m, 18H).

^{13}C NMR (101 MHz, Chloroform-*d*) δ 155.48, 149.07, 141.92, 134.82, 129.21, 128.39, 128.36, 125.82, 125.21, 79.06, 51.27, 40.90, 35.85 (t, *J* = 16.1 Hz), 34.37, 32.43, 31.38, 28.40.

HRMS (ESI) calcd for $C_{25}H_{34}DNO_2Na^+$ [$(M+Na)^+$] 405.2623, found 405.2625.

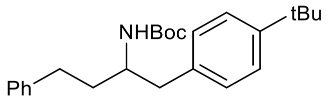

tert-butyl (1-(4-(tert-butyl)phenyl)-4-phenylbutan-2-yl)carbamate (**80-b**)

^{1}H NMR (400 MHz, Chloroform-*d*) δ 7.35 – 7.26 (m, 4H), 7.24 – 7.06 (m, 5H), 4.40 (d, *J* = 9.2 Hz, 1H), 4.00 – 3.62 (m, 1H), 2.90 – 2.51 (m, 4H), 1.91 – 1.80 (m, 1H), 1.69 – 1.58 (m, 1H), 1.52 – 1.29 (m, 18H).

^{13}C NMR (101 MHz, Chloroform-*d*) δ 155.47, 149.04, 141.90, 134.82, 129.20, 128.38, 128.35, 125.81, 125.20, 79.03, 51.32, 40.92, 36.21, 34.35, 32.52, 31.37, 28.40.

Synthesis of (*Z*)-Tertiary enamides

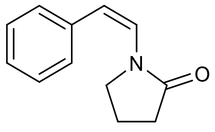

(*Z*)-1-styrylpyrrolidin-2-one (***Z*-75**)

According to the reported literature[24], ***Z*-75** was obtained as colorless oil.

^{1}H NMR (500 MHz, Chloroform-*d*) δ 7.53 – 7.07 (m, 5H), 6.80 (d, *J* = 9.7 Hz, 1H), 6.00 (d, *J* = 9.8 Hz, 1H), 3.45 – 3.12 (m, 2H), 2.63 – 2.35 (m, 2H), 2.08 – 1.85 (m, 2H).

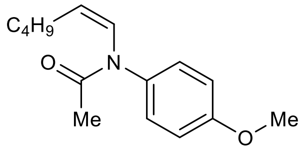

(*Z*)-*N*-(hex-1-en-1-yl)-*N*-(4-methoxyphenyl)acetamide (***Z*-82**)

According to the reported literature[25], ***Z*-82** was obtained as brown oil.

^{1}H NMR (500 MHz, Chloroform-*d*) δ 7.10 (d, *J* = 8.4 Hz, 2H), 6.88 (d, *J* = 8.4 Hz, 2H), 6.63 (d, *J* = 9.3 Hz, 0.73H), 6.26 (d, *J* = 7.9 Hz, 0.27H), 5.31 (q, *J* = 7.8 Hz, 0.27H), 4.88 (q, *J* = 7.9 Hz, 0.72H), 3.79 (s, 3H), 2.17 (s, 0.79H), 1.88 (s, 2.81H), 1.55 – 1.38 (m, 1.45H), 1.28 – 1.17 (m, 1.15H), 1.10 – 1.00 (m, 2.94H), 0.87 – 0.61 (m, 3H).

Supplementary Note 4

Effect of Configuration of Enamide and Ligand on Coupling Yields and Enantioselectiveties

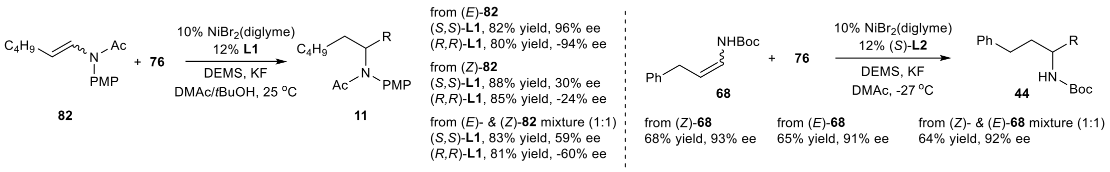

Effect of configuration of ligand on enantioselectivities: when different enantiomers of ligands were used in the reaction, we found that the major enantiomer of the product was determined by the configuration of the ligands.

Effect of configuration of Enamide on coupling yields and enantioselectivities: for tertiary enamides, the (*E*)- or (*Z*)-enamides afforded comparable coupling yields but quite different enantioselectivities, the (*E*)-substrate afforded a much higher level of enantioselectivity. For secondary enecarbamates, both (*E*)- and (*Z*)-enecarbamates afforded comparable coupling yields and enantioselectiveties.

Study of *Z/E* Interconversion of Enamide and Enecarbamate.

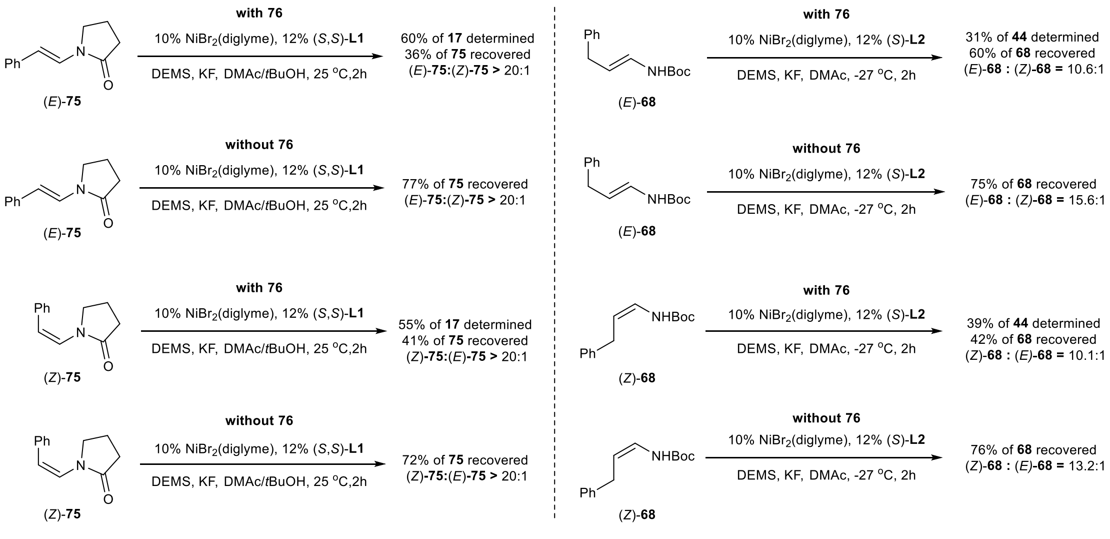

Considering the similar results obtained from (*E*)- and (*Z*)-secondary enecarbamate, one possibility is that the configuration of enecarbamate has isomerized under the

reaction conditions. The reaction was designed as follows: the olefin recovery test was carried out in the presence or absence of alkyl iodine. Although when compared with tertiary enamide, the *E/Z* isomerization of secondary enecarbamate was slightly higher, this degree of isomerization is still not enough to affect the reaction.

Supplementary Note 5

Proposed Mechanism and DFT Calculations

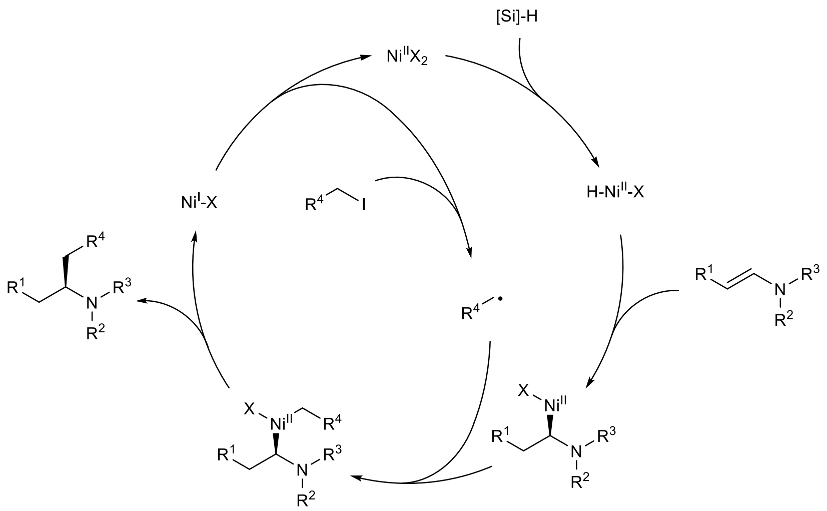

Supplementary Figure 16. Proposed Mechanism

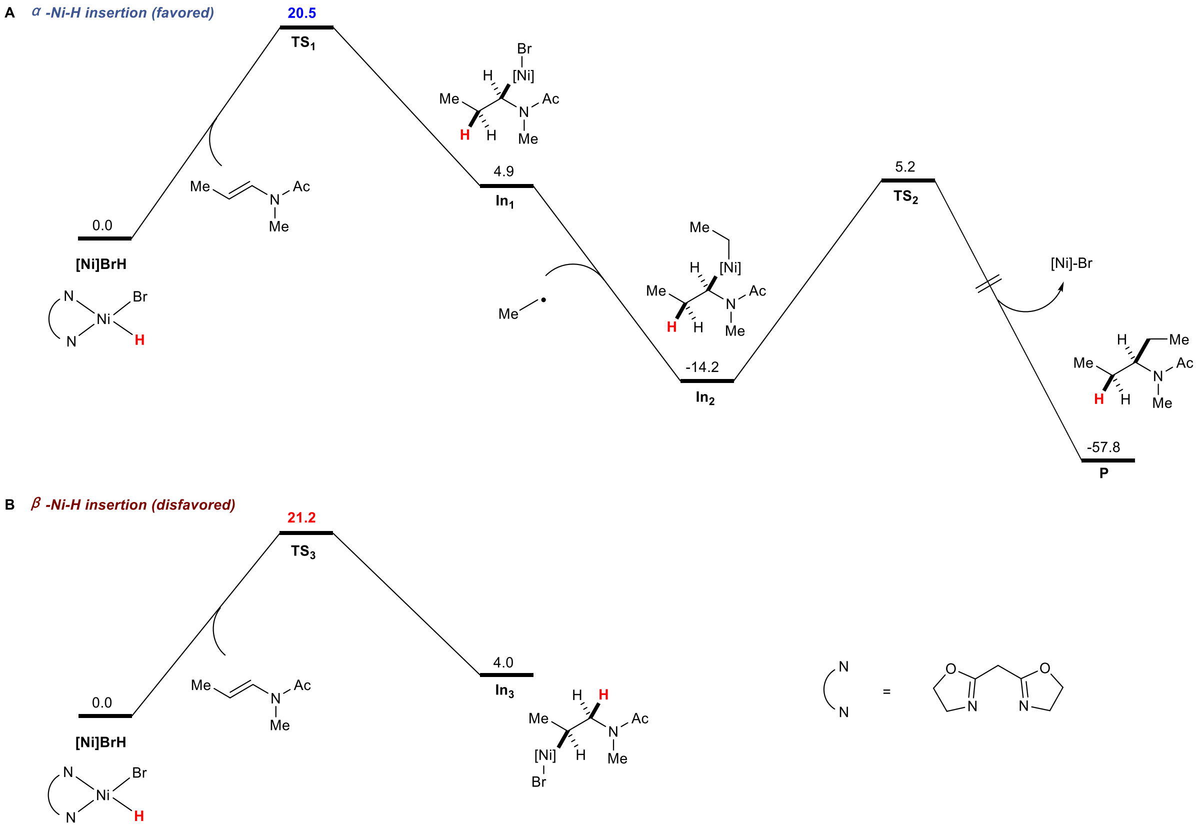

Supplementary Figure 17. DFT Calculations

Gibbs free energy profiles of (A) α-Ni-H insertion (favored) and subsequent transformation, and (B) β-Ni-H insertion (disfavored) (in kcal/mol). Complexes are computed with the simplified ligand.

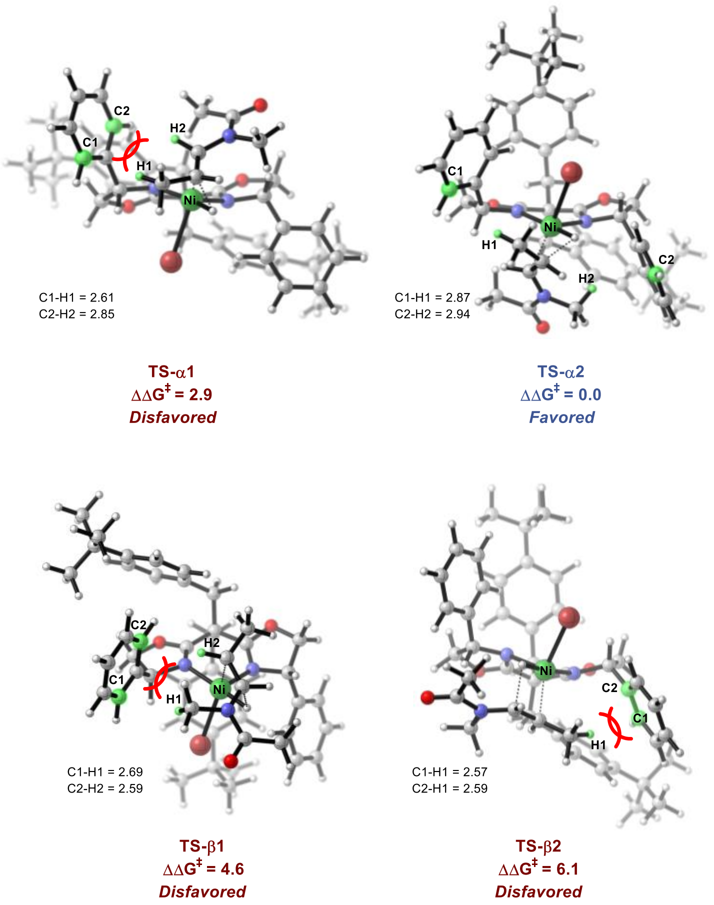

Supplementary Figure 18. Optimized structures of **TS-α1**, **TS-α2**, **TS-β1**, and **TS-β2**.

Relative Gibbs free energies and bond distances are given in kcal/mol and angstroms, respectively.

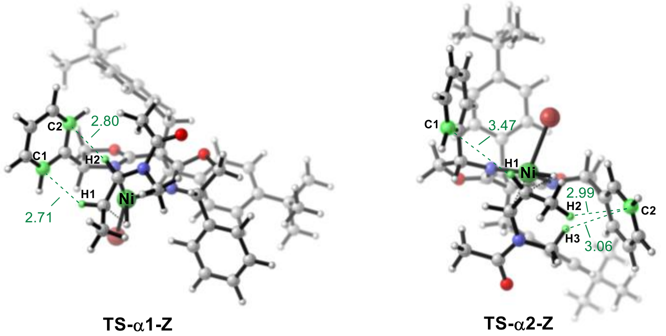

TS-α1-Z
$\Delta\Delta G^{\ddagger}$ = 1.6

TS-α2-Z
$\Delta\Delta G^{\ddagger}$ = 0.0

Supplementary Figure 19. Optimized structures of **TS-α1-Z** and **TS-α2-Z** (with the *Z*- substrate).

Relative Gibbs free energies and bond distances are given in kcal/mol and angstroms, respectively.

Computational details.

All calculations were performed with Gaussian 16 suite of programs.[26] The B3LYP functional[27,28] including the implicit solvent model SMD[29] and Grimme empirical dispersion correction (GD3BJ)[30] was used for the geometry optimization of all structures in DMA (*N*, *N*-dimethylacetamide). LANL2DZ basis set[31-33] was used for nickel and the 6-31G* basis set was used for the other atoms. Frequency calculations were performed at the same level of geometry optimization to verify that each of the transition states has one imaginary frequency. The single-point energies were computed with the SDD basis set[34-36] for nickel and the 6-311G(d,p) basis set for the other atoms based on the optimized structures. The 3D diagrams of molecules were generated using CYLView.[37]

Supplementary Figures

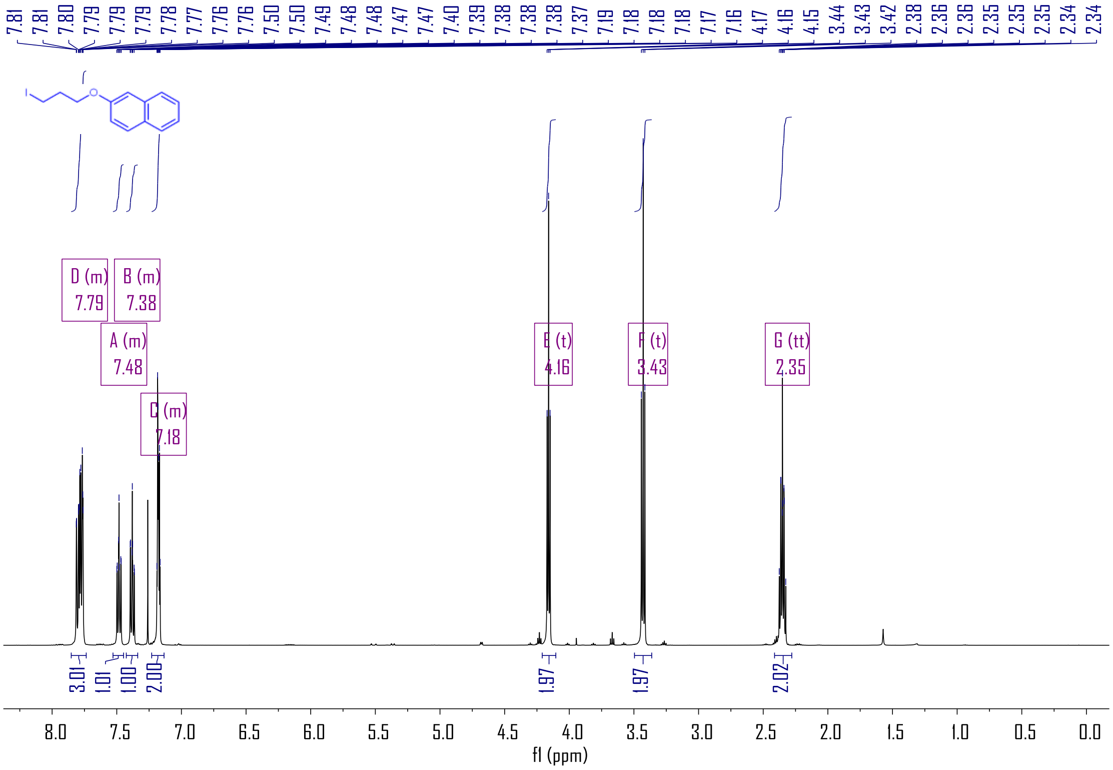

Supplementary Figure 20. ^{1}H NMR spectra for **RM1**

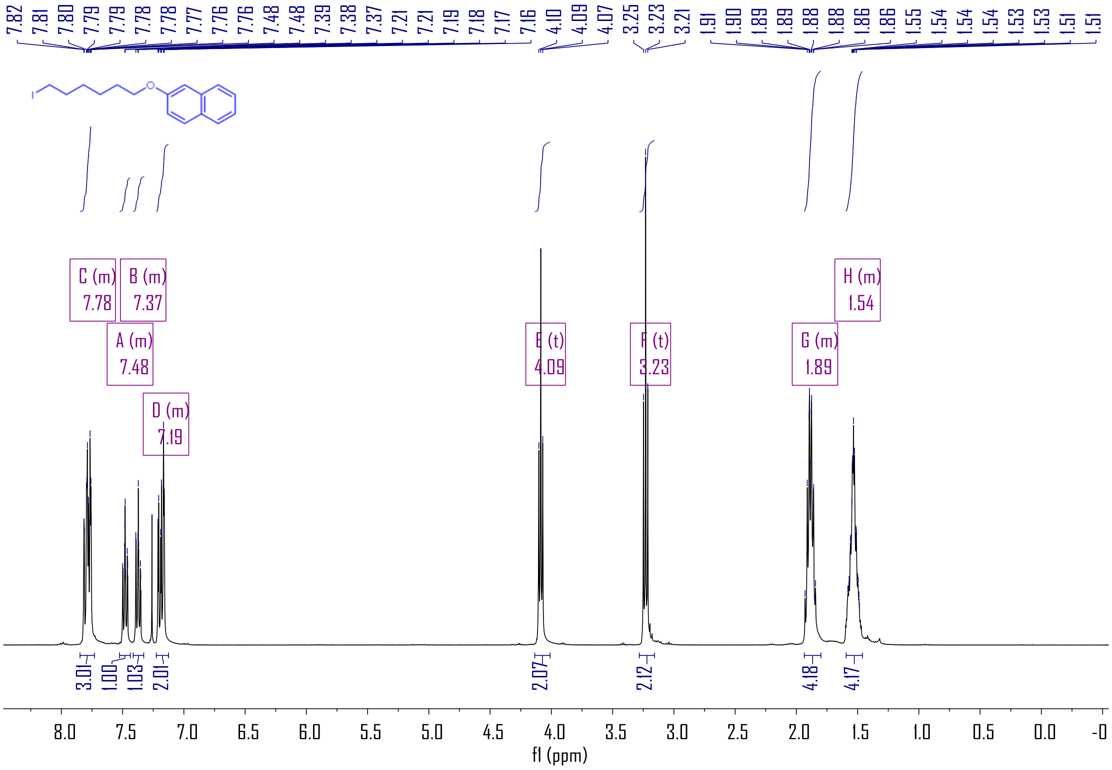

Supplementary Figure 21. ^{1}H NMR spectra for **RM2**

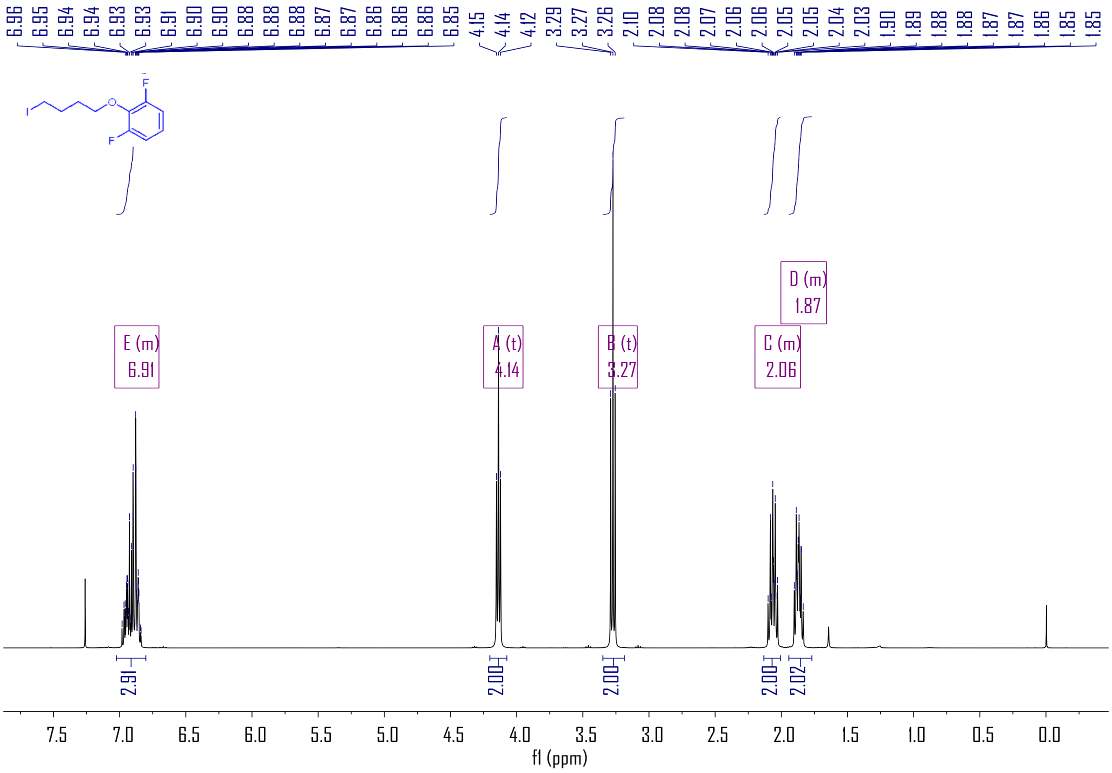

Supplementary Figure 22. ^{1}H NMR spectra for **RM3**

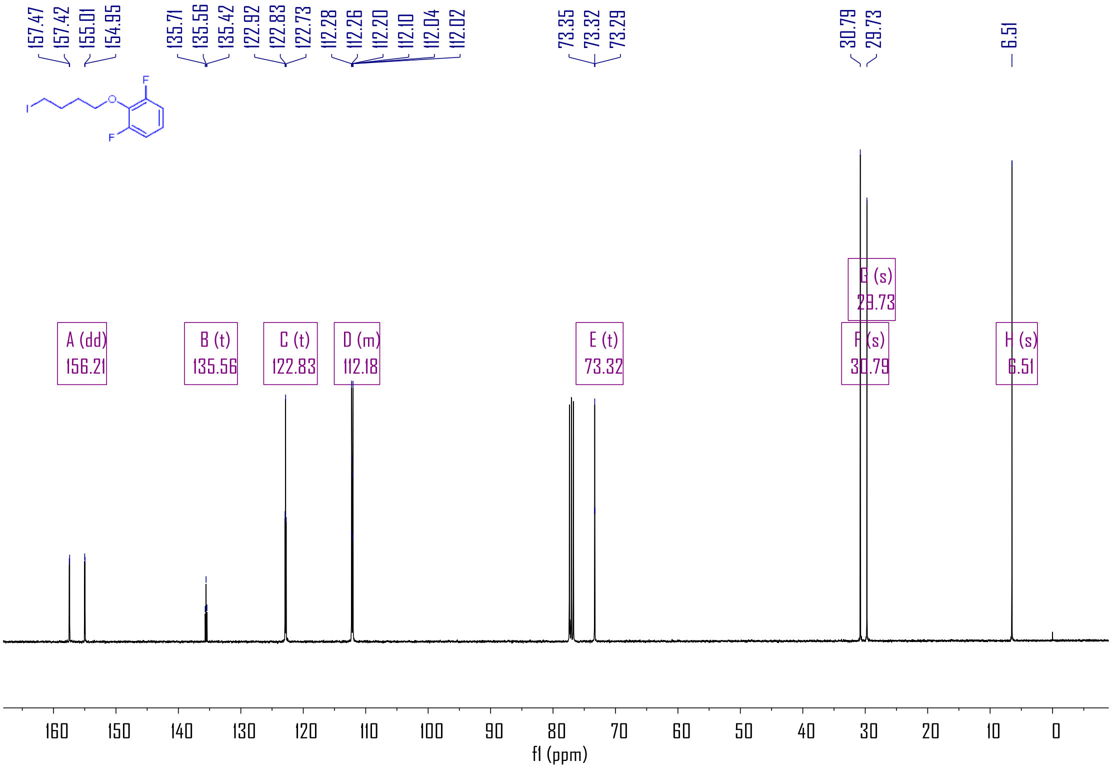

Supplementary Figure 23. ^{13}C NMR spectra for **RM3**

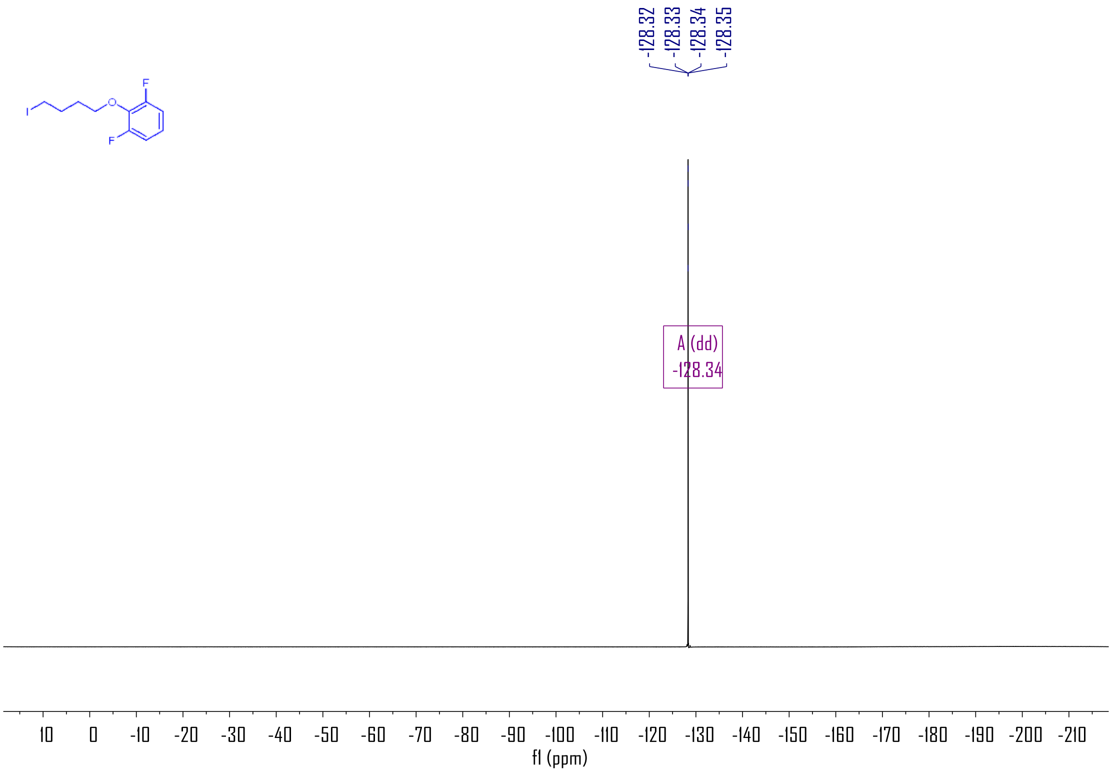

Supplementary Figure 24. ^{19}F NMR spectra for **RM3**

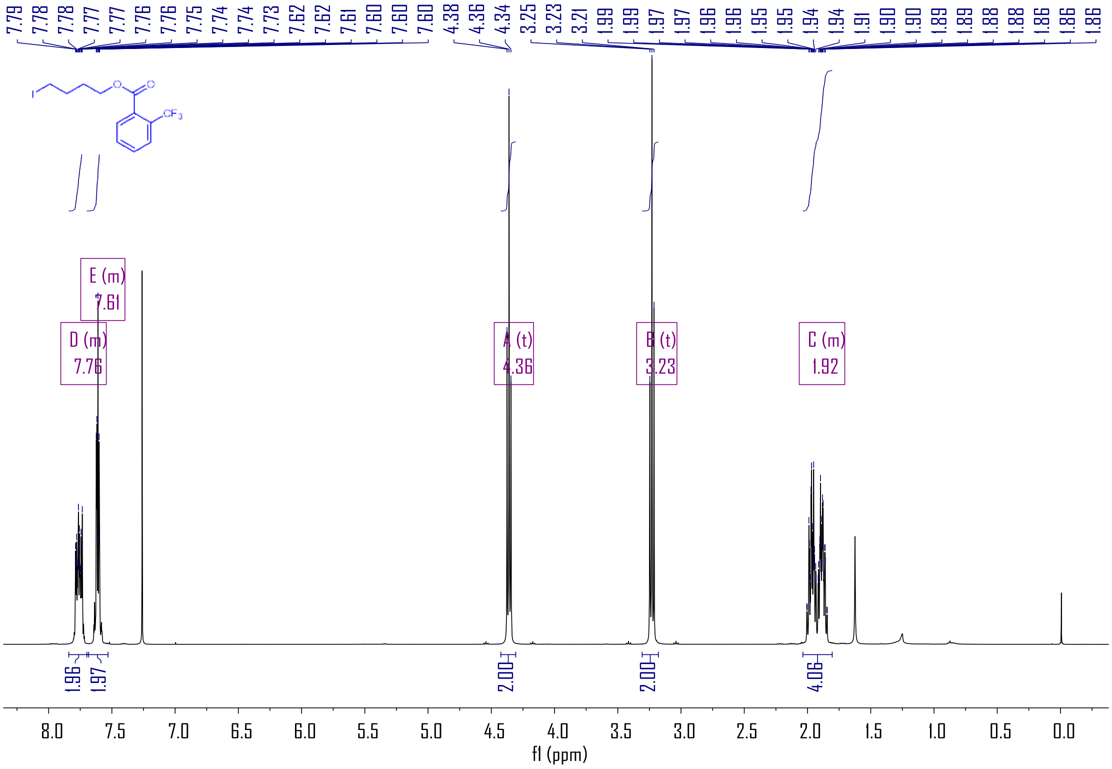

Supplementary Figure 25. ^{1}H NMR spectra for **RM4**

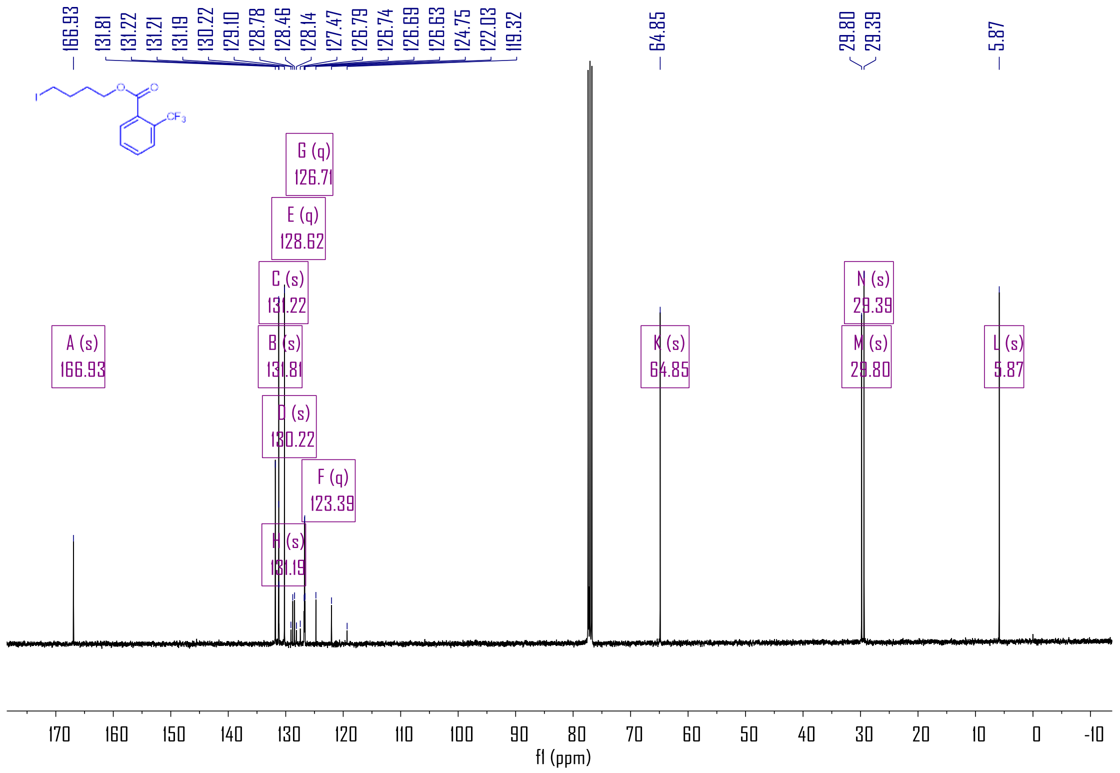

Supplementary Figure 26. ^{13}C NMR spectra for **RM4**

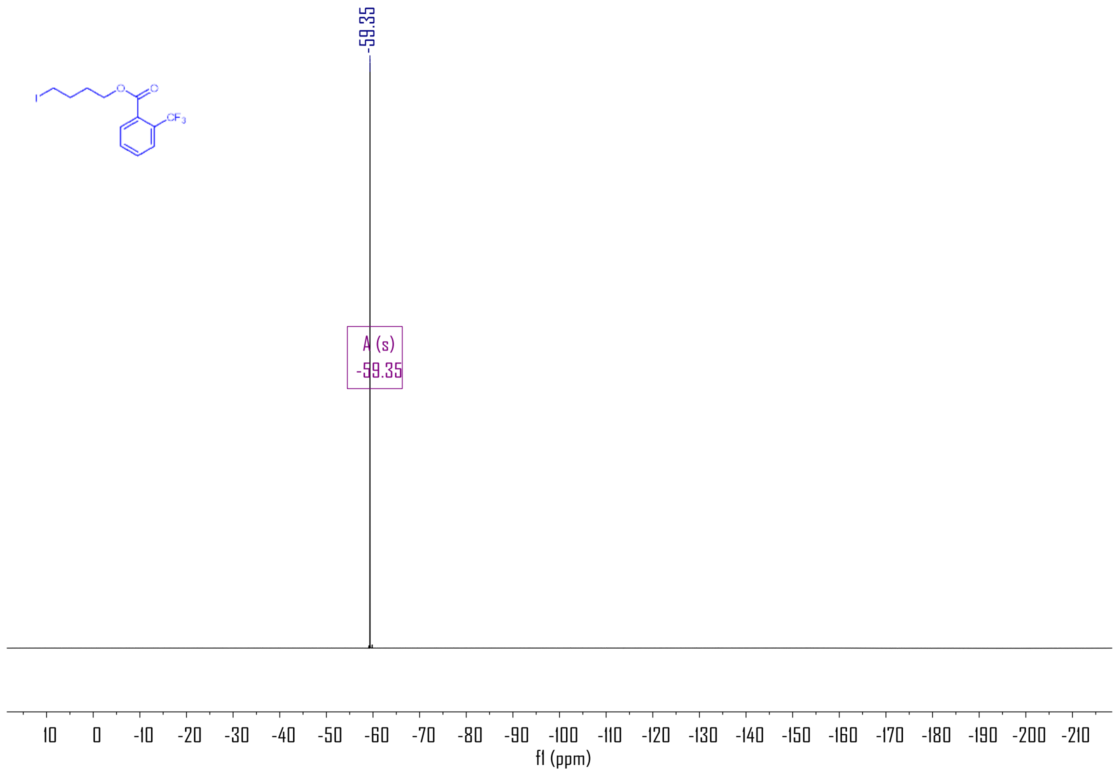

Supplementary Figure 27. ^{19}F NMR spectra for **RM4**

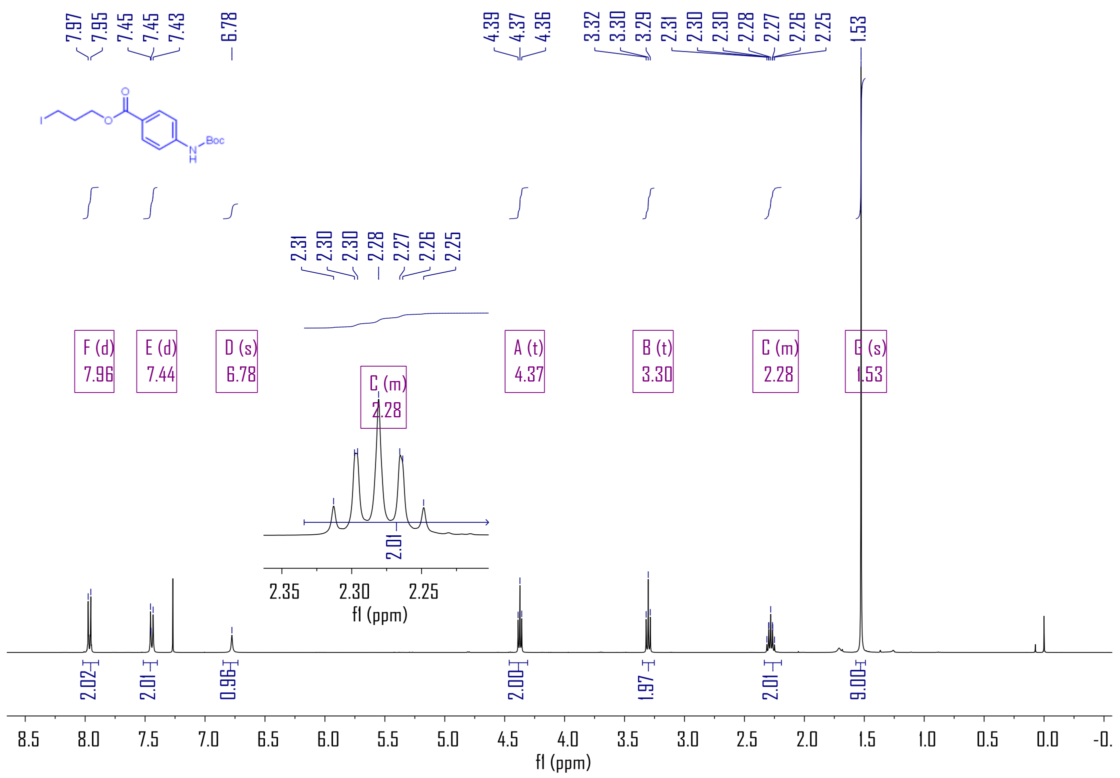

Supplementary Figure 28. ^{1}H NMR spectra for **RM5**

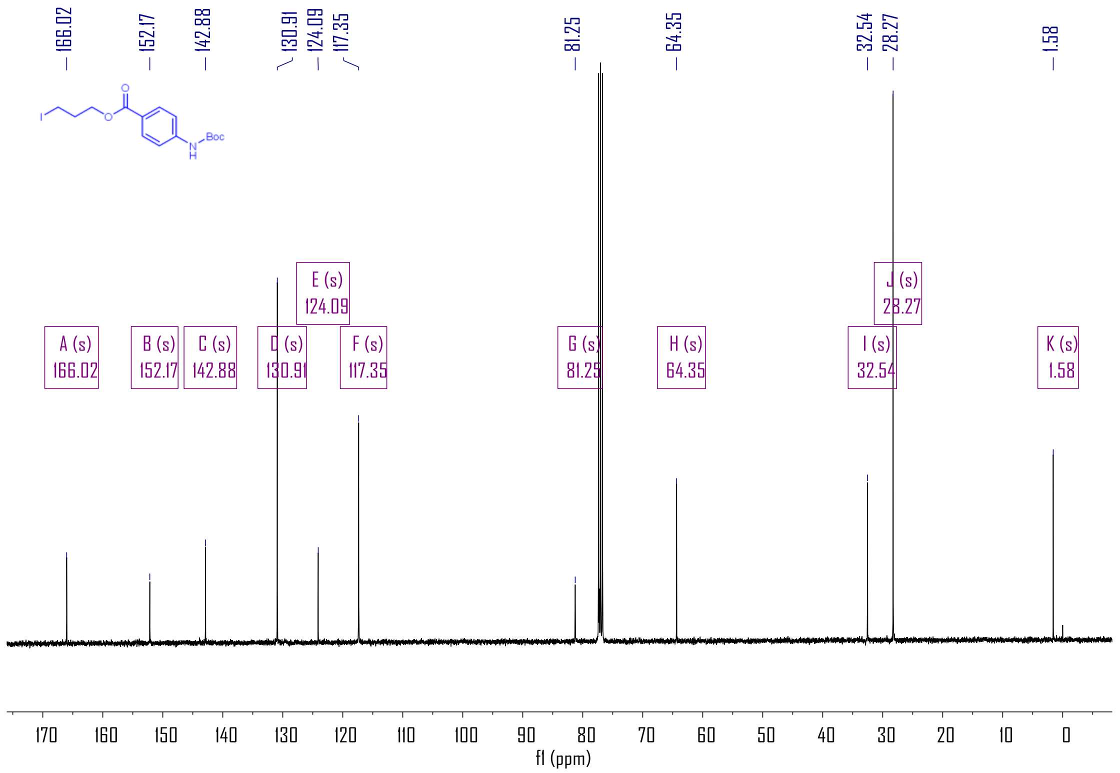

Supplementary Figure 29. ^{13}C NMR spectra for **RM5**

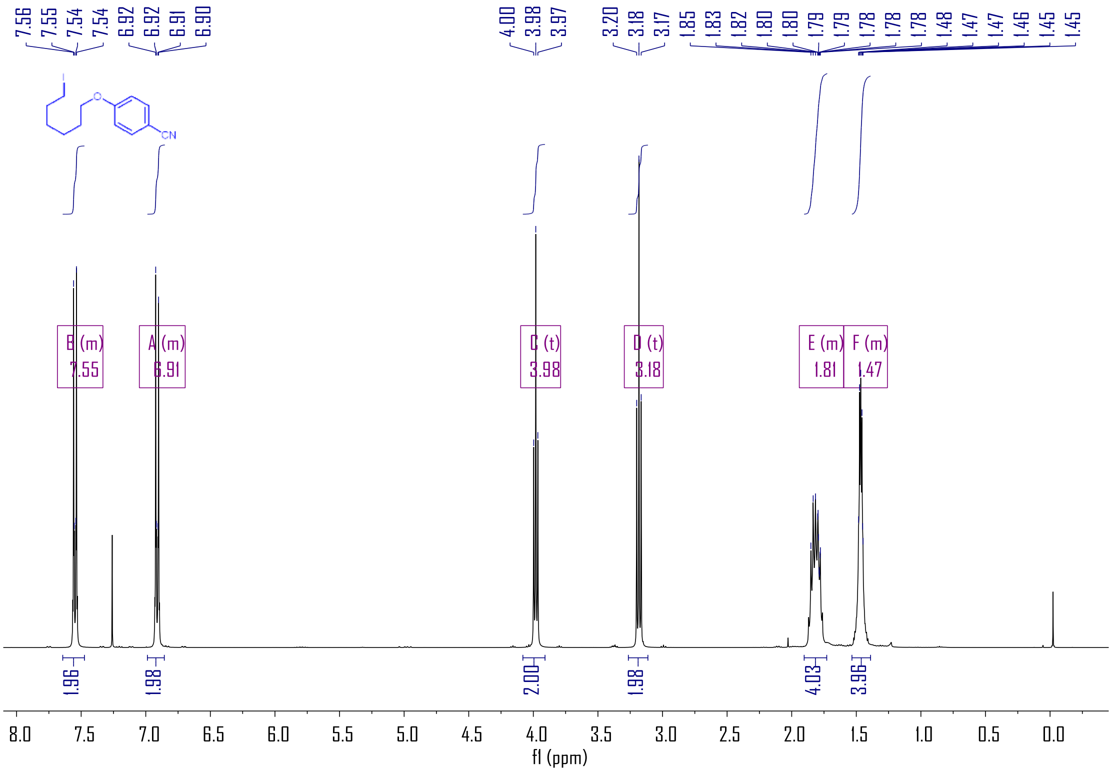

Supplementary Figure 30. ^{1}H NMR spectra for **RM6**

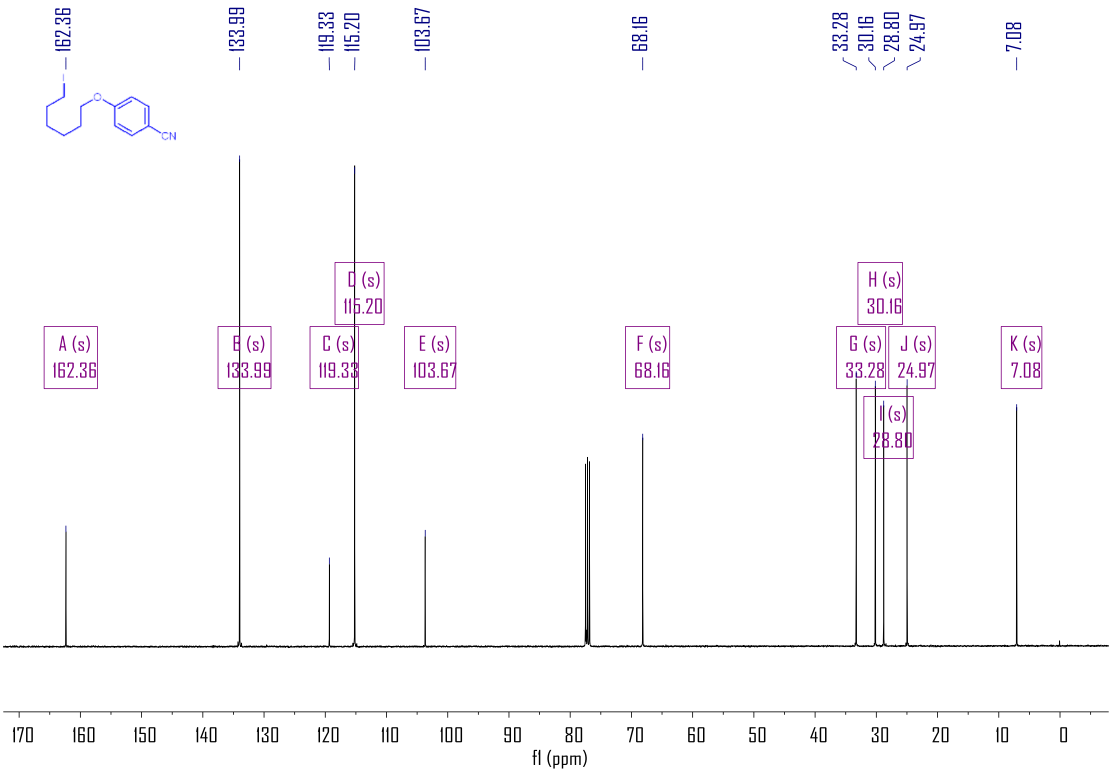

Supplementary Figure 31. ^{13}C NMR spectra for **RM6**

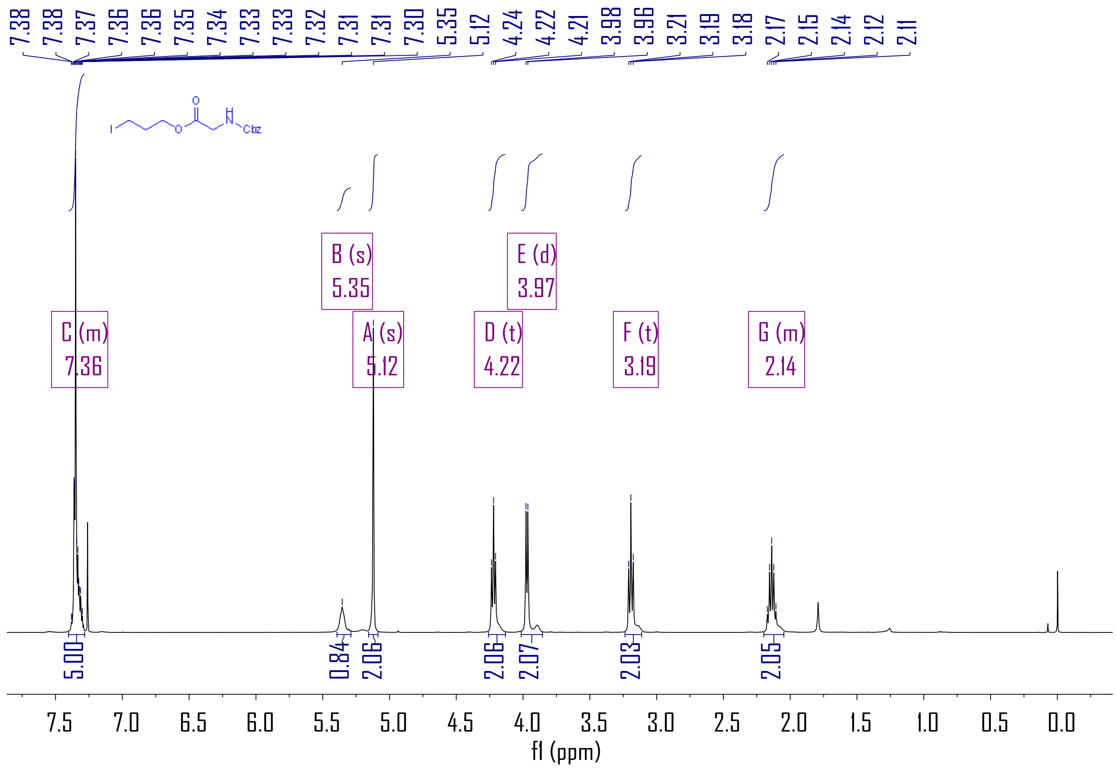

Supplementary Figure 32. ^{1}H NMR spectra for **RM7**

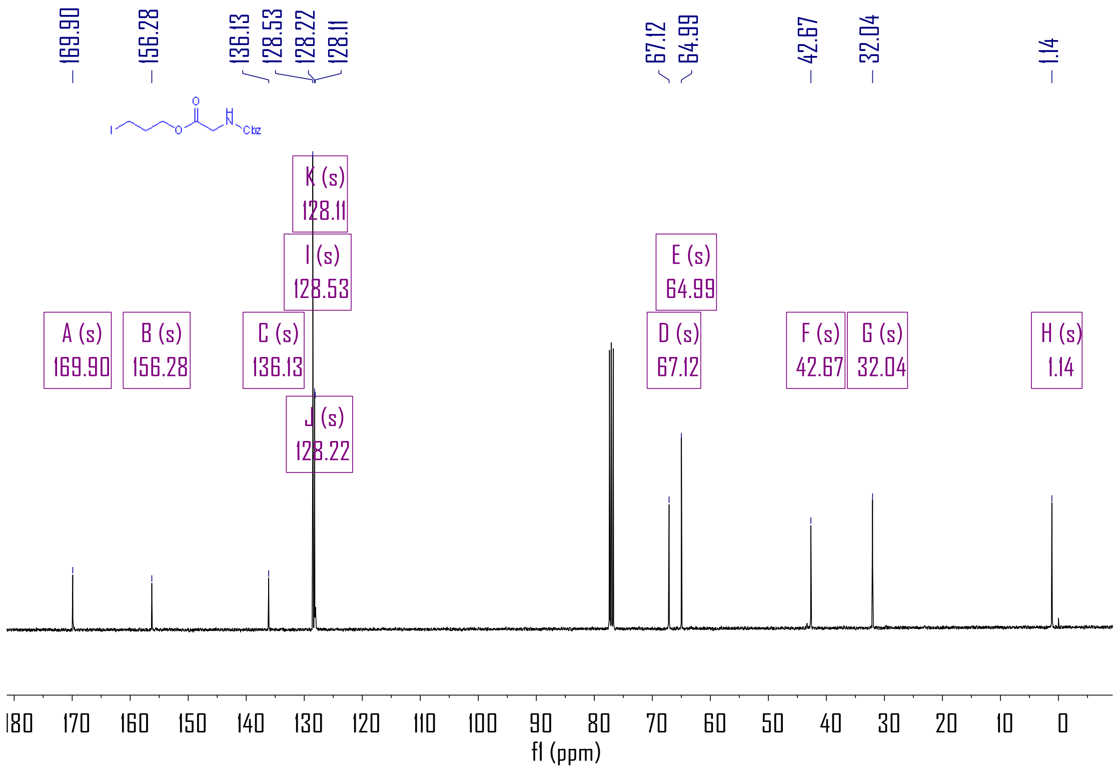

Supplementary Figure 33. ^{13}C NMR spectra for **RM7**

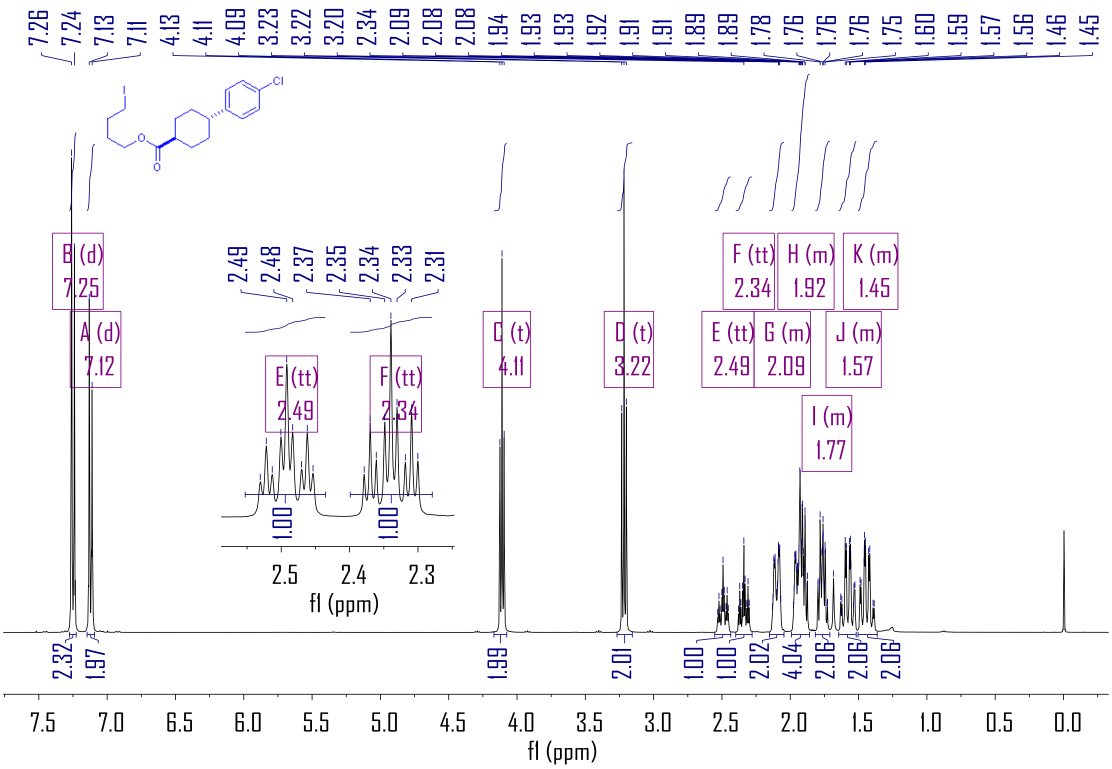

Supplementary Figure 34. ^{1}H NMR spectra for **RM8**

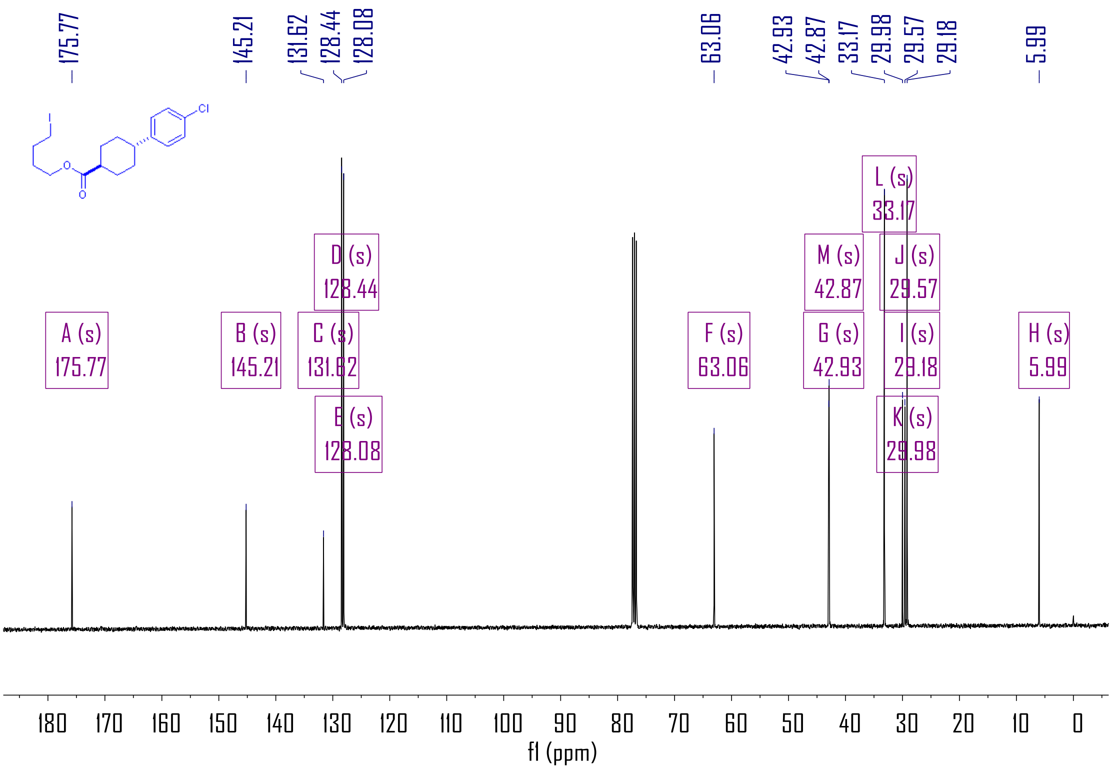

Supplementary Figure 35. ^{13}C NMR spectra for **RM8**

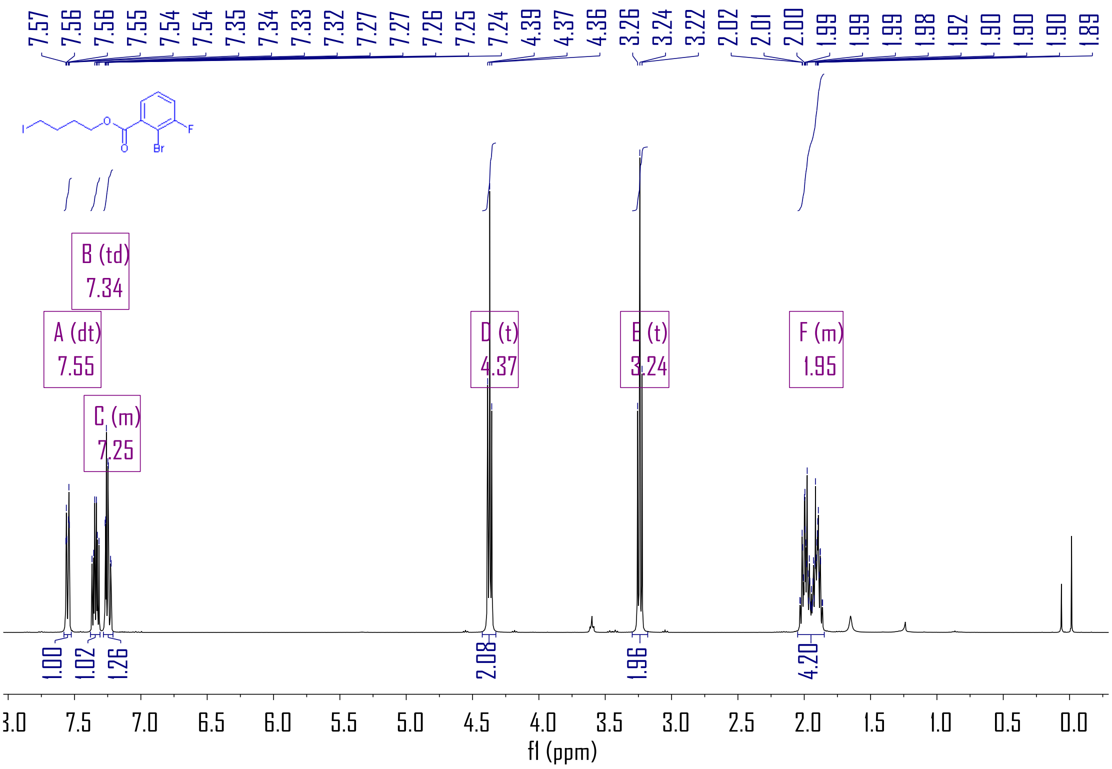

Supplementary Figure 36. ^{1}H NMR spectra for **RM9**

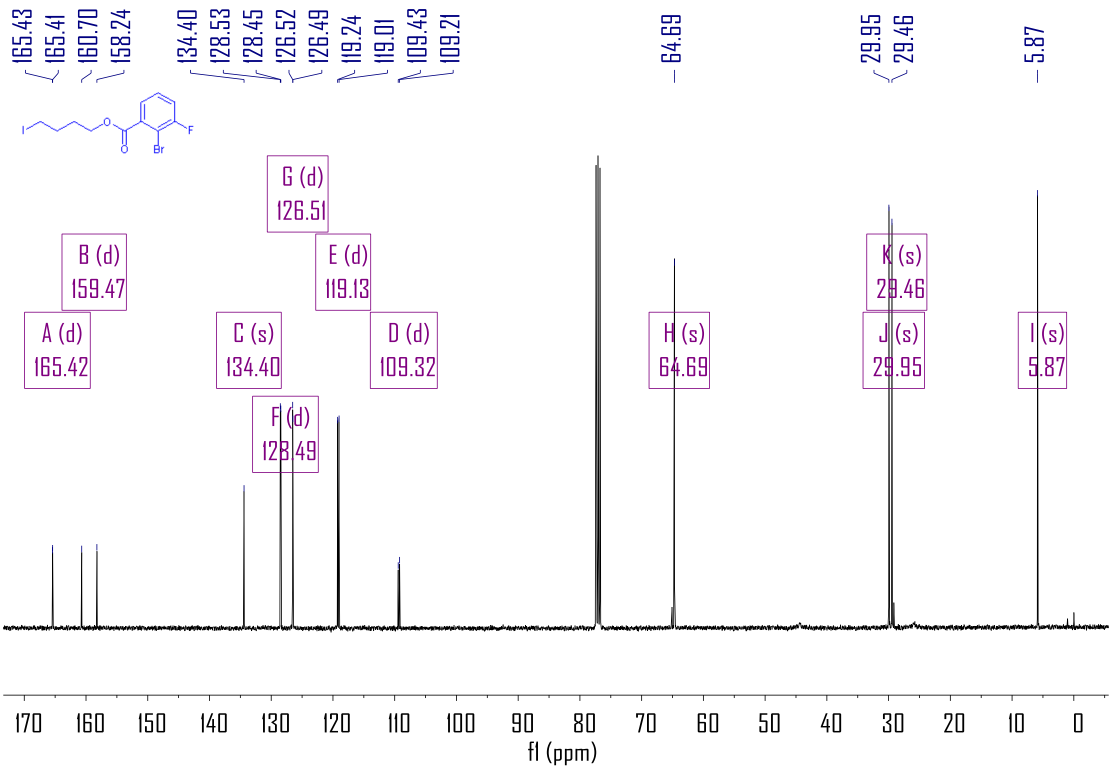

Supplementary Figure 37. ^{13}C NMR spectra for **RM9**

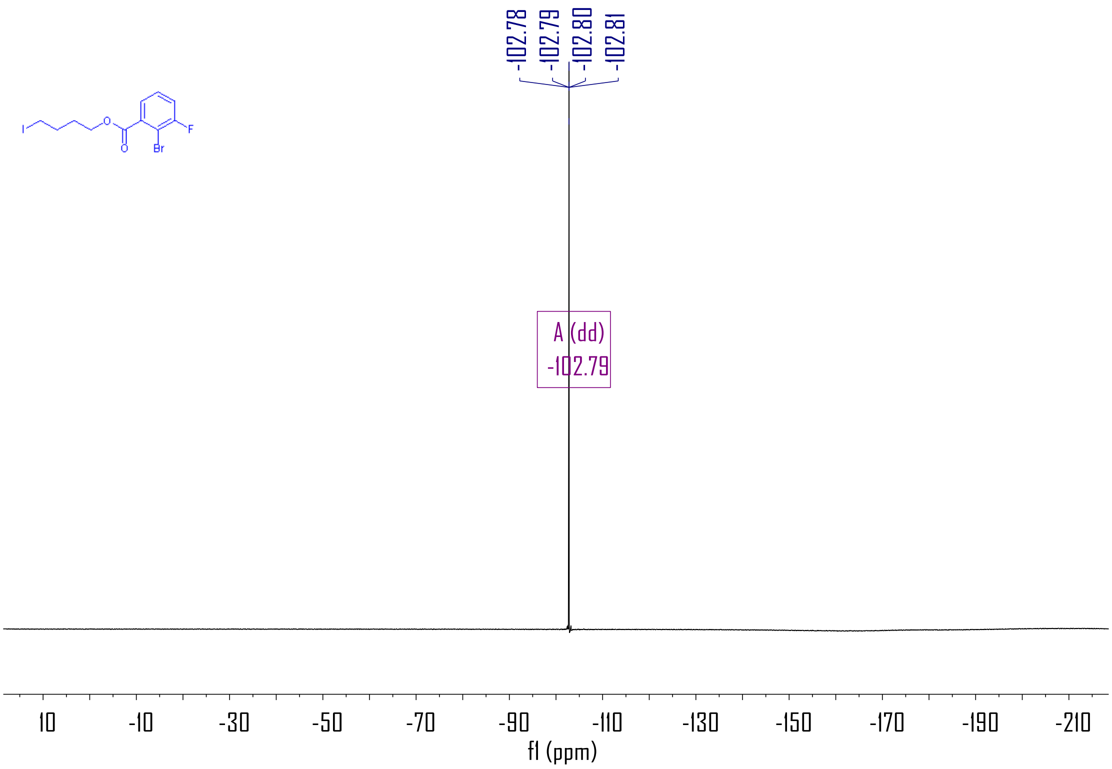

Supplementary Figure 38. ^{19}F NMR spectra for **RM9**

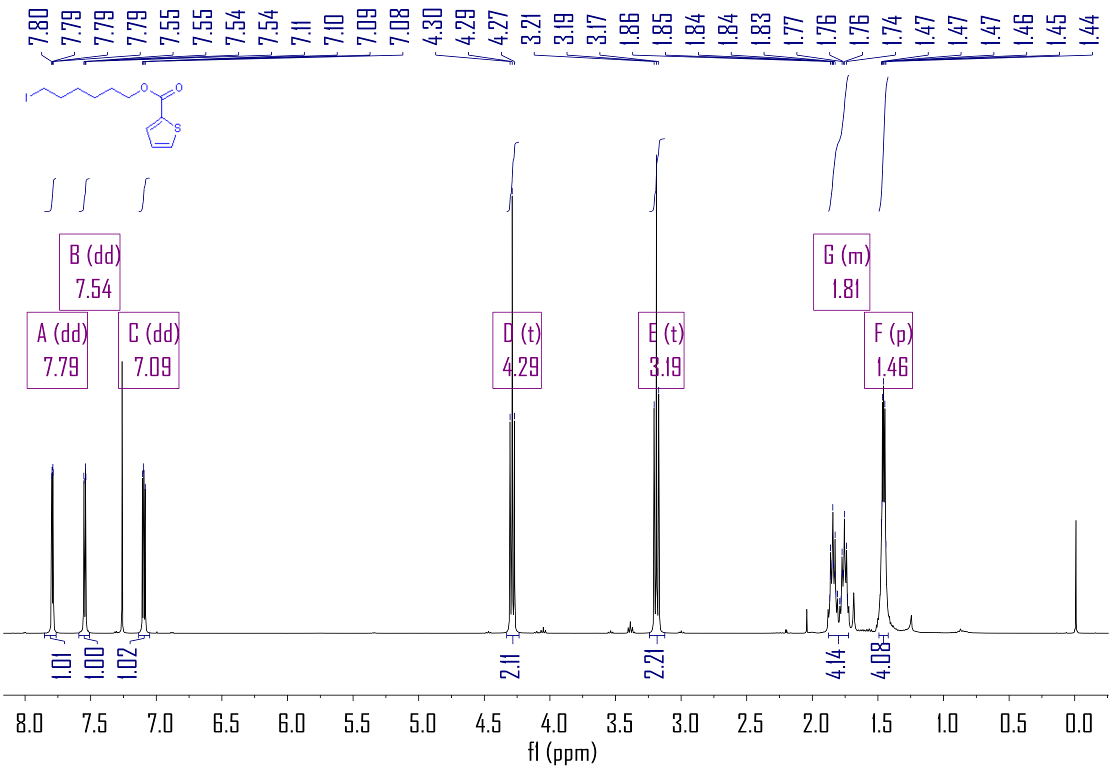

Supplementary Figure 39. ^{1}H NMR spectra for **RM10**

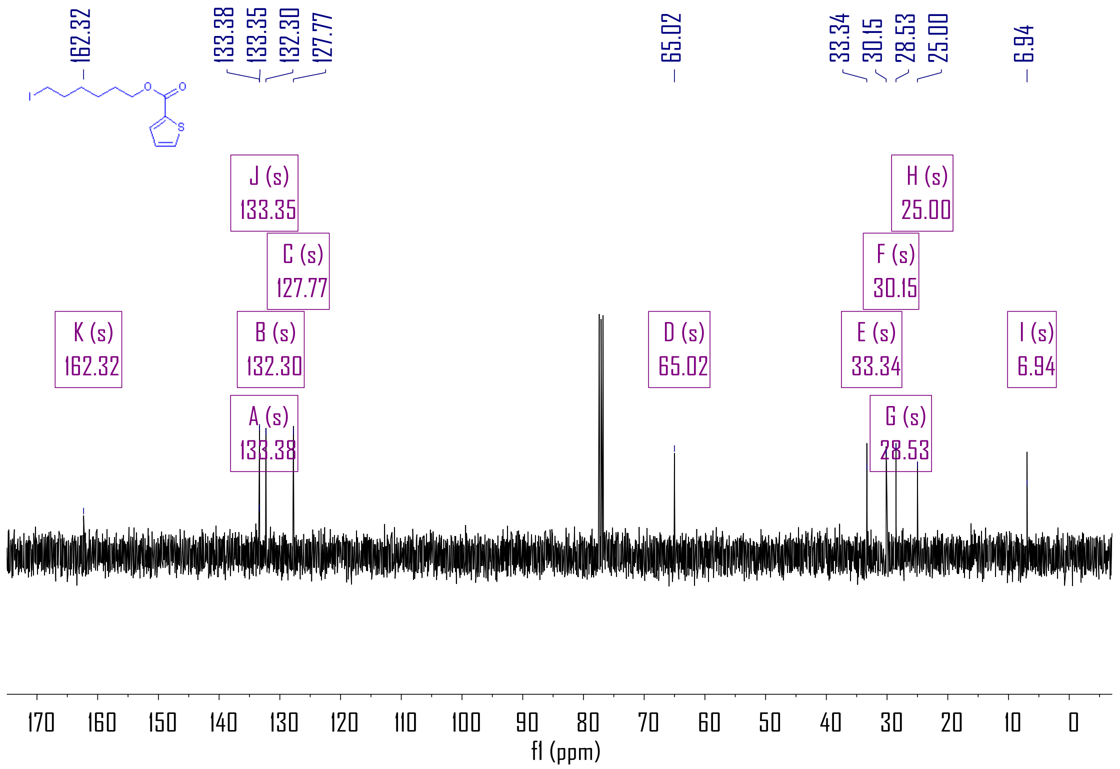

Supplementary Figure 40. ^{13}C NMR spectra for **RM10**

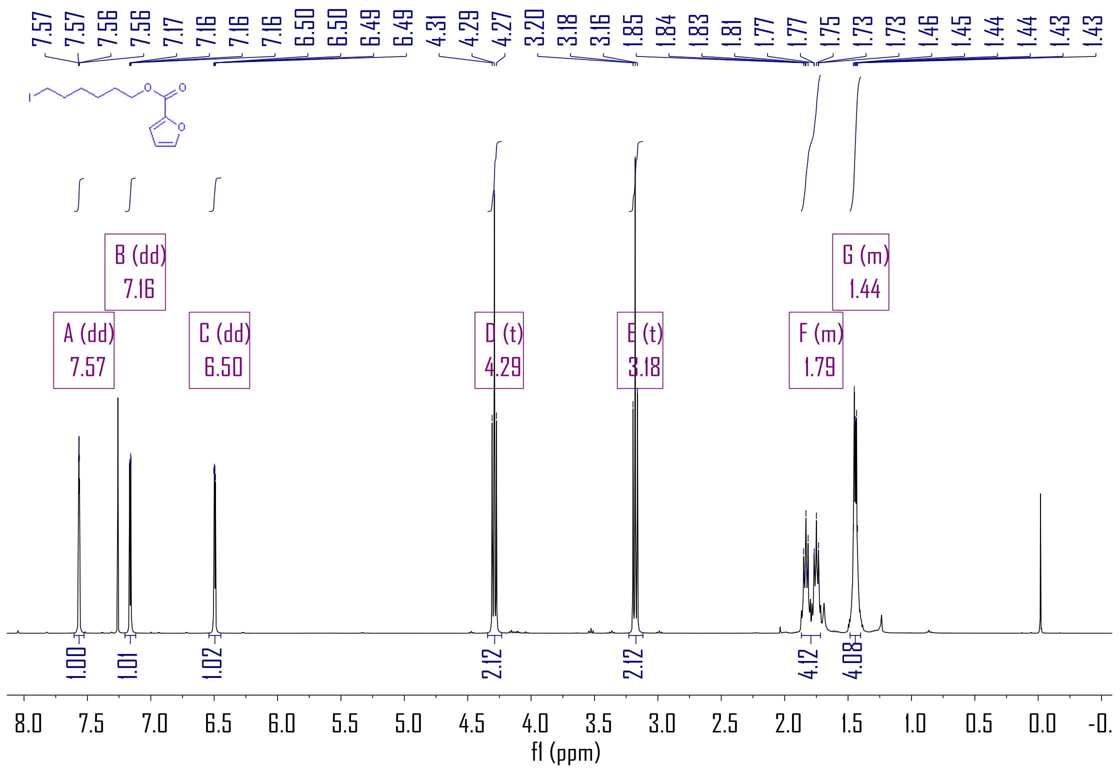

Supplementary Figure 41. ^{1}H NMR spectra for **RM11**

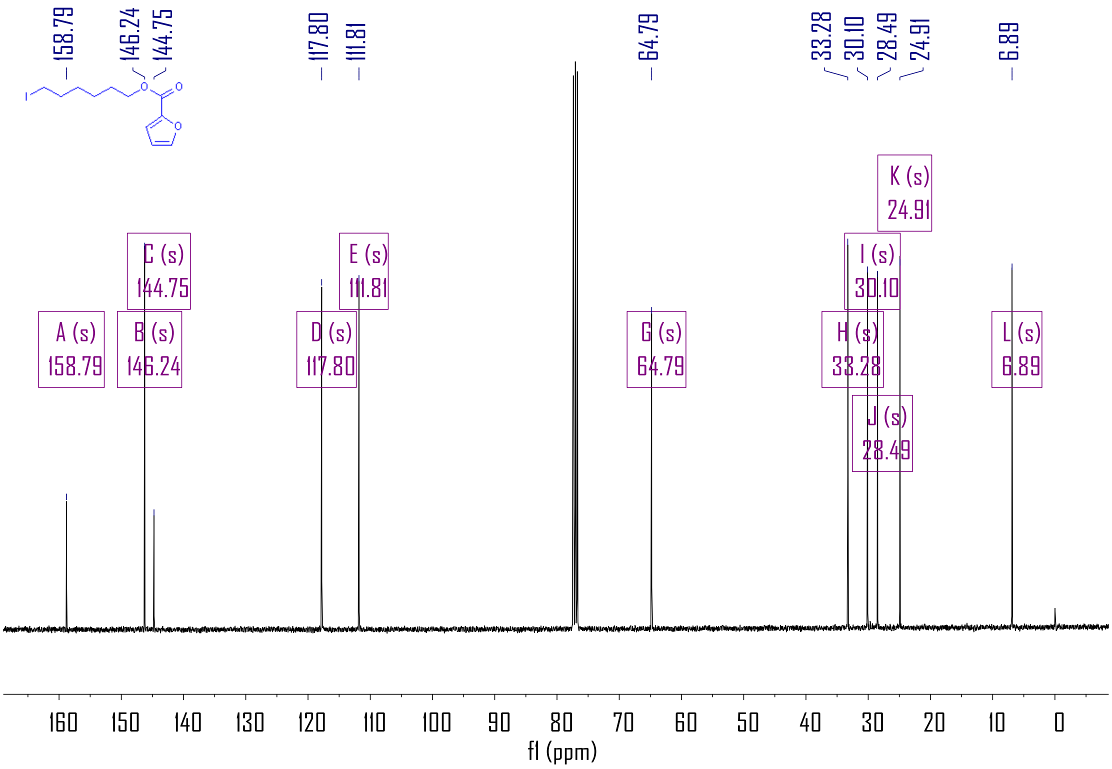

Supplementary Figure 42. ^{13}C NMR spectra for **RM11**

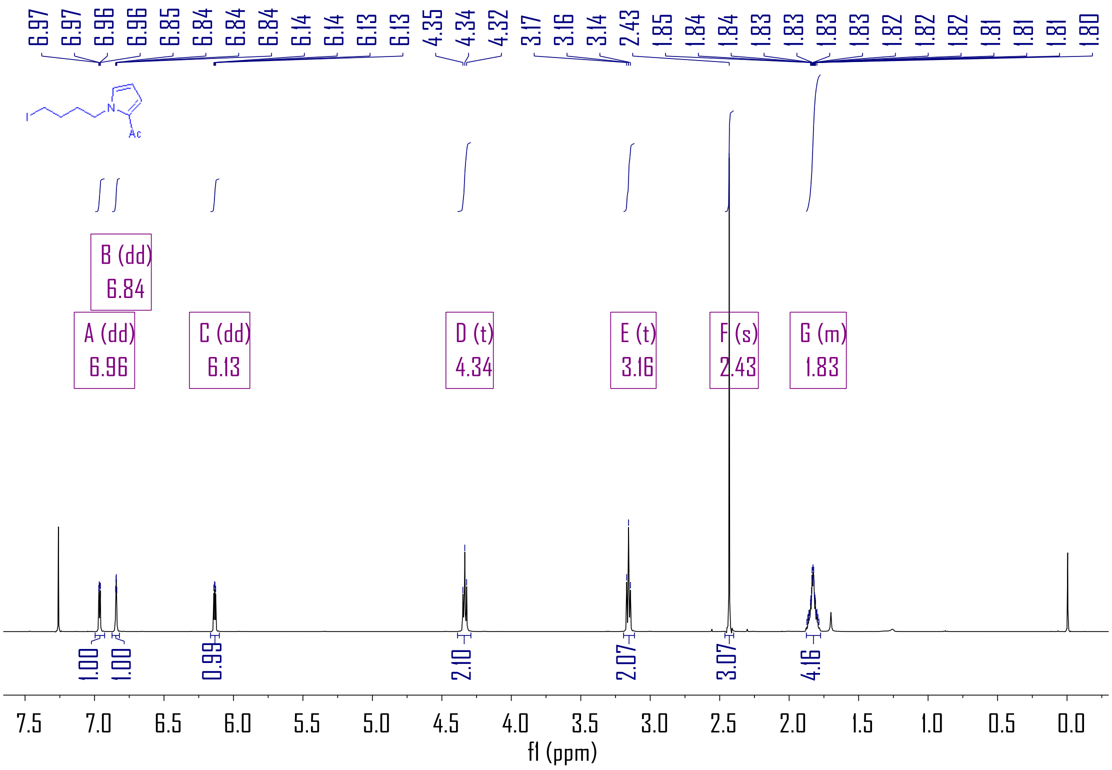

Supplementary Figure 43. ^{1}H NMR spectra for **RM12**

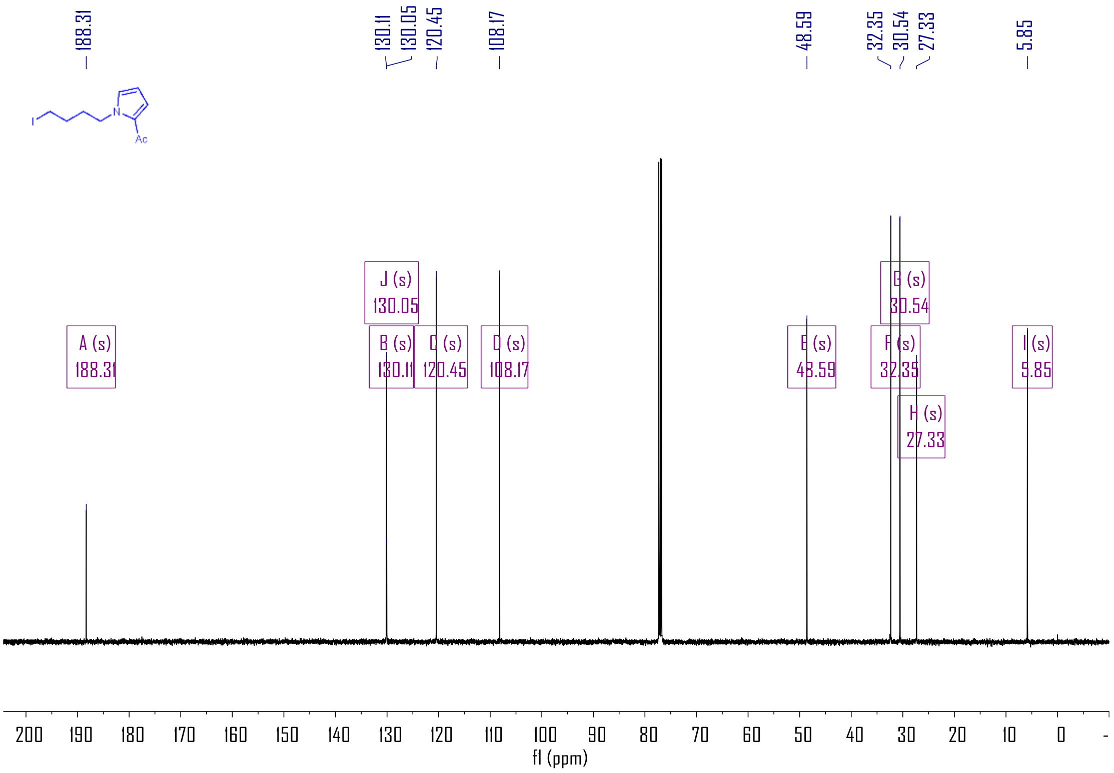

Supplementary Figure 44. ^{13}C NMR spectra for **RM12**

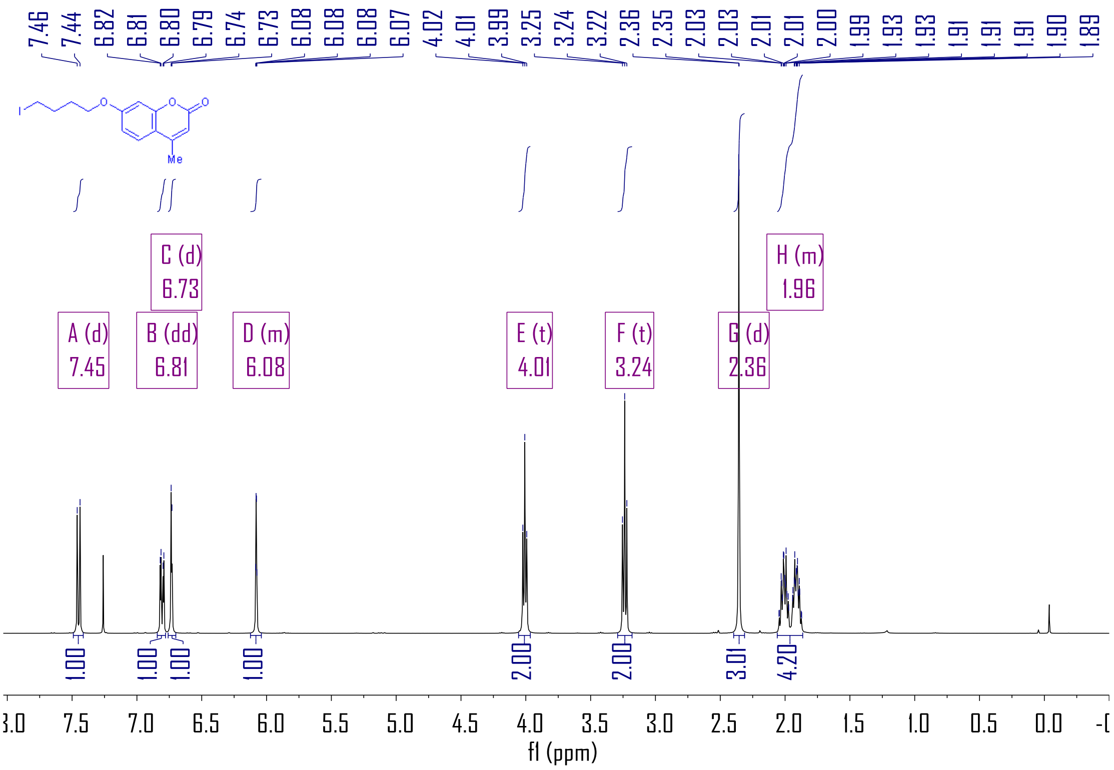

Supplementary Figure 45. ^{1}H NMR spectra for **RM13**

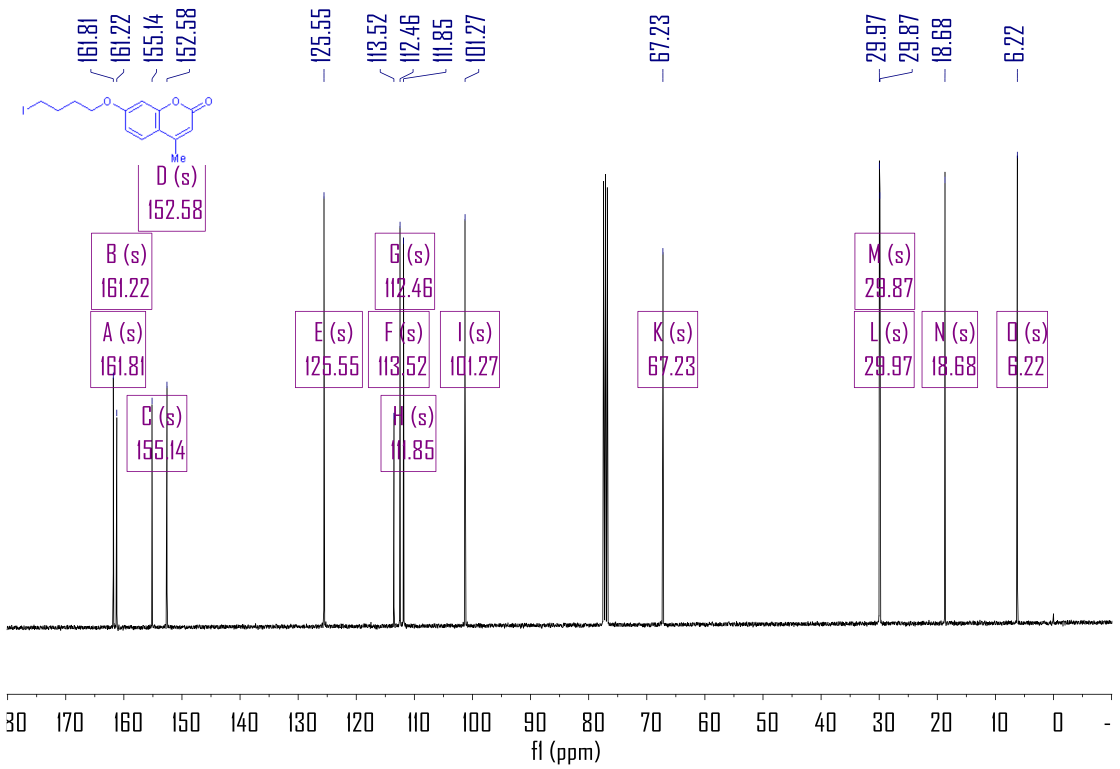

Supplementary Figure 46. ^{13}C NMR spectra for **RM13**

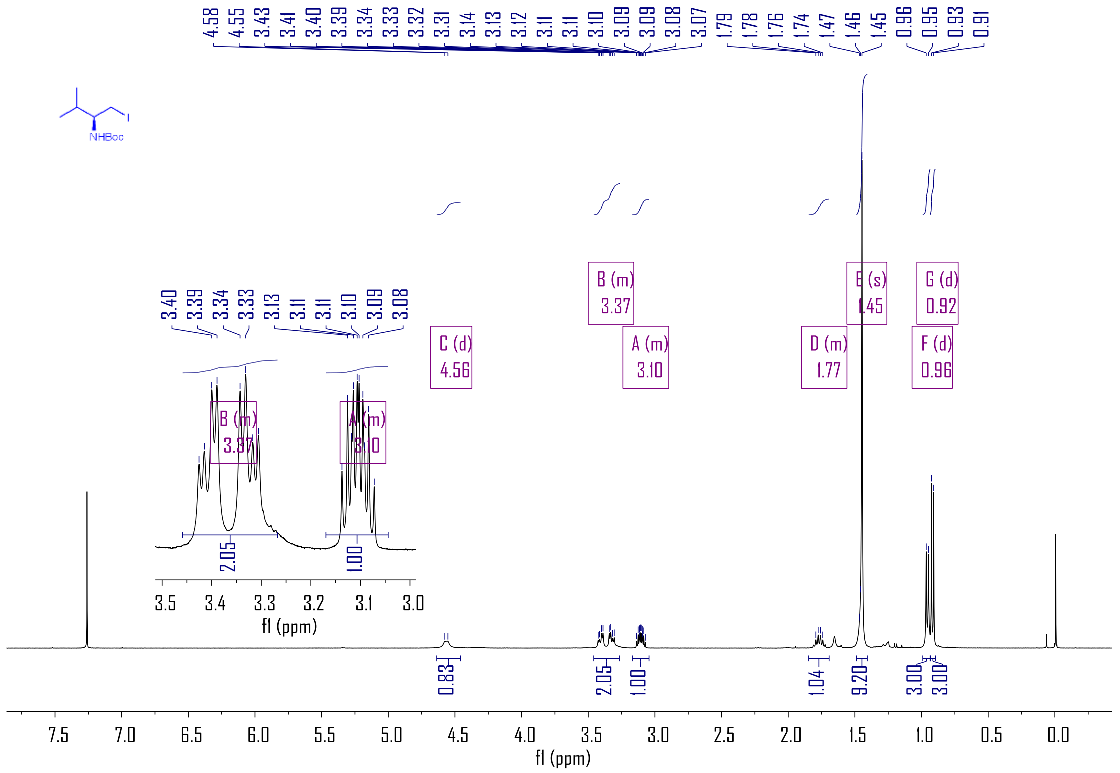

Supplementary Figure 47. ^{1}H NMR spectra for **RM14**

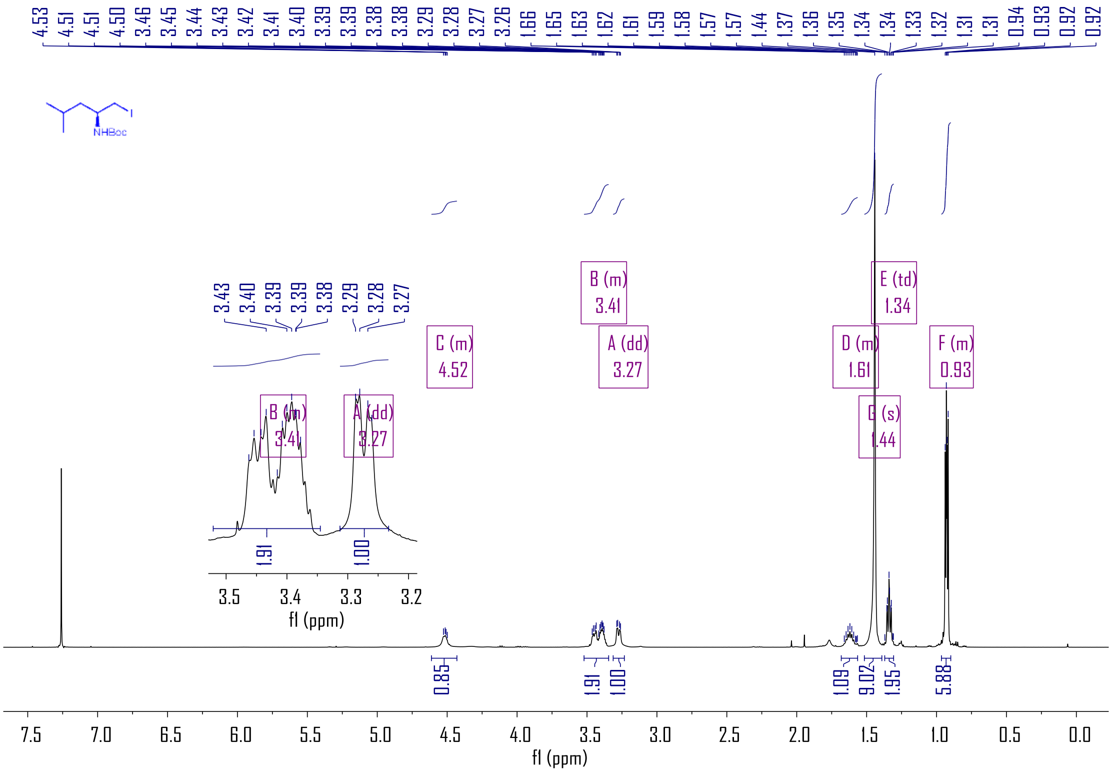

Supplementary Figure 48. ^{13}C NMR spectra for **RM15**

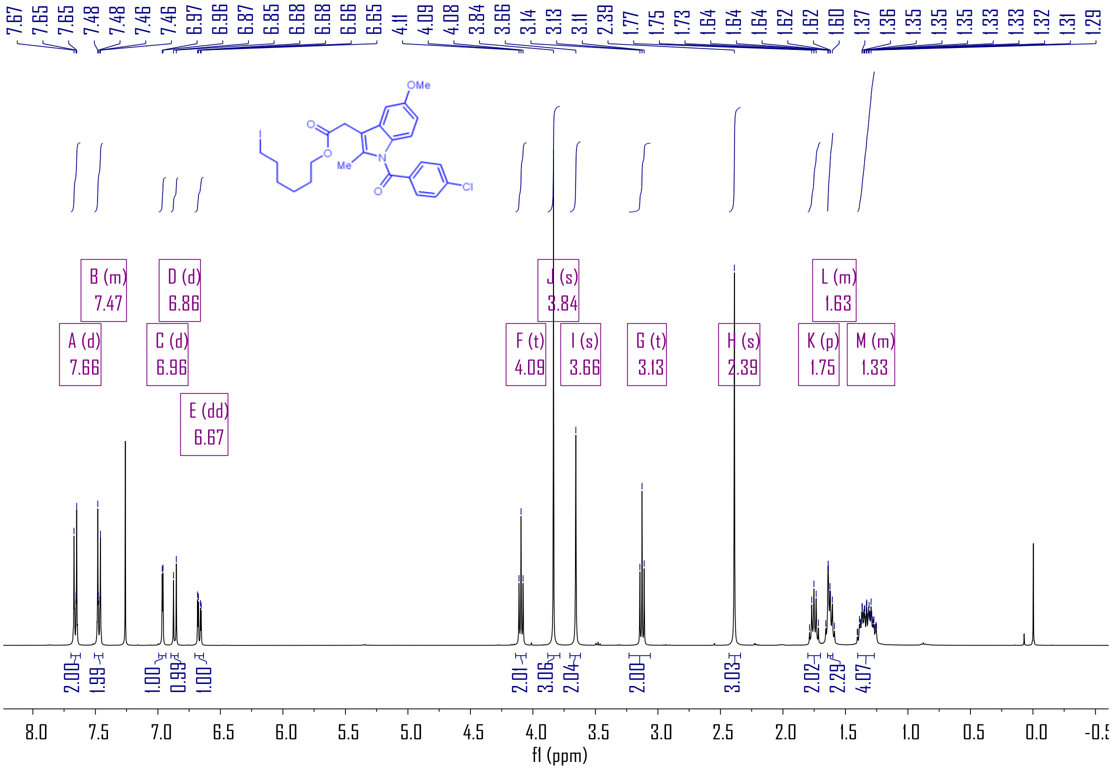

Supplementary Figure 49. ^{1}H NMR spectra for **RM16**

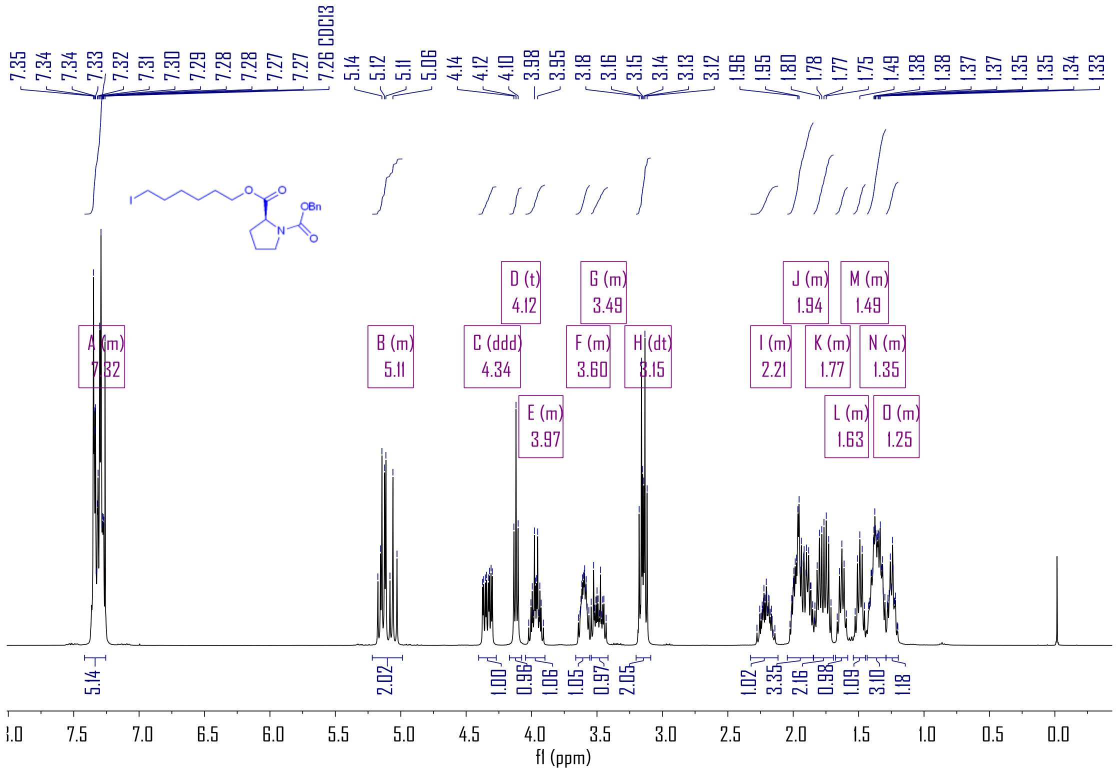

Supplementary Figure 50. ^{1}H NMR spectra for **RM17**

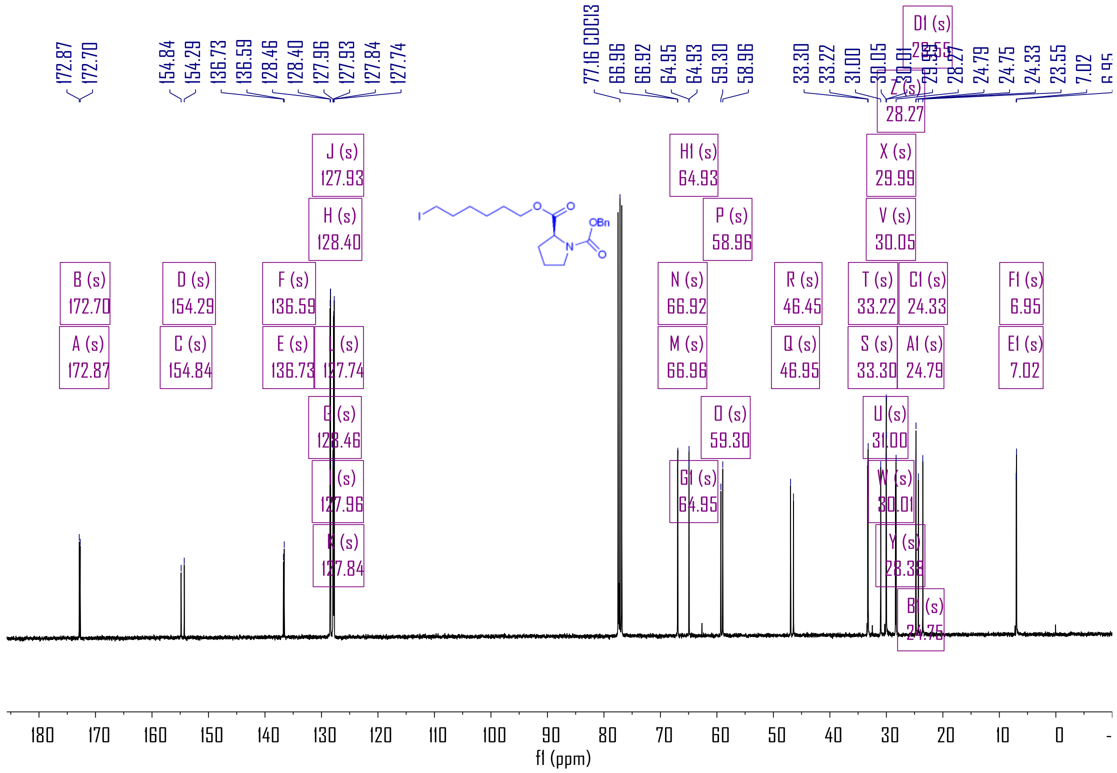

Supplementary Figure 51. ^{13}C NMR spectra for **RM17**

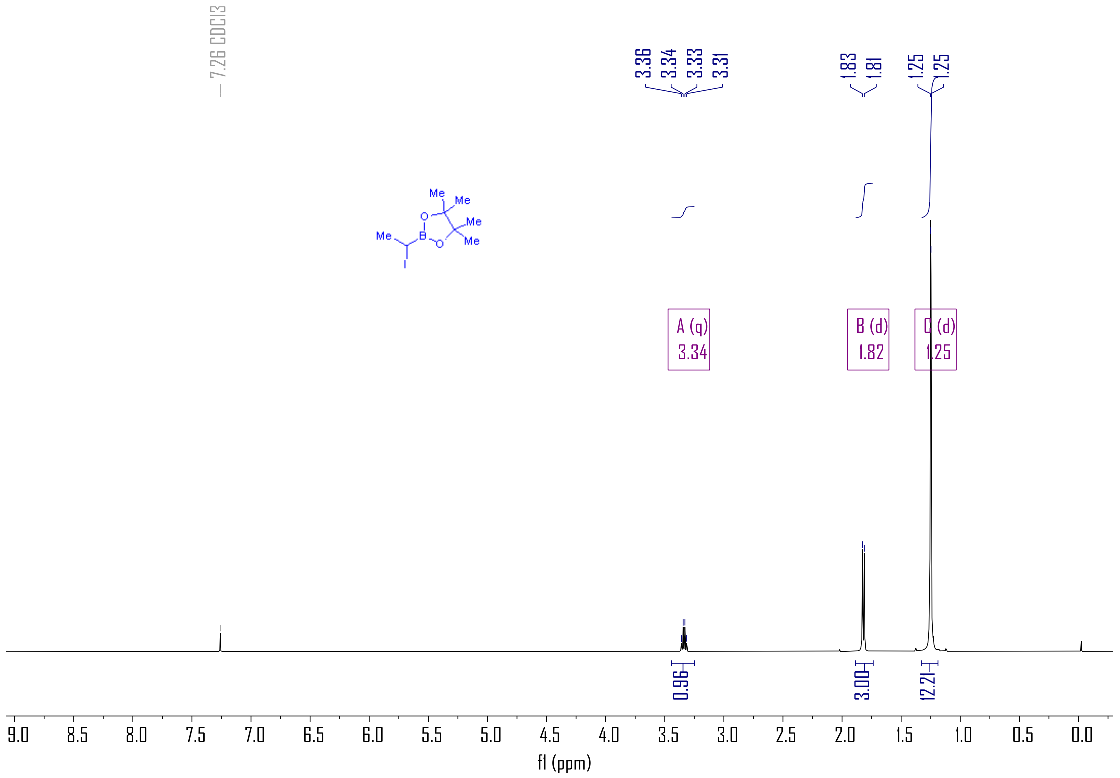

Supplementary Figure 52. ^{1}H NMR spectra for **RM18**

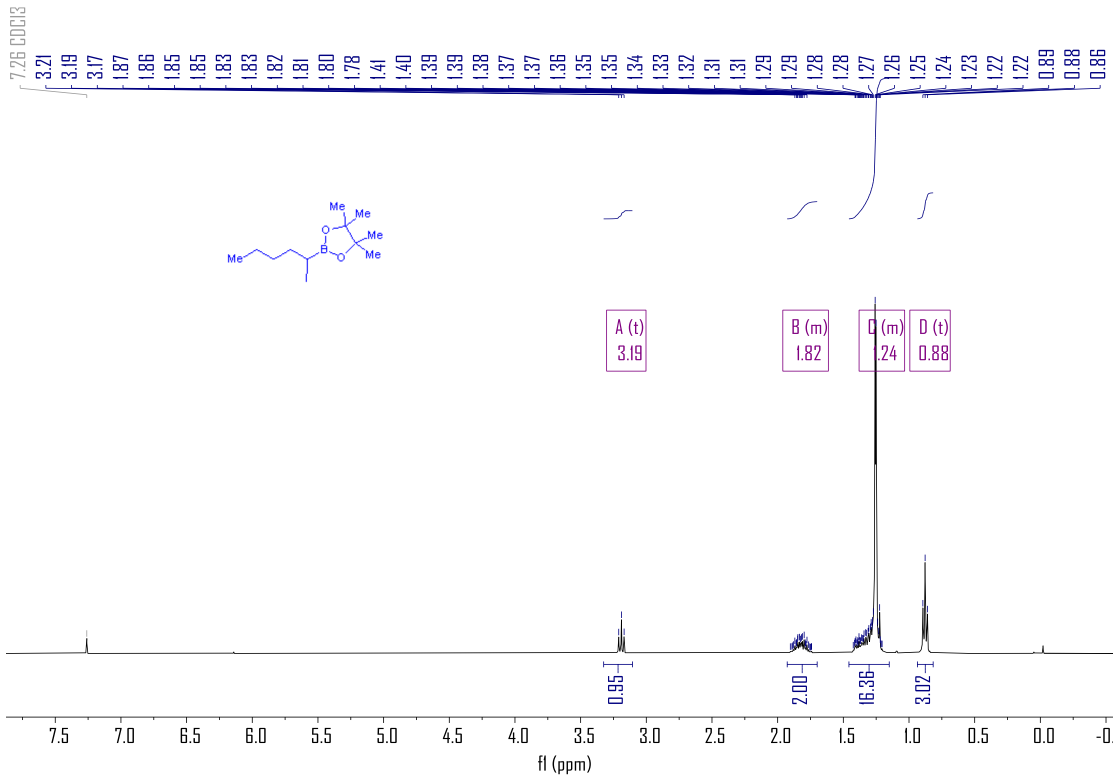

Supplementary Figure 53. ^{1}H NMR spectra for **RM19**

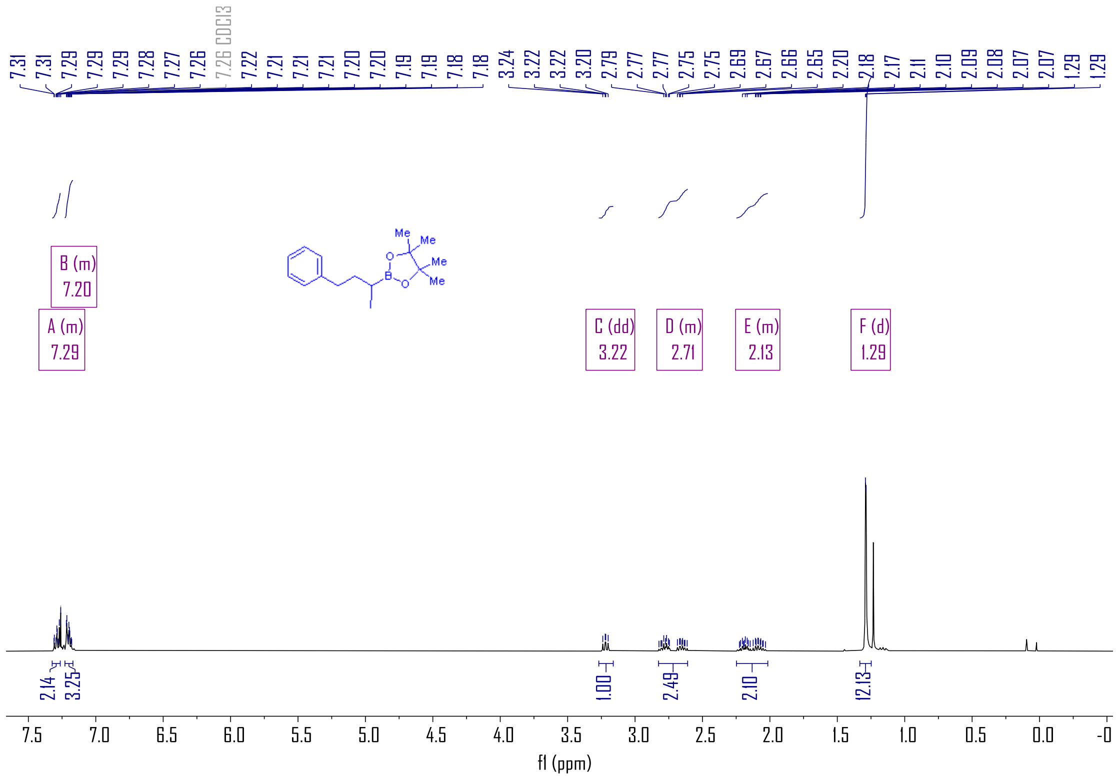

Supplementary Figure 54. ^{1}H NMR spectra for **RM20**

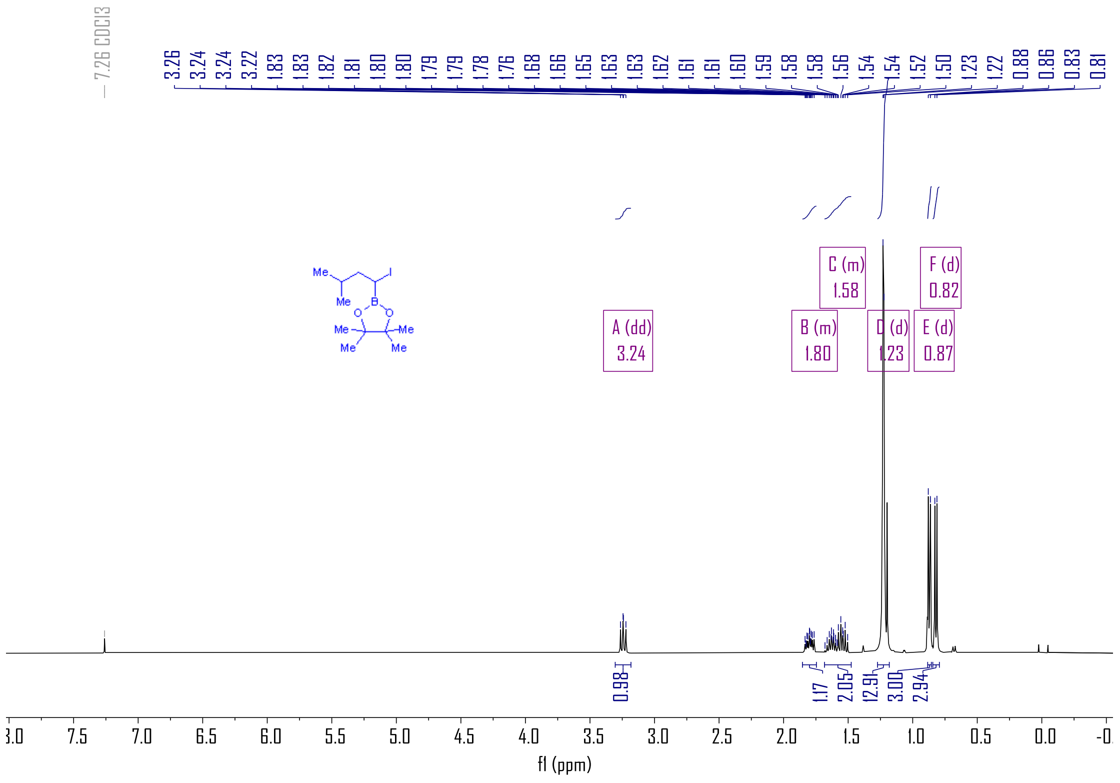

Supplementary Figure 55. ^{1}H NMR spectra for **RM21**

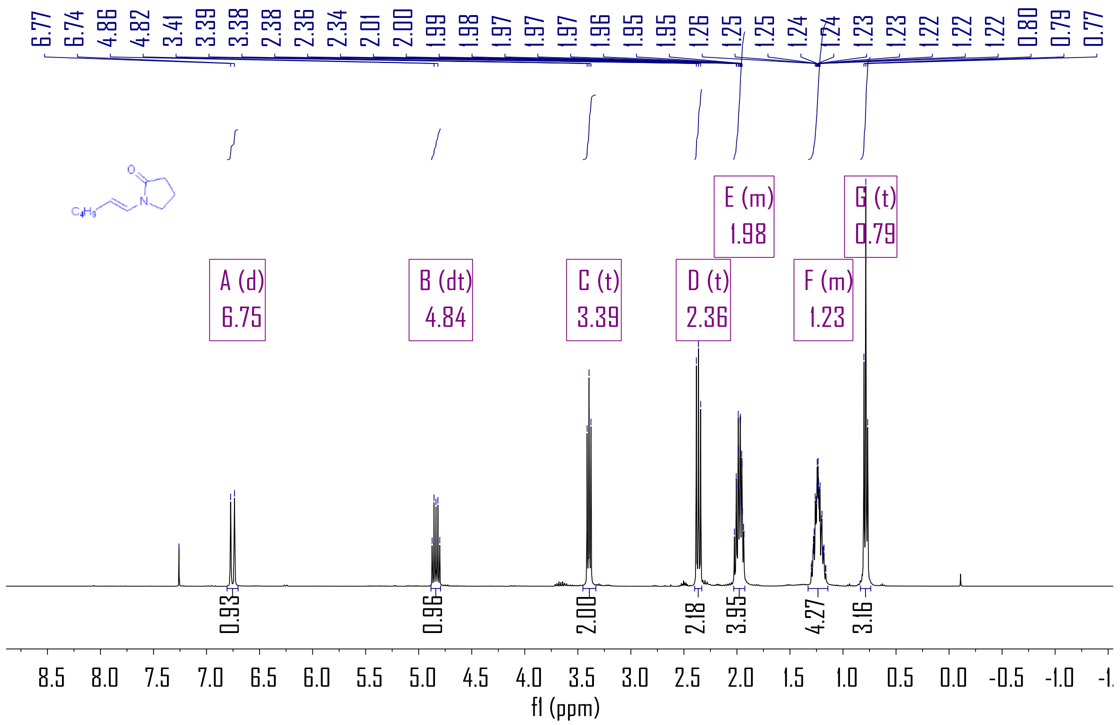

Supplementary Figure 56. ^{1}H NMR spectra for **RM22**

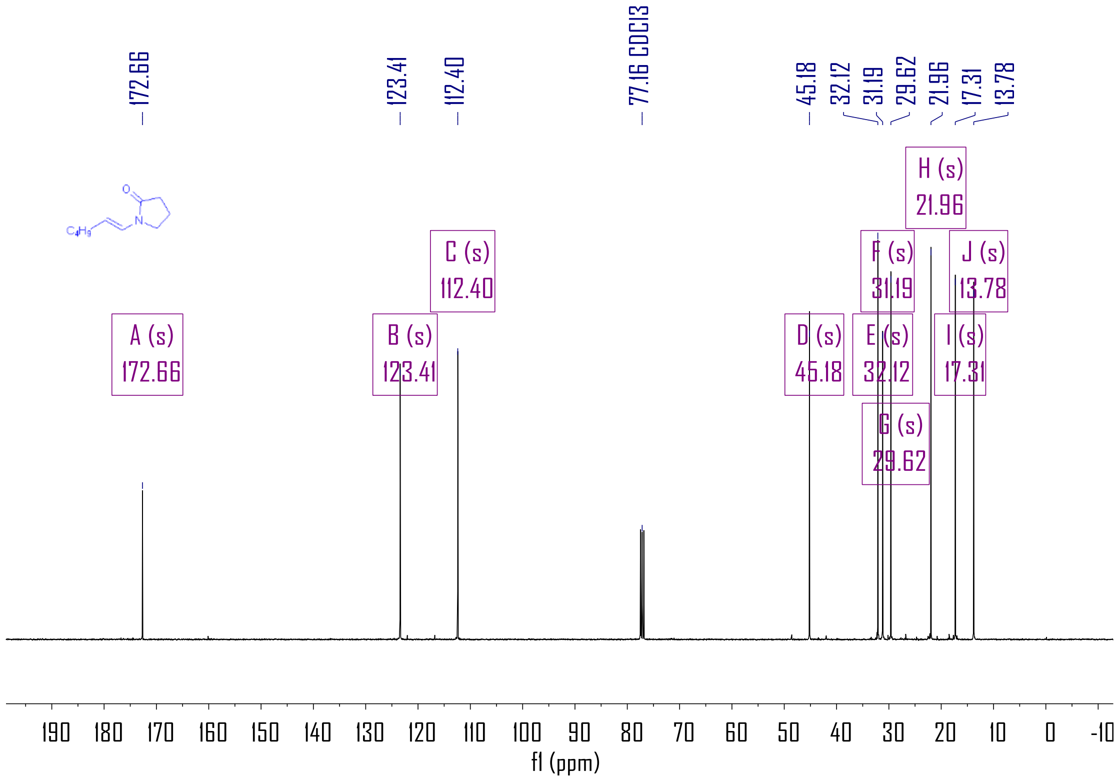

Supplementary Figure 57. ^{13}C NMR spectra for **RM22**

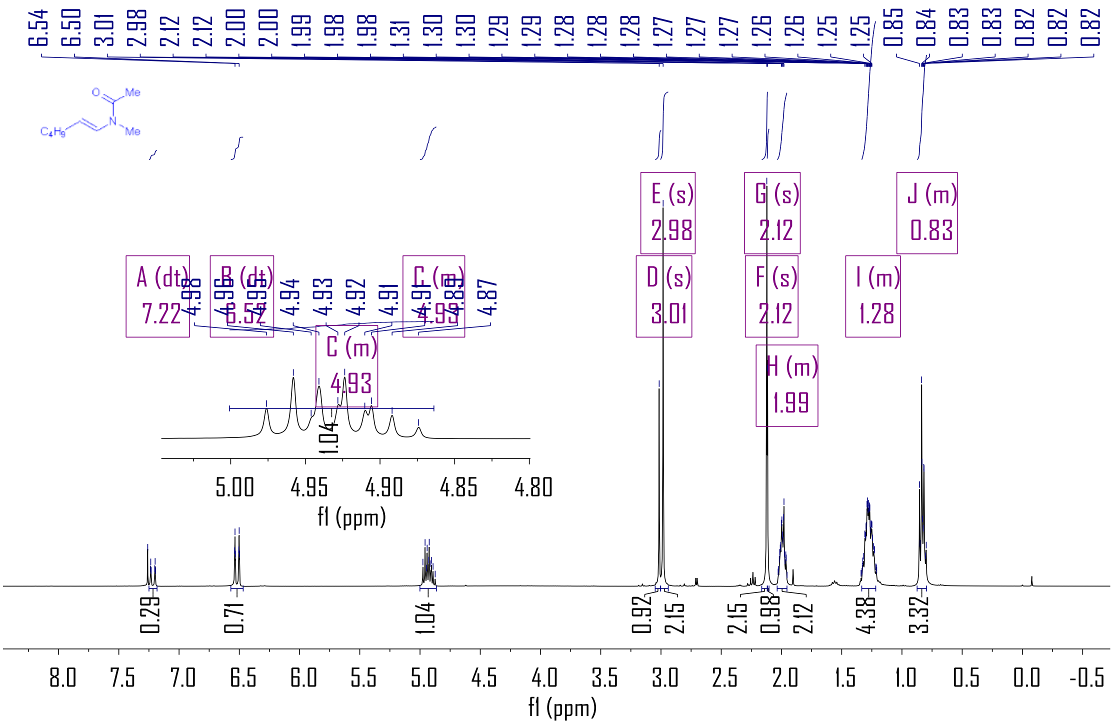

Supplementary Figure 58. ^{1}H NMR spectra for **RM23**

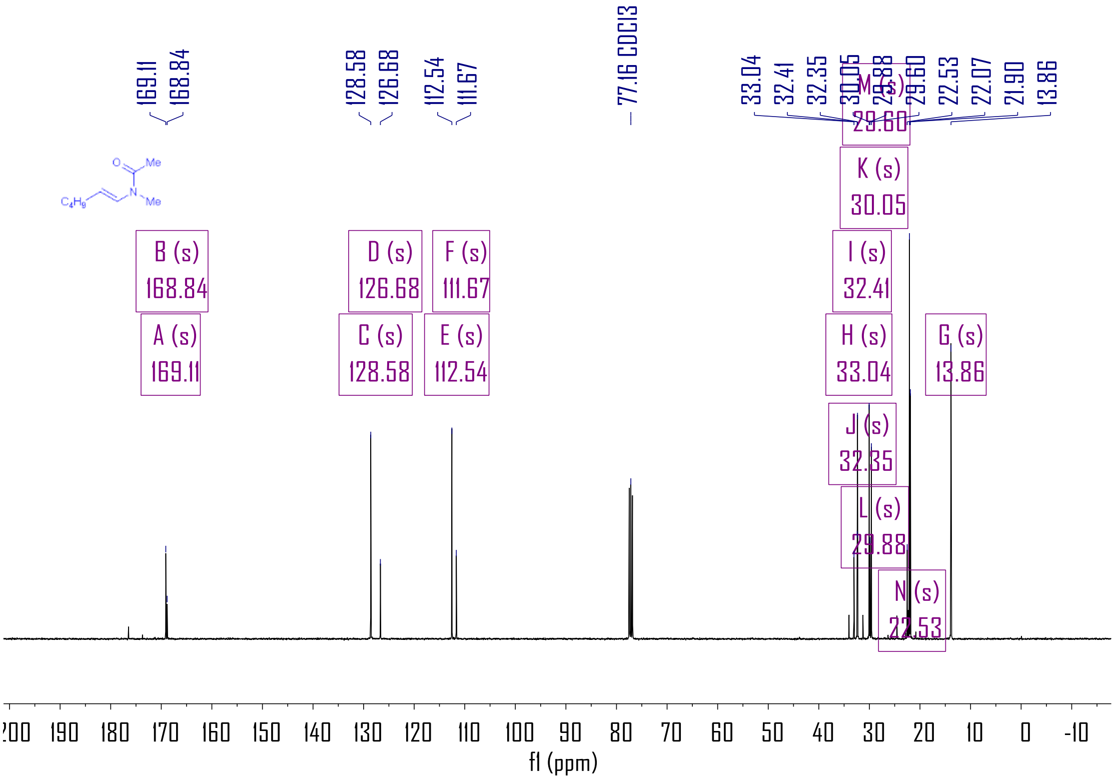

Supplementary Figure 59. ^{13}C NMR spectra for **RM23**

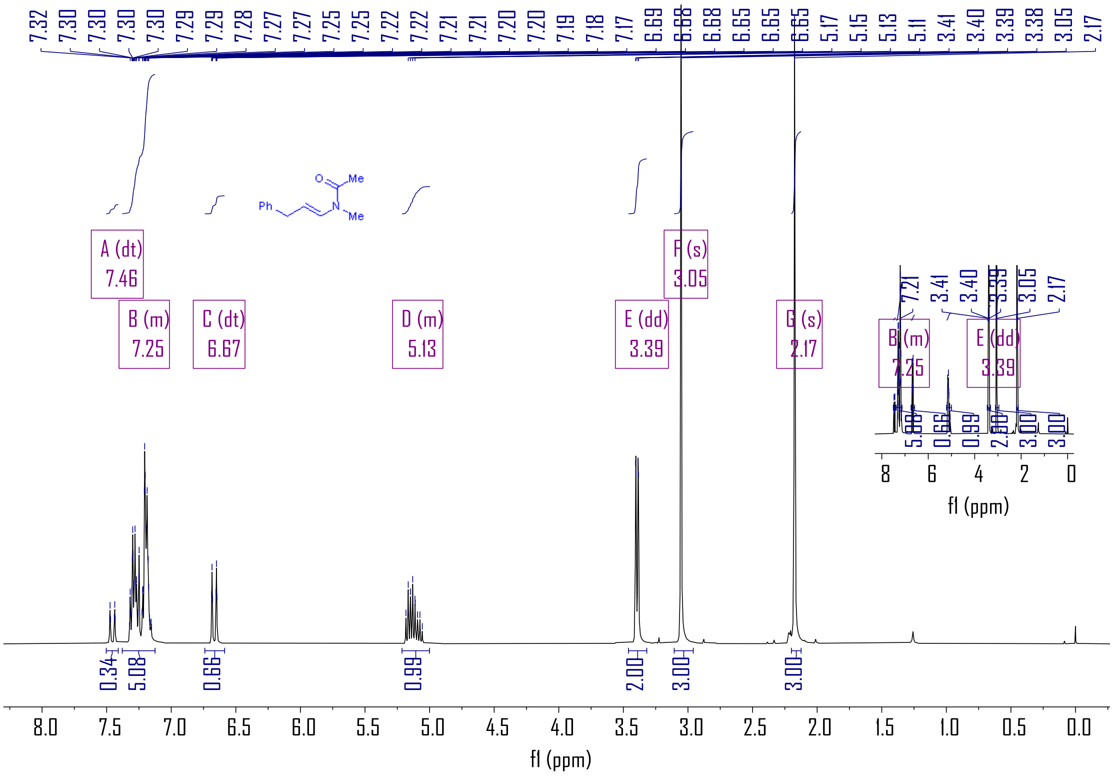

Supplementary Figure 60. ^{1}H NMR spectra for **RM24**

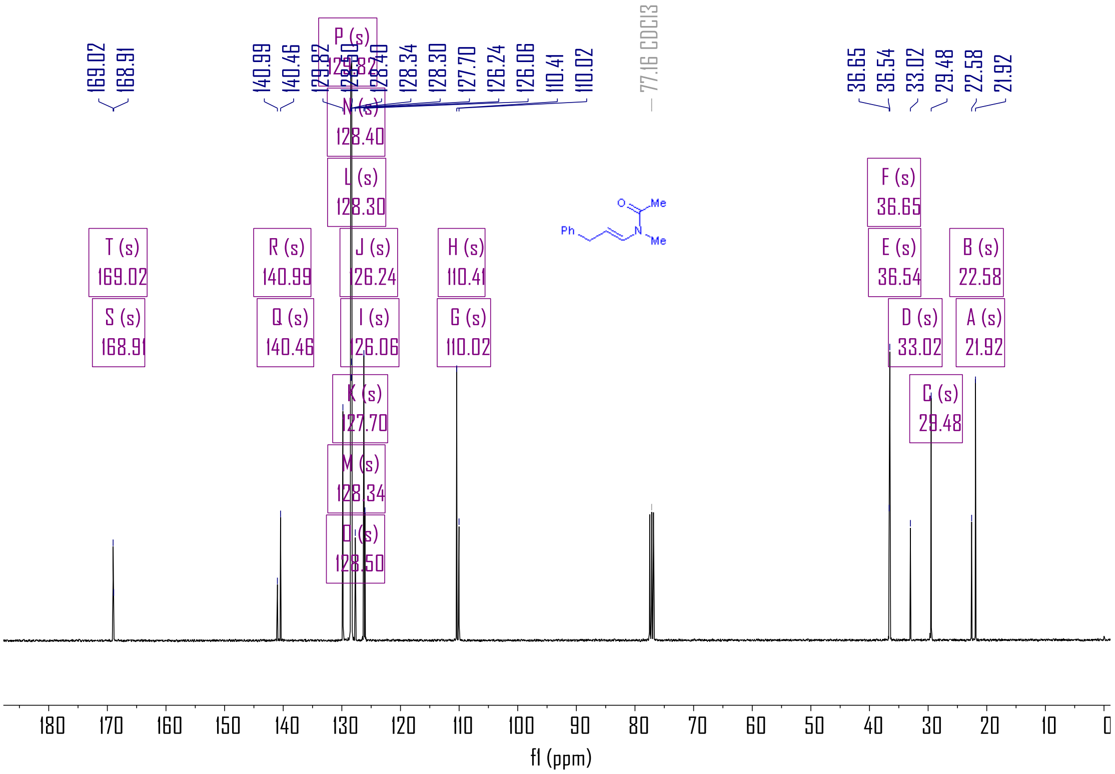

Supplementary Figure 61. ^{13}C NMR spectra for **RM24**

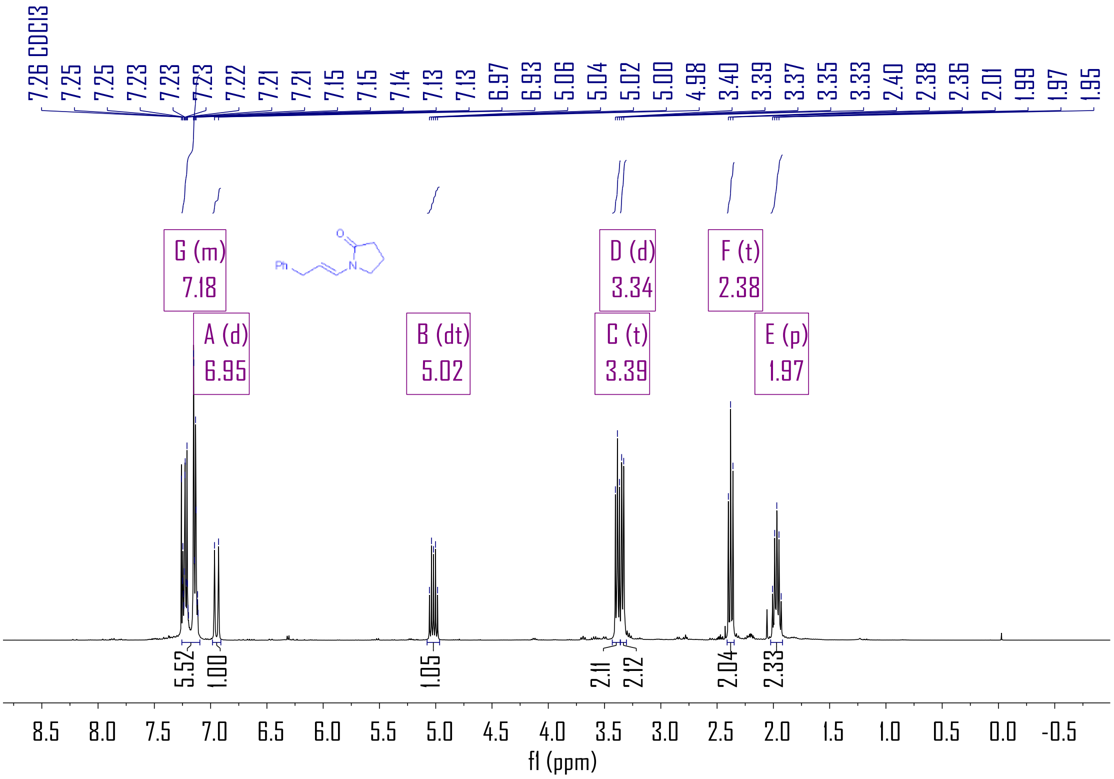

Supplementary Figure 62. ^{1}H NMR spectra for **RM25**

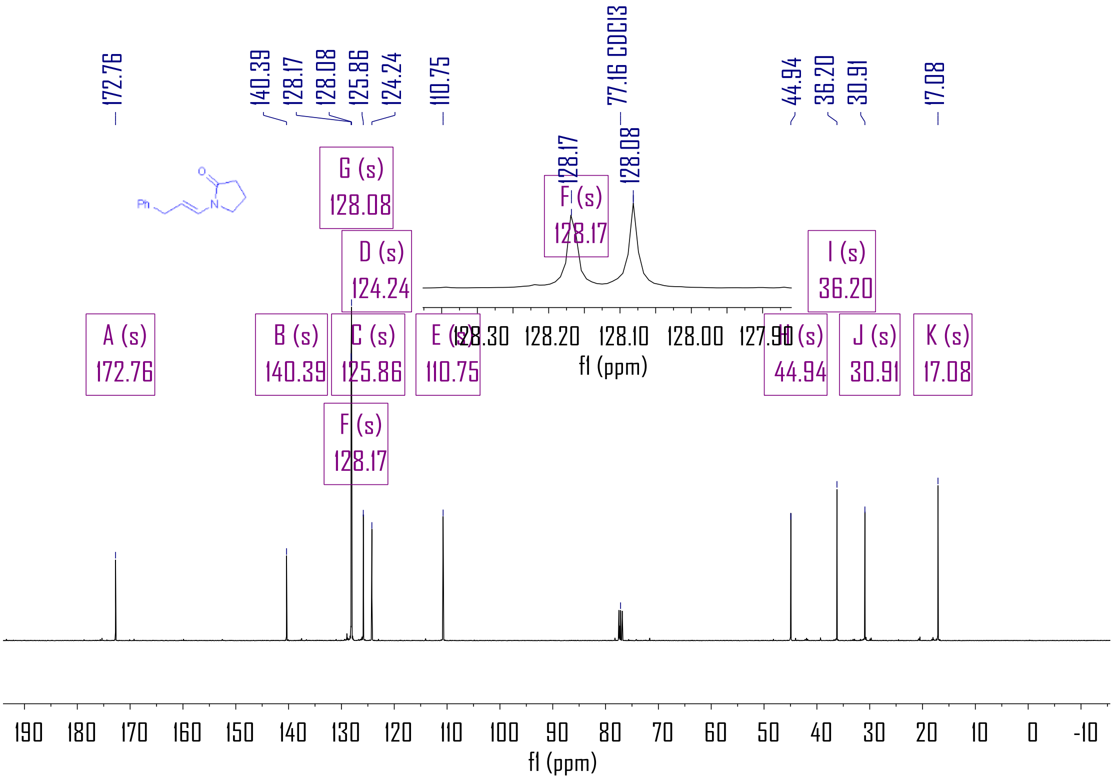

Supplementary Figure 63. ^{13}C NMR spectra for **RM25**

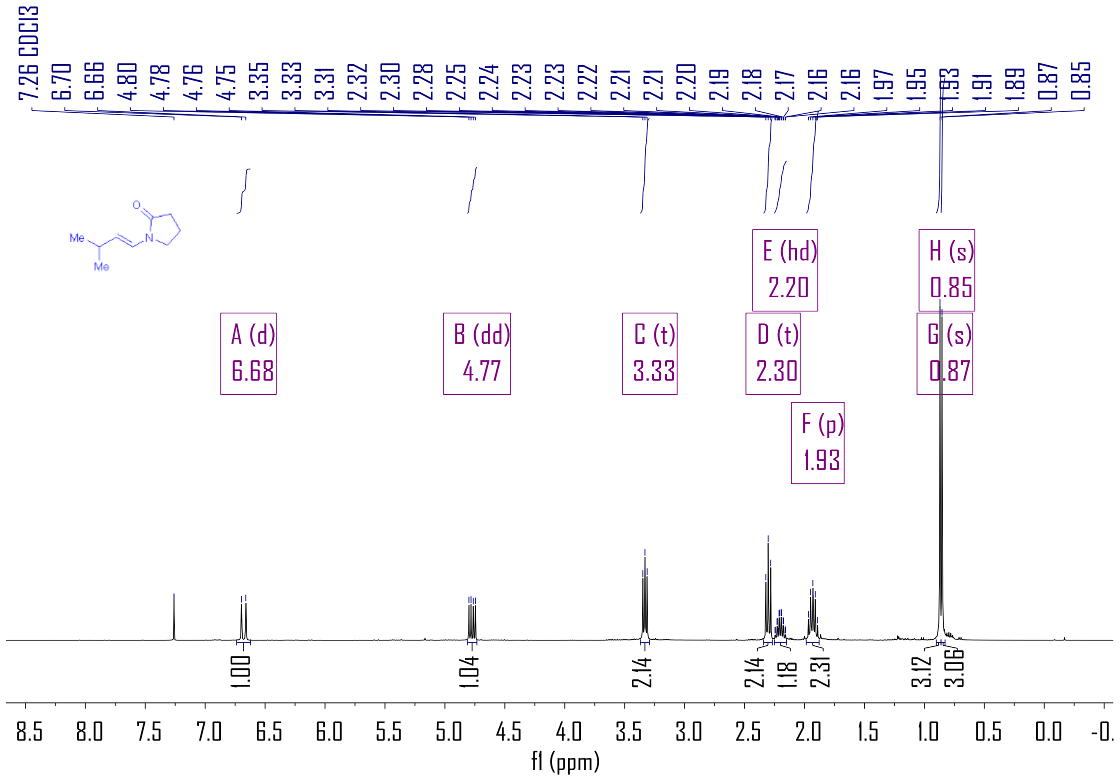

Supplementary Figure 64. ^{1}H NMR spectra for **RM26**

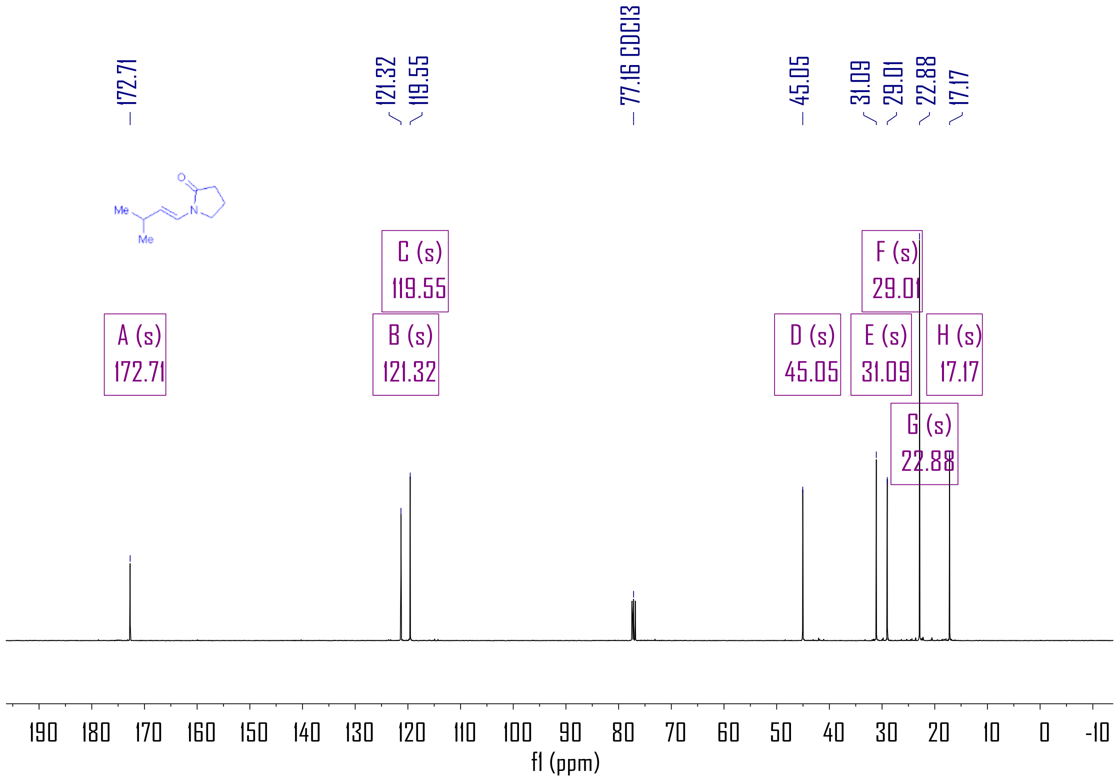

Supplementary Figure 65. ^{13}C NMR spectra for **RM26**

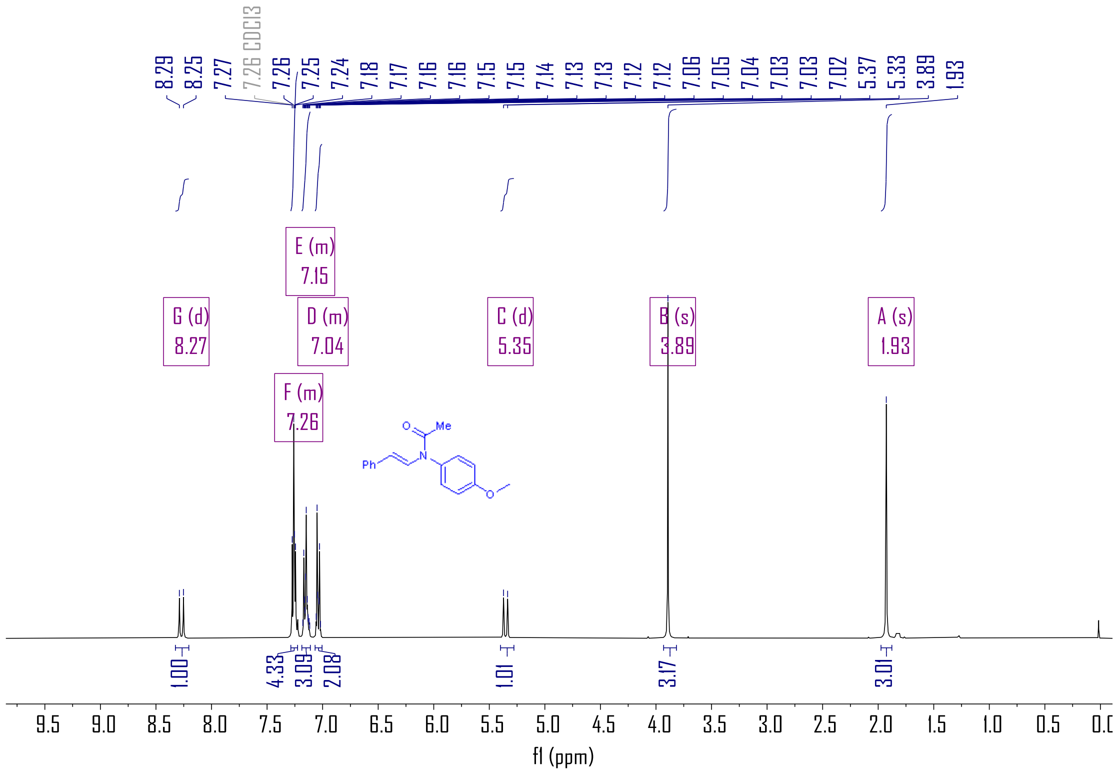

Supplementary Figure 66. ^{1}H NMR spectra for **RM27**

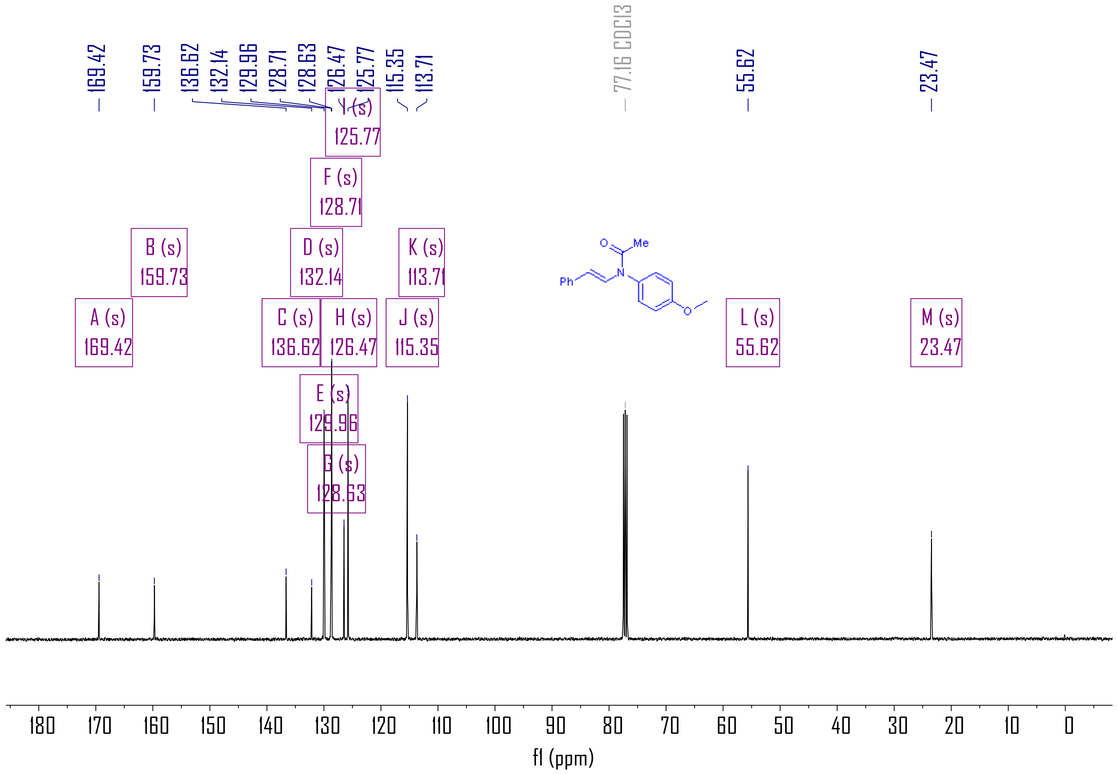

Supplementary Figure 67. ^{13}C NMR spectra for **RM27**

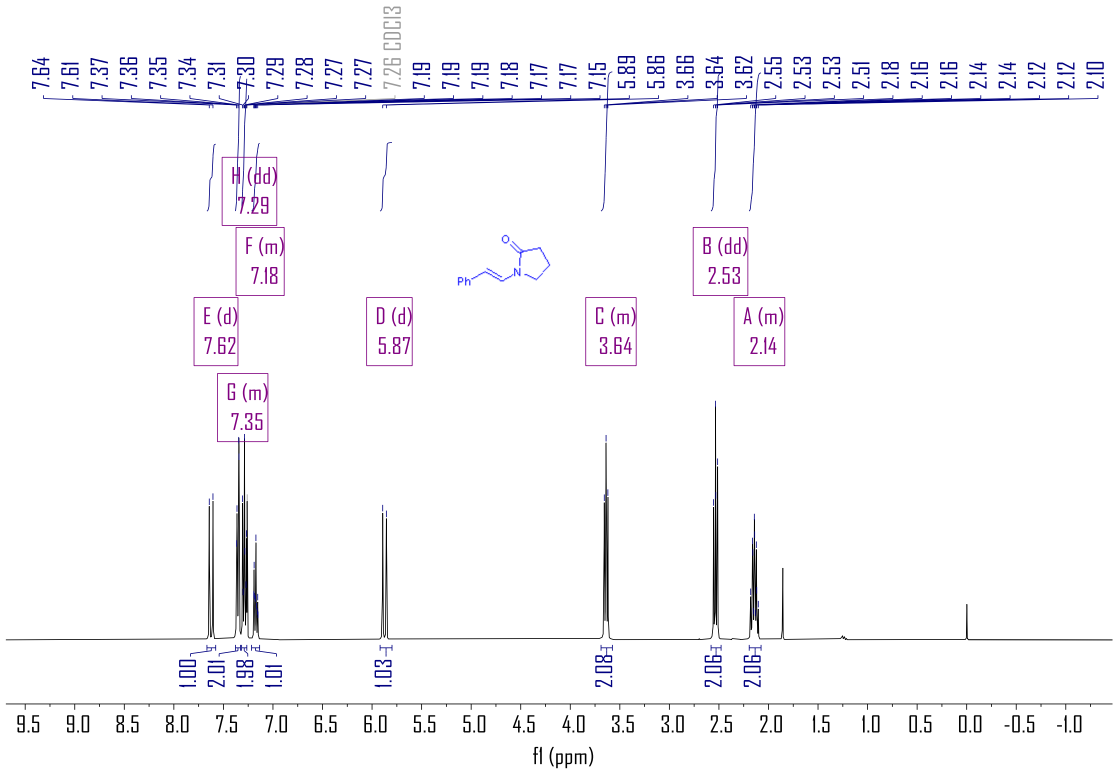

Supplementary Figure 68. ^{1}H NMR spectra for **RM28**

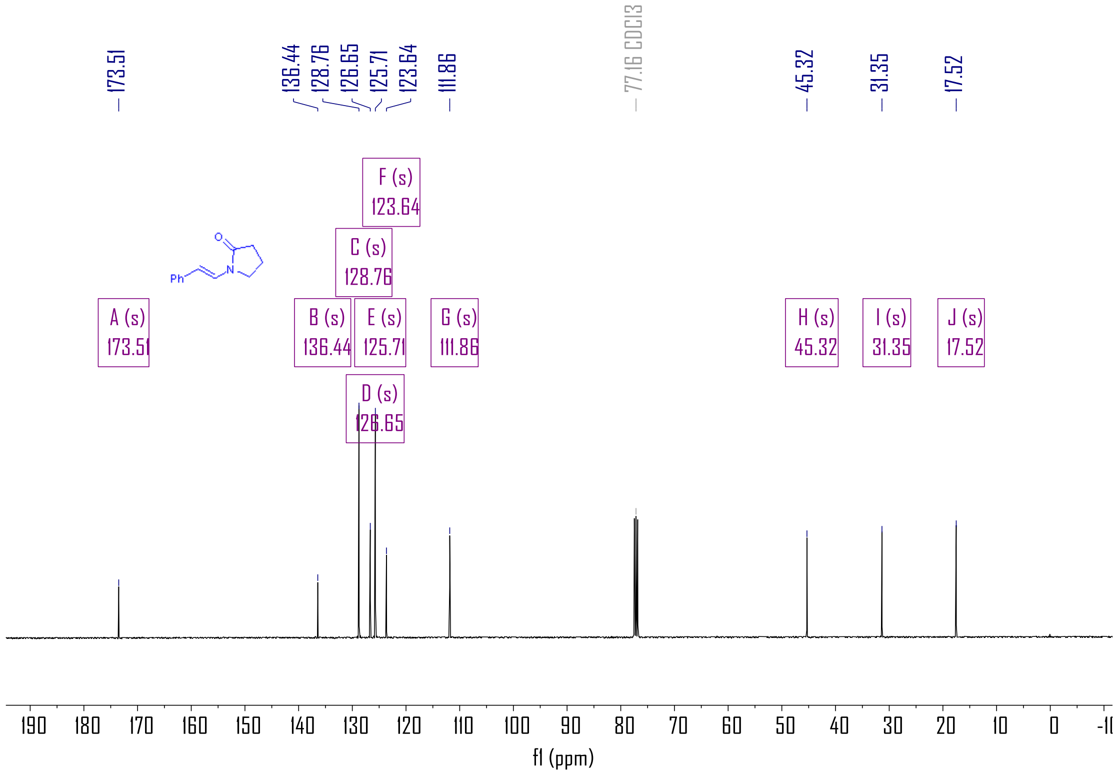

Supplementary Figure 69. ^{13}C NMR spectra for **RM28**

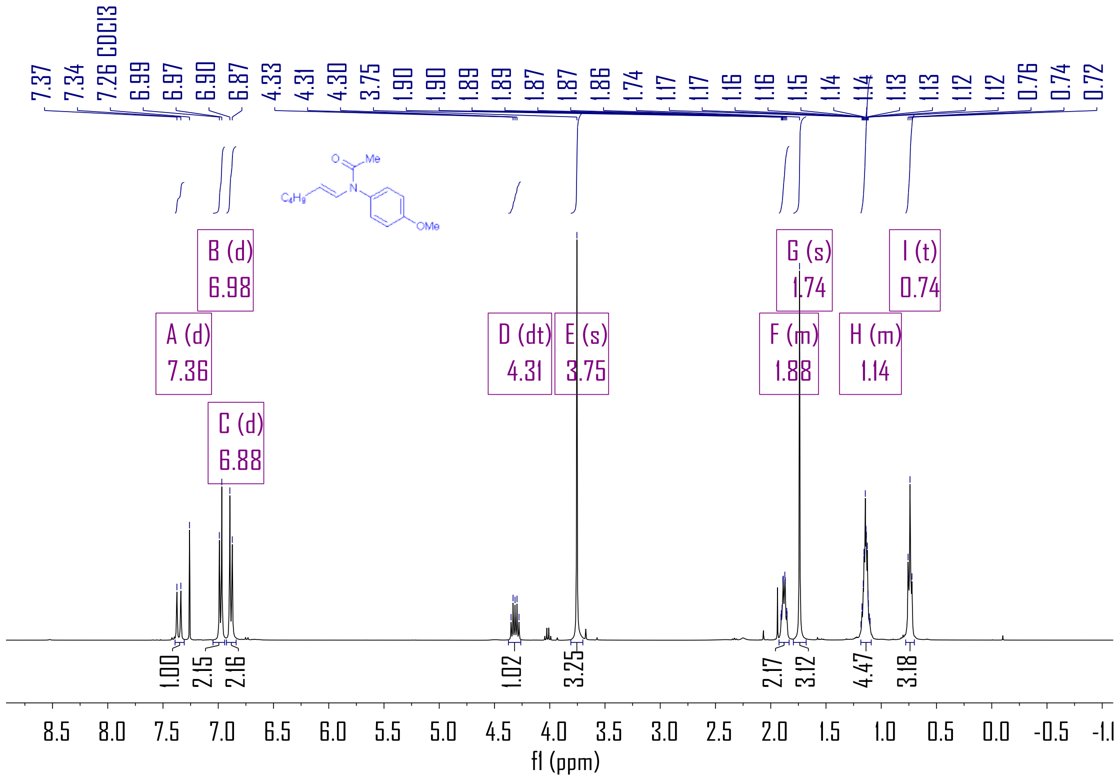

Supplementary Figure 70. ^{1}H NMR spectra for **RM29**

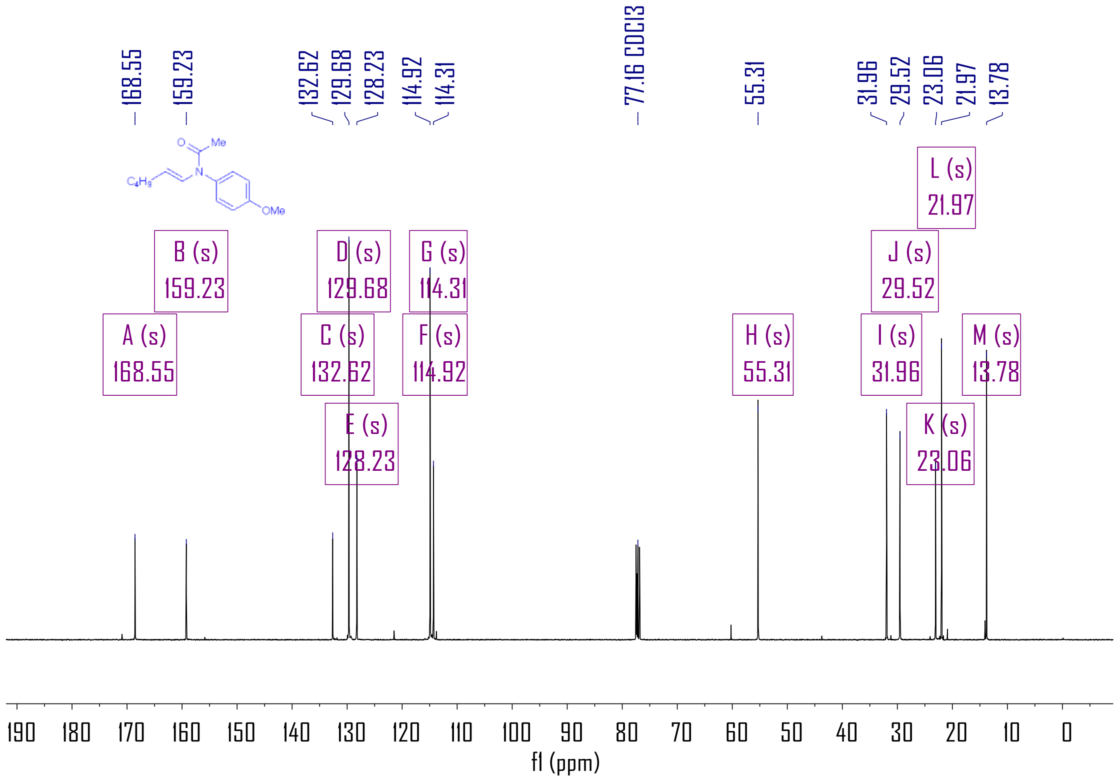

Supplementary Figure 71. ^{13}C NMR spectra for **RM29**

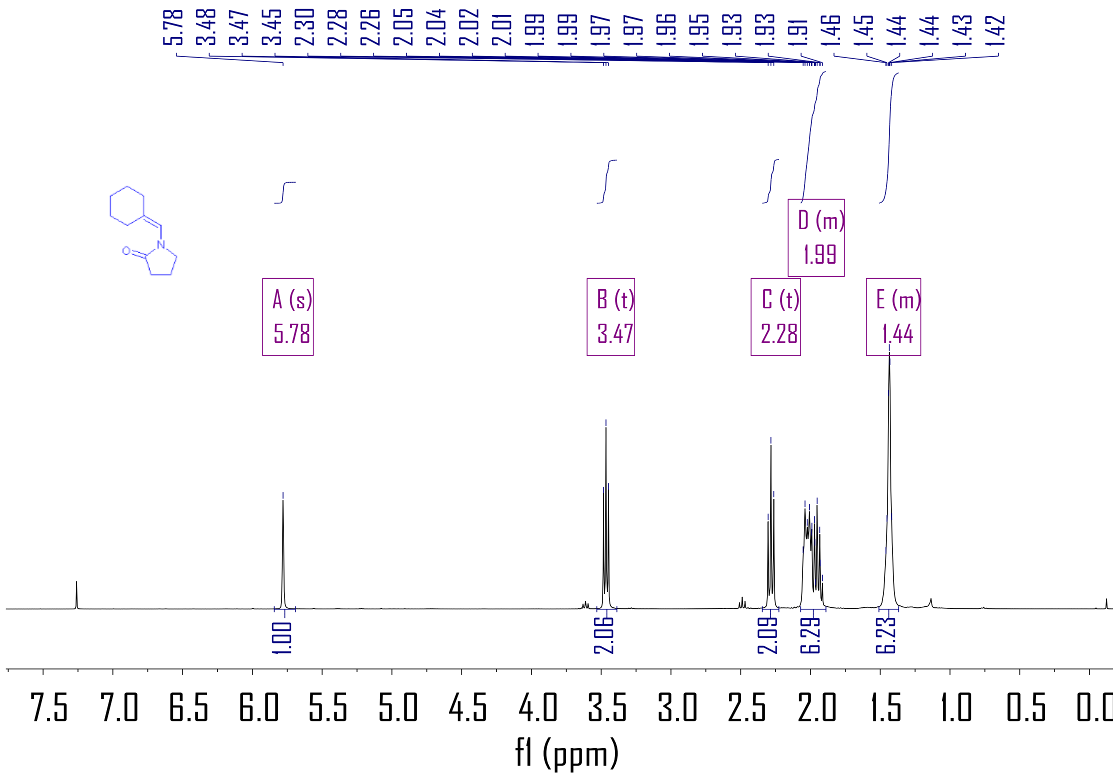

Supplementary Figure 72. ^{1}H NMR spectra for **RM30**

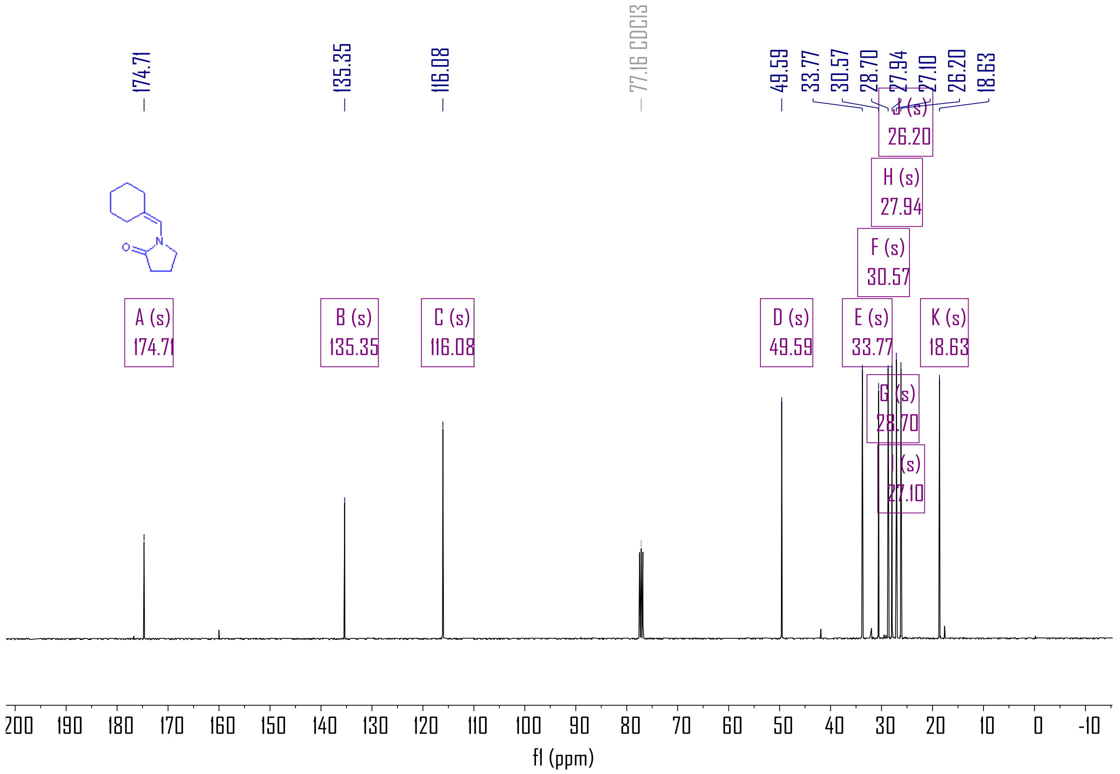

Supplementary Figure 73. ^{13}C NMR spectra for **RM30**

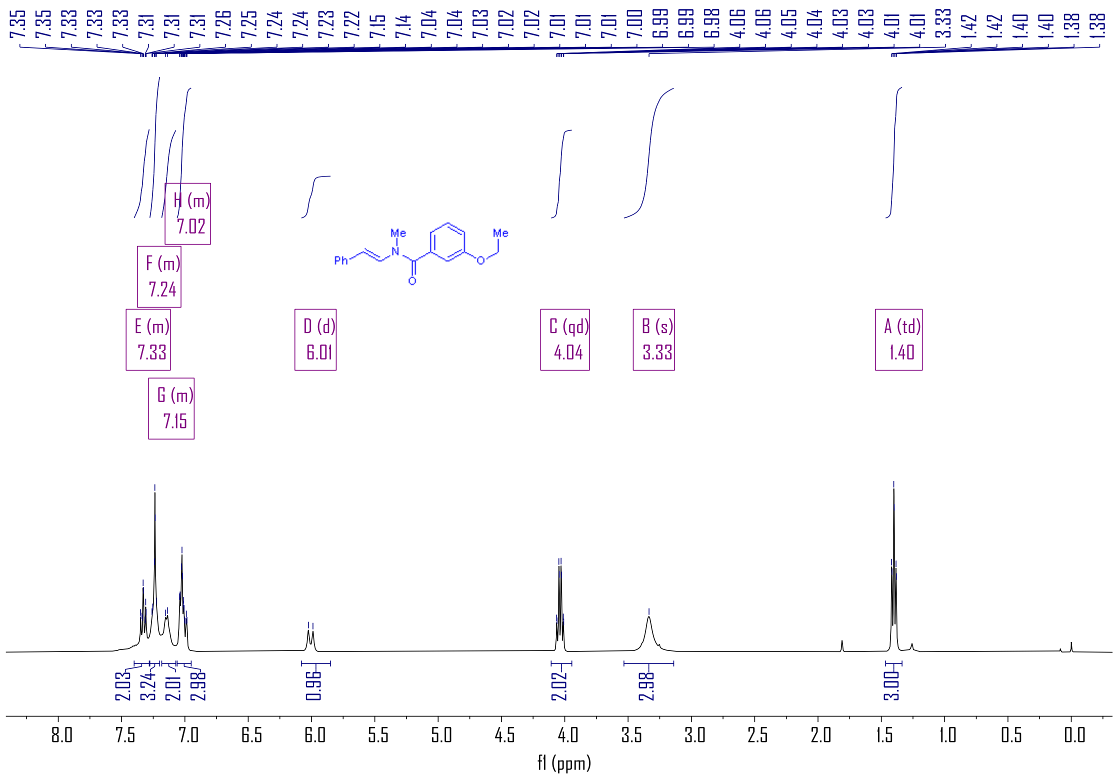

Supplementary Figure 74. ^{1}H NMR spectra for **RM31**

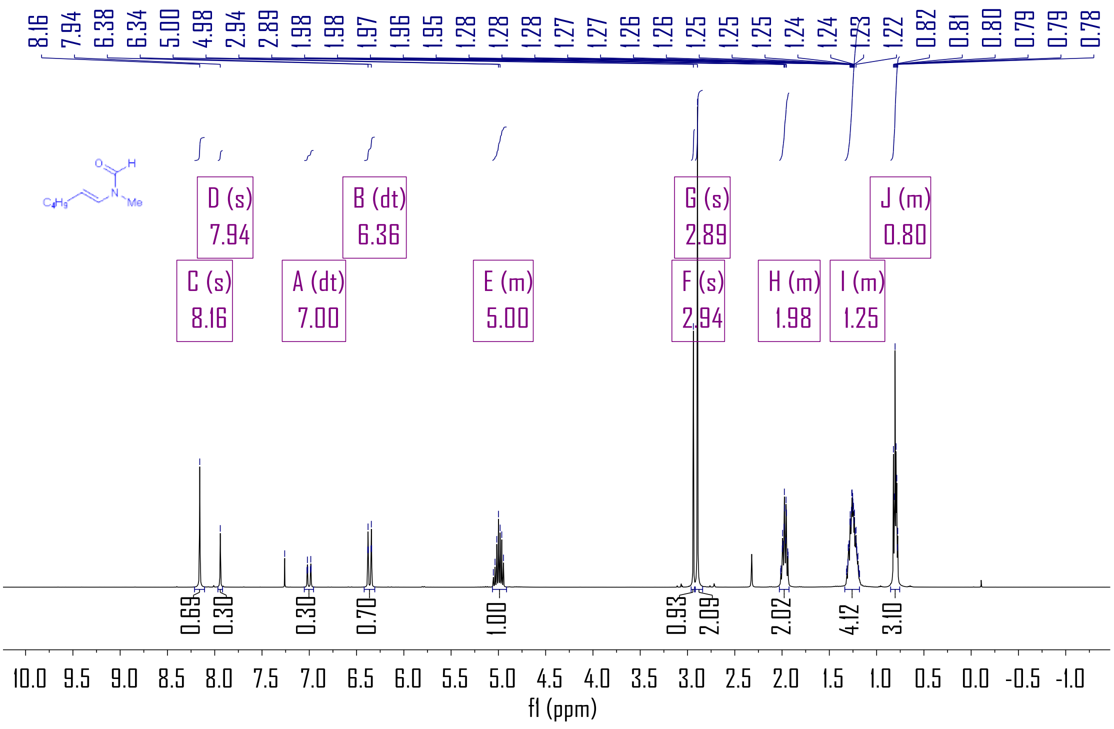

Supplementary Figure 75. ^{1}H NMR spectra for **RM32**

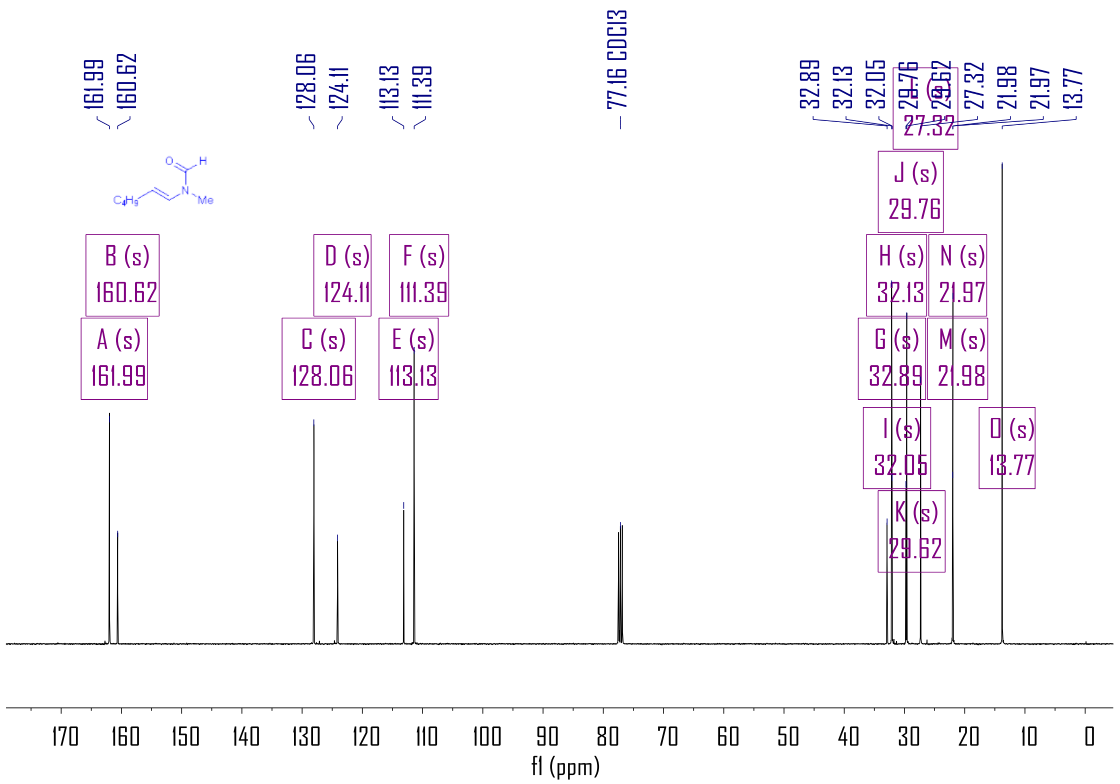

Supplementary Figure 76. ^{13}C NMR spectra for **RM32**

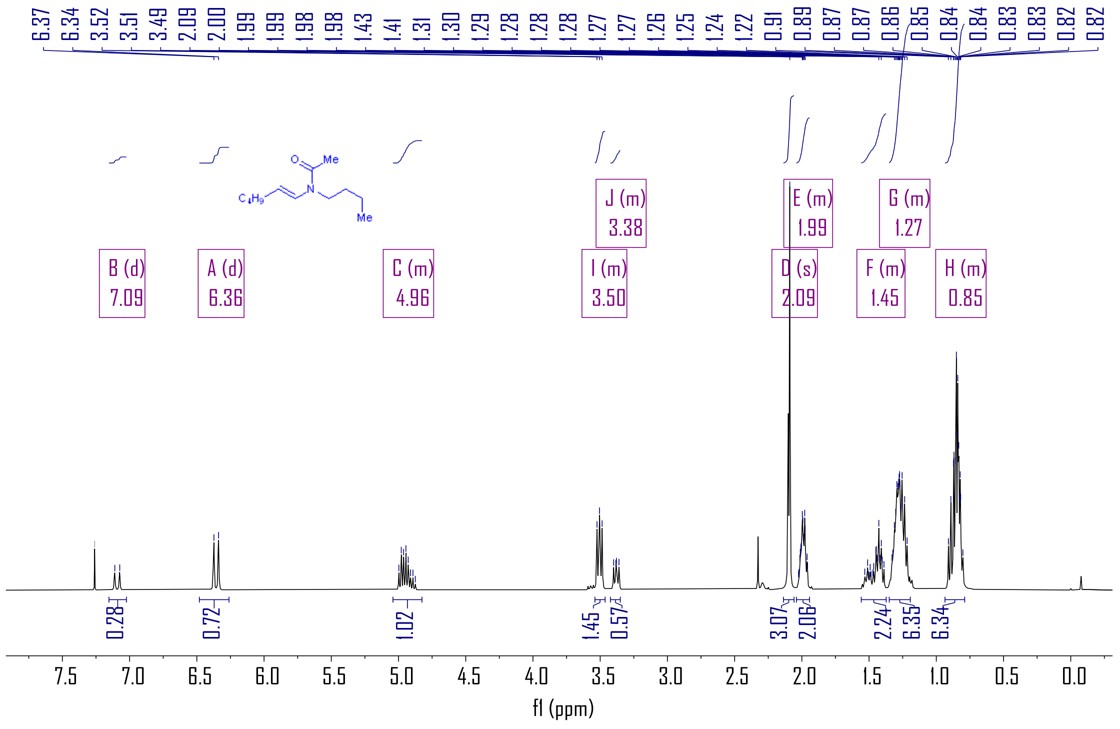

Supplementary Figure 77. ^{1}H NMR spectra for **RM33**

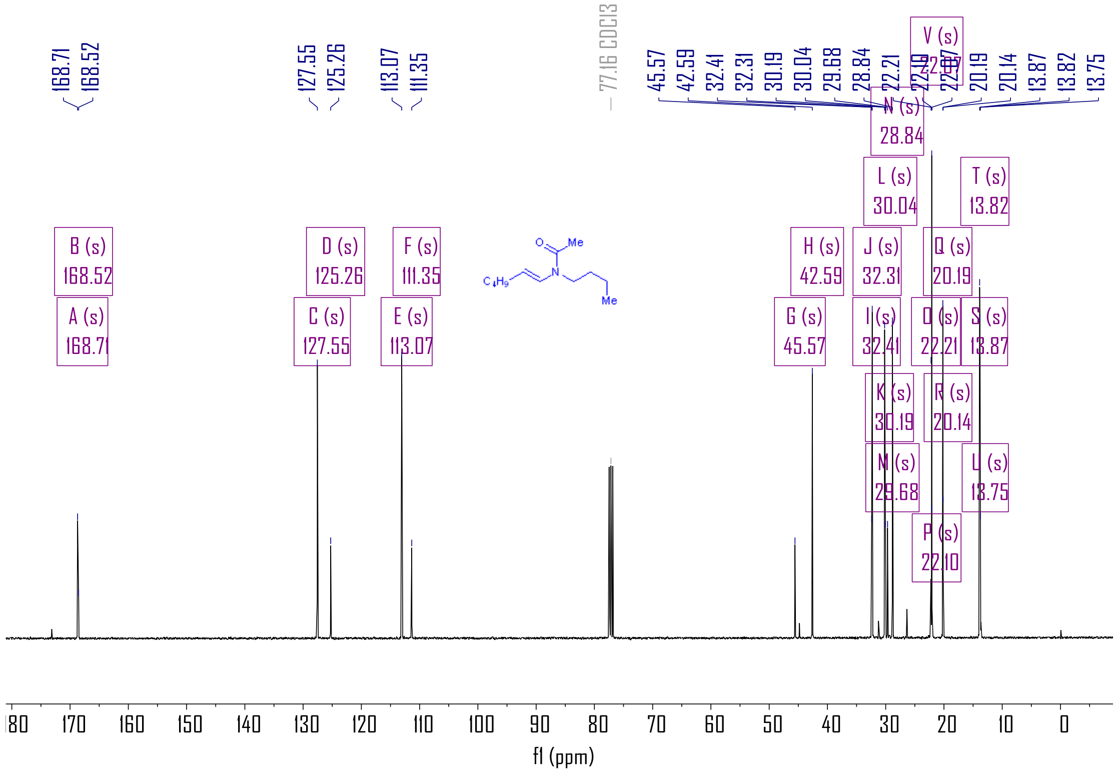

Supplementary Figure 78. ^{13}C NMR spectra for **RM33**

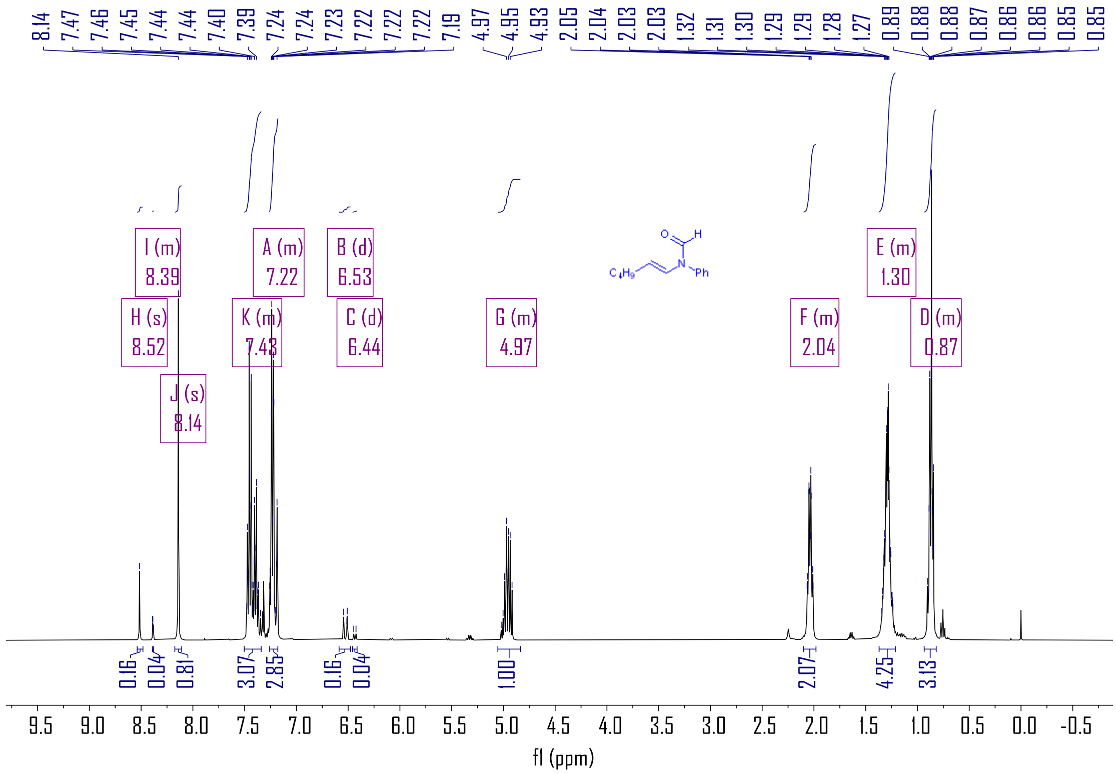

Supplementary Figure 79. ^{1}H NMR spectra for **RM34**

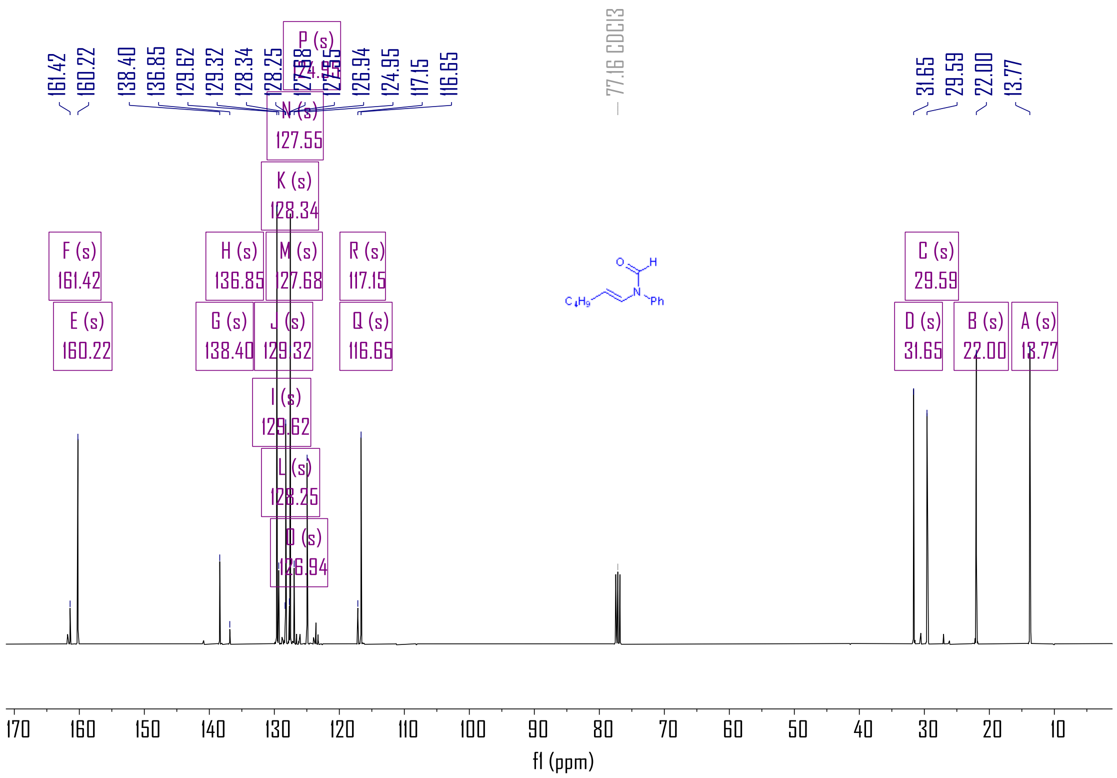

Supplementary Figure 80. ^{13}C NMR spectra for **RM34**

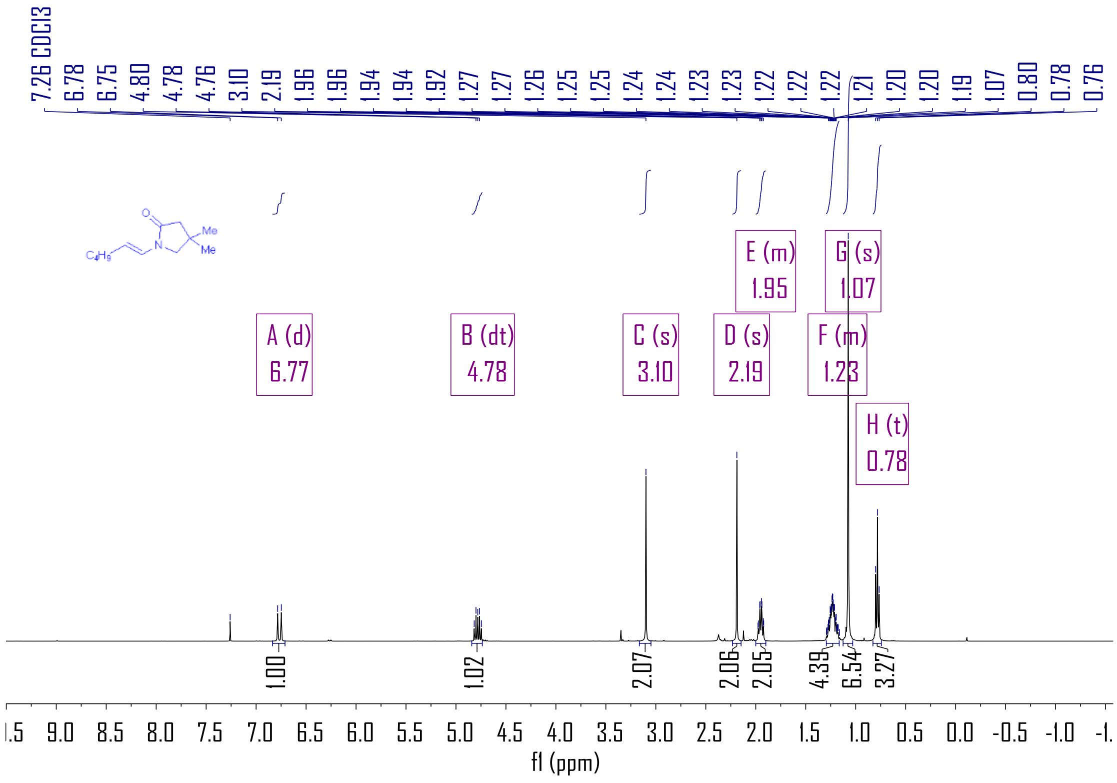

Supplementary Figure 81. ^{1}H NMR spectra for **RM35**

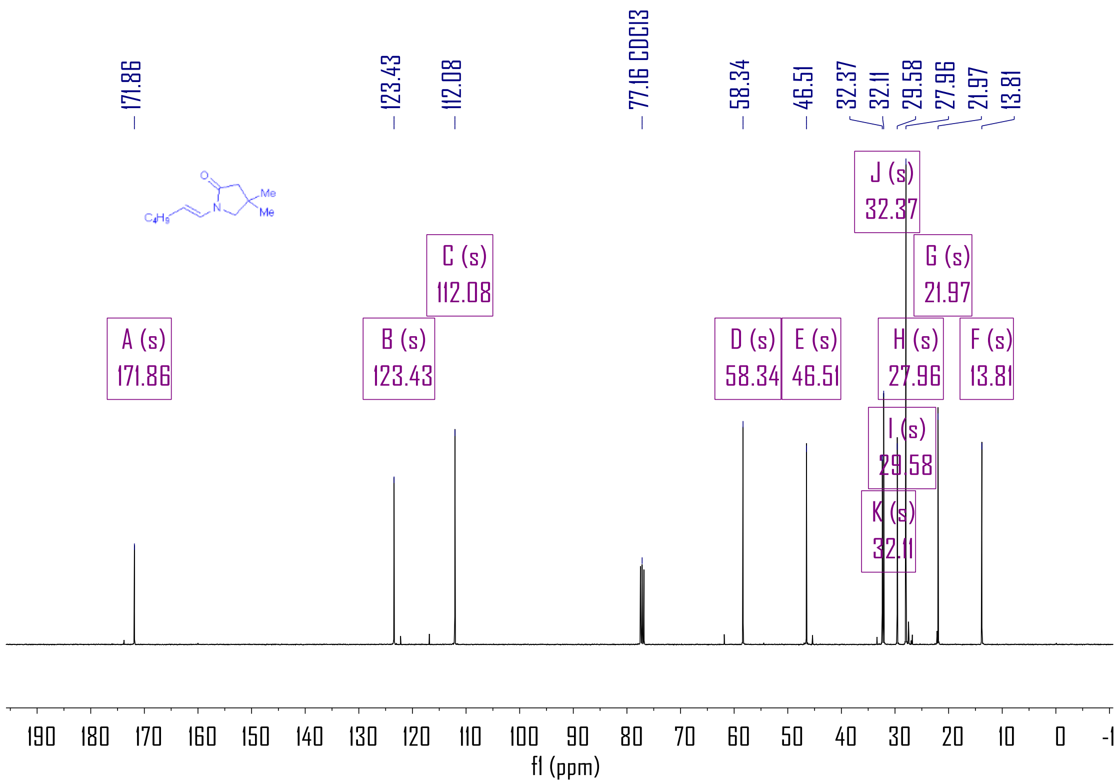

Supplementary Figure 82. ^{13}C NMR spectra for **RM35**

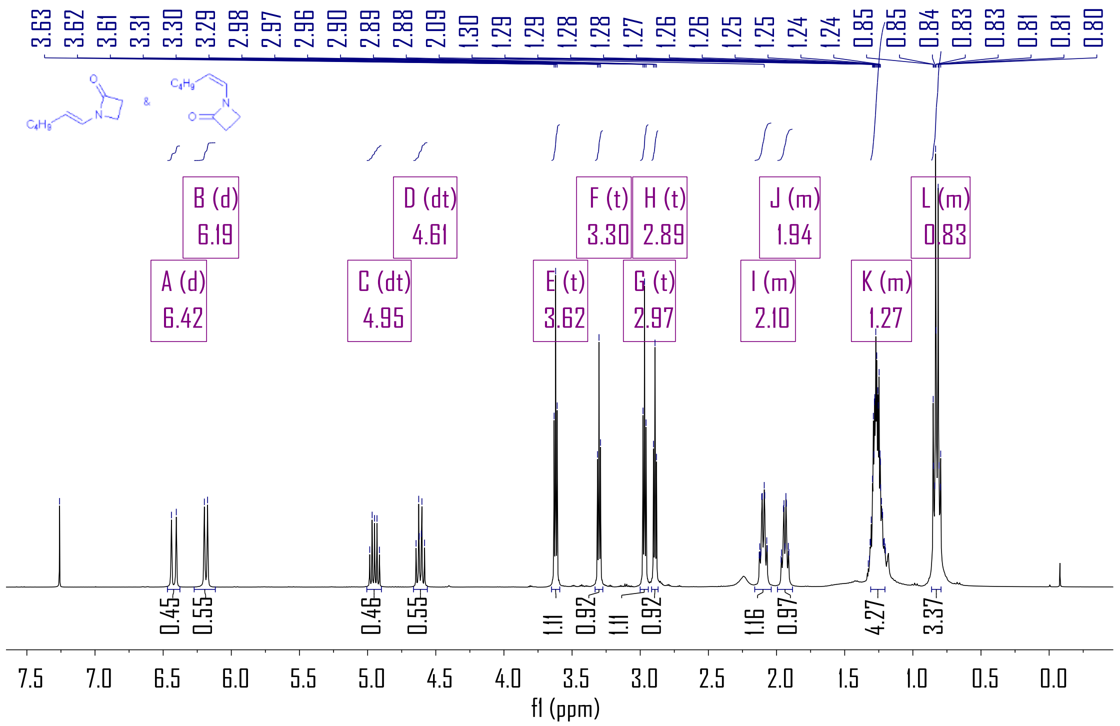

Supplementary Figure 83. ^{1}H NMR spectra for **RM36**

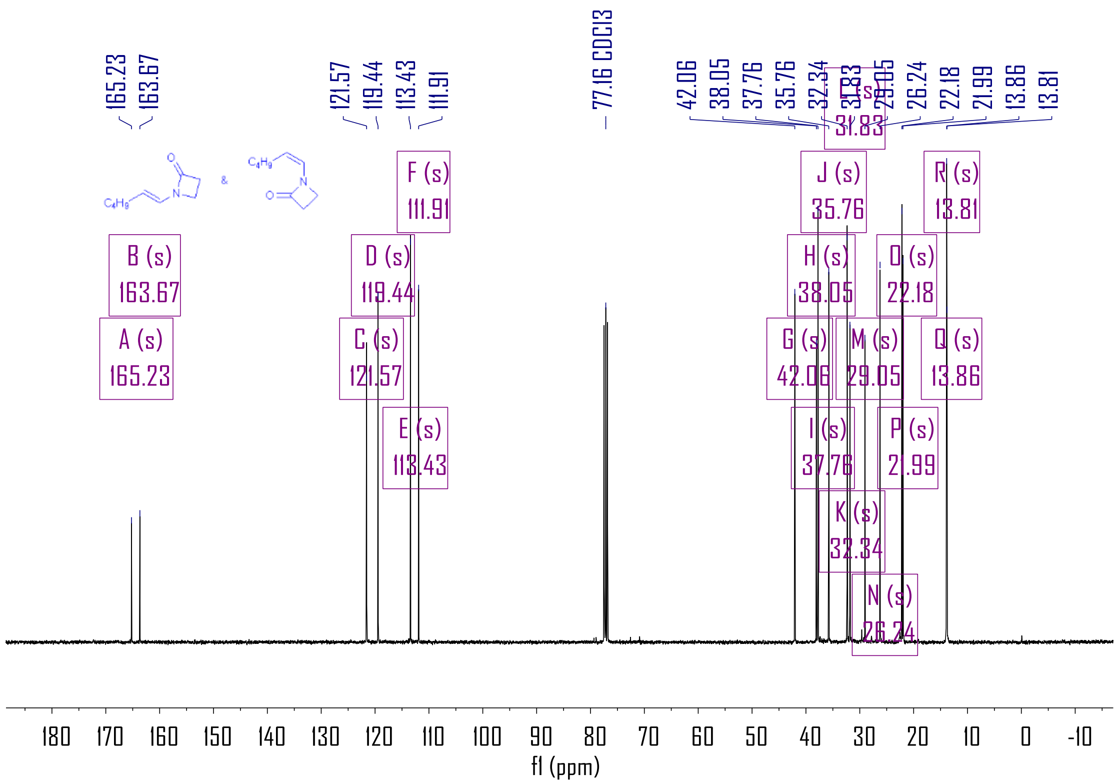

Supplementary Figure 84. ^{13}C NMR spectra for **RM36**

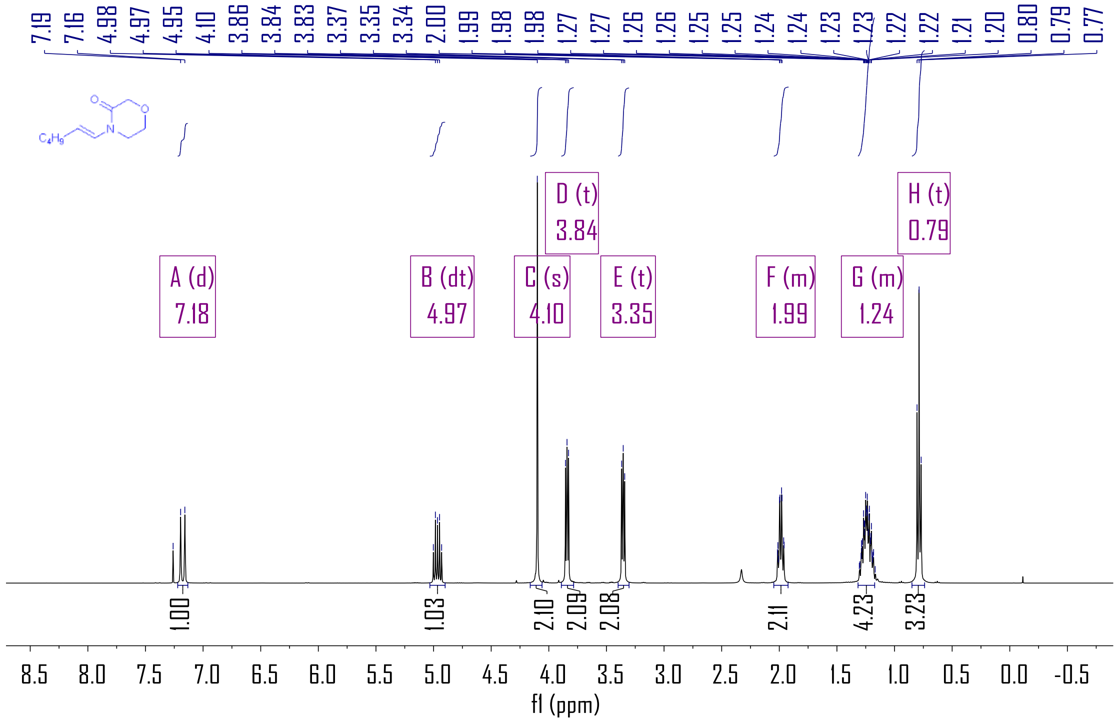

Supplementary Figure 85. ^{1}H NMR spectra for **RM37**

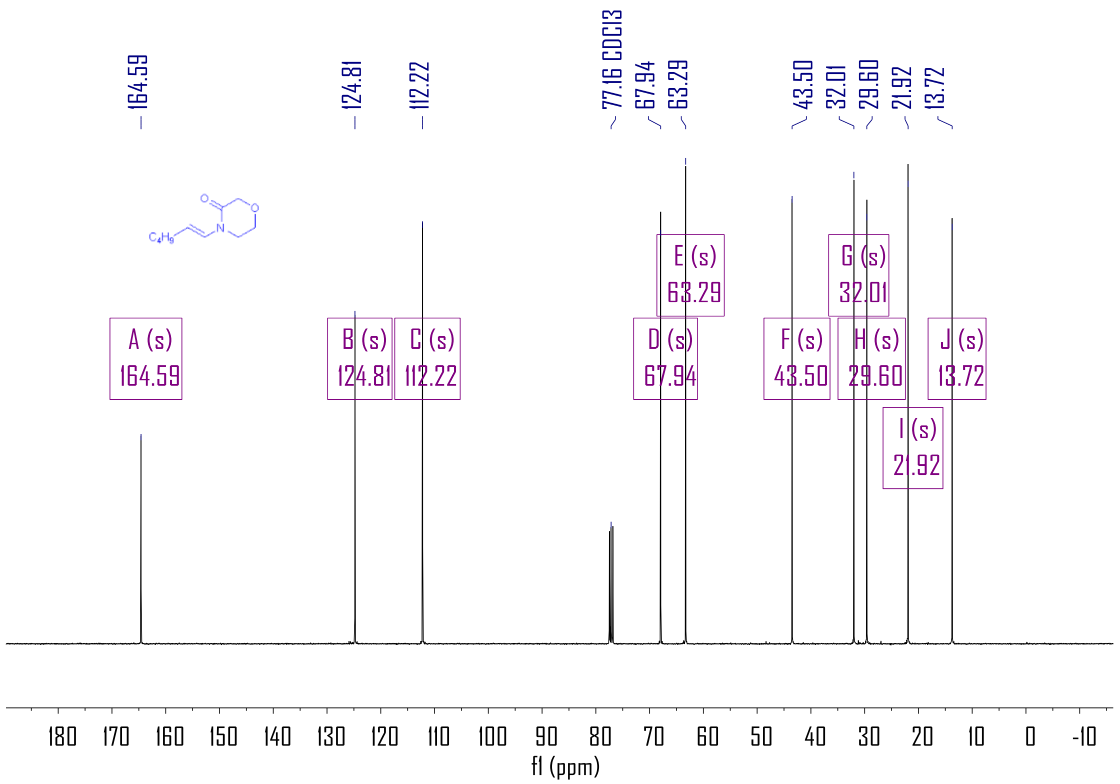

Supplementary Figure 86. ^{13}C NMR spectra for **RM37**

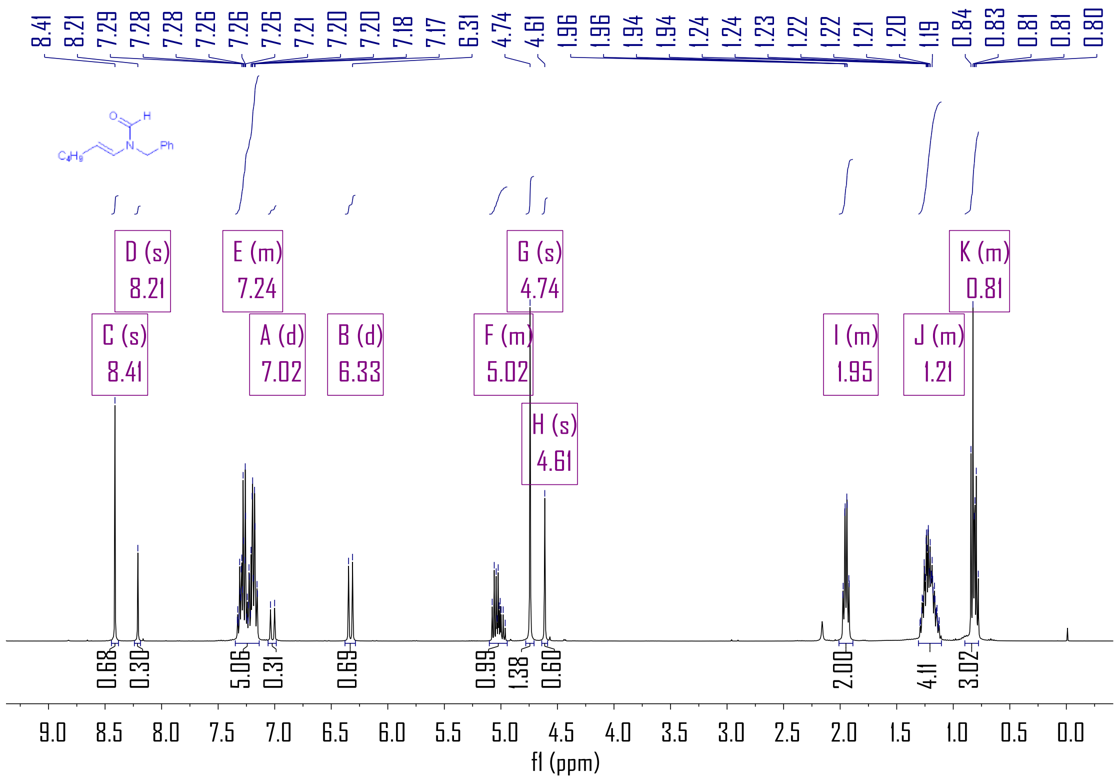

Supplementary Figure 87. ^{1}H NMR spectra for **RM38**

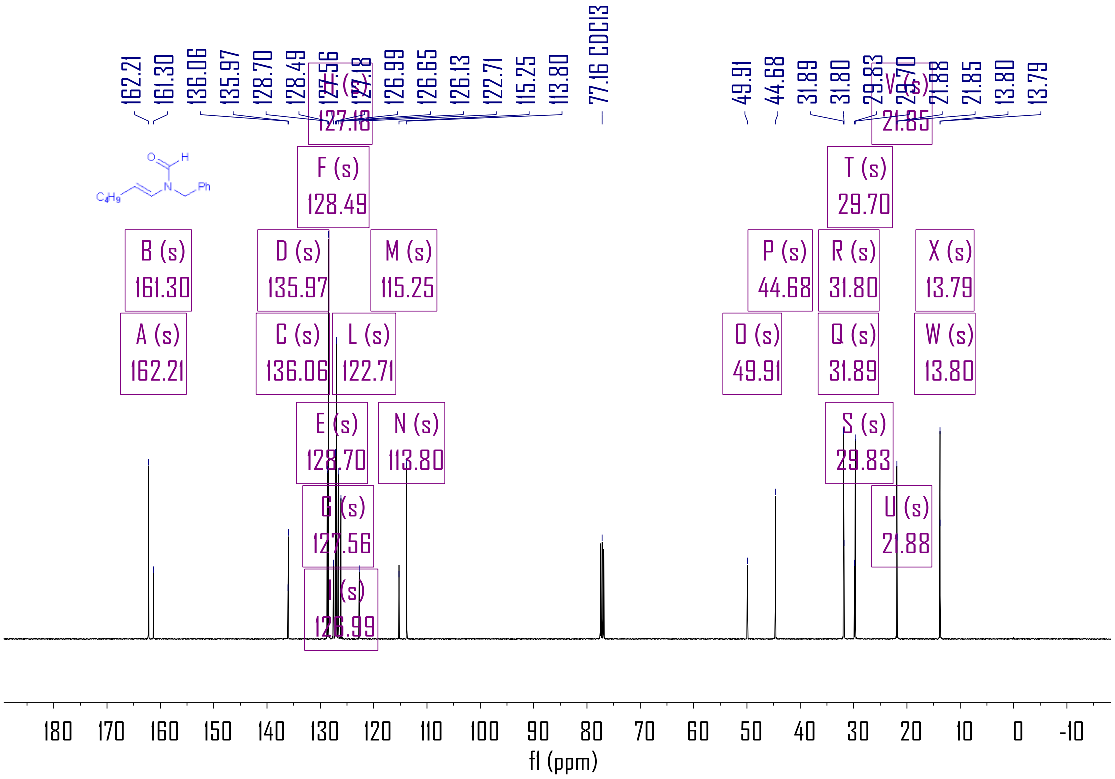

Supplementary Figure 88. ^{13}C NMR spectra for **RM38**

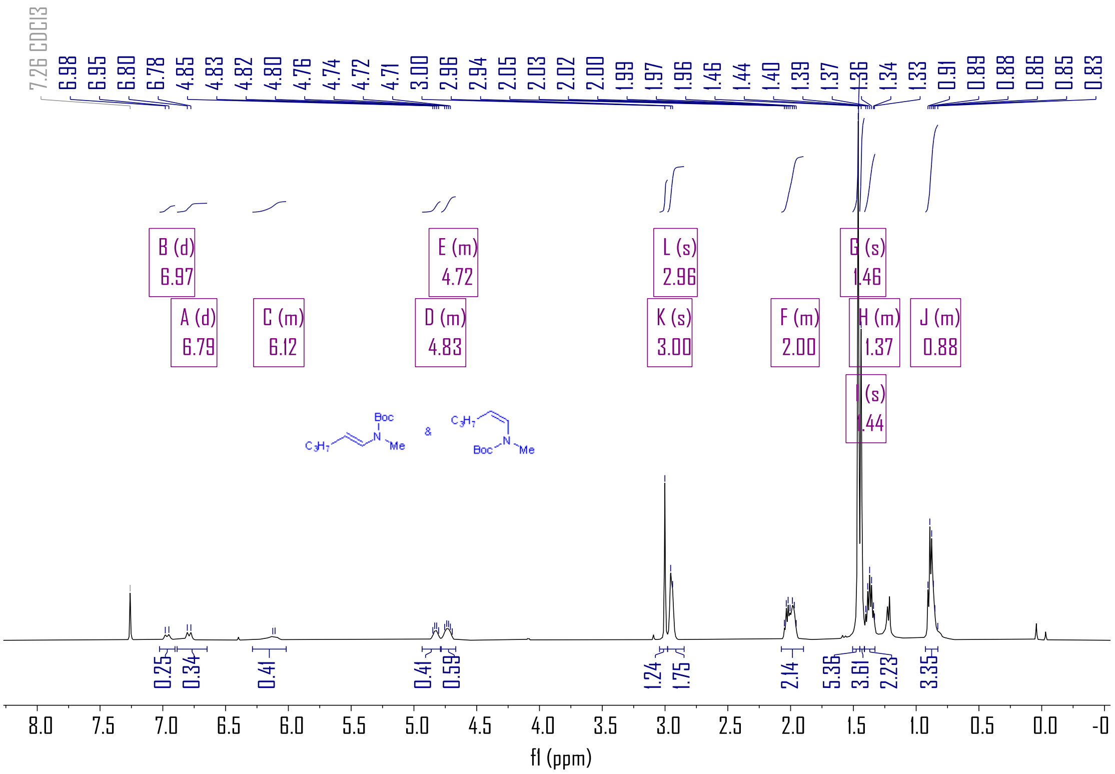

Supplementary Figure 89. ^{1}H NMR spectra for **RM39**

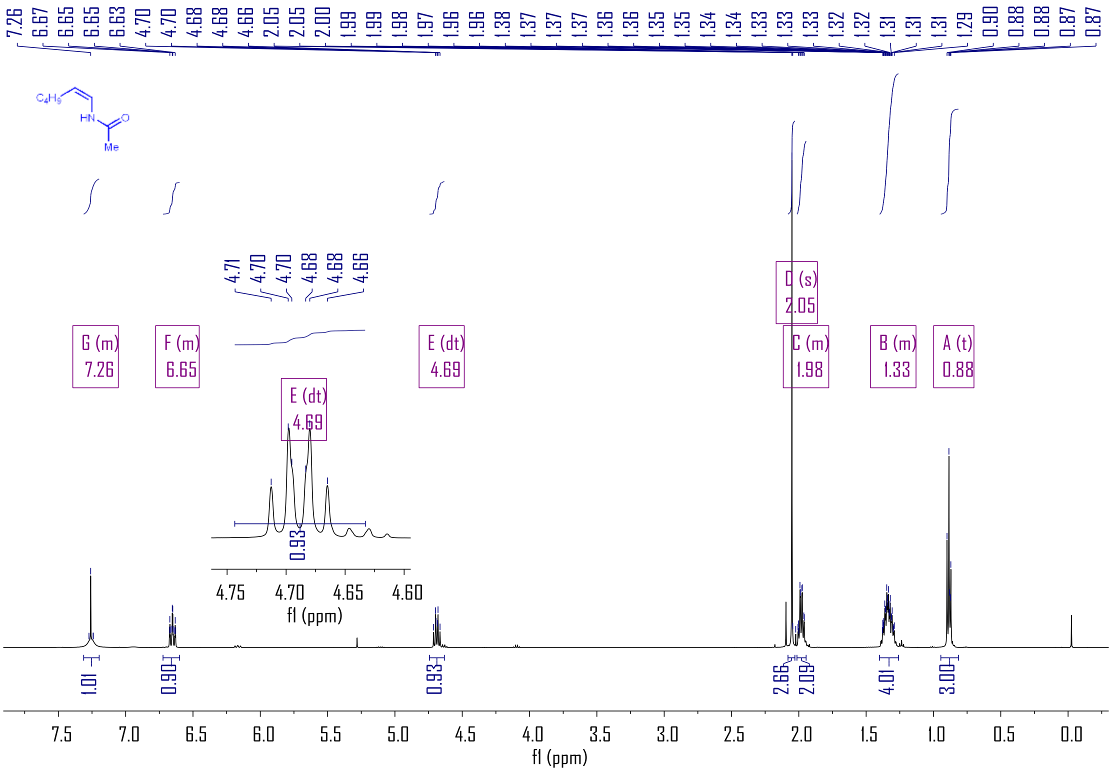

Supplementary Figure 90. ^{1}H NMR spectra for **RM40**

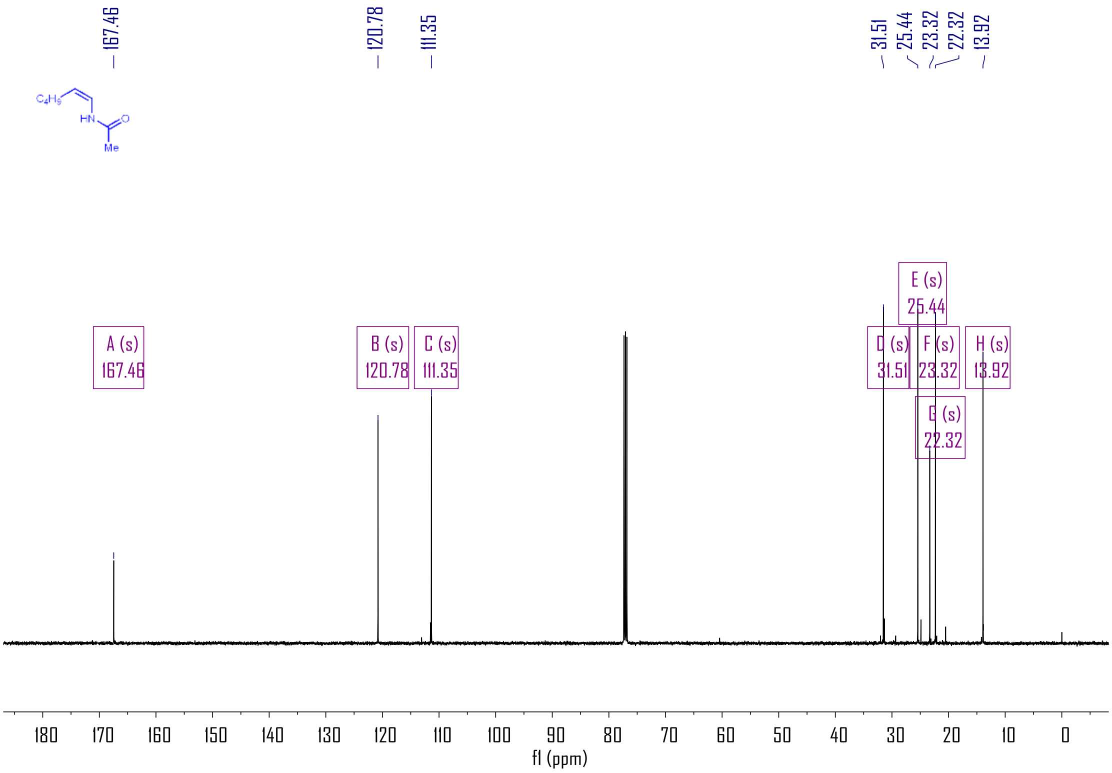

Supplementary Figure 91. ^{13}C NMR spectra for **RM40**

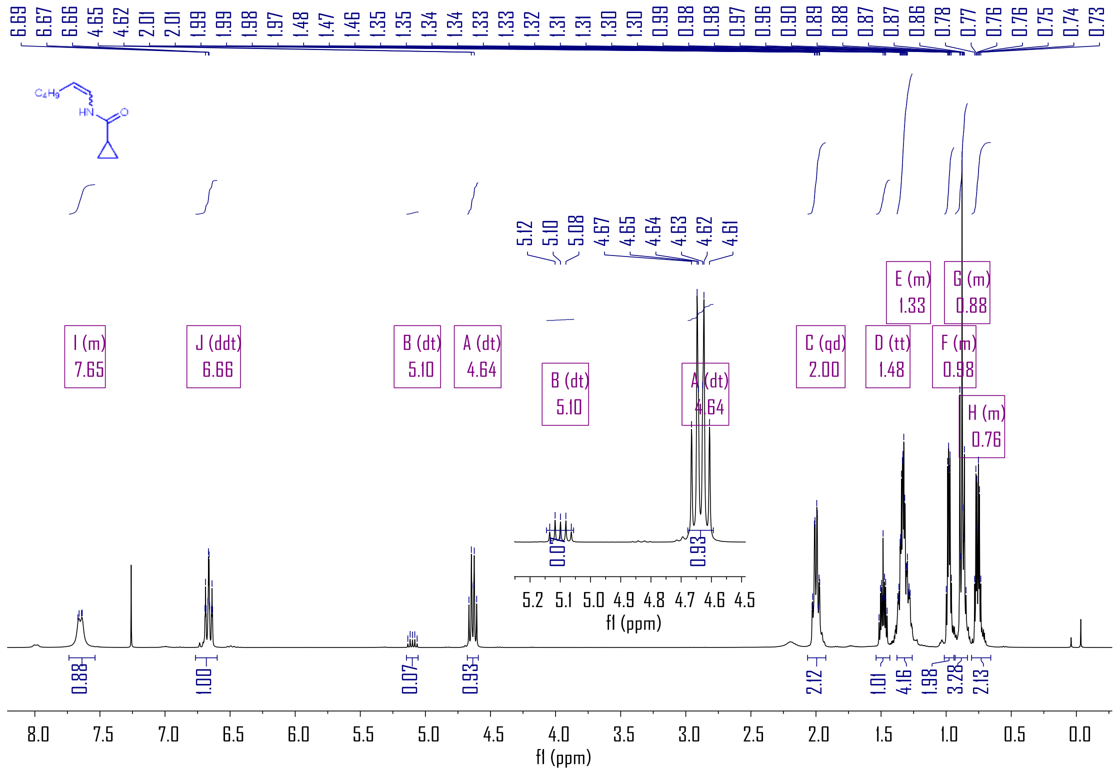

Supplementary Figure 92. ^{1}H NMR spectra for **RM41**

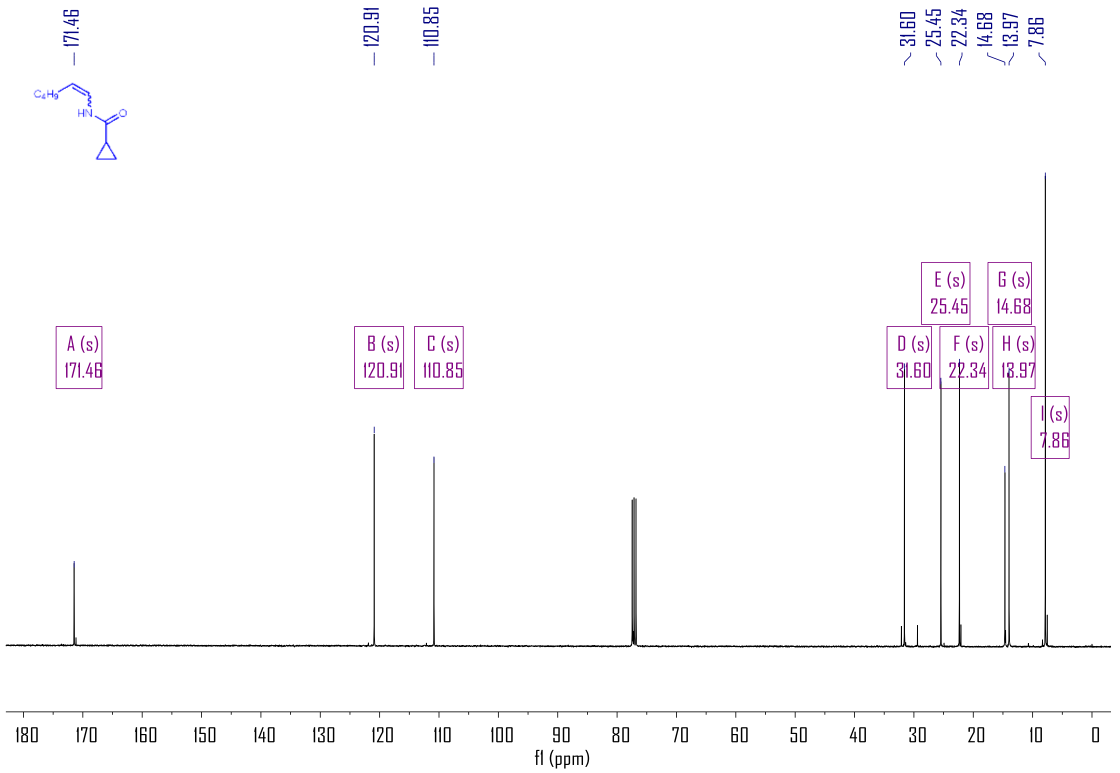

Supplementary Figure 93. ^{13}C NMR spectra for **RM41**

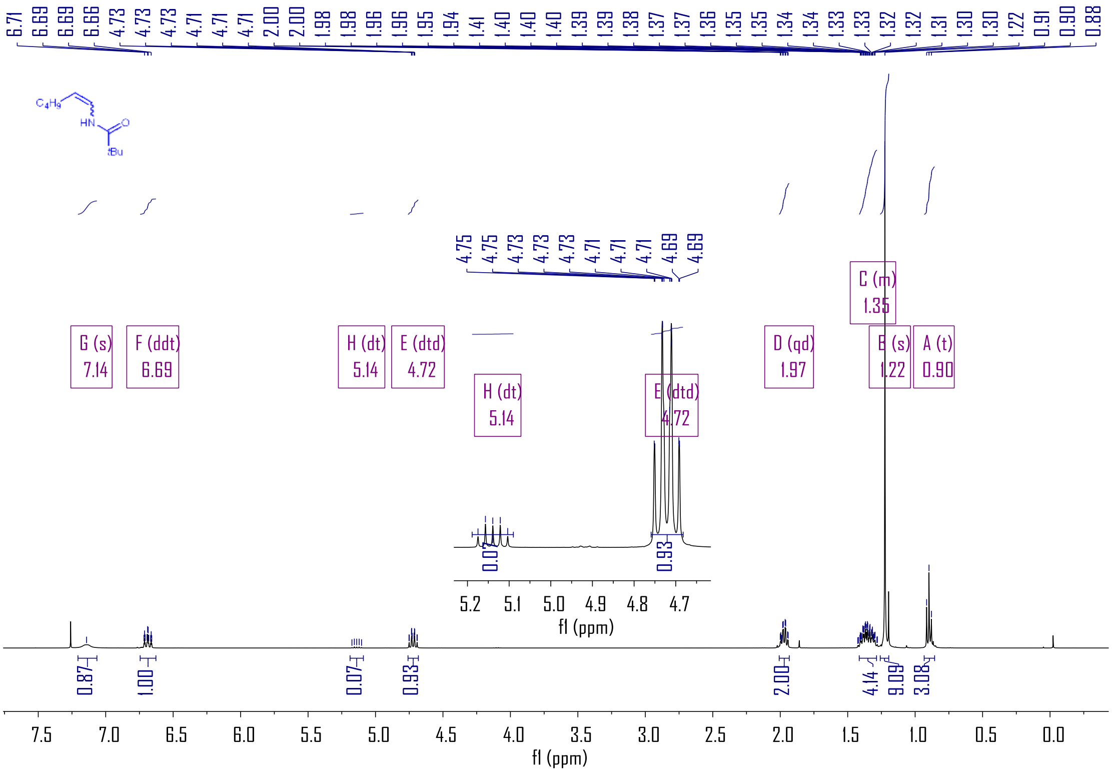

Supplementary Figure 94. ^{1}H NMR spectra for **RM42**

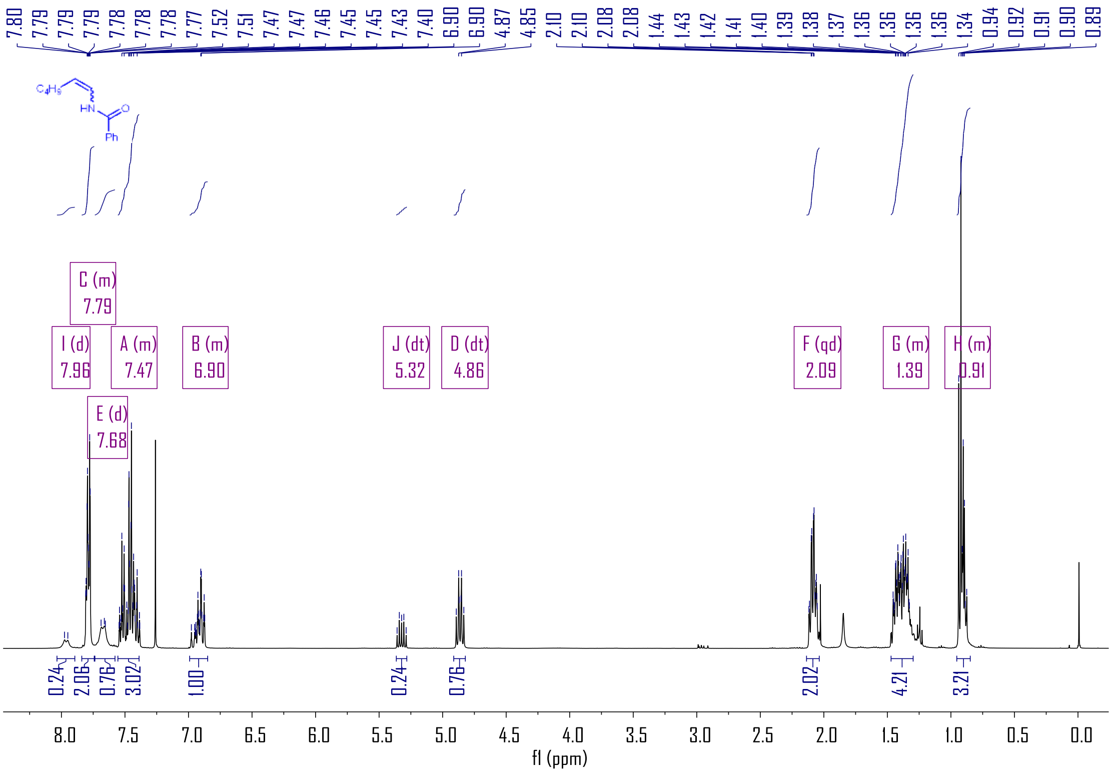

Supplementary Figure 95. ^{1}H NMR spectra for **RM43**

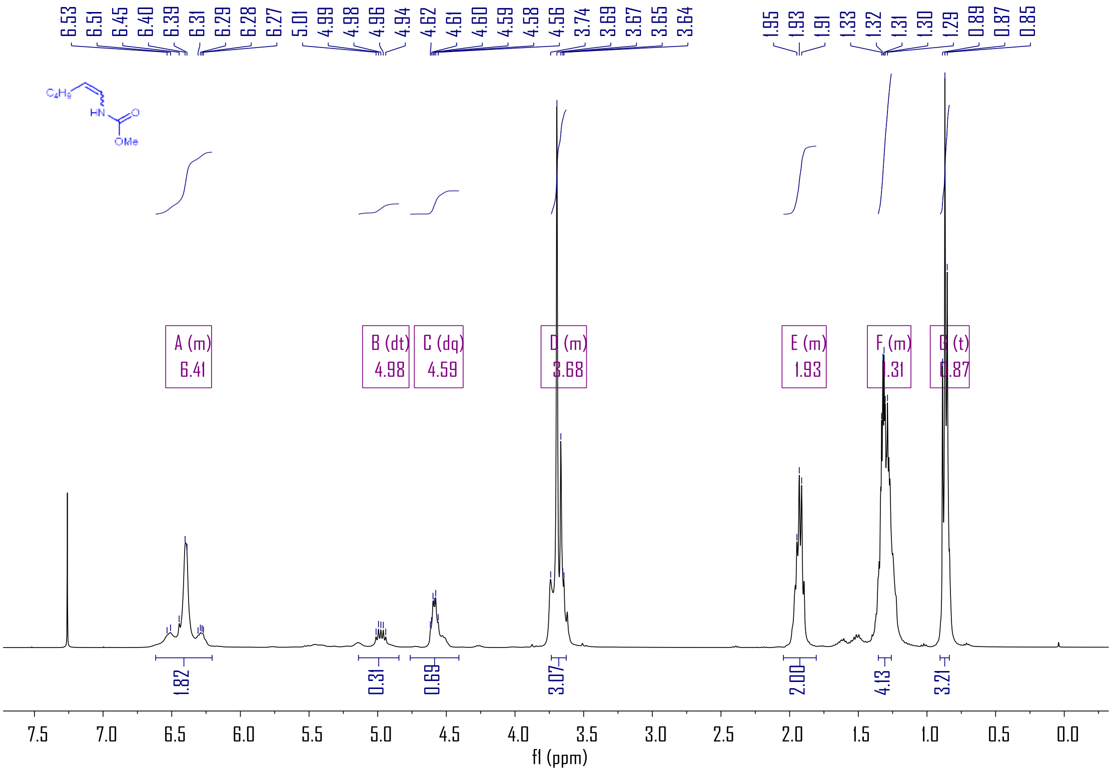

Supplementary Figure 96. ^{1}H NMR spectra for **RM44**

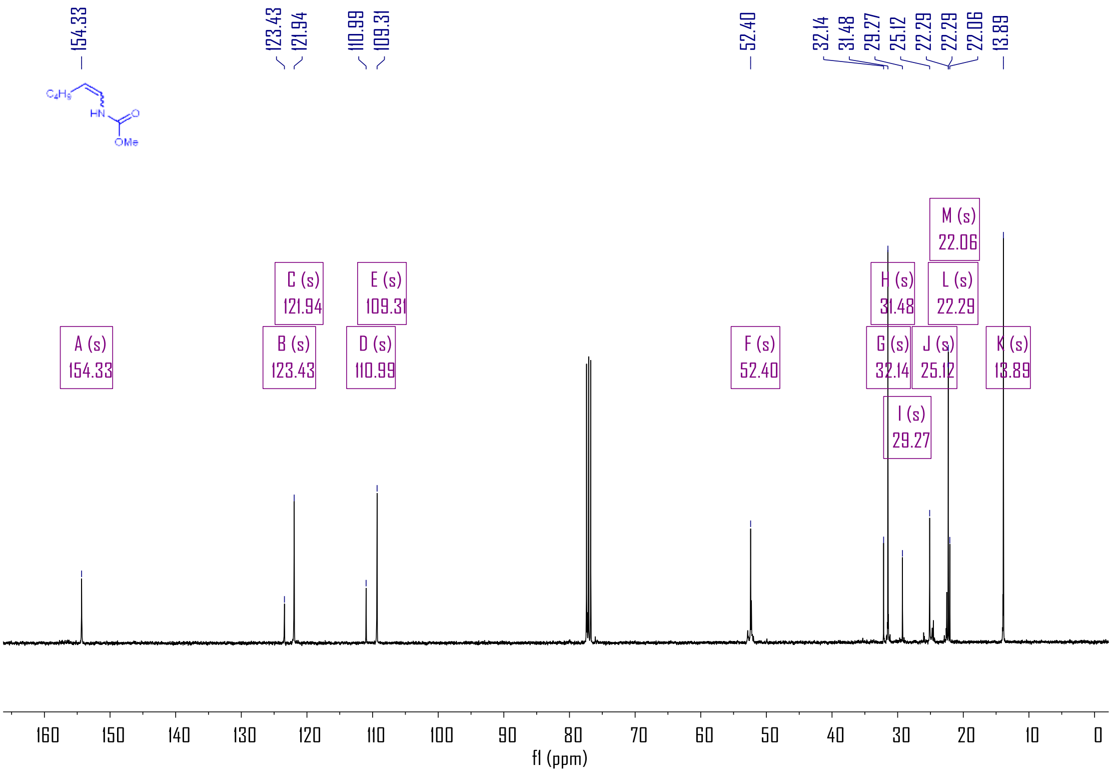

Supplementary Figure 97. ^{13}C NMR spectra for **RM44**

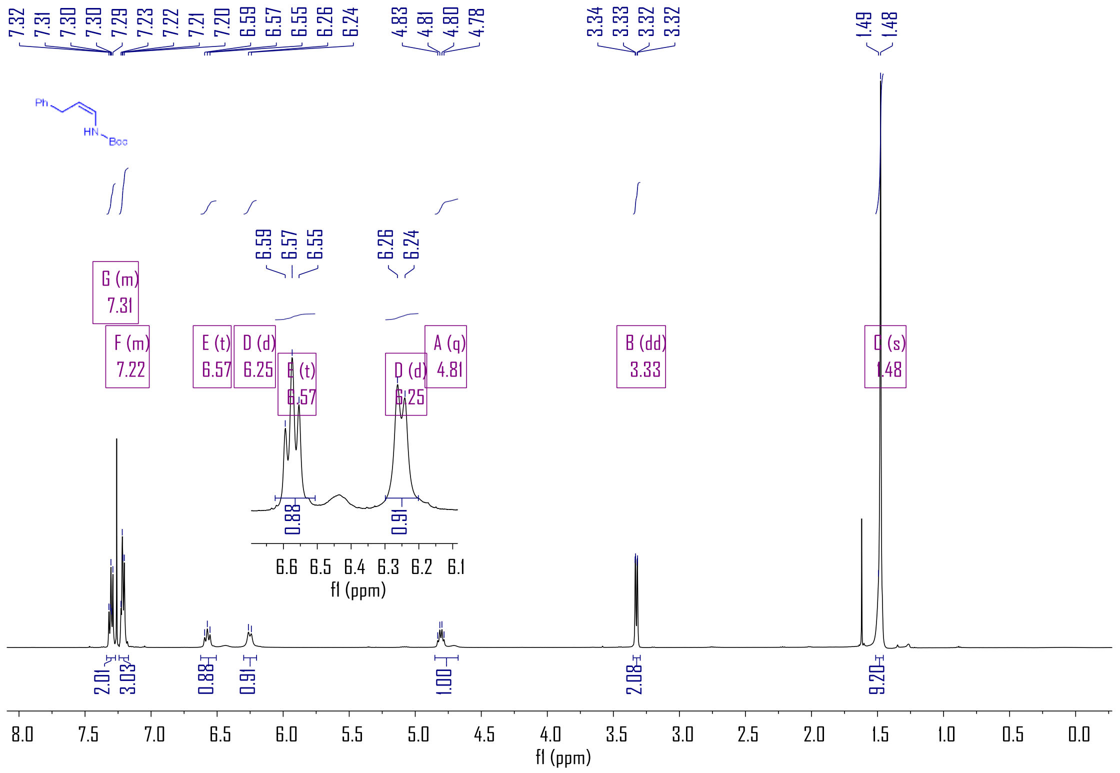

Supplementary Figure 98. ^{1}H NMR spectra for **RM45**

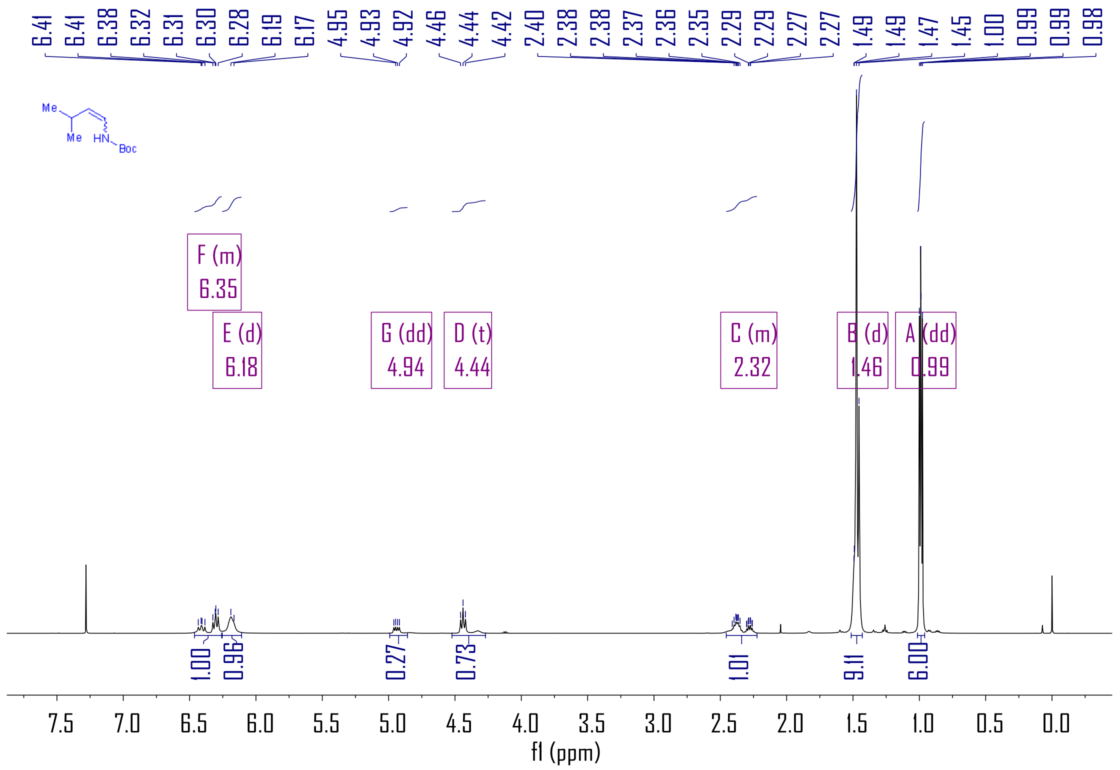

Supplementary Figure 99. ^{1}H NMR spectra for **RM46**

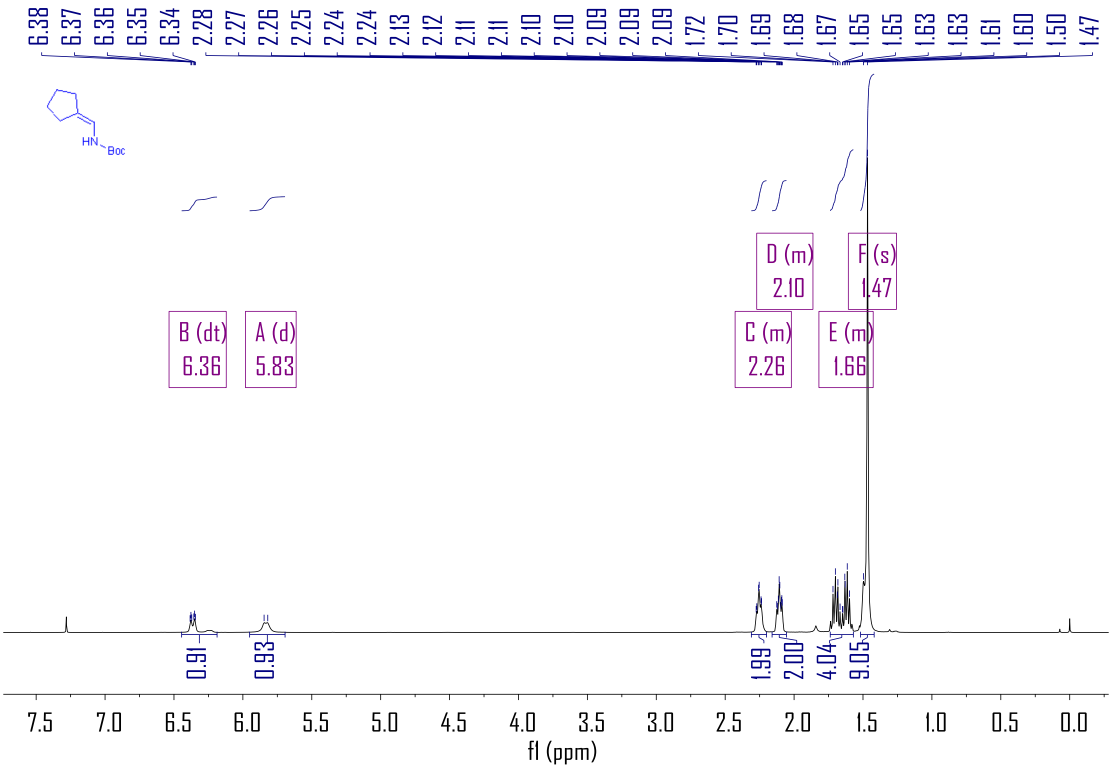

Supplementary Figure 100. ^{1}H NMR spectra for **RM47**

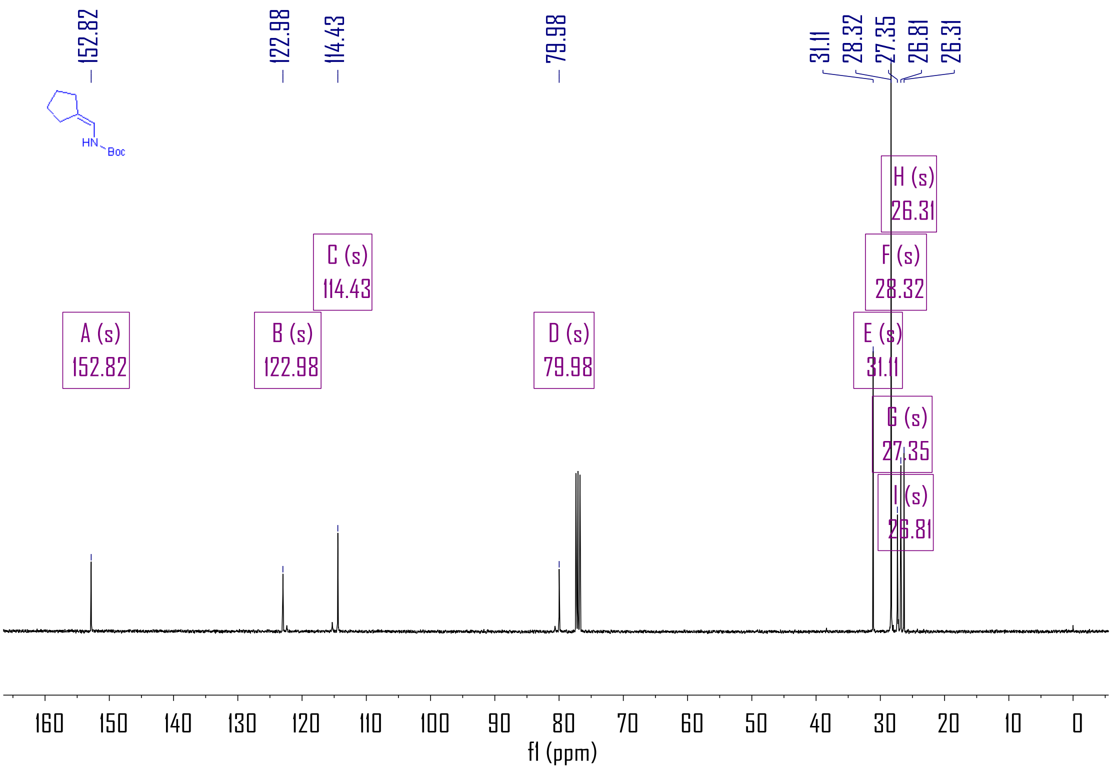

Supplementary Figure 101. ^{13}C NMR spectra for **RM47**

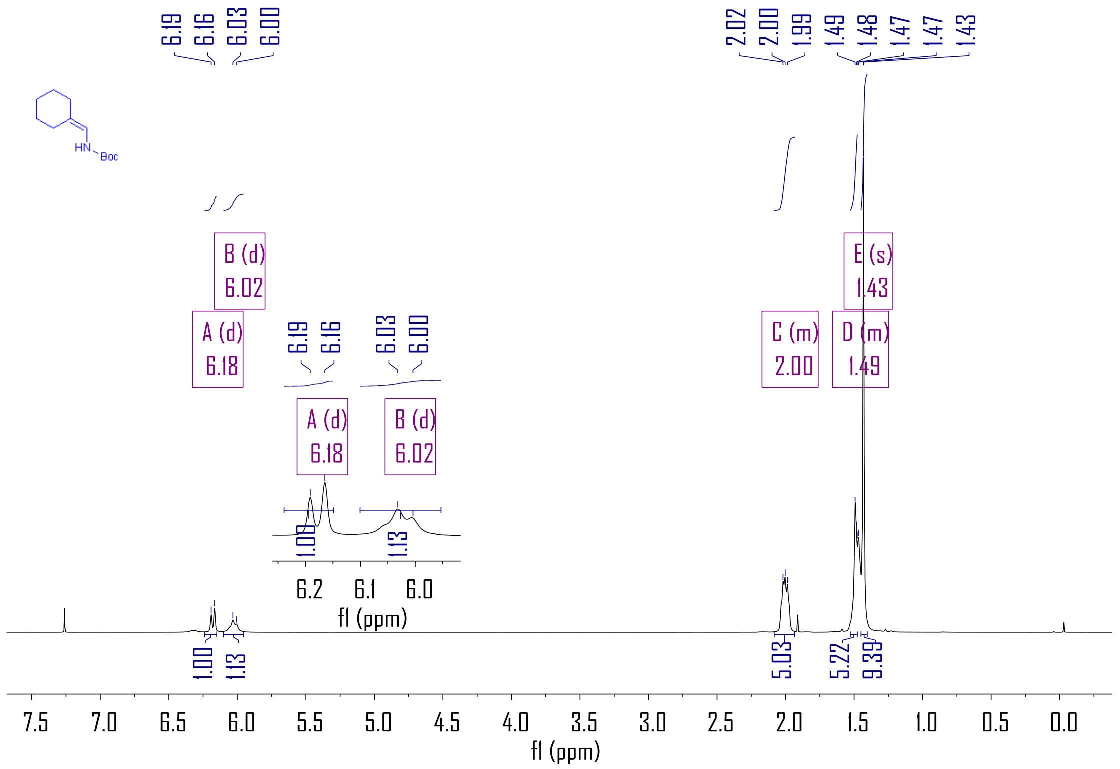

Supplementary Figure 102. ^{1}H NMR spectra for **RM48**

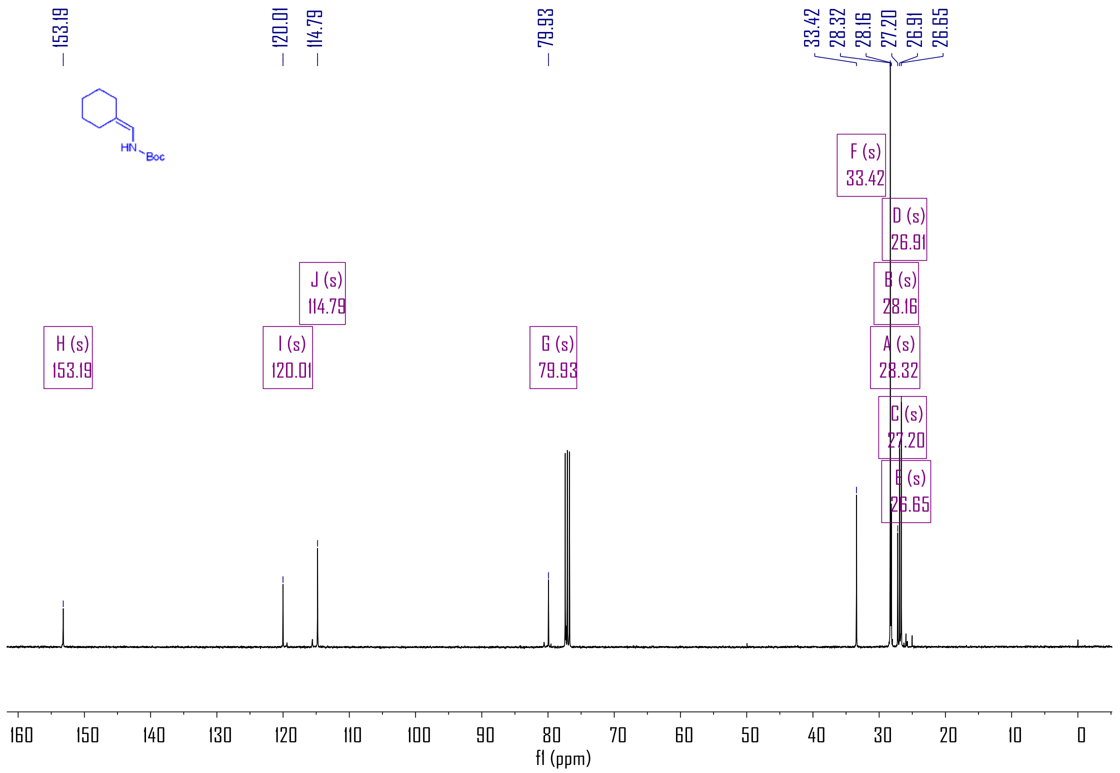

Supplementary Figure 103. ^{13}C NMR spectra for **RM48**

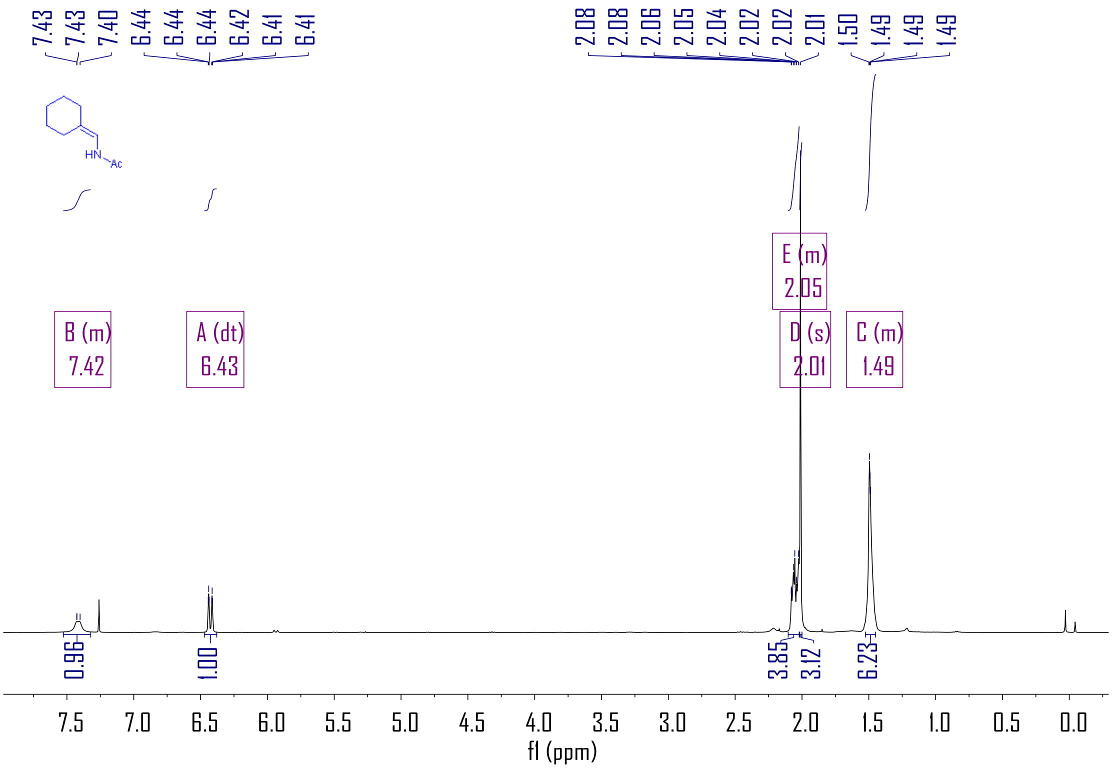

Supplementary Figure 104. ^{1}H NMR spectra for **RM49**

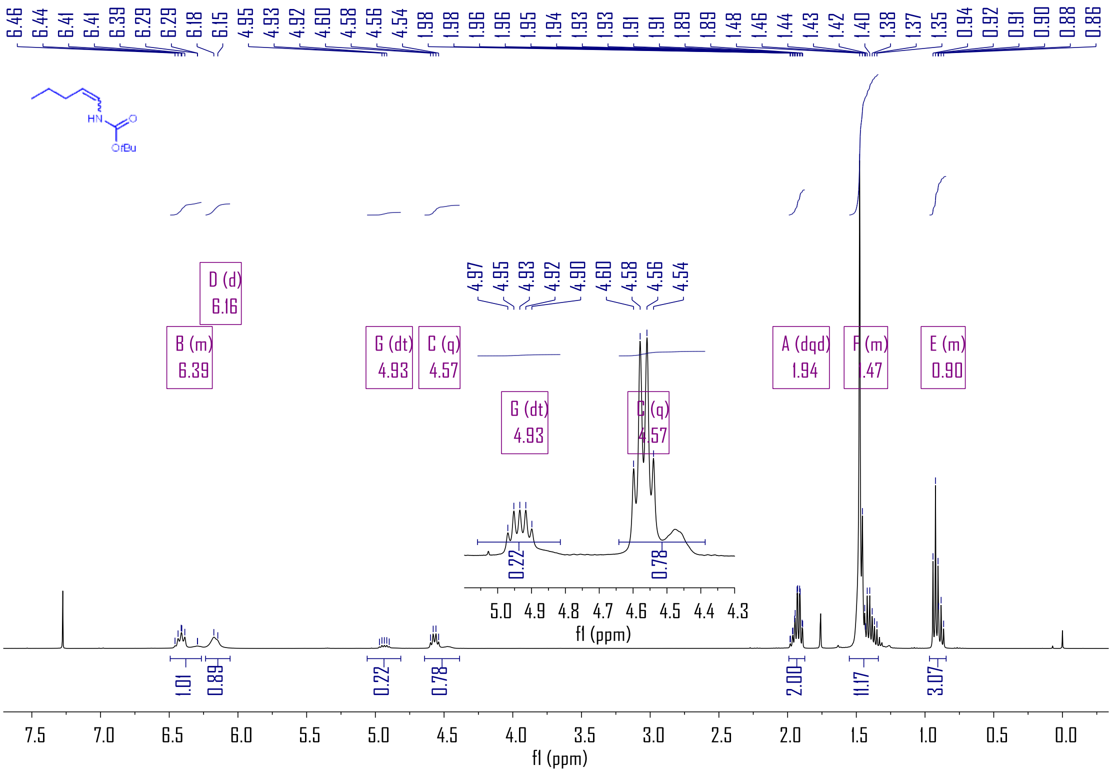

Supplementary Figure 105. ^{1}H NMR spectra for **RM50**

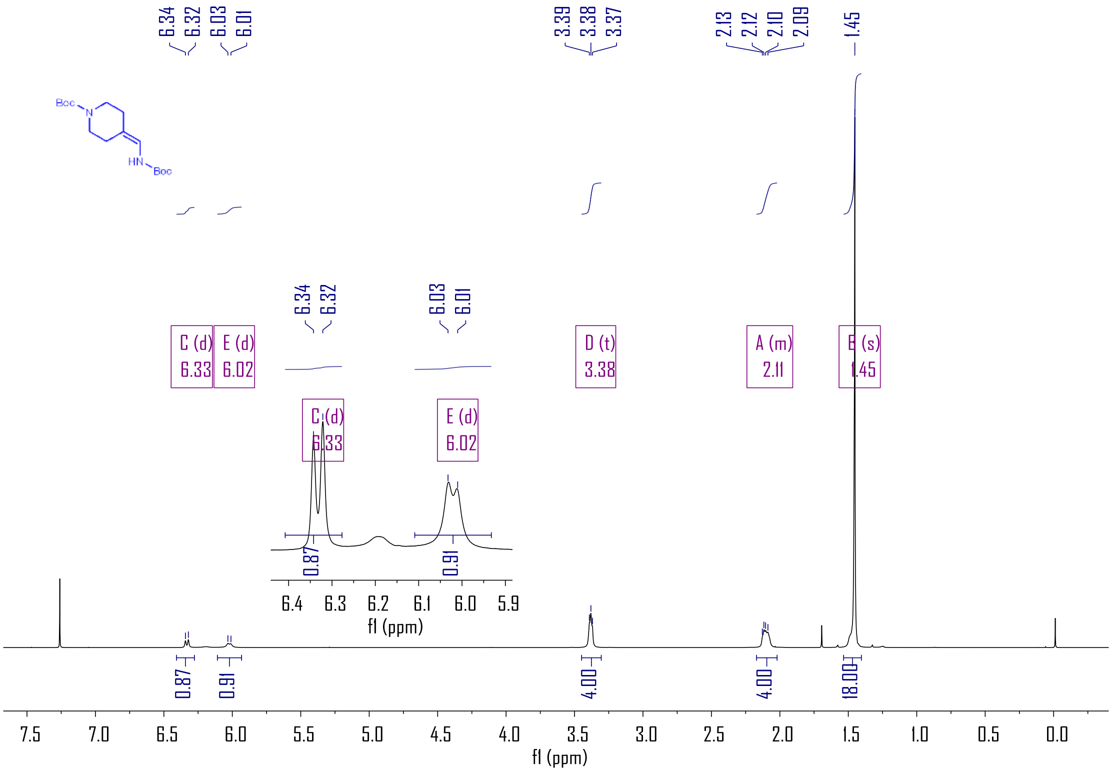

Supplementary Figure 106. ^{1}H NMR spectra for **RM51**

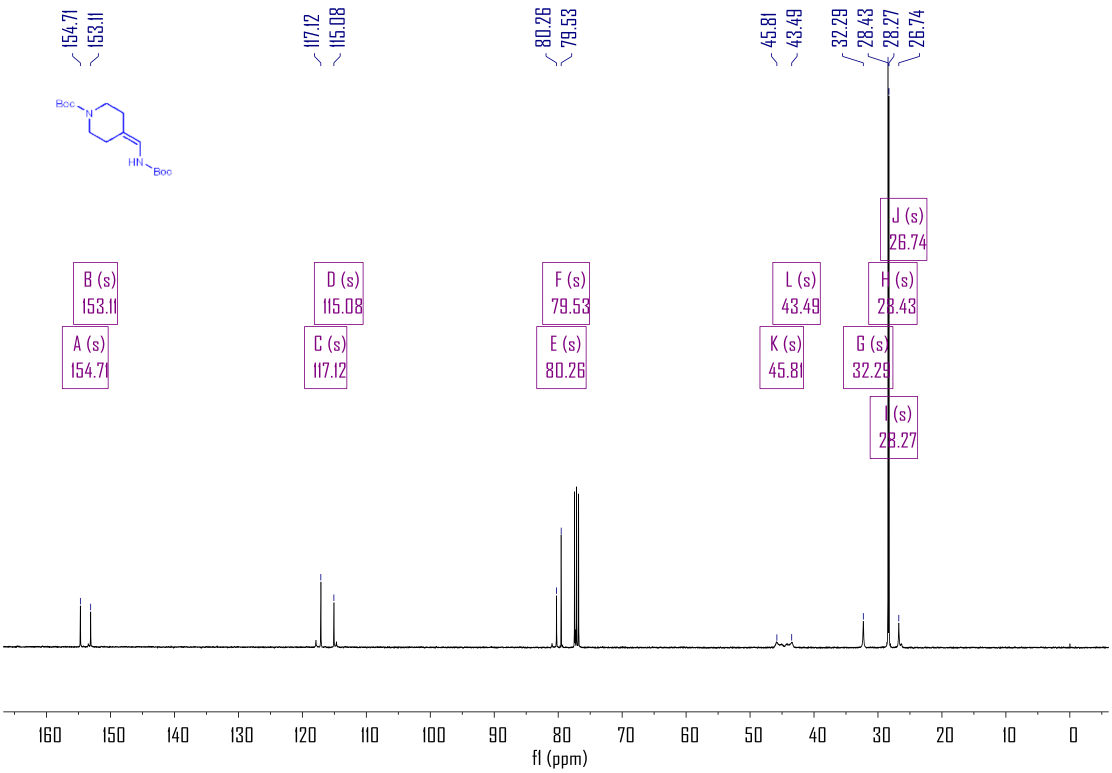

Supplementary Figure 107. ^{13}C NMR spectra for **RM51**

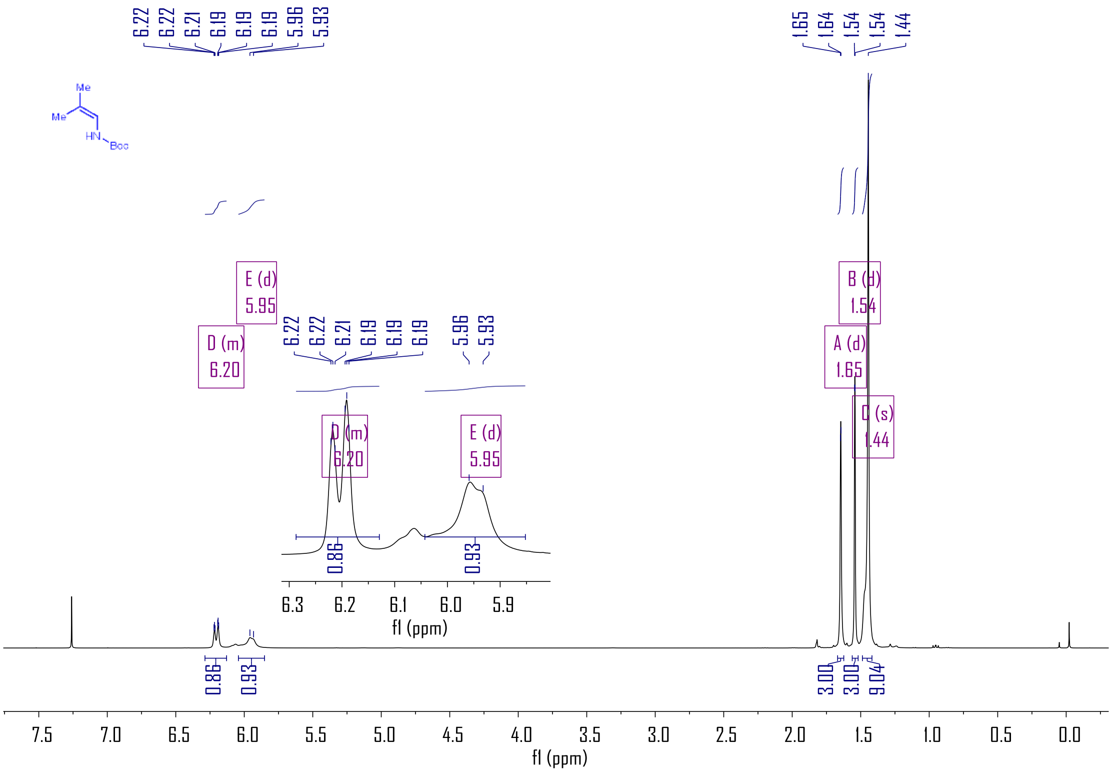

Supplementary Figure 108. ^{1}H NMR spectra for **RM52**

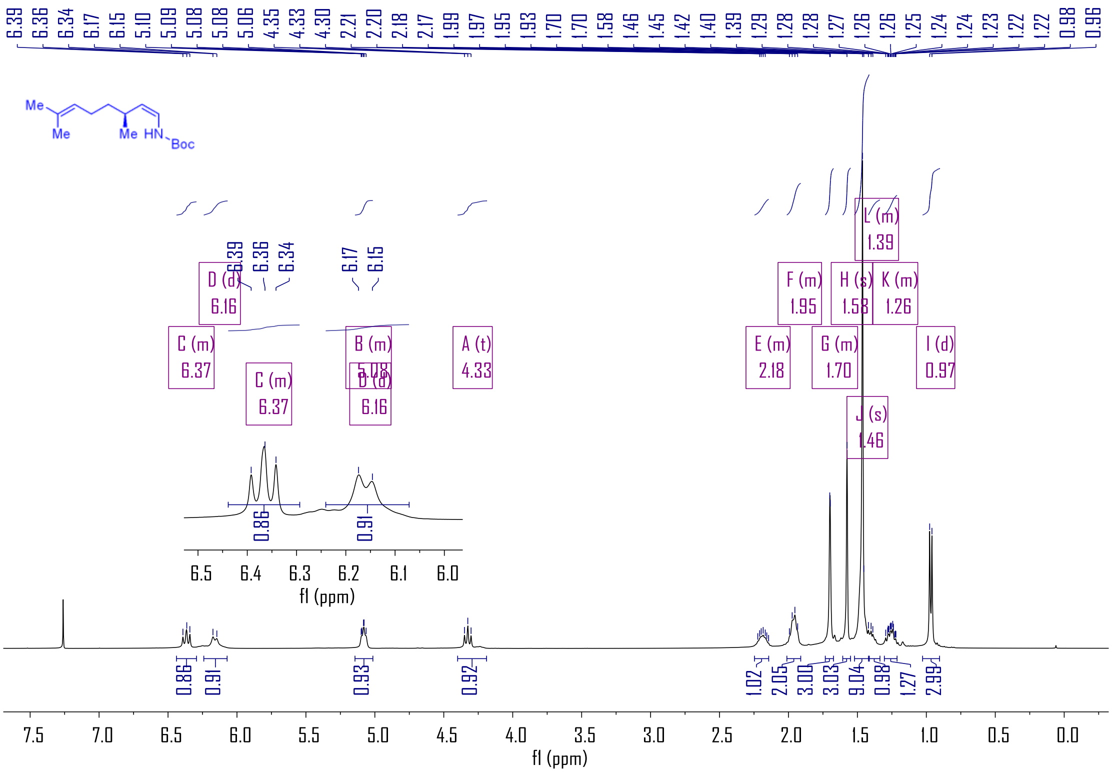

Supplementary Figure 109. ^{1}H NMR spectra for **RM53**

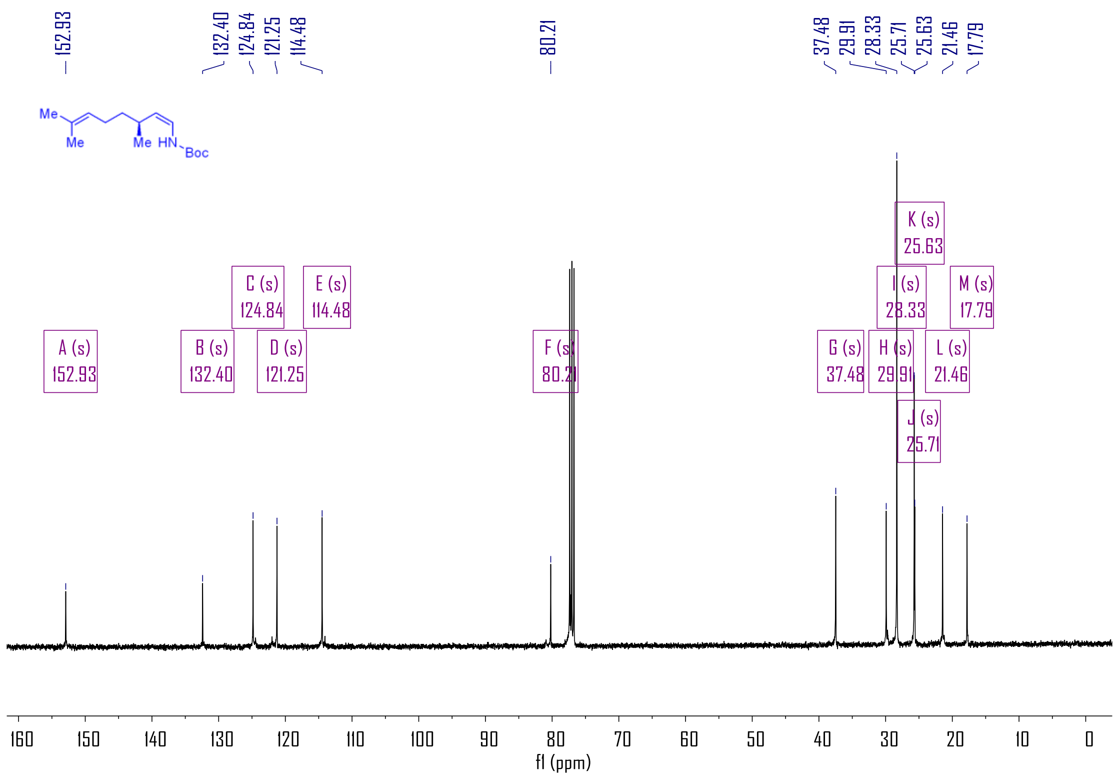

Supplementary Figure 110. ^{13}C NMR spectra for **RM53**

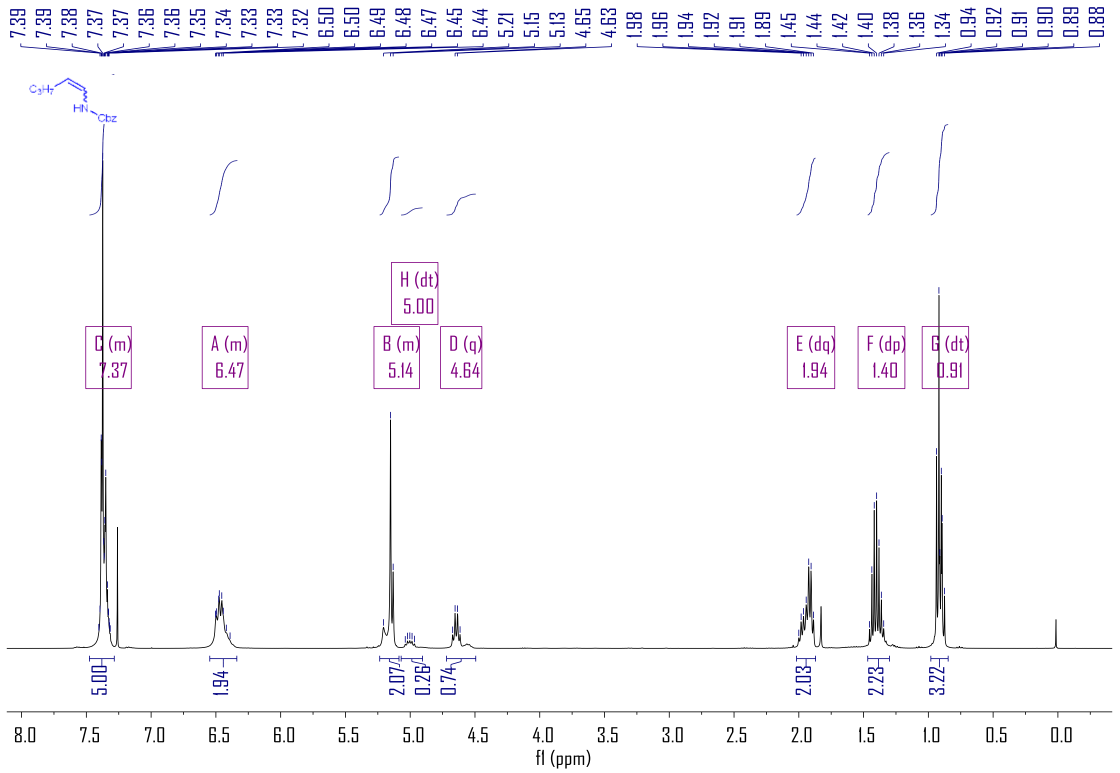

Supplementary Figure 111. ^{1}H NMR spectra for **RM54**

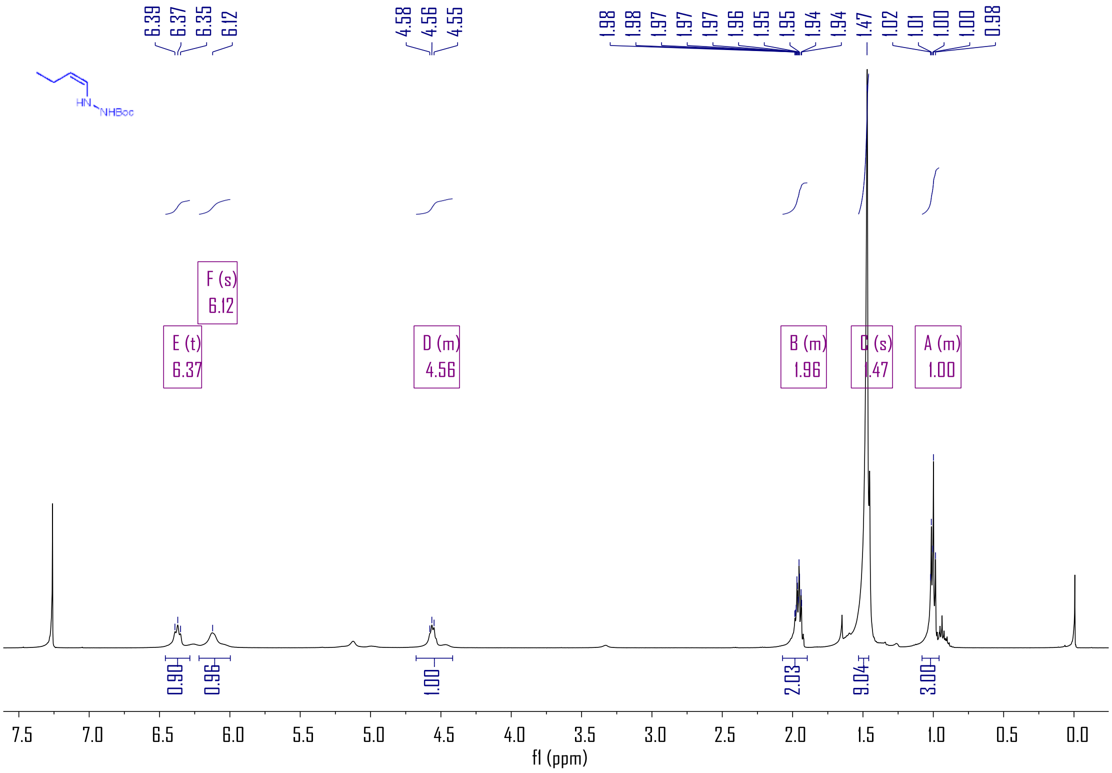

Supplementary Figure 112. ^{1}H NMR spectra for **RM55**

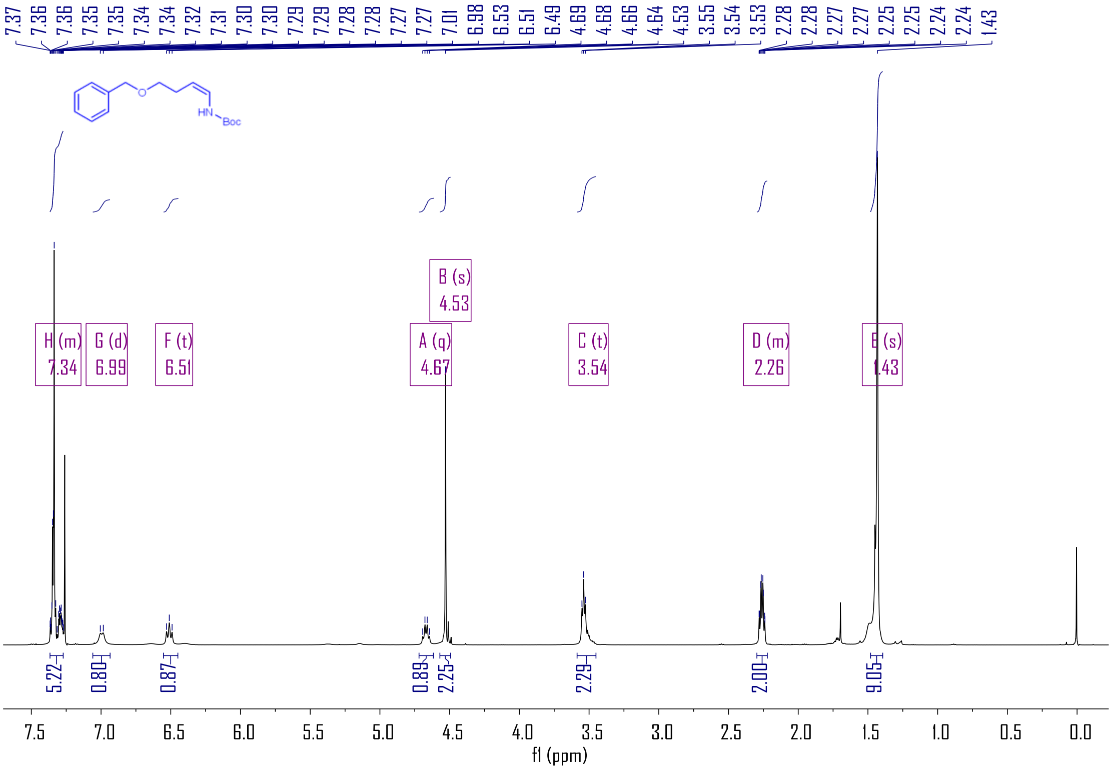

Supplementary Figure 113. ^{1}H NMR spectra for **RM56**

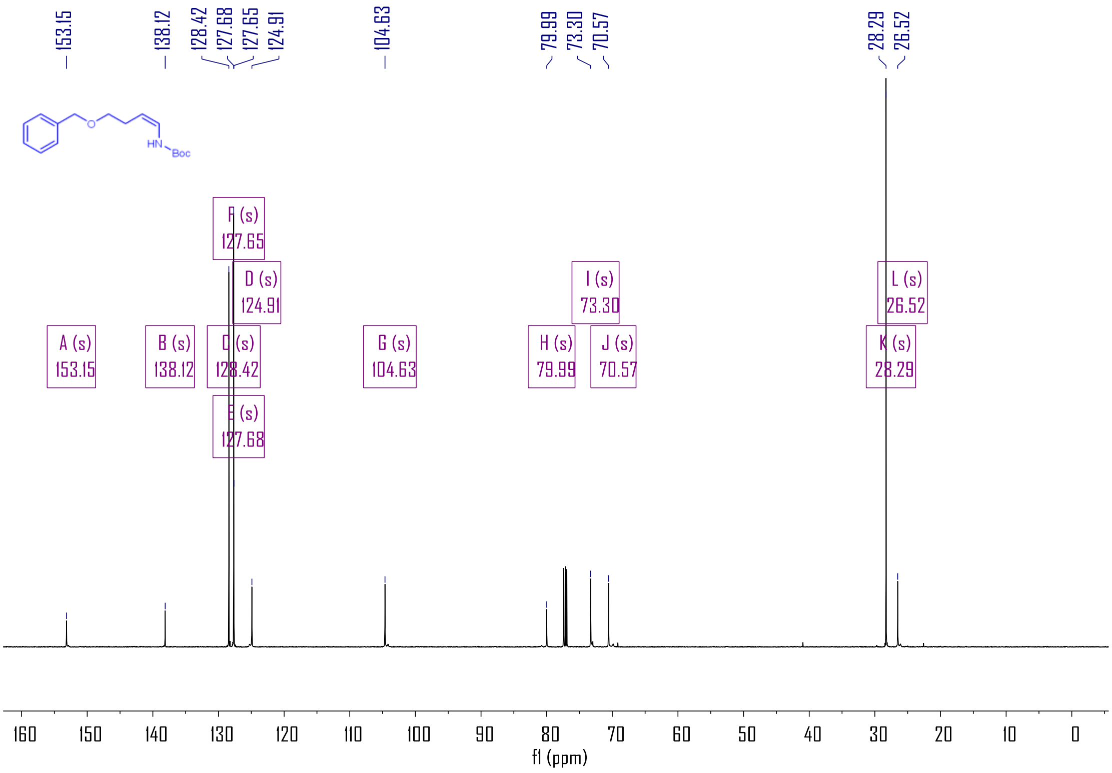

Supplementary Figure 114. ^{13}C NMR spectra for **RM56**

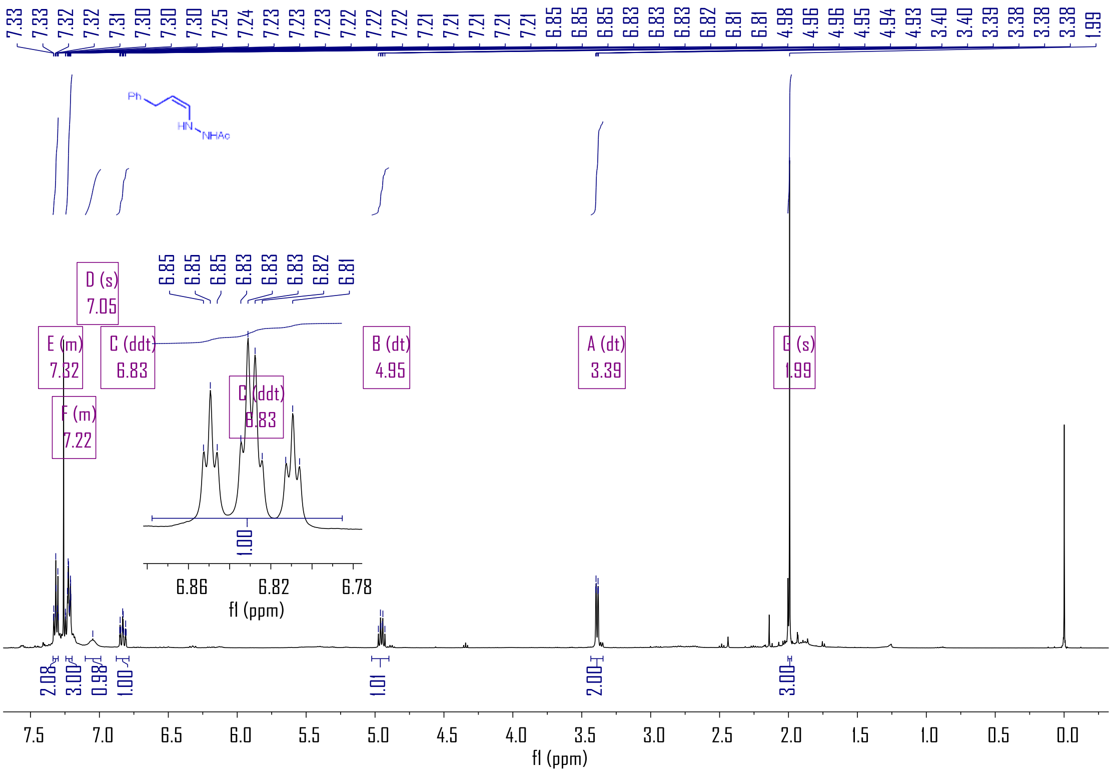

Supplementary Figure 115. ^{1}H NMR spectra for **RM57**

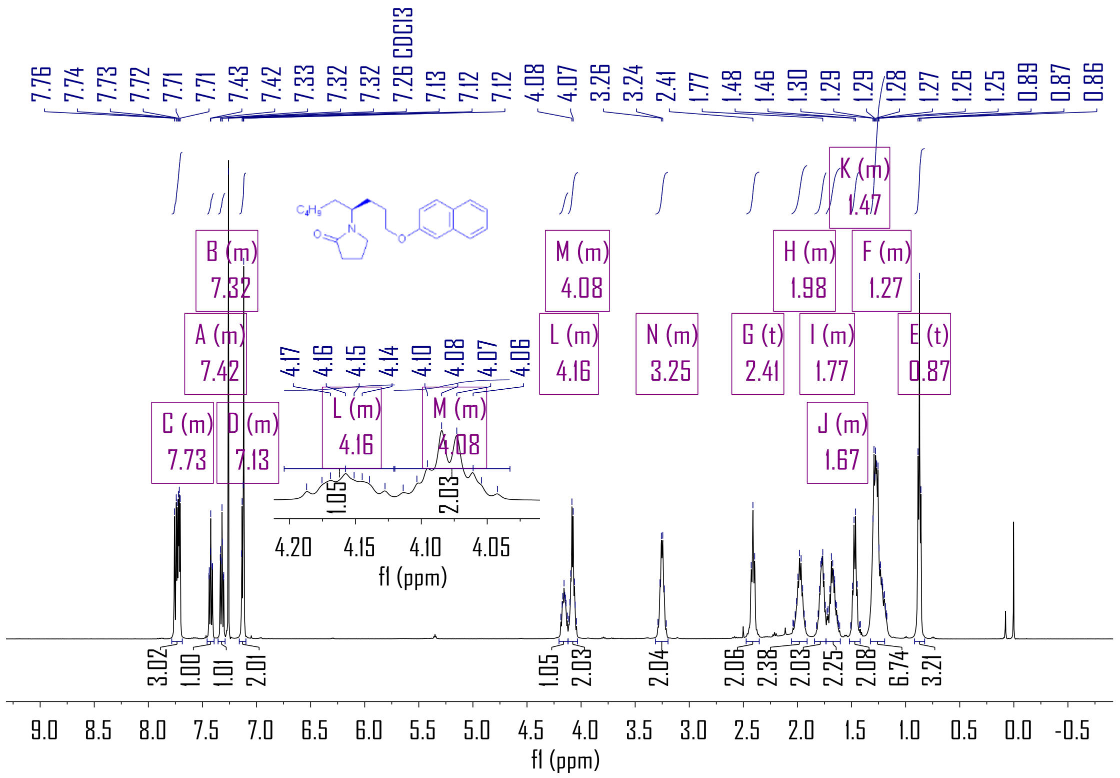

Supplementary Figure 116. ^{1}H NMR spectra for **1**

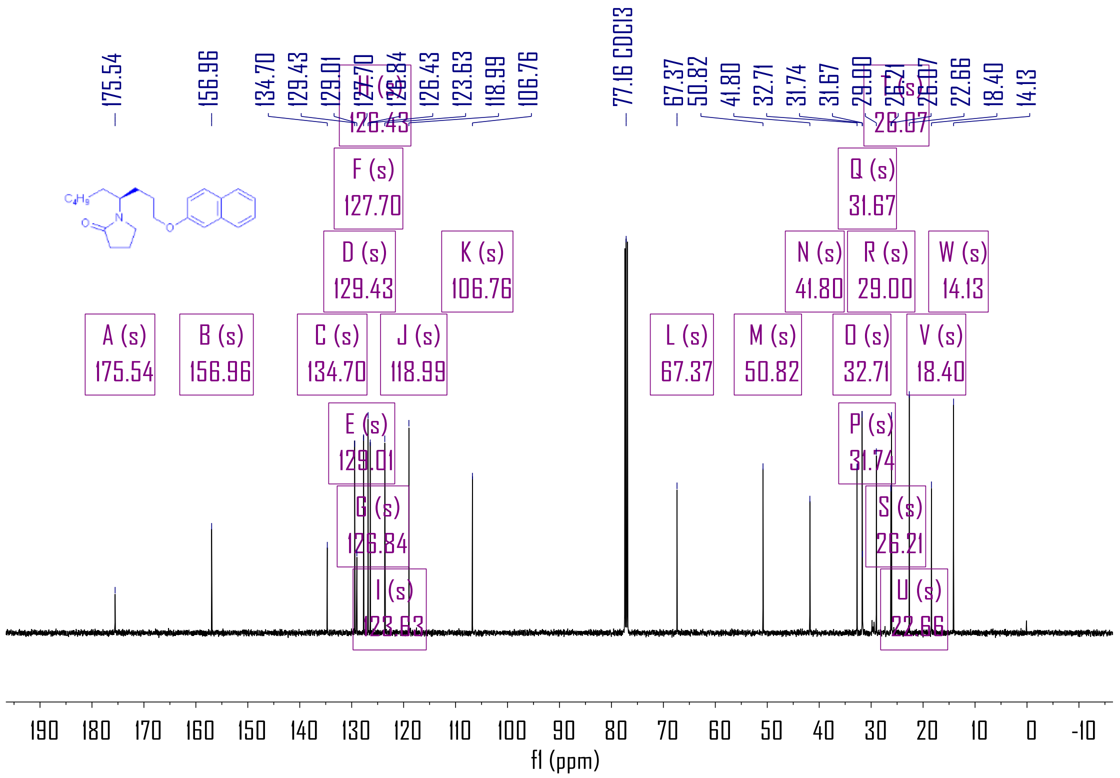

Supplementary Figure 117. ^{13}C NMR spectra for **1**

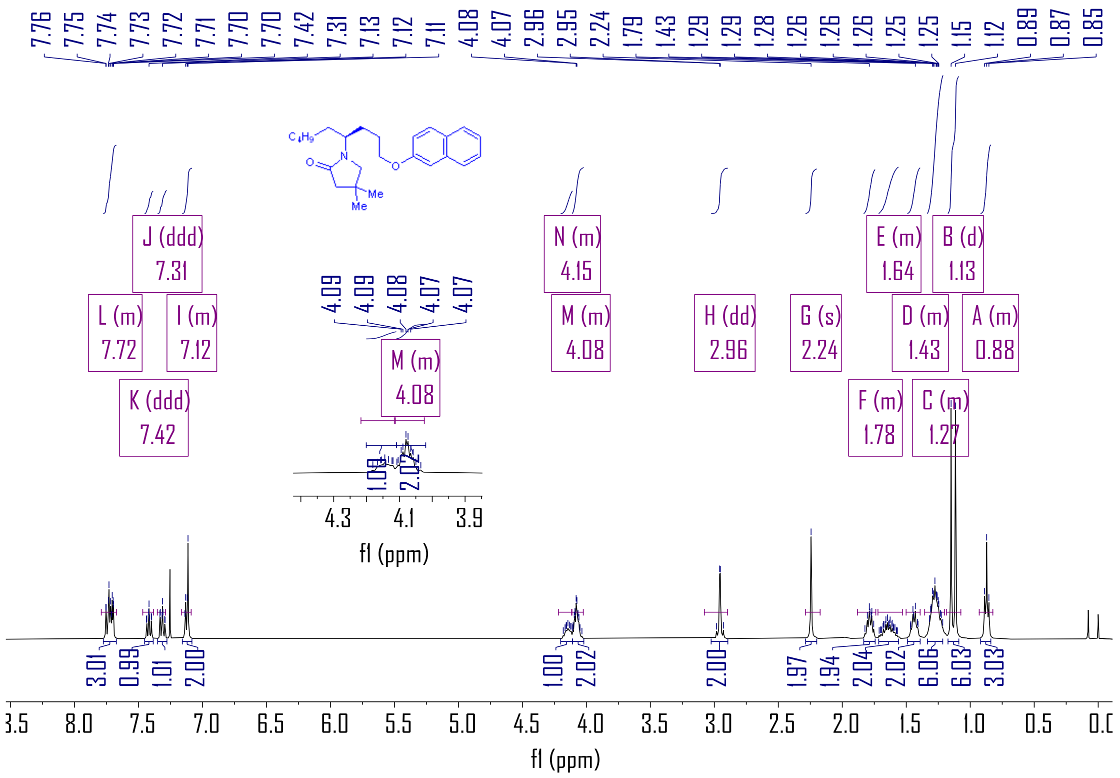

Supplementary Figure 118. ^{1}H NMR spectra for **2**

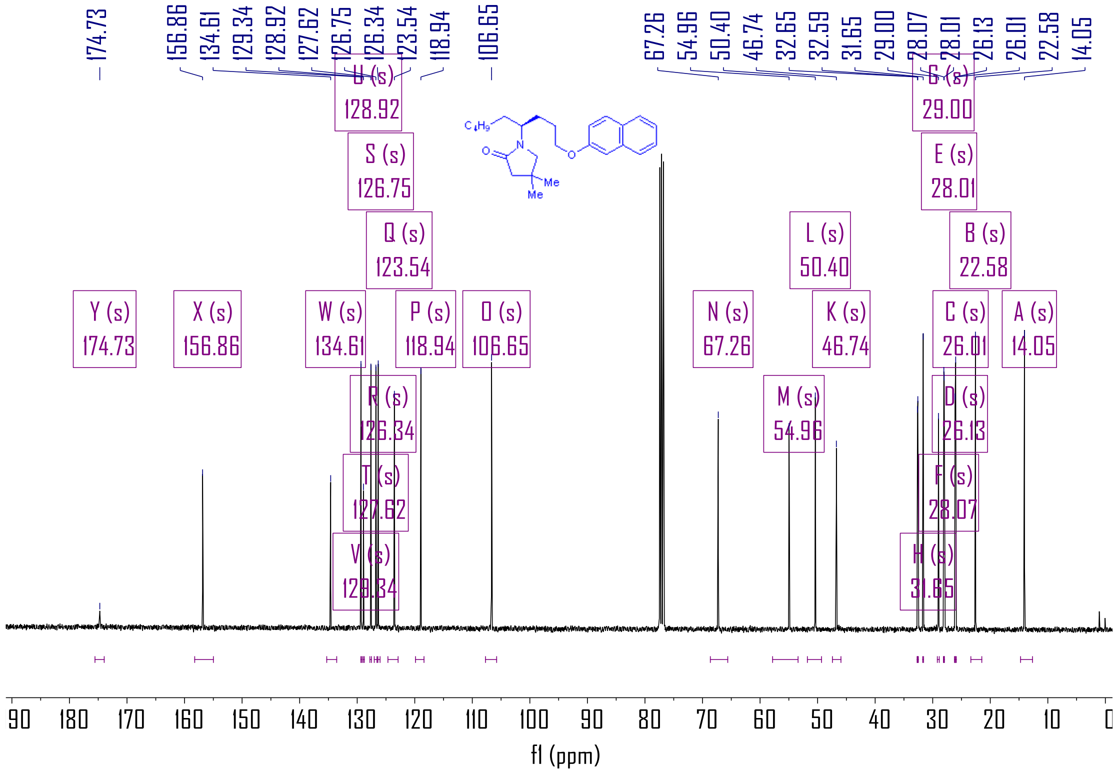

Supplementary Figure 119. ^{13}C NMR spectra for **2**

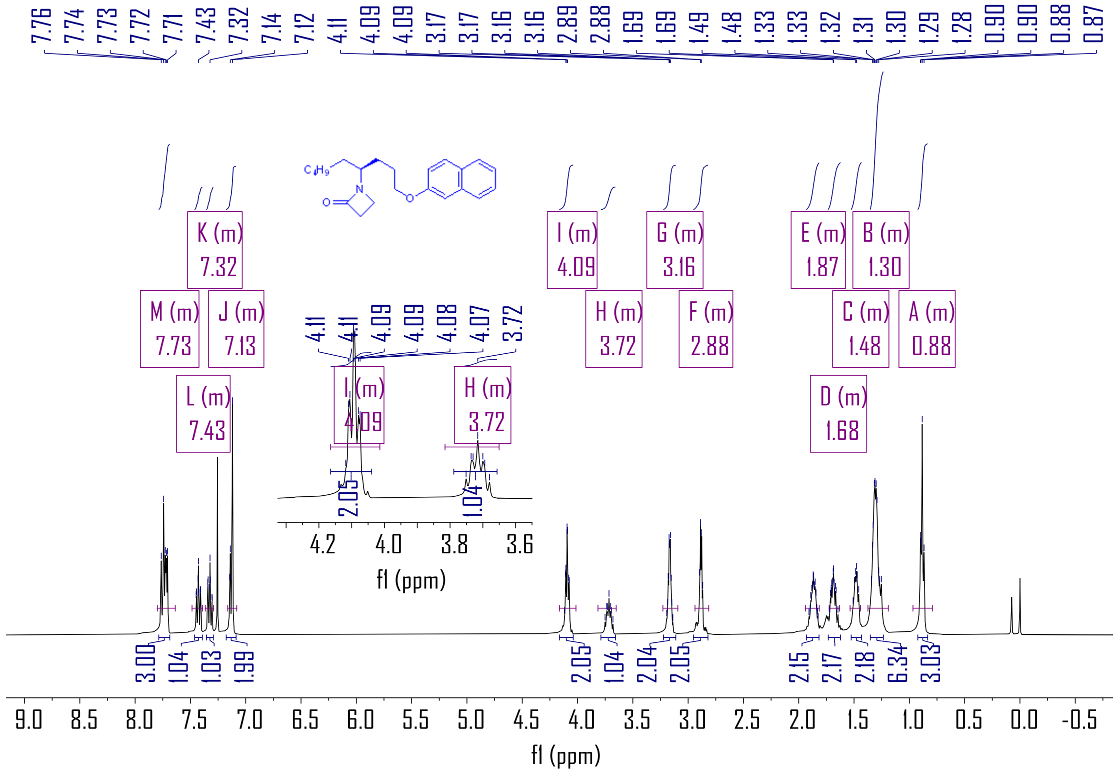

Supplementary Figure 120. ^{1}H NMR spectra for **3**

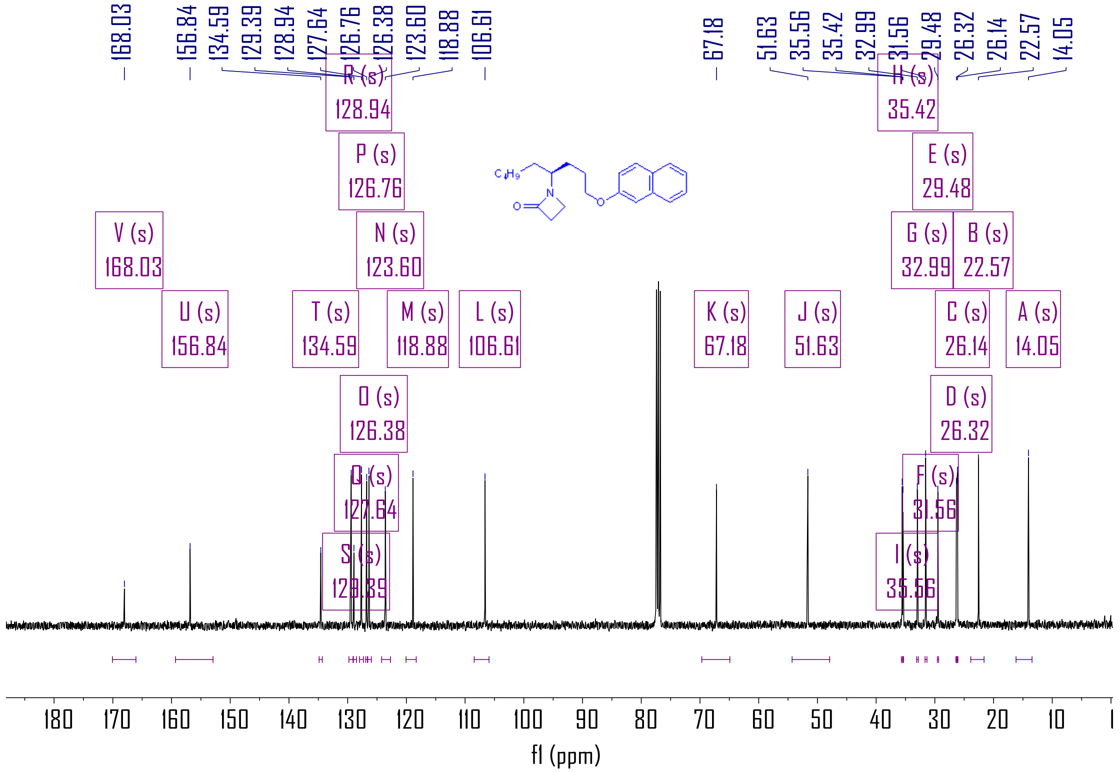

Supplementary Figure 121. ^{13}C NMR spectra for **3**

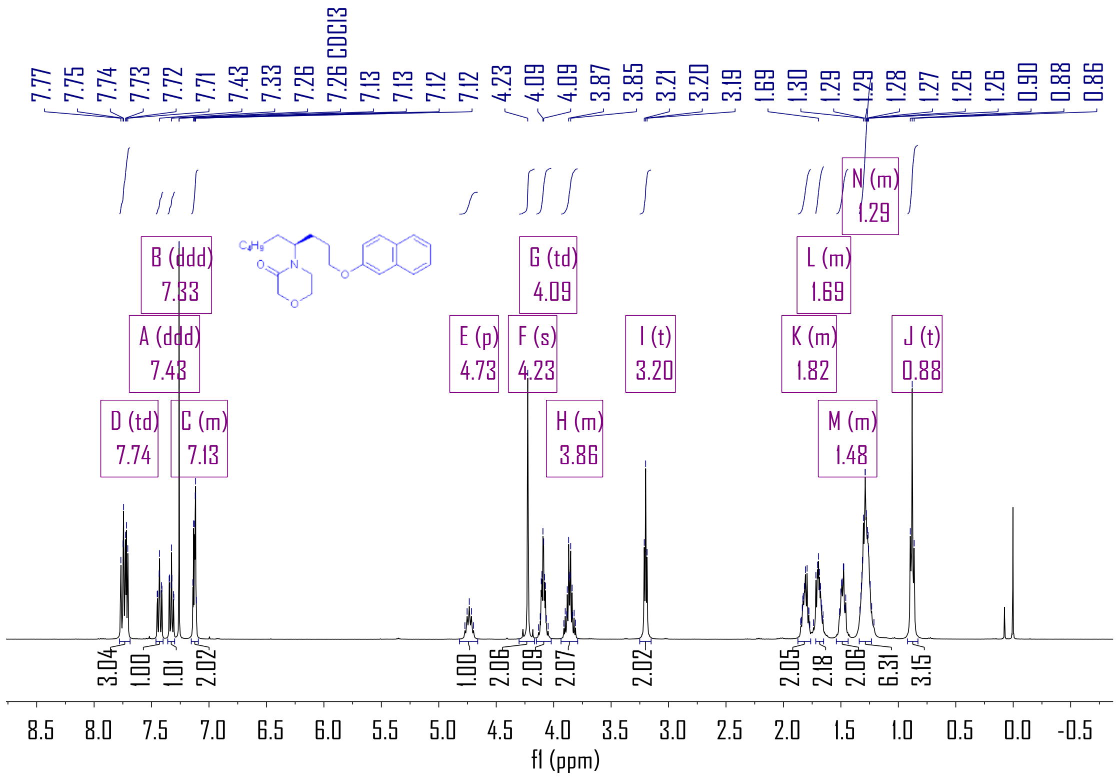

Supplementary Figure 122. ^{1}H NMR spectra for **4**

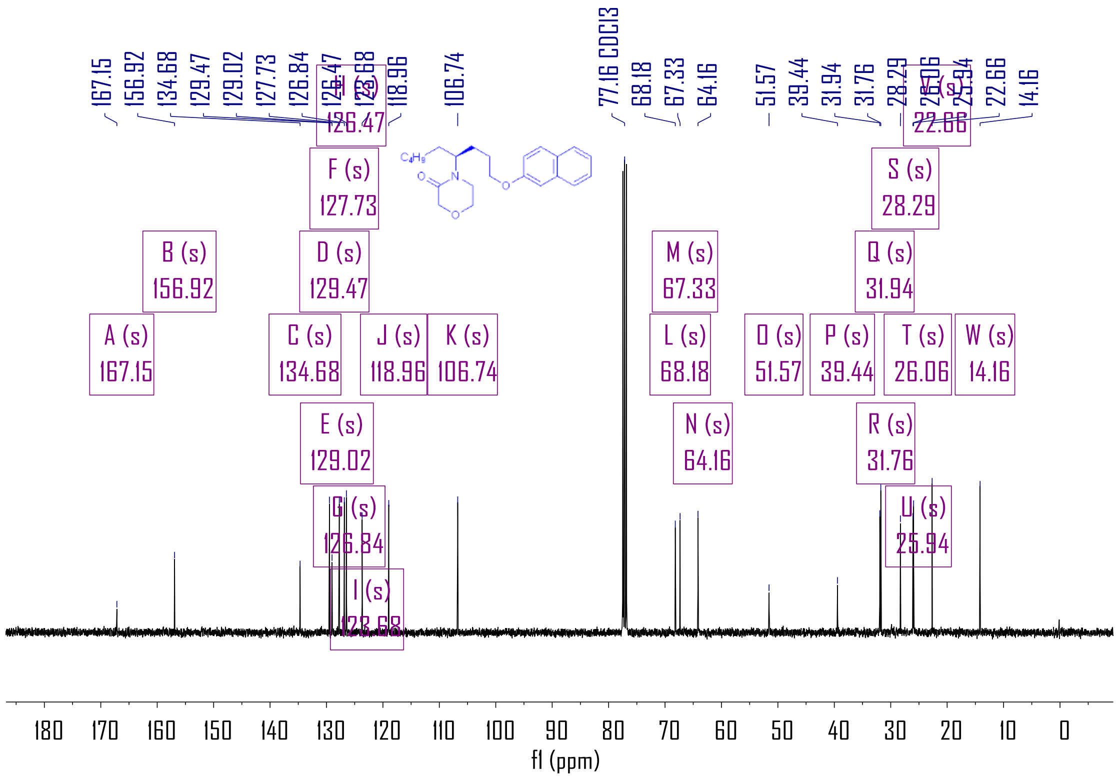

Supplementary Figure 123. ^{13}C NMR spectra for **4**

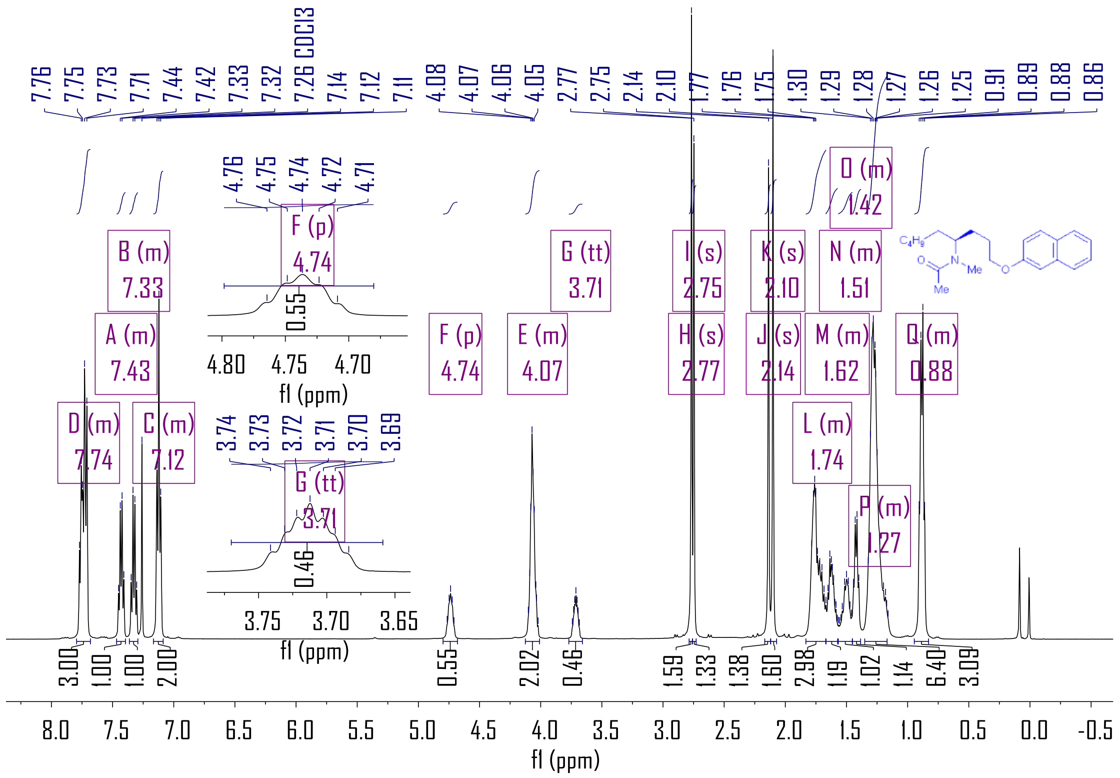

Supplementary Figure 124. ^{1}H NMR spectra for **5**

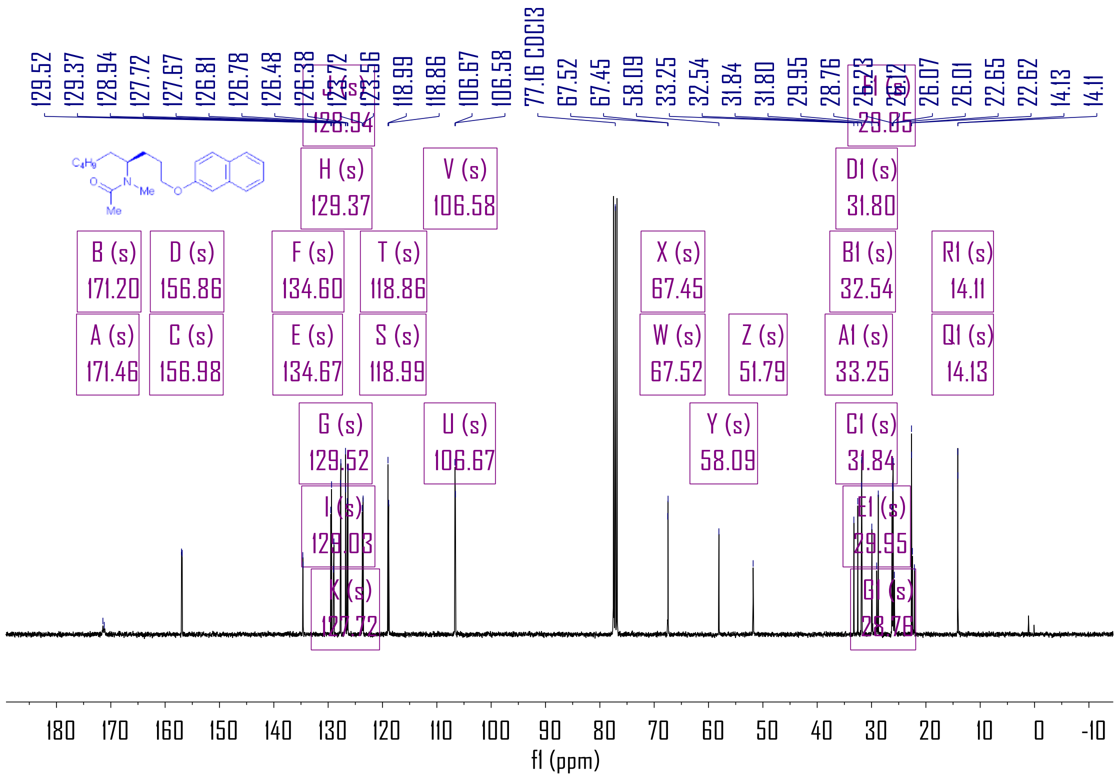

Supplementary Figure 125. ^{13}C NMR spectra for **5**

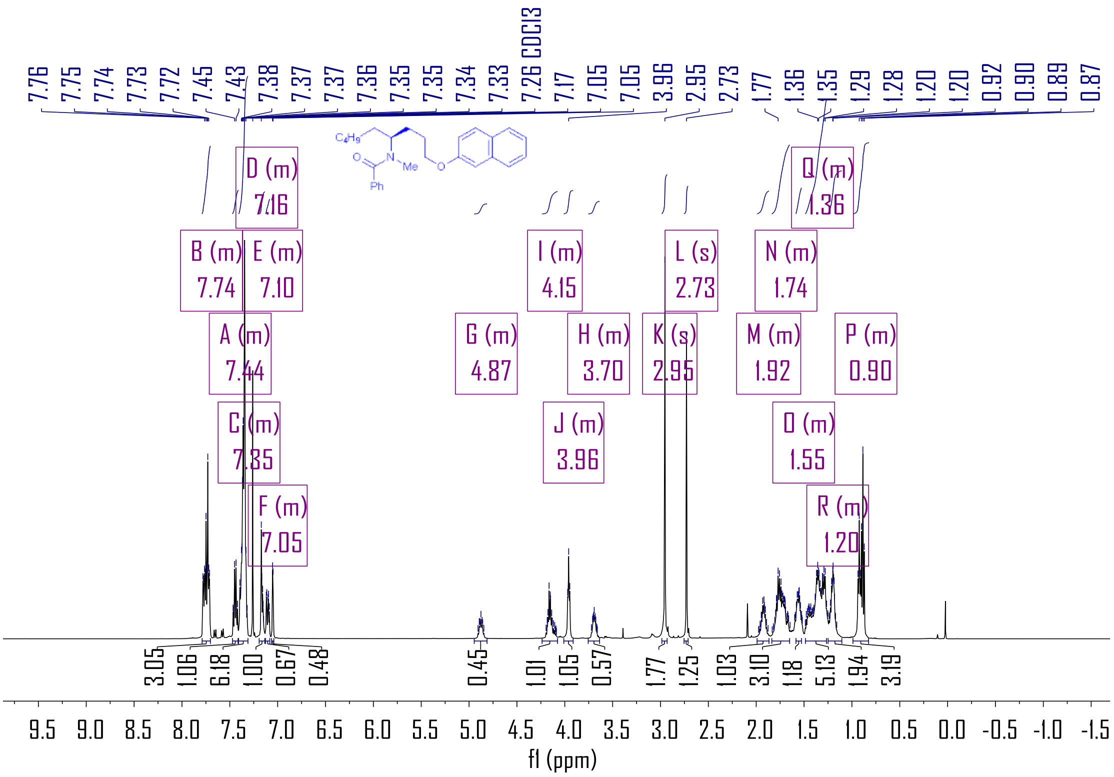

Supplementary Figure 126. ^{1}H NMR spectra for **6**

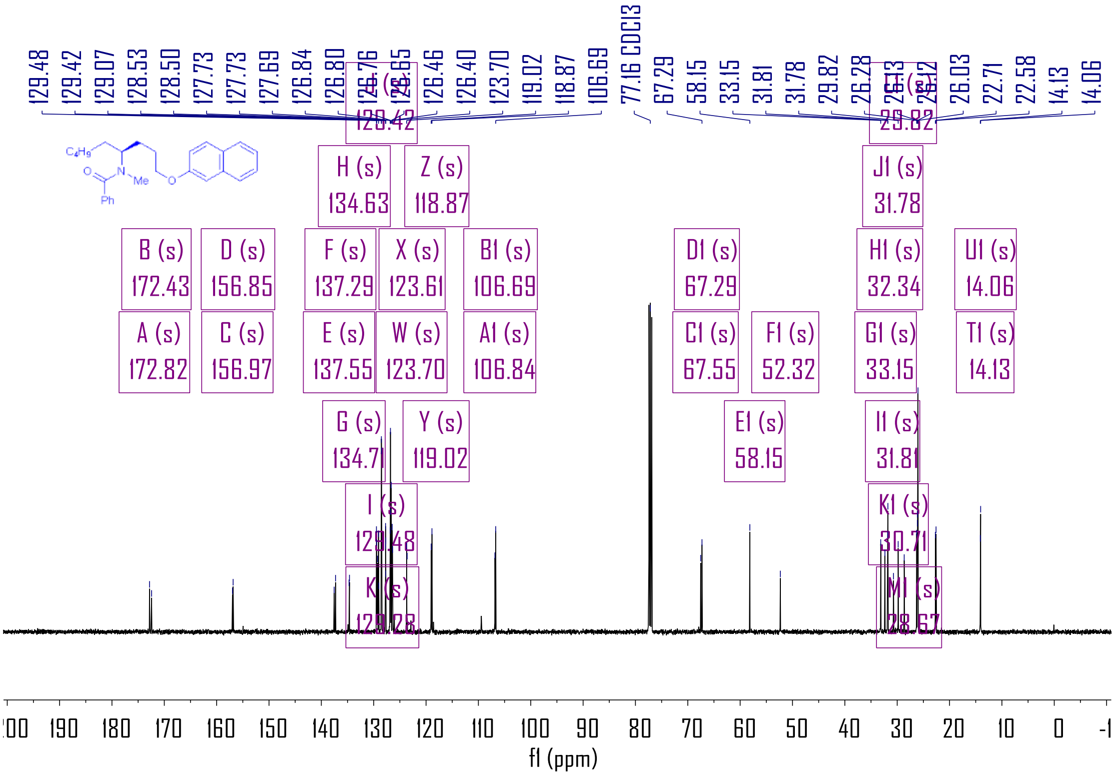

Supplementary Figure 127. ^{13}C NMR spectra for **6**

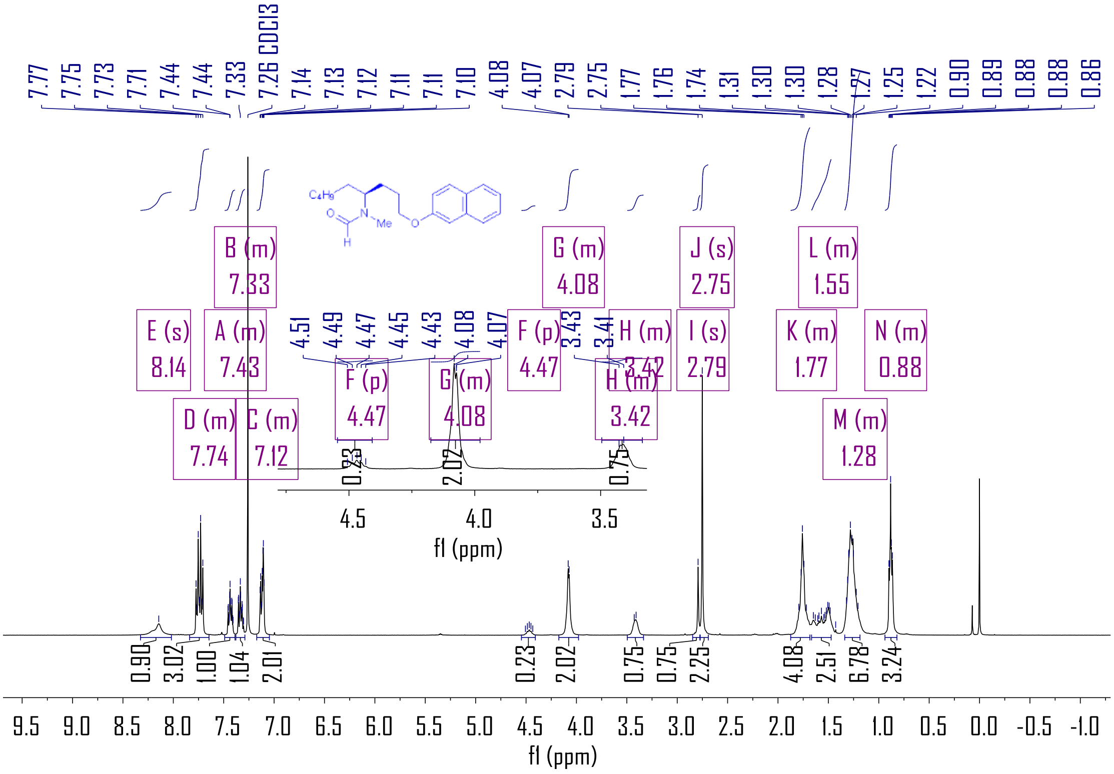

Supplementary Figure 128. ^{1}H NMR spectra for **7**

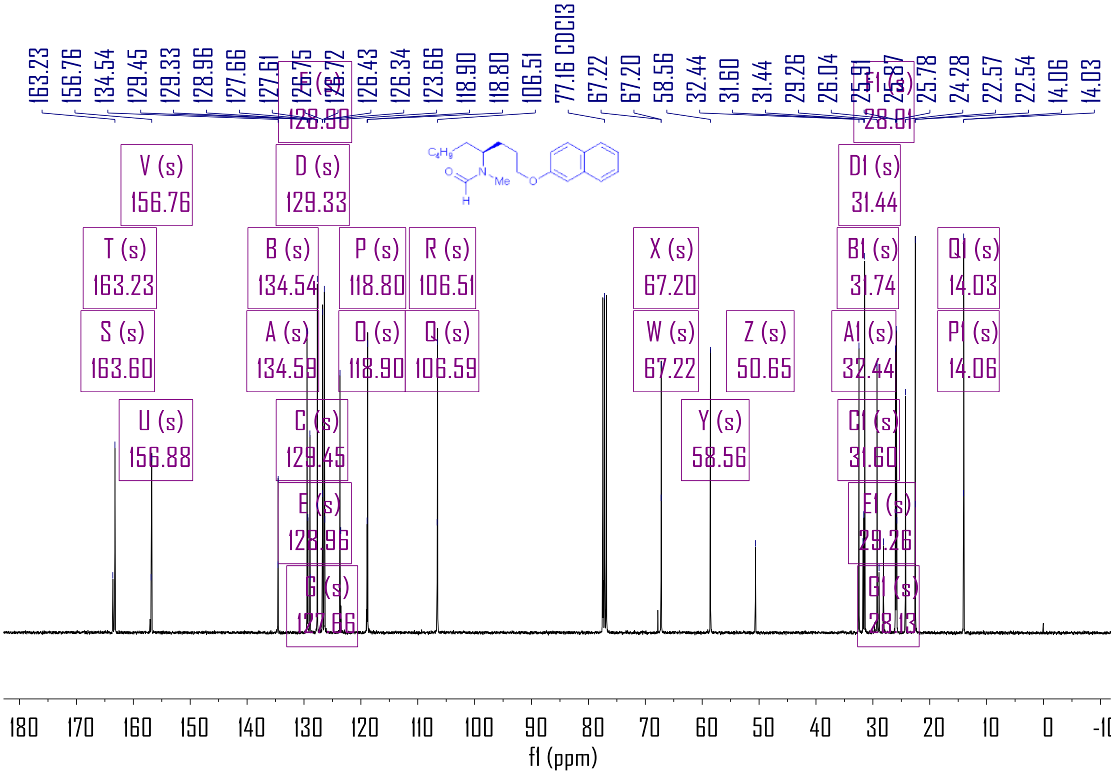

Supplementary Figure 129. ^{13}C NMR spectra for **7**

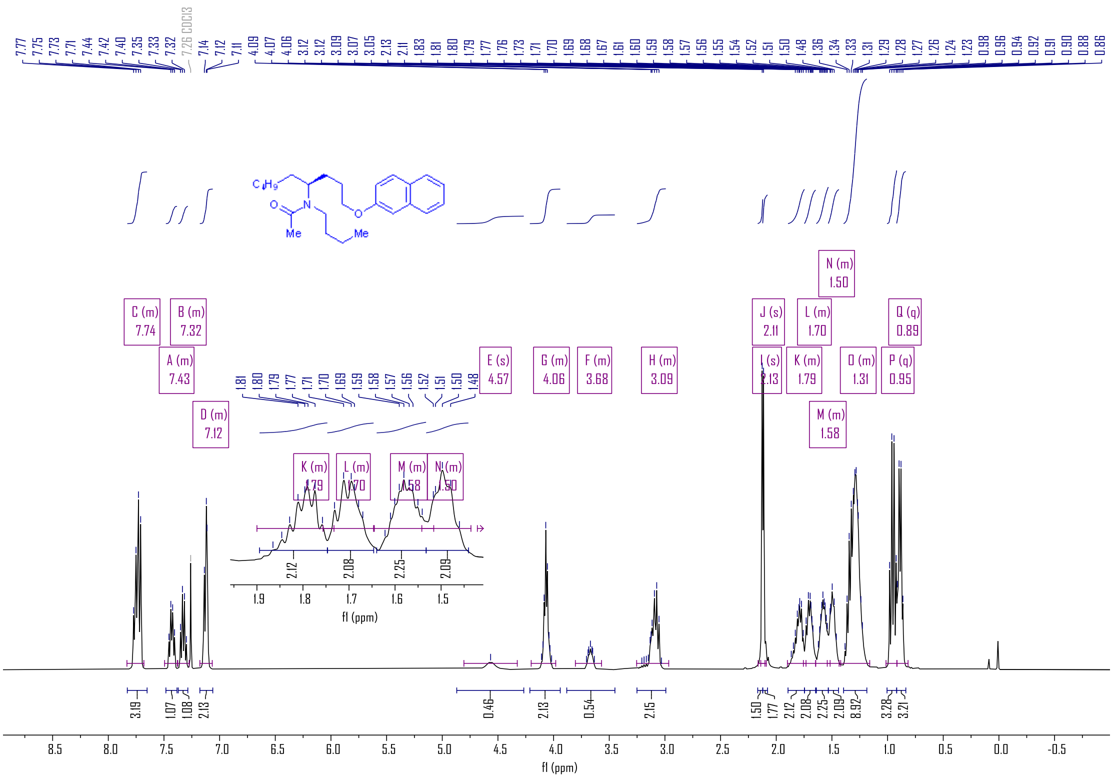

Supplementary Figure 130. ^{1}H NMR spectra for **8**

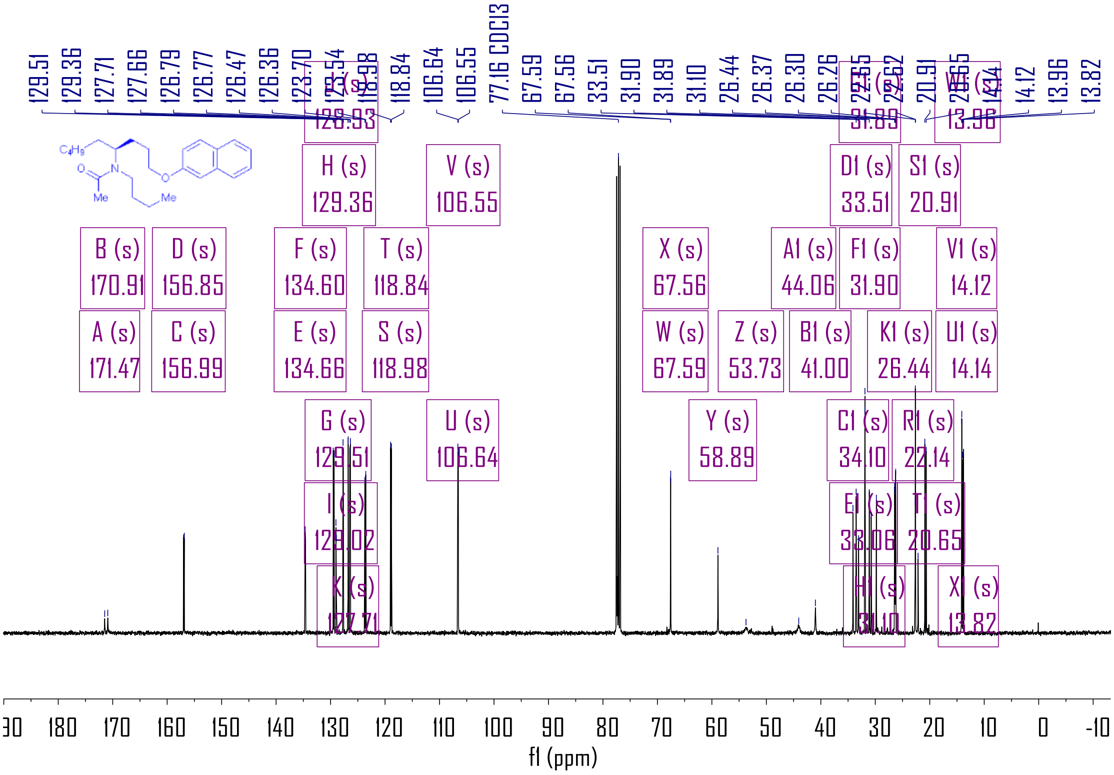

Supplementary Figure 131. ^{13}C NMR spectra for **8**

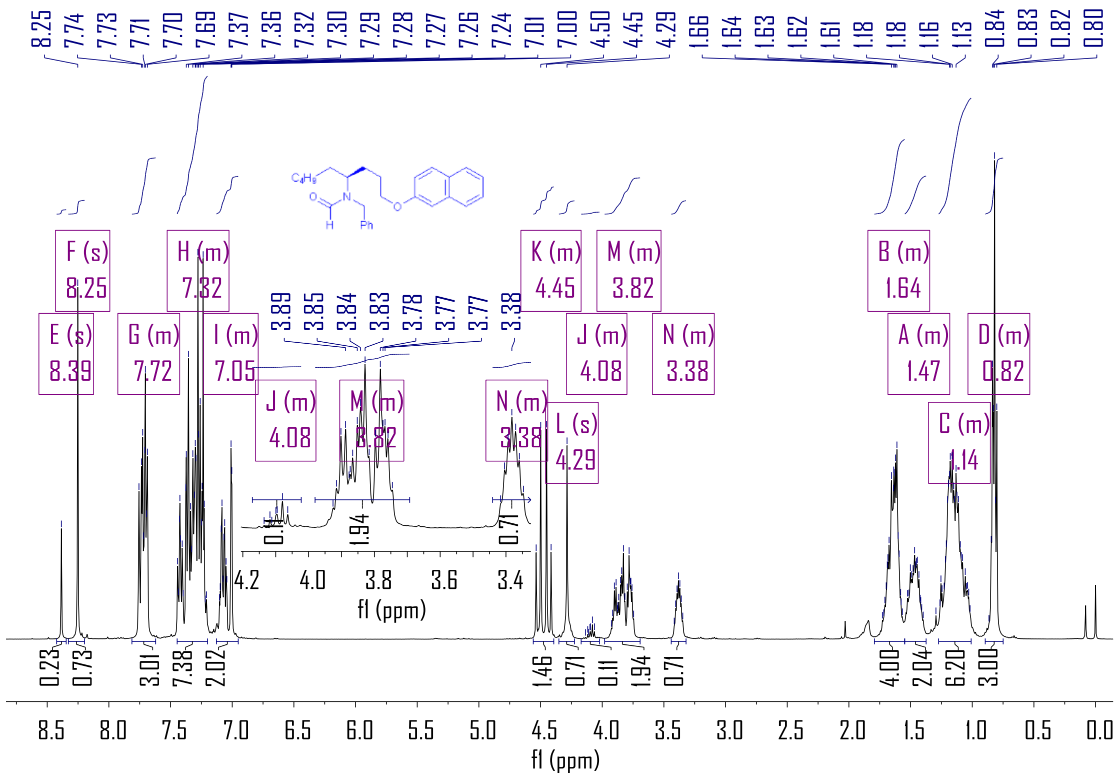

Supplementary Figure 132. ^{1}H NMR spectra for **9**

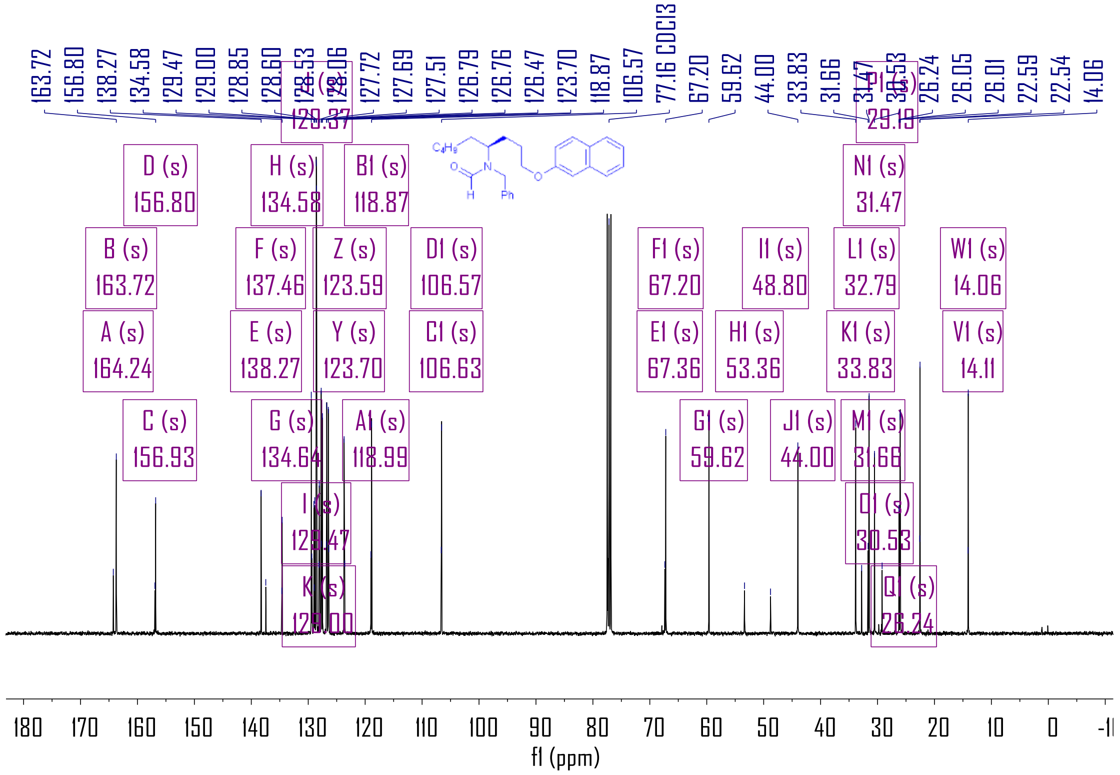

Supplementary Figure 133. ^{13}C NMR spectra for **9**

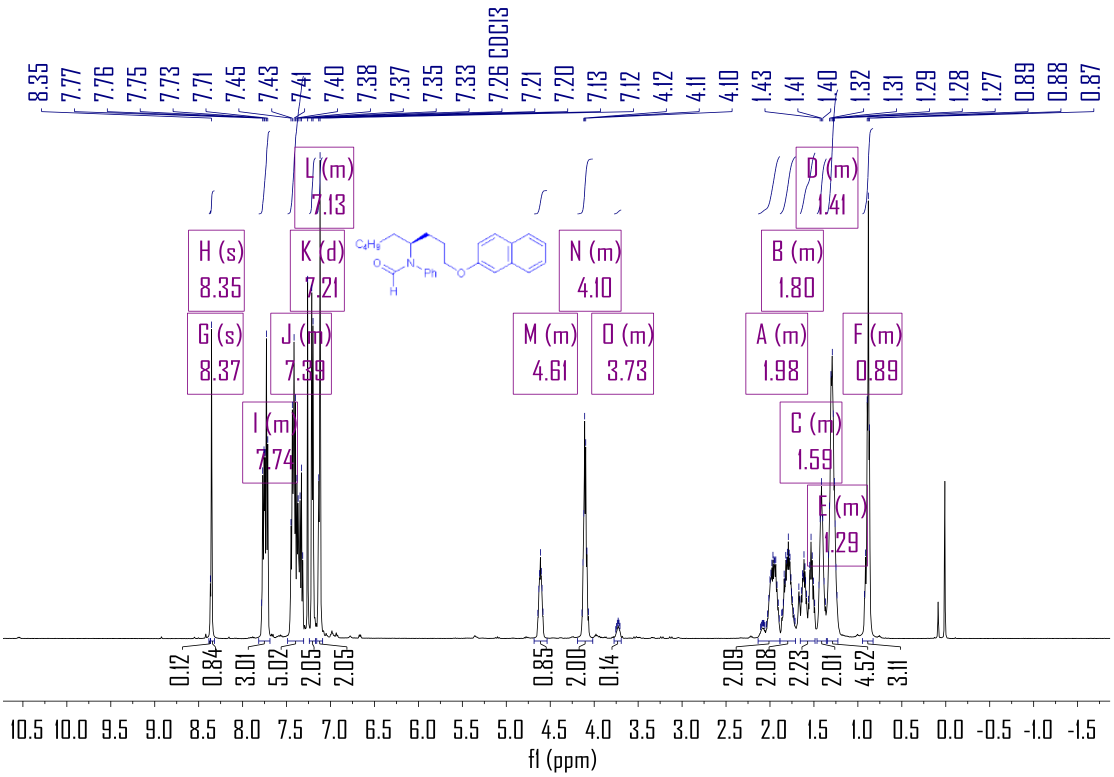

Supplementary Figure 134. ^{1}H NMR spectra for **10**

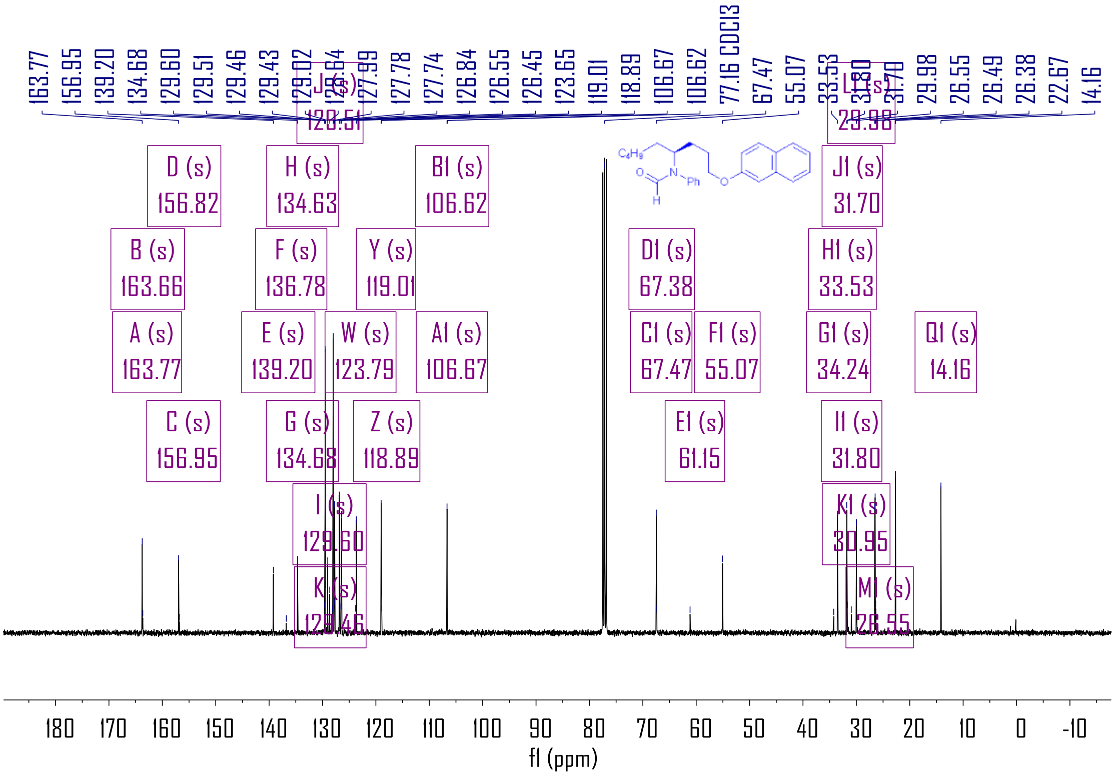

Supplementary Figure 135. ^{13}C NMR spectra for **10**

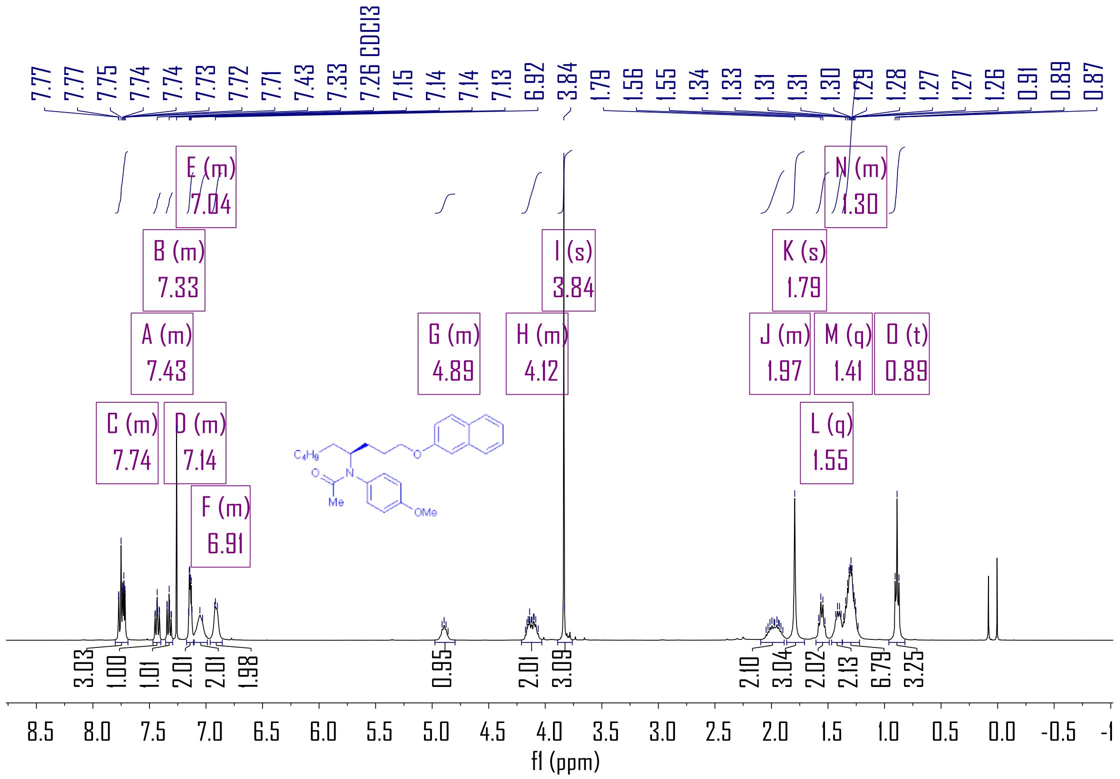

Supplementary Figure 136. ^{1}H NMR spectra for **11**

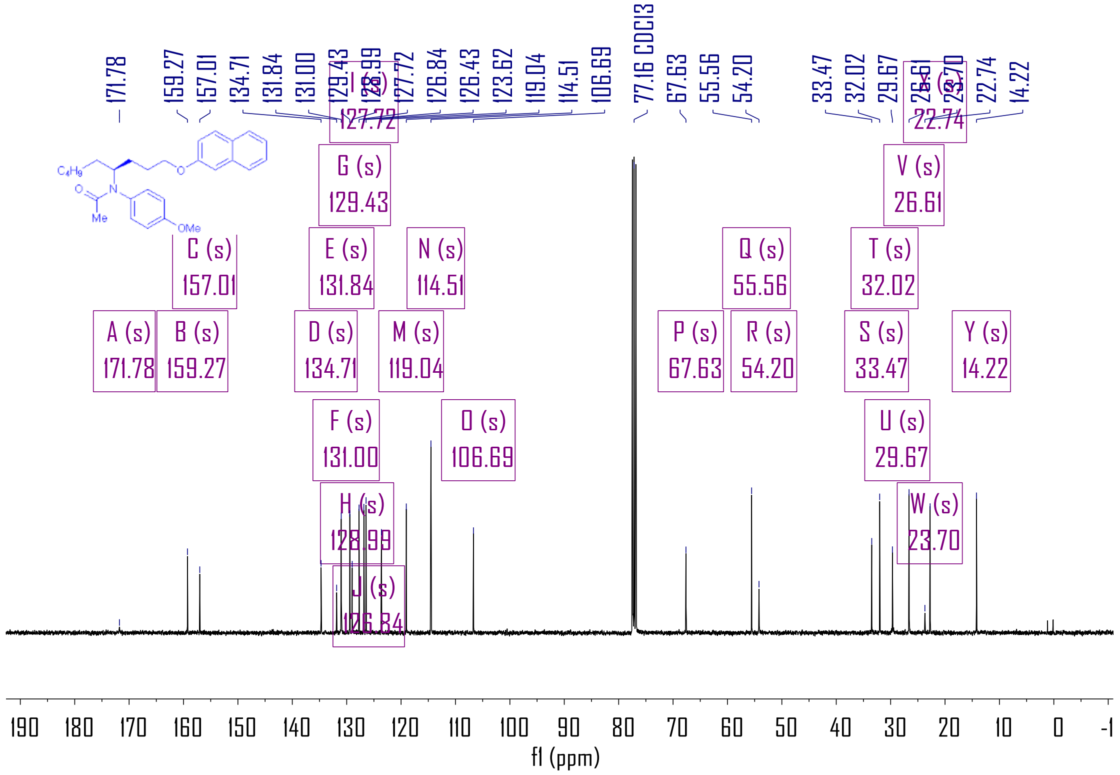

Supplementary Figure 137. ^{13}C NMR spectra for **11**

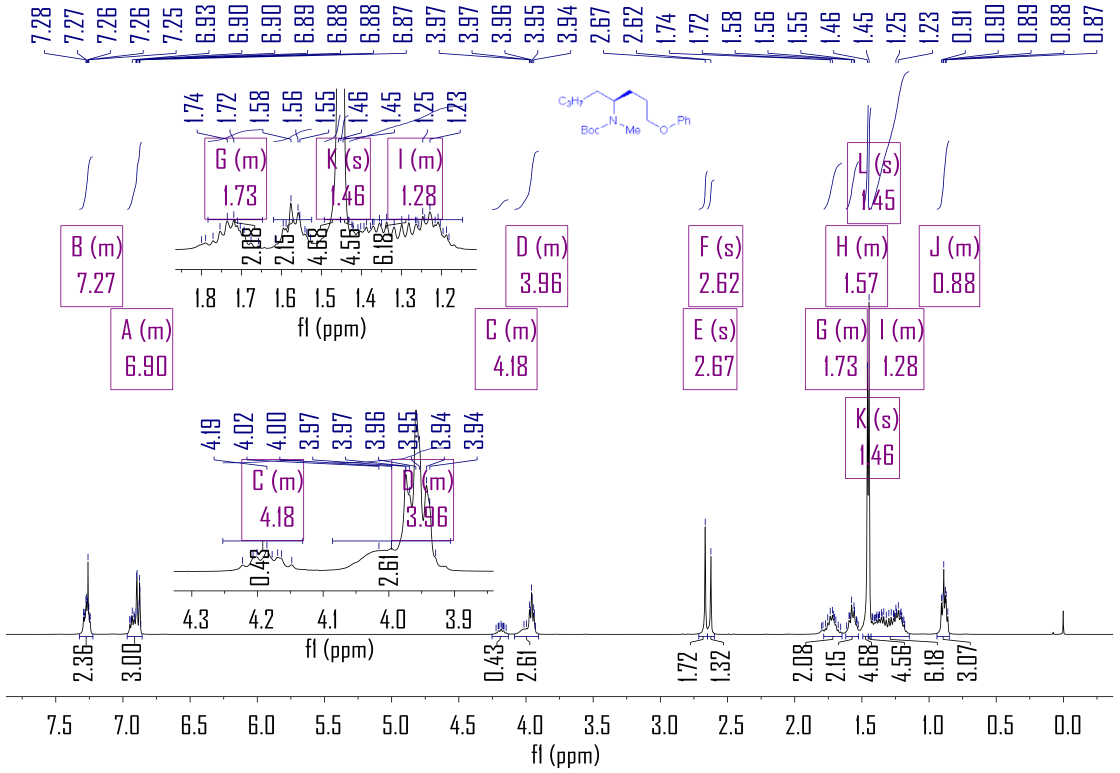

Supplementary Figure 138. ^{1}H NMR spectra for **12**

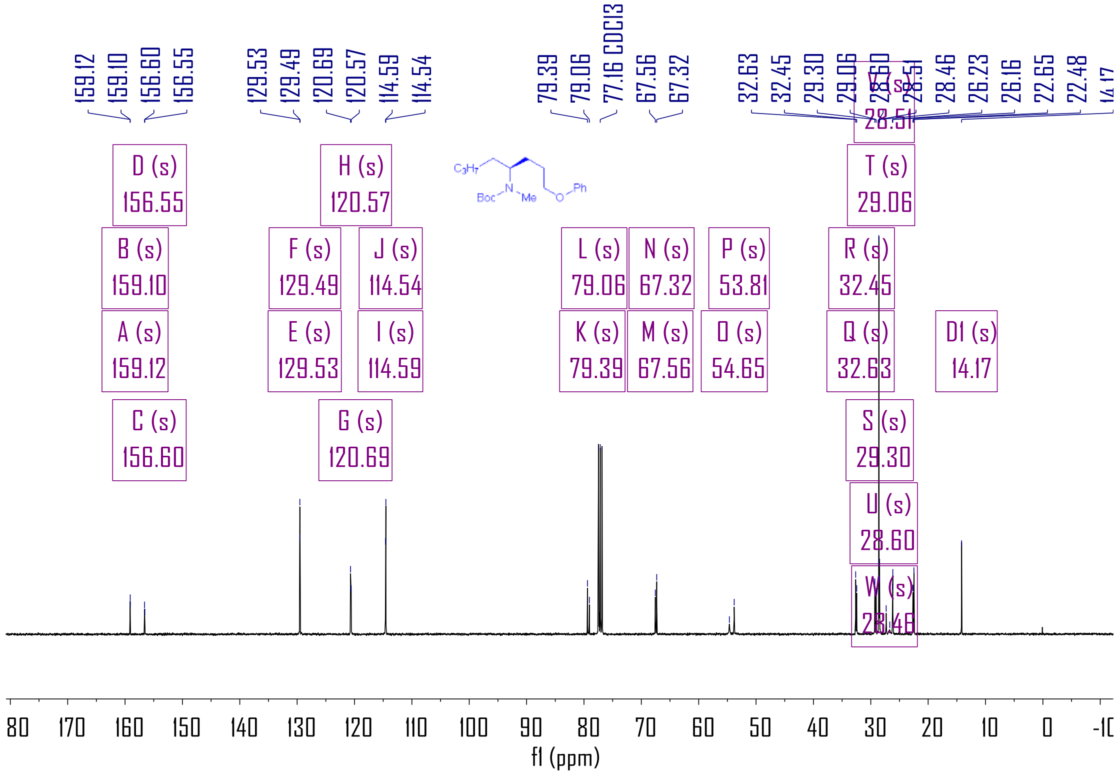

Supplementary Figure 139. ^{13}C NMR spectra for **12**

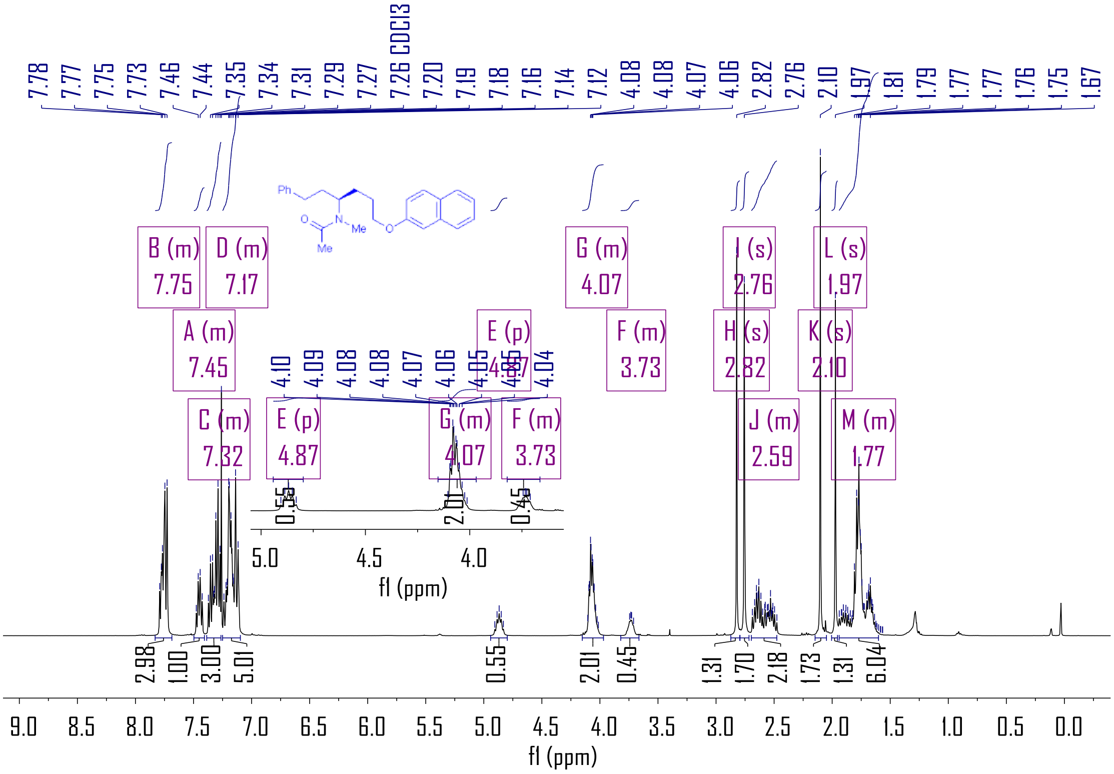

Supplementary Figure 140. ^{1}H NMR spectra for **13**

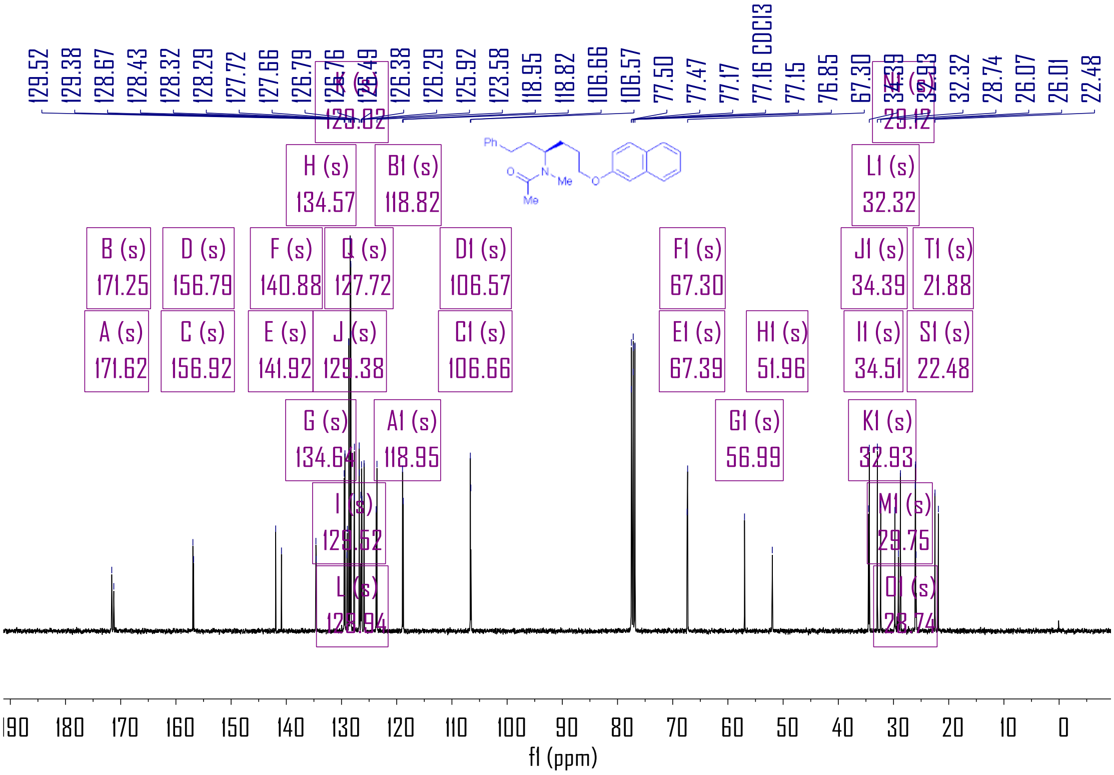

Supplementary Figure 141. ^{13}C NMR spectra for **13**

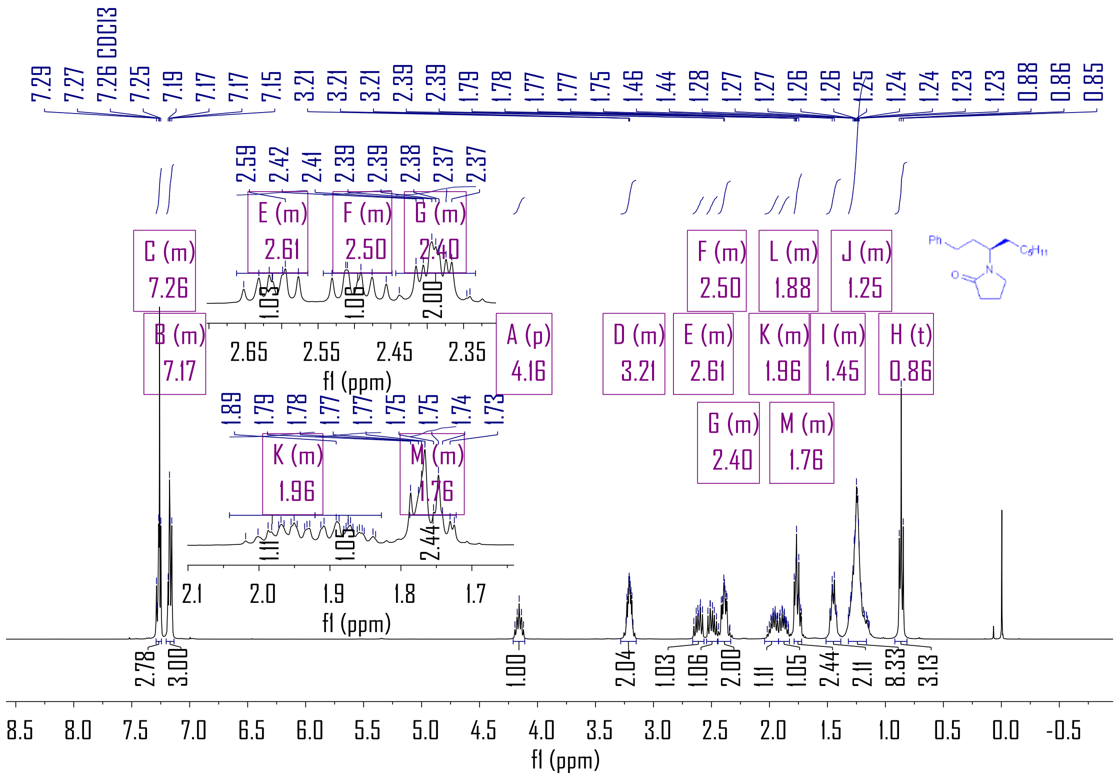

Supplementary Figure 142. ^{1}H NMR spectra for **14**

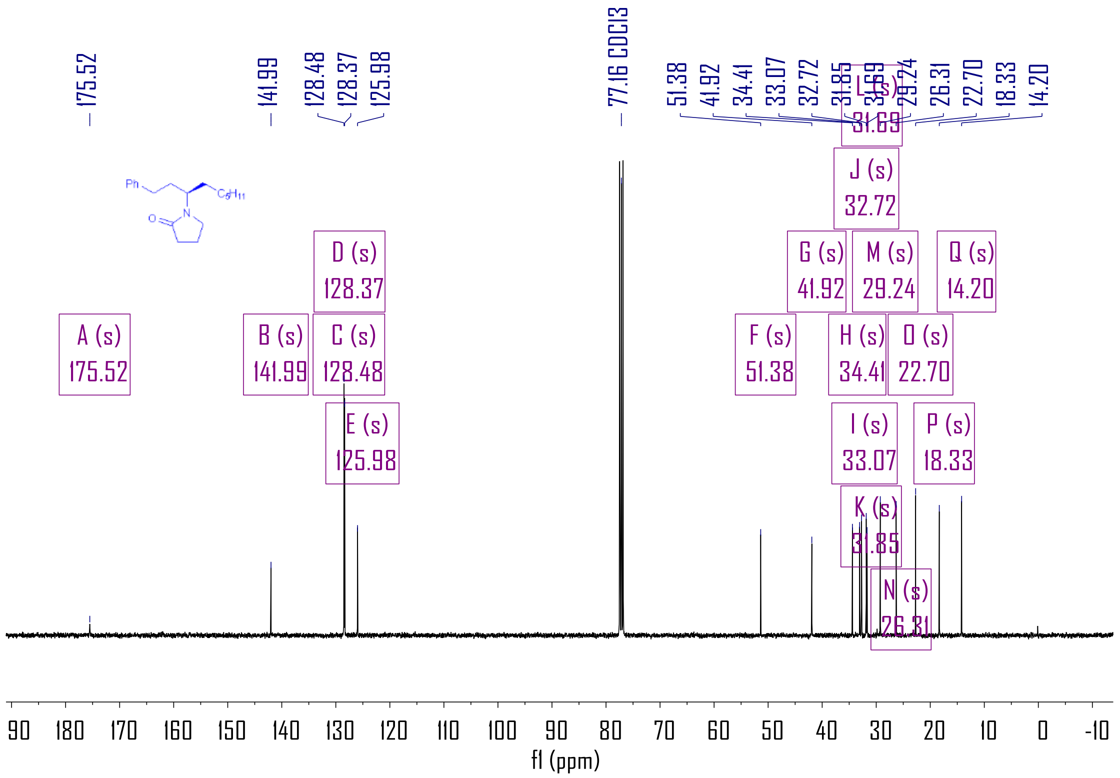

Supplementary Figure 143. ^{13}C NMR spectra for **14**

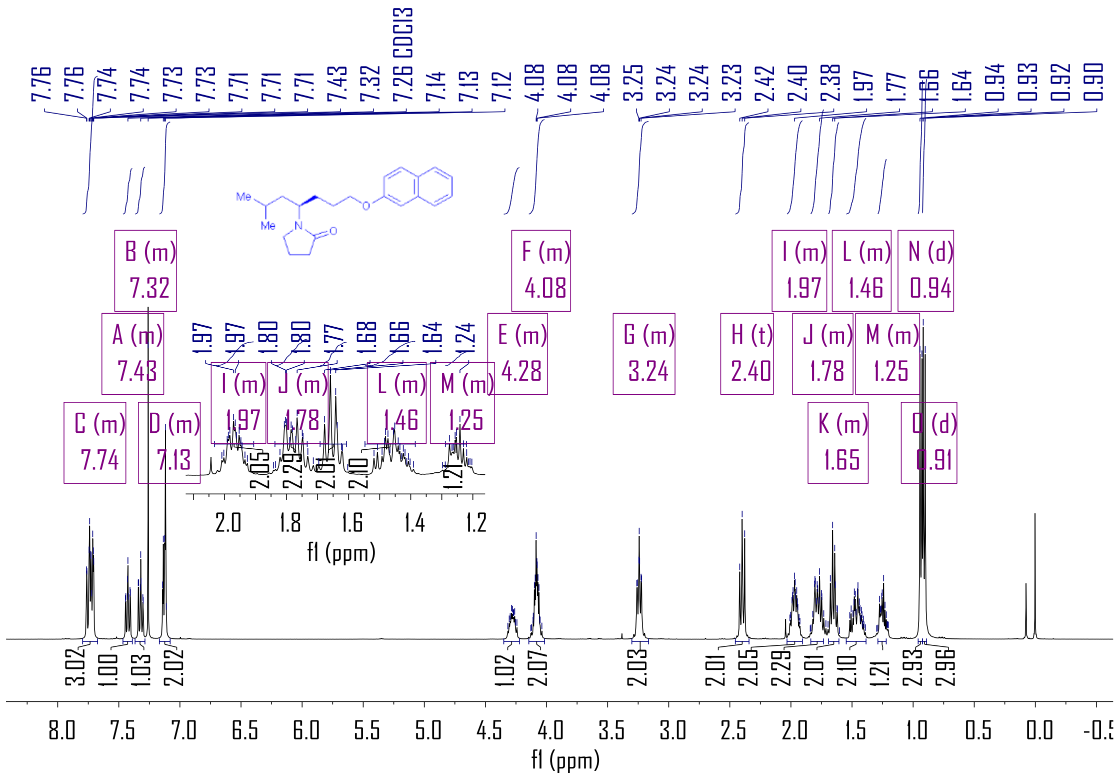

Supplementary Figure 144. ^{1}H NMR spectra for **15**

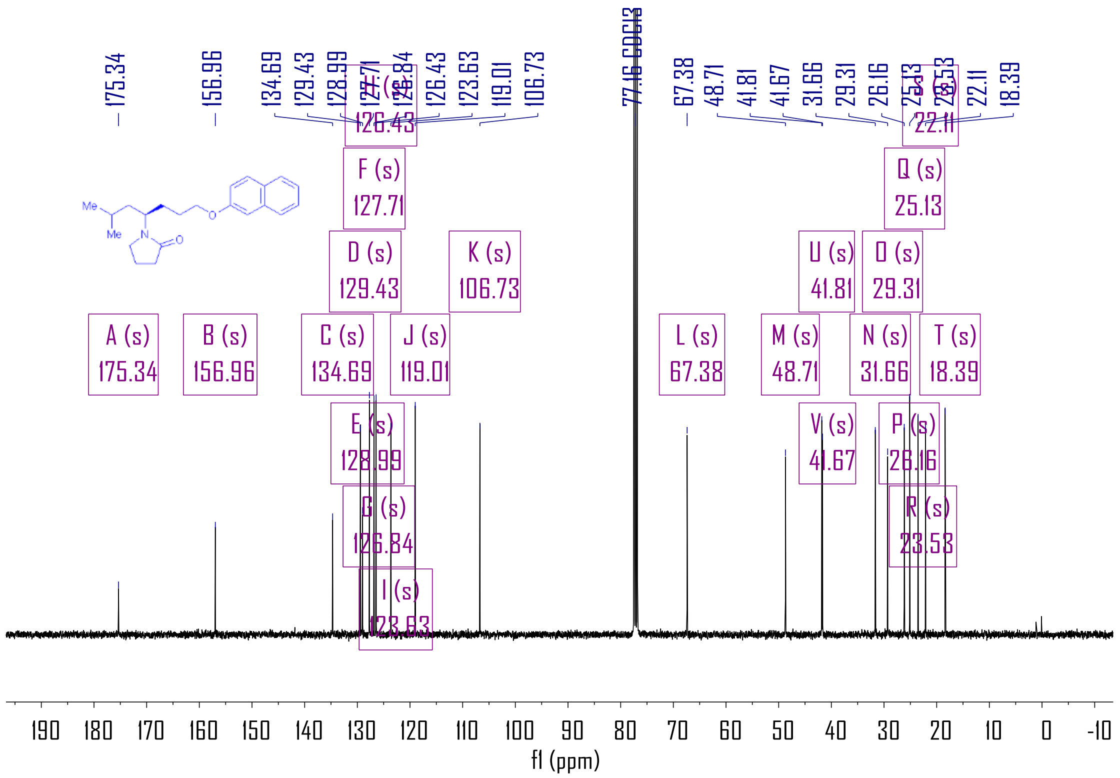

Supplementary Figure 145. ^{13}C NMR spectra for **15**

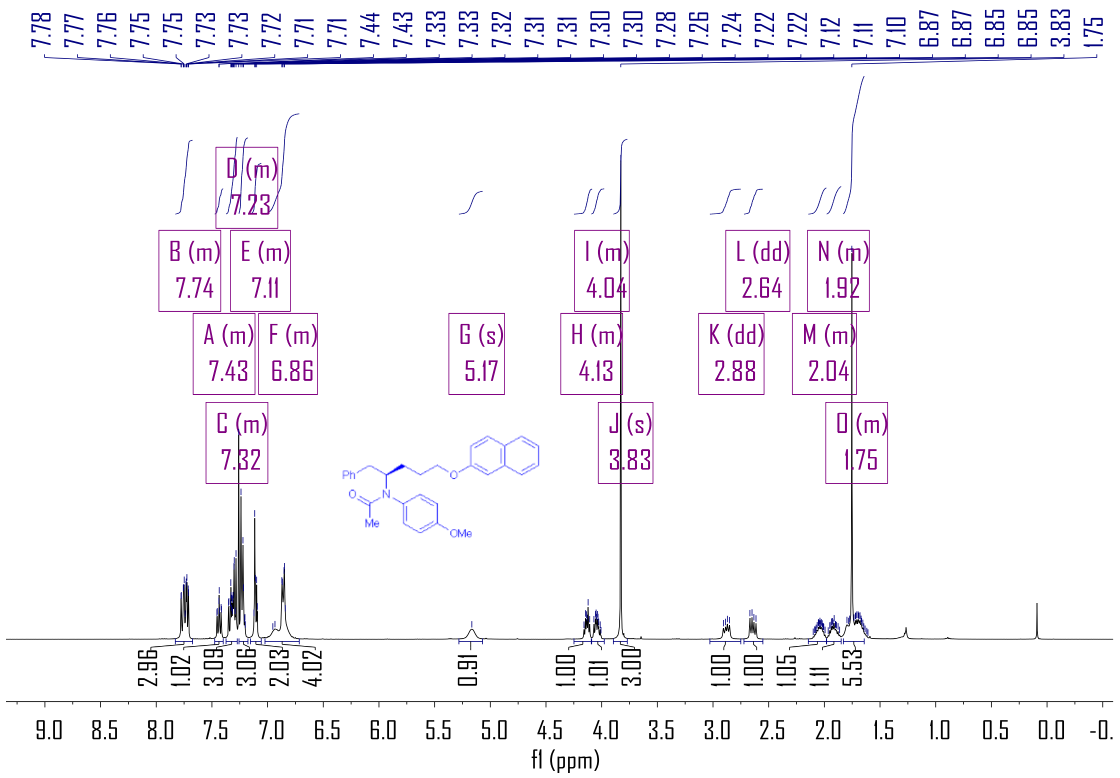

Supplementary Figure 146. ^{1}H NMR spectra for **16**

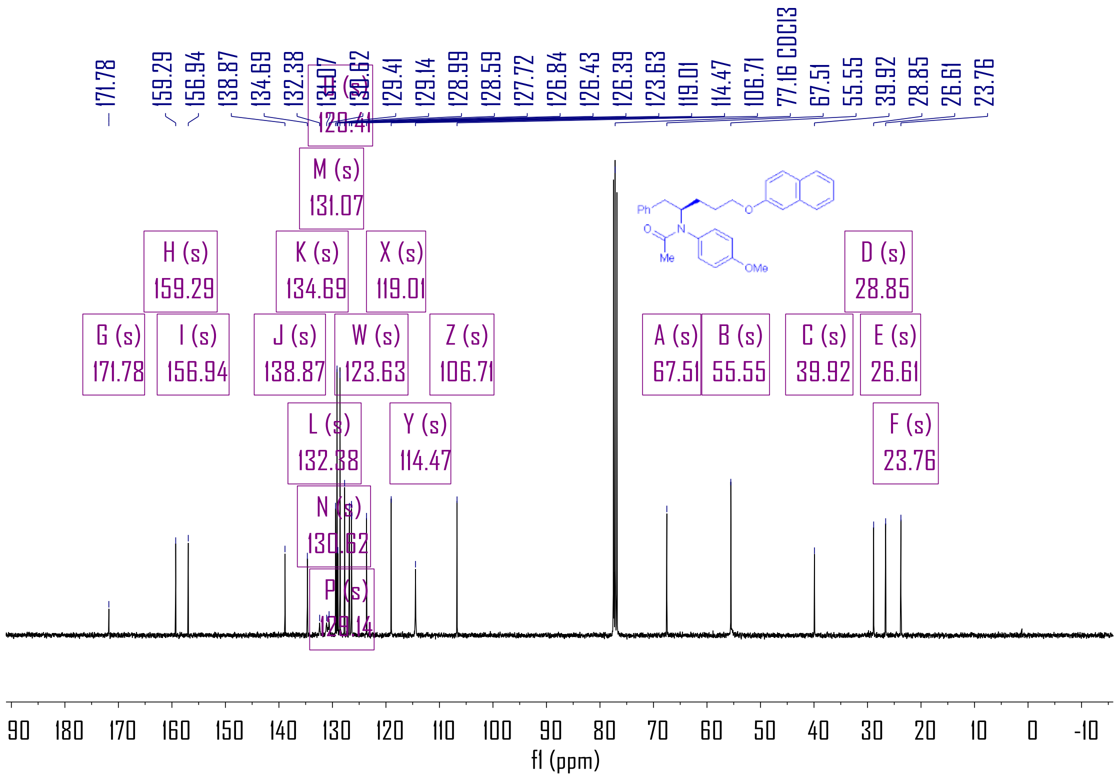

Supplementary Figure 147. ^{13}C NMR spectra for **16**

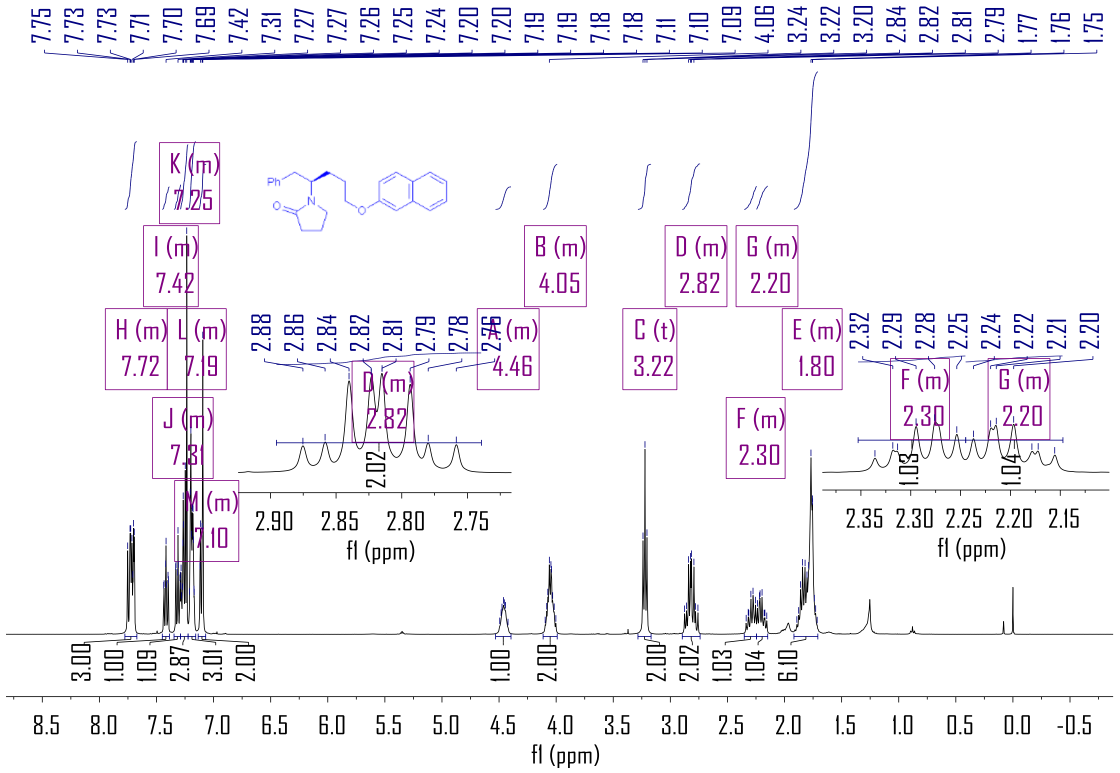

Supplementary Figure 148. ^{1}H NMR spectra for **17**

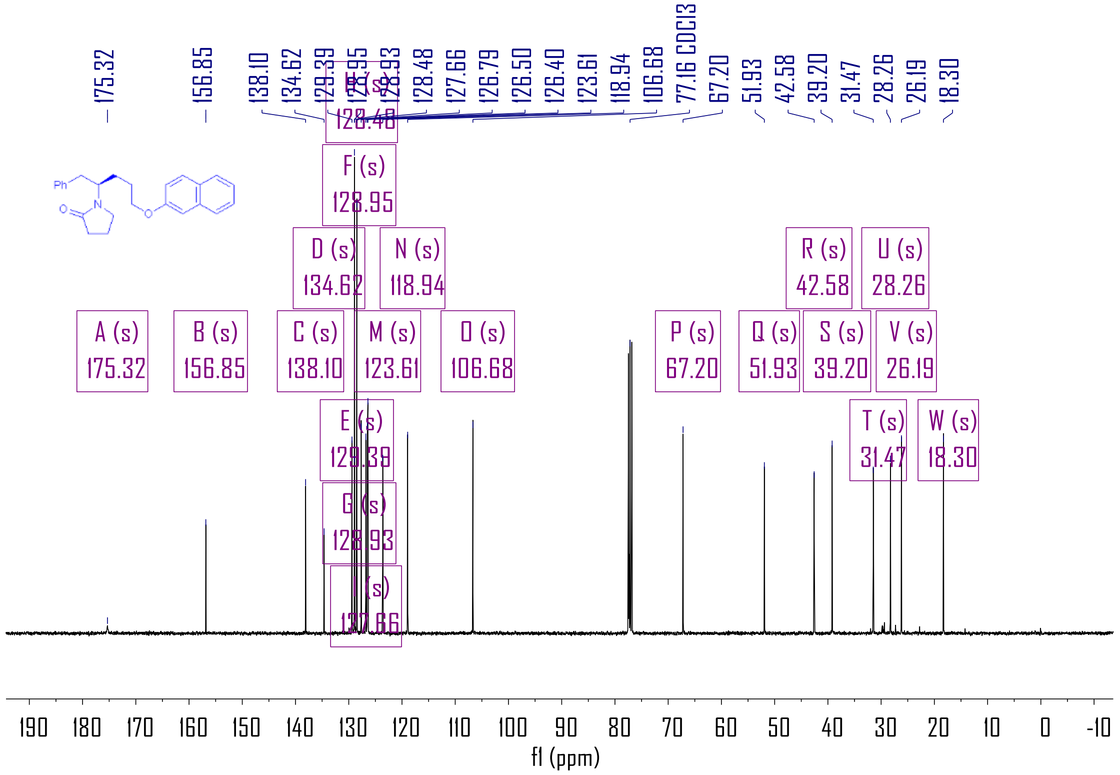

Supplementary Figure 149. ^{13}C NMR spectra for **17**

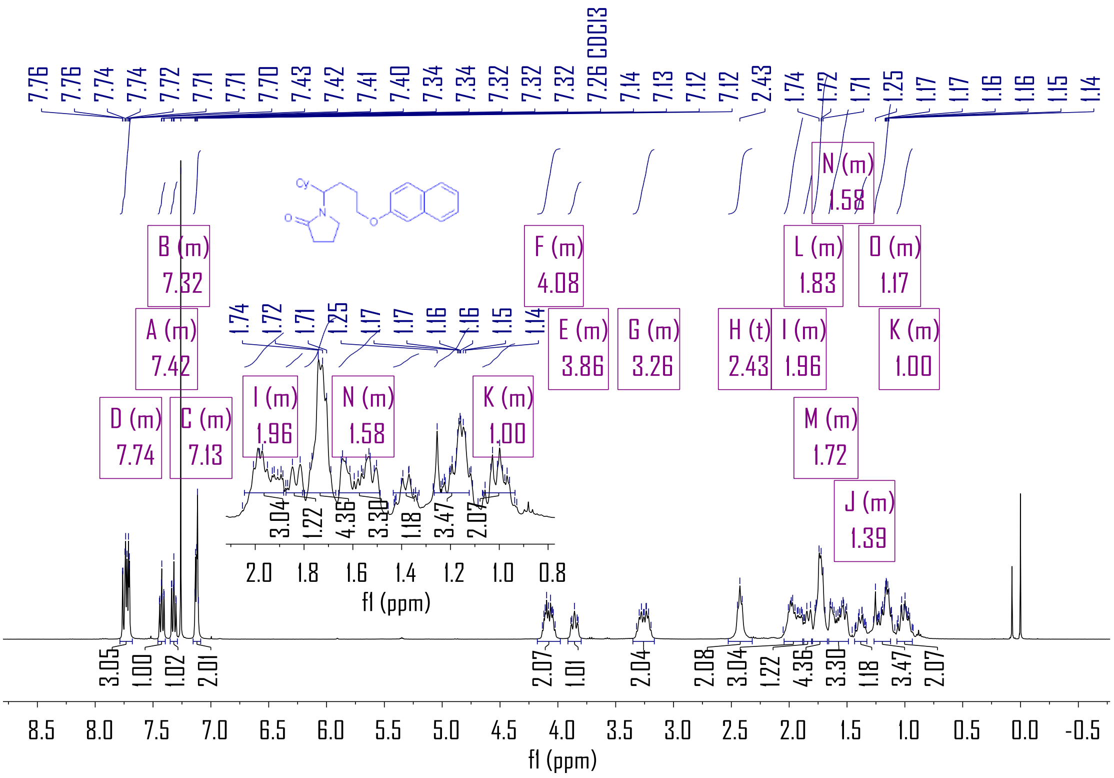

Supplementary Figure 150. ^{1}H NMR spectra for **18**

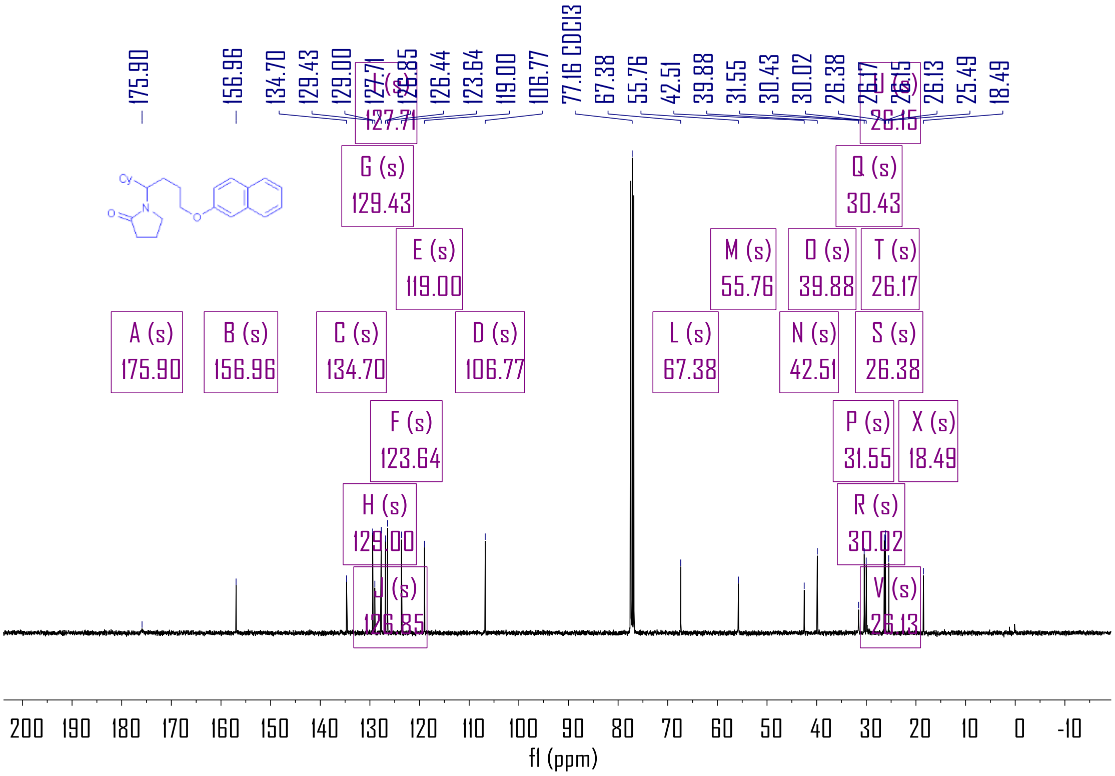

Supplementary Figure 151. ^{13}C NMR spectra for **18**

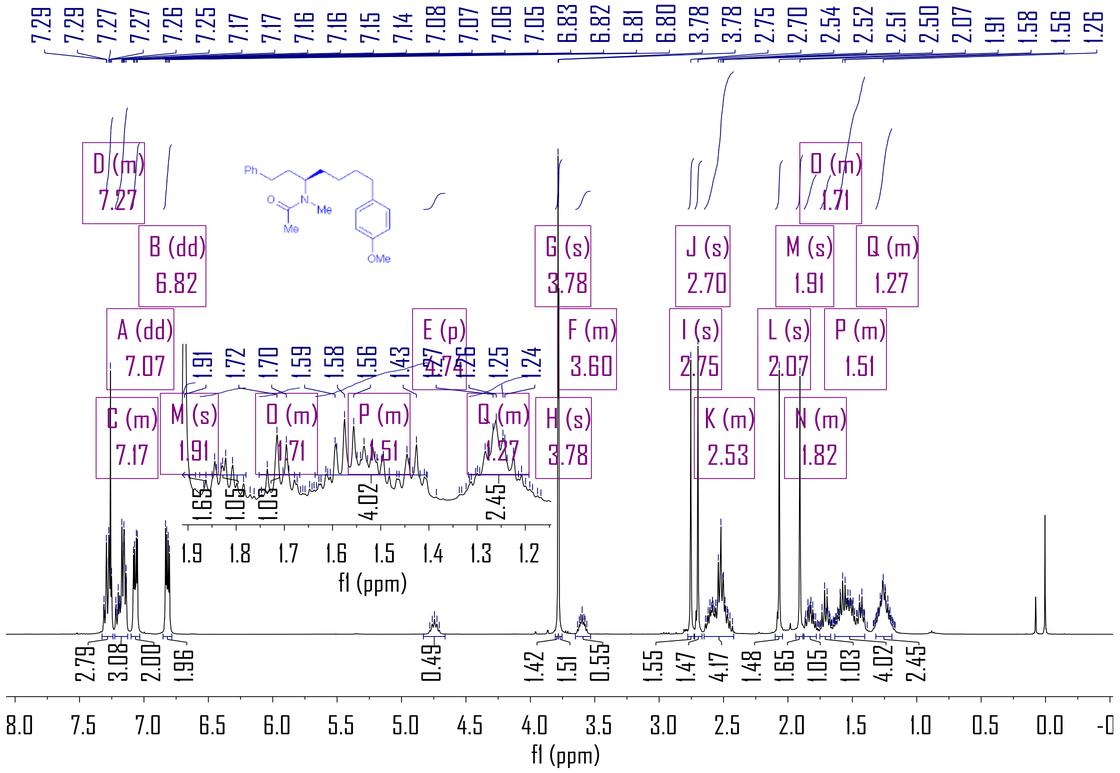

Supplementary Figure 152. ^{1}H NMR spectra for **19**

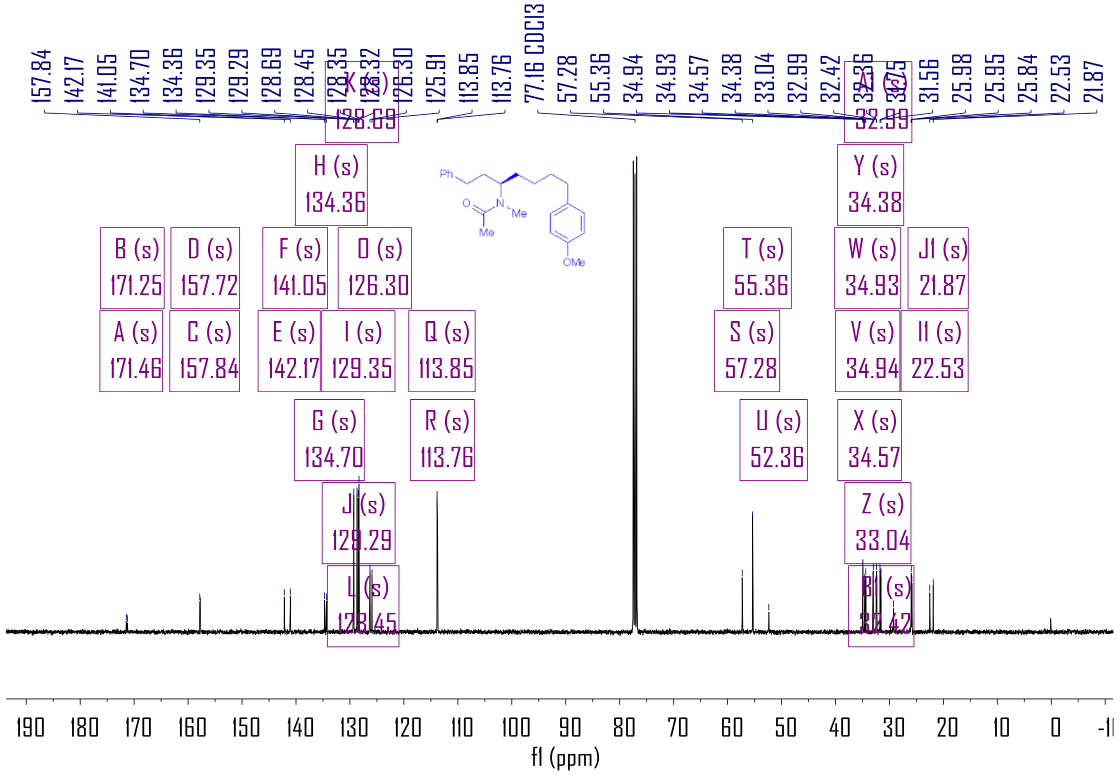

Supplementary Figure 153. ^{13}C NMR spectra for **19**

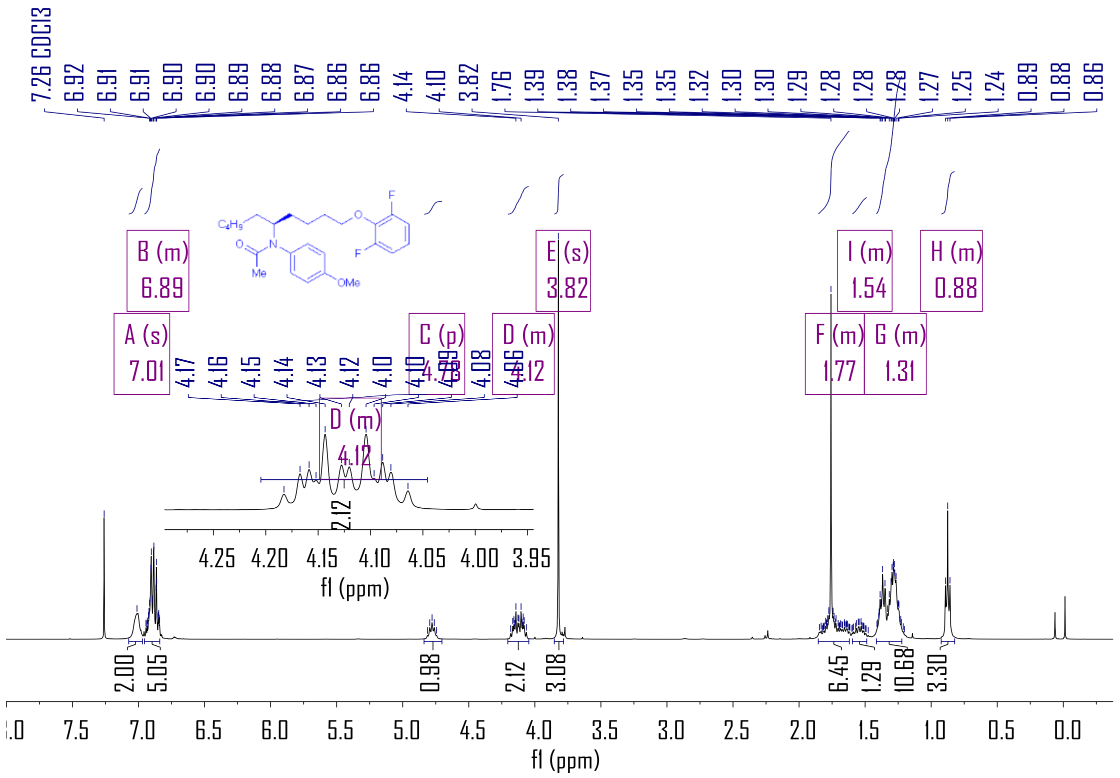

Supplementary Figure 154. ^{1}H NMR spectra for **20**

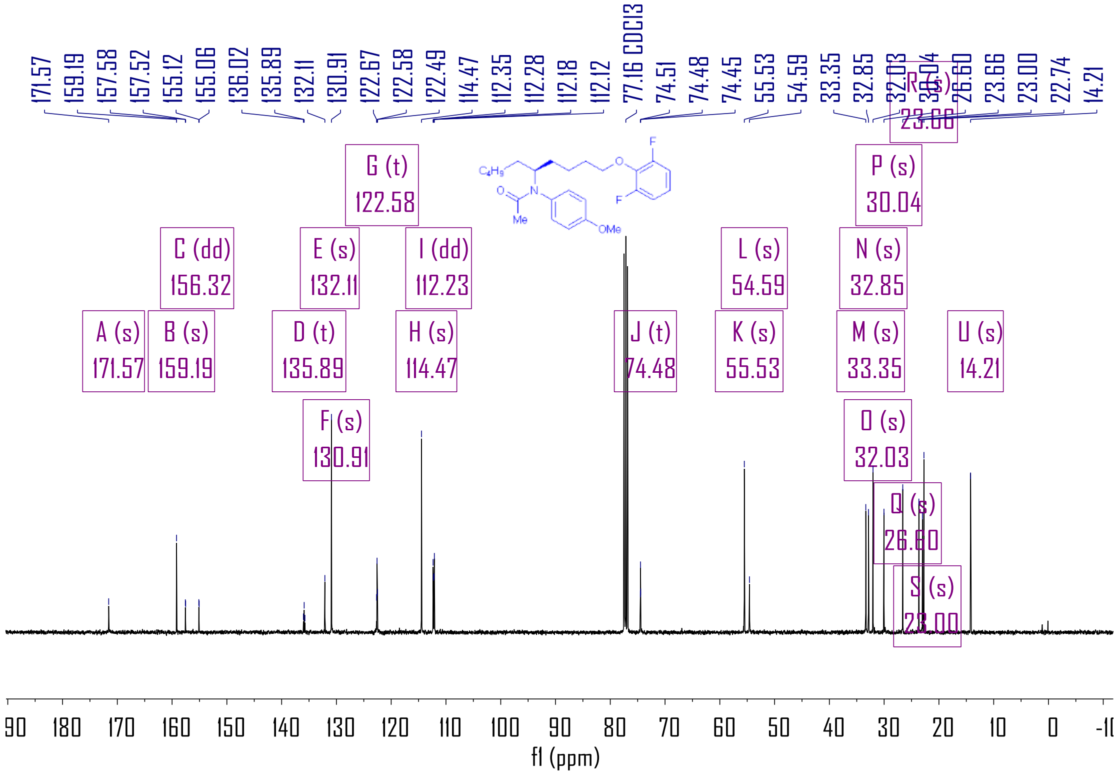

Supplementary Figure 155. ^{13}C NMR spectra for **20**

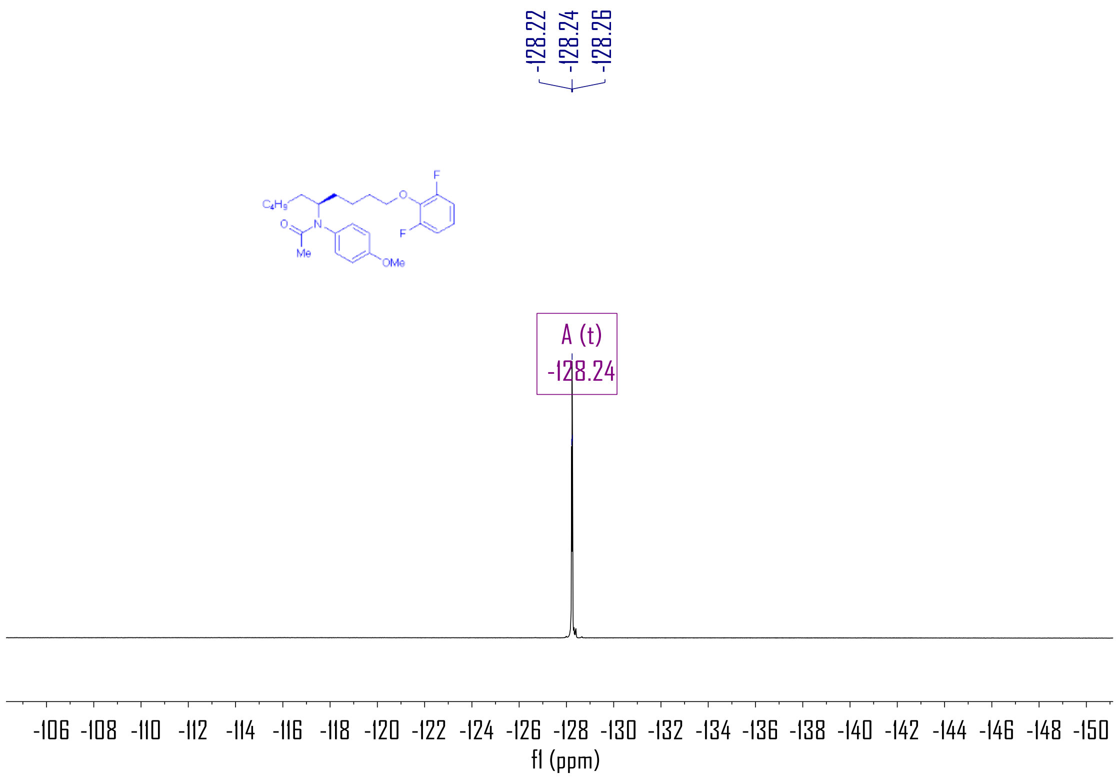

Supplementary Figure 156. ^{19}F NMR spectra for **20**

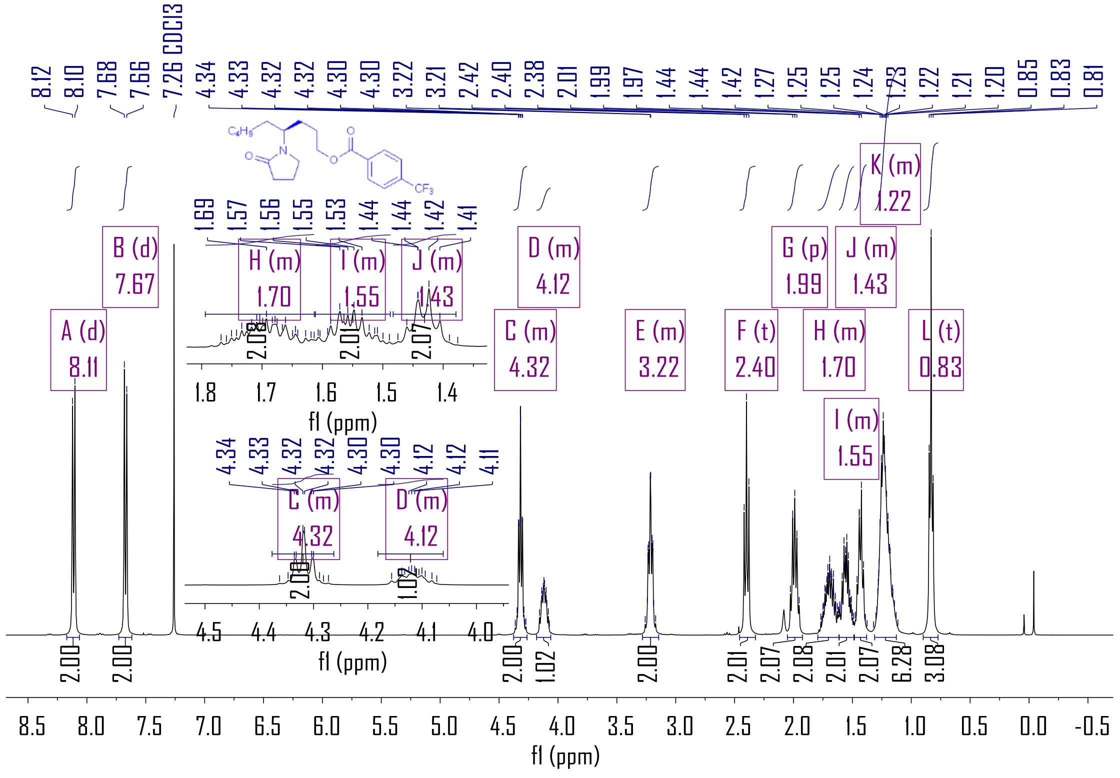

Supplementary Figure 157. ^{1}H NMR spectra for **21**

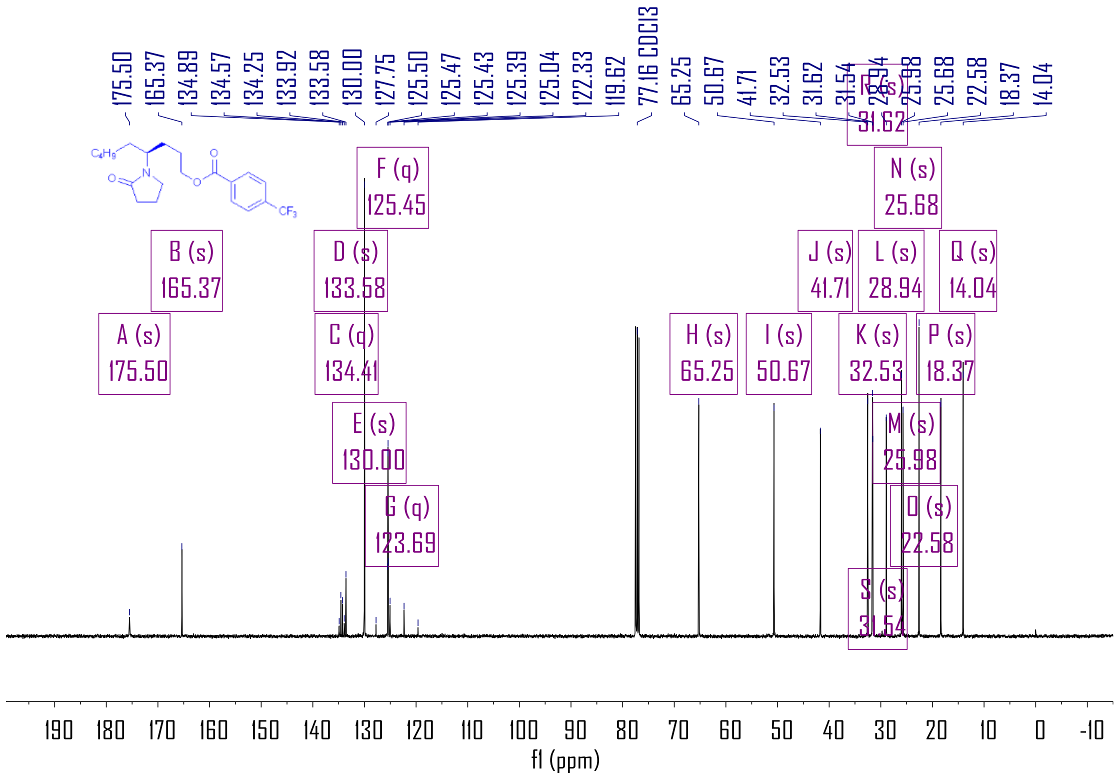

Supplementary Figure 158. ^{13}C NMR spectra for **21**

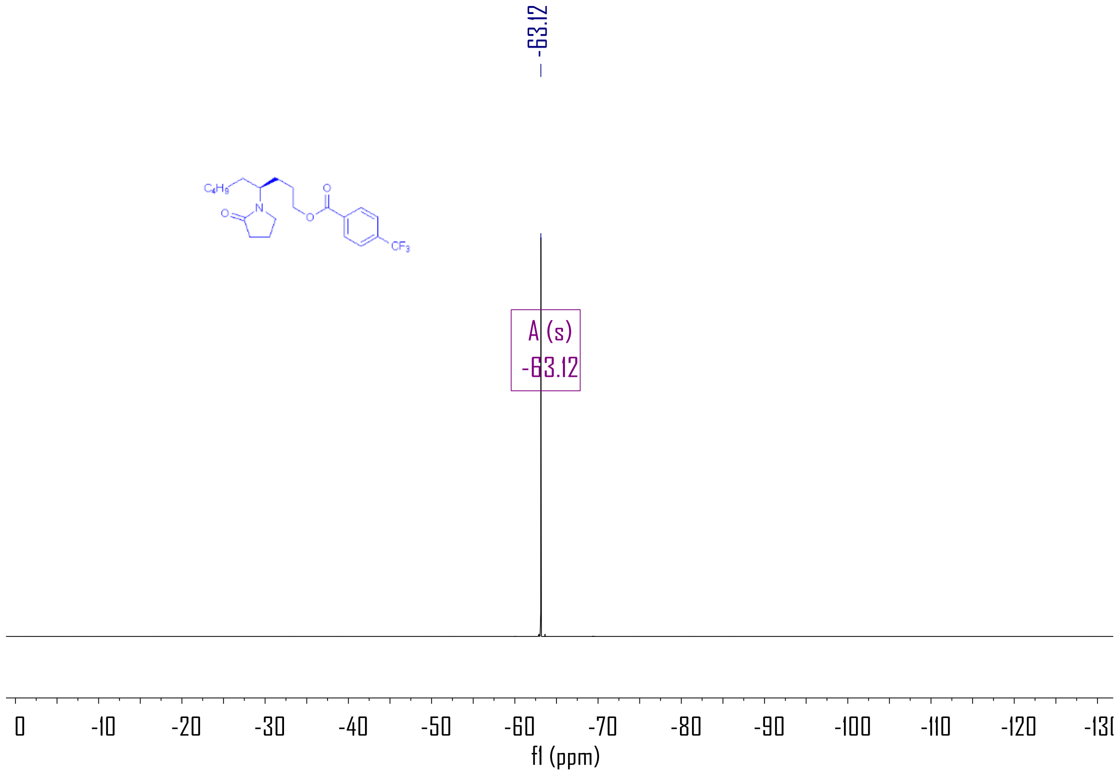

Supplementary Figure 159. ^{19}F NMR spectra for **21**

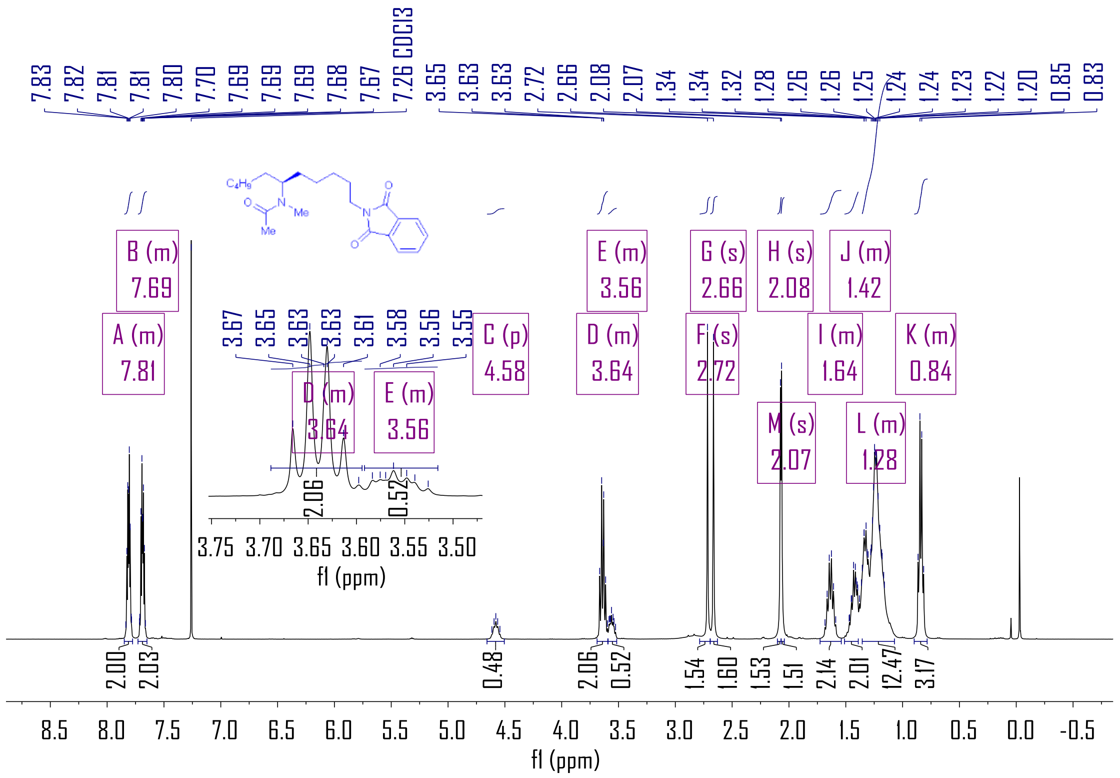

Supplementary Figure 160. ^{1}H NMR spectra for **22**

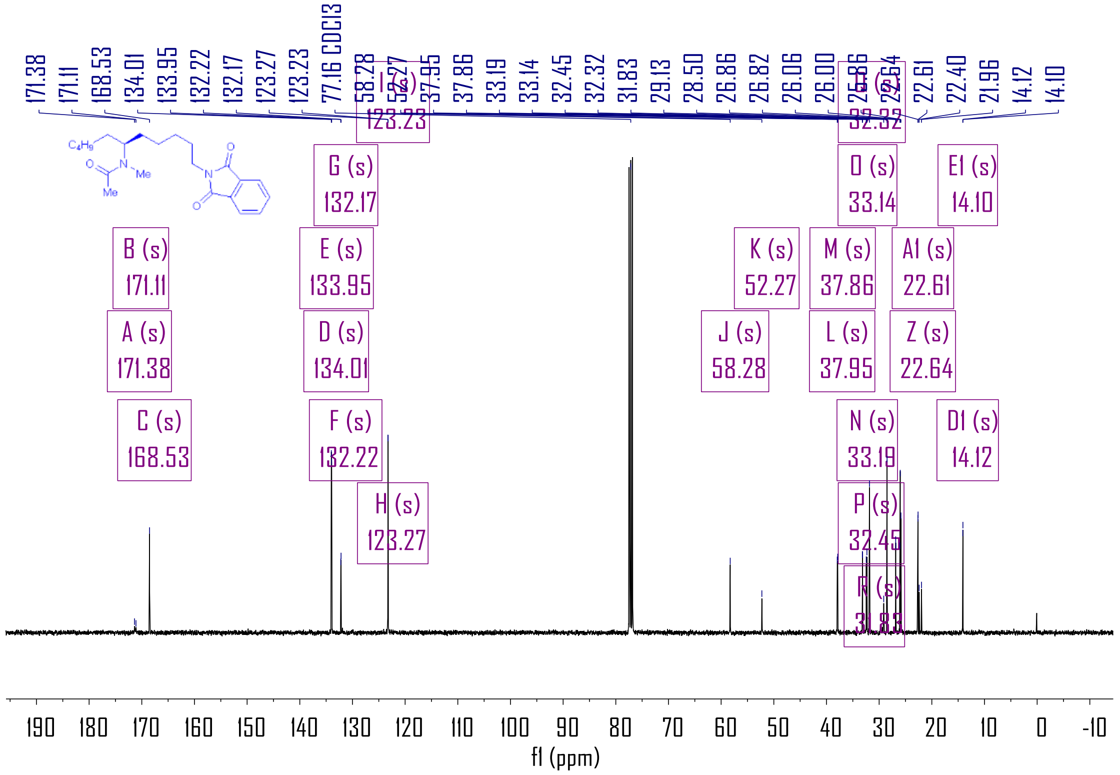

Supplementary Figure 161. ^{13}C NMR spectra for **22**

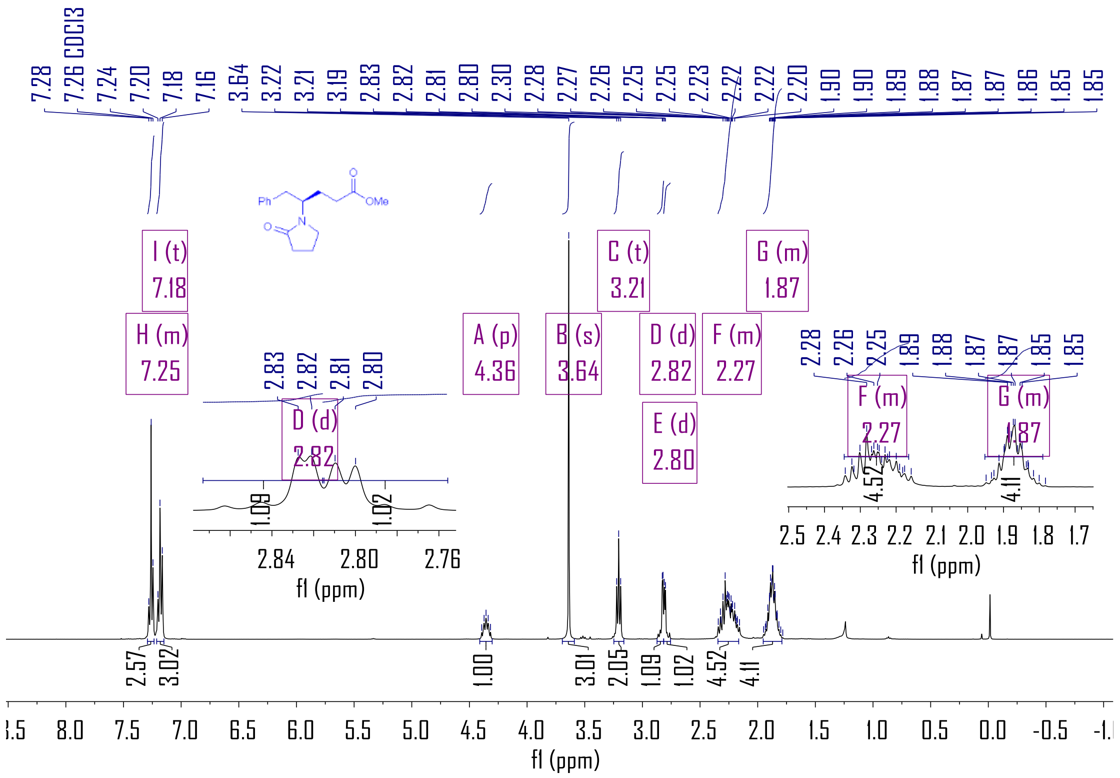

Supplementary Figure 162. ^{1}H NMR spectra for **23**

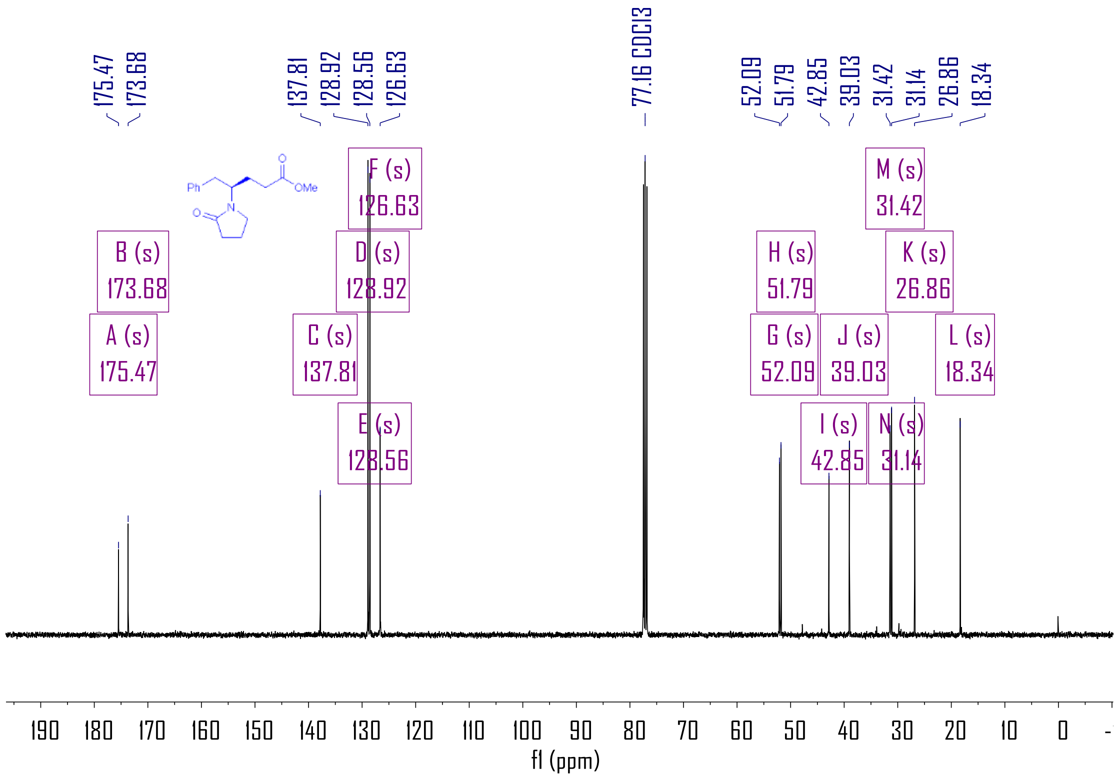

Supplementary Figure 163. ^{13}C NMR spectra for **23**

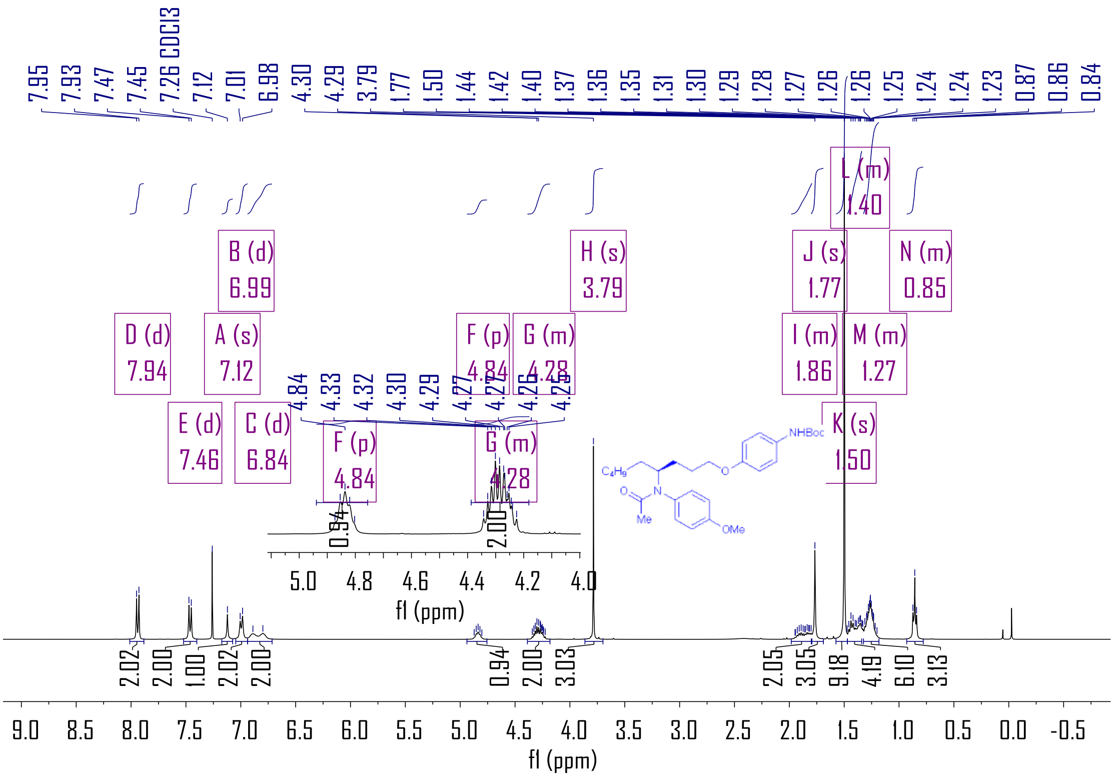

Supplementary Figure 164. ^{1}H NMR spectra for **24**

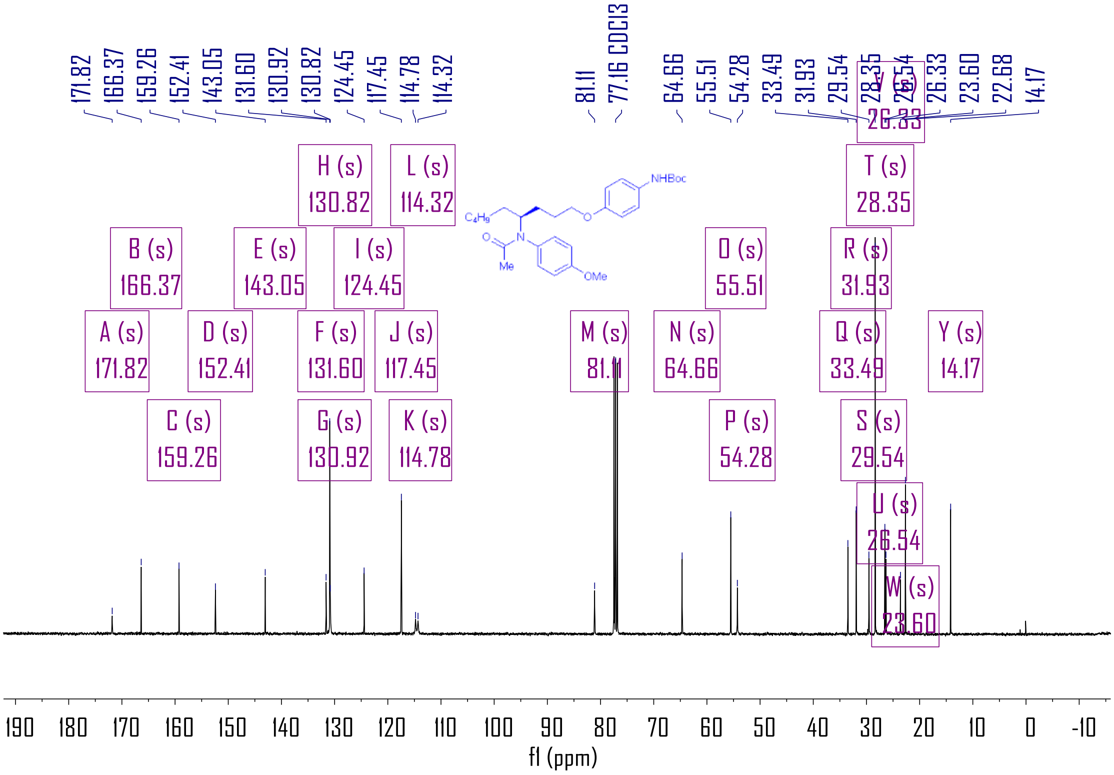

Supplementary Figure 165. ^{13}C NMR spectra for **24**

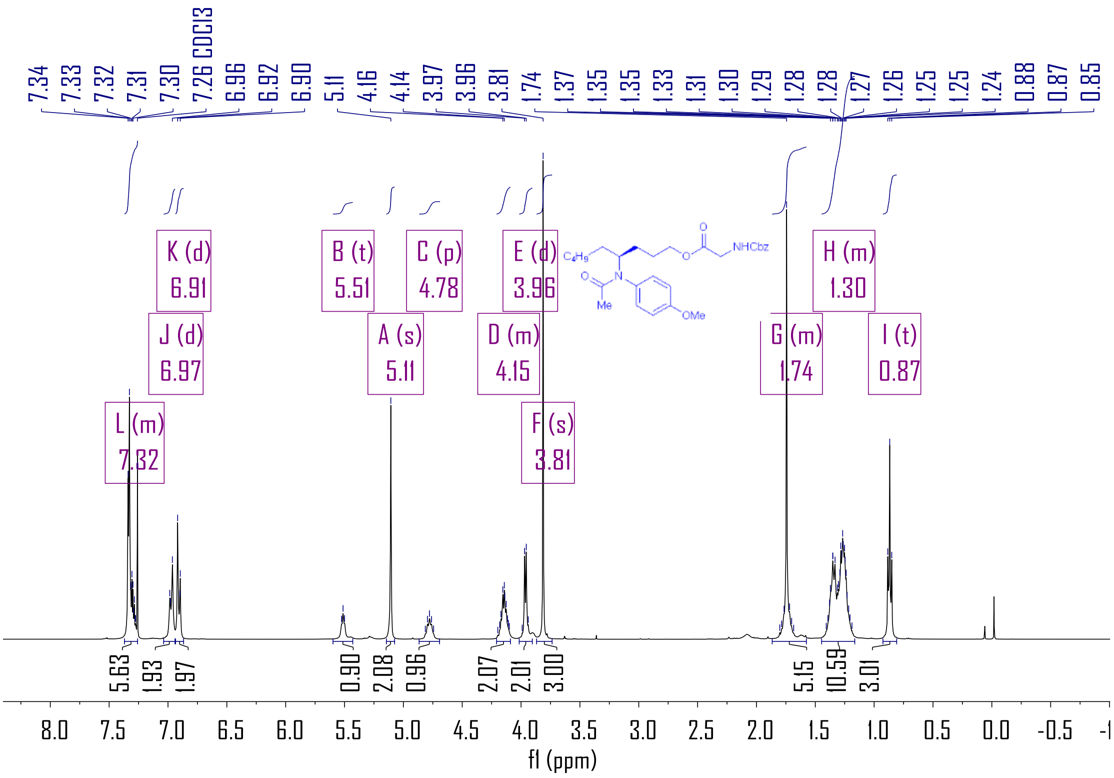

Supplementary Figure 166. ^{1}H NMR spectra for **25**

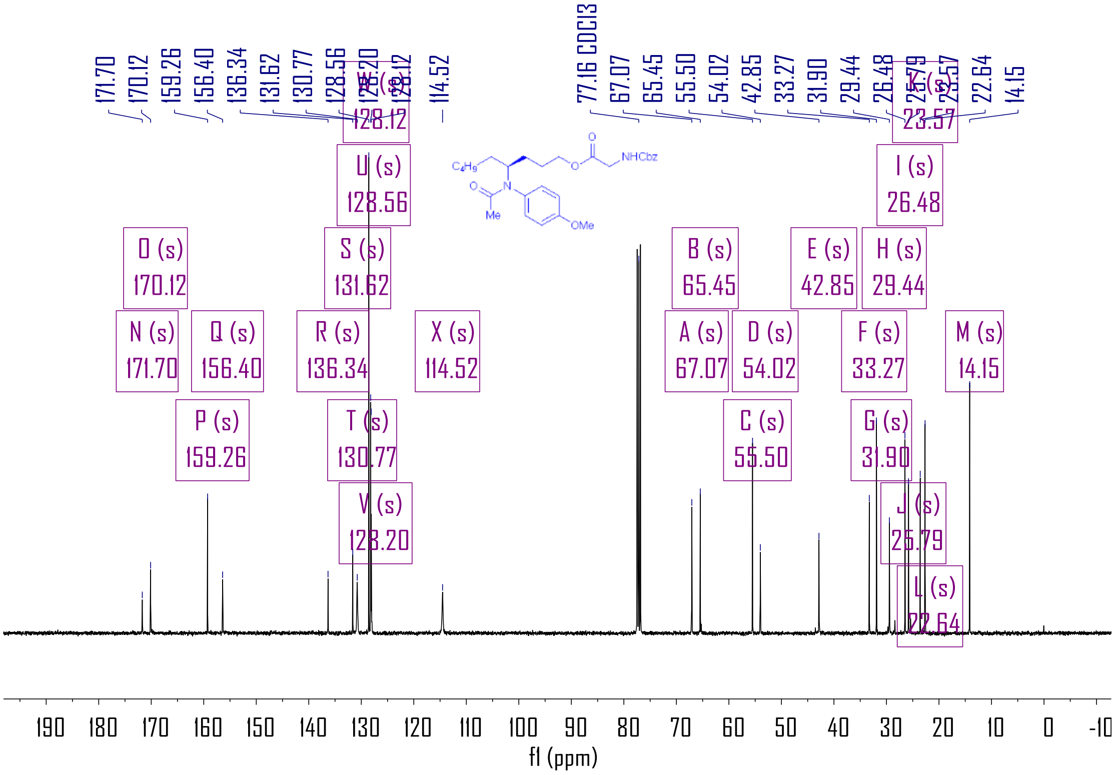

Supplementary Figure 167. ^{13}C NMR spectra for **25**

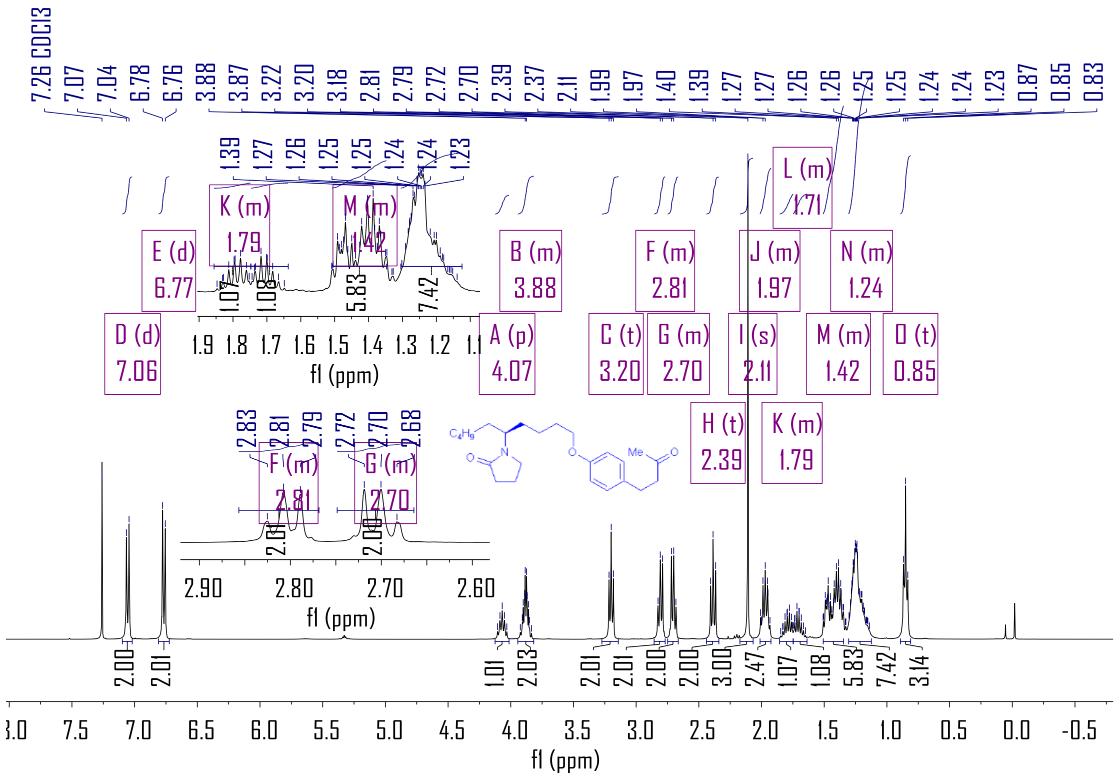

Supplementary Figure 168. ^{1}H NMR spectra for **26**

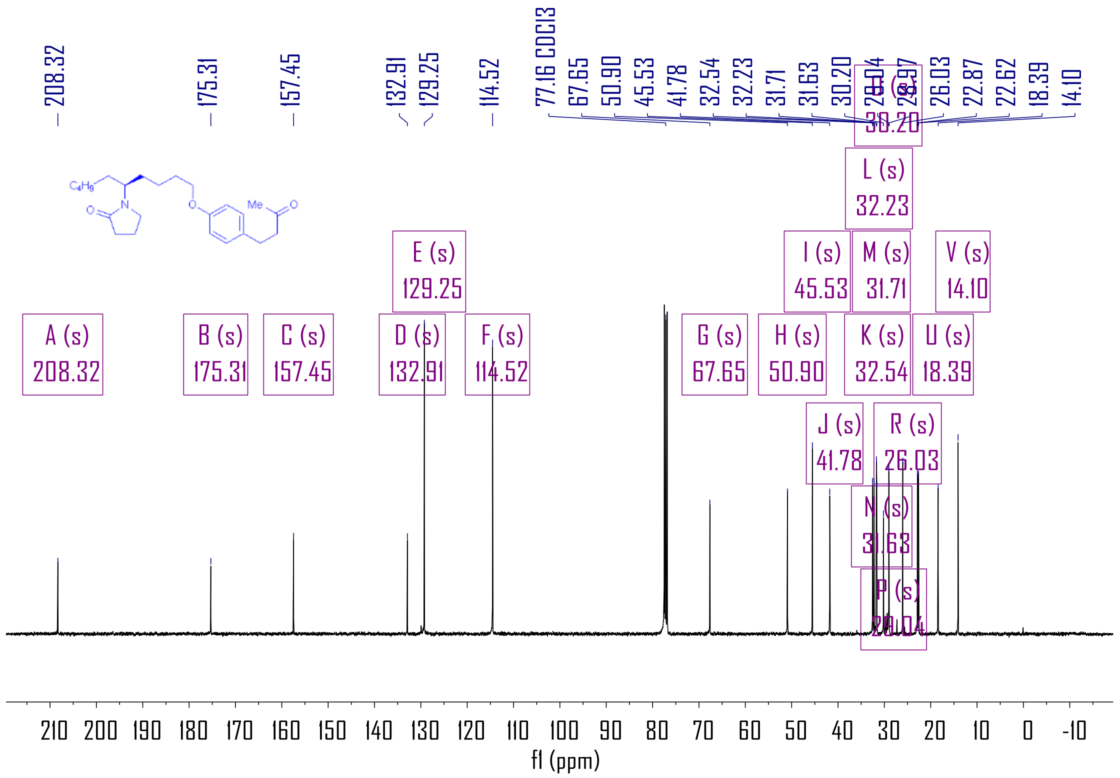

Supplementary Figure 169. ^{13}C NMR spectra for **26**

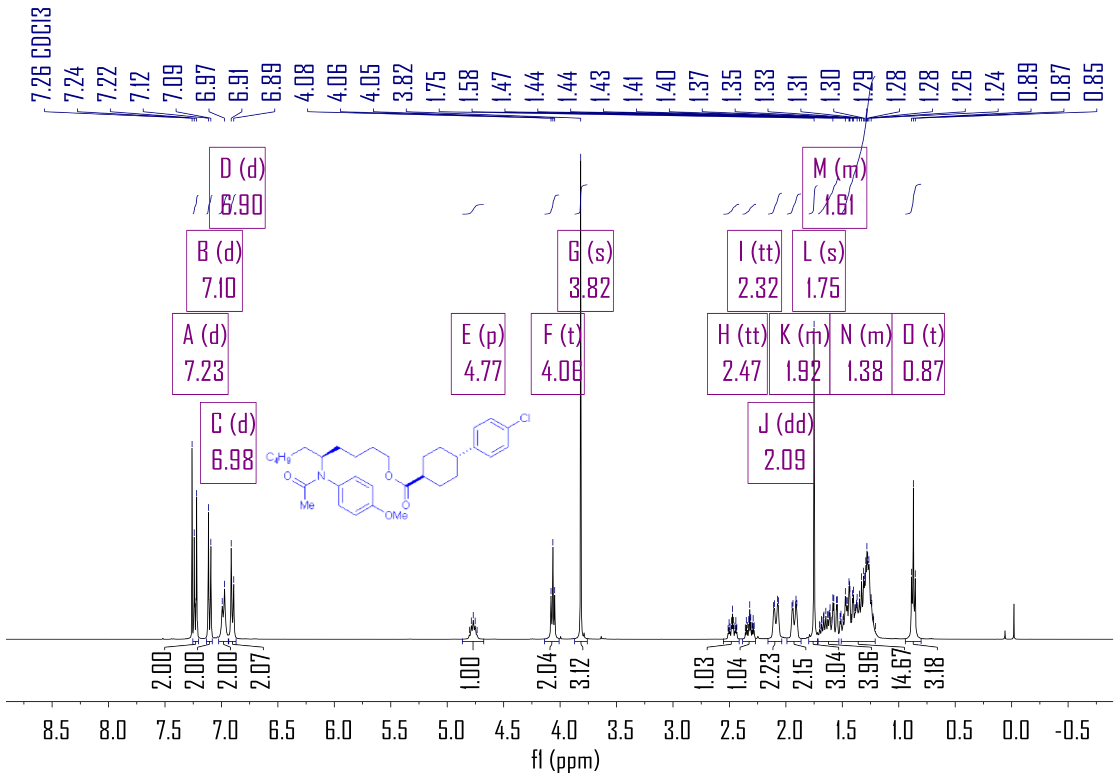

Supplementary Figure 170. ^{1}H NMR spectra for **27**

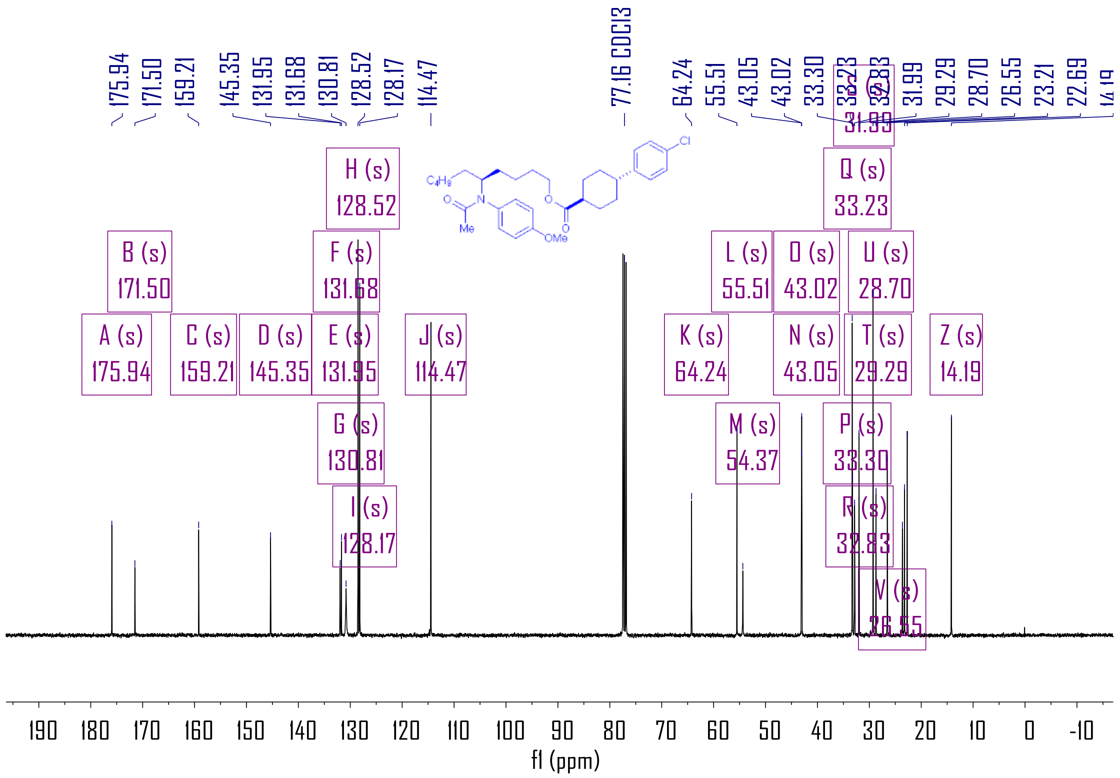

Supplementary Figure 171. ^{13}C NMR spectra for **27**

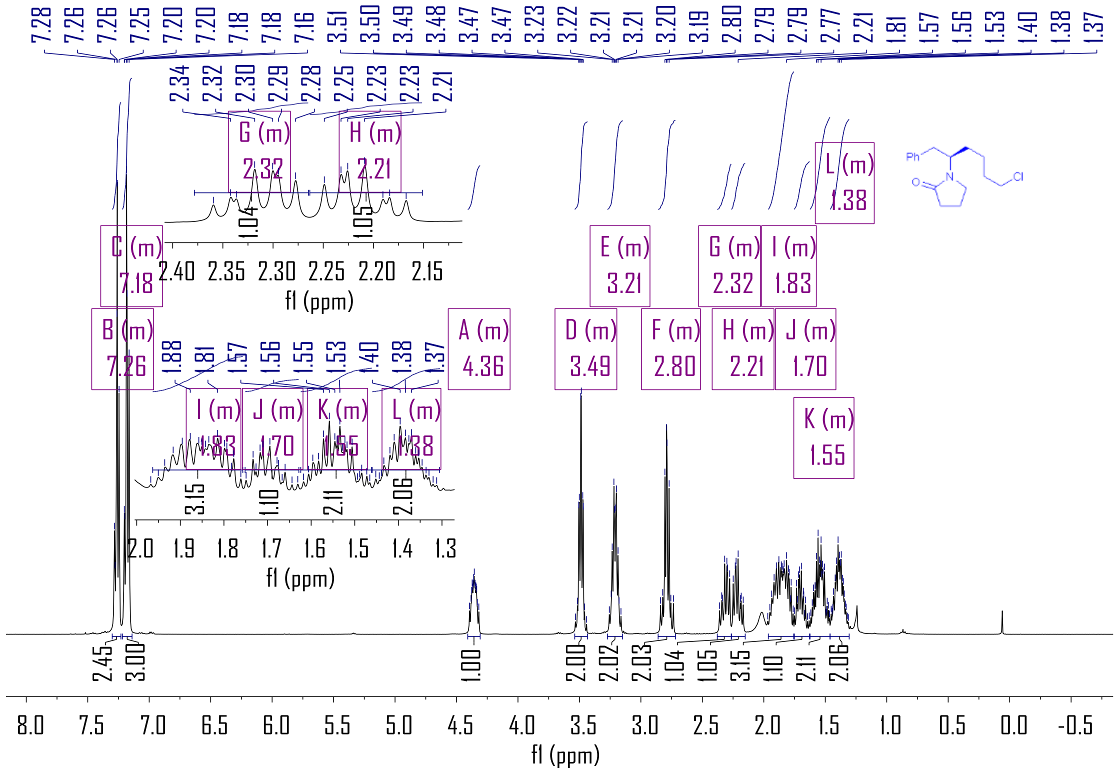

Supplementary Figure 172. ^{1}H NMR spectra for **28**

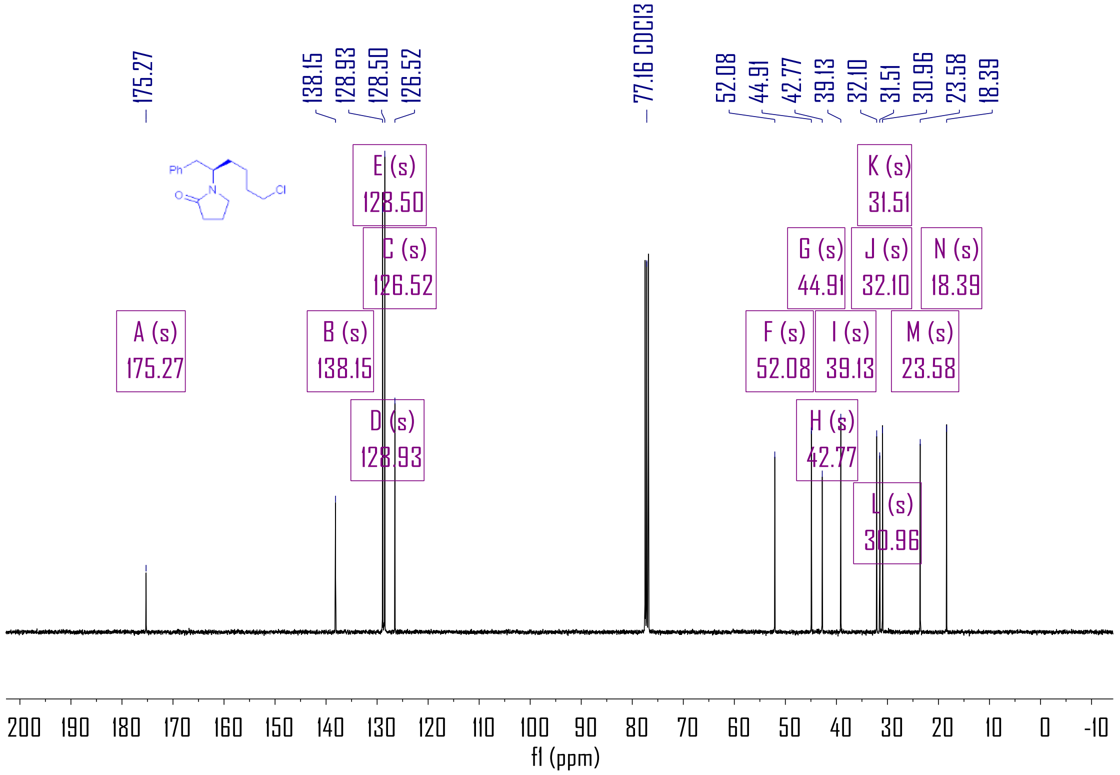

Supplementary Figure 173. ^{13}C NMR spectra for **28**

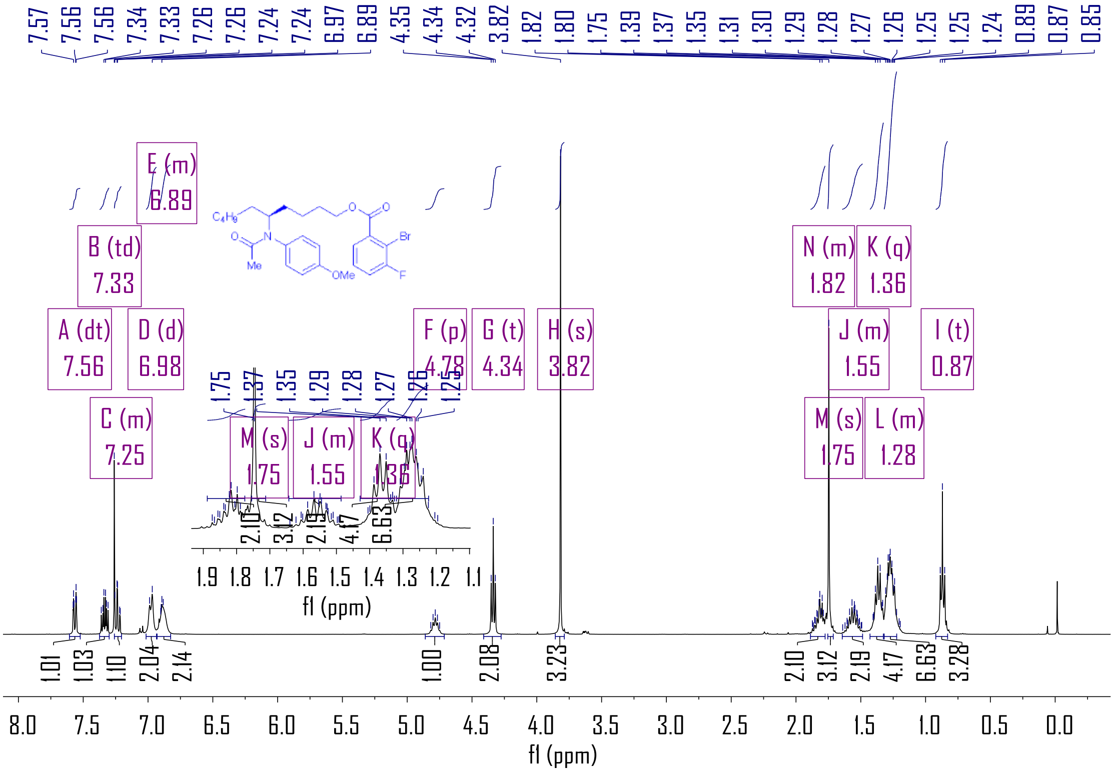

Supplementary Figure 174. ^{1}H NMR spectra for **29**

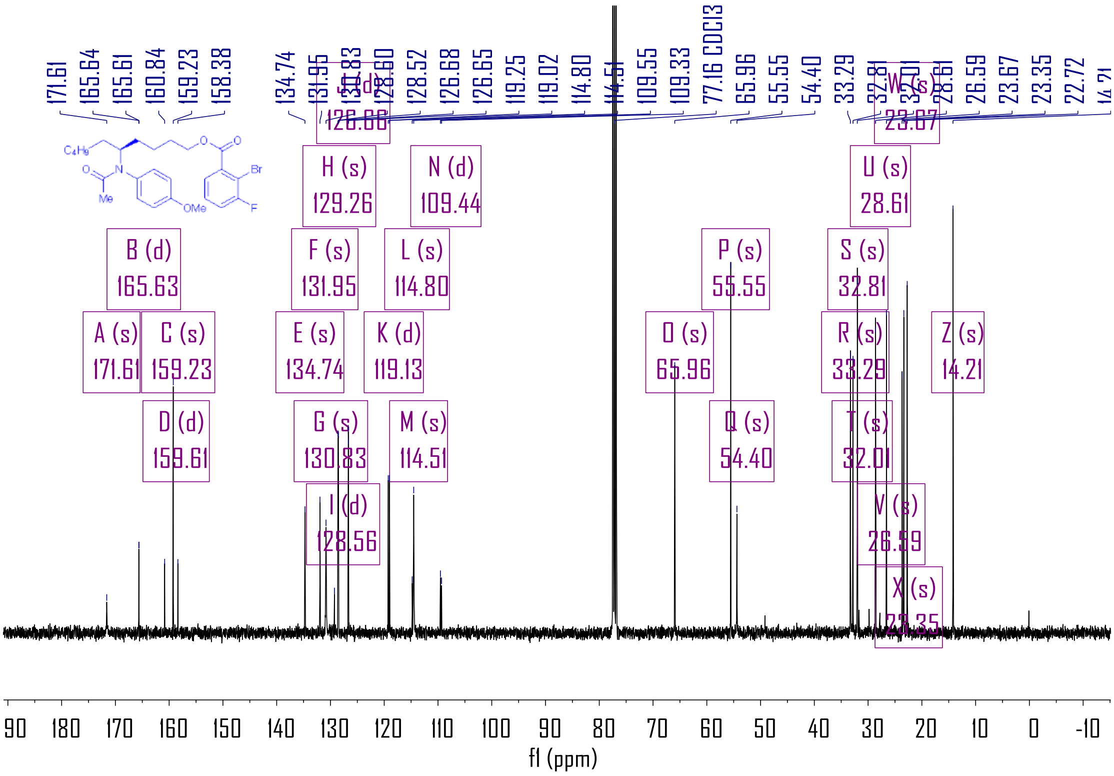

Supplementary Figure 175. ^{13}C NMR spectra for **29**

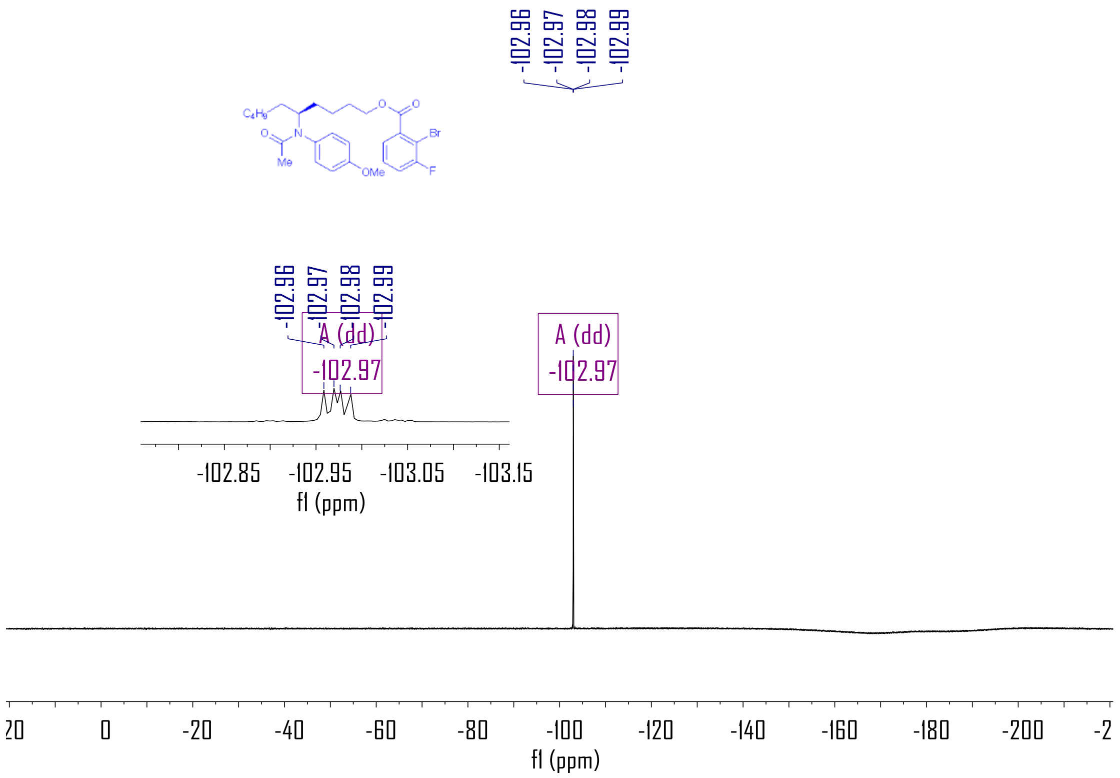

Supplementary Figure 176. ^{19}F NMR spectra for **29**

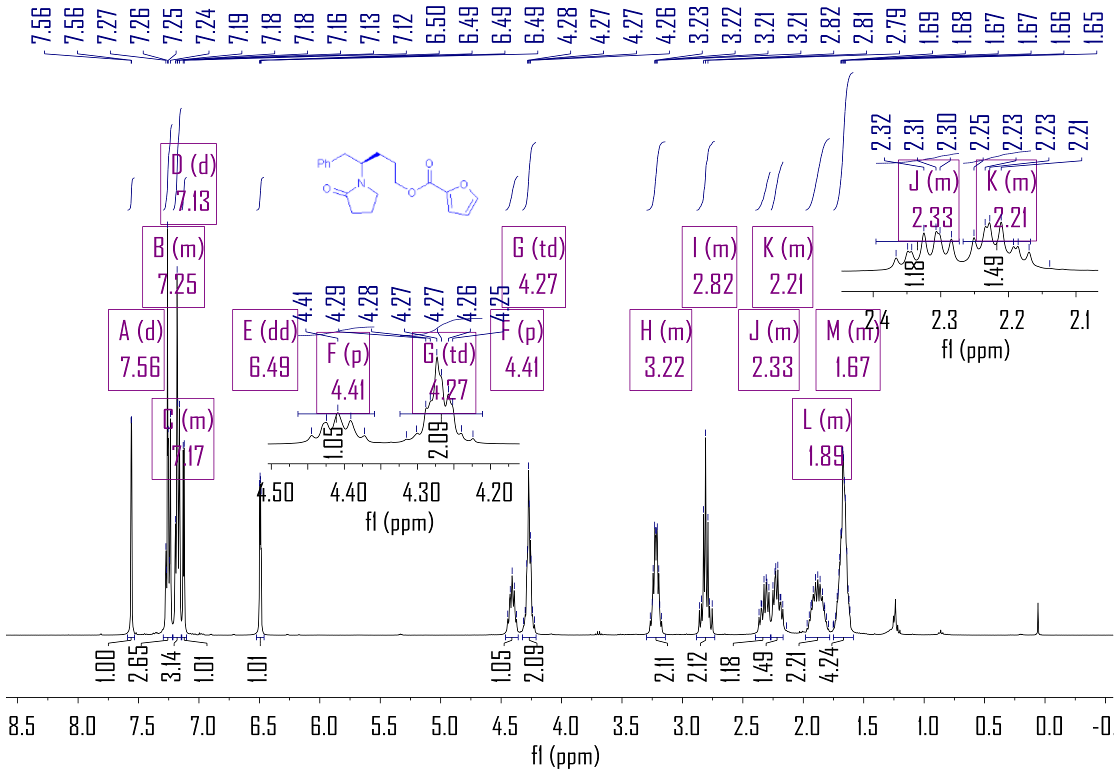

Supplementary Figure 177. ^{1}H NMR spectra for **30**

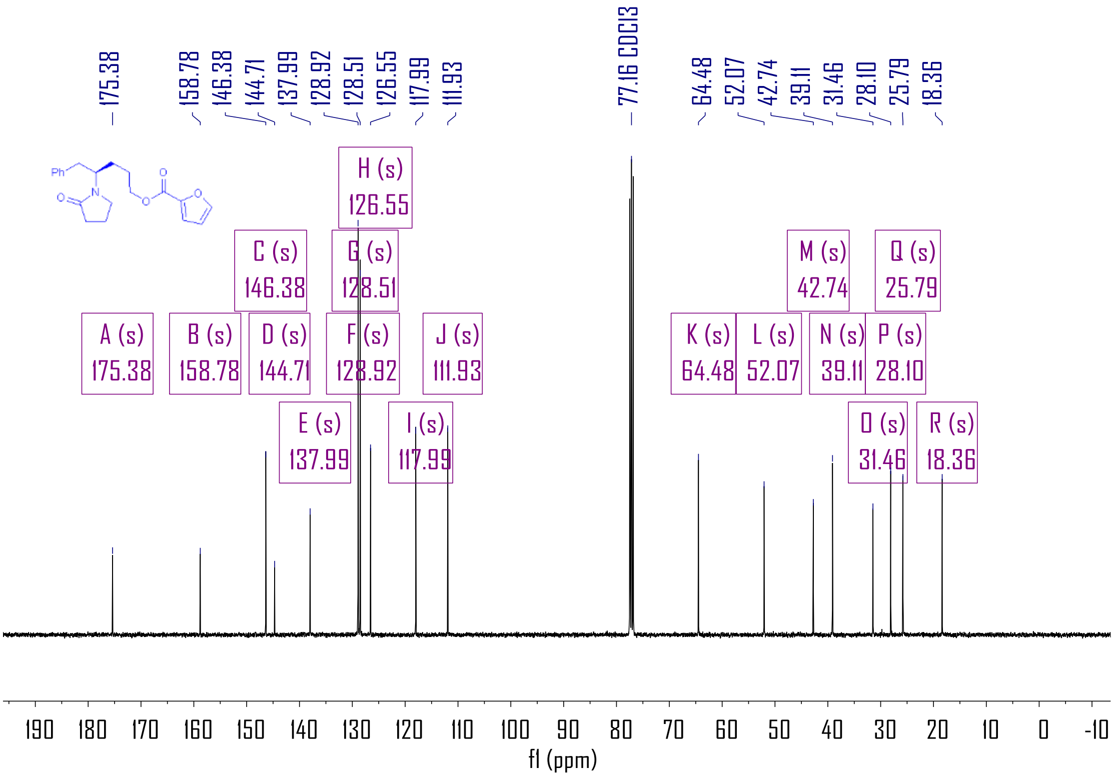

Supplementary Figure 178. ^{13}C NMR spectra for **30**

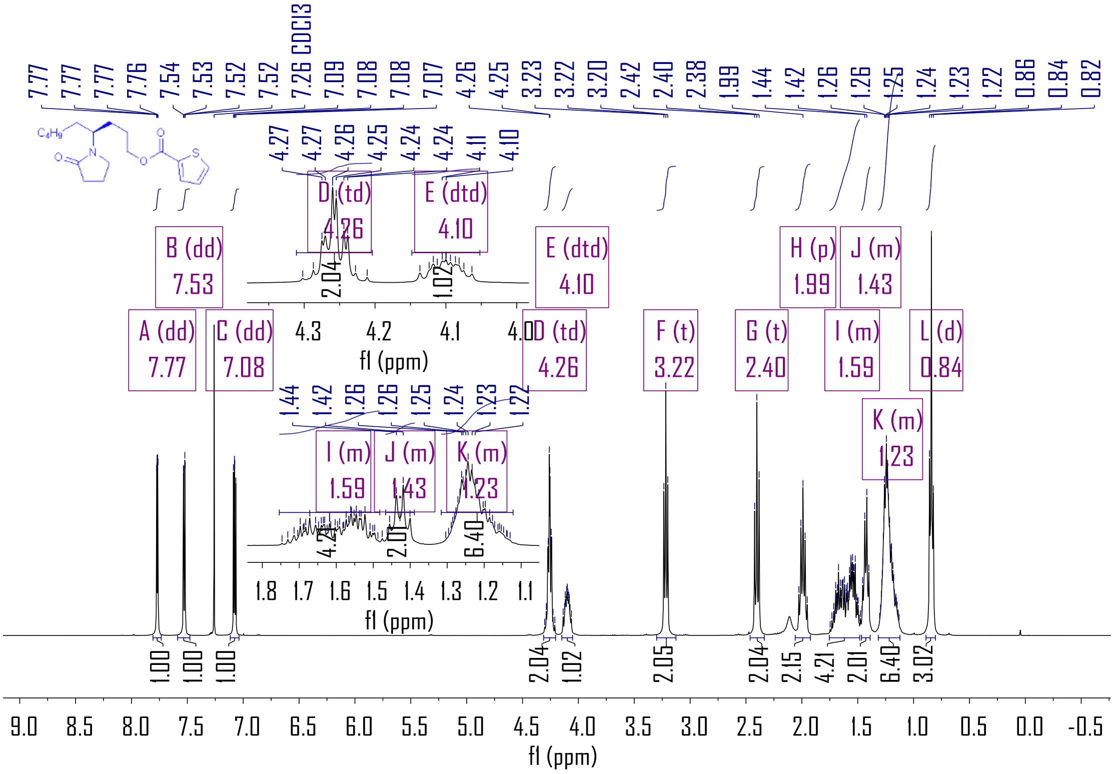

Supplementary Figure 179. ^{1}H NMR spectra for **31**

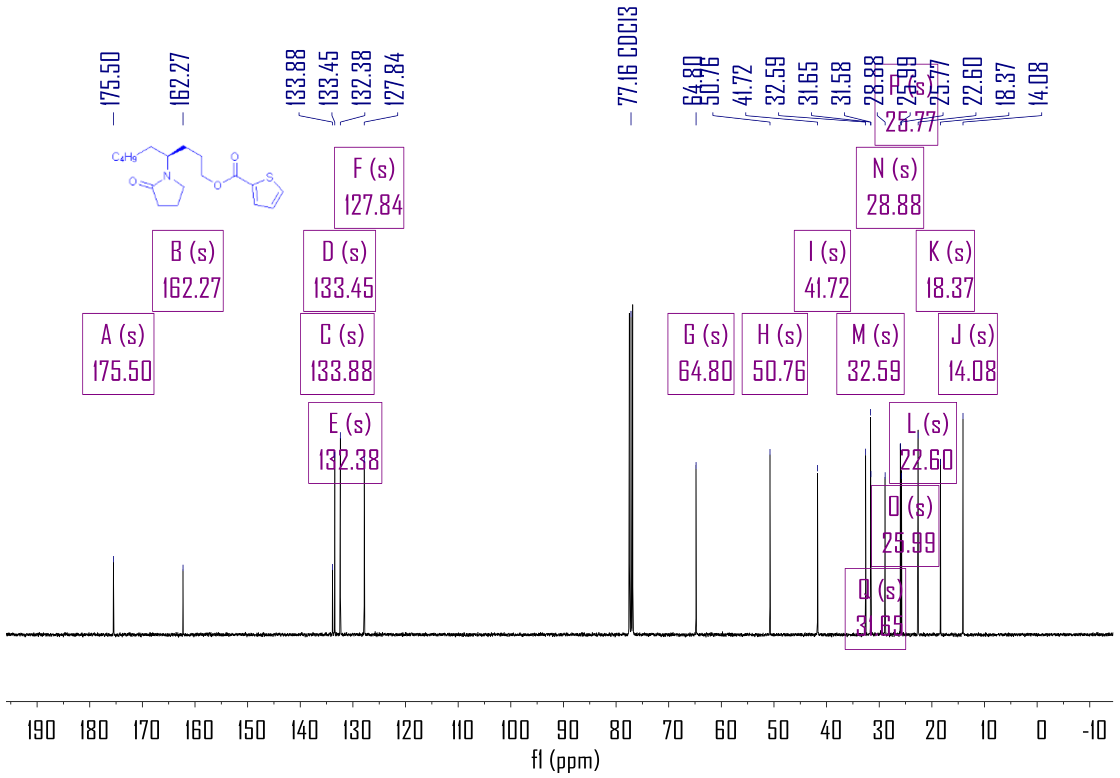

Supplementary Figure 180. ^{13}C NMR spectra for **31**

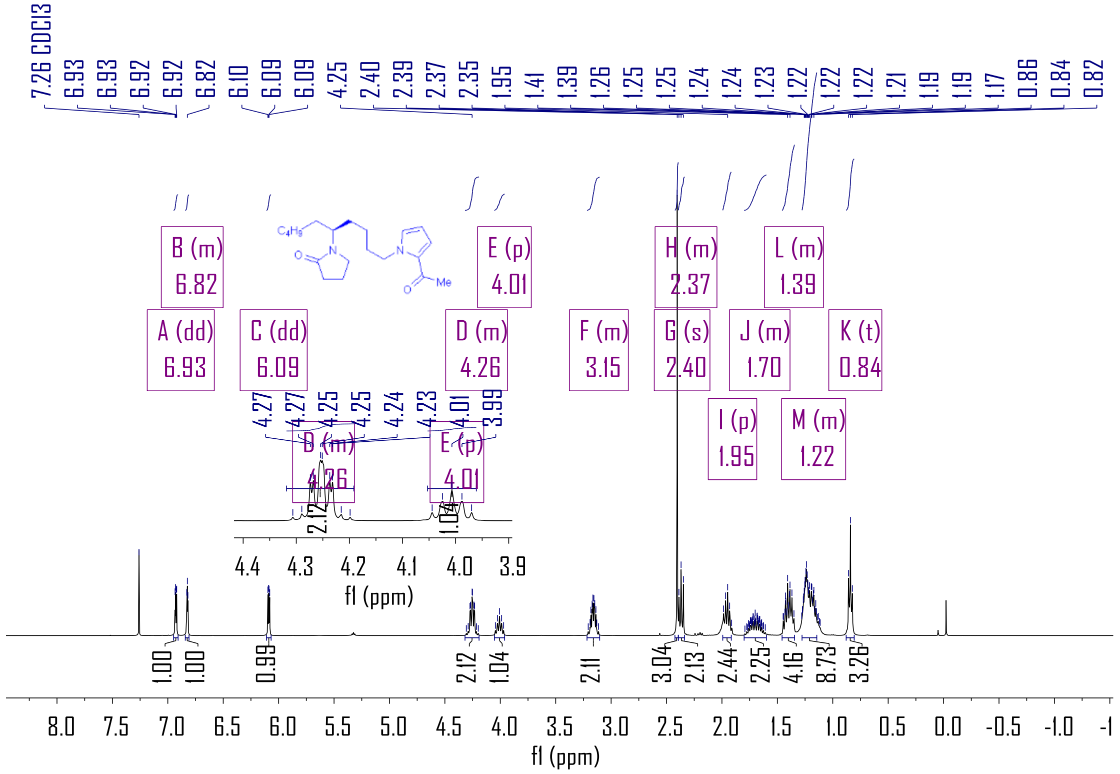

Supplementary Figure 181. ^{1}H NMR spectra for **32**

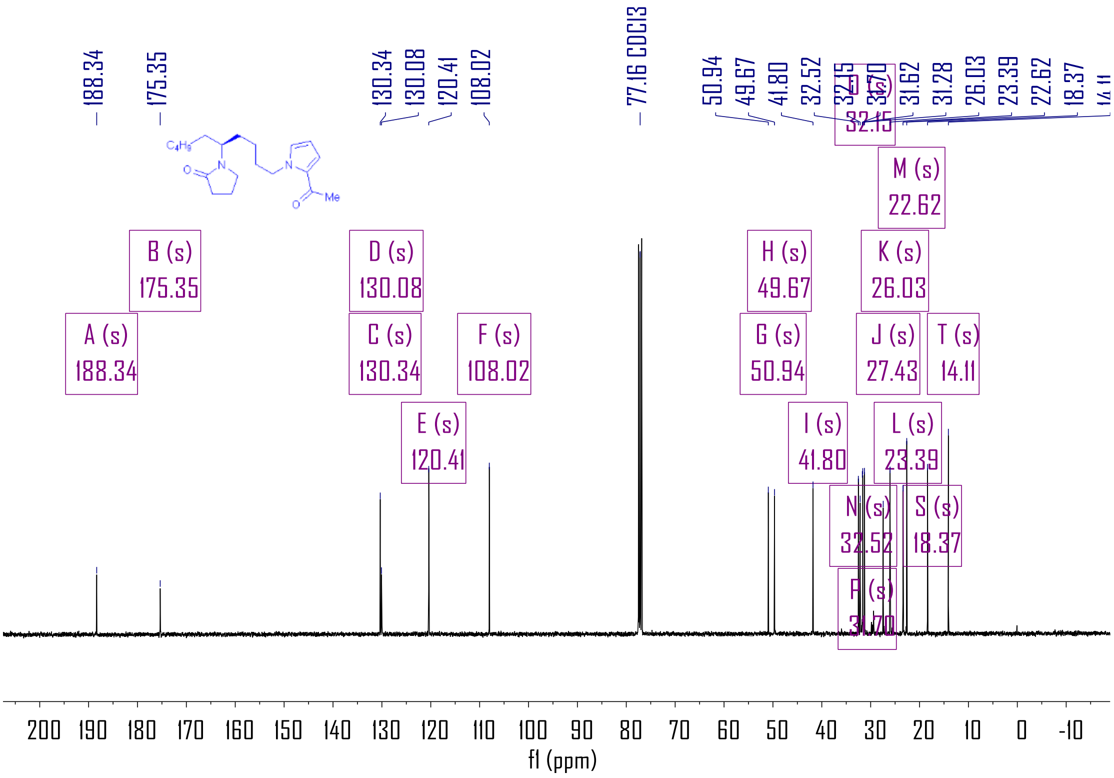

Supplementary Figure 182. ^{13}C NMR spectra for **32**

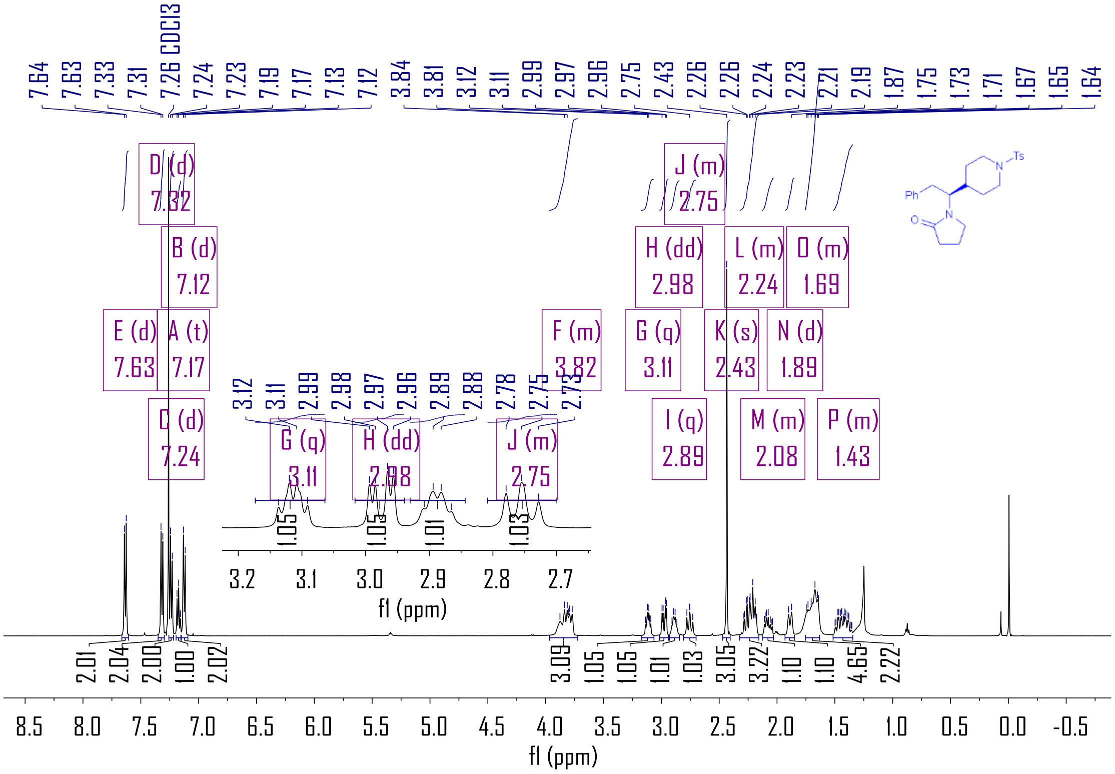

Supplementary Figure 183. ^{1}H NMR spectra for **33**

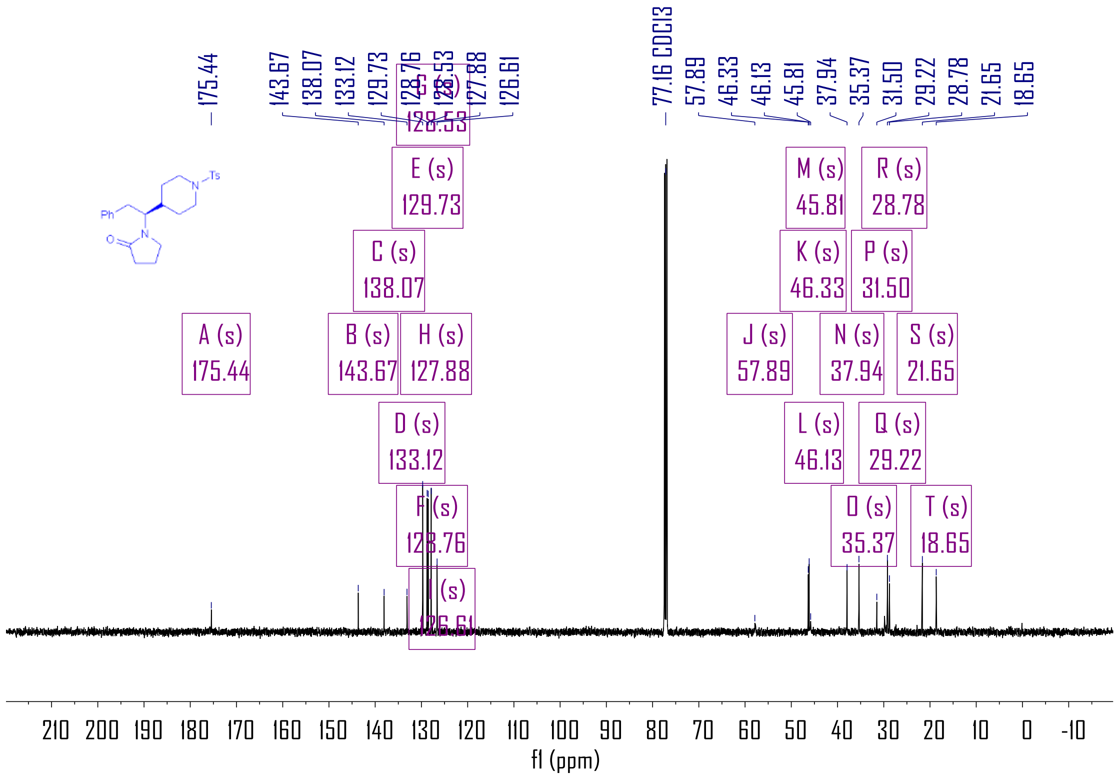

Supplementary Figure 184. ^{13}C NMR spectra for **33**

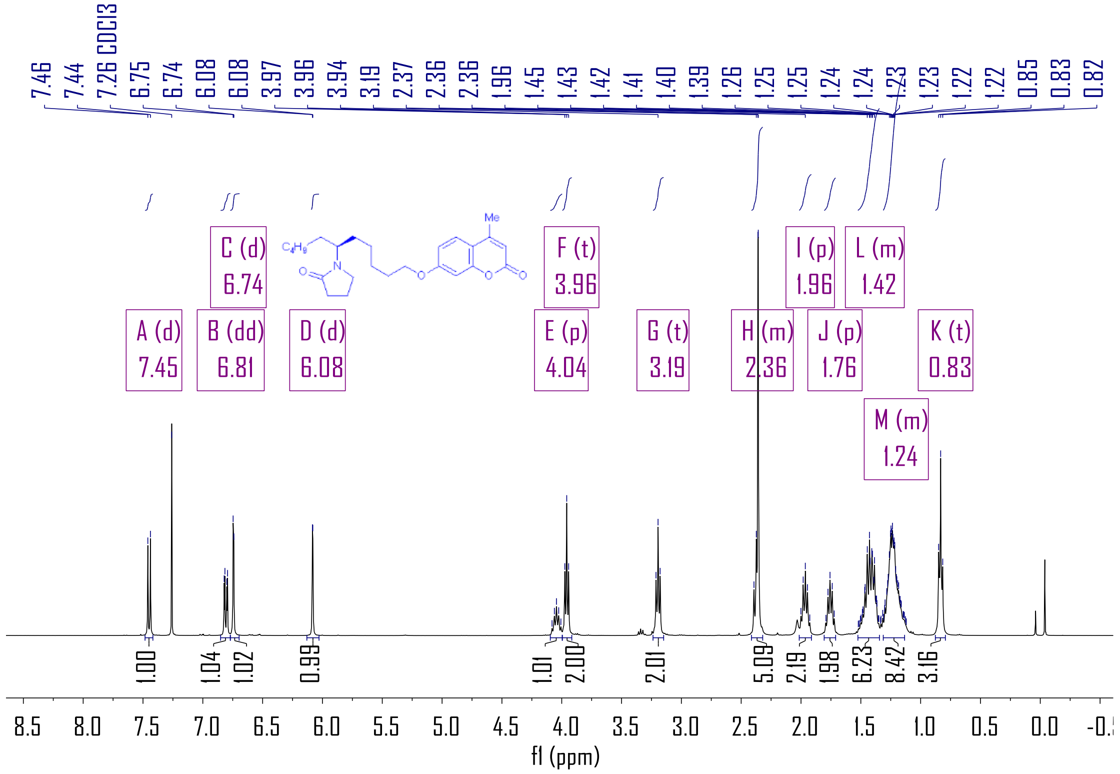

Supplementary Figure 185. ^{1}H NMR spectra for **34**

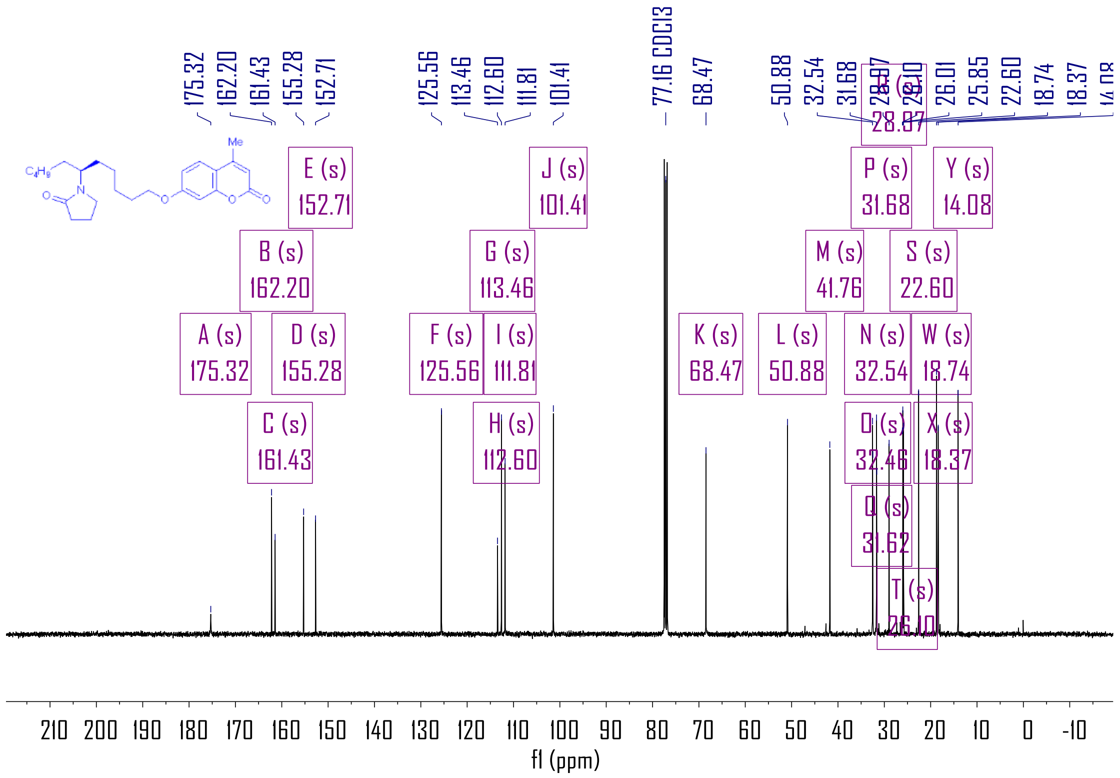

Supplementary Figure 186. ^{13}C NMR spectra for **34**

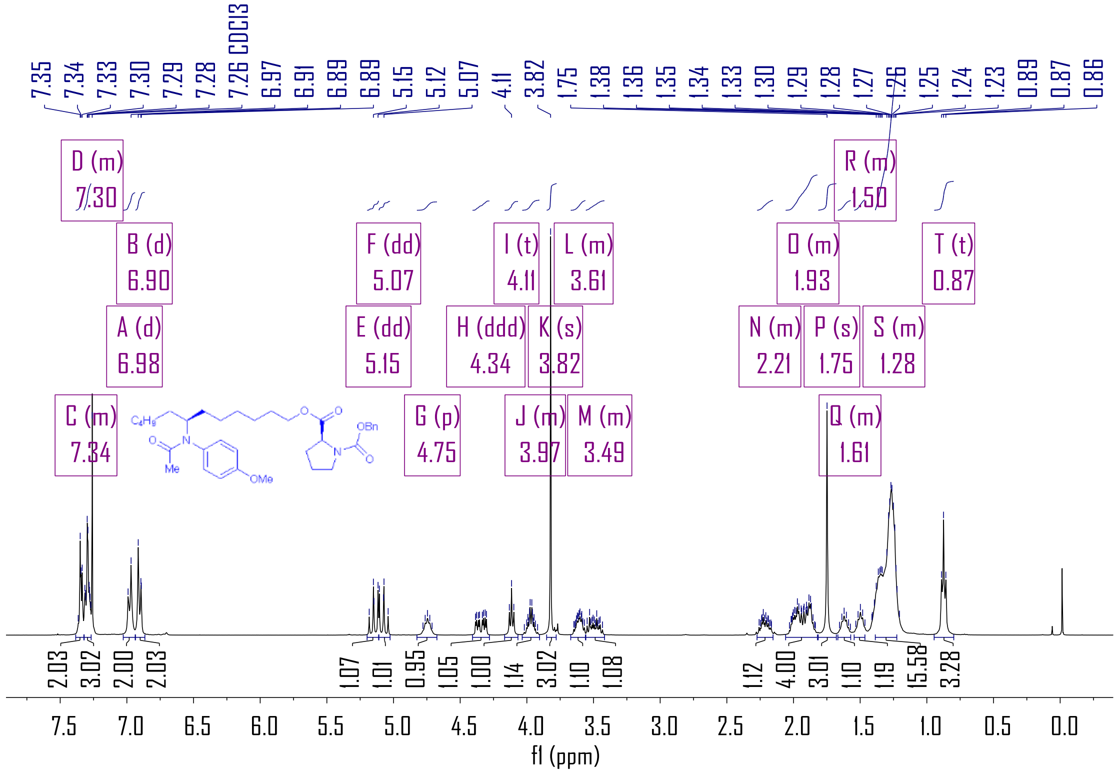

Supplementary Figure 187. ^{1}H NMR spectra for **35**

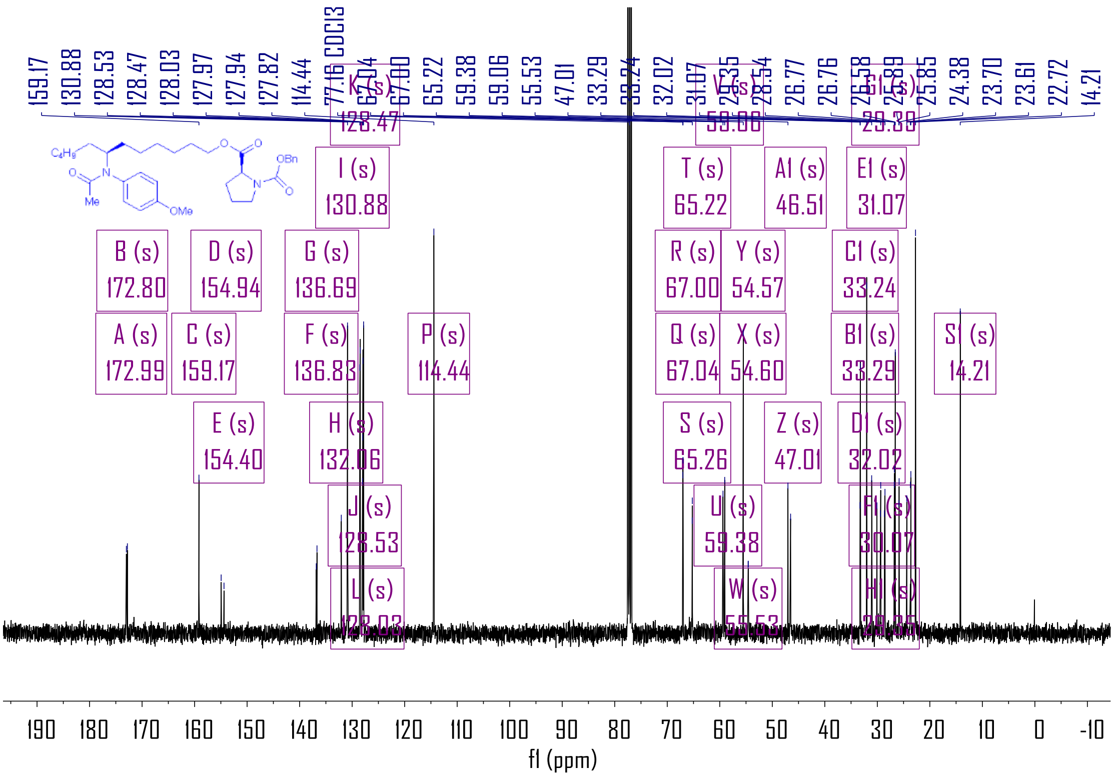

Supplementary Figure 188. ^{13}C NMR spectra for **35**

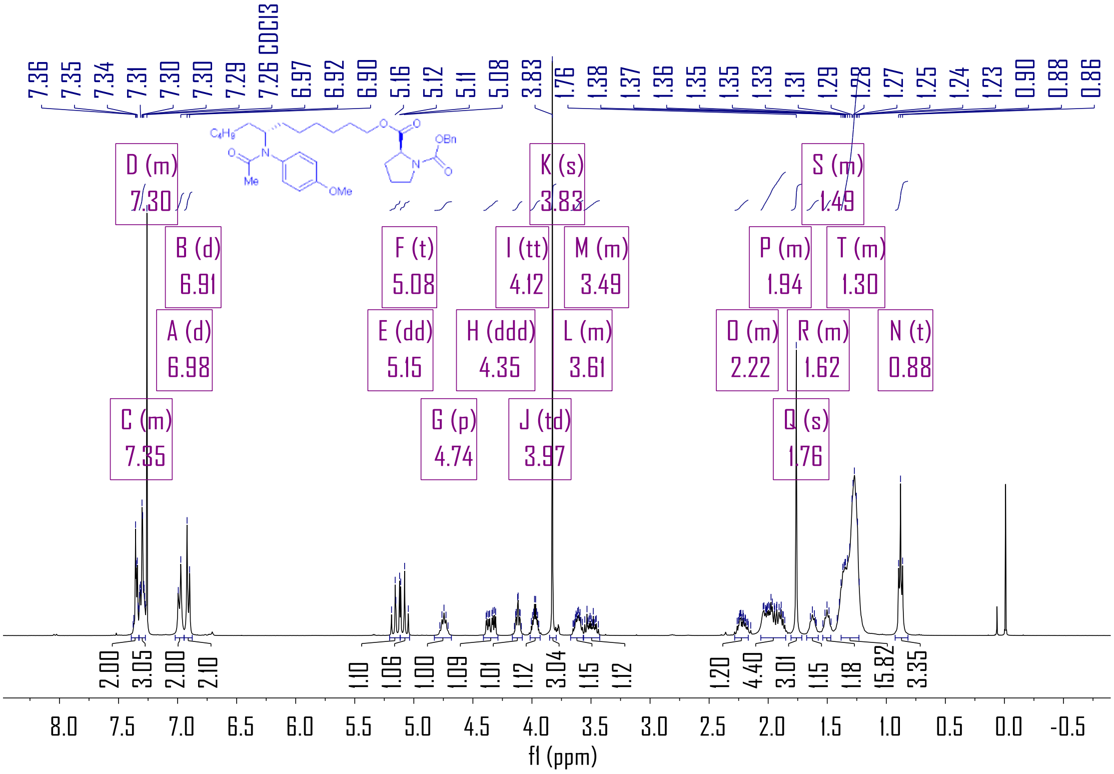

Supplementary Figure 189. ^{1}H NMR spectra for **36**

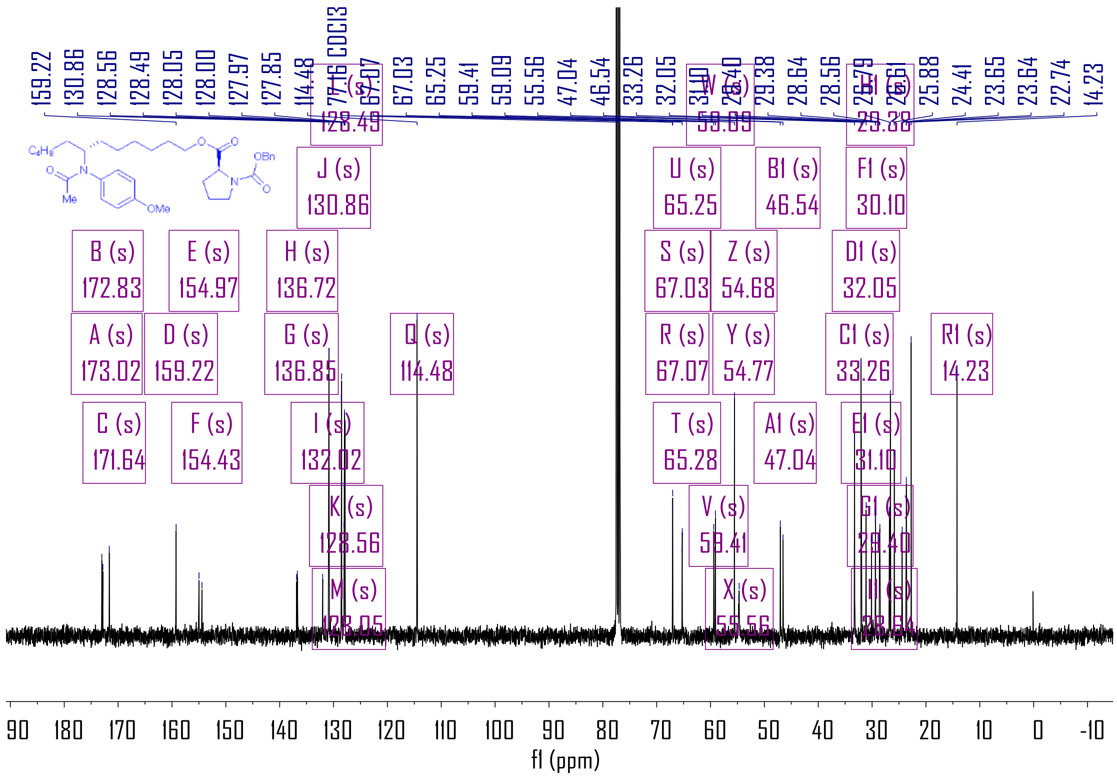

Supplementary Figure 190. ^{13}C NMR spectra for **36**

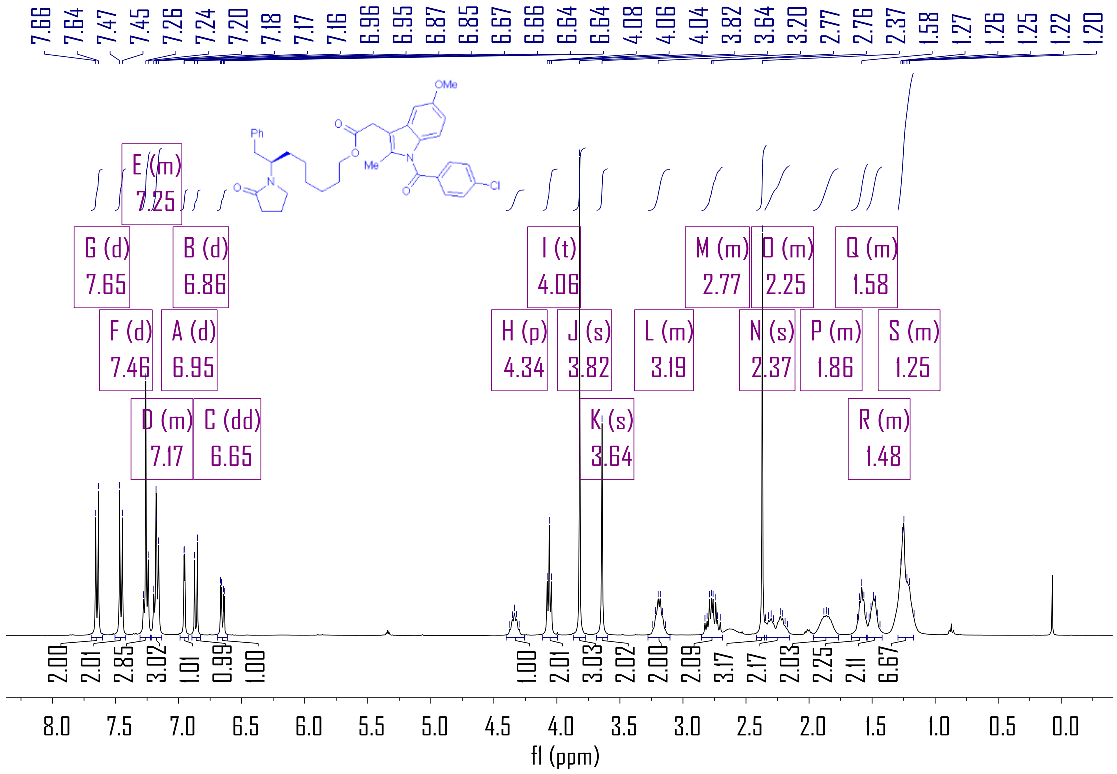

Supplementary Figure 191. ^{1}H NMR spectra for **37**

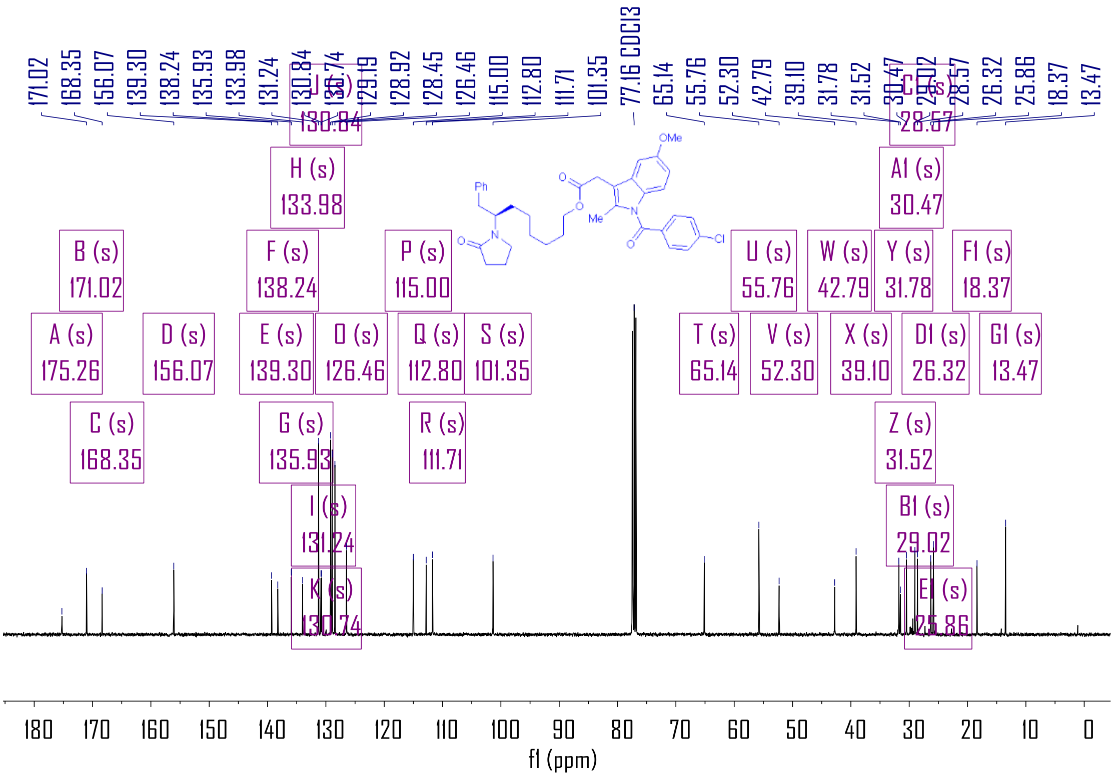

Supplementary Figure 192. ^{13}C NMR spectra for **37**

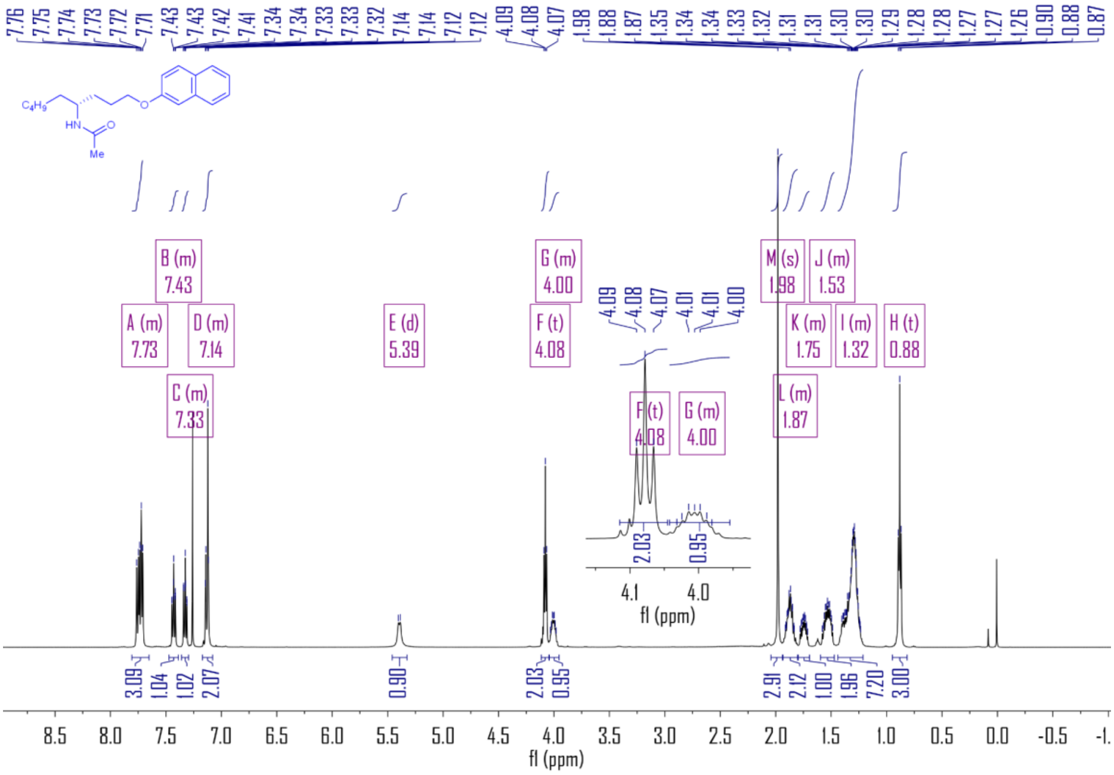

Supplementary Figure 193. ^{1}H NMR spectra for **38**

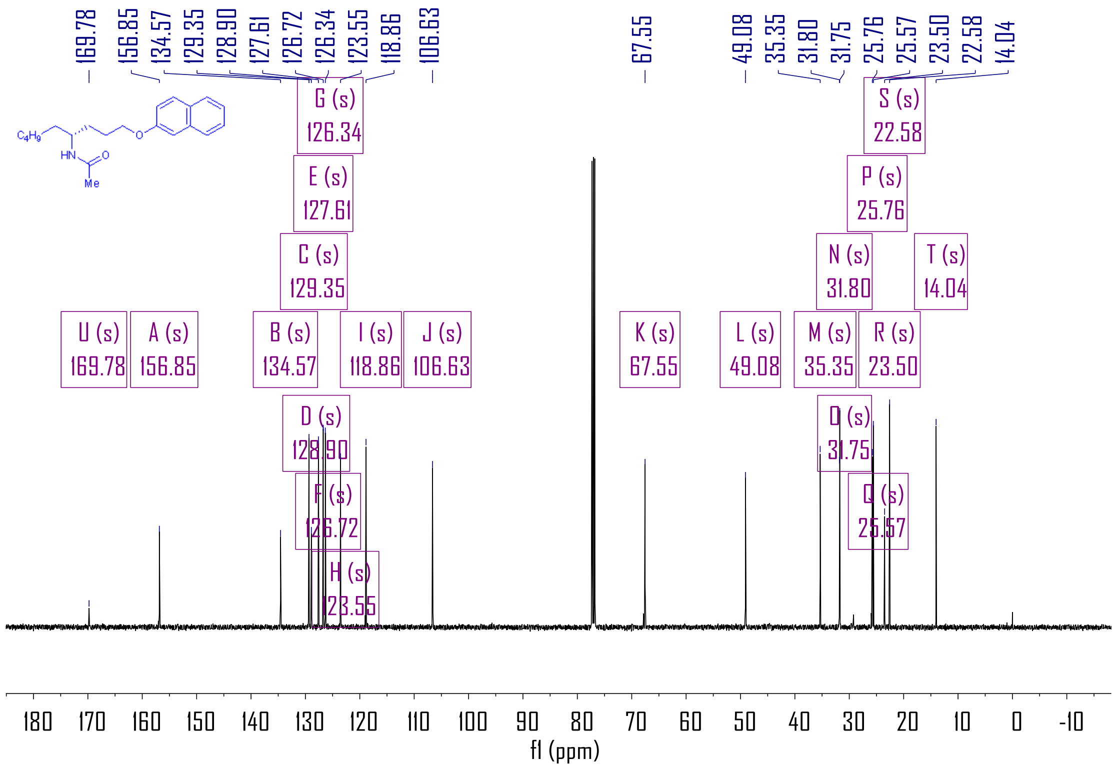

Supplementary Figure 194. ^{13}C NMR spectra for **38**

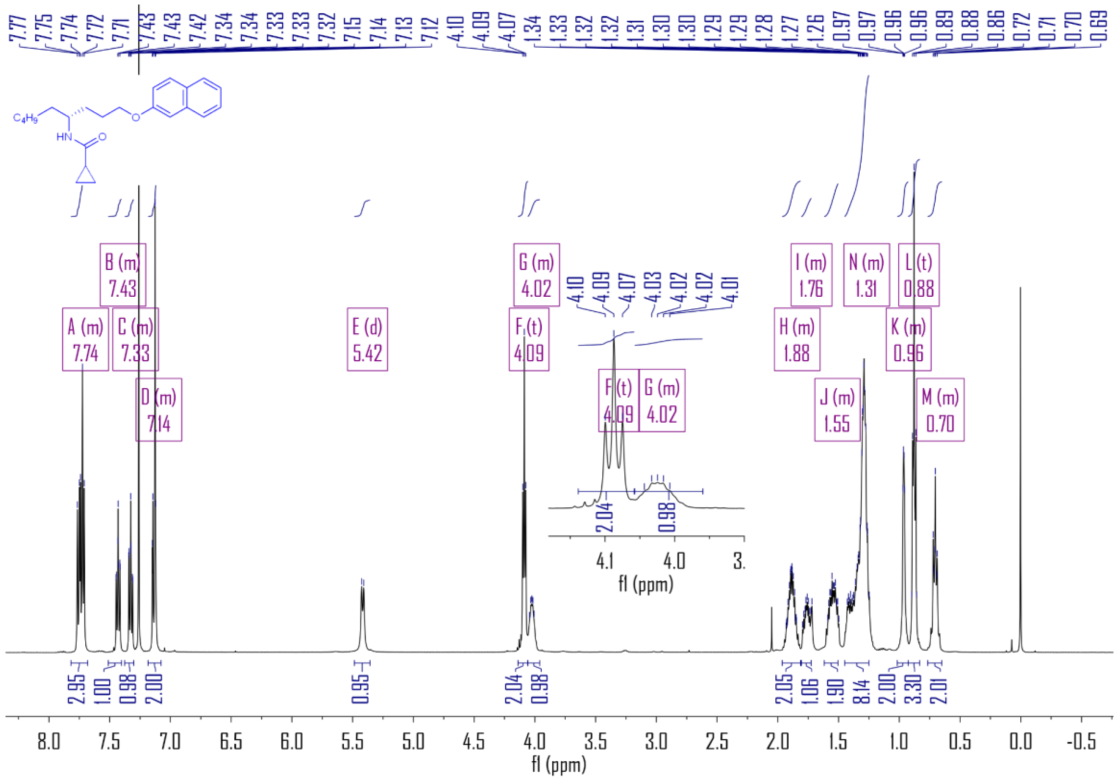

Supplementary Figure 195. ^{1}H NMR spectra for **38**

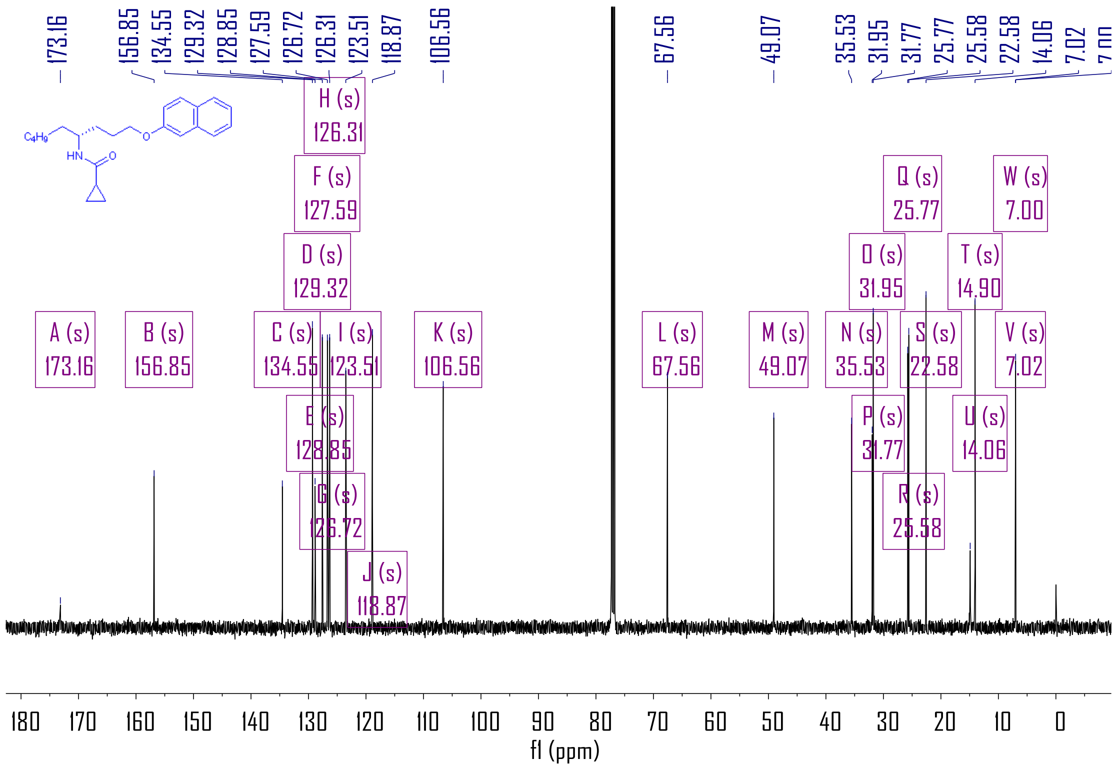

Supplementary Figure 196. ^{13}C NMR spectra for **38**

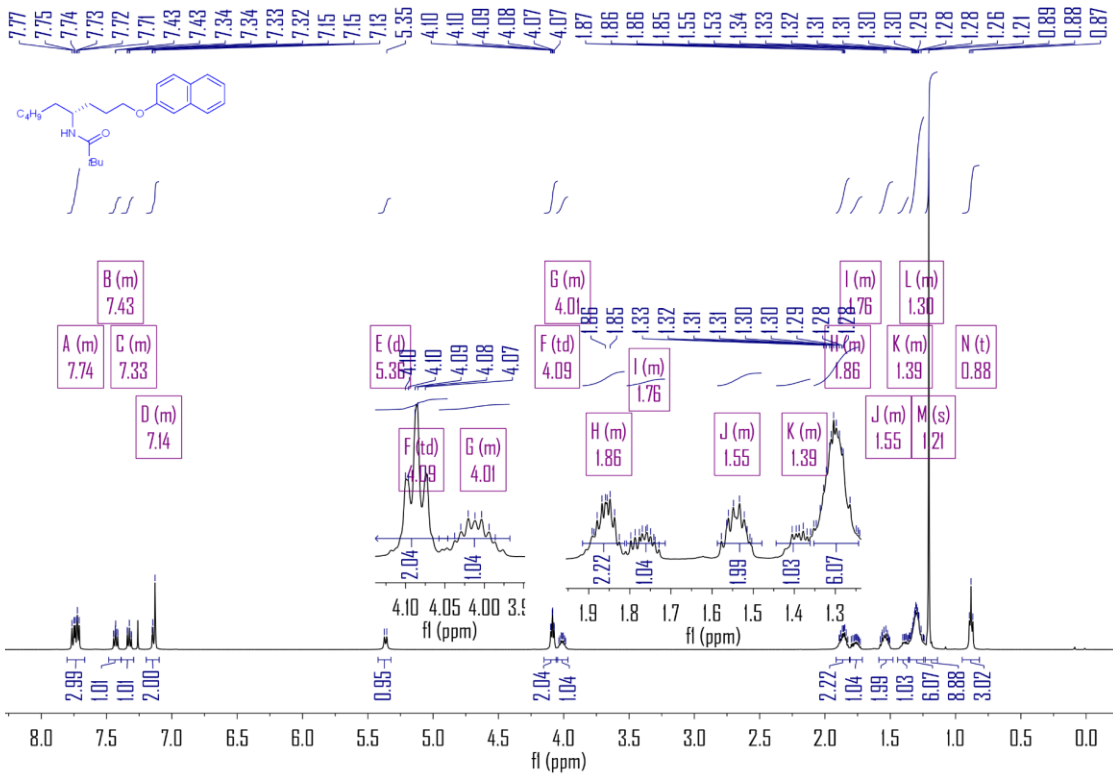

Supplementary Figure 197. ^{1}H NMR spectra for **40**

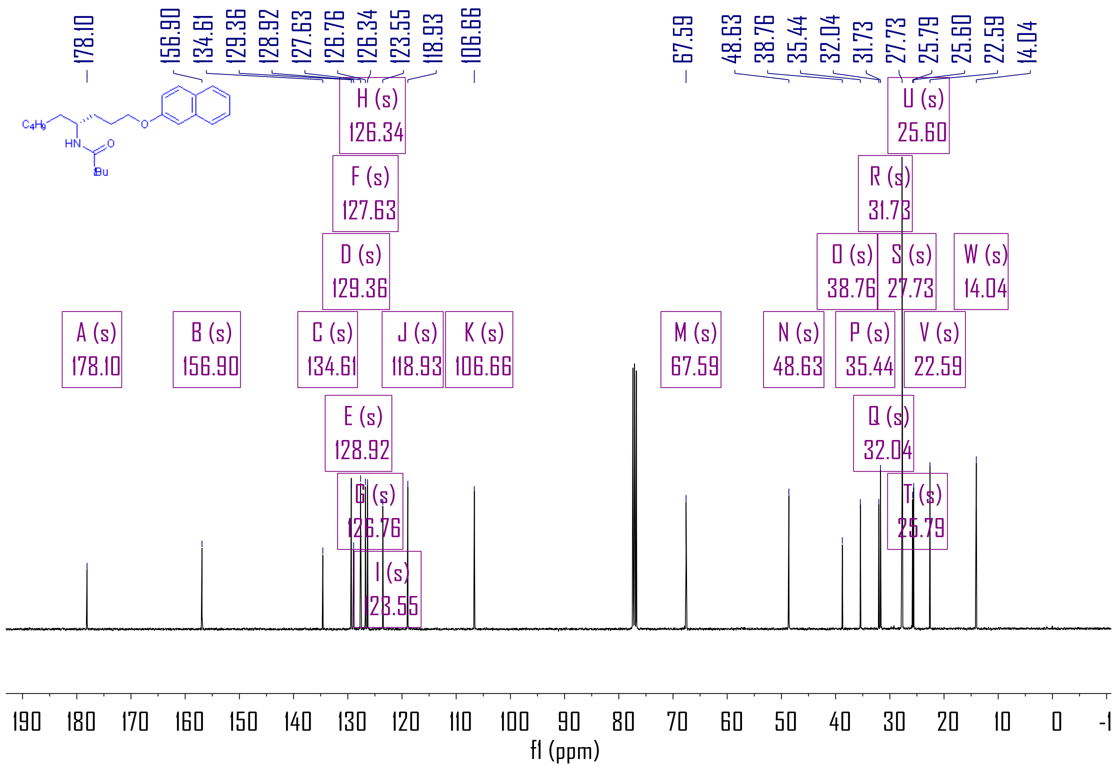

Supplementary Figure 198. ^{13}C NMR spectra for **40**

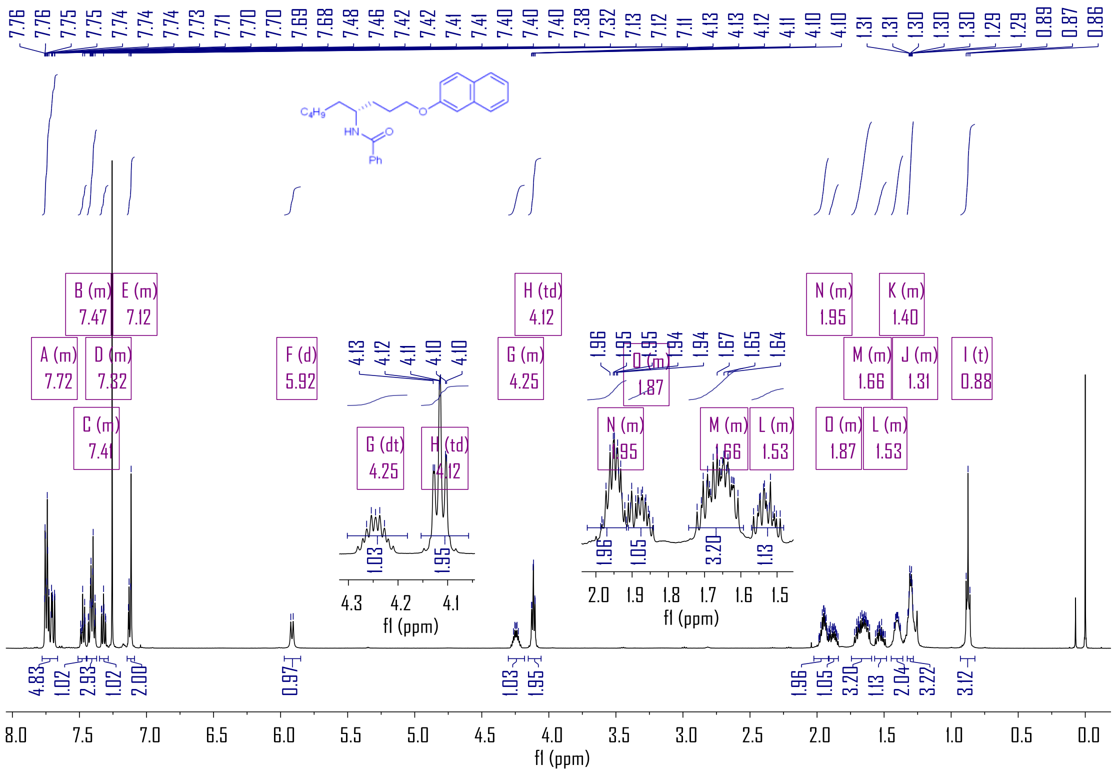

Supplementary Figure 199. ^{1}H NMR spectra for **41**

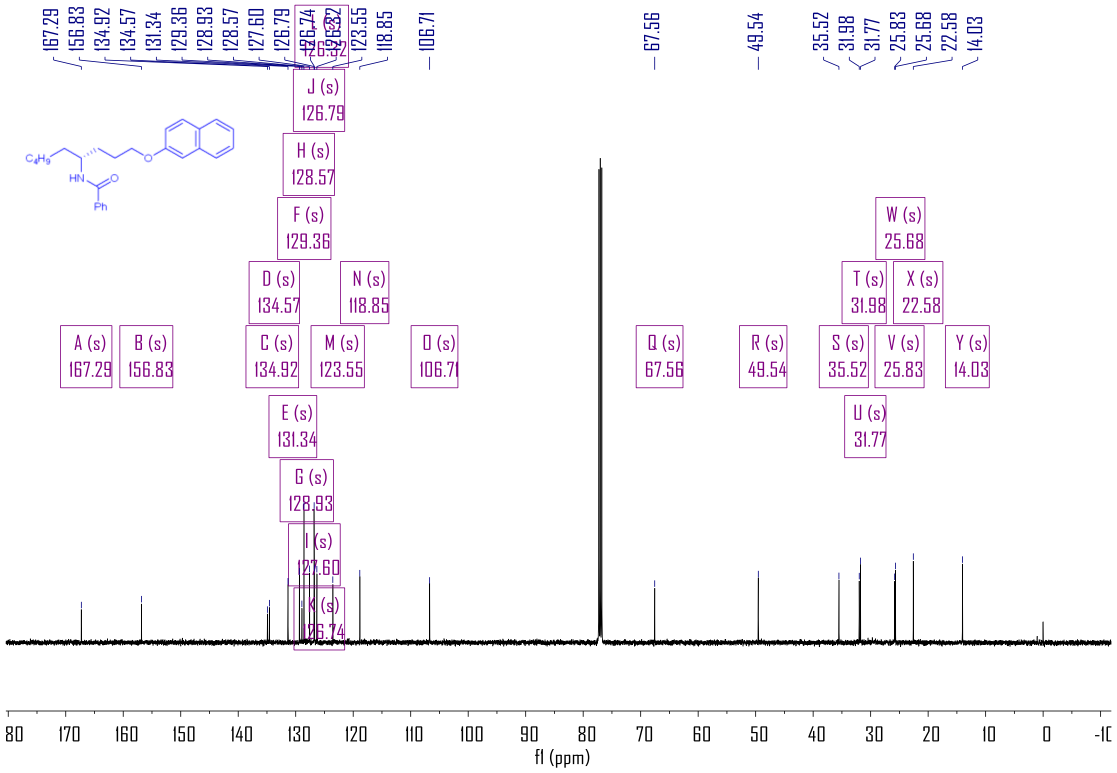

Supplementary Figure 200. ^{13}C NMR spectra for **41**

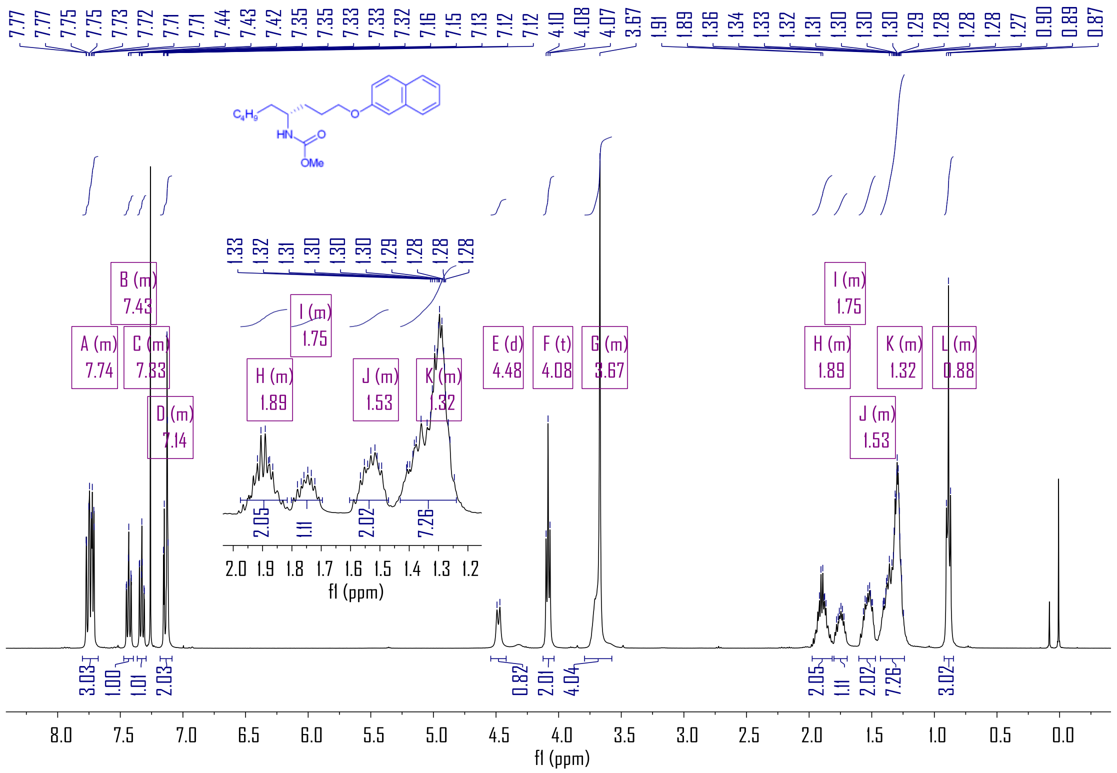

Supplementary Figure 201. ^{1}H NMR spectra for **42**

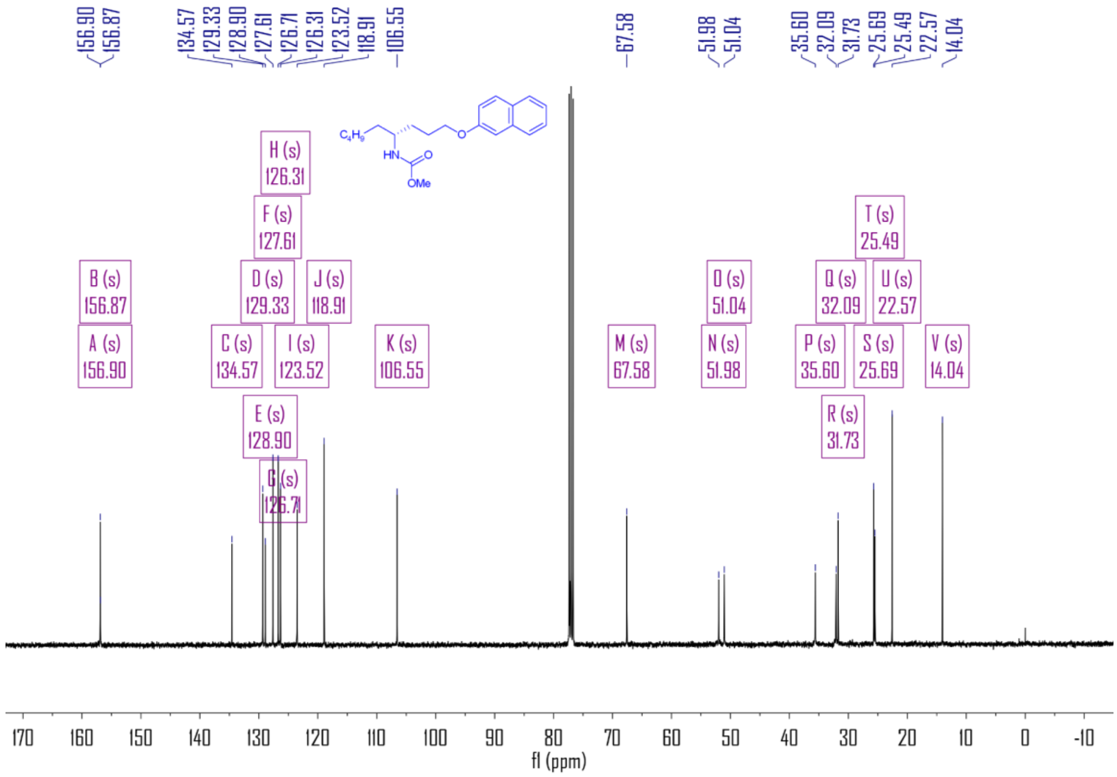

Supplementary Figure 202. ^{13}C NMR spectra for **42**

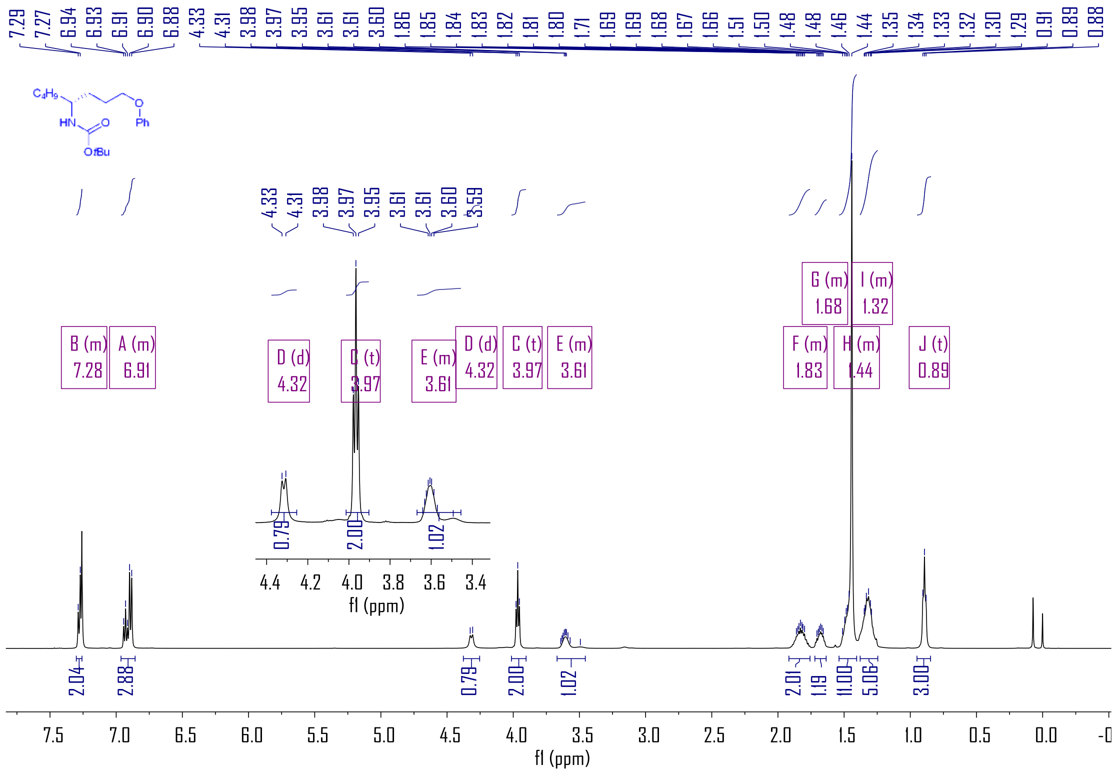

Supplementary Figure 203. ^{1}H NMR spectra for **43**

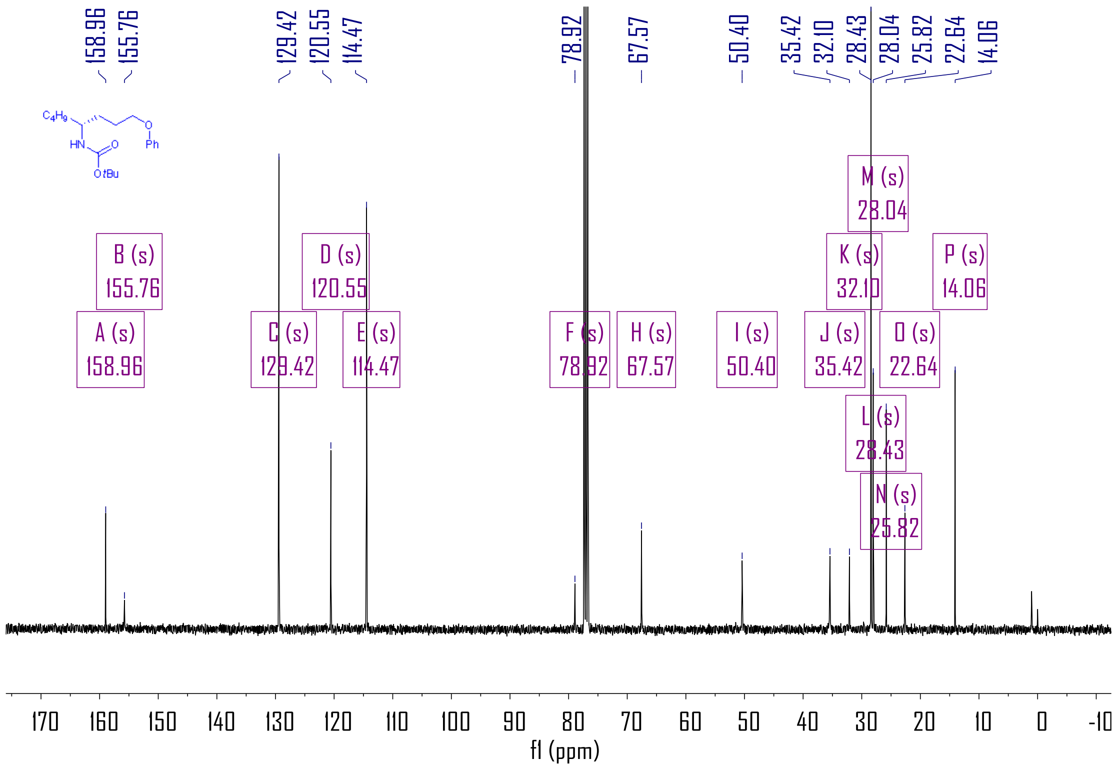

Supplementary Figure 204. ^{13}C NMR spectra for **43**

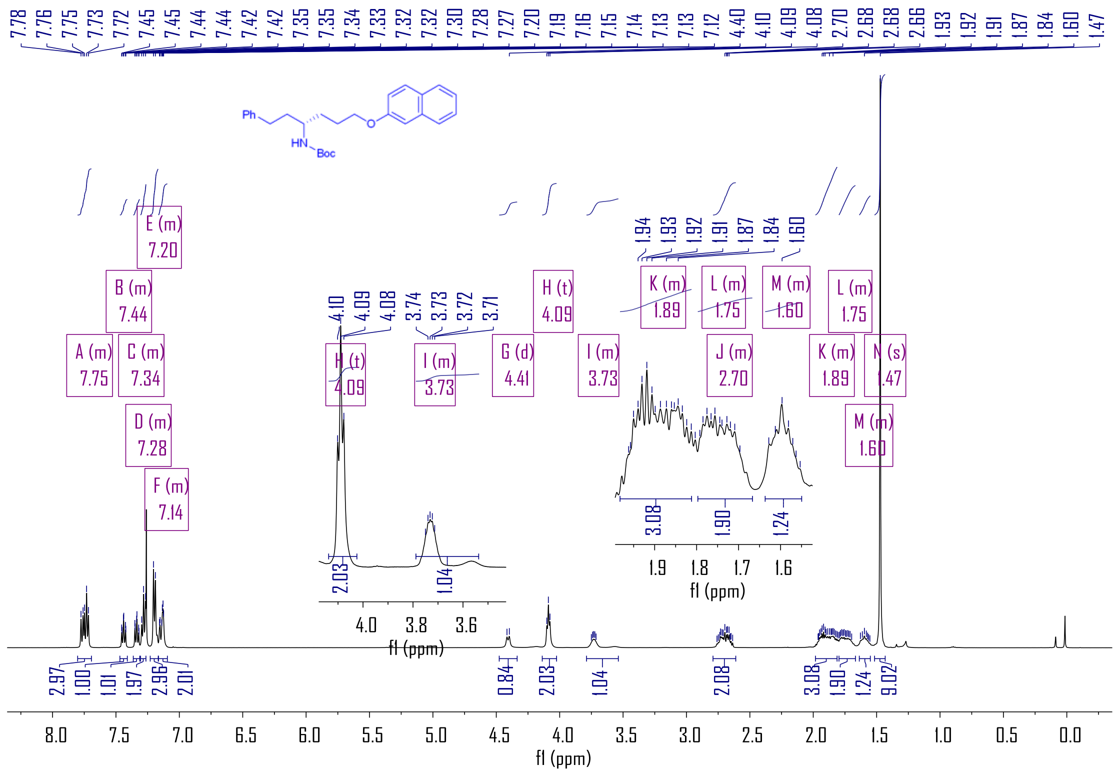

Supplementary Figure 205. ^{1}H NMR spectra for **44**

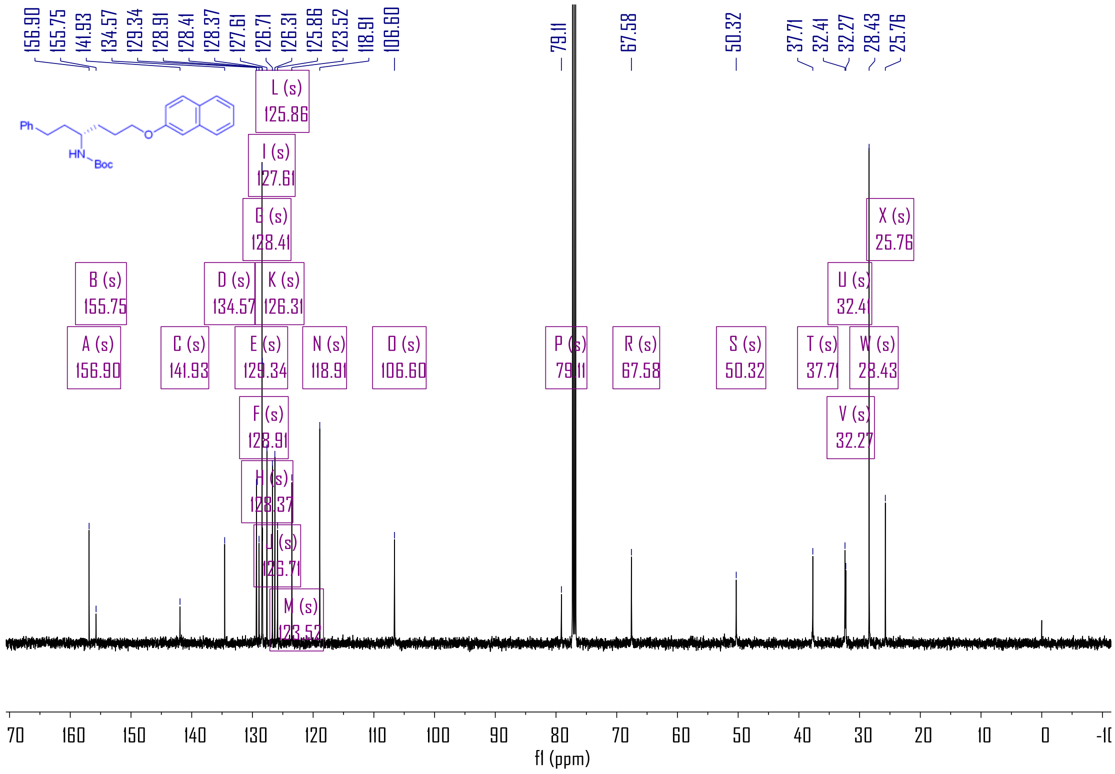

Supplementary Figure 206. ^{13}C NMR spectra for **44**

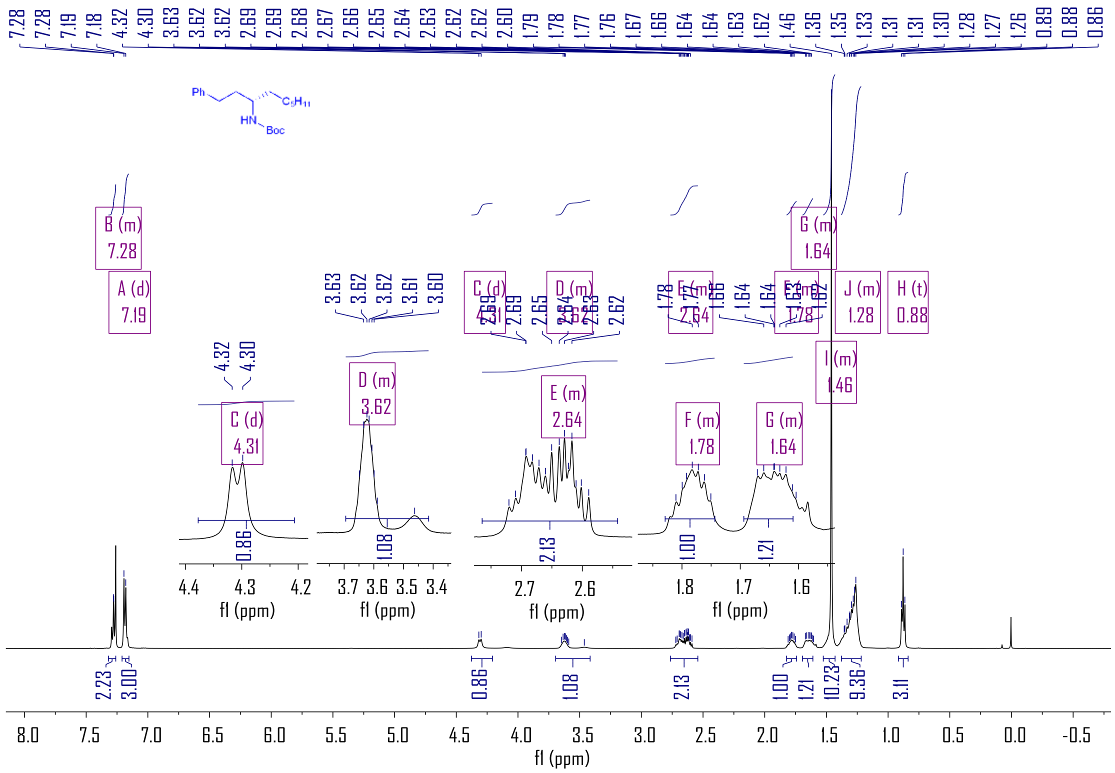

Supplementary Figure 207. ^{1}H NMR spectra for **45**

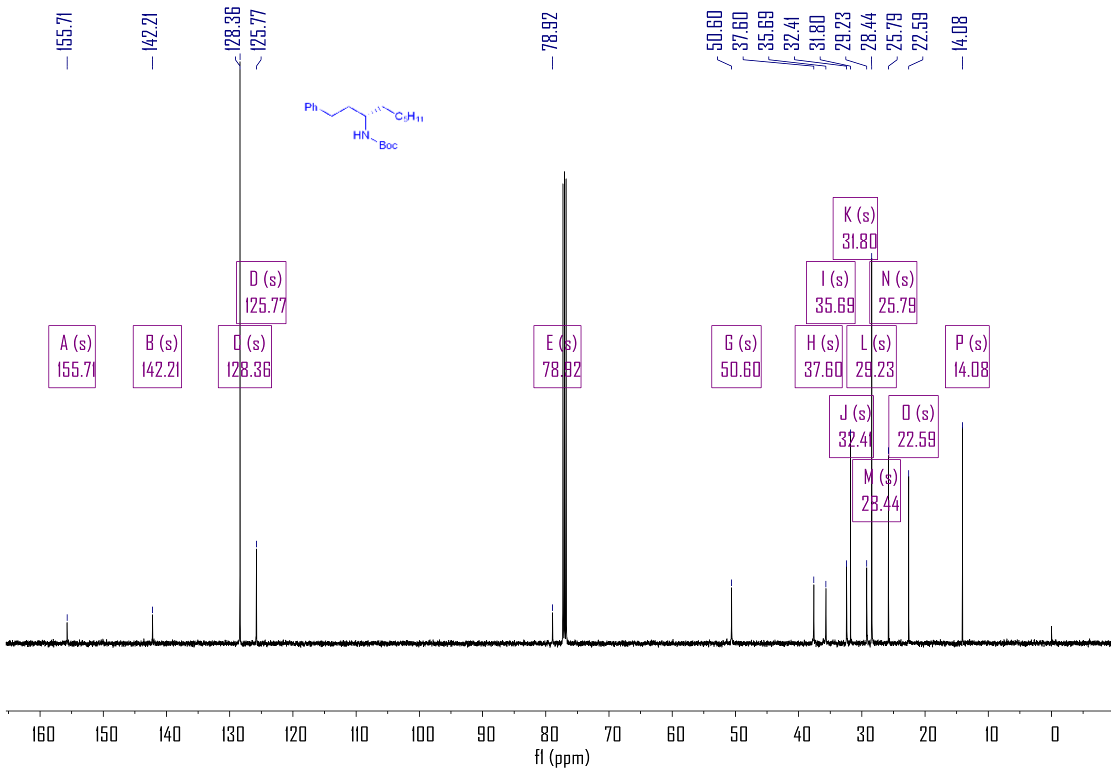

Supplementary Figure 208. ^{13}C NMR spectra for **45**

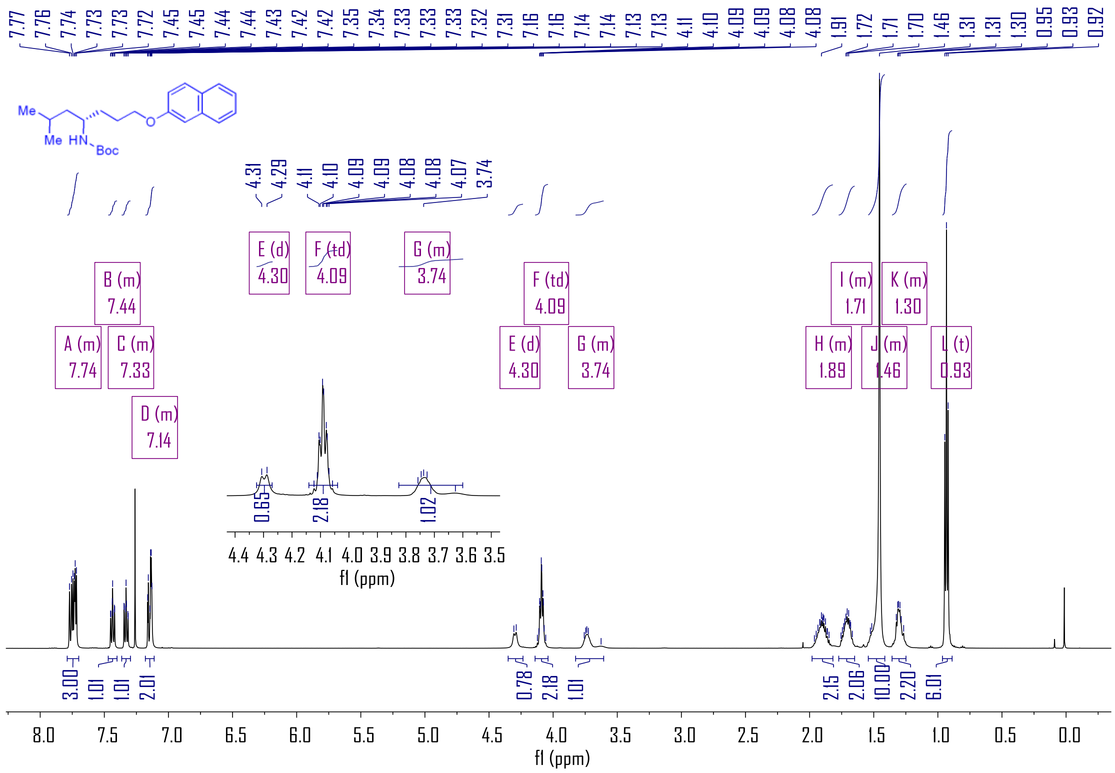

Supplementary Figure 209. ^{1}H NMR spectra for **46**

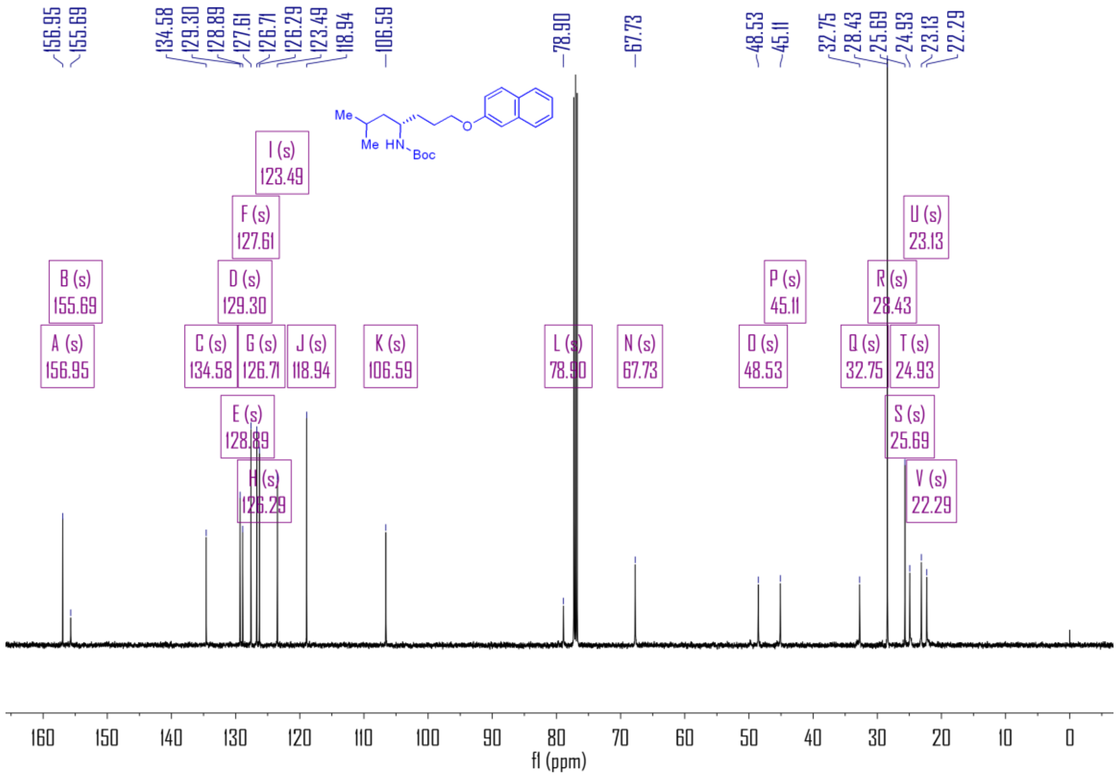

Supplementary Figure 210. ^{13}C NMR spectra for **46**

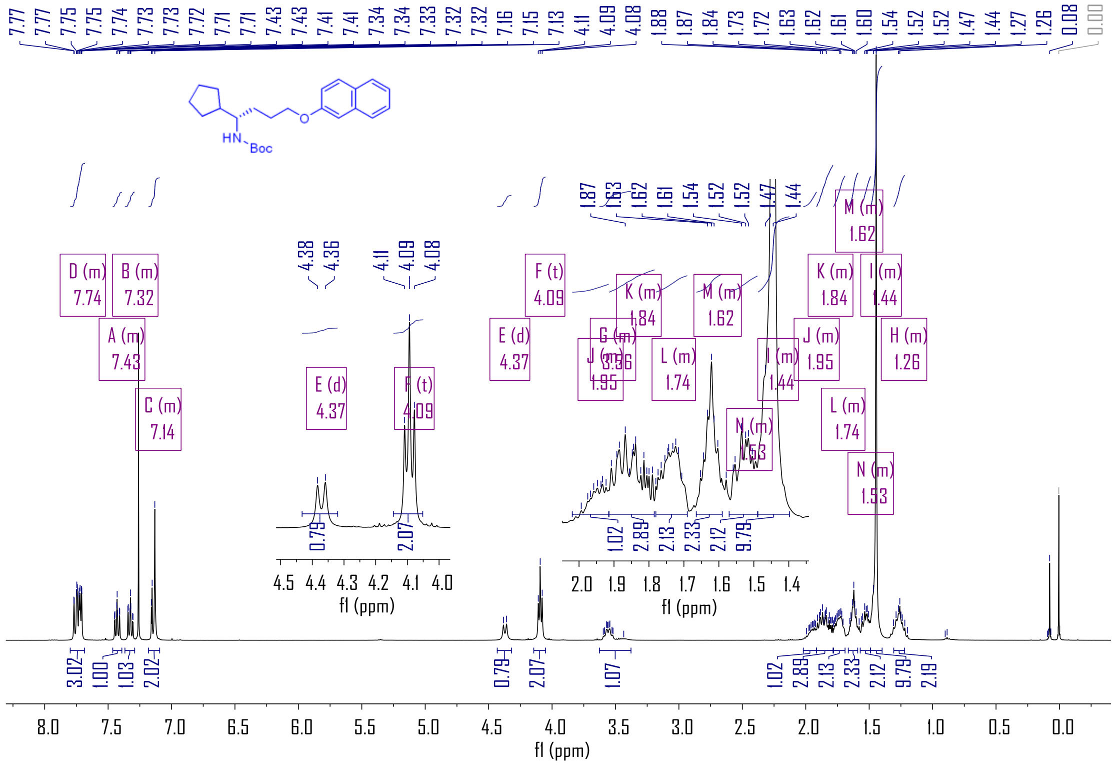

Supplementary Figure 211. ^{1}H NMR spectra for **47**

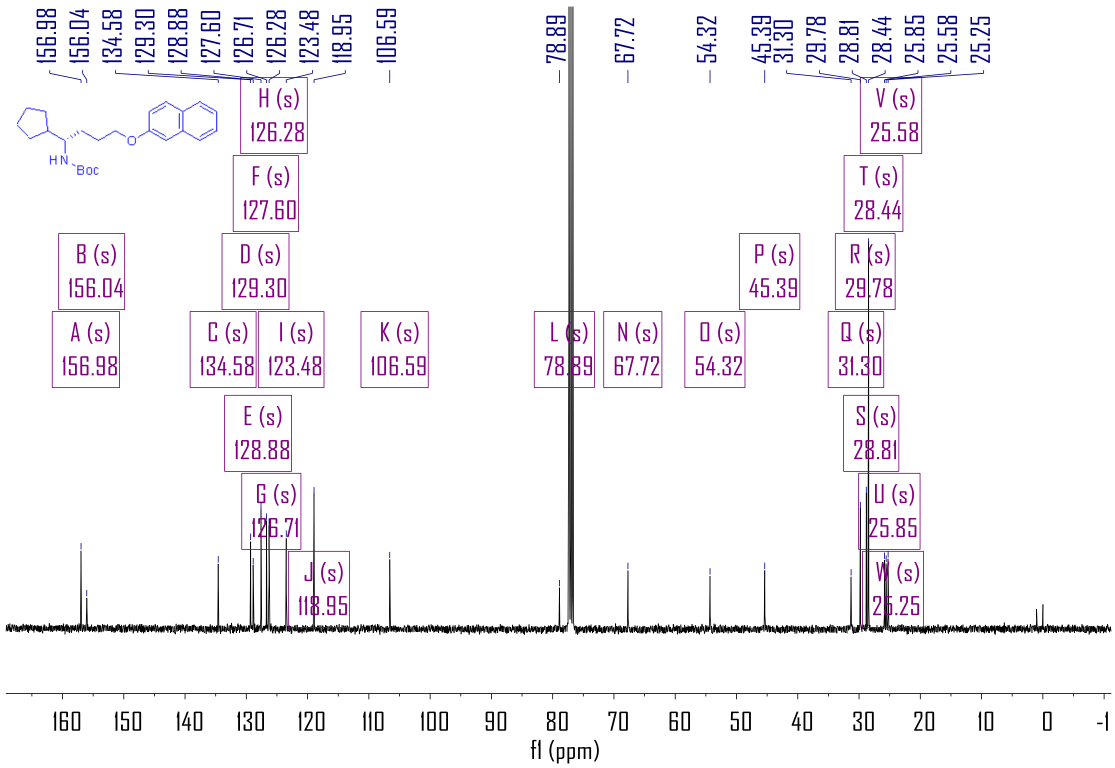

Supplementary Figure 212. ^{13}C NMR spectra for **47**

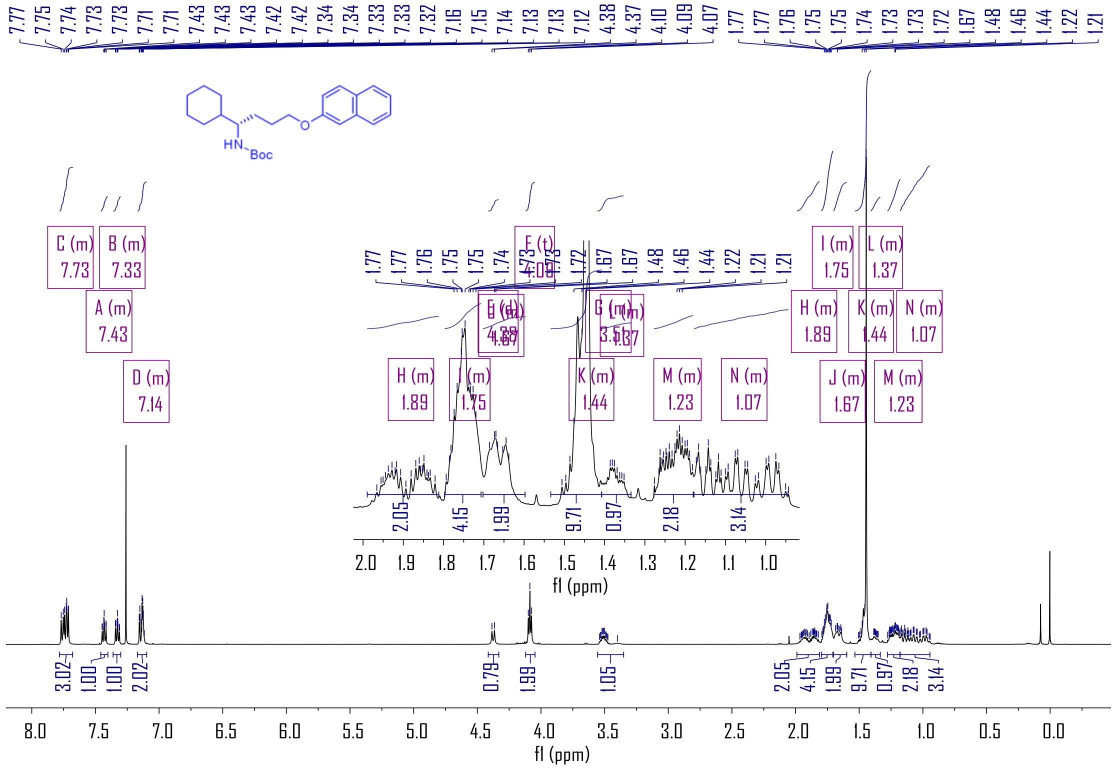

Supplementary Figure 213. ^{1}H NMR spectra for **48**

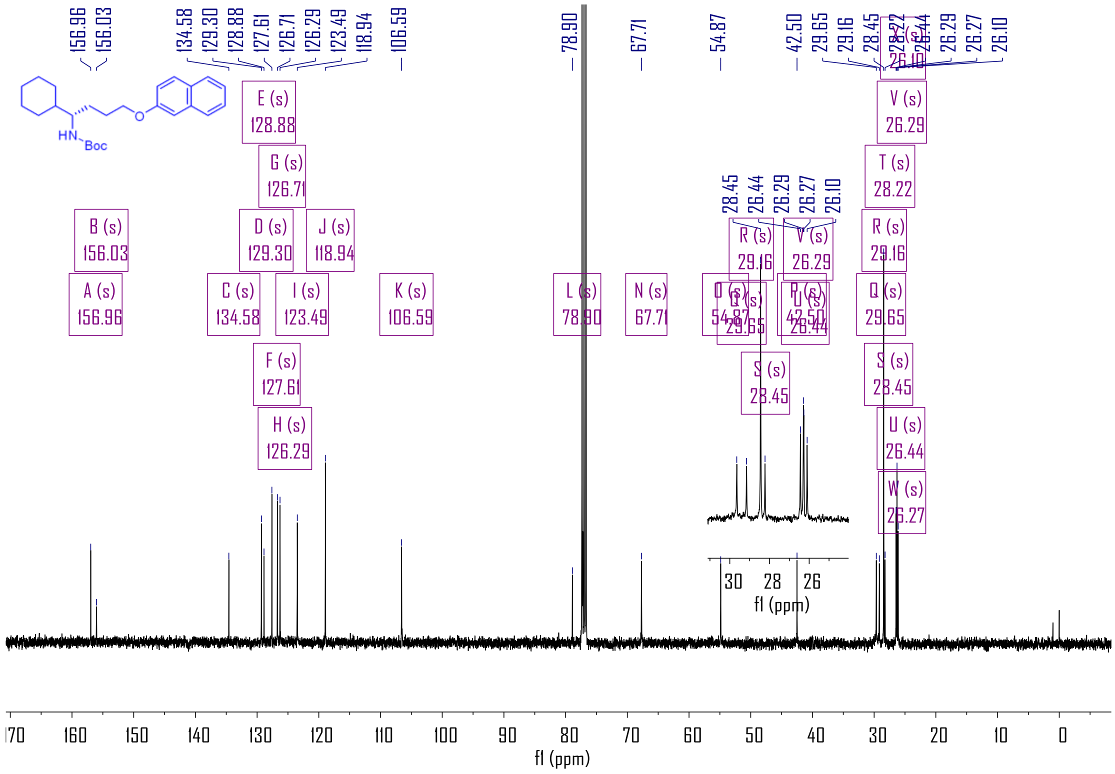

Supplementary Figure 214. ^{13}C NMR spectra for **48**

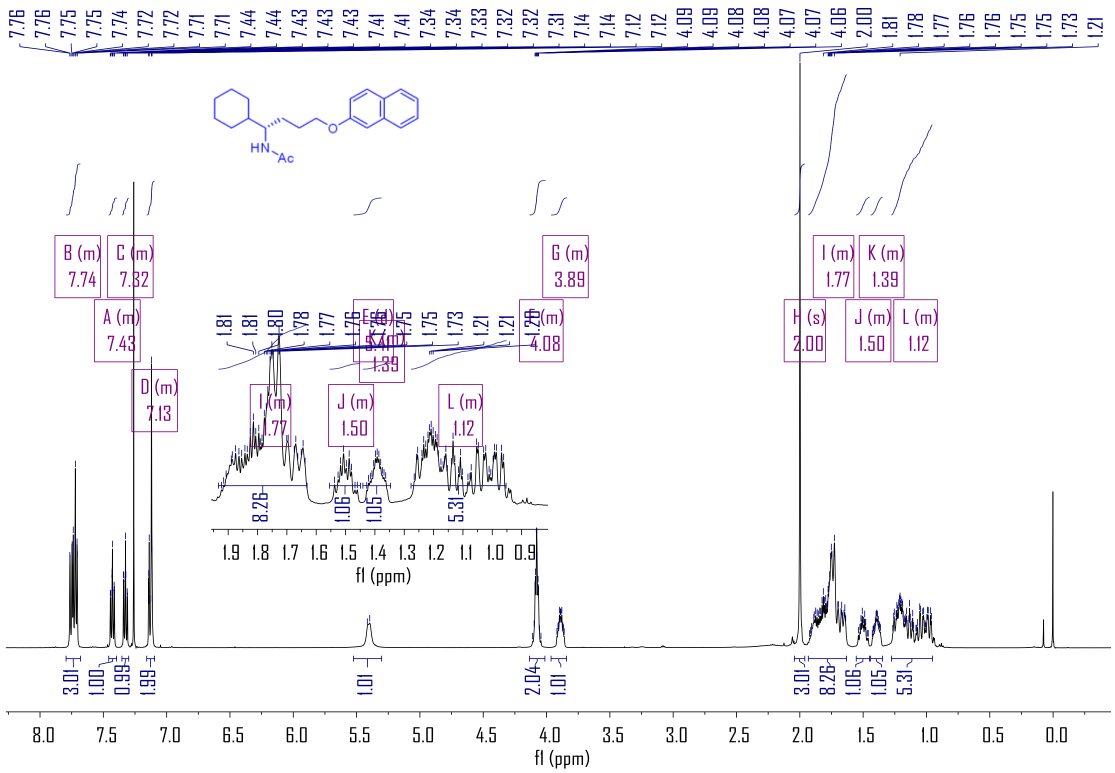

Supplementary Figure 215. ^{1}H NMR spectra for **49**

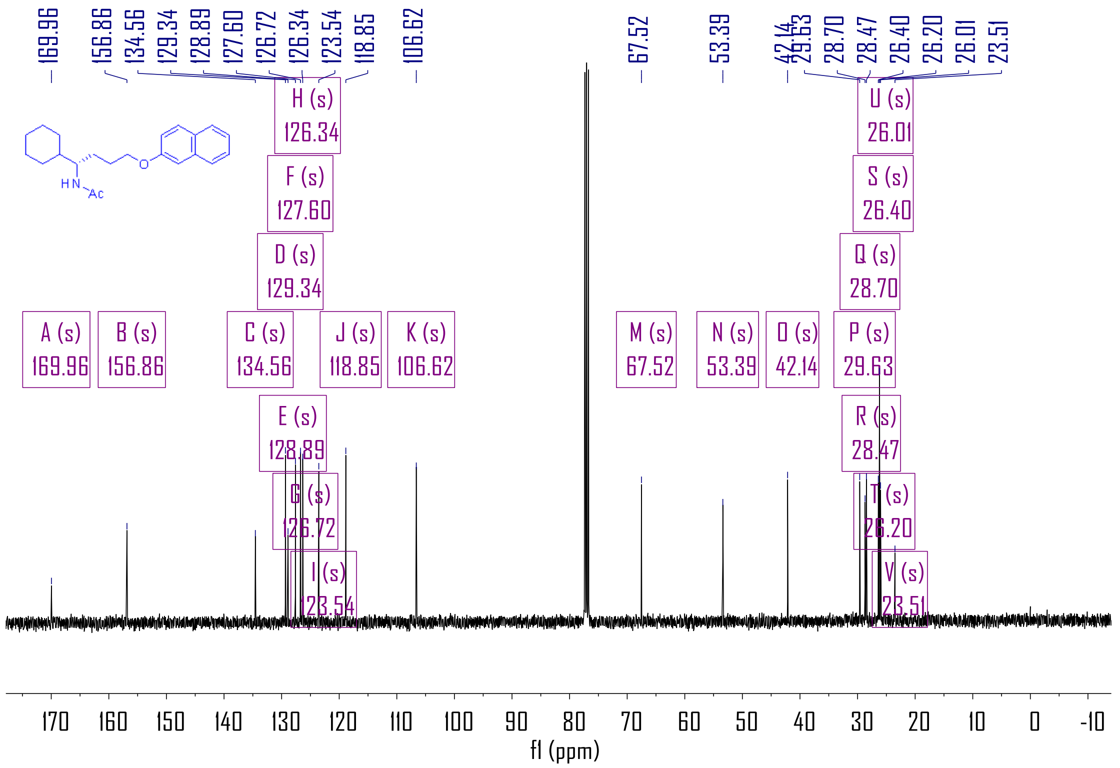

Supplementary Figure 216. ^{13}C NMR spectra for **49**

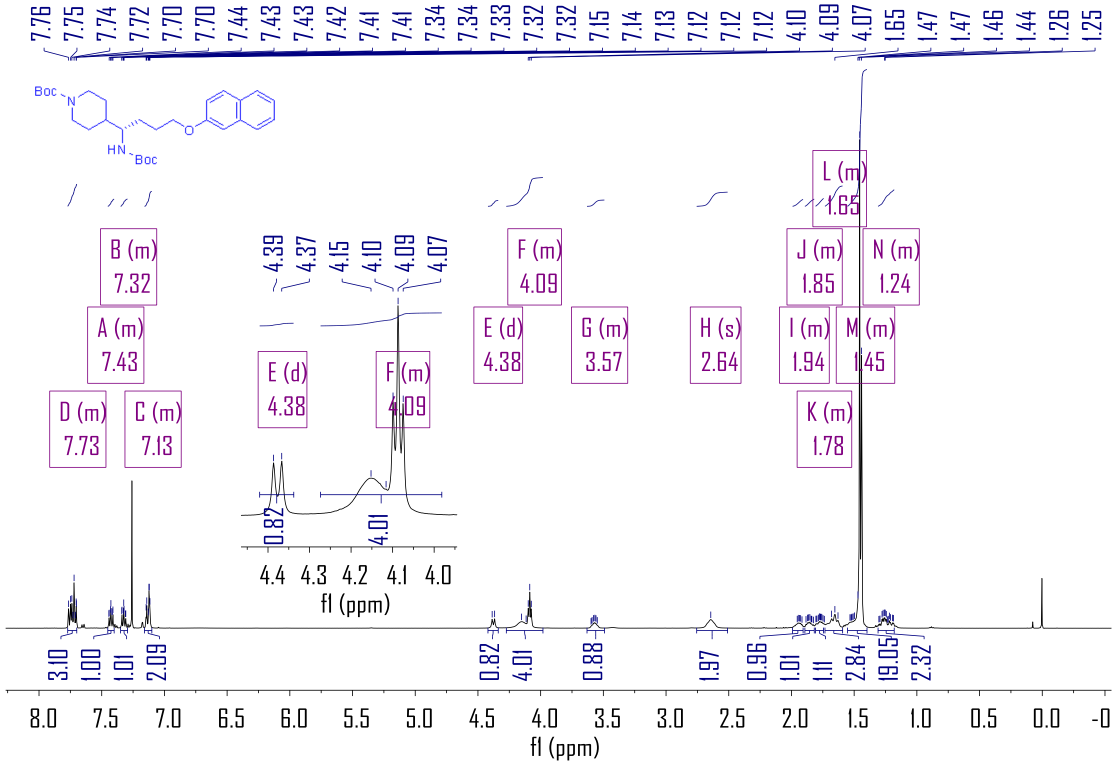

Supplementary Figure 217. ^{1}H NMR spectra for **50**

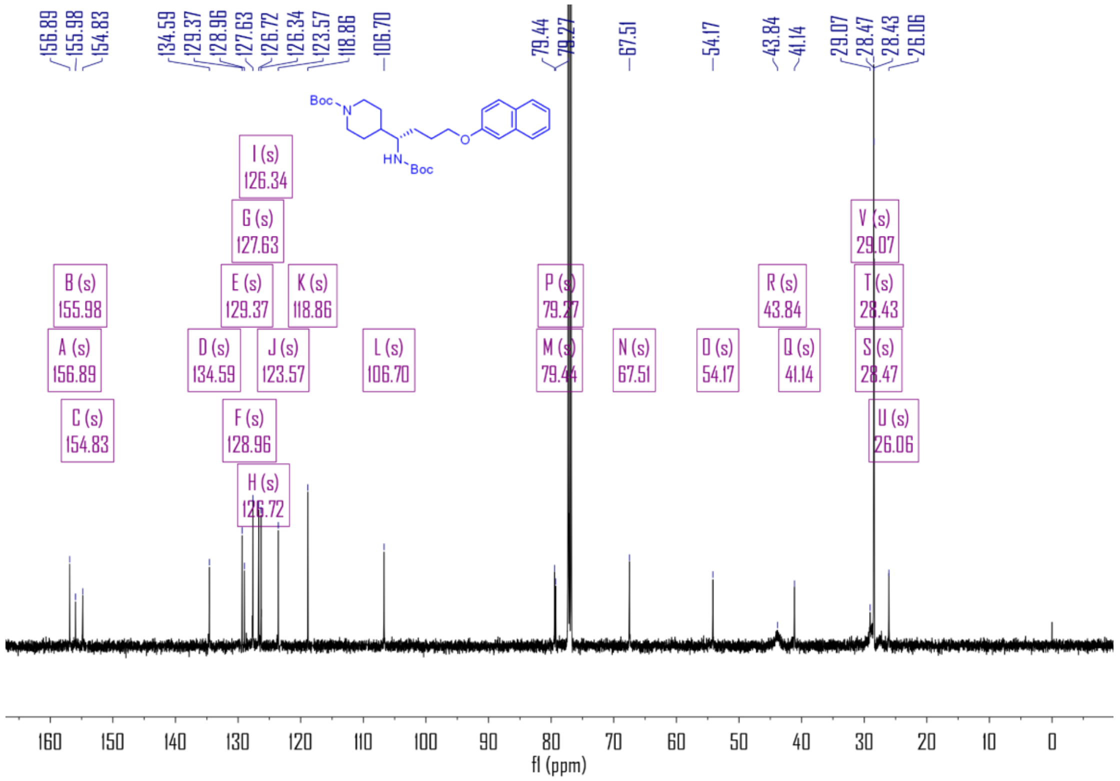

Supplementary Figure 218. ^{13}C NMR spectra for **50**

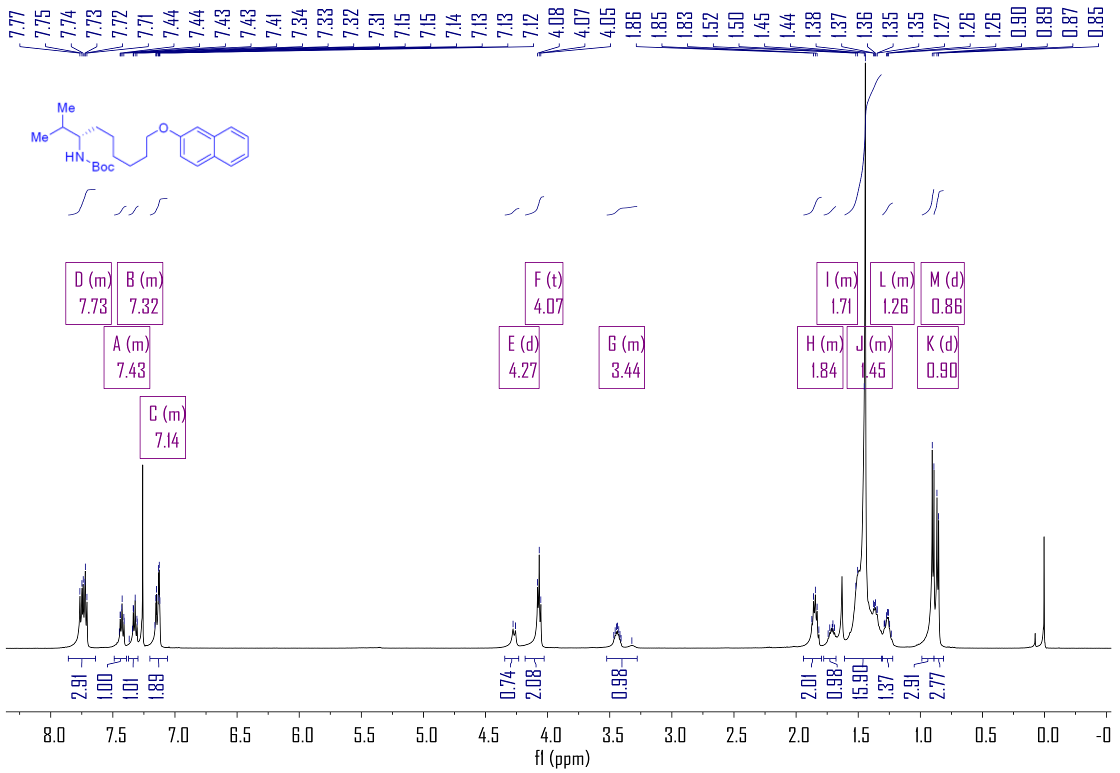

Supplementary Figure 219. ^{1}H NMR spectra for **51**

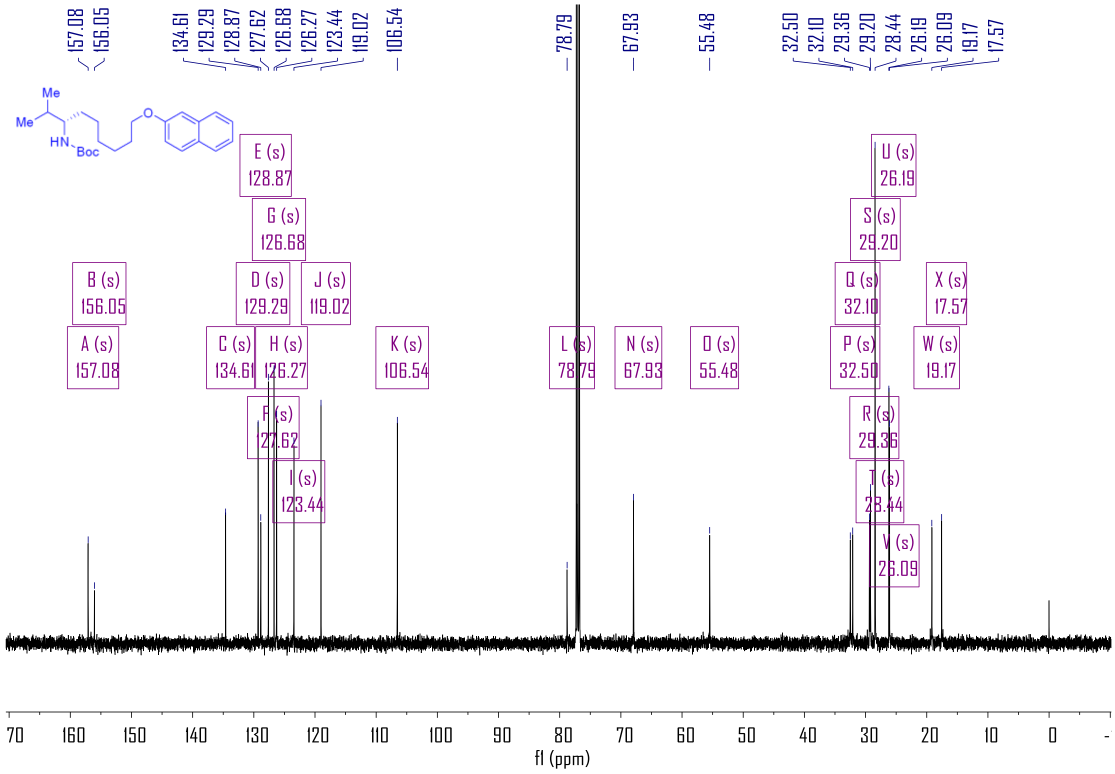

Supplementary Figure 220. ^{13}C NMR spectra for **51**

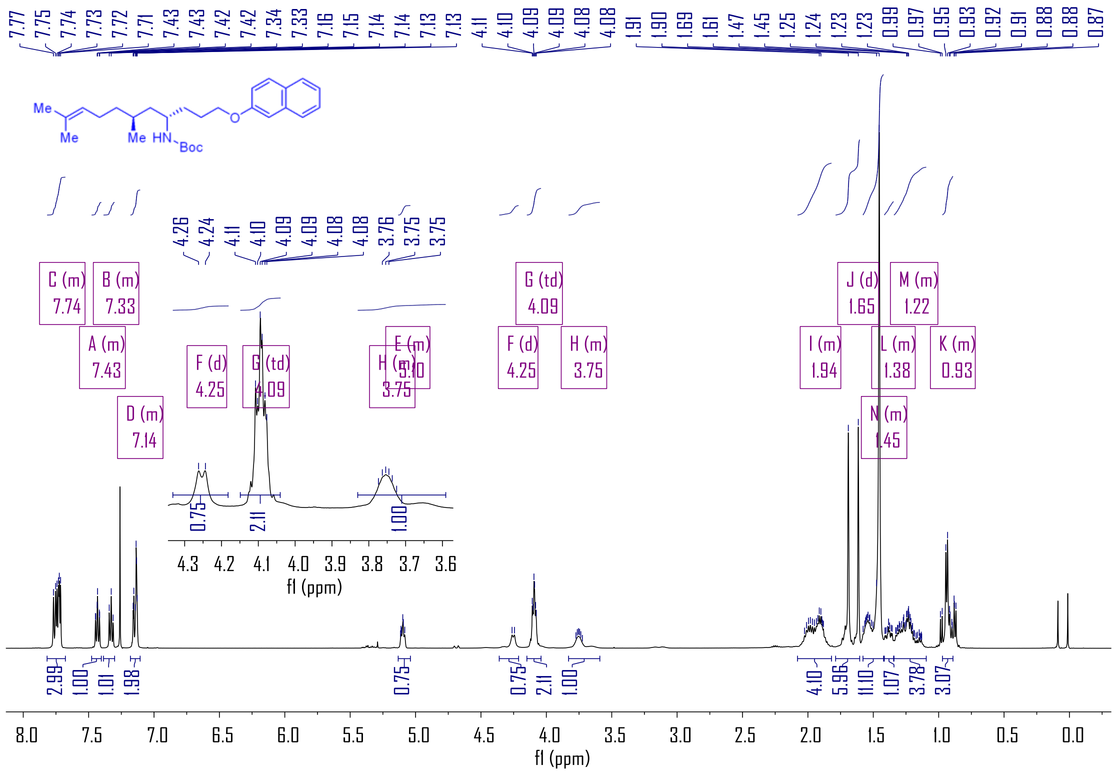

Supplementary Figure 221. ^{1}H NMR spectra for **52**

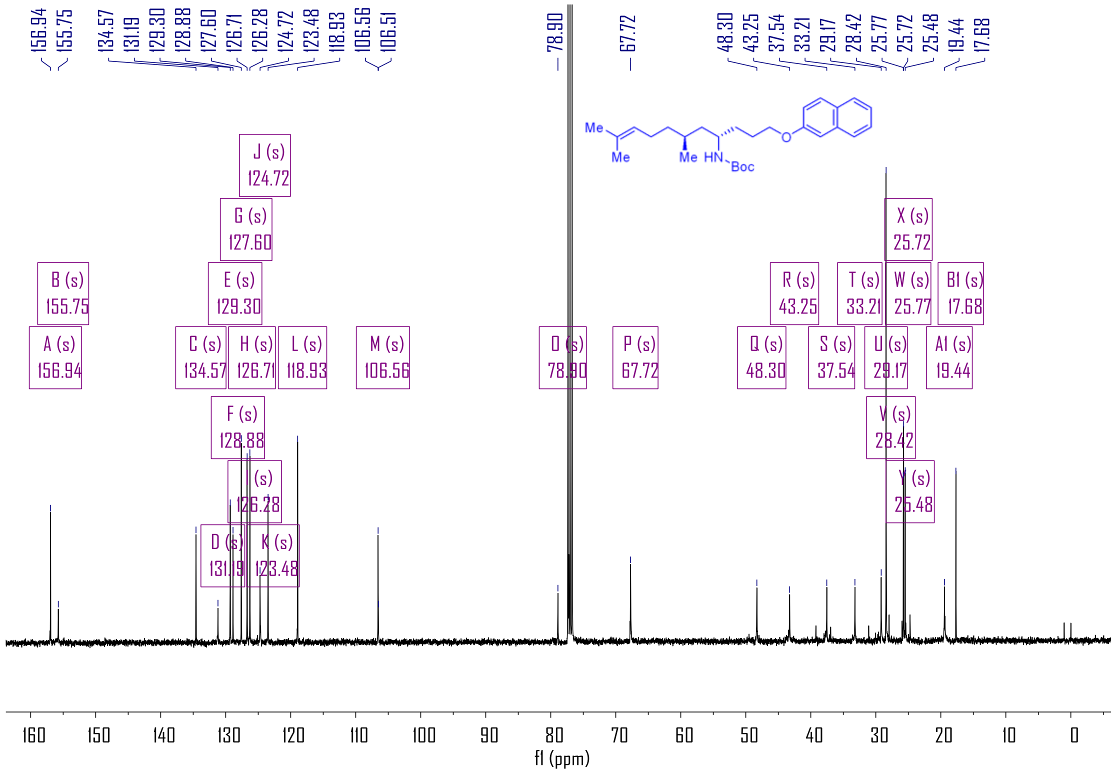

Supplementary Figure 222. ^{13}C NMR spectra for **52**

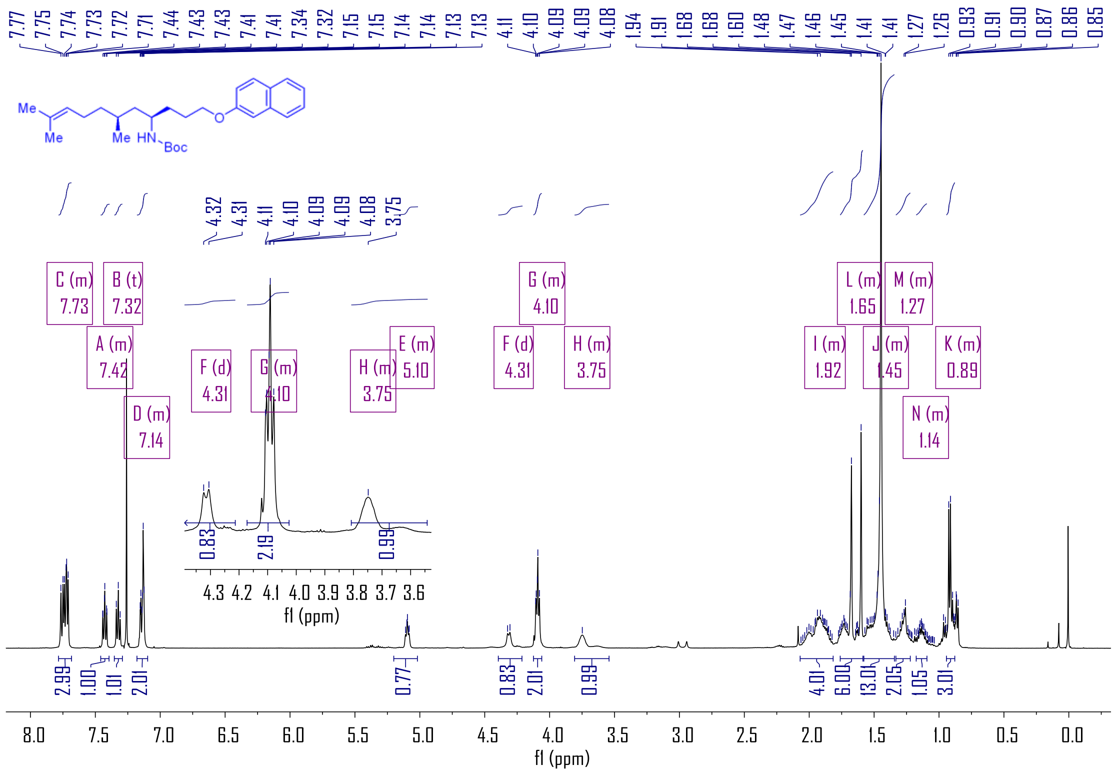

Supplementary Figure 223. ^{1}H NMR spectra for **53**

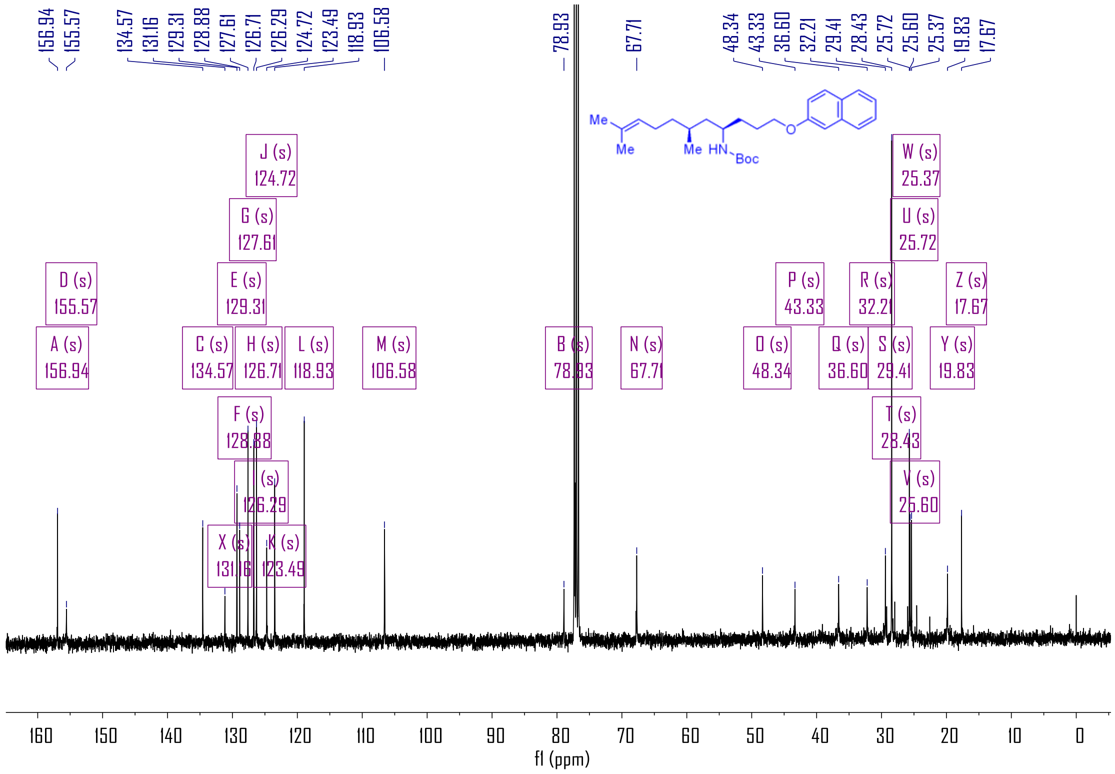

Supplementary Figure 224. ^{13}C NMR spectra for **53**

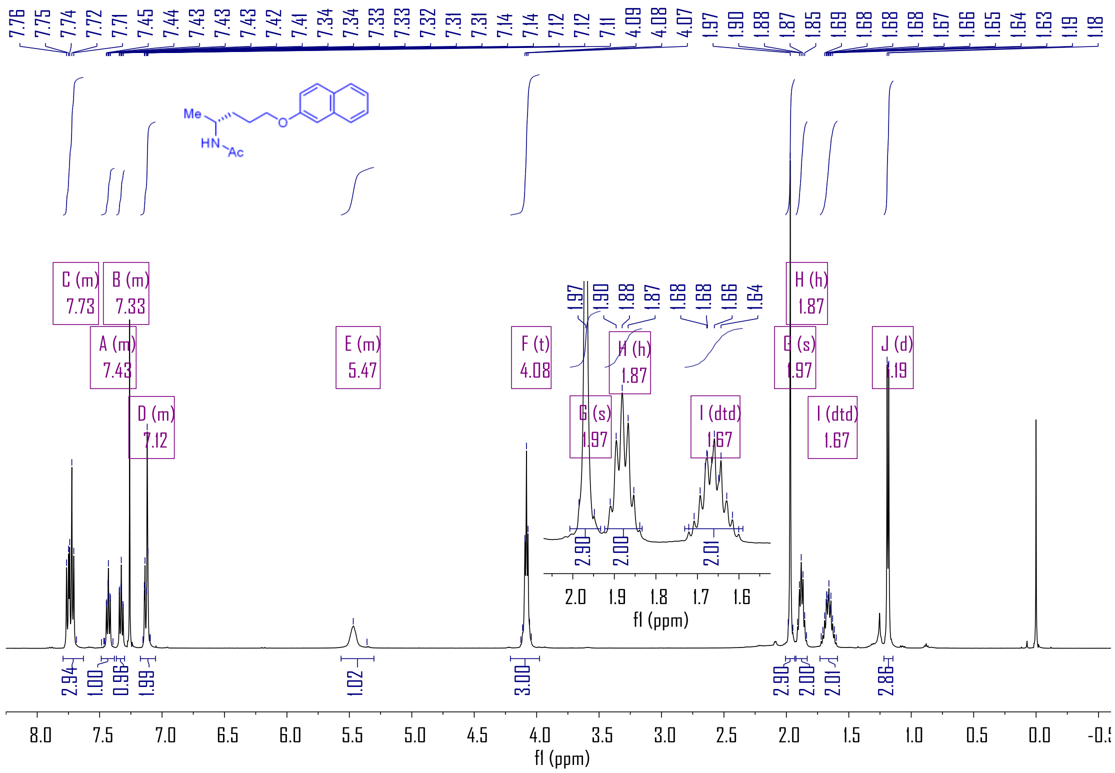

Supplementary Figure 225. ^{1}H NMR spectra for **54**

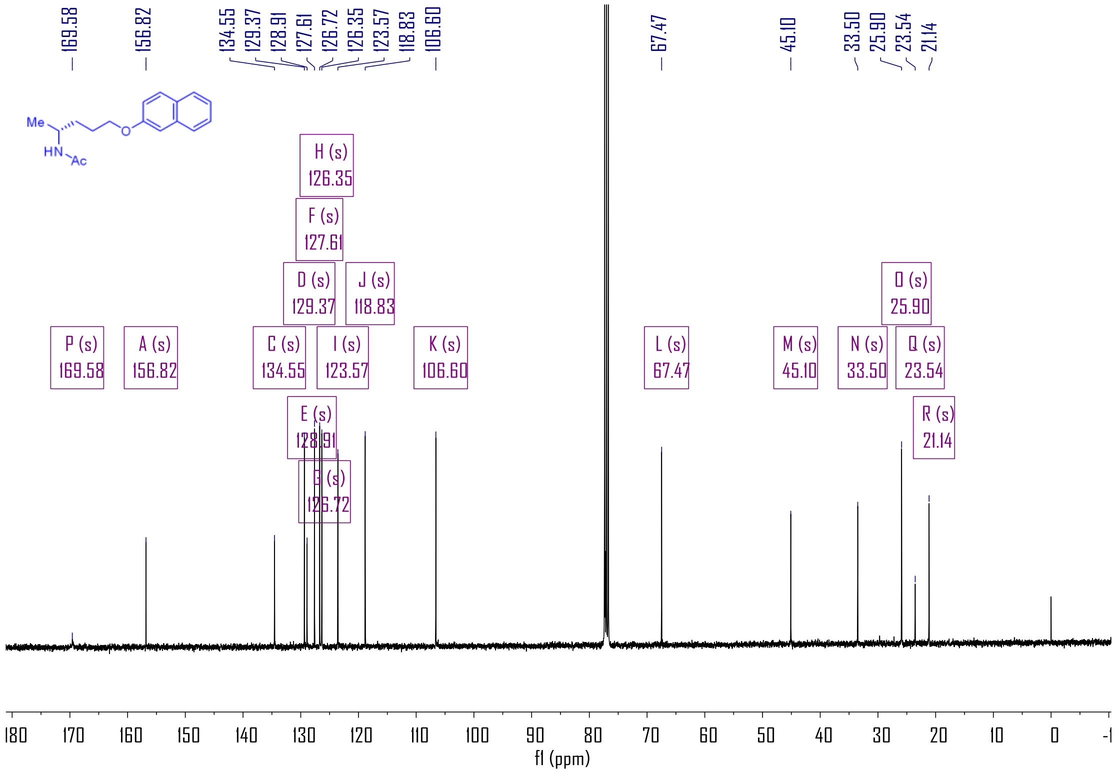

Supplementary Figure 226. ^{13}C NMR spectra for **54**

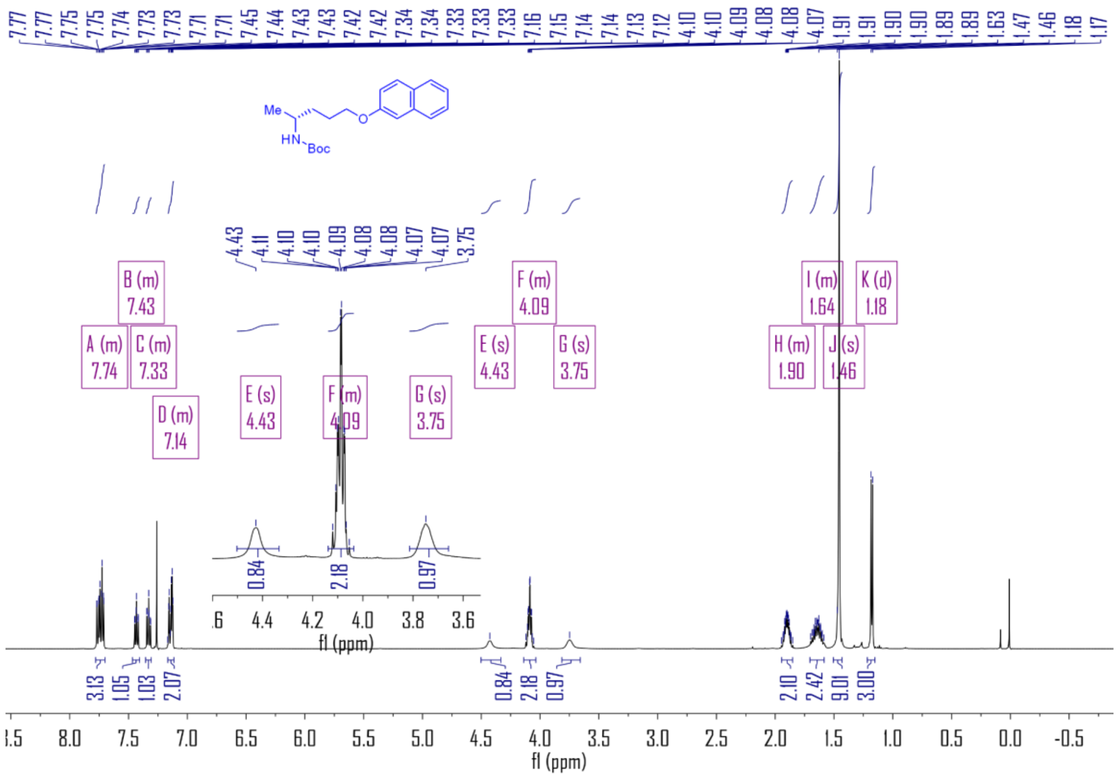

Supplementary Figure 227. ^{1}H NMR spectra for **55**

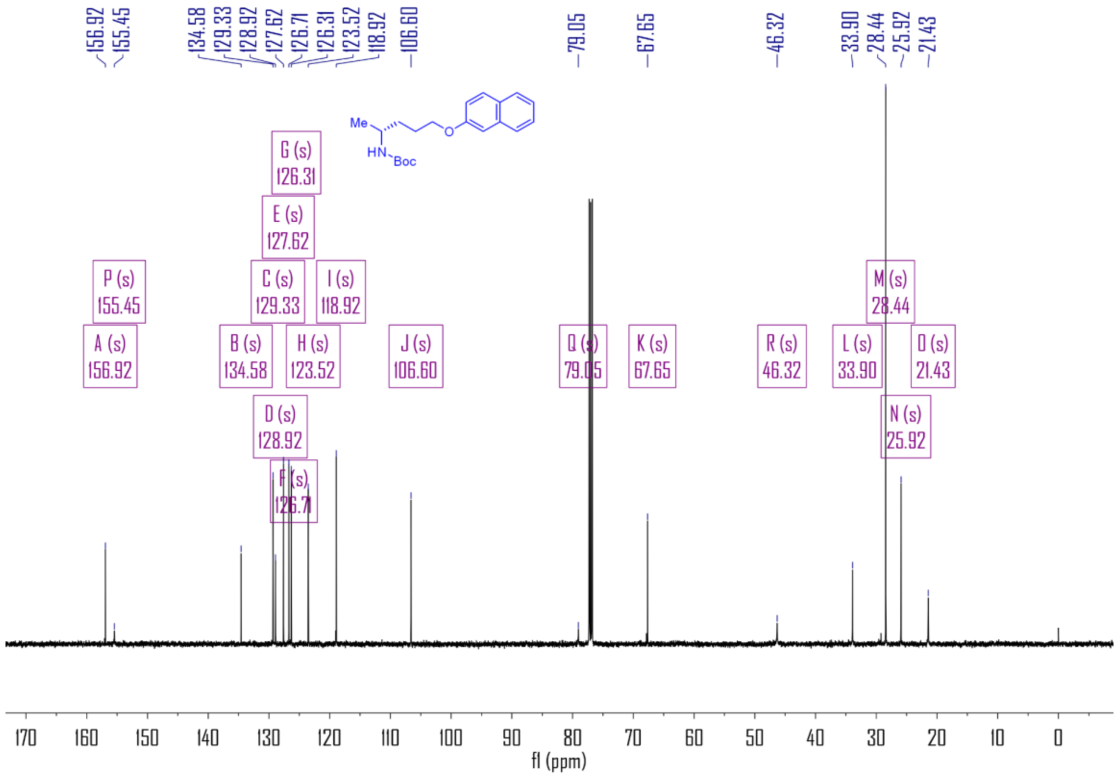

Supplementary Figure 228. ^{13}C NMR spectra for **55**

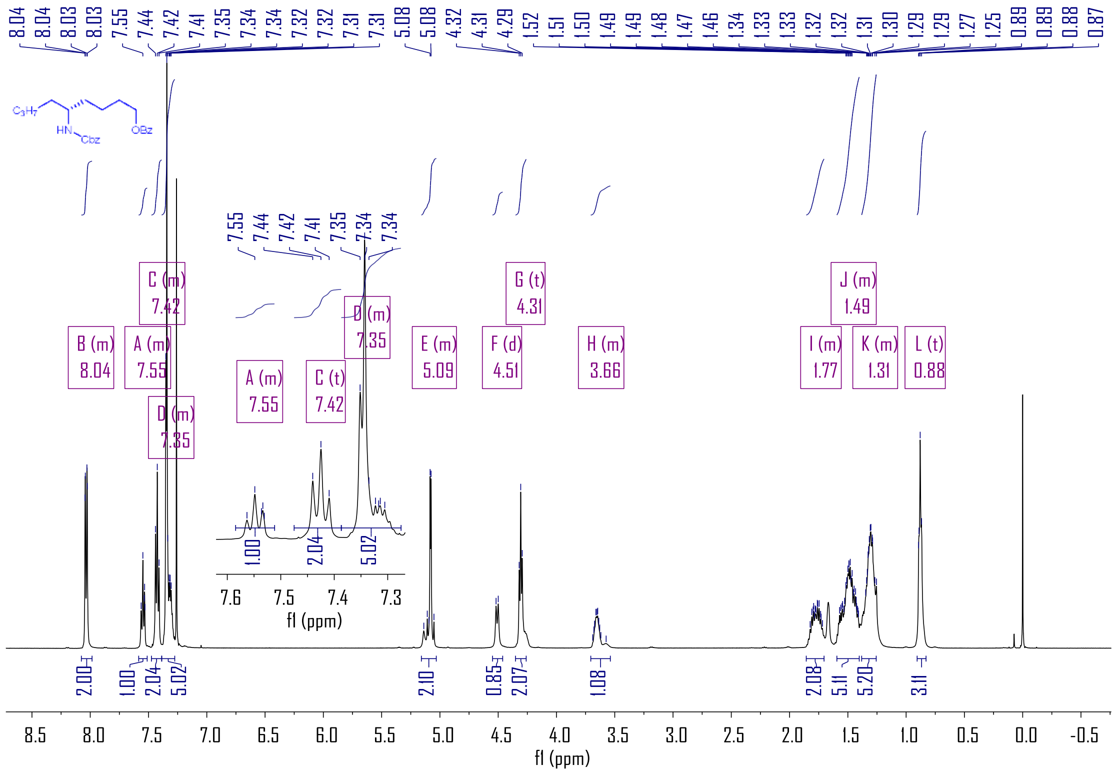

Supplementary Figure 229. ^{1}H NMR spectra for **56**

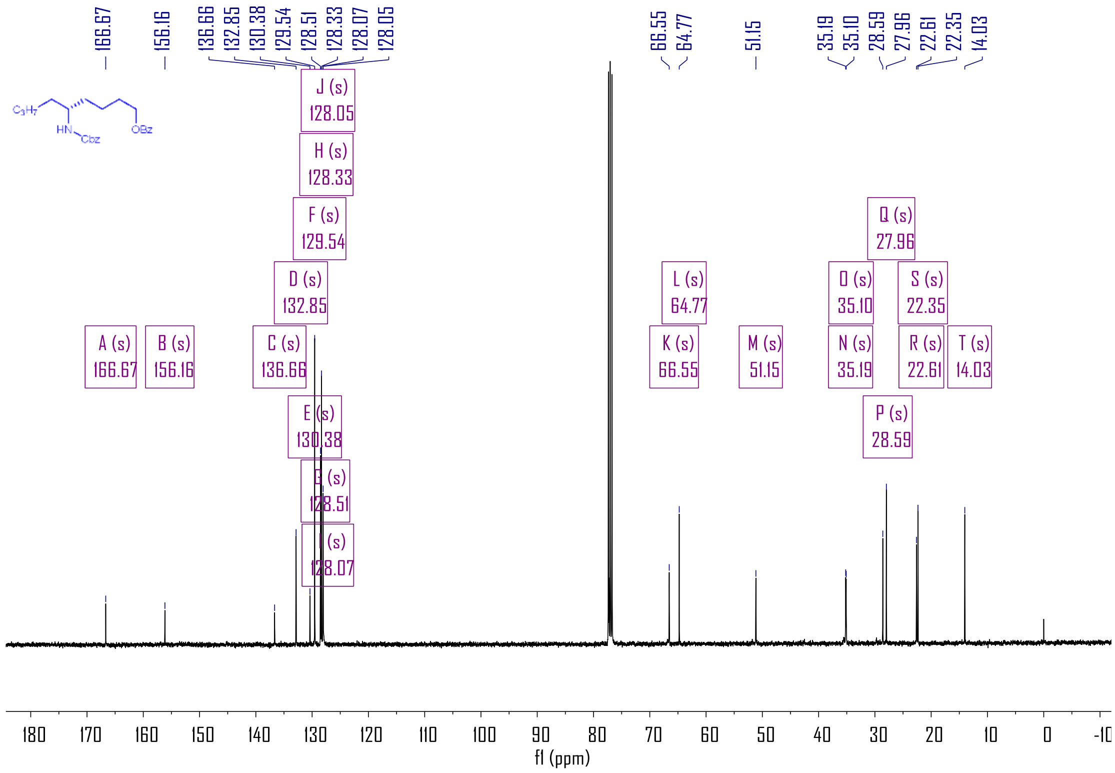

Supplementary Figure 230. ^{13}C NMR spectra for **56**

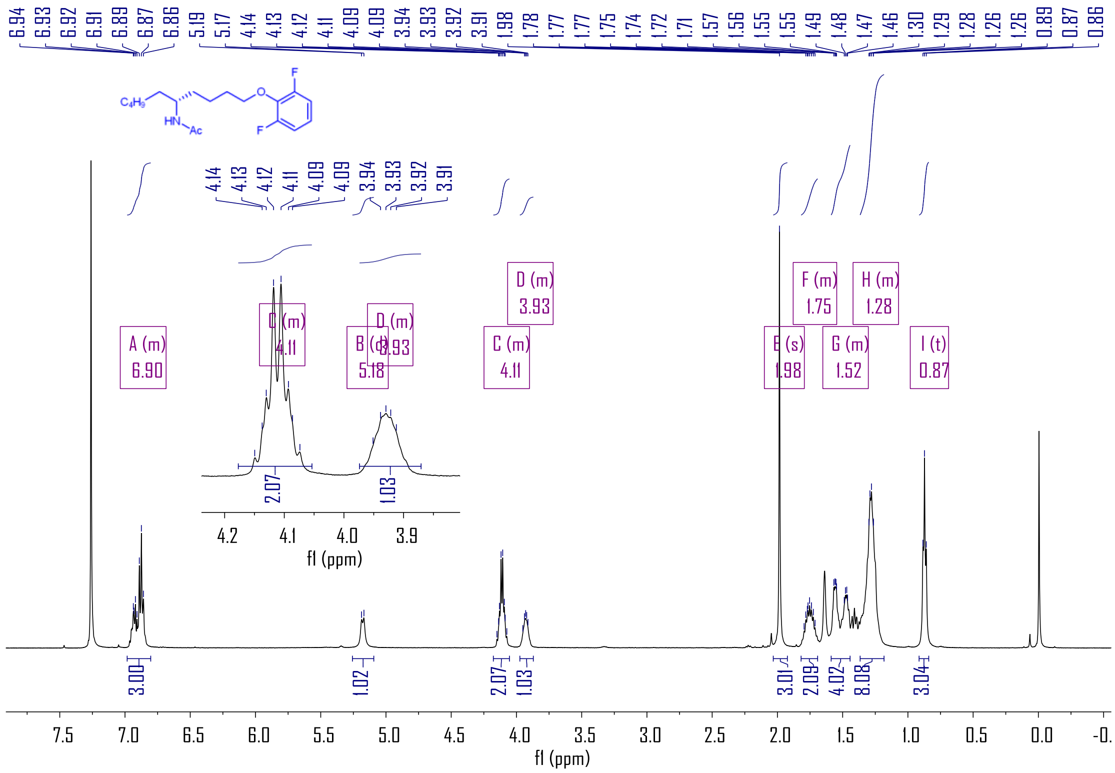

Supplementary Figure 231. ^{1}H NMR spectra for **57**

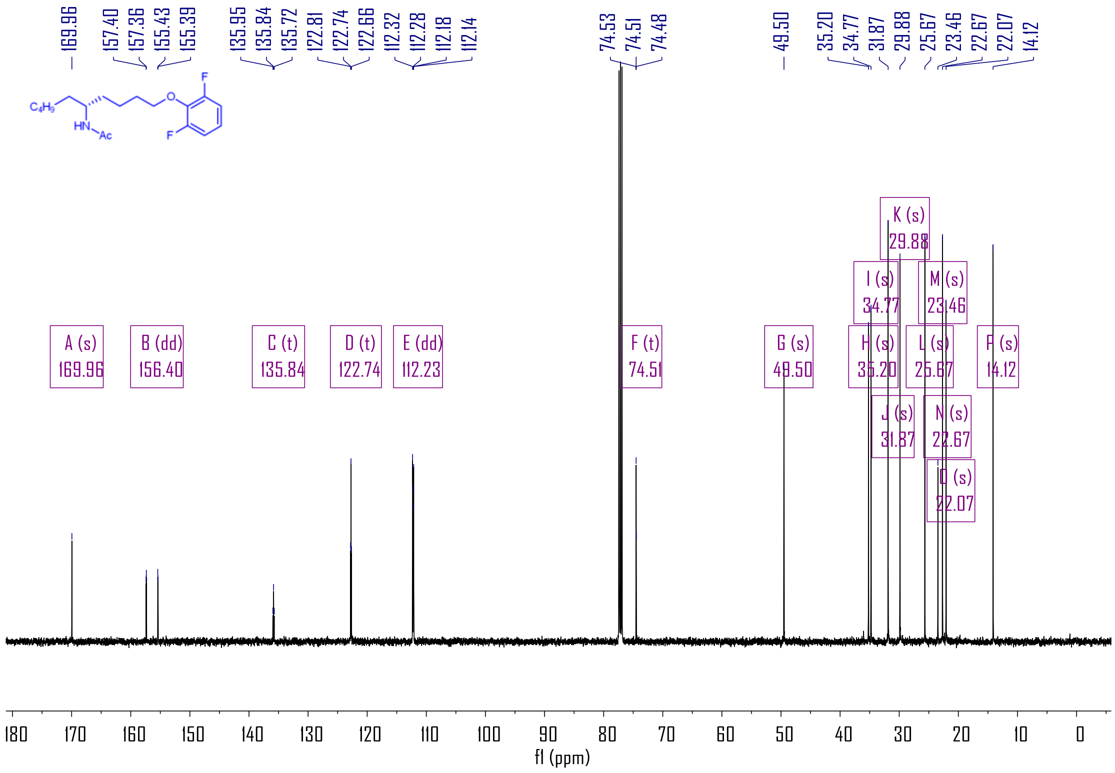

Supplementary Figure 232. ^{13}C NMR spectra for **57**

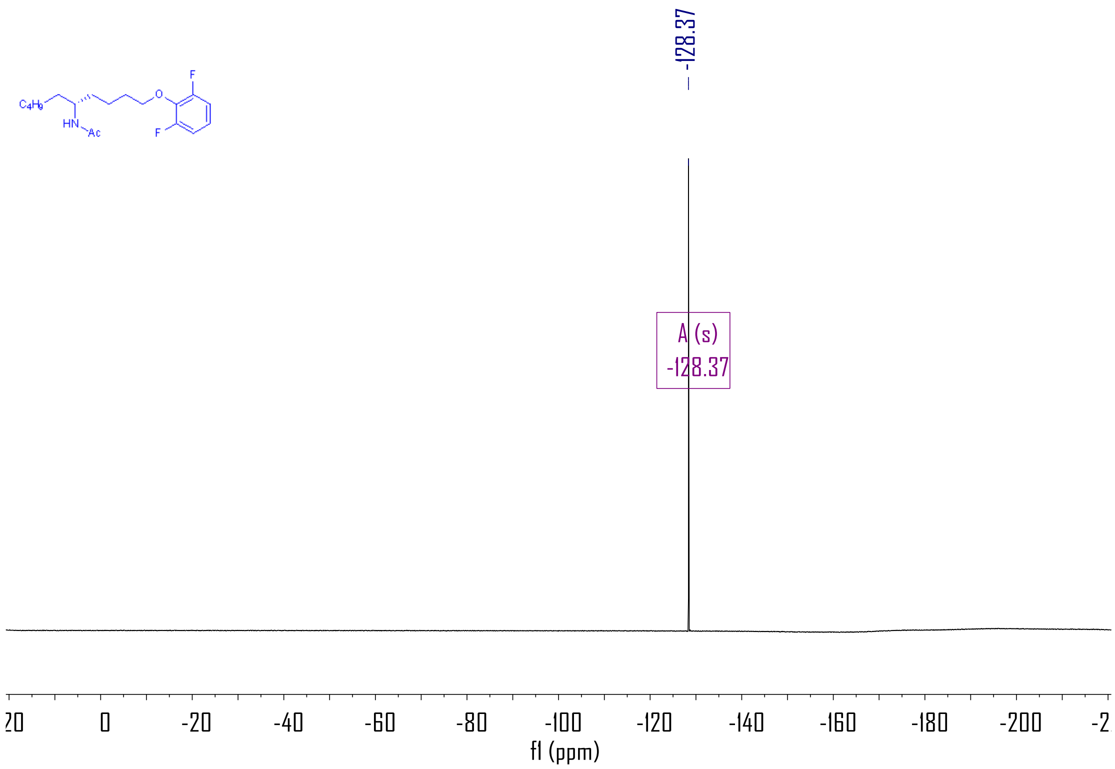

Supplementary Figure 233. ^{19}F NMR spectra for **57**

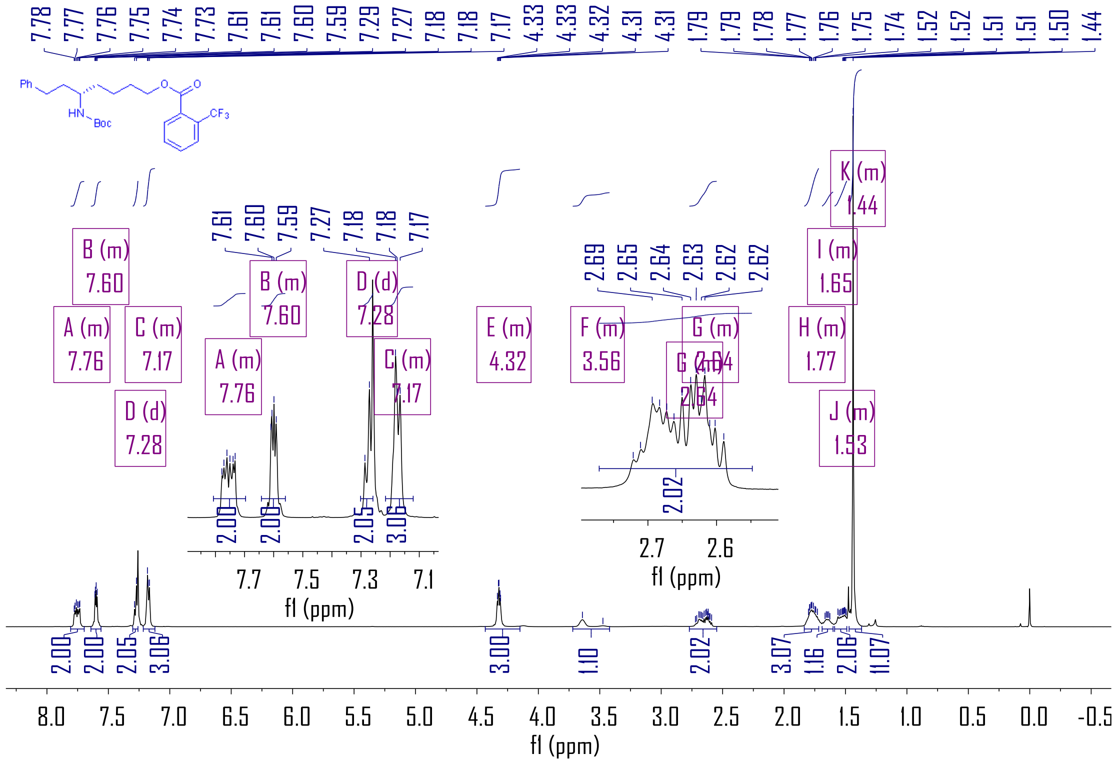

Supplementary Figure 234. ^{1}H NMR spectra for **58**

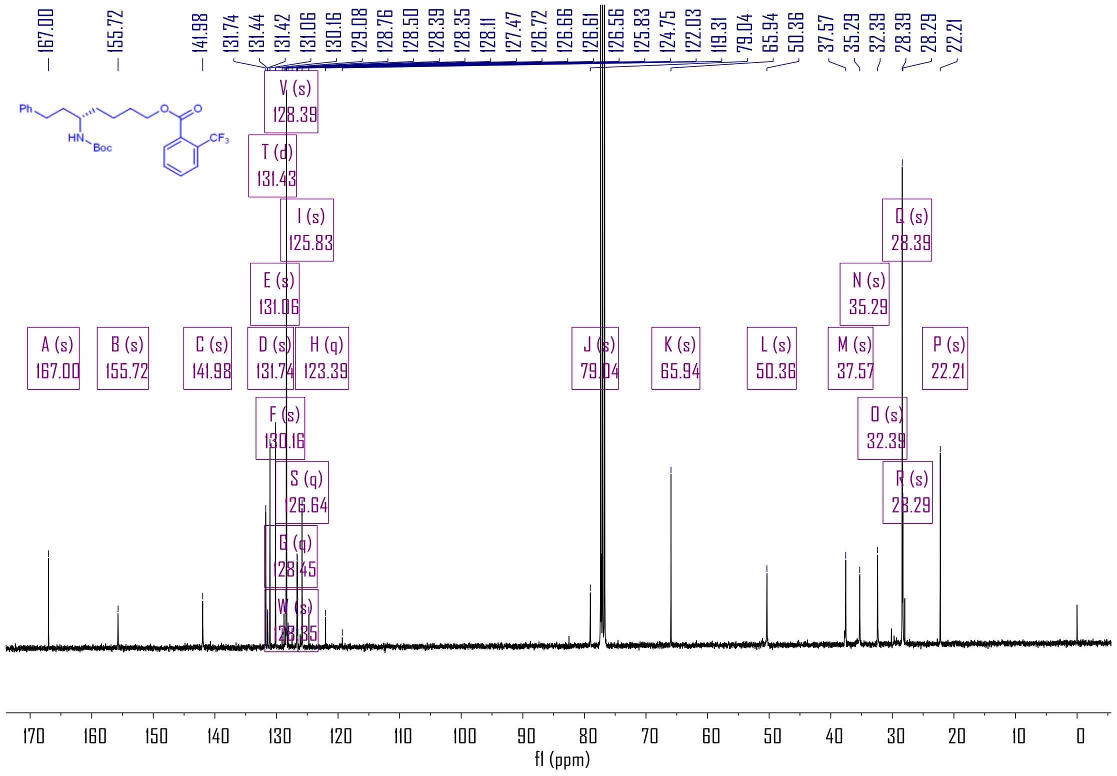

Supplementary Figure 235. ^{13}C NMR spectra for **58**

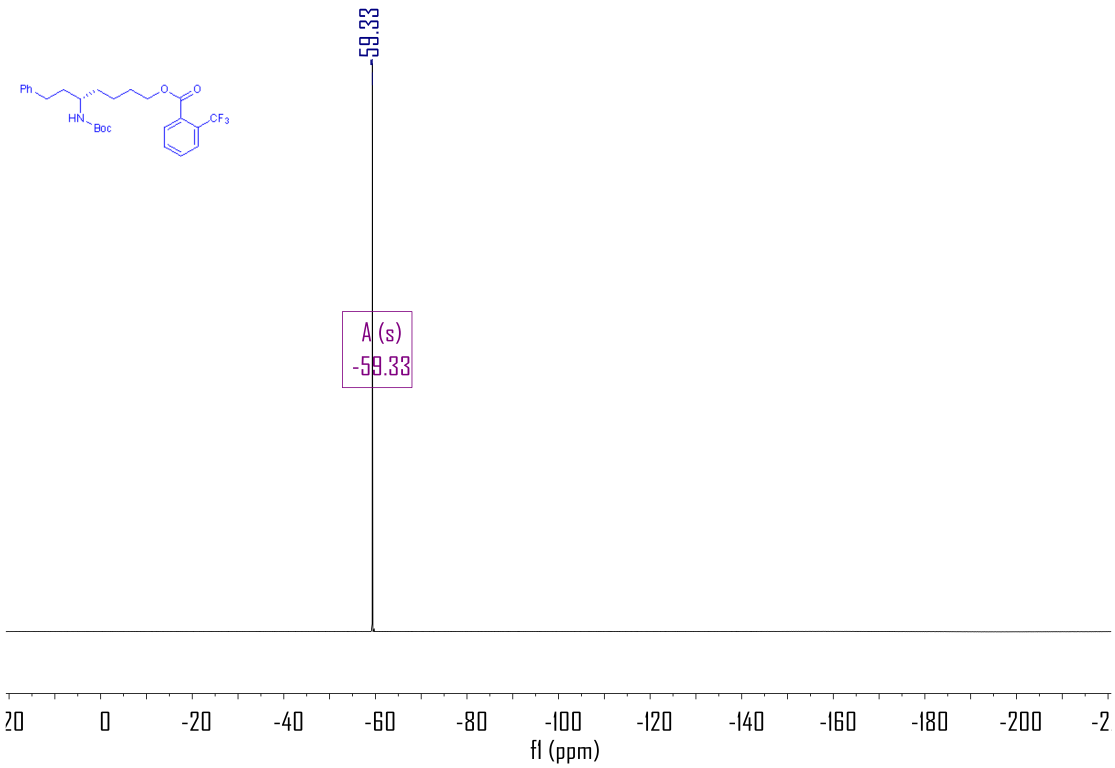

Supplementary Figure 236. ^{19}F NMR spectra for **58**

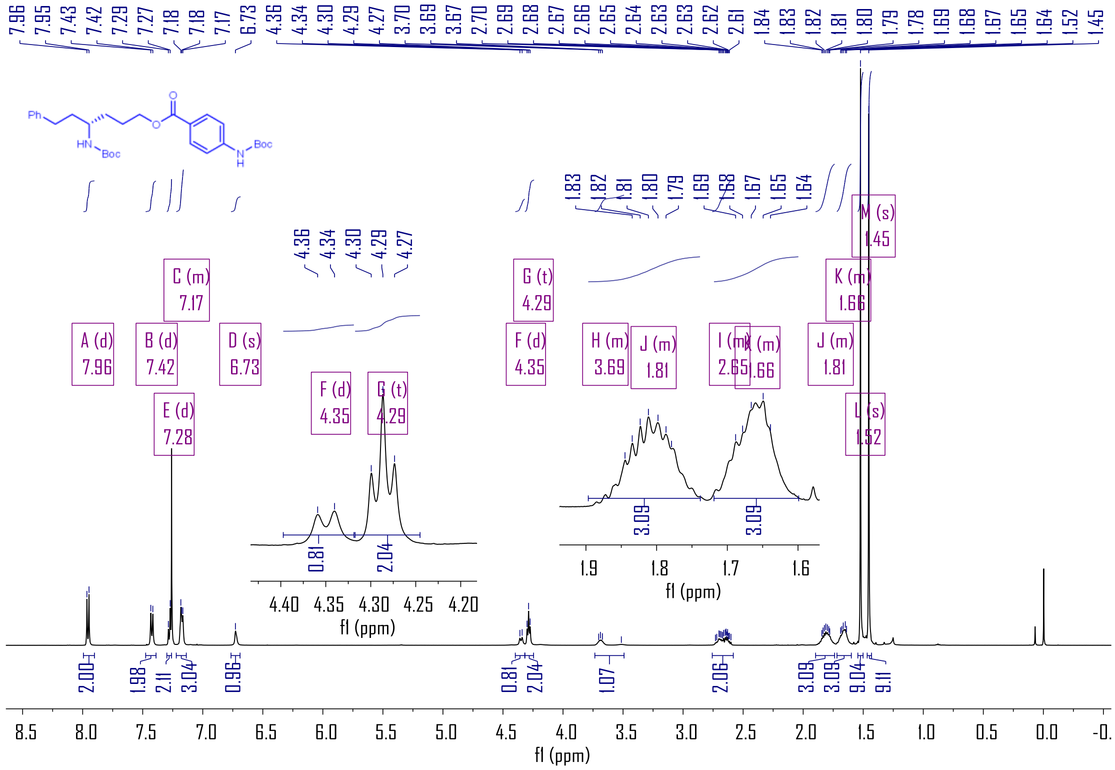

Supplementary Figure 237. ^{1}H NMR spectra for **59**

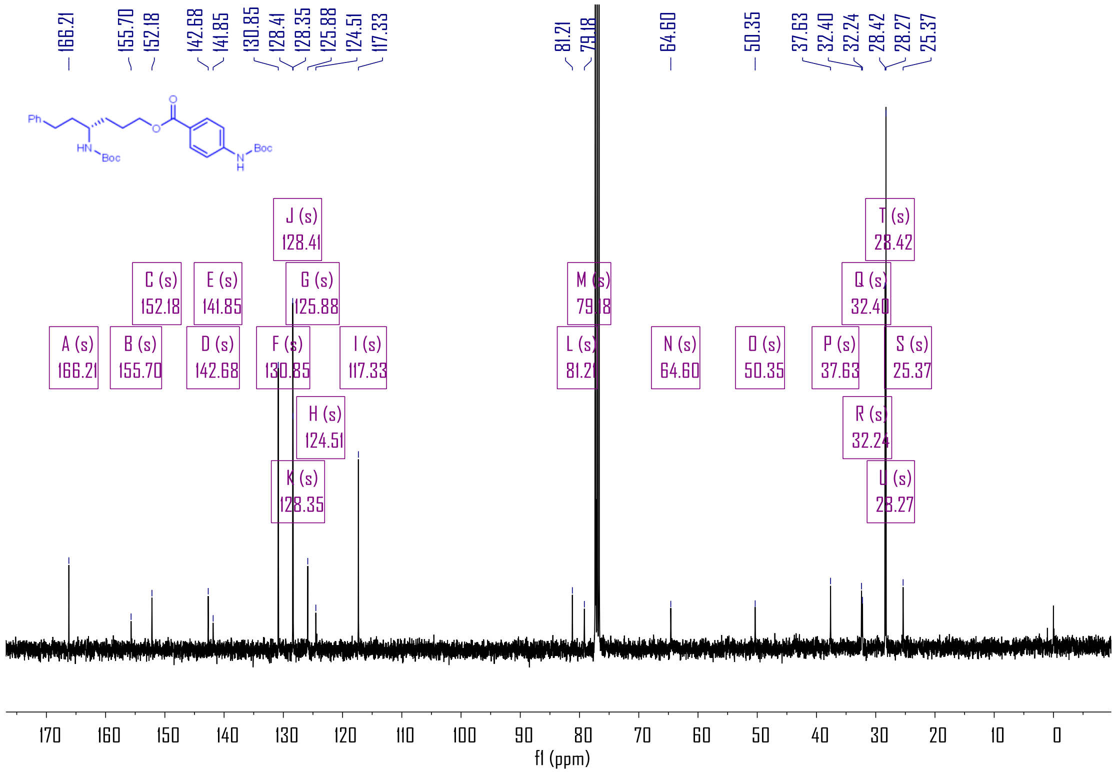

Supplementary Figure 238. ^{13}C NMR spectra for **59**

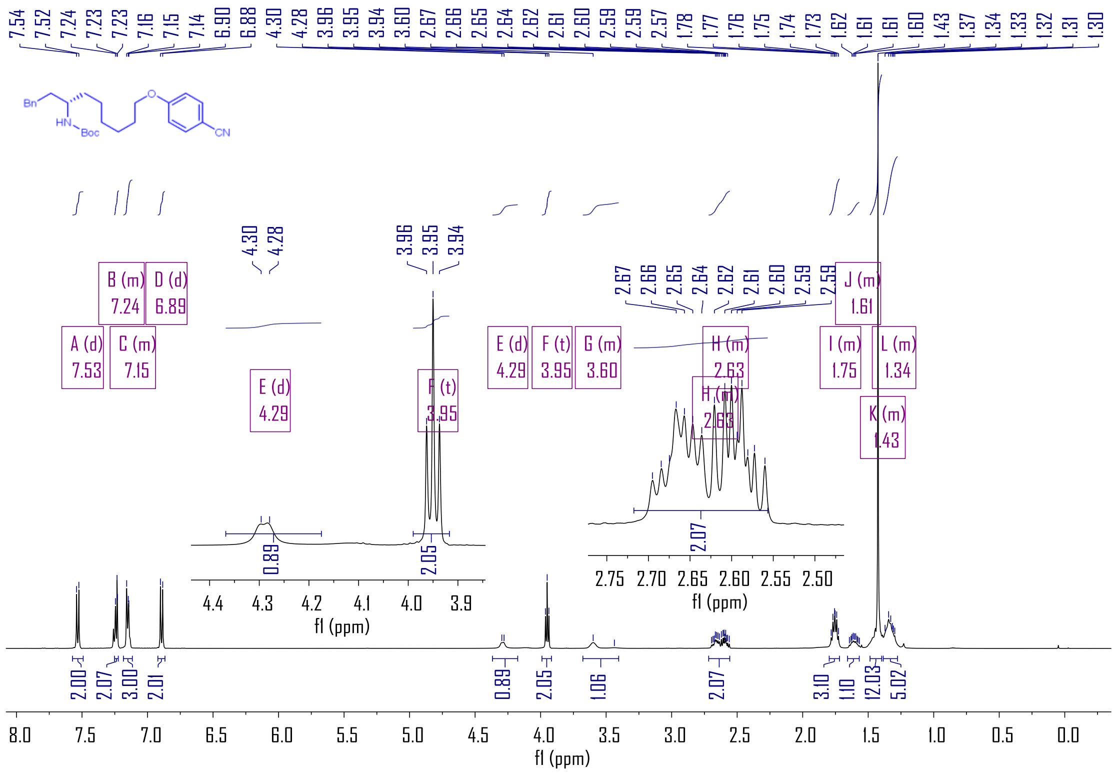

Supplementary Figure 239. ^{1}H NMR spectra for **60**

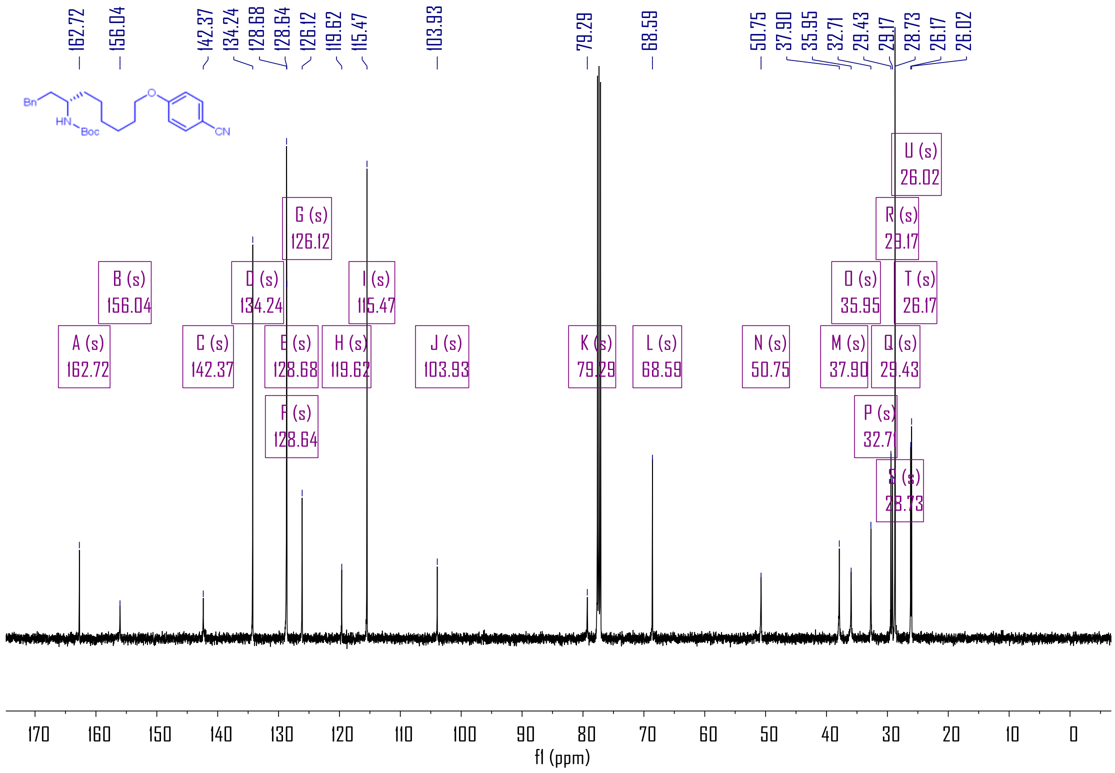

Supplementary Figure 240. ^{13}C NMR spectra for **60**

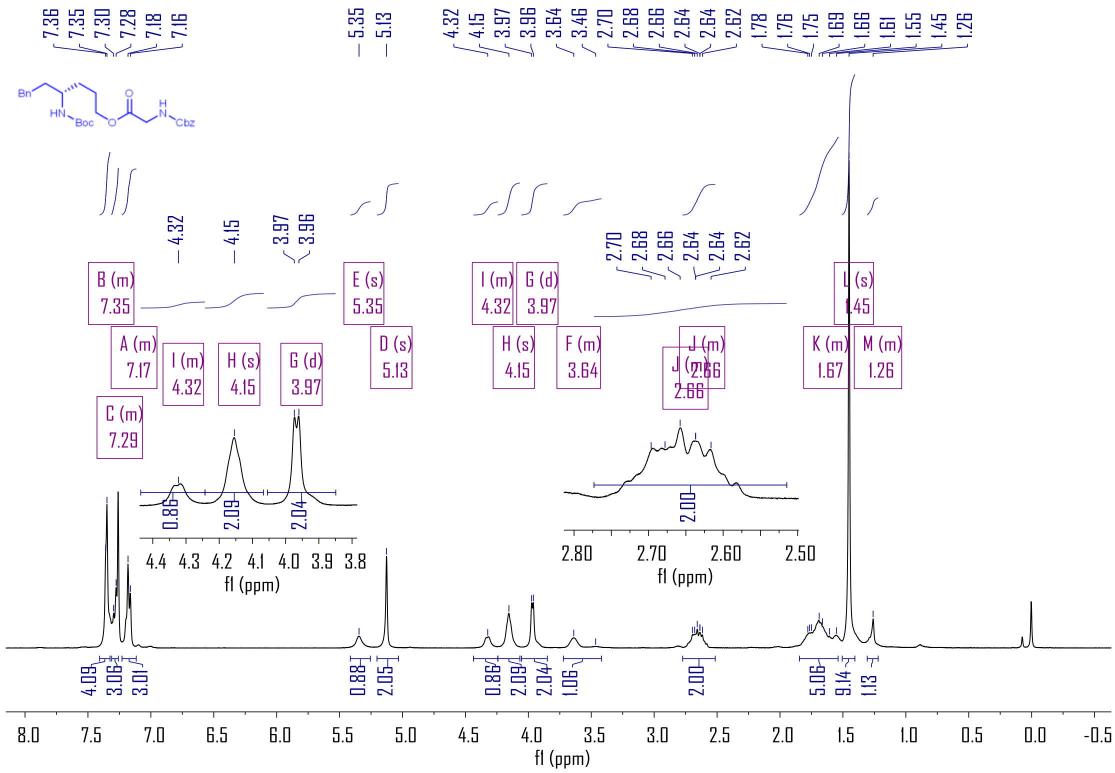

Supplementary Figure 241. ^{1}H NMR spectra for **61**

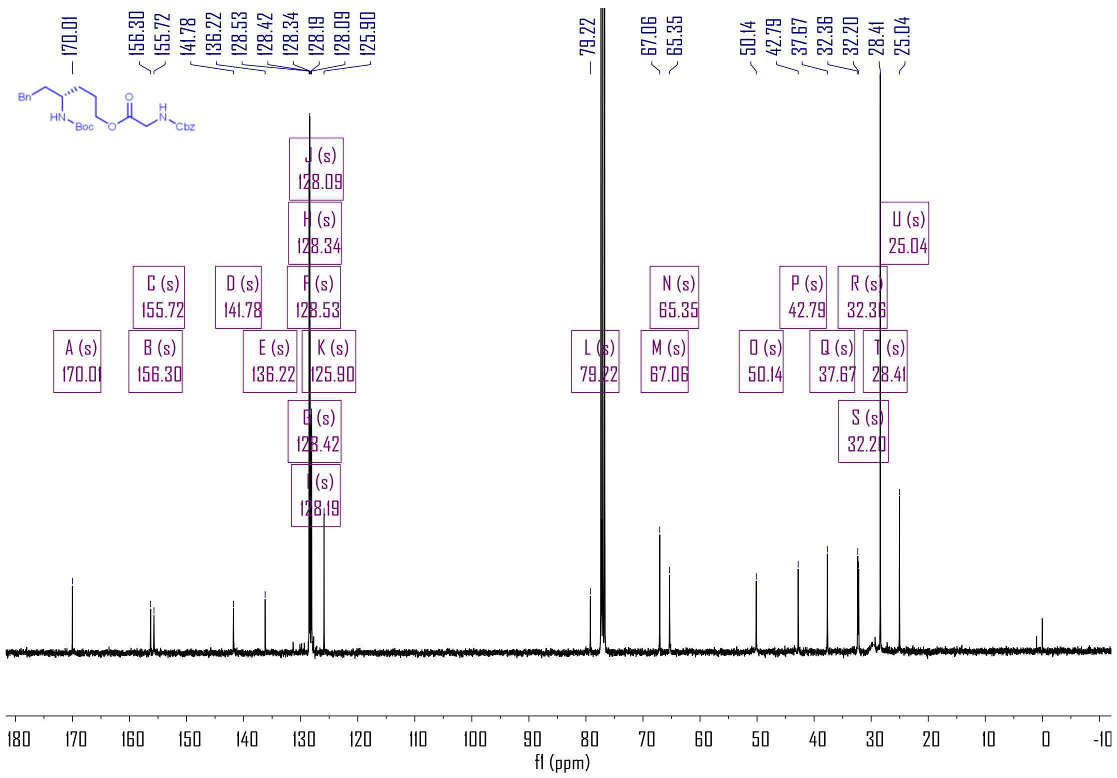

Supplementary Figure 242. ^{13}C NMR spectra for **61**

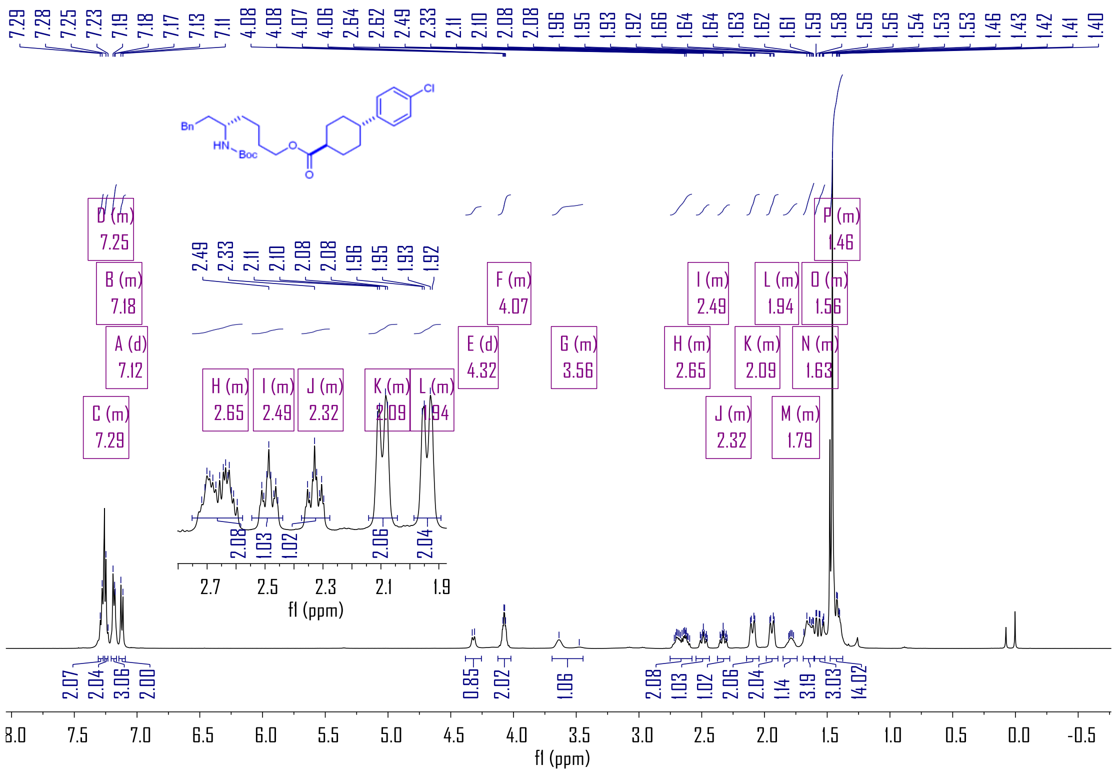

Supplementary Figure 243. ^{1}H NMR spectra for **62**

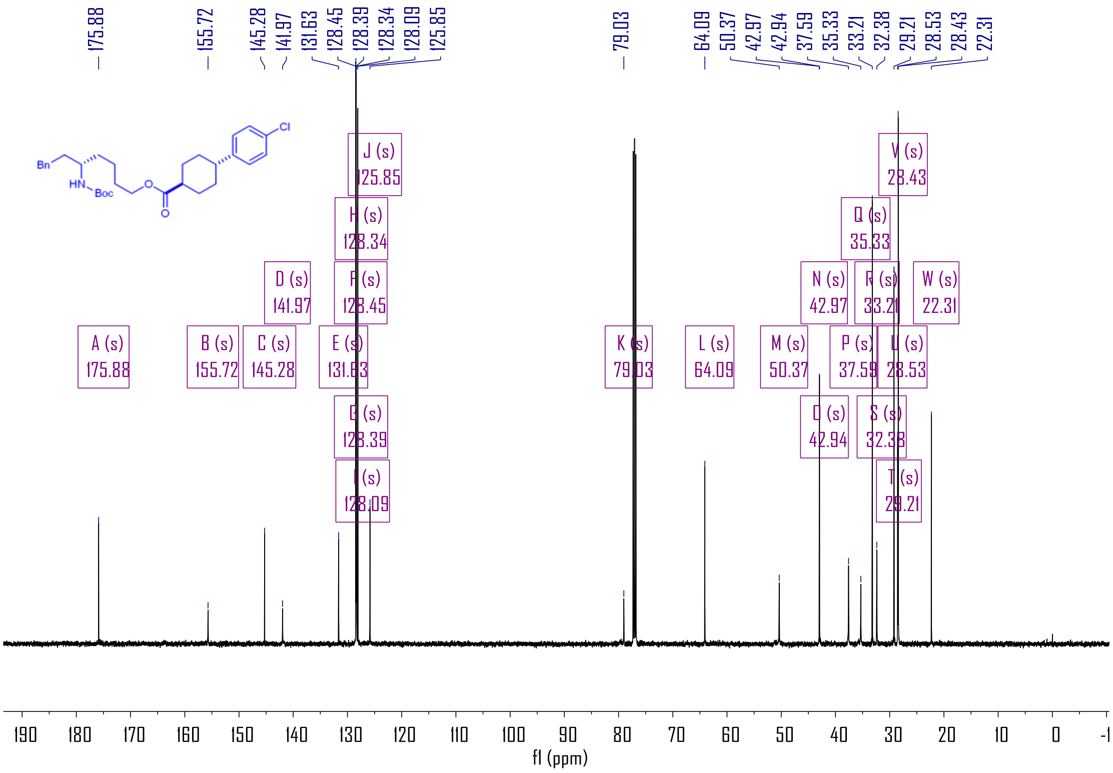

Supplementary Figure 244. ^{13}C NMR spectra for **62**

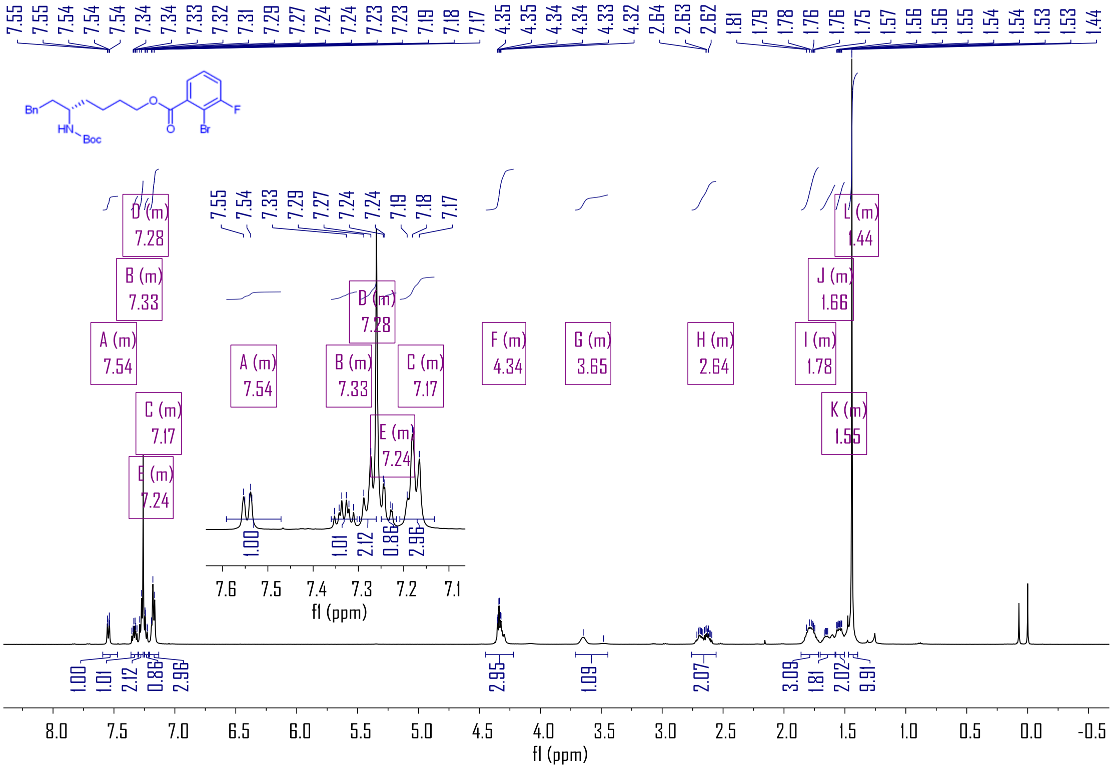

Supplementary Figure 245. ^{1}H NMR spectra for **63**

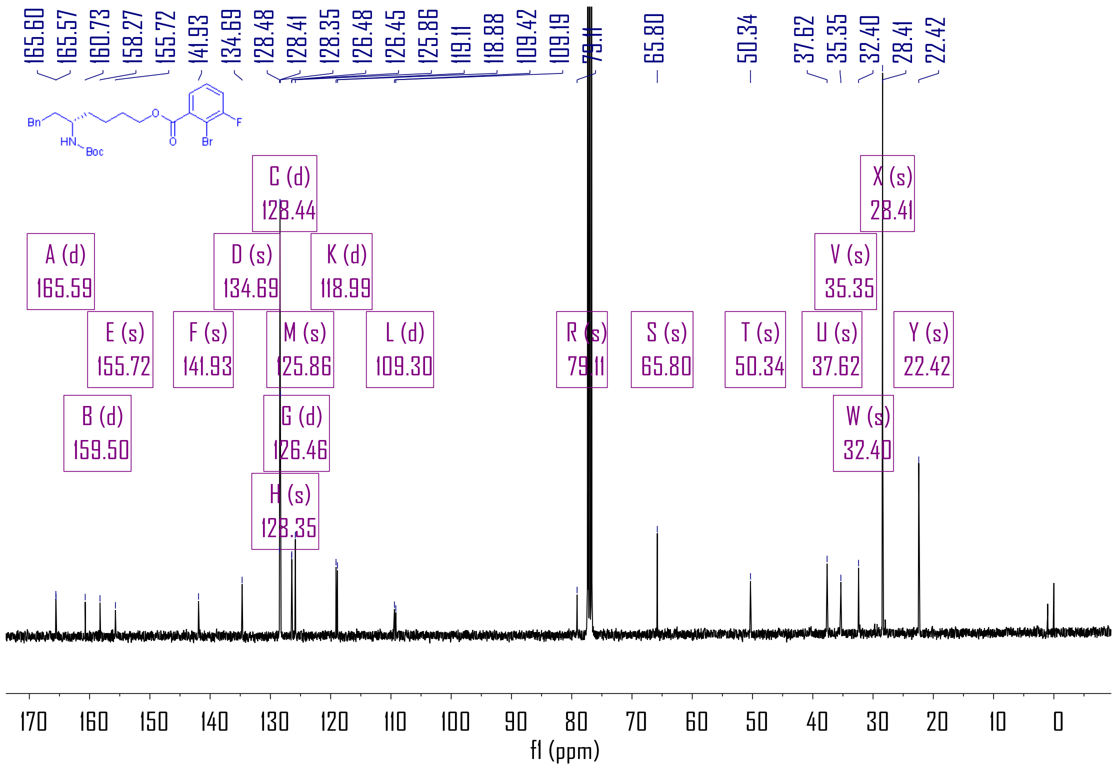

Supplementary Figure 246. ^{13}C NMR spectra for **63**

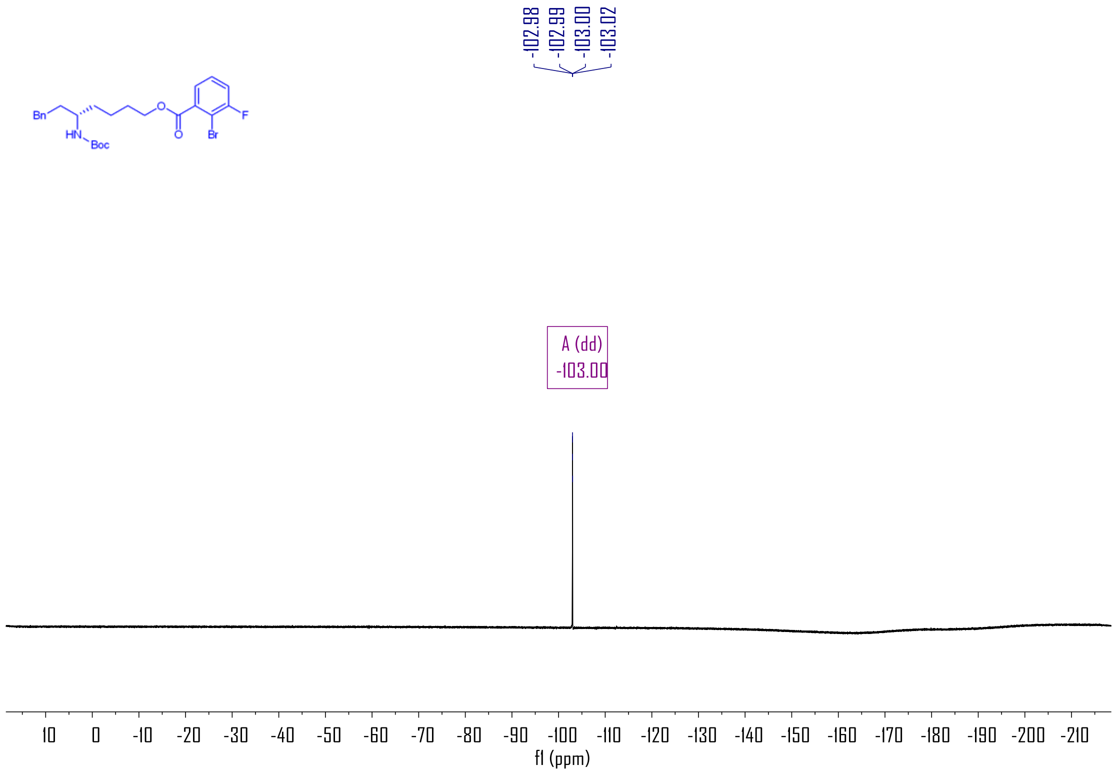

Supplementary Figure 247. ^{19}F NMR spectra for **63**

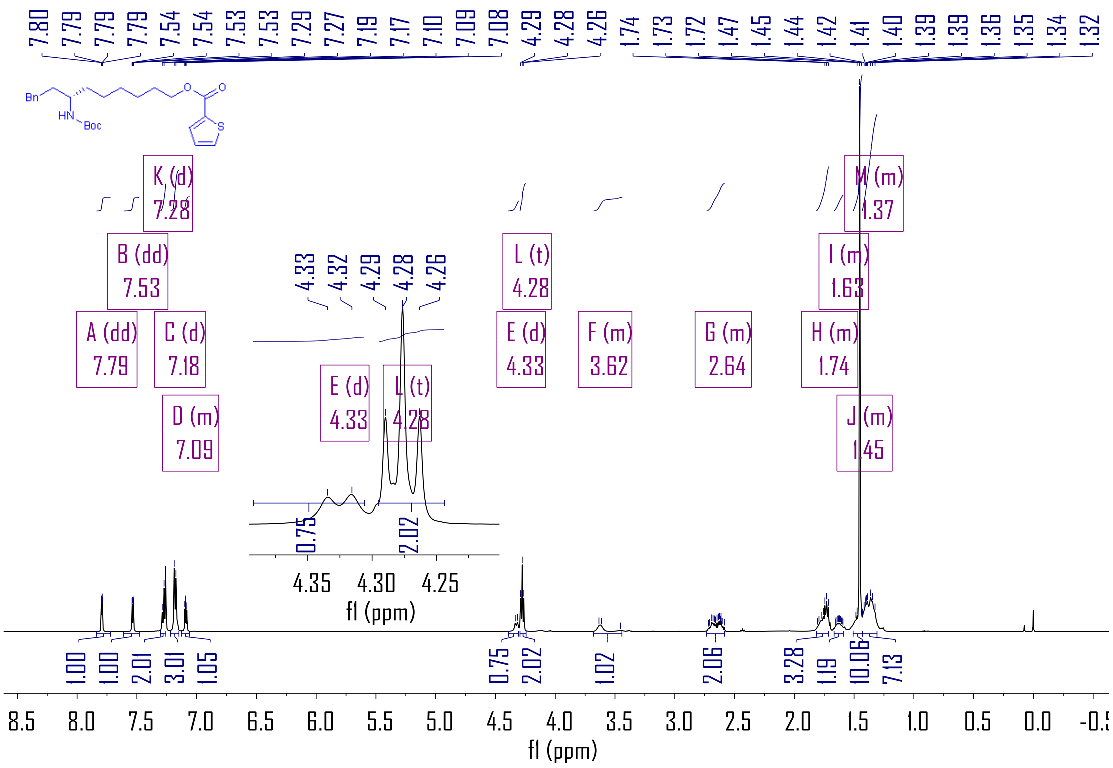

Supplementary Figure 248. ^{1}H NMR spectra for **64**

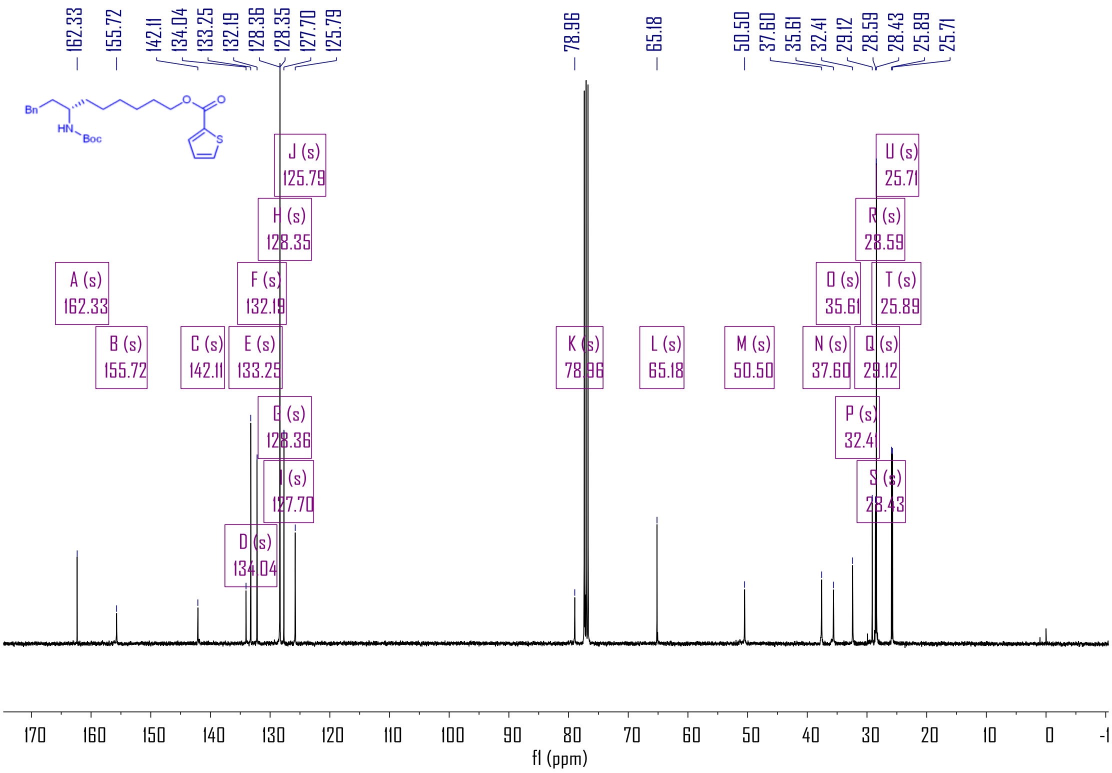

Supplementary Figure 249. ^{13}C NMR spectra for **64**

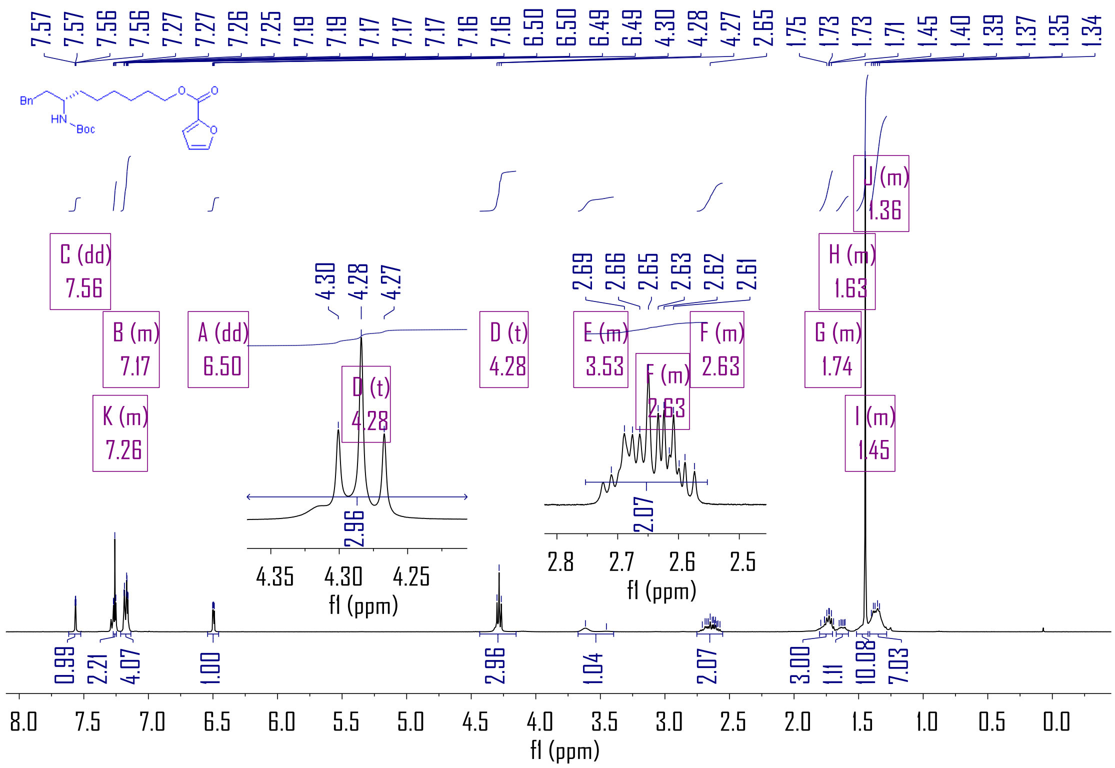

Supplementary Figure 250. ^{1}H NMR spectra for **65**

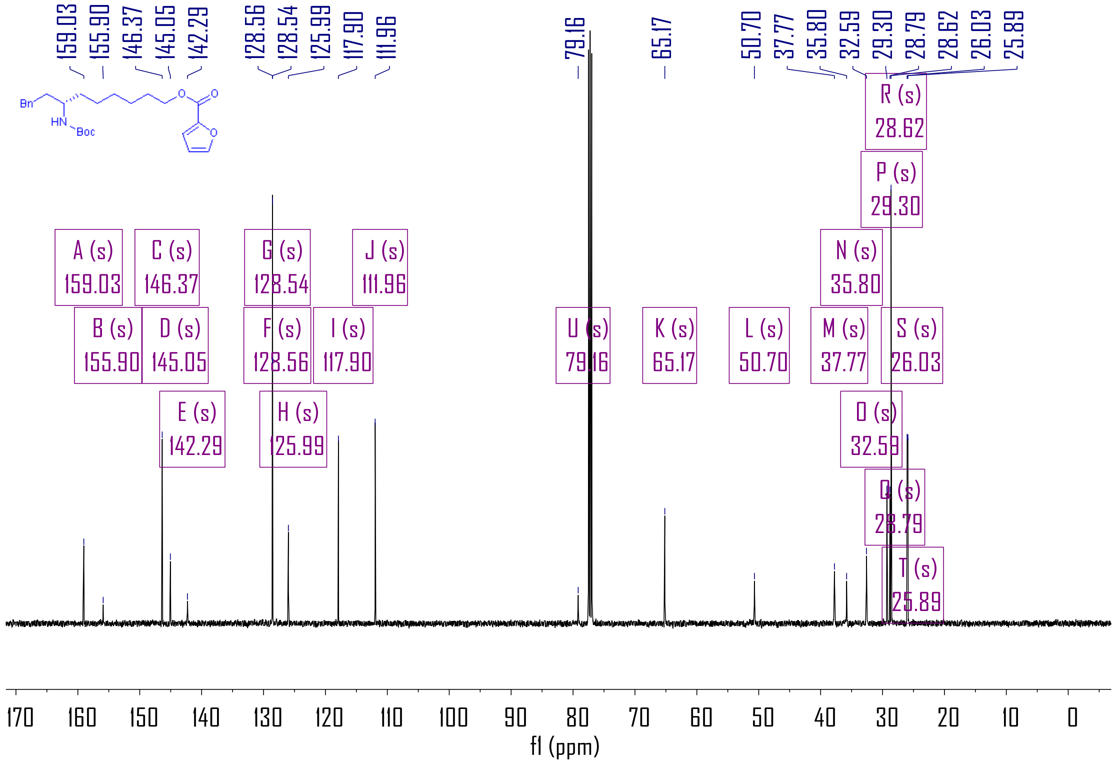

Supplementary Figure 251. ^{13}C NMR spectra for **65**

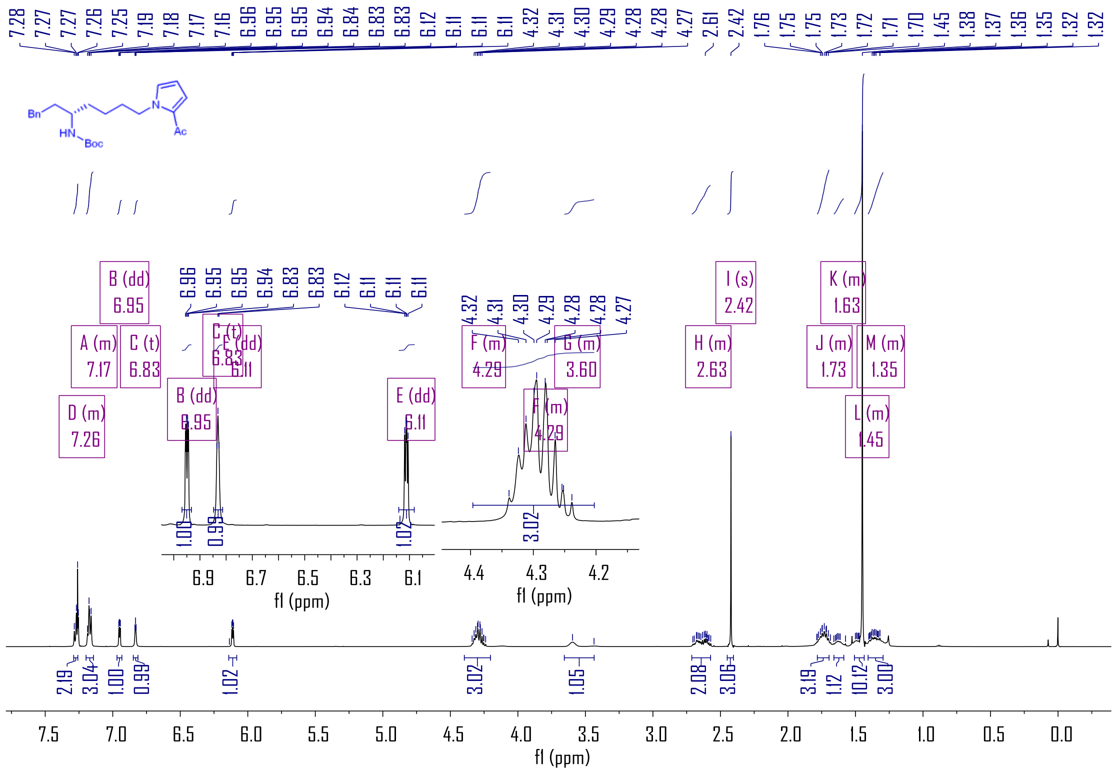

Supplementary Figure 252. ^{1}H NMR spectra for **66**

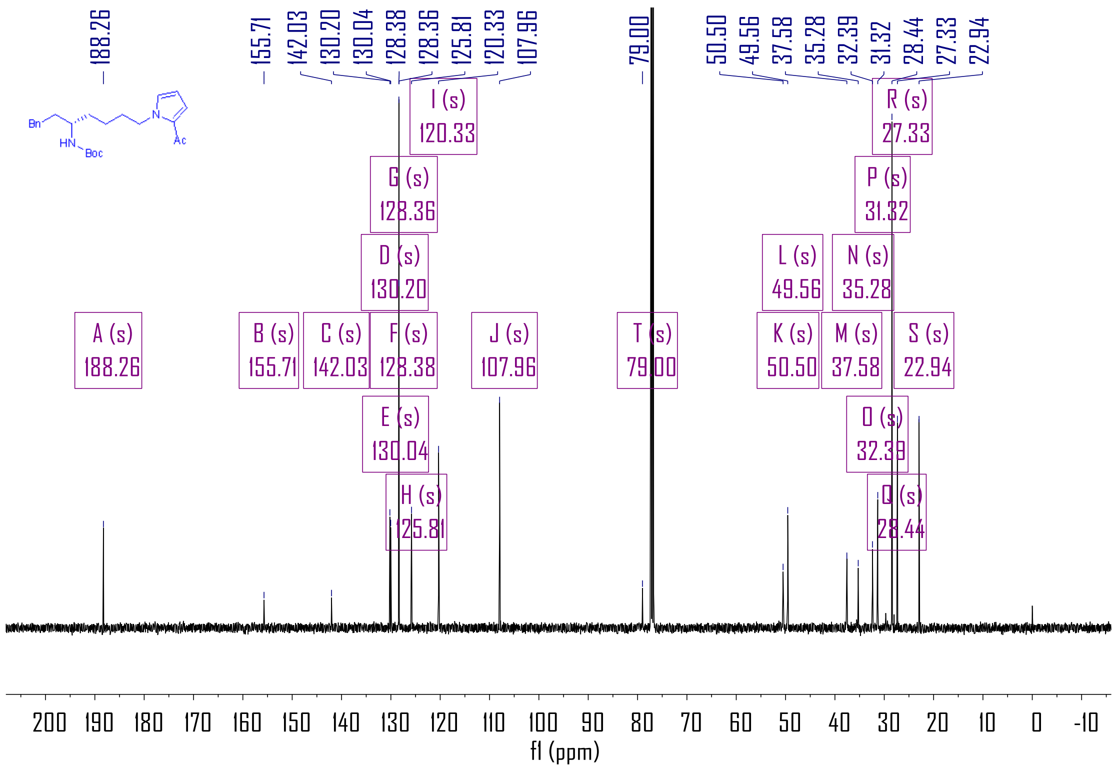

Supplementary Figure 253. ^{13}C NMR spectra for **66**

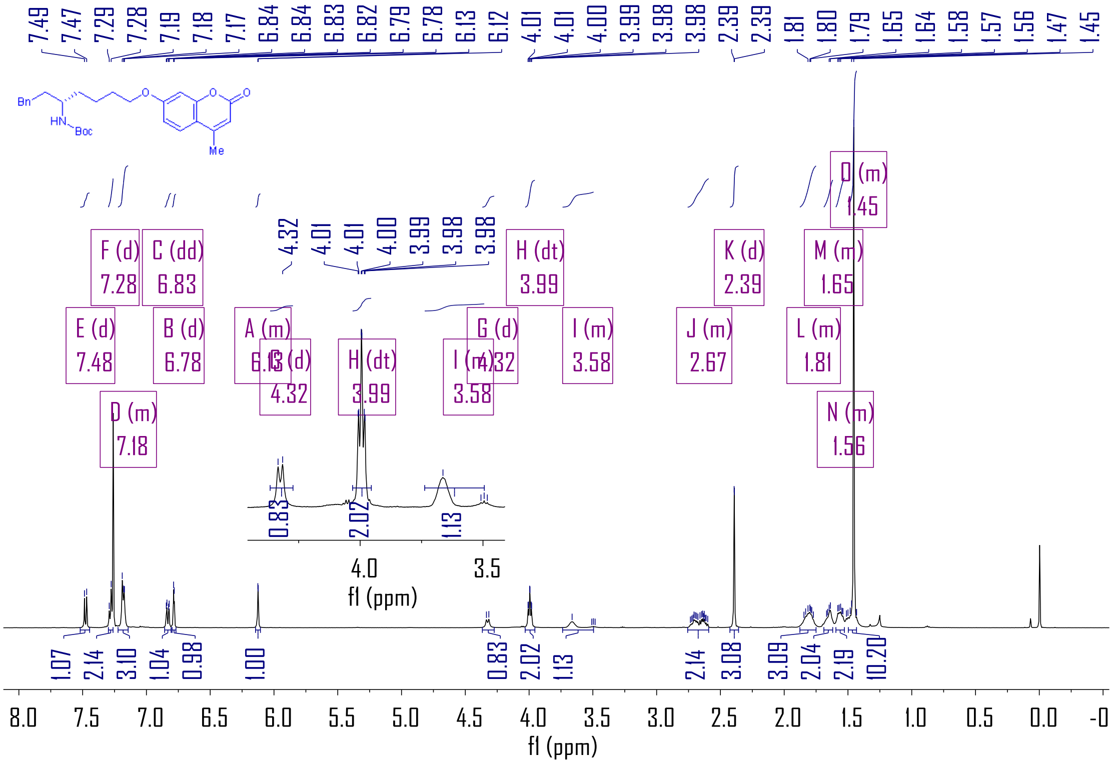

Supplementary Figure 254. ^{1}H NMR spectra for **67**

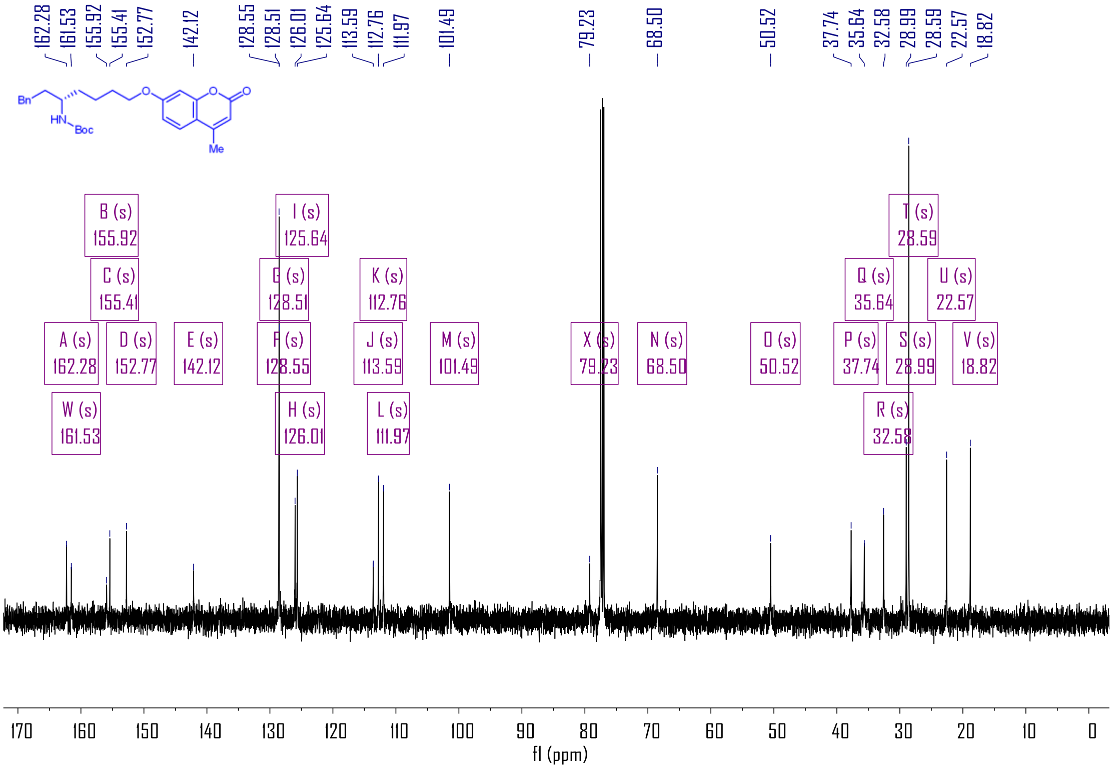

Supplementary Figure 255. ^{13}C NMR spectra for **67**

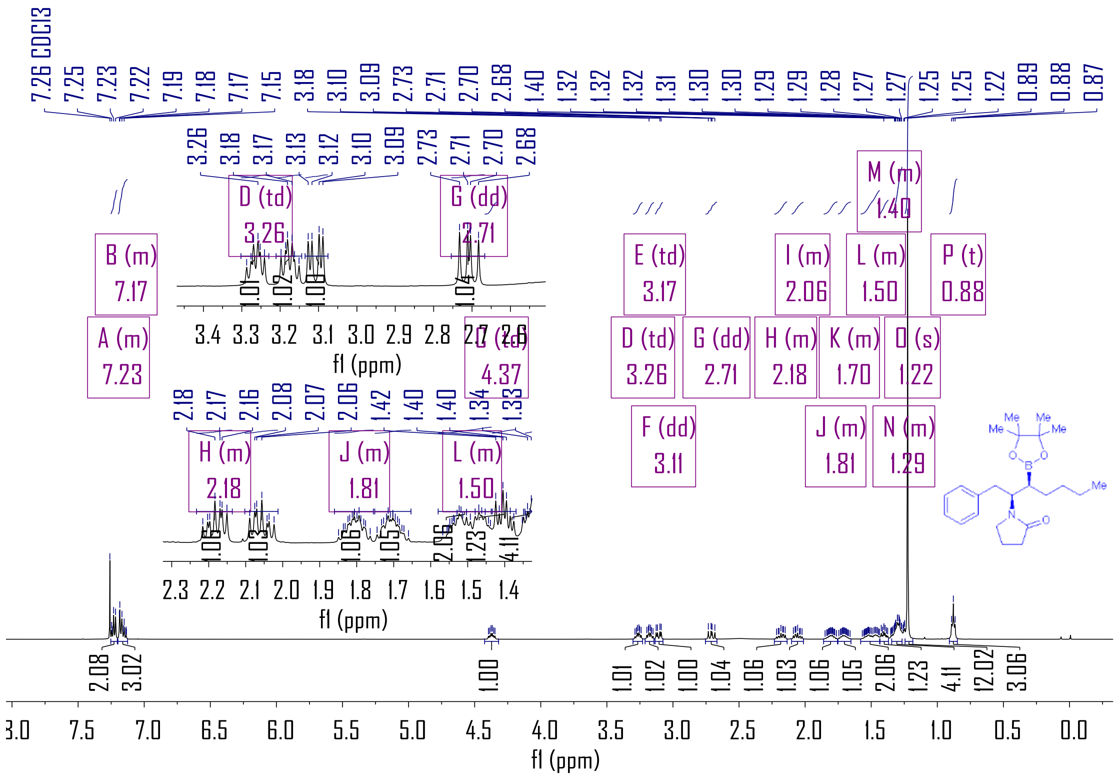

Supplementary Figure 256. ^{1}H NMR spectra for **83**

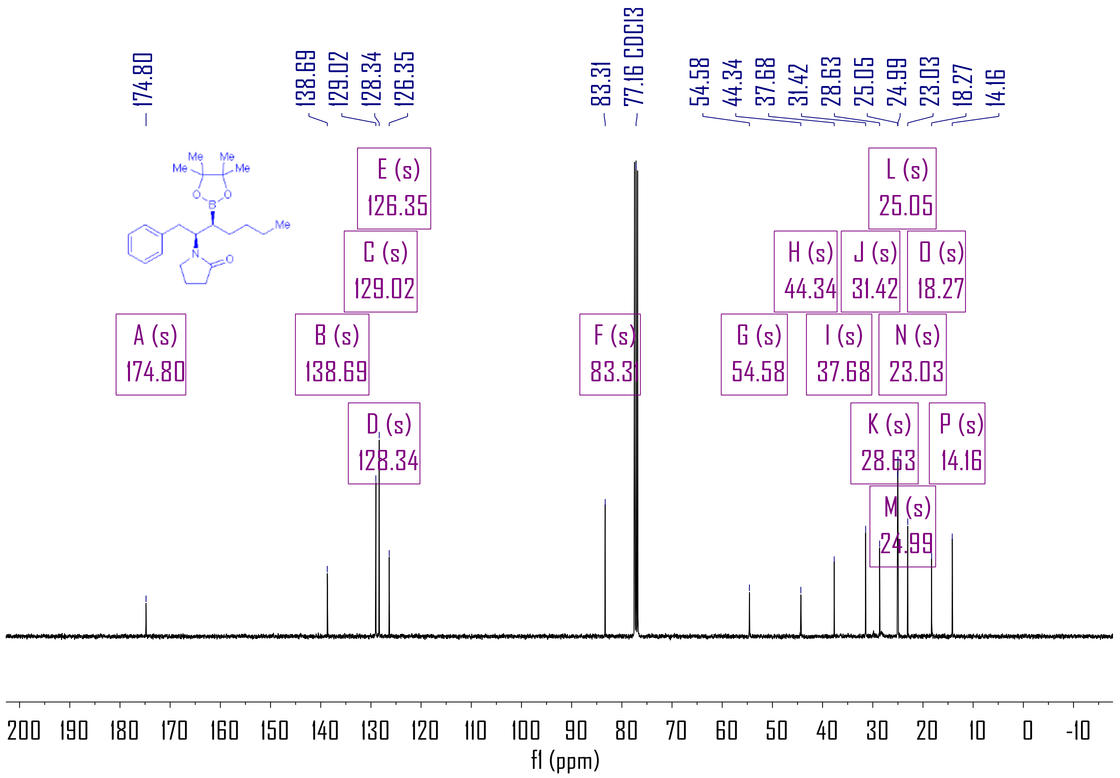

Supplementary Figure 257. ^{13}C NMR spectra for **83**

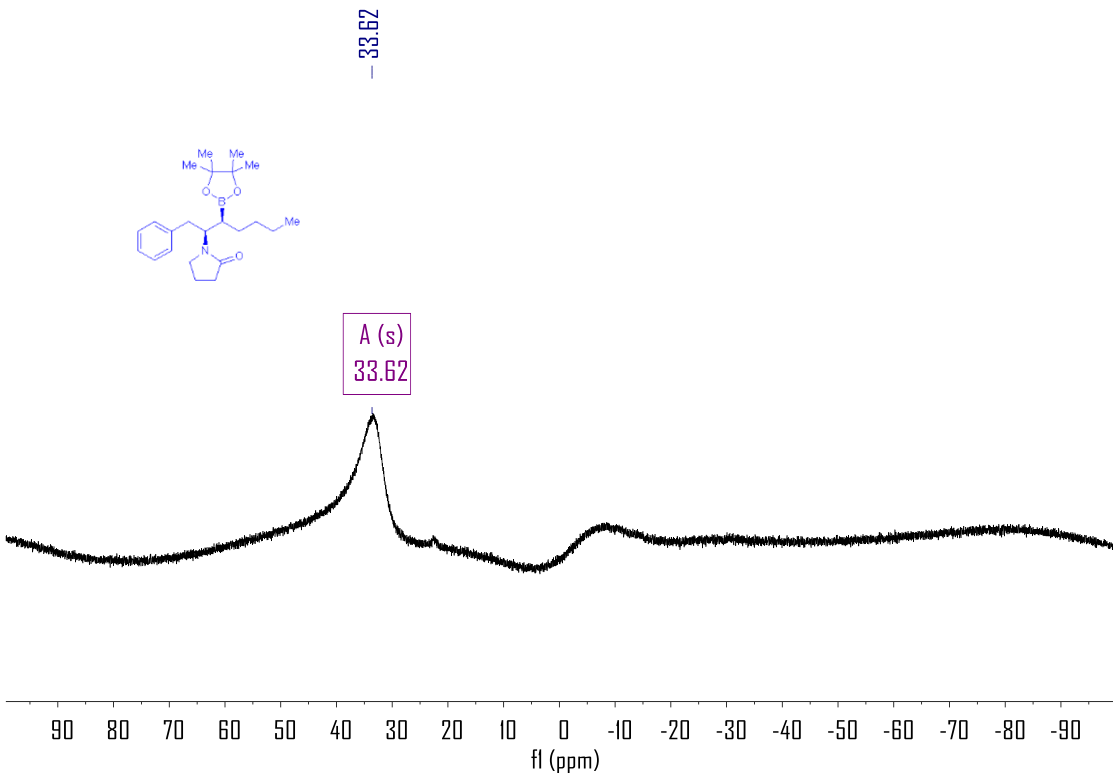

Supplementary Figure 258. ^{11}B NMR spectra for **83**

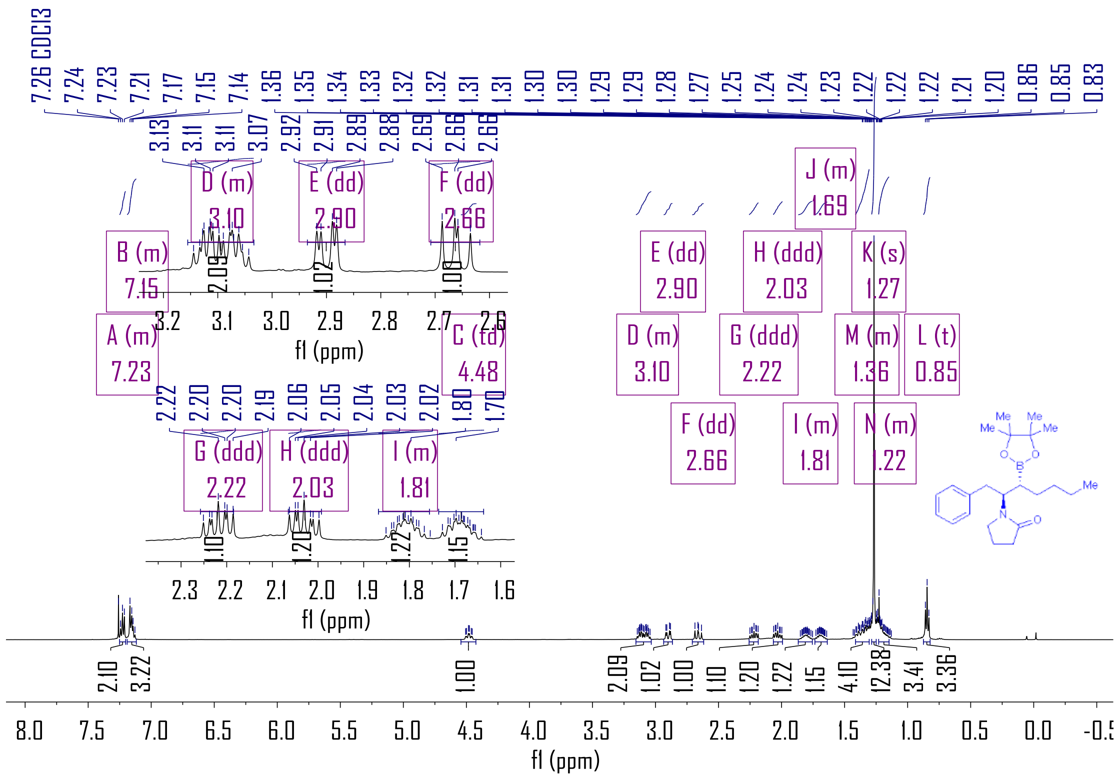

Supplementary Figure 259. ^{1}H NMR spectra for **84**

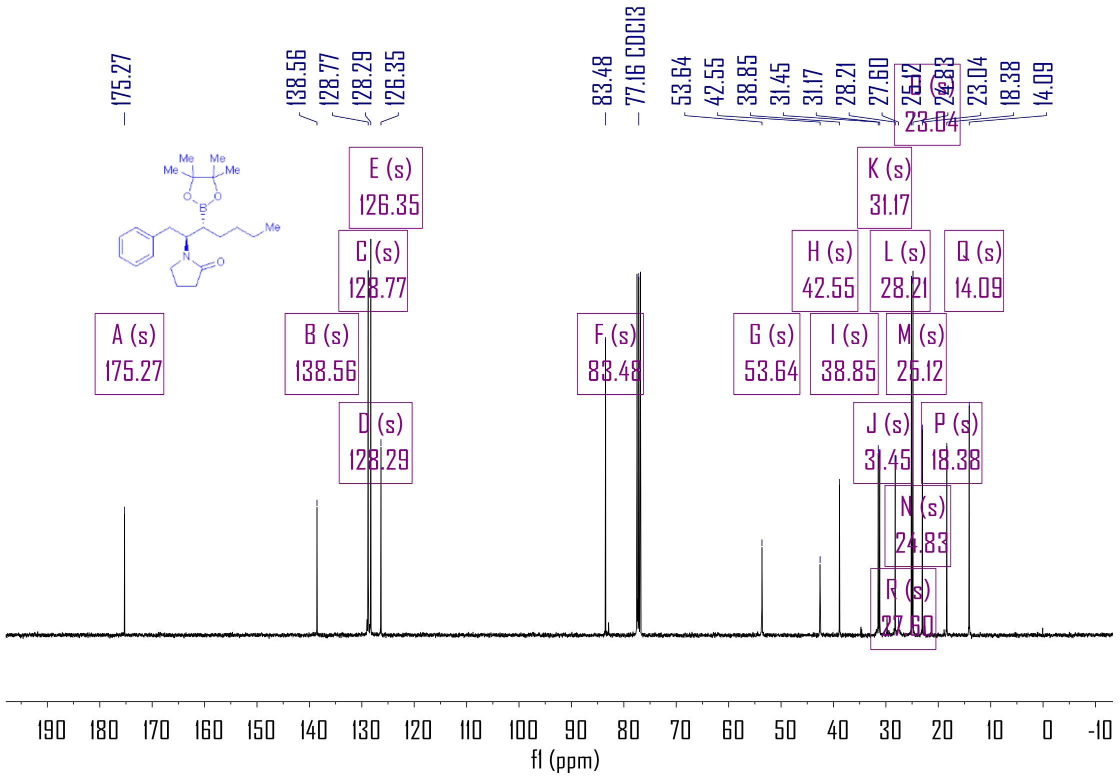

Supplementary Figure 260. ^{13}C NMR spectra for **84**

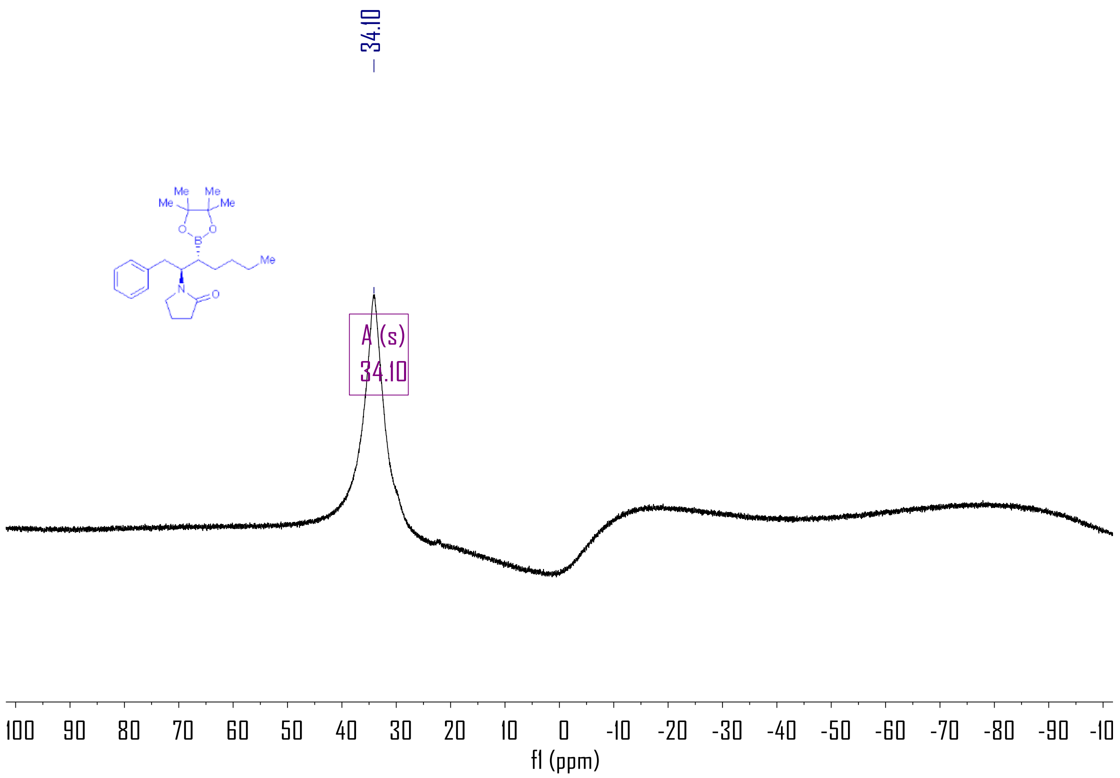

Supplementary Figure 261. ^{11}B NMR spectra for **84**

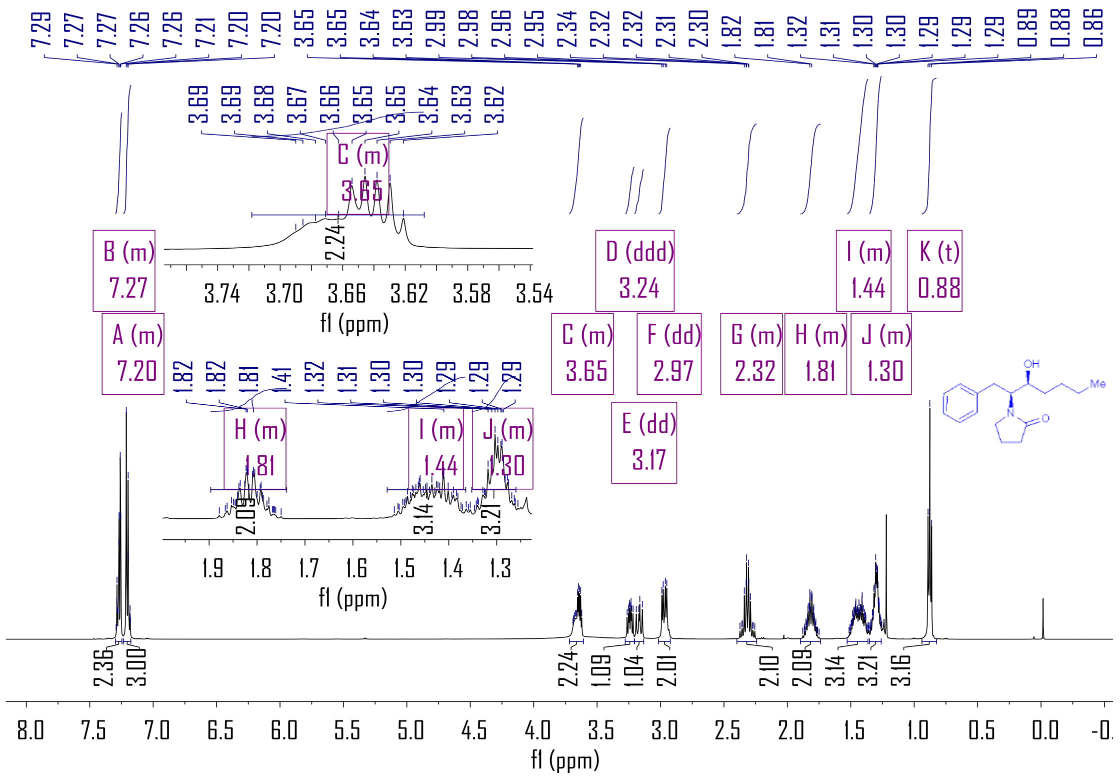

Supplementary Figure 262. ^{1}H NMR spectra for **83-OH**

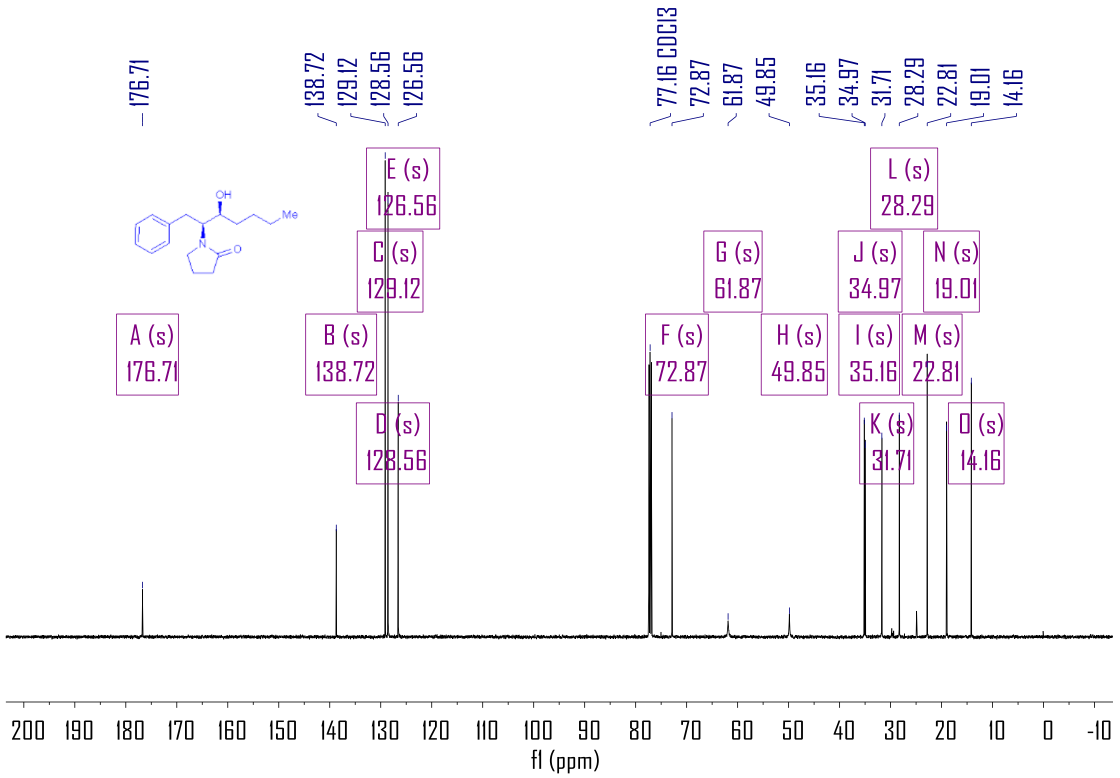

Supplementary Figure 263. ^{13}C NMR spectra for **83-OH**

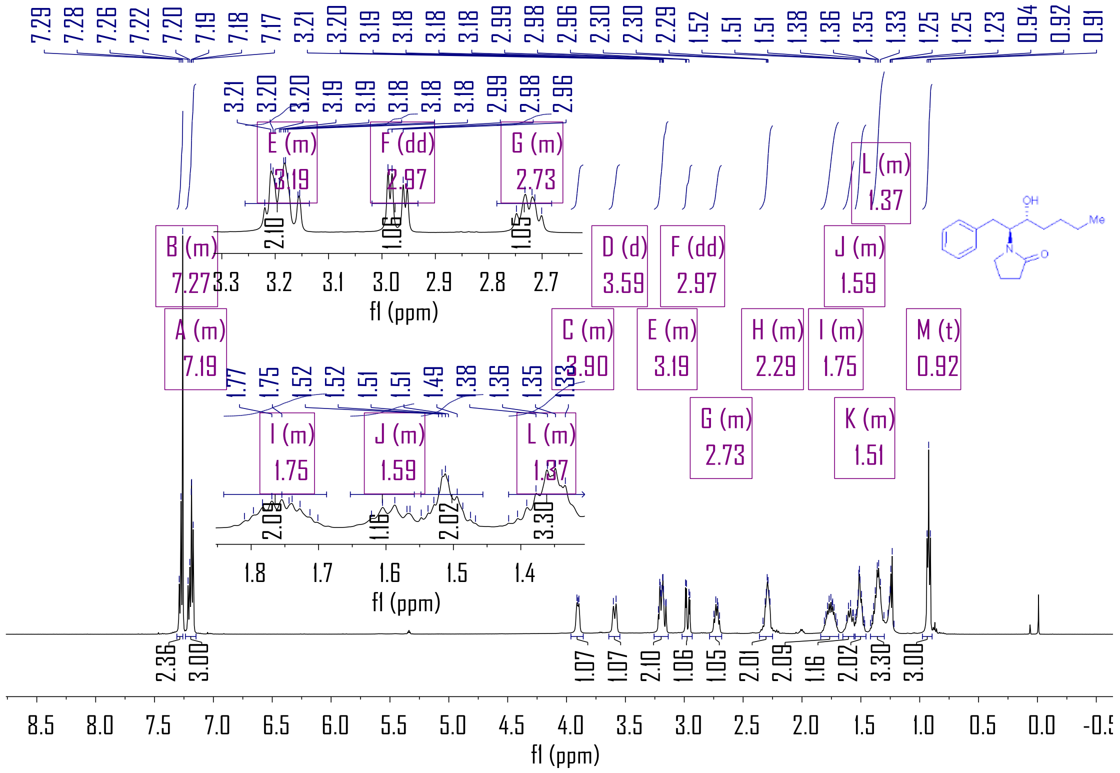

Supplementary Figure 264. ^{1}H NMR spectra for **84-OH**

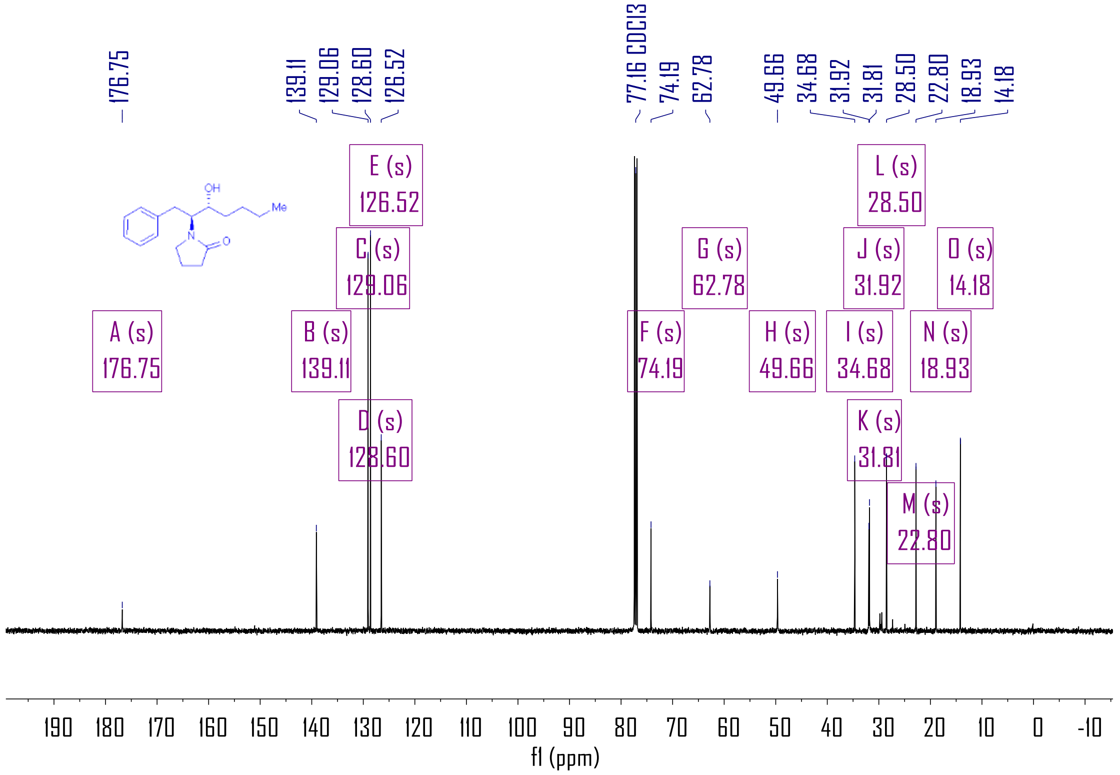

Supplementary Figure 265. ^{13}C NMR spectra for **84-OH**

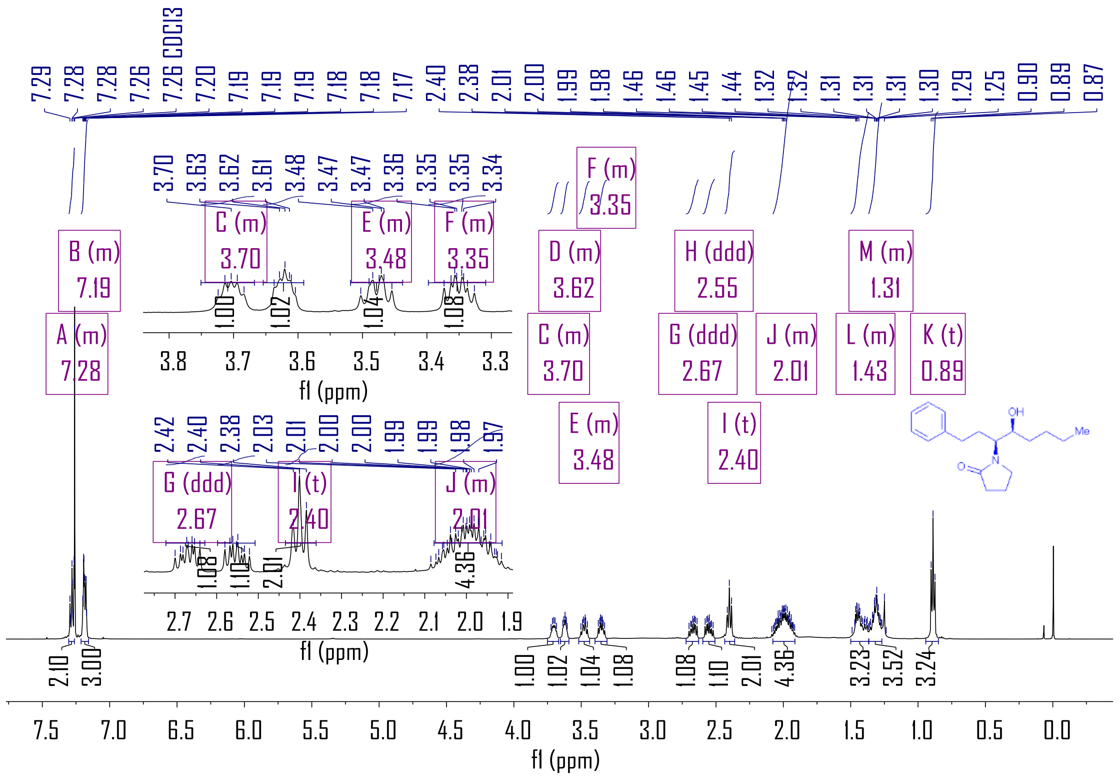

Supplementary Figure 266. ^{1}H NMR spectra for **85**

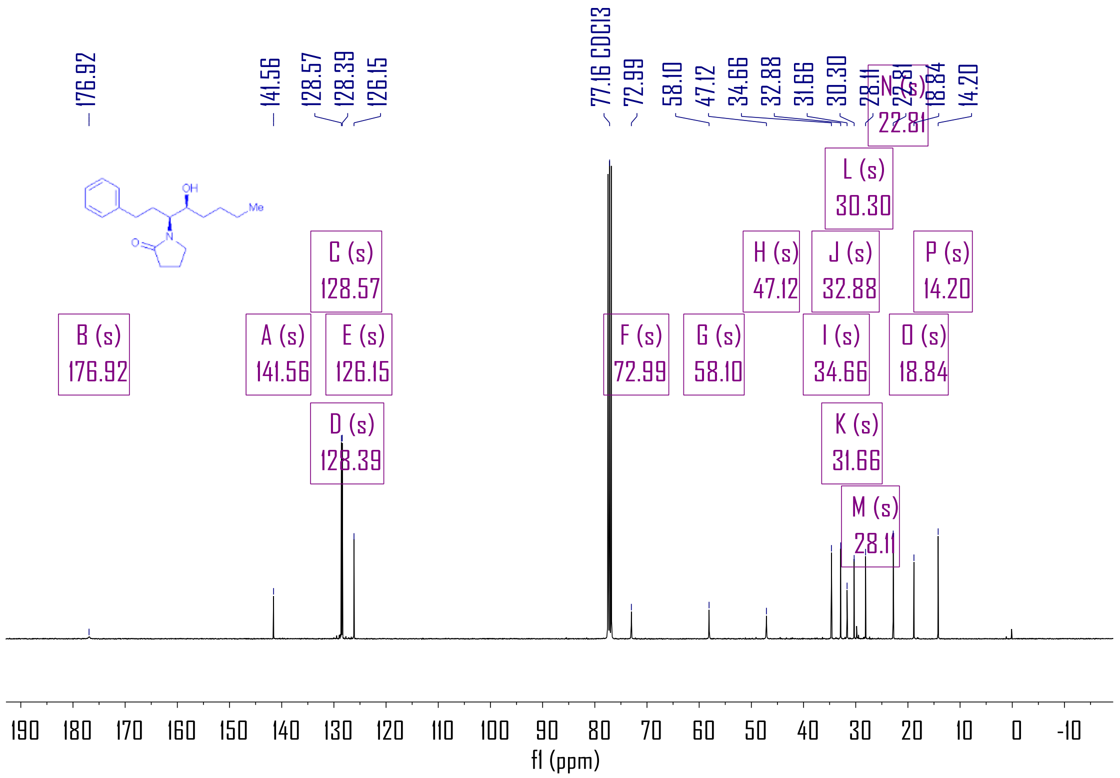

Supplementary Figure 267. ^{13}C NMR spectra for **85**

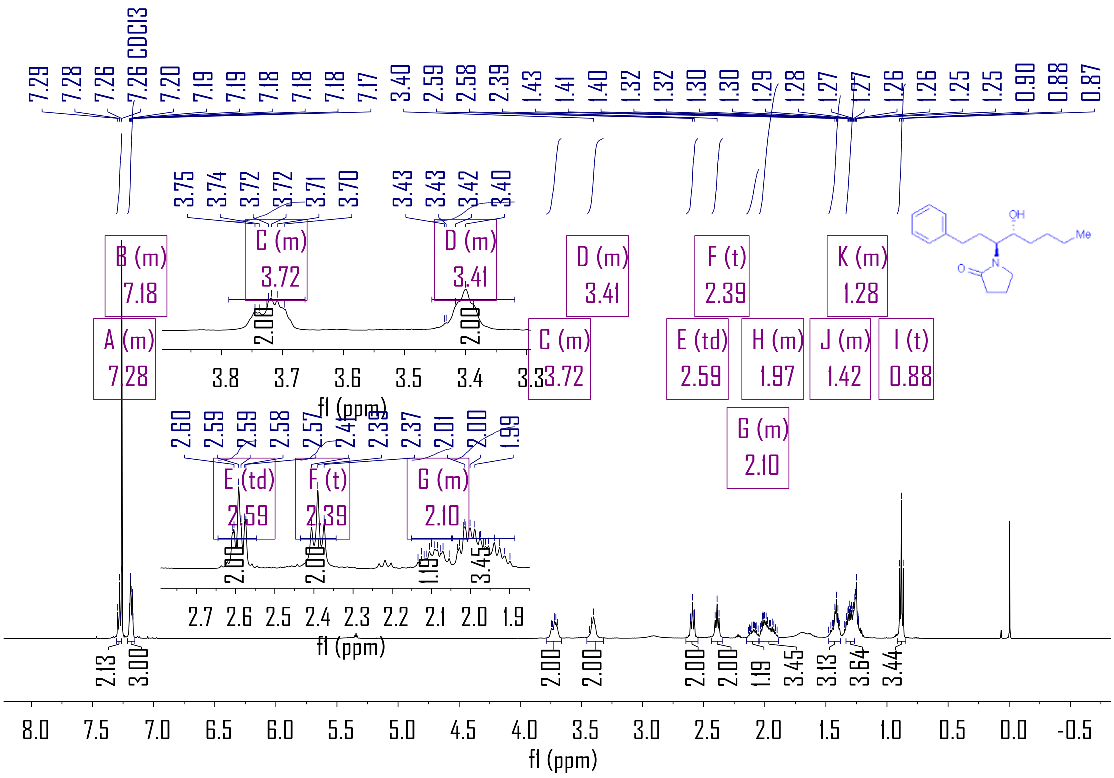

Supplementary Figure 268. ^{1}H NMR spectra for **86**

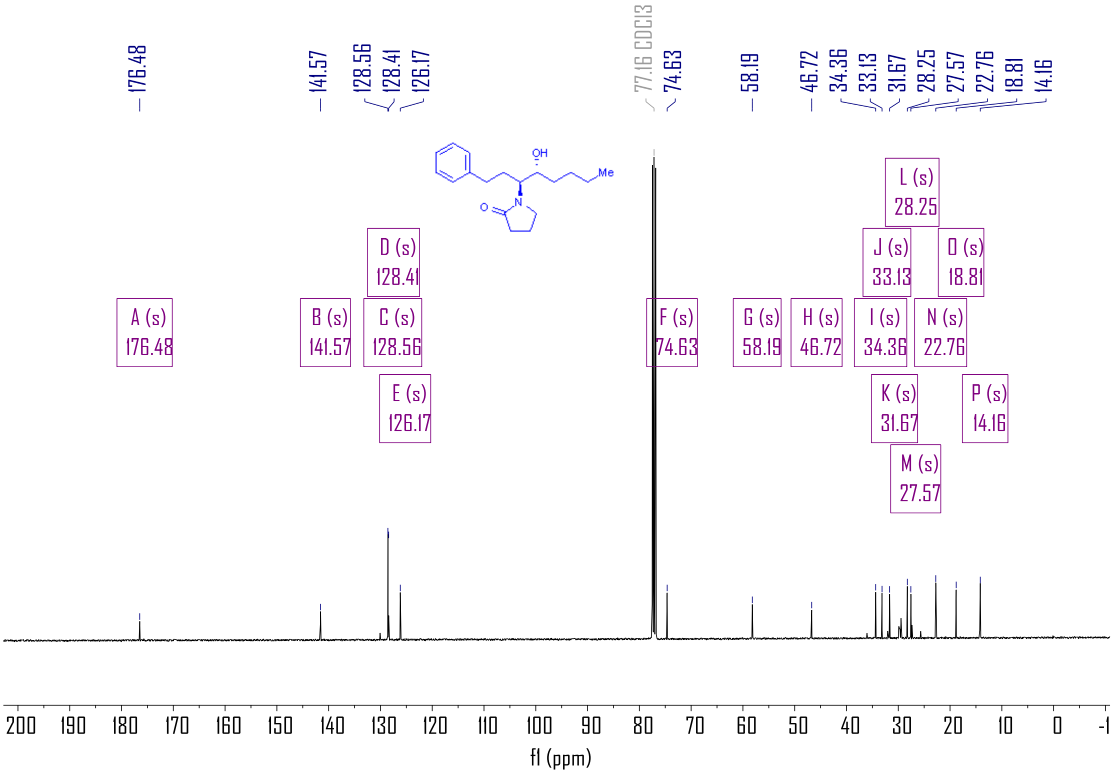

Supplementary Figure 269. ^{13}C NMR spectra for **86**

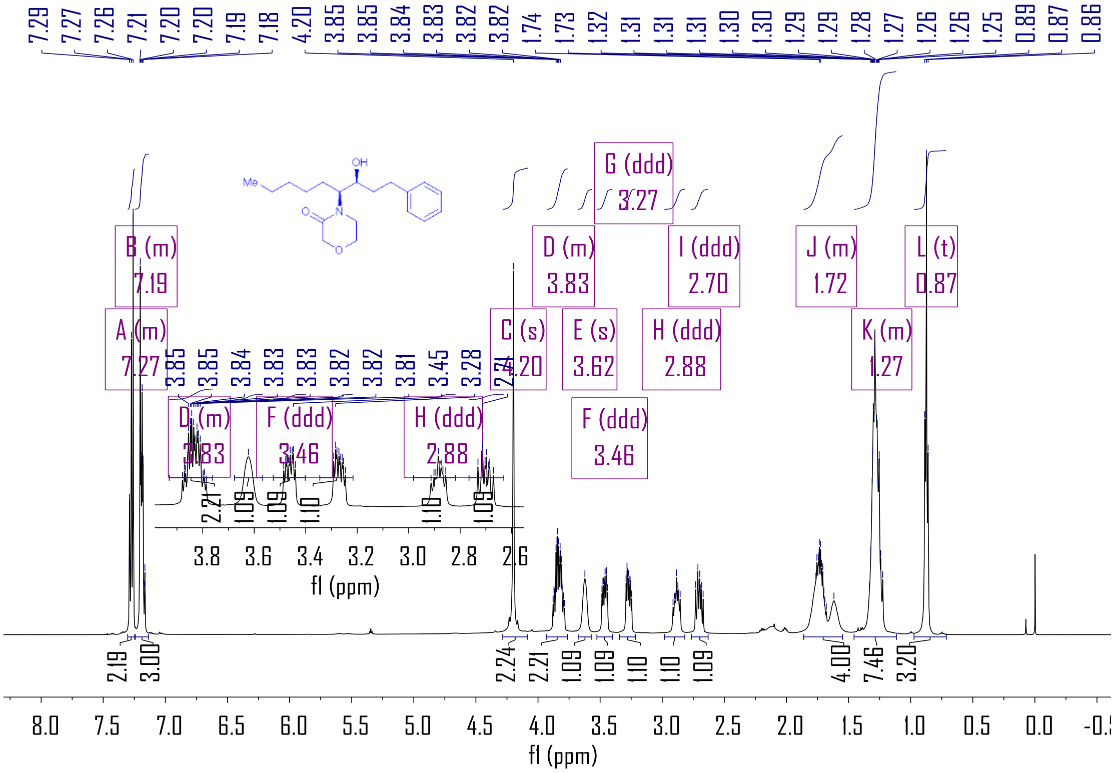

Supplementary Figure 270. ^{1}H NMR spectra for **87**

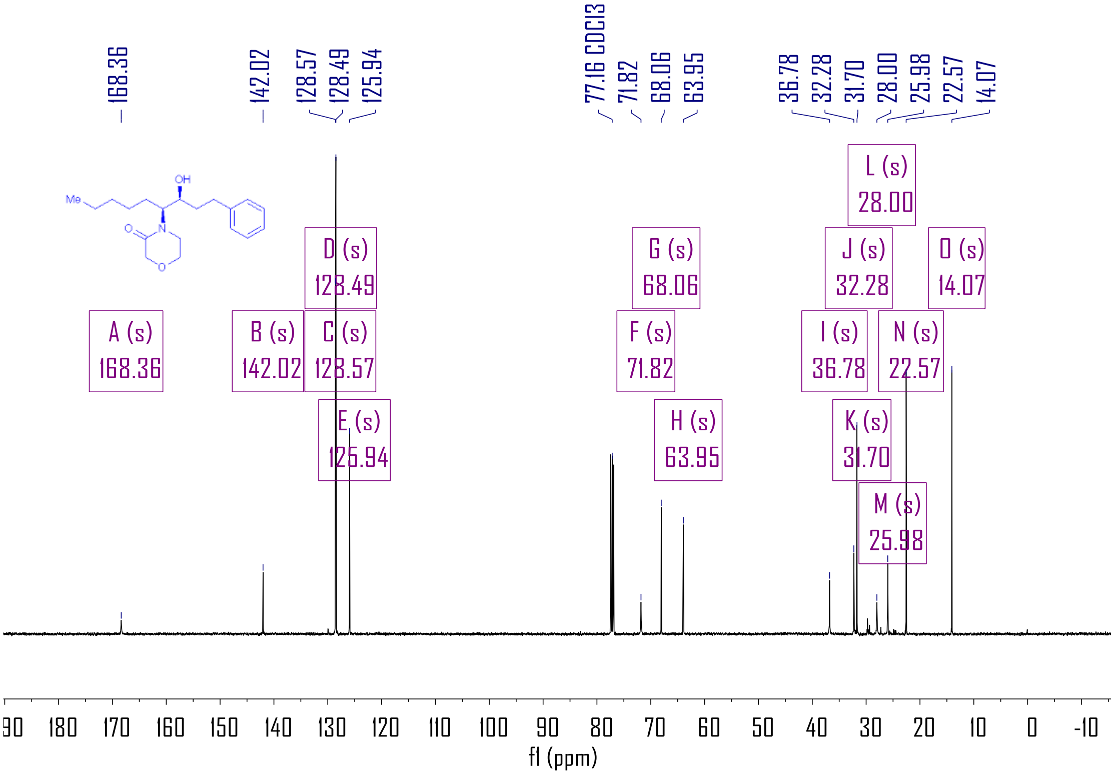

Supplementary Figure 271. ^{13}C NMR spectra for **87**

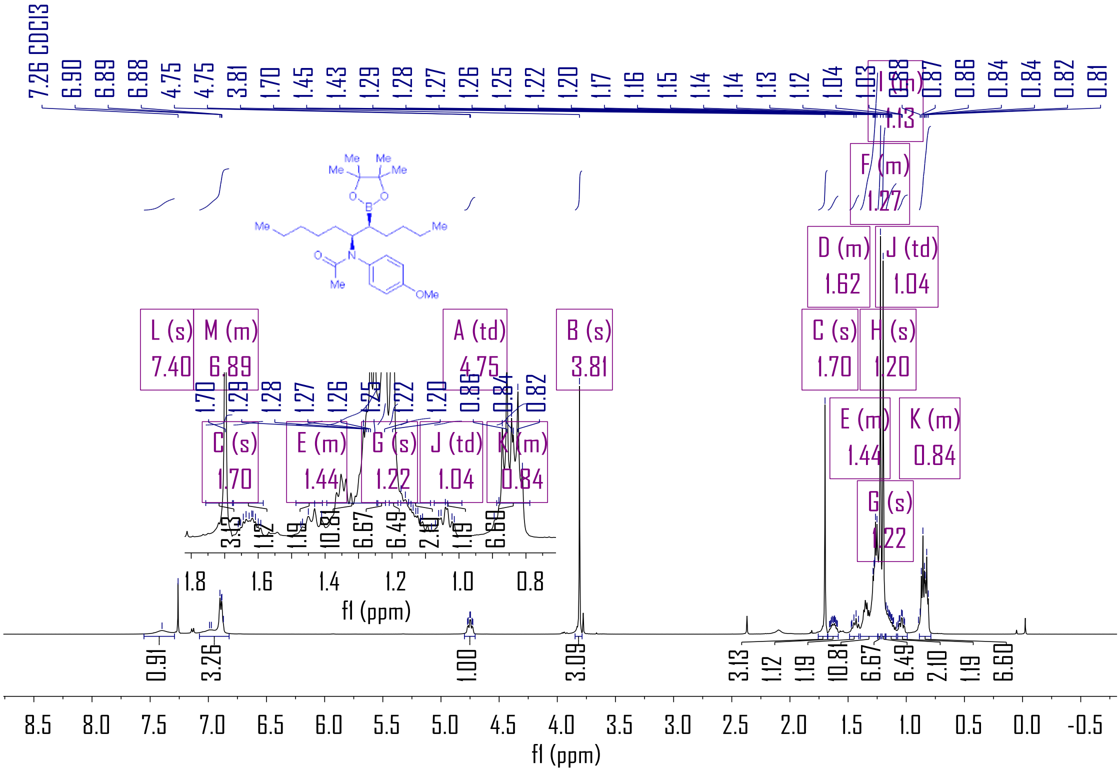

Supplementary Figure 272. ^{1}H NMR spectra for **88**

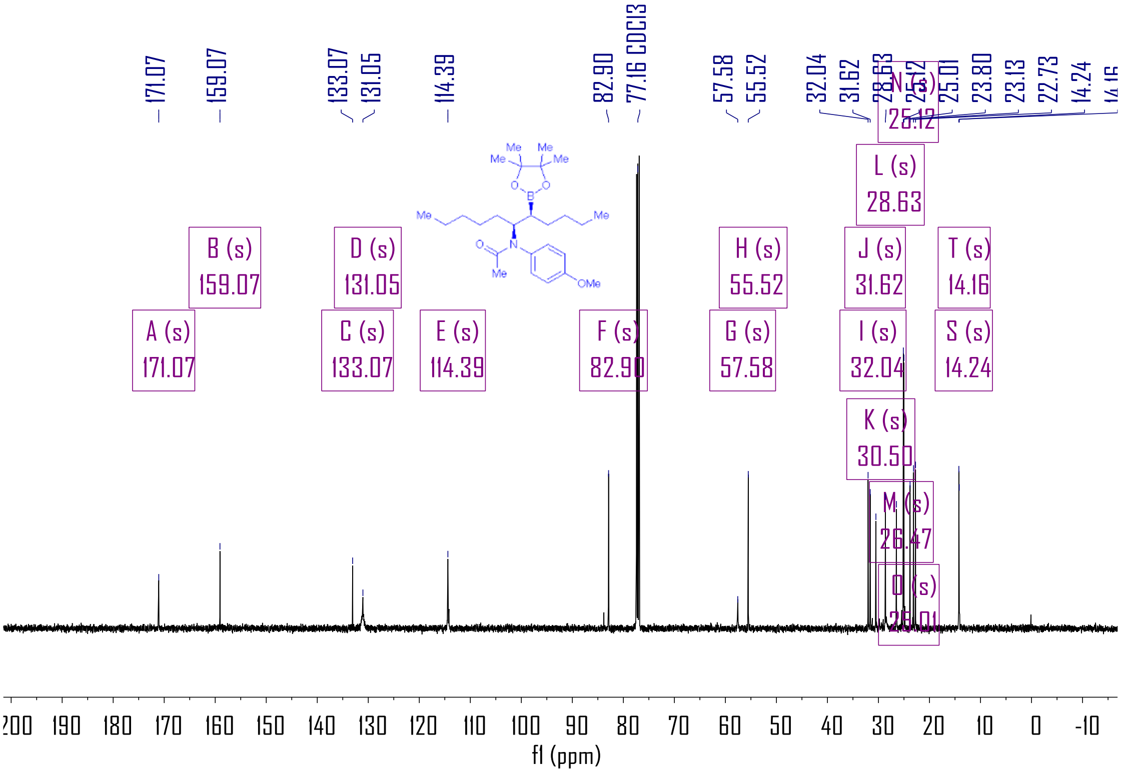

Supplementary Figure 273. ^{13}C NMR spectra for **88**

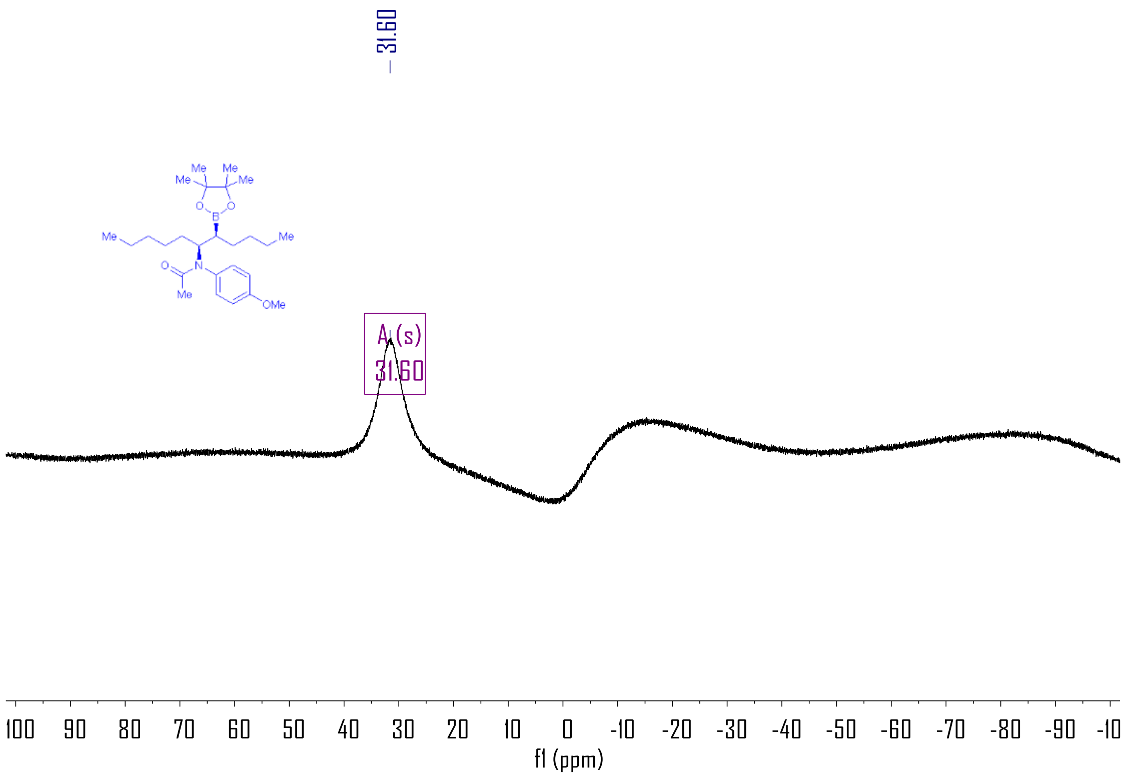

Supplementary Figure 274. ^{11}B NMR spectra for **88**

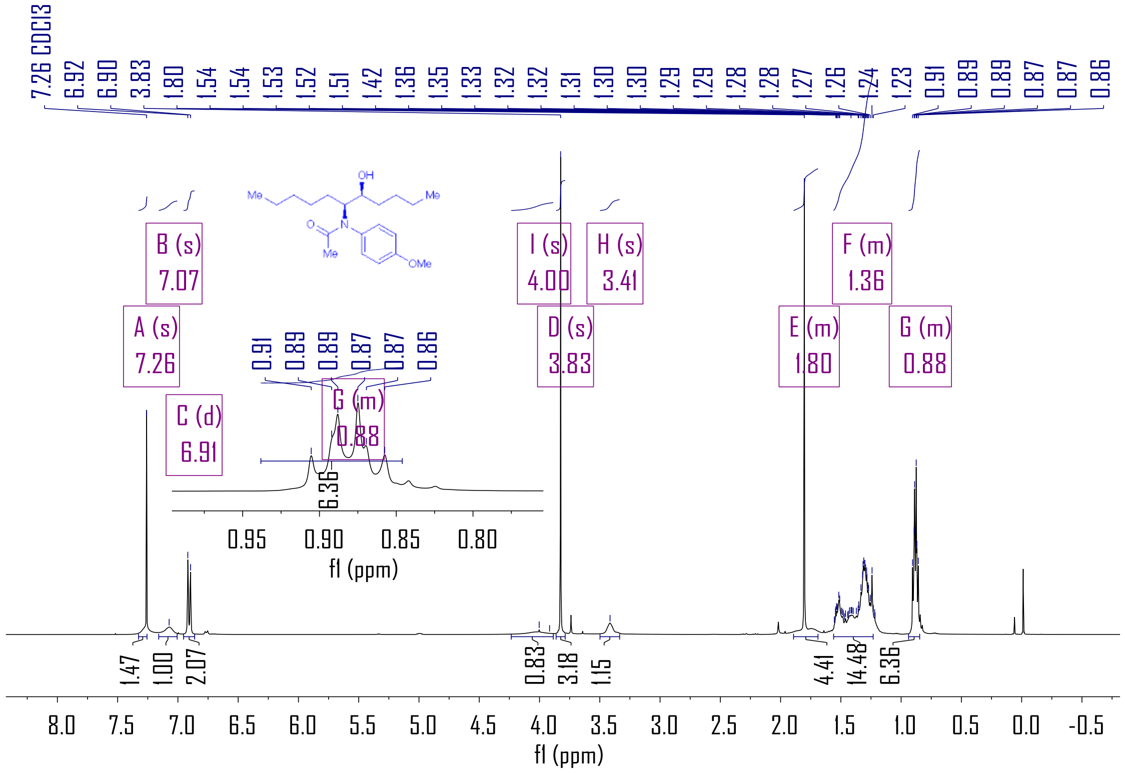

Supplementary Figure 275. ^{1}H NMR spectra for **88-OH**

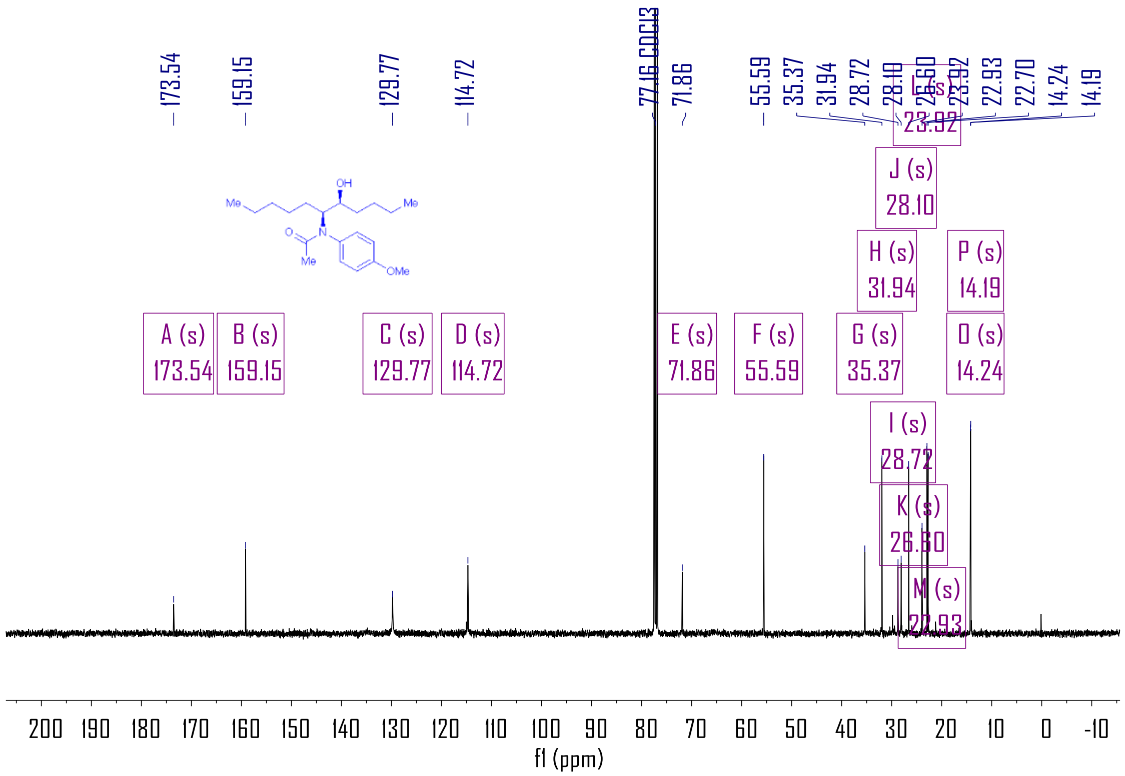

Supplementary Figure 276. ^{13}C NMR spectra for **88-OH**

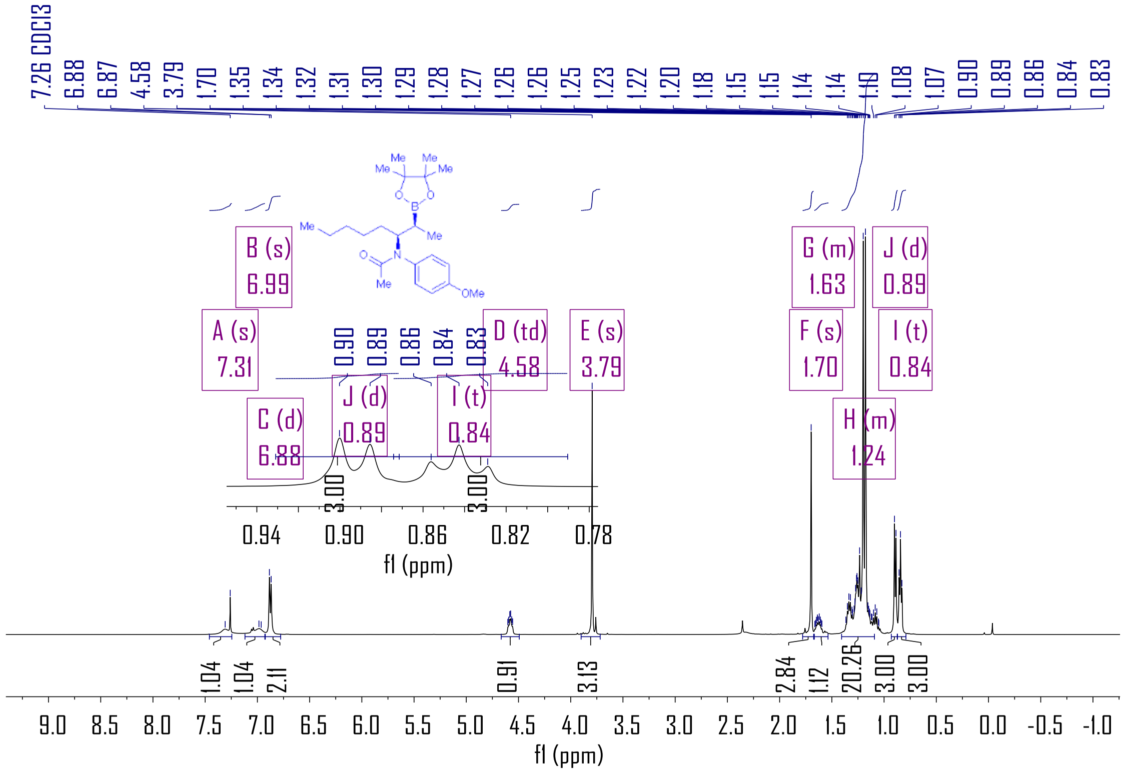

Supplementary Figure 277. ^{1}H NMR spectra for **89**

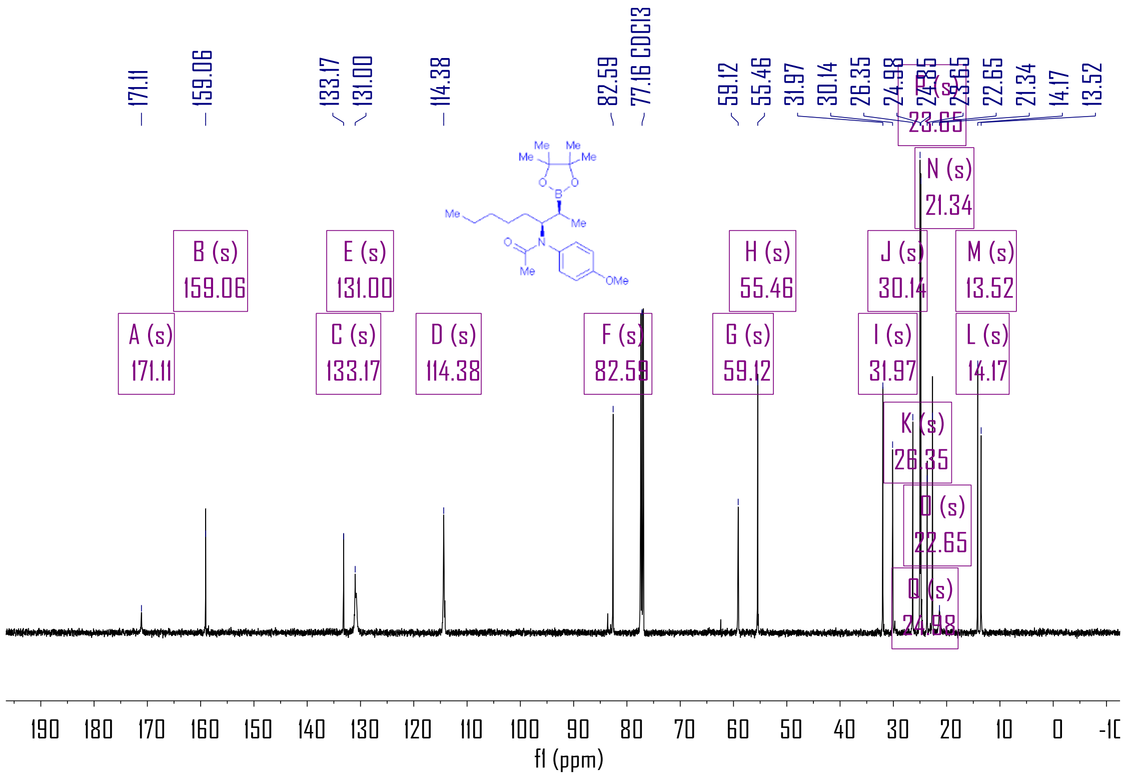

Supplementary Figure 278. ^{13}C NMR spectra for **89**

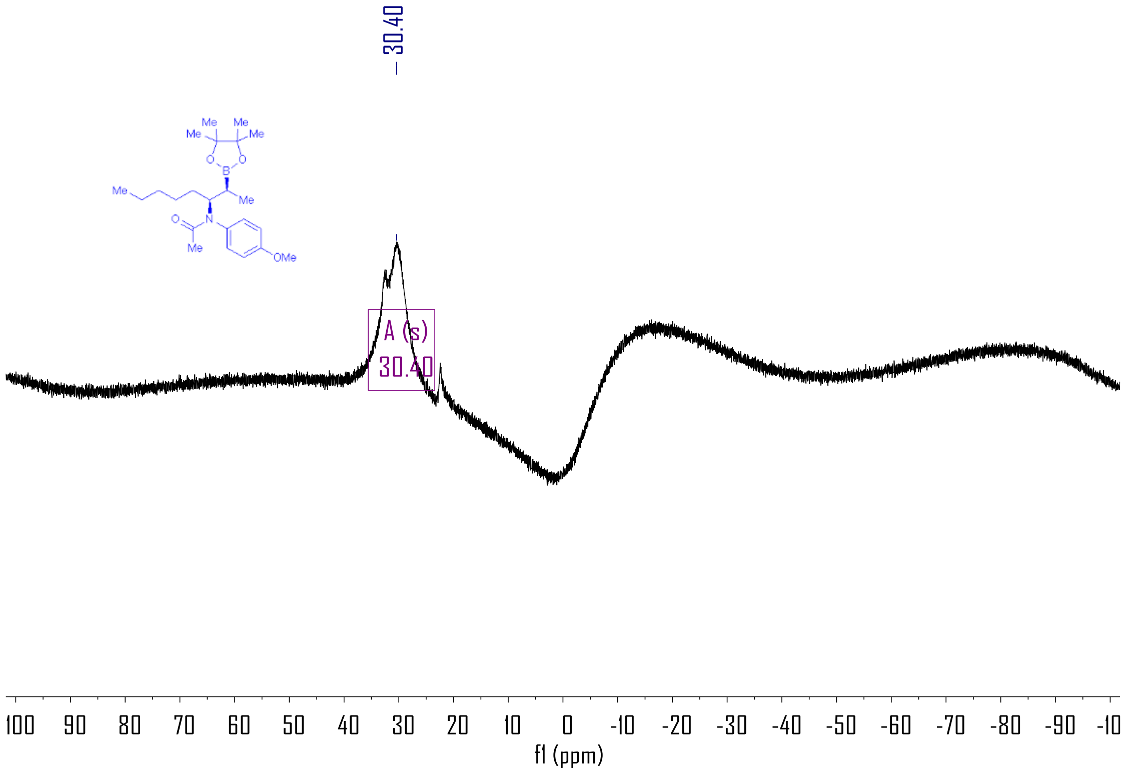

Supplementary Figure 279. ^{11}B NMR spectra for **89**

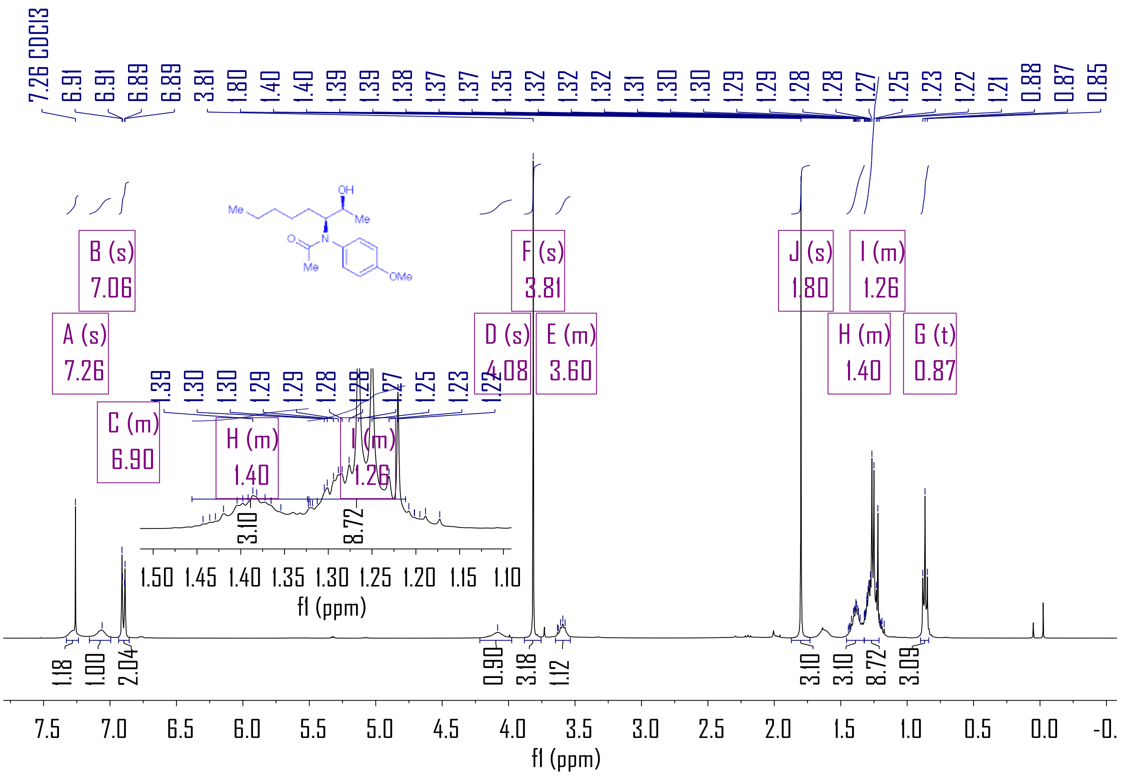

Supplementary Figure 280. ^{1}H NMR spectra for **89-OH**

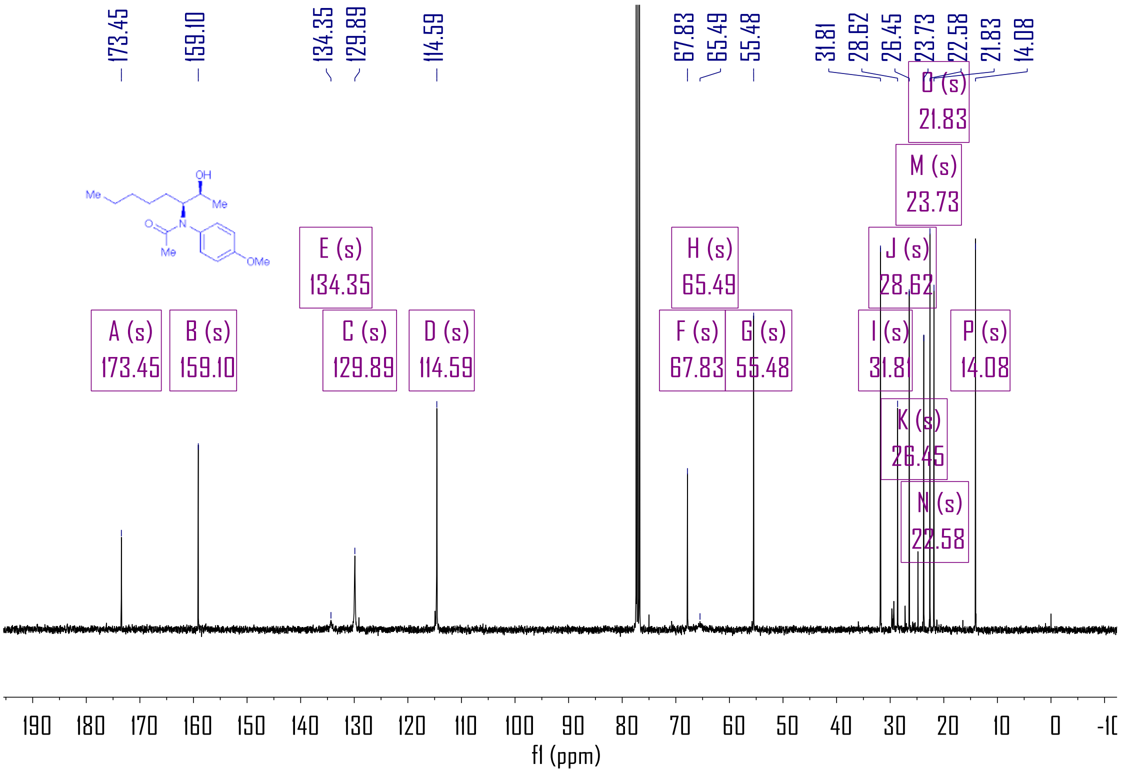

Supplementary Figure 281. ^{13}C NMR spectra for **89-OH**

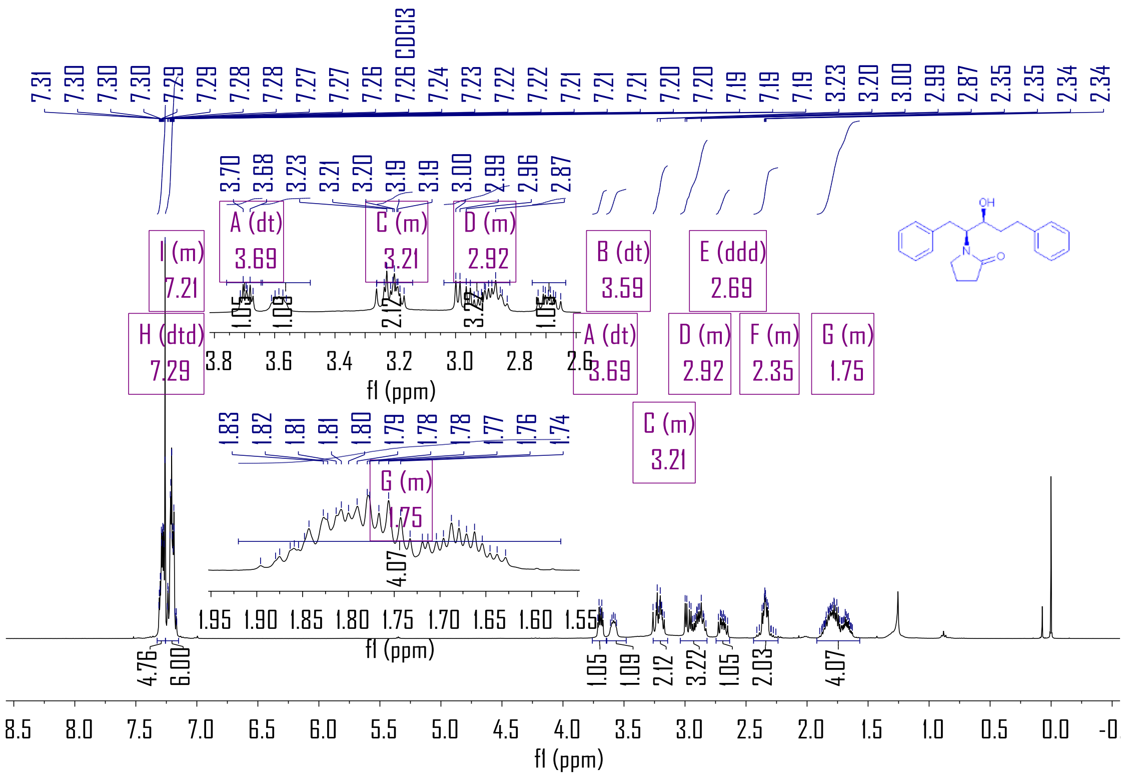

Supplementary Figure 282. ^{1}H NMR spectra for **90**

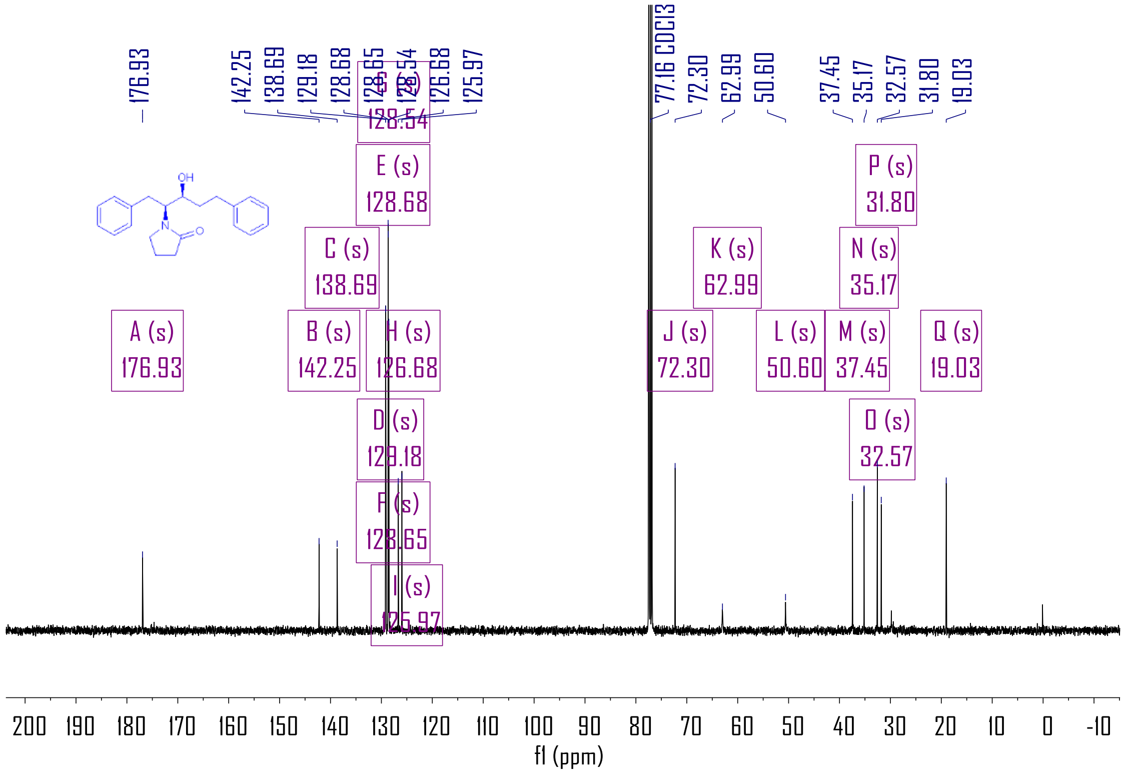

Supplementary Figure 283. ^{13}C NMR spectra for **90**

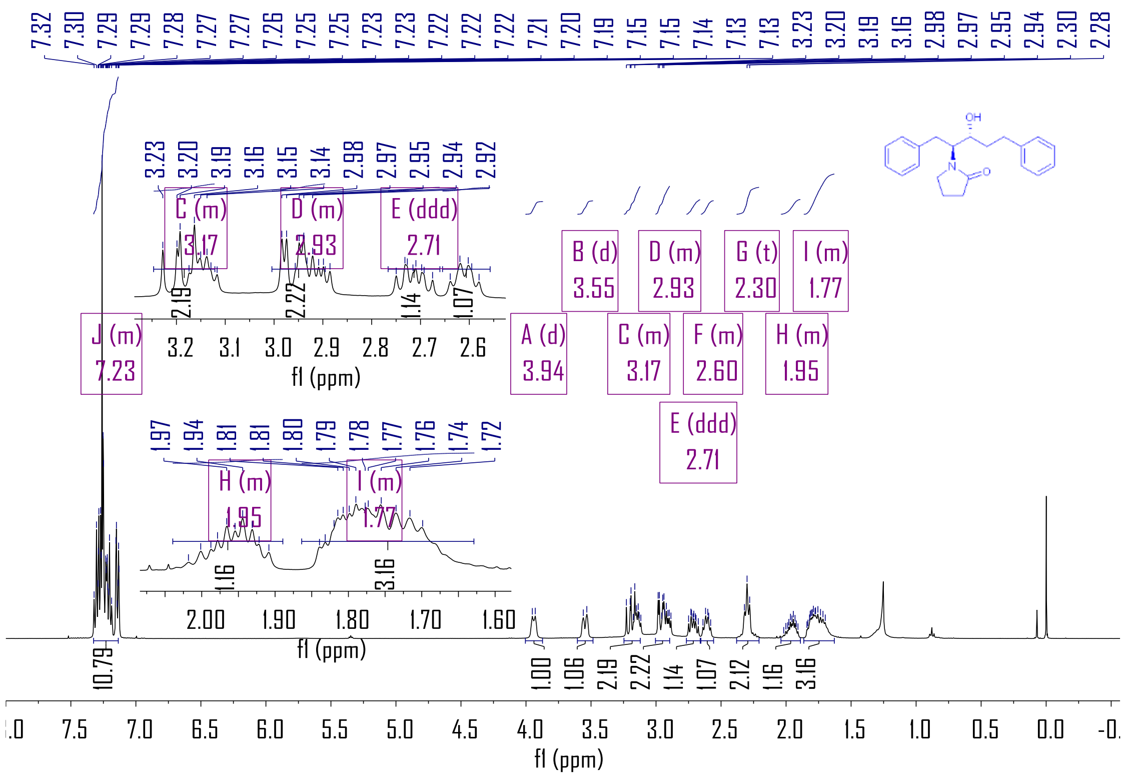

Supplementary Figure 284. ^{1}H NMR spectra for **91**

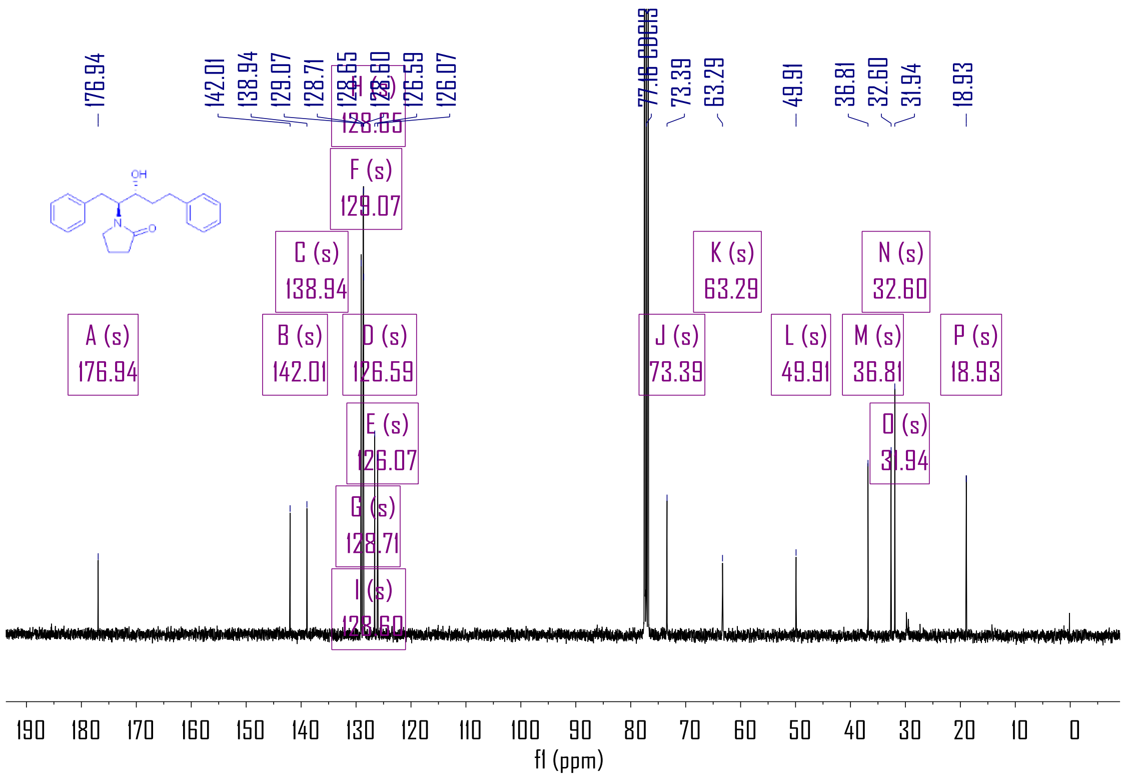

Supplementary Figure 285. ^{13}C NMR spectra for **91**

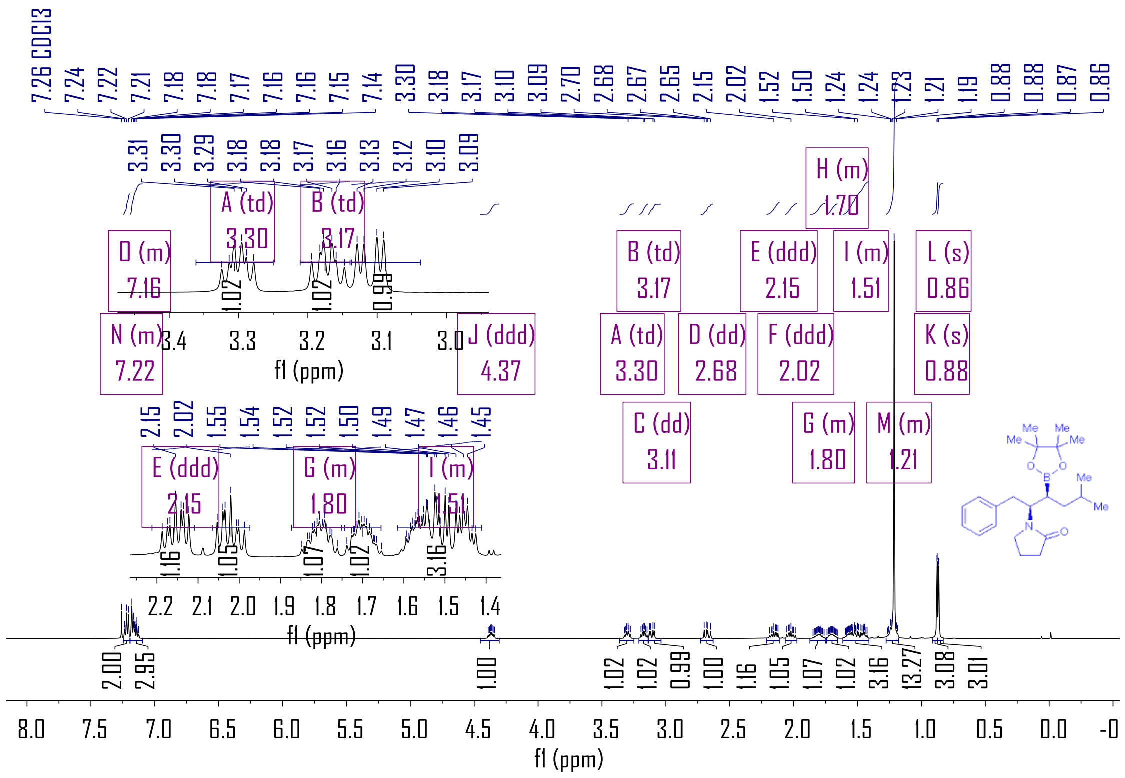

Supplementary Figure 286. ^{1}H NMR spectra for **92**

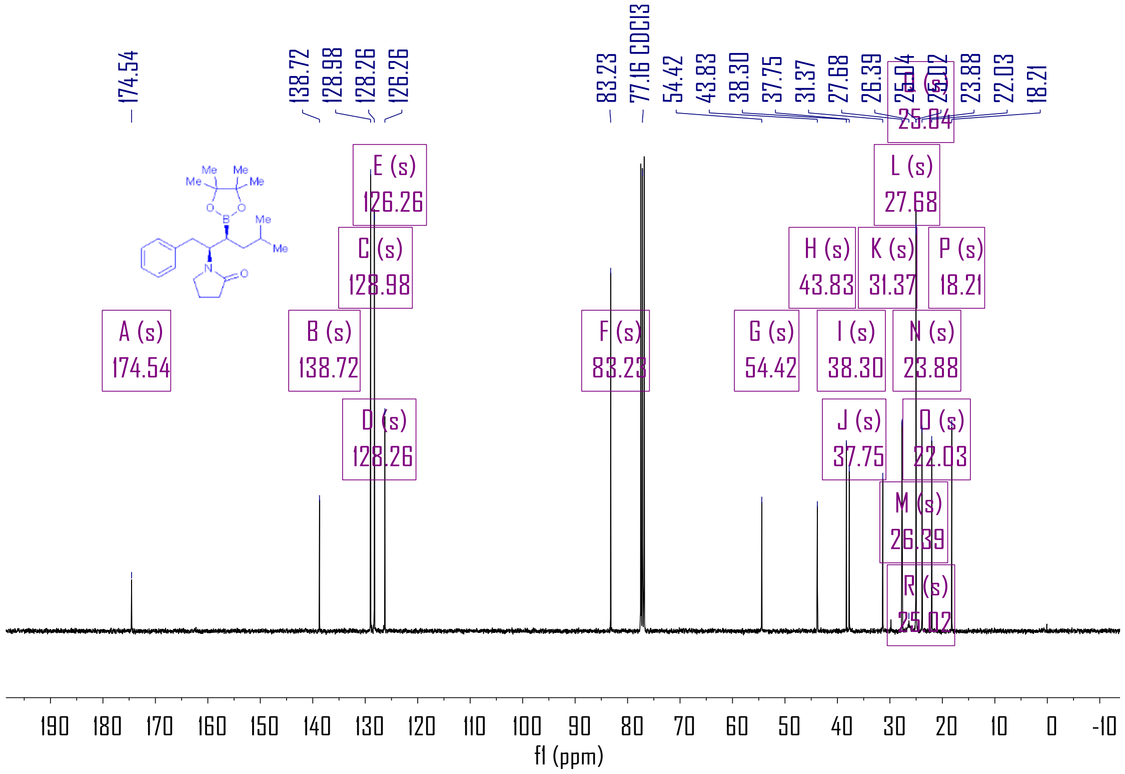

Supplementary Figure 287. ^{13}C NMR spectra for **92**

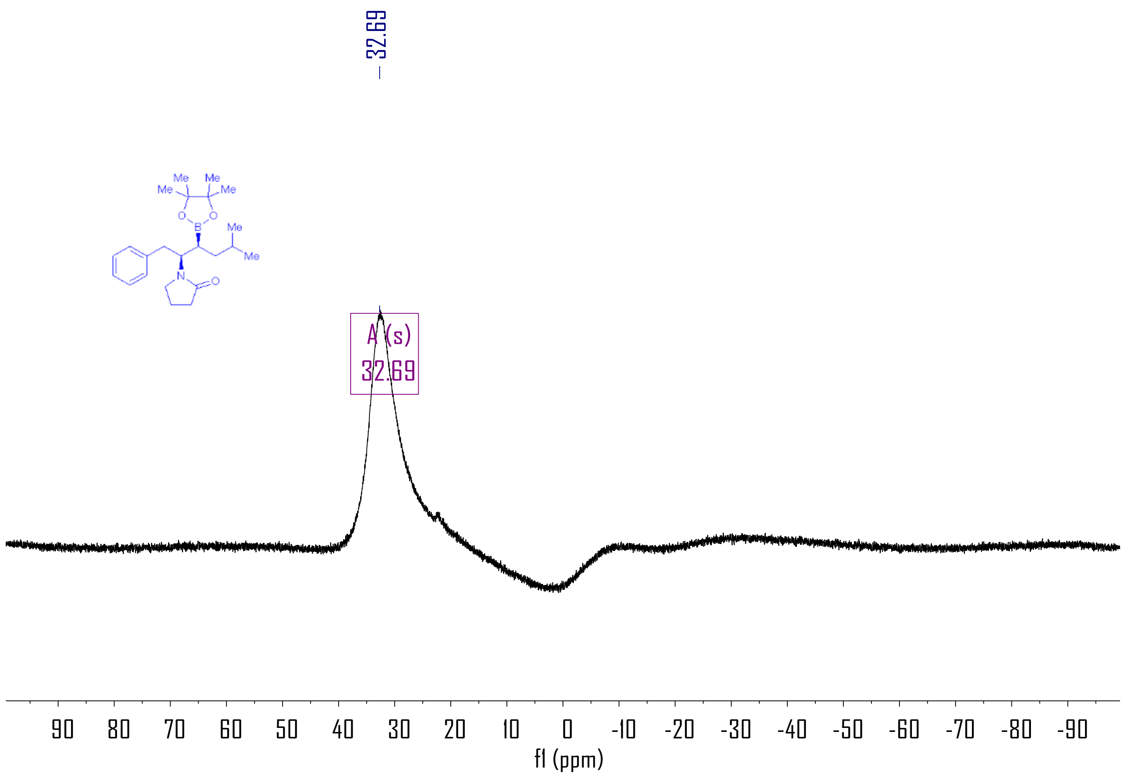

Supplementary Figure 288. ^{11}B NMR spectra for **92**

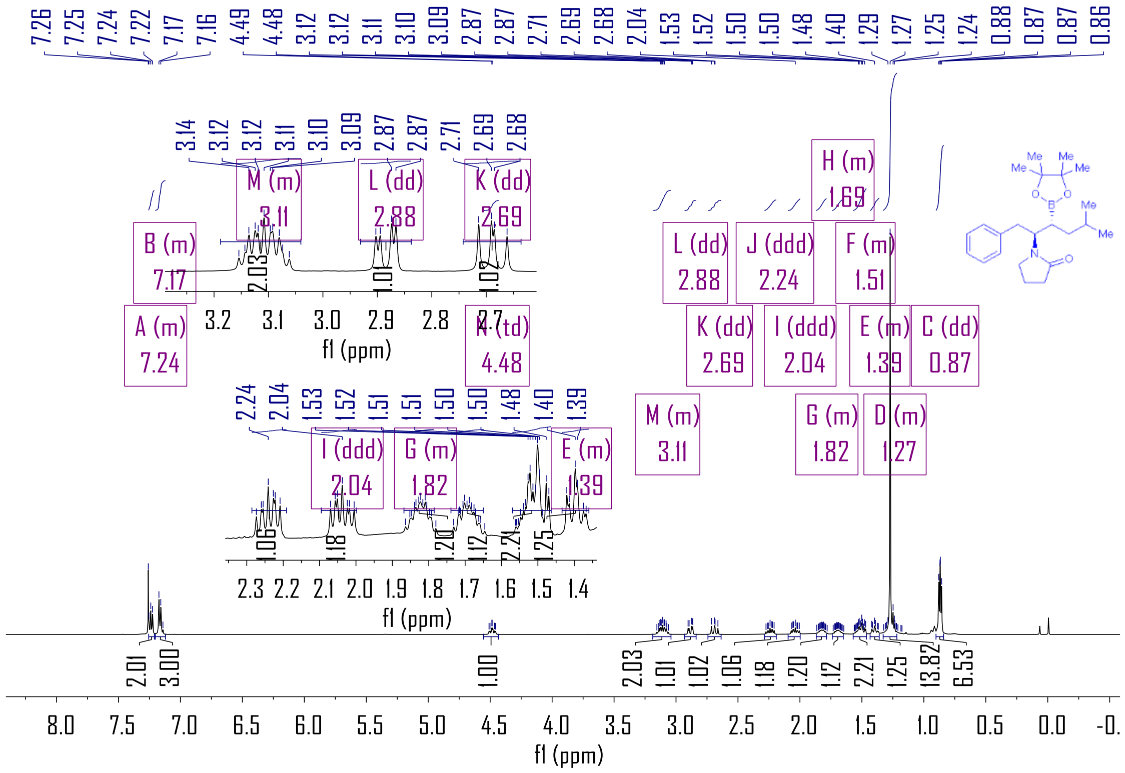

Supplementary Figure 289. ^{1}H NMR spectra for **93**

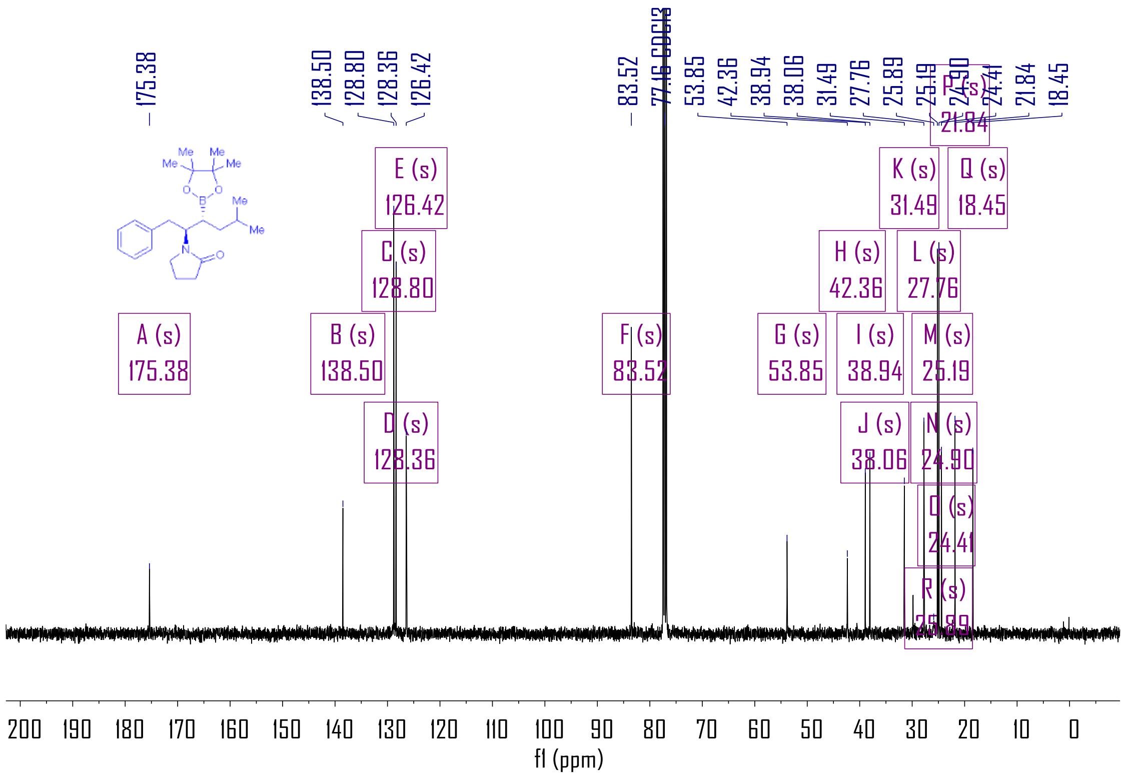

Supplementary Figure 290. ^{13}C NMR spectra for **93**

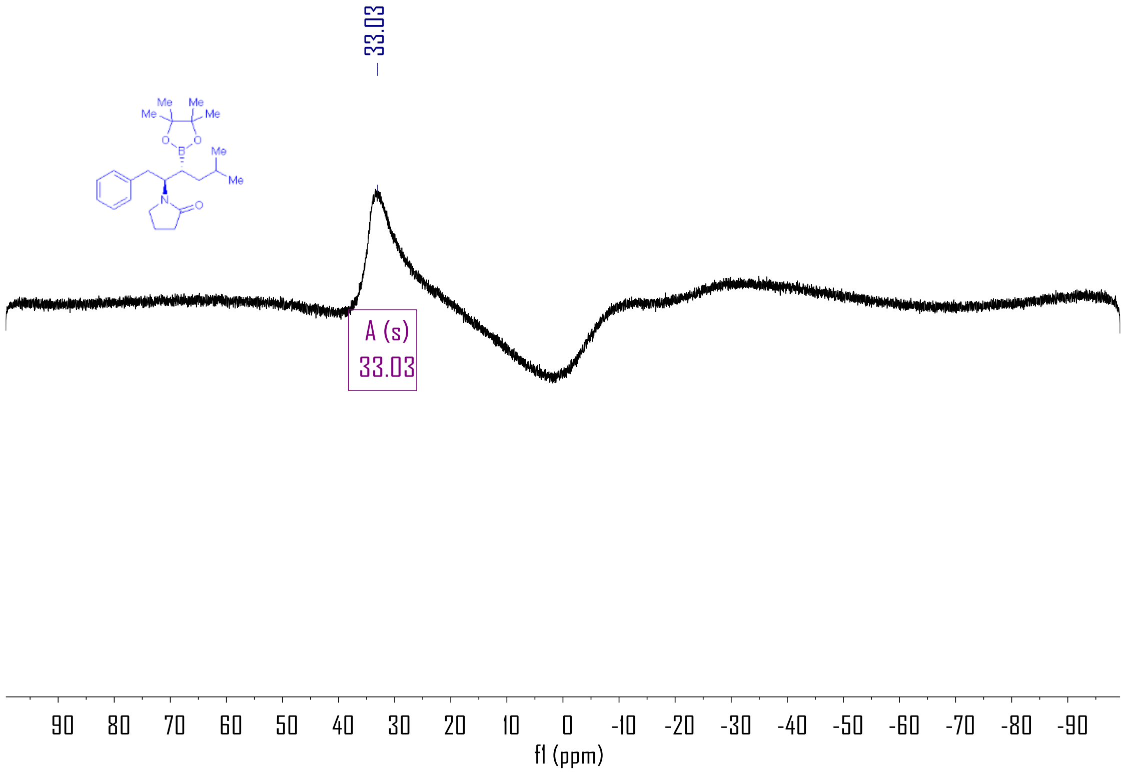

Supplementary Figure 291. ^{11}B NMR spectra for **93**

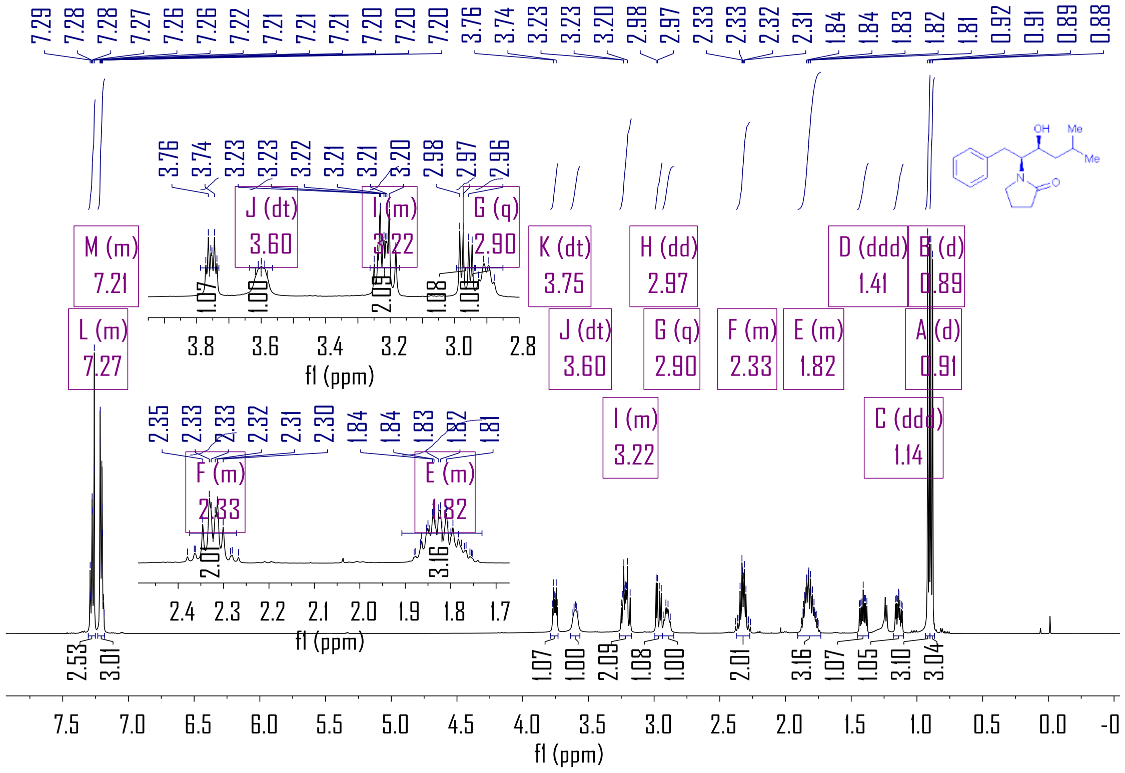

Supplementary Figure 292. ^{1}H NMR spectra for **95**

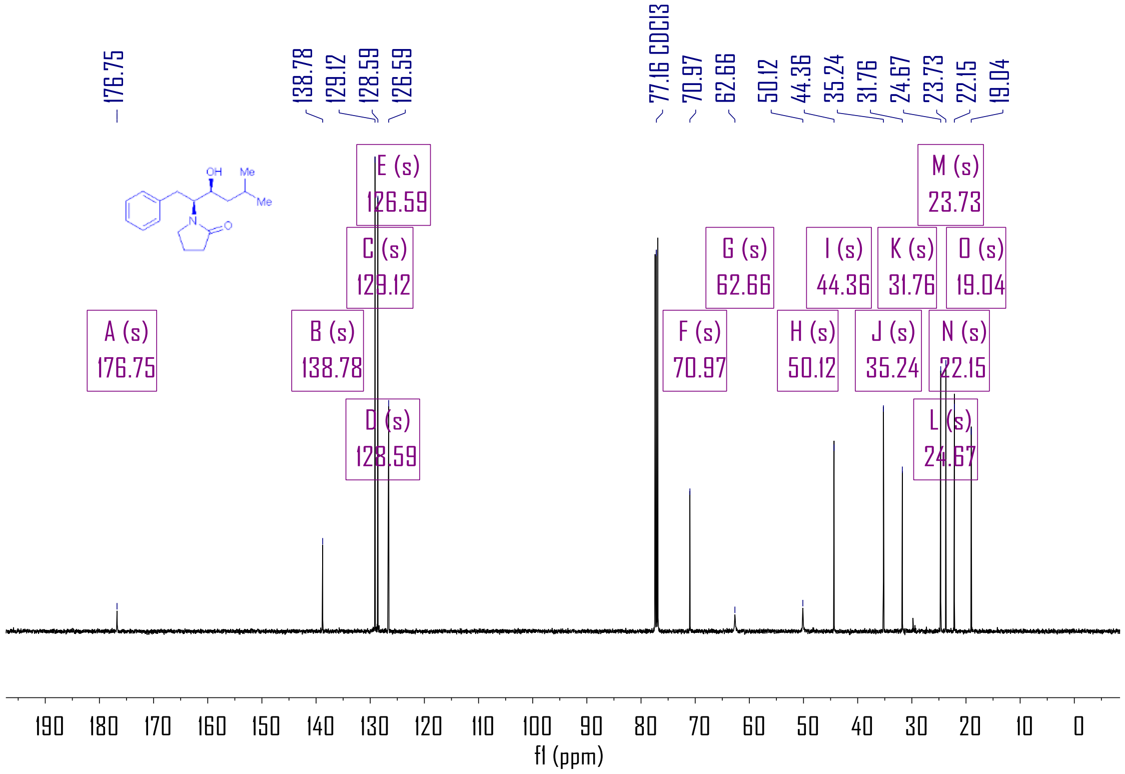

Supplementary Figure 293. ^{13}C NMR spectra for **95**

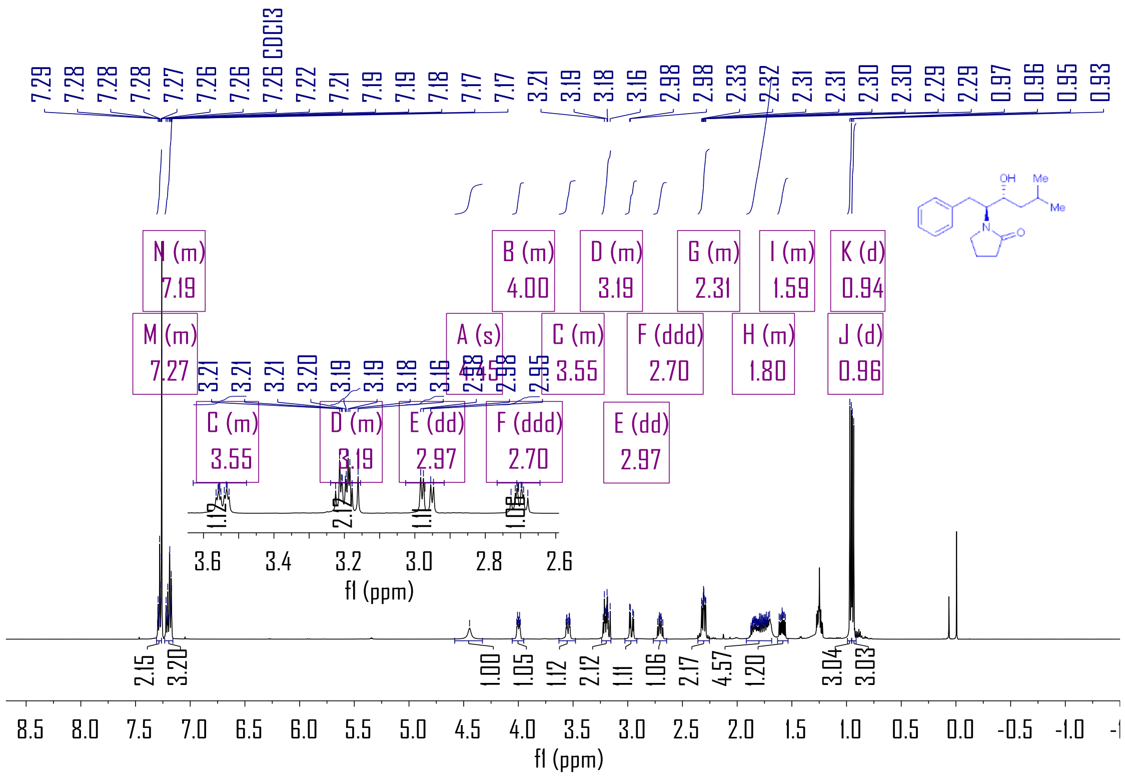

Supplementary Figure 294. ^{1}H NMR spectra for **93-OH**

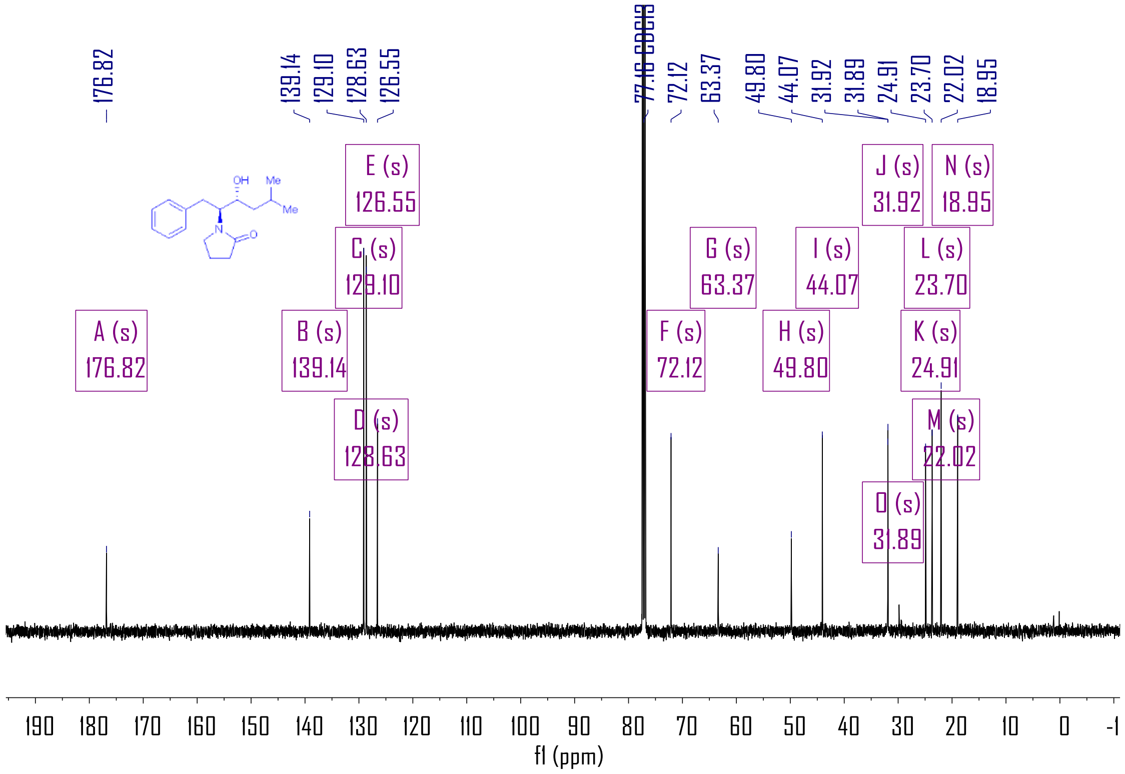

Supplementary Figure 295. ^{13}C NMR spectra for **93-OH**

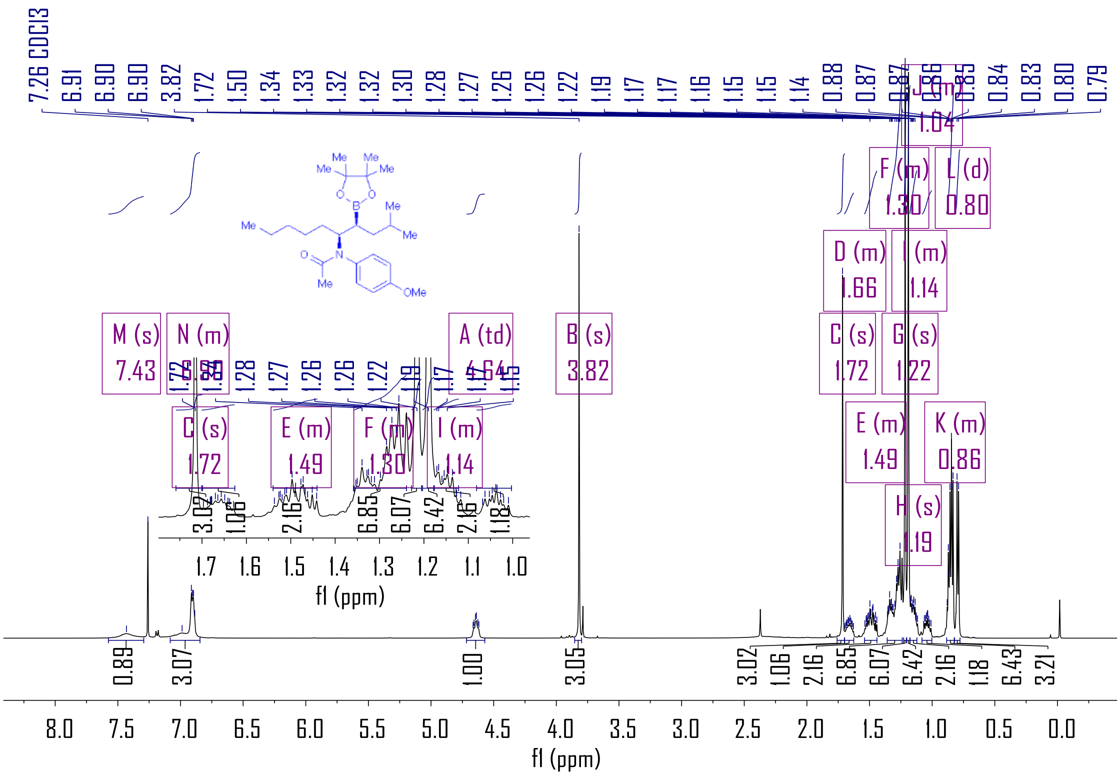

Supplementary Figure 296. ^{1}H NMR spectra for **94**

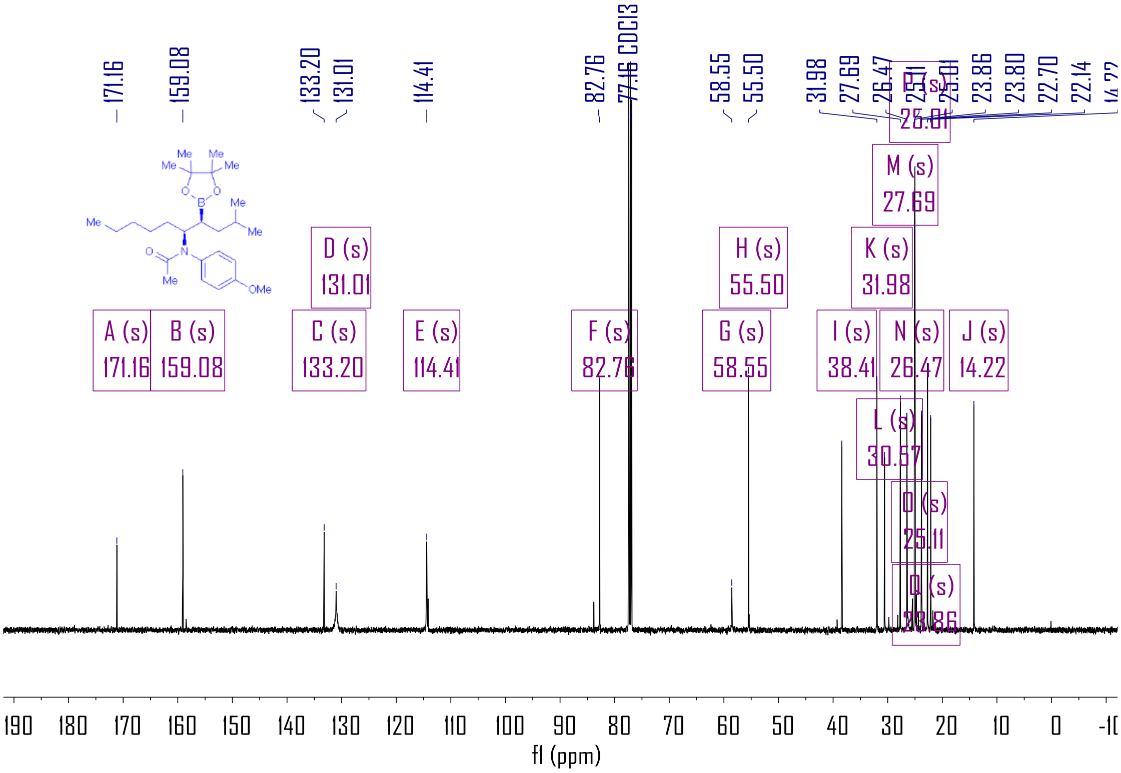

Supplementary Figure 297. ^{13}C NMR spectra for **94**

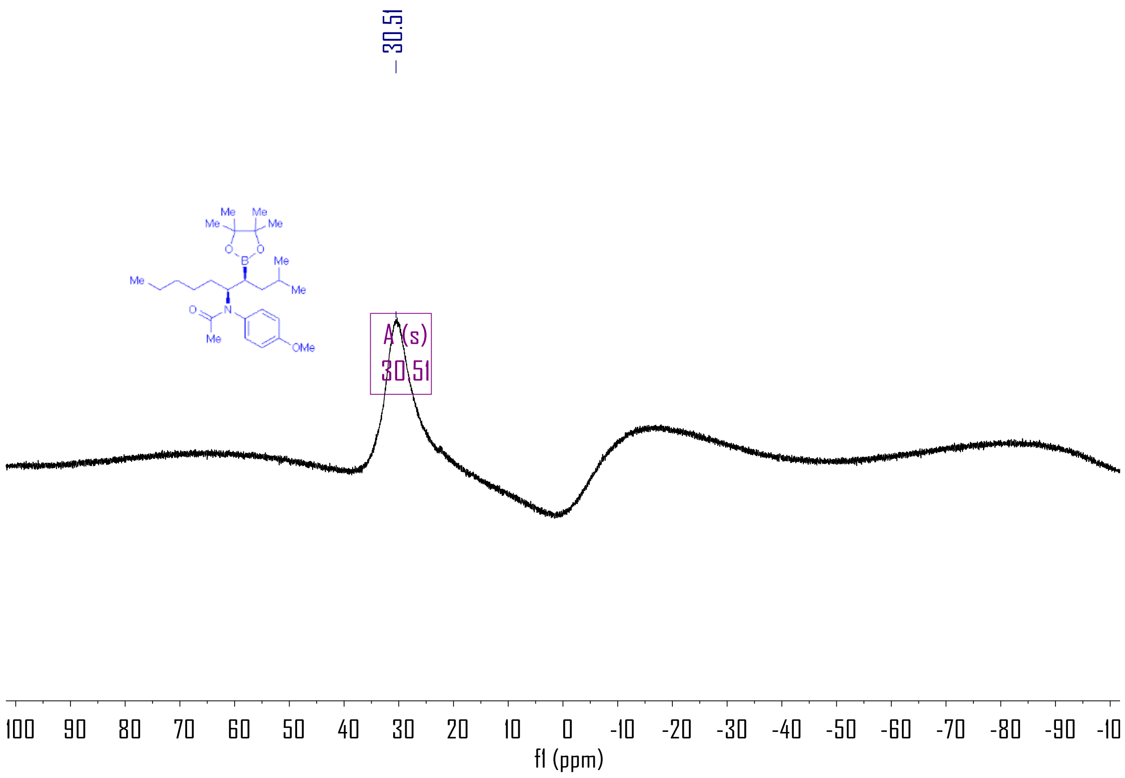

Supplementary Figure 298. ^{11}B NMR spectra for **94**

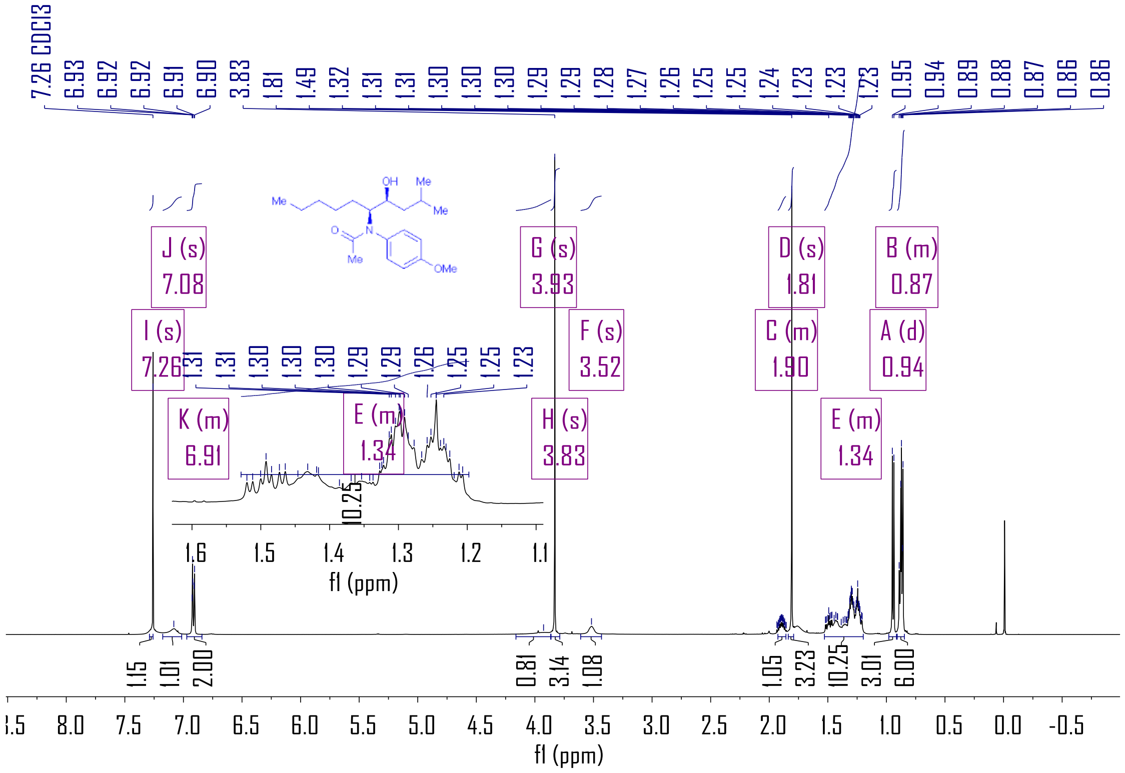

Supplementary Figure 299. ^{1}H NMR spectra for **94-OH**

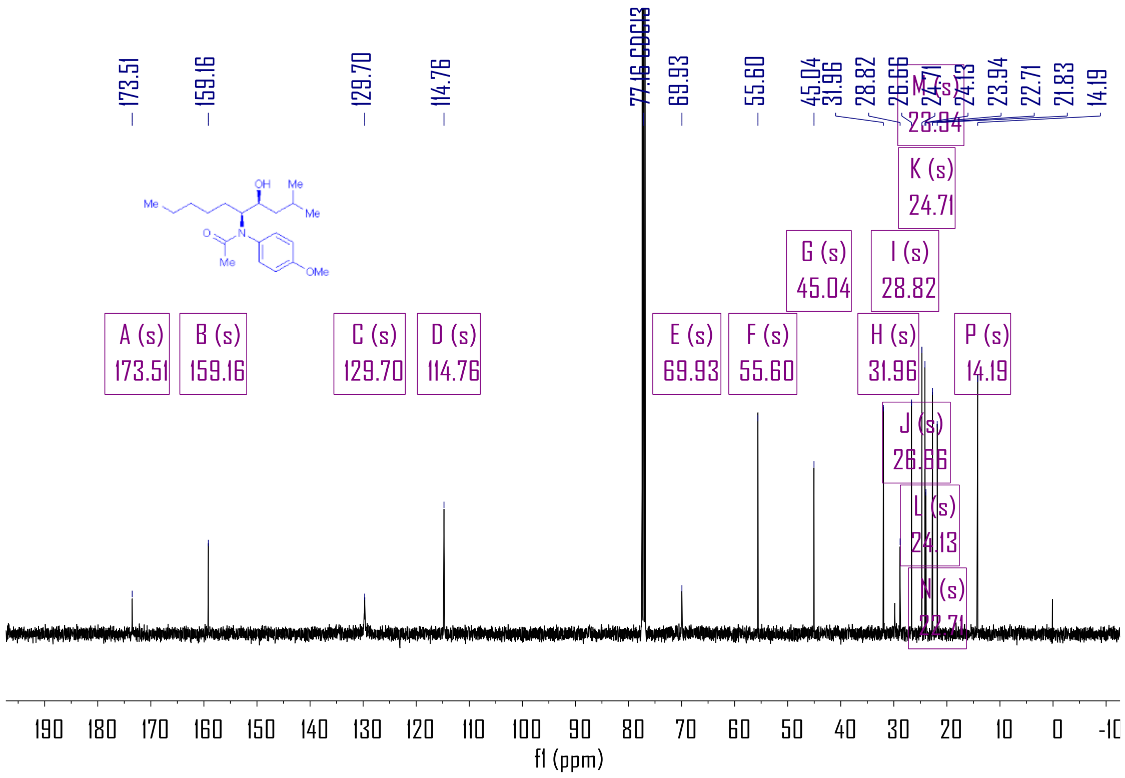

Supplementary Figure 300. ^{13}C NMR spectra for **94-OH**

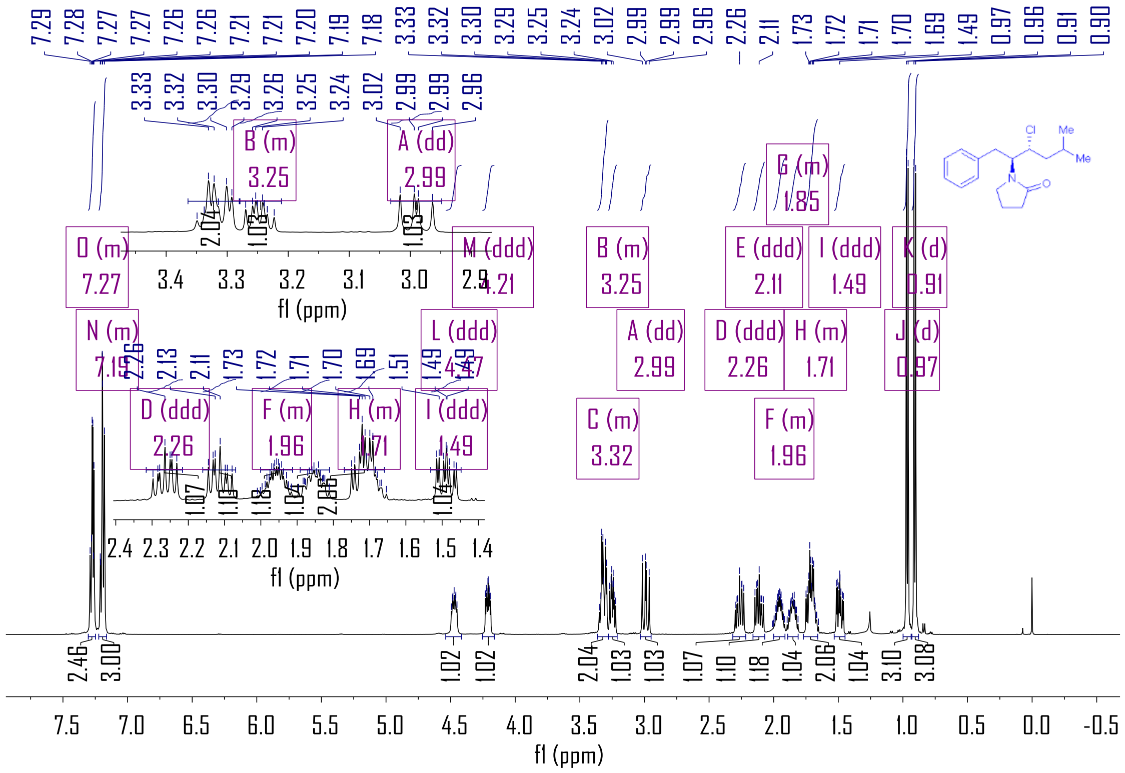

Supplementary Figure 301. ^{1}H NMR spectra for **96**

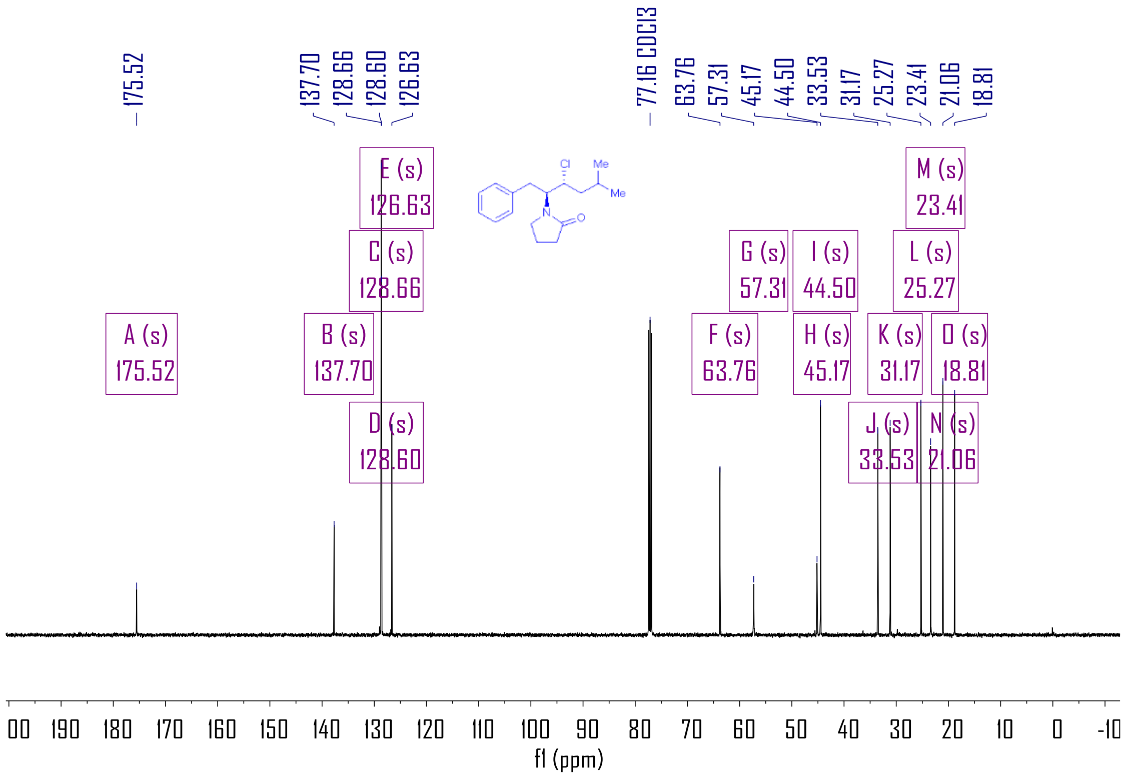

Supplementary Figure 302. ^{13}C NMR spectra for **96**

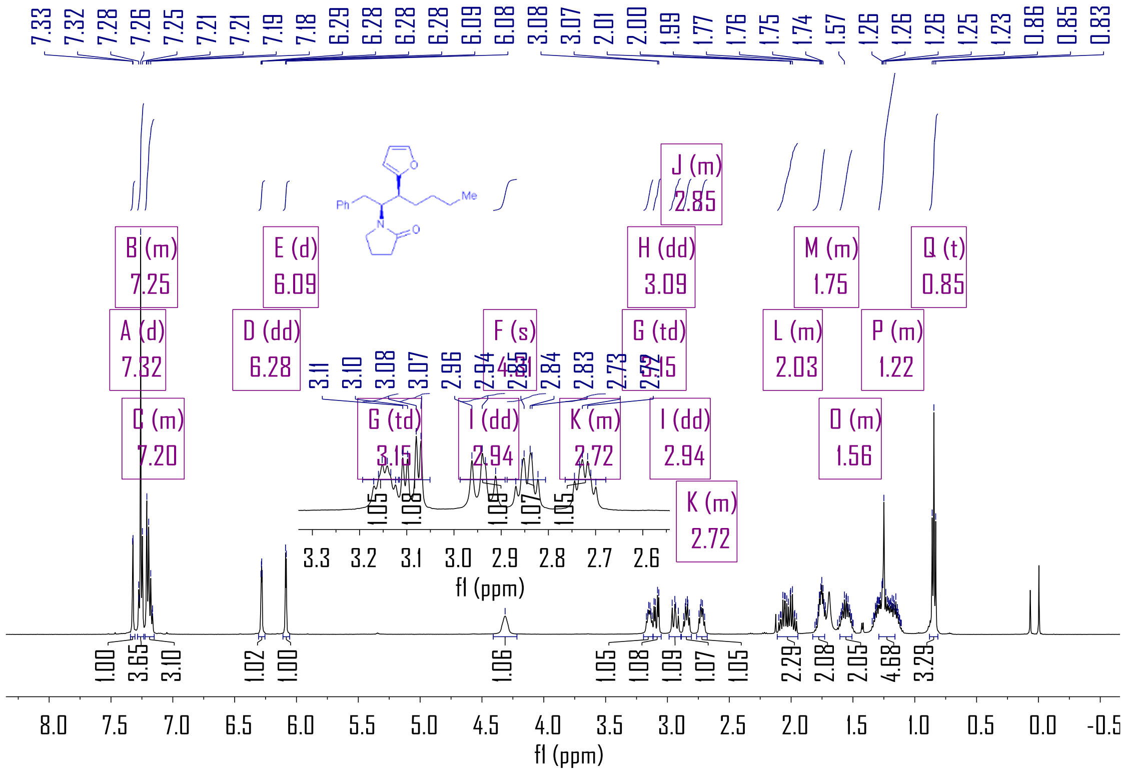

Supplementary Figure 303. ^{1}H NMR spectra for **97**

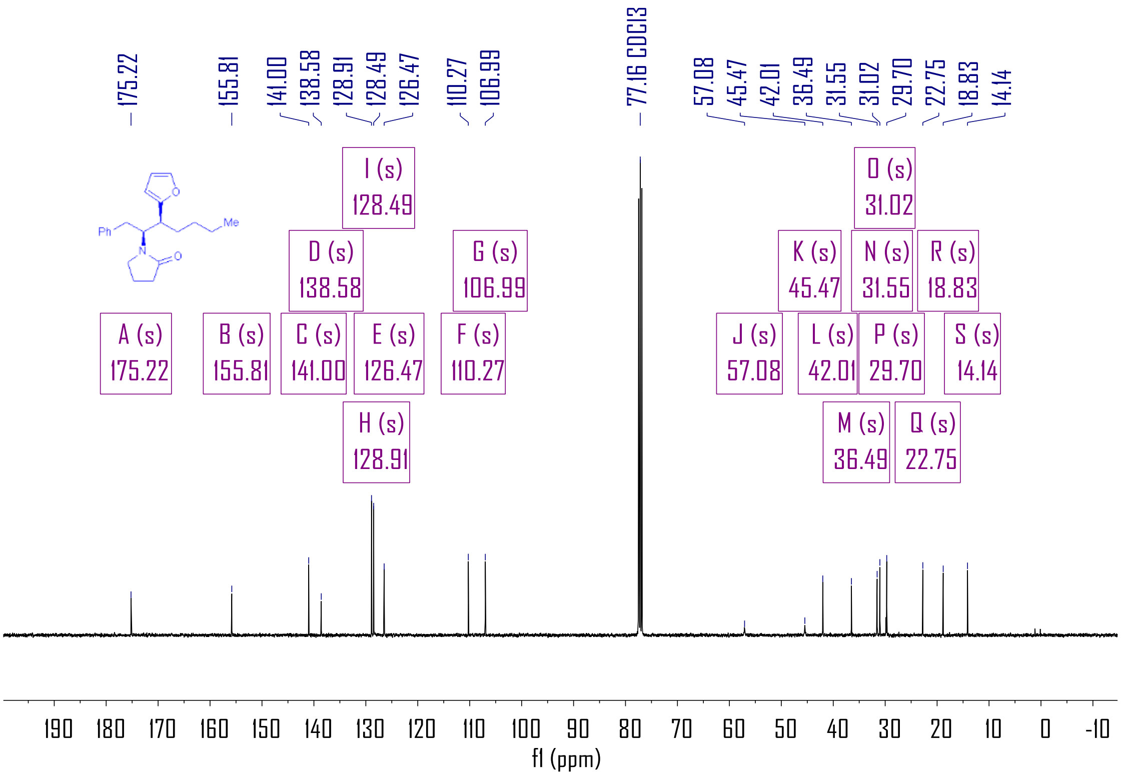

Supplementary Figure 304. ^{13}C NMR spectra for **97**

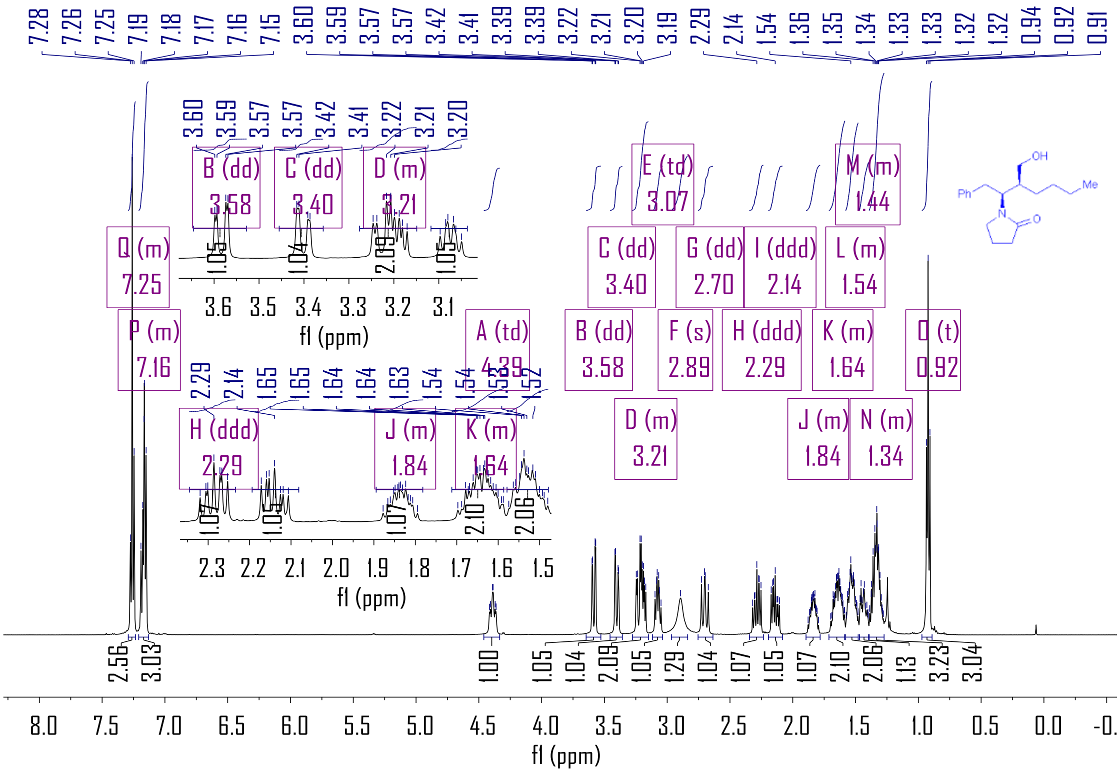

Supplementary Figure 305. ^{1}H NMR spectra for **98**

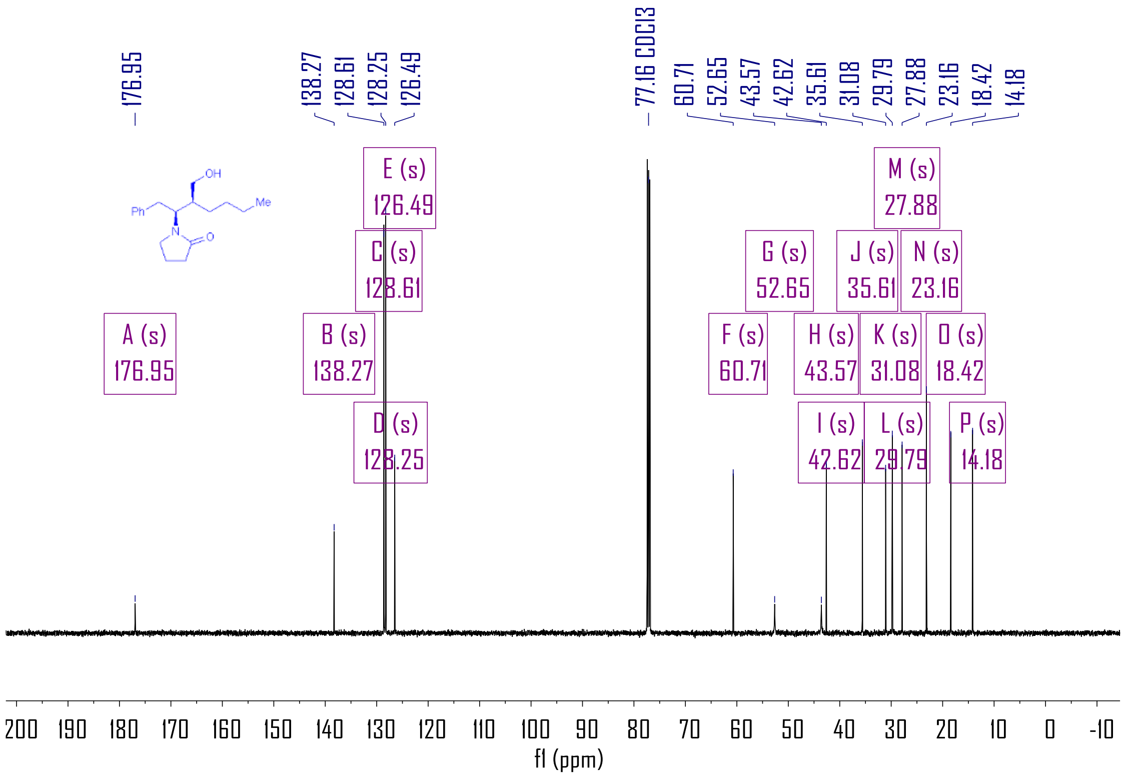

Supplementary Figure 306. ^{13}C NMR spectra for **98**

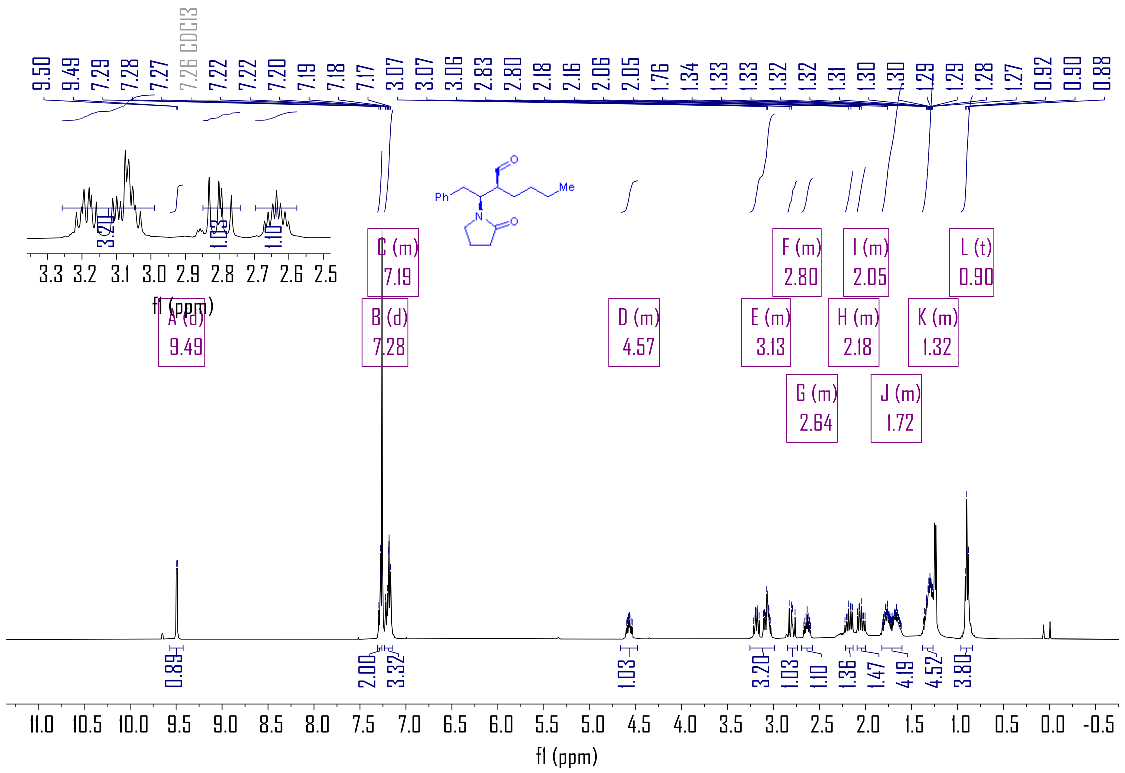

Supplementary Figure 307. ^{1}H NMR spectra for **99**

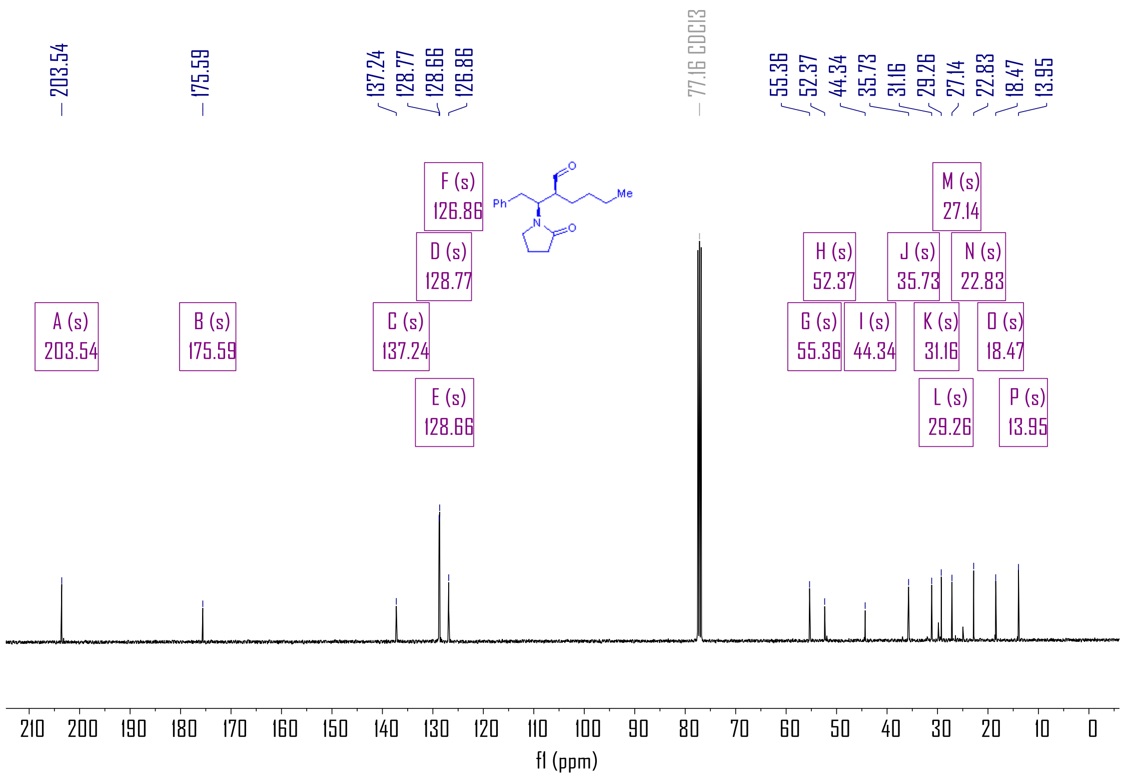

Supplementary Figure 308. ^{13}C NMR spectra for **99**

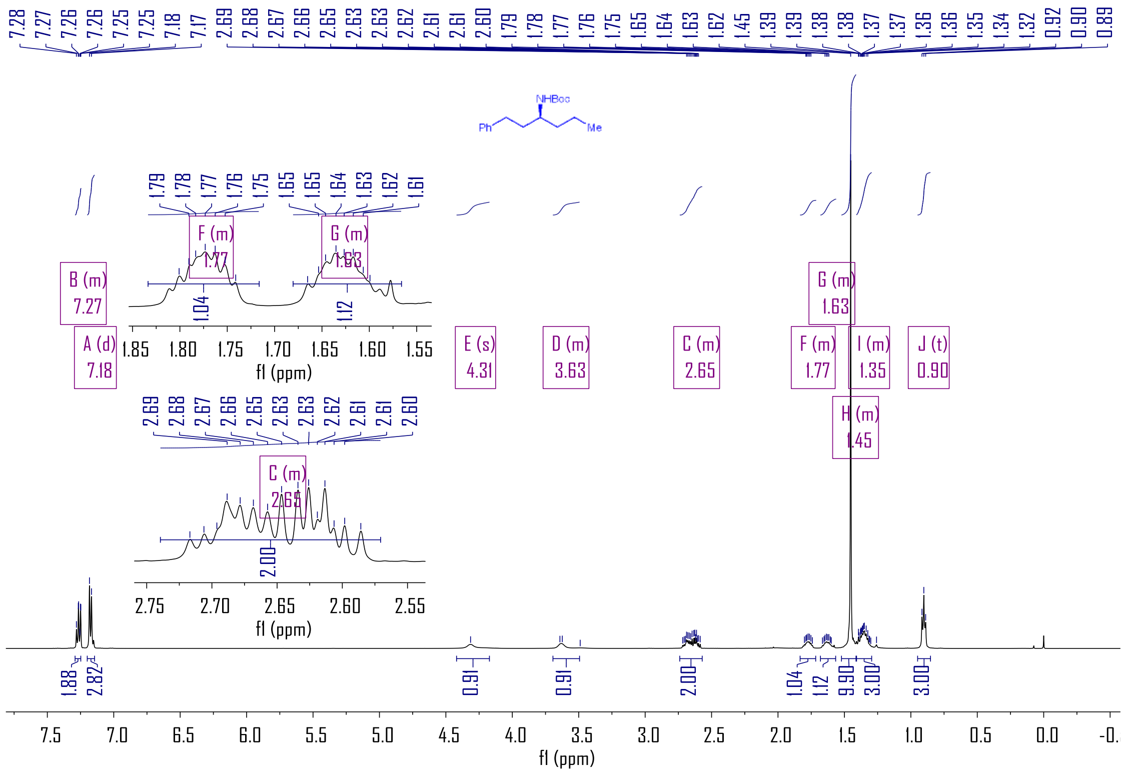

Supplementary Figure 309. ^{1}H NMR spectra for **100-Boc**

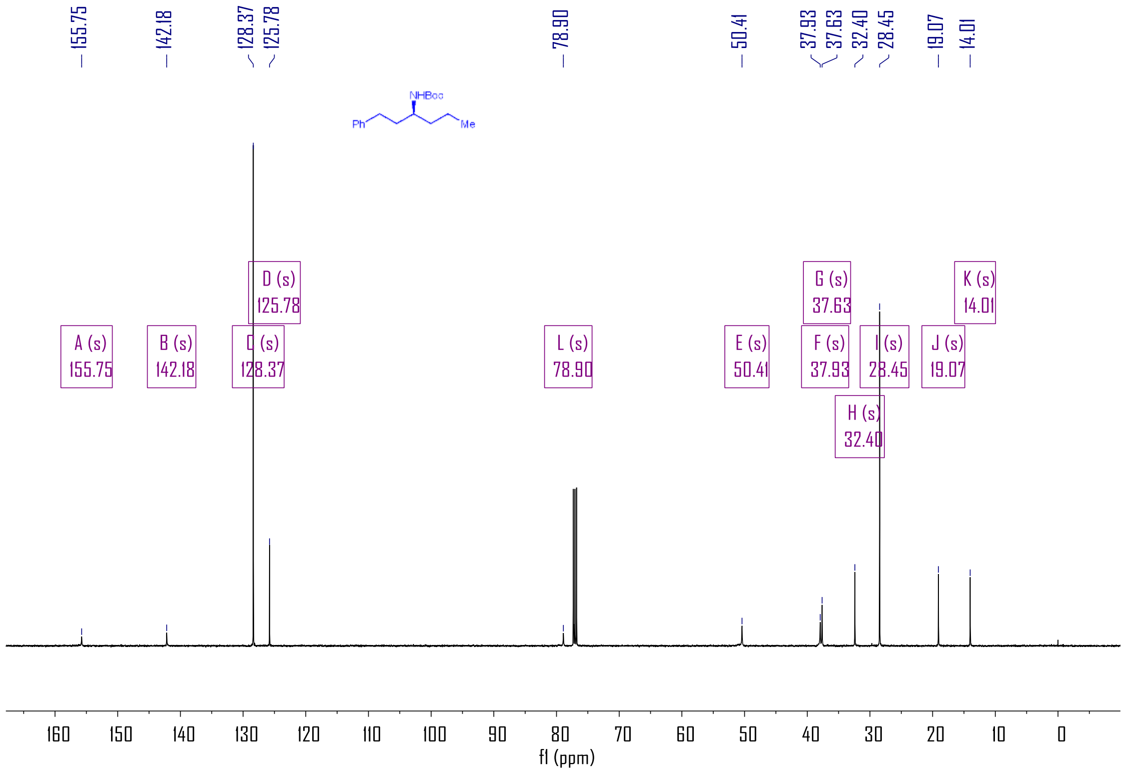

Supplementary Figure 310. ^{13}C NMR spectra for **100-Boc**

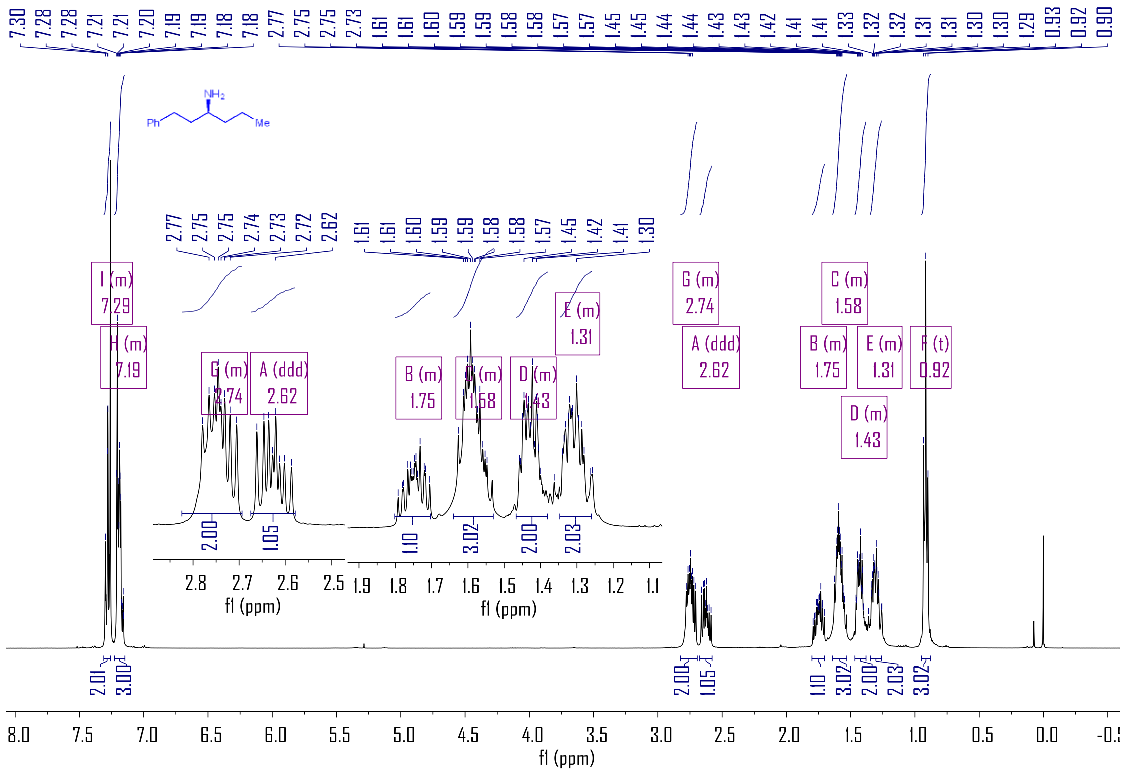

Supplementary Figure 311. ^{1}H NMR spectra for **100**

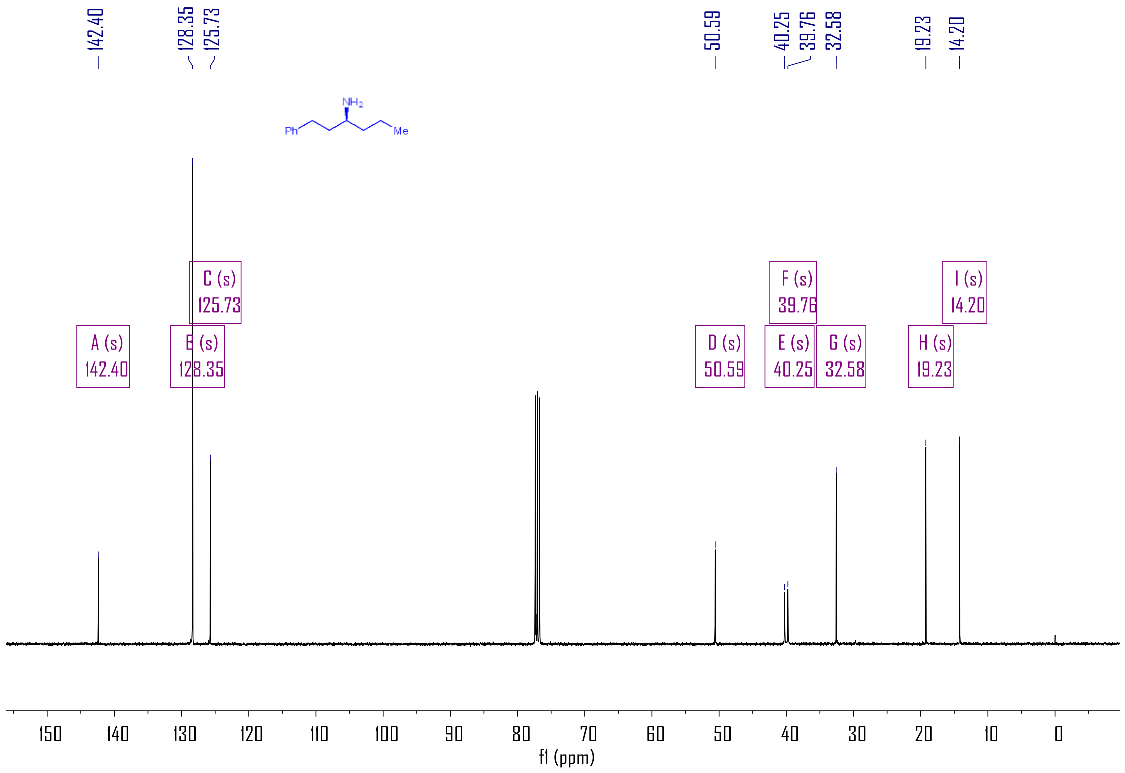

Supplementary Figure 312. ^{13}C NMR spectra for **100**

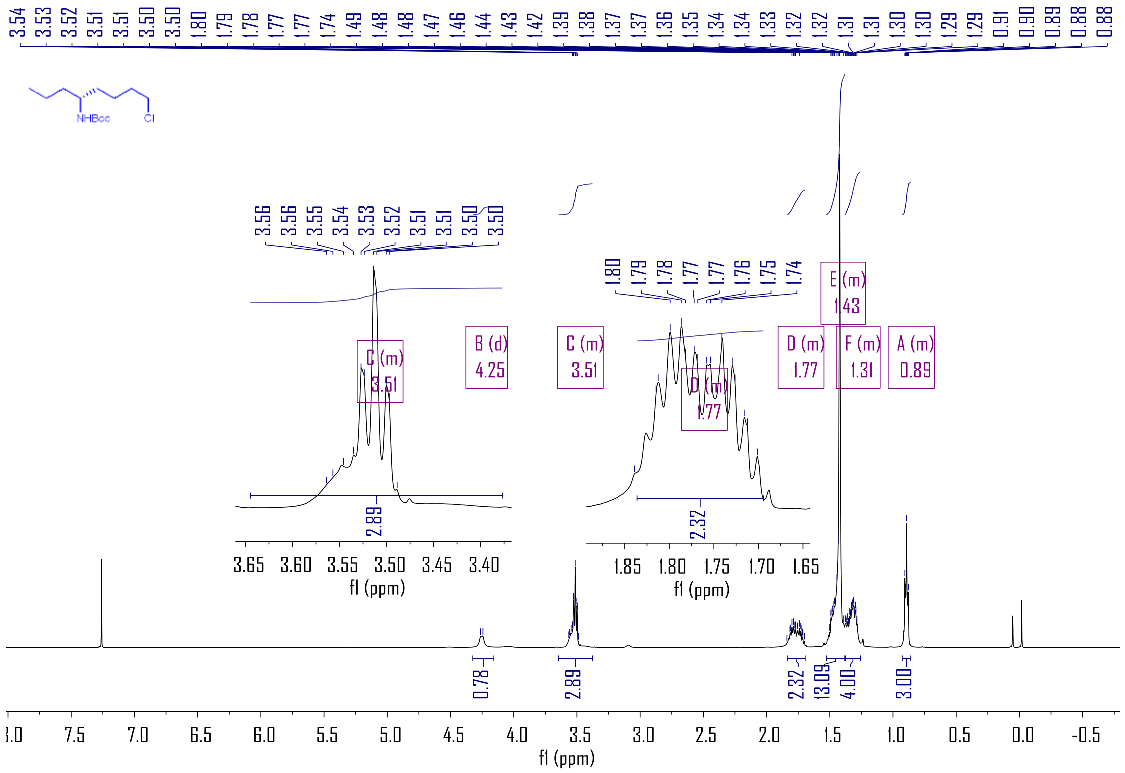

Supplementary Figure 313. ^{1}H NMR spectra for **106**

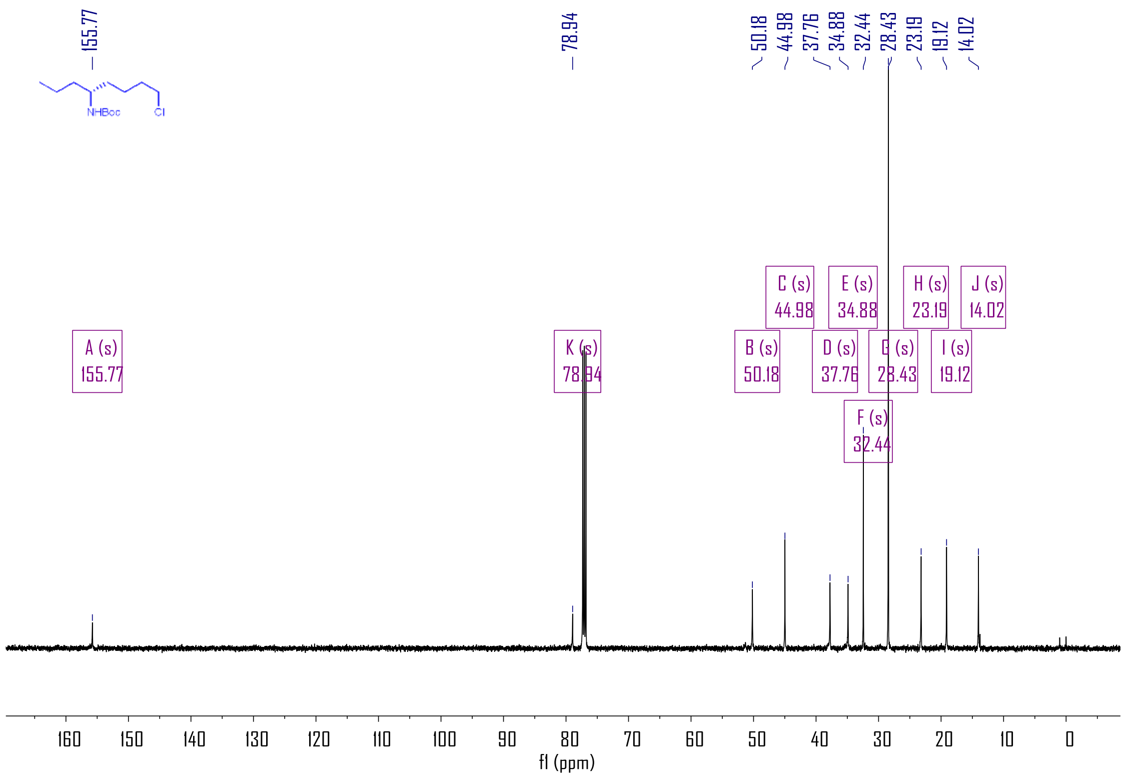

Supplementary Figure 314. ^{13}C NMR spectra for **106**

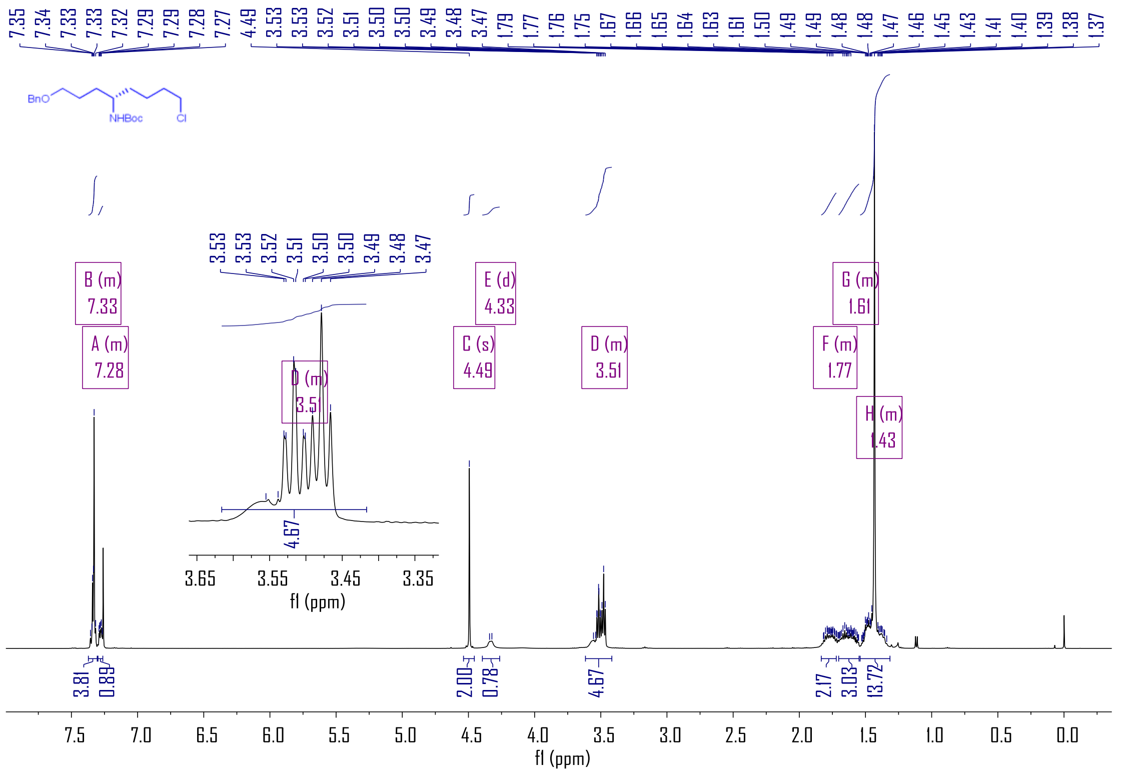

Supplementary Figure 315. ^{1}H NMR spectra for **107**

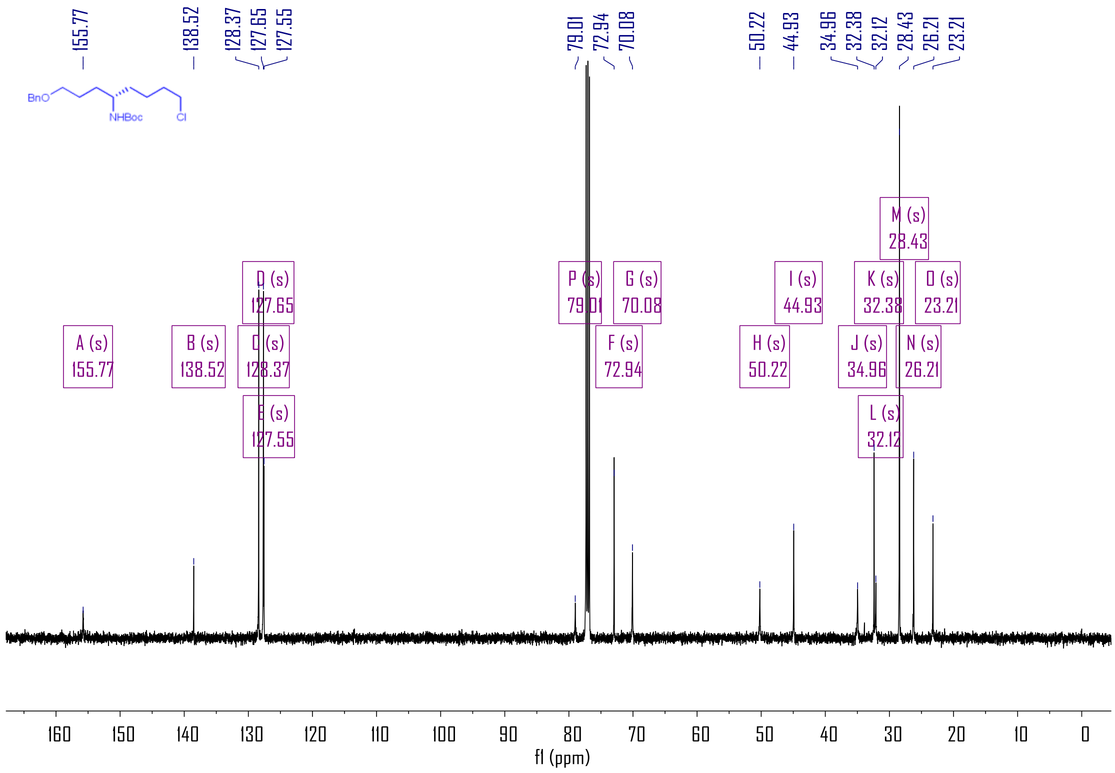

Supplementary Figure 316. ^{13}C NMR spectra for **107**

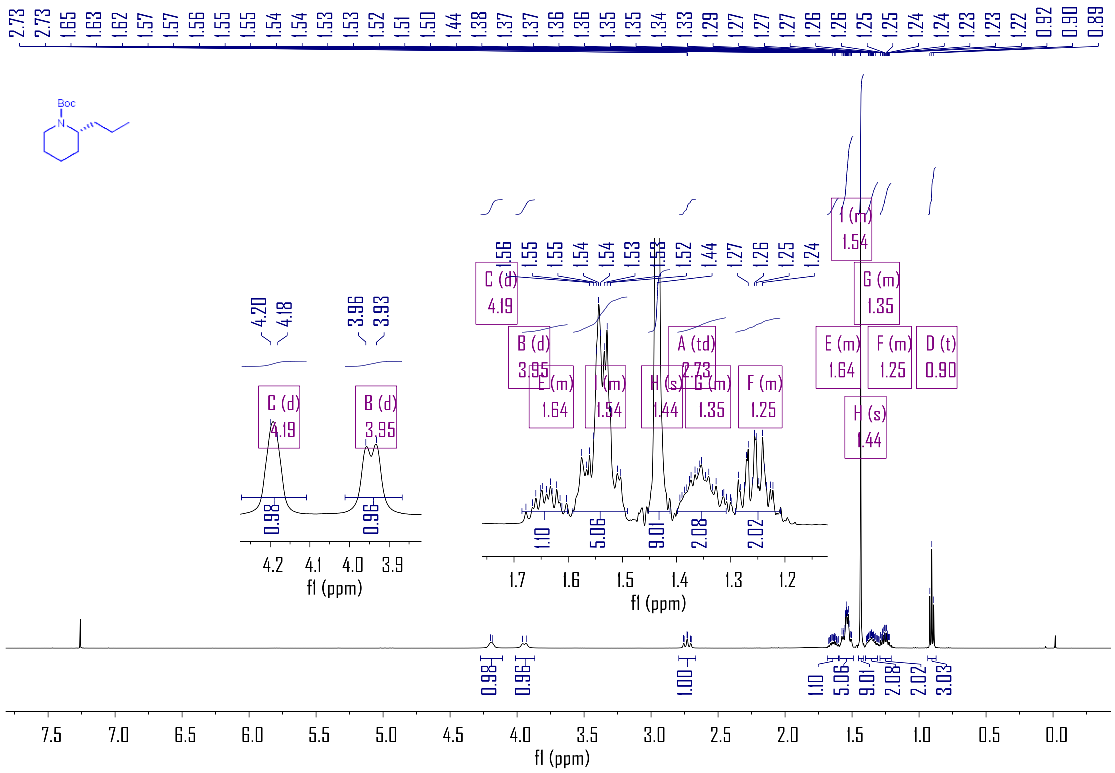

Supplementary Figure 317. ^{1}H NMR spectra for **108**

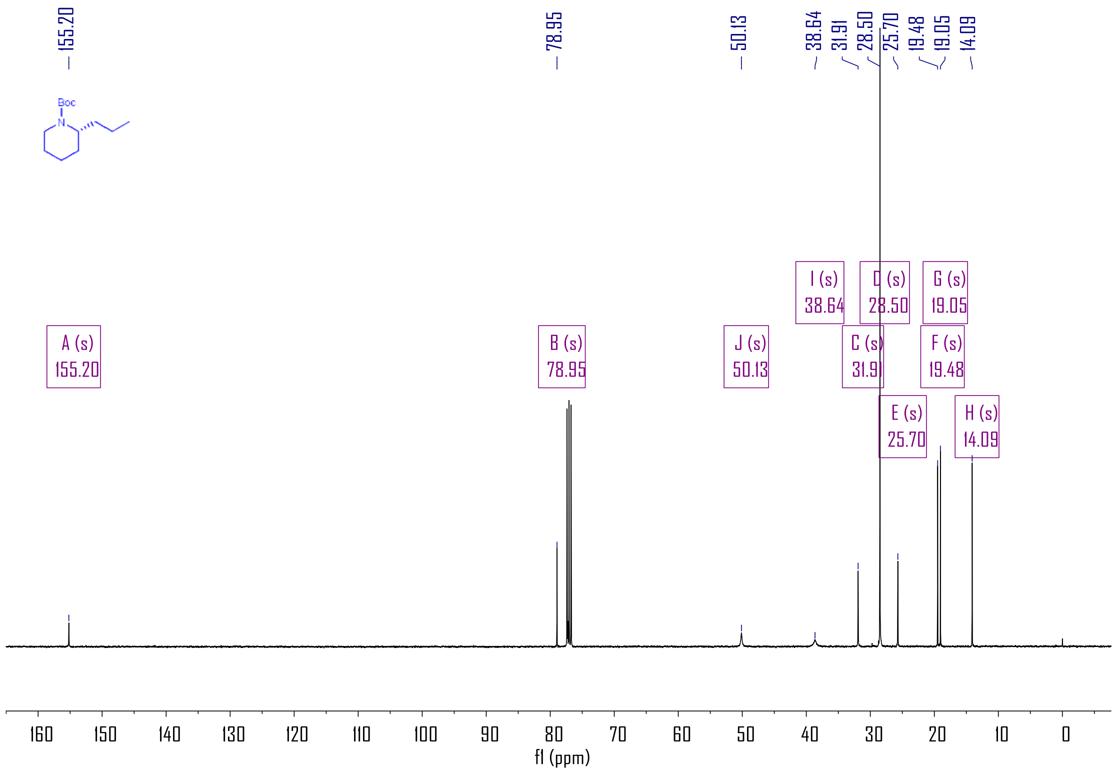

Supplementary Figure 318. ^{13}C NMR spectra for **108**

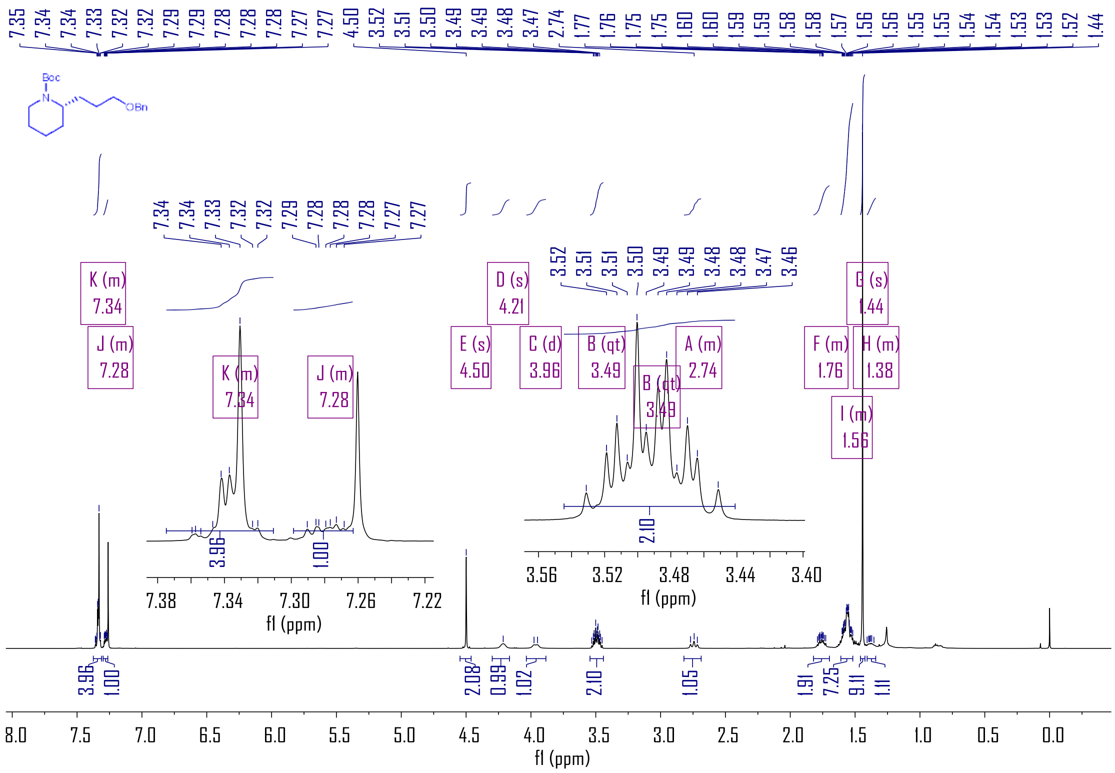

Supplementary Figure 319. ^{1}H NMR spectra for **109**

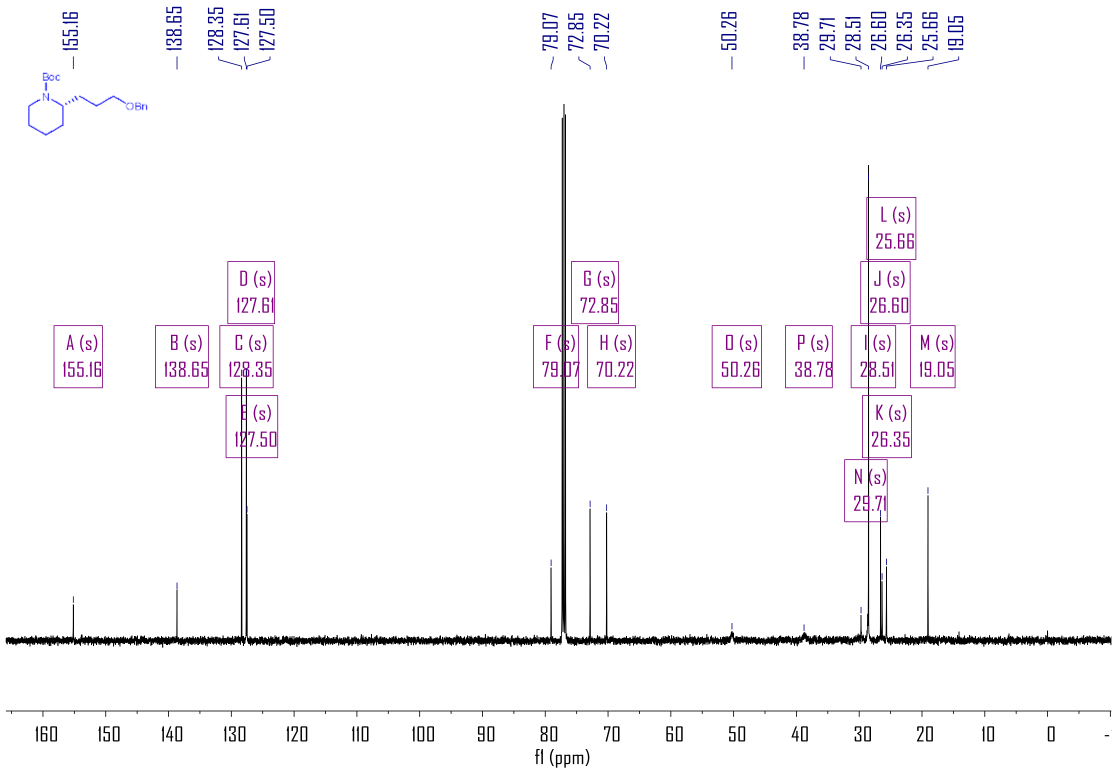

Supplementary Figure 320. ^{13}C NMR spectra for **109**

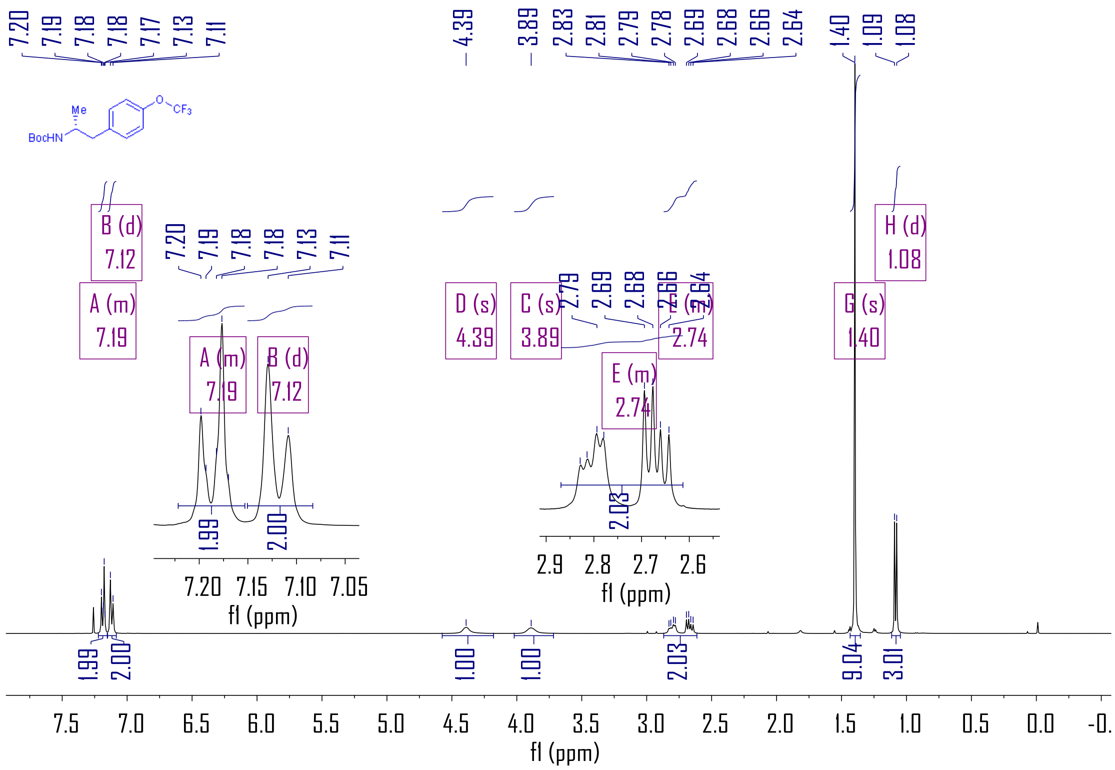

Supplementary Figure 321. ^{1}H NMR spectra for **111**

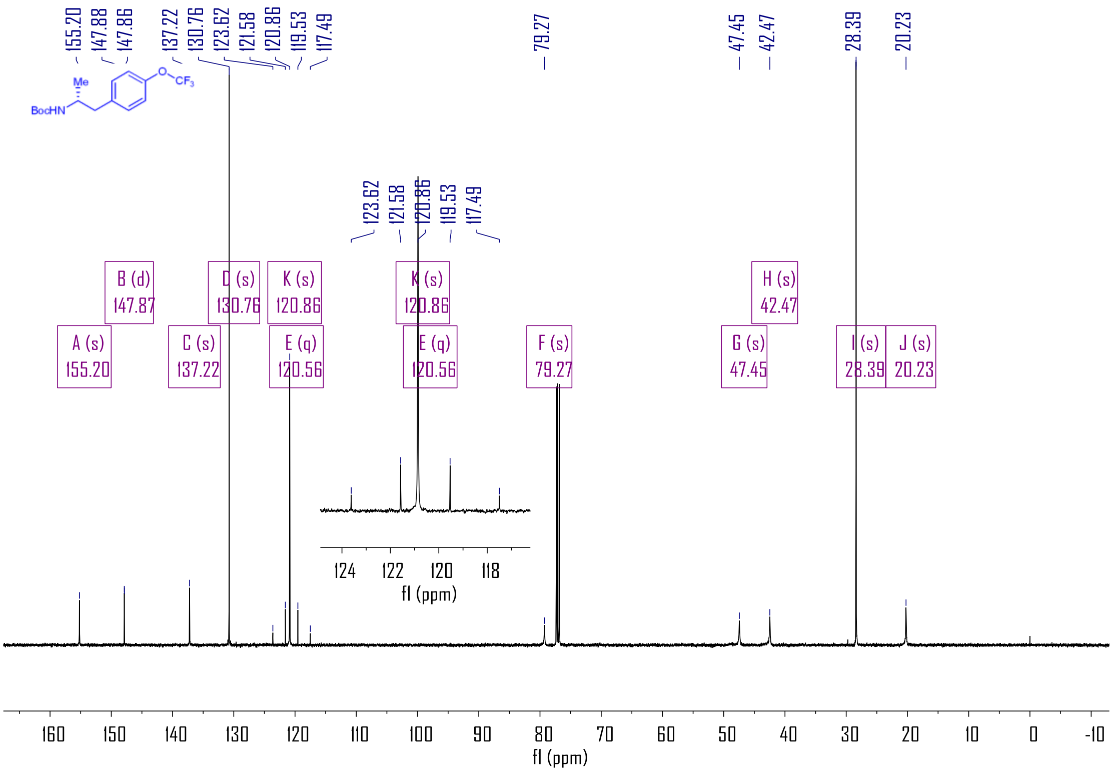

Supplementary Figure 322. ^{13}C NMR spectra for **111**

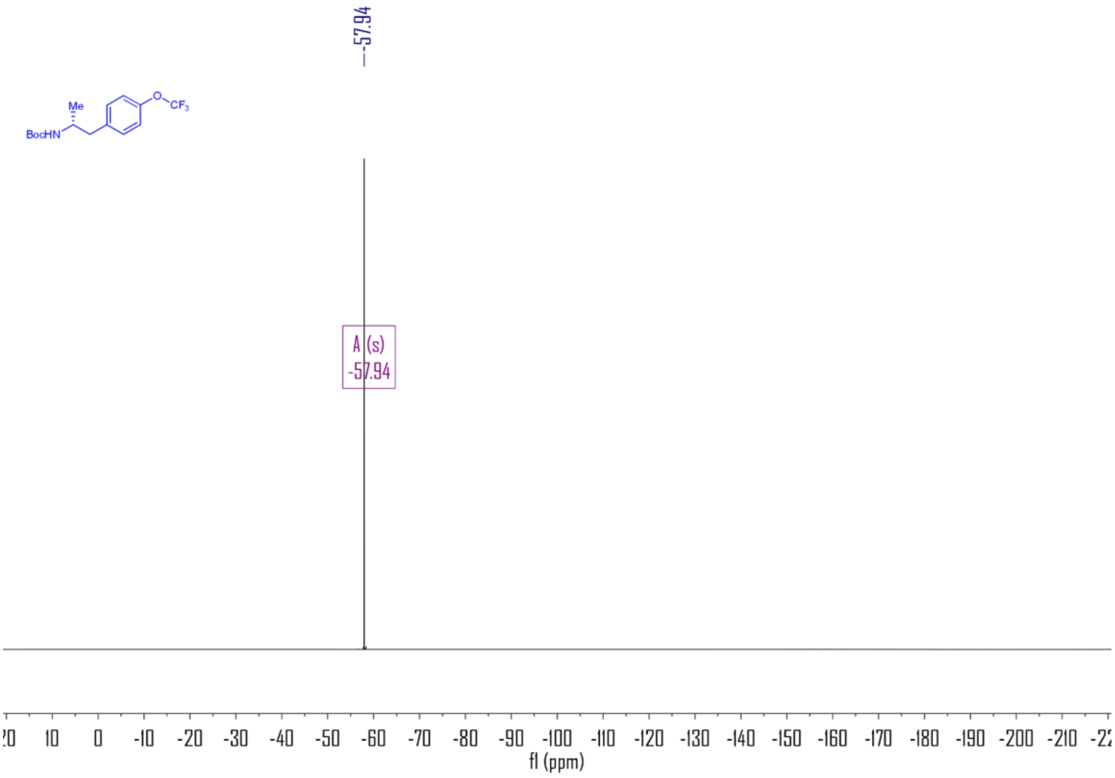

Supplementary Figure 323. ^{19}F NMR spectra for **111**

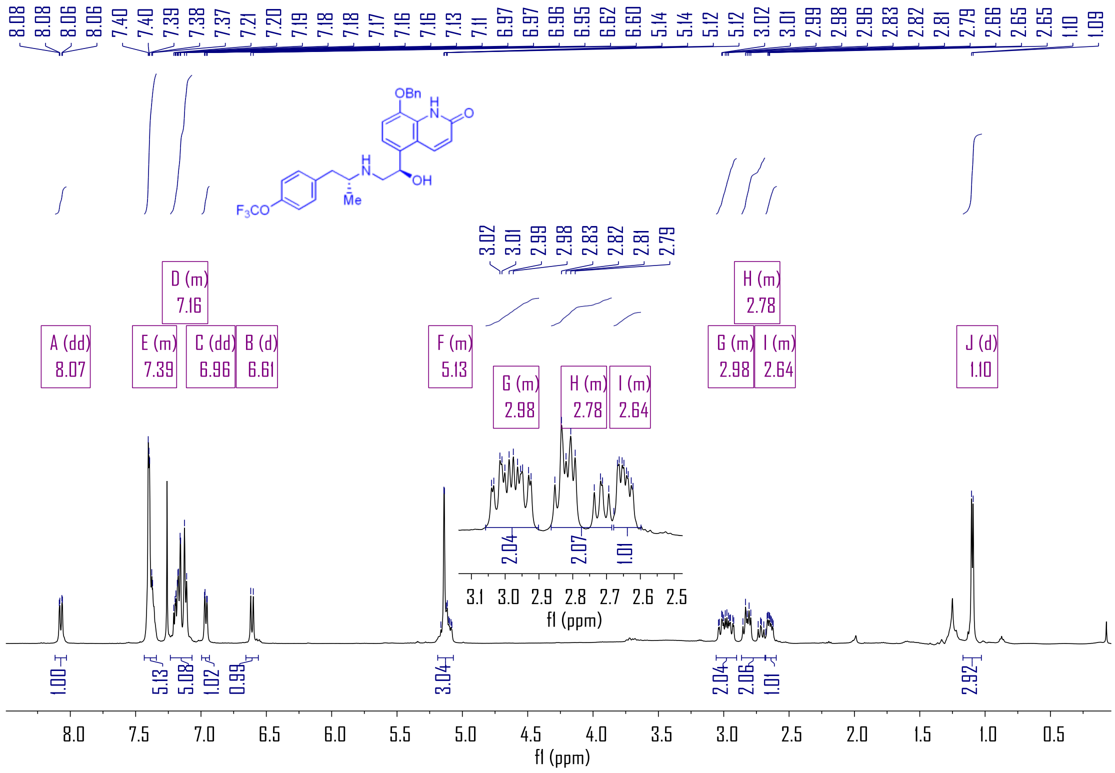

Supplementary Figure 324. ^{1}H NMR spectra for **113**

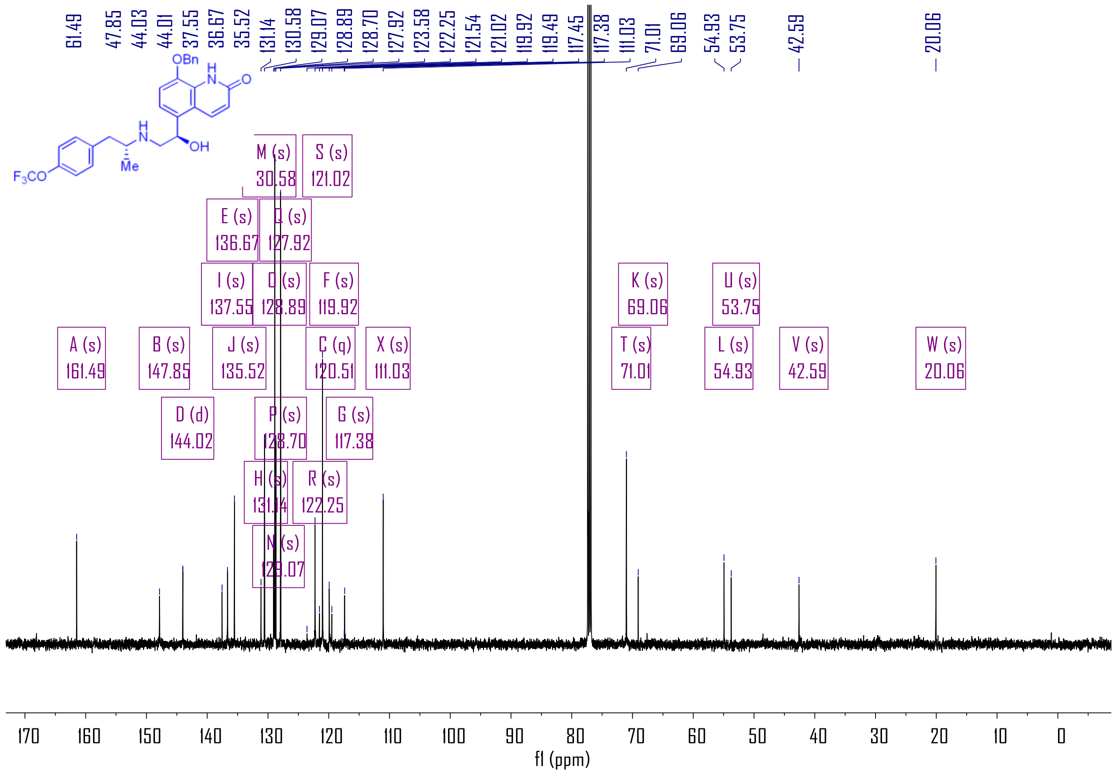

Supplementary Figure 325. ^{13}C NMR spectra for **113**

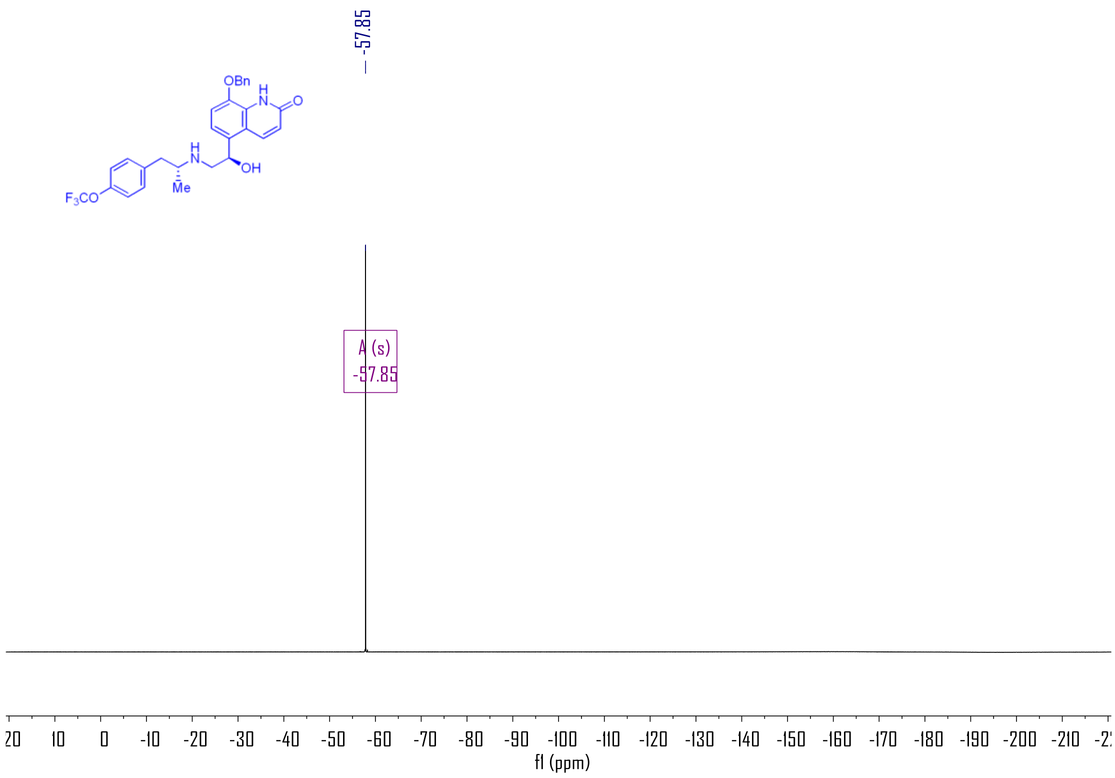

Supplementary Figure 326. ^{19}F NMR spectra for **113**

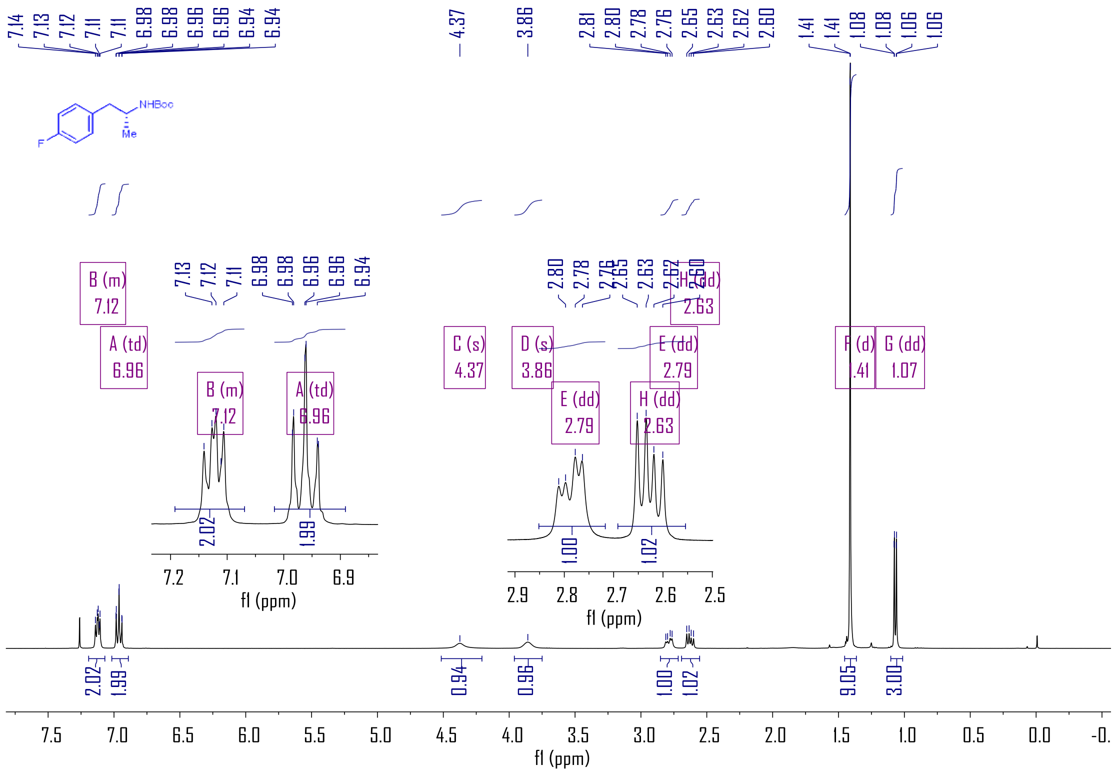

Supplementary Figure 327. ^{1}H NMR spectra for **114**

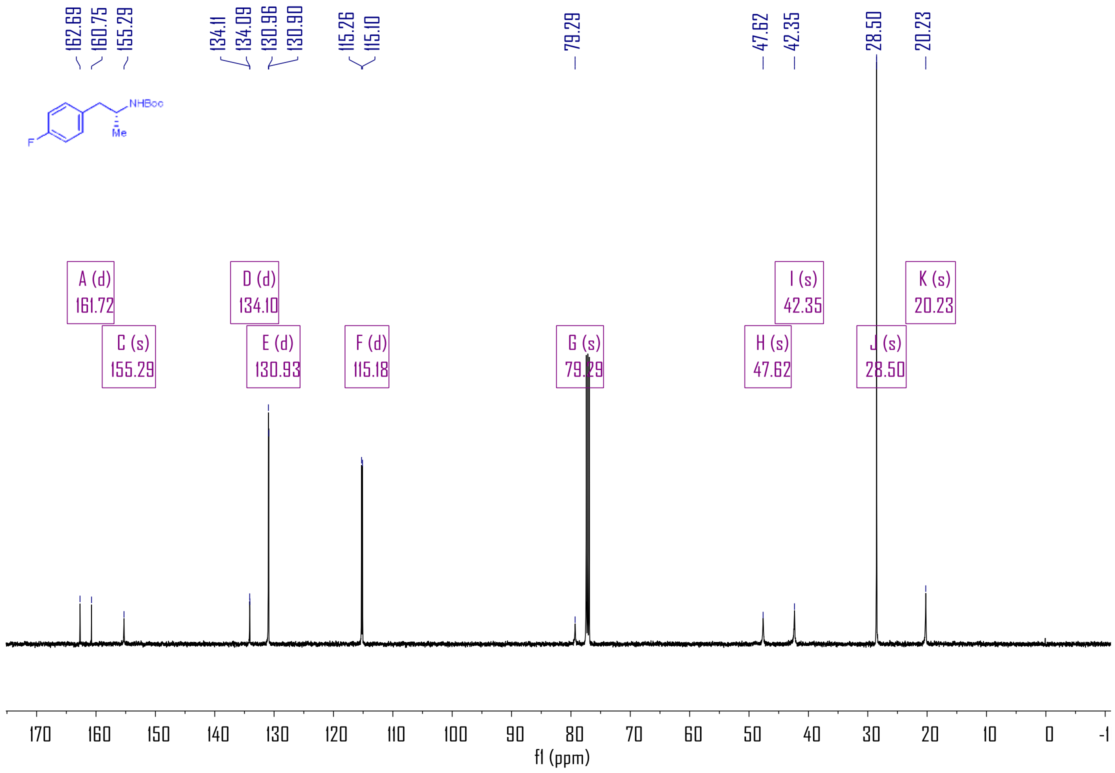

Supplementary Figure 328. ^{13}C NMR spectra for **114**

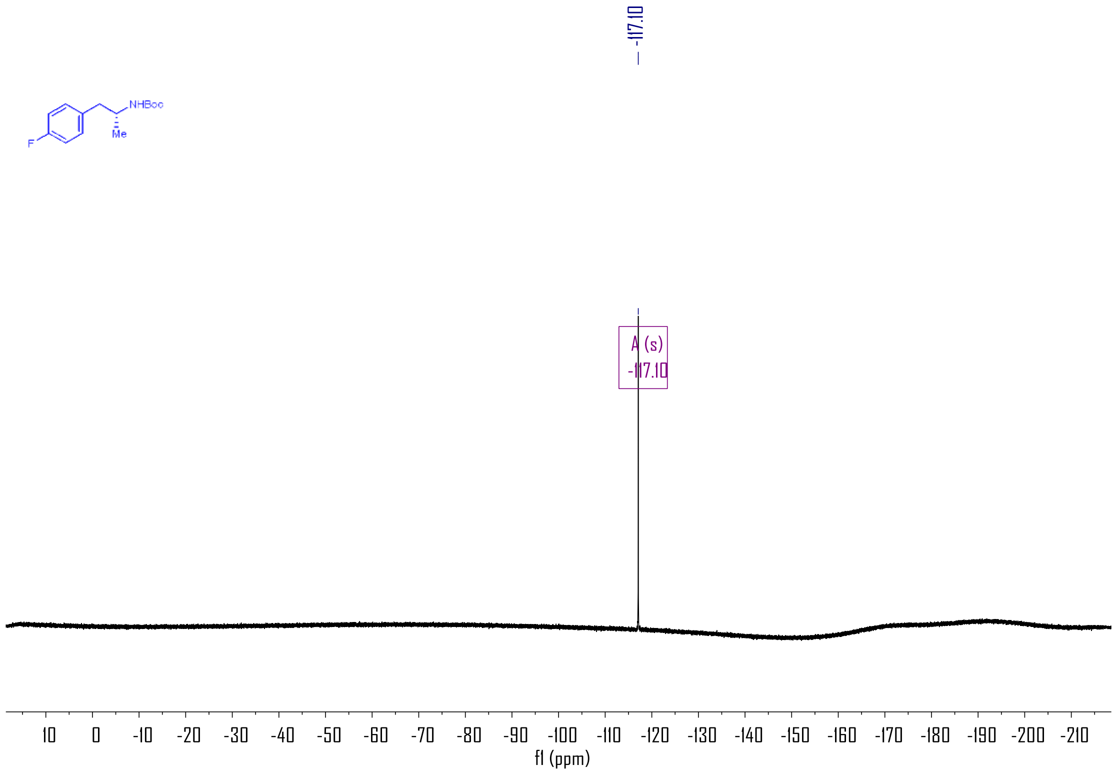

Supplementary Figure 329. ^{19}F NMR spectra for **114**

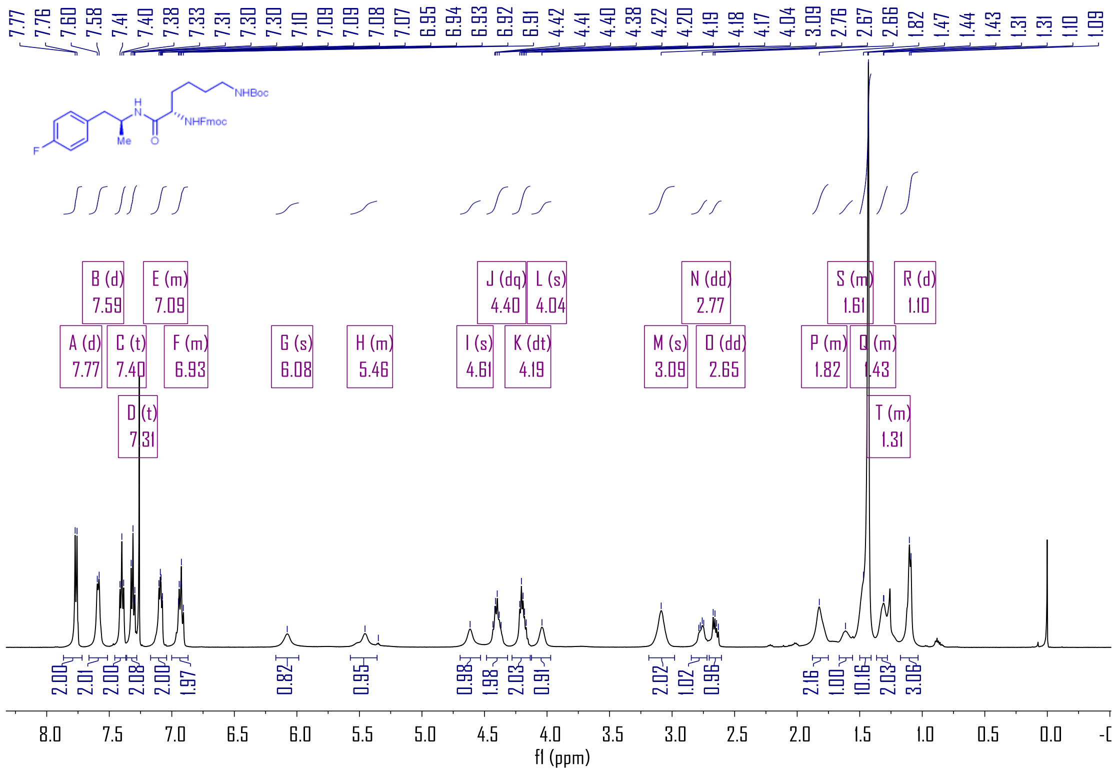

Supplementary Figure 330. ^{1}H NMR spectra for **115**

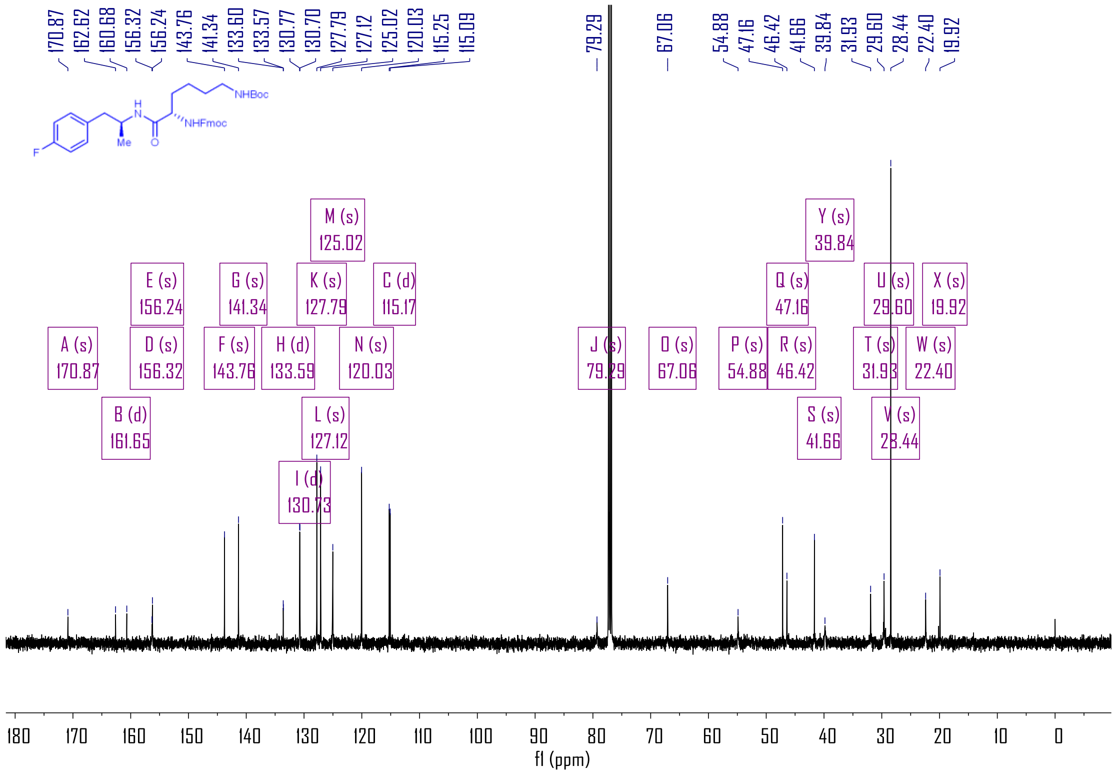

Supplementary Figure 331. ^{13}C NMR spectra for **115**

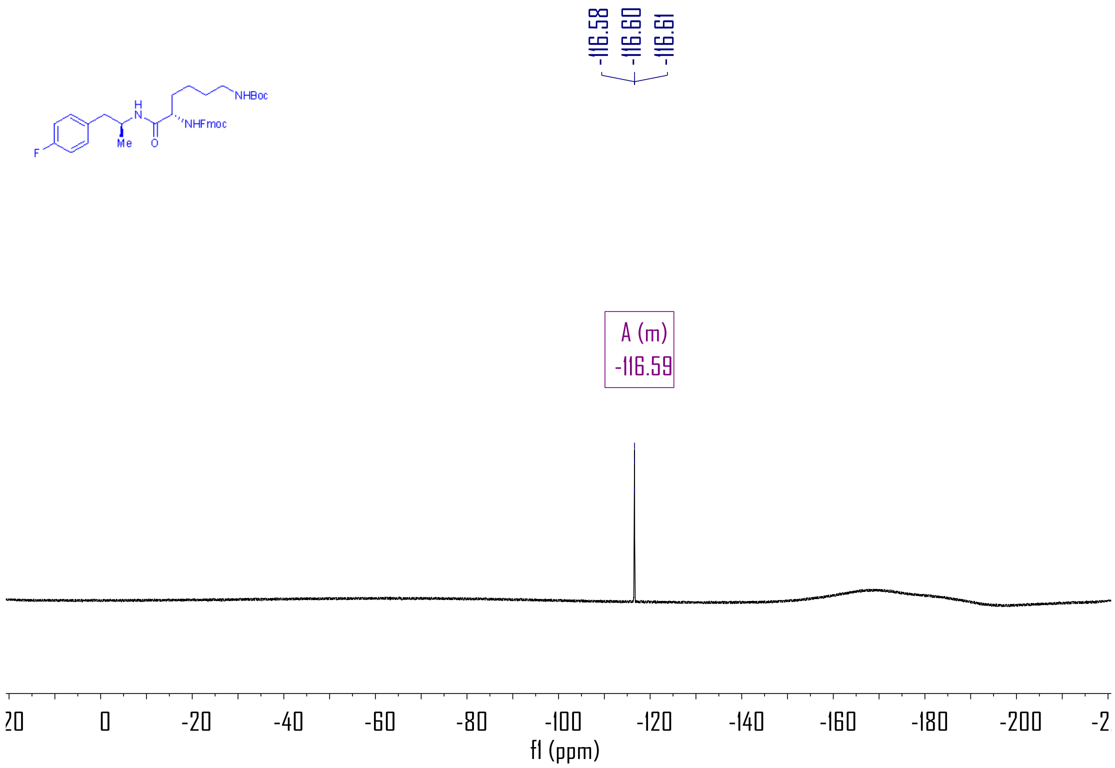

Supplementary Figure 332. ^{19}F NMR spectra for **115**

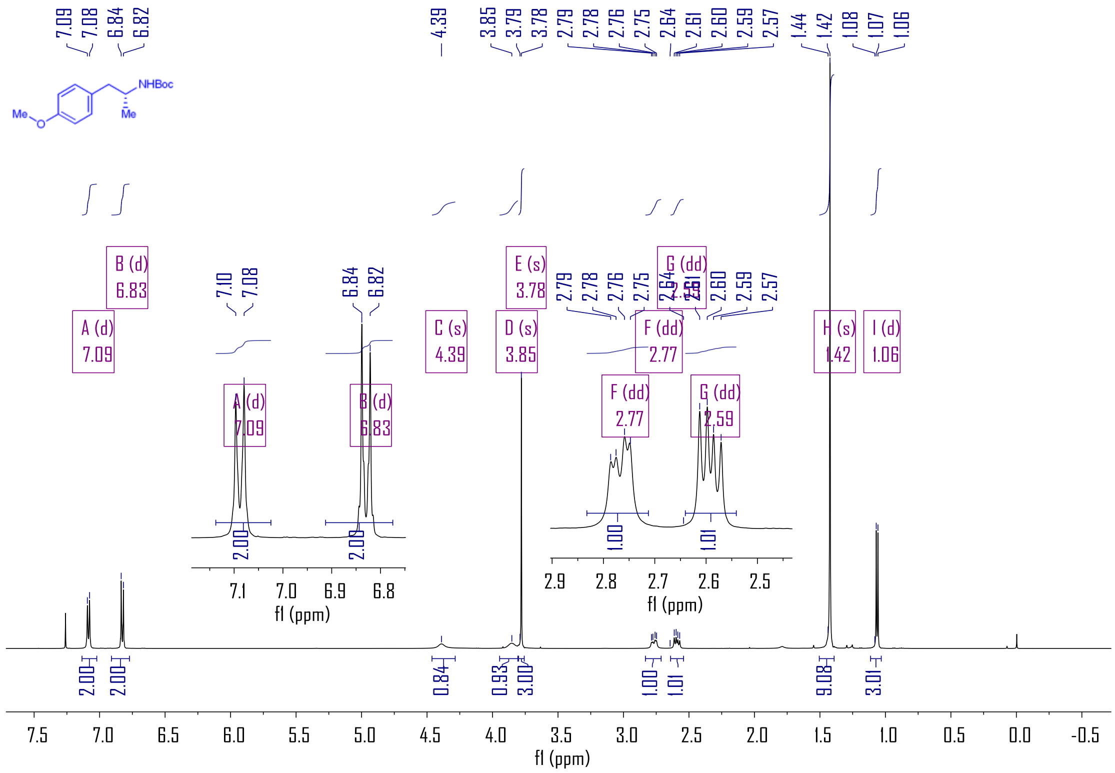

Supplementary Figure 333. ^{1}H NMR spectra for **116**

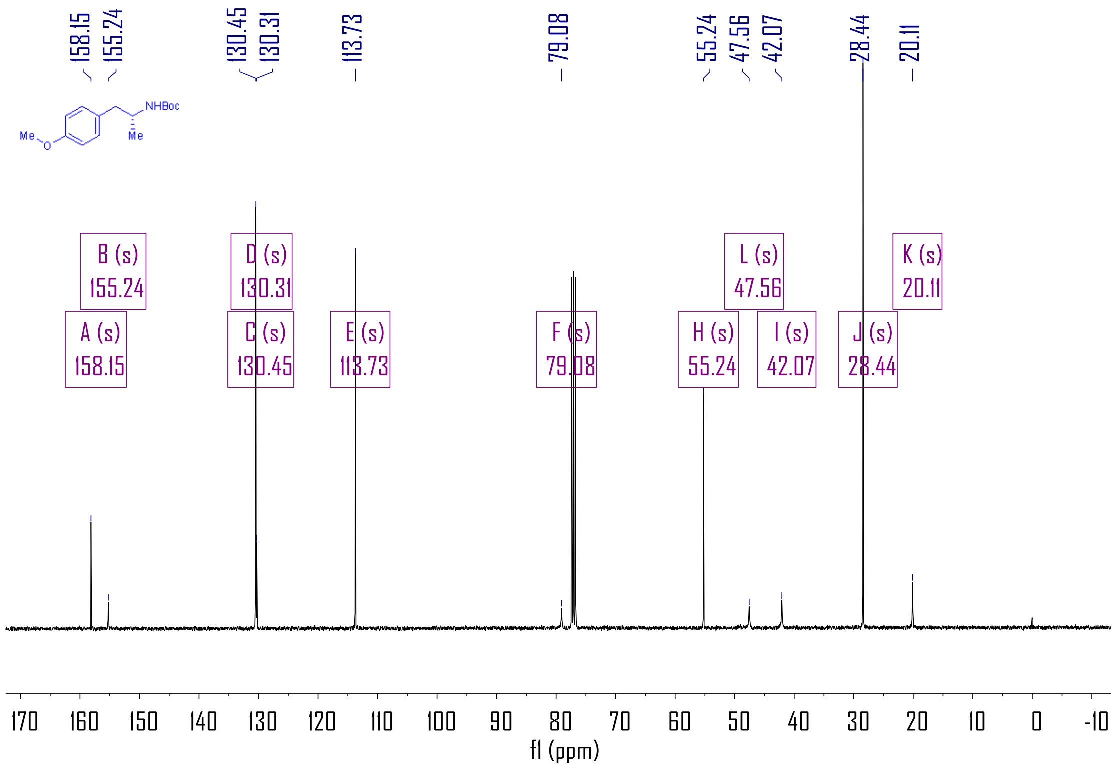

Supplementary Figure 334. ^{13}C NMR spectra for **116**

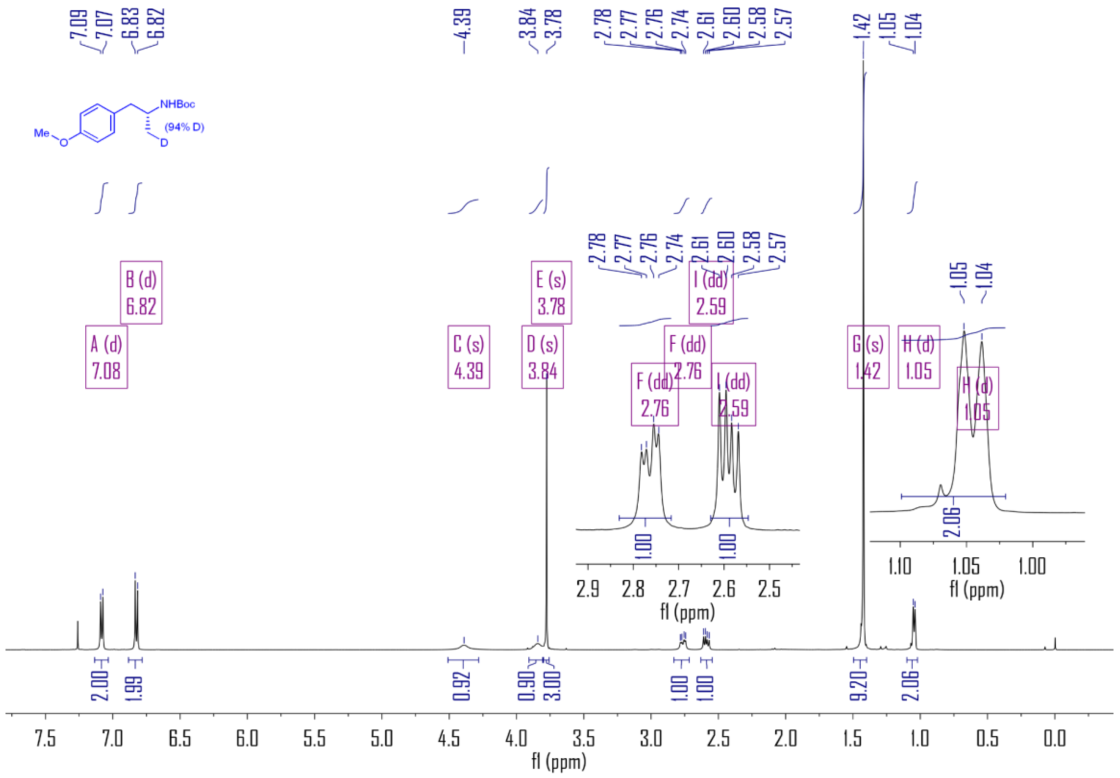

Supplementary Figure 335. ^{1}H NMR spectra for **117**

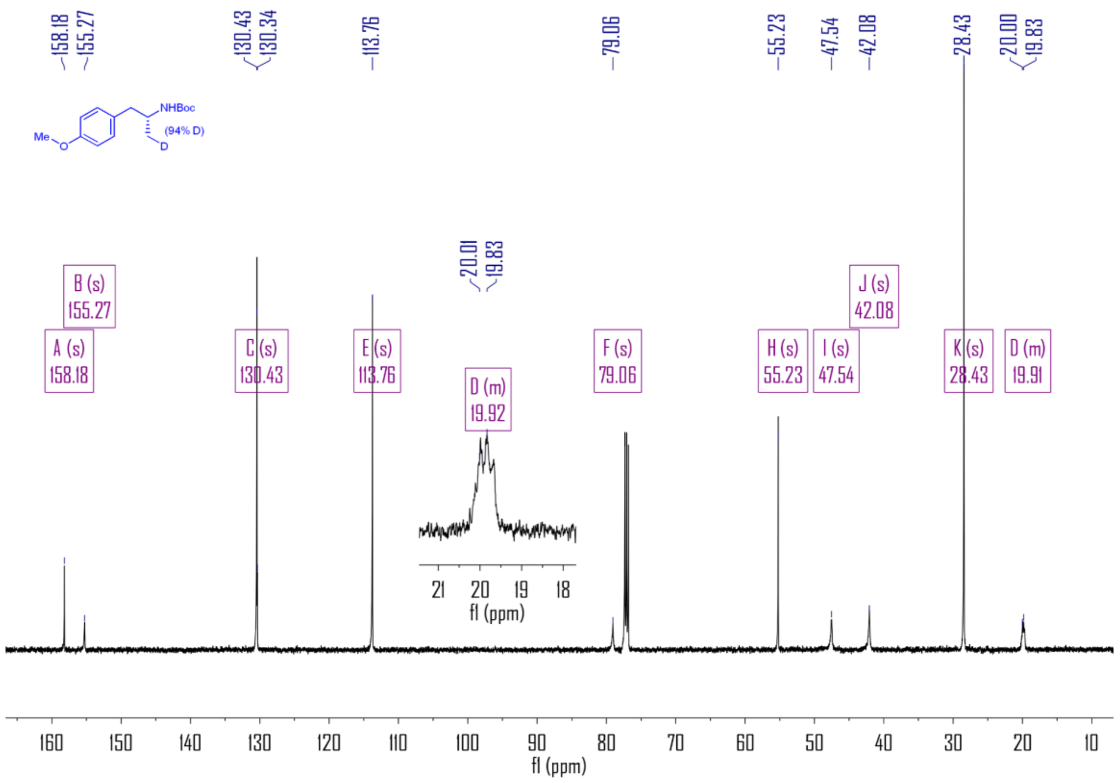

Supplementary Figure 336. ^{13}C NMR spectra for **117**

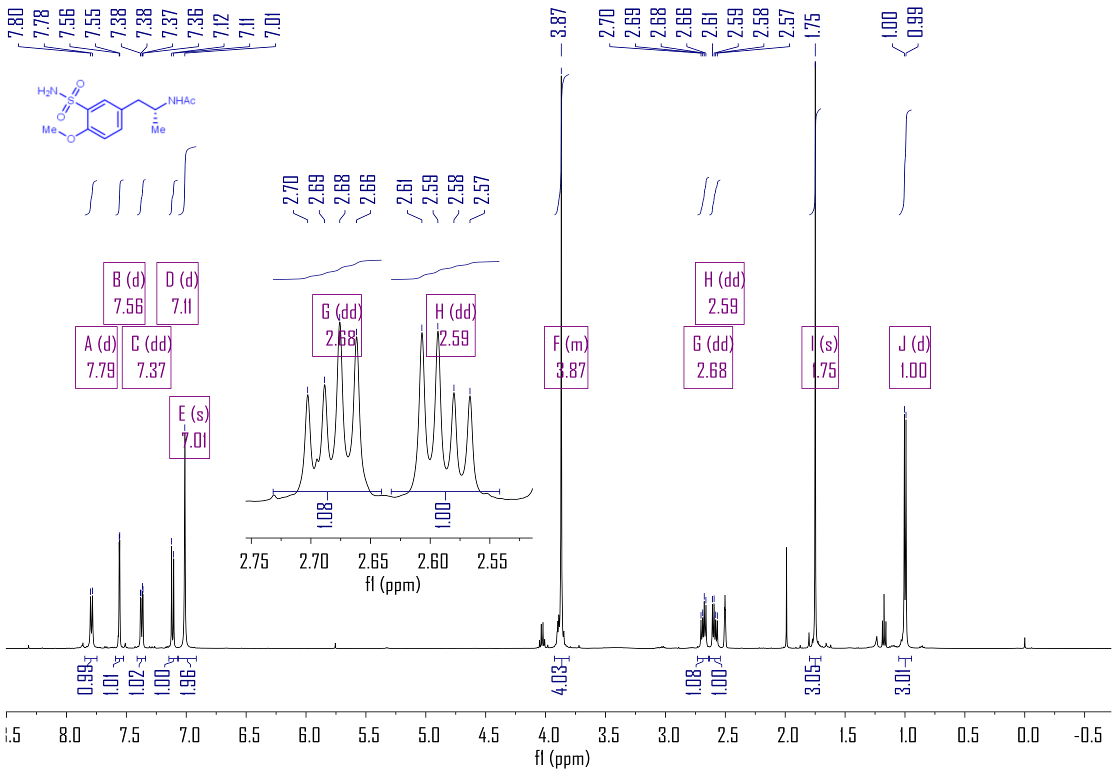

Supplementary Figure 337. ^{1}H NMR spectra for **118**

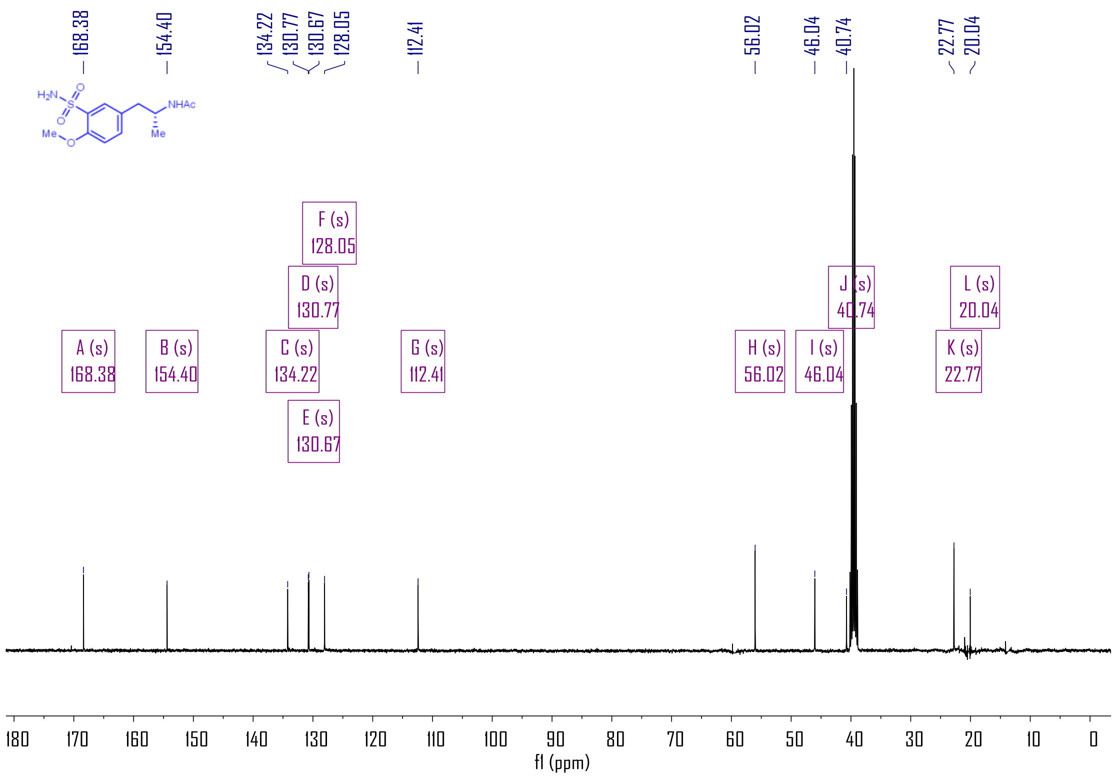

Supplementary Figure 338. ^{13}C NMR spectra for **118**

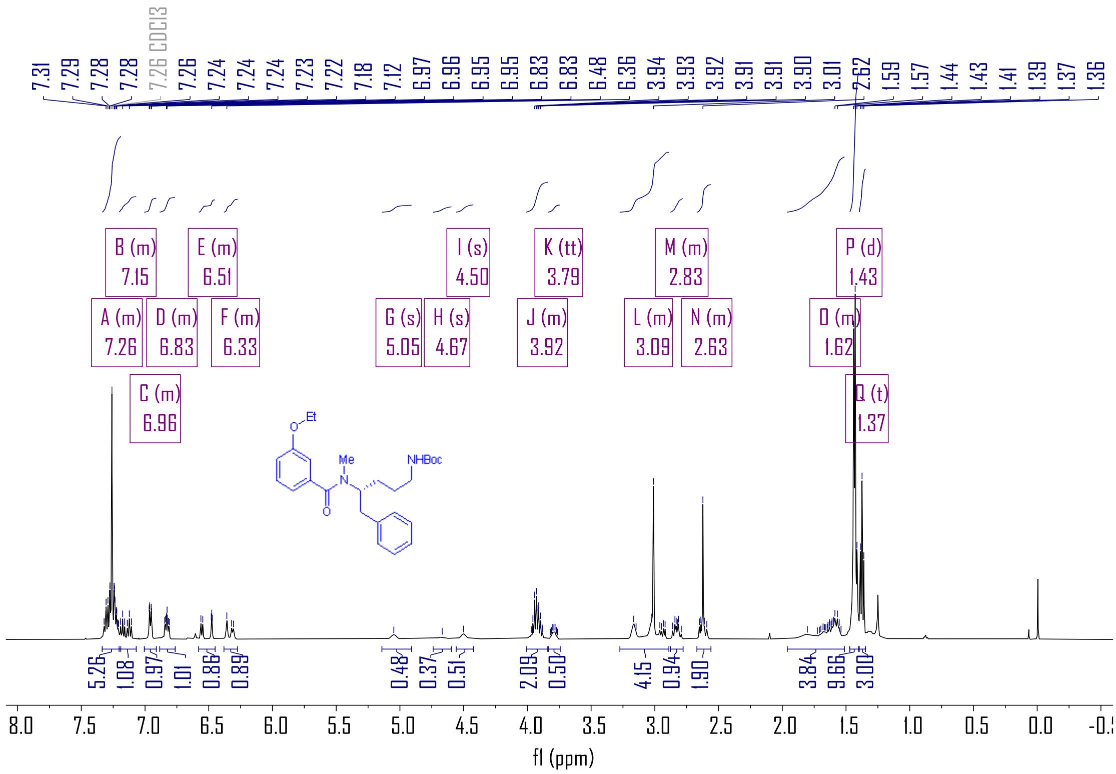

Supplementary Figure 339. ^{1}H NMR spectra for **121**

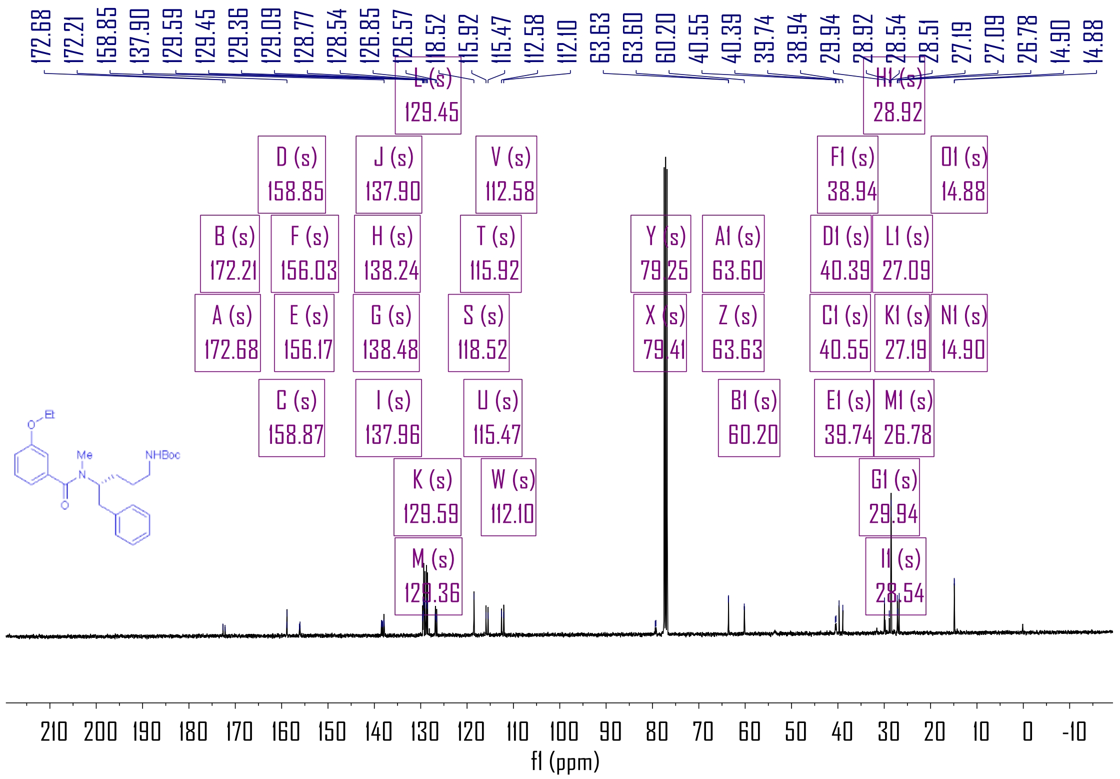

Supplementary Figure 340. ^{13}C NMR spectra for **121**

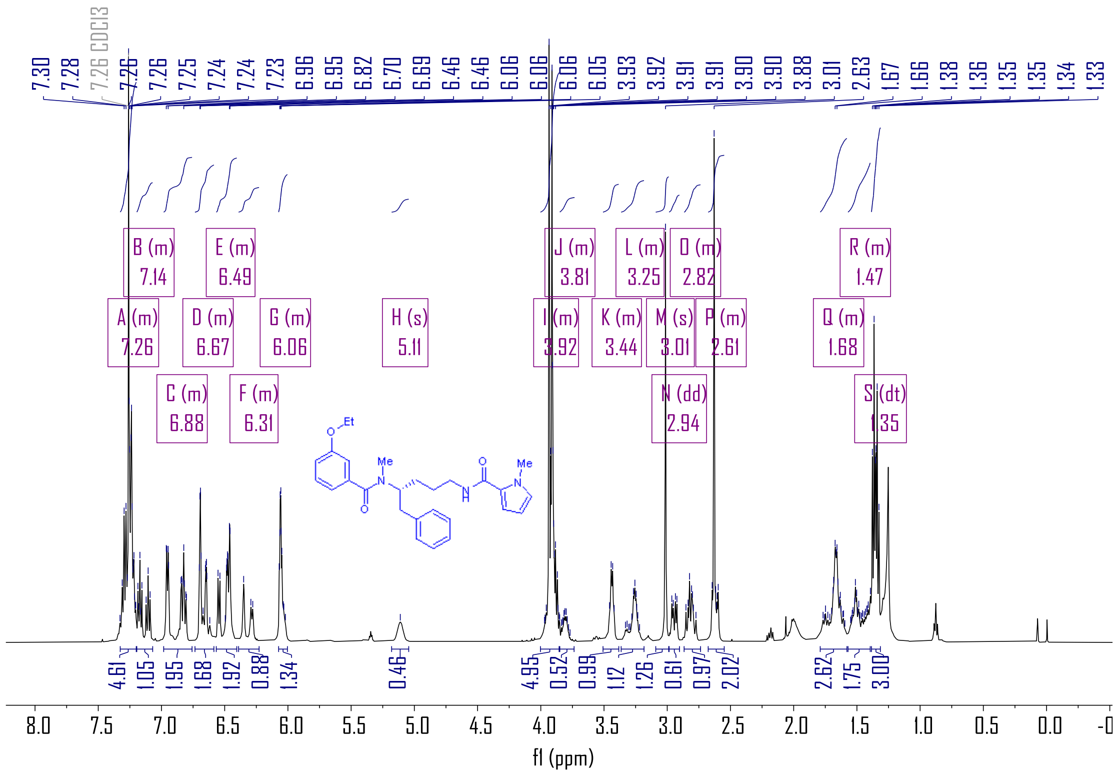

Supplementary Figure 341. ^{1}H NMR spectra for **122**

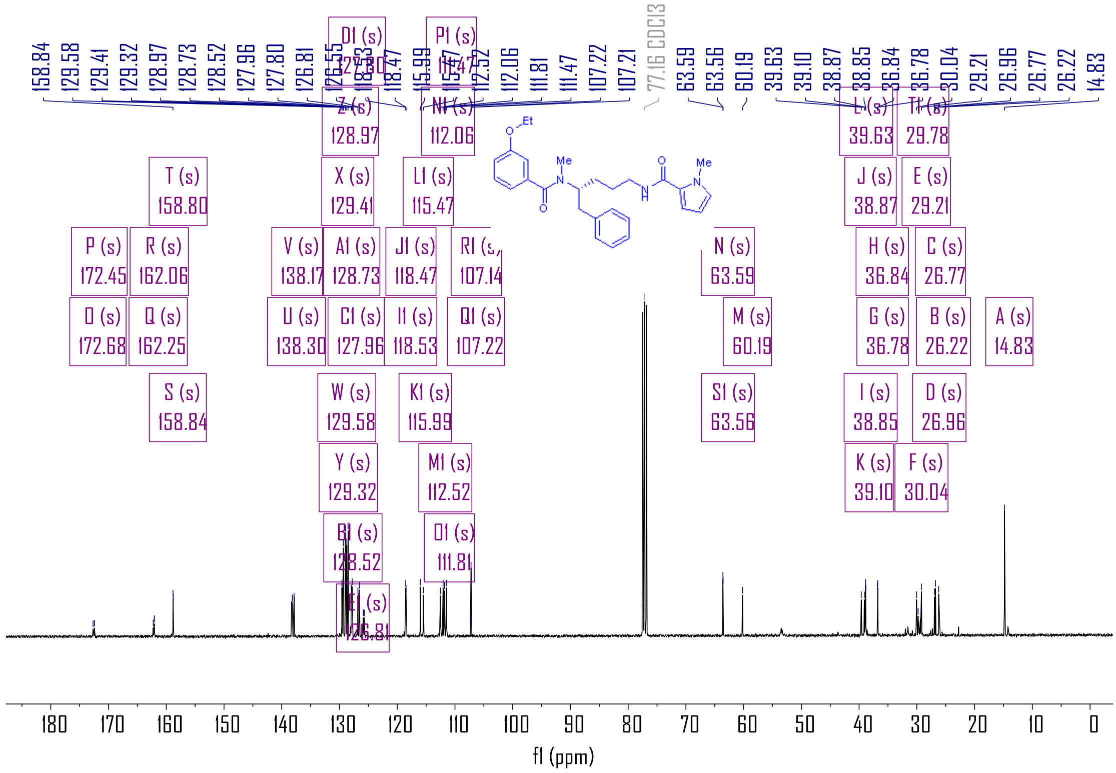

Supplementary Figure 342. ^{13}C NMR spectra for **122**

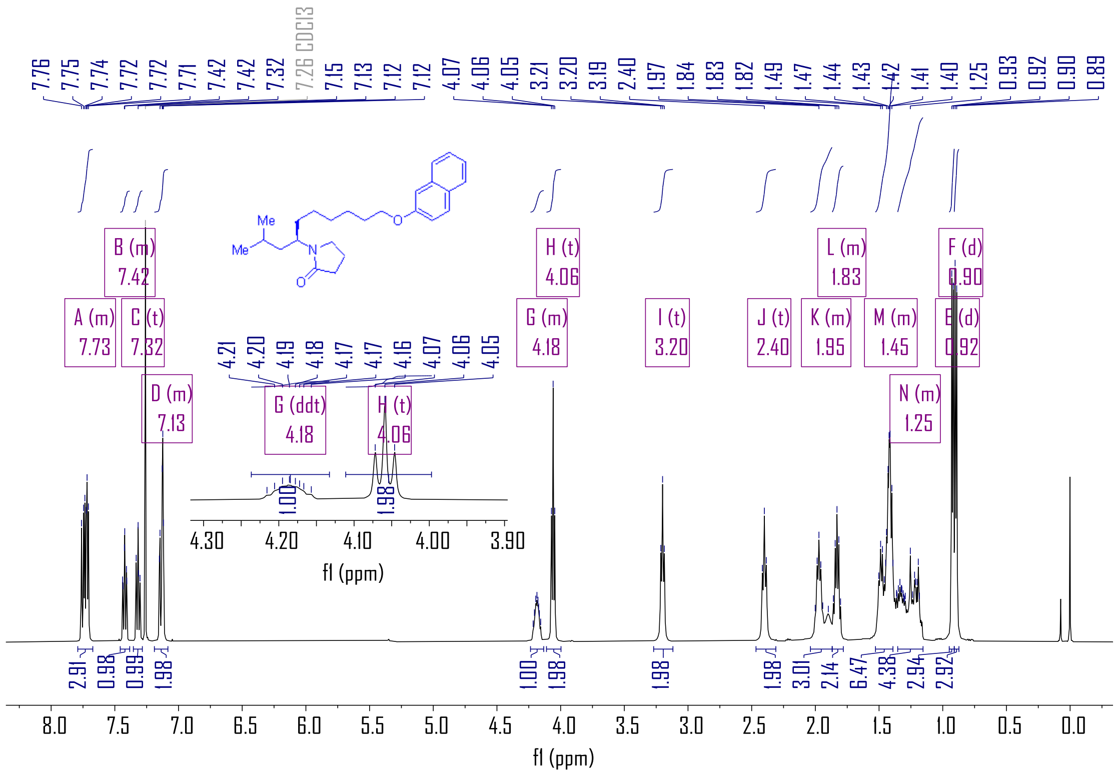

Supplementary Figure 343. ^{1}H NMR spectra for **CP1**

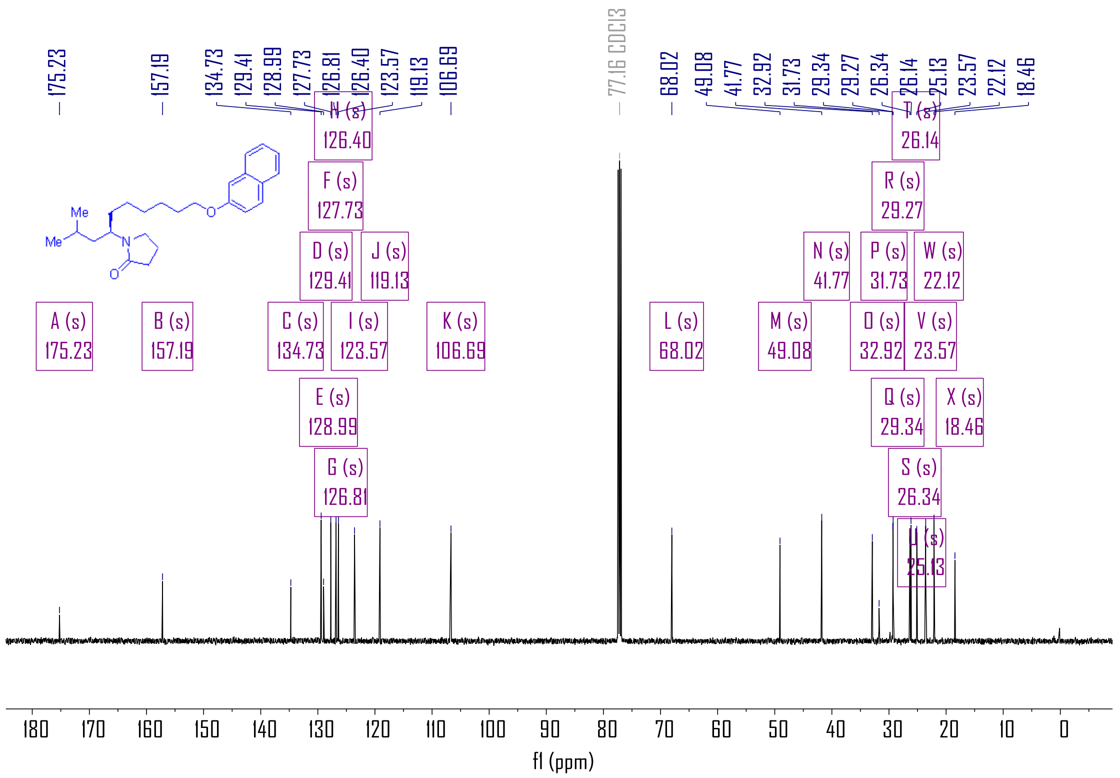

Supplementary Figure 344. ^{13}C NMR spectra for **CP1**

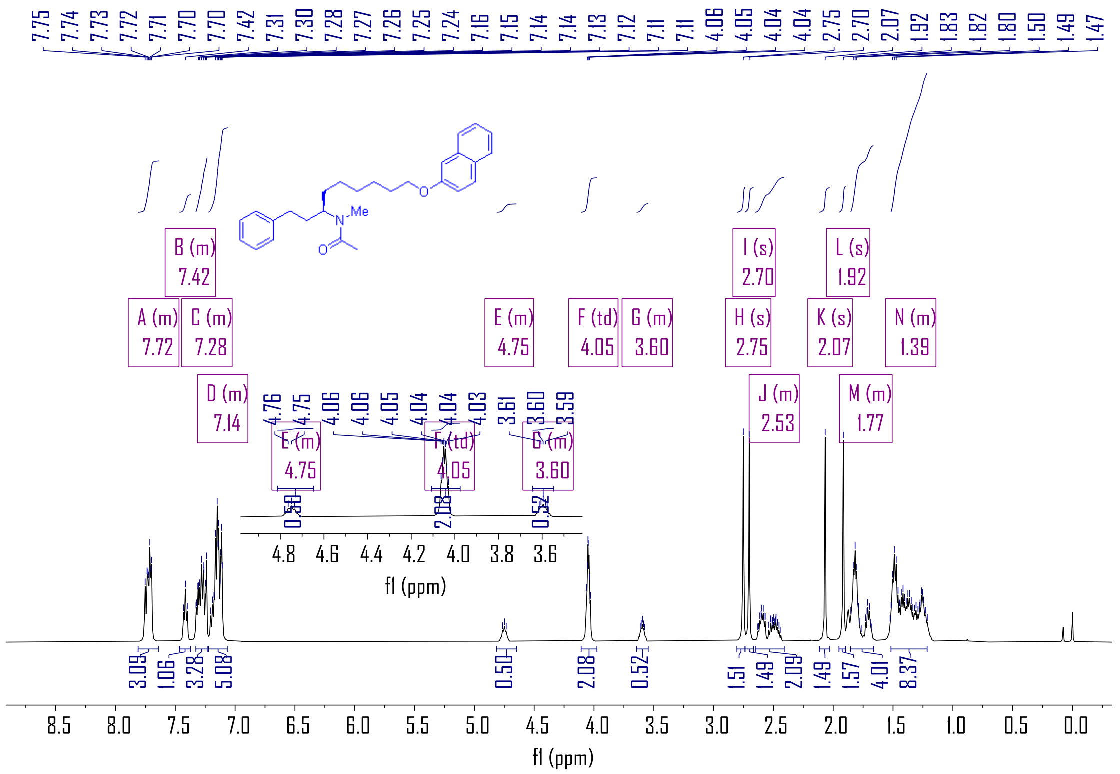

Supplementary Figure 345. ^{1}H NMR spectra for **CP2**

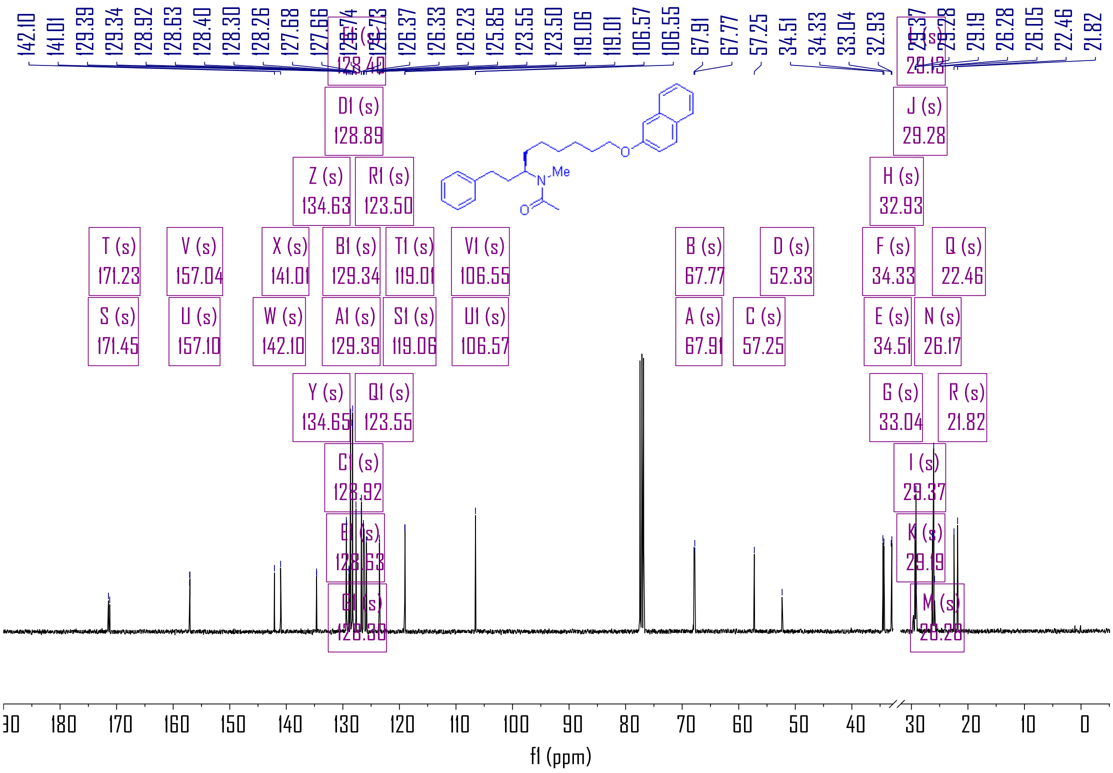

Supplementary Figure 346. ^{13}C NMR spectra for **CP2**

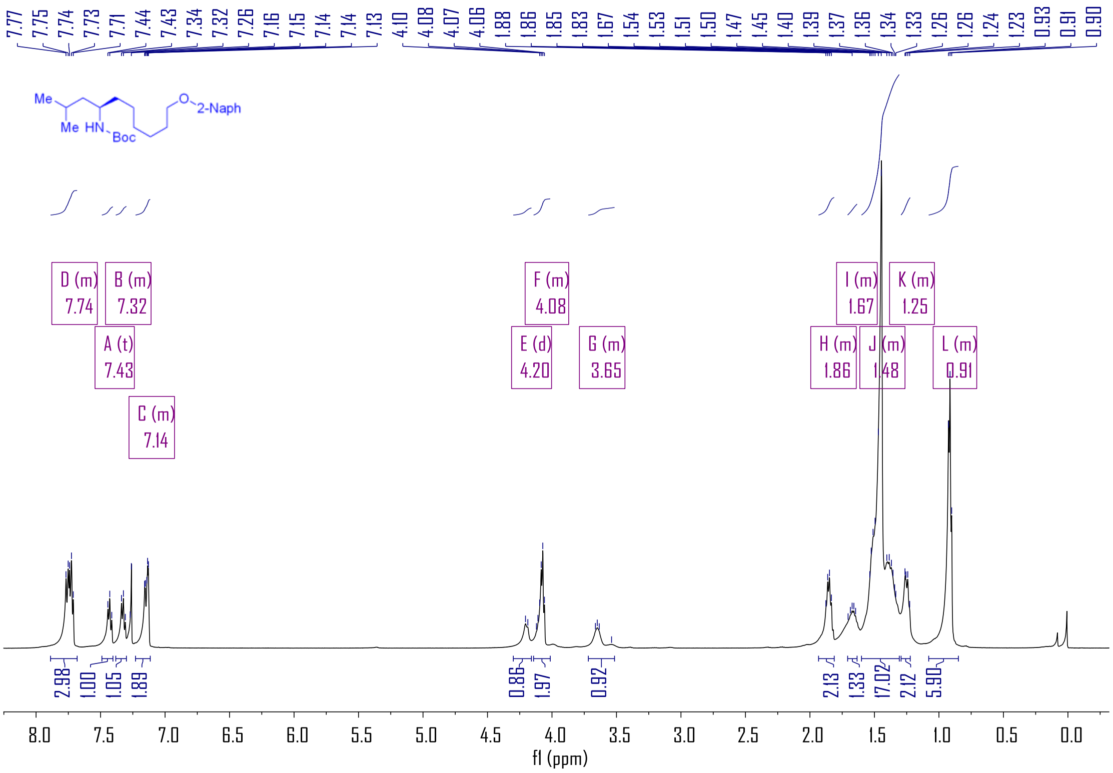

Supplementary Figure 347. ^{1}H NMR spectra for **CP3**

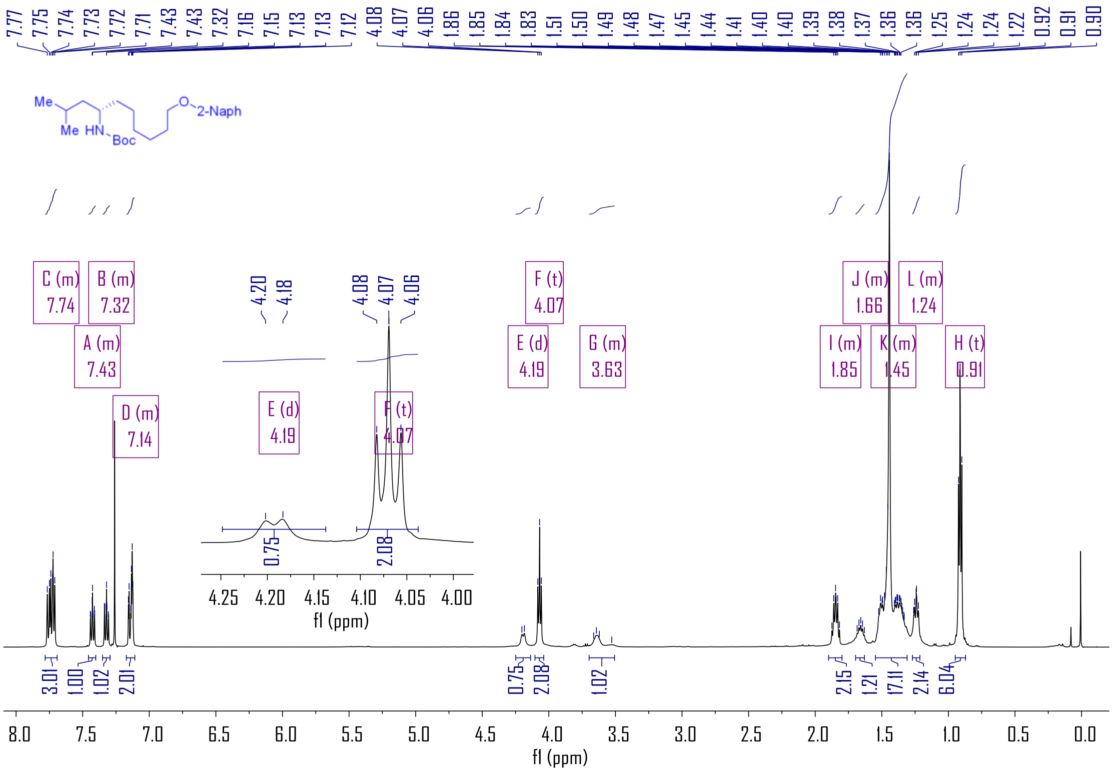

Supplementary Figure 348. ^{1}H NMR spectra for **46-b**

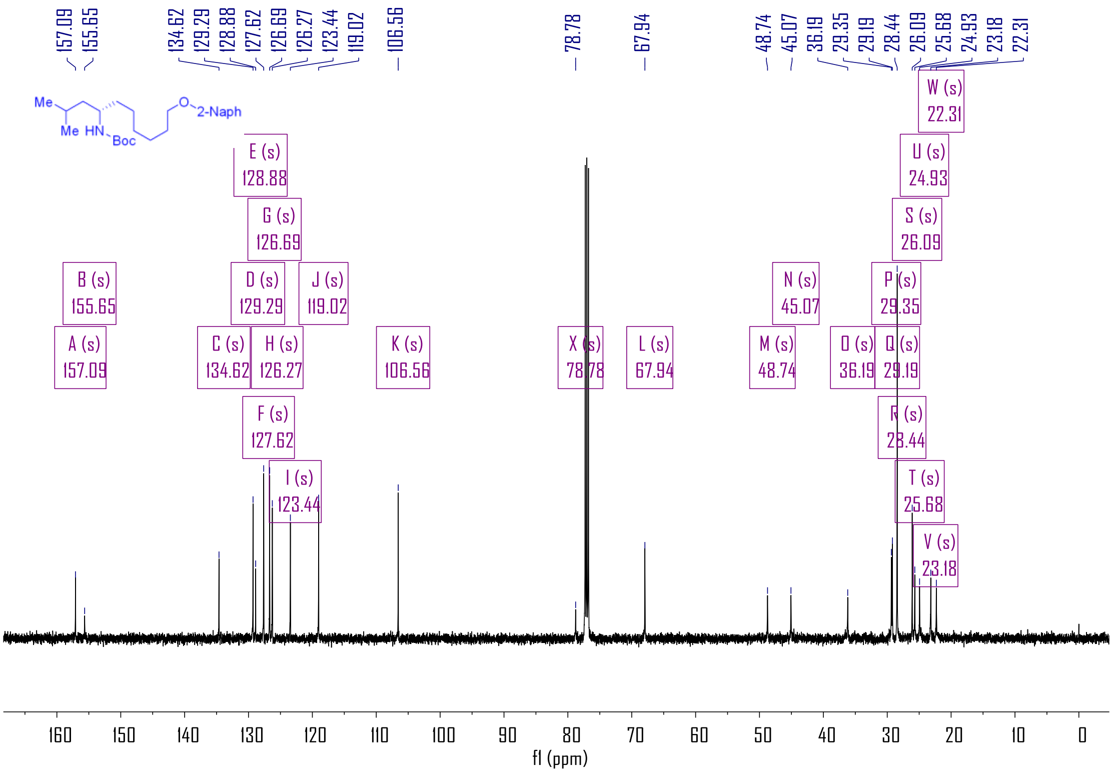

Supplementary Figure 349. ^{13}C NMR spectra for **46-b**

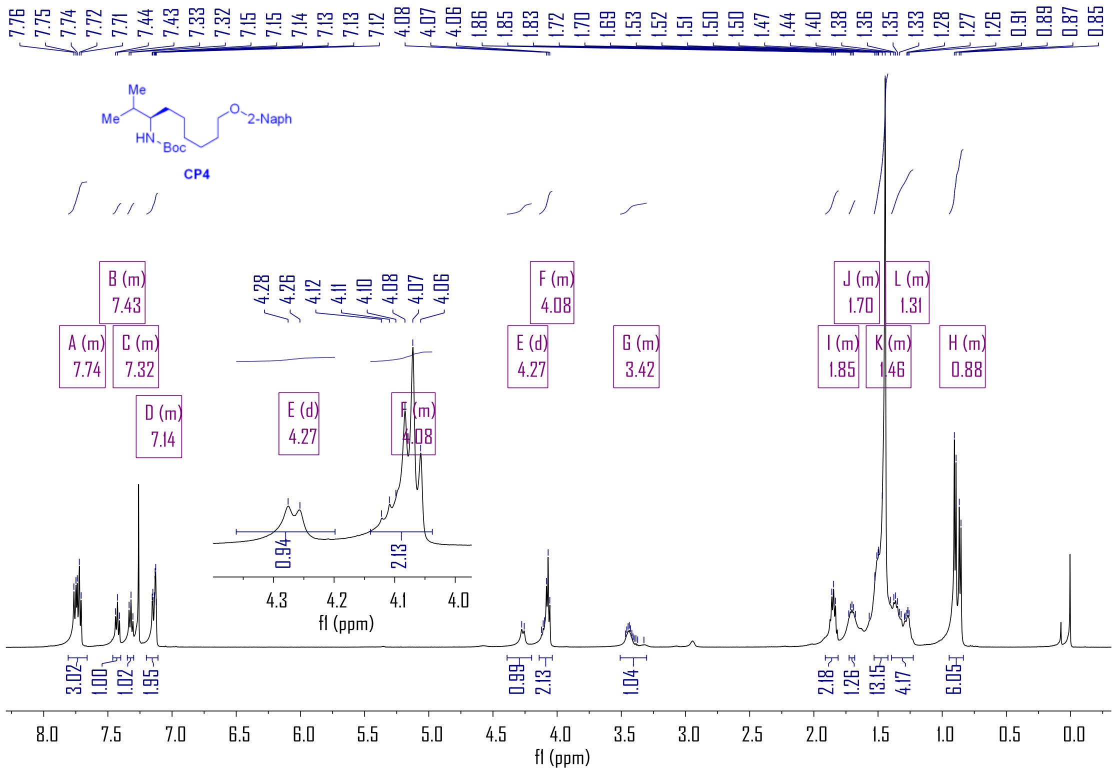

Supplementary Figure 350. ^{1}H NMR spectra for **CP4**

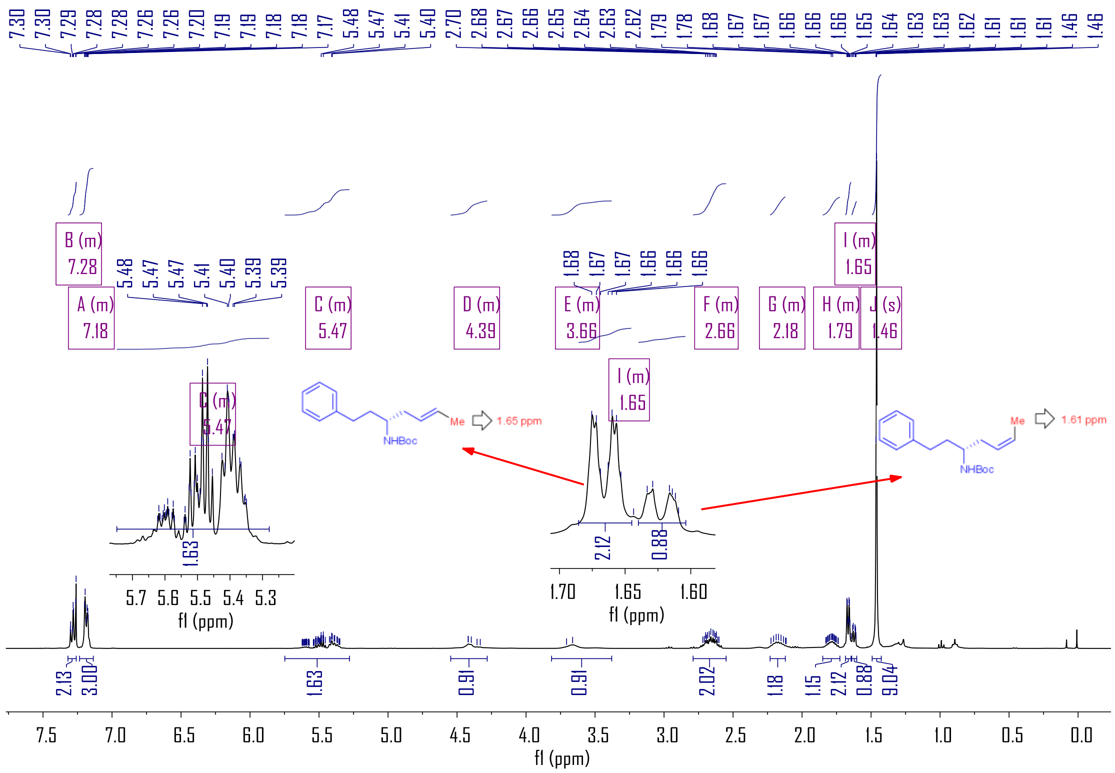

Supplementary Figure 351. ^{1}H NMR spectra for **70**

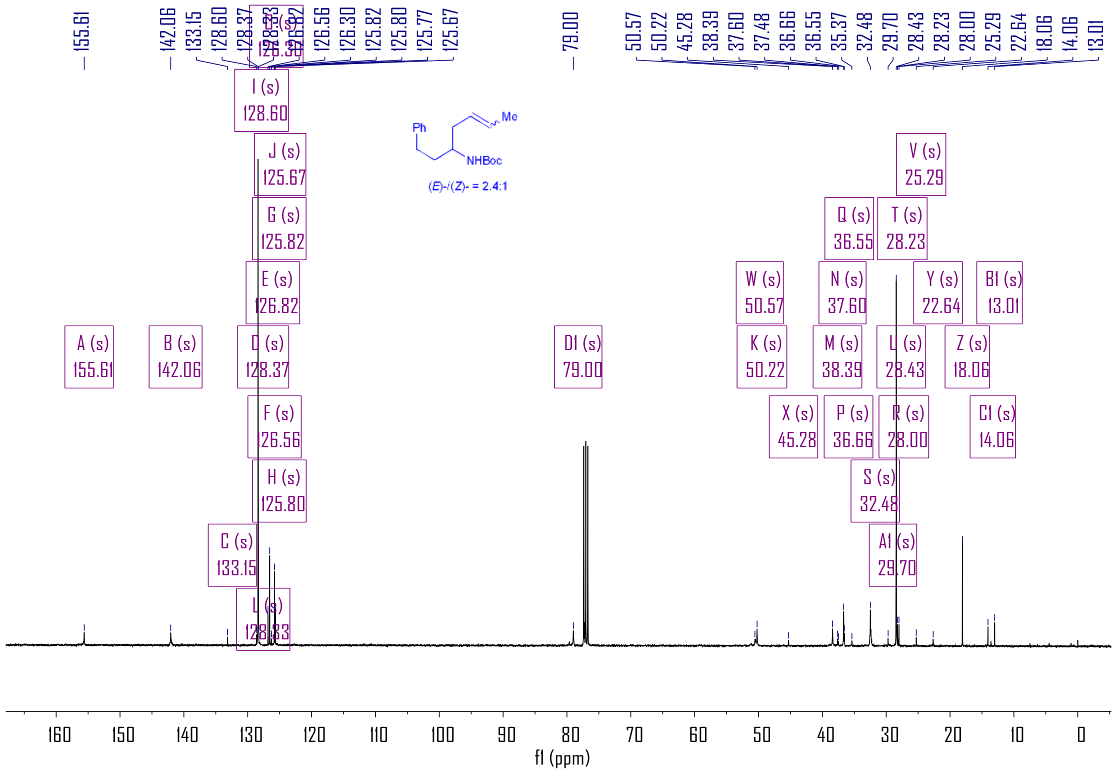

Supplementary Figure 352. ^{13}C NMR spectra for **70**

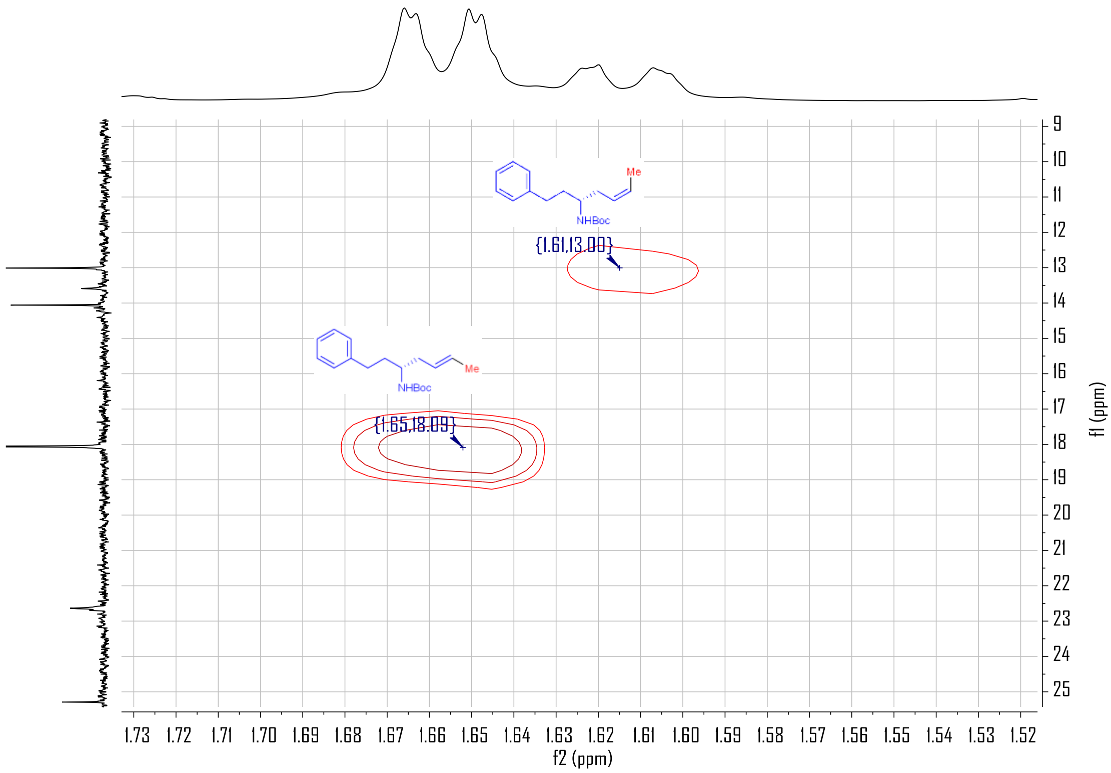

Supplementary Figure 353. HSQC spectra for **70**

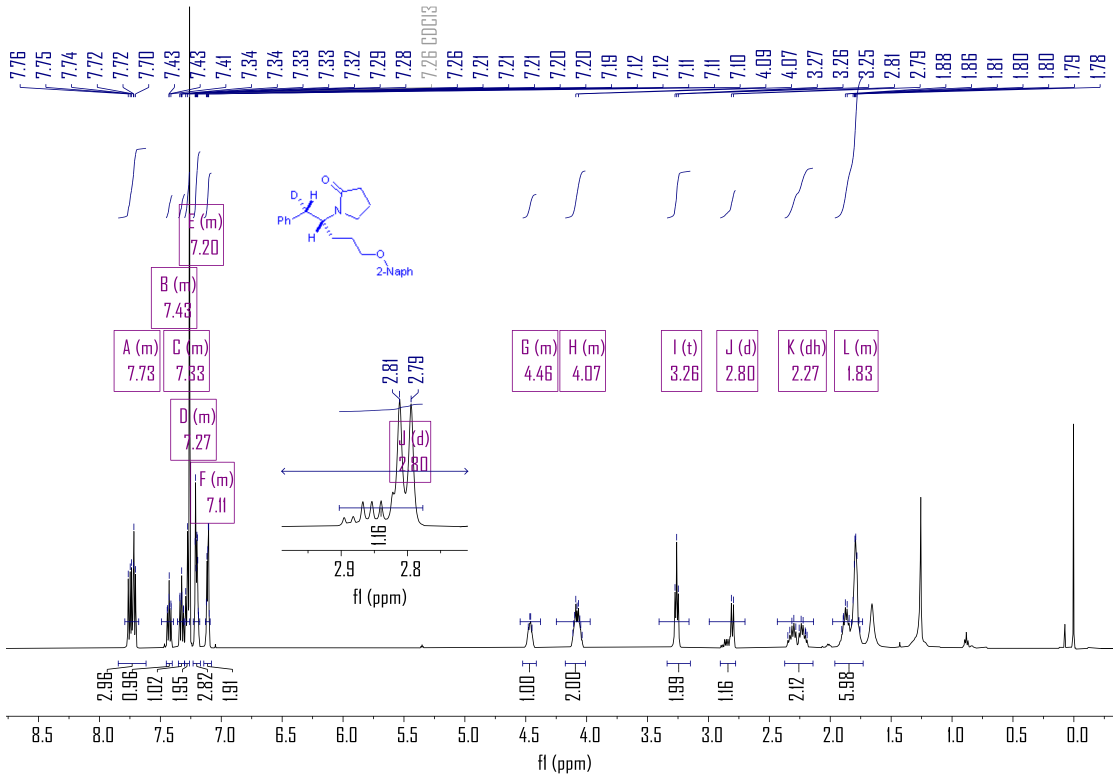

Supplementary Figure 354. ^{1}H NMR spectra for **77**

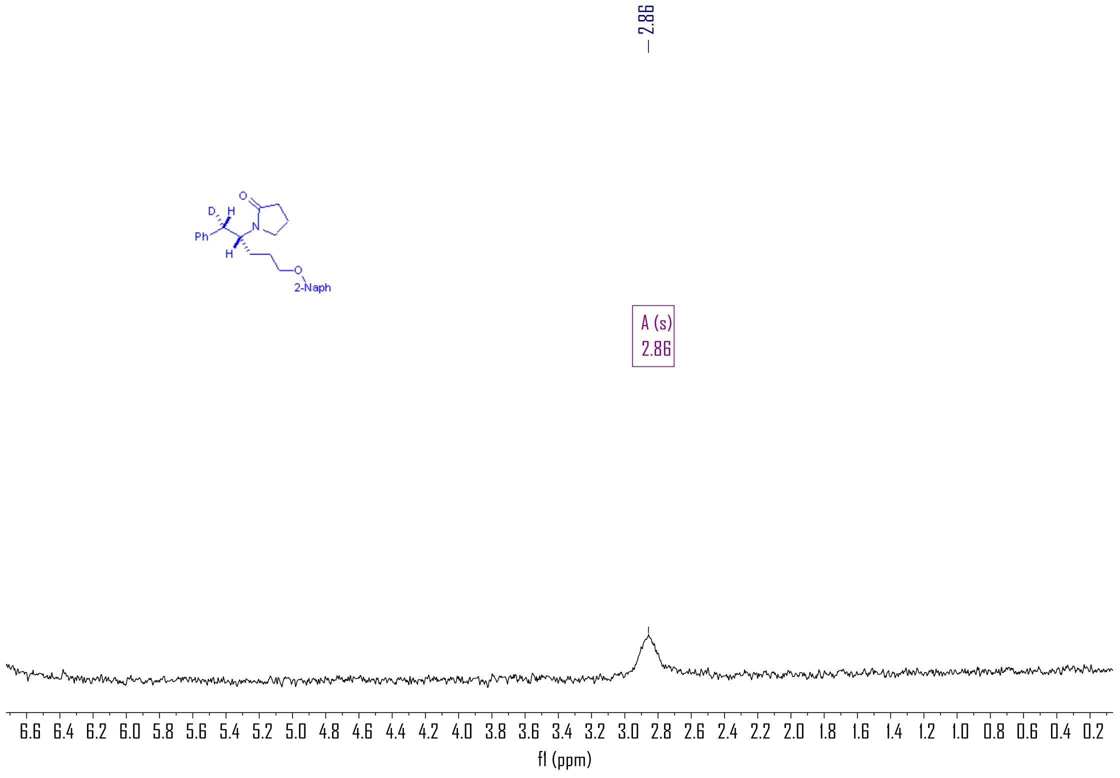

Supplementary Figure 355. ^{2}H NMR spectra for **77**

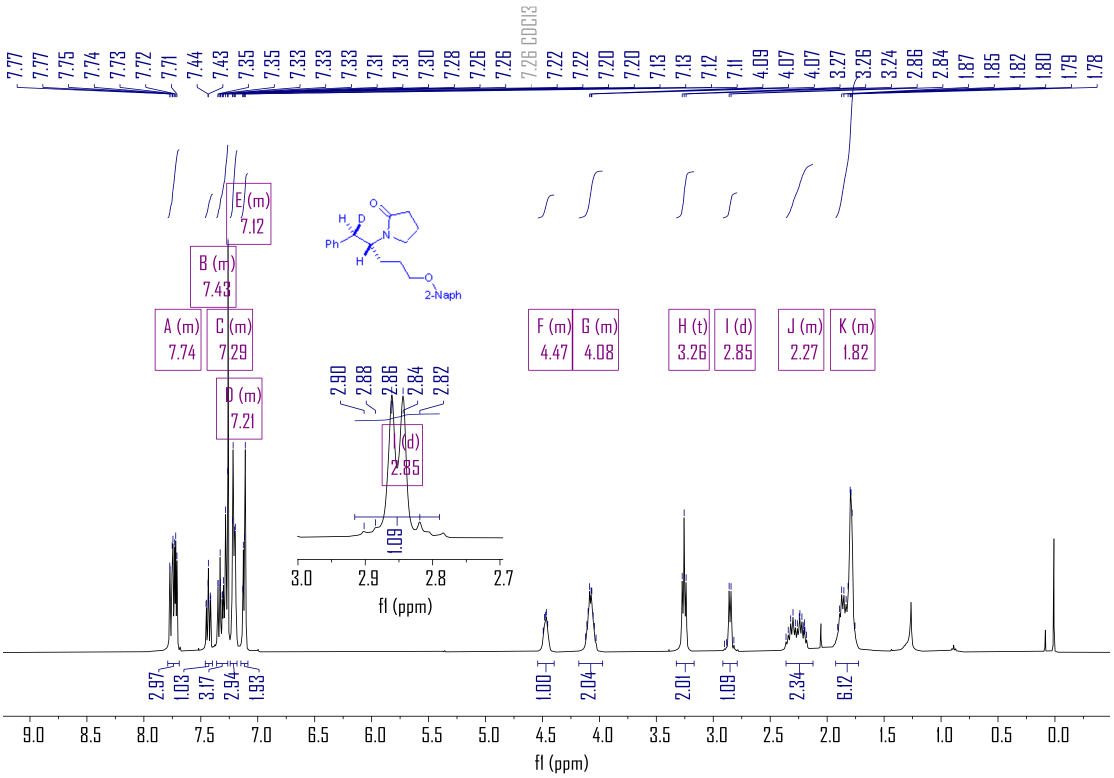

Supplementary Figure 356. ^{1}H NMR spectra for **78**

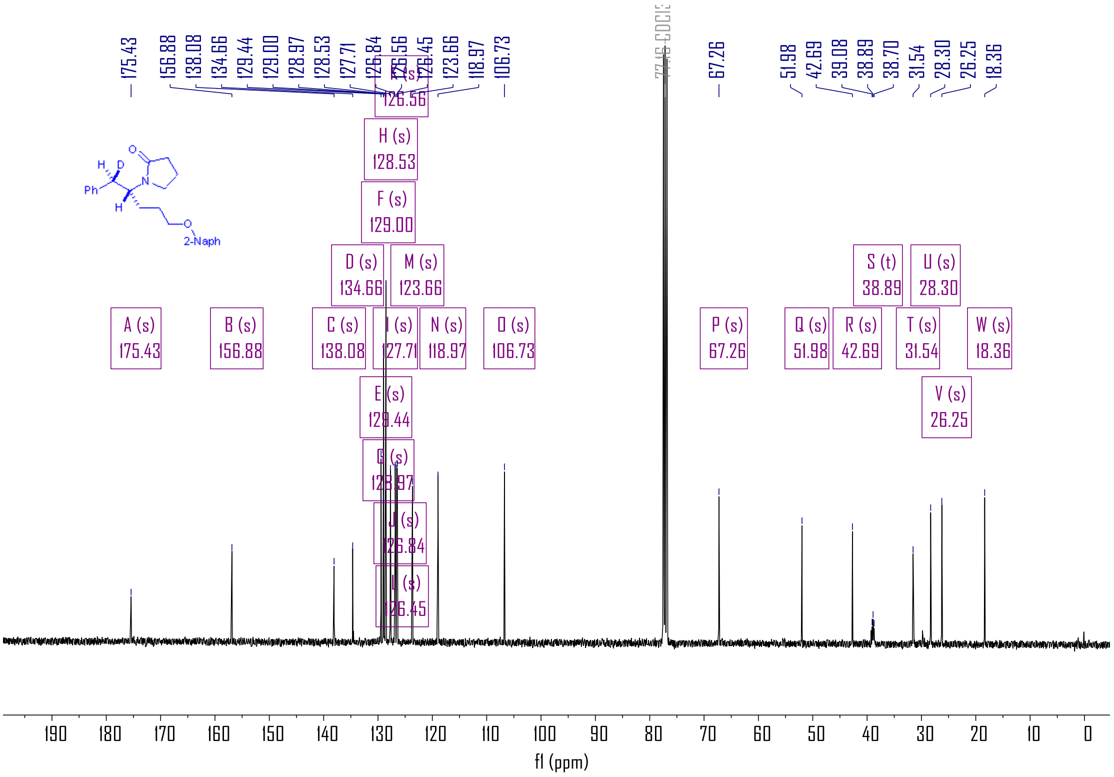

Supplementary Figure 357. ^{13}C NMR spectra for **78**

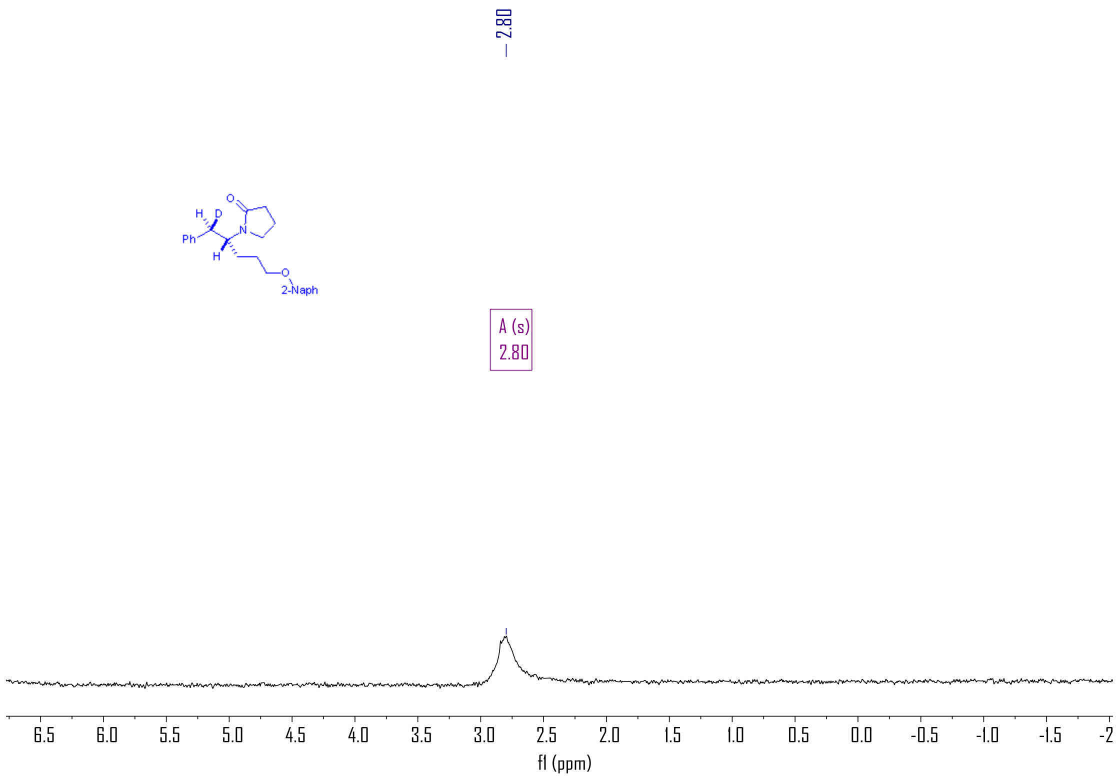

Supplementary Figure 358. ^{2}H NMR spectra for **78**

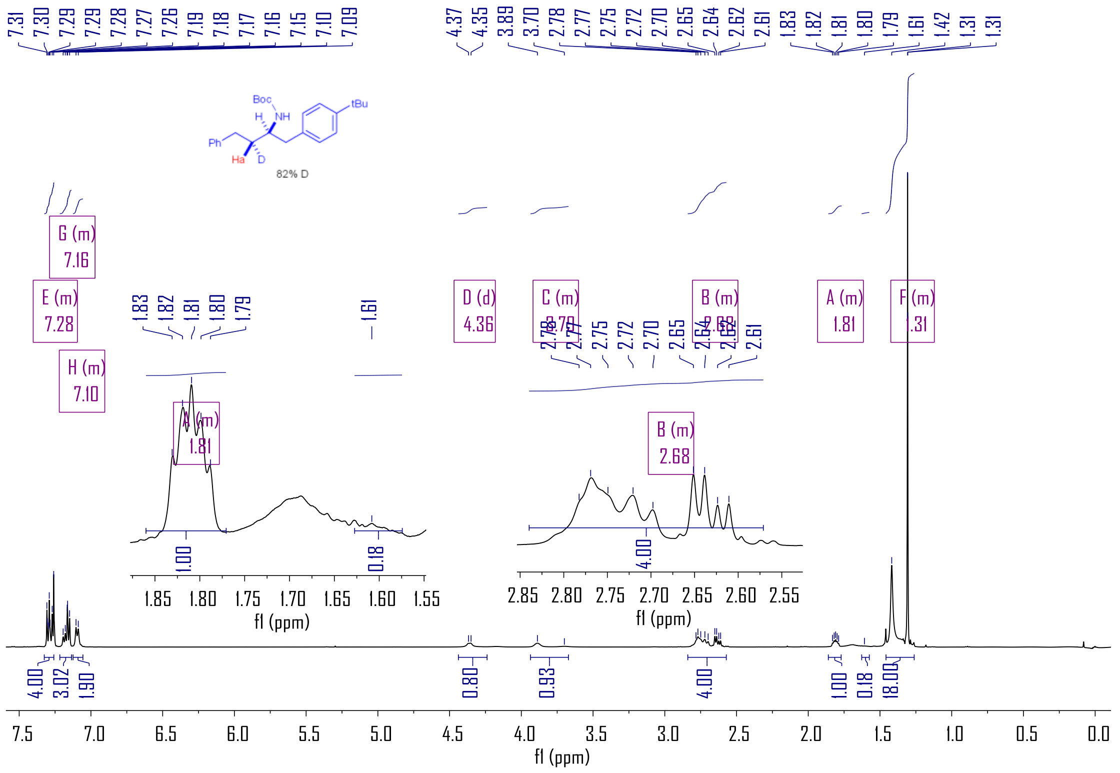

Supplementary Figure 359. ^{1}H NMR spectra for **80**

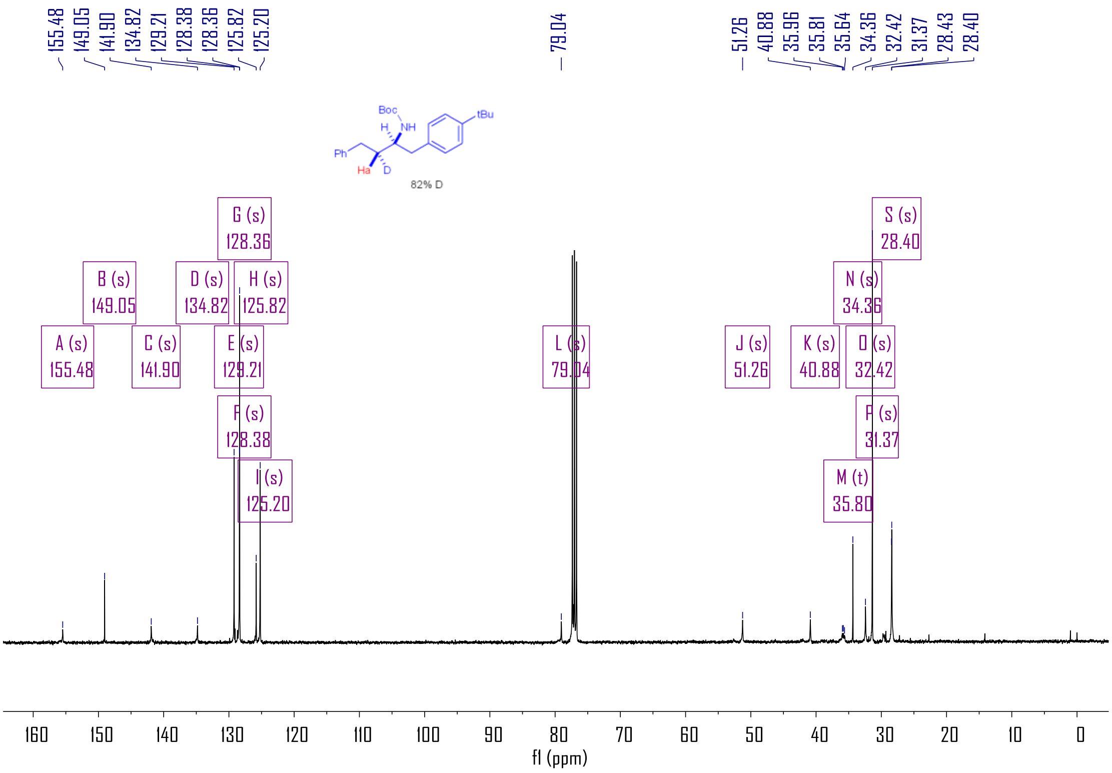

Supplementary Figure 360. ^{13}C NMR spectra for **80**

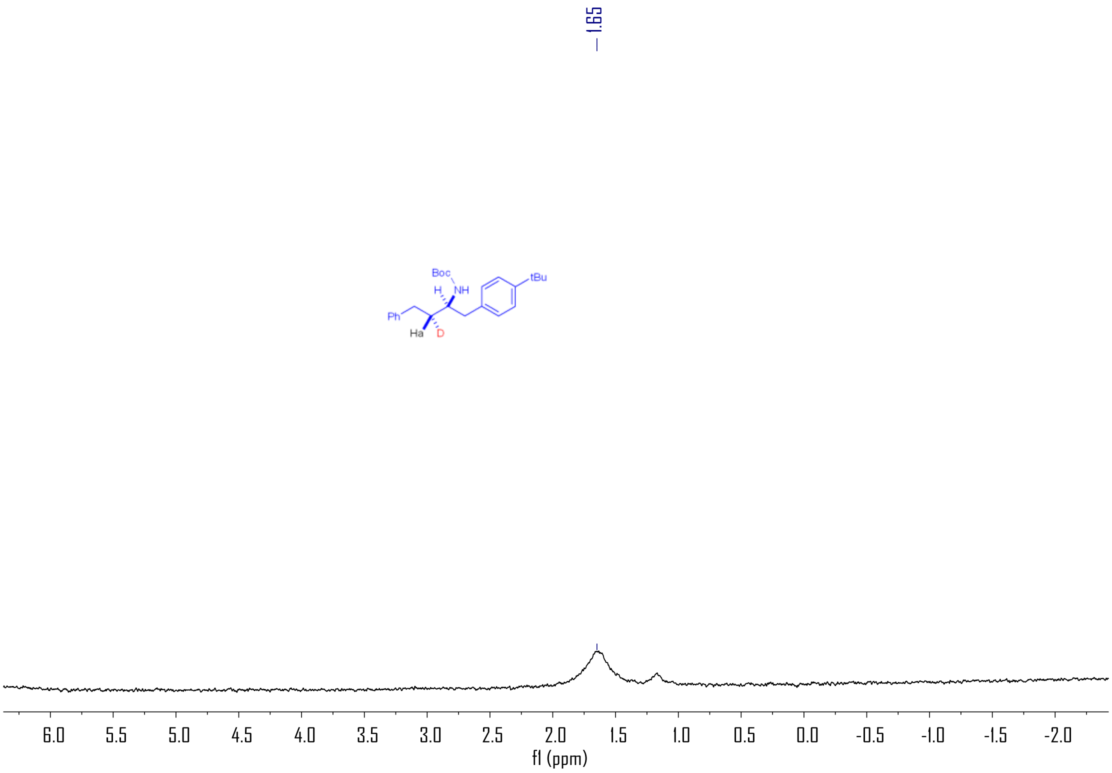

Supplementary Figure 361. ^{2}H NMR spectra for **80**

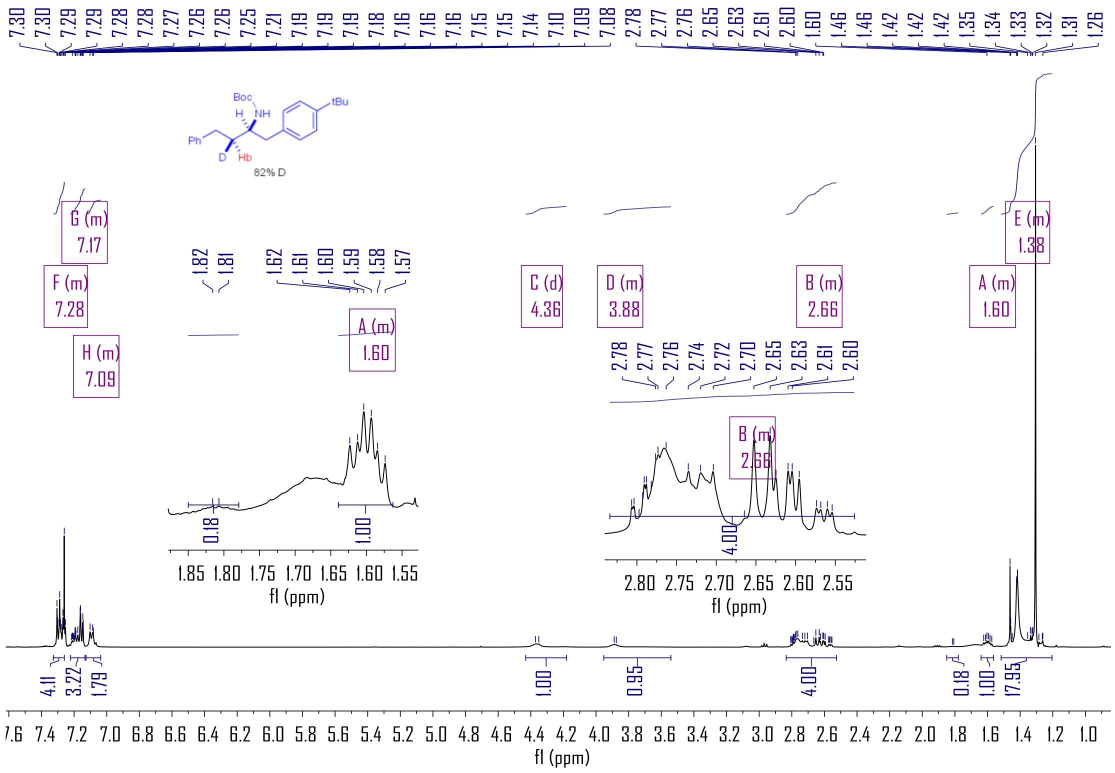

Supplementary Figure 362. ^{1}H NMR spectra for **81**

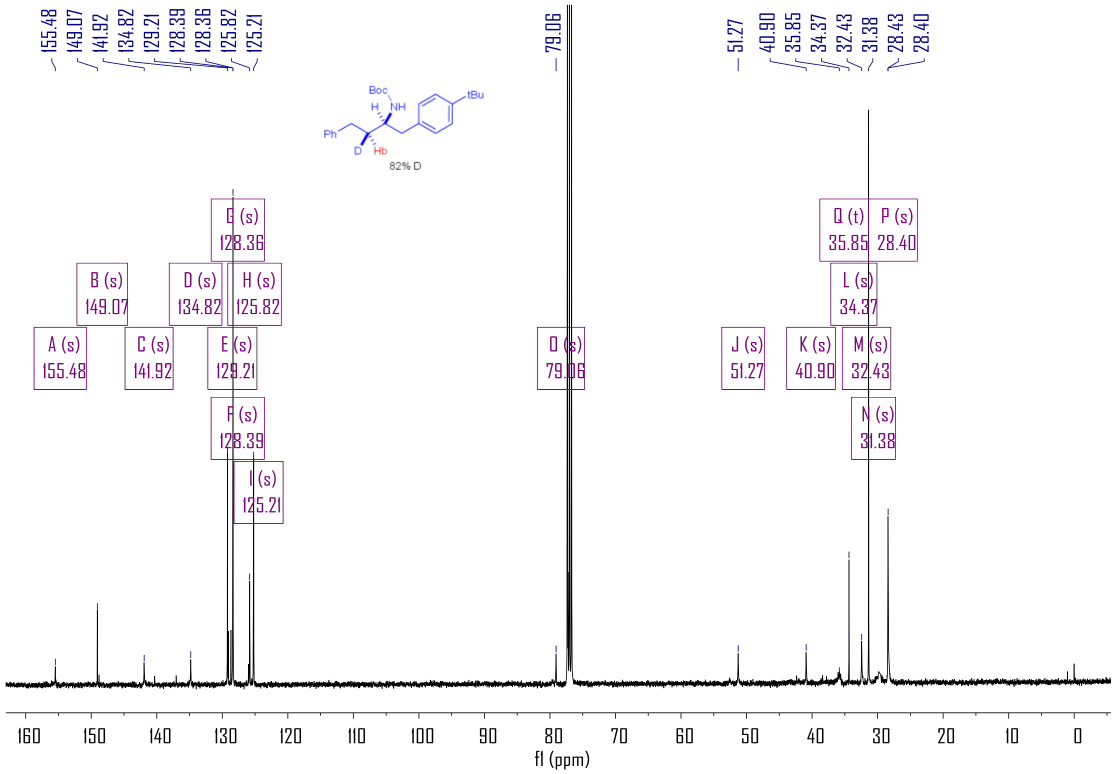

Supplementary Figure 363. ^{13}C NMR spectra for **81**

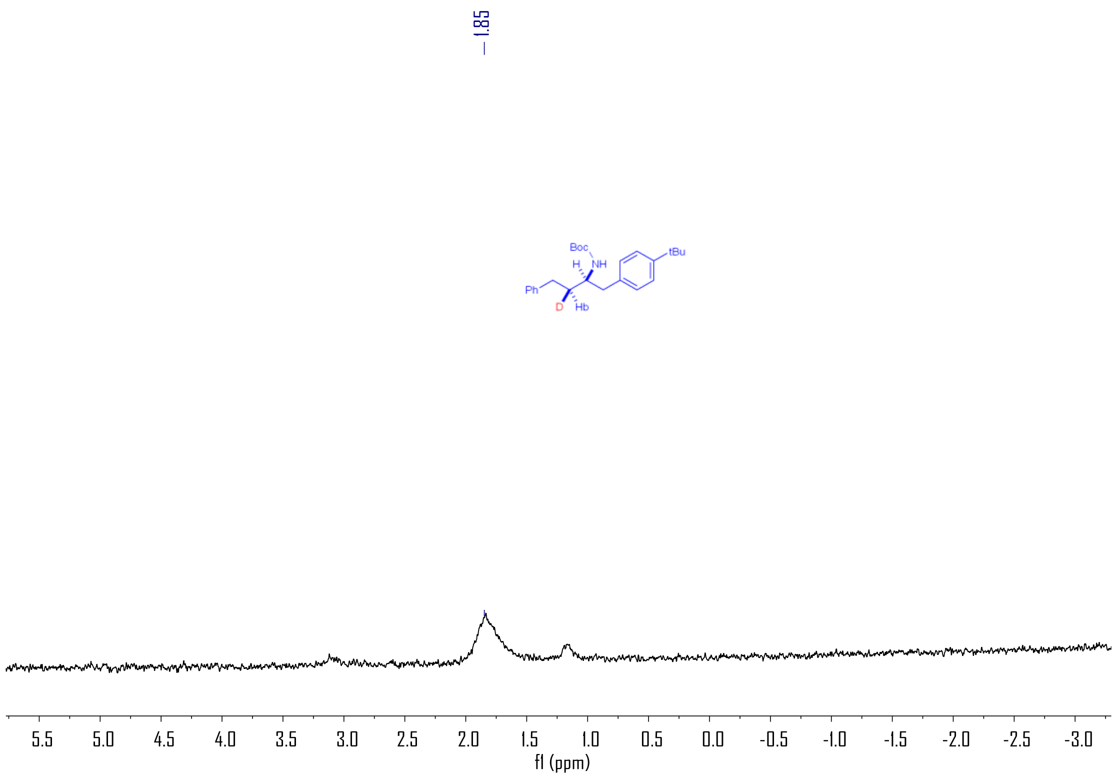

Supplementary Figure 364. ^{2}H NMR spectra for **81**

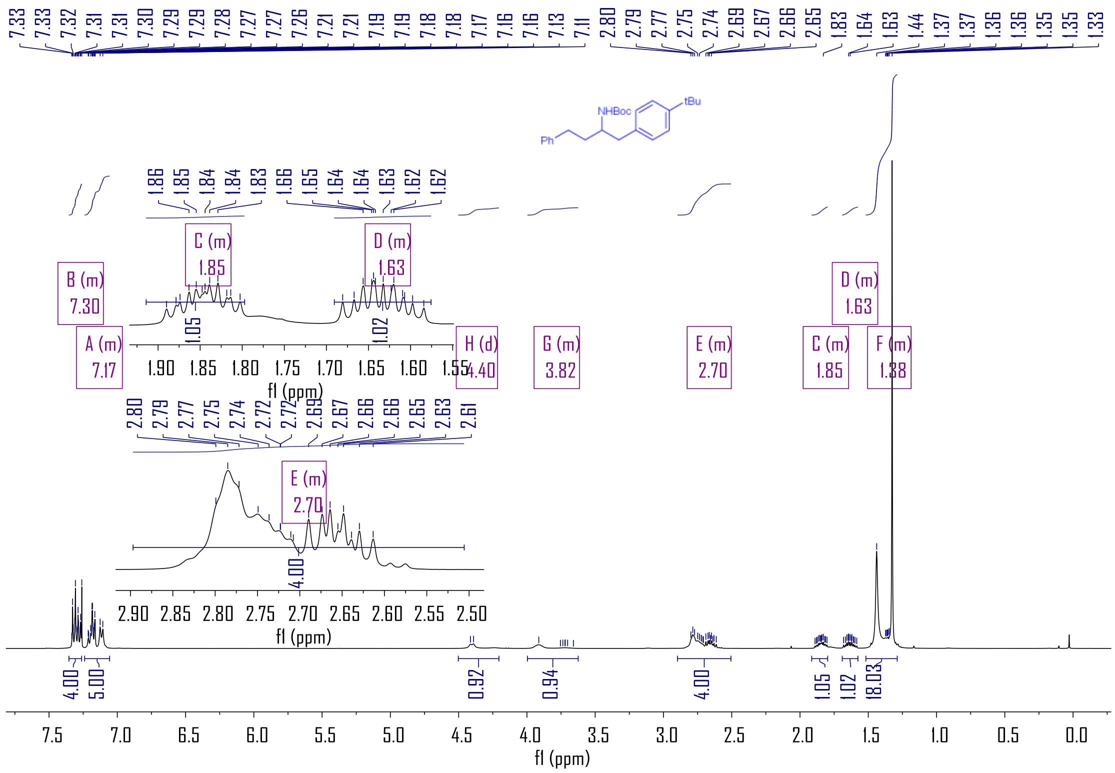

Supplementary Figure 365. ^{1}H NMR spectra for **80b**

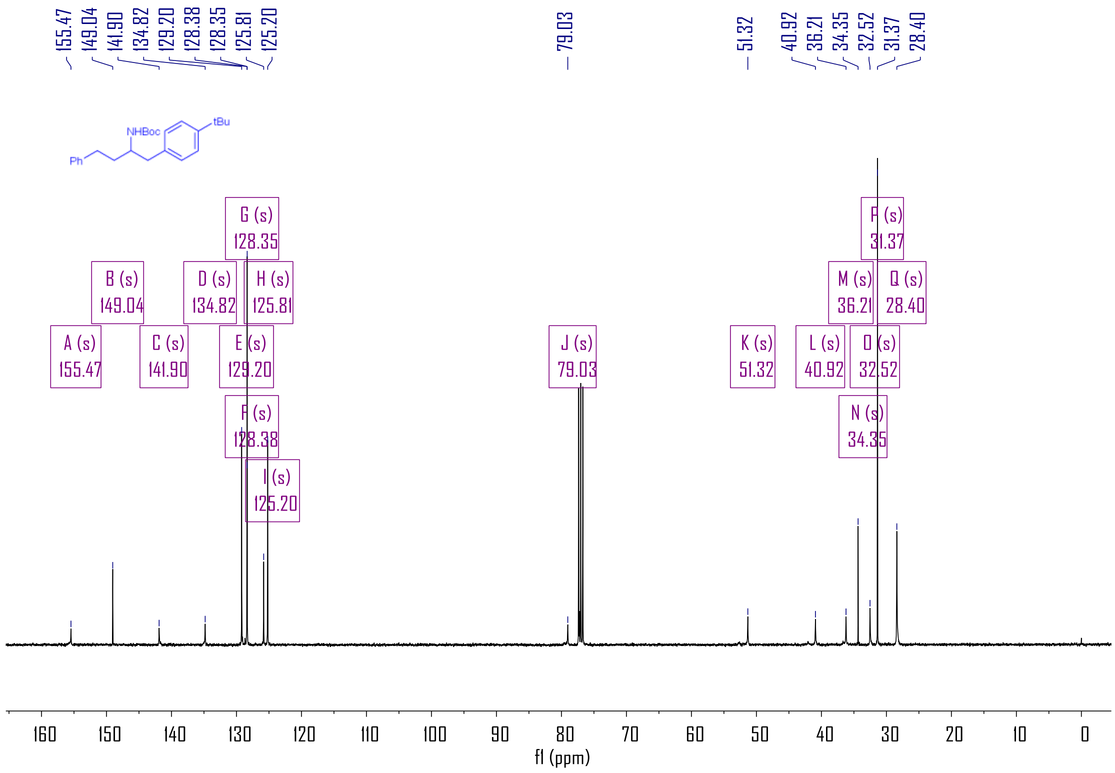

Supplementary Figure 366. ^{13}C NMR spectra for **80b**

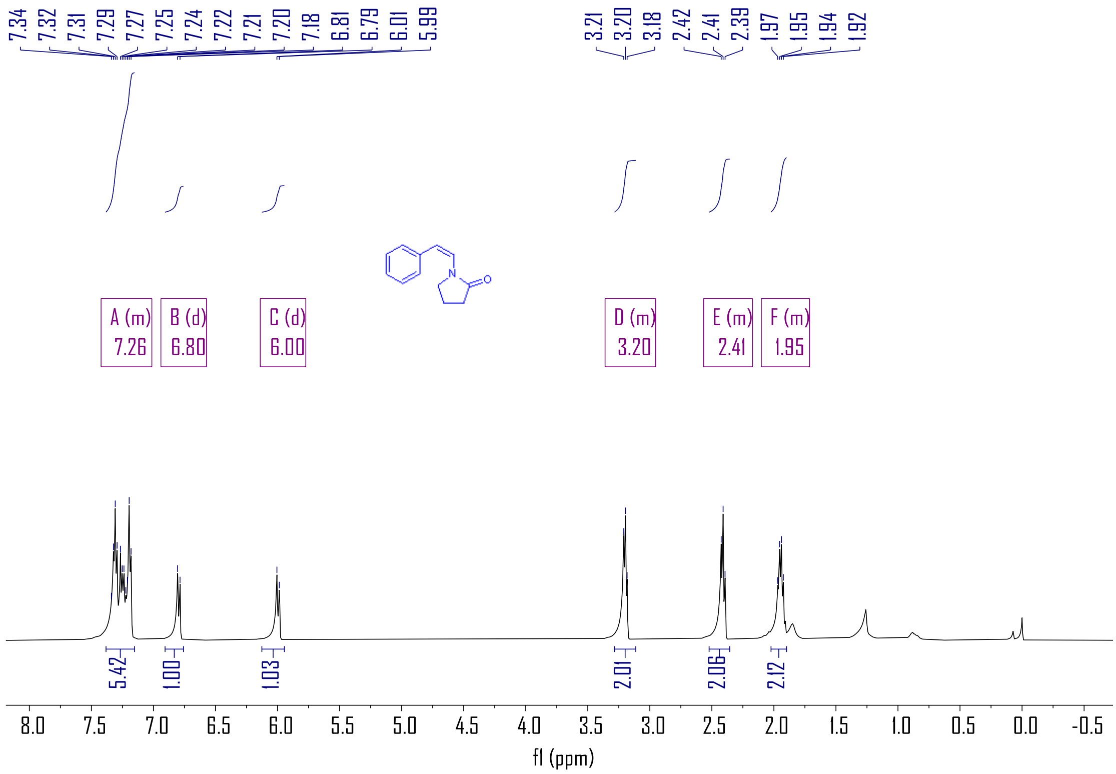

Supplementary Figure 367. ^{1}H NMR spectra for ***Z*-75**

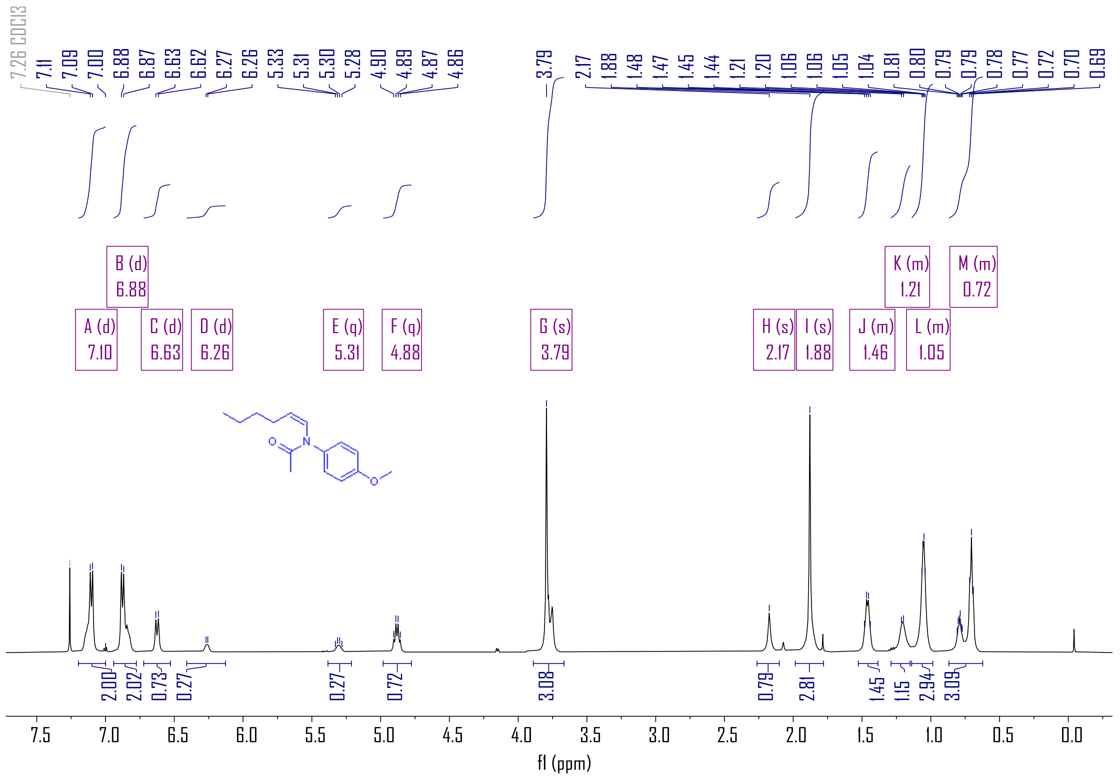

Supplementary Figure 368. ^{1}H NMR spectra for ***Z*-82**

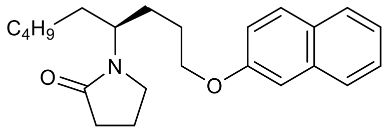

(*S*)-1-(1-(naphthalen-2-yloxy)nonan-4-yl)pyrrolidin-2-one (**1**)

HPLC analysis: The ee was determined to be 96% on a CHIRALCEL OD-H column (20% *i*PrOH in hexane, 2.0 mL/min, 40 ^{o}C); retention times for compound obtained using (*S*,*S*)-**L1**: 3.8 min (minor), 4.2 min (major).

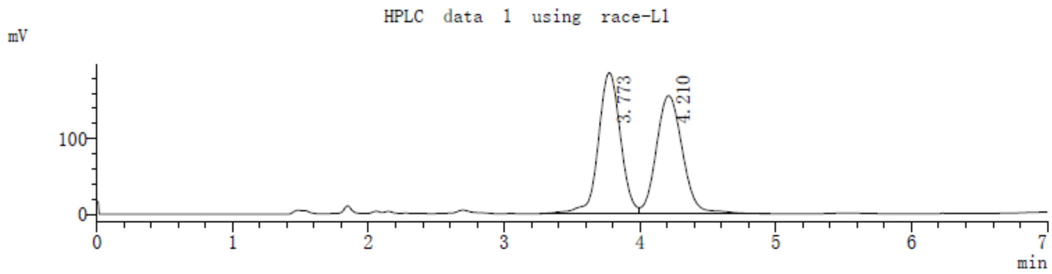

检测器A 214nm

Peak[#]	RetTime[min]	Height[uV]	Width[min]	Area[uV*s]	Area[%]
1	3.773	186485	0.171	2113508	50.343
2	4.210	156008	0.205	2084707	49.657

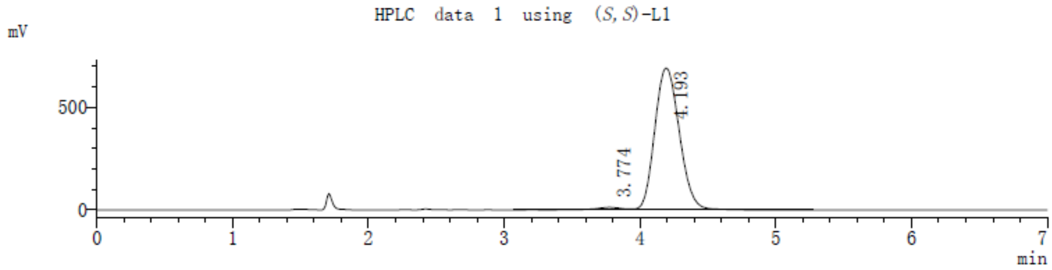

检测器A 214nm

Peak[#]	RetTime[min]	Height[uV]	Width[min]	Area[uV*s]	Area[%]
1	3.774	12322	0.195	176293	1.983
2	4.193	691473	0.197	8712908	98.017

Supplementary Figure 369. HPLC spectra for **1**

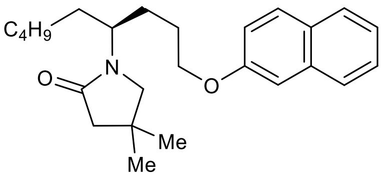

(*S*)-4,4-dimethyl-1-(1-(naphthalen-2-yloxy)nonan-4-yl)pyrrolidin-2-one (**2**)

HPLC analysis: The ee was determined to be 97% on a CHIRALCEL OD-H column (10% *i*PrOH in hexane, 1.0 mL/min, 40 ^{o}C); retention times for compound obtained using (*S*,*S*)-**L1**: 11.7 min (minor), 14.4 min (major).

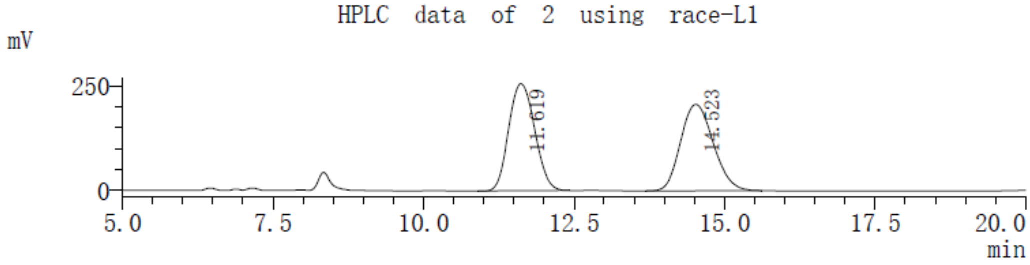

检测器A 214nm

Peak[#]	RetTime[min]	Height[uV]	Width[min]	Area[uV*s]	Area[%]
1	11.619	255817	0.481	7704558	49.771
2	14.523	206388	0.594	7775523	50.229

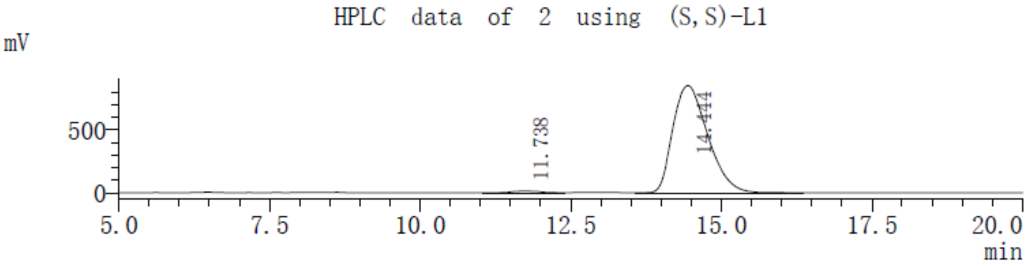

检测器A 214nm

Peak[#]	RetTime[min]	Height[uV]	Width[min]	Area[uV*s]	Area[%]
1	11.738	13983	0.498	429517	1.237
2	14.444	849274	0.635	34293290	98.763

Supplementary Figure 370. HPLC spectra for **2**

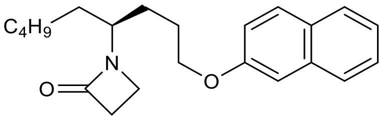

(*S*)-1-(1-(naphthalen-2-yloxy)nonan-4-yl)azetidin-2-one (**3**)

HPLC analysis: The ee was determined to be 89% on a CHIRALCEL OD-H column (15% *i*PrOH in hexane, 1.5 mL/min, 40 ºC); retention times for compound obtained using (*S*,*S*)-**L1**: 6.4 min (minor), 7.0 min (major).

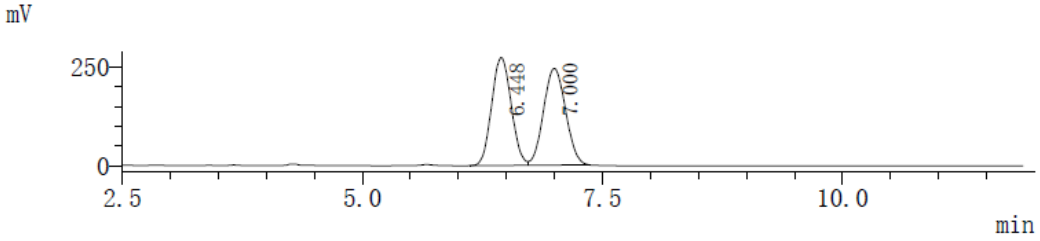

检测器A Ch1 214nm

Peak[#]	RetTime[min]	Height[uV]	Width[min]	Area[uV*s]	Area[%]
1	6.448	274698	0.225	3899864	49.742
2	7.000	246071	0.254	3940326	50.258

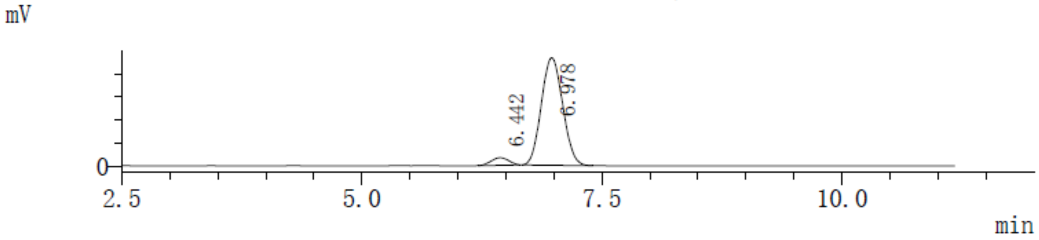

检测器A Ch1 214nm

Peak[#]	RetTime[min]	Height[uV]	Width[min]	Area[uV*s]	Area[%]
1	6.442	16315	0.207	207359	5.426
2	6.978	233211	0.245	3614025	94.574

Supplementary Figure 371. HPLC spectra for **3**

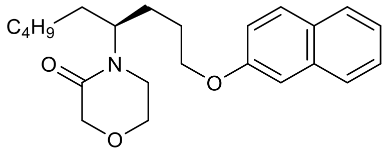

(*S*)-4-(1-(naphthalen-2-yloxy)nonan-4-yl)morpholin-3-one (**4**)

HPLC analysis: The ee was determined to be 98% on a CHIRALPAK AD-H column (10% *i*PrOH in hexane, 1.0 mL/min, 40 °C); retention times for compound obtained using (*S*,*S*)-**L1**: 10.1 min (minor), 10.8 min (major).

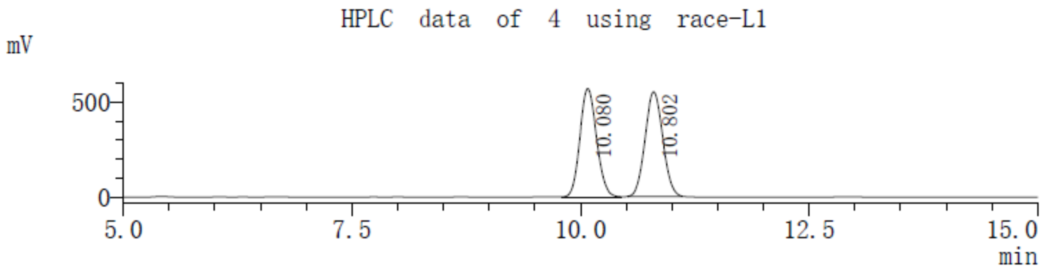

检测器A Ch1 214nm

Peak[#]	RetTime[min]	Height[uV]	Width[min]	Area[uV*s]	Area[%]
1	10.080	571438	0.197	7234501	49.337
2	10.802	552156	0.211	7428821	50.663

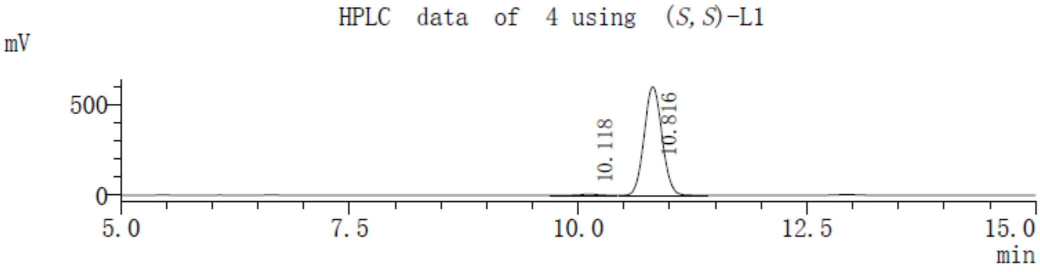

检测器A Ch1 214nm

Peak[#]	RetTime[min]	Height[uV]	Width[min]	Area[uV*s]	Area[%]
1	10.118	6287	0.194	82228	0.998
2	10.816	600284	0.212	8154014	99.002

Supplementary Figure 372. HPLC spectra for **4**

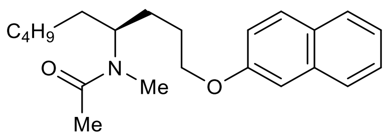

(*S*)-*N*-methyl-*N*-(1-(naphthalen-2-yloxy) nonan-4-yl)acetamide (**5**)

HPLC analysis: The ee was determined to be 92% on a CHIRALPAK AD-H column (7% *i*PrOH in hexane, 0.7 mL/min, 40 °C); retention times for compound obtained using (*S*,*S*)-**L1**: 13.3 min (minor), 14.1 min (major).

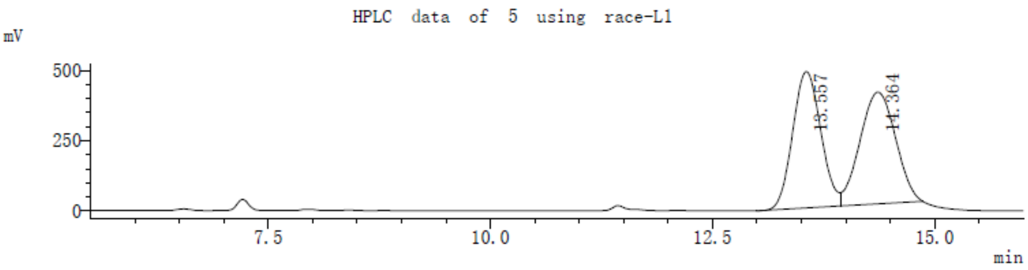

检测器A 214nm

Peak[#]	RetTime[min]	Height[uV]	Width[min]	Area[uV*s]	Area[%]
1	13.557	487089	0.347	10739399	49.060
2	14.364	398618	0.450	11150810	50.940

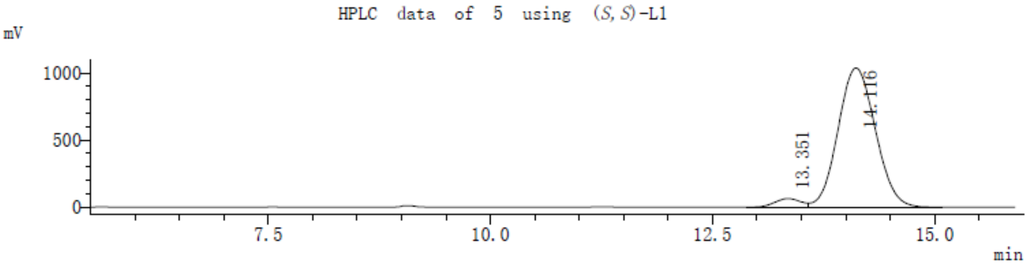

检测器A 214nm

Peak[#]	RetTime[min]	Height[uV]	Width[min]	Area[uV*s]	Area[%]
1	13.351	64976	0.366	1359815	4.269
2	14.116	1040152	0.459	30494293	95.731

Supplementary Figure 373. HPLC spectra for **5**

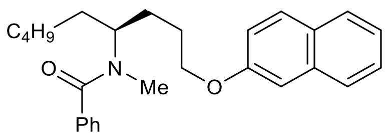

(*S*)-*N*-methyl-*N*-(1-(naphthalen-2-yloxy)nonan-4-yl)benzamide (**6**)

<u>HPLC analysis:</u> The ee was determined to be 92% on a CHIRALPAK AS-H column (10% *i*PrOH in hexane, 0.7 mL/min, 40 °C); retention times for compound obtained using (*S*,*S*)-**L1**: 7.4 min (minor), 7.9 min (major).

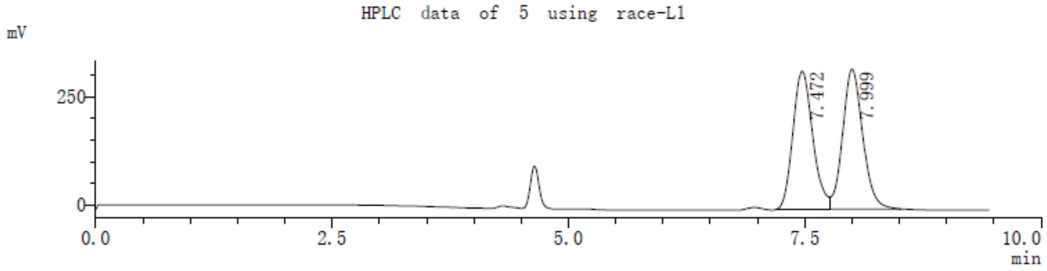

检测器A 214nm

Peak[#]	RetTime[min]	Height[uV]	Width[min]	Area[uV*s]	Area[%]
1	7.472	319855	0.224	4679382	49.440
2	7.999	324404	0.224	4785480	50.560

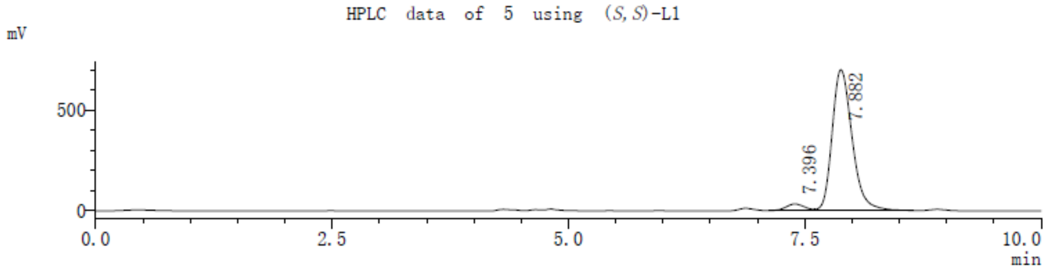

检测器A 214nm

Peak[#]	RetTime[min]	Height[uV]	Width[min]	Area[uV*s]	Area[%]
1	7.396	32943	0.217	452084	4.211
2	7.882	699998	0.222	10282661	95.789

Supplementary Figure 374. HPLC spectra for **6**

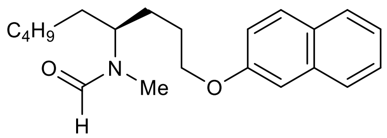

(*S*)-*N*-methyl-*N*-(1-(naphthalen-2-yloxy)nonan-4-yl)formamide (**7**)

HPLC analysis: The ee was determined to be 93% on a CHIRALPAK AS-H column (10% *i*PrOH in hexane, 1.0 mL/min, 40 °C); retention times for compound obtained using (*S*,*S*)-**L1**: 10.8 min (minor), 9.6 min (major).

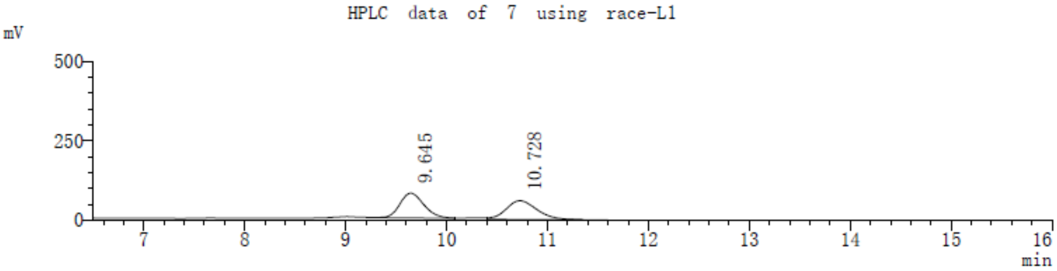

检测器A 214nm

Peak[#]	RetTime[min]	Height[uV]	Width[min]	Area[uV*s]	Area[%]
1	9.645	77960	0.254	1289127	50.520
2	10.728	59169	0.327	1262580	49.480

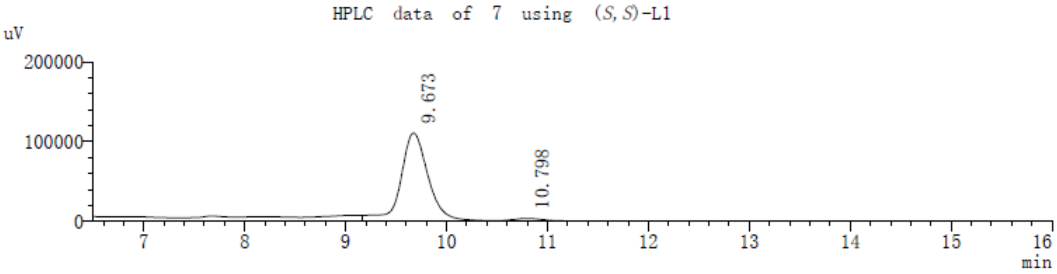

检测器A 214nm

Peak[#]	RetTime[min]	Height[uV]	Width[min]	Area[uV*s]	Area[%]
1	9.673	111070	0.269	2123229	96.692
2	10.798	3285	0.336	72645	3.308

Supplementary Figure 375. HPLC spectra for **7**

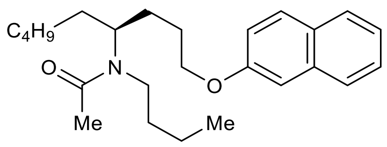

(*S*)-*N*-butyl-*N*-(1-(naphthalen-2-yloxy)nonan-4-yl)acetamide (**8**)

HPLC analysis: The ee was determined to be 90% on a CHIRALCEL OD-H column (20% *i*PrOH in hexane, 2.0 mL/min, 40 °C); retention times for compound obtained using (*S*,*S*)-**L1**: 3.7 min (minor), 2.8 min (major).

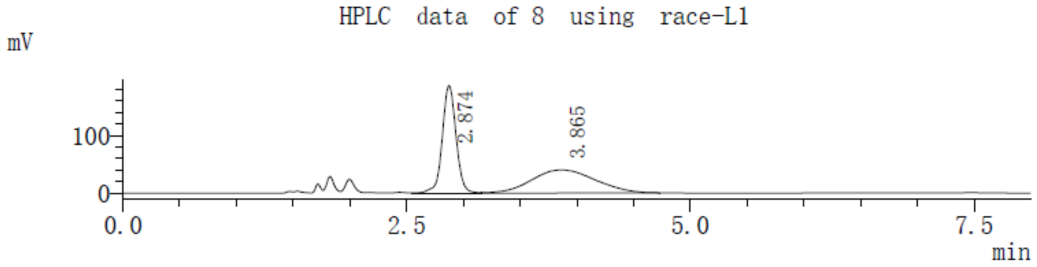

检测器A 214nm

Peak[#]	RetTime[min]	Height[uV]	Width[min]	Area[uV*s]	Area[%]
1	2.874	186246	0.126	1570439	50.232
2	3.865	39706	0.618	1555903	49.768

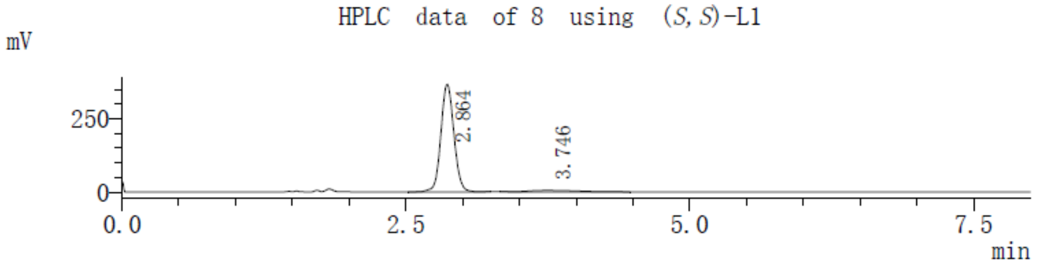

检测器A 214nm

Peak[#]	RetTime[min]	Height[uV]	Width[min]	Area[uV*s]	Area[%]
1	2.864	366708	0.122	2930764	95.148
2	3.746	5249	0.444	149443	4.852

Supplementary Figure 376. HPLC spectra for **8**

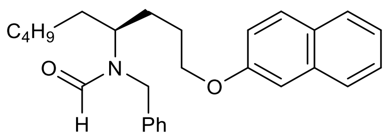

(*S*)-*N*-benzyl-*N*-(1-(naphthalen-2-yloxy)nonan-4-yl)formamide (**9**)

<u>HPLC analysis:</u> The ee was determined to be 91% on a CHIRALPAK AD-H column (8% *i*PrOH in hexane, 0.8 mL/min, 40 ^{o}C); retention times for compound obtained using (*S*,*S*)-**L1**: 16.3 min (minor), 15.2 min (major).

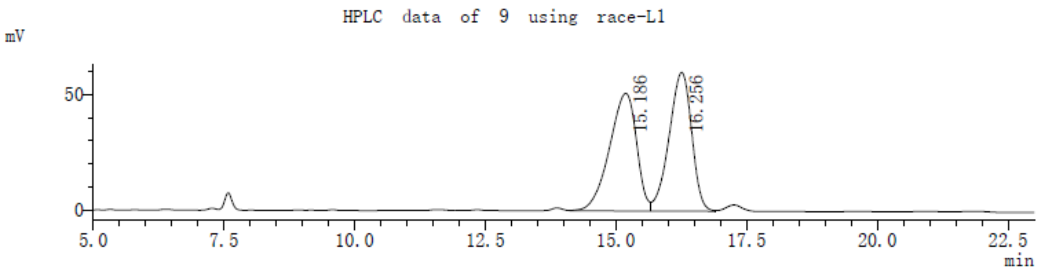

检测器A 214nm

Peak[#]	RetTime[min]	Height[uV]	Width[min]	Area[uV*s]	Area[%]
1	15.186	51043	0.564	1846151	50.489
2	16.256	60224	0.468	1810378	49.511

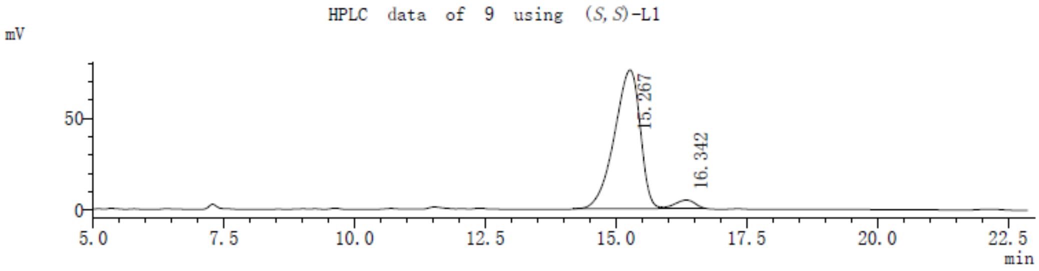

检测器A 214nm

Peak[#]	RetTime[min]	Height[uV]	Width[min]	Area[uV*s]	Area[%]
1	15.267	75400	0.534	2591758	95.530
2	16.342	4561	0.428	121272	4.470

Supplementary Figure 377. HPLC spectra for **9**

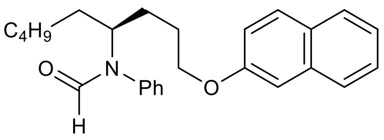

(*S*)-*N*-(1-(naphthalen-2-yloxy)nonan-4-yl)-*N*-phenylformamide (**10**)

HPLC analysis: The ee was determined to be 91% on a CHIRALPAK AS-H column (7% *i*PrOH in hexane, 0.7 mL/min, 40 °C); retention times for compound obtained using (*S*,*S*)-**L1**: 13.5 min (minor), 11.5 min (major).

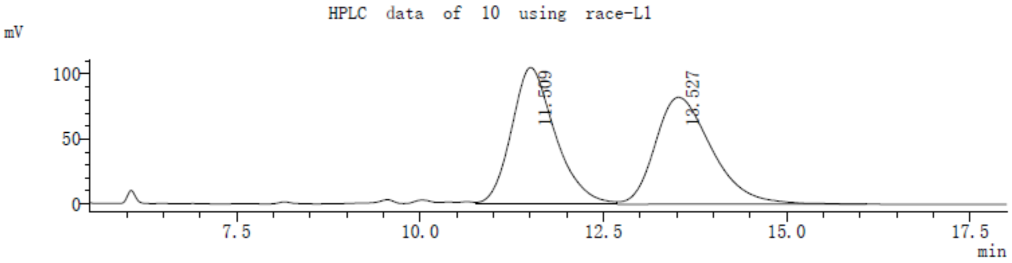

检测器A 214nm

Peak[#]	RetTime[min]	Height[uV]	Width[min]	Area[uV*s]	Area[%]
1	11.509	105031	0.625	4378349	49.850
2	13.527	82119	0.814	4404689	50.150

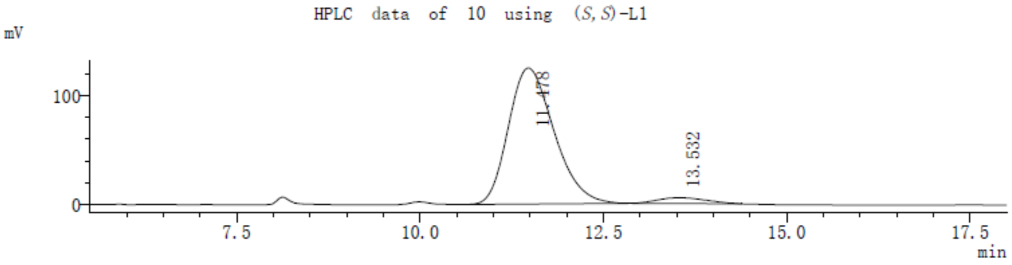

检测器A 214nm

Peak[#]	RetTime[min]	Height[uV]	Width[min]	Area[uV*s]	Area[%]
1	11.478	124657	0.671	5404055	95.604
2	13.532	5430	0.753	248479	4.396

Supplementary Figure 378. HPLC spectra for **10**

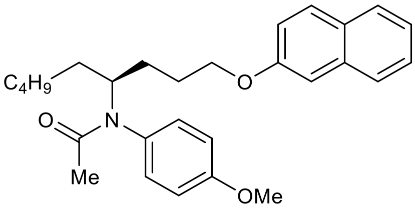

(*S*)-*N*-(4-methoxyphenyl)-*N*-(1-(naphthalen-2-yloxy)nonan-4-yl)acetamide (**11**)

HPLC analysis: The ee was determined to be 94% on a CHIRALPAK AD-H column (15% *i*PrOH in hexane, 1.5 mL/min, 40 °C); retention times for compound obtained using (*S*,*S*)-**L1**: 5.4 min (minor), 4.6 min (major).

HPLC data of 11 using race-L1

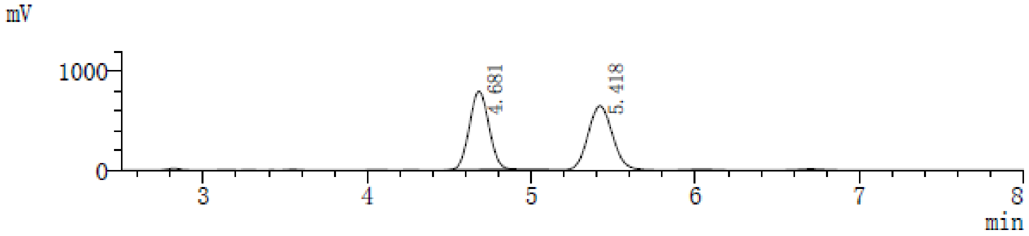

检测器A 214nm

Peak[#]	RetTime[min]	Height[uV]	Width[min]	Area[uV*s]	Area[%]
1	4.681	788320	0.131	6554743	49.824
2	5.418	644579	0.160	6601050	50.176

HPLC data of 11 using (*S*, *S*)-L1

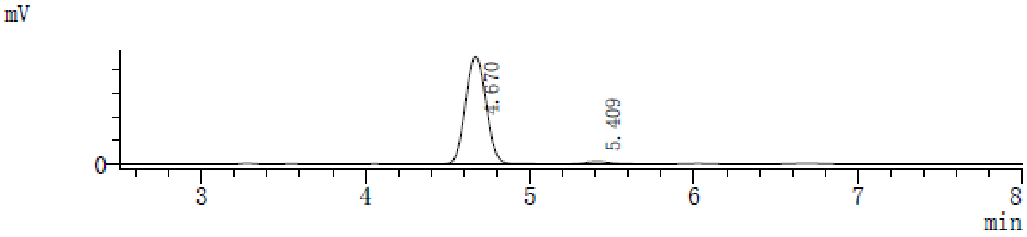

检测器A 214nm

Peak[#]	RetTime[min]	Height[uV]	Width[min]	Area[uV*s]	Area[%]
1	4.670	2273559	0.137	19824631	97.176
2	5.409	54829	0.161	576213	2.824

Supplementary Figure 379. HPLC spectra for **11**

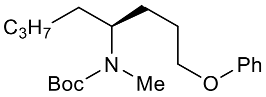

tert-butyl (*S*)-methyl(1-phenoxyoctan-4-yl)carbamate (**12**)

<u>**HPLC analysis:**</u> The ee was determined to be 86% on a CHIRALCEL OD-H column (10% *i*PrOH in hexane, 1.0 mL/min, 40 °C); retention times for compound obtained using (*S*,*S*)-**L1**: 3.9 min (minor), 3.5 min (major).

HPLC data of 12 using race-L1

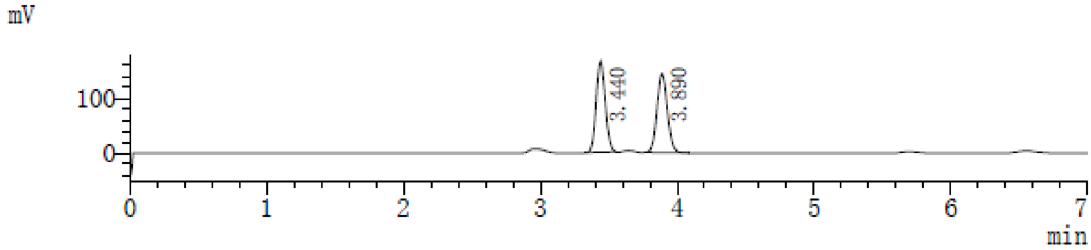

检测器A 214nm

Peak[#]	RetTime[min]	Height[uV]	Width[min]	Area[uV*s]	Area[%]
1	3.440	166433	0.072	764189	50.118
2	3.890	143751	0.082	760599	49.882

HPLC data of 12 using (*S, S*)-L1

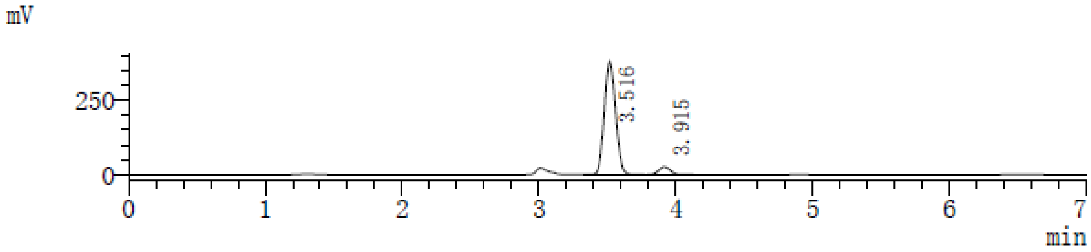

检测器A Ch1 214nm

Peak[#]	RetTime[min]	Height[uV]	Width[min]	Area[uV*s]	Area[%]
1	3.516	382214	0.090	2208179	92.779
2	3.915	27227	0.095	171860	7.221

Supplementary Figure 380. HPLC spectra for **12**

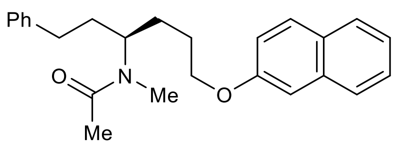

(*S*)-*N*-methyl-*N*-(6-(naphthalen-2-yloxy)-1-phenylhexan-3-yl)acetamide (**13**)

<u>HPLC analysis:</u> The ee was determined to be 94% on a CHIRALPAK AD-H column (8% *i*PrOH in hexane, 0.8 mL/min, 40 °C); retention times for compound obtained using (*S*,*S*)-**L1**: 19.6 min (minor), 21.0 min (major).

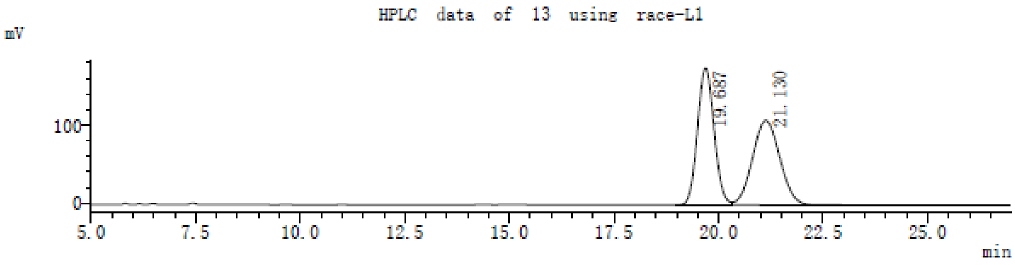

检测器A 214nm

Peak[#]	RetTime[min]	Height[uV]	Width[min]	Area[uV*s]	Area[%]
1	19.687	175969	0.431	4882065	49.699
2	21.130	108465	0.713	4941217	50.301

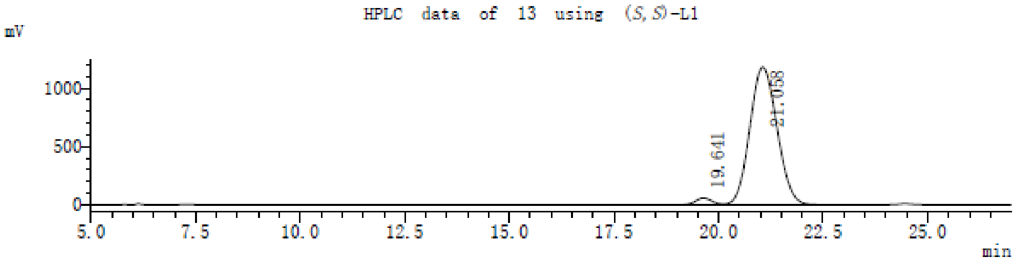

检测器A 214nm

Peak[#]	RetTime[min]	Height[uV]	Width[min]	Area[uV*s]	Area[%]
1	19.641	59395	0.422	1604658	2.917
2	21.058	1179811	0.710	53408948	97.083

Supplementary Figure 381. HPLC spectra for **13**

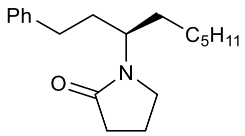

(*R*)-1-(1-phenylnonan-3-yl)pyrrolidin-2-one (**14**)

<u>HPLC analysis:</u> The ee was determined to be 91% on a CHIRALCEL OD-H column (10% *i*PrOH in hexane, 1.0 mL/min, 40 °C); retention times for compound obtained using (*S*,*S*)-**L1**: 5.1 min (minor), 5.8 min (major).

HPLC data of 14 using race-L1

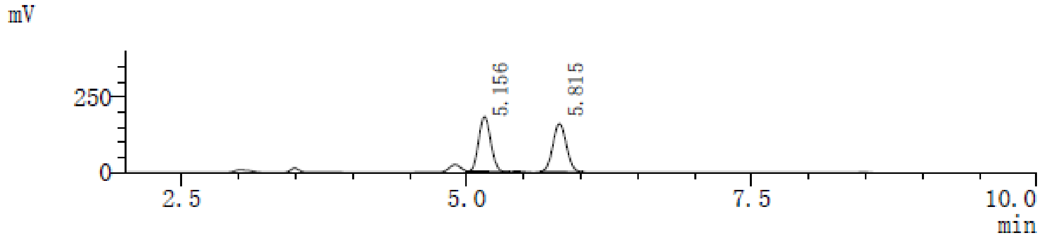

检测器A Ch1 214nm

Peak[#]	RetTime[min]	Height[uV]	Width[min]	Area[uV*s]	Area[%]
1	5.156	181919	0.117	1341262	50.208
2	5.815	160900	0.130	1330153	49.792

HPLC data of 14 using (*S*, *S*)-L1

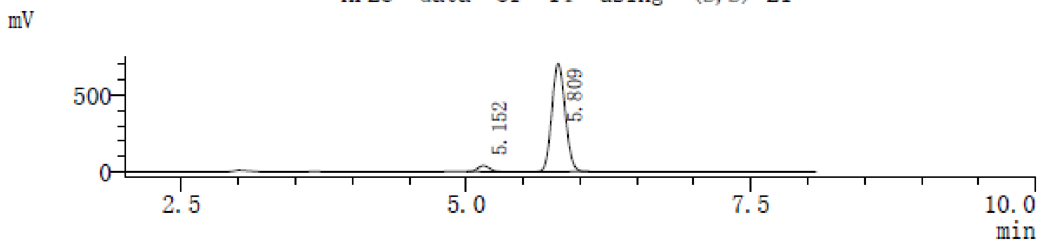

检测器A Ch1 214nm

Peak[#]	RetTime[min]	Height[uV]	Width[min]	Area[uV*s]	Area[%]
1	5.152	37812	0.115	273960	4.506
2	5.809	705203	0.129	5806178	95.494

Supplementary Figure 382. HPLC spectra for **14**

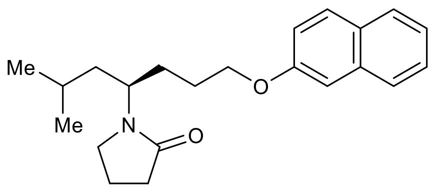

(*R*)-1-(6-methyl-1-(naphthalen-2-yloxy)heptan-4-yl)pyrrolidin-2-one (**15**)

<u>HPLC analysis:</u> The ee was determined to be 93% on a CHIRALCEL OD-H column (15% *i*PrOH in hexane, 1.5 mL/min, 40 °C); retention times for compound obtained using (*S*,*S*)-**L1**: 6.1 min (minor), 7.4 min (major).

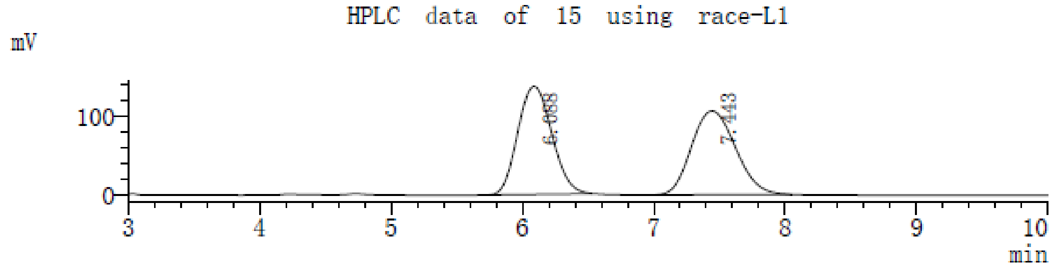

检测器A 214nm

Peak[#]	RetTime[min]	Height[uV]	Width[min]	Area[uV*s]	Area[%]
1	6.088	135834	0.285	2439616	49.762
2	7.443	105509	0.370	2462970	50.238

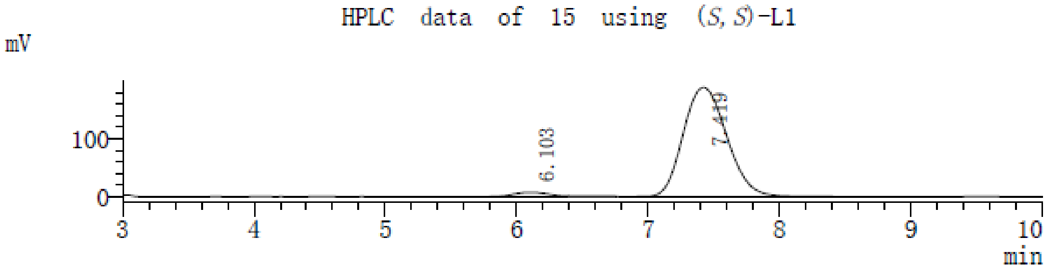

检测器A 214nm

Peak[#]	RetTime[min]	Height[uV]	Width[min]	Area[uV*s]	Area[%]
1	6.103	7741	0.290	149945	3.333
2	7.419	188455	0.363	4348393	96.667

Supplementary Figure 383. HPLC spectra for **15**

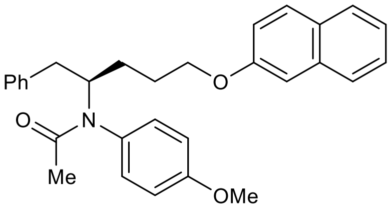

(*R*)-*N*-(4-methoxyphenyl)-*N*-(5-(naphthalen-2-yloxy)-1-phenylpentan-2-yl)acetamide (**16**)

<u>HPLC analysis:</u> The ee was determined to be 97% on a CHIRALPAK AS-H column (10% *i*PrOH in hexane, 1.0 mL/min, 40 °C); retention times for compound obtained using (*S*,*S*)-**L1**: 5.3 min (minor), 6.8 min (major).

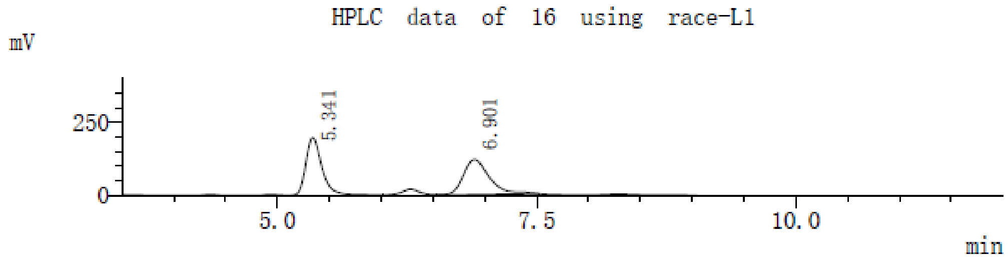

检测器A Ch1 214nm

Peak[#]	RetTime[min]	Height[uV]	Width[min]	Area[uV*s]	Area[%]
1	5.341	198262	0.160	2150635	50.212
2	6.901	120746	0.258	2132435	49.788

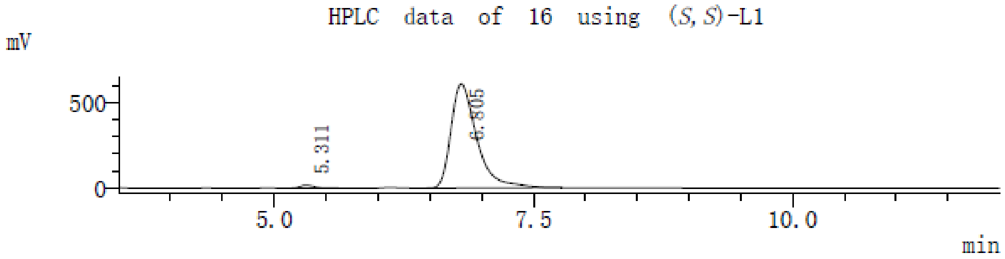

检测器A Ch1 214nm

Peak[#]	RetTime[min]	Height[uV]	Width[min]	Area[uV*s]	Area[%]
1	5.311	16598	0.154	169909	1.589
2	6.805	605802	0.253	10522804	98.411

Supplementary Figure 384. HPLC spectra for **16**

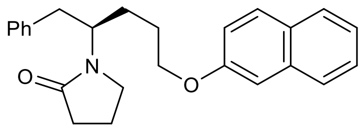

(*R*)-1-(5-(naphthalen-2-yloxy)-1-phenylpentan-2-yl)pyrrolidin-2-one (**17**)

<u>HPLC analysis:</u> The ee was determined to be 97% on a CHIRALPAK AD-H column (15% *i*PrOH in hexane, 1.5 mL/min, 40 °C); retention times for compound obtained using (*S*,*S*)-**L1**: 6.3 min (minor), 7.6 min (major).

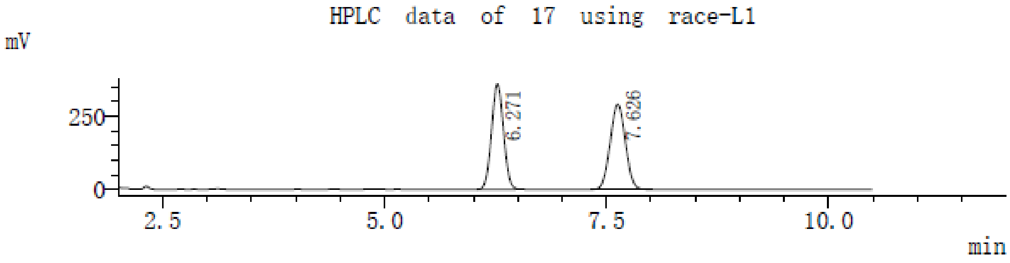

检测器A Ch1 214nm

Peak[#]	RetTime[min]	Height[uV]	Width[min]	Area[uV*s]	Area[%]
1	6.271	358660	0.147	3369035	49.321
2	7.626	290295	0.187	3461816	50.679

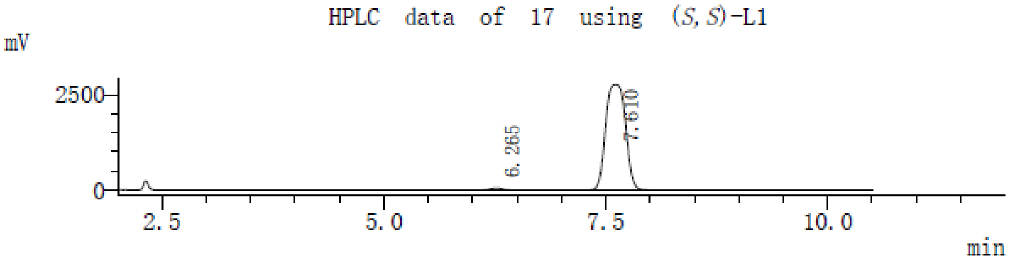

检测器A Ch1 214nm

Peak[#]	RetTime[min]	Height[uV]	Width[min]	Area[uV*s]	Area[%]
1	6.265	60119	0.145	551625	1.274
2	7.610	2756649	0.251	42762694	98.726

Supplementary Figure 385. HPLC spectra for **17**

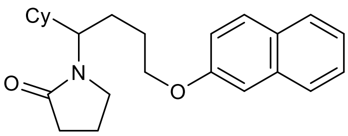

1-(4-(argiooxy)-1-cyclohexylbutyl)pyrrolidin-2-one (**18**)

HPLC analysis: The ee was determined to be <2% on a CHIRALCEL OD-H column (10% *i*PrOH in hexane, 1.0 mL/min, 40 ºC); retention times for compound obtained using (*S*,*S*)-**L1**: 11.9 min (minor), 15.6 min (major).

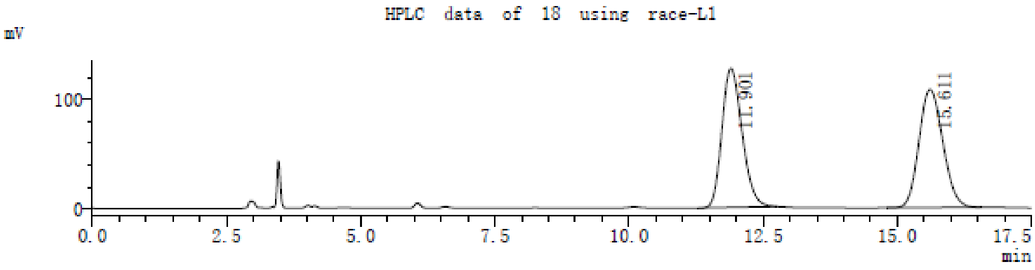

检测器A 214nm

Peak[#]	RetTime[min]	Height[uV]	Width[min]	Area[uV*s]	Area[%]
1	11.901	127531	0.406	3341824	49.869
2	15.611	108244	0.486	3359330	50.131

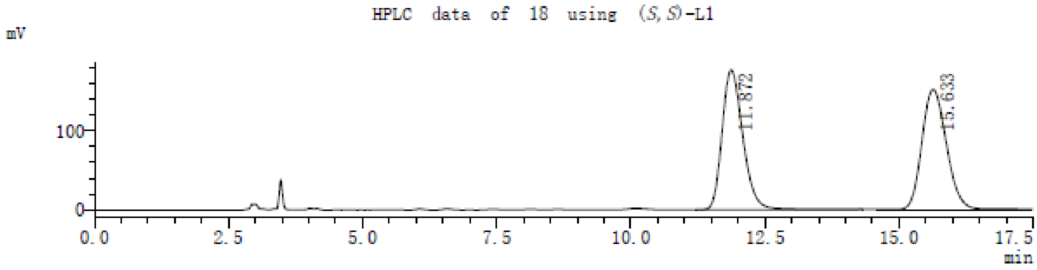

检测器A 214nm

Peak[#]	RetTime[min]	Height[uV]	Width[min]	Area[uV*s]	Area[%]
1	11.872	175648	0.411	4745256	49.621
2	15.633	151148	0.494	4817656	50.379

Supplementary Figure 386. HPLC spectra for **18**

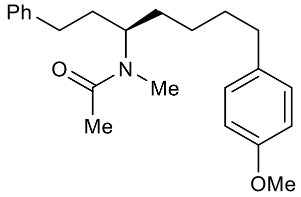

(*R*)-*N*-(7-(4-methoxyphenyl)-1-phenylheptan-3-yl)-*N*-methylacetamide (**19**)

HPLC analysis: The ee was determined to be 89% on a CHIRALPAK AD-H column (3% *i*PrOH in hexane, 0.3 mL/min, 40 °C); retention times for compound obtained using (*S*,*S*)-**L1**: 98.4 min (minor), 94.0 min (major).

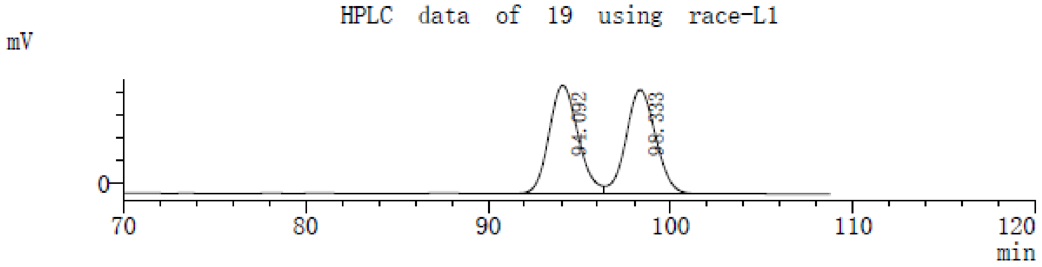

检测器A 214nm

Peak[#]	RetTime[min]	Height[uV]	Width[min]	Area[uV*s]	Area[%]
1	94.092	94960	1.708	10555427	50.639
2	98.333	91196	1.740	10288902	49.361

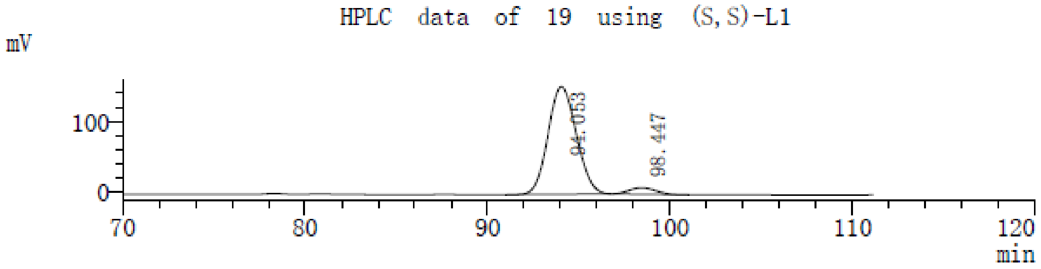

检测器A 214nm

Peak[#]	RetTime[min]	Height[uV]	Width[min]	Area[uV*s]	Area[%]
1	94.053	151919	1.693	16398385	94.491
2	98.447	9303	1.649	956118	5.509

Supplementary Figure 387. HPLC spectra for **19**

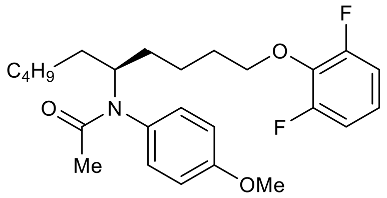

(S)-N-(1-(2,6-difluorophenoxy)decan-5-yl)-N-(4-methoxyphenyl)acetamide (**20**)

HPLC analysis: The ee was determined to be 91% on a CHIRALPAK AD-H column (15% *i*PrOH in hexane, 0.5 mL/min, 40 °C); retention times for compound obtained using (S,S)-**L1**: 24.3 min (minor), 22.9 min (major).

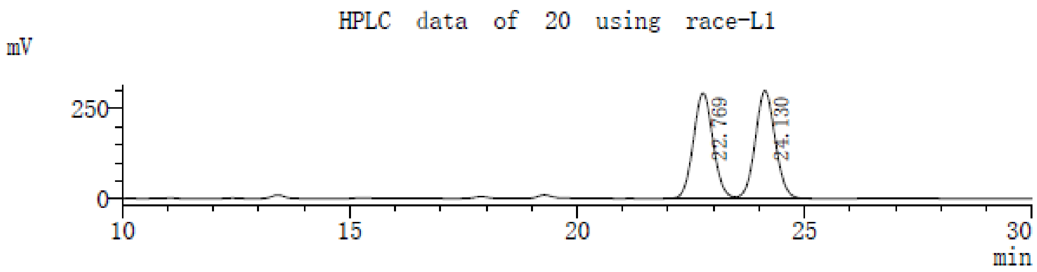

检测器A 214nm

Peak[#]	RetTime[min]	Height[uV]	Width[min]	Area[uV*s]	Area[%]
1	22.769	293332	0.468	8875938	49.080
2	24.130	299945	0.471	9208551	50.920

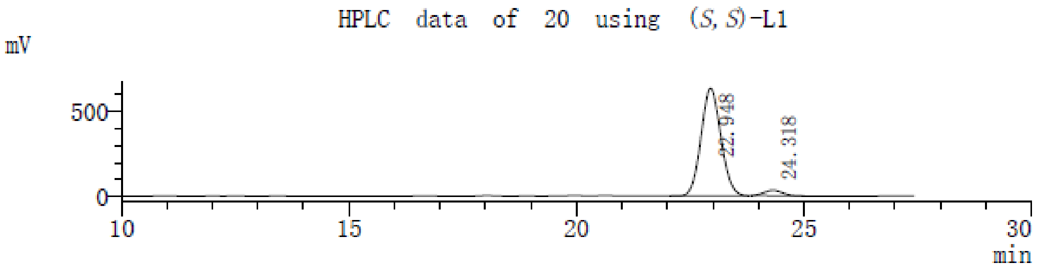

检测器A 214nm

Peak[#]	RetTime[min]	Height[uV]	Width[min]	Area[uV*s]	Area[%]
1	22.948	635925	0.473	19366305	95.421
2	24.318	32709	0.461	929395	4.579

Supplementary Figure 388. HPLC spectra for **20**

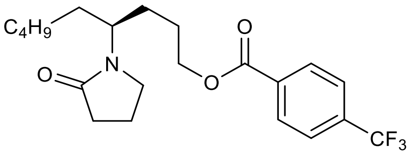

(*S*)-4-(2-oxopyrrolidin-1-yl)nonyl 4-(trifluoromethyl)benzoate (**21**)

HPLC analysis: The ee was determined to be 95% on a CHIRALCEL OD-H column (15% *i*PrOH in hexane, 1.5 mL/min, 40 °C); retention times for compound obtained using (*S*,*S*)-**L1**: 3.9 min (minor), 3.5 min (major).

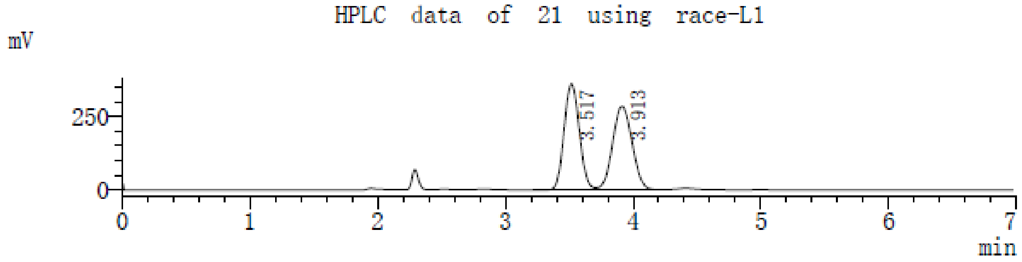

检测器A 214nm

Peak[#]	RetTime[min]	Height[uV]	Width[min]	Area[uV*s]	Area[%]
1	3.517	361555	0.131	3013360	49.765
2	3.913	284417	0.168	3041818	50.235

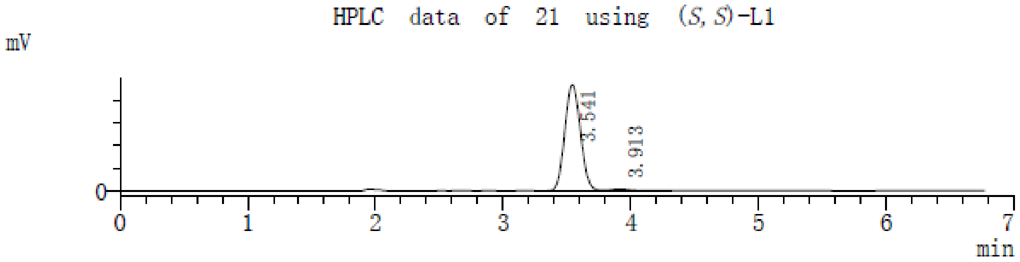

检测器A 214nm

Peak[#]	RetTime[min]	Height[uV]	Width[min]	Area[uV*s]	Area[%]
1	3.541	233234	0.135	2003376	97.776
2	3.913	3426	0.227	45558	2.224

Supplementary Figure 389. HPLC spectra for **21**

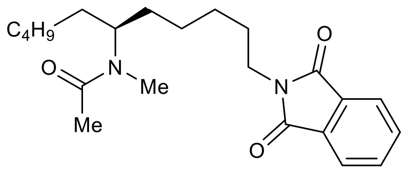

(*S*)-*N*-(1-(1,3-dioxoisoindolin-2-yl)undecan-6-yl)-*N*-methylacetamide (**22**)

HPLC analysis: The ee was determined to be 90% on a CHIRALPAK AD-H column (7% *i*PrOH in hexane, 0.7 mL/min, 40 °C); retention times for compound obtained using (*S*,*S*)-**L1**: 27.8 min (minor), 29.3 min (major).

HPLC data of 22 using race-L1

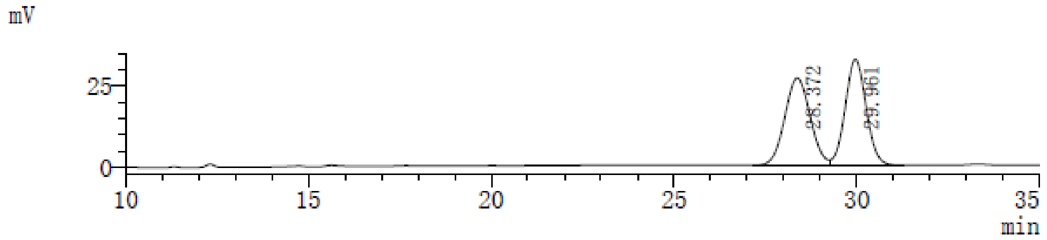

检测器A 214nm

Peak[#]	RetTime[min]	Height[uV]	Width[min]	Area[uV*s]	Area[%]
1	28.372	26826	0.756	1293889	49.809
2	29.961	32542	0.621	1303789	50.191

HPLC data of 22 using (*S*, *S*)-L1

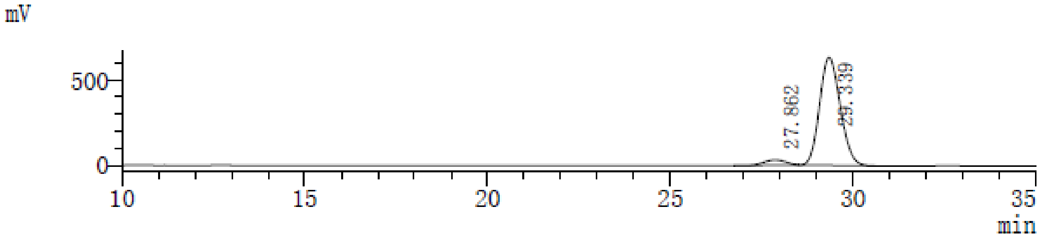

检测器A 214nm

Peak[#]	RetTime[min]	Height[uV]	Width[min]	Area[uV*s]	Area[%]
1	27.862	31668	0.673	1305051	4.999
2	29.339	632538	0.612	24799384	95.001

Supplementary Figure 390. HPLC spectra for **22**

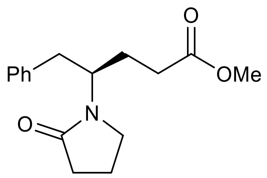

methyl (*R*)-4-(2-oxopyrrolidin-1-yl)-5-phenylpentanoate (**23**)

<u>HPLC analysis:</u> The ee was determined to be 91% on a CHIRALPAK AD-H column (15% *i*PrOH in hexane, 1.5 mL/min, 40 °C); retention times for compound obtained using (*S*,*S*)-**L1**: 4.7 min (minor), 5.4 min (major).

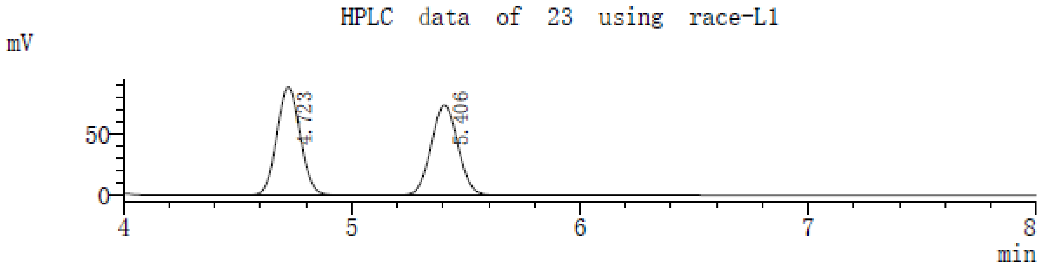

检测器A Ch1 214nm

Peak[#]	RetTime[min]	Height[uV]	Width[min]	Area[uV*s]	Area[%]
1	4.723	89180	0.112	639591	51.323
2	5.406	74236	0.127	606610	48.677

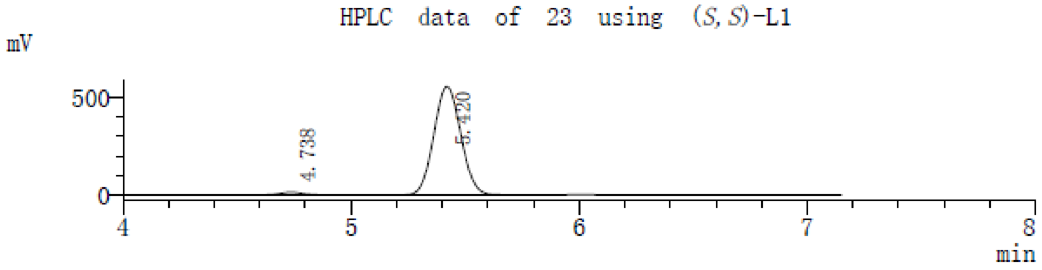

检测器A Ch1 214nm

Peak[#]	RetTime[min]	Height[uV]	Width[min]	Area[uV*s]	Area[%]
1	4.738	13537	0.112	96887	2.074
2	5.420	556158	0.129	4575080	97.926

Supplementary Figure 391. HPLC spectra for **23**

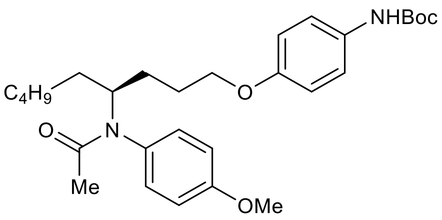

tert-butyl (*S*)-(4-((4-(*N*-(4-methoxyphenyl)acetamido)nonyl)oxy)phenyl)carbamate **(24)**

<u>HPLC analysis:</u> The ee was determined to be 96% on a CHIRALPAK AD-H column (10% *i*PrOH in hexane, 1.0 mL/min, 40 °C); retention times for compound obtained using (*S*,*S*)-**L1**: 17.8 min (minor), 30.9 min (major).

HPLC data of 24 using race-L1

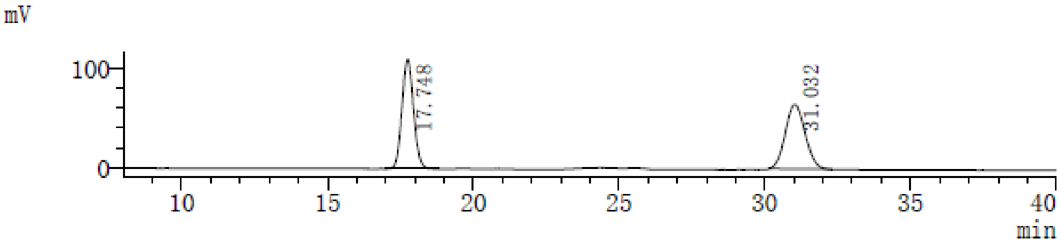

检测器A Ch1 214nm

Peak[#]	RetTime[min]	Height[uV]	Width[min]	Area[uV*s]	Area[%]
1	17.748	109608	0.431	3040446	49.980
2	31.032	64412	0.743	3042841	50.020

HPLC data of 24 using (*S*, *S*)-L1

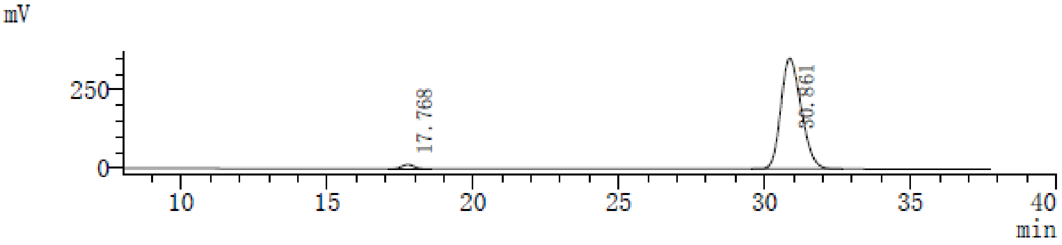

检测器A Ch1 214nm

Peak[#]	RetTime[min]	Height[uV]	Width[min]	Area[uV*s]	Area[%]
1	17.768	12354	0.430	341432	1.960
2	30.861	351861	0.757	17074276	98.040

Supplementary Figure 392. HPLC spectra for **24**

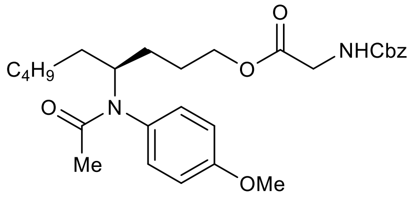

(*S*)-4-(*N*-(4-methoxyphenyl)acetamido)nonyl ((benzyloxy)carbonyl)glycinate (**25**)

HPLC analysis: The ee was determined to be 95% on a CHIRALPAK AD-H column (15% *i*PrOH in hexane, 1.5 mL/min, 40 °C); retention times for compound obtained using (*S*,*S*)-**L1**: 16.5 min (minor), 13.2 min (major).

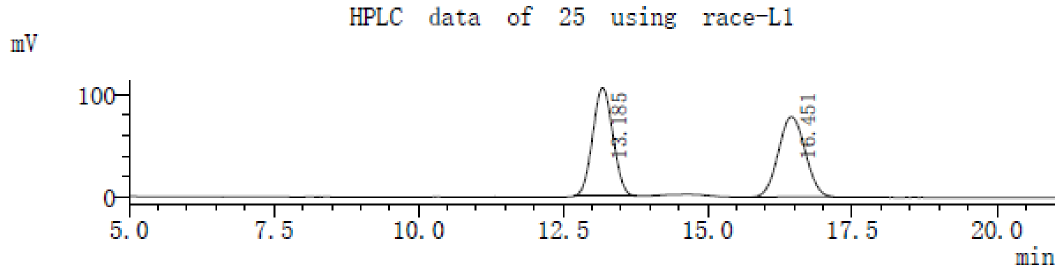

检测器A Ch1 214nm

Peak[#]	RetTime[min]	Height[uV]	Width[min]	Area[uV*s]	Area[%]
1	13.185	106037	0.387	2597567	50.437
2	16.451	78890	0.511	2552531	49.563

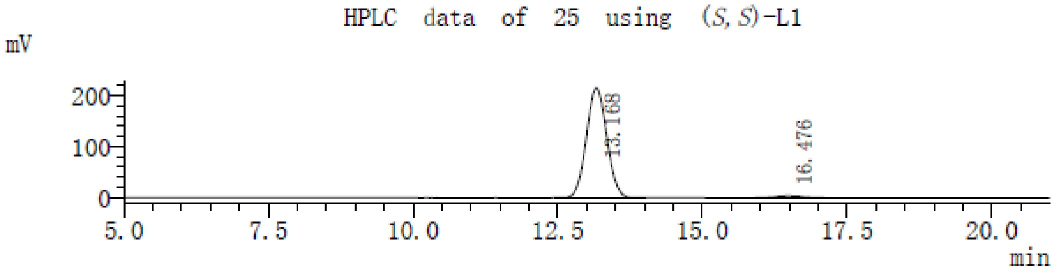

检测器A Ch1 214nm

Peak[#]	RetTime[min]	Height[uV]	Width[min]	Area[uV*s]	Area[%]
1	13.168	214603	0.387	5288342	97.542
2	16.476	3847	0.547	133251	2.458

Supplementary Figure 393. HPLC spectra for **25**

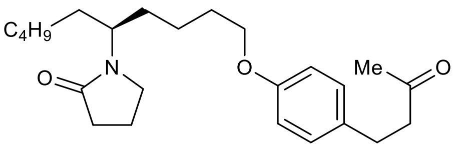

(*S*)-1-(1-(4-(3-oxobutyl)phenoxy)decan-5-yl)pyrrolidin-2-one (**26**)

HPLC analysis: The ee was determined to be 96% on a CHIRALCEL OD-H column (15% *i*PrOH in hexane, 1.5 mL/min, 40 °C); retention times for compound obtained using (*S*,*S*)-**L1**: 5.9 min (minor), 8.3 min (major).

HPLC data of 26 using race-L1

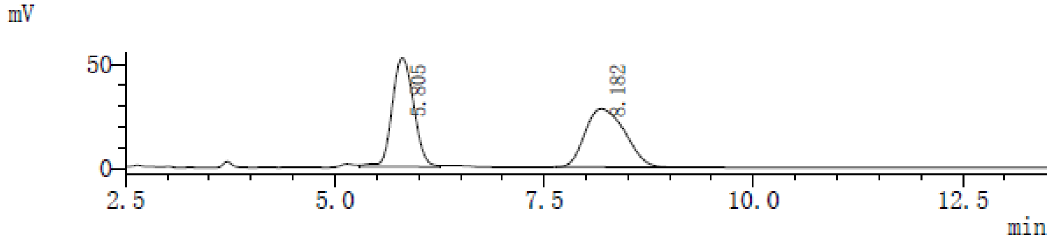

检测器A 214nm

Peak[#]	RetTime[min]	Height[uV]	Width[min]	Area[uV*s]	Area[%]
1	5.805	52156	0.278	929176	49.952
2	8.182	28014	0.543	930960	50.048

HPLC data of 26 using (*S*, *S*)-L1

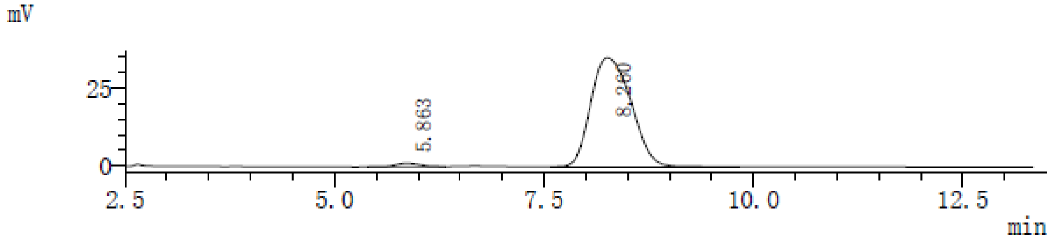

检测器A 214nm

Peak[#]	RetTime[min]	Height[uV]	Width[min]	Area[uV*s]	Area[%]
1	5.863	1146	0.296	21556	1.814
2	8.260	34968	0.541	1166694	98.186

Supplementary Figure 394. HPLC spectra for **26**

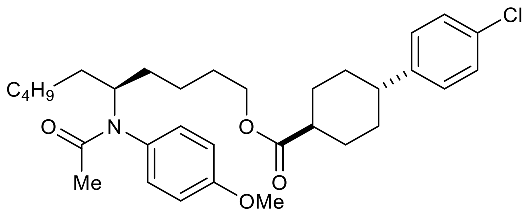

(S)-5-(N-(4-methoxyphenyl)acetamido)decyl (1r,4S)-4-(4-chlorophenyl)cyclohexane-1-carboxylate (**27**)

HPLC analysis: The ee was determined to be 96% on a CHIRALPAK AD-H column (7% iPrOH in hexane, 0.7 mL/min, 40 °C); retention times for compound obtained using (S,S)-**L1**: 22.4 min (minor), 23.6 min (major).

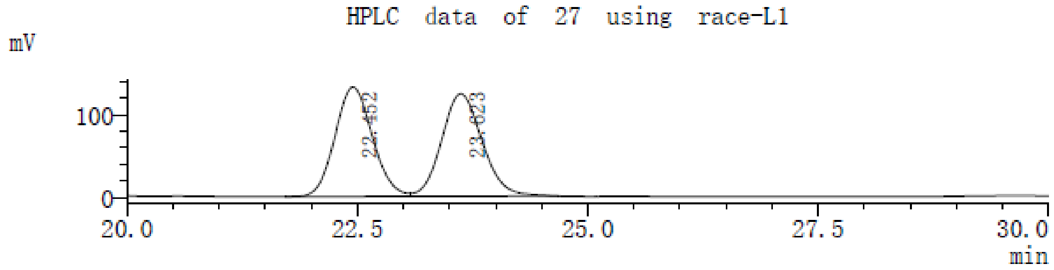

检测器A Ch1 214nm

Peak[#]	RetTime[min]	Height[uV]	Width[min]	Area[uV*s]	Area[%]
1	22.452	132478	0.439	3744224	49.642
2	23.623	124127	0.469	3798234	50.358

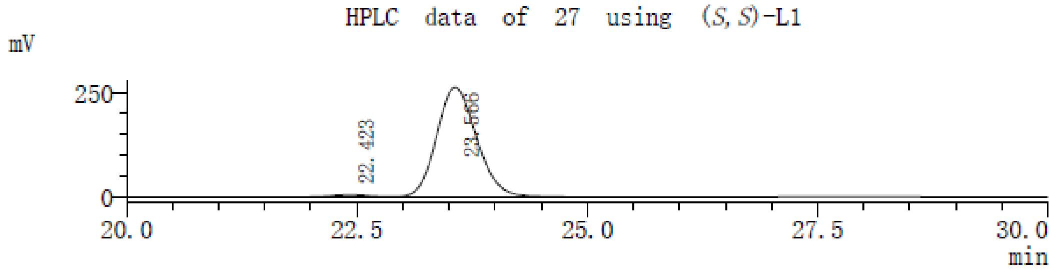

检测器A Ch1 214nm

Peak[#]	RetTime[min]	Height[uV]	Width[min]	Area[uV*s]	Area[%]
1	22.423	5165	0.436	143893	1.766
2	23.566	262771	0.469	8002147	98.234

Supplementary Figure 395. HPLC spectra for **27**

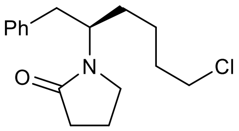

(*R*)-1-(6-chloro-1-phenylhexan-2-yl)pyrrolidin-2-one (**28**)

<u>HPLC analysis:</u> The ee was determined to be 98% on a CHIRALPAK AS-H column (15% *i*PrOH in hexane, 1.5 mL/min, 40 °C); retention times for compound obtained using (*S*,*S*)-**L1**: 2.9 min (minor), 3.8 min (major).

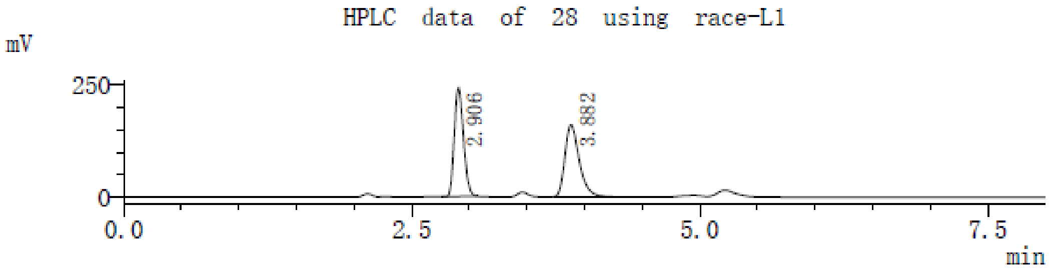

检测器A Ch1 214nm

Peak[#]	RetTime[min]	Height[uV]	Width[min]	Area[uV*s]	Area[%]
1	2.906	241722	0.088	1352199	49.406
2	3.882	160192	0.131	1384718	50.594

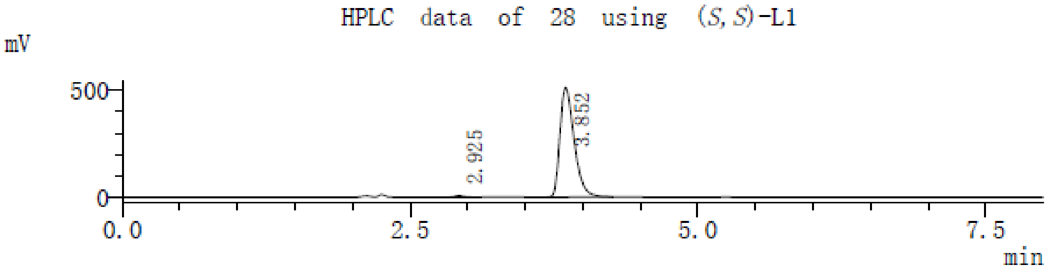

检测器A Ch1 214nm

Peak[#]	RetTime[min]	Height[uV]	Width[min]	Area[uV*s]	Area[%]
1	2.925	7338	0.082	38102	0.855
2	3.852	511894	0.132	4420016	99.145

Supplementary Figure 396. HPLC spectra for **28**

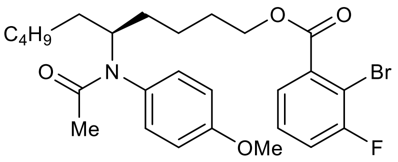

(*S*)-5-(*N*-(4-methoxyphenyl)acetamido)decyl 2-bromo-3-fluorobenzoate (**29**)

HPLC analysis: The ee was determined to be 96% on a CHIRALPAK AD-H column (10% *i*PrOH in hexane, 1.0 mL/min, 40 °C); retention times for compound obtained using (*S*,*S*)-**L1**: 14.3 min (minor), 11.4 min (major).

HPLC data of 29 using race-L1

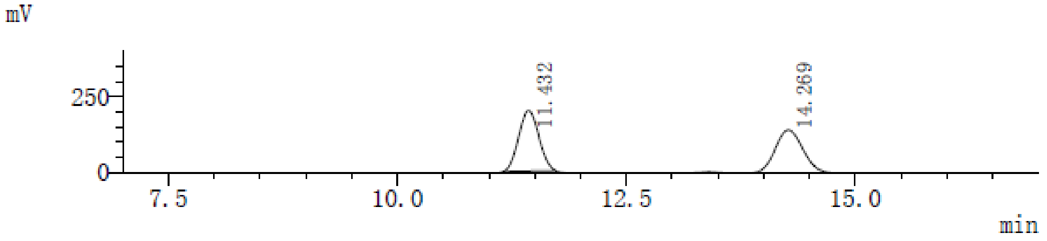

检测器A Ch1 214nm

Peak[#]	RetTime[min]	Height[uV]	Width[min]	Area[uV*s]	Area[%]
1	11.432	201061	0.242	3057160	50.714
2	14.269	142470	0.328	2971061	49.286

HPLC data of 29 using (*S*, *S*)-L1

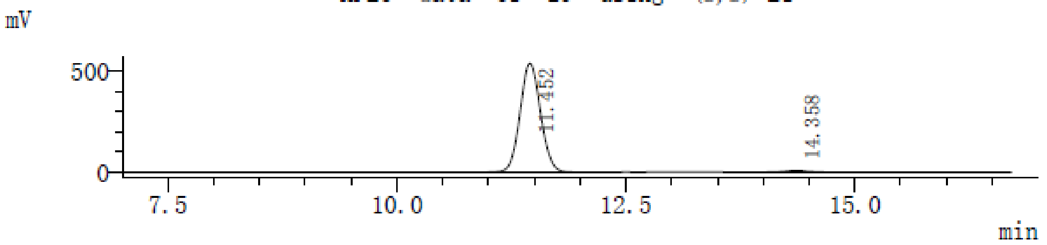

检测器A Ch1 214nm

Peak[#]	RetTime[min]	Height[uV]	Width[min]	Area[uV*s]	Area[%]
1	11.452	536083	0.241	8353631	98.144
2	14.358	7992	0.314	157942	1.856

Supplementary Figure 397. HPLC spectra for **29**

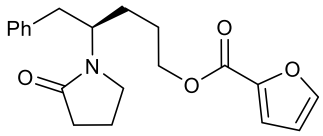

(*R*)-4-(2-oxopyrrolidin-1-yl)-5-phenylpentyl furan-2-carboxylate (**30**)

<u>HPLC analysis:</u> The ee was determined to be 97% on a CHIRALPAK AS-H column (15% *i*PrOH in hexane, 1.5 mL/min, 40 °C); retention times for compound obtained using (*S*,*S*)-**L1**: 4.4 min (minor), 7.1 min (major).

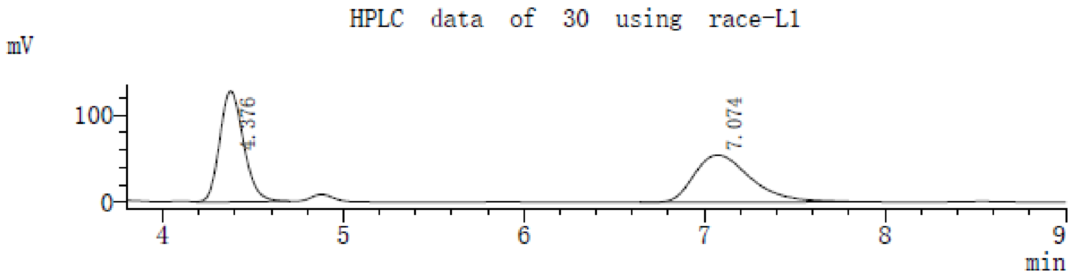

检测器A Ch1 214nm

Peak[#]	RetTime[min]	Height[uV]	Width[min]	Area[uV*s]	Area[%]
1	4.376	127347	0.141	1173165	49.863
2	7.074	54157	0.331	1179620	50.137

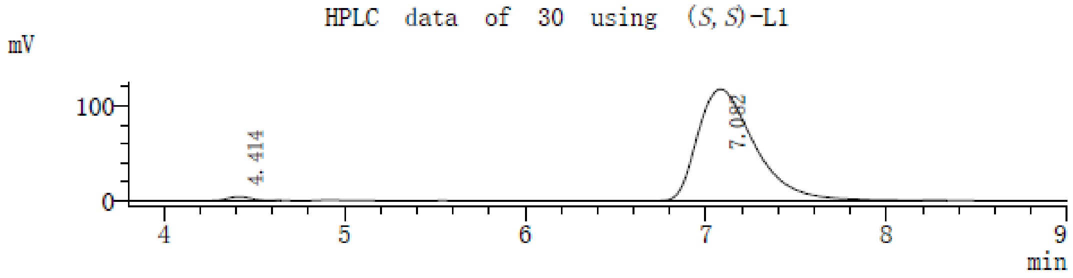

检测器A Ch1 214nm

Peak[#]	RetTime[min]	Height[uV]	Width[min]	Area[uV*s]	Area[%]
1	4.414	4475	0.137	39657	1.510
2	7.082	117447	0.332	2587402	98.490

Supplementary Figure 398. HPLC spectra for **30**

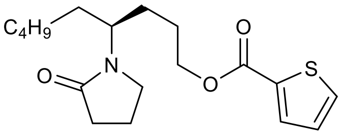

(*S*)-4-(2-oxopyrrolidin-1-yl)nonyl thiophene-2-carboxylate (**31**)

<u>HPLC analysis:</u> The ee was determined to be 97% on a CHIRALCEL OD-H column (15% *i*PrOH in hexane, 1.5 mL/min, 40 °C); retention times for compound obtained using (*S*,*S*)-**L1**: 4.1 min (minor), 4.8 min (major).

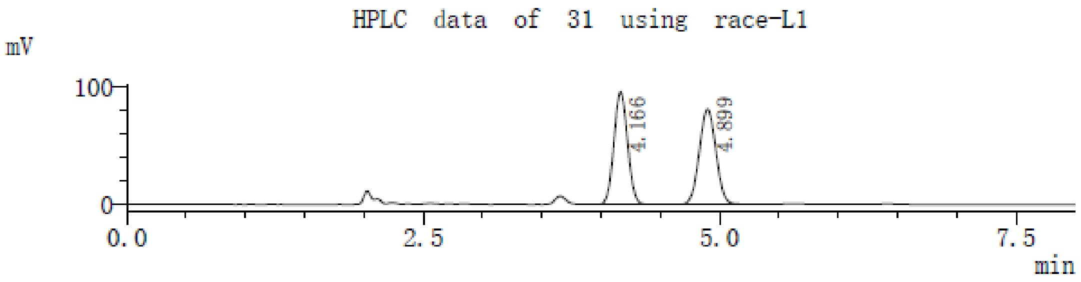

检测器A Ch1 214nm

Peak[#]	RetTime[min]	Height[uV]	Width[min]	Area[uV*s]	Area[%]
1	4.166	96162	0.126	775583	49.100
2	4.899	81724	0.153	804014	50.900

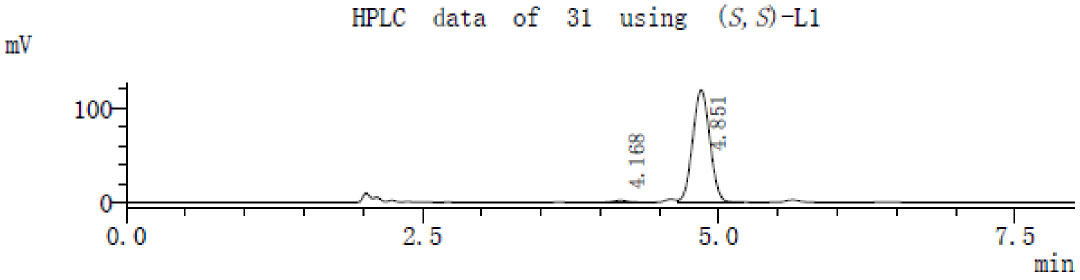

检测器A Ch1 214nm

Peak[#]	RetTime[min]	Height[uV]	Width[min]	Area[uV*s]	Area[%]
1	4.168	2324	0.125	18685	1.527
2	4.851	119043	0.159	1205004	98.473

Supplementary Figure 399. HPLC spectra for **31**

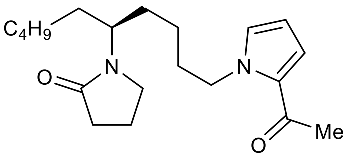

(*S*)-1-(1-(2-acetyl-1*H*-pyrrol-1-yl)decan-5-yl)pyrrolidin-2-one (**32**)

HPLC analysis: The ee was determined to be 97% on a CHIRALCEL OD-H column (15% *i*PrOH in hexane, 1.5 mL/min, 40 °C); retention times for compound obtained using (*S*,*S*)-**L1**: 4.1 min (minor), 3.6 min (major).

HPLC data of 32 using race-L1

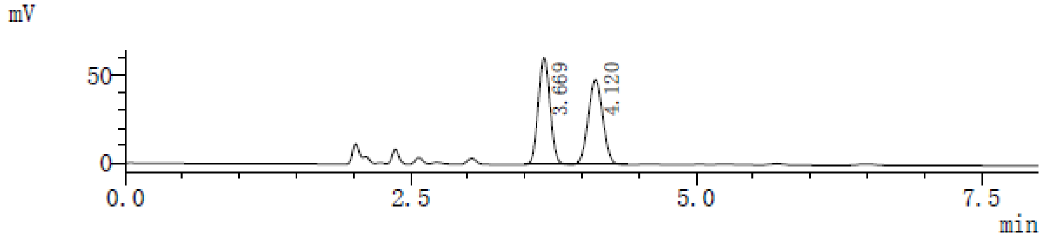

检测器A Ch1 214nm

Peak[#]	RetTime[min]	Height[uV]	Width[min]	Area[uV*s]	Area[%]
1	3.669	61032	0.112	439082	50.578
2	4.120	48305	0.139	429053	49.422

HPLC data of 32 using (*S*, *S*)-L1

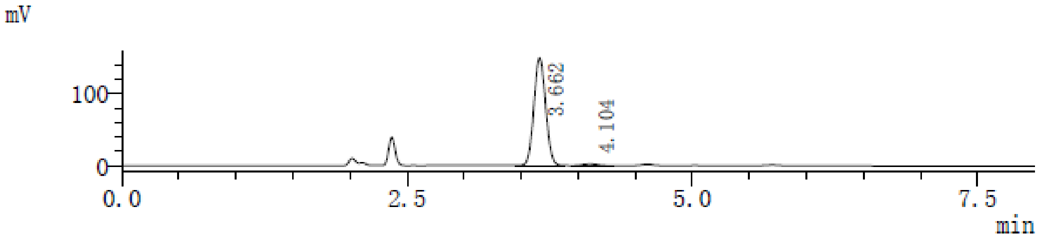

检测器A Ch1 214nm

Peak[#]	RetTime[min]	Height[uV]	Width[min]	Area[uV*s]	Area[%]
1	3.662	149642	0.113	1082959	98.345
2	4.104	2078	0.141	18228	1.655

Supplementary Figure 400. HPLC spectra for **32**

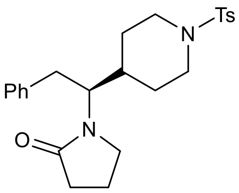

(*S*)-1-(2-phenyl-1-(1-tosylpiperidin-4-yl)ethyl)pyrrolidin-2-one (**33**)

HPLC analysis: The ee was determined to be 87% on a CHIRALCEL OD-H column (15% *i*PrOH in hexane, 1.5 mL/min, 40 °C); retention times for compound obtained using (*S*,*S*)-**L1**: 20.2 min (minor), 17.5 min (major).

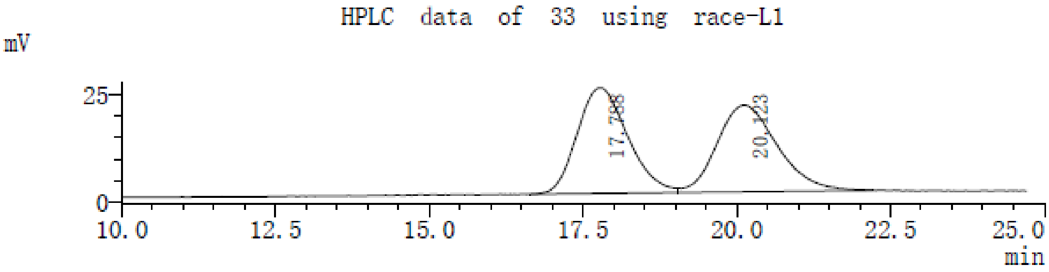

检测器A Ch1 214nm

Peak[#]	RetTime[min]	Height[uV]	Width[min]	Area[uV*s]	Area[%]
1	17.788	24469	0.890	1399487	50.501
2	20.123	20066	1.044	1371722	49.499

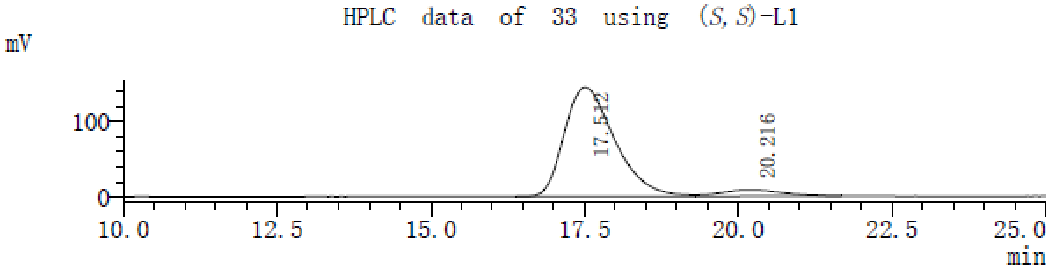

检测器A Ch1 214nm

Peak[#]	RetTime[min]	Height[uV]	Width[min]	Area[uV*s]	Area[%]
1	17.512	143869	0.875	8185752	93.691
2	20.216	8133	1.062	551171	6.309

Supplementary Figure 401. HPLC spectra for **33**

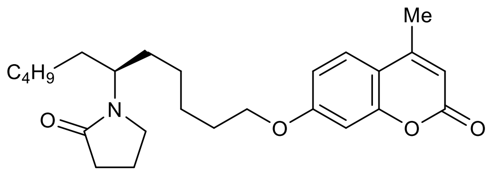

(*S*)-1-(1-((4-methyl-2-oxo-2*H*-chromen-7-yl)oxy)undecan-6-yl)pyrrolidin-2-one (**34**)

<u>HPLC analysis:</u> The ee was determined to be 96% on a CHIRALCEL OD-H column (15% *i*PrOH in hexane, 1.5 mL/min, 40 °C); retention times for compound obtained using (*S*,*S*)-**L1**: 14.7 min (minor), 12.7 min (major).

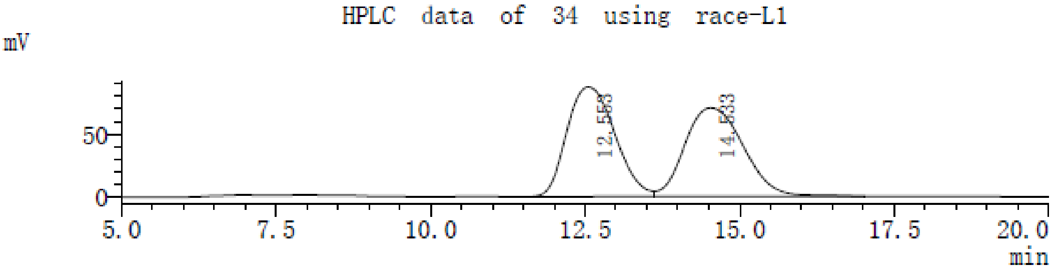

检测器A 214nm

Peak[#]	RetTime[min]	Height[uV]	Width[min]	Area[uV*s]	Area[%]
1	12.553	86700	0.866	4673706	50.399
2	14.533	70074	1.043	4599619	49.601

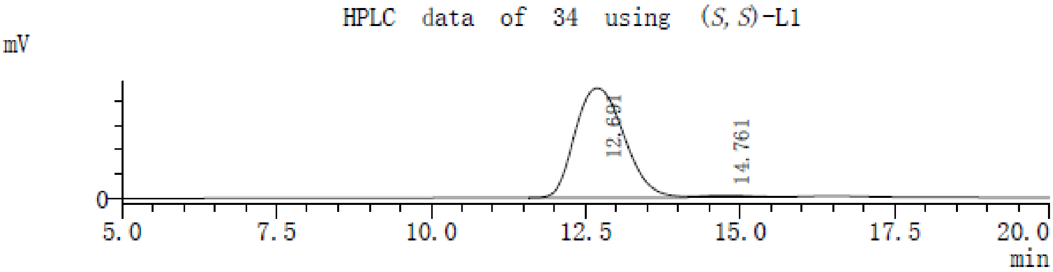

检测器A 214nm

Peak[#]	RetTime[min]	Height[uV]	Width[min]	Area[uV*s]	Area[%]
1	12.691	89466	0.879	4939564	97.930
2	14.761	1559	--	104432	2.070

Supplementary Figure 402. HPLC spectra for **34**

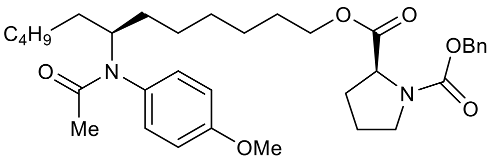

1-benzyl 2-((*S*)-7-(*N*-(4-methoxyphenyl)acetamido)dodecyl) (*S*)-pyrrolidine-1,2-dicarboxylate (**35**)

<u>HPLC analysis:</u> The dr was determined to be 97:3 on a CHIRALCEL OD-H column (7% *i*PrOH in hexane, 0.7 mL/min, 40 °C); retention times for compound obtained using (*S*,*S*)-**L1**: 49.1 min (minor), 44.6 min (major).

HPLC data of 35.36 using race-L1

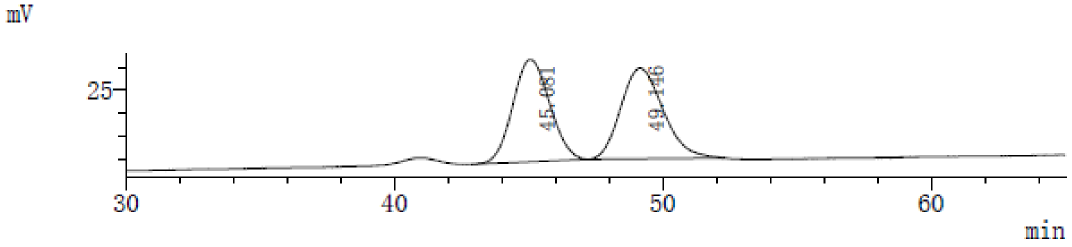

检测器A Ch1 214nm

Peak[#]	RetTime[min]	Height[uV]	Width[min]	Area[uV*s]	Area[%]
1	45.081	22359	1.506	2133877	49.544
2	49.146	19803	1.701	2173188	50.456

HPLC data of 35 using (*S*, *S*)-L1

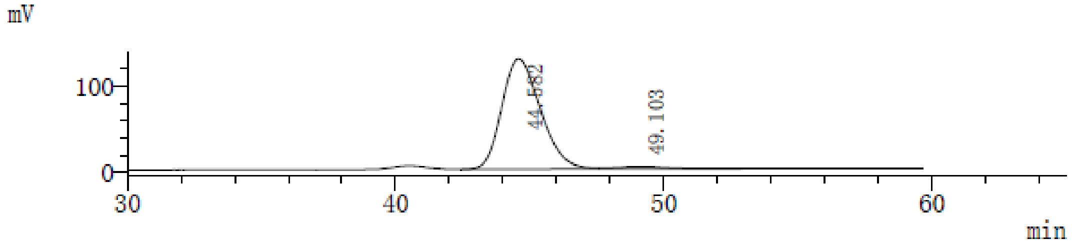

检测器A Ch1 214nm

Peak[#]	RetTime[min]	Height[uV]	Width[min]	Area[uV*s]	Area[%]
1	44.582	127463	1.559	12829478	97.105
2	49.103	2832	2.122	382450	2.895

HPLC data of 36 using (*R*, *R*)-L1

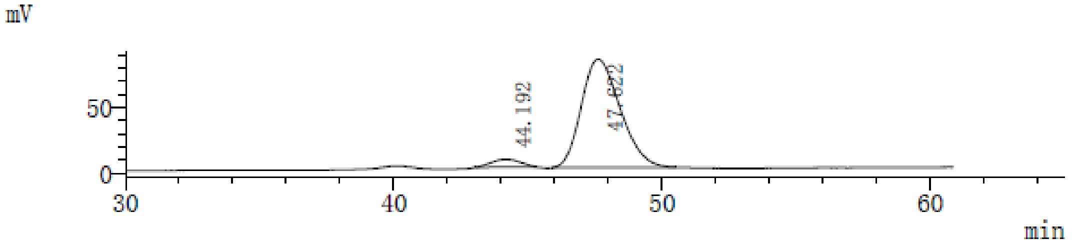

检测器A Ch1 214nm

Peak[#]	RetTime[min]	Height[uV]	Width[min]	Area[uV*s]	Area[%]
1	44.192	5960	1.237	441413	5.048
2	47.622	82397	1.575	8303337	94.952

Supplementary Figure 403. HPLC spectra for **35**

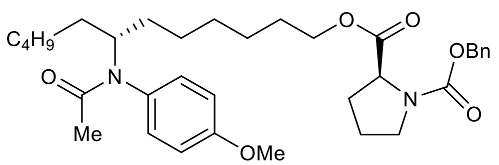

1-benzyl 2-((*R*)-7-(*N*-(4-methoxyphenyl)acetamido)dodecyl) (*S*)-pyrrolidine-1,2-dicarboxylate (**36**)

HPLC analysis: The dr was determined to be 5:95 on a CHIRALCEL OD-H column (7% *i*PrOH in hexane, 0.7 mL/min, 40 °C); retention times for compound obtained using (*R*,*R*)-**L1**: 44.2 min (minor), 47.6 min (major).

HPLC data of 35.36 using race-L1

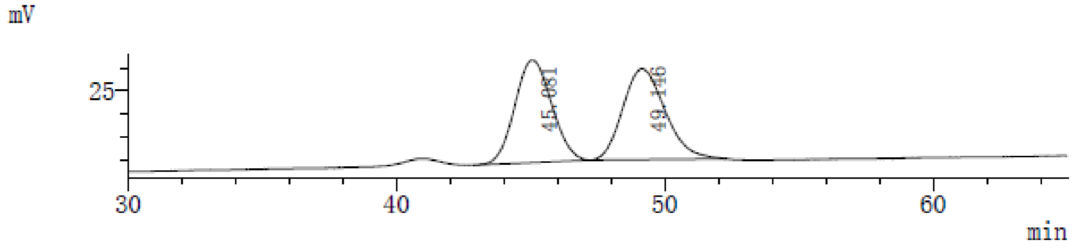

检测器A Ch1 214nm

Peak[#]	RetTime[min]	Height[uV]	Width[min]	Area[uV*s]	Area[%]
1	45.081	22359	1.506	2133877	49.544
2	49.146	19803	1.701	2173188	50.456

HPLC data of 35 using (*S*, *S*)-L1

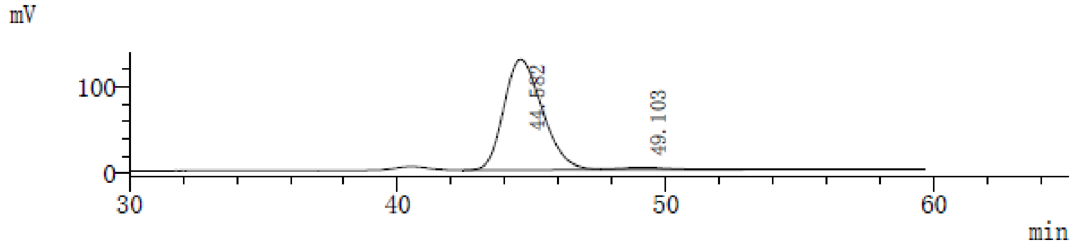

检测器A Ch1 214nm

Peak[#]	RetTime[min]	Height[uV]	Width[min]	Area[uV*s]	Area[%]
1	44.582	127463	1.559	12829478	97.105
2	49.103	2832	2.122	382450	2.895

HPLC data of 36 using (*R*, *R*)-L1

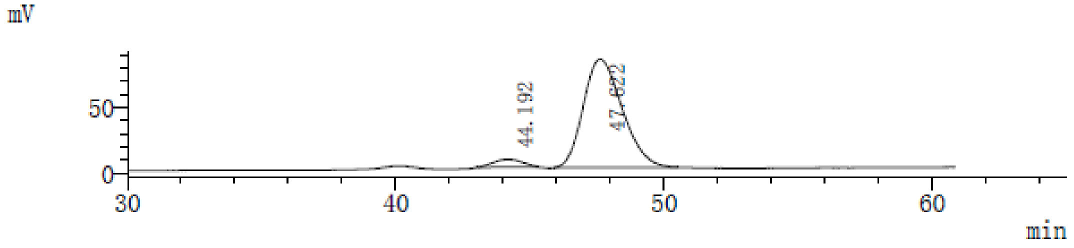

检测器A Ch1 214nm

Peak[#]	RetTime[min]	Height[uV]	Width[min]	Area[uV*s]	Area[%]
1	44.192	5960	1.237	441413	5.048
2	47.622	82397	1.575	8303337	94.952

Supplementary Figure 404. HPLC spectra for **36**

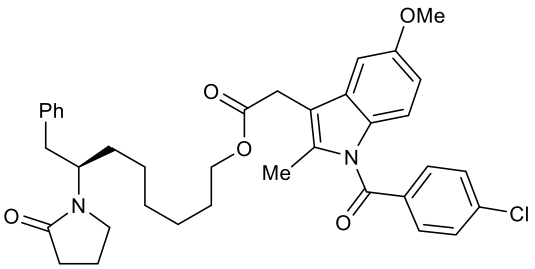

(*R*)-7-(2-oxopyrrolidin-1-yl)-8-phenyloctyl 2-(1-(4-chlorobenzoyl)-5-methoxy-2-methyl-1*H*-indol-3-yl)acetate (**37**)

<u>HPLC analysis:</u> The ee was determined to be 97% on a CHIRALPAK AS-H column (20% *i*PrOH in hexane, 2.0 mL/min, 40 °C); retention times for compound obtained using (*S*,*S*)-**L1**: 3.8 min (minor), 6.4 min (major).

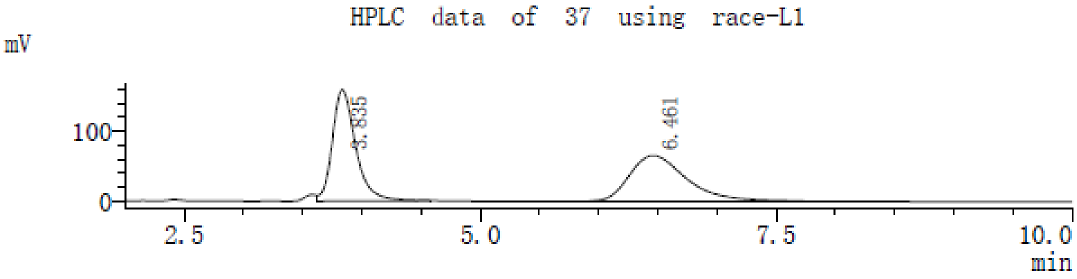

检测器A Ch1 214nm

Peak[#]	RetTime[min]	Height[uV]	Width[min]	Area[uV*s]	Area[%]
1	3.835	160182	0.202	2193746	50.068
2	6.461	65640	0.500	2187812	49.932

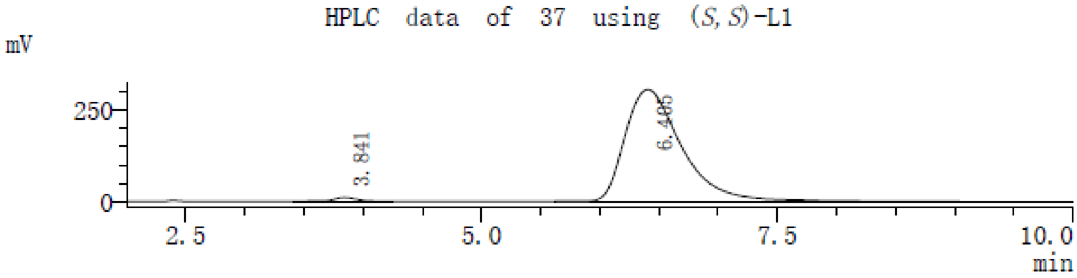

检测器A Ch1 214nm

Peak[#]	RetTime[min]	Height[uV]	Width[min]	Area[uV*s]	Area[%]
1	3.841	11138	0.200	156648	1.492
2	6.405	306952	0.497	10340919	98.508

Supplementary Figure 405. HPLC spectra for **37**

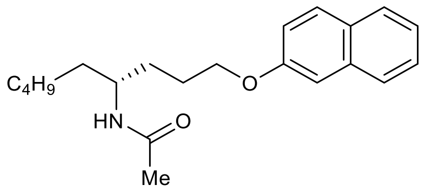

(*R*)-*N*-(1-(naphthalen-2-yloxy)nonan-4-yl)acetamide (**38**)

<u>HPLC analysis</u>: The ee was determined to be 90% on a CHIRALPAK AD-H column (10% *i*PrOH in hexane, 1.0 mL/min, 40 ^{o}C); retention times for compound obtained using (*S*)-**L2**: 6.7 min (minor), 7.1 min (major).

HPLC data of 38 using rac-L2

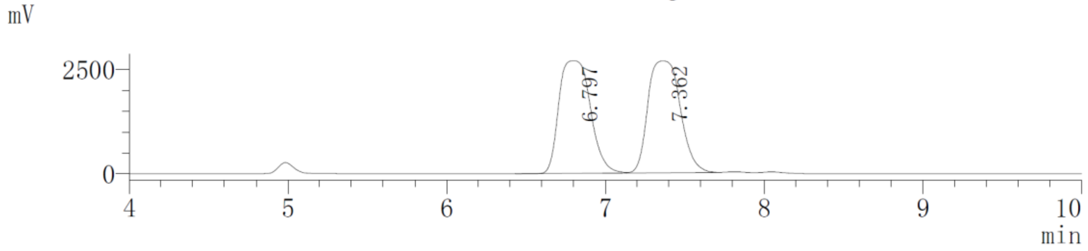

检测器A 214nm

Peak[#]	RetTime[min]	Height[uV]	Width[min]	Area[uV*s]	Area[%]
1	6.797	2704217	0.219	37174987	49.576
2	7.362	2681443	0.225	37811391	50.424

HPLC data of 38 using (S)-L2

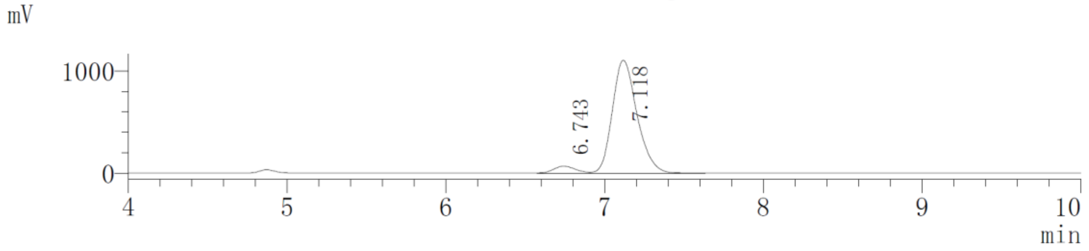

检测器A 214nm

Peak[#]	RetTime[min]	Height[uV]	Width[min]	Area[uV*s]	Area[%]
1	6.743	68356	0.147	640436	5.104
2	7.118	1104107	0.164	11906384	94.896

Supplementary Figure 406. HPLC spectra for **38**

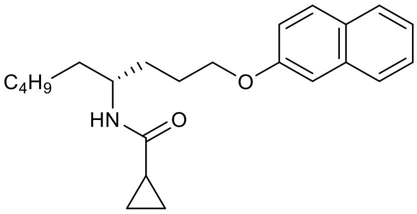

(*R*)-*N*-(1-(naphthalen-2-yloxy)nonan-4-yl)cyclopropanecarboxamide (**39**)

HPLC analysis: The ee was determined to be 93% on a CHIRALPAK IG column (8% *i*PrOH in hexane, 0.8 mL/min, 40 °C); retention times for compound obtained using (*S*)-**L2**: 24.4 min (major), 25.6 min (minor).

HPLC data of 39 using rac-L2

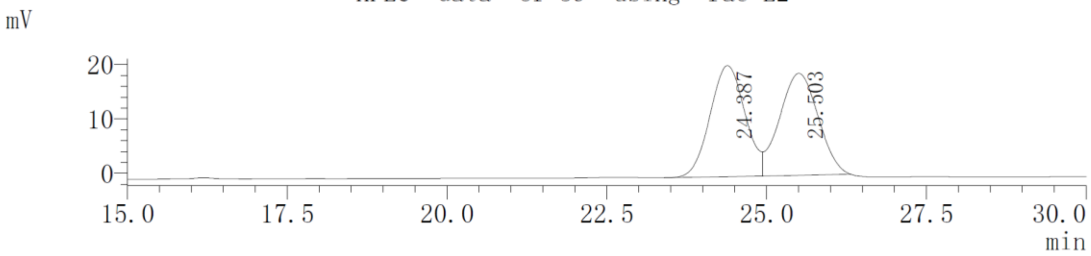

检测器A 214nm

Peak[#]	RetTime[min]	Height[uV]	Width[min]	Area[uV*s]	Area[%]
1	24.387	20371	0.602	777337	49.651
2	25.503	18723	0.672	788260	50.349

HPLC data of 39 using (S)-L2

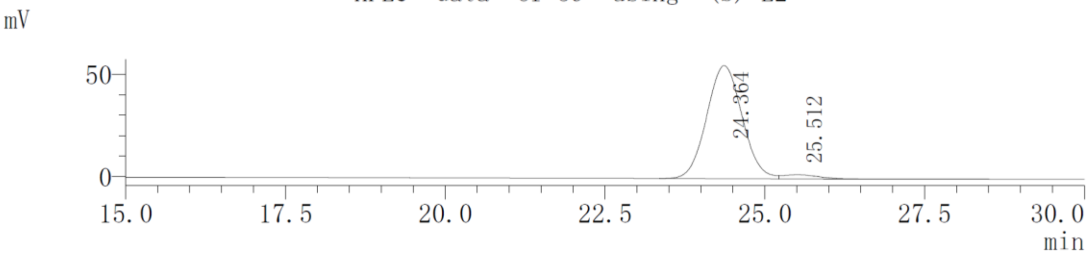

检测器A 214nm

Peak[#]	RetTime[min]	Height[uV]	Width[min]	Area[uV*s]	Area[%]
1	24.364	55346	0.599	2135186	96.631
2	25.512	1962	--	74440	3.369

Supplementary Figure 407. HPLC spectra for **39**

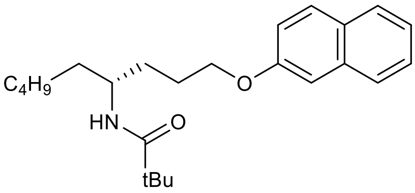

(*R*)-*N*-(1-(naphthalen-2-yloxy)nonan-4-yl)pivalamide (**40**)

<u>HPLC analysis</u>: The ee was determined to be 86% on a CHIRALCEL OJ-H column (10% *i*PrOH in hexane, 1.0 mL/min, 40 °C); retention times for compound obtained using (*S*)-**L2**: 12.8 min (major), 15.0 min (minor).

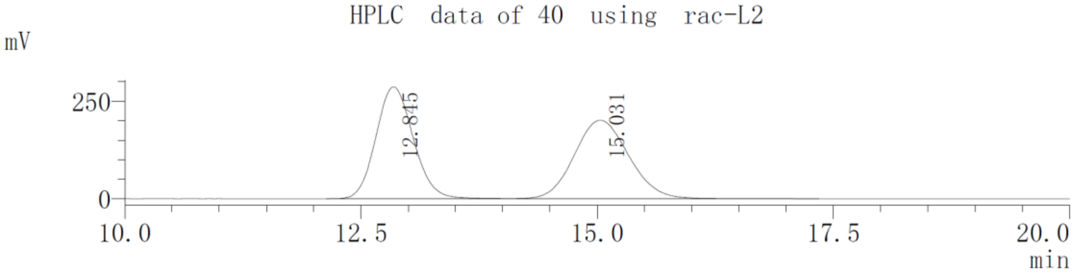

检测器A Ch1 214nm

Peak[#]	RetTime[min]	Height[uV]	Width[min]	Area[uV*s]	Area[%]
1	12.845	287527	0.425	7943554	49.391
2	15.031	201846	0.624	8139540	50.609

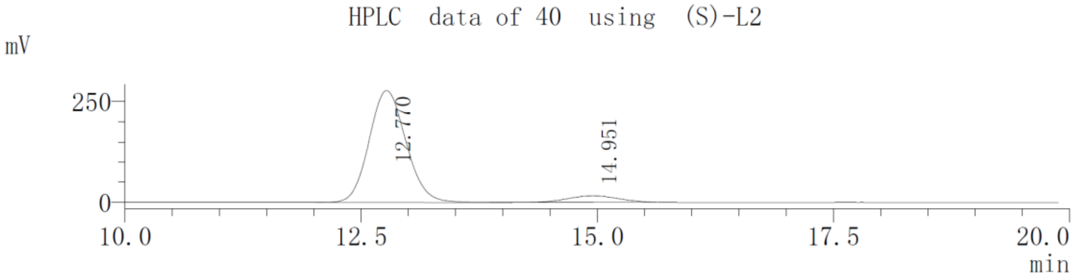

检测器A Ch1 214nm

Peak[#]	RetTime[min]	Height[uV]	Width[min]	Area[uV*s]	Area[%]
1	12.770	278491	0.420	7611909	92.757
2	14.951	16043	0.592	594365	7.243

Supplementary Figure 408. HPLC spectra for **40**

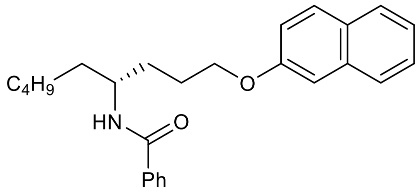

(*R*)-*N*-(1-(naphthalen-2-yloxy)nonan-4-yl)benzamide (**41**)

<u>HPLC analysis</u>: The ee was determined to be 86% on a CHIRALCEL OD-H column (20% *i*PrOH in hexane, 2.0 mL/min, 40 ^{o}C); retention times for compound obtained using (*S*)-**L2**: 8.5 min (minor), 9.6 min (major).

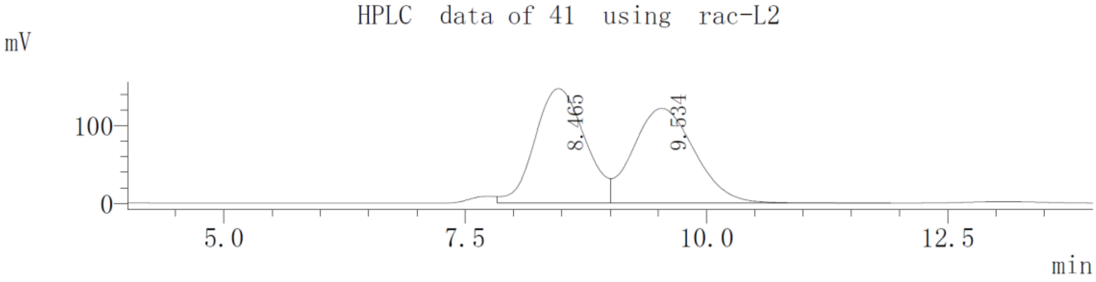

检测器A 214nm

Peak[#]	RetTime[min]	Height[uV]	Width[min]	Area[uV*s]	Area[%]
1	8.465	147157	0.570	5317060	49.710
2	9.534	121712	0.697	5379127	50.290

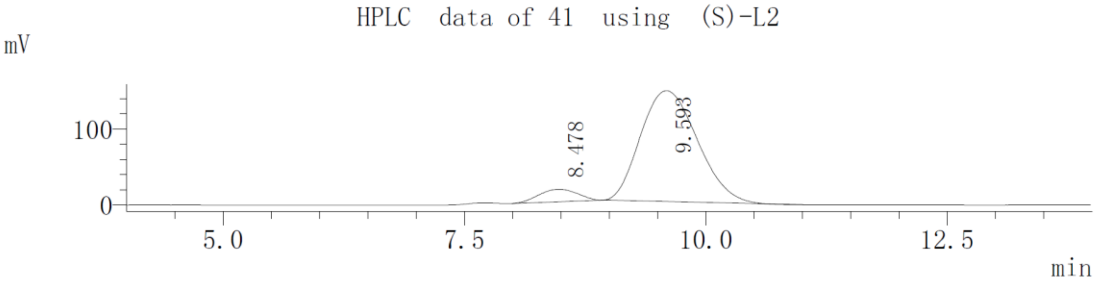

检测器A 214nm

Peak[#]	RetTime[min]	Height[uV]	Width[min]	Area[uV*s]	Area[%]
1	8.478	16460	0.461	457863	7.116
2	9.593	145855	0.655	5976061	92.884

Supplementary Figure 409. HPLC spectra for **41**

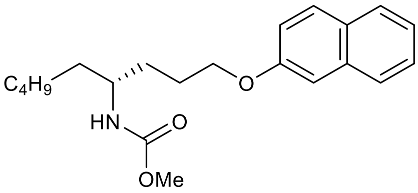

methyl (*R*)-(1-(naphthalen-2-yloxy)nonan-4-yl)carbamate (**42**)

<u>HPLC analysis</u>: The ee was determined to be 91% on a CHIRALPAK AS-H column (3% *i*PrOH in hexane, 0.5 mL/min, 40 °C); retention times for compound obtained using (*S*)-**L2**: 13.7 min (minor), 15.4 min (major).

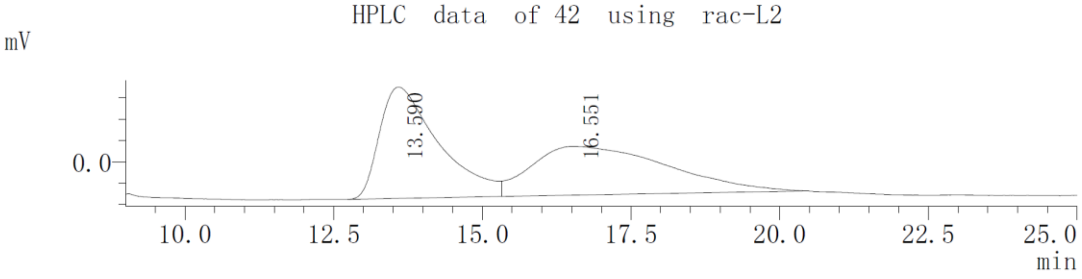

检测器A 254nm

Peak[#]	RetTime[min]	Height[uV]	Width[min]	Area[uV*s]	Area[%]
1	13.590	2609	1.018	180468	49.838
2	16.551	1147	2.575	181644	50.162

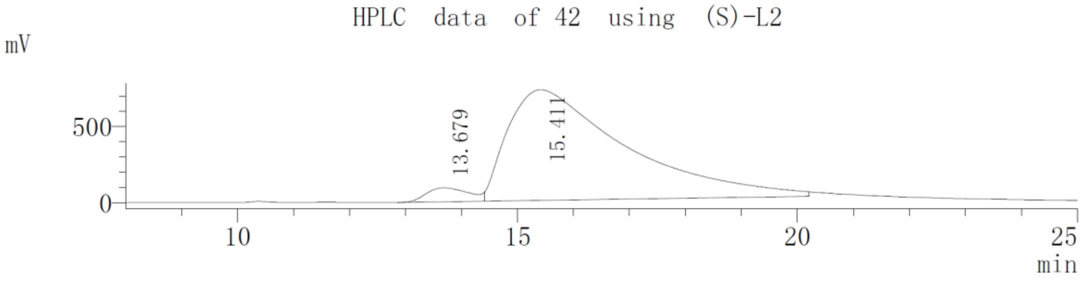

检测器A 214nm

Peak[#]	RetTime[min]	Height[uV]	Width[min]	Area[uV*s]	Area[%]
1	13.679	92164	0.930	4959064	4.441
2	15.411	728095	2.144	106713610	95.559

Supplementary Figure 410. HPLC spectra for **42**

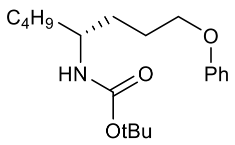

tert-butyl (*R*)-(1-phenoxyoctan-4-yl)carbamate (**43**)

<u>HPLC analysis</u>: The ee was determined to be 92% on a CHIRALPAK AD-H column (10% *i*PrOH in hexane, 1.0 mL/min, 40 °C); retention times for compound obtained using (*S*)-**L2**: 4.3 min (major), 4.8 min (minor).

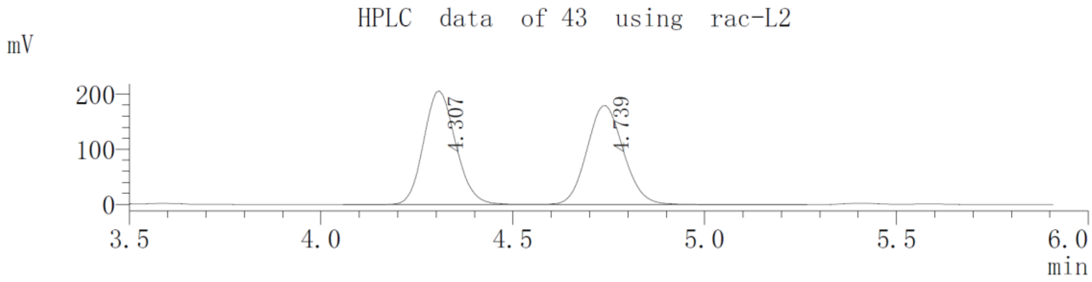

检测器A 214nm

Peak[#]	RetTime[min]	Height[uV]	Width[min]	Area[uV*s]	Area[%]
1	4.307	206447	0.089	1200642	50.204
2	4.739	179735	0.102	1190883	49.796

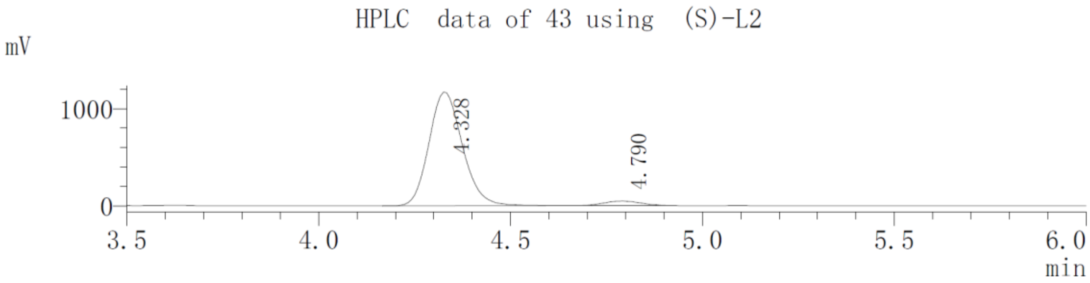

检测器A 214nm

Peak[#]	RetTime[min]	Height[uV]	Width[min]	Area[uV*s]	Area[%]
1	4.328	1171542	0.093	7094088	95.774
2	4.790	47063	0.105	313023	4.226

Supplementary Figure 411. HPLC spectra for **43**

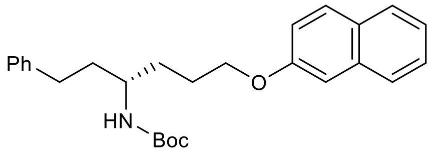

tert-butyl (*R*)-(6-(naphthalen-2-yloxy)-1-phenylhexan-3-yl)carbamate (**44**)

HPLC analysis: The ee was determined to be 93% on a CHIRALPAK AD-H column (15% *i*PrOH in hexane, 1.5 mL/min, 40 °C); retention times for compound obtained using (*S*)-**L2**: 4.4 min (major), 5.7 min (minor).

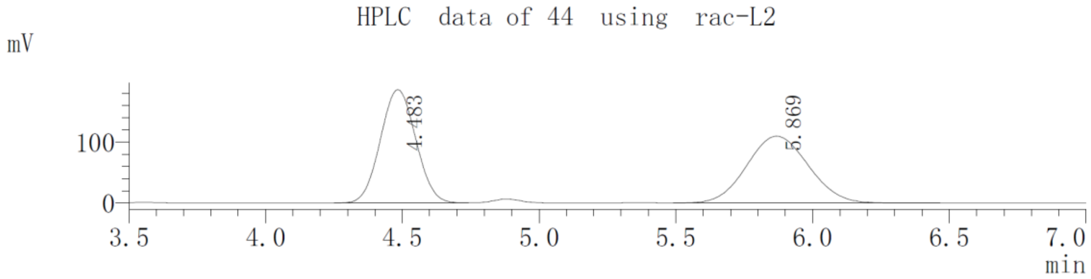

检测器A 214nm

Peak[#]	RetTime[min]	Height[uV]	Width[min]	Area[uV*s]	Area[%]
1	4.483	186535	0.142	1682584	49.364
2	5.869	109747	0.247	1725964	50.636

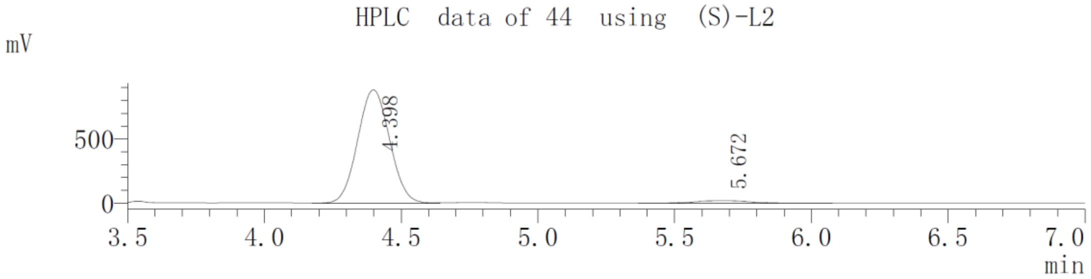

检测器A 214nm

Peak[#]	RetTime[min]	Height[uV]	Width[min]	Area[uV*s]	Area[%]
1	4.398	885518	0.128	7214612	96.434
2	5.672	20208	0.205	266758	3.566

Supplementary Figure 412. HPLC spectra for **44**

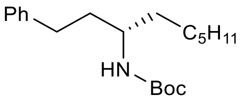

tert-butyl (*S*)-(1-phenylnonan-3-yl)carbamate (**45**)

<u>HPLC analysis</u>: The ee was determined to be 93% on a CHIRALCEL OD-H column (10% *i*PrOH in hexane, 1.0 mL/min, 40 °C); retention times for compound obtained using (*S*)-**L2**: 3.5 min (major), 3.9 min (minor).

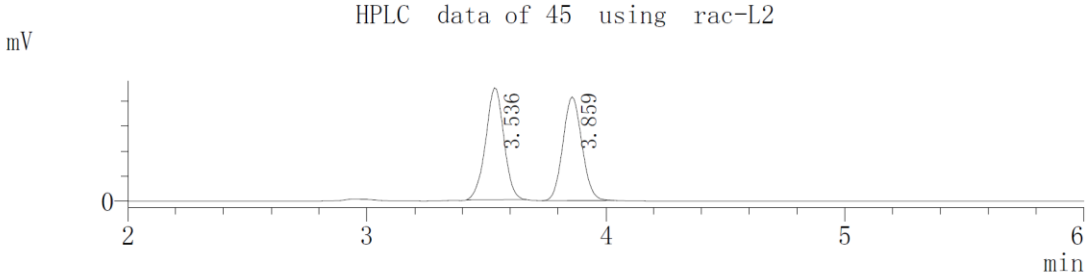

检测器A 214nm

Peak[#]	RetTime[min]	Height[uV]	Width[min]	Area[uV*s]	Area[%]
1	3.536	448780	0.081	2380481	50.901
2	3.859	415090	0.086	2296173	49.099

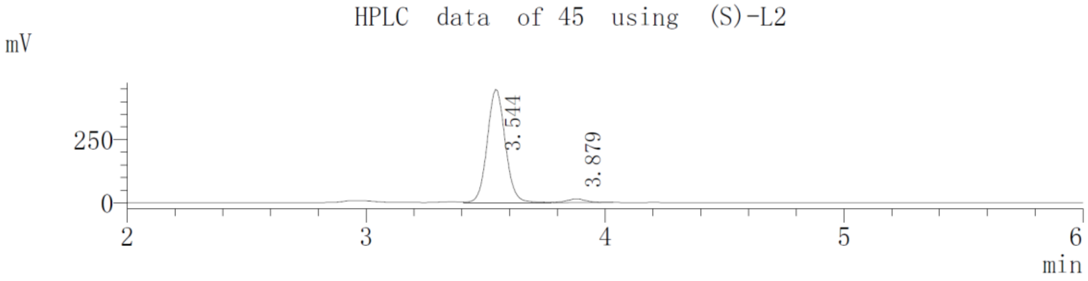

检测器A 214nm

Peak[#]	RetTime[min]	Height[uV]	Width[min]	Area[uV*s]	Area[%]
1	3.544	449160	0.080	2354964	96.645
2	3.879	13993	0.090	81757	3.355

Supplementary Figure 413. HPLC spectra for **45**

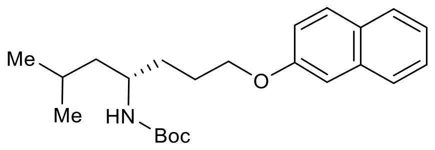

tert-butyl (*S*)-(6-methyl-1-(naphthalen-2-yloxy)heptan-4-yl)carbamate (**46**)

<u>HPLC analysis</u>: The ee was determined to be 93% on a CHIRALPAK AD-H column (10% *i*PrOH in hexane, 1.0 mL/min, 40 °C); retention times for compound obtained using (*S*)-**L2**: 5.6 min (major), 7.5 min (minor).

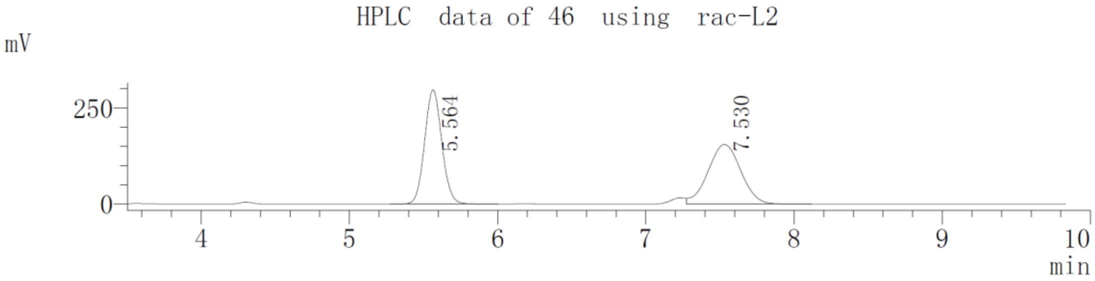

检测器A 214nm

Peak[#]	RetTime[min]	Height[uV]	Width[min]	Area[uV*s]	Area[%]
1	5.564	297754	0.124	2399006	50.184
2	7.530	155292	0.239	2381460	49.816

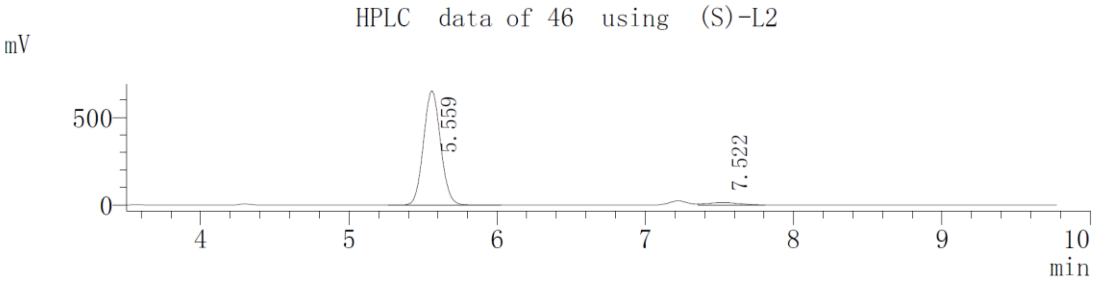

检测器A 214nm

Peak[#]	RetTime[min]	Height[uV]	Width[min]	Area[uV*s]	Area[%]
1	5.559	654524	0.124	5234185	96.496
2	7.522	13260	0.241	190079	3.504

Supplementary Figure 414. HPLC spectra for **46**

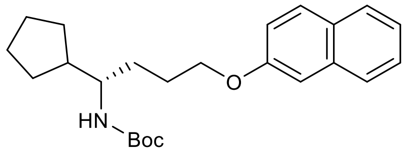

tert-butyl (*S*)-(1-cyclopentyl-4-(naphthalen-2-yloxy)butyl)carbamate (**47**)

HPLC analysis: The ee was determined to be 97% on a CHIRALPAK AD-H column (10% *i*PrOH in hexane, 1.0 mL/min, 40 °C); retention times for compound obtained using (*S*)-**L2**: 6.1 min (major), 9.0 min (minor).

HPLC data of 47 using rac-L2

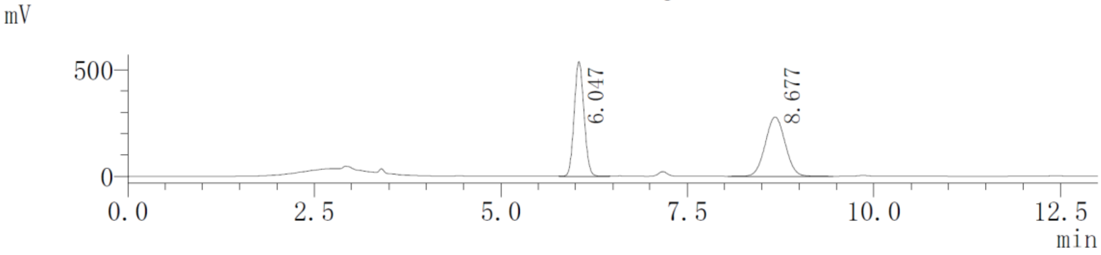

检测器A 214nm

Peak[#]	RetTime[min]	Height[uV]	Width[min]	Area[uV*s]	Area[%]
1	6.047	538674	0.143	4929139	48.899
2	8.677	278246	0.289	5151154	51.101

HPLC data of 47 using (S)-L2

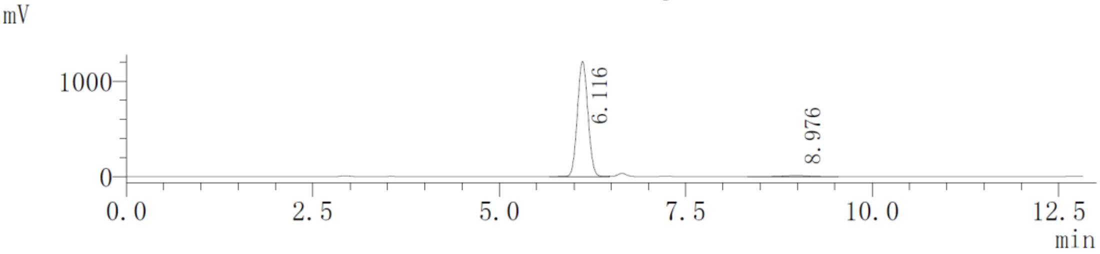

检测器A 214nm

Peak[#]	RetTime[min]	Height[uV]	Width[min]	Area[uV*s]	Area[%]
1	6.116	1207916	0.153	11835689	98.509
2	8.976	7861	0.353	179095	1.491

Supplementary Figure 415. HPLC spectra for **47**

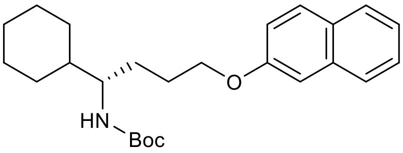

tert-butyl (*S*)-(1-cyclohexyl-4-(naphthalen-2-yloxy)butyl)carbamate (**48**)

<u>HPLC analysis</u>: The ee was determined to be 98% on a CHIRALCEL OD-H column (10% *i*PrOH in hexane, 1.0 mL/min, 40 °C); retention times for compound obtained using (*S*)-**L2**: 6.3 min (major), 7.3 min (minor).

HPLC data of 48 using rac-L2

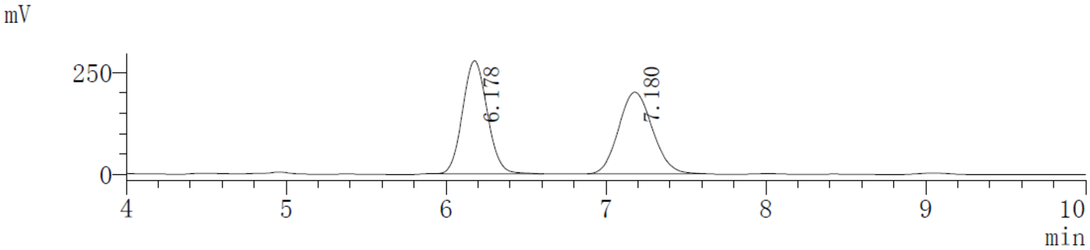

检测器A 214nm

Peak[#]	RetTime[min]	Height[uV]	Width[min]	Area[uV*s]	Area[%]
1	6.178	276700	0.166	2976045	50.370
2	7.180	199448	0.229	2932304	49.630

HPLC data of 48 using (S)-L2

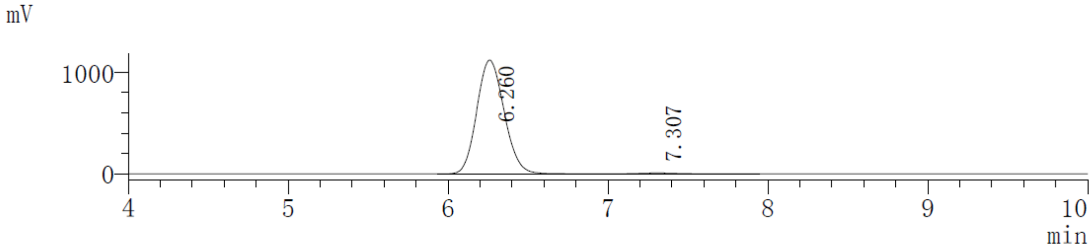

检测器A 214nm

Peak[#]	RetTime[min]	Height[uV]	Width[min]	Area[uV*s]	Area[%]
1	6.260	1121874	0.185	13376908	98.881
2	7.307	7954	0.268	151328	1.119

HPLC data of 48 using (S)-L2 at 0°C

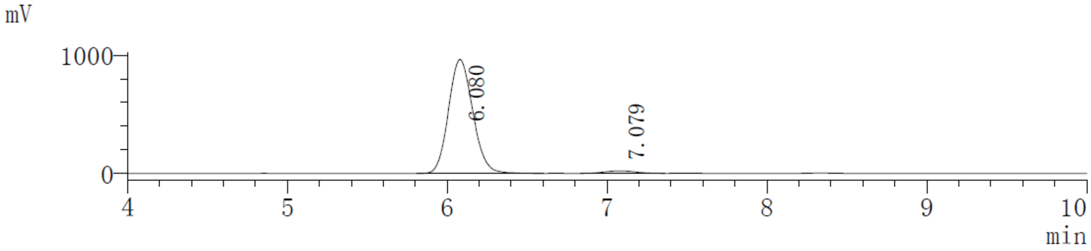

检测器A 214nm

Peak[#]	RetTime[min]	Height[uV]	Width[min]	Area[uV*s]	Area[%]
1	6.080	963158	0.164	10219943	97.307
2	7.079	20802	0.217	282874	2.693

Supplementary Figure 416. HPLC spectra for **48**

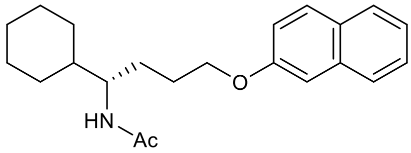

(*S*)-*N*-(1-cyclohexyl-4-(naphthalen-2-yloxy)butyl)acetamide (**49**)

<u>HPLC analysis</u>: The ee was determined to be 86% on a CHIRALPAK AD-H column (15% *i*PrOH in hexane, 1.5 mL/min, 40 ^{o}C); retention times for compound obtained using (*S*)-**L2**: 3.5 min (minor), 4.3 min (major).

HPLC data of 49 using rac-L2

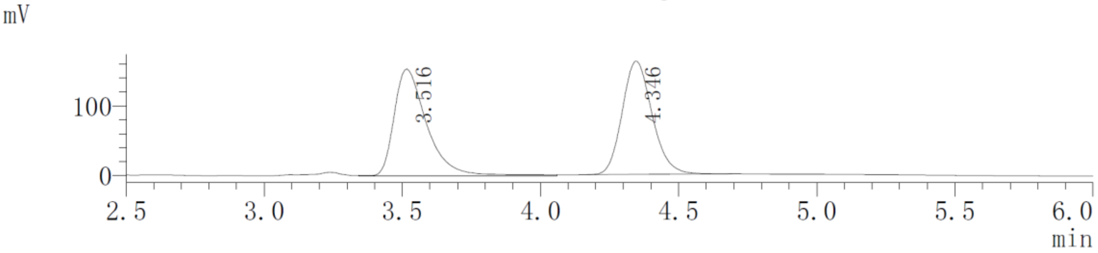

检测器A 214nm

Peak[#]	RetTime[min]	Height[uV]	Width[min]	Area[uV*s]	Area[%]
1	3.516	153345	0.120	1271156	50.916
2	4.346	162081	0.115	1225412	49.084

HPLC data of 49 using (S)-L2

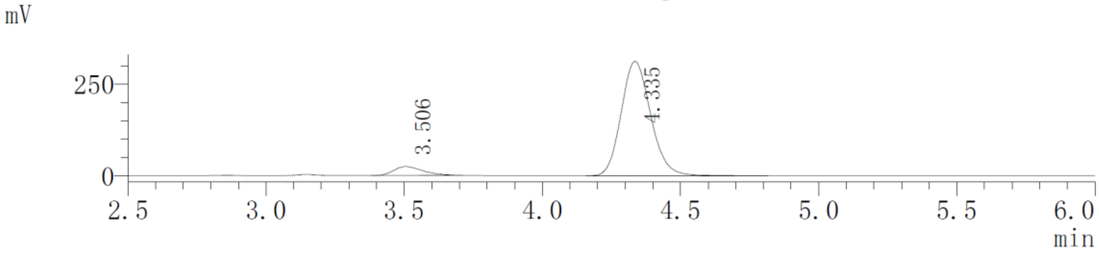

检测器A 214nm

Peak[#]	RetTime[min]	Height[uV]	Width[min]	Area[uV*s]	Area[%]
1	3.506	24239	0.114	179276	7.161
2	4.335	312875	0.114	2324234	92.839

Supplementary Figure 417. HPLC spectra for **49**

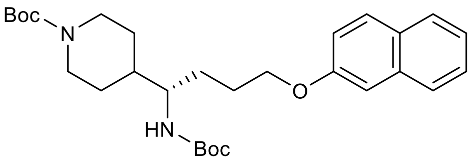

tert-butyl (*S*)-4-(1-((tert-butoxycarbonyl)amino)-4-(naphthalen-2-yloxy)butyl)piperidine-1-carboxylate (**50**)

<u>HPLC analysis</u>: The ee was determined to be 92% on a CHIRALPAK AD-H column (7% *i*PrOH in hexane, 0.7 mL/min, 40 ^{o}C); retention times for compound obtained using (*S*)-**L2**: 20.5 min (major), 35.6 min (minor).

HPLC data of 50 using rac-L2

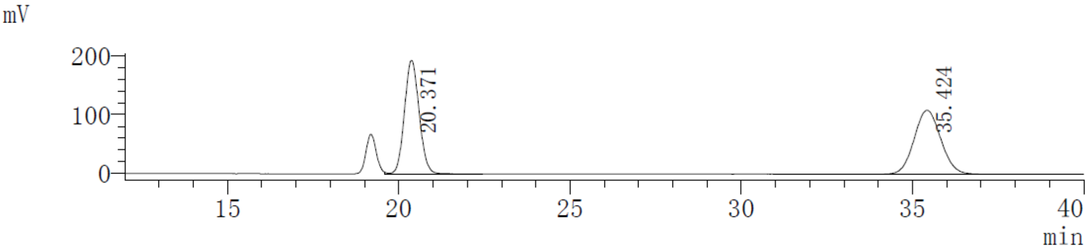

检测器A Ch1 214nm

Peak[#]	RetTime[min]	Height[uV]	Width[min]	Area[uV*s]	Area[%]
1	20.371	192995	0.462	5765588	49.691
2	35.424	108363	0.840	5837294	50.309

HPLC data of 50 using (S)-L2

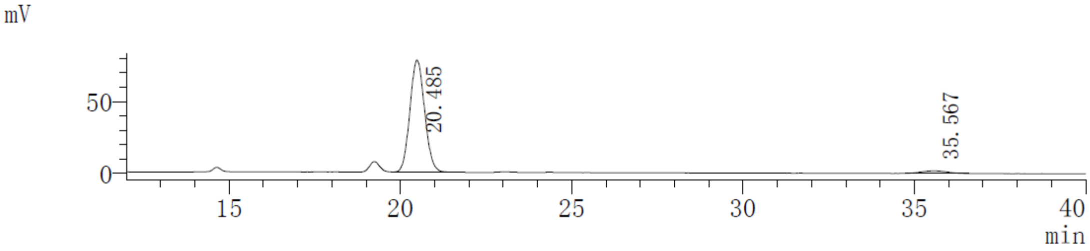

检测器A Ch1 214nm

Peak[#]	RetTime[min]	Height[uV]	Width[min]	Area[uV*s]	Area[%]
1	20.485	77891	0.465	2331987	96.155
2	35.567	1796	0.821	93260	3.845

HPLC data of 50 using (S)-L2 at 0℃

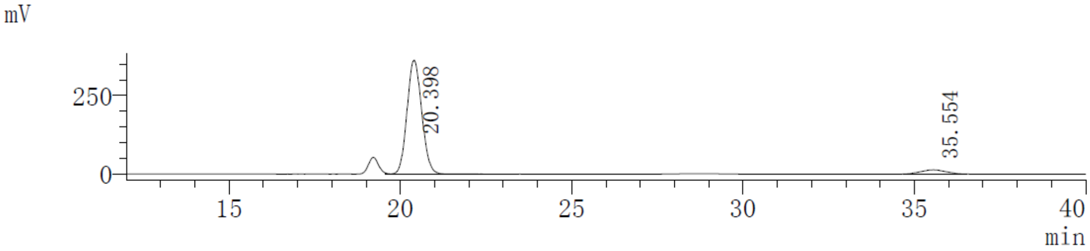

检测器A Ch1 214nm

Peak[#]	RetTime[min]	Height[uV]	Width[min]	Area[uV*s]	Area[%]
1	20.398	363082	0.463	10851695	94.343
2	35.554	12872	0.813	650654	5.657

Supplementary Figure 418. HPLC spectra for **50**

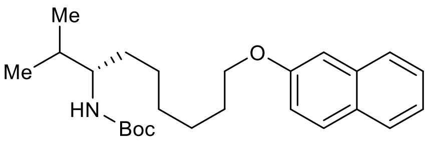

tert-butyl (*S*)-(2-methyl-9-(naphthalen-2-yloxy)nonan-3-yl)carbamate (**51**)

HPLC analysis: The ee was determined to be 98% on a CHIRALPAK AD-H column (10% *i*PrOH in hexane, 1.0 mL/min, 40 °C); retention times for compound obtained using (*S*)-**L2**: 5.2 min (major), 5.6 min (minor).

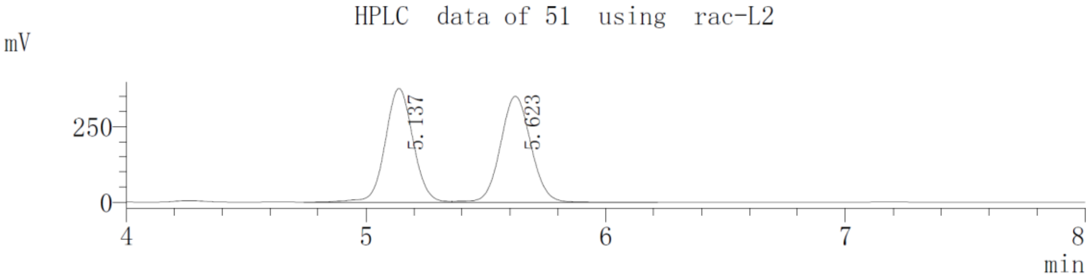

检测器A Ch1 214nm

Peak[#]	RetTime[min]	Height[uV]	Width[min]	Area[uV*s]	Area[%]
1	5. 137	376089	0. 124	3054927	49. 665
2	5. 623	350811	0. 135	3096110	50. 335

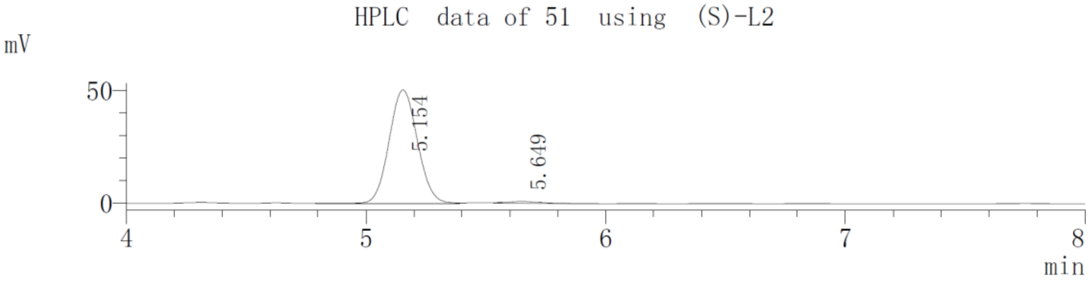

检测器A Ch1 214nm

Peak[#]	RetTime[min]	Height[uV]	Width[min]	Area[uV*s]	Area[%]
1	5. 154	50461	0. 128	417879	98. 250
2	5. 649	825	0. 145	7444	1. 750

Supplementary Figure 419. HPLC spectra for **51**

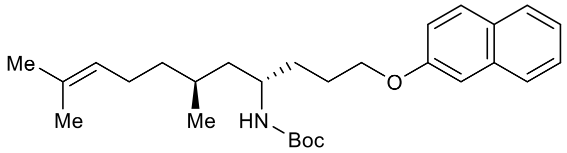

tert-butyl ((4*S*,6*S*)-6,10-dimethyl-1-(naphthalen-2-yloxy)undec-9-en-4-yl)carbamate (**52**)

HPLC analysis: The dr was determined to be 98:2 on a CHIRALPAK AD-H column (5% *i*PrOH in hexane, 0.5 mL/min, 40 °C); retention times for compound obtained using (*S*)-**L2**: 12.6 min (major), 15.3 min (minor).

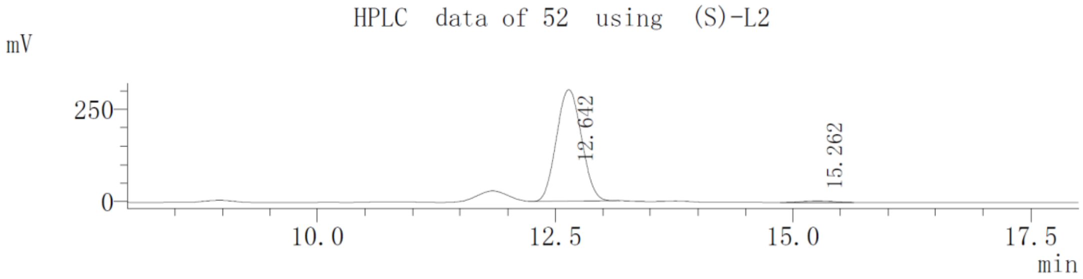

检测器A Ch1 214nm

Peak[#]	RetTime[min]	Height[uV]	Width[min]	Area[uV*s]	Area[%]
1	12.642	301464	0.292	5537708	98.500
2	15.262	4049	0.332	84334	1.500

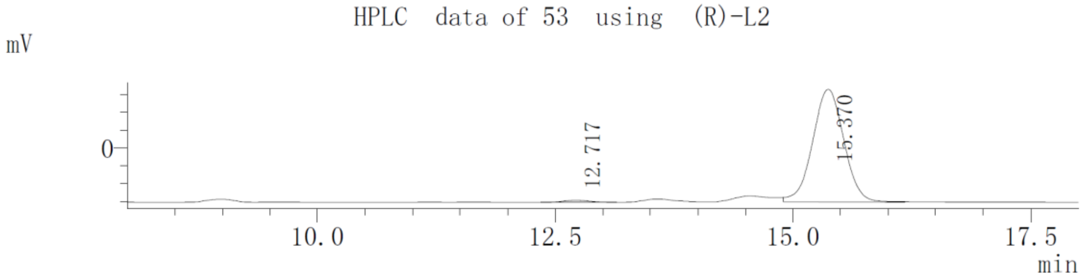

检测器A Ch1 214nm

Peak[#]	RetTime[min]	Height[uV]	Width[min]	Area[uV*s]	Area[%]
1	12.717	2460	0.296	45786	1.553
2	15.370	126711	0.350	2902078	98.447

Supplementary Figure 420. HPLC spectra for **52**

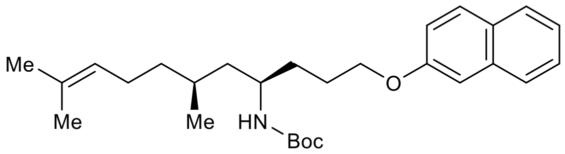

tert-butyl ((4*R*,6*S*)-6,10-dimethyl-1-(naphthalen-2-yloxy)undec-9-en-4-yl)carbamate (**53**)

<u>HPLC analysis</u>: The dr was determined to be 3:97 on a CHIRALPAK AD-H column (5% *i*PrOH in hexane, 0.5 mL/min, 40 °C); retention times for compound obtained using (*R*)-**L2**: 12.7 min (major), 15.4 min (minor).

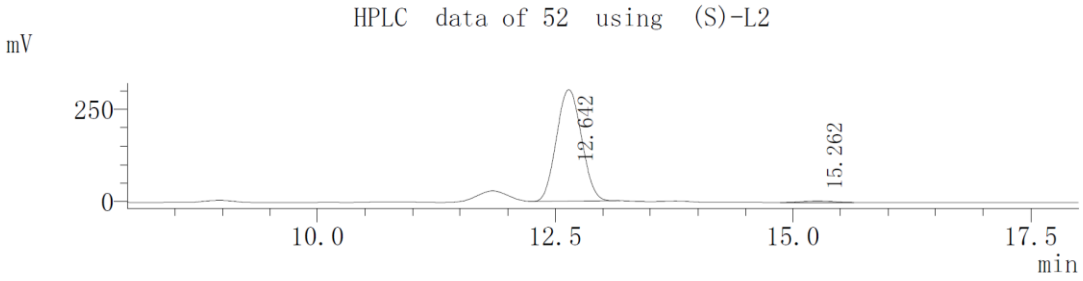

检测器A Ch1 214nm

Peak[#]	RetTime[min]	Height[uV]	Width[min]	Area[uV*s]	Area[%]
1	12. 642	301464	0. 292	5537708	98. 500
2	15. 262	4049	0. 332	84334	1. 500

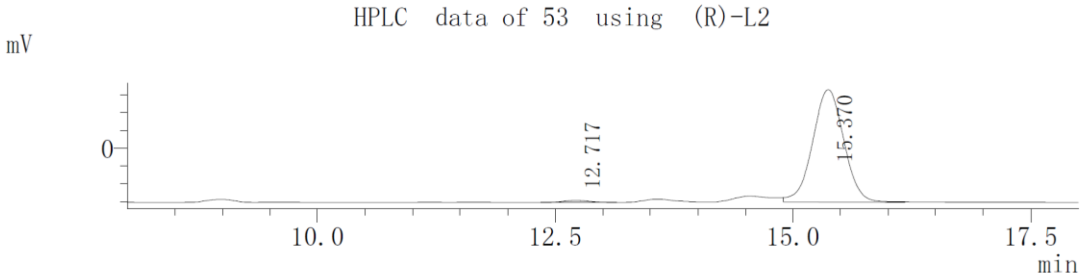

检测器A Ch1 214nm

Peak[#]	RetTime[min]	Height[uV]	Width[min]	Area[uV*s]	Area[%]
1	12. 717	2460	0. 296	45786	1. 553
2	15. 370	126711	0. 350	2902078	98. 447

Supplementary Figure 421. HPLC spectra for **53**

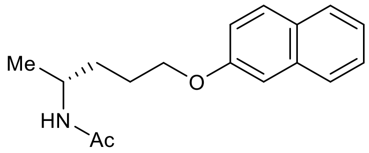

(*R*)-*N*-(5-(naphthalen-2-yloxy)pentan-2-yl)acetamide (**54**)

<u>HPLC analysis</u>: The ee was determined to be 85% on a CHIRALCEL OD-H column (20% *i*PrOH in hexane, 2.0 mL/min, 40 °C); retention times for compound obtained using (*S*)-**L2**: 4.1 min (major), 4.7 min (minor).

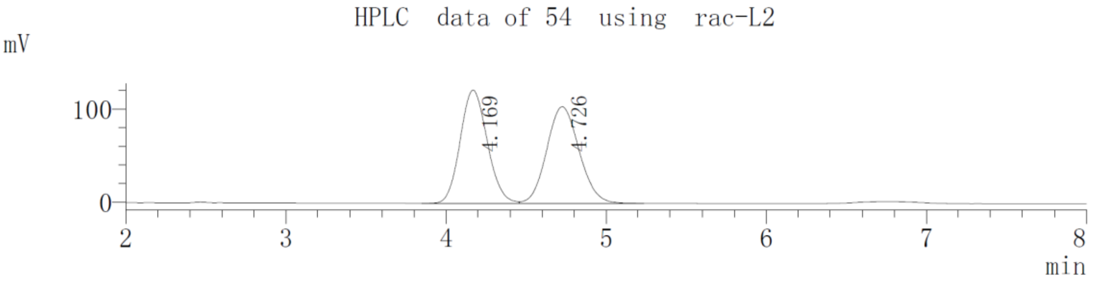

检测器A Ch1 214nm

Peak[#]	RetTime[min]	Height[uV]	Width[min]	Area[uV*s]	Area[%]
1	4.169	121605	0.183	1430871	49.538
2	4.726	103757	0.218	1457559	50.462

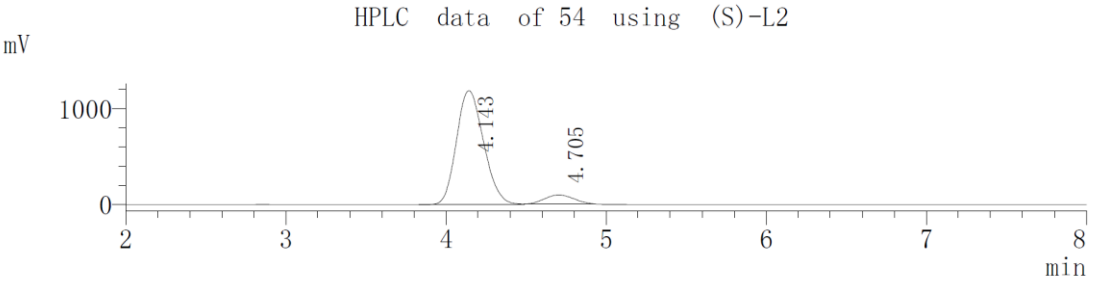

检测器A Ch1 214nm

Peak[#]	RetTime[min]	Height[uV]	Width[min]	Area[uV*s]	Area[%]
1	4.143	1185814	0.184	14021974	92.404
2	4.705	92891	0.201	1152665	7.596

Supplementary Figure 422. HPLC spectra for **54**

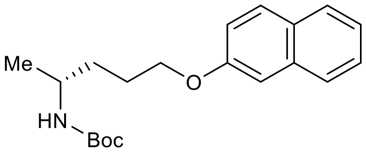

tert-butyl (*R*)-(5-(naphthalen-2-yloxy)pentan-2-yl)carbamate (**55**)

HPLC analysis: The ee was determined to be 88% on a CHIRALPAK AD-H column (15% *i*PrOH in hexane, 1.5 mL/min, 40 ^{o}C); retention times for compound obtained using (*S*)-**L2**: 3.4 min (major), 3.8min (minor).

HPLC data of 55 using rac-L2

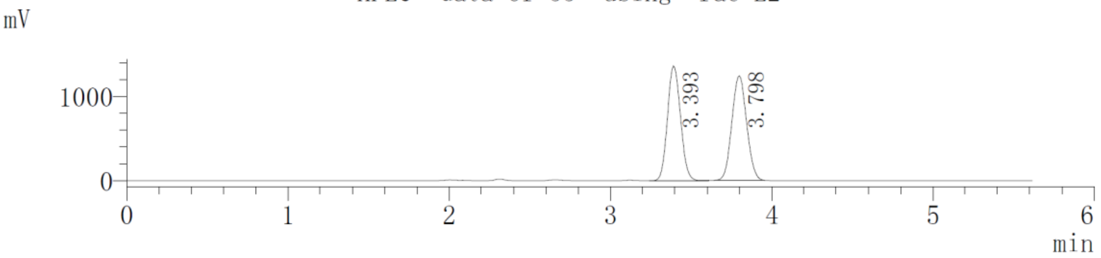

检测器A Ch1 214nm

Peak[#]	RetTime[min]	Height[uV]	Width[min]	Area[uV*s]	Area[%]
1	3.393	1363676	0.088	7646062	49.239
2	3.798	1240460	0.100	7882349	50.761

HPLC data of 55 using (S)-L2

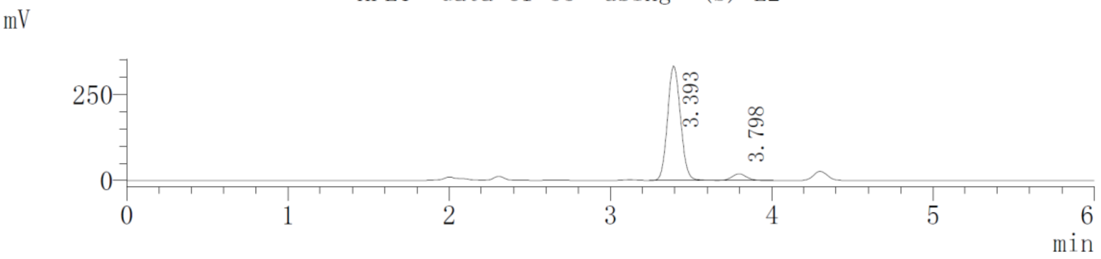

检测器A Ch1 214nm

Peak[#]	RetTime[min]	Height[uV]	Width[min]	Area[uV*s]	Area[%]
1	3.393	332465	0.087	1857531	93.862
2	3.798	19179	0.098	121479	6.138

Supplementary Figure 423. HPLC spectra for **55**

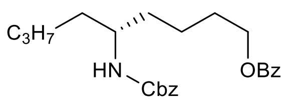

(*R*)-5-(((benzyloxy)carbonyl)amino)nonyl benzoate (**56**)

HPLC analysis: The ee was determined to be 91% on a CHIRALCEL OD-H column (10% *i*PrOH in hexane, 1.0 mL/min, 40 ^{o}C); retention times for compound obtained using (*S*)-**L2**: 9.9 min (major), 13.1min (minor).

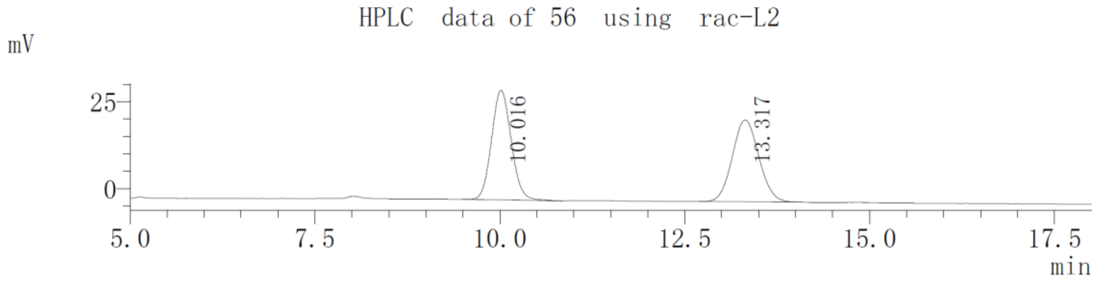

检测器A Ch1 214nm

Peak[#]	RetTime[min]	Height[uV]	Width[min]	Area[uV*s]	Area[%]
1	10.016	31490	0.285	583630	49.837
2	13.317	23488	0.388	587438	50.163

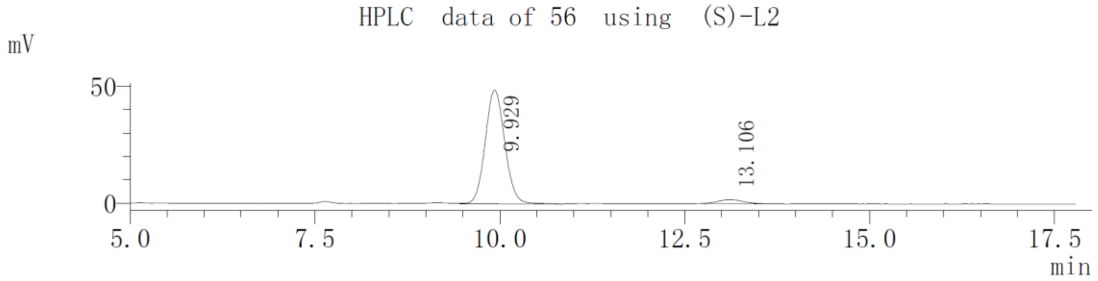

检测器A Ch1 214nm

Peak[#]	RetTime[min]	Height[uV]	Width[min]	Area[uV*s]	Area[%]
1	9.929	48701	0.283	895671	95.714
2	13.106	1726	0.373	40104	4.286

Supplementary Figure 424. HPLC spectra for **56**

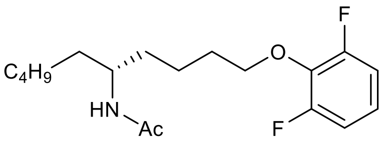

(*R*)-*N*-(1-(2,6-difluorophenoxy)decan-5-yl)acetamide (**57**)

<u>HPLC analysis</u>: The ee was determined to be 90% on a CHIRALPAK AD-H column (5% *i*PrOH in hexane, 0.5 mL/min, 40 °C); retention times for compound obtained using (*S*)-**L2**: 15.0 min (minor), 15.9min (major).

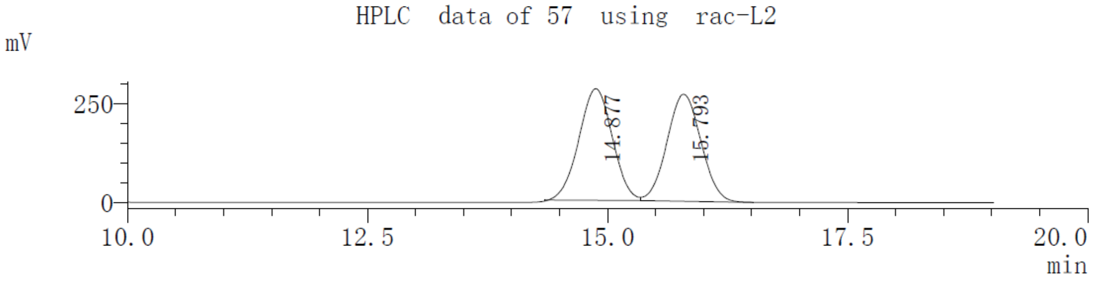

检测器A 214nm

Peak[#]	RetTime[min]	Height[uV]	Width[min]	Area[uV*s]	Area[%]
1	14. 877	282960	0. 380	6871261	50. 698
2	15. 793	271407	0. 385	6682067	49. 302

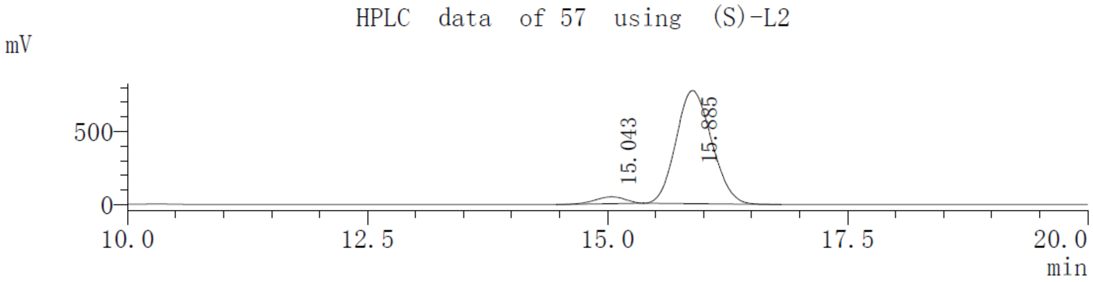

检测器A 214nm

Peak[#]	RetTime[min]	Height[uV]	Width[min]	Area[uV*s]	Area[%]
1	15. 043	47520	0. 352	1040890	4. 937
2	15. 885	775617	0. 406	20043718	95. 063

Supplementary Figure 425. HPLC spectra for **57**

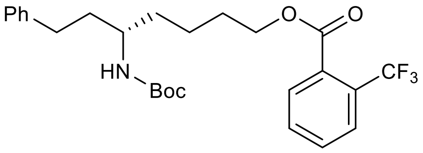

(*S*)-5-((*tert*-butoxycarbonyl)amino)-7-phenylheptyl 2-(trifluoromethyl)benzoate (**58**)

<u>HPLC analysis</u>: The ee was determined to be 93% on a CHIRALCEL OD-H column (10% *i*PrOH in hexane, 1.0 mL/min, 40 °C); retention times for compound obtained using (*S*)-**L2**: 7.3 min (major), 8.3min (minor).

HPLC data of 58 using rac-L2

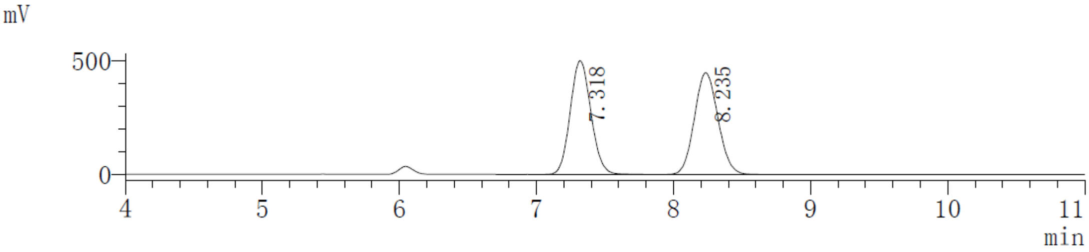

检测器A Ch1 214nm

Peak[#]	RetTime[min]	Height[uV]	Width[min]	Area[uV*s]	Area[%]
1	7.318	500319	0.163	5262916	49.501
2	8.235	447339	0.187	5368915	50.499

HPLC data of 58 using (S)-L2

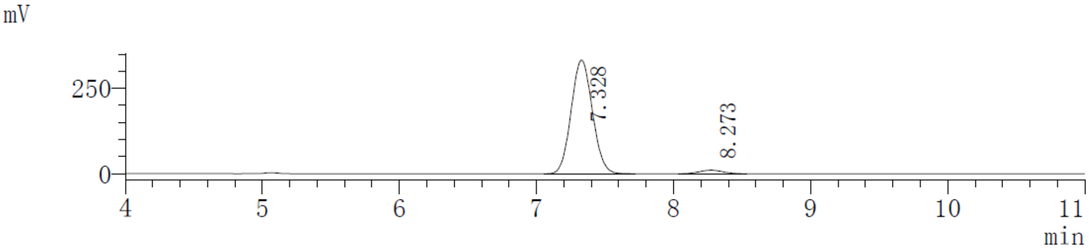

检测器A Ch1 214nm

Peak[#]	RetTime[min]	Height[uV]	Width[min]	Area[uV*s]	Area[%]
1	7.328	332590	0.168	3589955	96.528
2	8.273	10672	0.190	129142	3.472

Supplementary Figure 426. HPLC spectra for **58**

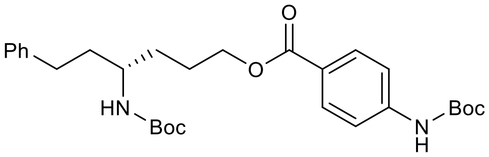

(*R*)-4-((*tert*-butoxycarbonyl)amino)-6-phenylhexyl-4-((*tert*-butoxycarbonyl)amino-benzoate (**59**)

<u>HPLC analysis</u>: The ee was determined to be 92% on a CHIRALPAK AD-H column (15% *i*PrOH in hexane, 1.5 mL/min, 40 ºC); retention times for compound obtained using (*S*)-**L2**: 5.8 min (major), 6.2min (minor).

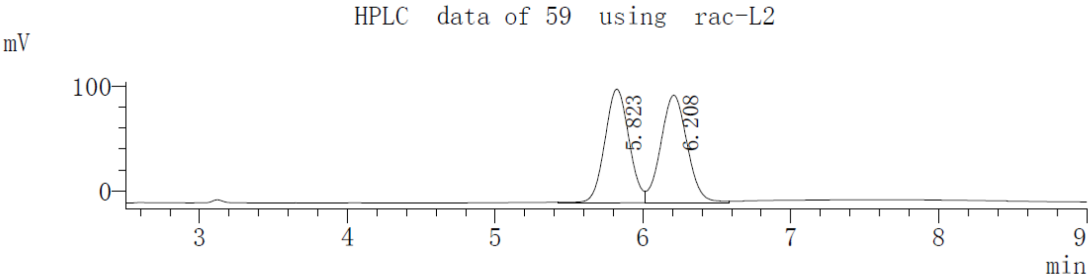

检测器A Ch1 214nm

Peak[#]	RetTime[min]	Height[uV]	Width[min]	Area[uV*s]	Area[%]
1	5.823	108453	0.177	1239400	49.304
2	6.208	102655	0.191	1274377	50.696

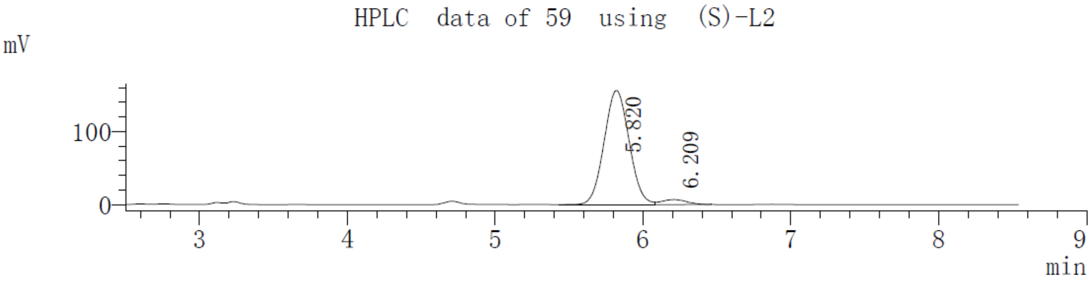

检测器A Ch1 214nm

Peak[#]	RetTime[min]	Height[uV]	Width[min]	Area[uV*s]	Area[%]
1	5.820	156259	0.184	1845082	95.852
2	6.209	6753	0.213	79850	4.148

Supplementary Figure 427. HPLC spectra for **59**

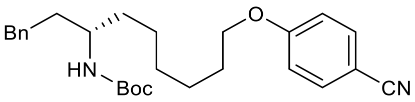

tert-butyl (*S*)-(9-(4-cyanophenoxy)-1-phenylnonan-3-yl)carbamate (**60**)

HPLC analysis: The ee was determined to be 93% on a CHIRALPAK AD-H column (10% *i*PrOH in hexane, 1.0 mL/min, 40 °C); retention times for compound obtained using (*S*)-**L2**: 15.3 min (minor), 15.9min (major).

HPLC data of 60 using rac-L2

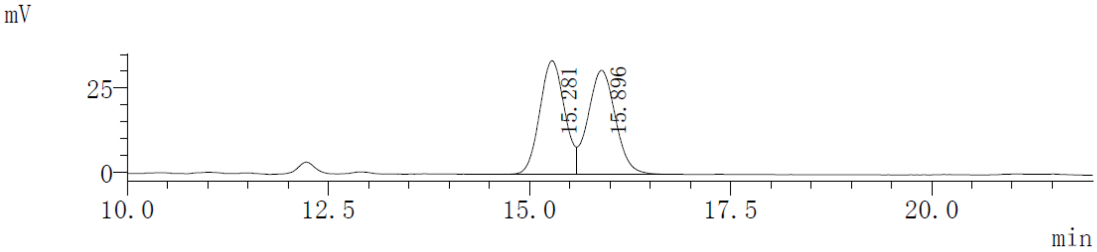

检测器A Ch1 214nm

Peak[#]	RetTime[min]	Height[uV]	Width[min]	Area[uV*s]	Area[%]
1	15.281	33683	0.340	729886	50.017
2	15.896	30822	0.370	729389	49.983

HPLC data of 60 using (S)-L2

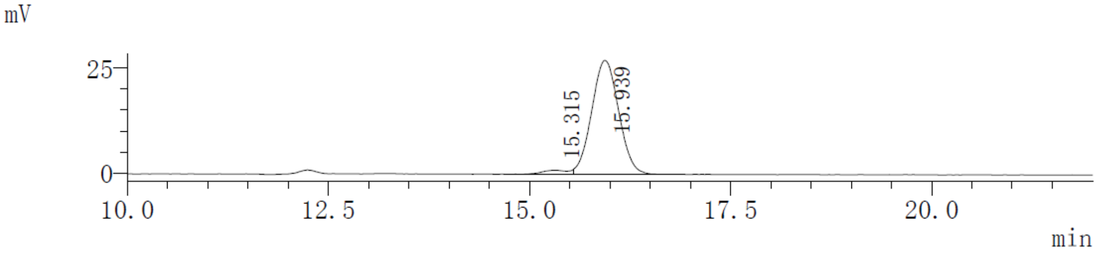

检测器A Ch1 214nm

Peak[#]	RetTime[min]	Height[uV]	Width[min]	Area[uV*s]	Area[%]
1	15.315	972	--	22952	3.501
2	15.939	27098	0.365	632616	96.499

Supplementary Figure 428. HPLC spectra for **60**

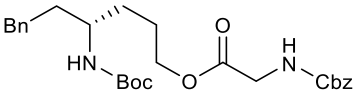

(*R*)-4-((*tert*-butoxycarbonyl)amino)-6-phenylhexyl ((benzyloxy)carbonyl)glycinate (**61**)

<u>HPLC analysis</u>: The ee was determined to be 92% on a CHIRALCEL OD-H column (20% *i*PrOH in hexane, 2.0 mL/min, 40 °C); retention times for compound obtained using (*S*)-**L2**: 7.0 min (major), 8.2min (minor).

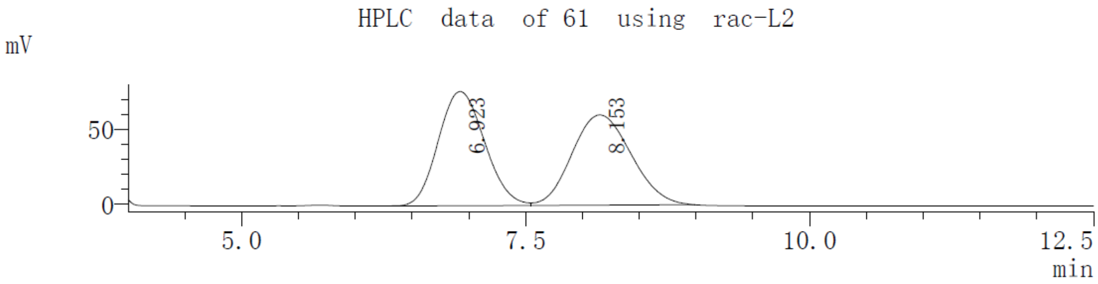

检测器A Ch1 214nm

Peak[#]	RetTime[min]	Height[uV]	Width[min]	Area[uV*s]	Area[%]
1	6.923	76044	0.447	2157556	49.760
2	8.153	60126	0.574	2178355	50.240

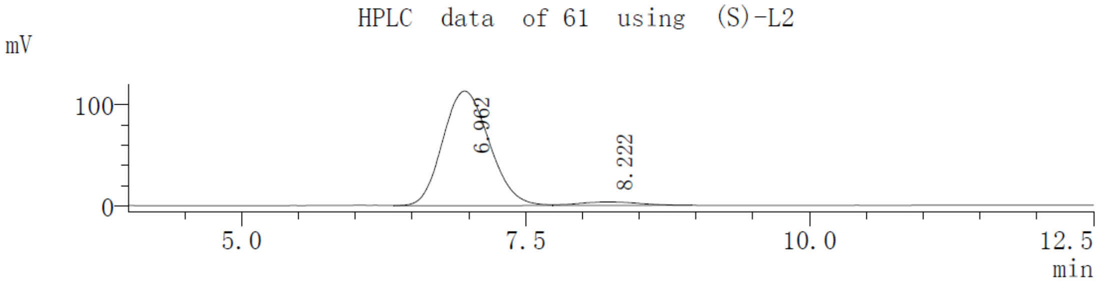

检测器A Ch1 214nm

Peak[#]	RetTime[min]	Height[uV]	Width[min]	Area[uV*s]	Area[%]
1	6.962	112998	0.448	3228166	96.186
2	8.222	3455	0.590	128003	3.814

Supplementary Figure 429. HPLC spectra for **61**

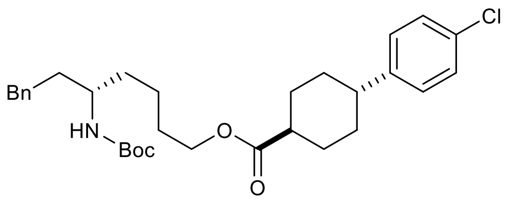

(*S*)-5-((*tert*-butoxycarbonyl)amino)-7-phenylheptyl (1*r*,4*S*)-4-(4 chlorophenyl) cyclohexane-1-carboxylate (**62**)

<u>HPLC analysis</u>: The ee was determined to be 93% on a CHIRALPAK AD-H column (5% *i*PrOH in hexane, 0.5 mL/min, 40 °C); retention times for compound obtained using (*S*)-**L2**: 34.8 min (minor), 36.4min (major).

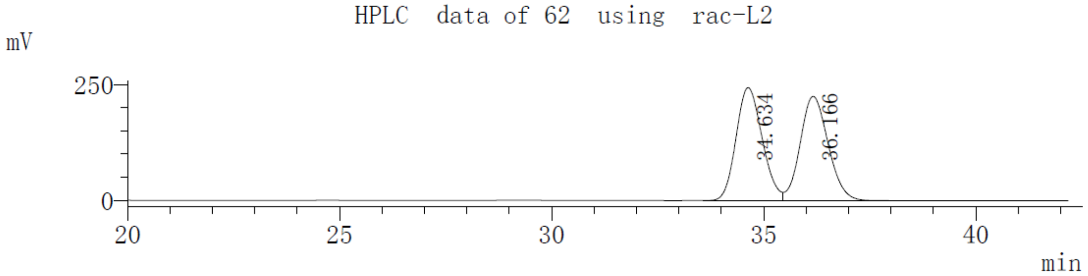

检测器A Ch1 214nm

Peak[#]	RetTime[min]	Height[uV]	Width[min]	Area[uV*s]	Area[%]
1	34.634	244784	0.669	10503252	50.274
2	36.166	225960	0.710	10388731	49.726

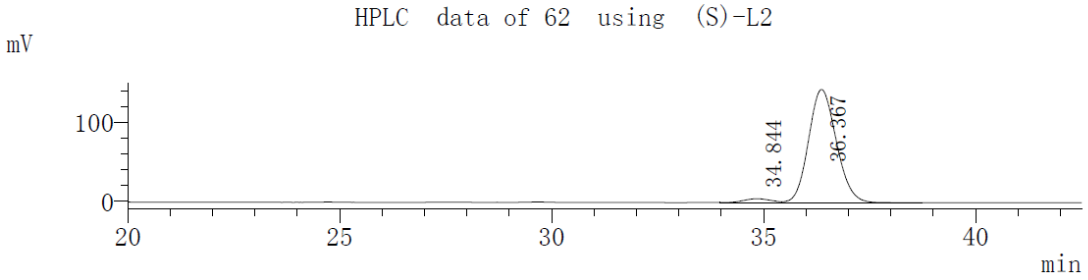

检测器A Ch1 214nm

Peak[#]	RetTime[min]	Height[uV]	Width[min]	Area[uV*s]	Area[%]
1	34.844	5145	0.694	225941	3.347
2	36.367	142901	0.706	6524738	96.653

Supplementary Figure 430. HPLC spectra for **62**

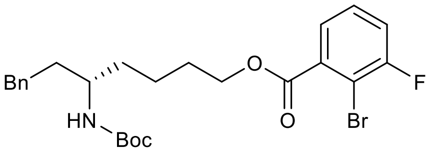

(*S*)-5-((*tert*-butoxycarbonyl)amino)-7-phenylheptyl 2-bromo-3-fluorobenzoate (**63**)

HPLC analysis: The ee was determined to be 91% on a CHIRALPAK AD-H column (10% *i*PrOH in hexane, 1.0 mL/min, 40 °C); retention times for compound obtained using (*S*)-**L2**: 8.6 min (major), 10.4min (minor).

HPLC data of 63 using rac-L2

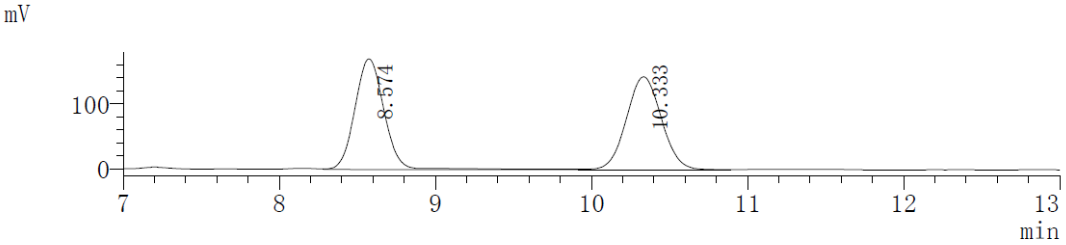

检测器A Ch1 214nm

Peak[#]	RetTime[min]	Height[uV]	Width[min]	Area[uV*s]	Area[%]
1	8.574	169968	0.199	2242069	50.265
2	10.333	142567	0.242	2218384	49.735

HPLC data of 63 using (S)-L2

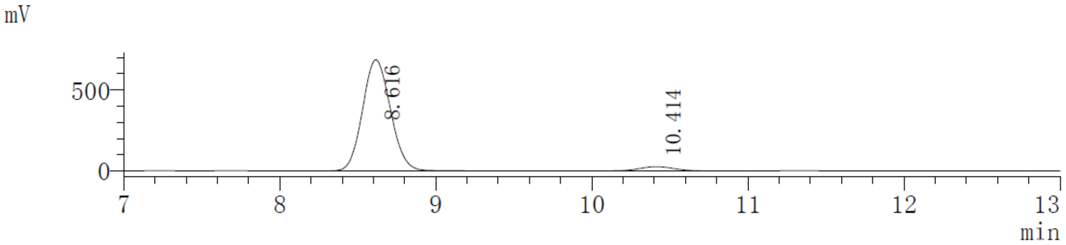

检测器A Ch1 214nm

Peak[#]	RetTime[min]	Height[uV]	Width[min]	Area[uV*s]	Area[%]
1	8.616	687189	0.194	8551411	95.495
2	10.414	26724	0.237	403455	4.505

Supplementary Figure 431. HPLC spectra for **63**

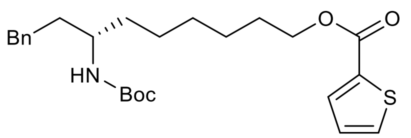

(*S*)-7-((*tert*-butoxycarbonyl)amino)-9-phenylnonyl thiophene-2-carboxylate (**64**)

HPLC analysis: The ee was determined to be 91% on a CHIRALPAK AD-H column (10% *i*PrOH in hexane, 1.0 mL/min, 40 °C); retention times for compound obtained using (*S*)-**L2**: 8.4 min (minor), 9.0 min (major).

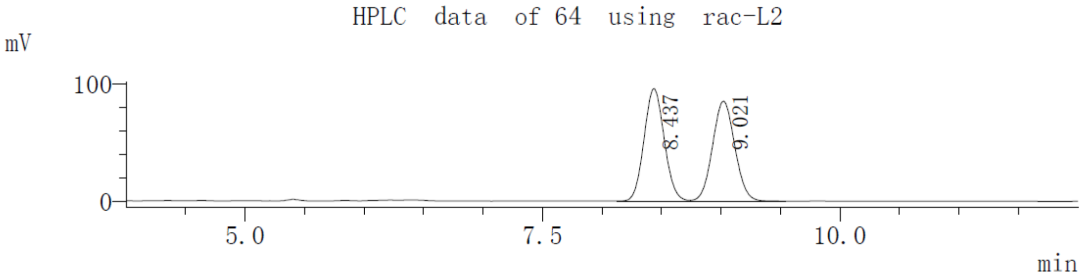

检测器A Ch1 214nm

Peak[#]	RetTime[min]	Height[uV]	Width[min]	Area[uV*s]	Area[%]
1	8.437	95992	0.184	1137521	50.529
2	9.021	85273	0.203	1113683	49.471

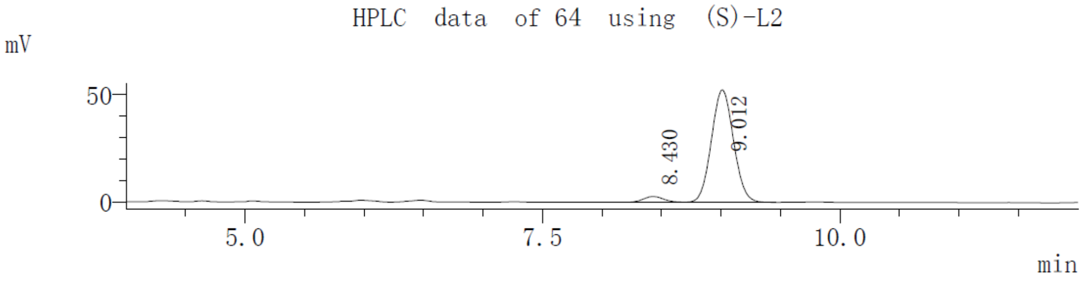

检测器A Ch1 214nm

Peak[#]	RetTime[min]	Height[uV]	Width[min]	Area[uV*s]	Area[%]
1	8.430	2718	0.184	32510	4.572
2	9.012	52380	0.201	678509	95.428

Supplementary Figure 432. HPLC spectra for **64**

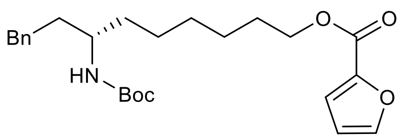

(*S*)-7-((*tert*-butoxycarbonyl)amino)-9-phenylnonyl furan-2-carboxylate (**65**)

HPLC analysis: The ee was determined to be 93% on a CHIRALPAK AD-H column (10% *i*PrOH in hexane, 1.0 mL/min, 40 °C); retention times for compound obtained using (*S*)-**L2**: 8.8 min (minor), 9.6 min (major).

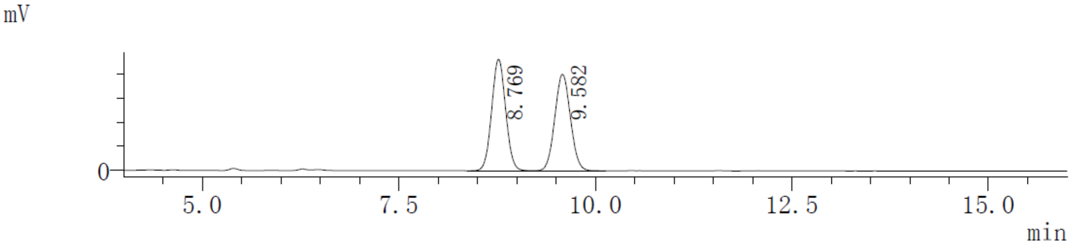

检测器A Ch1 214nm

Peak[#]	RetTime[min]	Height[uV]	Width[min]	Area[uV*s]	Area[%]
1	8.769	92767	0.191	1150774	50.815
2	9.582	80368	0.215	1113852	49.185

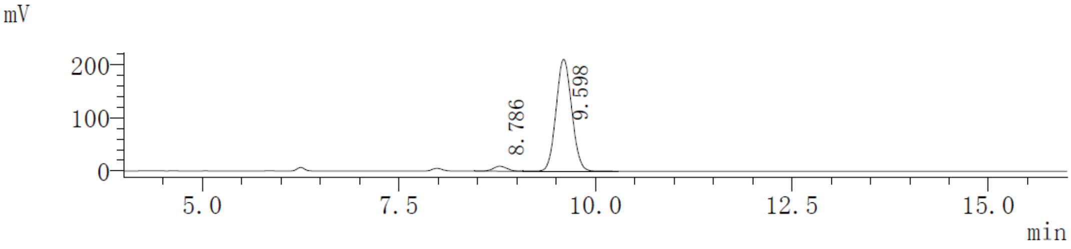

检测器A Ch1 214nm

Peak[#]	RetTime[min]	Height[uV]	Width[min]	Area[uV*s]	Area[%]
1	8.786	9162	0.191	112936	3.704
2	9.598	210965	0.216	2936368	96.296

Supplementary Figure 433. HPLC spectra for **65**

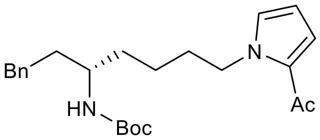

tert-butyl (*S*)-(7-(2-acetyl-1H-pyrrol-1-yl)-1-phenylheptan-3-yl)carbamate (**66**)

HPLC analysis: The ee was determined to be 95% on a CHIRALPAK AD-H column (15% *i*PrOH in hexane, 1.5 mL/min, 40 ºC); retention times for compound obtained using (*S*)-**L2**: 4.0 min (major), 4.3 min (minor).

HPLC data of 66 using rac-L2

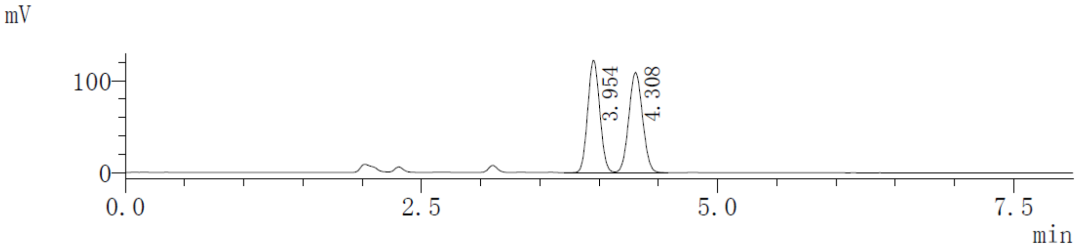

检测器A Ch1 214nm

Peak[#]	RetTime[min]	Height[uV]	Width[min]	Area[uV*s]	Area[%]
1	3.954	122684	0.110	866888	49.458
2	4.308	109683	0.126	885895	50.542

HPLC data of 66 using (S)-L2

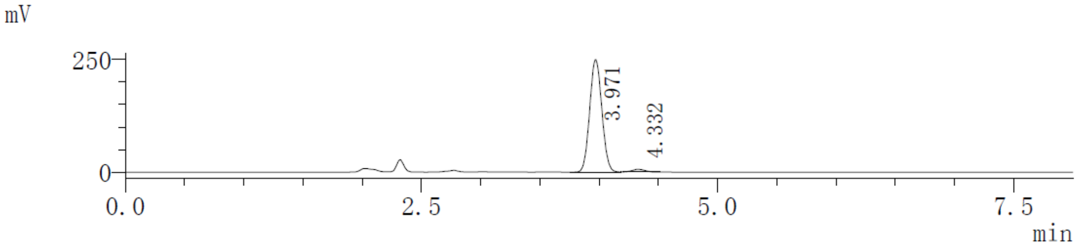

检测器A Ch1 214nm

Peak[#]	RetTime[min]	Height[uV]	Width[min]	Area[uV*s]	Area[%]
1	3.971	249819	0.112	1790861	97.504
2	4.332	5989	0.123	45841	2.496

Supplementary Figure 434. HPLC spectra for **66**

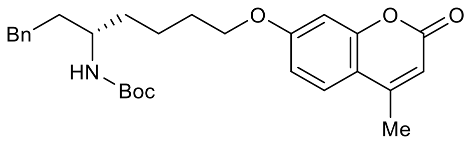

tert-butyl (*S*)-(7-((4-methyl-2-oxo-2H-chromen-7-yl)oxy)-1-phenylheptan-3-yl) carbamate (**67**)

HPLC analysis: The ee was determined to be 92% on a CHIRALPAK AD-H column (15% *i*PrOH in hexane, 1.5 mL/min, 40 °C); retention times for compound obtained using (*S*)-**L2**: 10.0 min (major), 13.8 min (minor).

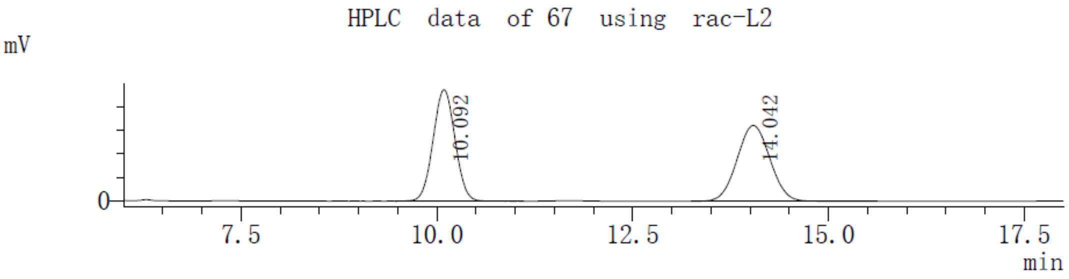

检测器A Ch1 214nm

Peak[#]	RetTime[min]	Height[uV]	Width[min]	Area[uV*s]	Area[%]
1	10.092	94683	0.304	1844957	49.565
2	14.042	64333	0.457	1877331	50.435

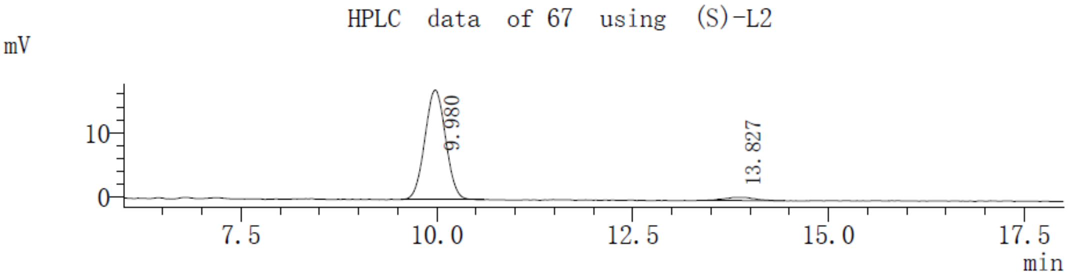

检测器A Ch1 214nm

Peak[#]	RetTime[min]	Height[uV]	Width[min]	Area[uV*s]	Area[%]
1	9.980	17011	0.290	315790	96.127
2	13.827	478	0.413	12724	3.873

Supplementary Figure 435. HPLC spectra for **67**

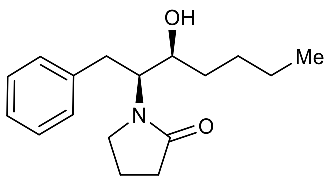

1-((2*S*,3*S*)-3-hydroxy-1-phenylheptan-2-yl)pyrrolidin-2-one (**83-OH**)

HPLC analysis: The ee was determined to be 97% on a CHIRALPAK AD-H column (8% *i*PrOH in hexane, 0.8 mL/min, 40 °C); retention times for compound obtained using (*S*,*S*)-**L1**: 11.4 min (minor), 13.3 min (major).

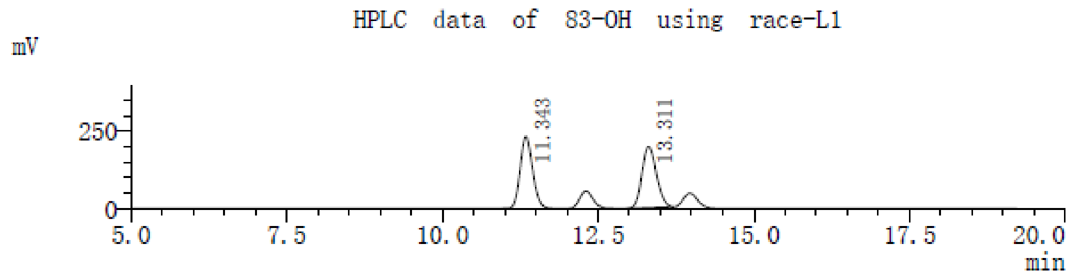

检测器A Ch1 214nm

Peak[#]	RetTime[min]	Height[uV]	Width[min]	Area[uV*s]	Area[%]
1	12.304	56653	0.230	843661	50.536
2	13.972	48966	0.262	825780	49.464

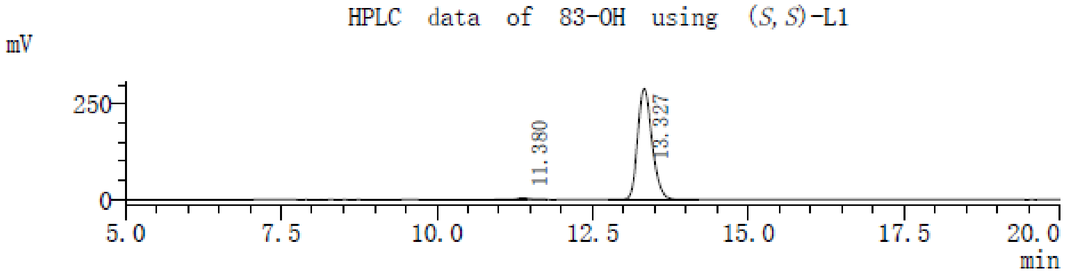

检测器A Ch1 214nm

Peak[#]	RetTime[min]	Height[uV]	Width[min]	Area[uV*s]	Area[%]
1	11.380	4532	0.201	62712	1.325
2	13.327	290017	0.247	4670238	98.675

Supplementary Figure 436. HPLC spectra for **83-OH**

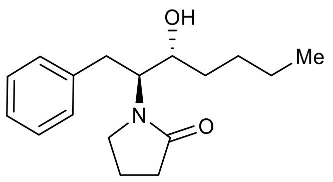

1-((2*S*,3*R*)-3-hydroxy-1-phenylheptan-2-yl)pyrrolidin-2-one (**84-OH**)

<u>HPLC analysis:</u> The ee was determined to be 97% on a CHIRALPAK AD-H column (8% *i*PrOH in hexane, 0.8 mL/min, 40 °C); retention times for compound obtained using (*S*,*S*)-**L1**: 13.9 min (minor), 12.3 min (major).

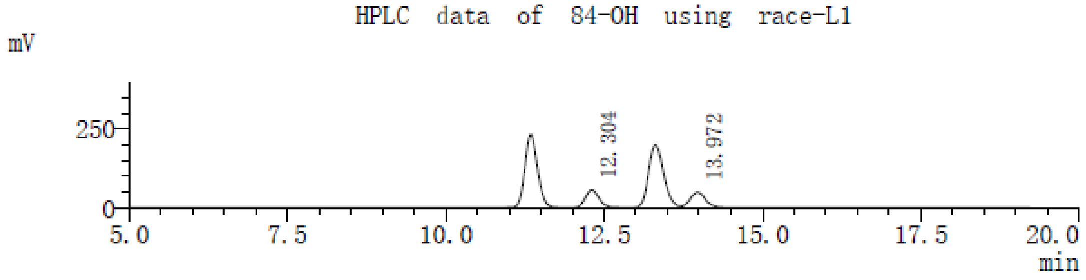

检测器A Ch1 214nm

Peak[#]	RetTime[min]	Height[uV]	Width[min]	Area[uV*s]	Area[%]
1	12.304	56653	0.230	843661	50.536
2	13.972	48966	0.262	825780	49.464

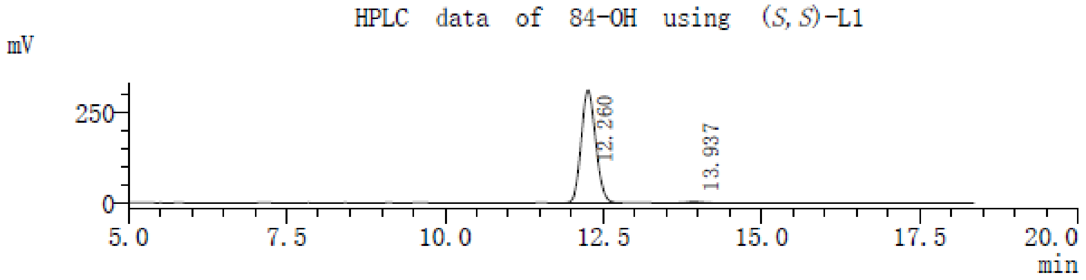

检测器A Ch1 214nm

Peak[#]	RetTime[min]	Height[uV]	Width[min]	Area[uV*s]	Area[%]
1	12.260	310845	0.248	4976423	98.653
2	13.937	3828	0.270	67931	1.347

Supplementary Figure 437. HPLC spectra for **84-OH**

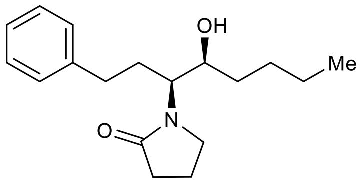

1-((3*S*,4*S*)-4-hydroxy-1-phenyloctan-3-yl)pyrrolidin-2-one (**85**)

<u>HPLC analysis:</u> The ee was determined to be 94% on a CHIRALPAK AD-H column (10% *i*PrOH in hexane, 1.0 mL/min, 40 °C); retention times for compound obtained using (*S*,*S*)-**L1**: 7.1 min (minor), 11.1 min (major).

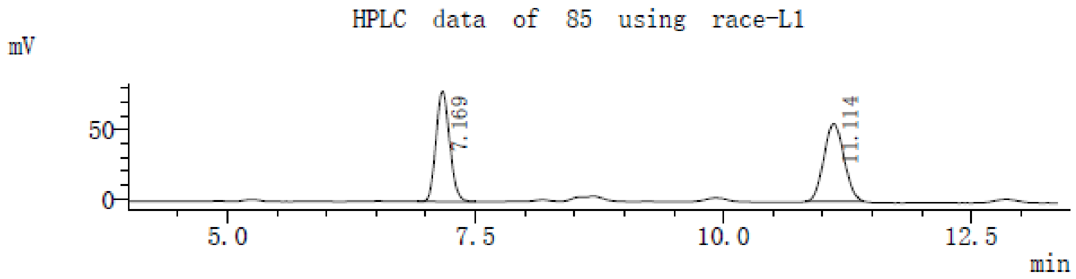

检测器A Ch1 214nm

Peak[#]	RetTime[min]	Height[uV]	Width[min]	Area[uV*s]	Area[%]
1	7.169	79391	0.149	764034	49.051
2	11.114	55971	0.223	793599	50.949

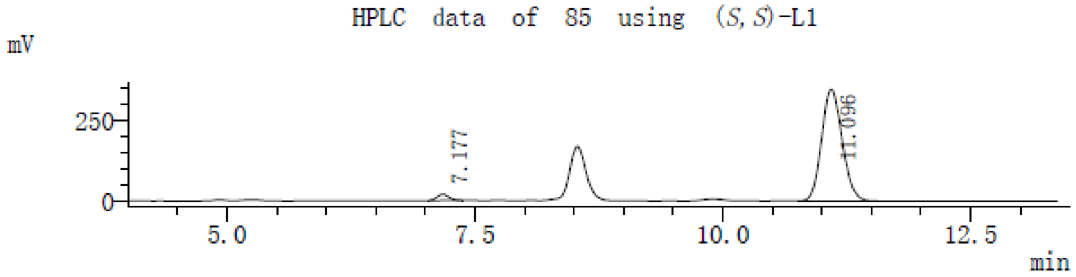

检测器A Ch1 214nm

Peak[#]	RetTime[min]	Height[uV]	Width[min]	Area[uV*s]	Area[%]
1	7.177	19142	0.133	160613	3.066
2	11.096	349535	0.224	5078430	96.934

Supplementary Figure 438. HPLC spectra for **85**

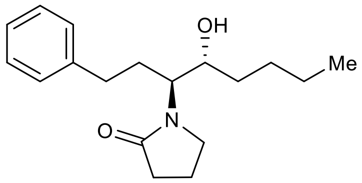

1-((3*S*,4*R*)-4-hydroxy-1-phenyloctan-3-yl)pyrrolidin-2-one (**86**)

HPLC analysis: The ee was determined to be 94% on a CHIRALCEL OD-H column (10% *i*PrOH in hexane, 1.0 mL/min, 40 °C); retention times for compound obtained using (*S*,*S*)-**L1**: 6.8 min (minor), 7.9 min (major).

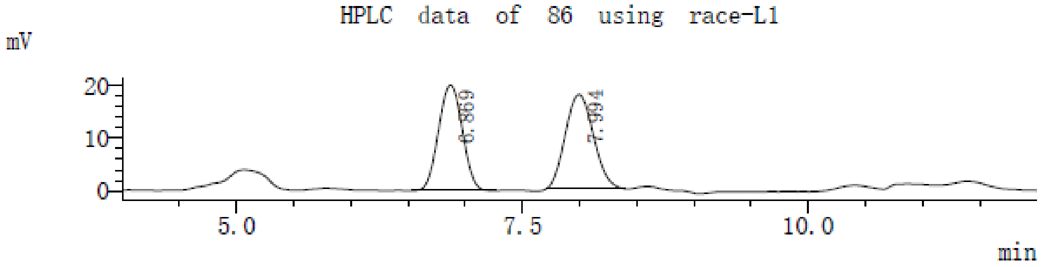

检测器A Ch1 214nm

Peak[#]	RetTime[min]	Height[uV]	Width[min]	Area[uV*s]	Area[%]
1	6.869	20053	0.233	292801	48.683
2	7.994	17928	0.274	308641	51.317

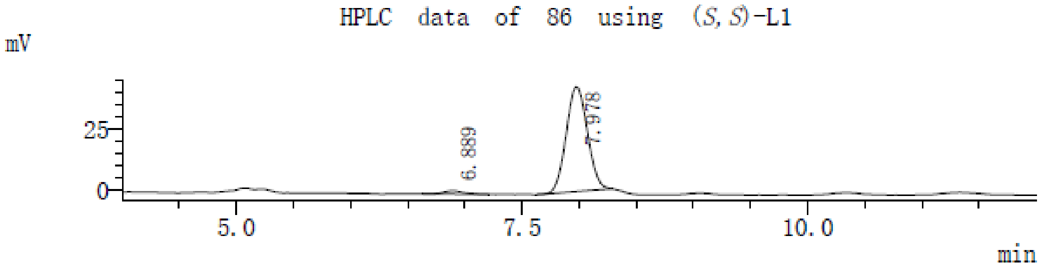

检测器A Ch1 214nm

Peak[#]	RetTime[min]	Height[uV]	Width[min]	Area[uV*s]	Area[%]
1	6.889	1322	0.181	15827	2.742
2	7.978	43231	0.204	561362	97.258

Supplementary Figure 439. HPLC spectra for **86**

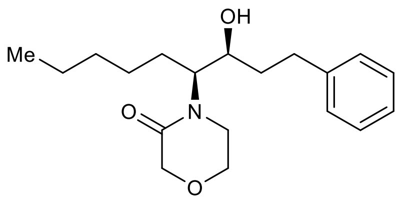

4-((3*S*,4*S*)-3-hydroxy-1-phenylnonan-4-yl)morpholin-3-one (**87**)

HPLC analysis: The ee was determined to be 92% on a CHIRALPAK AD-H column (10% *i*PrOH in hexane, 1.0 mL/min, 40 °C); retention times for compound obtained using (*S*,*S*)-**L1**: 7.1 min (minor), 9.6 min (major).

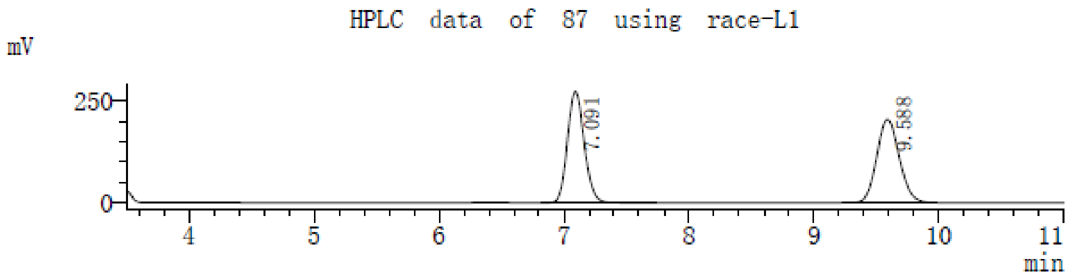

检测器A Ch1 214nm

Peak[#]	RetTime[min]	Height[uV]	Width[min]	Area[uV*s]	Area[%]
1	7.091	274700	0.144	2560275	49.801
2	9.588	205522	0.195	2580719	50.199

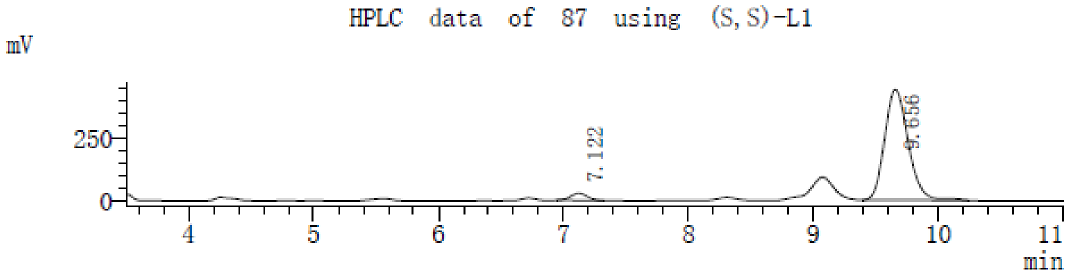

检测器A Ch1 214nm

Peak[#]	RetTime[min]	Height[uV]	Width[min]	Area[uV*s]	Area[%]
1	7.122	27803	0.141	248363	4.104
2	9.656	441722	0.200	5802924	95.896

Supplementary Figure 440. HPLC spectra for **87**

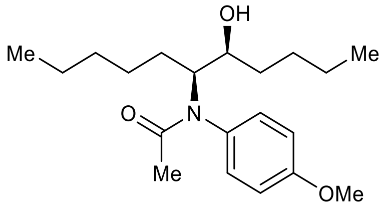

N-((5*S*,6*S*)-5-hydroxyundecan-6-yl)-*N*-(4-methoxyphenyl)acetamide (**88-OH**)

HPLC analysis: The ee was determined to be 91% on a CHIRALPAK AS-H column (10% *i*PrOH in hexane, 1.0 mL/min, 40 °C); retention times for compound obtained using (*S*,*S*)-**L1**: 5.9 min (minor), 6.8 min (major).

HPLC data of 88-OH using race-L1

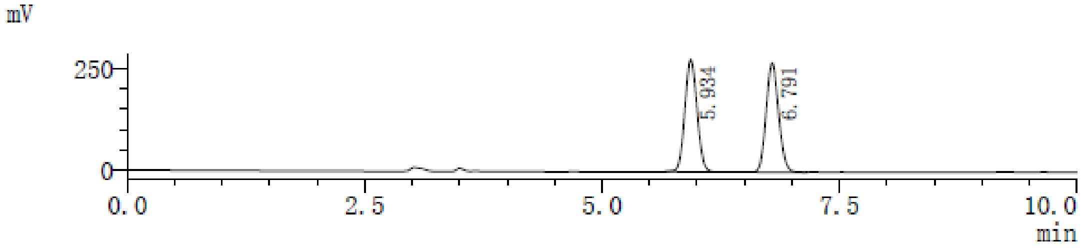

检测器A Ch1 214nm

Peak[#]	RetTime[min]	Height[uV]	Width[min]	Area[uV*s]	Area[%]
1	5.934	276512	0.139	2465769	49.949
2	6.791	269297	0.143	2470799	50.051

HPLC data of 88-OH using (*S*, *S*)-L1

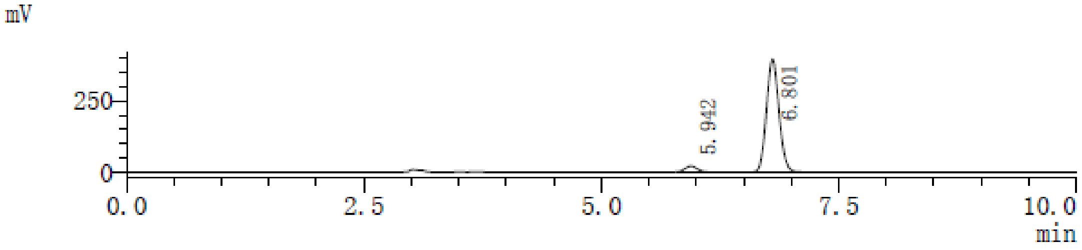

检测器A Ch1 214nm

Peak[#]	RetTime[min]	Height[uV]	Width[min]	Area[uV*s]	Area[%]
1	5.942	20510	0.135	173128	4.525
2	6.801	395843	0.143	3653027	95.475

Supplementary Figure 441. HPLC spectra for **88-OH**

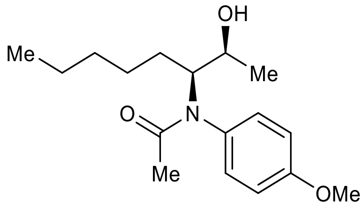

N-((2*S*,3*S*)-2-hydroxyoctan-3-yl)-*N*-(4-methoxyphenyl)acetamide (**89-OH**)

<u>HPLC analysis:</u> The ee was determined to be 93% on a CHIRALPAK AD-H column (10% *i*PrOH in hexane, 1.0 mL/min, 40 °C); retention times for compound obtained using (*S*,*S*)-**L1**: 5.7 min (minor), 6.9 min (major).

HPLC data of 89-OH using race-L1

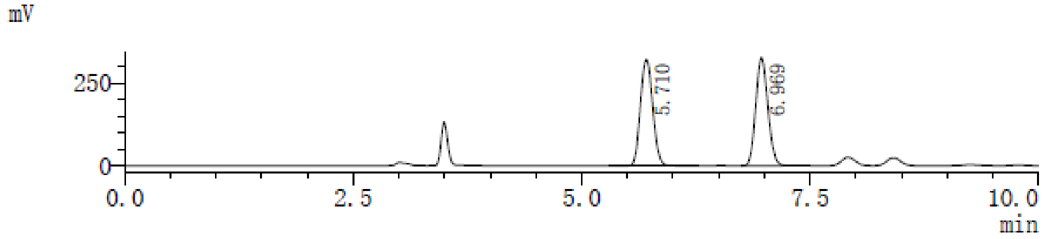

检测器A Ch1 214nm

Peak[#]	RetTime[min]	Height[uV]	Width[min]	Area[uV*s]	Area[%]
1	5.710	320388	0.149	3031437	50.190
2	6.969	326026	0.143	3008473	49.810

HPLC data of 89-OH using (S,S)-L1 in 25 °C

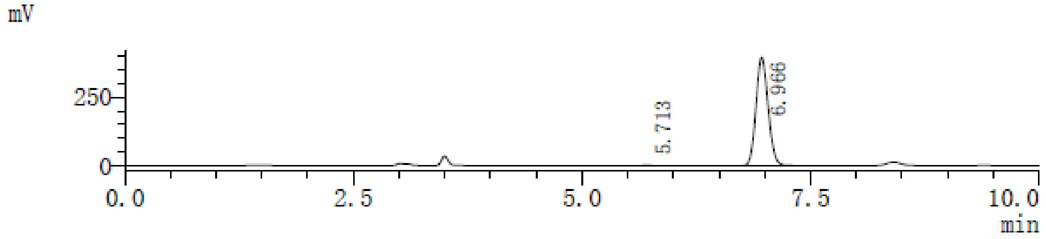

检测器A Ch1 214nm

Peak[#]	RetTime[min]	Height[uV]	Width[min]	Area[uV*s]	Area[%]
1	5.713	600	0.150	6052	0.164
2	6.966	396709	0.144	3673457	99.836

HPLC data of 89-OH using (S,S)-L1 in 40 °C

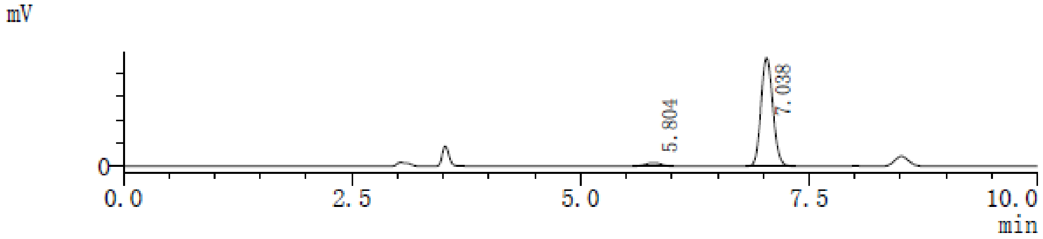

检测器A Ch1 214nm

Peak[#]	RetTime[min]	Height[uV]	Width[min]	Area[uV*s]	Area[%]
1	5.804	6929	0.185	78782	3.582
2	7.038	233914	0.141	2120630	96.418

Supplementary Figure 442. HPLC spectra for **89-OH**

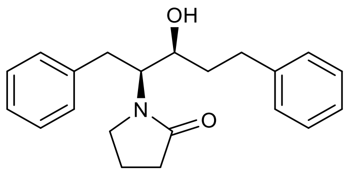

1-((2*S*,3*S*)-3-hydroxy-1,5-diphenylpentan-2-yl)pyrrolidin-2-one (**90**)

HPLC analysis: The ee was determined to be 97% on a CHIRALPAK AD-H column (10% *i*PrOH in hexane, 1.0 mL/min, 40 °C); retention times for compound obtained using (*S*,*S*)-**L1**: 10.9 min (minor), 13.1 min (major).

HPLC data of 90 using race-L1

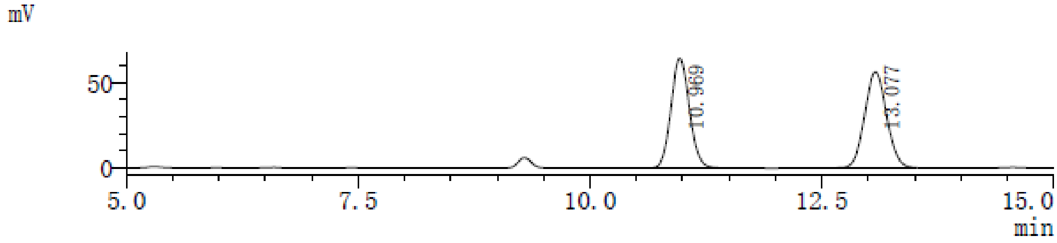

检测器A Ch1 214nm

Peak[#]	RetTime[min]	Height[uV]	Width[min]	Area[uV*s]	Area[%]
1	10.969	64454	0.214	891903	49.089
2	13.077	56374	0.255	925008	50.911

HPLC data of 90 using (*S*, *S*)-L1

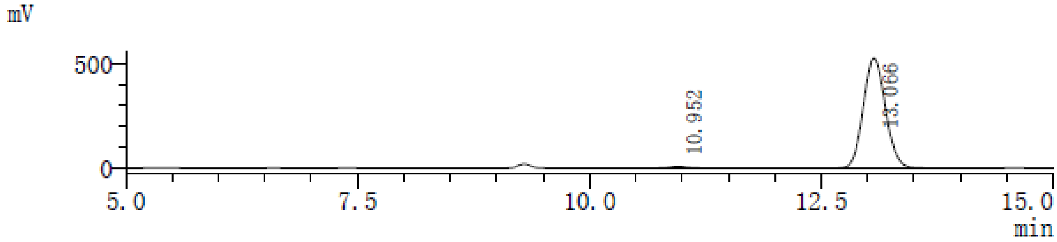

检测器A Ch1 214nm

Peak[#]	RetTime[min]	Height[uV]	Width[min]	Area[uV*s]	Area[%]
1	10.952	8417	0.217	116240	1.305
2	13.066	526454	0.259	8788953	98.695

Supplementary Figure 443. HPLC spectra for **90**

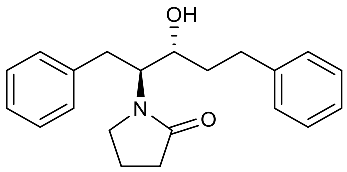

1-((2*S*,3*R*)-3-hydroxy-1,5-diphenylpentan-2-yl)pyrrolidin-2-one (**91**)

<u>HPLC analysis:</u> The ee was determined to be 98% on a CHIRALPAK AD-H column (10% *i*PrOH in hexane, 1.0 mL/min, 40 °C); retention times for compound obtained using (*S*,*S*)-**L1**: 12.3 min (minor), 10.9 min (major).

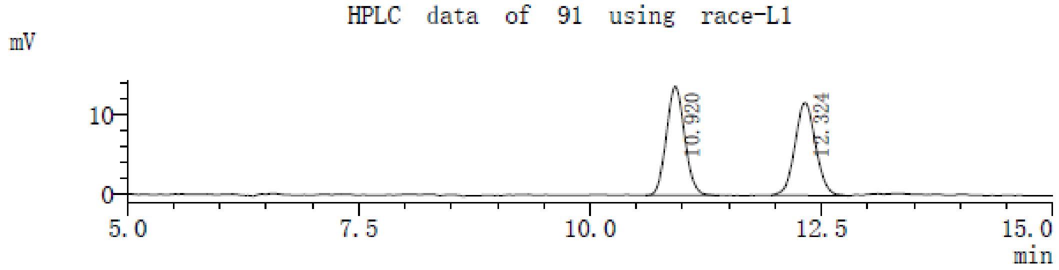

检测器A Ch1 214nm

Peak[#]	RetTime[min]	Height[uV]	Width[min]	Area[uV*s]	Area[%]
1	10.920	13830	0.214	190485	50.742
2	12.324	11795	0.241	184911	49.258

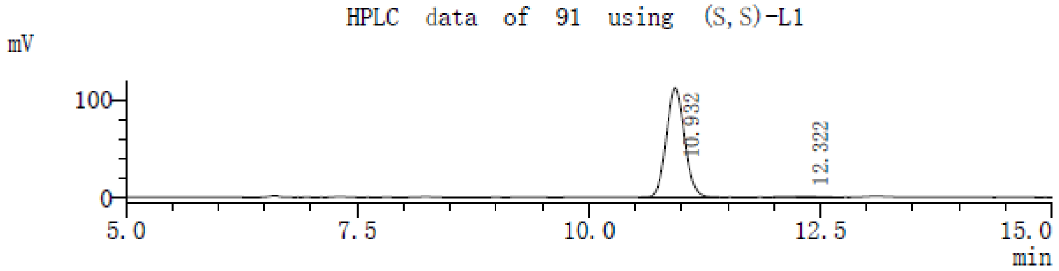

检测器A Ch1 214nm

Peak[#]	RetTime[min]	Height[uV]	Width[min]	Area[uV*s]	Area[%]
1	10.932	112301	0.213	1547970	99.035
2	12.322	924	0.249	15081	0.965

Supplementary Figure 444. HPLC spectra for **91**

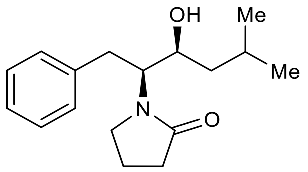

1-((2*S*,3*S*)-3-hydroxy-5-methyl-1-phenylhexan-2-yl)pyrrolidin-2-one (**95**)

HPLC analysis: The ee was determined to be 98% on a CHIRALPAK AD-H column (10% *i*PrOH in hexane, 1.0 mL/min, 40 °C); retention times for compound obtained using (*S*,*S*)-**L1**: 6.9 min (minor), 7.3 min (major).

HPLC data of 92-OH using race-L1

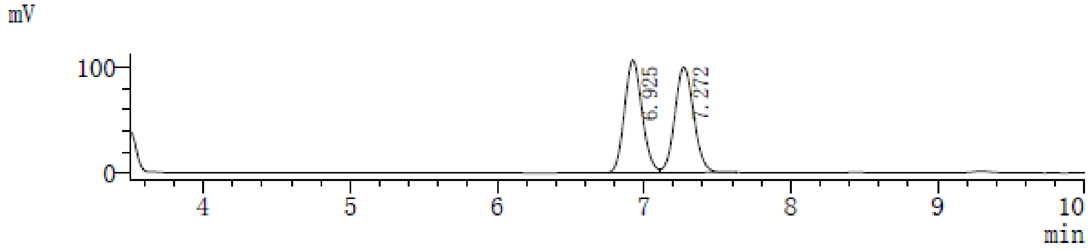

检测器A Ch1 214nm

Peak[#]	RetTime[min]	Height[uV]	Width[min]	Area[uV*s]	Area[%]
1	6.925	107032	0.129	890720	49.862
2	7.272	100153	0.138	895659	50.138

HPLC data of 92-OH using (S,S)-L1

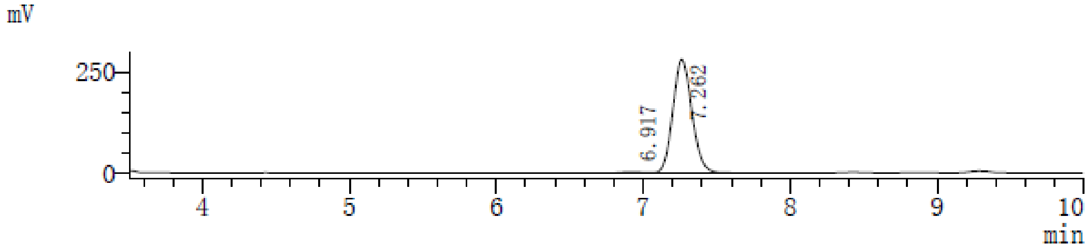

检测器A Ch1 214nm

Peak[#]	RetTime[min]	Height[uV]	Width[min]	Area[uV*s]	Area[%]
1	6.917	2866	0.129	23318	0.907
2	7.262	281330	0.139	2546609	99.093

Supplementary Figure 445. HPLC spectra for **95**

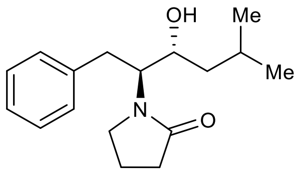

1-((2*S*,3*R*)-3-hydroxy-5-methyl-1-phenylhexan-2-yl)pyrrolidin-2-one **(93-OH)**

HPLC analysis: The ee was determined to be 98% on a CHIRALPAK AD-H column (10% *i*PrOH in hexane, 1.0 mL/min, 40 °C); retention times for compound obtained using (*S*,*S*)-**L1**: 8.6 min (minor), 7.8 min (major).

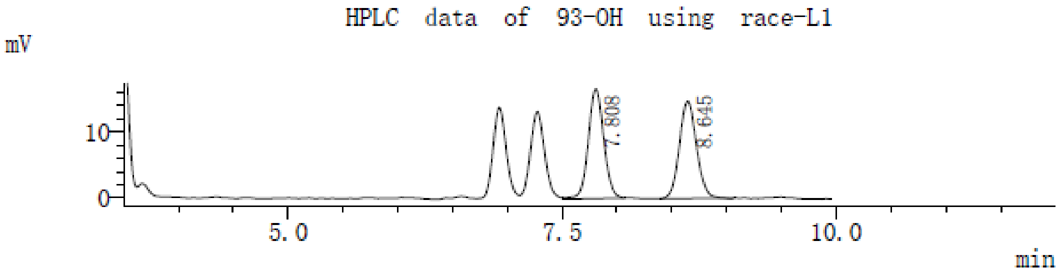

检测器A Ch1 214nm

Peak[#]	RetTime[min]	Height[uV]	Width[min]	Area[uV*s]	Area[%]
1	7.808	16568	0.150	163561	50.561
2	8.645	14703	0.167	159933	49.439

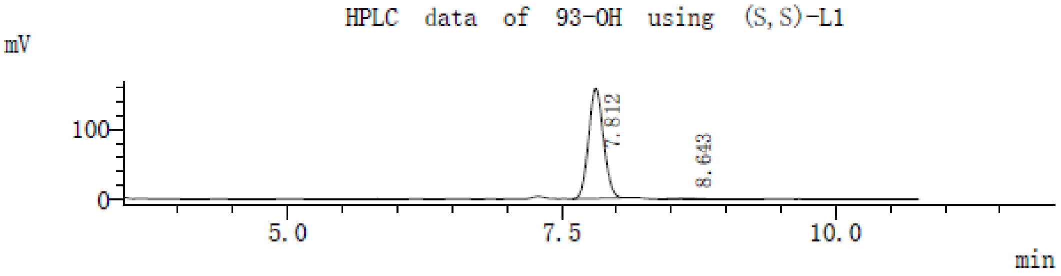

检测器A Ch1 214nm

Peak[#]	RetTime[min]	Height[uV]	Width[min]	Area[uV*s]	Area[%]
1	7.812	156628	0.148	1491440	99.091
2	8.643	1399	0.153	13686	0.909

Supplementary Figure 446. HPLC spectra for **93-OH**

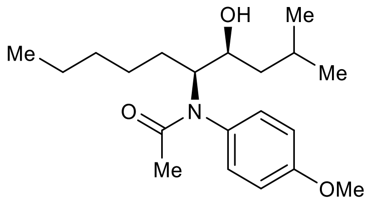

N-((4*S*,5*S*)-4-hydroxy-2-methyldecan-5-yl)-*N*-(4-methoxyphenyl)acetamide (**94-OH**)

<u>HPLC analysis:</u> The ee was determined to be 92% on a CHIRALPAK AD-H column (8% *i*PrOH in hexane, 0.8 mL/min, 40 ^{o}C); retention times for compound obtained using (*S*,*S*)-**L1**: 5.4 min (minor), 5.8 min (major).

HPLC data of 94-OH using race-L1

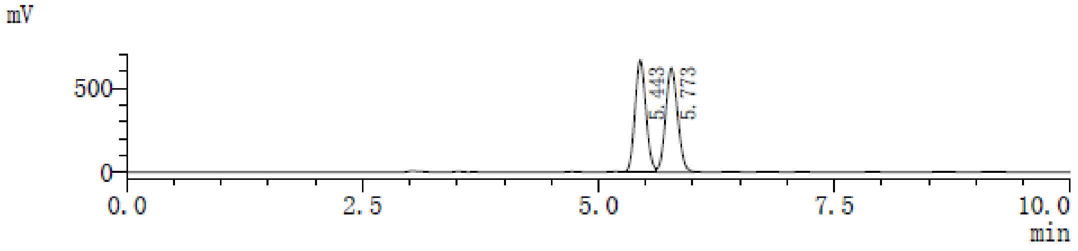

检测器A Ch1 214nm

Peak[#]	RetTime[min]	Height[uV]	Width[min]	Area[uV*s]	Area[%]
1	5.443	664746	0.126	5379834	49.510
2	5.773	615727	0.139	5486221	50.490

HPLC data of 94-OH using (*S*, *S*)-L1

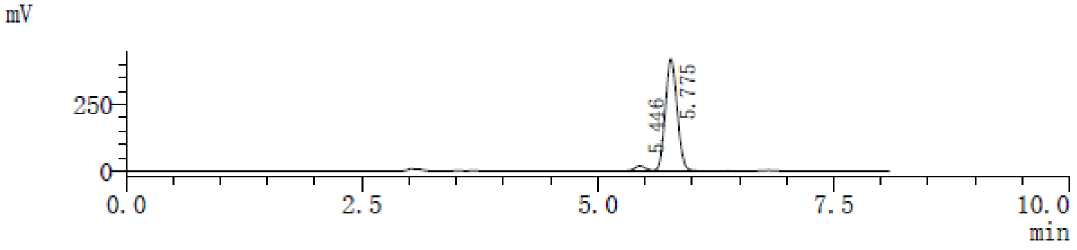

检测器A Ch1 214nm

Peak[#]	RetTime[min]	Height[uV]	Width[min]	Area[uV*s]	Area[%]
1	5.446	19566	0.125	154565	4.027
2	5.775	421885	0.137	3683949	95.973

Supplementary Figure 447. HPLC spectra for **94-OH**

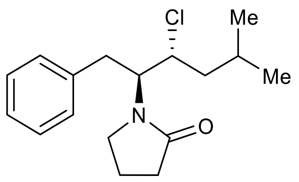

1-((2*S*,3*R*)-3-chloro-5-methyl-1-phenylhexan-2-yl)pyrrolidin-2-one (**96**)

HPLC analysis: The ee was determined to be 97% on a CHIRALPAK AD-H column (10% *i*PrOH in hexane, 1.0 mL/min, 40 °C); retention times for compound obtained using (*S*,*S*)-**L1**: 4.9 min (minor), 5.7 min (major).

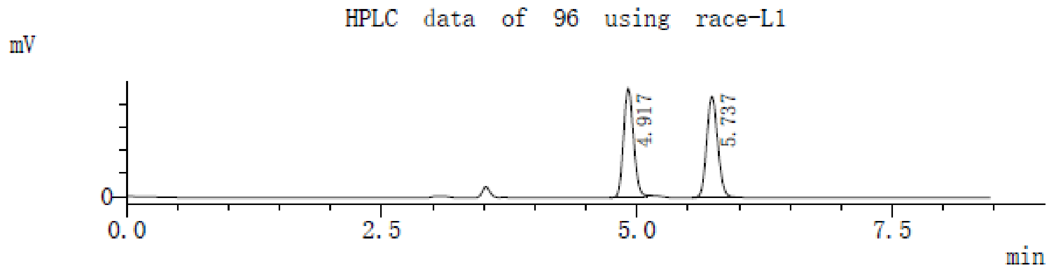

检测器A Ch1 214nm

Peak[#]	RetTime[min]	Height[uV]	Width[min]	Area[uV*s]	Area[%]
1	4.917	471742	0.106	3232014	49.699
2	5.737	436795	0.117	3271121	50.301

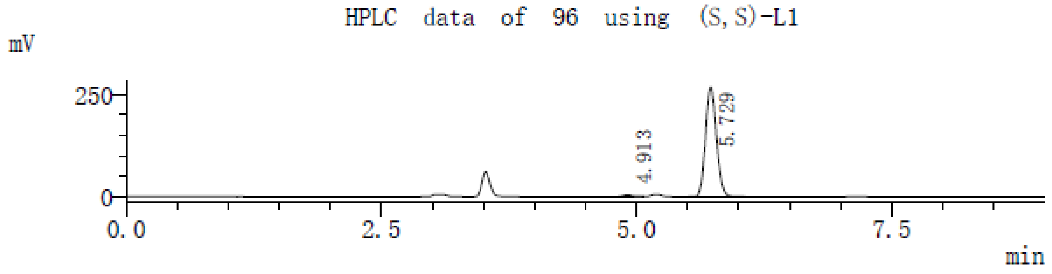

检测器A Ch1 214nm

Peak[#]	RetTime[min]	Height[uV]	Width[min]	Area[uV*s]	Area[%]
1	4.913	4169	0.101	26410	1.305
2	5.729	267342	0.116	1998037	98.695

Supplementary Figure 448. HPLC spectra for **96**

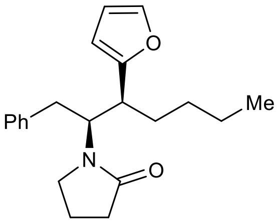

1-((2*S*,3*S*)-3-(furan-2-yl)-1-phenylheptan-2-yl)pyrrolidin-2-one (**97**)

HPLC analysis: The ee was determined to be 97% on a CHIRALPAK AD-H column (10% *i*PrOH in hexane, 1.0 mL/min, 40 °C); retention times for compound obtained using (*S*,*S*)-**L1**: 6.7 min (minor), 7.3 min (major).

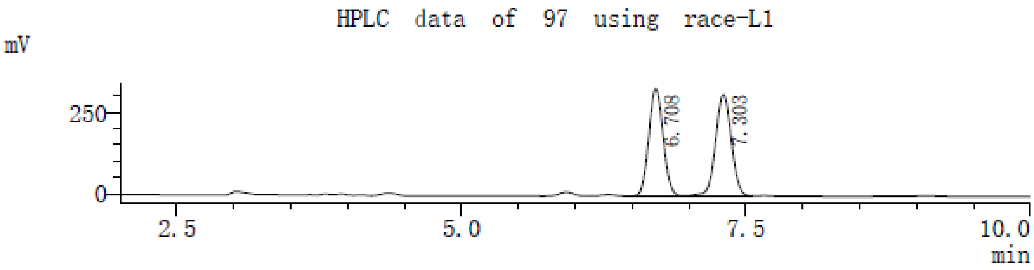

检测器A Ch1 214nm

Peak[#]	RetTime[min]	Height[uV]	Width[min]	Area[uV*s]	Area[%]
1	6.708	330536	0.138	2923548	49.085
2	7.303	312530	0.149	3032494	50.915

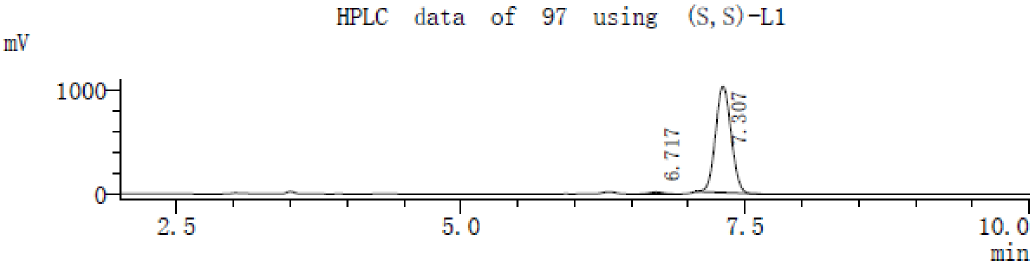

检测器A Ch1 214nm

Peak[#]	RetTime[min]	Height[uV]	Width[min]	Area[uV*s]	Area[%]
1	6.717	17913	0.135	152171	1.534
2	7.307	1024939	0.150	9767849	98.466

Supplementary Figure 449. HPLC spectra for **97**

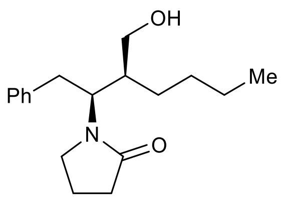

1-((2*S*,3*S*)-3-(hydroxymethyl)-1-phenylheptan-2-yl)pyrrolidin-2-one (**98**)

HPLC analysis: The ee was determined to be 97% on a CHIRALPAK AD-H column (15% *i*PrOH in hexane, 1.5 mL/min, 40 °C); retention times for compound obtained using (*S*,*S*)-**L1**: 4.0 min (minor), 5.1 min (major).

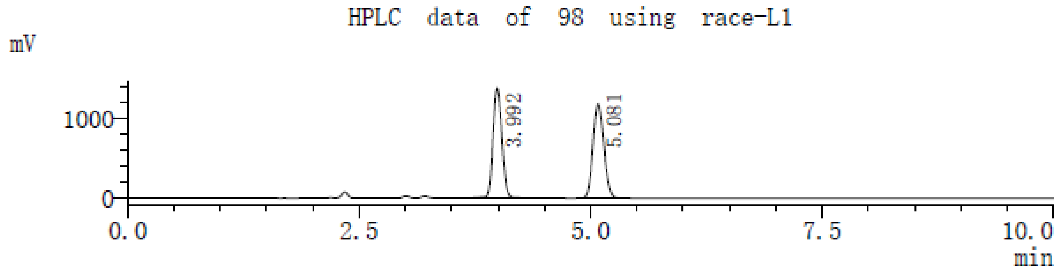

检测器A Ch1 214nm

Peak[#]	RetTime[min]	Height[uV]	Width[min]	Area[uV*s]	Area[%]
1	3.992	1368392	0.105	9203627	49.188
2	5.081	1171344	0.128	9507652	50.812

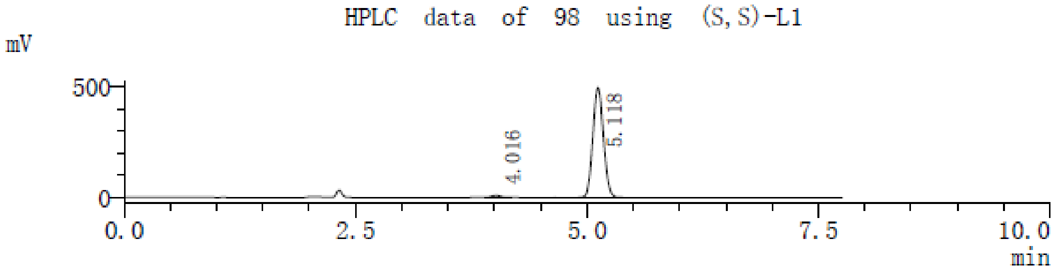

检测器A Ch1 214nm

Peak[#]	RetTime[min]	Height[uV]	Width[min]	Area[uV*s]	Area[%]
1	4.016	8719	0.102	55131	1.370
2	5.118	498267	0.124	3968736	98.630

Supplementary Figure 450. HPLC spectra for **98**

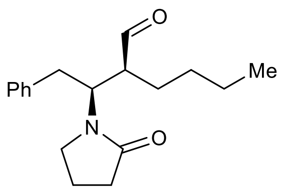

(*S*)-2-((*S*)-1-(2-oxopyrrolidin-1-yl)-2-phenylethyl)hexanal (**99**)

<u>HPLC analysis:</u> The ee was determined to be 97% on a CHIRALPAK AD-H column (8% *i*PrOH in hexane, 0.8 mL/min, 40 ^{o}C); retention times for compound obtained using (*S*,*S*)-**L1**: 10.6 min (minor), 11.2 min (major).

HPLC data of 99 using race-L1

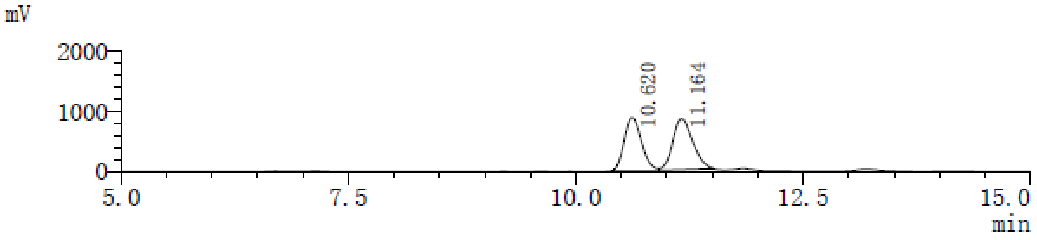

检测器A Ch1 214nm

Peak[#]	RetTime[min]	Height[uV]	Width[min]	Area[uV*s]	Area[%]
1	10.620	892751	0.206	11785751	47.910
2	11.164	847286	0.234	12814157	52.090

HPLC data of 99 using (S,S)-L1

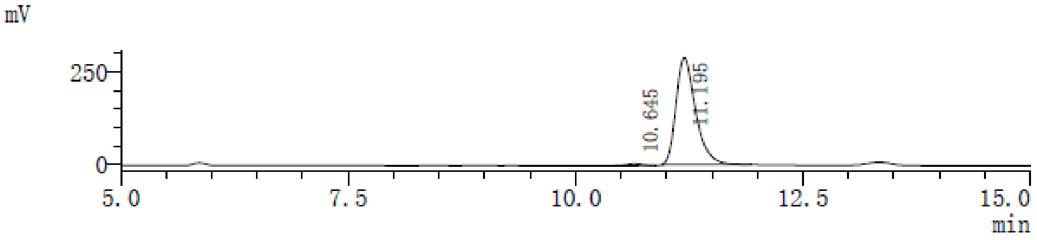

检测器A Ch1 214nm

Peak[#]	RetTime[min]	Height[uV]	Width[min]	Area[uV*s]	Area[%]
1	10.645	4836	0.191	58525	1.338
2	11.195	288771	0.223	4317056	98.662

Supplementary Figure 451. HPLC spectra for **99**

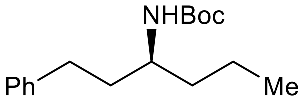

tert-butyl (*S*)-(1-phenylhexan-3-yl)carbamate (**100-Boc**)

HPLC analysis: The ee was determined to be 90% & 91% on a CHIRALPAK AD-H column (10% *i*PrOH in hexane, 1.0 mL/min, 40 °C); retention times for compound obtained using (*S*)-**L2** & (*R*)-**L2**: 4.3 min (minor), 4.6 min (major).

HPLC data of 100-Boc using rac-L2

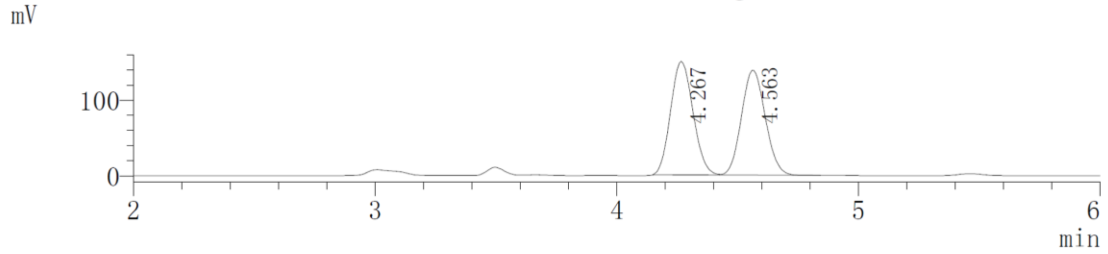

检测器A Ch1 214nm

Peak[#]	RetTime[min]	Height[uV]	Width[min]	Area[uV*s]	Area[%]
1	4.267	150532	0.102	970180	50.519
2	4.563	138773	0.107	950228	49.481

HPLC data of 100-Boc using (S)-L2

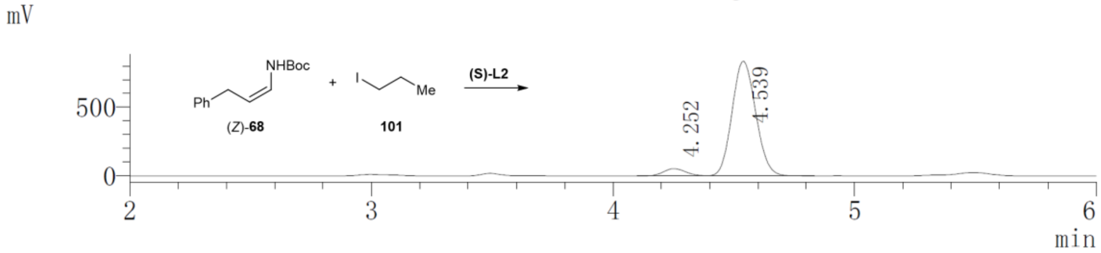

检测器A Ch1 214nm

Peak[#]	RetTime[min]	Height[uV]	Width[min]	Area[uV*s]	Area[%]
1	4.252	52756	0.101	340475	5.619
2	4.539	838542	0.107	5718535	94.381

Supplementary Figure 452. HPLC spectra for **100-Boc** from **(*Z*)-68** and **101**

HPLC data of 100-Boc using rac-L2

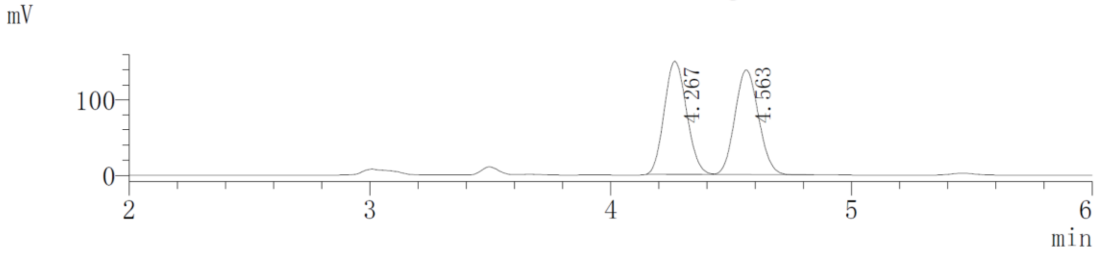

检测器A Ch1 214nm

Peak[#]	RetTime[min]	Height[uV]	Width[min]	Area[uV*s]	Area[%]
1	4.267	150532	0.102	970180	50.519
2	4.563	138773	0.107	950228	49.481

HPLC data of 100-Boc using (R)-L2

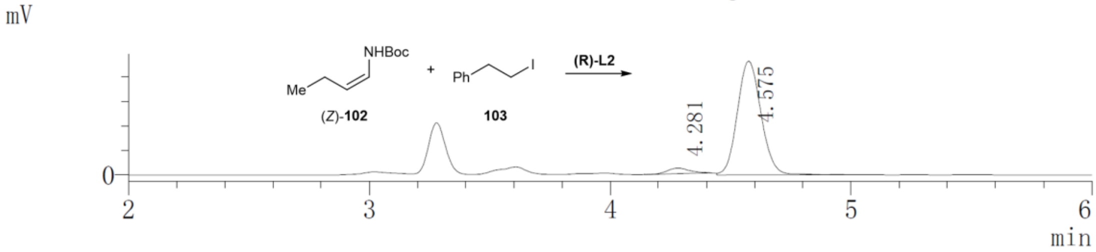

检测器A Ch1 214nm

Peak[#]	RetTime[min]	Height[uV]	Width[min]	Area[uV*s]	Area[%]
1	4.281	22718	0.098	148368	4.465
2	4.575	465011	0.105	3174238	95.535

Supplementary Figure 453. HPLC spectra for **100-Boc** from **(*Z*)-102** and **103**

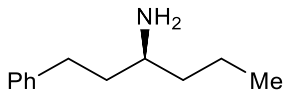

(*S*)-1-phenylhexan-3-amine (**100**)

<u>HPLC analysis</u>: The ee was determined by the product of **100** with $(Boc)_2O$ and Et_3N to be 88% & 91% on a CHIRALPAK AD-H column (10% *i*PrOH in hexane, 1.0 mL/min, 40 ^{o}C); retention times for compound obtained using (*S*)-**L**2 & (*R*)-**L**2: 4.3 min (major), 4.6 min (minor).

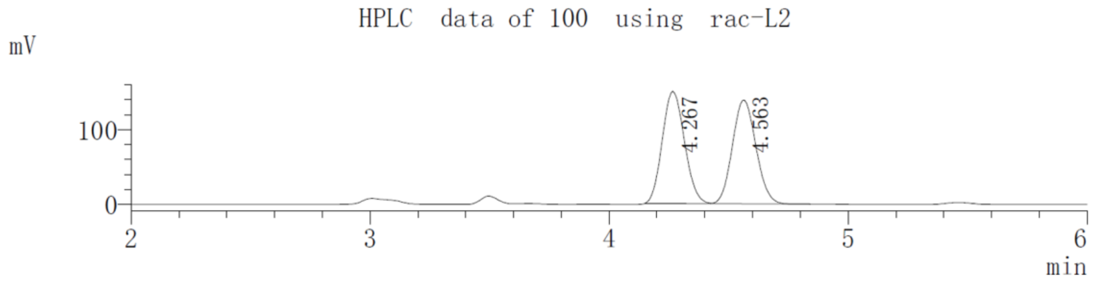

检测器A Ch1 214nm

Peak[#]	RetTime[min]	Height[uV]	Width[min]	Area[uV*s]	Area[%]
1	4.267	150532	0.102	970180	50.519
2	4.563	138773	0.107	950228	49.481

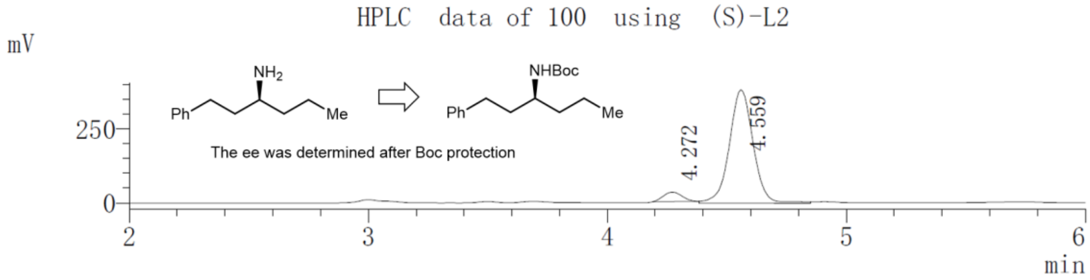

检测器A Ch1 214nm

Peak[#]	RetTime[min]	Height[uV]	Width[min]	Area[uV*s]	Area[%]
1	4.272	31600	0.087	170624	6.067
2	4.559	382055	0.103	2641576	93.933

Supplementary Figure 454. HPLC spectra for **100** from ***(Z)*-68** and **101**

HPLC data of 100 using rac-L2

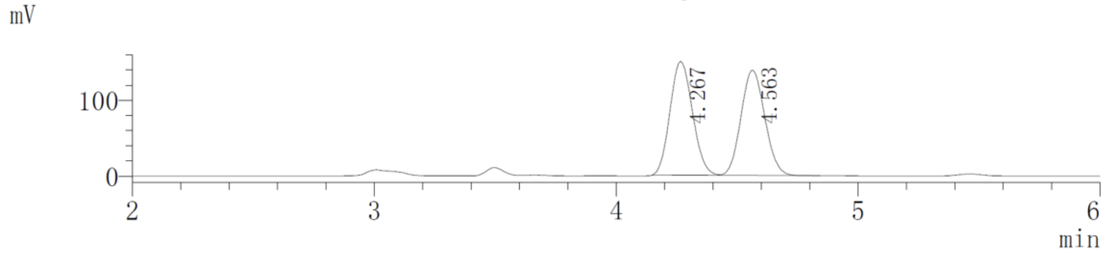

检测器A Ch1 214nm

Peak[#]	RetTime[min]	Height[uV]	Width[min]	Area[uV*s]	Area[%]
1	4.267	150532	0.102	970180	50.519
2	4.563	138773	0.107	950228	49.481

HPLC data of 100 using (R)-L2

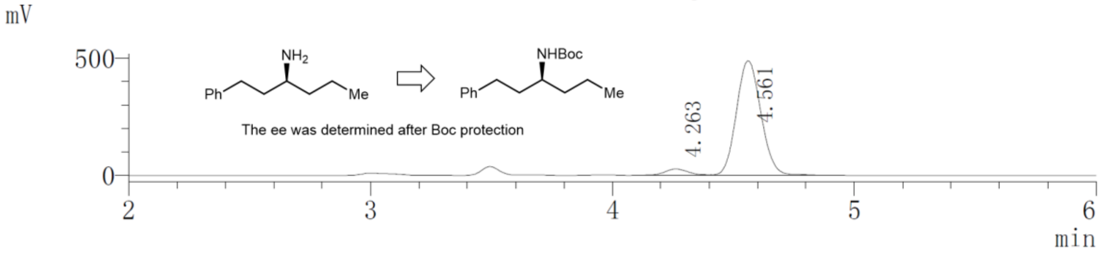

检测器A Ch1 214nm

Peak[#]	RetTime[min]	Height[uV]	Width[min]	Area[uV*s]	Area[%]
1	4.263	26093	0.101	170103	4.726
2	4.561	488495	0.109	3429090	95.274

Supplementary Figure 455. HPLC spectra for **100** from ***(Z)*-102** and **103**

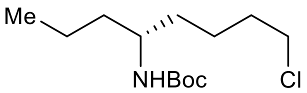

tert-butyl (*R*)-(8-chlorooctan-4-yl)carbamate (**106**)

<u>HPLC analysis</u>: The ee was determined by the product of **106** with NaI to be 91% on a CHIRALPAK AD-H column (8% *i*PrOH in hexane, 0.8 mL/min, 40 ^{o}C); retention times for compound obtained using (*S*)-**L2**: 5.3 min (major), 5.6 min (minor).

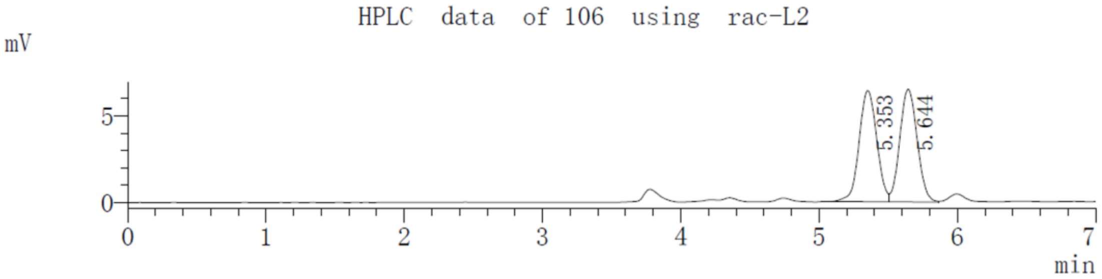

检测器A Ch2 254nm

Peak[#]	RetTime[min]	Height[uV]	Width[min]	Area[uV*s]	Area[%]
1	5.353	6412	0.138	57095	50.186
2	5.644	6510	0.137	56671	49.814

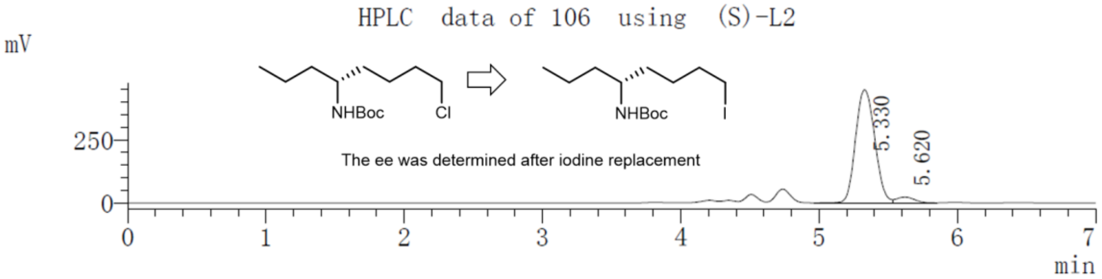

检测器A Ch2 254nm

Peak[#]	RetTime[min]	Height[uV]	Width[min]	Area[uV*s]	Area[%]
1	5.330	449054	0.154	4345313	95.251
2	5.620	23353	--	216648	4.749

Supplementary Figure 456. HPLC spectra for **106**

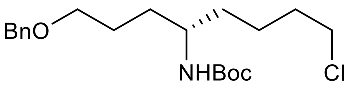

tert-butyl (*S*)-(1-(benzyloxy)-8-chlorooctan-4-yl)carbamate (**107**)

HPLC analysis: The ee was determined to be 89% on a CHIRALCEL OD-H column (5% *i*PrOH in hexane, 1.0 mL/min, 40 °C); retention times for compound obtained using (*S*)-**L2**: 7.0 min (major), 7.7 min (minor).

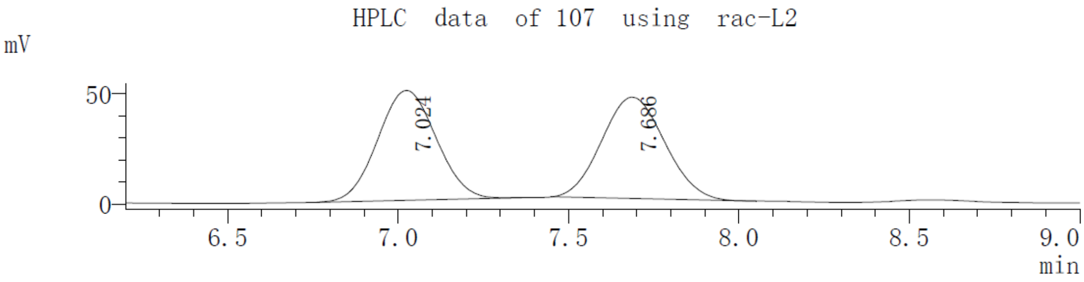

检测器A Ch1 214nm

Peak[#]	RetTime[min]	Height[uV]	Width[min]	Area[uV*s]	Area[%]
1	7.024	49637	0.188	592747	50.154
2	7.686	45784	0.204	589115	49.846

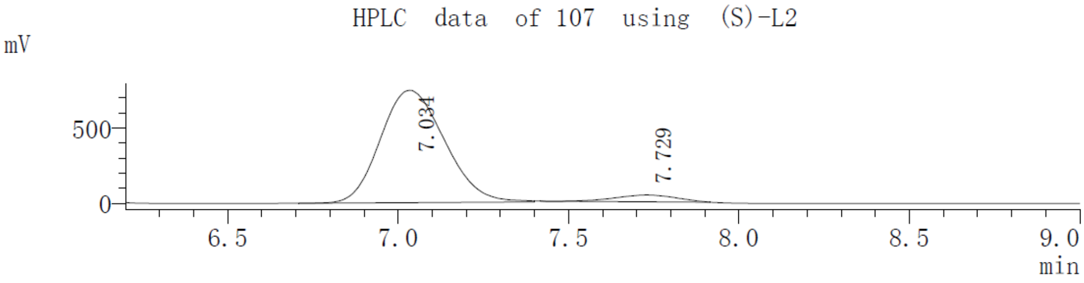

检测器A Ch1 214nm

Peak[#]	RetTime[min]	Height[uV]	Width[min]	Area[uV*s]	Area[%]
1	7.034	751618	0.207	10132877	94.537
2	7.729	46098	0.205	585564	5.463

Supplementary Figure 457. HPLC spectra for **107**

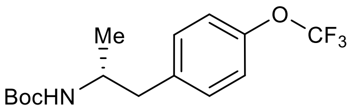

tert-butyl (*R*)-(1-(4-(trifluoromethoxy)phenyl)propan-2-yl)carbamate (**111**)

<u>HPLC analysis</u>: The ee was determined to be 81% on a CHIRALPAK AD-H column (5% *i*PrOH in hexane, 1.0 mL/min, 25 °C); retention times for compound obtained using (*S*)-**L2**: 4.8 min (minor), 5.2 min (major).

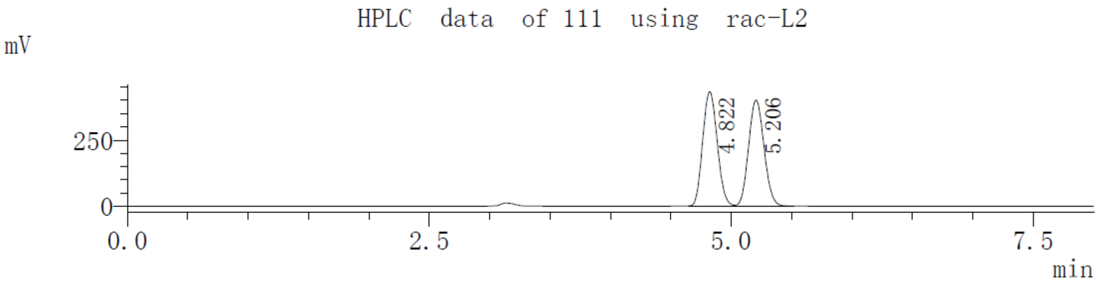

检测器A Ch1 214nm

Peak[#]	RetTime[min]	Height[uV]	Width[min]	Area[uV*s]	Area[%]
1	4.822	430894	0.133	3612578	50.386
2	5.206	399525	0.140	3557259	49.614

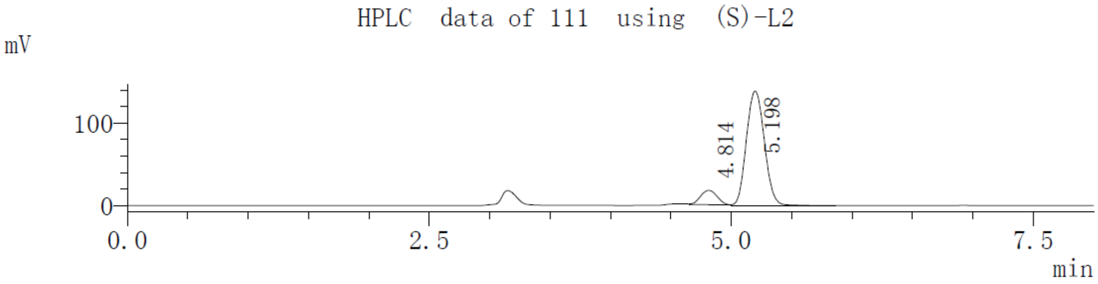

检测器A Ch1 214nm

Peak[#]	RetTime[min]	Height[uV]	Width[min]	Area[uV*s]	Area[%]
1	4.814	17215	0.154	163249	10.172
2	5.198	138946	0.164	1441701	89.828

Supplementary Figure 458. HPLC spectra for **111**

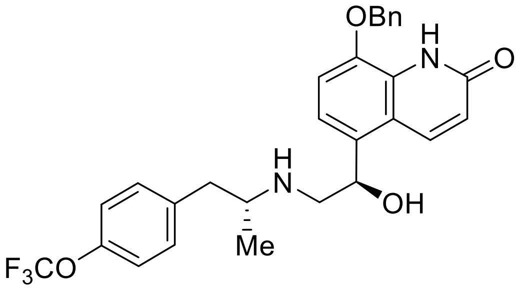

8-(benzyloxy)-5-((*R*)-1-hydroxy-2-(((*R*)-1-(4-(trifluoromethoxy)phenyl)propan-2-yl)amino)ethyl)quinolin-2(1*H*)-one (**113**)

<u>HPLC analysis</u>: The dr was determined to be 90.5:9.5 on a CHIRALCEL OD-H column (15% *i*PrOH in hexane, 1.5 mL/min, 40 °C); retention times for compound obtained using (*S*)-**L2**: 17.4 min (major), 27.0 min (minor).

HPLC data of 113 using rac-L2

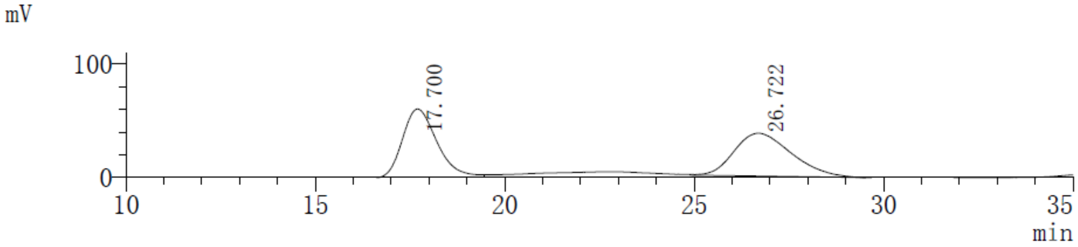

检测器A Ch1 214nm

Peak[#]	RetTime[min]	Height[uV]	Width[min]	Area[uV*s]	Area[%]
1	17.700	61177	0.973	3972000	49.916
2	26.722	37631	1.651	3985369	50.084

HPLC data of 113 using (S)-L2

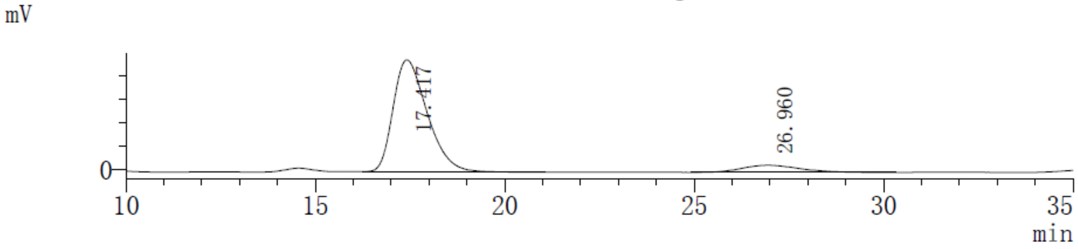

检测器A Ch1 214nm

Peak[#]	RetTime[min]	Height[uV]	Width[min]	Area[uV*s]	Area[%]
1	17.417	239591	0.965	15092850	90.537
2	26.960	15111	1.624	1577532	9.463

Supplementary Figure 459. HPLC spectra for **113**

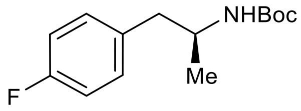

tert-butyl (*R*)-(1-(4-fluorophenyl)propan-2-yl)carbamate (**114**)

<u>HPLC analysis</u>: The ee was determined to be 80% on a CHIRALPAK AD-H column (5% *i*PrOH in hexane, 1.0 mL/min, 25 ^oC); retention times for compound obtained using (*R*)-**L2**: 5.8 min (major), 6.3 min (minor).

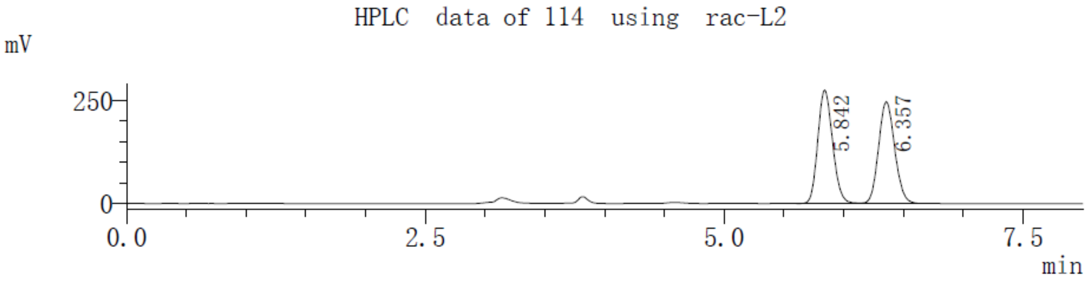

检测器A Ch1 214nm

Peak[#]	RetTime[min]	Height[uV]	Width[min]	Area[uV*s]	Area[%]
1	5.842	275974	0.133	2371808	50.554
2	6.357	247100	0.145	2319811	49.446

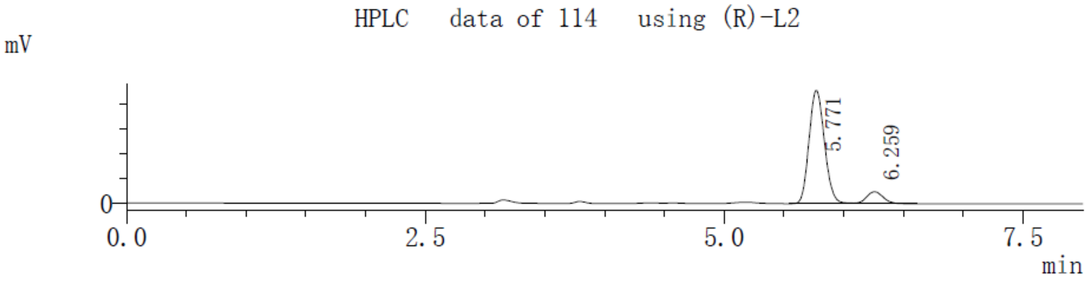

检测器A Ch1 214nm

Peak[#]	RetTime[min]	Height[uV]	Width[min]	Area[uV*s]	Area[%]
1	5.771	458997	0.140	4122757	89.755
2	6.259	48754	0.149	470594	10.245

Supplementary Figure 460. HPLC spectra for **114**

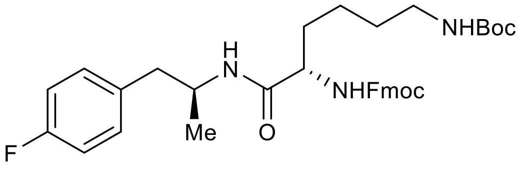

(9*H*-fluoren-9-yl)methyl *tert*-butyl ((*S*)-6-(((*S*)-1-(4-fluorophenyl)propan-2-yl)amino)-6-oxohexane-1,5-diyl)dicarbamate (**115**)

HPLC analysis: The dr was determined to be 90:10 on a CHIRALPAK AD-H column (15% *i*PrOH in hexane, 1.5 mL/min, 40 °C); retention times for compound obtained using (*R*)-**L2**: 6.7 min (minor), 8.0 min (major).

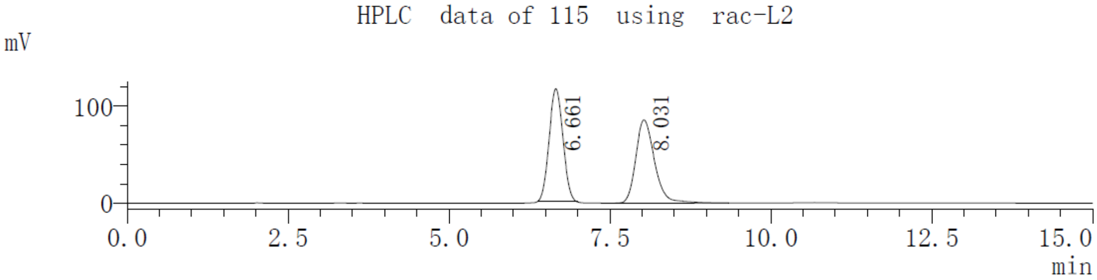

检测器A Ch2 254nm

Peak[#]	RetTime[min]	Height[uV]	Width[min]	Area[uV*s]	Area[%]
1	6.661	116091	0.236	1731438	50.036
2	8.031	85500	0.304	1728952	49.964

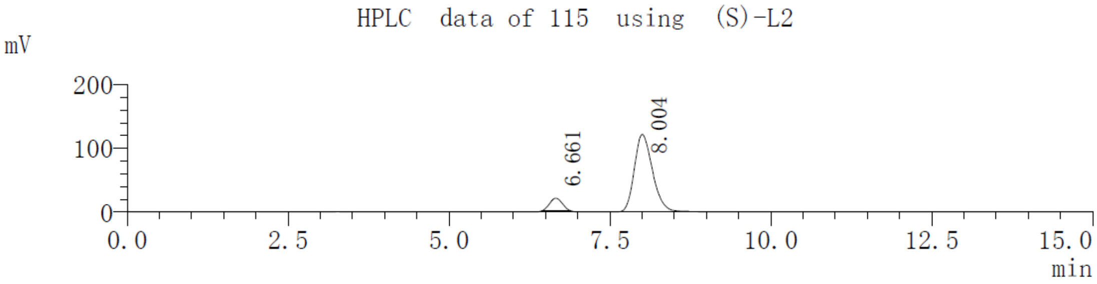

检测器A Ch2 254nm

Peak[#]	RetTime[min]	Height[uV]	Width[min]	Area[uV*s]	Area[%]
1	6.661	19686	0.221	265530	9.890
2	8.004	121975	0.302	2419379	90.110

Supplementary Figure 461. HPLC spectra for **115**

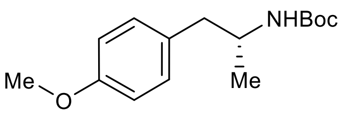

tert-butyl (*R*)-(1-(4-methoxyphenyl)propan-2-yl)carbamate (**116**)

HPLC analysis: The ee was determined to be 86% on a CHIRALCEL OJ-H column (10% *i*PrOH in hexane, 1.0 mL/min, 25 °C); retention times for compound obtained using (*S*)-**L2**: 4.6 min (minor), 5.2 min (major).

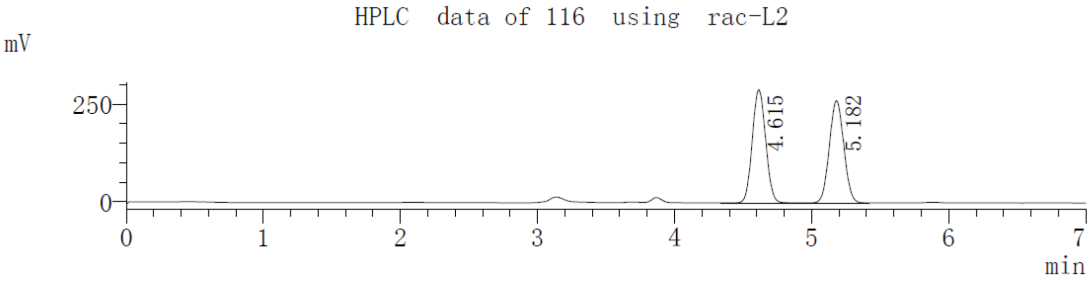

检测器A Ch1 214nm

Peak[#]	RetTime[min]	Height[uV]	Width[min]	Area[uV*s]	Area[%]
1	4.615	290864	0.106	1973777	50.591
2	5.182	262117	0.115	1927697	49.409

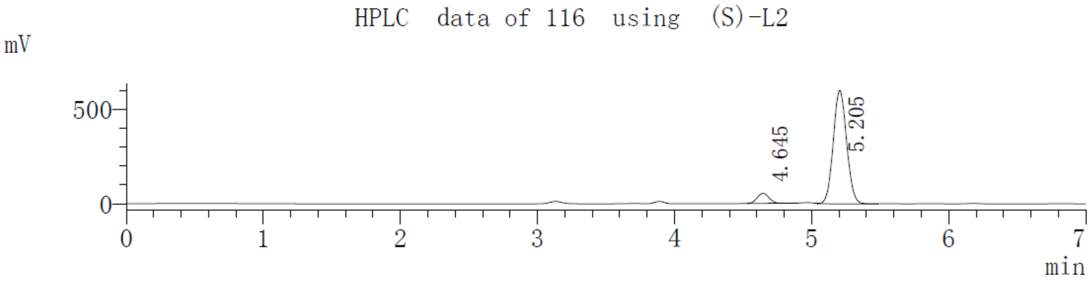

检测器A Ch1 214nm

Peak[#]	RetTime[min]	Height[uV]	Width[min]	Area[uV*s]	Area[%]
1	4.645	53031	0.095	316115	7.157
2	5.205	602484	0.106	4100625	92.843

Supplementary Figure 462. HPLC spectra for **116**

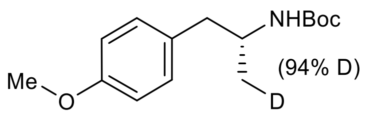

tert-butyl (*R*)-(1-(4-methoxyphenyl)propan-2-yl-3-d)carbamate (**117**)

<u>HPLC analysis</u>: The ee was determined to be 84% on a CHIRALCEL OJ-H column (10% *i*PrOH in hexane, 1.0 mL/min, 25 °C); retention times for compound obtained using (*S*)-**L2**: 4.6 min (minor), 5.2 min (major).

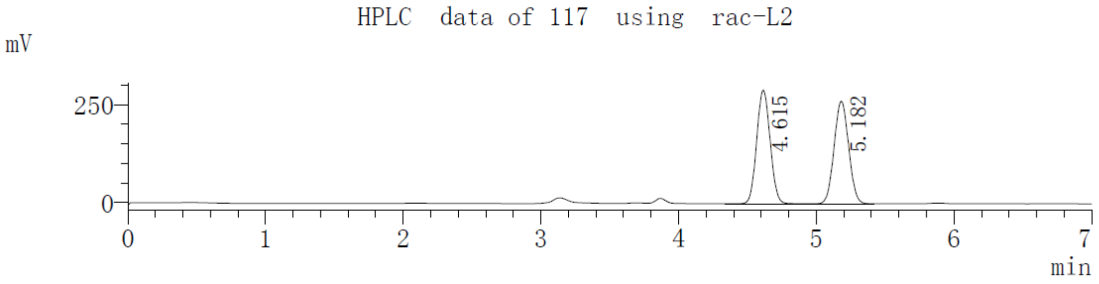

检测器A Ch1 214nm

Peak[#]	RetTime[min]	Height[uV]	Width[min]	Area[uV*s]	Area[%]
1	4.615	290864	0.106	1973777	50.591
2	5.182	262117	0.115	1927697	49.409

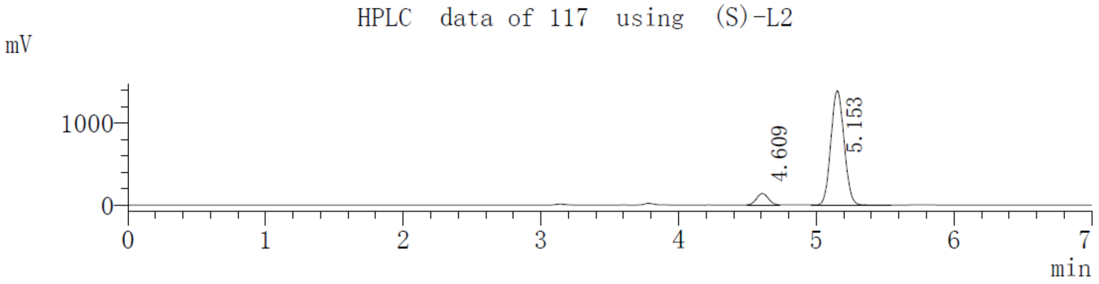

检测器A Ch1 214nm

Peak[#]	RetTime[min]	Height[uV]	Width[min]	Area[uV*s]	Area[%]
1	4.609	139754	0.093	822122	8.101
2	5.153	1393946	0.105	9326651	91.899

Supplementary Figure 463. HPLC spectra for **117**

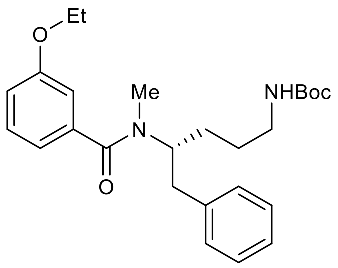

tert-butyl (*R*)-(4-(3-ethoxy-N-methylbenzamido)-5-phenylpentyl)carbamate (**121**)

<u>HPLC analysis:</u> The ee was determined to be 90% on a CHIRALPAK AD-H column (10% *i*PrOH in hexane, 1.0 mL/min, 40 °C); retention times for compound obtained using (*S*,*S*)-**L1**: 12.9 min (minor), 16.7 min (major).

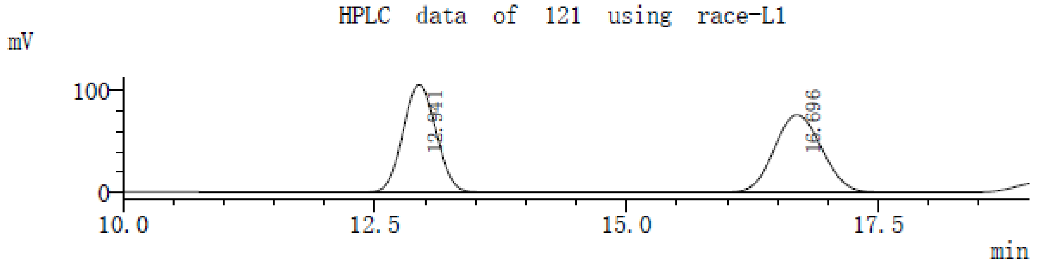

检测器A Ch1 214nm

Peak[#]	RetTime[min]	Height[uV]	Width[min]	Area[uV*s]	Area[%]
1	12.941	104913	0.361	2403063	49.390
2	16.696	75472	0.518	2462446	50.610

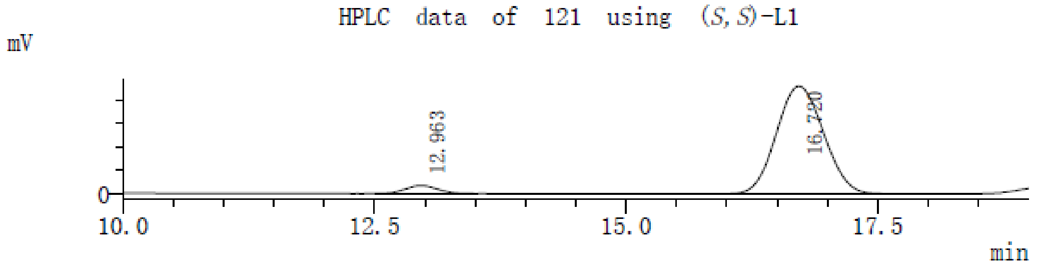

检测器A Ch1 214nm

Peak[#]	RetTime[min]	Height[uV]	Width[min]	Area[uV*s]	Area[%]
1	12.963	6957	0.357	157041	4.972
2	16.720	91981	0.516	3001666	95.028

Supplementary Figure 464. HPLC spectra for **121**

Supplementary References

[1] S. Pache, M. Lautens. Palladium-Catalyzed Sequential Alkylation−Alkenylation Reactions: New Three-Component Coupling Leading to Oxacycles. *Org. Lett.* **2003**, *5*, 4827.

[2] D. A. Quagliato, P. M. Andrae, E. M. Matelan. Efficient Procedure for the Reduction of α-Amino Acids to Enantiomerically Pure α-Methylamines. *J. Org. Chem.* **2000**, *65*, 5037.

[3] D. B. Diaz, C. C. G. Scully, S. K. Liew, S. Adachi, P. Trinchera, J. D. St Denis, A. K. Yudin. Synthesis of Aminoboronic Acid Derivatives from Amines and Amphoteric Boryl Carbonyl Compounds. *Angew. Chem. Int. Ed.* **2016**, *55*, 12659.

[4] H.-Y. Xu, Y. Zi, X.-P. Xu, S.-Y. Wang, S.-J. Ji. TFA-catalyzed C–N bond activation of enamides with indoles: efficient synthesis of 3,3-bisindolylpropanoates and other bisindolylalkanes. *Tetrahedron* **2013**, *69*, 1600.

[5] L. J. Goossen, J. E. Rauhaus, G. J. Deng. Ru-Catalyzed Anti-Markovnikov Addition of Amides to Alkynes: A Regio- and Stereoselective Synthesis of Enamides. *Angew. Chem. Int. Ed.* **2005**, *44*, 4042.

[6] L. J. Goossen, K. S. M. Salih, M. Blanchot. Synthesis of Secondary Enamides by Ruthenium-Catalyzed Selective Addition of Amides to Terminal Alkynes. *Angew. Chem. Int. Ed.* **2008**, *47*, 8492.

[7] T. Hashimoto, H. Nakatsu, Y. Takiguchi, K. Maruoka. Axially Chiral Dicarboxylic Acid Catalyzed Activation of Quinone Imine Ketals: Enantioselective Arylation of Enecarbamates. *J. Am. Chem. Soc.* **2013**, *135*, 16010.

[8] X.-Y. Bai, W. Zhao, X. Sun, B.-J. Li. Rhodium-Catalyzed Regiodivergent and Enantioselective Hydroboration of Enamides. *J. Am. Chem. Soc.* **2019**, *141*, 19870.

[9] X.-Y. Bai, W.-W. Zhang, Q. Li, B.-J. Li. Highly Enantioselective Synthesis of Propargyl Amides through Rh-Catalyzed Asymmetric Hydroalkynylation of

Enamides: Scope, Mechanism, and Origin of Selectivity. *J. Am. Chem. Soc.* **2018**, *140*, 506.

[10] M. Terada, K. Sorimachi. Enantioselective Friedel−Crafts Reaction of Electron-Rich Alkenes Catalyzed by Chiral Brønsted Acid. *J. Am. Chem. Soc.* **2007**, *129*, 292.

[11] W.-W. Zhang, S.-L. Zhang, B.-J. Li. Highly Enantioselective Synthesis of Propargyl Amide with Vicinal Stereocenters through Ir-Catalyzed Hydroalkynylation. *Angew. Chem. Int. Ed.* **2020**, *59*, 6874.

[12] A. Carboni, G. Dagousset, E. Magnier, G. Masson. Photoredox-Induced Three-Component Oxy-, Amino-, and Carbotrifluoromethylation of Enecarbamates. *Org. Lett.* **2014**, *16*, 1240.

[13] K. C. Cartwright, S. B. Lang, J. A. Tunge. Photoinduced Kochi Decarboxylative Elimination for the Synthesis of Enamides and Enecarbamates from N-Acyl Amino Acids. *J. Org. Chem.* **2019**, *84*, 2933.

[14] L. Longwitz, S. Jopp, T. Werner. Organocatalytic Chlorination of Alcohols by P(III)/P(V) Redox Cycling. *J. Org. Chem.* **2019**, *84*, 7863.

[15] Y. Xi, J. F. Hartwig. Diverse Asymmetric Hydrofunctionalization of Aliphatic Internal Alkenes through Catalytic Regioselective Hydroboration. *J. Am. Chem. Soc.* **2016**, *138*, 6703.

[16] J. Schmidt, J. Choi, A. T. Liu, M. Slusarczyk, G. C. Fu. A general, modular method for the catalytic asymmetric synthesis of alkylboronate esters. *Science* **2016**, *354*, 1265.

[17] J. M. Beierle, W. S. Horne, J. H. van Maarseveen, B. Waser, J. C. Reubi, M. R. Ghadiri. Conformationally Homogeneous Heterocyclic Pseudotetrapeptides as Three-Dimensional Scaffolds for Rational Drug Design: Receptor-Selective Somatostatin Analogues. *Angew. Chem. Int. Ed.* **2009**, *48*, 4725.

[18] G. K. Friestad, J.-C. Marie, Y. Suh, J. Qin. Mn-Mediated Coupling of Alkyl Iodides and Chiral N-Acylhydrazones: Optimization, Scope, and Evidence for a Radical Mechanism. *J. Org. Chem.* **2006**, *71*, 7016.

[19] J. R. Jacobsen, J. B. Aggen, T. J. Church, U. Klein, J. W. Pfeiffer, T. M. Pulido-Rios, G. R. Thomas, C. Yu, E. J. Moran. Multivalent design of long-acting β2-adrenoceptor agonists incorporating biarylamines. *Bioorg. Med. Chem. Lett.* **2014**, *24*, 2625.

[20] J. Fan, Y. Ye, G. Chu, Z. Zhang, Y. Fu, Y.-M. Li, J. Shi. Semisynthesis of Ubiquitin and SUMO-Rhodamine 110-Glycine through Aminolysis of Boc-Protected Thioester Counterparts. *J. Org. Chem.* **2019**, *84*, 14861.

[21] Z. Cai, G. Liu, G. Jiao, C. H. Senanayake, W. Tang. Practical Syntheses of N-Acetyl (*E*)-β-Arylenamides. *Synthesis-Stuttgart* **2013**, *45*, 3355.

[22] X. Lu, B. Xiao, Z. Zhang, T. Gong, W. Su, J. Yi, Y. Fu, L. Liu. Practical carbon–carbon bond formation from olefins through nickel-catalyzed reductive olefin hydrocarbonation. *Nat. Commun.* **2016**, *7*, 11129.

[23] N. Niljianskul, S. Zhu, S. L. Buchwald. Enantioselective Synthesis of α-Aminosilanes by Copper-Catalyzed Hydroamination of Vinylsilanes. *Angew. Chem. Int. Ed.* **2015**, *54*, 1638.

[24] F. B. Song, J. H. Snook, B. M. Foxman, B. B. Snider. Solid-state photodimerization of 2-phenylethenyl enamides. *Tetrahedron* **1998**, *54*, 13035.

[25] A. E. Buba, M. Arndt, L. J. Goossen, *Z*-Selective hydroamidation of terminal alkynes with secondary amides and imides catalyzed by a Ru/Yb-system. *J. Organomet. Chem.* **2011**, *696*, 170.

[26] M. J. Frisch, G. W. Trucks, H. B. Schlegel, G. E. Scuseria, M. A. Robb, J. R. Cheeseman, G. Scalmani, V. Barone, G. A. Petersson, H. Nakatsuji, X. Li, M. Caricato, A. V. Marenich, J. Bloino, B. G. Janesko, R. Gomperts, B. Mennucci, H. P. Hratchian, J. V. Ortiz, A. F. Izmaylov, J. L. Sonnenberg, D. Williams-Young, F. Ding, F. Lipparini, F. Egidi, J. Goings, B. Peng, A. Petrone, T. Henderson, D. Ranasinghe, V. G. Zakrzewski, J. Gao, N. Rega, G. Zheng, W. Liang, M. Hada, M. Ehara, K. Toyota, R. Fukuda, J. Hasegawa, M. Ishida, T. Nakajima, Y. Honda, O. Kitao, H. Nakai, T. Vreven, K. Throssell, J. A. Montgomery, Jr., J. E. Peralta, F. Ogliaro, M. J. Bearpark, J. J. Heyd, E. N.

Brothers, K. N. Kudin, V. N. Staroverov, T. A. Keith, R. Kobayashi, J. Normand, K. Raghavachari, A. P. Rendell, J. C. Burant, S. S. Iyengar, J. Tomasi, M. Cossi, J. M. Millam, M. Klene, C. Adamo, R. Cammi, J. W. Ochterski, R. L. Martin, K. Morokuma, O. Farkas, J. B. Foresman, and D. J. Fox, Gaussian 16, Revision C.01; Gaussian, Inc., Wallingford CT, **2019**.

[27] A. D. Becke. Density-functional thermochemistry. III. The role of exact exchange. *J. Chem. Phys.* **1993**, *98*, 5648.

[28] C. Lee, Yang, W. Parr, R. G. Development of the Colle-Salvetti correlation-energy formula into a functional of the electron density. *Phys. Rev. B: Condens. Matter Mater. Phys.* **1988**, *37*, 785.

[29] A. V. Marenich, C. J Cramer, D. G Truhlar. Universal Solvation Model Based on Solute Electron Density and on a Continuum Model of the Solvent Defined by the Bulk Dielectric Constant and Atomic Surface Tensions. *J. Phys. Chem. B* **2009**, *113*, 6378.

[30] S. Grimme, J Antony, S. Ehrlich, H. Krieg. A consistent and accurate ab initio parametrization of density functional dispersion correction (DFT-D) for the 94 elements H-Pu. *J. Chem. Phys.* **2010**, *132*, 154104.

[31] P. J. Hay, W. R. Wadt. Ab initio effective core potentials for molecular calculations. Potentials for the transition metal atoms Sc to Hg. *J. Chem. Phys.* **1985**, *82*, 270.

[32] W. R. Wadt, P. J. Hay. Ab initio effective core potentials for molecular calculations. Potentials for main group elements Na to Bi. *J. Chem. Phys.* **1985**, *82*, 284.

[33] P. J. Hay, W. R. Wadt. Ab initio effective core potentials for molecular calculations. Potentials for K to Au including the outermost core orbitals. *J. Chem. Phys.* **1985**, *82*, 299.

[34] L. von Szentpaly, P. Fuentealba, H. Preuss, H. Stoll. Pseudopotential calculations on Rb_2^+, Cs_2^+, RbH^+, CsH^+ and the mixed alkali dimer ions. *Chem. Phys. Lett.* **1982**, *93*, 555.

[35] M. Dolg, U. Wedig, H. Stoll, H. Preuss. Energyadjusted ab initio pseudopotentials for the first row transition elements. *J. Chem. Phys.* **1987**, *86*, 866.

[36] P. Schwerdtfeger, M. Dolg, W. H. E. Schwarz, G. A. Bowmaker, P. D. W. Boyd. Relativistic effects in gold chemistry. I. Diatomic gold compounds. *J. Chem. Phys.* **1989**, *91*, 1762.

[37] CYLview, 1.0b; C. Y. Legault, Université de Sherbrooke, **2009** (http://www.cylview.org).
