# Supplementary material for: Catalytic asymmetric reductive hydroalkylation of enamides and enecarbamates to chiral aliphatic amines
Source: Nat Commun. 2021 Feb 26;12:1313. doi: 10.1038/s41467-021-21600-x (PMC7910428; doi:10.1038/s41467-021-21600-x)
Supplement: Supplementary file 3 — Description of Additional Supplementary Files [file 41467_2021_21600_MOESM3_ESM.pdf]

Description of Additional Supplementary Files

File Name: Supplementary Data 1

Description: Cartesian coordinates of the calculated transition states.
