# Supplementary material for: Catalytic asymmetric reductive hydroalkylation of enamides and enecarbamates to chiral aliphatic amines
Source: Nat Commun. 2021 Feb 26;12:1313. doi: 10.1038/s41467-021-21600-x (PMC7910428; doi:10.1038/s41467-021-21600-x)
Supplement: Supplementary file 4 — Supplementary Data 1 [file 41467_2021_21600_MOESM4_ESM.zip › Supplementary Data 1.docx]

**Cartesian coordinates of the calculated transition states.**

**TS-α1**

Thermal Correction to Free Energy: 0.866797 Hartree

SCF energy: -2401.150346 Hartree

Gibbs free energy: -2400.283549 Hartree

Coordinates:

N 0.43716500 -3.45070400 2.25050600

C -1.05747900 -1.64852900 3.03409100

H -1.94329400 -2.27232400 3.20207600

H -1.04023100 -0.85020100 3.77740100

H -1.14429600 -1.22417500 2.03300200

O 0.97487800 -2.28283800 4.13710100

C 0.20195600 -2.46900100 3.19460300

C 1.60824000 -4.30748200 2.41464100

H 2.31954500 -4.17419900 1.59288100

H 2.09051100 -4.03554600 3.35108400

H 1.30146900 -5.35811000 2.44824700

C 0.90983700 0.23929700 0.69560000

C -1.27823600 0.10445900 -0.55703400

O 1.52749300 0.90136700 1.69453000

N 1.39878400 -0.88949700 0.34201200

O -2.16874400 0.76532400 -1.31134900

N -1.42235100 -1.16993100 -0.46320100

C 2.67527500 0.10473100 2.09107800

C -3.18406600 -0.18794500 -1.72222800

Ni 0.15284200 -2.45562100 -0.50092700

Br 0.46206100 -2.04508200 -3.01316300

H 3.56271900 0.72720700 1.97158400

H 2.54140200 -0.17116300 3.13785400

H -3.29241800 -0.11884300 -2.80498400

H -4.11689900 0.09773200 -1.23073200

C -2.63111200 -1.55171500 -1.23954400

H -2.29144300 -2.14935900 -2.08848800

C 2.62920400 -1.11472800 1.13340800

H 2.50009600 -2.02786300 1.71427500

C -0.23659400 1.00427200 0.06492800

H 1.12329000 -3.53300400 -0.73342700

C -1.03412500 -5.08791700 -0.87314400

H -1.28150200 -6.10106900 -0.52433400

H -1.95385100 -4.50119200 -0.89744200

H -0.65285000 -5.16826400 -1.89585800

C 0.00038300 -4.47218900 0.03897700

C -0.36886500 -3.59792500 1.09261300

H -1.41557300 -3.35171300 1.22430200

H 0.86642000 -5.11032300 0.19411100

C 3.86434100 -1.26886800 0.26367300

C 3.77152000 -1.50959800 -1.11150100

C 5.13153600 -1.17146800 0.85413800

C 4.92985800 -1.62114200 -1.88554300

H 2.79563100 -1.59304900 -1.57817100

C 6.28799300 -1.28514700 0.08041900

H 5.21902600 -0.99783600 1.92391900

C 6.19088500 -1.50327600 -1.29676000

H 4.84230000 -1.79421600 -2.95524500

H 7.26271100 -1.19943200 0.55308000

H 7.08946200 -1.58423500 -1.90226200

C -3.63005100 -2.34315500 -0.42732900

C -4.31980400 -3.40724400 -1.01883100

C -3.91473400 -2.00425600 0.90105600

C -5.27104100 -4.12875700 -0.29362800

H -4.10627600 -3.67369400 -2.05094400

C -4.85836600 -2.72809100 1.63079700

H -3.38989200 -1.17763300 1.36587700

C -5.53881700 -3.79422600 1.03548400

H -5.79643000 -4.95443900 -0.76551100

H -5.06294500 -2.45852600 2.66339400

H -6.27303900 -4.35863900 1.60355800

C 0.34959300 1.88807000 -1.11224300

H 0.42049800 1.24177800 -1.99162300

H -0.38758600 2.66403500 -1.32776500

C -0.89122300 1.88670900 1.19603800

H -1.04117100 1.23708500 2.06274100

H -0.13955200 2.62653600 1.47680500

C 1.70773600 2.47296000 -0.82556700

C 2.85249300 1.77219300 -1.22738700

C 1.88602500 3.65871700 -0.10793900

C 4.12540500 2.21132000 -0.87916700

H 2.73997900 0.85874900 -1.80157100

C 3.16593600 4.10440300 0.23444300

H 1.02009200 4.23898000 0.19975700

C 4.31281100 3.38253000 -0.12554300

H 4.98245700 1.62277200 -1.19267100

H 3.25672400 5.02568700 0.79898500

C -2.19314600 2.56760300 0.86285300

C -3.41191200 1.95367100 1.18342600

C -2.23876000 3.81651400 0.23883700

C -4.62566200 2.54638900 0.85245700

H -3.40612000 0.99442800 1.69420000

C -3.45917700 4.41228200 -0.09430700

H -1.31316600 4.33644100 0.00602800

C -4.67925100 3.78746600 0.19266500

H -5.54643000 2.03220400 1.11249600

H -3.44312800 5.37955400 -0.58381100

C 5.73139500 3.80657000 0.27580600

C 6.59092500 3.99937600 -0.99130900

H 7.60981800 4.29883900 -0.71676900

H 6.66016900 3.07778500 -1.57872100

H 6.16887900 4.78016200 -1.63531600

C 6.35566700 2.69677200 1.14956300

H 6.40434600 1.74099300 0.61790500

H 7.37650900 2.97216000 1.44234500

H 5.77008500 2.54458700 2.06435600

C 5.74532500 5.11871100 1.07484400

H 5.33060600 5.95194000 0.49583200

H 5.17580700 5.03345500 2.00738600

H 6.77730100 5.37724700 1.33814300

C -6.03869700 4.39633100 -0.17453500

C -6.79142800 3.42398100 -1.10799000

H -6.96269600 2.45403600 -0.62901100

H -7.76853900 3.83997900 -1.38263900

H -6.22410800 3.24985900 -2.03012100

C -6.86705000 4.61295700 1.10958700

H -7.84640500 5.04079900 0.86253200

H -7.03750700 3.67293000 1.64506100

H -6.35669900 5.30302500 1.79208500

C -5.89867400 5.74663300 -0.89421400

H -5.39495700 6.49173000 -0.26780700

H -5.33793000 5.65196100 -1.83117500

H -6.89326500 6.13619800 -1.13940800

**TS-α2**

Thermal Correction to Free Energy: 0.867470 Hartree

SCF energy: -2401.155607 Hartree

Gibbs free energy: -2400.288137 Hartree

Coordinates:

N 1.36528600 -3.40365500 -1.14966200

C 0.92762100 -2.77310500 -3.50713200

H 0.25613400 -1.94321800 -3.27666900

H 1.51529700 -2.51607300 -4.38944900

H 0.31094700 -3.65063400 -3.73177600

O 3.11389900 -2.99456900 -2.55365800

C 1.89520900 -3.05485200 -2.37431700

C 2.27940900 -3.75592100 -0.06771300

H 3.28738900 -3.48779100 -0.37308500

H 2.01179900 -3.21252000 0.83740200

H 2.23657900 -4.83308800 0.13303600

C -0.71173900 0.25573400 -1.85974000

C 0.83810600 0.82877300 0.05063100

O -1.14538400 0.61804500 -3.08463700

N -1.28607900 -0.75764200 -1.31757100

O 1.42278500 1.86468100 0.69242200

N 0.73729400 -0.27457900 0.68782900

C -2.30914600 -0.19734800 -3.38876200

C 1.87437100 1.36089900 1.98063400

Ni -0.66471700 -1.81596200 0.27597000

Br -2.23717000 -1.06637900 2.23097500

H -3.18283800 0.45809600 -3.38493200

H -2.16876000 -0.63232200 -4.37853400

H 1.51480400 2.04414200 2.75083200

H 2.96597700 1.36328100 1.96794800

C 1.24598200 -0.04484600 2.07047800

H 0.35777500 -0.03687200 2.70789700

C -2.34048100 -1.24181700 -2.24708400

H -2.03280500 -2.21783200 -2.63029500

C 0.43307200 1.12082300 -1.37597500

C -0.59464100 -3.91892400 0.28921800

H -0.26389200 -2.64763400 1.39856400

H 0.05754600 -4.44462800 0.98300800

C -2.02931100 -4.39014900 0.31140400

H -2.61804100 -3.92949700 -0.48698900

H -2.06947600 -5.48252800 0.18829300

H -2.50573100 -4.13573900 1.26309400

C -0.02304700 -3.39259500 -0.88714600

H -0.64080800 -3.34062000 -1.77066700

C 2.15962700 -1.15579500 2.53402500

C 1.63651600 -2.16178000 3.35683400

C 3.49333600 -1.24576300 2.11839200

C 2.42773700 -3.24361000 3.74856800

H 0.59944900 -2.09634000 3.67428000

C 4.28859700 -2.32238600 2.51365800

H 3.91371200 -0.48422000 1.47146100

C 3.75682100 -3.32766700 3.32555000

H 2.00746500 -4.01830300 4.38435800

H 5.32153100 -2.37877000 2.18069900

H 4.37478800 -4.16803800 3.62970300

C -3.70087300 -1.38073900 -1.60827100

C -4.53672800 -2.43233000 -2.00062100

C -4.16674400 -0.44036200 -0.68406600

C -5.83058100 -2.53921800 -1.48517900

H -4.17255000 -3.16700300 -2.71501500

C -5.45851300 -0.54577400 -0.16930400

H -3.51080100 0.35311000 -0.35042500

C -6.29483400 -1.59171800 -0.56949100

H -6.47129300 -3.35966500 -1.79685600

H -5.80766300 0.18309000 0.55618700

H -7.30043000 -1.67153000 -0.16516900

C 1.65799500 0.80587100 -2.32966100

H 1.68551900 -0.27488500 -2.48073100

H 1.43492200 1.27053700 -3.29396400

C 0.00680700 2.62262600 -1.51720700

H -0.09153700 2.82841900 -2.58496600

H 0.82731100 3.23269600 -1.13806700

C 2.99859600 1.24856500 -1.80606600

C 3.83397500 0.32404000 -1.17381800

C 3.43877200 2.57580900 -1.90143700

C 5.05742500 0.71592000 -0.62450000

H 3.53054200 -0.71770200 -1.11630000

C 4.65422800 2.96497000 -1.34520800

H 2.82311500 3.31204000 -2.41116800

C 5.48745900 2.04745500 -0.67992000

H 5.67259400 -0.03829200 -0.14708400

H 4.95671400 4.00462700 -1.43011400

C -1.28716500 2.96330200 -0.80883900

C -2.50899400 2.90406000 -1.48808200

C -1.31685400 3.32528400 0.54624800

C -3.71818800 3.14838800 -0.83255400

H -2.52067800 2.66030200 -2.54558200

C -2.52322300 3.57419100 1.19585300

H -0.38666900 3.40650900 1.09820000

C -3.75612200 3.47223200 0.52917300

H -4.63645600 3.07332400 -1.40364900

H -2.50016600 3.84683500 2.24690000

C 6.80252700 2.52267600 -0.04927000

C 7.70840800 3.13128400 -1.14032700

H 7.23260000 3.98597000 -1.63284300

H 8.65068700 3.48006900 -0.70030800

H 7.94700900 2.38740300 -1.90982100

C 7.56724800 1.37782800 0.63350600

H 6.98024500 0.91970800 1.43793000

H 7.84443600 0.59147400 -0.07785900

H 8.49130300 1.76706600 1.07573600

C 6.48996100 3.59757300 1.01388700

H 5.98373600 4.46489800 0.57698600

H 5.84419800 3.19081300 1.80153000

H 7.41719400 3.95027900 1.48184300

C -5.06332500 3.71294400 1.29426600

C -5.09056600 5.16597200 1.81351200

H -6.01500700 5.35306500 2.37370300

H -4.24630500 5.37174300 2.48045300

H -5.04641500 5.87940800 0.98187700

C -6.30213900 3.48637800 0.41345300

H -7.20869800 3.65061400 1.00694700

H -6.33156300 4.17922400 -0.43531800

H -6.33983000 2.46360000 0.02170000

C -5.13634100 2.74284000 2.49308100

H -5.08529200 1.70028900 2.15885800

H -4.31542400 2.90776600 3.19882400

H -6.07859600 2.88303200 3.03681000

**TS-β1**

Thermal Correction to Free Energy: 0.868228 Hartree

SCF energy: -2401.149065 Hartree

Gibbs free energy: -2400.280837 Hartree

Coordinates:

C 0.78413900 -1.36821500 0.02617000

C -0.67058800 -1.15996600 2.07779900

O 1.31677100 -2.24912000 -0.84143000

N 0.78751400 -0.13437600 -0.33371400

O -0.96902500 -1.65471500 3.30565100

N -1.26689900 -0.08900500 1.71514600

C 1.91758300 -1.48686800 -1.91896400

C -1.77355900 -0.63444900 3.96277200

Ni -0.34386700 1.34217400 0.35241800

Br -1.68373500 1.68907700 -1.77603200

H 3.00246600 -1.55977900 -1.80602700

H 1.60628200 -1.93339100 -2.86348400

H -1.13037700 -0.11934000 4.68068400

H -2.57643800 -1.13804300 4.49640900

C -2.19844100 0.29969800 2.81214300

H -1.97682400 1.33727200 3.07550200

C 1.38008000 -0.05787000 -1.69960400

H 0.55593200 0.16909100 -2.37932100

C 0.29549700 -2.02197600 1.29982200

C 0.13798300 3.31184000 1.37072900

H -1.23876000 2.41506600 0.81929200

H -0.47621500 3.53956000 2.23280300

C 1.07397400 2.27244100 1.51130500

H 1.94486000 2.26375000 0.86464000

N 0.23672600 4.36161100 0.44849000

C -0.74718300 5.33540200 0.34521100

C 1.22738700 4.25839500 -0.61758400

H 1.14888700 5.14912800 -1.23646600

H 1.02351500 3.36904900 -1.21975100

H 2.23358300 4.19044900 -0.19639800

C -1.87694400 5.29779800 1.35425100

H -2.41921000 4.34628000 1.31285100

H -1.51199600 5.43292400 2.37872800

H -2.56308500 6.11156100 1.11519700

O -0.69725600 6.20193100 -0.52392200

C 1.23608300 1.60936000 2.85809800

H 2.06765000 2.06174600 3.41926900

H 0.33167700 1.71308500 3.46683200

H 1.45441900 0.54397500 2.75989200

C -3.64378100 0.23031900 2.33064200

C -4.60942900 -0.60784200 2.89721600

C -4.01814100 1.05971500 1.26225400

C -5.92300400 -0.61197600 2.41524000

H -4.35935500 -1.26891800 3.71961600

C -5.32705500 1.06047200 0.78487900

H -3.26921100 1.68046200 0.78184000

C -6.28900800 0.22544500 1.36182200

H -6.65723400 -1.27108800 2.87053900

H -5.59427000 1.70854400 -0.04520700

H -7.31007000 0.22577700 0.99042100

C 2.44200400 1.00442500 -1.83825900

C 2.43678100 1.86004300 -2.94354700

C 3.46714300 1.11787700 -0.89178700

C 3.43935300 2.82087400 -3.10075300

H 1.63827800 1.78107400 -3.67613500

C 4.46491600 2.08130900 -1.04091500

H 3.48031400 0.45337600 -0.03544100

C 4.45363800 2.93663200 -2.14784000

H 3.42301100 3.48178200 -3.96300700

H 5.24917900 2.16465600 -0.29341300

H 5.23043600 3.68708500 -2.26553200

C -0.46858800 -3.34724500 0.91970100

H 0.25903800 -4.02417000 0.46776000

H -0.79576100 -3.79446900 1.86251400

C 1.52191000 -2.39776400 2.25117100

H 1.36354400 -3.43336000 2.56612000

H 1.44543100 -1.77997800 3.14661700

C -1.64386700 -3.13026100 -0.00203500

C -1.52699200 -3.29354800 -1.38568100

C -2.88335100 -2.71244900 0.50187200

C -2.60086500 -3.02452000 -2.23762800

H -0.58458800 -3.62918200 -1.80679400

C -3.94927000 -2.43400600 -0.34831200

H -3.01745700 -2.59813200 1.57309700

C -3.83213800 -2.57658600 -1.74180900

H -2.45949600 -3.16605700 -3.30316400

H -4.88440600 -2.09635500 0.08680700

C -5.02253400 -2.24156100 -2.64965500

C -6.21756800 -3.14660000 -2.28280200

H -6.52129900 -3.01057800 -1.23930700

H -7.08135300 -2.91108700 -2.91675200

H -5.96798400 -4.20465700 -2.42775900

C -4.69711200 -2.44600200 -4.13738000

H -3.87169400 -1.80322600 -4.46443900

H -4.42973800 -3.48575200 -4.35861300

H -5.57539600 -2.19297800 -4.74217100

C -5.41987300 -0.76562600 -2.43643500

H -5.74258900 -0.58320700 -1.40770100

H -4.57574700 -0.09721300 -2.64350500

H -6.24831500 -0.49544500 -3.10324600

C 2.91351100 -2.24197400 1.68496300

C 3.70958700 -1.15288100 2.04818100

C 3.46018800 -3.17575300 0.79209500

C 4.98507000 -0.96916200 1.50501000

H 3.33189000 -0.42914900 2.76356300

C 4.72189000 -2.98351600 0.23849700

H 2.88170500 -4.05084300 0.51184600

C 5.50950000 -1.86407000 0.56669900

H 5.55536000 -0.10015500 1.81276200

H 5.09769700 -3.71751800 -0.46821400

C 6.86265200 -1.65200200 -0.12283800

C 6.62137200 -1.48586100 -1.63941700

H 6.14099700 -2.37069300 -2.07081300

H 5.97898000 -0.61987600 -1.83841900

H 7.57362200 -1.33112100 -2.16167200

C 7.77213500 -2.87395100 0.12135600

H 8.73899700 -2.73167800 -0.37652900

H 7.95901100 -3.01455200 1.19273800

H 7.32903300 -3.79648900 -0.26814900

C 7.58782000 -0.39778000 0.38923700

H 7.00818500 0.51257000 0.19913900

H 7.79296400 -0.45604500 1.46440900

H 8.54816600 -0.29325500 -0.12826400

**TS-β2**

Thermal Correction to Free Energy: 0.869385 Hartree

SCF energy: -2401.147817 Hartree

Gibbs free energy: -2400.278432 Hartree

Coordinates:

N -0.63822700 -3.88304500 -1.84894700

C -2.28821000 -5.30444400 -0.65956600

H -2.43812400 -4.57606000 0.14496500

H -3.23422000 -5.79970700 -0.88168800

H -1.57016100 -6.05241800 -0.30490600

O -2.44134000 -4.70915300 -2.97601100

C -1.81085300 -4.62716800 -1.92392300

C -0.16425800 -3.19539700 -3.04925100

H -0.82985800 -3.45761400 -3.86836600

H -0.16941800 -2.11219800 -2.91067100

H 0.85644800 -3.51370400 -3.28128300

C -0.83128700 0.26886200 -1.73526400

C 0.90730900 0.91387600 -0.02492100

O -1.33532500 0.46801700 -2.98083200

N -1.43511500 -0.56318300 -0.97762900

O 1.50144700 2.01636300 0.46917800

N 0.86394900 -0.11242400 0.74797100

C -2.61122200 -0.23408200 -3.01580600

C 1.83640000 1.75547100 1.85731000

Ni -0.23395800 -1.75999400 0.53873100

Br -1.47043200 -1.58599900 2.74667200

H -3.40551900 0.51683000 -2.99368100

H -2.65915100 -0.80681300 -3.94205100

H 1.24860800 2.44036700 2.47213800

H 2.89701300 1.96845700 1.98885800

C 1.44498800 0.27965100 2.07396200

H 0.62498100 0.19335300 2.78787800

C -2.58076100 -1.11194200 -1.74102000

H -2.33674600 -2.13945100 -2.00641000

C 0.41200100 1.08132300 -1.45290600

C 0.08828400 -3.74944800 -0.65841300

H -1.13915100 -2.91313000 0.43653900

H -0.01443200 -4.57925900 0.02966700

C 2.31720900 -3.32053100 0.40652200

H 2.90688300 -2.46428800 0.73128300

H 1.94859600 -3.84193300 1.29586600

H 2.99533300 -4.00665000 -0.12378700

C 1.18706000 -2.90352100 -0.50486800

H 1.46616700 -2.27260000 -1.34198000

C 2.57035000 -0.62372800 2.52863000

C 2.29969700 -1.64454500 3.44707900

C 3.87519500 -0.47599200 2.04228200

C 3.31273800 -2.51063700 3.86339700

H 1.28418600 -1.76892300 3.81186400

C 4.88871500 -1.34203300 2.45397200

H 4.10537700 0.30293300 1.32482500

C 4.60936400 -2.36472400 3.36467300

H 3.08875100 -3.30076600 4.57520800

H 5.89464800 -1.21836800 2.06162400

H 5.39820900 -3.03872900 3.68739400

C -3.87385400 -1.12692400 -0.96436900

C -4.70174100 -2.25273200 -1.03099900

C -4.27790700 -0.02233700 -0.20514100

C -5.92229100 -2.27647700 -0.35127800

H -4.38802900 -3.11191900 -1.61877500

C -5.49576000 -0.04523700 0.47445100

H -3.63472800 0.84890600 -0.14195200

C -6.32189700 -1.17172900 0.40297500

H -6.55611900 -3.15724800 -0.40937100

H -5.80078700 0.81457100 1.06359300

H -7.26906500 -1.18731700 0.93528900

C 1.52587400 0.59199300 -2.46288300

H 1.27594400 1.02117700 -3.43719800

H 1.43938900 -0.49312400 -2.55511900

C 0.05893900 2.58767600 -1.72384700

H -0.09199900 2.68203100 -2.80112100

H 0.92645200 3.19207600 -1.45860900

C 2.94116600 0.93086200 -2.07079000

C 3.75972700 -0.04765400 -1.50233900

C 3.47692900 2.21616800 -2.23879300

C 5.06361200 0.24591500 -1.09477000

H 3.37552000 -1.05481300 -1.36929000

C 4.77008900 2.51054000 -1.81697200

H 2.87563100 2.99416500 -2.70094800

C 5.59261500 1.53510700 -1.22389500

H 5.65592200 -0.54863400 -0.65634900

H 5.14473500 3.52091800 -1.95212500

C -1.18134300 3.07364900 -1.00232700

C -2.42182000 3.06858600 -1.65004100

C -1.14935400 3.52592600 0.32534400

C -3.59009200 3.46003100 -0.99147500

H -2.47957400 2.75051700 -2.68623400

C -2.31465000 3.91888500 0.97905300

H -0.20337000 3.57989300 0.85129900

C -3.56750800 3.88125700 0.34406600

H -4.52503600 3.42537400 -1.53908500

H -2.24068000 4.26622100 2.00529300

C 6.99751000 1.91043200 -0.73611000

C 7.74253800 0.71203600 -0.12761700

H 7.87572500 -0.09737100 -0.85448600

H 8.73786100 1.02928800 0.20353200

H 7.21644600 0.30631700 0.74432000

C 7.82830300 2.45191300 -1.91830300

H 7.36703200 3.33840900 -2.36626800

H 8.83306200 2.73218000 -1.57920900

H 7.93349300 1.69254700 -2.70236900

C 6.87515000 3.00422200 0.34668200

H 6.38887700 3.90557100 -0.04127100

H 6.28819100 2.64414900 1.20034900

H 7.86893900 3.28935800 0.71317400

C -4.82694700 4.31658900 1.10362800

C -4.73883300 5.83087600 1.39024300

H -5.62411200 6.16716300 1.94414100

H -3.85369900 6.07376300 1.98860700

H -4.68348300 6.40317600 0.45650600

C -4.91878300 3.55386900 2.44218800

H -4.07070800 3.77568200 3.09809700

H -5.83401800 3.83804600 2.97536600

H -4.94222700 2.47069800 2.27810500

C -6.10992400 4.04207500 0.30302200

H -6.98299700 4.33354300 0.89784900

H -6.14028800 4.61549000 -0.63024600

H -6.21204600 2.97936700 0.05524300

**TS-α1-Z**

Thermal Correction to Free Energy: 0.866017 Hartree

SCF energy: -2401.143158 Hartree

Gibbs free energy: -2400.277141 Hartree

Coordinates:

N 0.24560100 -3.31738000 2.25657600

C -1.32500000 -1.52109800 2.93247300

H -2.21263500 -2.15950800 3.00946900

H -1.37827200 -0.74547500 3.69774000

H -1.33712400 -1.06397200 1.94175200

O 0.65072700 -2.10913400 4.15209700

C -0.06407700 -2.32276100 3.17129200

C 1.43583400 -4.12691900 2.50664200

H 2.15760500 -4.02304400 1.69281600

H 1.88514300 -3.77396800 3.43260000

H 1.16320900 -5.18022800 2.61259400

C 0.86625900 0.32912000 0.65802700

C -1.30346000 0.19385900 -0.62602100

O 1.47062700 1.00979600 1.65352300

N 1.35269300 -0.81307500 0.34315000

O -2.22795400 0.84466900 -1.35265900

N -1.39991900 -1.08454800 -0.57914800

C 2.58267700 0.20075800 2.11519300

C -3.23708000 -0.13125500 -1.73264100

Ni 0.13452700 -2.39196500 -0.54177200

Br 0.57483100 -1.98528600 -3.04262100

H 3.48559200 0.80681000 2.03509600

H 2.39224200 -0.06133500 3.15727000

H -3.44040200 -0.00757200 -2.79633100

H -4.13651400 0.08571300 -1.15121500

C -2.58758200 -1.48645400 -1.38019700

H -2.20540100 -1.97731300 -2.27865800

C 2.56230700 -1.02836800 1.17036700

H 2.41678300 -1.93468500 1.75755900

C -0.27302900 1.09488400 0.00970600

H 0.99882800 -3.57354900 -0.70816300

C -0.21174300 -4.36660300 -0.00970500

C -0.49318200 -3.46678700 1.06183400

H -1.51695300 -3.12901800 1.13945600

C 3.82308000 -1.19110500 0.33917200

C 3.77292600 -1.43748400 -1.03688800

C 5.07185700 -1.09523700 0.96849600

C 4.95458600 -1.55530600 -1.77407000

H 2.81241900 -1.52203600 -1.53400600

C 6.25151400 -1.21501300 0.23176900

H 5.12693100 -0.91854500 2.03992800

C 6.19693300 -1.43826800 -1.14699900

H 4.89952200 -1.73289700 -2.84520000

H 7.21118700 -1.13043600 0.73447700

H 7.11377800 -1.52417200 -1.72375800

C -3.46463500 -2.44834000 -0.61450300

C -3.66081100 -3.74831000 -1.09127900

C -4.05263400 -2.07285200 0.60110000

C -4.42436400 -4.66514500 -0.36354100

H -3.20909200 -4.04351500 -2.03499100

C -4.81374800 -2.98573800 1.33051700

H -3.90535600 -1.06710900 0.98229300

C -4.99845900 -4.28678600 0.85123200

H -4.56703900 -5.67229700 -0.74524900

H -5.26077700 -2.68364400 2.27364700

H -5.58940100 -4.99851100 1.42095200

C 0.31885300 1.97928600 -1.16508600

H 0.39673000 1.33387200 -2.04477800

H -0.41839000 2.75474900 -1.38365400

C -0.93081900 1.98057500 1.13803000

H -1.02083000 1.35255900 2.02759100

H -0.20692900 2.76355100 1.37177200

C 1.67324000 2.56583600 -0.86654100

C 2.82354000 1.85035400 -1.22510500

C 1.83969500 3.76758400 -0.17368700

C 4.08946200 2.29121700 -0.85520300

H 2.72007100 0.92413300 -1.78042100

C 3.11334800 4.21502200 0.19000200

H 0.96864400 4.35880900 0.09768000

C 4.26434900 3.47864300 -0.12382400

H 4.95107200 1.69162200 -1.13348500

H 3.19552600 5.14885400 0.73487900

C -2.27684500 2.58364600 0.83575600

C -3.44556700 1.91622300 1.22799500

C -2.41471800 3.80339800 0.16997000

C -4.70234400 2.42706200 0.92109000

H -3.36402700 0.98055000 1.77615300

C -3.67813800 4.31628600 -0.13894500

H -1.52815700 4.36325600 -0.11709300

C -4.84958600 3.63500900 0.21565300

H -5.58294800 1.87442700 1.23544900

H -3.73546100 5.26286800 -0.66456400

C 5.67461500 3.90250000 0.30589600

C 6.57090200 4.05734400 -0.94075300

H 7.58396900 4.35629300 -0.64494100

H 6.64958000 3.12105900 -1.50317700

H 6.17330200 4.82485700 -1.61554000

C 6.26475500 2.81003700 1.22422300

H 6.31897000 1.84057800 0.71838100

H 7.27974600 3.08401700 1.53795100

H 5.65294200 2.68638900 2.12607100

C 5.67684900 5.23401700 1.07233400

H 5.28504000 6.05547500 0.46139400

H 5.08097000 5.17633700 1.99036600

H 6.70314400 5.49154200 1.35789300

C -6.25359700 4.14913200 -0.12836500

C -6.97491100 3.09889800 -1.00041500

H -7.07500300 2.14030000 -0.48020100

H -7.98236700 3.44676700 -1.25981500

H -6.42542100 2.92150200 -1.93271200

C -7.05449900 4.36729600 1.17272100

H -8.06427300 4.72789800 0.94130200

H -7.15351000 3.44063800 1.74801200

H -6.56569100 5.11182800 1.81240900

C -6.21616500 5.47608300 -0.90210300

H -5.73866900 6.27319100 -0.32082100

H -5.67971300 5.37761800 -1.85280800

H -7.23916000 5.79717100 -1.12897600

H -1.08291200 -4.45566500 -0.65758000

C 0.58938400 -5.66283800 -0.03512300

H 0.05604100 -6.43154900 0.53811400

H 0.65968100 -6.01085900 -1.07121600

H 1.60224100 -5.58036300 0.35691400

**TS-α2-Z**

Thermal Correction to Free Energy: 0.870188 Hartree

SCF energy: -2401.149821 Hartree

Gibbs free energy: -2400.279633 Hartree

Coordinates:

N -1.37527400 -3.20797000 1.49675200

C -0.82169000 -2.62544300 3.85399600

H -0.19662800 -1.75635800 3.62749800

H -1.37370000 -2.42715600 4.77395700

H -0.16051100 -3.48418900 4.01223900

O -3.04666800 -2.81415400 2.98720200

C -1.83830000 -2.88557400 2.75862700

C -2.37708300 -3.39986000 0.44949100

H -3.26123300 -2.81987900 0.70738500

H -1.97459100 -3.04928900 -0.49669900

H -2.66598900 -4.45275000 0.36197800

C 0.73124100 0.37205900 1.76955200

C -0.80503600 0.77959600 -0.19178900

O 1.15852200 0.82754200 2.96642100

N 1.32820400 -0.66097300 1.29729500

O -1.38059100 1.76509400 -0.91803200

N -0.69289300 -0.36707900 -0.74365200

C 2.36580400 0.08717700 3.29507800

C -1.80480500 1.16725900 -2.17554600

Ni 0.71792700 -1.87895500 -0.17524200

Br 2.30851600 -1.28231700 -2.13007400

H 3.21222600 0.76883100 3.18414700

H 2.28637200 -0.24913900 4.32867100

H -1.42787900 1.79221800 -2.98590800

H -2.89607200 1.17215900 -2.18566700

C -1.17495300 -0.24013000 -2.14903400

H -0.27353300 -0.27531200 -2.76689800

C 2.38611200 -1.06297900 2.26528400

H 2.06507000 -1.99248100 2.74286800

C -0.43415500 1.16805800 1.22251200

C 0.61958900 -3.95368700 0.12955100

H 0.34156900 -2.87242300 -1.16655900

C 0.00249800 -3.24723800 1.18891000

H 0.62443200 -3.03266200 2.04132600

C -2.07008300 -1.39309300 -2.54345600

C -1.49252700 -2.49475100 -3.18891700

C -3.43113200 -1.43601300 -2.21901700

C -2.25563700 -3.62449600 -3.49007100

H -0.43463000 -2.46511400 -3.43433300

C -4.19769800 -2.56247600 -2.52303000

H -3.89861100 -0.59983500 -1.71139200

C -3.61110200 -3.66300100 -3.15305000

H -1.79182300 -4.47315200 -3.98544700

H -5.25216200 -2.58181900 -2.26117400

H -4.20716100 -4.54136500 -3.38496000

C 3.73203700 -1.29719800 1.62528500

C 4.38685600 -2.51719000 1.81705700

C 4.35542900 -0.29453200 0.87347300

C 5.65572500 -2.73454900 1.27261400

H 3.90315600 -3.29788100 2.39953800

C 5.62332000 -0.50600200 0.33624800

H 3.84341500 0.64599900 0.70193900

C 6.27788600 -1.72603900 0.53450100

H 6.15646400 -3.68602300 1.43020600

H 6.09987900 0.27863500 -0.24446800

H 7.26669500 -1.88875100 0.11402900

C -1.66421600 0.84749500 2.16229600

H -1.70733300 -0.23807600 2.27874500

H -1.43891000 1.28144100 3.14028400

C -0.06564700 2.68845600 1.29199100

H -0.02525900 2.96245400 2.34804100

H -0.88494300 3.24688800 0.83768700

C -2.98917900 1.33811900 1.64286400

C -3.80640800 0.48285000 0.89933300

C -3.42964200 2.65164400 1.85172400

C -5.01404900 0.92776700 0.35776600

H -3.49066100 -0.54242700 0.73216300

C -4.63259700 3.09457400 1.30712900

H -2.82492600 3.33348900 2.44382900

C -5.45009800 2.24682000 0.53871800

H -5.61350700 0.22564900 -0.21045700

H -4.93842800 4.12131100 1.48551800

C 1.25388800 3.02457100 0.62853700

C 2.42502100 3.12715800 1.38714700

C 1.36330800 3.21182800 -0.75753200

C 3.66487600 3.35607900 0.78463400

H 2.37272000 3.01823300 2.46587300

C 2.59933200 3.44416400 -1.35515300

H 0.47308100 3.16878900 -1.37524000

C 3.78507700 3.49952900 -0.60323300

H 4.54211100 3.41139700 1.41915600

H 2.63746800 3.57821300 -2.43224400

C -6.75837400 2.77962000 -0.05965000

C -7.68407100 3.26149200 1.07731300

H -7.22170800 4.06165000 1.66501600

H -8.62338700 3.64883700 0.66399500

H -7.92658500 2.43778500 1.75928700

C -7.50479000 1.70975100 -0.87205300

H -6.90300000 1.34319200 -1.71166200

H -7.78702600 0.85065200 -0.25274200

H -8.42499900 2.13885600 -1.28443000

C -6.44041100 3.96461300 -0.99642600

H -5.94640800 4.78307400 -0.46216700

H -5.78224600 3.64938300 -1.81501300

H -7.36456100 4.35993300 -1.43555300

C 5.13025600 3.71170400 -1.30855500

C 5.13718800 5.10055600 -1.98146500

H 6.08657200 5.26602200 -2.50565100

H 4.32758100 5.19591800 -2.71326500

H 5.01598900 5.89668900 -1.23713300

C 6.31589900 3.63328500 -0.33399900

H 7.25352500 3.76474000 -0.88590100

H 6.26934500 4.41707100 0.43062700

H 6.35981400 2.66268200 0.17344900

C 5.31802800 2.62367900 -2.38768000

H 5.27929400 1.62142000 -1.94644200

H 4.54445000 2.67940100 -3.16052700

H 6.29078200 2.74368300 -2.88004700

C 0.03798800 -5.07910200 -0.70721600

H -0.02684300 -5.98758400 -0.09338500

H -0.95292900 -4.87160500 -1.10993800

H 0.70316500 -5.29376700 -1.55031800

H 1.69058000 -4.07079700 0.28950000

**[Ni]BrH**

Thermal Correction to Free Energy: 0.143055 Hartree

SCF energy: -717.926511 Hartree

Gibbs free energy: -717.783456 Hartree

Coordinates:

C 0.60715700 1.94468300 -0.07624000

C 2.38623700 0.15782700 -0.09895900

O 0.36937800 3.26102900 -0.02723100

N -0.38921900 1.14782500 0.06322100

O 3.69725200 -0.09400900 -0.06533900

N 1.58865100 -0.84352800 0.03001000

C -1.07963700 3.42018400 0.12298000

C 3.85760700 -1.53674300 0.14183400

Ni -0.32485200 -0.84719000 -0.00546500

Br -2.69871100 -1.17154200 -0.06106100

H -1.25191900 4.05177300 0.99530200

H -1.44714600 3.91910900 -0.77621300

H 4.40256000 -1.67162300 1.07745300

H 4.44500800 -1.92334000 -0.69200000

C 2.41182200 -2.06485300 0.18310900

H 2.16456100 -2.55380200 1.12741900

H 2.18854300 -2.75299400 -0.63439200

C -1.59427400 1.97826100 0.28045700

H -1.99434000 1.77398300 1.27643400

H -2.35783700 1.71353100 -0.45050400

C 2.03727200 1.59102700 -0.30437600

H 2.66312600 2.20355300 0.35372600

H 2.31234700 1.87506200 -1.32993000

H -0.26031700 -2.31851800 -0.06202300

Thermal Correction to Free Energy: 0.130573 Hartree

SCF energy: -365.376536 Hartree

Gibbs free energy: -365.245963 Hartree

Coordinates:

N 0.37907000 0.39947800 -0.00026300

C 1.61814900 -1.75539800 -0.00025400

H 1.11872600 -2.16393500 -0.88557200

H 2.66028200 -2.07815600 -0.00089300

H 1.11988400 -2.16399600 0.88572000

O 2.66064000 0.39961400 0.00045200

C -2.05970000 0.27807600 -0.00014600

C -0.84330300 -0.28840600 0.00000700

H -2.16855100 1.35946200 -0.00053400

H -0.75671300 -1.36755400 0.00042700

C 1.60924500 -0.23873300 0.00001600

C -3.32687000 -0.52666900 0.00022400

H -3.12096000 -1.60318100 0.00049300

H -3.94526600 -0.30088100 0.88053400

H -3.94546400 -0.30135000 -0.88007100

C 0.34815800 1.85724700 -0.00011700

H -0.17434300 2.22516100 0.88989500

H 1.37375800 2.21911800 0.00006500

H -0.17404200 2.22534700 -0.89022100

**TS1**

Thermal Correction to Free Energy: 0.294346 Hartree

SCF energy: -1083.291129 Hartree

Gibbs free energy: -1082.996783 Hartree

Coordinates:

N -1.90718200 -0.77158900 -1.50228400

C -2.70161100 -2.52320900 0.06859100

H -1.89411200 -2.42618600 0.79851100

H -3.62626200 -2.75966700 0.59707000

H -2.44772900 -3.35419800 -0.59895200

O -4.01630500 -0.65473100 -0.65108100

C -2.93314000 -1.24156400 -0.70922900

C -2.15476500 0.40123700 -2.33545100

H -3.03677600 0.91214400 -1.95423500

H -1.29558500 1.07070900 -2.29300200

H -2.33122900 0.10581900 -3.37669100

C -0.61421100 -0.32123500 2.01476900

C -1.05564300 1.90020400 0.89330300

O -0.75411900 -0.96368600 3.18839600

N 0.24996900 -0.78542500 1.19137300

O -1.62069700 3.11236400 1.08325100

N -0.19743100 1.77694700 -0.04337000

C 0.20512400 -2.07088500 3.18260400

C -1.08687300 3.97985700 0.03243000

Ni 0.77052300 -0.07594300 -0.56936700

Br 3.21629900 0.30901900 0.04073800

H 0.88630200 -1.91687200 4.02167200

H -0.35756700 -2.99467700 3.32694100

H -0.68345000 4.87269100 0.51252400

H -1.91762500 4.25429200 -0.62227900

C -0.02459300 3.10157700 -0.66206500

H 0.99851200 3.44634700 -0.48367600

H -0.17819000 3.03369200 -1.74225000

C 0.88709300 -1.96305000 1.80404200

H 1.96372100 -1.79011700 1.86029900

H 0.70544900 -2.83973400 1.17478700

C -1.54230700 0.83846500 1.82912200

H -1.73566500 1.29024900 2.80611000

H -2.51095100 0.46356500 1.46870500

C 0.37483800 -1.07592300 -2.44606000

H 1.17091300 0.40351600 -1.89569200

H 0.11252200 -0.45505000 -3.29856000

C 1.48571600 -2.06787400 -2.69476400

H 1.64749400 -2.71216300 -1.82514700

H 1.23174200 -2.70405900 -3.55469900

H 2.42963300 -1.56140000 -2.91680000

C -0.61305400 -1.33748600 -1.48922300

H -0.52421900 -2.23995000 -0.90117500

**In1**

Thermal Correction to Free Energy: 0.302465 Hartree

SCF energy: -1083.324044 Hartree

Gibbs free energy: -1083.021579 Hartree

Coordinates:

N -2.63233100 -0.47028900 0.40395100

C -1.20899600 -0.31637500 2.42210200

H -0.36610600 0.19399400 1.94437300

H -1.31068700 0.04264400 3.44883700

H -0.95999900 -1.38213900 2.42774700

O -3.44501500 0.51150100 2.30121300

C -2.51588400 -0.05002200 1.70345000

C -3.98368100 -0.41727900 -0.15691900

H -4.50859200 -1.37344700 -0.01850100

H -4.53836600 0.36070400 0.36490000

H -3.96464000 -0.19239000 -1.22304600

C 2.26836200 1.58629100 0.32903400

C -0.00735400 2.44431300 -0.06032700

O 3.53362800 1.99832600 0.48073600

N 2.06429800 0.45499100 -0.23535000

O -0.60735400 3.61269200 -0.30693300

N -0.49329400 1.39260600 -0.62069900

C 4.37897300 0.89834100 -0.00225700

C -1.78981100 3.30833100 -1.11876500

Ni 0.21333700 -0.39689600 -0.25607000

Br 1.24475000 -2.51726600 0.47827200

H 5.10363800 1.32203500 -0.69779300

H 4.89419600 0.48129500 0.86645400

H -1.79925200 3.99884200 -1.96194000

H -2.66675500 3.47114800 -0.48752500

C -1.59215000 1.83530400 -1.50601000

H -1.28053300 1.70618000 -2.54698800

H -2.48622300 1.24880500 -1.32690500

C 3.37813200 -0.07923300 -0.64579500

H 3.43376600 -0.08144400 -1.73942600

H 3.48547900 -1.10200300 -0.28637600

C 1.19699000 2.50465300 0.82435000

H 1.57044100 3.52959300 0.86153400

H 0.91458600 2.22047600 1.84727000

C -1.92791100 -1.58130200 -1.72626300

H -2.11668100 -0.67160800 -2.30750400

H -2.86643800 -2.15620300 -1.75424700

C -0.85402100 -2.39918300 -2.44927700

H 0.07655200 -1.83083000 -2.55785000

H -0.60832200 -3.31358300 -1.90037100

H -1.20339000 -2.67845200 -3.45151000

C -1.56723900 -1.23819900 -0.27668500

H -1.41558300 -2.17136500 0.27404700

 (Ethyl radical)

Thermal Correction to Free Energy: 0.035323 Hartree

SCF energy: -79.188656 Hartree

Gibbs free energy: -79.153333 Hartree

Coordinates:

C 0.79421500 0.00000000 -0.02498100

H 1.35245100 0.92890000 0.05461900

H 1.35244000 -0.92890800 0.05461700

C -0.69330300 0.00000200 -0.00119900

H -1.09090300 -0.00014200 1.03078900

H -1.10972800 0.88939700 -0.49135500

H -1.10973300 -0.88926100 -0.49159300

**In2**

Thermal Correction to Free Energy: 0.364526 Hartree

SCF energy: -1162.569930 Hartree

Gibbs free energy: -1162.205404 Hartree

Coordinates:

N 1.86335800 0.50313700 -1.47167900

C 3.09533900 1.55911200 0.40360600

H 2.21415800 1.62091100 1.04108200

H 3.97088000 1.33172000 1.01423000

H 3.23391500 2.54020800 -0.06643700

O 3.82873800 -0.39622000 -0.77458200

C 2.96347400 0.47762900 -0.64493200

C 1.86342100 -0.47383700 -2.56233500

H 2.54217400 -0.14939000 -3.36014200

H 2.21070700 -1.44252300 -2.20430200

H 0.86373500 -0.57849900 -2.97180300

C 1.14896700 -1.31123400 1.64503500

C 0.19005200 -2.28339500 -0.48822300

O 1.78346700 -1.64882100 2.78169600

N 0.53940100 -0.18679900 1.61878200

O 0.00245800 -3.45784600 -1.11828300

N -0.52749800 -1.29247500 -0.86049700

C 1.67547600 -0.48836500 3.66987900

C -0.97646400 -3.20926300 -2.17832000

Ni -0.61187700 0.64004900 -0.01286000

Br -3.18291600 -0.14152200 0.82311600

H 1.36422700 -0.84835100 4.65051800

H 2.66730000 -0.03340500 3.73423600

H -1.75880700 -3.96401000 -2.08957500

H -0.45867900 -3.32037800 -3.13394600

C -1.45442300 -1.77192500 -1.90281400

H -2.47038400 -1.71712100 -1.50420100

H -1.38822200 -1.13268900 -2.78684100

C 0.64421400 0.40831200 2.96443300

H -0.34216500 0.37811200 3.43786500

H 0.96549500 1.44918100 2.90187000

C 1.26853400 -2.31793000 0.54814500

H 1.29334000 -3.31669900 0.99257100

H 2.24069700 -2.17737900 0.05343400

C 0.07309100 1.78087100 -2.62785000

H -0.56937400 0.95359200 -2.95094900

H 0.83910800 1.89144600 -3.41089300

C -0.75985000 3.06139700 -2.57923700

H -1.59097000 2.97967600 -1.87382800

H -0.15075100 3.92467800 -2.28854800

H -1.18142500 3.26811000 -3.56975200

C 0.78673600 1.44315600 -1.31794600

H 1.16389300 2.33687300 -0.83698100

C 0.00784500 3.57779900 0.92801700

H 0.89562200 3.27744200 1.49593100

H 0.35028900 3.98672300 -0.02902800

H -0.45110100 4.41635000 1.47616000

C -0.98718100 2.44423700 0.76801500

H -1.32012500 2.06897500 1.74155100

H -1.87844400 2.76501700 0.21908900

**TS2**

Thermal Correction to Free Energy: 0.364725 Hartree

SCF energy: -1162.539156 Hartree

Gibbs free energy: -1162.174431 Hartree

Coordinates:

N -0.59149300 -0.38266000 1.92315500

C 0.85841800 -2.38771600 2.12761500

H 0.93059100 -2.54556500 1.04917900

H 1.81537000 -2.63197500 2.59220600

H 0.09502600 -3.06982500 2.51981600

O 1.27070700 -0.28933300 3.21644700

C 0.53630200 -0.94701600 2.46824400

C -0.88762000 1.00182300 2.30520000

H -1.38757500 1.03445200 3.28013600

H 0.04201600 1.56281700 2.37588700

H -1.53036700 1.45307000 1.55038800

C 2.64309300 -0.75128500 -0.59061000

C 2.12913800 1.53910300 0.28542700

O 3.70586500 -1.40371900 -1.09508600

N 1.47839600 -1.08291900 -1.00980300

O 2.73413400 2.66563500 0.70469800

N 0.92752800 1.62656100 -0.15034000

C 3.16677500 -2.45305700 -1.96105300

C 1.72655200 3.72457600 0.62432200

Ni -0.36500200 -0.00960100 -0.57208100

Br -2.27120600 1.65479300 -1.46780800

H 3.71043500 -2.41814800 -2.90541600

H 3.34267600 -3.41060800 -1.46375000

H 2.17285500 4.56671900 0.09390500

H 1.47981200 4.02023200 1.64738500

C 0.55577400 3.05674500 -0.11818700

H 0.43719300 3.41403100 -1.14407600

H -0.40058000 3.18341700 0.38738200

C 1.67533100 -2.09451900 -2.07153800

H 1.41925100 -1.64602400 -3.03745600

H 1.02962600 -2.95731600 -1.91303400

C 2.96924900 0.30667700 0.41328600

H 4.02128800 0.58235900 0.31291300

H 2.83732000 -0.09404900 1.42733800

C -2.96047400 -0.73067900 1.30323200

H -3.25802900 0.09800000 0.65335300

H -3.02745400 -0.36670800 2.33595800

C -3.95052900 -1.89197400 1.16637400

H -4.04517200 -2.25259100 0.13842500

H -3.65871300 -2.74043400 1.79739700

H -4.94582500 -1.56473600 1.49038600

C -1.49521800 -1.10027100 1.03847600

H -1.32331900 -2.15263900 1.21589900

C -1.44634700 -3.15897200 -0.86134100

H -0.43859100 -3.43992500 -0.54067100

H -2.14701900 -3.57160500 -0.12862800

H -1.64174100 -3.66965500 -1.81520600

C -1.60309400 -1.65853200 -1.02866600

H -1.07228700 -1.32990900 -1.94013600

H -2.63528200 -1.33927600 -1.14229400

**[Ni]Br**

Thermal Correction to Free Energy: 0.129888 Hartree

SCF energy: -717.350432 Hartree

Gibbs free energy: -717.220544 Hartree

Coordinates:

C -1.91960900 -1.27327700 0.00054300

C -1.91677500 1.27689800 0.00030700

O -2.68482800 -2.37702500 0.00096800

N -0.64648100 -1.43239900 -0.00110800

O -2.67946800 2.38238400 -0.00122900

N -0.64328600 1.43308700 0.00065500

C -1.77556400 -3.52501200 -0.00010900

C -1.76752400 3.52828000 -0.00010000

Ni 0.73866000 -0.00121300 -0.00006400

H -1.98860700 -4.11363700 0.89391500

H -1.99131100 -4.11411900 -0.89314700

H -1.98018600 4.11637700 0.89437200

H -1.98096700 4.11886800 -0.89269700

Br 3.14171600 -0.00236900 0.00043700

C -0.36629400 2.88422900 -0.00140100

H 0.21966500 3.14348800 0.88430100

H 0.21707800 3.14127300 -0.88949000

C -0.37285500 -2.88420000 -0.00234000

H 0.21284800 -3.14418300 0.88332300

H 0.20955800 -3.14320600 -0.89047900

C -2.69687800 0.00267900 0.00212900

H -3.36038400 0.00351500 0.87663800

H -3.36450800 0.00325100 -0.86917100

**P1**

Thermal Correction to Free Energy: 0.206769 Hartree

SCF energy: -445.261046 Hartree

Gibbs free energy: -445.054277 Hartree

Coordinates:

N 0.85965700 -0.15062500 0.41747800

C 1.75700400 0.94743700 -1.61522700

H 1.21813900 1.88964300 -1.46582500

H 2.74349000 1.16589300 -2.02845400

H 1.19366700 0.35244900 -2.34284300

O 3.09865400 -0.07863000 0.09003900

C 1.96116900 0.19730100 -0.30853200

C 1.09596400 -0.90230800 1.64694500

H 1.47799300 -1.90890300 1.43740800

H 1.83149100 -0.38625400 2.26944000

H 0.16175200 -0.99141400 2.20231200

C -1.10562900 -1.34568700 -0.46505500

H -1.10789100 -2.01695100 0.40366500

H -0.41633400 -1.78334100 -1.19893300

C -2.51276800 -1.28617500 -1.06150700

H -3.25373500 -0.95397400 -0.32655100

H -2.55558100 -0.60144700 -1.91753900

H -2.82032000 -2.27818600 -1.41287200

C -0.53061000 0.01897800 -0.04115400

H -0.49014900 0.65265900 -0.92774300

C -2.34539400 1.78606800 0.41569400

H -1.79360400 2.55951600 -0.13309900

H -3.05424600 1.32741000 -0.28086400

H -2.92549900 2.28430500 1.20165500

C -1.38184800 0.75992900 1.02007000

H -0.70263800 1.28278800 1.70330300

H -1.94068000 0.03597100 1.62689200

**TS3**

Thermal Correction to Free Energy: 0.291996 Hartree

SCF energy: -1083.287702 Hartree

Gibbs free energy: -1082.995706 Hartree

Coordinates:

C -2.48401400 -1.24494500 -0.53755000

C -2.76066800 1.22549300 0.05283400

O -3.15658200 -2.38467200 -0.78611600

N -1.21227800 -1.33114800 -0.41787800

O -3.63296400 2.25874800 0.09928700

N -1.56083400 1.43331200 0.43253700

C -2.16379500 -3.45663600 -0.86660000

C -2.89697700 3.39202400 0.65726300

Ni 0.09656900 0.04535500 0.04649300

Br 0.75180400 -0.96949600 2.27311100

H -2.42709400 -4.20574000 -0.11785100

H -2.22980200 -3.89246800 -1.86543800

H -3.37428100 3.66408800 1.60155300

H -2.97653500 4.22091800 -0.04838400

C -1.46130800 2.84842400 0.82639500

H -1.10044700 2.92144500 1.85609100

H -0.73968800 3.35371500 0.17658500

C -0.82668700 -2.74312300 -0.58877400

H -0.33436800 -3.08177900 0.32560400

H -0.12070600 -2.82983600 -1.41917300

C -3.36711200 -0.04038700 -0.46254500

H -4.23414700 -0.29174000 0.16035400

H -3.77391900 0.15165100 -1.46451300

C 1.74938000 0.82437100 -1.21751500

H 1.14331700 1.06234000 0.33778000

H 1.88997100 1.89766800 -1.23650300

C 0.61407300 0.31383300 -1.88475600

H 0.66747200 -0.69291600 -2.28945600

N 2.94159900 0.09951300 -1.03675500

C 4.05962000 0.66840600 -0.44565500

C 2.91049600 -1.34737200 -1.21828400

H 3.88903900 -1.73855100 -0.94891800

H 2.69007000 -1.60073700 -2.25969300

H 2.14363200 -1.78003500 -0.56556200

C 3.99538200 2.14353900 -0.10574300

H 3.86716400 2.76039100 -1.00235700

H 3.16540500 2.36267400 0.57567100

H 4.93556700 2.41440100 0.37677200

O 5.06851900 0.00542200 -0.21601800

C -0.33053000 1.25379900 -2.59490200

H -1.34811600 0.84829700 -2.61687000

H -0.36823400 2.23284600 -2.10537500

H -0.01968900 1.41351500 -3.63857100

**In3**

Thermal Correction to Free Energy: 0.302327 Hartree

SCF energy: -1083.325361 Hartree

Gibbs free energy: -1083.023034 Hartree

Coordinates:

N 3.08414900 0.86628300 0.50906000

C 1.59112300 0.84331400 2.47649300

H 0.66954300 0.61631600 1.92692500

H 1.55581300 0.34542200 3.44745500

H 1.62949800 1.92671300 2.63237600

O 3.52969700 -0.54648100 2.22975700

C 2.80833000 0.32436800 1.72921600

C 4.18915600 0.28822900 -0.24369400

H 4.48397700 0.98597900 -1.03226600

H 5.03749300 0.11583700 0.42186400

H 3.90204600 -0.66710700 -0.69956500

C -2.73502600 -1.00995200 0.09626400

C -2.49075800 1.41747500 -0.17963700

O -3.63722400 -1.93708600 0.44342700

N -1.50977200 -1.21024100 0.40806500

O -3.22472400 2.49859700 0.10138200

N -1.21834000 1.49396800 -0.00210100

C -2.87359200 -3.03718000 1.04885200

C -2.27588200 3.56172400 0.44619100

Ni -0.04846200 -0.03374000 -0.40152000

Br 1.15626800 -2.05693800 -1.05288300

H -3.34442000 -3.27743900 2.00241100

H -2.94526200 -3.89125200 0.37111300

H -2.63377200 4.04328900 1.35569300

H -2.27252000 4.27426400 -0.38229600

C -0.94315500 2.81672800 0.60529600

H -0.66113800 2.66858500 1.65153400

H -0.13096300 3.32818800 0.09436900

C -1.44549500 -2.47158100 1.17049800

H -1.16676100 -2.24695300 2.20535700

H -0.68762900 -3.12283900 0.73566300

C -3.24503500 0.20231500 -0.62106600

H -4.30975700 0.33629600 -0.42354400

H -3.12113900 0.06406000 -1.70345600

C 0.73260300 2.05291100 -2.28893100

H 0.06147500 2.81067600 -1.87196800

H 1.53924600 2.59626100 -2.81047800

H 0.16541000 1.50909000 -3.05499900

C 2.19597100 1.77900500 -0.21562200

H 2.84576700 2.51234000 -0.72261100

H 1.62047000 2.34838400 0.51313900

C 1.30548000 1.08559900 -1.25274700

H 1.93361800 0.37338600 -1.79163600
