# Supplementary material for: Catalytic asymmetric reductive hydroalkylation of enamides and enecarbamates to chiral aliphatic amines
Source: Nat Commun. 2021 Feb 26;12:1313. doi: 10.1038/s41467-021-21600-x (PMC7910428; doi:10.1038/s41467-021-21600-x)
Supplement: Supplementary file 2 — Peer Review File [file 41467_2021_21600_MOESM2_ESM.pdf]

Editorial Note: This manuscript has been previously reviewed at another journal that is not operating a transparent peer review scheme. The manuscript was considered suitable for publication without further review at *Nature Communications.*
